# Formation of human long intergenic non-coding RNA genes, pseudogenes, and protein genes: Ancestral sequences are key players

**DOI:** 10.1371/journal.pone.0230236

**Published:** 2020-03-26

**Authors:** Nicholas Delihas

**Affiliations:** Department of Molecular Genetics and Microbiology, Renaissance School of Medicine, Stony Brook University, Stony Brook, N.Y., United States of America; University of Helsinki, FINLAND

## Abstract

Pathways leading to formation of non-coding RNA and protein genes are varied and complex. We report finding a conserved repeat sequence present in human and chimpanzee genomes that appears to have originated from a common primate ancestor. This sequence is repeatedly copied in human chromosome 22 (chr22) low copy repeats (LCR22) or segmental duplications and forms twenty-one different genes, which include the human long intergenic non-coding RNA (lincRNA) family *FAM230*, a newly discovered lincRNA gene family termed conserved long intergenic non-coding RNAs (clincRNA), pseudogene families, as well as the gamma-glutamyltransferase (*GGT*) protein gene family and the RNA pseudogenes that originate from *GGT* sequences. Of particular interest are the *GGT5* and *USP18* protein genes that appear to have formed from an homologous repeat sequence that also forms the clincRNA gene family. The data point to ancestral DNA sequences, conserved through evolution and duplicated in humans by chromosomal repeat sequences that may serve as functional genomic elements in the development of diverse genes.

## Introduction

Models presented for the pathways in formation of genes are diverse [1]. These include formation of long non-coding RNA (lncRNA) genes from protein genes [2–5], with one study based on similarities in open reading frames [5], and the reverse pathway of human protein gene formation from lncRNA genes that are found in rhesus macaque and chimpanzee [6]. Here we report new findings on an evolutionarily conserved repeat sequence that is present in multiple and diverse RNA and protein genes and propose that a similar sequence serves as a proto-gene forming unit, a nucleation site for formation of new genes, both non-coding RNA (ncRNA) and protein genes. The repeat sequence is highly prevalent in different segmental duplications or low copy repeats (LCR22) of human chromosome 22 (chr22), specifically in region 22q11.2. Chr22 has the largest number of segmental duplications per unit chromosomal length of any human chromosome [7]. These duplications are dynamic [8]. Several may have arisen after the separation of human and macaque lineages [9] and they are continuously evolving, as shown by comparisons and differences found in current human populations [10]. Segmental duplications have been considered to be important for new gene formation and human evolution [11–13]. Additionally, the 22q11.2 region in itself is of special interest as it is prone to genetic deletions formed during fetal development that result in a high rate of genetic abnormalities [14]. Segmental duplications have been shown to participate in the deletion process via meiotic nonallelic homologous recombination [9, 11].

In this paper we propose a model for the evolutionarily conserved human/chimpanzee repeat sequence and show that it serves as a starting point for formation of new lncRNA genes with subsequent base pair changes, sequence additions and/or deletions. The core sequence consists of the common sequence shared by the gamma-glutamyltransferase (*GGT*) protein gene family, where *GGT* is linked to three phylogenetically conserved and distinct sequences. In humans, these sequences form families of long intergenic non-coding RNA genes and pseudogenes that are linked to *GGT* sequences present in chromosomal segmental duplications. The presence of *GGT* in the long arm of human chr22 was determined several decades ago [15], and its duplication in segmental duplications has also been reported [11]. The *GGT* family is well characterized [16, 17].

In addition to the *GGT*-linked gene segments, we describe another protein gene family in LCR22s, the ubiquitin specific peptidase (*USP*) family that is also found linked to lincRNA genes. The formation of two specific genes, *GGT5* and *USP18* protein genes are presented here. We demonstrate that these genes originated in a primate ancestor and with use of the homologous sequence that forms the *clincRNA* genes; this suggests that the clincRNA ancestral sequence may be a nucleation for development of both lincRNA and protein genes.

The significance of chromosomal segmental duplications to gene development described here has parallels to the importance of human genome expansion of repeat units in the evolution of regulatory elements [18].

## Results

### Background on *GGT*-linked gene repeat sequences

**Fig 1:**
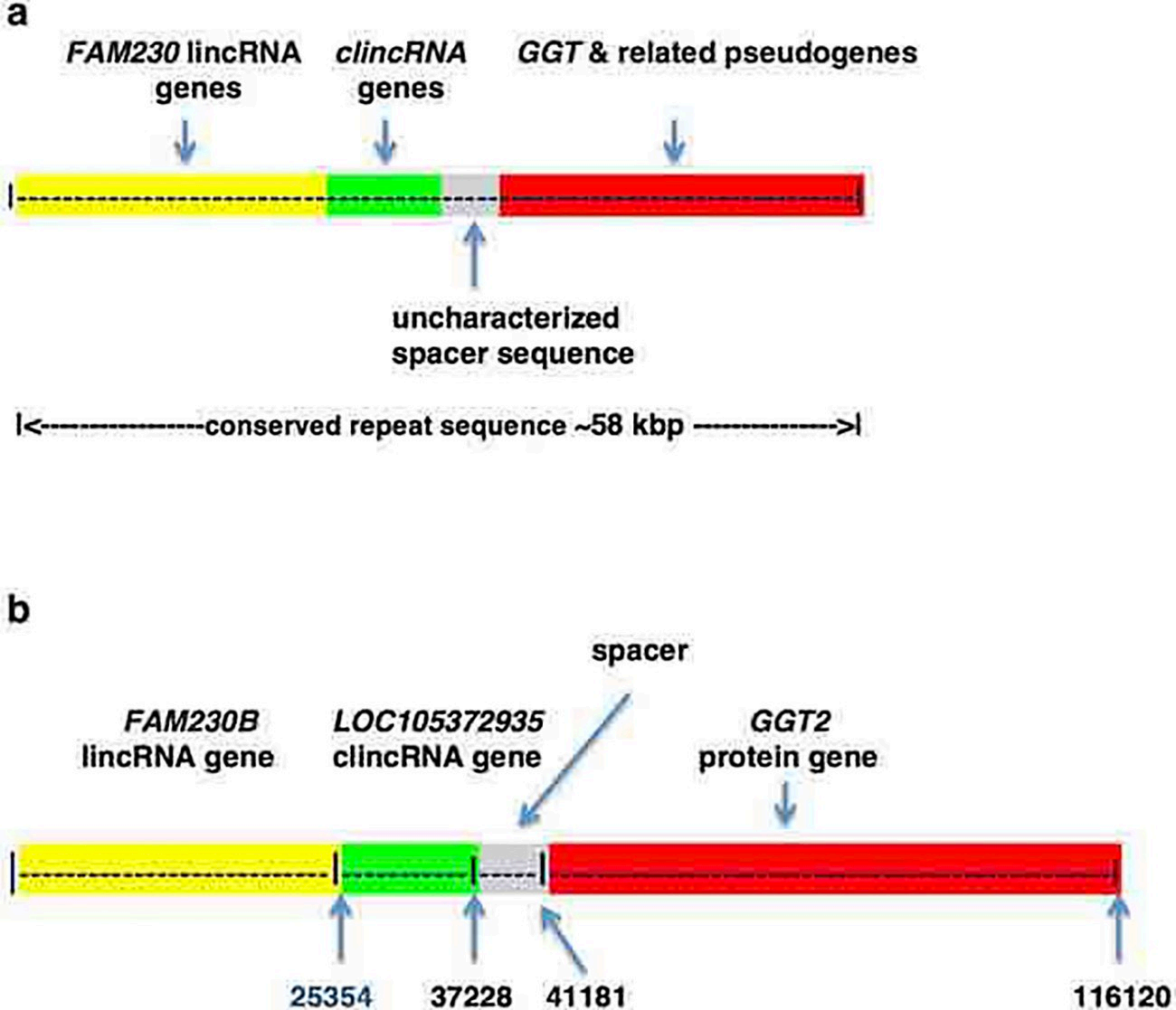
**a**. Diagrammatic representation of the conserved core sequence that comprises linked gene families found in the human chr22 LCR22A and D segmental duplications; these are: *FAM230* lincRNA gene family (highlighted in yellow), the *clincRNA* family genes (highlighted in green), a spacer sequence (highlighted in gray) and the *GGT* gene family (highlighted in red. **b.** Schematic of the linked gene segment *FAM230B*—*LOC105372935*—*GGT2*. Diagrams are approximate and not drawn to scale.

The DNA repeat sequence was detected in human chr22 segmental duplications LCR22A and LCR22D while analyzing the *FAM230* lincRNA family genes [19]. The repeat represents three gene families, whose sequences are linked (Fig 1A): the *FAM230* lincRNA gene family (highlighted in yellow), a newly found **c**onserved **l**ong **i**ntergenic **n**on-**c**oding RNA (*clincRNA*) gene family (highlighted in green) and the sequence of the *GGT* protein family as well as *GGT*-related pseudogenes (highlighted in red). An uncharacterized spacer sequence that resides between the *clincRNA* and *GGT* sequences (highlighted in gray) is also highly conserved in LCR22A and LCR22D. We refer to *GGT* as the sequence shared by *GGT1* and *GGT2* that comprises ~20,000 bp. Fig 1B is a representation of the linked gene segment *FAM230B*—*LOC105372935*—*GGT2*, which we use as a guide and model for sequence comparisons. Listed are bp numbers that show the ends of genes present in the linked gene segment, which comprises a total of 116,120 bp. The drawings are representational and not to scale.

Table 1 lists the linked gene segments, which represent copies of the conserved sequence, and their location in LCR22s. The *clincRNA* genes are those starting with the prefix LOC or AC and are linked to *FAM230* genes in LCR22A and LCR22D. Also grouped together in Table 1 are linked genes in segmental duplications LCR22E, LCR22G and LCR22H; these carry the repeat sequence but do not have the *FAM230* sequence, and some also differ with respect to the uncharacterized spacer sequence, which may be partially or totally missing. In segmental duplications LCR22E and LCR22H, pseudogenes *POM121* transmembrane nucleoporin like 1 pseudogene *POM121L1P* and the BCR activator of RhoGEF family pseudogene *BCRP3* are found linked to *GGT*; these pseudogenes stem from the *clincRNA* sequence. Thus the homologous sequence that forms the *clincRNA* gene family in LCR22A and D is found to generate pseudogenes in chromosomal segmental duplications LCR22E, and H. The *FAM230C* gene and linked genes reside in chr13 and not in chr22 or an LCR22 (Table 1).

**Table 1:** *GGT-linked* genes present in human LCR22s*.

Linked gene segments	LCR22	chr22 coordinates**
*FAM230B*—*LOC105372935*—*GGT2*	LCR22D	chr22:21166903–21283023
*FAM230E*—*LOC105377182*—*GGT3P*	LCR22A	chr:22:18733914–18791961
*FAM230A*—*AC023490*.*3*—*GGTLC3*	LCR22A	chr22:18487127–18518165
*FAM230J*—*LOC105372942*—*GGTLC5P*	LCR22A	chr22:18340163–18386526
*POM121L1P—GGTLC2*	LCR22E	chr22: 22631557–22647898
*POM121L10P—BCRP3*—*GGT1*	LCR22H	chr22:24583150–24650612
*POM121L9P (BCRP1)—GGTLC4P*—*GGT5*	LCR22G	chr22:24219654–24265524
*FAM230C*—*LOC101060145*—*GGT4P*	(chr13)	chr13:18195297–18271624

*The *FAM230* family consists of 10 genes. The genes *FAM230G*, *FAM230F*, *FAM230H and FAM230I* are not linked to a conserved ncRNA-*GGT* sequence and appear to have been formed separately in LCR22s. *FAM230D and FAM230G* are linked to *USP* genes.

**coordinates are those of the NCBI.

**Fig 2:**
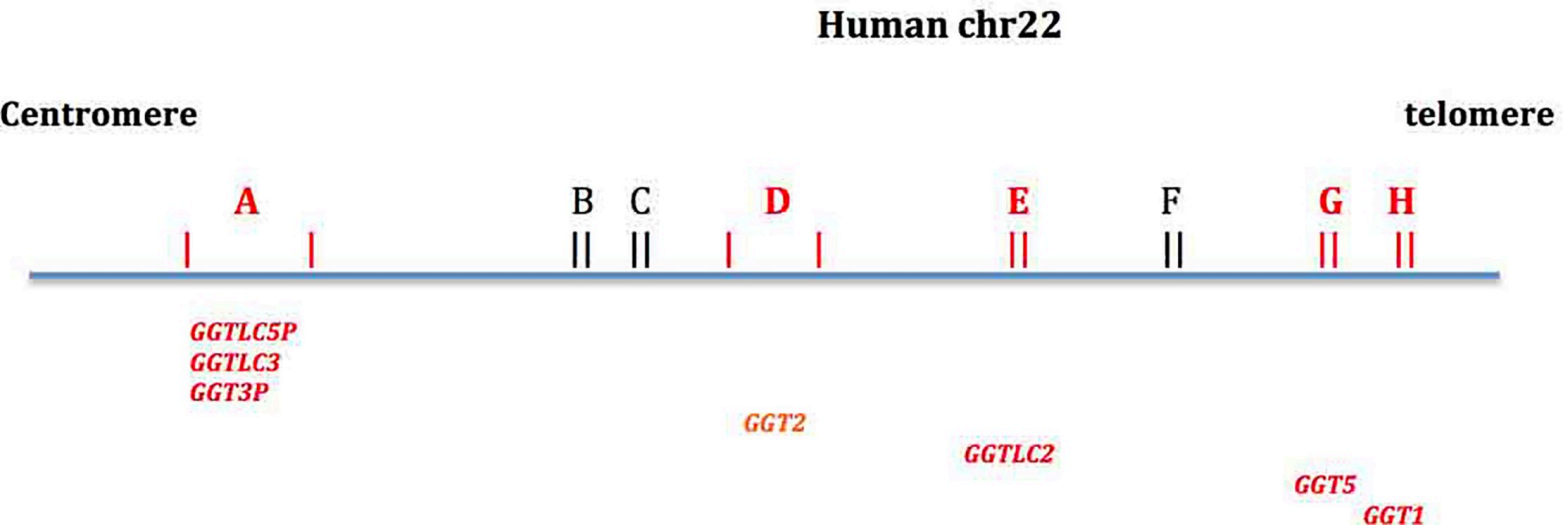
A schematic of segmental duplications found in the 22q.11.2 region of human chr22. A-H represent the eight LCR22s. The *GGT*-linked gene segments (in red) are represented by the *GGT*-related symbols in the drawing.

A diagrammatic representation of the eight LCR22s in human chr22 shows the location of the *GGT*-linked gene segments in LCR22s (Fig 2). The four linked-gene units that contain the *FAM230* gene family (Fig 1) are present only in LCR22A and LCR22D.

**Fig 3:**
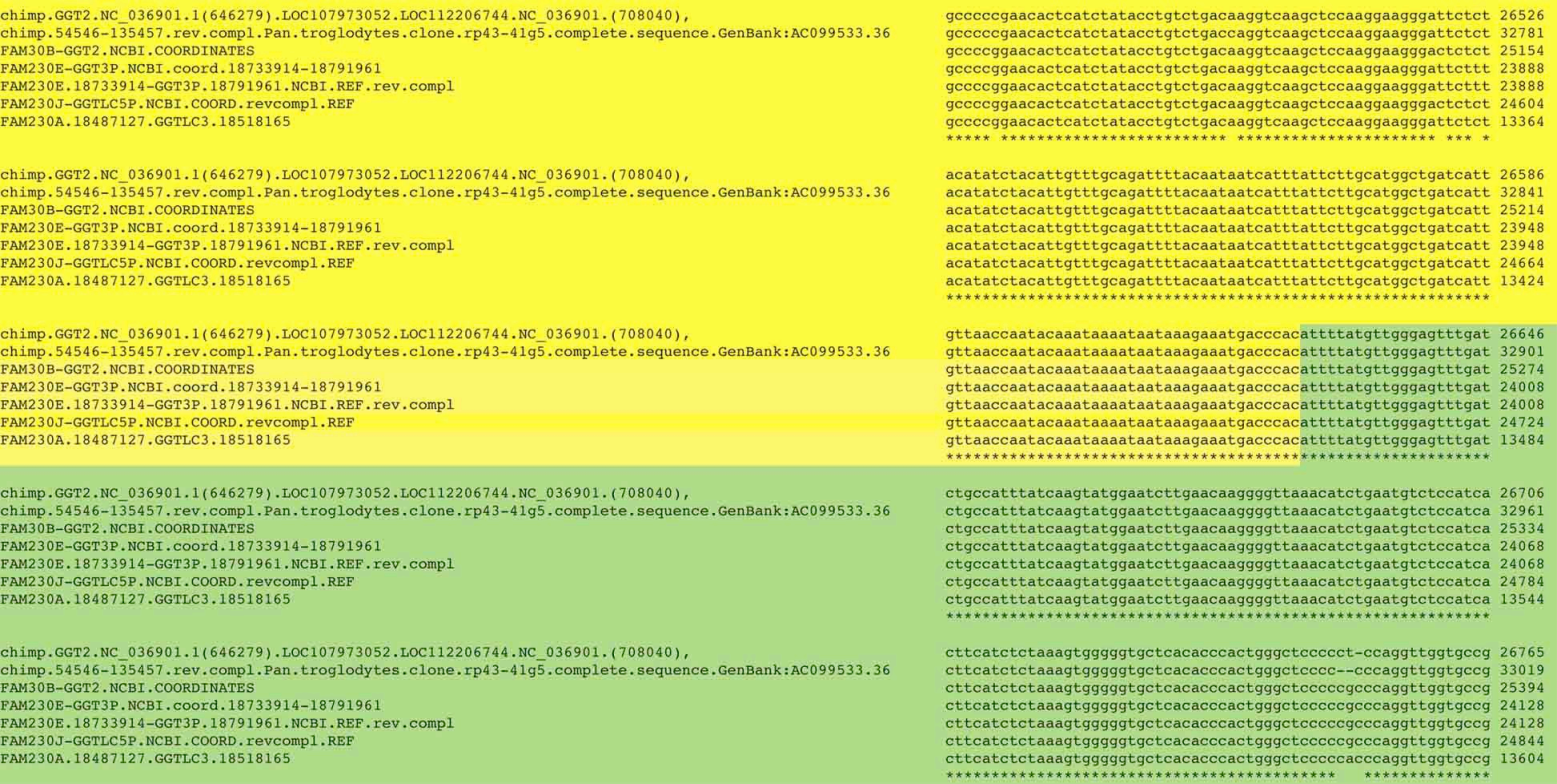
A segment of the alignment of four *FAM230-clincRNA-GGT linked genes* (Table 1) with two chimpanzee sequences. The complete sequence alignment is in S1 Fig. The yellow highlight denotes sequences of the *FAM230* genes, green highlight denotes the *clincRNA* genes with the *FAM230B—LOC105372935—GGT2* coordinates used for guideposts. The figure displays the 3’ end *FAM230B* gene/*LOC105372935* junction. The two chimpanzee sequences are from: Pan troglodytes isolate Yerkes chimp pedigree #C0471 (Clint) chromosome 22, Clint_PTRv2, NCBI Reference Sequence: NC_036901.1 and chimp.54546-135457.revcompl. from Pan.troglodytes.clone.rp43-41g5.GenBank:AC099533.36. The human sequences are from Homo sapiens chromosome 22, GRCh38.p12 Primary Assembly NCBI Reference Sequence: NC_000022.

The human DNA repeat sequence is also found present in the chimpanzee genome with high identity. A nt sequence alignment of four human *GGT*-linked gene sequences together with two homologous sequences from chr22 of the chimpanzee genome reveals the high similarity between most of the human and chimpanzee sequences (S1 Fig). Fig 3 shows a small segment of the sequences, which is taken from the complete nt sequence alignment of six *GGT*-linked gene segments. It visually displays the near perfect similarity in shared sequences at the *FAM230B* gene/*LOC105372935* (clincRNA) gene junction site (yellow/green highlighted junction). The divergence between the six sequences can be seen in (S1 Fig).

As a model for the conserved repeat sequence, the sequence of *FAM230B*-*LOC105372935*-*GGT2* is used here for all comparisons as it contains 94% of the length of the *GGT2*-linked gene counterpart in chimpanzee, *LOC112206744*-*LOC107973052-GGT2*, and displays a high nt sequence identity with the chimpanzee sequence (97%-98%). A phylogram representing a phylogenetic analysis shows a close similarity between the *GGT2*-linked gene sequence of chimpanzee and the human *FAM230B*-*LOC105372935*-*GGT2* (S2 Fig). A complete nt sequence alignment between the chimpanzee and human sequences is in S3 Fig. In this manuscript the term *FAM230-clincRNA -GGT* is used to signify the conserved repeat sequence (Fig 1A) and to represent the putative ancestral conserved sequence.

### Analyses of *GGT*-linked genes in segmental duplications LCR22A and LCR22D

**Fig 4:**
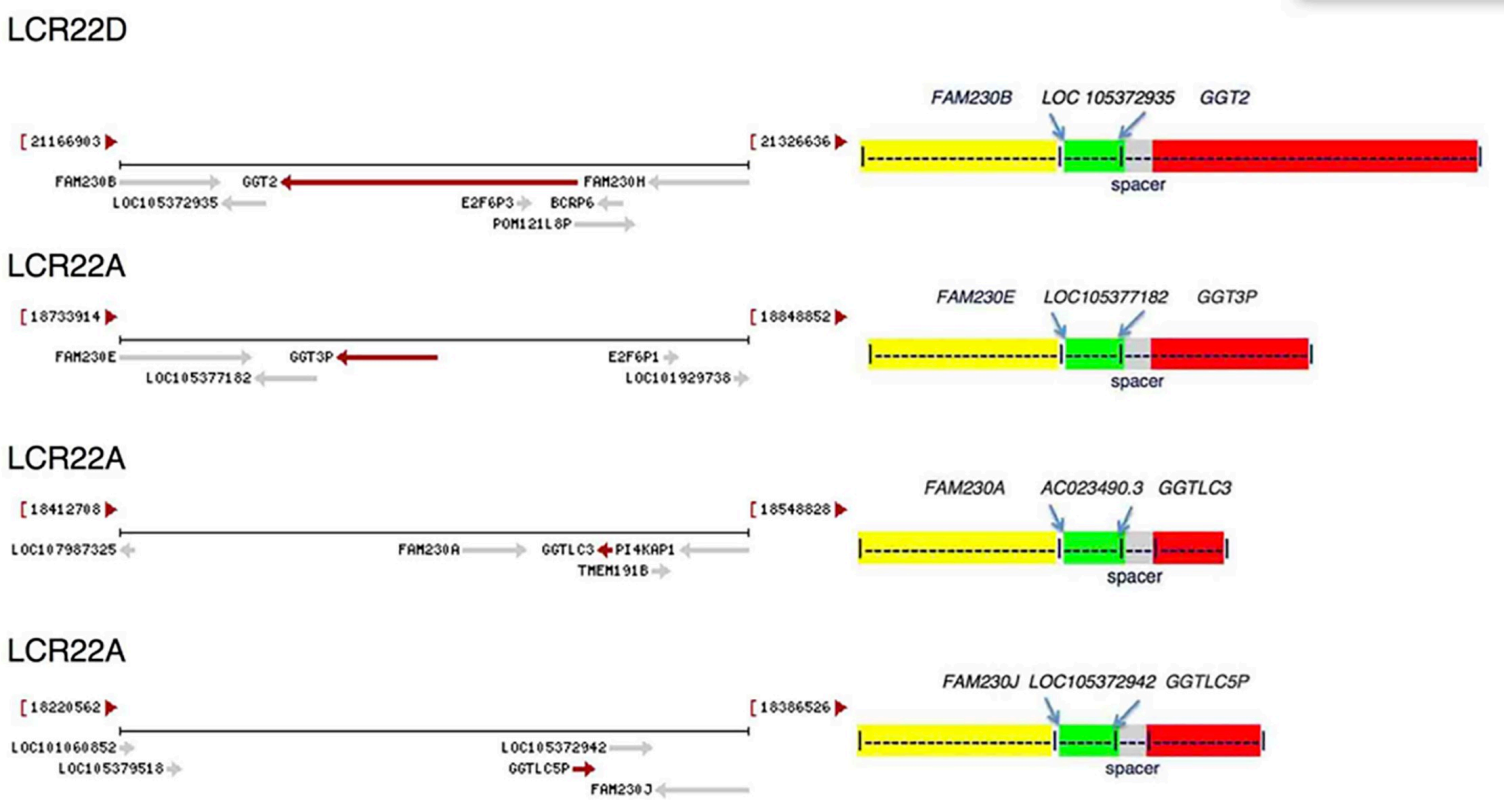
**Left panel**: *GGT* and the associated genes in LCR22s of chr22. The end chromosomal coordinates are shown in parentheses. The gene arrangement diagrams are directly from the NCBI website: https://www.ncbi.nlm.nih.gov/gene (21). **Right panel**: Schematic representation of *GGT*-linked genes (but not drawn to scale). *GGTLC5P* and its associated genes (bottom figure) are presented in the reverse orientation. Note: the *FAM230A* gene has a 50 kbp sequence gap, thus sequences from both Ensembl and NCBI were used for alignments to obtain more complete identity values. In addition, only Ensembl has annotated the clincRNA gene, *AC023490*.*3*.

NCBI displays maps of *GGT* genes and surrounding genes (www.ncbi.nlm.nih.gov/gene). These maps are shown in Fig 4, left panel. In the right panel of Fig 4, schematic diagrams represent homologous sequences, with color identification that depict the *GGT* associated gene families found in the LCR22 duplications. Table 2 shows the percent nt sequence identity obtained from sequence alignments of the *GGT*-linked gene segments with the sequence of *FAM230B-LOC105372935*-*GGT2*. Fig 1B serves as a guide for the association of the percent identity relative to each gene family as it shows the positional ends of genes. Table 2 shows the conservation of sequence, which reveals a 98%-99% identity throughout most of the lengths of the four segments. Lower identities largely correspond to changes between *FAM230* lincRNA genes.

**Table 2:** Percent identity of *FAM230*-linked genes relative to *FAM230B*-*LOC105372935*-*GGT2*.

*FAM230*-linked genes	% identity	nt positions from *FAM230B*-*LOC105372935*-*GGT2*
*FAM230E*-*LOC105377182*-*GGT3P*	98%	393–16584
	90%	16436–18838
	99%	18864–59136
*FAM230A*-*AC023490*.*3*-*GGTLC3*	99%	1507–11568
	97%	11517–16855
	98%	16647–40312
	99%	40295–42900
*FAM230J*-*LOC105372942*-GGTLC5P*	98%	1477–40312
	99%	40312–46974

*Note: small segment of *FAM230J*-*LOC105372942*-*GGTLC5P* at nt 16600–17494 has identity of 77%; this reflects changes in the *FAM230J* sequence relative to *FAM230B*

**Fig 5:**
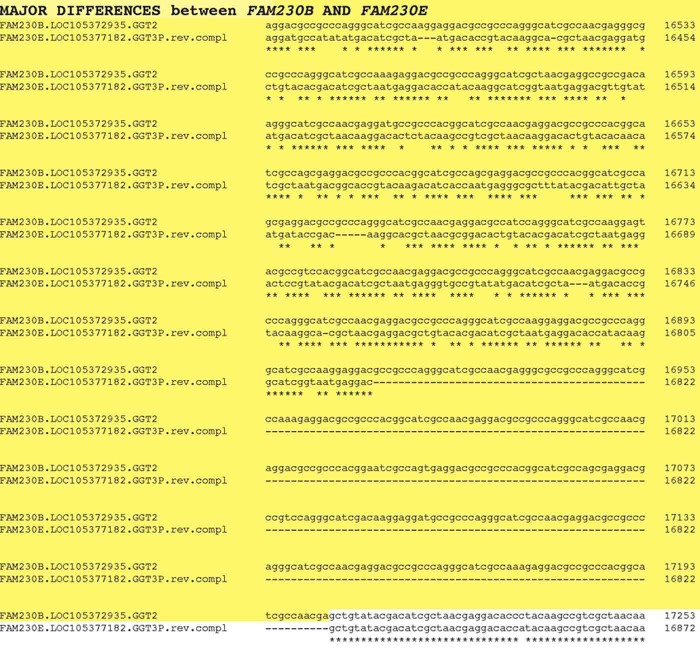
A section of the alignment of *FAM230B-LOC105372935*-*GGT2* and *FAM230E*-*LOC105377182*—*GGT3P* sequences with yellow highlighted sequences showing differences (point mutations, deletions/insertions) between the two *FAM230* genes.

A comparison of *FAM230E*-*LOC105377182*-*GGT3P* and *FAM230B—LOC105372935*—*GGT2* sequences indicates that the major sequence changes are between lincRNA *FAM230B* and *FAM230E* genes. Fig 5 shows significant mutational changes in one region involving a large sequence deletion and several point mutations between the two *FAM230* sequences. This region is followed by over 8 kbp that show no major additions/deletions/point mutation. These differences may show the development of the *FAM230* genes into distinct structures and possibly different functions. For example, lincRNA transcripts from *FAM230B* and *FAM230E* differ in nt sequence, length and exon sequences [20, 21]. Although the expression of RNA in normal somatic tissues from these genes is found only in testes [22, 19], the expression of circular RNAs (circRNA)s during fetal development shows differences between certain tissues [23] (S4 Fig). *FAM230E* circRNA is expressed in fetal heart tissue at 10 weeks and 17 weeks development, whereas *FAM230B* circRNA is not expressed in this tissue. This may be of significance in terms of possible *FAM230E* RNA gene function in the 22q11.2 chromosomal region during fetal development, as a 22q11.2 deletion results in abnormal heart development [14]. It has been shown there are genetic factors that may influence expression of circRNAs, resulting in differences in circRNA expression and the onset of various diseases [24].

The *clincRNA* genes *LOC105372935* and *LOC105377182* (Fig 4, right panel, top two drawings) are nearly identical, both in sequence and length. The RNA transcripts from these genes show small a difference in length but they are nearly the identical in sequence. The expression of RNA from these two genes in somatic tissues, as well as the expression of circular RNA during fetal development, are also nearly identical [21, 23]. Formation of the *clincRNA* genes may be recent, as they have not significantly diverged in sequence or in tissue-specific transcript expression.

*GGT3P* (Fig 4, right panel, second drawing from top) is an unprocessed pseudogene and comprises 18,273 bp. Its entire sequence is homologous to the 3’ end nt sequence of *GGT2* and *GGT1* protein genes. RNA transcript expression from *GGT2* and *GGT3P* in normal somatic tissues between the two genes is similar [21]. The expression of circular RNAs during fetal development also shows similar patterns [23].

*GGT2* (Fig 4, right panel, top drawing) is a complex gene that encodes thirteen different transcripts. Most transcripts differ in size due to the presence of multiple exons in the *GGT2* 5’ UTR (see: www.ncbi.nlm.nih.gov/gene/728441) (20). The transcript that is used here as a model for gene size is the longest (NCBI GenBank ACCESSION NM_001351304 XM_016999937). *GGT2* produces a protein product [www.uniprot.org/uniprot/P36268], however the protein is inactive in glutathione hydrolase activity and its enzymatic activity has not been fully characterized [17].

Two other *GGT*-linked gene segments found in LCR22A, *FAM230A*-*AC023490*.*3*-*GGTLC3* and *FAM230J*-*LOC105372942*-*GGTLC5P* also show a high identity with *FAM230B*-*LOC105372935*—*GGT2* (Table 2), but here there are regions of major sequence changes within *FAM230* and differences in *GGT*-related genes in sequence lengths. In these segments, the *GGT* sequence forms the protein gene *GGTLC3*, the gamma-glutamyltransferase light chain family member 3, and the unprocessed pseudogene *GGTLC5P* is the gamma-glutamyltransferase light chain 5 pseudogene. The *GGTLC3* sequence consists only of the 3’ end sequences of *GGT1/GGT2* and displays an identity of 97% with *GGT1*, but closer identity with *GGT2*, 99%. An alignment of the three gene sequences reveals thirty-nine point mutations and three deletion/insertion mutations that are unique to *GGT1* relative to the other two sequences, and only one point mutation that is unique to *GGT2* and there are no deletions/insertions. This highly biased mutational pattern suggests that *GGTLC3* originated from a sequence similar to that of *GGT2*.

### *GGT*-linked genes in segmental duplications LCR22E, H and G

**Fig 6:**
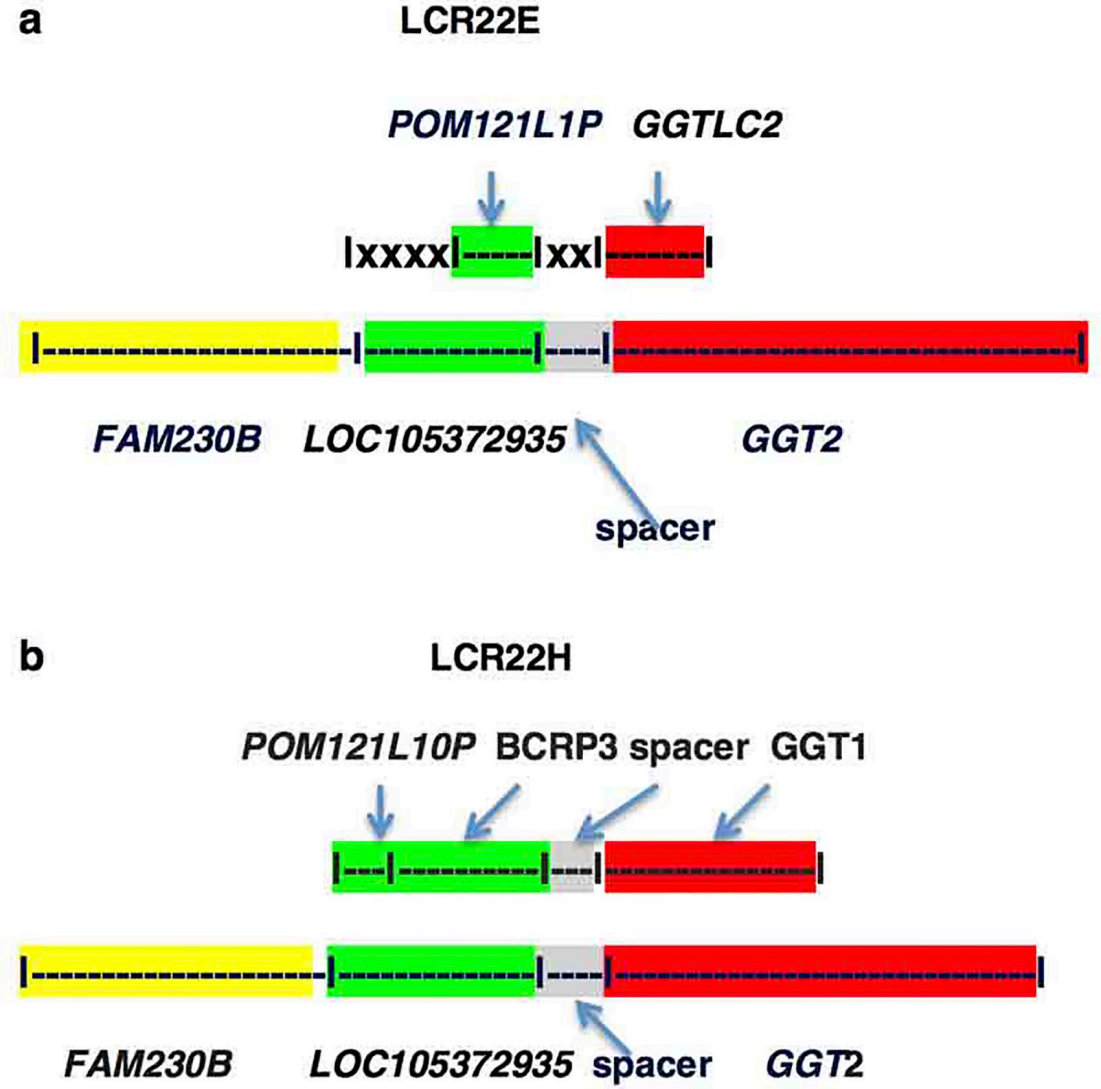
Schematic representation of *GGT*-linked genes in LCR22E, and H and comparisons with *FAM230B-LOC105372935*-*GGT2*. **a**. |xxx| represents the absence of parts of the *LOC105372935* clincRNA and the entire spacer sequences. The percent identity relative to nt positions of *FAM230B-LOC105372935-GGT2* are, *POM121L1P*, nt positions 28645–30907 96%; *GGTLC2*, positions 40838–43793, 96%; **b**. Percent identity of nt postions 28900–40312 of *FAM230B-LOC105372935-GGT2* with *POM121L10P—BCRP3*—spacer, 96%; with *GGT1*, nt positions, 40295 to 57246, 97%. The lengths of genes in the figure are not to scale.

Linked gene sequences in segmental duplications LCR22E, H and G (Table 1) differ from those in LCR22A and D. They do not carry the *FAM230* sequence, and in one case, sections of the clincRNA sequence and the spacer region are missing. Fig 6 shows a schematic drawing depicting the differences relative the *FAM230B-LOC105373935-GGT2* model.

### POM121L1P-GTTLC2

The *POM121L1P-GTTLC2* linked gene segment (in LCR22E) resides in a complex chromosomal region, the immunoglobulin lambda gene locus *IGL*. There are six genes packed into a space of ~3.2 kbp that also contains the *POM121L1P-GTTLC2* linked gene segment [20]. There is evidence that the conserved repeat sequence was duplicated in this chromosomal region but it is significantly different; there is a partial *clincRNA* gene sequence present in *POM121L1P*-*GGTLC2* and *FAM230* and spacer sequences are missing (Fig 6A) (the symbol |XXX| refers to clincRNA and spacer sequences missing).

*POM121L1P* is termed a POM121 transmembrane nucleoporin like 1 unprocessed pseudogene [22]. 2279 bp of the *POM121L1P* pseudogene sequence has an identity of 96% with aligned sequences of the *clincRNA* gene *LOC105372935* of *FAM230B*—*LOC105372935*—*GGT2*. Thus, part of the conserved sequence that forms *clincRNA* genes in LCR22A and LCR22D (green highlight, Fig 6) forms part of this pseudogene in LCR22E.

*GGTLC2* encodes a gamma-glutamyltransferase light chain 2 protein and displays glutathione hydrolase activity [https://www.ebi.ac.uk/interpro/protein/Q14390]. It shares most of its sequence with *GGT2* and *GGT1* and displays an identity of 96% with *GGT2* but 98% with *GGT1*, however a mutational analysis to determine the closeness of *GGTLC2* with *GGT1* relative to *GGT2* is inconclusive.

### POM121L10P-BCRP3-GGT1

*POM121L10P-BCRP3*-*GGT1* resides in LCR22H. Based on a sequence alignment with *FAM230B—LOC105372935—GGT2*, the *clincRNA* sequence and the entire uncharacterized spacer sequence are present and are linked to *GGT1* (Fig 6B). Sequences of the *POM121L10P and BCRP3* genes both stem from the *clincRNA* sequence and are highly similar to sections of the *clincRNA LOC105372935* sequence: 2264 bp of the *POM121L10P* sequence are homologous to *LOC105372935* (*clincRNA*) and an adjacent 6498 bp encoding *BCRP3* are also homologous to the clincRNA sequence with 96% identity. *BCRP3* is one of the eight *BCRP* family of pseudogenes that contain sequences from the breakpoint cluster region (*BCR*) gene. The *BCRP* pseudogenes are complex. Part of the *BCR* gene is in LCR22F. Of the eight *BCRP* family genes only one, *BCRP8* resides within the *BCR* gene sequence and thus stems from the *BCR* gene locus. Functions of *BCRP3* are needed to understand the relationship of this pseudogene to *BCR*.

GGT1 is a well-characterized enzyme. Over two decades ago it was pointed out that there are several human genes for *GGT* that produce different mRNAs but that *GGT1* produces an active gamma-glutamyltransferase enzyme [25]. This was confirmed by Heisterkamp et al. [17].

### POM121L9P(BCRP1)-GGTLC4P-GGT5

The *POM121L9P(BCRP1)*-*GTLC4P-GGT5* segment resides in LCR22G (Table 1). The *BCRP1* gene is situated entirely within the *POM121L9P* sequence and is an antisense gene. However, *GGT5* is an anomaly. Although gene positions relative to each other in chr22 are *POM121L9P(BCRP1)*-*GTLC4P-GGT5*, there is no evidence that *GGT5* originates from a *GGT* locus, however data point to the origin from a clincRNA sequence.

An alignment of the *POM121L9P (BCRP1)*-*GGTLC4P-GGT5* sequence with that of *FAM230B*-*LOC105372935*-*GGT2* shows that *POM121L9P (BCRP1)*-*GGTLC4P-GGT5* contains spacer and *GGT* sequences, and most of the *clincRNA* sequence. *POM121L9P (BCRP1)* carries sequences 5949 to 8219 of the clincRNA *LOC105372935* sequence. Of significance, a sequence alignment of the *GGT5* sequence with that of the *clincRNA LOC105372935* shows that *GGT5* contains part of the *clincRNA* sequence (positions 1–5958) but carries no *GGT* sequences (Table 3 and S5 Fig). *GGT5* is 25489 bp in length and carries 5958 bp that are homologous to the *LOC105372935* clincRNA sequence. Thus ~23% of *GGT5* contains clincRNA sequences with an identity of 90–92% (Table 3). It has been pointed out before that there is little nt sequence homology between the *GGT5* and *GGT1* genes [17].

**Table 3:** % Identity *GGT5* and *GGTLC4P* with *GGT2* and *clincRNA* gene *LOC105372935*.

Gene	bp length	# bps identity with *GGT2*	% identity with *GGT2*	# bps identity with *clincRNA* *LOC105372935*	% identity with *clincRNA* *LOC105372935*
*GGT5*	25489	0	0	5958	90–92
*GGTLC4P*	1553	1553	96	0	0

Table 3 shows the close similarity of pseudogene *GGTLC4P* with *GGT2* sequences, where the entire sequence of *GGTLC4P* consists of *GGT2* sequences. *GGTLC4P* also displays a high identity with *GGT1* (not shown).

Although there is no significant nt sequence homology between *GGT5* and *GGT2*, amino acid sequences of the protein products have similarities where approximately half of the amino acid residues are identical [17]. In addition, the GGT5 protein displays gamma-glutamyltransferase activity [17].

**Fig 7:**
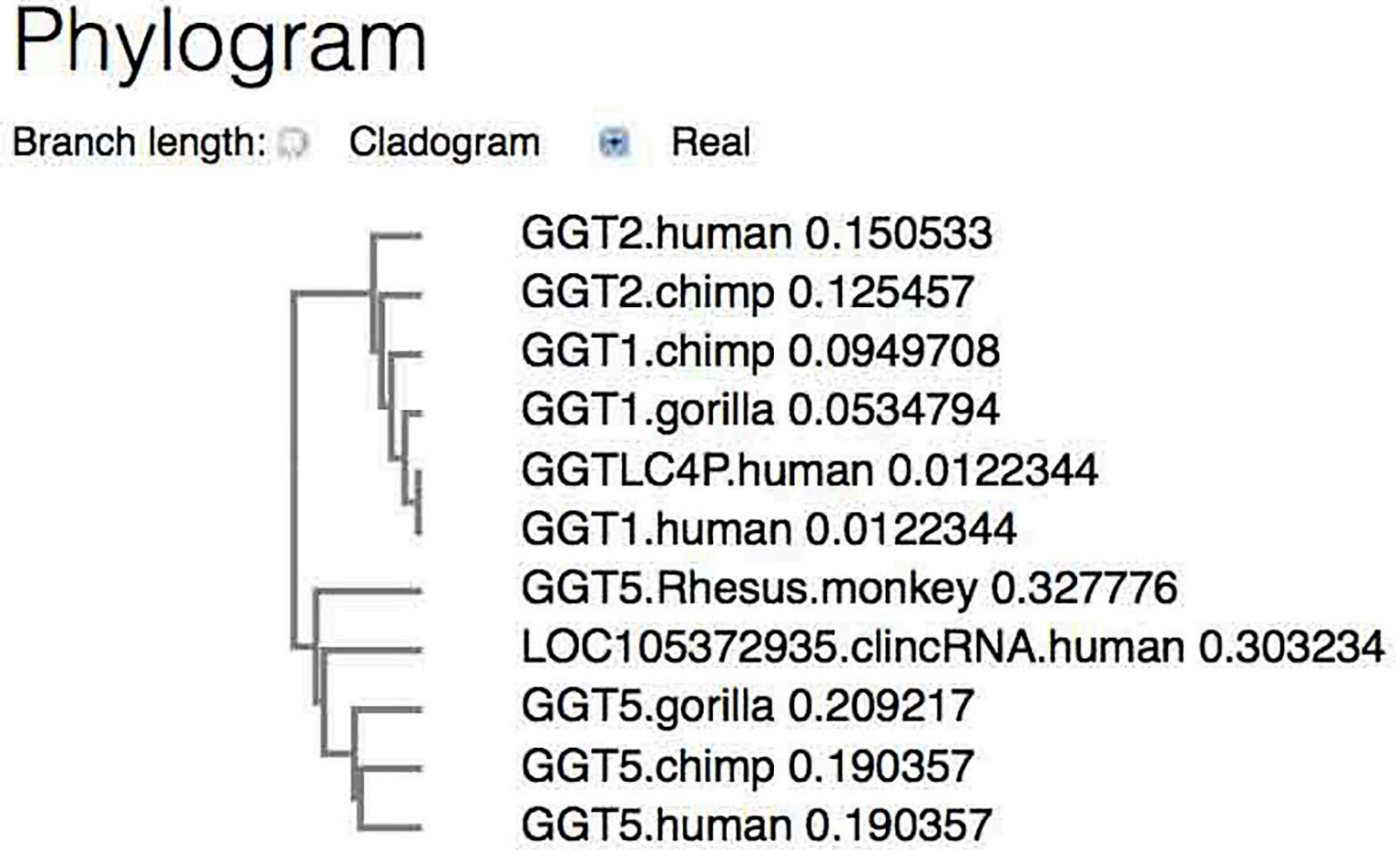
Phylogram shows the phylogenetic branch relationships between *GGT* genes and the *LOC105372935 lincRNA* gene. The gorilla *GGT1* and *GGT5* sequences were from Gorilla gorilla (western gorilla) chromosome 22, gorGor4, NCBI Reference Sequence: NC_018446.2, locus: NC_018446 [20]. The Rheus monkey *GGT5* sequence (annotated as *LOC720345*) was from the NCBI sequence of *Macaca mulatta* isolate AG07107 chromosome 10, Mmul_10, whole genome shotgun sequence, ACCESSION: NC_041763, REGION: complement 28391649..28419652). The phylogram was obtained using the EBI Clustal Omega sequence alignment and phylogeny programs.

The chimpanzee *GGT5* nt sequence is also present in the chimpanzee genome and it is found to be highly similar to the human sequence (with 98% identity over 90% of the human *GGT5* sequence). In addition, the chimpanzee gene also contains the clincRNA signature sequence. A phylogenetic analysis was performed with aligned *GGT* nt sequences that generated a phylogram tree. Included also are *GGT* sequences from the gorilla and Rhesus monkey, as these sequences are available from the NCBI (Fig 7). The tree shows that all *GGT5* genes and the human *clincRNA LOC105372935* gene form a branch that is separate from the branch grouping of *GGT1*, *GGT2*, and *GGTLC* (Fig 7). The close association of the various primate *GGT5* genes with the human clincRNA gene *LOC105372935* sequence is consistent with and adds to the data of Table 3. Because of the close similarities between the human and chimpanzee *GGT5* gene sequences as well as with the other two primate *GGT5* genes, *GGT5* appears to have originated in a common primate ancestor.

### FAM230C-LOC101060145-GGT4P

The *FAM230C-LOC101060145-GGT4P* linked gene locus is in chr13, which distinguishes it from the other *FAM230* family genes that are all in chr 22. What stands out in the *FAM230C-LOC101060145-GGT4P* linked gene segment is that no genes stem from the clincRNA sequence and that two pseudogenes, *LOC101060145* and *GGT4P* originate from the *GGT* sequence (S6 Fig). *LOC101060145* is annotated as a glutathione hydrolase light chain 1-like pseudogene by NCBI and *GGT4P* is a gamma-glutamyltransferase 4 pseudogene annotated by Ensembl. S6 Fig also shows that the *FAM230C*-*LOC101060145*-*GGT4P* linked gene sequence has a high identity with the *FAM230B-LOC105372935-GGT2* sequence. This shows the presence of the *FAM230*-*clincRNA*-spacer-*GGT* repeat sequence outside of chr22.

### *FAM230D-USP18* linked gene sequences

In addition to the *GGT*-linked genes, there is another example of the human *FAM230*-*lincRNA* sequence that forms genes, sequences that are linked to the *USP* protein genes; USP is an ubiquitin specific peptidase.

**Fig 8:**
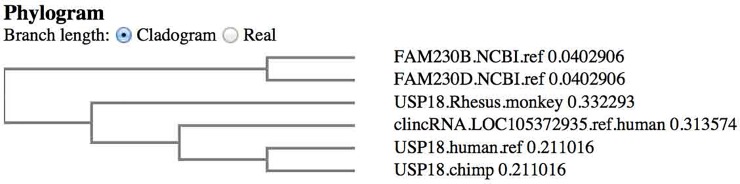
A phylogram determined from an alignment of primate *USP18* sequences with human *clincRNA LOC105372935* and two *FAM230* sequences. The phylogram was obtained using the EBI Clustal Omega sequence alignment and phylogeny programs.

The *USP18*-linked *FAM230D* sequence does not carry the uncharacterized spacer; the *FAM230D* gene is directly linked to *USP18*. Of major significance, a nt sequence alignment of the *USP18* gene sequence with the clincRNA DNA sequence shows that part of the clincRNA sequence forms part of the *USP18* gene (3174 bp of the clincRNA sequence is present in the *USP18* gene with 97% identity); the presence of the clincRNA sequence is also found in the *USP18* genes of other primates. Fig 8 shows the segregation of the clincRNA sequence with the primate *USP18* genes. The phylogram is similar to the *GGT5* gene findings, where the *USP18* protein genes cluster with the clincRNA sequence. But unlike the *GGT5* gene that does not contain *GGT* sequences, *USP18* and the related *USP41* share some sequences.

With respect to the *USP18* mRNA, the entire exon 11 consists of the clincRNA sequence. Exon 11 is the last and largest exon (626 nt) in the *USP18* mRNA and it has an identity of 99% compared with the clincRNA sequence. A section of a translated aa sequence of clincRNA shows an oligo-peptide sequence of QETAYLLVYMKMEC; this is the identical sequence of the last 14 aa of the *USP18* peptide chain. Thus the carboxy terminal amino acid sequence of the protein appears to have been formed from the clincRNA sequence. This suggests that information carried in the clincRNA nt sequence is used to partly form the USP18 protease. This further supports the concept that a sequence homologous to human *clincRNA LOC105372935* may participate in forming protein genes at chromosomal loci where the sequence is duplicated.

USP41 is termed ubiquitin specific peptidase 41, and the *USP41* gene is found linked to *FAM230G* in chr22. The *FAM230G-USP41* segment resides is in LCR22B. There are major changes in the *USP41*-linked sequence compared to the model *FAM230*-*clincRNA*-spacer segment of the conserved repeat depicted in Fig 1. In humans, *FAM230G* is linked directly to the *USP41* sequence without clincRNA or spacer sequences and the *FAM230G* gene lacks the 3’ half sequence of *FAM230B*.

*USP41* does not carry the clincRNA sequence. *USP18* and *USP41* share a 14170 bp sequence, but this is outside of the *USP18* sequence that contains the clincRNA sequence and there is no overlap.

## Discussion

The proposed ancestral proto-gene forming element is based on the findings that *GGT*, *USP*, and the three distinct linked DNA sequences, FAM230, clincRNA and spacer are conserved between humans, chimpanzee and other primates, and that different genes have formed from these sequences. The clincRNA sequence appears to have been the focal point in the development of human and non-human primate *GGT5* and *USP18* protein genes and formation of these genes occurred in a common primate ancestor. The informational content of the clincRNA DNA element appears to be such that it can serve as a foundation for development of either lncRNA or protein genes. The FAM230 DNA sequence is not as clear. Although FAM230 sequences do carry large open reading frames, for example, the nonsense mediated decay transcript from *FAM230A* (21) that translates to a 454 aa sequence, no human protein has yet been found to contain an FAM230 open reading frame, although several uncharacterized proteins such as unnamed protein product, GenBank: BAG63817.1 do carry the FAM230A nt sequence and the FAM230A open reading frame.

In humans, the FAM230-clincRNA-spacer-GGT sequence has been repeatedly duplicated in the genome by chromosomal expansion through segmental duplications where the FAM230-clincRNA sequences are found to form lincRNA gene and pseudogene families. The concept of segmental duplications as vehicles for the proliferation of *GGT*- and *USP*-related repeat sequences concerned with the development of new genes parallels the findings of the effects of human chromosomal expansion, which consists primarily of repeat sequences, on the evolution and development of gene regulation [18]. In addition, there is a parallel of the proposed ancestral proto-gene forming element described here with that of enhancer regulatory elements that have developed from ancestral sequences or proto-enhancers [26]. These studies point to the role of ancestral sequences in the evolution of regulatory elements, and in the work here, that of gene development.

The study here adds another aspect to the work of others that suggests a number of lncRNA genes originated from protein genes [2–5]. For example, Talyan *et al*. [5] showed that RNA and protein genes share partial open reading frames and that a number of RNA genes may have originated from protein genes. In the work presented here, some of the *GGT*-related RNA pseudogenes stem from protein *GGT* sequences, however the *BCRP* and *POM121* family pseudogenes and lincRNA genes originate from the evolutionarily conserved *clincRNA* or *FAM230* RNA sequences, and not from existing protein genes. On another scale, other lncRNAs have been shown to come from enhancer sequences [27]. Thus various studies show that lncRNAs can have very different origins. With respect to protein gene formation, open reading frames in lincRNA sequences may have the potential to provide a foundation for protein gene development, however lincRNA genes lack protein coding capacity, as was previously pointed out [28].

The clincRNA contributes to the structure of the USP18 protein. Part of the clincRNA sequence provides the entire exon 11, the last exon in the ubiquitin specific peptidase 1 (USP18) mRNA and it thus provides the carboxy terminal thirteen amino acid sequence of the protein. On the other hand, the entire putative ancestral clincRNA sequence is used to form the clincRNA family of genes. Thus the informational content of the ancestral DNA sequence is such that it can lead to the development of either lincRNA or protein genes.

There are sixteen lincRNA genes and pseudogenes found to arise from the *FAM230*-*clincRNA* sequence in humans, and in addition, the protein genes, *GGT5* and *USP18*. *GGT5* belongs to the *GGT* family of protein genes. It is a well-characterized gene whose protein product displays gamma-glutamyltransferase activity but the gene nt sequence displays no significant DNA sequence homology with other members of the *GGT* family, as shown by Heisterkamp et al [17] and the work presented here. Thus *GGT5* is an anomaly in that its DNA sequence does not stem from a *GGT* locus. Although its gene position in chr22 is: *POM121L9P(BCRP1)*-*GTLC4P-GGT5*, there is no evidence that *GGT5* contains *GGT* DNA sequences but data point to the origin from the *clincRNA* locus.

Why is *GGT5* formed from an unrelated DNA sequence and not from the *GGT* sequence itself? The primate cell may have performed its own “genetic and molecular engineering” to form a protein similar in aa sequence and function to the *GGT* family proteins but from a different genomic sequence. This does not address why the *GGT* nt sequence is not used to form the *GGT5* gene as it is for other *GGT*-related genes. Genes that are descended from an ancestral gene, share nucleotide sequences, have similar translated protein aa sequences and share similar functions are generally classified as a gene family. The *GGT5* gene offers an interesting variation to this definition.

The *clincRNA* gene family found in LCR22 A and D may have formed recently, as there is little or no difference in sequence or in RNA transcript expression in different tissues. Whether some of these genes will develop important functions or eventually disappear is not known. The *FAM230* genes show more development with differences in DNA nt sequence, specific RNA transcript structures and a differential expression of circRNAs in fetal tissues [21].

The uncharacterized spacer sequence is not found to form a part of genes. This sequence is highly conserved in homologous sequences of the linked gene loci found in LCR22A and D, and it is also present in chimpanzee counterparts and conserved to 97%. But it has totally dissipated in linked genes *POM121L1P-GGTLC2*, found in LCR22E and in *FAM230D-USP18* in LCR22A and *FAM230G-USP41* in LCR22B. Its evolutionary conservation points to a function, but perhaps a non-essential one as it has disappeared in some segmental duplications.

The formation of the *FAM230* lincRNA family, which consists of a total of eight lincRNA genes in chr22, is only partially understood. This gene family is formed from the 3’ half sequence of *FAM230C* with a remnant of the 5’ half *FAM230C* sequence found in the upstream region of seven of the genes [19, 29]. *FAM230C* resides in chr13. Six *FAM230* genes are part of the conserved repeat units described here, either with *GGT* or *USP*. The remaining three *FAM230* genes have not been found to be part of a repeat element and may have formed by a separate process. The origin of *FAM230C*, which is found in chr13 is also uncertain.

With the FAM230 sequences found linked to *GGT* and/or *USP* genes in chimpanzee and other primates, such as Rhesus monkey, gorilla or orangutan, gene annotations have not progressed enough to be able to compare genes that may stem from non-human primate FAM230 sequences with those of the human genes. Any major differences would be of interest.

## Methods

### Reference genomes for primate species

Homo sapiens chromosome 22, GRCh38.p12 Primary Assembly NCBI was the source of sequences and properties of RNA and protein genes. Pan troglodytes, isolate Yerkes chimp pedigree #C0471 (Clint) chromosome 22, Clint_PTRv2, NCBI was the source of chimpanzee sequences and protein genes. In addition, a cloned sequence from Pan troglodytes, clone rp43-41g5, complete sequence GenBank: AC099533.36 provided an additional copy of the conserved repeat sequence. Gorilla gorilla (western gorilla) chromosome 22, gorGor4, NCBI Reference Sequence: NC_018446.2, locus: NC_018446 [20] (was used to search for the presence of *GGT* genes and the conserved repeat sequence. The Rheus monkey *GGT5* sequence (annotated as *LOC720345*) was from the NCBI sequence of *Macaca mulatta* isolate AG07107 chromosome 10, Mmul_10, whole genome shotgun sequence, ACCESSION: NC_041763, REGION: complement 28391649..28419652).

### Gene properties and gene searches

NCBI and Ensembl websites: (https://www.ncbi.nlm.nih.gov/gene) [20–21] and (http://useast.ensembl.org/Homo_sapiens/Info/Index) [30,31] were used as the primary sources for gene properties. However *FAM230A* gene annotations provided a partial gene sequence as there is a 50 kbp unsequenced gap within the gene. For, sequence analysis both the *FAM230A* NMD RNA transcript sequence from NCBI and the *FAM230A* gene sequence provided by Ensembl was used. Additional databases employed for gene properties were: Gene Cards: GeneCards–the human gene database, (www.genecards.org) [32], HGNC: (Genenames.org) [33] RNAcentral: rnacentral.org/ [34]. For chimpanzee gene searches, the NCBI Reference sequence (RefSeq) database was used [20] (19). The NCBI annotation of chimpanzee protein genes are with the Gnomon-The NCBI eukaryotic gene prediction tool (https://www.ncbi.nlm.nih.gov/genome/annotation_euk/gnomon/).

### Genomic coordinates

The NCBI and Ensembl gene coordinates differ for a number of genes, especially at the 5’ ends. For uniformity, all coordinates used here were according to NCBI with the expectation of *AC023490*.*3* that has been annotated only by Ensembl. The description of linked gene segments in Table 1 is in the order of *FAM230*-clincRNA-*GGT* or pseudogenene-*GGT*. However, in the genome, the gene order for several of the repeat segments is in the reverse orientation. For consistency, all linked genes are shown in the same orientation as in Table 1.

### Blast, BLAT searches and sequence identity determinations

The Blast search engine (https://blast.ncbi.nlm.nih.gov/Blast.cgi?CMD=Web&PAGE_TYPE=BlastHome [35] and Blat search engine (http://useast.ensembl.org/Homo_sapiens/Tools/Blast?db=core) [31] were both used to find similarities is gene sequences and to initially detect gene families.

### Nucleotide and amino acid sequence alignments and identity determinations

**T**he EMBL-EBI Clustal Omega Multiple Sequence Alignment program,website: http://www.ebi.ac.uk/Tools/msa/clustalo/ was used for alignment of two or more nucleotide or amino acid sequences. This program was also used to determine phylogenetic relationships via generation of a phylogram.

The identity between two sequences was determined by the NCBI Basic Local Alignment Search Tools, blastn and blastp, align two or more sequences with the Program Selection: Optimize for Highly similar sequences (megablast) [35]. The identities represent only aligned sequences and do not including gaps sequences.

#### RNA expression

The expression of RNA from normal tissues were obtained from website: www.ncbi.nlm.nih.gov/gene/, human tissue-specific expression

(HPA) RNA-seq normal tissues [22]. The expression of circular RNAs: Tissue-specific circular RNA induction during human fetal development was obtained from website: www.ncbi.nlm.nih.gov/gene/, that presents data by Szabo et al [23], where RNA-seq was performed on 27 different human tissues with samples from 95 individuals.

### Protein properties

UniProtKB (uniprot.org/uniprot/) was the source of human protein amino acid sequences. For chimpanzee proteins, amino acid sequences and regions of the protein sequence that have predicted functional domains were from: www.ncbi.nlm.nih.gov/protein [36].

#### Availability of data on websites

Gene searches, gene properties, and gene transcript expression data:

www.ncbi.nlm.nih.gov/gene/

(http://useast.ensembl.org/Homo_sapiens/Info/Index

Additional databases for gene properties:

GeneCards–the human gene database: (www.genecards.org)

HGNC: (Genenames.org)

RNAcentral: (rnacentral.org/)

Blast and BLAT searches and sequence identity determinations:

(https://blast.ncbi.nlm.nih.gov/Blast.cgi?CMD=Web&PAGE_TYPE=BlastHome

(http://useast.ensembl.org/Homo_sapiens/Tools/Blast?db=core)

Nucleotide and amino acid sequence alignments:

The EMBL-EBI Clustal Omega Multiple Sequence Alignment program: (http://www.ebi.ac.uk/Tools/msa/clustalo/)

Protein properties:

UniProtKB (uniprot.org/uniprot/)

Predicted functional domains (www.ncbi.nlm.nih.gov/protein)

RepeatMasker analysis of nt sequences:

RepeatMasker program (www.repeatmasker.org/cgi-bin/WEBRepeatMasker)

### Addendum

After this paper was completed we became aware of an article on the multifaceted functions of the USP18 protease in the interferon response [37].

## Supporting information

S1 Fig Nt sequence alignment of two chimpanzee and four human sequences that contain the repeat core sequence. The chimp.*LOC112206744-LOC107973052-GGT2*.revcompl sequence was from Pan troglodytes isolate Yerkes chimp pedigree #C0471 (Clint) chromosome 22, Clint_PTRv2. The source of the other chimpanzee sequence is from a clone and is as shown below. The human sequences were from Homo sapiens chromosome 22, GRCh38.p12 Primary Assembly NCBI Reference Sequence: NC_000022 with the human *FAM230A* sequence from the NCBI NMD transcript. The Clustal Omega, Multiple sequence alignment program was used for sequence alignment.
(PDF)

S2 Fig Phylogram of four human FAM230-linked gene sequences and chimpanzee homolog. The four human sequences are from homo sapiens chromosome 22, GRDh38.p12 Primary Assembly NCBI reference Sequence: NC_000022 with the human *FAM230A* sequence from the NCBI NMD transcript. The Clustal Omega, Multiple sequence alignment program was used for sequence alignment and generation of the phylogram.
(PNG)

S3 Fig Nt sequence alignment of chimpanzee *LOC112206744-LOC107973052-GGT2* with human *FAM230B-LOC105372935-GGT2*. The Clustal Omega, Multiple sequence alignment program was used for sequence alignment
(PDF)

S4 Fig Circular RNA expression from *FAM230E* and *FAM230B* during human fetal development. The data are from Szabo et al [23] as shown on the NCBI websites for these genes.
(PDF)

S5 Fig Alignment of human GGT5 gene sequence with human LOC105372935 clincRNA gene sequence. The Clustal Omega, Multiple sequence alignment program was used for sequence alignment.
(PDF)

S6 Fig *FAM230B-LOC105372935-GGT2* sequence found in *FAM230C*-*LOC101060145-GGT4P*. **a.** Color highlighted sections represent the *FAM230B-LOC105372935* -*GGT2* sequences that are found in *FAM230C*-*LOC101060145-GGT4P* with the respective percent identities. xxx represents sequences from the clincRNA region (*LOC105372935*) of *FAM230BLOC105372935*-*GGT2* that are missing in FAM230C-*LOC101060145-GGT4P*. The unhighlighted section, |——| represents the 5’ half sequence of *FAM230C* that does not form part of *FAM230B*. **b.** Schematic of *FAM230B-LOC105372935-spacer*-*GGT2* for comparisons. The % identities shown are relative to the *FAM230B-LOC105372935* -*GGT2* sequence.
(PDF)
