# Supplementary material for: Formation of human long intergenic non-coding RNA genes, pseudogenes, and protein genes: Ancestral sequences are key players
Source: PLoS One. 2020 Mar 26;15(3):e0230236. doi: 10.1371/journal.pone.0230236 (PMC7098633; doi:10.1371/journal.pone.0230236)
Supplement: S1 Fig — The chimp.LOC112206744-LOC107973052-GGT2.revcompl sequence was from Pan troglodytes isolate Yerkes chimp pedigree #C0471 (Clint) chromosome 22, Clint_PTRv2. The source of the other chimpanzee sequence is from a clone and is as shown below. The human sequences were from Homo sapiens chromosome 22, GRCh38.p12 Primary Assembly NCBI Reference Sequence: NC_000022 with the human FAM230A sequence from the NCBI NMD transcript. The Clustal Omega, Multiple sequence alignment program was used for sequence alignment. (PDF) [file pone.0230236.s001.pdf]

Formation of human long intergenic non-coding RNA genes and pseudogenes: ancestral sequences are key players

Nicholas Delihas

S1 Fig. Nt sequence alignment of two chimpanzee and four human sequences that contain the repeat core sequence. The chimp.LOC112206744-LOC107973052-GGT2.revcompl sequence was from Pan troglodytes isolate Yerkes chimp pedigree #C0471 (Clint) chromosome 22, Clint_PTRv2. The source of the other chimpanzee sequence is from a clone and is as shown below. The human sequences were from Homo sapiens chromosome 22, GRCh38.p12 Primary Assembly NCBI Reference Sequence: NC_000022 with the human *FAM230A* sequence from the NCBI NMD transcript. The Clustal Omega, Multiple sequence alignment program was used for sequence alignment.

```
CLUSTAL O(1.2.4) multiple sequence alignment


chimp.LOC112206744-LOC107973052-GGT2.revcompl                                     ------------------------------------------------------------ 0
chimp54546-135457.revcompl.Pan.troglodytes.clone.rp43-41g5.GenBank:AC099533.36    AGCAGTTGGACCTCACAGTGTGGATTGTGCCTTCACCCTGGAATGTTTATGCCCTATCGC 60
human.FAM30B-LOC105372935-GGT2.NCBI.                                              agcagttggacctcacagtgtggattgtgccttcaccctggaatgtttatgccctatcac 60
human.FAM230E-LOC105377182-GGT3P.NCBI.                                            ------------------------------------------------------------ 0
human.FAM230J-LOC105372942-GGTLC5P.NCBI.rev.compl                                 ------------------------------------------------------------ 0
human.FAM230A.ncRNA.18487127.18500594-GGTLC3.18516335.18518165.rev.compl.REF      ------------------------------------------------------------ 0

chimp.LOC112206744-LOC107973052-GGT2.revcompl                                     ------------------------------------------------------------ 0
chimp54546-135457.revcompl.Pan.troglodytes.clone.rp43-41g5.GenBank:AC099533.36    CATGGTGATGGGATTAGGGATCTCCTGCCCTTGGTCCTAAGTGCCACTGTCTGTGCTGAG 120
human.FAM30B-LOC105372935-GGT2.NCBI.                                              catggtgatgggattagggatctcctgcccttggtcctaagtgccactatctgtgctgag 120
human.FAM230E-LOC105377182-GGT3P.NCBI.                                            ------------------------------------------------------------ 0
human.FAM230J-LOC105372942-GGTLC5P.NCBI.rev.compl                                 ------------------------------------------------------------ 0
human.FAM230A.ncRNA.18487127.18500594-GGTLC3.18516335.18518165.rev.compl.REF      ------------------------------------------------------------ 0

chimp.LOC112206744-LOC107973052-GGT2.revcompl                                     ------------------------------------------------------------ 0
chimp54546-135457.revcompl.Pan.troglodytes.clone.rp43-41g5.GenBank:AC099533.36    TTTTTCAAAGGTCAGAGCAGATTGAACCTCTGTGGTTTCATTTTCCCTGATTTTGATTTT 180
human.FAM30B-LOC105372935-GGT2.NCBI.                                              tttttcaaaggtcagagcagattgaaccattgtggtttcattttccctgattttgatttt 180
human.FAM230E-LOC105377182-GGT3P.NCBI.                                            ------------------------------------------------------------ 0
human.FAM230J-LOC105372942-GGTLC5P.NCBI.rev.compl                                 ------------------------------------------------------------ 0
human.FAM230A.ncRNA.18487127.18500594-GGTLC3.18516335.18518165.rev.compl.REF      ------------------------------------------------------------ 0

chimp.LOC112206744-LOC107973052-GGT2.revcompl                                     ----atggggaacctgtgtggctgcattcaaggtatgttcatactggcctgtcaaatgcg 56
chimp54546-135457.revcompl.Pan.troglodytes.clone.rp43-41g5.GenBank:AC099533.36    TCTTATGGGGAACCTGTGTGGCTGCATTCAAGGTATGTTCATACTGGCCTGTCAAATGCG 240
human.FAM30B-LOC105372935-GGT2.NCBI.                                              tcttatggggaacctgtgtggctgcattcaaggtatgttcatactggcctgtcaaatgcg 240
human.FAM230E-LOC105377182-GGT3P.NCBI.                                            ------------------------------------------------------------ 0
human.FAM230J-LOC105372942-GGTLC5P.NCBI.rev.compl                                 ------------------------------------------------------------ 0
human.FAM230A.ncRNA.18487127.18500594-GGTLC3.18516335.18518165.rev.compl.REF      ------------------------------------------------------------ 0

chimp.LOC112206744-LOC107973052-GGT2.revcompl                                     atcttttcaaattactagttaatgctttcaaaatatgttatttaaaaaattatcctctgt 116
chimp54546-135457.revcompl.Pan.troglodytes.clone.rp43-41g5.GenBank:AC099533.36    ATCTTTTCAAATTACTAGTTAATGCTTTCAAAATATGTTATTTAAAAAATTATCCTCTGT 300
human.FAM30B-LOC105372935-GGT2.NCBI.                                              atcttttcaaattactagttaatgctttcaaaatatgttatttaaaaaattagcctctgt 300
human.FAM230E-LOC105377182-GGT3P.NCBI.                                            ------------------------------------------------------------ 0
human.FAM230J-LOC105372942-GGTLC5P.NCBI.rev.compl                                 ------------------------------------------------------------ 0
human.FAM230A.ncRNA.18487127.18500594-GGTLC3.18516335.18518165.rev.compl.REF      ------------------------------------------------------------ 0

chimp.LOC112206744-LOC107973052-GGT2.revcompl                                     attttccatatggagttataaatatgtttcatggttatgttttattcctcaatttatata 176
chimp54546-135457.revcompl.Pan.troglodytes.clone.rp43-41g5.GenBank:AC099533.36    ATTTTCCATATGCAGTTATAAATATGTTTCATGGTTATGTTTTATTCCTCAATTTATAGA 360
human.FAM30B-LOC105372935-GGT2.NCBI.                                              attttccatatgcagttataaatatgtttcatgattatgttttattcctcaatttatata 360
human.FAM230E-LOC105377182-GGT3P.NCBI.                                            ------------------------------------------------------------ 0
human.FAM230J-LOC105372942-GGTLC5P.NCBI.rev.compl                                 ------------------------------------------------------------ 0
human.FAM230A.ncRNA.18487127.18500594-GGTLC3.18516335.18518165.rev.compl.REF      ------------------------------------------------------------ 0

chimp.LOC112206744-LOC107973052-GGT2.revcompl                                     tttgattattgtaccaagcagagtacctttgaaatttttcttcatttaaaaaatatgtct 236
chimp54546-135457.revcompl.Pan.troglodytes.clone.rp43-41g5.GenBank:AC099533.36    TTTGATTATTGTACCAAGTAGAGTACCTTTGAAATTTTTCTTCATTTAAAAAATATGTAT 420
human.FAM30B-LOC105372935-GGT2.NCBI.                                              tttgattattgtaccaagcagagtatctttgaaatttttcttcatttaaaaaatatgtat 420
human.FAM230E-LOC105377182-GGT3P.NCBI.                                            ------------------------------------------------------------ 0
human.FAM230J-LOC105372942-GGTLC5P.NCBI.rev.compl                                 ------------------------------------------------------------ 0
human.FAM230A.ncRNA.18487127.18500594-GGTLC3.18516335.18518165.rev.compl.REF      ------------------------------------------------------------ 0

chimp.LOC112206744-LOC107973052-GGT2.revcompl                                     cttggctcaggcctgtaatcccagcactttgggaggccaatgcaagaggatcacaaggtg 296
chimp54546-135457.revcompl.Pan.troglodytes.clone.rp43-41g5.GenBank:AC099533.36    CTTGGCTCAGGCCTGTAATCCCAGCACTTTGGGAGGCCAAGGCAAGAGGATCACAAGGTG 480
human.FAM30B-LOC105372935-GGT2.NCBI.                                              cttgactcaggcctgtaatcccagcactttgggaggccaaggcaagaggatcacaaggtg 480
human.FAM230E-LOC105377182-GGT3P.NCBI.                                            ------------------------------------------------------------ 0
human.FAM230J-LOC105372942-GGTLC5P.NCBI.rev.compl                                 ------------------------------------------------------------ 0
human.FAM230A.ncRNA.18487127.18500594-GGTLC3.18516335.18518165.rev.compl.REF      ------------------------------------------------------------ 0

chimp.LOC112206744-LOC107973052-GGT2.revcompl                                     aggagatcaagaccatcctggtcaatacagtgaaaccctgtctgtacgaaaagtacaaaa 356
chimp54546-135457.revcompl.Pan.troglodytes.clone.rp43-41g5.GenBank:AC099533.36    AGGAGATCAAGACCATCCTGCCCAATACAGTAAAACCCTGTCTCTATCAAAAATACAAAA 540
human.FAM30B-LOC105372935-GGT2.NCBI.                                              aggagatcaagaccatcctggccaatacagtgaaaccctgtctctactacaaatacaaaa 540
human.FAM230E-LOC105377182-GGT3P.NCBI.                                            ------------------------------------------------------------ 0
human.FAM230J-LOC105372942-GGTLC5P.NCBI.rev.compl                                 ------------------------------------------------------------ 0
human.FAM230A.ncRNA.18487127.18500594-GGTLC3.18516335.18518165.rev.compl.REF      ------------------------------------------------------------ 0

chimp.LOC112206744-LOC107973052-GGT2.revcompl                                     aattagccaggaatggtggcagctggtgtagtcccagtgtgaattgg------------- 403
chimp54546-135457.revcompl.Pan.troglodytes.clone.rp43-41g5.GenBank:AC099533.36    AATTAGCCTGGCATGGTGGCAGCTGCTGTAGTCACAGTGTGGTGTAGTCCCAGCTACCTG 600
human.FAM30B-LOC105372935-GGT2.NCBI.                                              atttagccaggcatggtggcagctggtgtagtcccagtgtgaattgg------------- 587
human.FAM230E-LOC105377182-GGT3P.NCBI.                                            ------------------------------------------------------------ 0
human.FAM230J-LOC105372942-GGTLC5P.NCBI.rev.compl                                 ------------------------------------------------------------ 0
human.FAM230A.ncRNA.18487127.18500594-GGTLC3.18516335.18518165.rev.compl.REF      ------------------------------------------------------------ 0

chimp.LOC112206744-LOC107973052-GGT2.revcompl                                     ------------------------------------------------------------ 403
chimp54546-135457.revcompl.Pan.troglodytes.clone.rp43-41g5.GenBank:AC099533.36    GGAGGCTGAGGCAGGACAATCGCTTGAACCCGTGAGGCAGAGGTTGCAGTGAGCCAAGAT 660
human.FAM30B-LOC105372935-GGT2.NCBI.                                              ------------------------------------------------------------ 587
human.FAM230E-LOC105377182-GGT3P.NCBI.                                            ------------------------------------------------------------ 0
human.FAM230J-LOC105372942-GGTLC5P.NCBI.rev.compl                                 ------------------------------------------------------------ 0
human.FAM230A.ncRNA.18487127.18500594-GGTLC3.18516335.18518165.rev.compl.REF      ------------------------------------------------------------ 0

chimp.LOC112206744-LOC107973052-GGT2.revcompl                                     -------------------------------gattcagtttattcccaaattcccaaatt 432
chimp54546-135457.revcompl.Pan.troglodytes.clone.rp43-41g5.GenBank:AC099533.36    GGCGCCATTGCACTCCAGCCTGTGCAACAGAACAAGACTCTGTTAAAAAAAAAAAAACAT 720
human.FAM30B-LOC105372935-GGT2.NCBI.                                              ------------------------------------------------------------ 587
human.FAM230E-LOC105377182-GGT3P.NCBI.                                            ------------------------------------------------------------ 0
human.FAM230J-LOC105372942-GGTLC5P.NCBI.rev.compl                                 ------------------------------------------------------------ 0
human.FAM230A.ncRNA.18487127.18500594-GGTLC3.18516335.18518165.rev.compl.REF      ------------------------------------------------------------ 0

chimp.LOC112206744-LOC107973052-GGT2.revcompl                                     atatatattatatatataatatattatatatacagtatatataatatattatatatacag 492
chimp54546-135457.revcompl.Pan.troglodytes.clone.rp43-41g5.GenBank:AC099533.36    ATATATATAATGTATATTATATATATTATATAT------TATAGAATATATGATATACAA 774
human.FAM30B-LOC105372935-GGT2.NCBI.                                              ------------------------------------------------------------ 587
human.FAM230E-LOC105377182-GGT3P.NCBI.                                            ------------------------------------------------------------ 0
human.FAM230J-LOC105372942-GGTLC5P.NCBI.rev.compl                                 ------------------------------------------------------------ 0
human.FAM230A.ncRNA.18487127.18500594-GGTLC3.18516335.18518165.rev.compl.REF      ------------------------------------------------------------ 0

chimp.LOC112206744-LOC107973052-GGT2.revcompl                                     tatataatatactatgtatacagtatatattatatactgtattatatac-attatatact 551
chimp54546-135457.revcompl.Pan.troglodytes.clone.rp43-41g5.GenBank:AC099533.36    TATGTAATATATAATATAT--GATACATAATATATAATATATGATATACAATTATATATC 832
human.FAM30B-LOC105372935-GGT2.NCBI.                                              ------------------------------------------------------------ 587
human.FAM230E-LOC105377182-GGT3P.NCBI.                                            ------------------------------------------------------------ 0
human.FAM230J-LOC105372942-GGTLC5P.NCBI.rev.compl                                 ------------------------------------------------------------ 0
human.FAM230A.ncRNA.18487127.18500594-GGTLC3.18516335.18518165.rev.compl.REF      ------------------------------------------------------------ 0

chimp.LOC112206744-LOC107973052-GGT2.revcompl                                     gtatacattatatactgtattatatattatatactgtatatataatatatattatatata 611
chimp54546-135457.revcompl.Pan.troglodytes.clone.rp43-41g5.GenBank:AC099533.36    ATATATATTAAATGTCACATTATATATATTATATATTATATATAATATACATAATGTATA 892
human.FAM30B-LOC105372935-GGT2.NCBI.                                              ------------------------------------------------------------ 587
human.FAM230E-LOC105377182-GGT3P.NCBI.                                            ------------------------------------------------------------ 0
human.FAM230J-LOC105372942-GGTLC5P.NCBI.rev.compl                                 ------------------------------------------------------------ 0
human.FAM230A.ncRNA.18487127.18500594-GGTLC3.18516335.18518165.rev.compl.REF      ------------------------------------------------------------ 0

chimp.LOC112206744-LOC107973052-GGT2.revcompl                                     cagtatatagtacatattatatatactatatgtta--tataaaatatataatataaaata 669
chimp54546-135457.revcompl.Pan.troglodytes.clone.rp43-41g5.GenBank:AC099533.36    ACATATATAATATATAATATATAACAAATATATTAGGTATATAATATATCTTATATGACA 952
human.FAM30B-LOC105372935-GGT2.NCBI.                                              ------------------------------------------------------------ 587
human.FAM230E-LOC105377182-GGT3P.NCBI.                                            ------------------------------------------------------------ 0
human.FAM230J-LOC105372942-GGTLC5P.NCBI.rev.compl                                 ------------------------------------------------------------ 0
human.FAM230A.ncRNA.18487127.18500594-GGTLC3.18516335.18518165.rev.compl.REF      ------------------------------------------------------------ 0

chimp.LOC112206744-LOC107973052-GGT2.revcompl                                     tataatatataatatataatatataatatataatatgtataatatataatatataatata 729
chimp54546-135457.revcompl.Pan.troglodytes.clone.rp43-41g5.GenBank:AC099533.36    TATAATATATATTATGTTATATGACATATATAATATATCTTACATATTATATATTATA-- 1010
```

```
human.FAM30B-LOC105372935-GGT2.NCBI.                                              ------------------------------------------------------------ 587
human.FAM230E-LOC105377182-GGT3P.NCBI.                                            ------------------------------------------------------------ 0
human.FAM230J-LOC105372942-GGTLC5P.NCBI.rev.compl                                 ------------------------------------------------------------ 0
human.FAM230A.ncRNA.18487127.18500594-GGTLC3.18516335.18518165.rev.compl.REF      ------------------------------------------------------------ 0

chimp.LOC112206744-LOC107973052-GGT2.revcompl                                     tagtatatatatcgtataatatatataatatatattatatataatatatattataaatta 789
chimp54546-135457.revcompl.Pan.troglodytes.clone.rp43-41g5.GenBank:AC099533.36    ------ATATATTATATATAATATATTATATATAATATATATAATATATATTATATATTT 1064
human.FAM30B-LOC105372935-GGT2.NCBI.                                              ------------------------------gattcagtttattcccaaattcccaaatta 617
human.FAM230E-LOC105377182-GGT3P.NCBI.                                            ------------------------------------------------------------ 0
human.FAM230J-LOC105372942-GGTLC5P.NCBI.rev.compl                                 ------------------------------------------------------------ 0
human.FAM230A.ncRNA.18487127.18500594-GGTLC3.18516335.18518165.rev.compl.REF      ------------------------------------------------------------ 0

chimp.LOC112206744-LOC107973052-GGT2.revcompl                                     taatttataatttatatatattatatatataataataaattataattataaattataatt 849
chimp54546-135457.revcompl.Pan.troglodytes.clone.rp43-41g5.GenBank:AC099533.36    T-ATATATATGTTATATATATAATATATAC-ATTATATATCATATATATTATATGTAATA 1122
human.FAM30B-LOC105372935-GGT2.NCBI.                                              t-atatatata-tacatataaaatacatat-ataatatattaaatatattatatatataa 674
human.FAM230E-LOC105377182-GGT3P.NCBI.                                            ------------------------------------------------------------ 0
human.FAM230J-LOC105372942-GGTLC5P.NCBI.rev.compl                                 ------------------------------------------------------------ 0
human.FAM230A.ncRNA.18487127.18500594-GGTLC3.18516335.18518165.rev.compl.REF      ------------------------------------------------------------ 0

chimp.LOC112206744-LOC107973052-GGT2.revcompl                                     tataatatatattatatataatatatattatatattataatttataatatacattattta 909
chimp54546-135457.revcompl.Pan.troglodytes.clone.rp43-41g5.GenBank:AC099533.36    TATATCATATATTACATATAATATATATCATATATTAT--------------ATATGTAT 1168
human.FAM30B-LOC105372935-GGT2.NCBI.                                              tatatattatattatatataat-------------------------------------- 696
human.FAM230E-LOC105377182-GGT3P.NCBI.                                            ------------------------------------------------------------ 0
human.FAM230J-LOC105372942-GGTLC5P.NCBI.rev.compl                                 ------------------------------------------------------------ 0
human.FAM230A.ncRNA.18487127.18500594-GGTLC3.18516335.18518165.rev.compl.REF      ------------------------------------------------------------ 0

chimp.LOC112206744-LOC107973052-GGT2.revcompl                                     tatataatatataatgtatattatatattatatatttaatatataatatgttatatataa 969
chimp54546-135457.revcompl.Pan.troglodytes.clone.rp43-41g5.GenBank:AC099533.36    TATATATTATATATCATATATATTATATTATATATATCATATATAATATAATATATATAA 1228
human.FAM30B-LOC105372935-GGT2.NCBI.                                              ------------------------------------------------------------ 696
human.FAM230E-LOC105377182-GGT3P.NCBI.                                            ------------------------------------------------------------ 0
human.FAM230J-LOC105372942-GGTLC5P.NCBI.rev.compl                                 ------------------------------------------------------------ 0
human.FAM230A.ncRNA.18487127.18500594-GGTLC3.18516335.18518165.rev.compl.REF      ------------------------------------------------------------ 0

chimp.LOC112206744-LOC107973052-GGT2.revcompl                                     tatataatatat------------------------------------------------ 981
chimp54546-135457.revcompl.Pan.troglodytes.clone.rp43-41g5.GenBank:AC099533.36    TATGTATTATATATAATTATTATACATAAGTATATATAATAATTATATATAATATAAGGA 1288
human.FAM30B-LOC105372935-GGT2.NCBI.                                              ------------------------------------------------------------ 696
human.FAM230E-LOC105377182-GGT3P.NCBI.                                            ------------------------------------------------------------ 0
human.FAM230J-LOC105372942-GGTLC5P.NCBI.rev.compl                                 ------------------------------------------------------------ 0
human.FAM230A.ncRNA.18487127.18500594-GGTLC3.18516335.18518165.rev.compl.REF      ------------------------------------------------------------ 0

chimp.LOC112206744-LOC107973052-GGT2.revcompl                                     ------------------------------------------------------------ 981
chimp54546-135457.revcompl.Pan.troglodytes.clone.rp43-41g5.GenBank:AC099533.36    TGCAGGATGTAAAAGGAAATTATATATATGTTATATATATTATATTATATATATATGTAT 1348
human.FAM30B-LOC105372935-GGT2.NCBI.                                              -------------------------------------------------tatatatatat 707
human.FAM230E-LOC105377182-GGT3P.NCBI.                                            ------------------------------------------------------------ 0
human.FAM230J-LOC105372942-GGTLC5P.NCBI.rev.compl                                 ------------------------------------------------------------ 0
human.FAM230A.ncRNA.18487127.18500594-GGTLC3.18516335.18518165.rev.compl.REF      ------------------------------------------------------------ 0

chimp.LOC112206744-LOC107973052-GGT2.revcompl                                     ------------------------------------------------------------ 981
chimp54546-135457.revcompl.Pan.troglodytes.clone.rp43-41g5.GenBank:AC099533.36    ATATTTGGGGGTGCCCTATTTCCCATCTCATAACTTATTTTAAGAAGCACAGCATAATAA 1408
human.FAM30B-LOC105372935-GGT2.NCBI.                                              atatttgtgggtgccctatttcccgtctcataacttattttaagaagc-cagcataataa 766
human.FAM230E-LOC105377182-GGT3P.NCBI.                                            ------------------------------------------------------------ 0
human.FAM230J-LOC105372942-GGTLC5P.NCBI.rev.compl                                 ------------------------------------------------------------ 0
human.FAM230A.ncRNA.18487127.18500594-GGTLC3.18516335.18518165.rev.compl.REF      ------------------------------------------------------------ 0

chimp.LOC112206744-LOC107973052-GGT2.revcompl                                     ------------------------------------------------------------ 981
chimp54546-135457.revcompl.Pan.troglodytes.clone.rp43-41g5.GenBank:AC099533.36    TGTGTGGGCTTGGGATTCAGTTTTTGAAACAAAACACTGAGCCTTCAATGACCTTCCTGT 1468
human.FAM30B-LOC105372935-GGT2.NCBI.                                              tgtgtgggcttgggattcagtttttgaaacaaaacactgagcctttgatgaccttcctgt 826
human.FAM230E-LOC105377182-GGT3P.NCBI.                                            ------------------------------------------------------------ 0
human.FAM230J-LOC105372942-GGTLC5P.NCBI.rev.compl                                 ------------------------------------------------------------ 0
human.FAM230A.ncRNA.18487127.18500594-GGTLC3.18516335.18518165.rev.compl.REF      ------------------------------------------------------------ 0

chimp.LOC112206744-LOC107973052-GGT2.revcompl                                     ------------------------------------------------------------ 981
chimp54546-135457.revcompl.Pan.troglodytes.clone.rp43-41g5.GenBank:AC099533.36    ACATGTAAAAGCACACCTGTCTGCATGGCAGCAGTTGGACCTCACAGTGTGGATTGTGCC 1528
human.FAM30B-LOC105372935-GGT2.NCBI.                                              acttgtaaaagcccacctgtctgcatggcagcagttggacctcacagtgtggattgtgcc 886
human.FAM230E-LOC105377182-GGT3P.NCBI.                                            ------------------------------------------------------------ 0
human.FAM230J-LOC105372942-GGTLC5P.NCBI.rev.compl                                 ------------------------------------------------------------ 0
human.FAM230A.ncRNA.18487127.18500594-GGTLC3.18516335.18518165.rev.compl.REF      ------------------------------------------------------------ 0

chimp.LOC112206744-LOC107973052-GGT2.revcompl                                     ------------------------------------------------------------ 981
chimp54546-135457.revcompl.Pan.troglodytes.clone.rp43-41g5.GenBank:AC099533.36    TTCACCCTGGAATGTTTATGCCCTATCGCCATGGTGATGGGATTAGGGATCTCCTGCCTT 1588
human.FAM30B-LOC105372935-GGT2.NCBI.                                              ttcaccctggaatgtttatgccctatcgccatggtgatgggattagggatctcctgccct 946
human.FAM230E-LOC105377182-GGT3P.NCBI.                                            ----------------------------------------gattagggatctcctgccct 20
human.FAM230J-LOC105372942-GGTLC5P.NCBI.rev.compl                                 ----------------------------------------gattagggatctcctgccct 20
human.FAM230A.ncRNA.18487127.18500594-GGTLC3.18516335.18518165.rev.compl.REF      ------------------------------------------------------------ 0

chimp.LOC112206744-LOC107973052-GGT2.revcompl                                     ------------------------------------------------------------ 981
chimp54546-135457.revcompl.Pan.troglodytes.clone.rp43-41g5.GenBank:AC099533.36    TGGTCCTAAGTGCCACTATCTGTGCTGAGTTTTTCAAAGGTCAGAGCAGATTGAATCTTT 1648
human.FAM30B-LOC105372935-GGT2.NCBI.                                              tggtcctaagtgccactatctgtgctgagtttttcaaaggtcagagcagattgaaccatt 1006
human.FAM230E-LOC105377182-GGT3P.NCBI.                                            tggtcctaagtgccactatctgtgctgagtttttcaaaggtcagagcagattgaaccatt 80
human.FAM230J-LOC105372942-GGTLC5P.NCBI.rev.compl                                 tggtcgtaagtgccactacctgtgctgagtttttcaaaggtcagagcagattgaaccatt 80
human.FAM230A.ncRNA.18487127.18500594-GGTLC3.18516335.18518165.rev.compl.REF      ------------------------------------------------------------ 0

chimp.LOC112206744-LOC107973052-GGT2.revcompl                                     ------------------------------------------------------------ 981
chimp54546-135457.revcompl.Pan.troglodytes.clone.rp43-41g5.GenBank:AC099533.36    GTGGTTTCATTTTCCCTGATTTTGATTTTTCTCATGGGGAACCTGTGTGGCTGCATTCAA 1708
human.FAM30B-LOC105372935-GGT2.NCBI.                                              gtggtttcattttccctgattttgatttttcttatggggaacctgtgtggctgcattcaa 1066
human.FAM230E-LOC105377182-GGT3P.NCBI.                                            gtggtttcattttccctgattttgatttttcttatggggaacctgtgtggctgcattcaa 140
human.FAM230J-LOC105372942-GGTLC5P.NCBI.rev.compl                                 gtggtttcattttccctgattttgatttttcttatggggaacctgtgtggctgcattcaa 140
human.FAM230A.ncRNA.18487127.18500594-GGTLC3.18516335.18518165.rev.compl.REF      ------------------------------------------------------------ 0

chimp.LOC112206744-LOC107973052-GGT2.revcompl                                     ------------------------------------------------------------ 981
chimp54546-135457.revcompl.Pan.troglodytes.clone.rp43-41g5.GenBank:AC099533.36    GGTATGTTCATACTGGCCTGTCAAGTGTGATCTTTTCAAATTACTAGTTAATGCTTTCAA 1768
human.FAM30B-LOC105372935-GGT2.NCBI.                                              ggtatgttcatactggcctgtcaaatgcgatcttttcaaattactagttaatgctttcaa 1126
human.FAM230E-LOC105377182-GGT3P.NCBI.                                            ggtatgttcatactggcctgtcaaatgcgatcttttcaaattactagttaatgctttcaa 200
human.FAM230J-LOC105372942-GGTLC5P.NCBI.rev.compl                                 ggtatgttcatactggcctgtcaaatgcgatcttttcaaattactagttaatgctttcaa 200
human.FAM230A.ncRNA.18487127.18500594-GGTLC3.18516335.18518165.rev.compl.REF      ------------------------------------------------------------ 0

chimp.LOC112206744-LOC107973052-GGT2.revcompl                                     ------------------------------------------------------------ 981
chimp54546-135457.revcompl.Pan.troglodytes.clone.rp43-41g5.GenBank:AC099533.36    AATATGTTATTTAAAAAATTATCCTCTGTATTTTCCATATGCAGTTATAAATATGTTTCA 1828
human.FAM30B-LOC105372935-GGT2.NCBI.                                              aatatgttatttaaaaaattagcctctgtattttccatatgcagttataaatatgtttca 1186
human.FAM230E-LOC105377182-GGT3P.NCBI.                                            aatatgttatttaaaaaattagcctctgtattttccatatgcagttataaatatgtttca 260
human.FAM230J-LOC105372942-GGTLC5P.NCBI.rev.compl                                 aatatgttatttaaaaaattagcctctgtattttccatatgcagttataaatatgtttca 260
human.FAM230A.ncRNA.18487127.18500594-GGTLC3.18516335.18518165.rev.compl.REF      ------------------------------------------------------------ 0

chimp.LOC112206744-LOC107973052-GGT2.revcompl                                     ------------------------------------------------------------ 981
chimp54546-135457.revcompl.Pan.troglodytes.clone.rp43-41g5.GenBank:AC099533.36    TGGTTATGTTTTATTCCTCAATTTATAGATTTGATTATTGTACCAAGCAGAGTACCTTTG 1888
human.FAM30B-LOC105372935-GGT2.NCBI.                                              tgattatgttttattcctcaatttatatatttgattattgtaccaagcagagtatctttg 1246
human.FAM230E-LOC105377182-GGT3P.NCBI.                                            tgattatgttttattcctcaatttatatatttgattattgtaccaagcagagtatctttg 320
human.FAM230J-LOC105372942-GGTLC5P.NCBI.rev.compl                                 tgattatgttttattcctcaatttatatatttgattattgtaccaagcagagtatctttc 320
human.FAM230A.ncRNA.18487127.18500594-GGTLC3.18516335.18518165.rev.compl.REF      ------------------------------------------------------------ 0

chimp.LOC112206744-LOC107973052-GGT2.revcompl                                     ------------------------------------------------------------ 981
chimp54546-135457.revcompl.Pan.troglodytes.clone.rp43-41g5.GenBank:AC099533.36    AAATTTTTCTTCATTTAAAAAATATGTATCTTAACTCAGGCCTGTAATCCCAGCACATTG 1948
human.FAM30B-LOC105372935-GGT2.NCBI.                                              aaatttttcttcatttaaaaaatatgtatcttgactcaggcctgtaatcccagcactttg 1306
human.FAM230E-LOC105377182-GGT3P.NCBI.                                            aaatttttcttcatttaaaaaatatgtatcttgactcaggcctgtaatcccagcactttg 380
human.FAM230J-LOC105372942-GGTLC5P.NCBI.rev.compl                                 aaatttttcttcatttaaaaaatatgtatcttgactcaggcctataatcccagcactttg 380
human.FAM230A.ncRNA.18487127.18500594-GGTLC3.18516335.18518165.rev.compl.REF      ------------------------------------------------------------ 0

chimp.LOC112206744-LOC107973052-GGT2.revcompl                                     ------------------------------------------------------------ 981
chimp54546-135457.revcompl.Pan.troglodytes.clone.rp43-41g5.GenBank:AC099533.36    GGAGTCCAAGGCAAGAGGATCACAAGGTGAGGAGATCAAGACCATCCTGCCCAATACAGT 2008
human.FAM30B-LOC105372935-GGT2.NCBI.                                              ggaggccaaggcaagaggatcacaaggtgaggagatcaagaccatcctggccaatacagt 1366
human.FAM230E-LOC105377182-GGT3P.NCBI.                                            ggaggccaaggcaagaggatcacaaggtgaggagatcaagaccatcctggccattacagt 440
human.FAM230J-LOC105372942-GGTLC5P.NCBI.rev.compl                                 ggaggccaaggcaagaggatcacaaggtgaggagatcaagaccatcctggccaatacagt 440
human.FAM230A.ncRNA.18487127.18500594-GGTLC3.18516335.18518165.rev.compl.REF      ------------------------------------------------------------ 0

chimp.LOC112206744-LOC107973052-GGT2.revcompl                                     ------------------------------------------------------------ 981
chimp54546-135457.revcompl.Pan.troglodytes.clone.rp43-41g5.GenBank:AC099533.36    GAAACCCTGTCTCTACGAAAAATACAAAAAATTAGCCAGGCATGGTGGCAGCTGGTGTAG 2068
human.FAM30B-LOC105372935-GGT2.NCBI.                                              gaaaccctgtctctactacaaatacaaaaaattagccaggcatggtggcagctggtgtag 1426
```

```
human.FAM230E-LOC105377182-GGT3P.NCBI.                                            gaaaccctgtctctactacaaatacaaaaaattagccaggcatggtggcagctggtgtag 500
human.FAM230J-LOC105372942-GGTLC5P.NCBI.rev.compl                                 gaaaccctgtctctactacaaatacaaaaaattagccaggcatggtggcagctggtgtag 500
human.FAM230A.ncRNA.18487127.18500594-GGTLC3.18516335.18518165.rev.compl.REF      ------------------------------------------------------------ 0

chimp.LOC112206744-LOC107973052-GGT2.revcompl                                     ------------------------------------------------------------ 981
chimp54546-135457.revcompl.Pan.troglodytes.clone.rp43-41g5.GenBank:AC099533.36    TCCCAATGTGAATCGGG---------ATTCAGTTTATTCCCAAATTCCCAAAT------- 2112
human.FAM30B-LOC105372935-GGT2.NCBI.                                              tcccagtgtgaattgggattcagtttattcagtttattcccaaattcccaaat------- 1479
human.FAM230E-LOC105377182-GGT3P.NCBI.                                            tcccagtgtgaattgggattcagtttattcagtttattcccaaattccaaattatatat- 559
human.FAM230J-LOC105372942-GGTLC5P.NCBI.rev.compl                                 tcccagtgtgaattgggattcagtttattc--------ccaaattcccaaattatatata 552
human.FAM230A.ncRNA.18487127.18500594-GGTLC3.18516335.18518165.rev.compl.REF      ------------------------------------------------------------ 0

chimp.LOC112206744-LOC107973052-GGT2.revcompl                                     ------------------------------------------------------------ 981
chimp54546-135457.revcompl.Pan.troglodytes.clone.rp43-41g5.GenBank:AC099533.36    ------------------------------------------------------------ 2112
human.FAM30B-LOC105372935-GGT2.NCBI.                                              ------------------------------------------------------------ 1479
human.FAM230E-LOC105377182-GGT3P.NCBI.                                            ------------------------------------------------------------ 559
human.FAM230J-LOC105372942-GGTLC5P.NCBI.rev.compl                                 tatattatatatatatatataaaaaatatatatatatataaaatatatatatataatgtatt 612
human.FAM230A.ncRNA.18487127.18500594-GGTLC3.18516335.18518165.rev.compl.REF      ------------------------------------------------------------ 0

chimp.LOC112206744-LOC107973052-GGT2.revcompl                                     ------------------------------------------------------------ 981
chimp54546-135457.revcompl.Pan.troglodytes.clone.rp43-41g5.GenBank:AC099533.36    ------------------------------------------------------------ 2112
human.FAM30B-LOC105372935-GGT2.NCBI.                                              ------------------------------------------------------------ 1479
human.FAM230E-LOC105377182-GGT3P.NCBI.                                            ------------------------------------------------------------ 559
human.FAM230J-LOC105372942-GGTLC5P.NCBI.rev.compl                                 taatatattatatataatatattatatatattatatataatatattatatatataatata 672
human.FAM230A.ncRNA.18487127.18500594-GGTLC3.18516335.18518165.rev.compl.REF      ------------------------------------------------------------ 0

chimp.LOC112206744-LOC107973052-GGT2.revcompl                                     ------------------------------------------------------------ 981
chimp54546-135457.revcompl.Pan.troglodytes.clone.rp43-41g5.GenBank:AC099533.36    ------------------------------------------------------------ 2112
human.FAM30B-LOC105372935-GGT2.NCBI.                                              ------------------------------------------------------------ 1479
human.FAM230E-LOC105377182-GGT3P.NCBI.                                            ------------------------------------------------------------ 559
human.FAM230J-LOC105372942-GGTLC5P.NCBI.rev.compl                                 tataatatattaaatataatatataatatatataatatattatatatattatagataata 732
human.FAM230A.ncRNA.18487127.18500594-GGTLC3.18516335.18518165.rev.compl.REF      ------------------------------------------------------------ 0

chimp.LOC112206744-LOC107973052-GGT2.revcompl                                     ------------------------------------------------------------ 981
chimp54546-135457.revcompl.Pan.troglodytes.clone.rp43-41g5.GenBank:AC099533.36    ------------------------------------------------------------ 2112
human.FAM30B-LOC105372935-GGT2.NCBI.                                              ------------------------------------------------------------ 1479
human.FAM230E-LOC105377182-GGT3P.NCBI.                                            ------------------------------------------------------------ 559
human.FAM230J-LOC105372942-GGTLC5P.NCBI.rev.compl                                 tatattatattatatataatatatattatacattatataattatatattatgtatataat 792
human.FAM230A.ncRNA.18487127.18500594-GGTLC3.18516335.18518165.rev.compl.REF      ------------------------------------------------------------ 0

chimp.LOC112206744-LOC107973052-GGT2.revcompl                                     ------------------------------------------------------------ 981
chimp54546-135457.revcompl.Pan.troglodytes.clone.rp43-41g5.GenBank:AC099533.36    ------------------------------------------------------------ 2112
human.FAM30B-LOC105372935-GGT2.NCBI.                                              ------------------------------------------------------------ 1479
human.FAM230E-LOC105377182-GGT3P.NCBI.                                            ------------------------------------------------------------ 559
human.FAM230J-LOC105372942-GGTLC5P.NCBI.rev.compl                                 atataatatataatatatattatataattatatattatgtacattatatattatttatat 852
human.FAM230A.ncRNA.18487127.18500594-GGTLC3.18516335.18518165.rev.compl.REF      ------------------------------------------------------------ 0

chimp.LOC112206744-LOC107973052-GGT2.revcompl                                     ------------------------------------------------------------ 981
chimp54546-135457.revcompl.Pan.troglodytes.clone.rp43-41g5.GenBank:AC099533.36    ------------------------------------------------------------ 2112
human.FAM30B-LOC105372935-GGT2.NCBI.                                              ------------------------------------------------------------ 1479
human.FAM230E-LOC105377182-GGT3P.NCBI.                                            ------------------------------------------------------------ 559
human.FAM230J-LOC105372942-GGTLC5P.NCBI.rev.compl                                 atattatatataacatataatatatataataaataatatatatattatatatatcatata 912
human.FAM230A.ncRNA.18487127.18500594-GGTLC3.18516335.18518165.rev.compl.REF      ------------------------------------------------------------ 0

chimp.LOC112206744-LOC107973052-GGT2.revcompl                                     -----------attgtatataatatatattatattatatattataatacataatataata 1030
chimp54546-135457.revcompl.Pan.troglodytes.clone.rp43-41g5.GenBank:AC099533.36    -----------TATATATATAATATATATAATATGTATTATATATACAGTGTATATAATA 2161
human.FAM30B-LOC105372935-GGT2.NCBI.                                              -----------tatat-------------------------------------------- 1484
human.FAM230E-LOC105377182-GGT3P.NCBI.                                            ------------------------------------------------------------ 559
human.FAM230J-LOC105372942-GGTLC5P.NCBI.rev.compl                                 atatataatatatata-------------------------------------------- 928
human.FAM230A.ncRNA.18487127.18500594-GGTLC3.18516335.18518165.rev.compl.REF      ------------------------------------------------------------ 0

chimp.LOC112206744-LOC107973052-GGT2.revcompl                                     tataatat------attatatattatatatattatata------atatattatatattat 1078
chimp54546-135457.revcompl.Pan.troglodytes.clone.rp43-41g5.GenBank:AC099533.36    TATATTATATATACATTATATATAATACATATTATATACACTGTATATATAATATATATT 2221
human.FAM30B-LOC105372935-GGT2.NCBI.                                              ------------------------------------------------------------ 1484
human.FAM230E-LOC105377182-GGT3P.NCBI.                                            ------------------------------------------------------------ 559
human.FAM230J-LOC105372942-GGTLC5P.NCBI.rev.compl                                 ------------------------------------------------------------ 928
human.FAM230A.ncRNA.18487127.18500594-GGTLC3.18516335.18518165.rev.compl.REF      ------------------------------------------------------------ 0

chimp.LOC112206744-LOC107973052-GGT2.revcompl                                     atatatatataatatataatatattatatatatgt-----------tatatattatatat 1127
chimp54546-135457.revcompl.Pan.troglodytes.clone.rp43-41g5.GenBank:AC099533.36    ATATATAAAATATAGATAATATATATTATATATACAGTAGGTAAAATATATATTACATAT 2281
human.FAM30B-LOC105372935-GGT2.NCBI.                                              ------------------------------------------------------------ 1484
human.FAM230E-LOC105377182-GGT3P.NCBI.                                            ------------------------------------------------------------ 559
human.FAM230J-LOC105372942-GGTLC5P.NCBI.rev.compl                                 ------------------------------------------------------------ 928
human.FAM230A.ncRNA.18487127.18500594-GGTLC3.18516335.18518165.rev.compl.REF      ------------------------------------------------------------ 0

chimp.LOC112206744-LOC107973052-GGT2.revcompl                                     atatgttatatattatagaatatgttatatattatatattatagaatatgttatatatta 1187
chimp54546-135457.revcompl.Pan.troglodytes.clone.rp43-41g5.GenBank:AC099533.36    ACTGTATATATAATATATATTATATATATTATATTATATTATATTATATTATATATATTA 2341
human.FAM30B-LOC105372935-GGT2.NCBI.                                              ------------------------------------------------------------ 1484
human.FAM230E-LOC105377182-GGT3P.NCBI.                                            ------------------------------------------------------------ 559
human.FAM230J-LOC105372942-GGTLC5P.NCBI.rev.compl                                 ------------------------------------------------------------ 928
human.FAM230A.ncRNA.18487127.18500594-GGTLC3.18516335.18518165.rev.compl.REF      ------------------------------------------------------------ 0

chimp.LOC112206744-LOC107973052-GGT2.revcompl                                     tatattatata------atatgttatatattatatattataga----------------- 1224
chimp54546-135457.revcompl.Pan.troglodytes.clone.rp43-41g5.GenBank:AC099533.36    TATATTATATATATGATATATGATATATAATATATATTATATATACAGTATATGTAATAT 2401
human.FAM30B-LOC105372935-GGT2.NCBI.                                              ------------------------------------------------------------ 1484
human.FAM230E-LOC105377182-GGT3P.NCBI.                                            ------------------------------------------------------------ 559
human.FAM230J-LOC105372942-GGTLC5P.NCBI.rev.compl                                 ------------------------------------------------------------ 928
human.FAM230A.ncRNA.18487127.18500594-GGTLC3.18516335.18518165.rev.compl.REF      ------------------------------------------------------------ 0

chimp.LOC112206744-LOC107973052-GGT2.revcompl                                     -----------------------------------------------------------a 1225
chimp54546-135457.revcompl.Pan.troglodytes.clone.rp43-41g5.GenBank:AC099533.36    GTATTATATATACAGTATATGTAATATGTATTATATATATTATTTATAATATGTATTATA 2461
human.FAM30B-LOC105372935-GGT2.NCBI.                                              ------------------------------------------------------------ 1484
human.FAM230E-LOC105377182-GGT3P.NCBI.                                            ------------------------------------------------------------ 559
human.FAM230J-LOC105372942-GGTLC5P.NCBI.rev.compl                                 ------------------------------------------------------------ 928
human.FAM230A.ncRNA.18487127.18500594-GGTLC3.18516335.18518165.rev.compl.REF      ------------------------------------------------------------ 0

chimp.LOC112206744-LOC107973052-GGT2.revcompl                                     tatgttatatattatataatatgttatatattatatattatataatatgttatatattat 1285
chimp54546-135457.revcompl.Pan.troglodytes.clone.rp43-41g5.GenBank:AC099533.36    TATATTATTTATAATAT-GTTTGATATATATTATATATAATAT-ATATTATATATATTAT 2519
human.FAM30B-LOC105372935-GGT2.NCBI.                                              ------------------------------------------------------------ 1484
human.FAM230E-LOC105377182-GGT3P.NCBI.                                            ------------------------------------------------------------ 559
human.FAM230J-LOC105372942-GGTLC5P.NCBI.rev.compl                                 ------------------------------------------------------------ 928
human.FAM230A.ncRNA.18487127.18500594-GGTLC3.18516335.18518165.rev.compl.REF      ------------------------------------------------------------ 0

chimp.LOC112206744-LOC107973052-GGT2.revcompl                                     atatattatataatatgttatatattatataatatgt----tatatattatatatattat 1341
chimp54546-135457.revcompl.Pan.troglodytes.clone.rp43-41g5.GenBank:AC099533.36    AAATAATATATAGTATA-TATATTATATATAATATATAGTATATATATTATATATAATAT 2578
human.FAM30B-LOC105372935-GGT2.NCBI.                                              ------------------------------------------------------------ 1484
human.FAM230E-LOC105377182-GGT3P.NCBI.                                            ------------------------------------------------------------ 559
human.FAM230J-LOC105372942-GGTLC5P.NCBI.rev.compl                                 ------------------------------------------------------------ 928
human.FAM230A.ncRNA.18487127.18500594-GGTLC3.18516335.18518165.rev.compl.REF      ------------------------------------------------------------ 0

chimp.LOC112206744-LOC107973052-GGT2.revcompl                                     ataatatgt----tatatattatatatattatataatatgt------tatatattatata 1391
chimp54546-135457.revcompl.Pan.troglodytes.clone.rp43-41g5.GenBank:AC099533.36    ATAATATATAGTATATATATTATATATTATATATAATATATATAGTATATATATTATATA 2638
human.FAM30B-LOC105372935-GGT2.NCBI.                                              ------------------------------------------------------------ 1484
human.FAM230E-LOC105377182-GGT3P.NCBI.                                            ------------------------------------------------------------ 559
human.FAM230J-LOC105372942-GGTLC5P.NCBI.rev.compl                                 ------------------------------------------------------------ 928
human.FAM230A.ncRNA.18487127.18500594-GGTLC3.18516335.18518165.rev.compl.REF      ------------------------------------------------------------ 0

chimp.LOC112206744-LOC107973052-GGT2.revcompl                                     ttatataatatgttatatattatatatattatataatatgttatatattatatattatat 1451
chimp54546-135457.revcompl.Pan.troglodytes.clone.rp43-41g5.GenBank:AC099533.36    TAGTAT-ATAGTATATATATTATTTATAATATATAGTA---TATATATTATTTATAATAT 2694
human.FAM30B-LOC105372935-GGT2.NCBI.                                              ------------------------------------------------------------ 1484
human.FAM230E-LOC105377182-GGT3P.NCBI.                                            ------------------------------------------------------------ 559
human.FAM230J-LOC105372942-GGTLC5P.NCBI.rev.compl                                 ------------------------------------------------------------ 928
human.FAM230A.ncRNA.18487127.18500594-GGTLC3.18516335.18518165.rev.compl.REF      ------------------------------------------------------------ 0

chimp.LOC112206744-LOC107973052-GGT2.revcompl                                     aatatgttatatattatatatattatataatatgttatatattatatattatataatatg 1511
chimp54546-135457.revcompl.Pan.troglodytes.clone.rp43-41g5.GenBank:AC099533.36    -ATAGTATATATATTATTTATATTCCAGGATATATTATATTTTATAATATATATTATTTC 2753
human.FAM30B-LOC105372935-GGT2.NCBI.                                              ------------------------------------------------------------ 1484
human.FAM230E-LOC105377182-GGT3P.NCBI.                                            ------------------------------------------------------------ 559
```

```
human.FAM230J-LOC105372942-GGTLC5P.NCBI.rev.compl                          ------------------------------------------------------------ 928
human.FAM230A.ncRNA.18487127.18500594-GGTLC3.18516335.18518165.rev.compl.REF ------------------------------------------------------------ 0

chimp.LOC112206744-LOC107973052-GGT2.revcompl                              t------tatatattatatatattatataatatatattatatcttatataatataattta 1565
chimp54546-135457.revcompl.Pan.troglodytes.clone.rp43-41g5.GenBank:AC099533.36 CATACTATATATAATGTGTATATTATATATTATATATTTAATA-TATAATGTATATATTG 2812
human.FAM30B-LOC105372935-GGT2.NCBI.                                       ------------------------------------------------------------ 1484
human.FAM230E-LOC105377182-GGT3P.NCBI.                                     ------------------------------------------------------------ 559
human.FAM230J-LOC105372942-GGTLC5P.NCBI.rev.compl                          ------------------------------------------------------------ 928
human.FAM230A.ncRNA.18487127.18500594-GGTLC3.18516335.18518165.rev.compl.REF ------------------------------------------------------------ 0

chimp.LOC112206744-LOC107973052-GGT2.revcompl                              tatattatatattatacaatatatattatatattatattatatattatacaatatatatt 1625
chimp54546-135457.revcompl.Pan.troglodytes.clone.rp43-41g5.GenBank:AC099533.36 TATGTAATATATTATATTATATGTTATATGTAATATATTATATATTATATGTTATATGTA 2872
human.FAM30B-LOC105372935-GGT2.NCBI.                                       ------------------------------------------------------------ 1484
human.FAM230E-LOC105377182-GGT3P.NCBI.                                     ------------------------------------------------------------ 559
human.FAM230J-LOC105372942-GGTLC5P.NCBI.rev.compl                          ------------------------------------------------------------ 928
human.FAM230A.ncRNA.18487127.18500594-GGTLC3.18516335.18518165.rev.compl.REF ------------------------------------------------------------ 0

chimp.LOC112206744-LOC107973052-GGT2.revcompl                              atata-tatattatatattatacaatatatattatataatatataatatatattatat-- 1682
chimp54546-135457.revcompl.Pan.troglodytes.clone.rp43-41g5.GenBank:AC099533.36 ATATATTATATTATATATTATATGTAATATATTATATATTATATGTAATATATTATATAT 2932
human.FAM30B-LOC105372935-GGT2.NCBI.                                       ------------------------------------------------------------ 1484
human.FAM230E-LOC105377182-GGT3P.NCBI.                                     ------------------------------------------------------------ 559
human.FAM230J-LOC105372942-GGTLC5P.NCBI.rev.compl                          ------------------------------------------------------------ 928
human.FAM230A.ncRNA.18487127.18500594-GGTLC3.18516335.18518165.rev.compl.REF ------------------------------------------------------------ 0

chimp.LOC112206744-LOC107973052-GGT2.revcompl                              --tatataatatatatataatttccttttacatgctgcatccttcaacattccatccccc 1740
chimp54546-135457.revcompl.Pan.troglodytes.clone.rp43-41g5.GenBank:AC099533.36 TACATATAATATATATATAATTTCCTTTTACATCCTGCATCCTTCAACGTTCCATCCCCC 2992
human.FAM30B-LOC105372935-GGT2.NCBI.                                       --------atatatatataatttccttttacatcctgcatccttcaacgttacatccccc 1536
human.FAM230E-LOC105377182-GGT3P.NCBI.                                     --------atatatatataatttccttttacatcctgcatccttcaacgttccatccccc 611
human.FAM230J-LOC105372942-GGTLC5P.NCBI.rev.compl                          --------atatatatataatttccttttacatcctgcatccttcaacgttccattcccc 980
human.FAM230A.ncRNA.18487127.18500594-GGTLC3.18516335.18518165.rev.compl.REF ------------------------------------------------------------ 0

chimp.LOC112206744-LOC107973052-GGT2.revcompl                              accccacagattaagttattccccaggggagaatatggcaaactctattttaatgcagtt 1800
chimp54546-135457.revcompl.Pan.troglodytes.clone.rp43-41g5.GenBank:AC099533.36 ACCCCACAGATTAAGTTATTCCCCAAGGGAGAATATGGCAAAGTCTATTTTAATGCAGTA 3052
human.FAM30B-LOC105372935-GGT2.NCBI.                                       accccacagattaagttattccccaggggagaatatggcaaagtctattttaatgcagtt 1596
human.FAM230E-LOC105377182-GGT3P.NCBI.                                     accccacagattaagttattccccaggggagaatatggcaaagtctattttaatgcagtt 671
human.FAM230J-LOC105372942-GGTLC5P.NCBI.rev.compl                          accccacagattaagttattccccaggggagaatatggcagagtctattttaatgctgtt 1040
human.FAM230A.ncRNA.18487127.18500594-GGTLC3.18516335.18518165.rev.compl.REF ------------------------------------------------------------ 0

chimp.LOC112206744-LOC107973052-GGT2.revcompl                              tttagcctaatt------------------------------------------------ 1812
chimp54546-135457.revcompl.Pan.troglodytes.clone.rp43-41g5.GenBank:AC099533.36 GTTACCCTAATTAAGAACTTATGAGGCCGGGCGCGGTGGCTCACGACTGTAATCCCAGCA 3112
human.FAM30B-LOC105372935-GGT2.NCBI.                                       tttaacccaatt------------------------------------------------ 1608
human.FAM230E-LOC105377182-GGT3P.NCBI.                                     tttaacccaatt------------------------------------------------ 683
human.FAM230J-LOC105372942-GGTLC5P.NCBI.rev.compl                          tttaacccaatt------------------------------------------------ 1052
human.FAM230A.ncRNA.18487127.18500594-GGTLC3.18516335.18518165.rev.compl.REF ------------------------------------------------------------ 0

chimp.LOC112206744-LOC107973052-GGT2.revcompl                              ------------------------------------------------------------ 1812
chimp54546-135457.revcompl.Pan.troglodytes.clone.rp43-41g5.GenBank:AC099533.36 CTTTGGGAGGCCGAGGTGGGGGGATCACGAGGTCAGGAGATCGAGACCATCCTGGCTAAC 3172
human.FAM30B-LOC105372935-GGT2.NCBI.                                       ------------------------------------------------------------ 1608
human.FAM230E-LOC105377182-GGT3P.NCBI.                                     ------------------------------------------------------------ 683
human.FAM230J-LOC105372942-GGTLC5P.NCBI.rev.compl                          ------------------------------------------------------------ 1052
human.FAM230A.ncRNA.18487127.18500594-GGTLC3.18516335.18518165.rev.compl.REF ------------------------------------------------------------ 0

chimp.LOC112206744-LOC107973052-GGT2.revcompl                              ------------------------------------------------------------ 1812
chimp54546-135457.revcompl.Pan.troglodytes.clone.rp43-41g5.GenBank:AC099533.36 ATGGTGAAACCCCGTCTCTACTAAAAATACAAAAAATTAGCCGGGCGTGGTGGCGGGCGC 3232
human.FAM30B-LOC105372935-GGT2.NCBI.                                       ------------------------------------------------------------ 1608
human.FAM230E-LOC105377182-GGT3P.NCBI.                                     ------------------------------------------------------------ 683
human.FAM230J-LOC105372942-GGTLC5P.NCBI.rev.compl                          ------------------------------------------------------------ 1052
human.FAM230A.ncRNA.18487127.18500594-GGTLC3.18516335.18518165.rev.compl.REF ------------------------------------------------------------ 0

chimp.LOC112206744-LOC107973052-GGT2.revcompl                              ------------------------------------------------------------ 1812
chimp54546-135457.revcompl.Pan.troglodytes.clone.rp43-41g5.GenBank:AC099533.36 CTGTAGTCCCAGCTACTTGGGAGGCTGAGGCAGGAGAATGGCGTGAACCCGGGAGGCGGA 3292
human.FAM30B-LOC105372935-GGT2.NCBI.                                       ------------------------------------------------------------ 1608
human.FAM230E-LOC105377182-GGT3P.NCBI.                                     ------------------------------------------------------------ 683
human.FAM230J-LOC105372942-GGTLC5P.NCBI.rev.compl                          ------------------------------------------------------------ 1052
human.FAM230A.ncRNA.18487127.18500594-GGTLC3.18516335.18518165.rev.compl.REF ------------------------------------------------------------ 0

chimp.LOC112206744-LOC107973052-GGT2.revcompl                              ------------------------------------------------------------ 1812
chimp54546-135457.revcompl.Pan.troglodytes.clone.rp43-41g5.GenBank:AC099533.36 GCTTGCAGTGAGCCAAGATCGCGCCACTGCACTCCAGCCTGGGCGACAGAGCGAGACTCC 3352
human.FAM30B-LOC105372935-GGT2.NCBI.                                       ------------------------------------------------------------ 1608
human.FAM230E-LOC105377182-GGT3P.NCBI.                                     ------------------------------------------------------------ 683
human.FAM230J-LOC105372942-GGTLC5P.NCBI.rev.compl                          ------------------------------------------------------------ 1052
human.FAM230A.ncRNA.18487127.18500594-GGTLC3.18516335.18518165.rev.compl.REF ------------------------------------------------------------ 0

chimp.LOC112206744-LOC107973052-GGT2.revcompl                              -------------------------aagaa-ttatgaaatcattactttccaaatatttg 1846
chimp54546-135457.revcompl.Pan.troglodytes.clone.rp43-41g5.GenBank:AC099533.36 GTCTCAAAAAAAAAAAAAAAAAAAAAGAACTTATGAAATCATTACTTTCCAAATCTTTG 3412
human.FAM30B-LOC105372935-GGT2.NCBI.                                       -------------------------aagaacctatgaaatcattactttccaaaactttg 1643
human.FAM230E-LOC105377182-GGT3P.NCBI.                                     -------------------------aagaacctatgaaatcattactttccaaaactttg 718
human.FAM230J-LOC105372942-GGTLC5P.NCBI.rev.compl                          -------------------------aagaacctatgaaatcattactttccaaaactttg 1087
human.FAM230A.ncRNA.18487127.18500594-GGTLC3.18516335.18518165.rev.compl.REF ------------------------------------------------------------ 0

chimp.LOC112206744-LOC107973052-GGT2.revcompl                              gaacaaagccacagtagtatgcatgggttggaggcttttcacagaataaaatgtacctat 1906
chimp54546-135457.revcompl.Pan.troglodytes.clone.rp43-41g5.GenBank:AC099533.36 GAACAAAGCCACAGTAGTAGGGATGGGTTGGAGGCTTTTCACACAATAAAATGTACCTCT 3472
human.FAM30B-LOC105372935-GGT2.NCBI.                                       gaacaaagccacagtagtatggatccgttggaggcttttcacacaataaaatgtacctct 1703
human.FAM230E-LOC105377182-GGT3P.NCBI.                                     gaacaaagccacagtagtatggatccgttggaggcttttcacacaataaaatgtacatct 778
human.FAM230J-LOC105372942-GGTLC5P.NCBI.rev.compl                          gaacaaagccacagtagtaaggatccgttggaggcttttcacacaataaaatgtaactct 1147
human.FAM230A.ncRNA.18487127.18500594-GGTLC3.18516335.18518165.rev.compl.REF ------------------------------------------------------------ 0

chimp.LOC112206744-LOC107973052-GGT2.revcompl                              ctttgtttttaacatgtttttcccttcctctcttctttctttgtgaaatgtgtatttact 1966
chimp54546-135457.revcompl.Pan.troglodytes.clone.rp43-41g5.GenBank:AC099533.36 CTTTGTTTTTAACATGTTTTTCCCTTCCTCTCTTCTTTCTTTGTGAAATGTGTATTTACT 3532
human.FAM30B-LOC105372935-GGT2.NCBI.                                       ctttgtttttaacatgtttttcccttcctctcttctttttttgtgaaatgtgtatttact 1763
human.FAM230E-LOC105377182-GGT3P.NCBI.                                     ctttgtttttaacatgtttttcccttcctctcttctttttttgtgaaatgtgtatttact 838
human.FAM230J-LOC105372942-GGTLC5P.NCBI.rev.compl                          ctttgtttttaacatgtttttcccttcctctcttctttttttgtgaaatgtgtatttact 1207
human.FAM230A.ncRNA.18487127.18500594-GGTLC3.18516335.18518165.rev.compl.REF ------------------------------------------------------------ 0

chimp.LOC112206744-LOC107973052-GGT2.revcompl                              ttaataaatttgtagtaagtcacttccattcacatattaatttttttaaagtaataagtat 2026
chimp54546-135457.revcompl.Pan.troglodytes.clone.rp43-41g5.GenBank:AC099533.36 TTAATAAGTTTGTAGTAAGTCACTTCTATTCACATATTAATTTTTTAAAGTAATAAGTAT 3592
human.FAM30B-LOC105372935-GGT2.NCBI.                                       ttaatatatttgtagtaagtcacttccatgcacatattaatttttttaaagtaataagtat 1823
human.FAM230E-LOC105377182-GGT3P.NCBI.                                     ttaatatatttgtagtaagtcacttccatgcacatattaatttttttaaagtaataagtat 898
human.FAM230J-LOC105372942-GGTLC5P.NCBI.rev.compl                          ttaatatatttgtagtaagtcacttccatgcacatattaatttttttaaagtaataagcat 1267
human.FAM230A.ncRNA.18487127.18500594-GGTLC3.18516335.18518165.rev.compl.REF ------------------------------------------------------------ 0

chimp.LOC112206744-LOC107973052-GGT2.revcompl                              gtgtattgtctactagtgaaataaaacacacatttattttattctttggaagttatcca 2086
chimp54546-135457.revcompl.Pan.troglodytes.clone.rp43-41g5.GenBank:AC099533.36 GTGTATTGTCTACGTGTGAAATAAAACACACATTTATTTTATGCTTTGGAAGTTATCCA 3652
human.FAM30B-LOC105372935-GGT2.NCBI.                                       gtgtattgtctacgtgtgaaagaaaacacacatttattttatgctttggaagttatcca 1883
human.FAM230E-LOC105377182-GGT3P.NCBI.                                     gtgtattgtctacgtgtgaaagaaaacacacatttattttatgccttggaagttatcca 958
human.FAM230J-LOC105372942-GGTLC5P.NCBI.rev.compl                          gtgtattgtctacgtgtgaaagaaaacacacatttattttatgctttggaagttatcca 1327
human.FAM230A.ncRNA.18487127.18500594-GGTLC3.18516335.18518165.rev.compl.REF ------------------------------------------------------------ 0

chimp.LOC112206744-LOC107973052-GGT2.revcompl                              gaatcatggcattgtcaatcacagtcaatcacccaacctactcacctttccagtgtaatc 2146
chimp54546-135457.revcompl.Pan.troglodytes.clone.rp43-41g5.GenBank:AC099533.36 GAATCATGGAATTGTCAATCACAGTCAATCACCCAACCTACTCACCTTTCCAGTGTAATC 3712
human.FAM30B-LOC105372935-GGT2.NCBI.                                       gaatcatggaattgtcaatcacagtcaatcacccaacctactcacctttccagtgtaatc 1943
human.FAM230E-LOC105377182-GGT3P.NCBI.                                     gaatcatggaattgtcaatcacagtcaatcacccaacctactcacctttccagtgtaatc 1018
human.FAM230J-LOC105372942-GGTLC5P.NCBI.rev.compl                          gaatcatggaattgtcaatcacagtcaatcacccaacctactcacctttccagtgtaatc 1387
human.FAM230A.ncRNA.18487127.18500594-GGTLC3.18516335.18518165.rev.compl.REF ------------------------------------------------------------ 0

chimp.LOC112206744-LOC107973052-GGT2.revcompl                              ttagtctaa-tttttttttgttatccaatgagatgcagtatttcaactcagagagataa 2205
chimp54546-135457.revcompl.Pan.troglodytes.clone.rp43-41g5.GenBank:AC099533.36 TTAGTCAAG--TTTTTTTTGTTATCCAATGAGATGCAGTATTTCAACTCAGAAAGATAA 3770
human.FAM30B-LOC105372935-GGT2.NCBI.                                       ttagtcaaa-tttttttttgttatccaatgagatgcagtatttcaactcagaaagataa 2002
human.FAM230E-LOC105377182-GGT3P.NCBI.                                     ttagtcaaa-tttttttttgttatccaatgagatgcagtatttcaactcagaaagataa 1077
human.FAM230J-LOC105372942-GGTLC5P.NCBI.rev.compl                          ttagtcaaatttttttttttgttatccaatgagatgcagtatttcaactcagaaagataa 1447
human.FAM230A.ncRNA.18487127.18500594-GGTLC3.18516335.18518165.rev.compl.REF ------------------------------------------------------------ 0

chimp.LOC112206744-LOC107973052-GGT2.revcompl                              atagaatgaattggtagagactattaactaagaatatacagttttatttatgctcagaag 2265
chimp54546-135457.revcompl.Pan.troglodytes.clone.rp43-41g5.GenBank:AC099533.36 ATAGAATGAATTGATAGAGACTATTAACTAAGAATATACAGTTTTATTTATACTCAGAAG 3830
human.FAM30B-LOC105372935-GGT2.NCBI.                                       atagagtgaatttatagagactattaactaagaacatacagttttatttatactcagaag 2062
human.FAM230E-LOC105377182-GGT3P.NCBI.                                     atagagtgaatttatagagactattaactaagaacatacagttttatttatactcagaag 1137
human.FAM230J-LOC105372942-GGTLC5P.NCBI.rev.compl                          atagagtgaatttatagagactattaactaagaacatacagtttgatttatactcagaag 1507
```

```
human.FAM230A.ncRNA.18487127.18500594-GGTLC3.18516335.18518165.rev.compl.REF        ------------------------------------------------------------ 0

chimp.LOC112206744-LOC107973052-GGT2.revcompl                                        caagtagattatgtatatatgtatgaagataaaaattaaaaggataattgtgtaaatttg 2325
chimp54546-135457.revcompl.Pan.troglodytes.clone.rp43-41g5.GenBank:AC099533.36      CAAGTAGATTATGTATATATGTATGAAGATAAAAATTAAAAGGATAATTGTGTAAATTTG 3890
human.FAM30B-LOC105372935-GGT2.NCBI.                                                 caagtagattatgtacatatatatgaagataaaaattaaaaggataattgtgtaaatttg 2122
human.FAM230E-LOC105377182-GGT3P.NCBI.                                               caagtagattatgtacatatatatgaagataaaaattaaaaggataattgtgtaaatttg 1197
human.FAM230J-LOC105372942-GGTLC5P.NCBI.rev.compl                                    caagtagattatgtacatatatatgaagataaaaattaaaaggataattgtgtaaatttg 1567
human.FAM230A.ncRNA.18487127.18500594-GGTLC3.18516335.18518165.rev.compl.REF        ------------------------------------------------------------ 0

chimp.LOC112206744-LOC107973052-GGT2.revcompl                                        catgtagagagctttgaaaacctgtttacttgttaatgctgttttgatgtattgtgtgtc 2385
chimp54546-135457.revcompl.Pan.troglodytes.clone.rp43-41g5.GenBank:AC099533.36      CATGTGGAGAGCTTTGAAAACCTGTTTACTTGTTAATGCTGTTTTGATGTATTGTGTGTC 3950
human.FAM30B-LOC105372935-GGT2.NCBI.                                                 catgtagagggctttgaaaacctgtttacttgttaatgctgttttgatgtat--tgtgtc 2180
human.FAM230E-LOC105377182-GGT3P.NCBI.                                               catgtagagggctttgaaaacctgtttacttgttaatgctgttttgatgtat--tgtgtc 1255
human.FAM230J-LOC105372942-GGTLC5P.NCBI.rev.compl                                    catgtagagggctttgaaaacctgtttacttgtgaatgctgttttgatgtat--tgtgtc 1625
human.FAM230A.ncRNA.18487127.18500594-GGTLC3.18516335.18518165.rev.compl.REF        ------------------------------------------------------------ 0

chimp.LOC112206744-LOC107973052-GGT2.revcompl                                        tttgttctcccgacccagcatccagagctctctgctggagctaagtgctcatcagttcca 2445
chimp54546-135457.revcompl.Pan.troglodytes.clone.rp43-41g5.GenBank:AC099533.36      TTTGTTCTCCCGACCCAGCATCCAGAGCTCTCTGCTGGAGCTAAGTGCTCATCAGTTCCA 4010
human.FAM30B-LOC105372935-GGT2.NCBI.                                                 tttgttctcccgacccatcgtccagagctctctgcaggagctaagtgctcatcagttcca 2240
human.FAM230E-LOC105377182-GGT3P.NCBI.                                               tttgttctcccgacccatcgtccagagctctctgcaggagctaagtgctcatcagttcca 1315
human.FAM230J-LOC105372942-GGTLC5P.NCBI.rev.compl                                    tttgttctcccgacccatcatccagagctctctgcaggagctaagtgctcatcagttcca 1685
human.FAM230A.ncRNA.18487127.18500594-GGTLC3.18516335.18518165.rev.compl.REF        ------------------------------------------------------------ 0

chimp.LOC112206744-LOC107973052-GGT2.revcompl                                        tgacttggaaactgtctaagtttagaggcacttgtatttgttagtaaataagtcaagatg 2505
chimp54546-135457.revcompl.Pan.troglodytes.clone.rp43-41g5.GenBank:AC099533.36      TGACTTGGAAACTGTCTAAGTTTAGAGGCACTTGTATTTGTTAGTAAATAAGTCAAGATG 4070
human.FAM30B-LOC105372935-GGT2.NCBI.                                                 tgacttggaaactgtctaagtttagaggcacttgtatttgttagtaaataaggcaagatg 2300
human.FAM230E-LOC105377182-GGT3P.NCBI.                                               tgacttggaaactgtctaagtttagaggcacttgtatttgttagtaaataaggcaagatg 1375
human.FAM230J-LOC105372942-GGTLC5P.NCBI.rev.compl                                    tgacttggaaactgtctaagtttagaggcacttgtatttgttagtaaataaggcaagatg 1745
human.FAM230A.ncRNA.18487127.18500594-GGTLC3.18516335.18518165.rev.compl.REF        ------------------------------------------------------------ 0

chimp.LOC112206744-LOC107973052-GGT2.revcompl                                        atattgtttcacaggctttagtgcagaagactgaatagagaagctgctccacccagtaca 2565
chimp54546-135457.revcompl.Pan.troglodytes.clone.rp43-41g5.GenBank:AC099533.36      ATATTGTTTCACAGGCTTTAGTGCAGAAGACTGAATAGAGAAGCTGCTCCACCCAGTACA 4130
human.FAM30B-LOC105372935-GGT2.NCBI.                                                 atattgtttcacaggttttagtgccgaagactgaatagataagctgctccacccagtaca 2360
human.FAM230E-LOC105377182-GGT3P.NCBI.                                               atattgtttcacaggttttagtgccgaagactgaatagataagctgctccacccagtaca 1435
human.FAM230J-LOC105372942-GGTLC5P.NCBI.rev.compl                                    atattgtttcacaggttttagtgccgaagactgaatagataagctgctccacccagtaca 1805
human.FAM230A.ncRNA.18487127.18500594-GGTLC3.18516335.18518165.rev.compl.REF        ------------------------------------------------------------ 0

chimp.LOC112206744-LOC107973052-GGT2.revcompl                                        ctggtgttcatttcatggtcatctcatctgttaaccatggatataaaacacttatcttca 2625
chimp54546-135457.revcompl.Pan.troglodytes.clone.rp43-41g5.GenBank:AC099533.36      CTGGTGTTCATTTCATGGTCATCTCATCTGTTAACCATGGATATAAAACACTTATCTTCA 4190
human.FAM30B-LOC105372935-GGT2.NCBI.                                                 ctggtgttcatttcatggtcatctcatctgttaaccatggatataaaacatttatcttca 2420
human.FAM230E-LOC105377182-GGT3P.NCBI.                                               ctggtgttcatttcatggtcatctcatctgttaaccatggatataaaacatttatcttca 1495
human.FAM230J-LOC105372942-GGTLC5P.NCBI.rev.compl                                    ctggtgttcatttcatggtcatctcatctgttaaccatggatataaaacatttatcttca 1865
human.FAM230A.ncRNA.18487127.18500594-GGTLC3.18516335.18518165.rev.compl.REF        ------------------------------------------------------------ 0

chimp.LOC112206744-LOC107973052-GGT2.revcompl                                        atgatcttcctgtacatgtaaaaacacacctgtctgcatggcagcagttggacctcacag 2685
chimp54546-135457.revcompl.Pan.troglodytes.clone.rp43-41g5.GenBank:AC099533.36      ATGATCTTCCTGTACATATAAAAACACACCTGTCTACATGGCAGCAGTTGGACCTCACAA 4250
human.FAM30B-LOC105372935-GGT2.NCBI.                                                 at---------------------------------------------------------- 2422
human.FAM230E-LOC105377182-GGT3P.NCBI.                                               at---------------------------------------------------------- 1497
human.FAM230J-LOC105372942-GGTLC5P.NCBI.rev.compl                                    at---------------------------------------------------------- 1867
human.FAM230A.ncRNA.18487127.18500594-GGTLC3.18516335.18518165.rev.compl.REF        ------------------------------------------------------------ 0

chimp.LOC112206744-LOC107973052-GGT2.revcompl                                        tgtggattgtgccttcaccctggaatgtctatgatgccctatcgaccatggtgatgggat 2745
chimp54546-135457.revcompl.Pan.troglodytes.clone.rp43-41g5.GenBank:AC099533.36      TGTGGATTGTGCCTTCACCCTGGAATGTCTATGATGCCCTATCGACCATGGTGATGGGAT 4310
human.FAM30B-LOC105372935-GGT2.NCBI.                                                 ------------------------------------------------------------ 2422
human.FAM230E-LOC105377182-GGT3P.NCBI.                                               ------------------------------------------------------------ 1497
human.FAM230J-LOC105372942-GGTLC5P.NCBI.rev.compl                                    ------------------------------------------------------------ 1867
human.FAM230A.ncRNA.18487127.18500594-GGTLC3.18516335.18518165.rev.compl.REF        ------------------------------------------------------------ 0

chimp.LOC112206744-LOC107973052-GGT2.revcompl                                        tagggatctcttcccctttgctcggccactgtctgtgccagccaggccactgggccattg 2805
chimp54546-135457.revcompl.Pan.troglodytes.clone.rp43-41g5.GenBank:AC099533.36      TAGGGATCTCTTGCCCTTTGCTCTGCCACTGTCTGTGCCAGCCAGGCCACTGGGCCATTG 4370
human.FAM30B-LOC105372935-GGT2.NCBI.                                                 ------------------------------------------------------------ 2422
human.FAM230E-LOC105377182-GGT3P.NCBI.                                               ------------------------------------------------------------ 1497
human.FAM230J-LOC105372942-GGTLC5P.NCBI.rev.compl                                    ------------------------------------------------------------ 1867
human.FAM230A.ncRNA.18487127.18500594-GGTLC3.18516335.18518165.rev.compl.REF        ------------------------------------------------------------ 0

chimp.LOC112206744-LOC107973052-GGT2.revcompl                                        tggcggatggtgatgccctttgtgtggagcgcactgagtgtgcgctgaggcagacacagt 2865
chimp54546-135457.revcompl.Pan.troglodytes.clone.rp43-41g5.GenBank:AC099533.36      TGGCAGATGGTGATGCCCTTTGTGTGGAGCGCACTGAGTGTGCGCTGAGGCAGACACAGT 4430
human.FAM30B-LOC105372935-GGT2.NCBI.                                                 ------------------------------------------------------------ 2422
human.FAM230E-LOC105377182-GGT3P.NCBI.                                               ------------------------------------------------------------ 1497
human.FAM230J-LOC105372942-GGTLC5P.NCBI.rev.compl                                    ------------------------------------------------------------ 1867
human.FAM230A.ncRNA.18487127.18500594-GGTLC3.18516335.18518165.rev.compl.REF        ------------------------------------------------------------ 0

chimp.LOC112206744-LOC107973052-GGT2.revcompl                                        acttttgacaactttatctgcttctttcaaaactgggtctgcattatgataaagtggtcg 2925
chimp54546-135457.revcompl.Pan.troglodytes.clone.rp43-41g5.GenBank:AC099533.36      ACTTTTGACAACTTTATCTGCTTCTTTCAAAACTGGGTCTGCATTATGATAAAGTGGTCG 4490
human.FAM30B-LOC105372935-GGT2.NCBI.                                                 ------------------------------------------------------------ 2422
human.FAM230E-LOC105377182-GGT3P.NCBI.                                               ------------------------------------------------------------ 1497
human.FAM230J-LOC105372942-GGTLC5P.NCBI.rev.compl                                    ------------------------------------------------------------ 1867
human.FAM230A.ncRNA.18487127.18500594-GGTLC3.18516335.18518165.rev.compl.REF        ------------------------------------------------------------ 0

chimp.LOC112206744-LOC107973052-GGT2.revcompl                                        gttttaggccaagcaggtgtatgtcatagagcaatgacttgggagttcagaatcctgggc 2985
chimp54546-135457.revcompl.Pan.troglodytes.clone.rp43-41g5.GenBank:AC099533.36      GTTTTAGGCCAAGCAGGTGTATGTCATAGAACAATGACTTGGGAGTTCAGAATCCTGGGC 4550
human.FAM30B-LOC105372935-GGT2.NCBI.                                                 ------------------------------------------------------------ 2422
human.FAM230E-LOC105377182-GGT3P.NCBI.                                               ------------------------------------------------------------ 1497
human.FAM230J-LOC105372942-GGTLC5P.NCBI.rev.compl                                    ------------------------------------------------------------ 1867
human.FAM230A.ncRNA.18487127.18500594-GGTLC3.18516335.18518165.rev.compl.REF        ------------------------------------------------------------ 0

chimp.LOC112206744-LOC107973052-GGT2.revcompl                                        gtgaataagcctctgagccttattaagctgtgaaatctaaagcaagacatttcactgata 3045
chimp54546-135457.revcompl.Pan.troglodytes.clone.rp43-41g5.GenBank:AC099533.36      GTGAATAAGCCTCTGAGCCTTATTAAGCTGTGAAATCTAAAGCAAGACATTTCACTGATA 4610
human.FAM30B-LOC105372935-GGT2.NCBI.                                                 ------------------------------------------------------------ 2422
human.FAM230E-LOC105377182-GGT3P.NCBI.                                               ------------------------------------------------------------ 1497
human.FAM230J-LOC105372942-GGTLC5P.NCBI.rev.compl                                    ------------------------------------------------------------ 1867
human.FAM230A.ncRNA.18487127.18500594-GGTLC3.18516335.18518165.rev.compl.REF        ------------------------------------------------------------ 0

chimp.LOC112206744-LOC107973052-GGT2.revcompl                                        attaagcctcatgagcacttatactgtacactttacctacatcaaatcattcggccatca 3105
chimp54546-135457.revcompl.Pan.troglodytes.clone.rp43-41g5.GenBank:AC099533.36      ATTAAGCCTCATGAGCACTTATACTGTACACTTTACCTACATGAAATCATTCGGCCATCG 4670
human.FAM30B-LOC105372935-GGT2.NCBI.                                                 ------------------------------------------------------------ 2422
human.FAM230E-LOC105377182-GGT3P.NCBI.                                               ------------------------------------------------------------ 1497
human.FAM230J-LOC105372942-GGTLC5P.NCBI.rev.compl                                    ------------------------------------------------------------ 1867
human.FAM230A.ncRNA.18487127.18500594-GGTLC3.18516335.18518165.rev.compl.REF        ------------------------------------------------------------ 0

chimp.LOC112206744-LOC107973052-GGT2.revcompl                                        ccacaaacaaatggcatgattgatttactatcacctttcgctgggtagaaaaagacagag 3165
chimp54546-135457.revcompl.Pan.troglodytes.clone.rp43-41g5.GenBank:AC099533.36      CCACAGACAAATGGCATGATTGATTTACTATCACCTTTTGCTGGGCAGAAAAAGACAGAG 4730
human.FAM30B-LOC105372935-GGT2.NCBI.                                                 ------------------------------------------------------------ 2422
human.FAM230E-LOC105377182-GGT3P.NCBI.                                               ------------------------------------------------------------ 1497
human.FAM230J-LOC105372942-GGTLC5P.NCBI.rev.compl                                    ------------------------------------------------------------ 1867
human.FAM230A.ncRNA.18487127.18500594-GGTLC3.18516335.18518165.rev.compl.REF        ------------------------------------------------------------ 0

chimp.LOC112206744-LOC107973052-GGT2.revcompl                                        atgaatgtgcccaagcttatgagcccctcagcaaaagagccaagatgggaacccaagcat 3225
chimp54546-135457.revcompl.Pan.troglodytes.clone.rp43-41g5.GenBank:AC099533.36      ATGAATGTGCCCAAGCTTATGAGCCCCTCAGCAAAAGAGCCAAGATGGGAACCCAAGCAT 4790
human.FAM30B-LOC105372935-GGT2.NCBI.                                                 ------------------------------------------------------------ 2422
human.FAM230E-LOC105377182-GGT3P.NCBI.                                               ------------------------------------------------------------ 1497
human.FAM230J-LOC105372942-GGTLC5P.NCBI.rev.compl                                    ------------------------------------------------------------ 1867
human.FAM230A.ncRNA.18487127.18500594-GGTLC3.18516335.18518165.rev.compl.REF        ------------------------------------------------------------ 0

chimp.LOC112206744-LOC107973052-GGT2.revcompl                                        acagccccaatgctgaggctctgaactactgacctgccctcagcactcagccttgggatc 3285
chimp54546-135457.revcompl.Pan.troglodytes.clone.rp43-41g5.GenBank:AC099533.36      ACAGCCCCAATGCTGAGGCTCTGAACTACTGACCTGCCCTCAGCACTCAGCCTTGGGATC 4850
human.FAM30B-LOC105372935-GGT2.NCBI.                                                 ------------------------------------------------------------ 2422
human.FAM230E-LOC105377182-GGT3P.NCBI.                                               ------------------------------------------------------------ 1497
human.FAM230J-LOC105372942-GGTLC5P.NCBI.rev.compl                                    ------------------------------------------------------------ 1867
human.FAM230A.ncRNA.18487127.18500594-GGTLC3.18516335.18518165.rev.compl.REF        ------------------------------------------------------------ 0

chimp.LOC112206744-LOC107973052-GGT2.revcompl                                        atgagtcactgtgcaagggagttccaacatctgcatgtatgtctggaatgatctgagcct 3345
chimp54546-135457.revcompl.Pan.troglodytes.clone.rp43-41g5.GenBank:AC099533.36      ATGAGTCACTGTGCAAGGGAGTTCCAACATCTGCATGTATGTCTGGAATGATCTGAGCCT 4910
human.FAM30B-LOC105372935-GGT2.NCBI.                                                 ------------------------------------------------------------ 2422
human.FAM230E-LOC105377182-GGT3P.NCBI.                                               ------------------------------------------------------------ 1497
human.FAM230J-LOC105372942-GGTLC5P.NCBI.rev.compl                                    ------------------------------------------------------------ 1867
human.FAM230A.ncRNA.18487127.18500594-GGTLC3.18516335.18518165.rev.compl.REF        ------------------------------------------------------------ 0
```

```
chimp.LOC112206744-LOC107973052-GGT2.revcompl    gtagagttcctacacactggccacattatagggtggtgtctgtggtcacacagctcaggg 3405
chimp54546-135457.revcompl.Pan.troglodytes.clone.rp43-41g5.GenBank:AC099533.36    GTAGAGTTCCTACACACTGGCCACATTATAGGGTGGTGTCTGTGGTCACACAGCTCAGGG 4970
human.FAM30B-LOC105372935-GGT2.NCBI.    ------------------------------------------------------------ 2422
human.FAM230E-LOC105377182-GGT3P.NCBI.    ------------------------------------------------------------ 1497
human.FAM230J-LOC105372942-GGTLC5P.NCBI.rev.compl    ------------------------------------------------------------ 1867
human.FAM230A.ncRNA.18487127.18500594-GGTLC3.18516335.18518165.rev.compl.REF    ------------------------------------------------------------ 0

chimp.LOC112206744-LOC107973052-GGT2.revcompl    caggtattagtacatgaatagcttagatgtgtcatagtctttatgtgaaaggcacataaa 3465
chimp54546-135457.revcompl.Pan.troglodytes.clone.rp43-41g5.GenBank:AC099533.36    CAGGTATTAGTACATGAATAGCTTAGCTGTGTCATAGTCTTTATGTGAAAGGCACATAAA 5030
human.FAM30B-LOC105372935-GGT2.NCBI.    ------------------------------------------------------------ 2422
human.FAM230E-LOC105377182-GGT3P.NCBI.    ------------------------------------------------------------ 1497
human.FAM230J-LOC105372942-GGTLC5P.NCBI.rev.compl    ------------------------------------------------------------ 1867
human.FAM230A.ncRNA.18487127.18500594-GGTLC3.18516335.18518165.rev.compl.REF    ------------------------------------------------------------ 0

chimp.LOC112206744-LOC107973052-GGT2.revcompl    agcccaaatgcttttgaactggttgcggattttgagttggaggacaacacttgggacaac 3525
chimp54546-135457.revcompl.Pan.troglodytes.clone.rp43-41g5.GenBank:AC099533.36    AGCCCAAATGCTTTTGAACTGGTTGCGGATTTTGAGTTGGAGGACAACACTTGGGACAAC 5090
human.FAM30B-LOC105372935-GGT2.NCBI.    ------------------------------------------------------------ 2422
human.FAM230E-LOC105377182-GGT3P.NCBI.    ------------------------------------------------------------ 1497
human.FAM230J-LOC105372942-GGTLC5P.NCBI.rev.compl    ------------------------------------------------------------ 1867
human.FAM230A.ncRNA.18487127.18500594-GGTLC3.18516335.18518165.rev.compl.REF    ------------------------------------------------------------ 0

chimp.LOC112206744-LOC107973052-GGT2.revcompl    cggtcacattccttcagtgcgcggccactcctcagcacatatcccaaagccggtaaataa 3585
chimp54546-135457.revcompl.Pan.troglodytes.clone.rp43-41g5.GenBank:AC099533.36    CGGTCACATTCCTTCAGTGCGCGGCCACTCCTCAGCACATATCCCAAAGCCGGTAAATAA 5150
human.FAM30B-LOC105372935-GGT2.NCBI.    ------------------------------------------------------------ 2422
human.FAM230E-LOC105377182-GGT3P.NCBI.    ------------------------------------------------------------ 1497
human.FAM230J-LOC105372942-GGTLC5P.NCBI.rev.compl    ------------------------------------------------------------ 1867
human.FAM230A.ncRNA.18487127.18500594-GGTLC3.18516335.18518165.rev.compl.REF    ------------------------------------------------------------ 0

chimp.LOC112206744-LOC107973052-GGT2.revcompl    tgggaagaccctgtactgatgtttgatggtgttatgaacttcaaatgacccctatagaaa 3645
chimp54546-135457.revcompl.Pan.troglodytes.clone.rp43-41g5.GenBank:AC099533.36    TGGGAAGACCCTGTACTGATGTTTGATGGTGTTATGAACTTCAAATGACCCCTATAGAAA 5210
human.FAM30B-LOC105372935-GGT2.NCBI.    ------------------------------------------------------------ 2422
human.FAM230E-LOC105377182-GGT3P.NCBI.    ------------------------------------------------------------ 1497
human.FAM230J-LOC105372942-GGTLC5P.NCBI.rev.compl    ------------------------------------------------------------ 1867
human.FAM230A.ncRNA.18487127.18500594-GGTLC3.18516335.18518165.rev.compl.REF    ------------------------------------------------------------ 0

chimp.LOC112206744-LOC107973052-GGT2.revcompl    tgttaaaaaaaaaaaaaaaaaaaaagtctgtgagctacacaacctggggacaagacaagt 3705
chimp54546-135457.revcompl.Pan.troglodytes.clone.rp43-41g5.GenBank:AC099533.36    TGTT--------AAAAAAAAAAAAAGTCTGTGAGCTACACAACCTGGGGACAAGACAAGT 5262
human.FAM30B-LOC105372935-GGT2.NCBI.    ------------------------------------------------------------ 2422
human.FAM230E-LOC105377182-GGT3P.NCBI.    ------------------------------------------------------------ 1497
human.FAM230J-LOC105372942-GGTLC5P.NCBI.rev.compl    ------------------------------------------------------------ 1867
human.FAM230A.ncRNA.18487127.18500594-GGTLC3.18516335.18518165.rev.compl.REF    ------------------------------------------------------------ 0

chimp.LOC112206744-LOC107973052-GGT2.revcompl    aagaacacaaaataccagtaaataatgagcctcatgtgtcaccctgcttgcaactccctc 3765
chimp54546-135457.revcompl.Pan.troglodytes.clone.rp43-41g5.GenBank:AC099533.36    AAGAACACAAAATACCACTAAATAATGAGCCTCATGTGTCACCCTGCTTGCAACTCCCTC 5322
human.FAM30B-LOC105372935-GGT2.NCBI.    ------------------------------------------------------------ 2422
human.FAM230E-LOC105377182-GGT3P.NCBI.    ------------------------------------------------------------ 1497
human.FAM230J-LOC105372942-GGTLC5P.NCBI.rev.compl    ------------------------------------------------------------ 1867
human.FAM230A.ncRNA.18487127.18500594-GGTLC3.18516335.18518165.rev.compl.REF    ------------------------------------------------------------ 0

chimp.LOC112206744-LOC107973052-GGT2.revcompl    ggcagctggtttgaagagcctcagtgctcaccaggggccatctccccgctctgccctctc 3825
chimp54546-135457.revcompl.Pan.troglodytes.clone.rp43-41g5.GenBank:AC099533.36    GGCAGCTGGTTTGAAGAGCCTCAGTGCTCACCAGGGGCCATCTCCCCGCTCTGCCCTCTC 5382
human.FAM30B-LOC105372935-GGT2.NCBI.    ------------------------------------------------------------ 2422
human.FAM230E-LOC105377182-GGT3P.NCBI.    ------------------------------------------------------------ 1497
human.FAM230J-LOC105372942-GGTLC5P.NCBI.rev.compl    ------------------------------------------------------------ 1867
human.FAM230A.ncRNA.18487127.18500594-GGTLC3.18516335.18518165.rev.compl.REF    ------------------------------------------------------------ 0

chimp.LOC112206744-LOC107973052-GGT2.revcompl    tcgctgtgggttgcctgggctgaacataggcagcccctcacatgatgactgcaaacacag 3885
chimp54546-135457.revcompl.Pan.troglodytes.clone.rp43-41g5.GenBank:AC099533.36    TCGCTGTGGGTTGCCTGGGCTGAACATAGGCAGCCCCTCACATGATGACTGCAAACACAG 5442
human.FAM30B-LOC105372935-GGT2.NCBI.    ------------------------------------------------------------ 2422
human.FAM230E-LOC105377182-GGT3P.NCBI.    ------------------------------------------------------------ 1497
human.FAM230J-LOC105372942-GGTLC5P.NCBI.rev.compl    ------------------------------------------------------------ 1867
human.FAM230A.ncRNA.18487127.18500594-GGTLC3.18516335.18518165.rev.compl.REF    ------------------------------------------------------------ 0

chimp.LOC112206744-LOC107973052-GGT2.revcompl    ggcaaaggaagactgaacggaaaatgtatggttatagtggttattttaaaatataagtat 3945
chimp54546-135457.revcompl.Pan.troglodytes.clone.rp43-41g5.GenBank:AC099533.36    GGCAAAGGAAGACTGAACGGAAAATGTATGGTTATAGTGGTTATTTTAAAATATAAGTAT 5502
human.FAM30B-LOC105372935-GGT2.NCBI.    ------------------------------------------------------------ 2422
human.FAM230E-LOC105377182-GGT3P.NCBI.    ------------------------------------------------------------ 1497
human.FAM230J-LOC105372942-GGTLC5P.NCBI.rev.compl    ------------------------------------------------------------ 1867
human.FAM230A.ncRNA.18487127.18500594-GGTLC3.18516335.18518165.rev.compl.REF    ------------------------------------------------------------ 0

chimp.LOC112206744-LOC107973052-GGT2.revcompl    ttcagtattttctaatctttaataattgattattttaggggaggtttttttttttttttt 4005
chimp54546-135457.revcompl.Pan.troglodytes.clone.rp43-41g5.GenBank:AC099533.36    TTCAGTATTTTCTAATCTTTAATAATTGATTATTTTAGGGGAGG--TTTTTTTTTTTTTT 5560
human.FAM30B-LOC105372935-GGT2.NCBI.    ------------------------------------------------------------ 2422
human.FAM230E-LOC105377182-GGT3P.NCBI.    ------------------------------------------------------------ 1497
human.FAM230J-LOC105372942-GGTLC5P.NCBI.rev.compl    ------------------------------------------------------------ 1867
human.FAM230A.ncRNA.18487127.18500594-GGTLC3.18516335.18518165.rev.compl.REF    ------------------------------------------------------------ 0

chimp.LOC112206744-LOC107973052-GGT2.revcompl    tttgagacagagtcttgcgctgttgcccaggctggagtgcagtggcatgatctcagctca 4065
chimp54546-135457.revcompl.Pan.troglodytes.clone.rp43-41g5.GenBank:AC099533.36    TTTGAGACAGAGTCTTGCACTGTTGCCCAGGCTGGAGTGCAGTGGCATGATCTCAGCTCA 5620
human.FAM30B-LOC105372935-GGT2.NCBI.    ------------------------------------------------------------ 2422
human.FAM230E-LOC105377182-GGT3P.NCBI.    ------------------------------------------------------------ 1497
human.FAM230J-LOC105372942-GGTLC5P.NCBI.rev.compl    ------------------------------------------------------------ 1867
human.FAM230A.ncRNA.18487127.18500594-GGTLC3.18516335.18518165.rev.compl.REF    ------------------------------------------------------------ 0

chimp.LOC112206744-LOC107973052-GGT2.revcompl    ctgcaacctctacctcctgtgttcaagcgattctcctgcctcagcctcccgagtagctgg 4125
chimp54546-135457.revcompl.Pan.troglodytes.clone.rp43-41g5.GenBank:AC099533.36    CTGCAACCTCTGCCTCCTGTGTTCAAGCGATTCTCCTGCCTCAGCCTCCCGAGTAGCTGG 5680
human.FAM30B-LOC105372935-GGT2.NCBI.    ------------------------------------------------------------ 2422
human.FAM230E-LOC105377182-GGT3P.NCBI.    ------------------------------------------------------------ 1497
human.FAM230J-LOC105372942-GGTLC5P.NCBI.rev.compl    ------------------------------------------------------------ 1867
human.FAM230A.ncRNA.18487127.18500594-GGTLC3.18516335.18518165.rev.compl.REF    ------------------------------------------------------------ 0

chimp.LOC112206744-LOC107973052-GGT2.revcompl    gactacaggcacgtgccaccacgcctggcaaagttttgtgtttttagtagagacggggt 4185
chimp54546-135457.revcompl.Pan.troglodytes.clone.rp43-41g5.GenBank:AC099533.36    GACTACAGGCACGTGCCACCACGCCTGGCAAAGTTTTGTGTTTTTAGTAGAGACGGGGT 5740
human.FAM30B-LOC105372935-GGT2.NCBI.    ------------------------------------------------------------ 2422
human.FAM230E-LOC105377182-GGT3P.NCBI.    ------------------------------------------------------------ 1497
human.FAM230J-LOC105372942-GGTLC5P.NCBI.rev.compl    ------------------------------------------------------------ 1867
human.FAM230A.ncRNA.18487127.18500594-GGTLC3.18516335.18518165.rev.compl.REF    ------------------------------------------------------------ 0

chimp.LOC112206744-LOC107973052-GGT2.revcompl    ttcactctgttagccaggatggtctcaatcttctgacctcatgatccacccgcctcggcc 4245
chimp54546-135457.revcompl.Pan.troglodytes.clone.rp43-41g5.GenBank:AC099533.36    TTCACTCTGTTAGCCAGGATGGTCTCAATCTTCTGACCTCATGATCCACCCGCCTCGGCC 5800
human.FAM30B-LOC105372935-GGT2.NCBI.    ------------------------------------------------------------ 2422
human.FAM230E-LOC105377182-GGT3P.NCBI.    ------------------------------------------------------------ 1497
human.FAM230J-LOC105372942-GGTLC5P.NCBI.rev.compl    ------------------------------------------------------------ 1867
human.FAM230A.ncRNA.18487127.18500594-GGTLC3.18516335.18518165.rev.compl.REF    ------------------------------------------------------------ 0

chimp.LOC112206744-LOC107973052-GGT2.revcompl    tcccaaagtgctgggattacaggtgtgagccaccacacctggcctgatttttattttt 4305
chimp54546-135457.revcompl.Pan.troglodytes.clone.rp43-41g5.GenBank:AC099533.36    TCCCAAAGTGCTGGGATTACAGGTGTGAGCCACCACACCTGGCCTGATTTTTACTTTTT 5860
human.FAM30B-LOC105372935-GGT2.NCBI.    ------------------------------------------------------------ 2422
human.FAM230E-LOC105377182-GGT3P.NCBI.    ------------------------------------------------------------ 1497
human.FAM230J-LOC105372942-GGTLC5P.NCBI.rev.compl    ------------------------------------------------------------ 1867
human.FAM230A.ncRNA.18487127.18500594-GGTLC3.18516335.18518165.rev.compl.REF    ------------------------------------------------------------ 0

chimp.LOC112206744-LOC107973052-GGT2.revcompl    tatttttgagatgaagtctctgtcacccaggctggagtgcagtggtgcaatctcagctc 4365
chimp54546-135457.revcompl.Pan.troglodytes.clone.rp43-41g5.GenBank:AC099533.36    TATTTTTGAGATGAAGTCTCTGTCACCCAGGCTGGAGTGCAGTGGTGCAATCTCAGCTC 5920
human.FAM30B-LOC105372935-GGT2.NCBI.    ------------------------------------------------------------ 2422
human.FAM230E-LOC105377182-GGT3P.NCBI.    ------------------------------------------------------------ 1497
human.FAM230J-LOC105372942-GGTLC5P.NCBI.rev.compl    ------------------------------------------------------------ 1867
human.FAM230A.ncRNA.18487127.18500594-GGTLC3.18516335.18518165.rev.compl.REF    ------------------------------------------------------------ 0

chimp.LOC112206744-LOC107973052-GGT2.revcompl    actgcaatctctgcctccaggttcaagggattgtcctgcctcagcctcctgagtagctgg 4425
chimp54546-135457.revcompl.Pan.troglodytes.clone.rp43-41g5.GenBank:AC099533.36    ACTGCAATCTCTGCCTCCAGGTTCAAGGGATTCTCCTGCCTCAGCCTCCTGAGTAGCTGG 5980
human.FAM30B-LOC105372935-GGT2.NCBI.    ------------------------------------------------------------ 2422
human.FAM230E-LOC105377182-GGT3P.NCBI.    ------------------------------------------------------------ 1497
human.FAM230J-LOC105372942-GGTLC5P.NCBI.rev.compl    ------------------------------------------------------------ 1867
human.FAM230A.ncRNA.18487127.18500594-GGTLC3.18516335.18518165.rev.compl.REF    ------------------------------------------------------------ 0
```

```
chimp.LOC112206744-LOC107973052-GGT2.revcompl                                     ----gtttttttttttttttagttgagatggggtttaccatgttggtcaggctggtctcg 4481
chimp54546-135457.revcompl.Pan.troglodytes.clone.rp43-41g5.GenBank:AC099533.36    TTTTTTTTTTTTTTTTTTTTAGTTGAGATGGGGTTTACCATGTTGGTCAGGCTGGTCTCG 6040
human.FAM30B-LOC105372935-GGT2.NCBI.                                              --------------------------gatggggtttaccatgttggtcaggctggtctcg 2457
human.FAM230E-LOC105377182-GGT3P.NCBI.                                            --------------------------gatggggtttaccatgttggtcaggctggtctcg 1532
human.FAM230J-LOC105372942-GGTLC5P.NCBI.rev.compl                                 --------------------------gatggggtttaccatgttggtcaggctggtctcg 1902
human.FAM230A.ncRNA.18487127.18500594-GGTLC3.18516335.18518165.rev.compl.REF      ------------------------------------------------------------ 0

chimp.LOC112206744-LOC107973052-GGT2.revcompl                                     aactcctgacctcaaatgatccgcccacctccaccttccaaactgctgggattacaggtg 4541
chimp54546-135457.revcompl.Pan.troglodytes.clone.rp43-41g5.GenBank:AC099533.36    AACTCCTGACCTCAAATGATCCGCCCACCTCCACCTTCCAAACTGCTGGGATTACAGGTG 6100
human.FAM30B-LOC105372935-GGT2.NCBI.                                              aactcctgacctcaaatgatccacccacctccaccttccaaactgctgggattacaggtg 2517
human.FAM230E-LOC105377182-GGT3P.NCBI.                                            aactcctgacctcaaatgatccacccacctccaccttccaaactgctgggattacaggtg 1592
human.FAM230J-LOC105372942-GGTLC5P.NCBI.rev.compl                                 aactcctgacctcaaatgatccacccacctccaccttccaaactgctgggattacaggtg 1962
human.FAM230A.ncRNA.18487127.18500594-GGTLC3.18516335.18518165.rev.compl.REF      ------------------------------------------------------------ 0

chimp.LOC112206744-LOC107973052-GGT2.revcompl                                     tgagccactatgcctggctgattattttcataaccaagaaaagaaataaatataattaat 4601
chimp54546-135457.revcompl.Pan.troglodytes.clone.rp43-41g5.GenBank:AC099533.36    TGAGCCACTATGCCTGGCTGATTATTTTCATAACCAAGAAAAGAAATAAATATAATTAAT 6160
human.FAM30B-LOC105372935-GGT2.NCBI.                                              tgagccactatgcctgactgattattttcataaccaagaaaagaaataaatacaattaat 2577
human.FAM230E-LOC105377182-GGT3P.NCBI.                                            tgagccactatgcctgactgattattttcataaccaagaaaagaaataaatacaattaat 1652
human.FAM230J-LOC105372942-GGTLC5P.NCBI.rev.compl                                 tgagccactatgcctgactgattattttcataaccaagaaaagaaataaatacaattaat 2022
human.FAM230A.ncRNA.18487127.18500594-GGTLC3.18516335.18518165.rev.compl.REF      ------------------------------------------------------------ 0

chimp.LOC112206744-LOC107973052-GGT2.revcompl                                     gctggtgcatggtattaaatctagtttttaaaaaattcacacataaaccaggcagaaccc 4661
chimp54546-135457.revcompl.Pan.troglodytes.clone.rp43-41g5.GenBank:AC099533.36    GCTGGTGCATGGTATTAAATCTAGTTTTTAAAAAAATCACACATAAACCAGGCAGAACCC 6220
human.FAM30B-LOC105372935-GGT2.NCBI.                                              gctggtgcatggtattaaatctagtttttaaaaaattcacacataaacaaggcagaaccc 2637
human.FAM230E-LOC105377182-GGT3P.NCBI.                                            gctggtgcatggtattaaatctagtttttaaaaaattcacacataaacaaggcagaaccc 1712
human.FAM230J-LOC105372942-GGTLC5P.NCBI.rev.compl                                 gctggtgcatggtattaaatctagtttttaaaaaattcacacataaacagggcagaaccc 2082
human.FAM230A.ncRNA.18487127.18500594-GGTLC3.18516335.18518165.rev.compl.REF      ------------------------------------------------------------ 0

chimp.LOC112206744-LOC107973052-GGT2.revcompl                                     tataccctccatgataaatgcagtagcagtgtatgtgggtctgtggaggttgaaagggac 4721
chimp54546-135457.revcompl.Pan.troglodytes.clone.rp43-41g5.GenBank:AC099533.36    TATACCCTCCATGATAAATGCAGTAGCAGTGTATGTGGGTCTGTGGAGGTTGAAAGGGAC 6280
human.FAM30B-LOC105372935-GGT2.NCBI.                                              tataccctccatgataaatgcagtagcagtgtatgtgggtctgtggaggttgaaagggac 2697
human.FAM230E-LOC105377182-GGT3P.NCBI.                                            tataccctccatgataaatgcagtagcagtgtatgtgggtctgtggaggttgaaagggac 1772
human.FAM230J-LOC105372942-GGTLC5P.NCBI.rev.compl                                 tataccctccatgataaatgcagtagcagtgtatgtgggtctgtggaggttgaaagggac 2142
human.FAM230A.ncRNA.18487127.18500594-GGTLC3.18516335.18518165.rev.compl.REF      ------------------------------------------------------------ 0

chimp.LOC112206744-LOC107973052-GGT2.revcompl                                     ttggtagatgtcaagaaggtagtggcagtcctgctgggcttttaaagggtctgaagaagt 4781
chimp54546-135457.revcompl.Pan.troglodytes.clone.rp43-41g5.GenBank:AC099533.36    TTGGTAGATGTCAAGAAGGTAGTGGCAGTCCTGCTGGGCTTTTAAAGGGTCTGAAGAAGT 6340
human.FAM30B-LOC105372935-GGT2.NCBI.                                              ttggtagatgtcaagaagctagtggcagtcctgctgggcttttaaagggtctgaagaagt 2757
human.FAM230E-LOC105377182-GGT3P.NCBI.                                            ttggtagatgtcaagaaggtagtggcagtcctgctgggcttttaaagggtctgaagaagt 1832
human.FAM230J-LOC105372942-GGTLC5P.NCBI.rev.compl                                 ttggtcgatgtcaagaaggtagtggcagtcctgctgggcttttaaagggtctgaagaagt 2202
human.FAM230A.ncRNA.18487127.18500594-GGTLC3.18516335.18518165.rev.compl.REF      ------------------------------------------------------------ 0

chimp.LOC112206744-LOC107973052-GGT2.revcompl                                     gacaggatgctgtggttgaatcctagcatgtattttagcatttgttcatttggagtttga 4841
chimp54546-135457.revcompl.Pan.troglodytes.clone.rp43-41g5.GenBank:AC099533.36    GACAGGATGCTGTGGTTGAATCCTAGCATGTATTTTAGCATTTGTTCATTTGGAGTTTGA 6400
human.FAM30B-LOC105372935-GGT2.NCBI.                                              gacaggatgctgtggttgaatcctagcatgtattttagcatttgttcatttggag-ttga 2816
human.FAM230E-LOC105377182-GGT3P.NCBI.                                            gacaggatgctgtggttgaatcctagcatgtattttagcatttgttcatttggag-ttga 1891
human.FAM230J-LOC105372942-GGTLC5P.NCBI.rev.compl                                 gacaggatgctgtggttgaatcgtagcatgtgttttagcatttgttcatttggag-ttga 2261
human.FAM230A.ncRNA.18487127.18500594-GGTLC3.18516335.18518165.rev.compl.REF      ------------------------------------------------------------ 0

chimp.LOC112206744-LOC107973052-GGT2.revcompl                                     ttatttcacgttgctttcatttgccattacctggaaagccaagggctctactctcatttc 4901
chimp54546-135457.revcompl.Pan.troglodytes.clone.rp43-41g5.GenBank:AC099533.36    TTATTTCACGTTGCTTTCATTTGCCATTACCTGGAAAGCCAAGGGCTGTACTCTTATTTC 6460
human.FAM30B-LOC105372935-GGT2.NCBI.                                              ttatttcacgttgctttcatttgccattacctggaaagccaagggctctactctcatttc 2876
human.FAM230E-LOC105377182-GGT3P.NCBI.                                            ttatttcacgttgctttcatttgccattacctggaaagccaagggctctactctcatttc 1951
human.FAM230J-LOC105372942-GGTLC5P.NCBI.rev.compl                                 ttatttcacgttgctttcatttgccattacctggaaagccaagggctctactctcatttc 2321
human.FAM230A.ncRNA.18487127.18500594-GGTLC3.18516335.18518165.rev.compl.REF      ------------------------------------------------------------ 0

chimp.LOC112206744-LOC107973052-GGT2.revcompl                                     cttgctgctctttctttgccttccttggtccatgaagaagatggtccaggagaagctcat 4961
chimp54546-135457.revcompl.Pan.troglodytes.clone.rp43-41g5.GenBank:AC099533.36    CTTGCTACTCTTTCTTTGCCTTCCTTGGTCCATGAAGAAGATGGTCCAGGAGAAGCTCAT 6520
human.FAM30B-LOC105372935-GGT2.NCBI.                                              cttgctgctctttctttgccttccttggtccgtgaagaagatggtccaggagaagctcat 2936
human.FAM230E-LOC105377182-GGT3P.NCBI.                                            cttgctgctctttctttgccttccttggtccgtgaagaagatggtccaggagaagctcat 2011
human.FAM230J-LOC105372942-GGTLC5P.NCBI.rev.compl                                 cttgctgctctttctttgccttccttggtccgtgaagaagatggtccaggagaagctcat 2381
human.FAM230A.ncRNA.18487127.18500594-GGTLC3.18516335.18518165.rev.compl.REF      ------------------------------------------------------------ 0

chimp.LOC112206744-LOC107973052-GGT2.revcompl                                     tccatgcttgttaaccaggcacacccctaagttccagtccctgagtcattcatgagtagc 5021
chimp54546-135457.revcompl.Pan.troglodytes.clone.rp43-41g5.GenBank:AC099533.36    TCCATGCTTGTTAACCAGGCACACCCCTAAGTTCCAGTCCCTGAGTCATTCATGAGTAGC 6580
human.FAM30B-LOC105372935-GGT2.NCBI.                                              tccatgcttgttaaccaggcacgcccctaagttccagtccctgagtcattcatgagtagc 2996
human.FAM230E-LOC105377182-GGT3P.NCBI.                                            tccatgcttgttaaccaggcacgcccctaagttccagtccctgagtcattcatgagtagc 2071
human.FAM230J-LOC105372942-GGTLC5P.NCBI.rev.compl                                 tccatgcttgttaaccaggcacgcccctaagttccagtccctgagtcattcatgagtagc 2441
human.FAM230A.ncRNA.18487127.18500594-GGTLC3.18516335.18518165.rev.compl.REF      ------------------------------------------------------------ 0

chimp.LOC112206744-LOC107973052-GGT2.revcompl                                     actgccaatgaactgacagccatgctgtgtccctccacatcccctaggtgactcgaagaa 5081
chimp54546-135457.revcompl.Pan.troglodytes.clone.rp43-41g5.GenBank:AC099533.36    ACTGCCAATGAACTGACAGCCGTGCTGTGTCCCTCCACATCCCCTAGGTGACTCGAAGAA 6640
human.FAM30B-LOC105372935-GGT2.NCBI.                                              actgccaatgaactgacagccatgctgtgtccctccacatcccctaggtgactcgaagaa 3056
human.FAM230E-LOC105377182-GGT3P.NCBI.                                            actgccaatgaactgacagccatgctgtgtccctccacatcccctaggtgactcgaagaa 2131
human.FAM230J-LOC105372942-GGTLC5P.NCBI.rev.compl                                 actgccaatgaactgacagccatgctgtgtccctccacatcccctaggtgactcgaagaa 2501
human.FAM230A.ncRNA.18487127.18500594-GGTLC3.18516335.18518165.rev.compl.REF      ------------------------------------------------------------ 0

chimp.LOC112206744-LOC107973052-GGT2.revcompl                                     gccttccaaaaagcatgtgaaaagggagccctcctctactaccaaggtaaagtagcctgt 5141
chimp54546-135457.revcompl.Pan.troglodytes.clone.rp43-41g5.GenBank:AC099533.36    GCCTTCCAAAAAGCATGTGAAAAGGGAGCCCTCCTCTACTACCAAGGTAAAGTAGCCTGT 6700
human.FAM30B-LOC105372935-GGT2.NCBI.                                              gccttccaaaaagcatgtgaaaaggaagccctactctactaccaaggtaaagtagcctgt 3116
human.FAM230E-LOC105377182-GGT3P.NCBI.                                            gccttccaaaaagcgtgtgaaaaggaagccctactctactaccaaggtaaagtagcctgt 2191
human.FAM230J-LOC105372942-GGTLC5P.NCBI.rev.compl                                 gccttccaaaaagcgtgtgaaaaggaagccctactctactaccaaggtaaagtagcctgt 2561
human.FAM230A.ncRNA.18487127.18500594-GGTLC3.18516335.18518165.rev.compl.REF      ------------------------------------------------------------ 0

chimp.LOC112206744-LOC107973052-GGT2.revcompl                                     ctttgcctaagatgtaaatgttgttttcttggatcctttatttttcagttgatatctgct 5201
chimp54546-135457.revcompl.Pan.troglodytes.clone.rp43-41g5.GenBank:AC099533.36    CTTTGCCTAAGATGTAAATGTTGTTTTCTTGGATCCTTTATTTTTCAGTTGATATCAGCT 6760
human.FAM30B-LOC105372935-GGT2.NCBI.                                              ctttgcctaagatgtaaatgttgttttcttggatcctttatttttcagttgatatcagct 3176
human.FAM230E-LOC105377182-GGT3P.NCBI.                                            ctttgcctaagatgtaaatgttgttttcttggatcctttatttttcagttgatatcagct 2251
human.FAM230J-LOC105372942-GGTLC5P.NCBI.rev.compl                                 ctttgcctaagatgtaaatgttgttttcttggatcctttatttttcagttgatatcagct 2621
human.FAM230A.ncRNA.18487127.18500594-GGTLC3.18516335.18518165.rev.compl.REF      ------------------------------------------------------------ 0

chimp.LOC112206744-LOC107973052-GGT2.revcompl                                     atgggaaaattatccactacattatagatgttagataatatttccttggggatggaggag 5261
chimp54546-135457.revcompl.Pan.troglodytes.clone.rp43-41g5.GenBank:AC099533.36    ATGGGAAAATTATCCACTACATTATAGATGTTAGATAATATTTCCTTGGGAATGGAGGAG 6820
human.FAM30B-LOC105372935-GGT2.NCBI.                                              atgggaaaattctccactacattataggtgttagataatatttccttggggatggaggag 3236
human.FAM230E-LOC105377182-GGT3P.NCBI.                                            atgggaaaattctccactacattataggtgttagataatatttccttggggatggaggag 2311
human.FAM230J-LOC105372942-GGTLC5P.NCBI.rev.compl                                 atgggaaaattctccactacattataggtgttagataatatttccttggggatggaggag 2681
human.FAM230A.ncRNA.18487127.18500594-GGTLC3.18516335.18518165.rev.compl.REF      ------------------------------------------------------------ 0

chimp.LOC112206744-LOC107973052-GGT2.revcompl                                     gtgtattttaccaactgacacctgattccagaggatgtgcaaaattggcagtgtcagata 5321
chimp54546-135457.revcompl.Pan.troglodytes.clone.rp43-41g5.GenBank:AC099533.36    GTGTATTTTACCAACTGACACCTGATTCCAGAGGACGTGCAAAATTGGCAGTGTCAGATA 6880
human.FAM30B-LOC105372935-GGT2.NCBI.                                              gtgtattttaccaactgacacctgattccagaggacgtgcaaaattggcagtgtcagata 3296
human.FAM230E-LOC105377182-GGT3P.NCBI.                                            gtgtattttaccaactgacacctgattccagaggacgtgcaaaattggcagtgtcagata 2371
human.FAM230J-LOC105372942-GGTLC5P.NCBI.rev.compl                                 gtgtattttaccaactgacacctgattccagaggacgtgcaaaattggcagtgtcagata 2741
human.FAM230A.ncRNA.18487127.18500594-GGTLC3.18516335.18518165.rev.compl.REF      ------------------------------------------------------------ 0

chimp.LOC112206744-LOC107973052-GGT2.revcompl                                     gtacactgcgtgttaagggatgttttcttcaggaacaagctttccactttagataagaat 5381
chimp54546-135457.revcompl.Pan.troglodytes.clone.rp43-41g5.GenBank:AC099533.36    GTACACTGCGTGTTAAGGGATGTTTTCTTCAGGAACAAGCTTTCCACTTTAGATAAGAAT 6940
human.FAM30B-LOC105372935-GGT2.NCBI.                                              gtacactgggtgttaagggatgttttcttcaggaacaagctttccactttagataagaat 3356
human.FAM230E-LOC105377182-GGT3P.NCBI.                                            gtacactgggtgttaagggatgttttcttcaggaacaagctttccactttagataagaat 2431
human.FAM230J-LOC105372942-GGTLC5P.NCBI.rev.compl                                 gtacactgggtgttaagggatgttttcttcaggaacaagctttccactttagataagaat 2801
human.FAM230A.ncRNA.18487127.18500594-GGTLC3.18516335.18518165.rev.compl.REF      ------------------------------------------------------------ 0

chimp.LOC112206744-LOC107973052-GGT2.revcompl                                     tctgcaattgctactcaaaaattacctagacagaaacattcttcaagaaaagctcctgtg 5441
chimp54546-135457.revcompl.Pan.troglodytes.clone.rp43-41g5.GenBank:AC099533.36    TCTGCAATTGCTACTCAAAAATTACCTAGACAGAAACATTCTTCAAGAAAAGCTCCTGTG 7000
human.FAM30B-LOC105372935-GGT2.NCBI.                                              tctgcaattgctactcaaaaattacctagacagaaacattcttcaagaaaagctcctgtg 3416
human.FAM230E-LOC105377182-GGT3P.NCBI.                                            tctgcaattgctactcaaaaattacctagacagaaacattcttcaagaaaagctcctgtg 2491
human.FAM230J-LOC105372942-GGTLC5P.NCBI.rev.compl                                 tctgcaattgctactcaaaaattacctagacagaaacattcttcaagaaaagctcctgtg 2861
human.FAM230A.ncRNA.18487127.18500594-GGTLC3.18516335.18518165.rev.compl.REF      ------------------------------------------------------------ 0

chimp.LOC112206744-LOC107973052-GGT2.revcompl                                     ctttcctaagggaactctactctagagttggggcttttgacttgaaccttatttccaatc 5501
chimp54546-135457.revcompl.Pan.troglodytes.clone.rp43-41g5.GenBank:AC099533.36    CTTTCCTAAGGGAACTCTACTCTAGAGTTGGGGCTTTTGACTTGAACCTTATTTCCAATC 7060
human.FAM30B-LOC105372935-GGT2.NCBI.                                              ctttcctaagggaactctactctagagttggggcttttgacttgaaccttatttccagtc 3476
human.FAM230E-LOC105377182-GGT3P.NCBI.                                            ctttcctaagggaactctactctagagttggggcttttgacttgaaccttatttccagtc 2551
human.FAM230J-LOC105372942-GGTLC5P.NCBI.rev.compl                                 ctttcctaagggaactctactctagagttggggcttttgacttgaaccttatttccaatc 2921
human.FAM230A.ncRNA.18487127.18500594-GGTLC3.18516335.18518165.rev.compl.REF      ------------------------------------------------------------ 0
```

```
chimp.LOC112206744-LOC107973052-GGT2.revcompl                                        ttggttacccagagtttccaagtgaacaaaagacctgtgtgagccatccatagcatagcc 5561
chimp54546-135457.revcompl.Pan.troglodytes.clone.rp43-41g5.GenBank:AC099533.36       TTGGTTACCCAGAGTTTCCAAGTGAACAAAAGACCTGTGTGAGCCATCCATAGCATAGCC 7120
human.FAM30B-LOC105372935-GGT2.NCBI.                                                 ttggttacccagagtttccaagtgaacaaaagacctgtgtgagccatccatagcatagcc 3536
human.FAM230E-LOC105377182-GGT3P.NCBI.                                               ttggttacccagagtttccaagtgaacaaaagacctgtgtgagccatccatagcatagcc 2611
human.FAM230J-LOC105372942-GGTLC5P.NCBI.rev.compl                                    ttggttacccagagtttccaagtgaacaaaagacctgtgtgagccatccatagcatagcc 2981
human.FAM230A.ncRNA.18487127.18500594-GGTLC3.18516335.18518165.rev.compl.REF         ------------------------------------------------------------ 0

chimp.LOC112206744-LOC107973052-GGT2.revcompl                                        tgattctcagagtgttttccttctctaattacaggtgacttcagggagcacattcaatgg 5621
chimp54546-135457.revcompl.Pan.troglodytes.clone.rp43-41g5.GenBank:AC099533.36       TGATTCTCAGAGTGTTTTCCTTCTCTAATTACAGGTGACTTCAGGGAGCACATTCAATGG 7180
human.FAM30B-LOC105372935-GGT2.NCBI.                                                 tgattctcagagtgttttccttctctaattacaggtgacttcagggagcacattcaatgg 3596
human.FAM230E-LOC105377182-GGT3P.NCBI.                                               tgattctcagagtgttttccttctctaattacaggtgacttcagggagcacattcaatgg 2671
human.FAM230J-LOC105372942-GGTLC5P.NCBI.rev.compl                                    tgattctcagagtgttttccttctctaattacaggtgacttcagggagcacattcaatgg 3041
human.FAM230A.ncRNA.18487127.18500594-GGTLC3.18516335.18518165.rev.compl.REF         ------------------------------------------------------------ 0

chimp.LOC112206744-LOC107973052-GGT2.revcompl                                        tacgtattctggaatcactcactggttgttagaaaaggattctataggaaatctggagct 5681
chimp54546-135457.revcompl.Pan.troglodytes.clone.rp43-41g5.GenBank:AC099533.36       TACGTATTCTGGAATCACTCACTGGTTGTTAGAAAAGGATTCTATAGGAAATCTGGAGCT 7240
human.FAM30B-LOC105372935-GGT2.NCBI.                                                 tacgtattctggaatcactcactggttgttagaaaaggattctacaggaaatctggagct 3656
human.FAM230E-LOC105377182-GGT3P.NCBI.                                               tacgtattctggaatcactcactggttgttagaaaaggattctacaggaaatctggagct 2731
human.FAM230J-LOC105372942-GGTLC5P.NCBI.rev.compl                                    tacgtattctggaatcactcactggttgttagaaaaggattctacaggaaatctggagct 3101
human.FAM230A.ncRNA.18487127.18500594-GGTLC3.18516335.18518165.rev.compl.REF         ------------------------------------------------------------ 0

chimp.LOC112206744-LOC107973052-GGT2.revcompl                                        taactgctggcttttgtctagagaggctccatgatccaagacatctggtgggaatgagga 5741
chimp54546-135457.revcompl.Pan.troglodytes.clone.rp43-41g5.GenBank:AC099533.36       TAACTGCTGGCTTTTGTCTGGAAAGCCTCCATGATCCAAGACATCTGGTGGGAATGAGGA 7300
human.FAM30B-LOC105372935-GGT2.NCBI.                                                 taactgctggcttttgtctggagagcctccatgatccaagacatctggtgggaatgagga 3716
human.FAM230E-LOC105377182-GGT3P.NCBI.                                               taactgctggcttttgtctggagagcctccatgatccaagacatctggtgggaatgagga 2791
human.FAM230J-LOC105372942-GGTLC5P.NCBI.rev.compl                                    taactgctggcttttgtccggagagcctccatgatccaagacatctggtgggaatgagga 3161
human.FAM230A.ncRNA.18487127.18500594-GGTLC3.18516335.18518165.rev.compl.REF         ------------------------------------------------------------ 0

chimp.LOC112206744-LOC107973052-GGT2.revcompl                                        tgtgggatatagtaaaggaaactggttttccaggtgacatactctttttatctatgtata 5801
chimp54546-135457.revcompl.Pan.troglodytes.clone.rp43-41g5.GenBank:AC099533.36       TGTGGGGTATAGTAAAGGAAACTGGTTTTCCAGGTGACATACTCTTTTTATCTACGTATA 7360
human.FAM30B-LOC105372935-GGT2.NCBI.                                                 tgtagggtatagtaaaagaaactggttttcctggtgacatactctttttatctatgtata 3776
human.FAM230E-LOC105377182-GGT3P.NCBI.                                               tgtagggtatagtaaaagaaactggttttcctggtgacatactctttttatctatgtata 2851
human.FAM230J-LOC105372942-GGTLC5P.NCBI.rev.compl                                    tgtagggtatagtaaaagaaactggttttcctggtgacatactctttttatctatgtata 3221
human.FAM230A.ncRNA.18487127.18500594-GGTLC3.18516335.18518165.rev.compl.REF         ------------------------------------------------------------ 0

chimp.LOC112206744-LOC107973052-GGT2.revcompl                                        gtttctgggaatgtgttcacattaggttgtgtgtgggtatgtgtgtattagggcgggggt 5861
chimp54546-135457.revcompl.Pan.troglodytes.clone.rp43-41g5.GenBank:AC099533.36       GTTTCTGGGAATGTGTTCACATTAGGTTGTGTGTGGGTATGTGTGTATTAGGGCGGGGGT 7420
human.FAM30B-LOC105372935-GGT2.NCBI.                                                 gtttctgggaacatgttcacattaggttgtgtgtgggtatgtgtgtattagggcgggggt 3836
human.FAM230E-LOC105377182-GGT3P.NCBI.                                               gtttctgggaacatgttcacattaggttgtgtgtgggtatgtgtgtattagggcgggggt 2911
human.FAM230J-LOC105372942-GGTLC5P.NCBI.rev.compl                                    gtttctgggaacatgttcacattaggttgtgtgtgggtatgtgtgtattagggcgggggt 3281
human.FAM230A.ncRNA.18487127.18500594-GGTLC3.18516335.18518165.rev.compl.REF         ------------------------------------------------------------ 0

chimp.LOC112206744-LOC107973052-GGT2.revcompl                                        gggatgaggtggtctgtgtgcacatctgcatgatttgcttgtgaatgtgtgtctatgtgt 5921
chimp54546-135457.revcompl.Pan.troglodytes.clone.rp43-41g5.GenBank:AC099533.36       GGGGTGAGGTGGTCTGTGTGCACGTCTGCATGATTTGCTTGTGAATGTGTGTCTATGTGT 7480
human.FAM30B-LOC105372935-GGT2.NCBI.                                                 ggggtgaggtggtctgtgtgcaagtctgcatgatttgcttgtgaatgtgtgtctatgtgt 3896
human.FAM230E-LOC105377182-GGT3P.NCBI.                                               ggggtgaggtggtctgtgtgcaagtctgcatgatttgcttgtgaatgtgtgtctatgtgt 2971
human.FAM230J-LOC105372942-GGTLC5P.NCBI.rev.compl                                    ggggtgaggtggtctgtgtgcaagtctgcatgatttgcttgtgaatgtgtgtctatctgt 3341
human.FAM230A.ncRNA.18487127.18500594-GGTLC3.18516335.18518165.rev.compl.REF         ------------------------------------------------------------ 0

chimp.LOC112206744-LOC107973052-GGT2.revcompl                                        gtttcccccaggaaaaaaatgttgtgtttacccagcacaactctcagtgccatttttctt 5981
chimp54546-135457.revcompl.Pan.troglodytes.clone.rp43-41g5.GenBank:AC099533.36       GTTTCCCCCAGGAAAAAAATGTTGTGTTTACCCAGCACAACTCTCAGTGCCATTTTTCTT 7540
human.FAM30B-LOC105372935-GGT2.NCBI.                                                 gtttcccctaggaaaaaaatgttgtgtttacccagcacaactctcagtgccatttttctt 3956
human.FAM230E-LOC105377182-GGT3P.NCBI.                                               gtttcccctaggaaaaaaatgttgtgtttacccagcacaactctcagtgccatgtttctt 3031
human.FAM230J-LOC105372942-GGTLC5P.NCBI.rev.compl                                    gtttcccccaggaaaaaaatgttgtgtttacccagcacaactctcagtgccatttttctt 3401
human.FAM230A.ncRNA.18487127.18500594-GGTLC3.18516335.18518165.rev.compl.REF         ------------------------------------------------------------ 0

chimp.LOC112206744-LOC107973052-GGT2.revcompl                                        aatttaacaaatcagaccacatactttacttacattagttcacacctcatcatcatcatg 6041
chimp54546-135457.revcompl.Pan.troglodytes.clone.rp43-41g5.GenBank:AC099533.36       AATTTAACAAATCAGACCACATACTTTACTTACATTAGTTCACACCTCATCATCATCATG 7600
human.FAM30B-LOC105372935-GGT2.NCBI.                                                 aatttaacaaatcagaccacatactttacttacattagttcacacctcatcatcatcatg 4016
human.FAM230E-LOC105377182-GGT3P.NCBI.                                               aatttaacaaatcagaccacatactttacttacattagttcacacctcatcatcatcatg 3091
human.FAM230J-LOC105372942-GGTLC5P.NCBI.rev.compl                                    aatttaacaaatcagaccacatactttacttacattagttcacacctcatcatcatcatg 3461
human.FAM230A.ncRNA.18487127.18500594-GGTLC3.18516335.18518165.rev.compl.REF         ------------------------------------------------------------ 0

chimp.LOC112206744-LOC107973052-GGT2.revcompl                                        cccatatgttgtgagcttgtttattgagcccacatgccagatggagaaactaagccacat 6101
chimp54546-135457.revcompl.Pan.troglodytes.clone.rp43-41g5.GenBank:AC099533.36       CCCATATGTCGTGAGCTTGTTTATTGAGCCCACATGCCAGATGGAGAAACTAAGCCACAT 7660
human.FAM30B-LOC105372935-GGT2.NCBI.                                                 cccatatgttgtgagcttgtttattgagcccacatgccagatggagaaactcagccacat 4076
human.FAM230E-LOC105377182-GGT3P.NCBI.                                               cccatatgttgtgagcttgtttattgagcccacatgccagatggagaaactaagccacat 3151
human.FAM230J-LOC105372942-GGTLC5P.NCBI.rev.compl                                    cccatatgttgtgagcttgtttattgagcccacatgccagatggagaaactaagccacat 3521
human.FAM230A.ncRNA.18487127.18500594-GGTLC3.18516335.18518165.rev.compl.REF         ------------------------------------------------------------ 0

chimp.LOC112206744-LOC107973052-GGT2.revcompl                                        aaataaatgtgccctggttcacttgctgcatagtgaagagtcaaaatgtttactcatacg 6161
chimp54546-135457.revcompl.Pan.troglodytes.clone.rp43-41g5.GenBank:AC099533.36       AAATAAATGTGCCCTGGTTCACTTGCTGCATAGTGAAGAGTCAAAATGTTTACTCATACG 7720
human.FAM30B-LOC105372935-GGT2.NCBI.                                                 aaataaatgtgccctggttcacttgctgcatagtgaagagtcaaaatgtttactcatacg 4136
human.FAM230E-LOC105377182-GGT3P.NCBI.                                               aaataaatgtgccctggttcacttgctgcatagtgaagagtcaaaatgtttactcaaacg 3211
human.FAM230J-LOC105372942-GGTLC5P.NCBI.rev.compl                                    aaataaatgtgctctggttcacttgctgcatagtgaagagtcaaaatgtttcctcatacg 3581
human.FAM230A.ncRNA.18487127.18500594-GGTLC3.18516335.18518165.rev.compl.REF         ------------------------------------------------------------ 0

chimp.LOC112206744-LOC107973052-GGT2.revcompl                                        gtgctaatgttgaaggcctgaactacaacctctatttatcagccagtgaagagatcacta 6221
chimp54546-135457.revcompl.Pan.troglodytes.clone.rp43-41g5.GenBank:AC099533.36       GTGCTAATGTTGAAGGCCTGAACTACAACCTCTATTTATCAGCCAGTGAAGAGATCACTA 7780
human.FAM30B-LOC105372935-GGT2.NCBI.                                                 gtgctaatgttgaaggcctgaactacaacctctatttatcagccagtgaagagatcacta 4196
human.FAM230E-LOC105377182-GGT3P.NCBI.                                               gtgctaatgttgaaggcctgaactacaacctctatttatcagccagtgaagagatcacta 3271
human.FAM230J-LOC105372942-GGTLC5P.NCBI.rev.compl                                    gtgctaatgttgaaggcctgaactacaacctctatttatcagccagtgaagagatcacta 3641
human.FAM230A.ncRNA.18487127.18500594-GGTLC3.18516335.18518165.rev.compl.REF         ------------------------------------------------------------ 0

chimp.LOC112206744-LOC107973052-GGT2.revcompl                                        ttcaccatgcaagggagttccagcaccctctatgcctggaattacccacgcctgcagaga 6281
chimp54546-135457.revcompl.Pan.troglodytes.clone.rp43-41g5.GenBank:AC099533.36       TTCACCATGCAAGGGAGTTCCAGCACCCTCTATGCCTGGAATTACCCATGCCTGCAGAGA 7840
human.FAM30B-LOC105372935-GGT2.NCBI.                                                 ttccccatgcaagggagttccagcaccctctatgcctggaattacccacacctgcagaga 4256
human.FAM230E-LOC105377182-GGT3P.NCBI.                                               ttcaccatgcaagggagttccagcaccctctatgcctggaattacccacgcctgcagaga 3331
human.FAM230J-LOC105372942-GGTLC5P.NCBI.rev.compl                                    ttcaccatgcaagggagttccagcaccctctatgcctggaattacccacgcctgcagaga 3701
human.FAM230A.ncRNA.18487127.18500594-GGTLC3.18516335.18518165.rev.compl.REF         ------------------------------------------------------------ 0

chimp.LOC112206744-LOC107973052-GGT2.revcompl                                        tcccaaacgccatccctcacataagagagcctcatgatctcataatccaggtagctatgt 6341
chimp54546-135457.revcompl.Pan.troglodytes.clone.rp43-41g5.GenBank:AC099533.36       TCCCAAACGCCATCCCTCACATAAGAGAGCCTCATGATCTCATAATCCAGGTAGCTATGT 7900
human.FAM30B-LOC105372935-GGT2.NCBI.                                                 tcccaaacgccatccctcacataagacagcctcatgatctcataatccaggtagctatgt 4316
human.FAM230E-LOC105377182-GGT3P.NCBI.                                               tcccaaacgccatccctcacataagacagcctcatgatctcataatccaggtagctatgt 3391
human.FAM230J-LOC105372942-GGTLC5P.NCBI.rev.compl                                    tcccaaacgccatccctcacataagacagcctcatgatctcataatccaggtagctatgt 3761
human.FAM230A.ncRNA.18487127.18500594-GGTLC3.18516335.18518165.rev.compl.REF         ------------------------------------------------------------ 0

chimp.LOC112206744-LOC107973052-GGT2.revcompl                                        agacatcttcctgcaggtgtcacatagtccttagtgggaaaccaacatagaaagcccatg 6401
chimp54546-135457.revcompl.Pan.troglodytes.clone.rp43-41g5.GenBank:AC099533.36       AGACATCTTCCTGCAGGTGTCACATAGTCCTTAGTGTGAAACCAACATAGAAAGCCCATG 7960
human.FAM30B-LOC105372935-GGT2.NCBI.                                                 agacatcttcctgcaggtgtcacatagtccttagtgtgaaaccaacatagaaagcccatg 4376
human.FAM230E-LOC105377182-GGT3P.NCBI.                                               agacatcttcctgcaggtgtcacatagtccttagtgtgaaaccaacatagaaagcccatg 3451
human.FAM230J-LOC105372942-GGTLC5P.NCBI.rev.compl                                    agacatcttcctgcaggtgtcacatagtccttagtgtgaaaccaacatagaaagcccatg 3821
human.FAM230A.ncRNA.18487127.18500594-GGTLC3.18516335.18518165.rev.compl.REF         ------------------------------------------------------------ 0

chimp.LOC112206744-LOC107973052-GGT2.revcompl                                        tttctgatcaaatcacaggttctgaaacactaagggaagcactaagtaggacaatgtggt 6461
chimp54546-135457.revcompl.Pan.troglodytes.clone.rp43-41g5.GenBank:AC099533.36       TTTCTGATCAAATCACAGGTTCTGAAACACTAAGTGAGGCACTAAGTAGGACAATGTGGT 8020
human.FAM30B-LOC105372935-GGT2.NCBI.                                                 tttctgatcaaatcacaggttctgaaacactaagggaggcactaagtaggacaacgtggt 4436
human.FAM230E-LOC105377182-GGT3P.NCBI.                                               tttctgatcaaatcacaggttctgaaacactaagggaggcactaagtaggacaacgtggt 3511
human.FAM230J-LOC105372942-GGTLC5P.NCBI.rev.compl                                    tttctgatcaaatcacaggttctgaaacactaagggaggcactaagtaggacaacgtggt 3881
human.FAM230A.ncRNA.18487127.18500594-GGTLC3.18516335.18518165.rev.compl.REF         ------------------------------------------------------------ 0

chimp.LOC112206744-LOC107973052-GGT2.revcompl                                        gcctgtgtgtcatagctgggtctcctcaagacatggatcaagtccaataagaattgggga 6521
chimp54546-135457.revcompl.Pan.troglodytes.clone.rp43-41g5.GenBank:AC099533.36       GCCTGCATGTCATAGCTGGGTCTCCTCAAGACATGGATCAAGTCCAATAAGAATTGGAGA 8080
human.FAM30B-LOC105372935-GGT2.NCBI.                                                 gcctgcgtgtcatagctgggtctcctcaagacatggatcaagtccagtaagaattgggga 4496
human.FAM230E-LOC105377182-GGT3P.NCBI.                                               gcctgcgtgtcatagctgggtctcctcaagacatggatcaagtccagtaagaattgggga 3571
human.FAM230J-LOC105372942-GGTLC5P.NCBI.rev.compl                                    gcctgcgtgtcatagctgggtctcctcaagacatggatcaagtccagtaagaattgggga 3941
human.FAM230A.ncRNA.18487127.18500594-GGTLC3.18516335.18518165.rev.compl.REF         ------------------------------------------------------------ 0

chimp.LOC112206744-LOC107973052-GGT2.revcompl                                        gatgctttagagtcttgatggagttatcaccacaagccctctgagctacacactttaggg 6581
chimp54546-135457.revcompl.Pan.troglodytes.clone.rp43-41g5.GenBank:AC099533.36       GATGCTTTAGAGTCTTGATGGAGTTATCACCACAAGCCCTCTGAGCTACACACTTTAGGG 8140
human.FAM30B-LOC105372935-GGT2.NCBI.                                                 gatgctttagagtcttgatggagttatcaccacaagccctctgagctacacactttaggg 4556
human.FAM230E-LOC105377182-GGT3P.NCBI.                                               gatgctttagagtcttgatggagttatcaccacaagccctctgagctacacactttaggg 3631
human.FAM230J-LOC105372942-GGTLC5P.NCBI.rev.compl                                    gatgctttagagtcttgatggagttatcaccacaagccctctgagctacacactttaggg 4001
human.FAM230A.ncRNA.18487127.18500594-GGTLC3.18516335.18518165.rev.compl.REF         ------------------------------------------------------------ 0

chimp.LOC112206744-LOC107973052-GGT2.revcompl                                        atcatgaccattaagtactcaaattaccacttggttgttatccgggtatccgtcgtcctt 6641
```

```
chimp54546-135457.revcompl.Pan.troglodytes.clone.rp43-41g5.GenBank:AC099533.36      ATCATGACCATTAAGTACTCAAATTACCATTTGGTTGTTATCTGGGTATCCGTCGTCCTT 8200
human.FAM30B-LOC105372935-GGT2.NCBI.                                                 atcatgaccattaagtactcaaattaccatttggttgctatccgggtatccgtcgtcctt 4616
human.FAM230E-LOC105377182-GGT3P.NCBI.                                               atcatgaccattaagtactcaaattaccatttggttgttatccgggtatccgtcgtcctt 3691
human.FAM230J-LOC105372942-GGTLC5P.NCBI.rev.compl                                    atcatgaccattaagtactcaaattaccatttggttgttatccgggtatccgtcgtcctt 4061
human.FAM230A.ncRNA.18487127.18500594-GGTLC3.18516335.18518165.rev.compl.REF         ------------------------------------------------------------ 0

chimp.LOC112206744-LOC107973052-GGT2.revcompl                                        gtggcaactctcttgtgaagctggtgtggacagcctcagtgctggagctgtgcctgcctt 6701
chimp54546-135457.revcompl.Pan.troglodytes.clone.rp43-41g5.GenBank:AC099533.36      GTGGCAACTCTCTTGTGAAGCTGGTGTGGACAGTCTCAGTGCTGGAGCTGTGCCTGCCTT 8260
human.FAM30B-LOC105372935-GGT2.NCBI.                                                 gtggcaaccctcttgtgaagctggtgtggacagcctcagtgctggagctgtgcctgcctt 4676
human.FAM230E-LOC105377182-GGT3P.NCBI.                                               gtggcaaccctcttgtgaagctggtgtggacagcctcagtgctggagctgtgcctgcctt 3751
human.FAM230J-LOC105372942-GGTLC5P.NCBI.rev.compl                                    gtggcaaccctcttgtgaagctggtgtggacagcctcagtgctggagctgtgcctccctt 4121
human.FAM230A.ncRNA.18487127.18500594-GGTLC3.18516335.18518165.rev.compl.REF         ------------------------------------------------------------ 0

chimp.LOC112206744-LOC107973052-GGT2.revcompl                                        ctgaatggaccctttctgtgttagcaggtgggtacaagcatggggggtcagcacactcagt 6761
chimp54546-135457.revcompl.Pan.troglodytes.clone.rp43-41g5.GenBank:AC099533.36      CTGAATGGACCCTTTCTGTGTTAGCAGGTGGGTACAAGCATGGGGGTCAGCACACTCAGT 8320
human.FAM30B-LOC105372935-GGT2.NCBI.                                                 ctgagtggaccctttctgtgttagcaggtgggtacaagcgtgggggtcagcacactcagt 4736
human.FAM230E-LOC105377182-GGT3P.NCBI.                                               ctgagtggaccctttctgtgttagcaggtgggtacaagcgtgggggtcagcacactcagt 3811
human.FAM230J-LOC105372942-GGTLC5P.NCBI.rev.compl                                    ctgagtggaccctttctgtgttagcaggtgggtacaagcgtgggggtcagcacactcagt 4181
human.FAM230A.ncRNA.18487127.18500594-GGTLC3.18516335.18518165.rev.compl.REF         ------------------------------------------------------------ 0

chimp.LOC112206744-LOC107973052-GGT2.revcompl                                        ggatttacacacacagcattgaagagtaaggctgggcttcattatttatacattttcaat 6821
chimp54546-135457.revcompl.Pan.troglodytes.clone.rp43-41g5.GenBank:AC099533.36      GGATTTACACACACAGCATTGAAGAGTAAGGCTGGGCTTCATTATTTATACATTTTCAAT 8380
human.FAM30B-LOC105372935-GGT2.NCBI.                                                 ggatttacacacacagcgttgaagagtaaggctgggcttcattatttatacattttcaat 4796
human.FAM230E-LOC105377182-GGT3P.NCBI.                                               ggatttacacacacagcgttgaagagtaaggctgggcttcgttatttatacattttcaat 3871
human.FAM230J-LOC105372942-GGTLC5P.NCBI.rev.compl                                    ggatttacacacacagcgttgaagagtaaggctgggcttcattatttatacattttcaat 4241
human.FAM230A.ncRNA.18487127.18500594-GGTLC3.18516335.18518165.rev.compl.REF         ------------------------------------------------------------ 0

chimp.LOC112206744-LOC107973052-GGT2.revcompl                                        aaatgatgatcttcataacataaaatcaatgacgtagtacactagaatactgtccctagt 6881
chimp54546-135457.revcompl.Pan.troglodytes.clone.rp43-41g5.GenBank:AC099533.36      AAATGATGATCTTCATAACATAAAATCAATGATGTAGTACACTAGAATACTGTCCCTAGT 8440
human.FAM30B-LOC105372935-GGT2.NCBI.                                                 aaatgatgatcttcataacataaaatcaatgatgtagtacactagaatactgtccctagt 4856
human.FAM230E-LOC105377182-GGT3P.NCBI.                                               aaatgatgatcttcataacataaaatcaatgatgtagtacactagaatactgtccctagt 3931
human.FAM230J-LOC105372942-GGTLC5P.NCBI.rev.compl                                    aaatgatgatcttcataacataaaatcaatgatgtagtacactagaatactgtccctagt 4301
human.FAM230A.ncRNA.18487127.18500594-GGTLC3.18516335.18518165.rev.compl.REF         ------------------------------------------------------------ 0

chimp.LOC112206744-LOC107973052-GGT2.revcompl                                        attgaatcttgtctctcaacaaagggttgcttaaagtcacatgacagattccattcaact 6941
chimp54546-135457.revcompl.Pan.troglodytes.clone.rp43-41g5.GenBank:AC099533.36      ATTGAATCTTGTCTCTCAACAAAGGGTTGCTTAAAGTCACATGACAGATTCCATTCAACT 8500
human.FAM30B-LOC105372935-GGT2.NCBI.                                                 attgaatcttgtctctcagcaaagggttgcttaaagtcacgtgacagattccattcaact 4916
human.FAM230E-LOC105377182-GGT3P.NCBI.                                               attgaatcttgtctctcagcaaagggttgcttaaagtcacgtgacagattccattcaact 3991
human.FAM230J-LOC105372942-GGTLC5P.NCBI.rev.compl                                    attgaatcttgtctctcagcaaagggttgcttaaagtcacgtgacagattccattcaact 4361
human.FAM230A.ncRNA.18487127.18500594-GGTLC3.18516335.18518165.rev.compl.REF         ------------------------------------------------------------ 0

chimp.LOC112206744-LOC107973052-GGT2.revcompl                                        gatgacacatgctgtagcagcagttaaagcagtcatttgaaaaggcttttactataaact 7001
chimp54546-135457.revcompl.Pan.troglodytes.clone.rp43-41g5.GenBank:AC099533.36      GACGACACATGCTGTAGCAGCAGTTAAAGCAGTCATTTGAAAAGGCTTTTACTATAAACT 8560
human.FAM30B-LOC105372935-GGT2.NCBI.                                                 gatgacacatgctgtagcagcagttaaagcagtcatttgaaaaggcttttactataaact 4976
human.FAM230E-LOC105377182-GGT3P.NCBI.                                               gatgacacatgctgtagcagcagttaaagcagtcatttgaaaaggcttttactataaact 4051
human.FAM230J-LOC105372942-GGTLC5P.NCBI.rev.compl                                    gatgacacatgctgtagcagcagttaaagcagtcatttgaaaaggcttttactataaact 4421
human.FAM230A.ncRNA.18487127.18500594-GGTLC3.18516335.18518165.rev.compl.REF         ------------------------------------------------------------ 0

chimp.LOC112206744-LOC107973052-GGT2.revcompl                                        tatgtgtgagcctgaagtgggggataaaagaggtgattagctcccctgtgccatgtttct 7061
chimp54546-135457.revcompl.Pan.troglodytes.clone.rp43-41g5.GenBank:AC099533.36      TATGTGTGAGCCTGAAGTGGGGGATAAAAGAGGTGATTAGCTCCCCTGTGCCATGTTTCT 8620
human.FAM30B-LOC105372935-GGT2.NCBI.                                                 tacgtgtgagcctgaagtgggggataaaagaggcgattagctcccctgtgccatgtttct 5036
human.FAM230E-LOC105377182-GGT3P.NCBI.                                               tatgtgtgagcctgaagtgggggataaaagaggtgattagctcccctgtgccatgtttct 4111
human.FAM230J-LOC105372942-GGTLC5P.NCBI.rev.compl                                    tatgtgtgagcctgaagtgggggataaaagaggcgattagctcccctgtgccatgtttct 4481
human.FAM230A.ncRNA.18487127.18500594-GGTLC3.18516335.18518165.rev.compl.REF         ------------------------------------------------------------ 0

chimp.LOC112206744-LOC107973052-GGT2.revcompl                                        attatgtgcatggtggaggaaaattacacaggaaggtgatggagagaacagagcaaagga 7121
chimp54546-135457.revcompl.Pan.troglodytes.clone.rp43-41g5.GenBank:AC099533.36      ATTATGTGCGTGGTGGAGGAAAATTACACAGGAAGGTGATGGAGAGAACAGAGCAAAGGT 8680
human.FAM30B-LOC105372935-GGT2.NCBI.                                                 attatgtgcgtggtggaggaaaattacacaggaaggtgatggagagaacagagcaaagga 5096
human.FAM230E-LOC105377182-GGT3P.NCBI.                                               gttatgtgcgtggtggaggaaaattacacaggaaggtgatggagagaacagagcaaagga 4171
human.FAM230J-LOC105372942-GGTLC5P.NCBI.rev.compl                                    attatgtgcgtggtggaggaaaattacacaggaaggtgatggagagaacagagcaaagga 4541
human.FAM230A.ncRNA.18487127.18500594-GGTLC3.18516335.18518165.rev.compl.REF         ------------------------------------------------------------ 0

chimp.LOC112206744-LOC107973052-GGT2.revcompl                                        ttggacaggtccattgaacccataagactatggtgaggttagtgaatgagattggtcatt 7181
chimp54546-135457.revcompl.Pan.troglodytes.clone.rp43-41g5.GenBank:AC099533.36      TTGGACGGGTCCATTGAACCCATAAGACTATGGTGAGGTTAGTGAATGAGATTGGTCATT 8740
human.FAM30B-LOC105372935-GGT2.NCBI.                                                 ttggacaggtccattgaacccataagactatggtgaggttagtgaatgagattggtcatt 5156
human.FAM230E-LOC105377182-GGT3P.NCBI.                                               ttggacaggtccattgaacccataagactatggtgaggttagtgaatgagactggtcatt 4231
human.FAM230J-LOC105372942-GGTLC5P.NCBI.rev.compl                                    ttggacaggtccattgaacccataagactatggtgaggttagtgaatgagattggtcatt 4601
human.FAM230A.ncRNA.18487127.18500594-GGTLC3.18516335.18518165.rev.compl.REF         ------------------------------------------------------------ 0

chimp.LOC112206744-LOC107973052-GGT2.revcompl                                        ttaggtcaaattctacccagagctggtgcagccactgcccattcttagccagaccttatt 7241
chimp54546-135457.revcompl.Pan.troglodytes.clone.rp43-41g5.GenBank:AC099533.36      TTAGGTCAAATTTTACCCAGAGCTGGTGCAGCCACTGCCCATTCTTAGCCAGACCTTATT 8800
human.FAM30B-LOC105372935-GGT2.NCBI.                                                 ttaggtcaaattttacccagagctggtgcagccactgcccattcttagccagaccttatt 5216
human.FAM230E-LOC105377182-GGT3P.NCBI.                                               ttaggtcaaattttacccagagctggtacagccactgccgattcttagccagaccttatt 4291
human.FAM230J-LOC105372942-GGTLC5P.NCBI.rev.compl                                    ttaggtcaaattttacccagagctggtgcagccactgcccattcttagccagaccttatt 4661
human.FAM230A.ncRNA.18487127.18500594-GGTLC3.18516335.18518165.rev.compl.REF         ------------------------------------------------------------ 0

chimp.LOC112206744-LOC107973052-GGT2.revcompl                                        gcaggcagctctgatcaatagtcaaggaggcagtggggcttgcagacttaattcattaaa 7301
chimp54546-135457.revcompl.Pan.troglodytes.clone.rp43-41g5.GenBank:AC099533.36      GCAGGCAGCTCTGATCAATAGTCAAGGAGGCAGTGGGGGTTGCAGACTTAATTCATTAAA 8860
human.FAM30B-LOC105372935-GGT2.NCBI.                                                 gcaggcagctctgatcaatagtcaaggaggcagtggggcttgcagacttaattcattaaa 5276
human.FAM230E-LOC105377182-GGT3P.NCBI.                                               gcaggcagctctgatcaatagtcaaggaggcagtgggggttgcagactttattcattaaa 4351
human.FAM230J-LOC105372942-GGTLC5P.NCBI.rev.compl                                    gcaggcagctctgatcaatagtcaaggaggcagtggggcttgcagacttaattcattaaa 4721
human.FAM230A.ncRNA.18487127.18500594-GGTLC3.18516335.18518165.rev.compl.REF         ------------------------------------------------------------ 0

chimp.LOC112206744-LOC107973052-GGT2.revcompl                                        tcaccaaagcaccagcccacacggccacttttccagttaattcacagtagcttgcatatt 7361
chimp54546-135457.revcompl.Pan.troglodytes.clone.rp43-41g5.GenBank:AC099533.36      TCACCAAAGCACCAGCCCACACGGCCACTTTTCCAGTTAATTCACAGTAGCTTGCATATT 8920
human.FAM30B-LOC105372935-GGT2.NCBI.                                                 tcaccaaagcaccagcccaaacggccacttttccagttaattgacagtagcttgcatatt 5336
human.FAM230E-LOC105377182-GGT3P.NCBI.                                               tcaccaaagcaccagcccacacggccacttttccagttaattcacagtagcttgcatatt 4411
human.FAM230J-LOC105372942-GGTLC5P.NCBI.rev.compl                                    tcaccaaagcaccagcccacacggccacttttccagttaattgacagtagcttgcatatt 4781
human.FAM230A.ncRNA.18487127.18500594-GGTLC3.18516335.18518165.rev.compl.REF         ------------------------------------------------------------ 0

chimp.LOC112206744-LOC107973052-GGT2.revcompl                                        caggtttgatcagtggaagggaagttactctttgcagacccatcttttgacaatcatttt 7421
chimp54546-135457.revcompl.Pan.troglodytes.clone.rp43-41g5.GenBank:AC099533.36      CAGGTTTGATCAGTGGAAGGGAAGTTACTCTTTGCAGACCCATCTTTTGACAATCATTTT 8980
human.FAM30B-LOC105372935-GGT2.NCBI.                                                 caggtttgatcagtggaagggaagttactctttgcagacccatcttttgacaatcatttt 5396
human.FAM230E-LOC105377182-GGT3P.NCBI.                                               caggtttgatcagtggaagggaagttactctttgcagactcat-ttttgacaatcatttt 4470
human.FAM230J-LOC105372942-GGTLC5P.NCBI.rev.compl                                    caggtttgatcagtggaagggaagttactctttgcagacccat-ttttgacaatcatttt 4840
human.FAM230A.ncRNA.18487127.18500594-GGTLC3.18516335.18518165.rev.compl.REF         ------------------------------------------------------------ 0

chimp.LOC112206744-LOC107973052-GGT2.revcompl                                        gcagtgtcagaaggtctgagcagcctcgggaggcaagcagtccctggtccctcagtgtag 7481
chimp54546-135457.revcompl.Pan.troglodytes.clone.rp43-41g5.GenBank:AC099533.36      GCAGTGTCAGAAGGTCTGAGCAGCCTCGGGAGGCAAGCAGTCCCTGGTCCCTCAGTGTAG 9040
human.FAM30B-LOC105372935-GGT2.NCBI.                                                 gtggtgtcggaaggtctgagcagcctcgggaggcaagcagtccctggtccctcagtgtag 5456
human.FAM230E-LOC105377182-GGT3P.NCBI.                                               gcggtgtcagaaggtctgagcagcctcgggaggcaagcagtccctggtccctcagtgtag 4530
human.FAM230J-LOC105372942-GGTLC5P.NCBI.rev.compl                                    gcggtgtcagaaggtgtgagcagcctcgggaggcaagcagtccctggtccctcagtgtag 4900
human.FAM230A.ncRNA.18487127.18500594-GGTLC3.18516335.18518165.rev.compl.REF         ------------------------------------------------------------ 0

chimp.LOC112206744-LOC107973052-GGT2.revcompl                                        tcactggaggagacagtcactgagaggcagctggcagggtgaagggaaaggggaggcagg 7541
chimp54546-135457.revcompl.Pan.troglodytes.clone.rp43-41g5.GenBank:AC099533.36      TCACTGGAGGAGACAGTCACTGAGAGGCAGCTGGCAGGGTGAAGGGAAAGGGGAGGCAGG 9100
human.FAM30B-LOC105372935-GGT2.NCBI.                                                 tcactggaggagacagtcactgagaggcagctggcagggtgaagggaaaggggaggcagg 5516
human.FAM230E-LOC105377182-GGT3P.NCBI.                                               tcactggaggagacagtcactgagaggcagctggcagggtgaagggaaaggggaggcagg 4590
human.FAM230J-LOC105372942-GGTLC5P.NCBI.rev.compl                                    tcactggaggagacagtcactgagaggcagctggcagggtgaagggcaaggggaggcagg 4960
human.FAM230A.ncRNA.18487127.18500594-GGTLC3.18516335.18518165.rev.compl.REF         ------------------------------------------------------------ 0

chimp.LOC112206744-LOC107973052-GGT2.revcompl                                        ccacagagatgacagcctttaagctgtcatactgg-gaggtcaaggatctgaaagaggaa 7600
chimp54546-135457.revcompl.Pan.troglodytes.clone.rp43-41g5.GenBank:AC099533.36      CCACAGAGATGACAGCCTTTAAGCTGTCATACTGG-GAGGTCAAGGATCTGAAAGAGGAA 9159
human.FAM30B-LOC105372935-GGT2.NCBI.                                                 ccacagagatgacagcctttaagctgtcatactgg-gaggtcaaggatctgaaagaggaa 5575
human.FAM230E-LOC105377182-GGT3P.NCBI.                                               ccacagagatgacagtctttaagctgtcatactggtgaggtcaaggatctgaaagaggaa 4650
human.FAM230J-LOC105372942-GGTLC5P.NCBI.rev.compl                                    ccacagagatgacagcctttaagctgtcatactgg-gaggtcaaggatctgaaagaggaa 5019
human.FAM230A.ncRNA.18487127.18500594-GGTLC3.18516335.18518165.rev.compl.REF         ------------------------------------------------------------ 0

chimp.LOC112206744-LOC107973052-GGT2.revcompl                                        ggagaattctttatcattaaggacctgtccttatctcaggcatttcctccagagcatcac 7660
chimp54546-135457.revcompl.Pan.troglodytes.clone.rp43-41g5.GenBank:AC099533.36      GGAGAATTCTTTATCATTAAGGACCTGTCCTTATCTCAGGCATTTCCTCCAGAGCATCAC 9219
human.FAM30B-LOC105372935-GGT2.NCBI.                                                 ggagaattctttatcattaaggacctgtccttatctcaggcatttcctccagagcatcac 5635
human.FAM230E-LOC105377182-GGT3P.NCBI.                                               ggagaattctttatcattaaggacctgtccttatctcaggcatttcctccagagcatcac 4710
human.FAM230J-LOC105372942-GGTLC5P.NCBI.rev.compl                                    ggagaattctttatcattaaggacctgtccttatctcaggcatttcctccagagcaccac 5079
human.FAM230A.ncRNA.18487127.18500594-GGTLC3.18516335.18518165.rev.compl.REF         ------------------------------------------------------------ 0

chimp.LOC112206744-LOC107973052-GGT2.revcompl                                        ctttgtccacccacacaccttgggctaggaggactggagaaagacagtgaggggtctctt 7720
chimp54546-135457.revcompl.Pan.troglodytes.clone.rp43-41g5.GenBank:AC099533.36      TTTTGTCCACCCACACACCTTGGGCTAGGAGGACTGGAGAAAGACAGTGAGGGGTCTCTT 9279
```

```
human.FAM30B-LOC105372935-GGT2.NCBI.                                               ctttgtccacccacacaccttgggctaggaggactggagaaagacagtgaggggtctctt 5695
human.FAM230E-LOC105377182-GGT3P.NCBI.                                             ctttgtccacccacacaccttgggctaggaggactggagaaagacagtgaggggtctctt 4770
human.FAM230J-LOC105372942-GGTLC5P.NCBI.rev.compl                                  ctttgtccacccacacaccttgggctaggaggactggagaaagacagtgaggggtctctt 5139
human.FAM230A.ncRNA.18487127.18500594-GGTLC3.18516335.18518165.rev.compl.REF       ------------------------------------------------------------ 0

chimp.LOC112206744-LOC107973052-GGT2.revcompl                                      gggtctctggcacagggcgtgatgaagaggtggcagtttttcaggaatctctctctctag 7780
chimp54546-135457.revcompl.Pan.troglodytes.clone.rp43-41g5.GenBank:AC099533.36     GGGTCTCTGGCACAGGGCGTGATGAAGAGGTGGCAGTTTTTCAGGAATCTCTCTCTCTAG 9339
human.FAM30B-LOC105372935-GGT2.NCBI.                                               gggtctctggcacagggcgtgatgaagaggtggcagtttttcaggaatctctctctctag 5755
human.FAM230E-LOC105377182-GGT3P.NCBI.                                             gggtctctggcacagggcgtgatgaagaggtggcagtttttcaggaatctctctctctag 4830
human.FAM230J-LOC105372942-GGTLC5P.NCBI.rev.compl                                  gggtctgtggcacagggcgtgatgaagaggtggcagtttttcaggaatctctctctctag 5199
human.FAM230A.ncRNA.18487127.18500594-GGTLC3.18516335.18518165.rev.compl.REF       ------------------------------------------------------------ 0

chimp.LOC112206744-LOC107973052-GGT2.revcompl                                      ggaaccaaatacatttcccatctcagctccttcactcagcggggttgaggttctgctcgt 7840
chimp54546-135457.revcompl.Pan.troglodytes.clone.rp43-41g5.GenBank:AC099533.36     GGAACCAAATACATTTCCCATCTCAGGTCCTTCACTCAGCGGGGTTGAGGTTCTGCTCGT 9399
human.FAM30B-LOC105372935-GGT2.NCBI.                                               ggaaccaaatacatttcccatctcaggtccttcactcagcggggttgaggttctgctcgt 5815
human.FAM230E-LOC105377182-GGT3P.NCBI.                                             ggaaccaaatacatttcccatctcaggtccttcactcagcggggttgaggttctgctcgt 4890
human.FAM230J-LOC105372942-GGTLC5P.NCBI.rev.compl                                  ggaaccaaatacatttcccatctcaggtccttcactcagcggggttgaggttctgctcgt 5259
human.FAM230A.ncRNA.18487127.18500594-GGTLC3.18516335.18518165.rev.compl.REF       ------------------------------------------------------------ 0

chimp.LOC112206744-LOC107973052-GGT2.revcompl                                      cacttatcatctctgaatgtcagcaccctcaagtgtaaaatctcagccacagccccttct 7900
chimp54546-135457.revcompl.Pan.troglodytes.clone.rp43-41g5.GenBank:AC099533.36     CACTTATCATCTCTGAATGTCAGCACCCTCAAGTGTAAAATCTCAGCCACAGCCCCTCCT 9459
human.FAM30B-LOC105372935-GGT2.NCBI.                                               cacttatcatctctgaatgtcagcaccctcaagtgtaaaatctcagccacagcccctcct 5875
human.FAM230E-LOC105377182-GGT3P.NCBI.                                             cacttatcatctctgaatgtcagcaccctcaagtgtaaaatctcagccacagcccctcct 4950
human.FAM230J-LOC105372942-GGTLC5P.NCBI.rev.compl                                  cacttatcatctctgaatgtcagcaccctcaagtgtaaaatctcagccacagcccctcct 5319
human.FAM230A.ncRNA.18487127.18500594-GGTLC3.18516335.18518165.rev.compl.REF       ------------------------------------------------------------ 0

chimp.LOC112206744-LOC107973052-GGT2.revcompl                                      ctgcacccccctgcagggctgatgttctccataaaccataaggcatcatgcccacggaaaa 7960
chimp54546-135457.revcompl.Pan.troglodytes.clone.rp43-41g5.GenBank:AC099533.36     CTGCACCCCCTGCAGGGCTGATGTTCTCCATAAACCATAAGGCATCATGCCCACGGAAAA 9519
human.FAM30B-LOC105372935-GGT2.NCBI.                                               ctgcaccccctgcagggctgatgttctccataaaccataaggcgtcatgcccacggaaaa 5935
human.FAM230E-LOC105377182-GGT3P.NCBI.                                             ctgcaccccctgcagggctgatgttctccataaaccataaggcgtcatgcccacggaaaa 5010
human.FAM230J-LOC105372942-GGTLC5P.NCBI.rev.compl                                  ctgcaccccctgcagggctgatgttctccataaaccataaggcgtcatgcccacggaaaa 5379
human.FAM230A.ncRNA.18487127.18500594-GGTLC3.18516335.18518165.rev.compl.REF       ------------------------------------------------------------ 0

chimp.LOC112206744-LOC107973052-GGT2.revcompl                                      gccgaacaggaaagcatgctccactgccccagagccatccaagttcccccctccatattcc 8020
chimp54546-135457.revcompl.Pan.troglodytes.clone.rp43-41g5.GenBank:AC099533.36     GCCGAACAGGAAAGCATGCTCCACTGCCCCGGAGCCATCCAAGTTCCCCCTCCATATTCC 9579
human.FAM30B-LOC105372935-GGT2.NCBI.                                               gccgaacaggaaagcatgctccactgccccggagtcatgcaagttcccccctccatattcc 5995
human.FAM230E-LOC105377182-GGT3P.NCBI.                                             gccgaacaggaaagcatgctccactgccccggagccatccaagttcccccctccatattcc 5070
human.FAM230J-LOC105372942-GGTLC5P.NCBI.rev.compl                                  gccgaacaggaaagcatgctccactgccccggagccatccaagttcccccctccatattcc 5439
human.FAM230A.ncRNA.18487127.18500594-GGTLC3.18516335.18518165.rev.compl.REF       ------------------------------------------------------------ 0

chimp.LOC112206744-LOC107973052-GGT2.revcompl                                      gccactgctaagtgtccagcctattcctcctggcatgtagtaaacacttagagaacatta 8080
chimp54546-135457.revcompl.Pan.troglodytes.clone.rp43-41g5.GenBank:AC099533.36     GCCACTGCTAAGTGTCCAGCCTATTCCTCCTGGCATGTAGTAAACACTTAGAGAACATTA 9639
human.FAM30B-LOC105372935-GGT2.NCBI.                                               gccactgctaagtgtccagcttattcctcctggcatgtagtaaacacttagagaacatta 6055
human.FAM230E-LOC105377182-GGT3P.NCBI.                                             gccactgctaagtgtccagcttattcctcctggcctgtagtaaacacttagagaacatta 5130
human.FAM230J-LOC105372942-GGTLC5P.NCBI.rev.compl                                  gccactgctaagtgtccagcttattcctcctggcatgtagtaaacacttagagaacatta 5499
human.FAM230A.ncRNA.18487127.18500594-GGTLC3.18516335.18518165.rev.compl.REF       ------------------------------------------------------------ 0

chimp.LOC112206744-LOC107973052-GGT2.revcompl                                      ctgaagtaccagtcctctccaaggttttcctgtatttagtgatttttttagccctgtactg 8140
chimp54546-135457.revcompl.Pan.troglodytes.clone.rp43-41g5.GenBank:AC099533.36     CTGAAGTACCAGTCCTCTGTAAGGTTTTCCTGTATTTAGTGATTTTTTAGCCCTGTACTG 9699
human.FAM30B-LOC105372935-GGT2.NCBI.                                               ctgaagtaccagtcctctctaaggttttcctgtatttagtgatttttttagccctgcactg 6115
human.FAM230E-LOC105377182-GGT3P.NCBI.                                             ctgaagtaccagtcctctctaaggttttcctgtatttagtgatttttttagccctgtactg 5190
human.FAM230J-LOC105372942-GGTLC5P.NCBI.rev.compl                                  ctgaagtaccagtcctctctaaggttttcctgtatttagtgatttttttagccctgtactg 5559
human.FAM230A.ncRNA.18487127.18500594-GGTLC3.18516335.18518165.rev.compl.REF       ------------------------------------------------------------ 0

chimp.LOC112206744-LOC107973052-GGT2.revcompl                                      tgatactaagaagtagggcctaaatagggcctaaaaagtattgctaaaattacattatga 8200
chimp54546-135457.revcompl.Pan.troglodytes.clone.rp43-41g5.GenBank:AC099533.36     TGATACTAAGAAGTAGGGCCTAAATAGGGCCTAAAAAGTATTGCTAAAATTACATTATGA 9759
human.FAM30B-LOC105372935-GGT2.NCBI.                                               tgatactaagaagtagggcttaaatagggcctaaaaagtattgctaaaattacattatga 6175
human.FAM230E-LOC105377182-GGT3P.NCBI.                                             tgatactaagaagtagggcctaaatagggcctaaaaagtattgctaaaattacattatga 5250
human.FAM230J-LOC105372942-GGTLC5P.NCBI.rev.compl                                  tgatactaagaagtagggcttaaatagggcctaaaaagtattgctaaaattacattatga 5619
human.FAM230A.ncRNA.18487127.18500594-GGTLC3.18516335.18518165.rev.compl.REF       ------------------------------------------------------------ 0

chimp.LOC112206744-LOC107973052-GGT2.revcompl                                      cagtgcagagaactgagggcagagggaggacatgagcttgccaggtccacatggcttagt 8260
chimp54546-135457.revcompl.Pan.troglodytes.clone.rp43-41g5.GenBank:AC099533.36     CAGTGCAGAGAACTGAGGGCAGAGGGAGGACATGAGCTTGCCAGGTCTACATGGCTTAGT 9819
human.FAM30B-LOC105372935-GGT2.NCBI.                                               cagtgcagagaactgagggcagagggaggacatgagcttgccaggtccacatggcttagt 6235
human.FAM230E-LOC105377182-GGT3P.NCBI.                                             cagtgcagagaactgagggcagagggaagacatgagcttgccaggtccacatggcttagt 5310
human.FAM230J-LOC105372942-GGTLC5P.NCBI.rev.compl                                  cagtgcagagaactgagggcagagggaggacatgagcttgccaggtccacatggcttagt 5679
human.FAM230A.ncRNA.18487127.18500594-GGTLC3.18516335.18518165.rev.compl.REF       ------------------------------------------------------------ 0

chimp.LOC112206744-LOC107973052-GGT2.revcompl                                      ggaatttgaatccgggcccccactctgcaccagccctgcactcacagtcatcctgctgtg 8320
chimp54546-135457.revcompl.Pan.troglodytes.clone.rp43-41g5.GenBank:AC099533.36     GGAATTTGAATCCGGGCCCCCACTCTGCACCAGCCCCGCACTCACAGTCATCCTGCTGTA 9879
human.FAM30B-LOC105372935-GGT2.NCBI.                                               ggaatttgaatccgggcccccactctgcaccagccctgcactcacagtcatccggctgtg 6295
human.FAM230E-LOC105377182-GGT3P.NCBI.                                             ggaatttgaatccgggcccccactctgcaccagccctgcactcacagtcatccggctgtg 5370
human.FAM230J-LOC105372942-GGTLC5P.NCBI.rev.compl                                  ggaatttgaatccgggcccccactctgcaccagccctgcactcacagtcatccggctgtg 5739
human.FAM230A.ncRNA.18487127.18500594-GGTLC3.18516335.18518165.rev.compl.REF       ------------------------------------------------------------ 0

chimp.LOC112206744-LOC107973052-GGT2.revcompl                                      ttctcctctccaggaaggcactgcccacgcagtctgtctgatagaggtgttgagtgctca 8380
chimp54546-135457.revcompl.Pan.troglodytes.clone.rp43-41g5.GenBank:AC099533.36     TTCTCCTCTCCAGGAAGGCACTGCCCATGCAGTCTGTCTGATAGAGGTGTTGAGTGCTCA 9939
human.FAM30B-LOC105372935-GGT2.NCBI.                                               ttctcctctccaggaaggcactgcccacgcagtctgtctgatagaggtgttgagtgctca 6355
human.FAM230E-LOC105377182-GGT3P.NCBI.                                             ttcccctctccaggaaggcactgcccacgcagtctgtctgatagaggtgttgagtgctca 5430
human.FAM230J-LOC105372942-GGTLC5P.NCBI.rev.compl                                  ttcccctctccaggaaggcactgcccacgcagtctgtctgatagaggtgttgagtgctca 5799
human.FAM230A.ncRNA.18487127.18500594-GGTLC3.18516335.18518165.rev.compl.REF       ------------------------------------------------------------ 0

chimp.LOC112206744-LOC107973052-GGT2.revcompl                                      ctgaactccgtgatcttcctgaaacccaactttgattcagtgggttctgcttggaagcct 8440
chimp54546-135457.revcompl.Pan.troglodytes.clone.rp43-41g5.GenBank:AC099533.36     CTGAACTCCGTGATCTTCCTGAAACCCAACTTTGATTCAGTGGGCTCTGCTTGGAAGCCT 9999
human.FAM30B-LOC105372935-GGT2.NCBI.                                               ctgaactccgtgatcttcctgaaacccaactttgattcagtgggctctgcttggaagcct 6415
human.FAM230E-LOC105377182-GGT3P.NCBI.                                             ctgaactccgtgatcttcctgaaacccaactttgattcagtgggctctgcttggaagcct 5490
human.FAM230J-LOC105372942-GGTLC5P.NCBI.rev.compl                                  ctgaactccgtgatcttcctgaaacccaactttgattcagtgggctctgcttggaagcct 5859
human.FAM230A.ncRNA.18487127.18500594-GGTLC3.18516335.18518165.rev.compl.REF       ------------------------------------------------------------ 0

chimp.LOC112206744-LOC107973052-GGT2.revcompl                                      gtaaagaaaaggatcataagtttaaacttagaacagattatcactattttccctctggtc 8500
chimp54546-135457.revcompl.Pan.troglodytes.clone.rp43-41g5.GenBank:AC099533.36     GTAAAGAAAAGGATCATAAGTTTAAACTTAGAACAGATTATCACTATTTTCCCTCTGGTC 10059
human.FAM30B-LOC105372935-GGT2.NCBI.                                               gtaaagaaaaggatcataagtttaaacttagaacagattatcactattttccctctggtc 6475
human.FAM230E-LOC105377182-GGT3P.NCBI.                                             gtaaagaaaaggatcataagtttaaacttagaacagattatcactattttccctctggcc 5550
human.FAM230J-LOC105372942-GGTLC5P.NCBI.rev.compl                                  gtaaa-aaaaggatcataagtttaaacttagaacagattatcactattttccctctggtc 5918
human.FAM230A.ncRNA.18487127.18500594-GGTLC3.18516335.18518165.rev.compl.REF       ------------------------------------------------------------ 0

chimp.LOC112206744-LOC107973052-GGT2.revcompl                                      ttctgtcagcaagatgtcaacagccctatctattgtaaatgcattaaccagcatcttctc 8560
chimp54546-135457.revcompl.Pan.troglodytes.clone.rp43-41g5.GenBank:AC099533.36     TTCTGTCAGCAAGATGTCAACAGCCCTATCTATTGTAAATGCATTAACCAGCATCTTCTC 10119
human.FAM30B-LOC105372935-GGT2.NCBI.                                               ttctgtcagcaagatgtcaacagccctatctattgtcaatgcattaaccagcatcttctc 6535
human.FAM230E-LOC105377182-GGT3P.NCBI.                                             ttctgtcagcaagatgtcaacagccctatctattgtcaatgcattaaccagcgtcttctc 5610
human.FAM230J-LOC105372942-GGTLC5P.NCBI.rev.compl                                  ttctgtcagcaagatgtcaacagccctatctattgtcaatgcattaaccagcatcttctc 5978
human.FAM230A.ncRNA.18487127.18500594-GGTLC3.18516335.18518165.rev.compl.REF       ------------------------------------------------------------ 0

chimp.LOC112206744-LOC107973052-GGT2.revcompl                                      tgatagagaatataagaagatatgctgtgcacaccaaccagtgttggagacctcatggct 8620
chimp54546-135457.revcompl.Pan.troglodytes.clone.rp43-41g5.GenBank:AC099533.36     TGATAGAGAATACAAGAAGATATGCTGTGCACACCAACCAGTGTCGGAGACCTCATGGCT 10179
human.FAM30B-LOC105372935-GGT2.NCBI.                                               tgatagagaatacaagaagatatgctgtgcacaccaaccagtgtaggagacctcatggct 6595
human.FAM230E-LOC105377182-GGT3P.NCBI.                                             tgatagagaatacaagaagatatgctgtgcacaccaaccagtgtaggagacctcatggct 5670
human.FAM230J-LOC105372942-GGTLC5P.NCBI.rev.compl                                  tgatagagaatacaagaagatatgctgtgcacaccaaccagtgtaggagacctcatggct 6038
human.FAM230A.ncRNA.18487127.18500594-GGTLC3.18516335.18518165.rev.compl.REF       ------------------------------------------------------------ 0

chimp.LOC112206744-LOC107973052-GGT2.revcompl                                      cccgggtaaagaagaagaggtacccacaagaaggtactgtggaagttcattaattaagtt 8680
chimp54546-135457.revcompl.Pan.troglodytes.clone.rp43-41g5.GenBank:AC099533.36     CCCGGGTAAAGAAGAAGAGGTACCCACAAGAAGGTACTGTGGAAGTTCATTAATTAAGTT 10239
human.FAM30B-LOC105372935-GGT2.NCBI.                                               cccgggtaaagaagaagaggtacccacaagaaggtactgtggaagttcattaattaagtt 6655
human.FAM230E-LOC105377182-GGT3P.NCBI.                                             cccgggtaaagaagaagaggtacccacaagaaggtactgtggaagttcattaattaagtt 5730
human.FAM230J-LOC105372942-GGTLC5P.NCBI.rev.compl                                  cccgggtaaagaagaagaggtacccacaagaaggtactgtggaagttcattaattaagtt 6098
human.FAM230A.ncRNA.18487127.18500594-GGTLC3.18516335.18518165.rev.compl.REF       ------------------------------------------------------------ 0

chimp.LOC112206744-LOC107973052-GGT2.revcompl                                      gattcaagaattgcaattgcggggagtattcagtgtcccatatgtaagaggaaactatga 8740
chimp54546-135457.revcompl.Pan.troglodytes.clone.rp43-41g5.GenBank:AC099533.36     GATTCAAGAATTGCAATTGCGGGGAGTATTCAGTGTCCCATATGTAAGAGGAAACTATGA 10299
human.FAM30B-LOC105372935-GGT2.NCBI.                                               gattcaagaattgcagttgcggggagtattcagtgtcccatatgtaagaggaaactatga 6715
human.FAM230E-LOC105377182-GGT3P.NCBI.                                             gattcaagaattgcagttgcggggagtattcagtgtcccatatgtaagaggaaactatga 5790
human.FAM230J-LOC105372942-GGTLC5P.NCBI.rev.compl                                  gattcaagaattgcagttgcggggagtattcagtgtcccatatgtaagaggaaactatga 6158
human.FAM230A.ncRNA.18487127.18500594-GGTLC3.18516335.18518165.rev.compl.REF       ------------------------------------------------------------ 0

chimp.LOC112206744-LOC107973052-GGT2.revcompl                                      agagactaagccatatttttttaatgtgtcaggattctaatttgcctggtcagtaaatatt 8800
chimp54546-135457.revcompl.Pan.troglodytes.clone.rp43-41g5.GenBank:AC099533.36     AGAGACTAAGCCATATTTTTTAATGTGTCAGGATTCAAATTTGCCTGGTCAGTAAATATT 10359
human.FAM30B-LOC105372935-GGT2.NCBI.                                               agagactaagccatatttttttaatgtgtcaggattctaatttgcctggtcagtaaatatt 6775
```

```
human.FAM230E-LOC105377182-GGT3P.NCBI.                                              agagactaagccatatttttaatgtgtcaggattctaatttgcctggtcagtaaatatt 5850
human.FAM230J-LOC105372942-GGTLC5P.NCBI.rev.compl                                   agagactaagccatatttttaatgtgtcaggattctaatttgcctggtcagtaaatatt 6218
human.FAM230A.ncRNA.18487127.18500594-GGTLC3.18516335.18518165.rev.compl.REF        ------------------------------------------------------------ 0

chimp.LOC112206744-LOC107973052-GGT2.revcompl                                       gctaccaccacaaaagtaaatatctacttaaaagtcaattttggttcatgttgaatgata 8860
chimp54546-135457.revcompl.Pan.troglodytes.clone.rp43-41g5.GenBank:AC099533.36      GCTACCACCACAAAAGTAAATATCTACTTAAAAGACAATTTTGGTTCATGTTTAATGATA 10419
human.FAM30B-LOC105372935-GGT2.NCBI.                                                gctaccaccacaaaagtaaatatctacttaaaagtcaattttggttcatgtttaatgata 6835
human.FAM230E-LOC105377182-GGT3P.NCBI.                                              gctaccaccacaaaagtaaatatctacttaaaagtcaattttggttcatgtttaatgata 5910
human.FAM230J-LOC105372942-GGTLC5P.NCBI.rev.compl                                   gctaccaccacaaaagtaaatatctacttaaaagtcaattttggttcatgtttaatgata 6278
human.FAM230A.ncRNA.18487127.18500594-GGTLC3.18516335.18518165.rev.compl.REF        ------------------------------------------------------------ 0

chimp.LOC112206744-LOC107973052-GGT2.revcompl                                       gacaatgtttcaagctaatgtctagaacttacctggttgttaaacataagcatagatctc 8920
chimp54546-135457.revcompl.Pan.troglodytes.clone.rp43-41g5.GenBank:AC099533.36      GACAATGTTTCAAGCTAATGTCTAGAACTTACCTGGTTGTTAAACATAAGCATAGATCTC 10479
human.FAM30B-LOC105372935-GGT2.NCBI.                                                gacaatgtttcaagctaatgtctagaacttacctggttgttaaacataagcatagatctc 6895
human.FAM230E-LOC105377182-GGT3P.NCBI.                                              gacaatgtttcaagctaatgtctagaacttacctggttgttaaacaaaagcatagatctc 5970
human.FAM230J-LOC105372942-GGTLC5P.NCBI.rev.compl                                   gacaatgcttcaagctaatgtctagaacttacctggttgttaaacataagcatagatctc 6338
human.FAM230A.ncRNA.18487127.18500594-GGTLC3.18516335.18518165.rev.compl.REF        ------------------------------------------------------------ 0

chimp.LOC112206744-LOC107973052-GGT2.revcompl                                       cctgaaagagtggtgctatattattatttttcaattaatatatttctttagagagtttta 8980
chimp54546-135457.revcompl.Pan.troglodytes.clone.rp43-41g5.GenBank:AC099533.36      CCTGAAAGAGTGGTGCTATATTATTATTTTTCAATTAATATATTTCTTTAGAGAGTTTTA 10539
human.FAM30B-LOC105372935-GGT2.NCBI.                                                cctgaaagagtggtgctatattattatttttcaattaatatatttctttagagagtttta 6955
human.FAM230E-LOC105377182-GGT3P.NCBI.                                              cctgaaagagtggtgctatattattacttttcaattaatatatttctttagagaatttta 6030
human.FAM230J-LOC105372942-GGTLC5P.NCBI.rev.compl                                   cctgaaagagtggtgctatattattatttttcaattaatatatttctttagagagtttta 6398
human.FAM230A.ncRNA.18487127.18500594-GGTLC3.18516335.18518165.rev.compl.REF        ------------------------------------------------------------ 0

chimp.LOC112206744-LOC107973052-GGT2.revcompl                                       aattgacataaaaactgagcatatggccgggcgtggtggctcacacttataatcccagca 9040
chimp54546-135457.revcompl.Pan.troglodytes.clone.rp43-41g5.GenBank:AC099533.36      AATTGACATAAAAACTGAGCATATGGCCGGGCGTGGTGGCTCACACTTATAATCCCAGCA 10599
human.FAM30B-LOC105372935-GGT2.NCBI.                                                aattgacataaaaactgagcatatggccgggcgtggtggctcacacttataatcccagca 7015
human.FAM230E-LOC105377182-GGT3P.NCBI.                                              aattgacataaaaactgagcatatggccgggtgtggtggctcacacttataatcccagca 6090
human.FAM230J-LOC105372942-GGTLC5P.NCBI.rev.compl                                   aattgacataaaaactgagcatatggccgtgtgtggtggctcacacttataatcccagca 6458
human.FAM230A.ncRNA.18487127.18500594-GGTLC3.18516335.18518165.rev.compl.REF        ------------------------------------------------------------ 0

chimp.LOC112206744-LOC107973052-GGT2.revcompl                                       ctttaggaggccaaggcaggcggatcatctgaggtcaagagttggagaccagcctggcca 9100
chimp54546-135457.revcompl.Pan.troglodytes.clone.rp43-41g5.GenBank:AC099533.36      CTTTAGGAGGCCAAGGCAGGCGGATCATCTGAGGTCAGGAGTTGGAGACCAGCCTGGCCA 10659
human.FAM30B-LOC105372935-GGT2.NCBI.                                                ctttaggaggccaaggcaggcggatcatctgaggtcaggagttggagaccagcctggcca 7075
human.FAM230E-LOC105377182-GGT3P.NCBI.                                              ctttaggaggccaaggcaggcggatcatctgaggtcaggagttggagaccagcctggcca 6150
human.FAM230J-LOC105372942-GGTLC5P.NCBI.rev.compl                                   ctttaggaggccaaggcaggcggatcatctgaggtcaggagttggagaccagcctggcca 6518
human.FAM230A.ncRNA.18487127.18500594-GGTLC3.18516335.18518165.rev.compl.REF        ------------------------------------------------------------ 0

chimp.LOC112206744-LOC107973052-GGT2.revcompl                                       acatggtgaaaccccatctctactaaaaatacaaaaaaattagccgggtgtggtggcagg 9160
chimp54546-135457.revcompl.Pan.troglodytes.clone.rp43-41g5.GenBank:AC099533.36      ACATGGTGAAACCCCATCTCTACTAAAAATACAAAAAAATTAGCCGGGTGTGGTGGCAGG 10719
human.FAM30B-LOC105372935-GGT2.NCBI.                                                acatggtgaaaccccatctctactaaaaatccaaaaaaattagccgggtgtggtggcagg 7135
human.FAM230E-LOC105377182-GGT3P.NCBI.                                              acatggtgaaaccccatctctactaaaaatacaaaaaaattagccgggtgtggtggcagg 6210
human.FAM230J-LOC105372942-GGTLC5P.NCBI.rev.compl                                   acatggtgaaaccccatctctactaaaaatccaaaaaaattagccgggtgtggtggcagg 6578
human.FAM230A.ncRNA.18487127.18500594-GGTLC3.18516335.18518165.rev.compl.REF        ------------------------------------------------------------ 0

chimp.LOC112206744-LOC107973052-GGT2.revcompl                                       tgcctgtaatcccagctactcaggaggctgaggcaggagaatcgcttgaacccaggaggc 9220
chimp54546-135457.revcompl.Pan.troglodytes.clone.rp43-41g5.GenBank:AC099533.36      TGCCTGTAATCCCAGCTACTCAGGAGGCTGAGGCAGGAGAATCGCTTGAACCCAGGAGGC 10779
human.FAM30B-LOC105372935-GGT2.NCBI.                                                cgcctgtaatcccagctactcaggaggctgaggcaggagaatcgcttgaacccaggaggc 7195
human.FAM230E-LOC105377182-GGT3P.NCBI.                                              cgcctgtaatcccagctactcaggaggctgaggcaggagaatcgcttgaacccaggaggc 6270
human.FAM230J-LOC105372942-GGTLC5P.NCBI.rev.compl                                   cgcctgtaatcccagctactcagaaggctgaggcaggagaatcgcttgaacccgggaggc 6638
human.FAM230A.ncRNA.18487127.18500594-GGTLC3.18516335.18518165.rev.compl.REF        ------------------------------------------------------------ 0

chimp.LOC112206744-LOC107973052-GGT2.revcompl                                       agaggttgcagtgagccaagatcatgccattgcactccagcctgggtgacaagagtgaaa 9280
chimp54546-135457.revcompl.Pan.troglodytes.clone.rp43-41g5.GenBank:AC099533.36      AGAGGTTGCAGTGAGCCAAGATCATGCCATTGCACTCCAGCCTGGGTGACAAGAGTGAAA 10839
human.FAM30B-LOC105372935-GGT2.NCBI.                                                agaggttgcagtgagccaagatcatgccattgcactccagcctgggtgacaagagtgaaa 7255
human.FAM230E-LOC105377182-GGT3P.NCBI.                                              agaggttgcagtgagccaagatcatgccattgcactccagcctgggtgac--gagtgaaa 6328
human.FAM230J-LOC105372942-GGTLC5P.NCBI.rev.compl                                   agaggttgcagtgagccaagatcatgccattgcactccagcctgggtgacaagagtgaaa 6698
human.FAM230A.ncRNA.18487127.18500594-GGTLC3.18516335.18518165.rev.compl.REF        ------------------------------------------------------------ 0

chimp.LOC112206744-LOC107973052-GGT2.revcompl                                       ctccatctca----aaataaataaataaataaataaataaataaaaattgagtata 9336
chimp54546-135457.revcompl.Pan.troglodytes.clone.rp43-41g5.GenBank:AC099533.36      CTCCATCTCA--------------------AAATAAATAAATAAATAAAAATTGAGTATA 10879
human.FAM30B-LOC105372935-GGT2.NCBI.                                                ctccatctca------------aaataaataaataaataaataaaaattgagtata 7303
human.FAM230E-LOC105377182-GGT3P.NCBI.                                              ctccatctca------------aaataaataaataaataaataaaaattgactata 6376
human.FAM230J-LOC105372942-GGTLC5P.NCBI.rev.compl                                   ctccatctcaaaataaataaataaataaataaataaataaataaaaattgagtata 6758
human.FAM230A.ncRNA.18487127.18500594-GGTLC3.18516335.18518165.rev.compl.REF        ------------------------------------------------------------ 0

chimp.LOC112206744-LOC107973052-GGT2.revcompl                                       taatatacgaagttcccatattattctgtctcctcaccctcacttcctaatttcacctat 9396
chimp54546-135457.revcompl.Pan.troglodytes.clone.rp43-41g5.GenBank:AC099533.36      TAATACACGAAGTTCCCATATTATTCTGTCTCCTCACCCTCACTTCCTAATTTCACCTAT 10939
human.FAM30B-LOC105372935-GGT2.NCBI.                                                taatacacgaagttcccatattattctgtctcctcaccctcacttcctaatttcacctat 7363
human.FAM230E-LOC105377182-GGT3P.NCBI.                                              taatacacgaagttcccatattattctgactcctcaccctcacttcctaatttcacctat 6436
human.FAM230J-LOC105372942-GGTLC5P.NCBI.rev.compl                                   taatacacgaagttcccatattattctgtctcctcaccctcacttcctaatttcacctat 6818
human.FAM230A.ncRNA.18487127.18500594-GGTLC3.18516335.18518165.rev.compl.REF        ------------------------------------------------------------ 0

chimp.LOC112206744-LOC107973052-GGT2.revcompl                                       tagtaacatcttacattactgtggtacatttgctagaataatgagaaaatattgacacat 9456
chimp54546-135457.revcompl.Pan.troglodytes.clone.rp43-41g5.GenBank:AC099533.36      TAGTAACATCTTACATTACTGTGGTACATTTGCTAGAATAATGAGAAAATATTGACACAT 10999
human.FAM30B-LOC105372935-GGT2.NCBI.                                                tagtaacatcttacattactgtggtacatttgctagaataatgagaaaatattgacacat 7423
human.FAM230E-LOC105377182-GGT3P.NCBI.                                              tagtaacatcttacattactgtggtacatttgctagaataatgagaacatattgatacat 6496
human.FAM230J-LOC105372942-GGTLC5P.NCBI.rev.compl                                   tagtaacatcttacattactgtggtacatttgctagaataatgagaaaatattgacacat 6878
human.FAM230A.ncRNA.18487127.18500594-GGTLC3.18516335.18518165.rev.compl.REF        ------------------------------------------------------------ 0

chimp.LOC112206744-LOC107973052-GGT2.revcompl                                       tattatctgaagtctacatttgcataatgttcattctttctgttatacatatatatgaat 9516
chimp54546-135457.revcompl.Pan.troglodytes.clone.rp43-41g5.GenBank:AC099533.36      TATTATCTGAAGTCTACATTTGCATAATGTTCATTCTTTCTGTTATACATATATATGAAT 11059
human.FAM30B-LOC105372935-GGT2.NCBI.                                                tattatctgaagtctacatttgcataatgttcattctttctgttatacatatatatgaat 7483
human.FAM230E-LOC105377182-GGT3P.NCBI.                                              tattatctgaagtctacatttgcataatgttcattctttctgttatacatatatatgaat 6556
human.FAM230J-LOC105372942-GGTLC5P.NCBI.rev.compl                                   tattatctgaagtctgcatttgcataatgttcattctttctgttatacatatatatgaat 6938
human.FAM230A.ncRNA.18487127.18500594-GGTLC3.18516335.18518165.rev.compl.REF        ------------------------------------------------------------ 0

chimp.LOC112206744-LOC107973052-GGT2.revcompl                                       tttgaaatatttaaaacattatgttcacccttatggtctcataaagaaaatgttcacttc 9576
chimp54546-135457.revcompl.Pan.troglodytes.clone.rp43-41g5.GenBank:AC099533.36      TTTGAAATATTTAAAACATTATGTTCACCCTTATGGTCTCATAAAGAAAATGTTCACTTC 11119
human.FAM30B-LOC105372935-GGT2.NCBI.                                                tttgaaatatttaaaacattatgttcacccttatggtctcataaagaaaatgttcacttc 7543
human.FAM230E-LOC105377182-GGT3P.NCBI.                                              tttgaaatatttaaaacattatgttcacccttatggtctcataaagaaaatgttcacttc 6616
human.FAM230J-LOC105372942-GGTLC5P.NCBI.rev.compl                                   tttgaaatatttaaaacattatgttcacccttatggtctcataaagaaaatgttcacttc 6998
human.FAM230A.ncRNA.18487127.18500594-GGTLC3.18516335.18518165.rev.compl.REF        ------------------------------------------------------------ 0

chimp.LOC112206744-LOC107973052-GGT2.revcompl                                       cctaaaaatcctctcttctcattaatctctgtcctctttctccagaaaccttggcaacta 9636
chimp54546-135457.revcompl.Pan.troglodytes.clone.rp43-41g5.GenBank:AC099533.36      CCTAAAAATCCTCTCTTCTCATTAATCTCTGTCCTCTTTCTCCAGAAACCTTGGCAACTA 11179
human.FAM30B-LOC105372935-GGT2.NCBI.                                                cctaaaaatcctctcttctcattaatctctgtcctctttctccagaaaccttggcaacta 7603
human.FAM230E-LOC105377182-GGT3P.NCBI.                                              cctaaaaatcctctcttctcattaatctctgtcctctttctccagaaaccttggcaacta 6676
human.FAM230J-LOC105372942-GGTLC5P.NCBI.rev.compl                                   cctaaaaatcctctcttctcattaatctctgtcctctttctccagaaaccttggcaacta 7058
human.FAM230A.ncRNA.18487127.18500594-GGTLC3.18516335.18518165.rev.compl.REF        ------------------------------------------------------------ 0

chimp.LOC112206744-LOC107973052-GGT2.revcompl                                       ttaatatttttactatcacttcagctttgccttttccagaatgtcatatagttggaatca 9696
chimp54546-135457.revcompl.Pan.troglodytes.clone.rp43-41g5.GenBank:AC099533.36      TTAATATTTTTACTACCGCTTCAGCTTTGCCTTTTCCAGAATGTCATATAGTTGGAATCA 11239
human.FAM30B-LOC105372935-GGT2.NCBI.                                                ttaatatttttactatcgcttcagctttgccttttccagaatgtcatatagttggaatca 7663
human.FAM230E-LOC105377182-GGT3P.NCBI.                                              ttaatatttttactatcgcttcagctttgccttttccagaatgtcatatagttggaatca 6736
human.FAM230J-LOC105372942-GGTLC5P.NCBI.rev.compl                                   ttaatatttttactatcgcttcagctttgccttttccagaatgtcatatagttggaatca 7118
human.FAM230A.ncRNA.18487127.18500594-GGTLC3.18516335.18518165.rev.compl.REF        ------------------------------------------------------------ 0

chimp.LOC112206744-LOC107973052-GGT2.revcompl                                       tatattatgtagtttttcagatgaatttattgcactaaattgatgtacgctttagctgc 9756
chimp54546-135457.revcompl.Pan.troglodytes.clone.rp43-41g5.GenBank:AC099533.36      TATATTATGTAGTTTTTTCAGATGAATTTATTGCACTAAATTGATGTACGCTTTAGCTGC 11299
human.FAM30B-LOC105372935-GGT2.NCBI.                                                tatattatgtagtttttcagatgaatttattgcactaaattgatgtacgctttagctgc 7723
human.FAM230E-LOC105377182-GGT3P.NCBI.                                              tatattatgtagtttttcagatgaatttattgcactaaattgatgtatgctttagctgc 6796
human.FAM230J-LOC105372942-GGTLC5P.NCBI.rev.compl                                   tatattatgtagtttttcagatgaatttattgcactaaattgatgtacgctttagctgc 7178
human.FAM230A.ncRNA.18487127.18500594-GGTLC3.18516335.18518165.rev.compl.REF        ------------------------------------------------------------ 0

chimp.LOC112206744-LOC107973052-GGT2.revcompl                                       tttcatgtctttttttatgccttaatggcaaaaaatggcacattaaatcaccaaataatat 9816
chimp54546-135457.revcompl.Pan.troglodytes.clone.rp43-41g5.GenBank:AC099533.36      TTTCATGTCTTTTTTATGCCTTAATGGCAAAAAATGGCACATTAAATCACCAAATAATAT 11359
human.FAM30B-LOC105372935-GGT2.NCBI.                                                tttcatgtcttttttatgccttaatggcaaaaaatggcacattaaatcaccaaataatat 7783
human.FAM230E-LOC105377182-GGT3P.NCBI.                                              tttcatgtcttttttatgccttaatggcaaaaaatggcacattaaatcaccaaataatat 6856
human.FAM230J-LOC105372942-GGTLC5P.NCBI.rev.compl                                   tttcatgtcttttttatgccttaatggcaaaaaatggcacattaaatcaccaaataatat 7238
human.FAM230A.ncRNA.18487127.18500594-GGTLC3.18516335.18518165.rev.compl.REF        ------------------------------------------------------------ 0

chimp.LOC112206744-LOC107973052-GGT2.revcompl                                       tgcattaaatgaattttgtcttttattcacctgttgaagaattcggtagatttcatga 9876
chimp54546-135457.revcompl.Pan.troglodytes.clone.rp43-41g5.GenBank:AC099533.36      TGCATTAAAAGAATTTTTGTCTTTTTATTCACCTGTTGAAGAATTCGGTAGATTTCATGA 11419
human.FAM30B-LOC105372935-GGT2.NCBI.                                                tgcattcaatgaattttgtcttttattcacctgttgaagaattcggtagatttcatga 7843
human.FAM230E-LOC105377182-GGT3P.NCBI.                                              tgcattaaatgaattttgtcttttattcacctgttgaagaattcggtagatttcatga 6916
```

```
human.FAM230J-LOC105372942-GGTLC5P.NCBI.rev.compl                          tgcattaaatgaatttttgtctttttattcacctgttgaagaattcggtagatttcatga 7298
human.FAM230A.ncRNA.18487127.18500594-GGTLC3.18516335.18518165.rev.compl.REF ------------------------------------------------------------ 0

chimp.LOC112206744-LOC107973052-GGT2.revcompl                              gagaaaccatctgggcctggtgctttctttttcagaatgctcttaatgtgaattcaactt 9936
chimp54546-135457.revcompl.Pan.troglodytes.clone.rp43-41g5.GenBank:AC099533.36 GAGAAACCATCTGGGCCTGGTGCTTTCTTTTTCGGAATGCTCTTAATGTGAATTCAACTT 11479
human.FAM30B-LOC105372935-GGT2.NCBI.                                       gagaaaccatctgggcctggtgctttctttttcggaatgctcttaatgtgaattcaactt 7903
human.FAM230E-LOC105377182-GGT3P.NCBI.                                     gagaaaccatctgggcctggtgctttctttttcggaatgctcttaatgtgaattcaactt 6976
human.FAM230J-LOC105372942-GGTLC5P.NCBI.rev.compl                          gagaaaccatctgggcctggtgctttctttttcggaatgctcttaatgtgaattcaactt 7358
human.FAM230A.ncRNA.18487127.18500594-GGTLC3.18516335.18518165.rev.compl.REF ------------------------------------------------------------ 0

chimp.LOC112206744-LOC107973052-GGT2.revcompl                              atttaatagacataagtttattcaaattaggatcctagcatgaccttgggaagattgcct 9996
chimp54546-135457.revcompl.Pan.troglodytes.clone.rp43-41g5.GenBank:AC099533.36 ATTTAATAGACATAAGTTTATTCAAATTAGGATCCTAGCATGACCTTGGGAAGATTGCCT 11539
human.FAM30B-LOC105372935-GGT2.NCBI.                                       atttaatagacataagtttattcaaattaggatcctagcatgaccttgggaagattgcct 7963
human.FAM230E-LOC105377182-GGT3P.NCBI.                                     atttaatagacataagtttattcaaattaggattctagcgtgaccttgggaagattgcct 7036
human.FAM230J-LOC105372942-GGTLC5P.NCBI.rev.compl                          atttaatagacataagtttattcaaattaggatcctagcatgaccttgggaagattgcct 7418
human.FAM230A.ncRNA.18487127.18500594-GGTLC3.18516335.18518165.rev.compl.REF ------------------------------------------------------------ 0

chimp.LOC112206744-LOC107973052-GGT2.revcompl                              ttcaaggaattgatacatttcactgaggttatcaaactgcggtcatagaactgttcatga 10056
chimp54546-135457.revcompl.Pan.troglodytes.clone.rp43-41g5.GenBank:AC099533.36 TTCAAGGAATTGATACATTTCACTGAGGTTATCAAACTGCGGTCATAGAACTGTTCATGA 11599
human.FAM30B-LOC105372935-GGT2.NCBI.                                       ttcaaggaattgatacatttcactgaggttatcaaactgcggtcatagaactgttcatga 8023
human.FAM230E-LOC105377182-GGT3P.NCBI.                                     ttcaaggaattgatacatttcactgaggttatcagactgcggtcatagaactgttcataa 7096
human.FAM230J-LOC105372942-GGTLC5P.NCBI.rev.compl                          ttcaaggaattgatacatttcactgaggttatcaaactgcgctcatagaactgttcatga 7478
human.FAM230A.ncRNA.18487127.18500594-GGTLC3.18516335.18518165.rev.compl.REF ------------------------------------------------------------ 0

chimp.LOC112206744-LOC107973052-GGT2.revcompl                              tattccttttaatgcctaacagttcagtagagatggctcctcttttatttctgaaattgg 10116
chimp54546-135457.revcompl.Pan.troglodytes.clone.rp43-41g5.GenBank:AC099533.36 TATTCCTTTTAATGCCTAACAGTTCAGTAGAGATGGCTCCTCTTTTATTTCTGAAATTGG 11659
human.FAM30B-LOC105372935-GGT2.NCBI.                                       tattccttttaatgcctaacagttcagtagagatggctcctcttttatttctgaaattgg 8083
human.FAM230E-LOC105377182-GGT3P.NCBI.                                     tattccttttaatgcctaacagttcagtagagatggctcctcttttatttctgaaattgg 7156
human.FAM230J-LOC105372942-GGTLC5P.NCBI.rev.compl                          tattccttttaatgcctaacagttcagtagagatggctcctcttttatttctgaaattgg 7538
human.FAM230A.ncRNA.18487127.18500594-GGTLC3.18516335.18518165.rev.compl.REF ------------------------------------------------------------ 0

chimp.LOC112206744-LOC107973052-GGT2.revcompl                              tcatttgtgttatcttctttttcttggttagcctgcatatcaattcattcattgtaatga 10176
chimp54546-135457.revcompl.Pan.troglodytes.clone.rp43-41g5.GenBank:AC099533.36 TCATTTGTGTTATCTTCTTTTTCTTGGTTAGCCTGCATATCAATTCATTCATTGTAATGA 11719
human.FAM30B-LOC105372935-GGT2.NCBI.                                       tcatttgtgttatcttctttttcttggttagcctgcatatcaattcattcattgtaatga 8143
human.FAM230E-LOC105377182-GGT3P.NCBI.                                     tcatttgtgttatcttctttttcttggttagcctgcatatcaattcattcattgtaatga 7216
human.FAM230J-LOC105372942-GGTLC5P.NCBI.rev.compl                          tcatttgtgttatcttctttttcttggttagcctgcatatcaattcattcattgtaatga 7598
human.FAM230A.ncRNA.18487127.18500594-GGTLC3.18516335.18518165.rev.compl.REF ------------------------------------------------------------ 0

chimp.LOC112206744-LOC107973052-GGT2.revcompl                              gcatatcaaagaaccagcttttggtttattgatttctgatggtttcagtgttttaatt 10236
chimp54546-135457.revcompl.Pan.troglodytes.clone.rp43-41g5.GenBank:AC099533.36 GCATATCAAAGAACCAGCTTTTGGTTTATTGATTTCTGATGATTTCAGTGTTTTAATT 11779
human.FAM30B-LOC105372935-GGT2.NCBI.                                       gcatatcaaagaaccagcttttggtttattgatttctgatgatttcagtgttttaatt 8203
human.FAM230E-LOC105377182-GGT3P.NCBI.                                     gcatatcaaagaaccagcttttggtttattgatttctgatgatttcagtgttttaatt 7276
human.FAM230J-LOC105372942-GGTLC5P.NCBI.rev.compl                          gcatatcaaagaaccagcttttggtttattgatttctgatgatttcagtgttttaatt 7658
human.FAM230A.ncRNA.18487127.18500594-GGTLC3.18516335.18518165.rev.compl.REF ------------------------------------------------------------ 0

chimp.LOC112206744-LOC107973052-GGT2.revcompl                              ttattgatttctgtgatgttgtttattacttttacttgctttccattgcattcctctatt 10296
chimp54546-135457.revcompl.Pan.troglodytes.clone.rp43-41g5.GenBank:AC099533.36 TTATTGATTTCTGTGATGTTGTTTATTACTTTTACTTGCTTTCCATTGCATTCCTCTATT 11839
human.FAM30B-LOC105372935-GGT2.NCBI.                                       ttattgatttctgtgatgttgtttattacttttacttgctttccattgcattcctctatt 8263
human.FAM230E-LOC105377182-GGT3P.NCBI.                                     ttattgatttctgtgatgttgtttattacttttacttgctttccattgcattcctctatt 7336
human.FAM230J-LOC105372942-GGTLC5P.NCBI.rev.compl                          ttattgatttctgtgatgttgtttattacttttacttgctttccattgcattcctctatt 7718
human.FAM230A.ncRNA.18487127.18500594-GGTLC3.18516335.18518165.rev.compl.REF ------------------------------------------------------------ 0

chimp.LOC112206744-LOC107973052-GGT2.revcompl                              ttctatagttccctaattgaaacatgatattactgatttaggtcttgtgatttttagta 10356
chimp54546-135457.revcompl.Pan.troglodytes.clone.rp43-41g5.GenBank:AC099533.36 TTCTATAGTTCCCTAATTGAAACATGATATTACTGATTTAGGTCTTGTGATTTTTAGTA 11899
human.FAM30B-LOC105372935-GGT2.NCBI.                                       ttctatagttccctaattgaaacatgatattactgatttaggtcttgtgatttttagta 8323
human.FAM230E-LOC105377182-GGT3P.NCBI.                                     ttctatagttccctaattgaaacatgatattactgatttaggtcttgtgatttttagta 7396
human.FAM230J-LOC105372942-GGTLC5P.NCBI.rev.compl                          ttctatagttccctaattgaaacatgatattactgatttaggtcttgtgatttttagta 7778
human.FAM230A.ncRNA.18487127.18500594-GGTLC3.18516335.18518165.rev.compl.REF ------------------------------------------------------------ 0

chimp.LOC112206744-LOC107973052-GGT2.revcompl                              tattgcatccaatgctatagatttccctctaaggactgcttttgctacatccagaaatct 10416
chimp54546-135457.revcompl.Pan.troglodytes.clone.rp43-41g5.GenBank:AC099533.36 TATTGCATCCAATGCTATAGATTTCCCTCTAAGGACTGCTTTTGCTACATCCAGAAATCT 11959
human.FAM30B-LOC105372935-GGT2.NCBI.                                       tattgcatccaatgctatagatttccctctaaggactgcttttgctacatccagaaatct 8383
human.FAM230E-LOC105377182-GGT3P.NCBI.                                     tattgcatccaatgctgtagatttccctctaaggactgcttttgctacatccagaaatct 7456
human.FAM230J-LOC105372942-GGTLC5P.NCBI.rev.compl                          tattgcatccaatgctatagatttccctctaaggactgcttttgctacatccagaaatct 7838
human.FAM230A.ncRNA.18487127.18500594-GGTLC3.18516335.18518165.rev.compl.REF ------------------------------------------------------------ 0

chimp.LOC112206744-LOC107973052-GGT2.revcompl                              tgccaagtcacatttcttttaatgtagttaaaagtatttttaattttctattgagactt 10476
chimp54546-135457.revcompl.Pan.troglodytes.clone.rp43-41g5.GenBank:AC099533.36 TGCCAAGTCACATTTCTTTTAATGTAGTTAAAAGTATTTTTAATTTTCTATTGAGACTT 12019
human.FAM30B-LOC105372935-GGT2.NCBI.                                       tgccaagtcacatttcttttaatgtagttaaaagtatttttaattttctattgagactt 8443
human.FAM230E-LOC105377182-GGT3P.NCBI.                                     tgccaagtgacatttcttttaatgtagttaaaagtatttttaattttctattgagactt 7516
human.FAM230J-LOC105372942-GGTLC5P.NCBI.rev.compl                          tgccaagtcacatttcttttaatgtagttaaaagtactttttaattttctattgagactt 7898
human.FAM230A.ncRNA.18487127.18500594-GGTLC3.18516335.18518165.rev.compl.REF ------------------------------------------------------------ 0

chimp.LOC112206744-LOC107973052-GGT2.revcompl                              cttctttaacccatgagttatttaaaagtgcattgctaatttgcaaatatttggggattt 10536
chimp54546-135457.revcompl.Pan.troglodytes.clone.rp43-41g5.GenBank:AC099533.36 CTTCTTTAACCCATGAGTTATTTAAAAGTGCATTGCTAATTTGCAAATATTTGGGGATTT 12079
human.FAM30B-LOC105372935-GGT2.NCBI.                                       cttctttaacccatgagttatttaaaagtgcattgctaatttgcaaatatttggggattt 8503
human.FAM230E-LOC105377182-GGT3P.NCBI.                                     cttcttttacccatgagttatttaaaagtgcattgctaatttgcaaatatttggggattt 7576
human.FAM230J-LOC105372942-GGTLC5P.NCBI.rev.compl                          cttctttaacccaacagttatttaaaagtgcattgctaatttgcaaatatttggggattt 7958
human.FAM230A.ncRNA.18487127.18500594-GGTLC3.18516335.18518165.rev.compl.REF ------------------------------------------------------------ 0

chimp.LOC112206744-LOC107973052-GGT2.revcompl                              tgtggctcttttacagttgttgatttttttgttgtcaggtatgtgttgcaaaagcagtcgt 10596
chimp54546-135457.revcompl.Pan.troglodytes.clone.rp43-41g5.GenBank:AC099533.36 TCTGGCTCTTTTACAGTTGTTGATTTTTTGTTGTCAGGTATGTGTTGCAAAAGCAGTCGT 12139
human.FAM30B-LOC105372935-GGT2.NCBI.                                       tgtggctcttttacagttgttgaatttttgttgtcaggtatgtgttgcaaaagcagtcgt 8563
human.FAM230E-LOC105377182-GGT3P.NCBI.                                     tgtggctcttttacagttgttgatttttttgttgtcaggtgtgtgttgcaaaagcagtcgt 7636
human.FAM230J-LOC105372942-GGTLC5P.NCBI.rev.compl                          tgtggctcttttacagttgttgatttttttgttgtcaggtgtgtgttgcaaaagcagtcgt 8018
human.FAM230A.ncRNA.18487127.18500594-GGTLC3.18516335.18518165.rev.compl.REF ------------------------------------------------------------ 0

chimp.LOC112206744-LOC107973052-GGT2.revcompl                              ctacctcatcttgccaccagccaagatggcccaggatgtgggctctccctgagtgaatct 10656
chimp54546-135457.revcompl.Pan.troglodytes.clone.rp43-41g5.GenBank:AC099533.36 CTACCTCATCTTGCCACCAGCCAAGATGGCCCAGGATGTGGGCTCTCCCTGAGTGAATCT 12199
human.FAM30B-LOC105372935-GGT2.NCBI.                                       ctacctcatcttgccaccacccaagatggcccaggatgtgggctctccctgagtgaatct 8623
human.FAM230E-LOC105377182-GGT3P.NCBI.                                     ctacctcatcttgccaccacccaagatggcccaggatgtgggctctccctgagtgaatct 7696
human.FAM230J-LOC105372942-GGTLC5P.NCBI.rev.compl                          ctacctcatcttgccaccacccaagatggcccaggatgtgggctctccctgagtgaatct 8078
human.FAM230A.ncRNA.18487127.18500594-GGTLC3.18516335.18518165.rev.compl.REF ------------------------------------------------------------ 0

chimp.LOC112206744-LOC107973052-GGT2.revcompl                              ttggcaatctgccaacctgatgtgttcggcctccttctttagtctgagtttcccttctgc 10716
chimp54546-135457.revcompl.Pan.troglodytes.clone.rp43-41g5.GenBank:AC099533.36 TTGGCAATCTGCCAACCTGATGTGTTCGGCCTCCTTCTTTAGTCTGAGTTTGCCTTCTGC 12259
human.FAM30B-LOC105372935-GGT2.NCBI.                                       ttggcaatctgccaacctgatgtgttcggcctccttctttagtctgagcttgccttctgc 8683
human.FAM230E-LOC105377182-GGT3P.NCBI.                                     ttggcaatctgccaacctgatgtgttcggcctccttctttagtctgagcttgccttctgc 7756
human.FAM230J-LOC105372942-GGTLC5P.NCBI.rev.compl                          ttggcaatctgccaacctgatgtgttcggcctccttctttagtctgagcttgccttctgc 8138
human.FAM230A.ncRNA.18487127.18500594-GGTLC3.18516335.18518165.rev.compl.REF ------------------------------------------------------------ 0

chimp.LOC112206744-LOC107973052-GGT2.revcompl                              ttagaaagggccattctcagttctggcagggagttttcccaacattgagaaggtggcatt 10776
chimp54546-135457.revcompl.Pan.troglodytes.clone.rp43-41g5.GenBank:AC099533.36 TTAGAAAGGGCCATTCTCAGTTCTGGCAGGGAGTTTTCCCAACATTGAGAAGGTGGCATT 12319
human.FAM30B-LOC105372935-GGT2.NCBI.                                       ttagaaagggccattctcagttctggcagggagttttcccaacattgagaaggtggcatt 8743
human.FAM230E-LOC105377182-GGT3P.NCBI.                                     ttagaaagggccattctcagttctggcagggagttttcccaacattgagaaggtggcatt 7816
human.FAM230J-LOC105372942-GGTLC5P.NCBI.rev.compl                          ttagaaagggccattctcagttctggcagggagttttcccaacattgagaaggtggcatt 8198
human.FAM230A.ncRNA.18487127.18500594-GGTLC3.18516335.18518165.rev.compl.REF ------------------------------------------------------------ 0

chimp.LOC112206744-LOC107973052-GGT2.revcompl                              cttactccccactgcagcctgcacctctgaccggtggtcagcagacaggacagaggtcct 10836
chimp54546-135457.revcompl.Pan.troglodytes.clone.rp43-41g5.GenBank:AC099533.36 CTTACTCCCCACTACAGCCTGCACCTCTGACCGGTGGTCAGCAGACAGGACAGAGGTCCT 12379
human.FAM30B-LOC105372935-GGT2.NCBI.                                       cttactccccactgcagcctgcacctctgaccggtggtcagcagacaggacagaggtcct 8803
human.FAM230E-LOC105377182-GGT3P.NCBI.                                     cttactccccactgcagcctgcacctctgaccggtggtcagcagacaggacagaggtcct 7876
human.FAM230J-LOC105372942-GGTLC5P.NCBI.rev.compl                          cttactccccactgcagcctgcacctctgaccggtggtcagcagacaggacagaggtcct 8258
human.FAM230A.ncRNA.18487127.18500594-GGTLC3.18516335.18518165.rev.compl.REF ------------------------------------------------------------ 0

chimp.LOC112206744-LOC107973052-GGT2.revcompl                              cagtagacagagttcagcggggtctctgaccaaagtgcatcttcagagtctgcacctacc 10896
chimp54546-135457.revcompl.Pan.troglodytes.clone.rp43-41g5.GenBank:AC099533.36 CATTAGACAGAGTTCAGCGGGGTCTCTGACCAAAGTGCATCTTCAGAGTCTACACCTACC 12439
human.FAM30B-LOC105372935-GGT2.NCBI.                                       catcagacagagttcagcagggtctctgaccaaagtgcatcttcagagtctgcacctacc 8863
human.FAM230E-LOC105377182-GGT3P.NCBI.                                     cattagacagagttcagcagggtctctgaccaaagtgcatcttcagagtctgcacctacc 7936
human.FAM230J-LOC105372942-GGTLC5P.NCBI.rev.compl                          cattagacagagttcagcagggtctctgaccaaagtgcatcttcagagtctgcacctacc 8318
human.FAM230A.ncRNA.18487127.18500594-GGTLC3.18516335.18518165.rev.compl.REF ------------------------------------------------------------ 0

chimp.LOC112206744-LOC107973052-GGT2.revcompl                              cactgtgaccacgggcaggctctgagtcctaaagcaggaggaactgtgcgaccatcctga 10956
chimp54546-135457.revcompl.Pan.troglodytes.clone.rp43-41g5.GenBank:AC099533.36 CACTGTGACCACGGGCAGGCTCTGAGTCCTAAAGCAGGAGGAGCTGTGCGACCATCCTGA 12499
human.FAM30B-LOC105372935-GGT2.NCBI.                                       cactgtgaccacgggcaggctctgagtcctaaagcaggaggaactgtgcgaccatcctga 8923
human.FAM230E-LOC105377182-GGT3P.NCBI.                                     cactgtgaccacgggcaggctctgagtcctaaagcaggaggaactgtgcgaccatcctga 7996
human.FAM230J-LOC105372942-GGTLC5P.NCBI.rev.compl                          cactgtgaccacgggcaggctctgagtcctaaagcaggaggaactgtgcgaccatcctga 8378
```

```
human.FAM230A.ncRNA.18487127.18500594-GGTLC3.18516335.18518165.rev.compl.REF        ------------------------------------------------------------ 0

chimp.LOC112206744-LOC107973052-GGT2.revcompl                                       ttggaaatttgtgaggatcaccgtgttactcaagtaaggtctttggaaagtgtcgtatta 11016
chimp54546-135457.revcompl.Pan.troglodytes.clone.rp43-41g5.GenBank:AC099533.36      TTGGAAATTTGTGAGGATCACCGTGTTACTCAAGTAAGGTCTTTGGAAAGTGTCGTATTA 12559
human.FAM30B-LOC105372935-GGT2.NCBI.                                                ttggaaatttgtgaggatcaccgtgttactcaagtaaggtctttggaaagtgtcgtatta 8983
human.FAM230E-LOC105377182-GGT3P.NCBI.                                              ttggaaatttgtgaggatcaccgtgttactcaagtaaggtctttggaaagtgtcgtatta 8056
human.FAM230J-LOC105372942-GGTLC5P.NCBI.rev.compl                                   ttggaaatttgtgaggatcaccgtgttactcaagtaaggtctttggaaagtgtcgtatta 8438
human.FAM230A.ncRNA.18487127.18500594-GGTLC3.18516335.18518165.rev.compl.REF        ------------------------------------------------------------ 0

chimp.LOC112206744-LOC107973052-GGT2.revcompl                                       ctactgtttgtgaactgcttgttggtggcctggctgagccacacactttatgaaaaccag 11076
chimp54546-135457.revcompl.Pan.troglodytes.clone.rp43-41g5.GenBank:AC099533.36      CTACTGTTTGTGAACTGCTTGTTGGTGGCCTGGCTGAGCCACACACTTTATGAAAACCAG 12619
human.FAM30B-LOC105372935-GGT2.NCBI.                                                ctactgtttgtgaactgcttgttggtggcctggctgagccacacactttatgaaaaccag 9043
human.FAM230E-LOC105377182-GGT3P.NCBI.                                              ctactgtttgtgaactgcttgttggtggcctggctgagccacacactttatgaaaaccag 8116
human.FAM230J-LOC105372942-GGTLC5P.NCBI.rev.compl                                   ctactgtttgtgaactgcttgttggtggcctggctgagccacacactttatgaaaaccag 8498
human.FAM230A.ncRNA.18487127.18500594-GGTLC3.18516335.18518165.rev.compl.REF        ------------------------------------------------------------ 0

chimp.LOC112206744-LOC107973052-GGT2.revcompl                                       gacccctcagctggtgtgggtgtctatgcagactgagaccctcatgtgaacagccttgtg 11136
chimp54546-135457.revcompl.Pan.troglodytes.clone.rp43-41g5.GenBank:AC099533.36      GACCCCTCAGCTGGTGTGGGTGTCTATGCAGCCTGAGACCCTCATGTGAACAGCCTCGTG 12679
human.FAM30B-LOC105372935-GGT2.NCBI.                                                gacccctcagctggtgtgggtgtctatgcagcctgagaccctcatgtgaacagcctcgtg 9103
human.FAM230E-LOC105377182-GGT3P.NCBI.                                              gacccctcagctggtgtgggtgtctatgcagcctgagaccctcatgtgaacagcctcgtg 8176
human.FAM230J-LOC105372942-GGTLC5P.NCBI.rev.compl                                   gacccctcagctggtgtgggtgtctatgcagcctgagaccctcatgtgaacagcctcgtg 8558
human.FAM230A.ncRNA.18487127.18500594-GGTLC3.18516335.18518165.rev.compl.REF        ------------------------------------------------------------ 0

chimp.LOC112206744-LOC107973052-GGT2.revcompl                                       gcagctgtctttgccccttgccaccatcagtgcctccttgttcctgggcactgctttctc 11196
chimp54546-135457.revcompl.Pan.troglodytes.clone.rp43-41g5.GenBank:AC099533.36      GCAGCTGTCTTTGCCCCTTGCCACCATCAGTGCCTCCTTGTTCCTGGGCACTGCTTTCTC 12739
human.FAM30B-LOC105372935-GGT2.NCBI.                                                gcagctgtctttgccccttgccaccatcagtgcctccttgttcctgggcactgctttctc 9163
human.FAM230E-LOC105377182-GGT3P.NCBI.                                              gcagctgtctttgccccttgccaccatcagtgcctccttgttcctgggcactgctttctc 8236
human.FAM230J-LOC105372942-GGTLC5P.NCBI.rev.compl                                   gcagctgtctttgccccttgccaccatcagtgcctccttgttcctgggcactgctttctc 8618
human.FAM230A.ncRNA.18487127.18500594-GGTLC3.18516335.18518165.rev.compl.REF        ------------------------------------------------------------ 0

chimp.LOC112206744-LOC107973052-GGT2.revcompl                                       tgatggtgctccattgttttcctgcacctcagtgtctacagctggatgtctcttctgcaa 11256
chimp54546-135457.revcompl.Pan.troglodytes.clone.rp43-41g5.GenBank:AC099533.36      TGATGGTGCTCCATTGTTTTCCTGCACCTCAGTGTCTACAGCTGGATGTCTCTTCTGCAA 12799
human.FAM30B-LOC105372935-GGT2.NCBI.                                                tgatggtgctccattgttttcctgcacctcagtgtctacagctggatgtctcttccgcaa 9223
human.FAM230E-LOC105377182-GGT3P.NCBI.                                              tgatggtgctccattgttttcctgcacctcagtgtctacagctggatgtctcttccacag 8296
human.FAM230J-LOC105372942-GGTLC5P.NCBI.rev.compl                                   tgatggtgctccattgttttcctgcacctcagtgtctacagctggatgtctcttccgcaa 8678
human.FAM230A.ncRNA.18487127.18500594-GGTLC3.18516335.18518165.rev.compl.REF        ------------------------------------------------------------ 0

chimp.LOC112206744-LOC107973052-GGT2.revcompl                                       tctaggcgagggggcatcaatggcagttctgctgtggcactgccctccttcttagcttgt 11316
chimp54546-135457.revcompl.Pan.troglodytes.clone.rp43-41g5.GenBank:AC099533.36      TCTAGGCGAGGGGGCATCAATGGCAGTTCTGCTGTGGCACTGCCCTCCTTCTTAGCTTGT 12859
human.FAM30B-LOC105372935-GGT2.NCBI.                                                tctaggcgagggggcatcaacggcagttctgctgtggcactgccctccttcttagcttgt 9283
human.FAM230E-LOC105377182-GGT3P.NCBI.                                              tctaggcgagggggcatcaatggcagttctgctgtggcactgccctccttcttagcttgt 8356
human.FAM230J-LOC105372942-GGTLC5P.NCBI.rev.compl                                   tctaggcgagggggcatcaatggcagttctgctgtggcactgccctccttcttagcttgt 8738
human.FAM230A.ncRNA.18487127.18500594-GGTLC3.18516335.18518165.rev.compl.REF        ------------------------------------------------------------ 0

chimp.LOC112206744-LOC107973052-GGT2.revcompl                                       cttgctctgtcttaggctccctcaaagatcccacccttcaggttcttccacaagtttctt 11376
chimp54546-135457.revcompl.Pan.troglodytes.clone.rp43-41g5.GenBank:AC099533.36      CTTGCTCTGTCTTAGGCTCCCTGAAAGATCCCACCCTTCGGGTTCTTCCACAAGTTTCTT 12919
human.FAM30B-LOC105372935-GGT2.NCBI.                                                cttgctctgtcttaggctccctcaaagatcccacccttcaggttcttccacaagtttctt 9343
human.FAM230E-LOC105377182-GGT3P.NCBI.                                              cttgctctgtcttaggctccctcaaagatcccacccttcgggttcttccacaagtttctt 8416
human.FAM230J-LOC105372942-GGTLC5P.NCBI.rev.compl                                   cttgctctgtcttaggctccctcaaagatcccacccttcaggttcttccacaagtttctt 8798
human.FAM230A.ncRNA.18487127.18500594-GGTLC3.18516335.18518165.rev.compl.REF        ------------------------------------------------------------ 0

chimp.LOC112206744-LOC107973052-GGT2.revcompl                                       actgaaatccgagcagaaaactatgaccaatatgaccaatcctatacccactgaagacat 11436
chimp54546-135457.revcompl.Pan.troglodytes.clone.rp43-41g5.GenBank:AC099533.36      ATTGAAATCCGAGCAGAAAACTATGACCAATATGACCAATCCTATACCCACTGAAGACAT 12979
human.FAM30B-LOC105372935-GGT2.NCBI.                                                attgaaatccgagcagaaaactatgaccaatatgaccaatcctatacccactgaagacat 9403
human.FAM230E-LOC105377182-GGT3P.NCBI.                                              attgaaatccgagcagaaaactatgaccaatatgaccaatcctatacccactgaagacat 8476
human.FAM230J-LOC105372942-GGTLC5P.NCBI.rev.compl                                   attgaaatccgagcagaaaactatgaccaatatgaccaatcctatacccactgaagacat 8858
human.FAM230A.ncRNA.18487127.18500594-GGTLC3.18516335.18518165.rev.compl.REF        ------------------------------------------------------------ 0

chimp.LOC112206744-LOC107973052-GGT2.revcompl                                       gaatgaagaattaaaacaattctccatggactctaccatatagatccctagaagtaattt 11496
chimp54546-135457.revcompl.Pan.troglodytes.clone.rp43-41g5.GenBank:AC099533.36      GAATGAAGAATTAAAACAATTCTCCATGGACTCTACCATATAGATCCCTAGAAGTAATTT 13039
human.FAM30B-LOC105372935-GGT2.NCBI.                                                gaatgaagaattaaaacaattctccatggactctaccatatagatccctagaagtaattt 9463
human.FAM230E-LOC105377182-GGT3P.NCBI.                                              gaatgaagaattaaaacaattctccatggactctaccatatagatccctagaagtaattt 8536
human.FAM230J-LOC105372942-GGTLC5P.NCBI.rev.compl                                   gaatgaagaattaaaacaattctccatggactctaccatatagatccctagaagtaattt 8918
human.FAM230A.ncRNA.18487127.18500594-GGTLC3.18516335.18518165.rev.compl.REF        ------------------------------------------------------------ 0

chimp.LOC112206744-LOC107973052-GGT2.revcompl                                       ctaaaaaaaaaaaaaaaa----caaggaagatgtaatagttttccataaattagaatacc 11552
chimp54546-135457.revcompl.Pan.troglodytes.clone.rp43-41g5.GenBank:AC099533.36      CT------AAAAAAAAAAAAATCAAGGAAGATGTAATAGTTTTCCATAAATTAGAATACC 13093
human.FAM30B-LOC105372935-GGT2.NCBI.                                                ctaaaaaaaaaaaaaaaaaaatcaaggaagatgtaatagttttccataaattagaatacc 9523
human.FAM230E-LOC105377182-GGT3P.NCBI.                                              ct-----aaaaaaaaaaaaaatcaaggaagatgtaatagttttccataaattagaatacc 8591
human.FAM230J-LOC105372942-GGTLC5P.NCBI.rev.compl                                   ct------aaaaaaaaaaaaatcaaggaaaatgtaatagttttccataaattagaatacc 8972
human.FAM230A.ncRNA.18487127.18500594-GGTLC3.18516335.18518165.rev.compl.REF        ------------------------------------------------------------ 0

chimp.LOC112206744-LOC107973052-GGT2.revcompl                                       ctacatgtacaattaaatgaaatggctagtatagtcttgaaaccaaaaccagataaggta 11612
chimp54546-135457.revcompl.Pan.troglodytes.clone.rp43-41g5.GenBank:AC099533.36      CTACATGTACAATTAAATGAAATGGCTAGTATAGTCTTGAAACCAAAACCAGATAAGGTA 13153
human.FAM30B-LOC105372935-GGT2.NCBI.                                                ctacatgtacaattaaatgaaatggctagtatagtcttgaaaccaaaaccagataaggta 9583
human.FAM230E-LOC105377182-GGT3P.NCBI.                                              ctacatgtacaattaaatgaaatggctagtatagtcttgaaaccaaatccagataaggta 8651
human.FAM230J-LOC105372942-GGTLC5P.NCBI.rev.compl                                   ctacatgtacaattaaatgaaatggctactatagtcttgaaaccaaaaccagataaggta 9032
human.FAM230A.ncRNA.18487127.18500594-GGTLC3.18516335.18518165.rev.compl.REF        ------------------------------------------------------------ 0

chimp.LOC112206744-LOC107973052-GGT2.revcompl                                       aattaaattctgtgatattttaaaatactgtaaattctgactaatgtgagttaatctcaa 11672
chimp54546-135457.revcompl.Pan.troglodytes.clone.rp43-41g5.GenBank:AC099533.36      AATTAAATTCTGTGATATTTTAAAATACTGTAAATTCTGACTAATGTGAGTT------AA 13207
human.FAM30B-LOC105372935-GGT2.NCBI.                                                aattaaattctgtgatattttaaaatactgtaaattctgactaatgtgagttaatctcaa 9643
human.FAM230E-LOC105377182-GGT3P.NCBI.                                              aattaaattctgtgatattttaaaatactttaaattctgactaatgtgagttaatctcaa 8711
human.FAM230J-LOC105372942-GGTLC5P.NCBI.rev.compl                                   aattaaattctgtgatattttaaaatactttaaattctgactaatgtgagttaatctcaa 9092
human.FAM230A.ncRNA.18487127.18500594-GGTLC3.18516335.18518165.rev.compl.REF        ------------------------------------------------------------ 0

chimp.LOC112206744-LOC107973052-GGT2.revcompl                                       tatatgaaatagtagattaacattgaaaatgcaataaataaaattagctacctcaagagt 11732
chimp54546-135457.revcompl.Pan.troglodytes.clone.rp43-41g5.GenBank:AC099533.36      TATATGAAATAGTAGATTAACATTGAAAATGCAATAAATAAAATTAGCTACCTCAAGAGT 13267
human.FAM30B-LOC105372935-GGT2.NCBI.                                                tatatgaaatagtagattaacattgaaaatgcaataaataaaattagctacctcaagagt 9703
human.FAM230E-LOC105377182-GGT3P.NCBI.                                              catatgaaatagtagattaacattgaaaatgcaataaataaaattagctacctcaagagt 8771
human.FAM230J-LOC105372942-GGTLC5P.NCBI.rev.compl                                   catatgaaatagtagattaacattgaaaatgcaataaataaaattagctacctcaagagt 9152
human.FAM230A.ncRNA.18487127.18500594-GGTLC3.18516335.18518165.rev.compl.REF        ------------------------------------------------------------ 0

chimp.LOC112206744-LOC107973052-GGT2.revcompl                                       ttaatggaaaaaaatgtgattattgcaatagattcaggaaattcatgaataacattcacc 11792
chimp54546-135457.revcompl.Pan.troglodytes.clone.rp43-41g5.GenBank:AC099533.36      TTAATGGAAAAAAATGTGATTATTGCAATAGATTCAGGAAATTCATGAATAACATTCACC 13327
human.FAM30B-LOC105372935-GGT2.NCBI.                                                ttaatggaaaaaaatgtgattattgcaatagattcaggaaattcatgaataacattcacc 9763
human.FAM230E-LOC105377182-GGT3P.NCBI.                                              ttaatggaaaaaaatgtgattattgcaatagattcaggaaattcatgaataacattcacc 8831
human.FAM230J-LOC105372942-GGTLC5P.NCBI.rev.compl                                   ttaatggaaaaaaatgtgattattgcaatagattcaggaaattcatgaataacattcacc 9212
human.FAM230A.ncRNA.18487127.18500594-GGTLC3.18516335.18518165.rev.compl.REF        ------------------------------------------------------------ 0

chimp.LOC112206744-LOC107973052-GGT2.revcompl                                       ctatatttgtaggacaactatctgattaactttaagtgacctgtgacaaccatttgaatt 11852
chimp54546-135457.revcompl.Pan.troglodytes.clone.rp43-41g5.GenBank:AC099533.36      CTATATTTGTAGGACAACTATCTGATTAACTTTAAGTGACCTGTGACAACCATTTGAATT 13387
human.FAM30B-LOC105372935-GGT2.NCBI.                                                ctatatttgtaggacagctatc----taactttaagtgacctgtgacaaccatttgaatt 9819
human.FAM230E-LOC105377182-GGT3P.NCBI.                                              ctatatttgtaggacaactatctgattaactttaagtgacctgtgacaaccacttgaatt 8891
human.FAM230J-LOC105372942-GGTLC5P.NCBI.rev.compl                                   ctatatttgtaggacaactatctgattaactttaagtgacctgtgacaaccacttgaatt 9272
human.FAM230A.ncRNA.18487127.18500594-GGTLC3.18516335.18518165.rev.compl.REF        ------------------------------------------------------------ 0

chimp.LOC112206744-LOC107973052-GGT2.revcompl                                       aatgctgctttcacagcatatctcttggcttgttaaaaacccgacaagaatttccgtaac 11912
chimp54546-135457.revcompl.Pan.troglodytes.clone.rp43-41g5.GenBank:AC099533.36      AATGCTGCTTTCACAGCATATCTCTTGGCTTGTTAAAAATCCGACAAGAATTTCCGTAAC 13447
human.FAM30B-LOC105372935-GGT2.NCBI.                                                aatgctgctttcacagcatatctcttggcttgttaaaaacccgacaagaatttccgtaac 9879
human.FAM230E-LOC105377182-GGT3P.NCBI.                                              aatgctgctttcacagcatatctcttggcttgttaaaaacccgacaagaatttccgtaac 8951
human.FAM230J-LOC105372942-GGTLC5P.NCBI.rev.compl                                   aatgctgctttcacagcatatctcttggcttgttaaaaacccgacaagaatttccgtaac 9332
human.FAM230A.ncRNA.18487127.18500594-GGTLC3.18516335.18518165.rev.compl.REF        ------------------------------------------------------------ 0

chimp.LOC112206744-LOC107973052-GGT2.revcompl                                       attaatttattttaacacctatattgggtgtgaacccaccataaagtttgcccactgaa 11972
chimp54546-135457.revcompl.Pan.troglodytes.clone.rp43-41g5.GenBank:AC099533.36      ATTAATTTATTTTTAACACCTATATTGGGTGTGAACCCACCATAAAGTTTGCCCACTGAA 13507
human.FAM30B-LOC105372935-GGT2.NCBI.                                                attaatttatttttaacacctatattgggtgtgaacccaccataaagtttgcccactgaa 9939
human.FAM230E-LOC105377182-GGT3P.NCBI.                                              a-----ttatttttaacacctatattgggtgtgaacccaccataaagtttgcccactgaa 9006
human.FAM230J-LOC105372942-GGTLC5P.NCBI.rev.compl                                   a-----ttatttttaacacctatattgggtgtgaacccaccataaagtttgcccactgaa 9387
human.FAM230A.ncRNA.18487127.18500594-GGTLC3.18516335.18518165.rev.compl.REF        ------------------------------------------------------------ 0

chimp.LOC112206744-LOC107973052-GGT2.revcompl                                       aaggtctacaatttgatgctttattaaattgatactgtgtgcacccatcaccaccatcta 12032
chimp54546-135457.revcompl.Pan.troglodytes.clone.rp43-41g5.GenBank:AC099533.36      AAGGTCTACAATTTGATGCTTTATTAAATTGATACTGTGTGCACCCATCACCACGATCTA 13567
human.FAM30B-LOC105372935-GGT2.NCBI.                                                aaggtctacaatttgatgctttattaaattgatactgtgtgcacccatcaccacgatcta 9999
human.FAM230E-LOC105377182-GGT3P.NCBI.                                              aaggtctagaatttgatgctttattaaattgatactgtgtgcacccaacaccacgatcta 9066
human.FAM230J-LOC105372942-GGTLC5P.NCBI.rev.compl                                   aaggtctagaatttgatgctttattaaattgatactgtgtgcacccaacaccacgatcta 9447
human.FAM230A.ncRNA.18487127.18500594-GGTLC3.18516335.18518165.rev.compl.REF        ------------------------------------------------------------ 0
```

```
chimp.LOC112206744-LOC107973052-GGT2.revcompl                                 atttaaatatgtttccctcacccaaattctctcttgcgcactggcagttaatccccaccc 12092
chimp54546-135457.revcompl.Pan.troglodytes.clone.rp43-41g5.GenBank:AC099533.36 ATTTAAATATGTTTCCCTCACCCAAATTCTCTCTTGCACACTGGCAGTTAATCCCCACCC 13627
human.FAM30B-LOC105372935-GGT2.NCBI.                                          atttaaatatgtttccctcacccaaattctctcttgcgcactggcagttaatccccactc 10059
human.FAM230E-LOC105377182-GGT3P.NCBI.                                        atttaaatatgtttccctcacccaagttctctcttgcgcactggcagttaatccccactc 9126
human.FAM230J-LOC105372942-GGTLC5P.NCBI.rev.compl                             atttaaatatgtttccctcacccaagttctctcttgcgcactggcagttaatccccactc 9507
human.FAM230A.ncRNA.18487127.18500594-GGTLC3.18516335.18518165.rev.compl.REF  ------------------------------------------------------------ 0

chimp.LOC112206744-LOC107973052-GGT2.revcompl                                 ccatctccagccctaagcaatactgctgtgacattccatctccataaatttcccacttgc 12152
chimp54546-135457.revcompl.Pan.troglodytes.clone.rp43-41g5.GenBank:AC099533.36 CCATCTCCAGCCCTAAGCAATACTGCTGTGACATTCCATCTCCATAAATTTCCCACTTGC 13687
human.FAM30B-LOC105372935-GGT2.NCBI.                                          ccatctccagccctaagcaatactgctgtgacattccatctccataaatttcccatttgc 10119
human.FAM230E-LOC105377182-GGT3P.NCBI.                                        ccatctccagccctaagcaatactgctgtgacattccatctccataaatttcccacttgc 9186
human.FAM230J-LOC105372942-GGTLC5P.NCBI.rev.compl                             ccatctccagccctaagcaatactgctgtgacattccatctccataaatttcccacttgc 9567
human.FAM230A.ncRNA.18487127.18500594-GGTLC3.18516335.18518165.rev.compl.REF  ------------------------------------------------------------ 0

chimp.LOC112206744-LOC107973052-GGT2.revcompl                                 ttaatagaaatggacatatatatatatttggaatctgacttccttcatttagcatactat 12212
chimp54546-135457.revcompl.Pan.troglodytes.clone.rp43-41g5.GenBank:AC099533.36 TTAATAGAATTGGACATATATATATATTTGGAATCTGACTTCCTTCATTTAGCATACTAT 13747
human.FAM30B-LOC105372935-GGT2.NCBI.                                          ttaatagaaatggacatatatatatatttggaatctgacttccttcatttagcatactat 10179
human.FAM230E-LOC105377182-GGT3P.NCBI.                                        tttatagaaatggacatatatatatatttggaatctgacttccctcatttagcatactat 9246
human.FAM230J-LOC105372942-GGTLC5P.NCBI.rev.compl                             tttatagaaatggacatatatatatatttggaatctgacttccctcatttagcatactat 9627
human.FAM230A.ncRNA.18487127.18500594-GGTLC3.18516335.18518165.rev.compl.REF  ------------------------------------------------------------ 0

chimp.LOC112206744-LOC107973052-GGT2.revcompl                                 gtttgaagttaattgacgtgttagcacgtgctggtcatgtgttttccttcatagtctgct 12272
chimp54546-135457.revcompl.Pan.troglodytes.clone.rp43-41g5.GenBank:AC099533.36 GTTTGAAGTTAATTGACGTGTTAGCACGTGCTGGTCATGTGTTTTCCTTCATAGTCTGCT 13807
human.FAM30B-LOC105372935-GGT2.NCBI.                                          gtttgaagttaattgacgtgttagcacgtgctggtcatgtgttttccttcatagtctgct 10239
human.FAM230E-LOC105377182-GGT3P.NCBI.                                        gtttgaagttaattgacgtgttagcacgtgctggtcatgtgttttccttcatagtctgct 9306
human.FAM230J-LOC105372942-GGTLC5P.NCBI.rev.compl                             gtttgaagttaattgacgtgttagcacgtgctggtcatgtgttttccttcatagtctgct 9687
human.FAM230A.ncRNA.18487127.18500594-GGTLC3.18516335.18518165.rev.compl.REF  ------------------------------------------------------------ 0

chimp.LOC112206744-LOC107973052-GGT2.revcompl                                 gtgtttactcatacagatagtgtttattcgtttatcagttaatggacatttaattgtttt 12332
chimp54546-135457.revcompl.Pan.troglodytes.clone.rp43-41g5.GenBank:AC099533.36 GTGTTTACTCATACAGATAGTGTTTATTCATTTATCAGTTAATGGACATTTAATTGTTTT 13867
human.FAM30B-LOC105372935-GGT2.NCBI.                                          gtgtttactcatacagatagtgtttattcatttatcagttaatggacatttaattgtttt 10299
human.FAM230E-LOC105377182-GGT3P.NCBI.                                        gtgtttattcatacagatagtgtttattcatttatcagttaatggacatttaattgtttt 9366
human.FAM230J-LOC105372942-GGTLC5P.NCBI.rev.compl                             gtgtttattcatacagatagtgtttattcatttatcagttaatggacatttaattgtttt 9747
human.FAM230A.ncRNA.18487127.18500594-GGTLC3.18516335.18518165.rev.compl.REF  ------------------------------------------------------------ 0

chimp.LOC112206744-LOC107973052-GGT2.revcompl                                 gttattttctttgatgagtaacgtagcttcgagcattcatatacagtcaggtaatgcata 12392
chimp54546-135457.revcompl.Pan.troglodytes.clone.rp43-41g5.GenBank:AC099533.36 GTTATTTTCTTTGATGAGTAATATAGCTTTGAGCATTCATATACAGTCACGTAATGCATA 13927
human.FAM30B-LOC105372935-GGT2.NCBI.                                          gttattttctttgatgagtaatgtagctttgagcattcatatacagtcatgtaatgcata 10359
human.FAM230E-LOC105377182-GGT3P.NCBI.                                        gttattttctttgatgagtaatgtagctttgtgcattcatatacagtcatgtaatgcata 9426
human.FAM230J-LOC105372942-GGTLC5P.NCBI.rev.compl                             gttattttctttgatgagtaatgtagctttgagcattcatatacagtcatgtaatgcata 9807
human.FAM230A.ncRNA.18487127.18500594-GGTLC3.18516335.18518165.rev.compl.REF  ------------------------------------------------------------ 0

chimp.LOC112206744-LOC107973052-GGT2.revcompl                                 atgacattttggtcaaaaaaaa-ttttttttttctgagacccaggctggagtgcagtggc 12451
chimp54546-135457.revcompl.Pan.troglodytes.clone.rp43-41g5.GenBank:AC099533.36 ATGACGTTTTCGTCAAAAAAAA-TTTTTTTTTCTGAGACCCAGGCTGGAGTGCAGTGGC 13986
human.FAM30B-LOC105372935-GGT2.NCBI.                                          atgacattttggtc-aaaaaaattttttttttctgagacccaggctggagtgcagtggc 10418
human.FAM230E-LOC105377182-GGT3P.NCBI.                                        atgacattttggtcaaaaaaaattttttttttctgagacccaggctggagtgcagtggc 9486
human.FAM230J-LOC105372942-GGTLC5P.NCBI.rev.compl                             atgacattttggtcaaaaaaaattttttttttctgagacccaggctggagtgcagtggc 9867
human.FAM230A.ncRNA.18487127.18500594-GGTLC3.18516335.18518165.rev.compl.REF  ------------------------------------------------------------ 0

chimp.LOC112206744-LOC107973052-GGT2.revcompl                                 acaatctcggctcactggaacctccacctcccaggtttaagcaattcttgtgcctcaacc 12511
chimp54546-135457.revcompl.Pan.troglodytes.clone.rp43-41g5.GenBank:AC099533.36 ACAATCTCGGCTCACTGGAACCTCCACCTCCCAGGTTTAAGCAATTCTCCTGCCTCAACT 14046
human.FAM30B-LOC105372935-GGT2.NCBI.                                          acaatctcggctcactggaacctccacctcccaggtttaagcaattctcatgcctcaacc 10478
human.FAM230E-LOC105377182-GGT3P.NCBI.                                        acaatctcggctcactggaacctccatctcccaggtttaagcaattctcatgcctcaacc 9546
human.FAM230J-LOC105372942-GGTLC5P.NCBI.rev.compl                             acaatctcggctcactggaacctccatctcccaggtttaagcaattctcgtgcctcaacc 9927
human.FAM230A.ncRNA.18487127.18500594-GGTLC3.18516335.18518165.rev.compl.REF  ------------------------------------------------------------ 0

chimp.LOC112206744-LOC107973052-GGT2.revcompl                                 tcccgagtagctgggacaactggcacacgccaccatgcctggataattttttgtattttca 12571
chimp54546-135457.revcompl.Pan.troglodytes.clone.rp43-41g5.GenBank:AC099533.36 TCCCGAGTAGCTGGGACAACTGTCACACGCCACCATGCCTGGATAATTTTTGTATTTTCA 14106
human.FAM30B-LOC105372935-GGT2.NCBI.                                          tcccgagtagctgggacaactggcacacgccaccatgcctggataatttttgtattttca 10538
human.FAM230E-LOC105377182-GGT3P.NCBI.                                        tcccgagtagctgggacaactggcacacaccaccacgcctggataatttttgtattttca 9606
human.FAM230J-LOC105372942-GGTLC5P.NCBI.rev.compl                             tcccgagtagctgggacaactggcacacaccaccacacctggataatttttgtattttca 9987
human.FAM230A.ncRNA.18487127.18500594-GGTLC3.18516335.18518165.rev.compl.REF  ------------------------------------------------------------ 0

chimp.LOC112206744-LOC107973052-GGT2.revcompl                                 gtagagacaggattttgctgtgttggtcaggctagtctcaaactcctagcctcaggtgat 12631
chimp54546-135457.revcompl.Pan.troglodytes.clone.rp43-41g5.GenBank:AC099533.36 GTAGAGACAGGATTTTGCTGTGTTGGTCAGGCTAGTCTCAAACTCCTAGCCTCAGGTGAT 14166
human.FAM30B-LOC105372935-GGT2.NCBI.                                          gtagagacaggattttgctgtgttggtcaggctagtctcaaactcccagcctcaggtgat 10598
human.FAM230E-LOC105377182-GGT3P.NCBI.                                        gtagagacaggattttgctgtgttggtcaggctagtctcaaactcctagcctcaggtgat 9666
human.FAM230J-LOC105372942-GGTLC5P.NCBI.rev.compl                             gtagagacaggattttgctgtgttggtcaggctagtctcaaactcctagcctcaggtgat 10047
human.FAM230A.ncRNA.18487127.18500594-GGTLC3.18516335.18518165.rev.compl.REF  ------------------------------------------------------------ 0

chimp.LOC112206744-LOC107973052-GGT2.revcompl                                 ccacccatctctgcctcccaaagtgctgggattataggcatgagccaccacacccagcct 12691
chimp54546-135457.revcompl.Pan.troglodytes.clone.rp43-41g5.GenBank:AC099533.36 CCACCCATCTCTGCCTCCCAAAGTGCTGGGATTATAGGCATGAGCCACCACACCCAGCCT 14226
human.FAM30B-LOC105372935-GGT2.NCBI.                                          ccacctatctctgcctcccaaagtgctgggattataggcatgagccaccacacccagcct 10658
human.FAM230E-LOC105377182-GGT3P.NCBI.                                        ccacccacctctgcctcccaaagtgctgggattataggcatgagccaccacacccagcct 9726
human.FAM230J-LOC105372942-GGTLC5P.NCBI.rev.compl                             ccacccacctctgcctcccaaagtgctgggattataggcatgagccaccacacccagcct 10107
human.FAM230A.ncRNA.18487127.18500594-GGTLC3.18516335.18518165.rev.compl.REF  ------------------------------------------------------------ 0

chimp.LOC112206744-LOC107973052-GGT2.revcompl                                 aatttttttaagaaacaagggaactatttctaaattacttttgccaatttatatttcta 12751
chimp54546-135457.revcompl.Pan.troglodytes.clone.rp43-41g5.GenBank:AC099533.36 AATTTTTTAAGAAACAAGGGAACTATTTCTAAATTACTTTTGCCAATTGATATTTCTA 14286
human.FAM30B-LOC105372935-GGT2.NCBI.                                          aattttttaagaaacaagggaactatttctaaattacttttgccaatttatatttcta 10718
human.FAM230E-LOC105377182-GGT3P.NCBI.                                        aattttttaagaaagaagggaactatttctaaattacttttgccaatttatattccta 9786
human.FAM230J-LOC105372942-GGTLC5P.NCBI.rev.compl                             aattttttaagaaagaagggaactatttctaaattacttttgccaatttatattccta 10167
human.FAM230A.ncRNA.18487127.18500594-GGTLC3.18516335.18518165.rev.compl.REF  ------------------------------------------------------------ 0

chimp.LOC112206744-LOC107973052-GGT2.revcompl                                 ccatgatgcatagcactaatttcaccgtacaatgtatggtaggccccaatatgtaagaaa 12811
chimp54546-135457.revcompl.Pan.troglodytes.clone.rp43-41g5.GenBank:AC099533.36 CCATGATGCATAGCACTAATTTCACTGTACAATGTATGGTAGGCCCCAGTATGTAAGAAA 14346
human.FAM30B-LOC105372935-GGT2.NCBI.                                          ccatgatgcatagcactaatttcaccgtacaatgtatggtaggccccaatatgtaagaaa 10778
human.FAM230E-LOC105377182-GGT3P.NCBI.                                        ccatgatgcatagcactaatttcaccgtacaatgtatggtaggccccaatatgtaagaaa 9846
human.FAM230J-LOC105372942-GGTLC5P.NCBI.rev.compl                             ccatgatgcatagcactaatttcaccgtacaatgtatggtaggccccaatatgtaagaaa 10227
human.FAM230A.ncRNA.18487127.18500594-GGTLC3.18516335.18518165.rev.compl.REF  ------------------------------------------------------------ 0

chimp.LOC112206744-LOC107973052-GGT2.revcompl                                 tgatgaaagtaacacataaagattagtataaaacaaataaaattatcattgttgctatca 12871
chimp54546-135457.revcompl.Pan.troglodytes.clone.rp43-41g5.GenBank:AC099533.36 TGATGAAAGTAACACATAAAGATTAGTATAAAACAAATAAGATTATCATTGTTGCTATCA 14406
human.FAM30B-LOC105372935-GGT2.NCBI.                                          tgatgaaaataacacataaagattggtataaaacaaataagattatcattgttgctatca 10838
human.FAM230E-LOC105377182-GGT3P.NCBI.                                        tgatgaaagtaacacataaagattagtataagacaaatgagattatcgttgttgctatca 9906
human.FAM230J-LOC105372942-GGTLC5P.NCBI.rev.compl                             tgatgaaagtaacacataaagattagtataagacaaatgagattatcgttgttgctatca 10287
human.FAM230A.ncRNA.18487127.18500594-GGTLC3.18516335.18518165.rev.compl.REF  ------------------------------------------------------------ 0

chimp.LOC112206744-LOC107973052-GGT2.revcompl                                 tctttgtcaaattctgaaaacaatctgagtatatttttatataaatatgcttggcaacat 12931
chimp54546-135457.revcompl.Pan.troglodytes.clone.rp43-41g5.GenBank:AC099533.36 TCTTTGTCAAATTCTGAAAACAATCTGAGTATATTTTTATATAAATATGCTTGGCAACAT 14466
human.FAM30B-LOC105372935-GGT2.NCBI.                                          tctttgtcaaattctgaaaacaatctgagtatatttttatataaatatgcttggcaacat 10898
human.FAM230E-LOC105377182-GGT3P.NCBI.                                        tctttgtcaaattctgaaaacaatctgagtatatttttatataaatatgcttggcaacat 9966
human.FAM230J-LOC105372942-GGTLC5P.NCBI.rev.compl                             tctttgtcaaattctgaaaacaatctgagtatatttttatataaatatgcttggcaacat 10347
human.FAM230A.ncRNA.18487127.18500594-GGTLC3.18516335.18518165.rev.compl.REF  ------------------------------------------------------------ 0

chimp.LOC112206744-LOC107973052-GGT2.revcompl                                 agctgaaaaacgcattatcagttacatttatcagtaacaaagacataaatttgaaggggg 12991
chimp54546-135457.revcompl.Pan.troglodytes.clone.rp43-41g5.GenBank:AC099533.36 AGCTGAAAAACGCATTATCAGTTACATTTATCAGTAACAAAGACATAAATTTGAAGGGGG 14526
human.FAM30B-LOC105372935-GGT2.NCBI.                                          agctgaaaaacgcattatcagttacatttatcagtaacaaagacataaatttgaaggggg 10958
human.FAM230E-LOC105377182-GGT3P.NCBI.                                        agctgaaaaaagcattatcagttacatttatcagtaacaaagacataaatttgaaggggg 10026
human.FAM230J-LOC105372942-GGTLC5P.NCBI.rev.compl                             agctgaaaaaagcattatcagttacatttatcagtaacaaagacataaatttgaaggggg 10407
human.FAM230A.ncRNA.18487127.18500594-GGTLC3.18516335.18518165.rev.compl.REF  ------------------------------------------------------------ 0

chimp.LOC112206744-LOC107973052-GGT2.revcompl                                 aaaaacacttgtactaacaacgcaatgtcagaattcacataaaaattctgctggtcactt 13051
chimp54546-135457.revcompl.Pan.troglodytes.clone.rp43-41g5.GenBank:AC099533.36 AAAAACACTTGTACTAACAACGCAATGTCAGAATTAACATAAAAATTCTGCTGGTCACTT 14586
human.FAM30B-LOC105372935-GGT2.NCBI.                                          aacaacacttgtactaacaacgcaatgtcagaattaacataaaaattctgctggtcactt 11018
human.FAM230E-LOC105377182-GGT3P.NCBI.                                        aaaaacacttgtactaacaatgcaatgtcagaattaacataaaaattctgctggtcactt 10086
human.FAM230J-LOC105372942-GGTLC5P.NCBI.rev.compl                             aaaaacacttgtactaacaatgcaatgtcagaattaacataaaaattctgctggtcactt 10467
human.FAM230A.ncRNA.18487127.18500594-GGTLC3.18516335.18518165.rev.compl.REF  ------------------------------------------------------------ 0

chimp.LOC112206744-LOC107973052-GGT2.revcompl                                 tggaatatttaattgcctggggcagtgtttagtagacaaatgagcatctatggagcaccc 13111
chimp54546-135457.revcompl.Pan.troglodytes.clone.rp43-41g5.GenBank:AC099533.36 TGGAATATTTAATTGCCTGGGGCAGTGTTTAGTAGACAAATGAGCATCTATGGAGCACCC 14646
human.FAM30B-LOC105372935-GGT2.NCBI.                                          tggaatatttaattgcctggggcagtgtttagtagacaaatgagcatctatggagcactc 11078
human.FAM230E-LOC105377182-GGT3P.NCBI.                                        tggaatatttaattgcctggggcagtgtttagtagacacatgagcatctatggagcaccc 10146
human.FAM230J-LOC105372942-GGTLC5P.NCBI.rev.compl                             tggaatatttaattgcctggggcagtgtttagtagacaaatgagcatctatggagcaccc 10527
human.FAM230A.ncRNA.18487127.18500594-GGTLC3.18516335.18518165.rev.compl.REF  ------------------------------------------------------------ 0
```

```
chimp.LOC112206744-LOC107973052-GGT2.revcompl                                      aaagtaggggaatcaacagaacttcggtttgaaaagttatctgggtttagagcgtgaaac 13171
chimp54546-135457.revcompl.Pan.troglodytes.clone.rp43-41g5.GenBank:AC099533.36     AAAGTAGGGGAATCAACAGAACTTGGATTTGAAAAGTTATCTGGGTTTAGAGCGTGAAAC 14706
human.FAM30B-LOC105372935-GGT2.NCBI.                                               aaagtaggggaatcaacagaacttgggtttgaaaagttatctgggtttagagcgtgaaac 11138
human.FAM230E-LOC105377182-GGT3P.NCBI.                                             aaagtaggggaatcaacagaacttgggtttcaaaagttatctgggtttagagcgtgaaac 10206
human.FAM230J-LOC105372942-GGTLC5P.NCBI.rev.compl                                  aaagtaggggaatcaacagaacttgggtttcaaaagttatctgggtttagagcgtgaaac 10587
human.FAM230A.ncRNA.18487127.18500594-GGTLC3.18516335.18518165.rev.compl.REF       ------------------------------------------------------------ 0

chimp.LOC112206744-LOC107973052-GGT2.revcompl                                      tttgttagaggacacacgccttgcatgagcgaggtgccttggtgtgtgtggacgtaccat 13231
chimp54546-135457.revcompl.Pan.troglodytes.clone.rp43-41g5.GenBank:AC099533.36     TTTGTTAGAGGACACACACCTTGCATGAGCGAGGTGCCTTGGTGTGTGTGGATGTACCAT 14766
human.FAM30B-LOC105372935-GGT2.NCBI.                                               tttgttagaggacacgcaccttgcatgagcgaggtgccctggtgtgtgtggacgtaccat 11198
human.FAM230E-LOC105377182-GGT3P.NCBI.                                             tttgttagaggacacacaccttgcatgagtgaggtgccttggtgtgtgtggacgtatcat 10266
human.FAM230J-LOC105372942-GGTLC5P.NCBI.rev.compl                                  tttgttagaggacacacaccttgcatgagtgaggtgccttggtgtgtgtggacgtatcat 10647
human.FAM230A.ncRNA.18487127.18500594-GGTLC3.18516335.18518165.rev.compl.REF       ------------------------------------------------------------ 0

chimp.LOC112206744-LOC107973052-GGT2.revcompl                                      tatgcttggaggtacagcataatggtggcttcctccagaaagggacatttggggtggatt 13291
chimp54546-135457.revcompl.Pan.troglodytes.clone.rp43-41g5.GenBank:AC099533.36     TATGCTTGGAGGTACAGCATAATGGTGGCTTCCTCCAGAAAGGGACATTTGGGGTAGATT 14826
human.FAM30B-LOC105372935-GGT2.NCBI.                                               tatgcttggaggtacagcataatggtggcttcctccagaaagggacatttggggtggatt 11258
human.FAM230E-LOC105377182-GGT3P.NCBI.                                             tatgcttggaggtacagcataatggtggcttcctccagaaagggacatttggggtagatt 10326
human.FAM230J-LOC105372942-GGTLC5P.NCBI.rev.compl                                  tatgcttggaggtacagcataatggtggcttcctccagaaagggacatttggggtagatt 10707
human.FAM230A.ncRNA.18487127.18500594-GGTLC3.18516335.18518165.rev.compl.REF       ------------------------------------------------------------ 0

chimp.LOC112206744-LOC107973052-GGT2.revcompl                                      cattccatctagacaacacagcctgatgtggcatggacatgaatggaggtgaaatggtca 13351
chimp54546-135457.revcompl.Pan.troglodytes.clone.rp43-41g5.GenBank:AC099533.36     CATTCCATCTAGACAACACAGCCTGATGTGGCATGGACATGAATGGAGGTGAAATGGTCA 14886
human.FAM30B-LOC105372935-GGT2.NCBI.                                               cattccatctagacaacacagcctgatgtggcatggacatgaatggaggtgaaatggtca 11318
human.FAM230E-LOC105377182-GGT3P.NCBI.                                             catgccatctagacaacacagcctgatgtggcatggacatgaatggaggtgaaatggtca 10386
human.FAM230J-LOC105372942-GGTLC5P.NCBI.rev.compl                                  catgccatctagacaacacagcctgatgtggcatggacatgaatggaggtgaaatggtca 10767
human.FAM230A.ncRNA.18487127.18500594-GGTLC3.18516335.18518165.rev.compl.REF       ------------------------------------------------------------ 0

chimp.LOC112206744-LOC107973052-GGT2.revcompl                                      gtagttgagaggatcagtcctgacaagggccaaggtgaaaaacctgggaaccccttcagg 13411
chimp54546-135457.revcompl.Pan.troglodytes.clone.rp43-41g5.GenBank:AC099533.36     GTAGTTGAGAGGATCAGTCCTGACAAGGGCCGAGGTGAAAAACCTGGGAACCCCTTCAGG 14946
human.FAM30B-LOC105372935-GGT2.NCBI.                                               gtagttgagaggatcagtcctgacaagggccgaggtgaaaaacctgggaaccccttcagg 11378
human.FAM230E-LOC105377182-GGT3P.NCBI.                                             gtagttgagaggatcagtcctgacaagggccgaggtgaaaaacctgggaaccccttcagg 10446
human.FAM230J-LOC105372942-GGTLC5P.NCBI.rev.compl                                  gtagttgagaggatcagtcctgacaagggccgaggtgaaaaacctgggaaccccttcagg 10827
human.FAM230A.ncRNA.18487127.18500594-GGTLC3.18516335.18518165.rev.compl.REF       ------------------------------------------------------------ 0

chimp.LOC112206744-LOC107973052-GGT2.revcompl                                      tacaaagtcttcagttgaaaaaggaggtggtcacaggaaatactgagacaggacagcaat 13471
chimp54546-135457.revcompl.Pan.troglodytes.clone.rp43-41g5.GenBank:AC099533.36     TACAAAGTCTTCAGTTGAAAAAGGAGGTGGTCACAGGAAATACTGAGACAGGACAGCAAT 15006
human.FAM30B-LOC105372935-GGT2.NCBI.                                               tacaaagtcttcagttgaaaaaggaggtggtcacaggaaatactgagacaggacagcaaa 11438
human.FAM230E-LOC105377182-GGT3P.NCBI.                                             tgcaaagtcttcagttgaaaaaggaggtggtcacaggaaatactgagatgggtcagcaat 10506
human.FAM230J-LOC105372942-GGTLC5P.NCBI.rev.compl                                  tgcaaagtcttcagttgaaaaaggaggtggtcacaggaaatactgagatgggtcagcaat 10887
human.FAM230A.ncRNA.18487127.18500594-GGTLC3.18516335.18518165.rev.compl.REF       ------------------------------------------------------------ 0

chimp.LOC112206744-LOC107973052-GGT2.revcompl                                      gcatgggagacagagttcttggccctgcagggtgagtagtgtggattctcaagttttctc 13531
chimp54546-135457.revcompl.Pan.troglodytes.clone.rp43-41g5.GenBank:AC099533.36     GCATGGGAGACAGAGTTCTTGGCCCTGCAAGGTGAGTAGTGTGGATTCTCAAGTTTTCTC 15066
human.FAM30B-LOC105372935-GGT2.NCBI.                                               gcatgggagacagagttcttggccctgcagggtgagtagtgtggattctcaagttttctc 11498
human.FAM230E-LOC105377182-GGT3P.NCBI.                                             gcatgggagacagagttcttggccctgcagggtgagtagtgtggattctcaagttttctc 10566
human.FAM230J-LOC105372942-GGTLC5P.NCBI.rev.compl                                  gcatgggagacagagttcttggccctgcagggtgagtagtgtggattctcaagttttctc 10947
human.FAM230A.ncRNA.18487127.18500594-GGTLC3.18516335.18518165.rev.compl.REF       ------------------------------------------------------------ 0

chimp.LOC112206744-LOC107973052-GGT2.revcompl                                      ctctctccattaatttctttcccaatgcagatgacttccatcatacagtcttcagcaacc 13591
chimp54546-135457.revcompl.Pan.troglodytes.clone.rp43-41g5.GenBank:AC099533.36     CTCTCTCCATTAATTTCTTTCCCAATGCAGATGACTTCCATCATACAGTCTTCAGCAACC 15126
human.FAM30B-LOC105372935-GGT2.NCBI.                                               ctctctccattaatttctttcccaatgcagatgacttccatcatacagtcttcagcaacc 11558
human.FAM230E-LOC105377182-GGT3P.NCBI.                                             ctctctccattaatttctttcccaatgcagatgacttccatcatacagtcttcagcaacc 10626
human.FAM230J-LOC105372942-GGTLC5P.NCBI.rev.compl                                  ctctctccattaatttctttcccaatgcagatgacttccatcatacagtcttcagcaacc 11007
human.FAM230A.ncRNA.18487127.18500594-GGTLC3.18516335.18518165.rev.compl.REF       ------------------ttcccaatgcagatgacttccatcatacagtcttcagcaacc 42
                                                                                                     ******************************************

chimp.LOC112206744-LOC107973052-GGT2.revcompl                                      ttgaaagattggacaagcttcagcccactcttgaaggtaaaggaaggcagctaacaagac 13651
chimp54546-135457.revcompl.Pan.troglodytes.clone.rp43-41g5.GenBank:AC099533.36     TTGAAAGATTGGACAAGCTTCAGCCCACTCTTGAAGGTAAAGGAAGGCAGCTAACAAGAC 15186
human.FAM30B-LOC105372935-GGT2.NCBI.                                               ttgaaagattggacaagcttcagcccactcttgaaggtaaaggaaggcagctaacaagac 11618
human.FAM230E-LOC105377182-GGT3P.NCBI.                                             ttgaaagattggacaagcttcagcccactcttgaaggtaaaggaaggcagctaacaagac 10686
human.FAM230J-LOC105372942-GGTLC5P.NCBI.rev.compl                                  ttgaaagattggacaagcttcagcccactcttgaaggtaaaggaaggcagctaacaagac 11067
human.FAM230A.ncRNA.18487127.18500594-GGTLC3.18516335.18518165.rev.compl.REF       ttgaaagattggacaagcttcagcccactcttgaaggtaaaggaaggcagctaacaagac 102
                                                                                   ************************************************************

chimp.LOC112206744-LOC107973052-GGT2.revcompl                                      tggcatctgggcttggctgtgcgtgttttctattgtggggaaatatatataacacaatat 13711
chimp54546-135457.revcompl.Pan.troglodytes.clone.rp43-41g5.GenBank:AC099533.36     TGGCATCTGGGCTTGGCTGTGCGTGTTTTCTATCGTGGGGAAATATATATAACACATTAT 15246
human.FAM30B-LOC105372935-GGT2.NCBI.                                               tggcatctgggcttggctgtgcgtgttttctatcgtggggaaatatatataacacaatat 11678
human.FAM230E-LOC105377182-GGT3P.NCBI.                                             tggcatctgggcttggctgtgcatgttttctatcgtggggaaatatatataacacattat 10746
human.FAM230J-LOC105372942-GGTLC5P.NCBI.rev.compl                                  tggcatctgggcttggctgtgcatgttttctatcgtggggaaatatatataacacattat 11127
human.FAM230A.ncRNA.18487127.18500594-GGTLC3.18516335.18518165.rev.compl.REF       tggcatctgggcttggctgtgcatgttttctatcgtggggaaatatatgtaacacattat 162
                                                                                   ********************** ********** ************** ******* ***

chimp.LOC112206744-LOC107973052-GGT2.revcompl                                      ttatcatttgaaccttttaaccaaagtgtgcactccatggcattcaatatattcacaggg 13771
chimp54546-135457.revcompl.Pan.troglodytes.clone.rp43-41g5.GenBank:AC099533.36     TTATCATTTGAACCTTTTAACCAAAGTGTGCACTCCATGGCATTCAATATATTCACAGGG 15306
human.FAM30B-LOC105372935-GGT2.NCBI.                                               ttatcatttgaaccttttaaccaaagtgtgcactccatggcattcaatatattcacaggg 11738
human.FAM230E-LOC105377182-GGT3P.NCBI.                                             ttatcatttgaaccttttaaccaaagtgtgcactccgtggcattcaatatattcacaggg 10806
human.FAM230J-LOC105372942-GGTLC5P.NCBI.rev.compl                                  ttatcatttgaaccttttaaccaaagtgtgcactccgtggcattcaatatattcacaggg 11187
human.FAM230A.ncRNA.18487127.18500594-GGTLC3.18516335.18518165.rev.compl.REF       ttatcatttgaaccttttaaccaaagtgtgcactccgtggcattcaatatattcacaggg 222
                                                                                   ************************************ ***********************

chimp.LOC112206744-LOC107973052-GGT2.revcompl                                      ttgcataaccaacaccactatctacacccacaattttgatgatttcttacaaaaccttgt 13831
chimp54546-135457.revcompl.Pan.troglodytes.clone.rp43-41g5.GenBank:AC099533.36     TTGCATAACCAACACCACTATCTACACCCACAATTTTGATGATTTCTTACAAAACCTTGT 15366
human.FAM30B-LOC105372935-GGT2.NCBI.                                               ttgcataaccaacaccactatctacacccacaattttgatgatttcttacgaaaccttgt 11798
human.FAM230E-LOC105377182-GGT3P.NCBI.                                             ttgcataaccaacaccactatctacacccacaattttgatgatttcttacaaaaccttgt 10866
human.FAM230J-LOC105372942-GGTLC5P.NCBI.rev.compl                                  ttgcataaccaacaccactatctacacccacaattttgatgatttcttacaaaaccttgt 11247
human.FAM230A.ncRNA.18487127.18500594-GGTLC3.18516335.18518165.rev.compl.REF       ttgcataaccaacaccactatctacacccacaattttgatgatttcttacaaaaccttgt 282
                                                                                   ************************************************** *********

chimp.LOC112206744-LOC107973052-GGT2.revcompl                                      ccactataagcaatatagcaccttccccctatttccagctcatggtgattcctatcccac 13891
chimp54546-135457.revcompl.Pan.troglodytes.clone.rp43-41g5.GenBank:AC099533.36     CCACAATAAGCAATATAGCACCTTCCCCCTATTTCCAGCCCATGGTGATTCCTATCCCAC 15426
human.FAM30B-LOC105372935-GGT2.NCBI.                                               ccacaataagcaatatagcaccttccccctatttccagcccatggtgattcctatcccac 11858
human.FAM230E-LOC105377182-GGT3P.NCBI.                                             ccacaataagcaatatagcgccttccccctatttccagcccatggtgattcctatcccac 10926
human.FAM230J-LOC105372942-GGTLC5P.NCBI.rev.compl                                  ccacaataagcaatatagcgccttccccctatttccagcccatggtgattcctatccgac 11307
human.FAM230A.ncRNA.18487127.18500594-GGTLC3.18516335.18518165.rev.compl.REF       ccacaataagcaatatagcaccttccccctatttccagcccatggtgattcctatccgac 342
                                                                                   **** ************** ******************* ***************** **

chimp.LOC112206744-LOC107973052-GGT2.revcompl                                      tttctcttgtatgaatttgactattctaggcacttcatgtaattacaattatacaatata 13951
chimp54546-135457.revcompl.Pan.troglodytes.clone.rp43-41g5.GenBank:AC099533.36     TTTCTCTTGTATGAATTTGACTATTCTAGGCACTTCATGTCATTACAATTATACAATATA 15486
human.FAM30B-LOC105372935-GGT2.NCBI.                                               tttctcttgtatgaatttgactattctaggcacttcatgtaattacaattatacaatata 11918
human.FAM230E-LOC105377182-GGT3P.NCBI.                                             --tctctcgtatgaatttgactattctaggcacttcatgtaattacaattatacaatata 10984
human.FAM230J-LOC105372942-GGTLC5P.NCBI.rev.compl                                  tttctcttgtatgaatttgactattctaggcacttcatgtaattacaattatacaatata 11367
human.FAM230A.ncRNA.18487127.18500594-GGTLC3.18516335.18518165.rev.compl.REF       tttctcttgtatgaatttgactattctaggcacttcatgtaattacaattatacaatata 402
                                                                                     ***** ******************************** *******************

chimp.LOC112206744-LOC107973052-GGT2.revcompl                                      ttccttttgtgtctggcttatttcactaagcataatgttctcaatgtccacccatgttgt 14011
chimp54546-135457.revcompl.Pan.troglodytes.clone.rp43-41g5.GenBank:AC099533.36     TTCCTTTTGTGTCTGGCTTATTTCACTAAGCATAATGTTCTCAATGTCCACCCATGTTGT 15546
human.FAM30B-LOC105372935-GGT2.NCBI.                                               ttccttttgtgtctggcttatttcactaagcataatgttctcaatgtccacccatgttgt 11978
human.FAM230E-LOC105377182-GGT3P.NCBI.                                             ttccttttgtgtctggcttatttcactaagcataatgttctcaatgtccacccatgttgt 11044
human.FAM230J-LOC105372942-GGTLC5P.NCBI.rev.compl                                  ttccttttgtgtctggcttatttcactaagcataatgttctcaatgtccacccatgttgt 11427
human.FAM230A.ncRNA.18487127.18500594-GGTLC3.18516335.18518165.rev.compl.REF       ttccttttgtgtctggcttatttcactaagcataatgttctcaatgtccacccatgttgt 462
                                                                                   ************************************************************

chimp.LOC112206744-LOC107973052-GGT2.revcompl                                      atcatctatcaaaattatgttcgtttttacagatggatgatgtagcattgcatgcagac 14071
chimp54546-135457.revcompl.Pan.troglodytes.clone.rp43-41g5.GenBank:AC099533.36     ATCATCTATCAAAATTATGTTCGTTTTTACAGATGGATGATGTAGCATTGCATGCAGAC 15606
human.FAM30B-LOC105372935-GGT2.NCBI.                                               atcatctttcaaaatgatgttcgtttttacagatggatgatgtagcattgcatgcagac 12038
human.FAM230E-LOC105377182-GGT3P.NCBI.                                             atcatctatcaaaatgatgttcatttttacagatggatgatgtagcattgcatgcagac 11104
human.FAM230J-LOC105372942-GGTLC5P.NCBI.rev.compl                                  atcatctatcaaaatgatgttcgtttttacagatggatgatgtagcattgcatgcagac 11487
human.FAM230A.ncRNA.18487127.18500594-GGTLC3.18516335.18518165.rev.compl.REF       atcatctatcaaaatgatgttcgtttttacagatggatgatgtagcattgcatgcagac 522
                                                                                   ******* ******* ****** *************************************

chimp.LOC112206744-LOC107973052-GGT2.revcompl                                      caccttgcttttattacattcatttgttcactgatggttggattatttccaccttttggc 14131
chimp54546-135457.revcompl.Pan.troglodytes.clone.rp43-41g5.GenBank:AC099533.36     CACCTTGCTTTTATTACATTCATTTGTTCACTGATGGTTGGATTATTTCCACCTTTTGGC 15666
human.FAM30B-LOC105372935-GGT2.NCBI.                                               caccttgcttttattacattcatttgttcactgatggttggattatttccaccttttggc 12098
human.FAM230E-LOC105377182-GGT3P.NCBI.                                             caccttgcttttattacattcatttgttcactgatggttggattatttccaccttttggc 11164
human.FAM230J-LOC105372942-GGTLC5P.NCBI.rev.compl                                  caccttgcttttattacattcatttgttcactgatggttggattatttccaccttttggc 11547
human.FAM230A.ncRNA.18487127.18500594-GGTLC3.18516335.18518165.rev.compl.REF       caccttgcttttattacattcatttgttcactgatggttggattatttccaccttttggc 582
                                                                                   ************************************************************

chimp.LOC112206744-LOC107973052-GGT2.revcompl                                      tcctgtgaaaagtgatgctacaaacattagtatacaaacatctgtttgatttctgttctc 14191
chimp54546-135457.revcompl.Pan.troglodytes.clone.rp43-41g5.GenBank:AC099533.36     TCCTGTGAAAAGTGATGCTACAAACATTAGTATACAAACATCTGTTTGATTTCTGTTCTC 15726
human.FAM30B-LOC105372935-GGT2.NCBI.                                               tcctgtgaaaagtgatgctacaaacattagtatacaaacatctgtttgatttccgttctc 12158
human.FAM230E-LOC105377182-GGT3P.NCBI.                                             tcctgtgaaaagtgatgctacaaacattagtatacaaacatctgtttgatttctgttctc 11224
human.FAM230J-LOC105372942-GGTLC5P.NCBI.rev.compl                                  tcctgtgaaaagtgatgctacaaacattagtatacaaacatctgtttgatttctgttctc 11607
human.FAM230A.ncRNA.18487127.18500594-GGTLC3.18516335.18518165.rev.compl.REF       tcctgtgaaaagtgatgctacaaacattagtatacaaacatctgtttgatttctgttctc 642
                                                                                   ***************************************************** ******
```

```
chimp.LOC112206744-LOC107973052-GGT2.revcompl                                      tattctttggggtgcctaagagtagagttcctgggtccaacaggggttctatatttaaac 14251
chimp54546-135457.revcompl.Pan.troglodytes.clone.rp43-41g5.GenBank:AC099533.36     TATTCTTTGGGGTGCCTAAGAGTAGAGTTCCTGGGTCCAACAGGGGTTCTATATTTAACC 15786
human.FAM30B-LOC105372935-GGT2.NCBI.                                               tattctttggggtgcctaagagtagagttcctgggtccaacaggggttctatatttaacc 12218
human.FAM230E-LOC105377182-GGT3P.NCBI.                                             tattctttggggtgcctaggagtagagttcctgggtccaacaggggttctatatttaacc 11284
human.FAM230J-LOC105372942-GGTLC5P.NCBI.rev.compl                                  tattctttggggtgcctaagagtagagttcctgggtccaacaggggttctatatttaacc 11667
human.FAM230A.ncRNA.18487127.18500594-GGTLC3.18516335.18518165.rev.compl.REF       tattctttggggtgcctaagagtagagttcctgggtccaacaggggttctatatttaacc 702
                                                                                   ****************** ************************************** *

chimp.LOC112206744-LOC107973052-GGT2.revcompl                                      ttctgagccactgcagactgtttttcagagtggctgcaactttatccatttctaccatca 14311
chimp54546-135457.revcompl.Pan.troglodytes.clone.rp43-41g5.GenBank:AC099533.36     TTCTGAGCCACTGCAGACTGTTTTTCACAGCGGCTGCAACTTTATCCATTTCTACCATCA 15846
human.FAM30B-LOC105372935-GGT2.NCBI.                                               ttctgagccactgcagactgtttttcacagtggctgcaactttatccatttctaccatca 12278
human.FAM230E-LOC105377182-GGT3P.NCBI.                                             ttctgagccactgcagactgtttttcacagtggctgcaactttatccatttctaccatca 11344
human.FAM230J-LOC105372942-GGTLC5P.NCBI.rev.compl                                  ttctgagccactgcagactgtttttcacagtggctgcaactttatccatttctaccatca 11727
human.FAM230A.ncRNA.18487127.18500594-GGTLC3.18516335.18518165.rev.compl.REF       ttctgagccactgcagactgtttttcacagtggctgcaactttatccatttctaccatca 762
                                                                                   *************************** ** *****************************

chimp.LOC112206744-LOC107973052-GGT2.revcompl                                      atgtatcagggttacaatttctttacgccctcttcacacttattttcctttaaatcatc 14371
chimp54546-135457.revcompl.Pan.troglodytes.clone.rp43-41g5.GenBank:AC099533.36     ATGTATCAGGGTTACAATTTCTTTACATCCTGTTCACACTTATTTTCCTTTAAATCATC 15906
human.FAM30B-LOC105372935-GGT2.NCBI.                                               atgtatcagggttacaatttctttacgtcctcttcacacttattttcctttaaatcatc 12338
human.FAM230E-LOC105377182-GGT3P.NCBI.                                             atgtatcagggttacaatttctttacatccttgttcacacttattttcctttaaatcatc 11404
human.FAM230J-LOC105372942-GGTLC5P.NCBI.rev.compl                                  atgtatcagggttacaatttctttacatctcttgttcacacttattttcctttaaatcatc 11787
human.FAM230A.ncRNA.18487127.18500594-GGTLC3.18516335.18518165.rev.compl.REF       atgtatcagggttacaatttctttacgtccttgttcacacttattttcctttaaatcatc 822
                                                                                   **************************  **** ****************************

chimp.LOC112206744-LOC107973052-GGT2.revcompl                                      ctagtaggtgtatattggtggctgcttgtgtttctcatttgcatttccctaatgactaat 14431
chimp54546-135457.revcompl.Pan.troglodytes.clone.rp43-41g5.GenBank:AC099533.36     CTAGTAGGTGTATATTGGTGGCTGCTTGTGTTTTTCATTTGCATTTCCCTAATGACTAAT 15966
human.FAM30B-LOC105372935-GGT2.NCBI.                                               ctagtaggtgtatattggtggctgcttgtgtttttcatttgcatttccctaatgactaat 12398
human.FAM230E-LOC105377182-GGT3P.NCBI.                                             ctagtaggtgtatattggtggctgcttgtgtttttcatttgcatttccctaatgactaat 11464
human.FAM230J-LOC105372942-GGTLC5P.NCBI.rev.compl                                  ctagtaggtgtatattggtggctgcttgtgtttttcatttgcatttccctaatgactaat 11847
human.FAM230A.ncRNA.18487127.18500594-GGTLC3.18516335.18518165.rev.compl.REF       ctagtaggtgtatattggtggctgcttgtgtttttcatttgcatttccctaatgactaat 882
                                                                                   ********************************* **************************

chimp.LOC112206744-LOC107973052-GGT2.revcompl                                      gatcctgagcagcttttcctgtgctactatctgtggctgtatcttctttagggaaatata 14491
chimp54546-135457.revcompl.Pan.troglodytes.clone.rp43-41g5.GenBank:AC099533.36     GATCCTGAGCAGCTTTTCCTGTGCTACTATCTGTGGCTGTATCTTCTTTAGGGAAATATA 16026
human.FAM30B-LOC105372935-GGT2.NCBI.                                               gatcctgagcagcttttcctgtgctactatctgtggctgtatcttctttagggaaatata 12458
human.FAM230E-LOC105377182-GGT3P.NCBI.                                             gatcctgagcagcttttcctgtgctactatttgtggctatatcttctttagggaaatata 11524
human.FAM230J-LOC105372942-GGTLC5P.NCBI.rev.compl                                  gatcctgagcagcttttcctgtgctactatttgtggctatatcttctttagggaaatata 11907
human.FAM230A.ncRNA.18487127.18500594-GGTLC3.18516335.18518165.rev.compl.REF       gatcctgagcagcttttcctgtgctactatctgtggctgtatcttctttagggaaatata 942
                                                                                   ****************************** ******* *********************

chimp.LOC112206744-LOC107973052-GGT2.revcompl                                      tgttgaagtcttttgcccatttttaaagagttgtctgatttttatttagttagtttgttg 14551
chimp54546-135457.revcompl.Pan.troglodytes.clone.rp43-41g5.GenBank:AC099533.36     TGTTGAAGTCTTTTGCCCATTTTTAAAGAGTTGTCTGATTTTTGTTTAGTTAGTTTGTTG 16086
human.FAM30B-LOC105372935-GGT2.NCBI.                                               tgttgaagtcttttgcccatttttaaagagttgtctgatttttatttagttagtttgttg 12518
human.FAM230E-LOC105377182-GGT3P.NCBI.                                             tgttgaagtcttttgcccatttttaaagagttgtctgatttttatttagttagtttgttg 11584
human.FAM230J-LOC105372942-GGTLC5P.NCBI.rev.compl                                  tgttgaagtcttttgcccatttttaaagagttgtctgatttttatttagttagtttgttg 11967
human.FAM230A.ncRNA.18487127.18500594-GGTLC3.18516335.18518165.rev.compl.REF       tgttgaagtcttttgcccatttttaaagagttgtctgatttttatttagttagtttgttg 1002
                                                                                   ******************************************* ****************

chimp.LOC112206744-LOC107973052-GGT2.revcompl                                      ttgtagattttgaatatatcttaaatatatttaaaatagattctaaattttagtctctt 14611
chimp54546-135457.revcompl.Pan.troglodytes.clone.rp43-41g5.GenBank:AC099533.36     TTGTAGATTTTTGAATATATCTTAAATATATTTAAAATAGATTCTAAATTTTAGTCTCTT 16146
human.FAM30B-LOC105372935-GGT2.NCBI.                                               ttgtagattttgaatatatcttaaatatatttaaaatatattctaaattttagtctctt 12578
human.FAM230E-LOC105377182-GGT3P.NCBI.                                             ctgtagattttgaatatatcttaaatatatttaaaa-atattctaaattttagtctctt 11643
human.FAM230J-LOC105372942-GGTLC5P.NCBI.rev.compl                                  ctgtagattttgaatatatcttaaatatatttaaaa-atattctaaattttagtctctt 12026
human.FAM230A.ncRNA.18487127.18500594-GGTLC3.18516335.18518165.rev.compl.REF       ctgtagattttgaatatatcttaaatatatttaaaa-atattctaaattttagtctctt 1061
                                                                                    *********************************** * *******************

chimp.LOC112206744-LOC107973052-GGT2.revcompl                                      acaagataaatgatttgcaaatatttccacctttgtgtagaactttagattcacaaactt 14671
chimp54546-135457.revcompl.Pan.troglodytes.clone.rp43-41g5.GenBank:AC099533.36     ACAAGATAAATGATTTGCAAATATTTCCCCCTTTGTGTAGAACTTTAGATTCACAAACTT 16206
human.FAM30B-LOC105372935-GGT2.NCBI.                                               acaagagaaatgatttgcaaatatttccacctttgtgtagaactttagattcacaaactt 12638
human.FAM230E-LOC105377182-GGT3P.NCBI.                                             acaagataaatgatttgcaaatatttccccctttgtgtagaactttagattcacaaactt 11703
human.FAM230J-LOC105372942-GGTLC5P.NCBI.rev.compl                                  acaagataaatgatttgcaaatatttccccctttgtgtagaactttagattcacaaactt 12086
human.FAM230A.ncRNA.18487127.18500594-GGTLC3.18516335.18518165.rev.compl.REF       acaagataaatgatttgcaaatatttccccctttgtgtagaactttagattcacaaactt 1121
                                                                                   ****** ********************* *******************************

chimp.LOC112206744-LOC107973052-GGT2.revcompl                                      cattaatttgtgtgaaatcctcagcagttgacccaaacagataagactgaagcagtatt 14731
chimp54546-135457.revcompl.Pan.troglodytes.clone.rp43-41g5.GenBank:AC099533.36     CATTAATTTGTATGAAATCCTCAGCAGTTGACCCCAAAGGATAAGACTGAAGCAGTATT 16266
human.FAM30B-LOC105372935-GGT2.NCBI.                                               cgttaatttgtatgaaatcctcagcagttgacccaaacagataagactgaagcagtatt 12698
human.FAM230E-LOC105377182-GGT3P.NCBI.                                             cattaatttgtatgaaatcctcagcagttgacccaaacagataagactgaagcagtatt 11763
human.FAM230J-LOC105372942-GGTLC5P.NCBI.rev.compl                                  cattaatttgtatgaaatcctcagcagttgacccaaacagataagactgaagcagtatt 12146
human.FAM230A.ncRNA.18487127.18500594-GGTLC3.18516335.18518165.rev.compl.REF       cattaatttgtatgaaatcctcagcagttgacccaaacagataagactgaagcagtatt 1181
                                                                                   * ********* **********************  *******************

chimp.LOC112206744-LOC107973052-GGT2.revcompl                                      ttaggaatagttgaaagtatgatcaccacaaaacataagcgtaatcaaatcctgcaagct 14791
chimp54546-135457.revcompl.Pan.troglodytes.clone.rp43-41g5.GenBank:AC099533.36     TTAGGAATAGTTGAAAGTATGATCACCACAAAACATAAGCGTAATCAAATCCTGCAAGCT 16326
human.FAM30B-LOC105372935-GGT2.NCBI.                                               ttaggaatagttgaaagtatgatcaccacaaaacataagcgtaatcaaatcctgcaagct 12758
human.FAM230E-LOC105377182-GGT3P.NCBI.                                             ttaggaatagttgaaagtatgatcaccacaaaacataagcgtaatcaaatcctgcaagct 11823
human.FAM230J-LOC105372942-GGTLC5P.NCBI.rev.compl                                  ttaggaatagttgaaagtatgatcaccacaaaacataagcgtaatcaaatcctgcaagct 12206
human.FAM230A.ncRNA.18487127.18500594-GGTLC3.18516335.18518165.rev.compl.REF       ttaggaatagttgaaagtatgatcaccacaaaacataagcgtaatcaaatcctgcaagct 1241
                                                                                   ************************************************************

chimp.LOC112206744-LOC107973052-GGT2.revcompl                                      acatgtaaagcacaatgacaaataaggcagcaaagggccatctggtgattagttcaccac 14851
chimp54546-135457.revcompl.Pan.troglodytes.clone.rp43-41g5.GenBank:AC099533.36     ACATGTAAGGCACAATGACAAATAAGGCAGCAAAGGGCCATCTGGTGATTAGTTCACCAC 16386
human.FAM30B-LOC105372935-GGT2.NCBI.                                               acatgtaaggcacaatgacaaataaggcagcaaagggccatctggtgattagttcaccac 12818
human.FAM230E-LOC105377182-GGT3P.NCBI.                                             acatgtaaggcacaatgacaaataaggcagcaaagggccatctggtgattagttcaccac 11883
human.FAM230J-LOC105372942-GGTLC5P.NCBI.rev.compl                                  acatgtaaggcacaatgacaaataaggcagcaaagggccatctggtgattagttcaccac 12266
human.FAM230A.ncRNA.18487127.18500594-GGTLC3.18516335.18518165.rev.compl.REF       acatgtaaggcacaatgacaaataaggcagcaaagggccatctggtgattagttcaccac 1301
                                                                                   ******** ***************************************************

chimp.LOC112206744-LOC107973052-GGT2.revcompl                                      acttgttgcaactgtttgcgctgcagagttaaaacacaccagcattcaacccatgtctcc 14911
chimp54546-135457.revcompl.Pan.troglodytes.clone.rp43-41g5.GenBank:AC099533.36     ACTTGTTGCAACTGTTTGTGCTGCAGAGTTAAAACACACCAGCATTCAACCCATGTCTCC 16446
human.FAM30B-LOC105372935-GGT2.NCBI.                                               acttgttgcaactgtttgcgctgcagagttaaaacacaccagcattcaacccgtgtctcc 12878
human.FAM230E-LOC105377182-GGT3P.NCBI.                                             acttgttgcaactgtttgcgctgcagagttaaaacataccagcattcaacccatgtctcc 11943
human.FAM230J-LOC105372942-GGTLC5P.NCBI.rev.compl                                  acttgttgcaactgtttgcgctgcagagttaaaacataccagcattcaacccatgtctcc 12326
human.FAM230A.ncRNA.18487127.18500594-GGTLC3.18516335.18518165.rev.compl.REF       acttgttgcaactgtttgcactgcagagttaaaacataccagcattcaacccatgtctcc 1361
                                                                                   ******************  **************** *************** *******

chimp.LOC112206744-LOC107973052-GGT2.revcompl                                      tctcttgaggtaaactgtcgtatgttggctggcctgaacaagcgtagatattctccatcc 14971
chimp54546-135457.revcompl.Pan.troglodytes.clone.rp43-41g5.GenBank:AC099533.36     TCTCTTGAAGTAAACTGTCGTATGTTGGCTGGCCTGAACAAGCGTAGATATTCTCCATCC 16506
human.FAM30B-LOC105372935-GGT2.NCBI.                                               tctcttgaagtaaactgtcgtatgttggctggcctgaacaagcgtagatattctccatcc 12938
human.FAM230E-LOC105377182-GGT3P.NCBI.                                             tgtcttgaagtaaactgtcgtatgttggctggcctgaacaagcgtagatattctccatcc 12003
human.FAM230J-LOC105372942-GGTLC5P.NCBI.rev.compl                                  tgtcttgaagtaaactgtcgtatgttggctggcctgaacaagcgtagatattctccatcc 12386
human.FAM230A.ncRNA.18487127.18500594-GGTLC3.18516335.18518165.rev.compl.REF       tgtcttgaagtaaactgtcgtatgttggctggcctgaacaagcgtagatattctccatcc 1421
                                                                                   * ****** ***************************************************

chimp.LOC112206744-LOC107973052-GGT2.revcompl                                      tcaattaatatgcatgcatgacaaagaaaaggaggcctggatgaaaaaatattgtgtgat 15031
chimp54546-135457.revcompl.Pan.troglodytes.clone.rp43-41g5.GenBank:AC099533.36     TCAATTAATATGCATGCATGACAAAGAAAAGGAGGCCTGGATGAAAAAATATTGTGTGAT 16566
human.FAM30B-LOC105372935-GGT2.NCBI.                                               tcaattaatatgcatgcatgacaaagaaaaggaggcctggatgaaaaaatattgtgtgat 12998
human.FAM230E-LOC105377182-GGT3P.NCBI.                                             tcagttaatatgcatgcatgacaaagaaaaggaggcctggatgaaaaaatattgtgtgat 12063
human.FAM230J-LOC105372942-GGTLC5P.NCBI.rev.compl                                  tcaattaatatgcatgcatgacaaagaaaaggaggcctggatgaaaaaatattgtgtgat 12446
human.FAM230A.ncRNA.18487127.18500594-GGTLC3.18516335.18518165.rev.compl.REF       tcaattaatatgcatgcatgacaaagaaaaggaggcctggatgaaaaaatactgtgtgat 1481
                                                                                   *** ************************************************ *******

chimp.LOC112206744-LOC107973052-GGT2.revcompl                                      taataattatgctttaattaattttaaaggatataatttcagtacttctaattctcccat 15091
chimp54546-135457.revcompl.Pan.troglodytes.clone.rp43-41g5.GenBank:AC099533.36     T-ATAATTATGCTTTAATTAATTTTAAAGGATATAATTTCAGTACTTCTAATTCTCCCAT 16625
human.FAM30B-LOC105372935-GGT2.NCBI.                                               taataattatgctttaattaattttaaaggatataatttcagtacttctaattctcccat 13058
human.FAM230E-LOC105377182-GGT3P.NCBI.                                             taataattatgctttaattaattttaaaggatataatttcagtacttctaattctcccat 12123
human.FAM230J-LOC105372942-GGTLC5P.NCBI.rev.compl                                  taataattatgctttaattaattttaaaggatataatttcagtacttctaattctcccat 12506
human.FAM230A.ncRNA.18487127.18500594-GGTLC3.18516335.18518165.rev.compl.REF       taataattatgctttaattaattttaaaggatataatttcagtacttctaattctcccat 1541
                                                                                   * **********************************************************

chimp.LOC112206744-LOC107973052-GGT2.revcompl                                      cagcagttataacaaaggattagtgaataaataccatagactgttttgcctggaattgaa 15151
chimp54546-135457.revcompl.Pan.troglodytes.clone.rp43-41g5.GenBank:AC099533.36     CAGCAGTTATAACAAAGGATTAGTGAATAAATACCATAGACTGTTTTGCCTAGAATTGAA 16685
human.FAM30B-LOC105372935-GGT2.NCBI.                                               cagcagttataacaaaggattagtgaataaataccatagactgttttgcctagaattgaa 13118
human.FAM230E-LOC105377182-GGT3P.NCBI.                                             cagcagttataacaaaggattagtgaataaataccatagactgttttgcctagaattgaa 12183
human.FAM230J-LOC105372942-GGTLC5P.NCBI.rev.compl                                  cagcagttataacaaaggattagtgaataaataccatagactgttttgcctagaattgaa 12566
human.FAM230A.ncRNA.18487127.18500594-GGTLC3.18516335.18518165.rev.compl.REF       cagcagttataacaaaggattagtgaataaataccatagactgttttgcctagaattgaa 1601
                                                                                   *************************************************** ********

chimp.LOC112206744-LOC107973052-GGT2.revcompl                                      tccaatctatctattaaactttgcttttattcaagtgcaaaatgctaaaacac---ataa 15208
chimp54546-135457.revcompl.Pan.troglodytes.clone.rp43-41g5.GenBank:AC099533.36     TCCAATCTATCTATTAAACTTTGCTTTTATTCAAGTGCAAAATGCTAAAACAC---ATAA 16742
human.FAM30B-LOC105372935-GGT2.NCBI.                                               tccaatctatctattaaactttgcttgtattcaagtgcaaaatgctaaaacac---ataa 13175
human.FAM230E-LOC105377182-GGT3P.NCBI.                                             tccaatctgtctattaaactttgcttttattcaagtgcaaaatgctaaaacacataataa 12243
human.FAM230J-LOC105372942-GGTLC5P.NCBI.rev.compl                                  tccaatctgtctattaaactttgcttttattcaagtgcaaaatgctaaaacacataataa 12626
human.FAM230A.ncRNA.18487127.18500594-GGTLC3.18516335.18518165.rev.compl.REF       tccaacctgtctattaaactttgcttttattcaagtgcaaaatgctaaaacacataataa 1661
                                                                                   ***** ** ***************** **************************   ****

chimp.LOC112206744-LOC107973052-GGT2.revcompl                                      ctgcagtgacagccactgtggatcctcagaggtaaaagtagtcttgggacataaatcctg 15268
chimp54546-135457.revcompl.Pan.troglodytes.clone.rp43-41g5.GenBank:AC099533.36     CTGCAGTGACAGCCACTGTGGATCCTCAGAGGTAAAAGTAGTCTTGGGACATAAATCCTG 16802
human.FAM30B-LOC105372935-GGT2.NCBI.                                               ctgcagtgacagccactgtggatcctcagaggtaaaagtagtcttgggacataaatcctg 13235
human.FAM230E-LOC105377182-GGT3P.NCBI.                                             ctgcagtgacagccactgtggatcctcagaggtaaaagtagtcttgggacataaatcctg 12303
human.FAM230J-LOC105372942-GGTLC5P.NCBI.rev.compl                                  ctgcagtgacagccactgtggatcctcagaggtaaaagtagtcttgggacataaatcctg 12686
human.FAM230A.ncRNA.18487127.18500594-GGTLC3.18516335.18518165.rev.compl.REF       ctgcagtgacagccactgtggatcctcagaggtaaaagtagtcttgggacataaatcctg 1721
                                                                                   ************************************************************

chimp.LOC112206744-LOC107973052-GGT2.revcompl                                      caagctaatattgtttttacaggtttagaaaaccatttagctgggtttcaaacctcacag 15328
```

```
chimp54546-135457.revcompl.Pan.troglodytes.clone.rp43-41g5.GenBank:AC099533.36      CAAGCTAATATTGTTTTTACAGGTTTAGAAAACCATTTAGCTGGGTTTCAAACCTCACAG 16862
human.FAM30B-LOC105372935-GGT2.NCBI.                                                 caagctaatattgtttttacaggtttagaaaaccatttagctgggtttcaaacctcacag 13295
human.FAM230E-LOC105377182-GGT3P.NCBI.                                               caag-taatattgtttttacaggtttagaaaaccatttagctgggtttcaaacctcacag 12362
human.FAM230J-LOC105372942-GGTLC5P.NCBI.rev.compl                                    caag-taatattgtttttacaggtttagaaaaccatttagctgggtttcaaacctcacag 12745
human.FAM230A.ncRNA.18487127.18500594-GGTLC3.18516335.18518165.rev.compl.REF         caag-taatattgtttttacaggtttagaaaaccatttagctgggtttcaaacctcacag 1780
                                                                                     **** *******************************************************

chimp.LOC112206744-LOC107973052-GGT2.revcompl                                        tgtgagcagtgggactctcatcaaactatagcatgtgcttcagtaccatttgtagactga 15388
chimp54546-135457.revcompl.Pan.troglodytes.clone.rp43-41g5.GenBank:AC099533.36      TGTGAGCAGTGGGACTCTCATCAAACTATAGCATGTGCTTCAGTACCATTTGTAGACTGA 16922
human.FAM30B-LOC105372935-GGT2.NCBI.                                                 tgtgagcagtgggactctcatcaaactatagcatgtgcttcagtaccatttgtagactga 13355
human.FAM230E-LOC105377182-GGT3P.NCBI.                                               tgtgagcagtgggactctcatcaaactatagcatgtgcttcagtaccatttgtagactga 12422
human.FAM230J-LOC105372942-GGTLC5P.NCBI.rev.compl                                    tgtgagcagtgggactctcatcaaactatagcatgtgcttcagtaccatttgtagactga 12805
human.FAM230A.ncRNA.18487127.18500594-GGTLC3.18516335.18518165.rev.compl.REF         tgtgagcagtgggactctcatcaaactatagcatgtgcttcagtaccatttgtagactga 1840
                                                                                     ************************************************************

chimp.LOC112206744-LOC107973052-GGT2.revcompl                                        ttcattcccatttcttaagttgccatcagcaaaatgccagggactctatttcttgctcc 15448
chimp54546-135457.revcompl.Pan.troglodytes.clone.rp43-41g5.GenBank:AC099533.36      CTCATTCCCATTTCTTAAGTTGCCATCAGCAAAATGCCAGGGACTCTATTTCTTGCTCC 16982
human.FAM30B-LOC105372935-GGT2.NCBI.                                                 ctcattcccatttcttcagttgccatcagcaaaatgccagggactctatttcttgctcc 13415
human.FAM230E-LOC105377182-GGT3P.NCBI.                                               ctcattcccattgccttaagttgccatcagcaaaatgccagggactctatttcttgctcc 12482
human.FAM230J-LOC105372942-GGTLC5P.NCBI.rev.compl                                    ctcattcccattgccttaagttgccatcagcaaaatgccagggactctatttcttgctcc 12865
human.FAM230A.ncRNA.18487127.18500594-GGTLC3.18516335.18518165.rev.compl.REF         ctcattcccattgccttaagttgccatcagcaaaatgccagggactctatttcttgctcc 1900
                                                                                      ***********  *** ******************************************

chimp.LOC112206744-LOC107973052-GGT2.revcompl                                        ttagctcctcgttcttgcctgtctttccacgagggaagattttctagcaggagctcaagc 15508
chimp54546-135457.revcompl.Pan.troglodytes.clone.rp43-41g5.GenBank:AC099533.36      TTAGCTCCTCGTTCTTGCCTGTCTTTCCACGAGGGAAGATTTTCTAGCAGGAGCTCAAGC 17042
human.FAM30B-LOC105372935-GGT2.NCBI.                                                 ttagctcctcgttcttgcctgtctttccacgagggaagattttctagcaggagctcaagc 13475
human.FAM230E-LOC105377182-GGT3P.NCBI.                                               ttagctcctcgttcttgcctgtctttccacgagggaggattttctagcaggagctcaagc 12542
human.FAM230J-LOC105372942-GGTLC5P.NCBI.rev.compl                                    ttagctcctcgttcttgcctgtctttccacgagggaggattttctagcaggagctcaagc 12925
human.FAM230A.ncRNA.18487127.18500594-GGTLC3.18516335.18518165.rev.compl.REF         ttagctcctcgttcttgcctgtctttccacgagggaggattttctagcaggagctcaagc 1960
                                                                                     ************************************ ***********************

chimp.LOC112206744-LOC107973052-GGT2.revcompl                                        tgtgcttttaatgaaacacatccacacacactgtcctgttgtccacattaagcagagctc 15568
chimp54546-135457.revcompl.Pan.troglodytes.clone.rp43-41g5.GenBank:AC099533.36      TGTGCTTTTAATGAAACACATCCACACACACTGTCCTGTTGTCCACATTAAGCAGAGCTC 17102
human.FAM30B-LOC105372935-GGT2.NCBI.                                                 tgtgcttttaatgaaacacatccacacacactgtcctgttgtccacattaagcagagctc 13535
human.FAM230E-LOC105377182-GGT3P.NCBI.                                               tgtgcttttaatgaaacacatccacacacactgtcctgttgtccacattaagcagagctc 12602
human.FAM230J-LOC105372942-GGTLC5P.NCBI.rev.compl                                    tgtgcttttaatgaaacacatccacacacactgtcctgttgtccacattaagcagagctc 12985
human.FAM230A.ncRNA.18487127.18500594-GGTLC3.18516335.18518165.rev.compl.REF         tgtgcttttaatgaaacacatccacacacactgtcctgttgtccacattaagcagagctc 2020
                                                                                     ************************************************************

chimp.LOC112206744-LOC107973052-GGT2.revcompl                                        cctgaataactcatgaacaaaagcatctatgactaactgttgctctgtgtcctcctagcc 15628
chimp54546-135457.revcompl.Pan.troglodytes.clone.rp43-41g5.GenBank:AC099533.36      CCTGAATAACTCATGAACAAAAGCATCTATGACTAACTGTTGCTCTGTGTCCTCCTAGCC 17162
human.FAM30B-LOC105372935-GGT2.NCBI.                                                 cctgaataactcatgaacaaaagcatctatgactaactgttgctctgtgtcctcctagcc 13595
human.FAM230E-LOC105377182-GGT3P.NCBI.                                               cctgaatagctcatgaacaaaagcatctatgactaactgttgctctgtgtcctcctagcc 12662
human.FAM230J-LOC105372942-GGTLC5P.NCBI.rev.compl                                    cctgaatagctcatgaacaaaagcatctatgactaactgttgctctgtgtcctcctagcc 13045
human.FAM230A.ncRNA.18487127.18500594-GGTLC3.18516335.18518165.rev.compl.REF         cctgaataactcatgaacaaaagcatctatgactaactgttgctctgtgtcctcctagcc 2080
                                                                                     ******* ****************************************************

chimp.LOC112206744-LOC107973052-GGT2.revcompl                                        tctgaggagtctctagttcacaaggacagaggagatggagagaggccagtcaacgcgagg 15688
chimp54546-135457.revcompl.Pan.troglodytes.clone.rp43-41g5.GenBank:AC099533.36      TCTGAGGAGTCTCTAGTTCACAAGGACAGAGGAGATGGAGAGAGGCCAGTCAACGCGAGG 17222
human.FAM30B-LOC105372935-GGT2.NCBI.                                                 tctgaggagtctctagttcacaaggacagaggagatggagagaggccagtcaacgcgagg 13655
human.FAM230E-LOC105377182-GGT3P.NCBI.                                               tctgaggagtctctagttcacaaggacagaggagatggagagaggccagtcaacgtgagg 12722
human.FAM230J-LOC105372942-GGTLC5P.NCBI.rev.compl                                    tctgaggagtctctagttcacaaggacagaggagatggagagaggccagtcaacgtgaag 13105
human.FAM230A.ncRNA.18487127.18500594-GGTLC3.18516335.18518165.rev.compl.REF         tctgaggagtctctagttcacaaggacagaggagatggagagaggccagtcaacgtgagg 2140
                                                                                     ******************************************************* ** *

chimp.LOC112206744-LOC107973052-GGT2.revcompl                                        gtaaggttgccttgctttctctgaaatagaaatgttcctttcttggtgtctttctttttc 15748
chimp54546-135457.revcompl.Pan.troglodytes.clone.rp43-41g5.GenBank:AC099533.36      GTAAGGTTGCCTTGCTTTCTCTGAAATAGAAATGTTCCTTTCTTGGTGTCTTTCTTTTTC 17282
human.FAM30B-LOC105372935-GGT2.NCBI.                                                 gtaaggttgccttgctttctctgaaatagaaatgttcctttcttggtgtctttctttttc 13715
human.FAM230E-LOC105377182-GGT3P.NCBI.                                               gtaaggttgccttgctttctctgaaatagaaatgttcctttcttgttgtctttctttttc 12782
human.FAM230J-LOC105372942-GGTLC5P.NCBI.rev.compl                                    gtaaggttgccttgctttctctgaaatagaaatgttcctttcttgttgtctttctttttc 13165
human.FAM230A.ncRNA.18487127.18500594-GGTLC3.18516335.18518165.rev.compl.REF         gtaaggttgccttgctttctctgaaatagaaatgttcctttcttgttgtctttctttttc 2200
                                                                                     ********************************************** *************

chimp.LOC112206744-LOC107973052-GGT2.revcompl                                        aactgactttacatgtgaaaaaatgacaatgtccatgacaggtattaaatgcagttttct 15808
chimp54546-135457.revcompl.Pan.troglodytes.clone.rp43-41g5.GenBank:AC099533.36      AACTGACTTTACATGTGAAAAAATGACAATGTCCATGACAGGTATTAAATGCAGTTTTCT 17342
human.FAM30B-LOC105372935-GGT2.NCBI.                                                 aactgactttacatgtgaaaaaatgacaatgtccatgacaggtattaaatgcagttttct 13775
human.FAM230E-LOC105377182-GGT3P.NCBI.                                               aactgactttacatgtgaaaagatgacaatgtccatgacaggtattaaatgcagttttct 12842
human.FAM230J-LOC105372942-GGTLC5P.NCBI.rev.compl                                    aactgactttacatgtgaaaagatgacaatgtccatgacaggtattaaatgcagttttct 13225
human.FAM230A.ncRNA.18487127.18500594-GGTLC3.18516335.18518165.rev.compl.REF         aactgactttacatgtgaaaagatgacaatgtccatgacaggtattaaatgcagttttct 2260
                                                                                     ******************** ***************************************

chimp.LOC112206744-LOC107973052-GGT2.revcompl                                        gaggggaggaagaagtgactcttagcaactgatatgtaatccaaaatgacatttagcta 15868
chimp54546-135457.revcompl.Pan.troglodytes.clone.rp43-41g5.GenBank:AC099533.36      GAGGGGAGGAAGAAGTGACTCTTAGCAACTGATATGTAATCCAAAATGGCATTTAGCTA 17402
human.FAM30B-LOC105372935-GGT2.NCBI.                                                 gaggggaggaagaagtgactcttagcaactgatatgtaatccaaaatggcatttagcta 13835
human.FAM230E-LOC105377182-GGT3P.NCBI.                                               gaggggaggaagaagtgactcttagcaactgatatgtaatccaaaatggcatttagcta 12902
human.FAM230J-LOC105372942-GGTLC5P.NCBI.rev.compl                                    gaggggaggaagaagtgactcttagcaactgatatgtaatccaaaatggcatttagcta 13285
human.FAM230A.ncRNA.18487127.18500594-GGTLC3.18516335.18518165.rev.compl.REF         gaggggaggaagaagtgactcttagcaactgatatgtaatacaaaatggcatttagcta 2320
                                                                                     ***************************************** ******* **********

chimp.LOC112206744-LOC107973052-GGT2.revcompl                                        tgacggcttcaggttgtagactgtatccttggggtccttgtccttggaagcaatgtcttc 15928
chimp54546-135457.revcompl.Pan.troglodytes.clone.rp43-41g5.GenBank:AC099533.36      TGACGGCTTCAGGTTGTAGACTGTATCCTTGGGGTCCTTGTCCTTGGAAGCAATGTCTTC 17462
human.FAM30B-LOC105372935-GGT2.NCBI.                                                 tgacggcttcaggttgtggactgtatccttggggtccttgtccttggaagcaatgtcttc 13895
human.FAM230E-LOC105377182-GGT3P.NCBI.                                               tgatggcttcaggttgtagactgtatccttggggtccttgtccttggaagcaatgtcttc 12962
human.FAM230J-LOC105372942-GGTLC5P.NCBI.rev.compl                                    tgatggcttcaggttgtagactgtatccttggggtccttgtccttggaagcaatgtcttc 13345
human.FAM230A.ncRNA.18487127.18500594-GGTLC3.18516335.18518165.rev.compl.REF         tgatggcttcaggttgtagactgtatccttggggtccttgtccttggaagcaatgtcttc 2380
                                                                                     *** ************* ******************************************

chimp.LOC112206744-LOC107973052-GGT2.revcompl                                        tccttggattcagtattttgcacttgccaacctacgtggacctgagagatccaccatcca 15988
chimp54546-135457.revcompl.Pan.troglodytes.clone.rp43-41g5.GenBank:AC099533.36      TCCTTGGATTCAGTATTTTGCACTTGCCAACCTACGTGGACCTGAGAGATCCACCATCCA 17522
human.FAM30B-LOC105372935-GGT2.NCBI.                                                 tccttggattcaggattttgcacttgccaacctacgtggacctgagagatccaccatcca 13955
human.FAM230E-LOC105377182-GGT3P.NCBI.                                               tccttggattcagtattttgcacttgccaacctacgtggacctgagagatccgccatcca 13022
human.FAM230J-LOC105372942-GGTLC5P.NCBI.rev.compl                                    tccttggattcagtattttgcacttgccaacctacgtggacctgagagatccgccatcca 13405
human.FAM230A.ncRNA.18487127.18500594-GGTLC3.18516335.18518165.rev.compl.REF         tccttggattcagtattttgcacttgccaacctacgtggacctgagagatccaccatcca 2440
                                                                                     ************* ************************************** *******

chimp.LOC112206744-LOC107973052-GGT2.revcompl                                        gaagctgatgtcttttccagtgtgtagcctacccttgttttggaggccttgaagttgact 16048
chimp54546-135457.revcompl.Pan.troglodytes.clone.rp43-41g5.GenBank:AC099533.36      GAAGCTGATGTCTTTTCCAGTGTGTAGCCTACCCTTGTTTTGGAGGCCTTGAAGTTGACT 17582
human.FAM30B-LOC105372935-GGT2.NCBI.                                                 gaagctgatgtcttttccagtgtgtatcctacccttgttttggaggccttgaagttgact 14015
human.FAM230E-LOC105377182-GGT3P.NCBI.                                               gaagctgatgtcttttccagtgtgtatcctacccttgttttggaggccttgaagttgact 13082
human.FAM230J-LOC105372942-GGTLC5P.NCBI.rev.compl                                    gaagctgatgtcttttccagtgtgtatcctacccttgttttggaggccttgaagttgact 13465
human.FAM230A.ncRNA.18487127.18500594-GGTLC3.18516335.18518165.rev.compl.REF         gaagctgatgtcttttccagtgtgtatcctacccttgttttggaggccttgaagttgact 2500
                                                                                     ************************* **********************************

chimp.LOC112206744-LOC107973052-GGT2.revcompl                                        acactttctgatcaagttttcagtattcattgagagaaacagagccttgtgcaaacaatc 16108
chimp54546-135457.revcompl.Pan.troglodytes.clone.rp43-41g5.GenBank:AC099533.36      ACACTTTCTGATCAAGTTTTCAGTATTCATTGAGAGAAACAGAGCCTTGTGCAAACAATC 17642
human.FAM30B-LOC105372935-GGT2.NCBI.                                                 acactttctgatcaagttttcagtattcattgagagaaacagagccttgtgcaaacaatc 14075
human.FAM230E-LOC105377182-GGT3P.NCBI.                                               acactttctgatcaagttttcaatattcattgagagaaacacagccttgtgcaaacaatc 13142
human.FAM230J-LOC105372942-GGTLC5P.NCBI.rev.compl                                    acactttctgatcaagttttcaatattcattgagagaaacacagccttgtgcaaacaatc 13525
human.FAM230A.ncRNA.18487127.18500594-GGTLC3.18516335.18518165.rev.compl.REF         acactttctgatcaagttttcaatattcattgagagaaacacagccttgtgcaaacaatc 2560
                                                                                     ********************** ****************** ******************

chimp.LOC112206744-LOC107973052-GGT2.revcompl                                        cacaacatgacatacccctcaaaaagctttgtttctgtattgcaggtggtgcaggtggcc 16168
chimp54546-135457.revcompl.Pan.troglodytes.clone.rp43-41g5.GenBank:AC099533.36      CACAACATGACATACCCCTCAAAAAGCTTTGTTTCTGTATTGCAGGTGGTGCAGATGGCC 17702
human.FAM30B-LOC105372935-GGT2.NCBI.                                                 cacaacatgagatacccctgaaaaagctttgtttctgtattgcaggtggtgcaggtggcc 14135
human.FAM230E-LOC105377182-GGT3P.NCBI.                                               cacaacatgacatacccctcaaaaagctttgtttctgtattgcaggtggtgcaggtggcc 13202
human.FAM230J-LOC105372942-GGTLC5P.NCBI.rev.compl                                    cacaacatgacatacccctcaaaaagctttgtttctgtattgcaggtggtgcaggtggcc 13585
human.FAM230A.ncRNA.18487127.18500594-GGTLC3.18516335.18518165.rev.compl.REF         cacaacatgacatacccctcaaaaagctttgtttctgtattgcaggtggtgcaggtggcc 2620
                                                                                     ********** ******* *********************************** *****

chimp.LOC112206744-LOC107973052-GGT2.revcompl                                        cctctgaggtgtgaatctagtaagtattctggaatcacttgccaagaaaacaatctggat 16228
chimp54546-135457.revcompl.Pan.troglodytes.clone.rp43-41g5.GenBank:AC099533.36      CCTCTGAGGTGTGACTCTAGTAAGTATTCTGGAATCACTTGCCAAGAAAACAATCTGGAT 17762
human.FAM30B-LOC105372935-GGT2.NCBI.                                                 cctctgaggcttgaatctagtaagtattctggaatcacttgccaagaaaacaatctggat 14195
human.FAM230E-LOC105377182-GGT3P.NCBI.                                               cctctgaggcgtgaatctagtaagtattctggaatcacttaccaagaaaacaatctggat 13262
human.FAM230J-LOC105372942-GGTLC5P.NCBI.rev.compl                                    cctctgaggcgtgaatctagtaagtattctggaatcacttaccaagaaaacaatctggat 13645
human.FAM230A.ncRNA.18487127.18500594-GGTLC3.18516335.18518165.rev.compl.REF         cctctgaggcatgaatctagtaagtattctggaatcacttaccaagaaaacaatctggat 2680
                                                                                     *********  *** ************************* *******************

chimp.LOC112206744-LOC107973052-GGT2.revcompl                                        gccaagaaaggtgtggcatccttgcctggtttcaatgtgaagagcctccctgatcctggg 16288
chimp54546-135457.revcompl.Pan.troglodytes.clone.rp43-41g5.GenBank:AC099533.36      GCCAAGAAAGGTGTGGCATCCTTGCCTGGTTTCAATGTGAAGAGCCTCCCTGATCCTGGG 17822
human.FAM30B-LOC105372935-GGT2.NCBI.                                                 gccaagaaaggtgtggcatccttgcctggtttcaatgtgaagagcctccctgatcctggg 14255
human.FAM230E-LOC105377182-GGT3P.NCBI.                                               gccaagaaaggtgtggcatccttgcctggtttcaatgtgaagagccaccctgatcctggg 13322
human.FAM230J-LOC105372942-GGTLC5P.NCBI.rev.compl                                    gccaagaaaggtgtggcatccttgcctggtttcaatgtgaagagccaccctgatcctggg 13705
human.FAM230A.ncRNA.18487127.18500594-GGTLC3.18516335.18518165.rev.compl.REF         gccaagaaaggtgtggcatccttgcctggtttcaatgtgaagagccaccctgatcctggg 2740
                                                                                     ********************************************** *************

chimp.LOC112206744-LOC107973052-GGT2.revcompl                                        attgtgataggaataagtataggggaagtgtttttttaaaacctgaattccccagggaaa 16348
chimp54546-135457.revcompl.Pan.troglodytes.clone.rp43-41g5.GenBank:AC099533.36      ATTGTGATAGGAATAAGTATAGGGGAAGTGTTTTTTTAAAACCTGAATTCCCCAGGGAAA 17882
human.FAM30B-LOC105372935-GGT2.NCBI.                                                 attgtgataggaataagtataggggaagtgtttttttaaaacctgaattccccagggaaa 14315
human.FAM230E-LOC105377182-GGT3P.NCBI.                                               attgtgataggaataagtataggggaagtgtttttttaaaacctgaattccccagggaaa 13382
human.FAM230J-LOC105372942-GGTLC5P.NCBI.rev.compl                                    attgtgataggaataagtataggggaagtgtttttttaaaacctgaattccccagggaaa 13765
human.FAM230A.ncRNA.18487127.18500594-GGTLC3.18516335.18518165.rev.compl.REF         attgtgataggaataagtataggggaagtgtttttttaaaacctgaattccccagggaaa 2800
                                                                                     ************************************************************

chimp.LOC112206744-LOC107973052-GGT2.revcompl                                        aattatggccaaattttgaggaagcagttgtgctccctttgggtggtgccgagttgggt 16408
chimp54546-135457.revcompl.Pan.troglodytes.clone.rp43-41g5.GenBank:AC099533.36      AATTATGGCCAAATTTTGAGGAAGCAGTTGTGCTCCCTTTGGGTGGTGCTGAGTTGGGT 17942
```

```
human.FAM30B-LOC105372935-GGT2.NCBI.                                          aattatggccaaattttgaggaagcagctgtgctcccttttgggtggtgctgagttgggt 14375
human.FAM230E-LOC105377182-GGT3P.NCBI.                                        aattatggccaaattttgaggaagcagctgtgctcccttttgggtggtgctgagttgggt 13442
human.FAM230J-LOC105372942-GGTLC5P.NCBI.rev.compl                             aattatggccaaattttgaggaagcagctgtgctcccttttgggtggtgctgagttgggt 13825
human.FAM230A.ncRNA.18487127.18500594-GGTLC3.18516335.18518165.rev.compl.REF  aattatggccaaattttgaggaagcagctgtgctcccttttgggtggtgctgagttgggt 2860
                                                                              *************************** ********************** *********

chimp.LOC112206744-LOC107973052-GGT2.revcompl                                 gcttgaggattggtggtgtcttgtgtgaggctgcatcgtgtggtgtgaatatgtgtgttt 16468
chimp54546-135457.revcompl.Pan.troglodytes.clone.rp43-41g5.GenBank:AC099533.36 GCTTGAGGATTGGTGGTGTCTTGTGTGAGGCTGCATCATGTGGTGTGAATGTGTGTGTTT 18002
human.FAM30B-LOC105372935-GGT2.NCBI.                                          gcttgaggattggtggtgtcttgtgtgaggctgcatcgtgtggtgtgaatgtgtgtgttt 14435
human.FAM230E-LOC105377182-GGT3P.NCBI.                                        gcttgaggattggtggtgtcttgtgtgaggctgcatcgtgtggtgtgaatgtgtgtgttt 13502
human.FAM230J-LOC105372942-GGTLC5P.NCBI.rev.compl                             gcttgaggattggtggtgtcttgtgtgaggctgcatcgtgtggtgtgaatgtgtgtgttt 13885
human.FAM230A.ncRNA.18487127.18500594-GGTLC3.18516335.18518165.rev.compl.REF  gcttgaggattggtggtgtcttgtgtgaggctgcatcgtgtggtgtgaatgtgtgtgttt 2920
                                                                              ************************************* ************ *********

chimp.LOC112206744-LOC107973052-GGT2.revcompl                                 ctgtacaggtgaggctgtgtgttttctcaggagagatttcccacttatacaacccaatca 16528
chimp54546-135457.revcompl.Pan.troglodytes.clone.rp43-41g5.GenBank:AC099533.36 CTGTACAGGTGAGGCTGTGTGTTTTCTCAGGAGAGATTTCCCACTTATACAACCCAATCA 18062
human.FAM30B-LOC105372935-GGT2.NCBI.                                          ctgtacaggtgaggctgtgtgttttctcaggagagatttcccacttatacaacccaatca 14495
human.FAM230E-LOC105377182-GGT3P.NCBI.                                        ctgtacaggtgaggctgtgtgttttctcaggagagatttcccacttatacaacccaatca 13562
human.FAM230J-LOC105372942-GGTLC5P.NCBI.rev.compl                             ctgtacaggtgaggctgtgtgttttctcaggagagatttcccatttatacaacccaatca 13945
human.FAM230A.ncRNA.18487127.18500594-GGTLC3.18516335.18518165.rev.compl.REF  ctgtacaggtgaggctgtgtgttttctcaggagagatttcccacttatacaacccaatca 2980
                                                                              ***************************************** ******************

chimp.LOC112206744-LOC107973052-GGT2.revcompl                                 ccagtgtccacttctaacaataaaatccacccccgctctactctctctgtacagtgactc 16588
chimp54546-135457.revcompl.Pan.troglodytes.clone.rp43-41g5.GenBank:AC099533.36 CCAGTGTCCACTTCTAACAATAAAATCCACCCCCGCTCTACTCTCTCTGTACAGTGACTC 18122
human.FAM30B-LOC105372935-GGT2.NCBI.                                          ccagtgtccacttctaacaataaaatccacccccgctctactctctctgtacagtgactc 14555
human.FAM230E-LOC105377182-GGT3P.NCBI.                                        ccagtgtccacttctaacaataaaatccacccccgctctactctctctgtacagtgactc 13622
human.FAM230J-LOC105372942-GGTLC5P.NCBI.rev.compl                             ccagtgtccacttctaacaataaaatccacccccgctctactctctctgtacagtgactc 14005
human.FAM230A.ncRNA.18487127.18500594-GGTLC3.18516335.18518165.rev.compl.REF  ccagtgtccacttctaacaataaaatccacccccgctctactctctctgtacagtgactc 3040
                                                                              ************************************************************

chimp.LOC112206744-LOC107973052-GGT2.revcompl                                 ctccaccctcactagagccatccccgggtctgccttattatccccactgctcaggtggag 16648
chimp54546-135457.revcompl.Pan.troglodytes.clone.rp43-41g5.GenBank:AC099533.36 CTCCACCCTCACCAGAGCCATCCCCAGATCTGCCTTATTATCCCCACTGCTCAGGTGGAG 18182
human.FAM30B-LOC105372935-GGT2.NCBI.                                          ctccaccctcaccagagccatccccgggtctgccttattatccccactgctcaggtggag 14615
human.FAM230E-LOC105377182-GGT3P.NCBI.                                        ctccaccctcaccagagccatccccgggtctgccttattatccccactgctcaggtggag 13682
human.FAM230J-LOC105372942-GGTLC5P.NCBI.rev.compl                             ctccaccctcaccagagccatccccgggtctgccttattatccccactgctcaggtggag 14065
human.FAM230A.ncRNA.18487127.18500594-GGTLC3.18516335.18518165.rev.compl.REF  ctccaccctcaccagagccatccccgggtctgccttattatccccactgctcaggtggag 3100
                                                                              ************ ************ * ********************************

chimp.LOC112206744-LOC107973052-GGT2.revcompl                                 aacctgaagggccaagggagtggccccagctcccgagttcctgaatgaaaaagtgaaaac 16708
chimp54546-135457.revcompl.Pan.troglodytes.clone.rp43-41g5.GenBank:AC099533.36 AACCTGAAGGGCCAAGGGAGTGGCCCCAGCTCCCGAGTTCCTGAATGAAAAAGTGAAAAC 18242
human.FAM30B-LOC105372935-GGT2.NCBI.                                          aacctgaagggccaagggagtggccccagctcccgagttcctgaatgaaacagtgaaaac 14675
human.FAM230E-LOC105377182-GGT3P.NCBI.                                        aacctgaagggccaagggagtggccccagctcccgagttcctgaatgaaacagtgaaaac 13742
human.FAM230J-LOC105372942-GGTLC5P.NCBI.rev.compl                             aacctgaagggccaagggagtggccccagctcccgagttcctgaatgaaaaagtgaaaac 14125
human.FAM230A.ncRNA.18487127.18500594-GGTLC3.18516335.18518165.rev.compl.REF  aacctgaagggccaagggagtggccccagctcccgagttcctgaatgaaaaagtgaaaac 3160
                                                                              ************************************************* **********

chimp.LOC112206744-LOC107973052-GGT2.revcompl                                 acgaacccagaagtgtgggccagtgctgatgctgacatgcacttagtcatggggtgttca 16768
chimp54546-135457.revcompl.Pan.troglodytes.clone.rp43-41g5.GenBank:AC099533.36 ACGAACCCAGGAGTGTGGGCCAGTGCTGACGCTGACATGCACTTAGTCATGGGGTGTTCA 18302
human.FAM30B-LOC105372935-GGT2.NCBI.                                          acgaacccaggagtgtggaccagtgctgacgctgacatgcacttagtcatggggtgttca 14735
human.FAM230E-LOC105377182-GGT3P.NCBI.                                        acgaacccaggagtgtgggccagtgctgacgctgacatgcacttagtcatggggtgttca 13802
human.FAM230J-LOC105372942-GGTLC5P.NCBI.rev.compl                             acgaacccaggagtgtgggccagtgctgacgctgacatgcacttagtcatggggtgttca 14185
human.FAM230A.ncRNA.18487127.18500594-GGTLC3.18516335.18518165.rev.compl.REF  acgaacccaggagtgtgggccagtgctgacgctgacatgcacttagtcatggggtgttca 3220
                                                                              ********** ******* ********** ******************************

chimp.LOC112206744-LOC107973052-GGT2.revcompl                                 ccaccacacagggagtccagcattcatgtataagccctaaagcaacgagcccaaaaggcc 16828
chimp54546-135457.revcompl.Pan.troglodytes.clone.rp43-41g5.GenBank:AC099533.36 CCACCACACAGGGAGTCCAGCATTCATGTATAAGCCCTAAAGCAACGAGCCCAAAAGGCC 18362
human.FAM30B-LOC105372935-GGT2.NCBI.                                          ccaccacacacggagtccagcattcatgtataagccctaaagcaacgagcccaaaaggcc 14795
human.FAM230E-LOC105377182-GGT3P.NCBI.                                        ccaccacacagggagtccagcattcatgtataaaccctaaggcaccgagcccaaaaggcc 13862
human.FAM230J-LOC105372942-GGTLC5P.NCBI.rev.compl                             ccaccacacagggagtccagcattcatgtataaaccctaaggcaccgagcccaaaaggcc 14245
human.FAM230A.ncRNA.18487127.18500594-GGTLC3.18516335.18518165.rev.compl.REF  ccaccacacagggagtccagcattcatgtataaaccctaaggcaccgagcccaaaaggcc 3280
                                                                              ********** ********************** ****** *** ***************

chimp.LOC112206744-LOC107973052-GGT2.revcompl                                 ccagacactgcccatcatcataaagtggcctccgtggtcacacaacccagggcagttata 16888
chimp54546-135457.revcompl.Pan.troglodytes.clone.rp43-41g5.GenBank:AC099533.36 CCAGACACTGCCCATCATCATAAAGTGGCCTCCGTGGTCACACAACCCAGGGCAGTTATA 18422
human.FAM30B-LOC105372935-GGT2.NCBI.                                          ccagacactgcccatcatcataaagtggcctccgtggtcacacaacccagggcagttata 14855
human.FAM230E-LOC105377182-GGT3P.NCBI.                                        ccaggcactgcccatcatcataaagtggcctccgtggtcacacaacccagggcagttata 13922
human.FAM230J-LOC105372942-GGTLC5P.NCBI.rev.compl                             ccagacactgcccatcatcataaagtggcctccgtggtcacacaacccagggcagttatg 14305
human.FAM230A.ncRNA.18487127.18500594-GGTLC3.18516335.18518165.rev.compl.REF  ccaggcactgcccatcatcataaagtggtctccgtggtcacacaacccagggcagttata 3340
                                                                              **** ********************** *******************************

chimp.LOC112206744-LOC107973052-GGT2.revcompl                                 ggctcatctccccacggacaggcatagtcatcagtgtgtcaaaagcacaaagatccccag 16948
chimp54546-135457.revcompl.Pan.troglodytes.clone.rp43-41g5.GenBank:AC099533.36 GGCTCATCTCCCCACGGACCGGCATAGTCATCAGTGTGTCAAAAGCACAAAGATCCCCAG 18482
human.FAM30B-LOC105372935-GGT2.NCBI.                                          gcctcatctccccacggaccgacatagtcatcagtgtgtcaaaagcacaaagatccccag 14915
human.FAM230E-LOC105377182-GGT3P.NCBI.                                        ggttcatctccccacggacaggcatagtcatcagtgtgtcaaaagcacaaagatccccag 13982
human.FAM230J-LOC105372942-GGTLC5P.NCBI.rev.compl                             ggttcatctccccacggacaggcatagtcatcagtgtgtcaaaagcacaaagatccccag 14365
human.FAM230A.ncRNA.18487127.18500594-GGTLC3.18516335.18518165.rev.compl.REF  ggttcatctccccacggacaggcatagtcatcagtgtgtcaaaagcacaaagatccccag 3400
                                                                              *  **************** * **************************************

chimp.LOC112206744-LOC107973052-GGT2.revcompl                                 gtgtttggctcagctcacagatcccttttttttt----tttaacttttaagttcaggggt 17004
chimp54546-135457.revcompl.Pan.troglodytes.clone.rp43-41g5.GenBank:AC099533.36 GTGTTTGGCTCAGCTCACAGATCCCCCTTTTTTT----TTTAACTTTTAAGTTCAGGGGT 18538
human.FAM30B-LOC105372935-GGT2.NCBI.                                          gtgtttggctcagctcacagatcccttttttt-----atttaacttttcagttcaggggt 14970
human.FAM230E-LOC105377182-GGT3P.NCBI.                                        gtgtttggctcagctcaccgatcctttttttttttt---tttaacttttcagttcaggggt 14039
human.FAM230J-LOC105372942-GGTLC5P.NCBI.rev.compl                             gtgtttggctcagctcaccgatccttttttttttttatttaacttttcagttcaggggt 14425
human.FAM230A.ncRNA.18487127.18500594-GGTLC3.18516335.18518165.rev.compl.REF  gtgtttggctcagctcaccgatcctttttttttttttttttaacttttaagttcaggggt 3460
                                                                              ****************** *****   *****      ********** ***********

chimp.LOC112206744-LOC107973052-GGT2.revcompl                                 acatgtgcaggatgtgcaggtttgctacatatataaatgtgtgtcatgagagtttgttgt 17064
chimp54546-135457.revcompl.Pan.troglodytes.clone.rp43-41g5.GenBank:AC099533.36 ACATGTGCAGGATGTGCAGGTTTGCTACATATATGAATGTGTGTTATGAGAGTTTGTTGT 18598
human.FAM30B-LOC105372935-GGT2.NCBI.                                          acatgtgcaggatgtgcaggtttgctacatatataaatgtgtgttatgagagtttttttgt 15030
human.FAM230E-LOC105377182-GGT3P.NCBI.                                        acatgtgcaggatgtgcaggtttgctacatatataaatgtgtgtcatgagagtttgttgt 14099
human.FAM230J-LOC105372942-GGTLC5P.NCBI.rev.compl                             acatgtgcaggatgtgcaggtttgctacatatataaatgtgtgtcatgagagtttgttgt 14485
human.FAM230A.ncRNA.18487127.18500594-GGTLC3.18516335.18518165.rev.compl.REF  acatgtgcaggatgtgcaggtttgctacatatataaatgtgtgtcatgagagtttgttgt 3520
                                                                              ********************************** ********* ********** ****

chimp.LOC112206744-LOC107973052-GGT2.revcompl                                 acagattattgcatcacccatatattgagcctaatatcagttatttttcctgatcctccc 17124
chimp54546-135457.revcompl.Pan.troglodytes.clone.rp43-41g5.GenBank:AC099533.36 ACAGATTATTGCATCACCCATATATTGAGGCTAATATCAGTTATTTTTCCTGATCCACCC 18658
human.FAM30B-LOC105372935-GGT2.NCBI.                                          acagattattgcatcacccatatattgagcctaatatcagttatttttcctgatccaccc 15090
human.FAM230E-LOC105377182-GGT3P.NCBI.                                        acagattattgcatcacccatatattgagcctaatatcagttatttttcctgatccaccc 14159
human.FAM230J-LOC105372942-GGTLC5P.NCBI.rev.compl                             acagattattgcatcacccatatattgagcctaatatcagttatttttcctgatccaccc 14545
human.FAM230A.ncRNA.18487127.18500594-GGTLC3.18516335.18518165.rev.compl.REF  acagattattgcatcacccatatattgagcctaatatcagttatttttcctgatccaccc 3580
                                                                              ***************************** ************************** ***

chimp.LOC112206744-LOC107973052-GGT2.revcompl                                 cctcctcccacctcccac---cctccagtaggccccacgctcacaaattctaagaggagt 17181
chimp54546-135457.revcompl.Pan.troglodytes.clone.rp43-41g5.GenBank:AC099533.36 CCTCCTCCCACCTCCCACCCTCCTCCAGTAGGCCCCACGCTCACAAATTCTAAGAGGAGT 18718
human.FAM30B-LOC105372935-GGT2.NCBI.                                          cctcctcccacctcccac---cctccagtaggccccacgctcacaaattctaagaggagt 15147
human.FAM230E-LOC105377182-GGT3P.NCBI.                                        tctcctcccacctcccac---cctccagtaggccccacgctcacaaattctaagaggagt 14216
human.FAM230J-LOC105372942-GGTLC5P.NCBI.rev.compl                             cctcctcccacctcccac---cctccagtaggccccacgctcacaaattctaagaggagt 14602
human.FAM230A.ncRNA.18487127.18500594-GGTLC3.18516335.18518165.rev.compl.REF  cctcctcccacctcccac---cctccagtaggccccacgctcacaaattctaagaggagt 3637
                                                                               *****************   ***************************************

chimp.LOC112206744-LOC107973052-GGT2.revcompl                                 gggggaccacaaaggccagtgtggcccacttcagttgtgaagttaatttgctcagcaact 17241
chimp54546-135457.revcompl.Pan.troglodytes.clone.rp43-41g5.GenBank:AC099533.36 GGGGGACCACAAAGGCCAGTGTGGCCCACTTCAGTTGTGAAGTTAATCTGCTCAGCAACT 18778
human.FAM30B-LOC105372935-GGT2.NCBI.                                          gggggaccacaaaggccagtgtggcccacttcagttgtgaagttaatttgctcagcaact 15207
human.FAM230E-LOC105377182-GGT3P.NCBI.                                        gggggaccacaaaggccagtgtggcccacttcagttgtgaagttaatttgctcagcaact 14276
human.FAM230J-LOC105372942-GGTLC5P.NCBI.rev.compl                             gggggaccacaaaggccagtgtggcccacttcagttgtgaagttaatttgctcagcaact 14662
human.FAM230A.ncRNA.18487127.18500594-GGTLC3.18516335.18518165.rev.compl.REF  gggggaccacaaaggccagtgtggcccacttcagttgtgaagttaatttgctcagcaact 3697
                                                                              *********************************************** ************

chimp.LOC112206744-LOC107973052-GGT2.revcompl                                 ggccaaagtctataaggatgggtgatgtattttagtagatttagtaatactatcttccca 17301
chimp54546-135457.revcompl.Pan.troglodytes.clone.rp43-41g5.GenBank:AC099533.36 GGCCAAAGTCTATAAGGATGGGTGATGTATTTTAGTAGATTTAGTAATACTATCTTCCCA 18838
human.FAM30B-LOC105372935-GGT2.NCBI.                                          ggccaaagtctataaggatgggtgatgtattttagtagatttagtaatactatcttccca 15267
human.FAM230E-LOC105377182-GGT3P.NCBI.                                        ggccaaagtctataaggatgggtgatgtattttagtagatttagtaatactatcttccca 14336
human.FAM230J-LOC105372942-GGTLC5P.NCBI.rev.compl                             ggccaaagtctataaggatgggtgatgtattttagtagatttagtaatactatcttccca 14722
human.FAM230A.ncRNA.18487127.18500594-GGTLC3.18516335.18518165.rev.compl.REF  ggccaaagtctataaggatgggtgatgtattttagtagatttagtaatactatcttccca 3757
                                                                              ************************************************************

chimp.LOC112206744-LOC107973052-GGT2.revcompl                                 agccctaaaatgctcaaatcctgccagccaaaaatggtgaggagggacagataggaactc 17361
chimp54546-135457.revcompl.Pan.troglodytes.clone.rp43-41g5.GenBank:AC099533.36 AGCCCTAAAATGCTCAAATCCTGCCAGCCAAAAATGGTGAGGAGGGACAGATAGGAACTC 18898
human.FAM30B-LOC105372935-GGT2.NCBI.                                          agccctaaaatgctcaaatcctgccagccaaaaatggtgaggagggacagataggaactc 15327
human.FAM230E-LOC105377182-GGT3P.NCBI.                                        agccctaaaatgctcaaatcctgccagccaaaaatggtgaggagggacagataggaactc 14396
human.FAM230J-LOC105372942-GGTLC5P.NCBI.rev.compl                             agccctaaaatgctcaaatcctgccagccaaaaatggtgaggagggacagataggaactc 14782
human.FAM230A.ncRNA.18487127.18500594-GGTLC3.18516335.18518165.rev.compl.REF  agccctaaaatgctcaaatcctgccagccaaaaatggtgaggagggacagataggaactc 3817
                                                                              ************************************************************

chimp.LOC112206744-LOC107973052-GGT2.revcompl                                 tgtgtggcacttggttattagcctggcttccatcccttagtggcaagtctcttgtatatg 17421
chimp54546-135457.revcompl.Pan.troglodytes.clone.rp43-41g5.GenBank:AC099533.36 TGTGTGGCACTTGGTTATTAGCCTGGCTTCCATCCCTTAGTGGCAACTCTCTTGTATATG 18958
human.FAM30B-LOC105372935-GGT2.NCBI.                                          tgtgtggcacttggttattagcctggcttccatcccttagtggcaactctcttgtatatg 15387
human.FAM230E-LOC105377182-GGT3P.NCBI.                                        tgtgtggcacttggttattagcctggcttccatcccttagtggcaactctcttgtatatg 14456
human.FAM230J-LOC105372942-GGTLC5P.NCBI.rev.compl                             tgtgtggcacttggttattagcctggcttccatcccttagtggcaactctcttgtatatg 14842
human.FAM230A.ncRNA.18487127.18500594-GGTLC3.18516335.18518165.rev.compl.REF  tgtgtggcacttggttattagcctggcttccatcccttagtggcaactctcttgtatatg 3877
                                                                              ******************************************** ***************

chimp.LOC112206744-LOC107973052-GGT2.revcompl                                 tgggttaaagaccctcagcctcaagccaagcctcctccatgaggagccatctcactattg 17481
chimp54546-135457.revcompl.Pan.troglodytes.clone.rp43-41g5.GenBank:AC099533.36 TGGGTTAAAGACCCTCAGCCTCAAGCCAAGCCTCCTCCATGAGGAGCCATCTCACTATTG 19018
human.FAM30B-LOC105372935-GGT2.NCBI.                                          tgggttaaagaccctcagcctcaagccaagcctcctccatgaggagccatctcactattg 15447
```

```
human.FAM230E-LOC105377182-GGT3P.NCBI.                                          tgggttaaagaccctcagcctcaagccaagcctcctccatgaggagccatctcactattg 14516
human.FAM230J-LOC105372942-GGTLC5P.NCBI.rev.compl                               tgggttaaagaccctcagcctcaagccaagcctcctccatgaggagccatctcactattg 14902
human.FAM230A.ncRNA.18487127.18500594-GGTLC3.18516335.18518165.rev.compl.REF    tgggttaaagaccctcagcctcaagccaagcctcctccatgaggagccatctcactattg 3937
                                                                                ************************************************************

chimp.LOC112206744-LOC107973052-GGT2.revcompl                                   actggctagtgccgggtatggccaccagcccaactgaaacaaaatgttgctttaaaacaa 17541
chimp54546-135457.revcompl.Pan.troglodytes.clone.rp43-41g5.GenBank:AC099533.36  ACTGGCTAGTGCCGGGTATGGCCACCAGCCCAACTGAAACAAAATGTTGCTTTAAAACAA 19078
human.FAM30B-LOC105372935-GGT2.NCBI.                                            accggctagtgccgggtatggccaccagcccaactgaaacaaaatgttgctttaaaacaa 15507
human.FAM230E-LOC105377182-GGT3P.NCBI.                                          accggctagtgccgggtatggccaccagcccaactgaaacaaaatgttgctttaaaacaa 14576
human.FAM230J-LOC105372942-GGTLC5P.NCBI.rev.compl                               actggctagtgccgggtatggccaccagcccaactgaaacaaaatgttgctttaaaacaa 14962
human.FAM230A.ncRNA.18487127.18500594-GGTLC3.18516335.18518165.rev.compl.REF    actggctagtgccgggtatggccaccagcccaactgaaacaaaatgttgctttaaaacaa 3997
                                                                                ** *********************************************************

chimp.LOC112206744-LOC107973052-GGT2.revcompl                                   gtgtaaatctcatacatacaacaggcaaatgcagaagcagtgtggtctcgcaagttgtaa 17601
chimp54546-135457.revcompl.Pan.troglodytes.clone.rp43-41g5.GenBank:AC099533.36  GTGTAAATCTCATACATACAACAGGCAAATGCAGAAGCAGTGTGGTCTCGCAAGTTGTAA 19138
human.FAM30B-LOC105372935-GGT2.NCBI.                                            gtgtaaatctcatacatacaacaggcaaatgcagaagcagtgtggtctcgcaagttgtaa 15567
human.FAM230E-LOC105377182-GGT3P.NCBI.                                          gtgtaaatctcgtacatacaacaggcaaatgcagaagcagtgtggtctcgcaagttgtaa 14636
human.FAM230J-LOC105372942-GGTLC5P.NCBI.rev.compl                               gtgtaaatctcatacatacaacaggcaaatgcagaagcagtgtggtctcgcaagttgtaa 15022
human.FAM230A.ncRNA.18487127.18500594-GGTLC3.18516335.18518165.rev.compl.REF    gtgtaaatctcatacatacaacaggcaaatgcagaagcagtgtggtctcgcaagttgtaa 4057
                                                                                *********** ************************************************

chimp.LOC112206744-LOC107973052-GGT2.revcompl                                   agaggacagtcgcaattttgctggacttcaacctgggtagaagacatgagggaactctgt 17661
chimp54546-135457.revcompl.Pan.troglodytes.clone.rp43-41g5.GenBank:AC099533.36  AGAGGACAGTCGCAATTTTGCTGGACTTCAACCTGGGTAGAAGACATGAGGGAACTCTGT 19198
human.FAM30B-LOC105372935-GGT2.NCBI.                                            agaggacagtcgcaattttgctggacttcaacctgggtagaagacatgagggaac-ctgt 15626
human.FAM230E-LOC105377182-GGT3P.NCBI.                                          agaggacagtcgcaattttgctggacttcaacctgggtagaagacacgagggaactctgt 14696
human.FAM230J-LOC105372942-GGTLC5P.NCBI.rev.compl                               agaggacagtcgcaattttgctggacttcaacctgggtagaagacacgagggaactctgt 15082
human.FAM230A.ncRNA.18487127.18500594-GGTLC3.18516335.18518165.rev.compl.REF    agaggacagtcgcaattttgctggacttcaacctgggtagaagacacgagggaactctgt 4117
                                                                                ********************************************* ********* ****

chimp.LOC112206744-LOC107973052-GGT2.revcompl                                   cactaaatcacggcagagttcaaggccacttgcagactatttcatgttacagaaggtggc 17721
chimp54546-135457.revcompl.Pan.troglodytes.clone.rp43-41g5.GenBank:AC099533.36  CACTGAATCACGGCAGAGTTCAAGGCCACTTGCAGACTATTTCATGTTATAGAAGGTGGC 19258
human.FAM30B-LOC105372935-GGT2.NCBI.                                            cactaaatcacggcagagttcaaggccacttgtagactatttcatgttacagaaggtggc 15686
human.FAM230E-LOC105377182-GGT3P.NCBI.                                          cactaaatcacggcagagttcaaggccgcttgtagactatttcgtgttatagaaggtggc 14756
human.FAM230J-LOC105372942-GGTLC5P.NCBI.rev.compl                               cactaaatcacggcagagttcaagg-----------ctatttcgtgttatagaaggtggc 15131
human.FAM230A.ncRNA.18487127.18500594-GGTLC3.18516335.18518165.rev.compl.REF    cactaaatcacggcagagttcaaggccgcttgtagactatttcgtgttatagaaggtggc 4177
                                                                                **** ********************           ******* ***** **********

chimp.LOC112206744-LOC107973052-GGT2.revcompl                                   ctttagctactaagcaaaggcctctgtttctcatttctttcctgttcatcttcttggtca 17781
chimp54546-135457.revcompl.Pan.troglodytes.clone.rp43-41g5.GenBank:AC099533.36  CTTTAGCTACTAAGCAAAGGCCTCTGTTTCTCATTTCTTTCCTGTTCATCTTCTTGGTCA 19318
human.FAM30B-LOC105372935-GGT2.NCBI.                                            ctttagctactaagcaaaggcctctgtttctcatttctttcctgttcatcttcttggtca 15746
human.FAM230E-LOC105377182-GGT3P.NCBI.                                          ctttagctactaggcaaaggcctctgtttctcatttctttcctgttcatctccttggtca 14816
human.FAM230J-LOC105372942-GGTLC5P.NCBI.rev.compl                               ctttagctactaggcaaaggcctctgtttctcatttctttcctgttcatctccttggtca 15191
human.FAM230A.ncRNA.18487127.18500594-GGTLC3.18516335.18518165.rev.compl.REF    ctttagctactaagcaaaggcctctgtttctcatttctttcctgttcatctccttggtca 4237
                                                                                ************ ************************************** ********

chimp.LOC112206744-LOC107973052-GGT2.revcompl                                   tccttcttccgcaagggaaacgagcccaagcaaaaggcagtttcaatattaatttgaccg 17841
chimp54546-135457.revcompl.Pan.troglodytes.clone.rp43-41g5.GenBank:AC099533.36  TCCTTCTTCCGCAAGGGAAATGAGCCCAAGCAAAAGGCAGTTTCAATATTAATTTGACCG 19378
human.FAM30B-LOC105372935-GGT2.NCBI.                                            tccttcttccgcaagggaaacgagcccaagcaaaaggcagtttcaatattaatttgaccg 15806
human.FAM230E-LOC105377182-GGT3P.NCBI.                                          tccttcttccgcaagggaaacgagcccaagcaaaaggcagtttcaatattaatttgaccg 14876
human.FAM230J-LOC105372942-GGTLC5P.NCBI.rev.compl                               tccttcttccgcaagggaaacgagcccaagcaaaaggcagtttcaatattaatttgaccg 15251
human.FAM230A.ncRNA.18487127.18500594-GGTLC3.18516335.18518165.rev.compl.REF    tccttcttccacaagggaaacgagcccaagcaaaaggcagtttcaatattaatttgaccg 4297
                                                                                ********** ********* ***************************************

chimp.LOC112206744-LOC107973052-GGT2.revcompl                                   aggttttgtgcagtttattatcatccaggtaatcaagtgcaacccagtctgcctagcagc 17901
chimp54546-135457.revcompl.Pan.troglodytes.clone.rp43-41g5.GenBank:AC099533.36  AGGTTTTGTGCAGTTTATTATCATCCAGGTAATCAAGTGCAACCCAGTCTGCCTAGCAGC 19438
human.FAM30B-LOC105372935-GGT2.NCBI.                                            aggttttgtgcagtttattatcatccaggtaatcaggtgcaacccagtctgcctagccgc 15866
human.FAM230E-LOC105377182-GGT3P.NCBI.                                          aggttttgtgcagttaattatcatccaggtaatcaggtgcaacccagtctgcctagcagc 14936
human.FAM230J-LOC105372942-GGTLC5P.NCBI.rev.compl                               aggttttgtgcagttaattatcatccaggtaatcaggtgcaacccagtctgcctagcagc 15311
human.FAM230A.ncRNA.18487127.18500594-GGTLC3.18516335.18518165.rev.compl.REF    aggttttgtgcagttaattatcatccaggtaatcaggtgcaacccagtctgcctagcagc 4357
                                                                                *************** ******************* ********************* **

chimp.LOC112206744-LOC107973052-GGT2.revcompl                                   ccccctatctctgctctgtgttttcatttaataaacattttggtctacttactatgtgct 17961
chimp54546-135457.revcompl.Pan.troglodytes.clone.rp43-41g5.GenBank:AC099533.36  CCCCCTATCTCTGCTCTGTGTTTTCATTTAATAAACATTTTGGTCTACTTACTATGTGCT 19498
human.FAM30B-LOC105372935-GGT2.NCBI.                                            ccccctatctctgctctgtgttttcatttaataaacattttggtctacttactatgtgct 15926
human.FAM230E-LOC105377182-GGT3P.NCBI.                                          ccccctatctctgctctgtgttttcatttaataaacattttggtctacttactatgtgct 14996
human.FAM230J-LOC105372942-GGTLC5P.NCBI.rev.compl                               ccccctatctctgctctgtgttttcatttaatgaacattttggtctacttactatgtgct 15371
human.FAM230A.ncRNA.18487127.18500594-GGTLC3.18516335.18518165.rev.compl.REF    ccccctatctctgctctgtgttttcatttaataaacattttggtctacttactatgtgct 4417
                                                                                ******************************** ***************************

chimp.LOC112206744-LOC107973052-GGT2.revcompl                                   agattttctcgagaccaagtaaatgagatagaatctttatgttggcagctaagttagatt 18021
chimp54546-135457.revcompl.Pan.troglodytes.clone.rp43-41g5.GenBank:AC099533.36  AGATTTTCTCGAGACCAAGTAAATGAGATAGAATCTTTATGTTGGCAGCTAAGTTAGATT 19558
human.FAM30B-LOC105372935-GGT2.NCBI.                                            agattttctcgagaccaagtaaatgagatagaatctttatgctggcagctaagttagatt 15986
human.FAM230E-LOC105377182-GGT3P.NCBI.                                          agattttctcgagaccaagtaaatgagatagaatctttatgttggcagctaagttagatt 15056
human.FAM230J-LOC105372942-GGTLC5P.NCBI.rev.compl                               agattttctcgagaccaagtaaatgagatagaatctttatgttggcagctaagttagatt 15431
human.FAM230A.ncRNA.18487127.18500594-GGTLC3.18516335.18518165.rev.compl.REF    agattttctcgagaccaagtaaatgagatagaatccttatgttggcagctaagttagatt 4477
                                                                                *********************************** ***** ******************

chimp.LOC112206744-LOC107973052-GGT2.revcompl                                   taacataactgacaaaaattaaaatttctgatttcttgtaaaaatattttgtatgtgtga 18081
chimp54546-135457.revcompl.Pan.troglodytes.clone.rp43-41g5.GenBank:AC099533.36  TAACATAACTGACAAAAATTAAAATTTCTGATTTCTTGTAAAAATATTTTGTATGTGTGA 19618
human.FAM30B-LOC105372935-GGT2.NCBI.                                            tcacataactgacaaaaattaaaatttctgatttcttgtaaaaatattttgtatgtgtga 16046
human.FAM230E-LOC105377182-GGT3P.NCBI.                                          tcacataactgacaaaaattaaaatttctgatttcttgtaaaaatattttgtatgtgtga 15116
human.FAM230J-LOC105372942-GGTLC5P.NCBI.rev.compl                               tcgcataactgacaaaaattaaaatttctgatttcttgtaaaaatattttgtatgtgtga 15491
human.FAM230A.ncRNA.18487127.18500594-GGTLC3.18516335.18518165.rev.compl.REF    tcacataactgacaaaaattaaaatttctgatttcttgtaaaaatattttgtatgtgtga 4537
                                                                                *  *********************************************************

chimp.LOC112206744-LOC107973052-GGT2.revcompl                                   atgcatactgaatgtaaagtggataaaaaactcacacttgcactcatggaaggcttttca 18141
chimp54546-135457.revcompl.Pan.troglodytes.clone.rp43-41g5.GenBank:AC099533.36  ATGCATACTGAATGTAAAGTGGATAAAAAACTCACACTTGCACTCATGGAAGGCTTTTCA 19678
human.FAM30B-LOC105372935-GGT2.NCBI.                                            atgcatactgaatgtaaagtggataaaaaactcacacttgcactcatggaaggcttttca 16106
human.FAM230E-LOC105377182-GGT3P.NCBI.                                          atgcatactgaatgtaaagtggataaaaaactcacacttgcactcatggaaggcttttca 15176
human.FAM230J-LOC105372942-GGTLC5P.NCBI.rev.compl                               atgcatactgaatgtaaagtggataaaaaactcacacttgcactcatggaaggcttttca 15551
human.FAM230A.ncRNA.18487127.18500594-GGTLC3.18516335.18518165.rev.compl.REF    atgcatactgaatgtaaagtggataaaaaactcacacttgcactcatggaaggcttttca 4597
                                                                                ************************************************************

chimp.LOC112206744-LOC107973052-GGT2.revcompl                                   tgaatttgtcaattttatttttatatttccccacttcaccggataatgcatacctgaa 18201
chimp54546-135457.revcompl.Pan.troglodytes.clone.rp43-41g5.GenBank:AC099533.36  TGAATTTGTCAATTTTATTTTTATATTTCCCCACTTCACCGGATAATGCATACCTGAA 19738
human.FAM30B-LOC105372935-GGT2.NCBI.                                            tgaatttgtcaattttatttttatatttccccacttcaccagataatgcatacctgaa 16166
human.FAM230E-LOC105377182-GGT3P.NCBI.                                          tgaatttgtcaattttatttttatatttccccacttcactggataatgcatacctgaa 15236
human.FAM230J-LOC105372942-GGTLC5P.NCBI.rev.compl                               tgaatttgtcaattttatttttatatttccccacttcactggataatgcatacctgaa 15611
human.FAM230A.ncRNA.18487127.18500594-GGTLC3.18516335.18518165.rev.compl.REF    tgaatttgtcaattttatttttatatttccccacttcactggataatgcatacctgaa 4657
                                                                                ***************************************  *******************

chimp.LOC112206744-LOC107973052-GGT2.revcompl                                   cctggaaattgattcccactgcagaaagtgttctgagccacatcccttagcttcactagt 18261
chimp54546-135457.revcompl.Pan.troglodytes.clone.rp43-41g5.GenBank:AC099533.36  CCTGGAAACTGATTCCCACTGCAGAAAGTGTTCTGAGCCACATCCCTTAGCTTCACTAGT 19798
human.FAM30B-LOC105372935-GGT2.NCBI.                                            cctggaaactgattcccactgcagaaagtgttctgagccacatcccttagcttcactagt 16226
human.FAM230E-LOC105377182-GGT3P.NCBI.                                          cctggaaactgatgcccactgcagaaagtgttctgagccacatcccttagcttcactagt 15296
human.FAM230J-LOC105372942-GGTLC5P.NCBI.rev.compl                               cctggaaactgatgcccactgcagaaagtgttctgagccacatcccttagcttcactagt 15671
human.FAM230A.ncRNA.18487127.18500594-GGTLC3.18516335.18518165.rev.compl.REF    cctggaaactgatgcccactgcagaaagtgttctgagccacatcccttagcttcactagt 4717
                                                                                ******** **** **********************************************

chimp.LOC112206744-LOC107973052-GGT2.revcompl                                   gcaggtccacctgggaggatgtcccagcatcagcttggcccatgctgtgatcagccacct 18321
chimp54546-135457.revcompl.Pan.troglodytes.clone.rp43-41g5.GenBank:AC099533.36  GCAGGTCCACCTGGGAGGATGTCCCAGCATCAGCTTGGCCCATGCTGTGATCAGCCACCT 19858
human.FAM30B-LOC105372935-GGT2.NCBI.                                            gcaggtccacctgggaggatgtcccagcatcagcttgacccatgctgtgatcagccacct 16286
human.FAM230E-LOC105377182-GGT3P.NCBI.                                          gcaggtccacctgggaggatgtcccagcatcagcttggcccatgctgtgatcagccacct 15356
human.FAM230J-LOC105372942-GGTLC5P.NCBI.rev.compl                               gcaggtccacctgggaggatgtcccagcatcagcttggcccatgctgtgatcagccacct 15731
human.FAM230A.ncRNA.18487127.18500594-GGTLC3.18516335.18518165.rev.compl.REF    gcaggtccacctgggaggatgtcccagcatcagcttggcccatgctgtgatcagccacct 4777
                                                                                ************************************* **********************

chimp.LOC112206744-LOC107973052-GGT2.revcompl                                   ccatgcaccacaccaagcaagcccctgggtgattcacagtctccaccaccagggcactga 18381
chimp54546-135457.revcompl.Pan.troglodytes.clone.rp43-41g5.GenBank:AC099533.36  CCATGCACCACACCAAGCAAGCCCCTGGGTGATTCACAGTCTCCACCACCAGGGCACTGA 19918
human.FAM30B-LOC105372935-GGT2.NCBI.                                            ccatgcatcacaccaagcaagcccctgggtgattcacagtctccaccaccagggcactga 16346
human.FAM230E-LOC105377182-GGT3P.NCBI.                                          ccatgcaccacaccaagcaagcccctgggtgattcacagtctccactaccagggcactga 15416
human.FAM230J-LOC105372942-GGTLC5P.NCBI.rev.compl                               ccatgcaccacaccaagcaagcccctgggtgattcacagtctccactaccagggcactga 15791
human.FAM230A.ncRNA.18487127.18500594-GGTLC3.18516335.18518165.rev.compl.REF    ccatgcaccacaccaagcaagcccctgggtgattcacagtctccactaccagggcactga 4837
                                                                                ******* ************************************** *************

chimp.LOC112206744-LOC107973052-GGT2.revcompl                                   ccttaactgtgtgttcttctagctccccatgaagacaccgtacacgacatcactaacgag 18441
chimp54546-135457.revcompl.Pan.troglodytes.clone.rp43-41g5.GenBank:AC099533.36  CCTTAACTCTGTGTTCTTCTAGCTCCCCATGAGGACACCGTACACGACATCACTAACGAG 19978
human.FAM30B-LOC105372935-GGT2.NCBI.                                            ccttaactctgtgtttttctagctccccatgaggacaccgtacacgacatcactaacgag 16406
human.FAM230E-LOC105377182-GGT3P.NCBI.                                          ccttaactctgtgttcttctagctccccatgaggacaccgtacacaacatcactaacgag 15476
human.FAM230J-LOC105372942-GGTLC5P.NCBI.rev.compl                               ccttaactctgtgttcttctagctccccatgaggacaccgtacacaacatcactaacgag 15851
human.FAM230A.ncRNA.18487127.18500594-GGTLC3.18516335.18518165.rev.compl.REF    ccttaactctgtgttcttctagctccccatgaggacaccgtacacaacatcactaacgag 4897
                                                                                ******** ****** **************** *********** ***************

chimp.LOC112206744-LOC107973052-GGT2.revcompl                                   gacgccacacacgacatcgccaacgaggacactgtacatgacaccgctaacgaggccgcc 18501
chimp54546-135457.revcompl.Pan.troglodytes.clone.rp43-41g5.GenBank:AC099533.36  GACGCCACACAGGACATCGCTAACAAGGACACTGTATACGACATCGCTAACGAGGCCGCC 20038
human.FAM30B-LOC105372935-GGT2.NCBI.                                            gacgccacacacgacatcgctaacgaggatactgtacacgacatcgctaacgaggccgcc 16466
human.FAM230E-LOC105377182-GGT3P.NCBI.                                          gatgcctcacacgatatcactaacgaggacgctgtccacggcatcgctaacgaggccgcc 15536
human.FAM230J-LOC105372942-GGTLC5P.NCBI.rev.compl                               gatgcctcacacgatatcactaacgaggacgctgtccacggcatcgctaacgaggccgcc 15911
human.FAM230A.ncRNA.18487127.18500594-GGTLC3.18516335.18518165.rev.compl.REF    gatgcctcacacgatatcactaacgaggacgctgtccacggcatcgctaacgaggccgcc 4957
                                                                                ** *** **** ** *** * *** ****  ****  * * ** ****************

chimp.LOC112206744-LOC107973052-GGT2.revcompl                                   gacaagggcatcgccagcgaggacgccgcccagggcatcgccaacgaggccgccgcccag 18561
chimp54546-135457.revcompl.Pan.troglodytes.clone.rp43-41g5.GenBank:AC099533.36  GACAAGGGCATCGCCAACGAGGACGCCGCCCAGGGCATCGCCAACGAGGACGCCTCCCAC 20098
human.FAM30B-LOC105372935-GGT2.NCBI.                                            gacaagggcatcgccaacgaggatgccgcccacggcatcgccaacgagtacgccgcccac 16526
human.FAM230E-LOC105377182-GGT3P.NCBI.                                          gcccagggcatcgccaacgaggacgccgcccagggcatcgccaacgaggacgccgcccac 15596
```

```
human.FAM230J-LOC105372942-GGTLC5P.NCBI.rev.compl                               gacaagggcatcgccaacgaggacgccgcccagggcatcgccaacgaggacgccgcccac 15971
human.FAM230A.ncRNA.18487127.18500594-GGTLC3.18516335.18518165.rev.compl.REF    gacaagggcaacgccaacgaggacgccgcccagggcatcgccaacgaggacgccgcccac 5017
                                                                                * * ****** ***** ****** ******** ***************  **** ****

chimp.LOC112206744-LOC107973052-GGT2.revcompl                                   ggcatcgtcagcgaggacgccgcccacggaatcgccaacgaggacgccgtccagggcatc 18621
chimp54546-135457.revcompl.Pan.troglodytes.clone.rp43-41g5.GenBank:AC099533.36  GGCATCGCCAACGAGGACACCGCCCACGGCATCGCCAGCGAGGACGCCGCCCACGGAATC 20158
human.FAM30B-LOC105372935-GGT2.NCBI.                                            ggcatcgccagcgaggacgccgcccacggcatcgccagcgaggacgccgcccagggcatc 16586
human.FAM230E-LOC105377182-GGT3P.NCBI.                                          ggaatcgccagcgaggacgccgcccagggcatcgccaacgaggtcgccgcccagggcatc 15656
human.FAM230J-LOC105372942-GGTLC5P.NCBI.rev.compl                               ggaatcgccagcgaggacgccgcccagggcatcgccaacgaggtcgccgcccagggcatc 16031
human.FAM230A.ncRNA.18487127.18500594-GGTLC3.18516335.18518165.rev.compl.REF    ggaatcgccagcgaggacgccgcccagggcatcgccaacgaggtcgccgcccagggcatc 5077
                                                                                ** **** ** ******* ******* ** ******* ***** ***** *** ** ***

chimp.LOC112206744-LOC107973052-GGT2.revcompl                                   gccaacgtggtcgccgcccacggaatcgccaacgaggacgccgcccagggcatcgc---- 18677
chimp54546-135457.revcompl.Pan.troglodytes.clone.rp43-41g5.GenBank:AC099533.36  GCCAACGAGGATGCCGCCCAGGGCATCGCCAA---------------------------- 20190
human.FAM30B-LOC105372935-GGT2.NCBI.                                            gccagcgaggacgccgcccagggcatcgccaacgaggacaccatccagggcatcgccaag 16646
human.FAM230E-LOC105377182-GGT3P.NCBI.                                          gccaacgaggacgccgcccagggcatcgccaacgaggacgccgtccatggcatcgccaac 15716
human.FAM230J-LOC105372942-GGTLC5P.NCBI.rev.compl                               gccaacgaggacgccgcccagggcatcgccaaggaggacgccgcccacggcatcgccaac 16091
human.FAM230A.ncRNA.18487127.18500594-GGTLC3.18516335.18518165.rev.compl.REF    gccaacgaggacgccgcccagggcatcgccaacgaggacgccgtccagggcatcgccaac 5137
                                                                                **** ** **  ******** ** ********

chimp.LOC112206744-LOC107973052-GGT2.revcompl                                   ------------------------------------------------------------ 18677
chimp54546-135457.revcompl.Pan.troglodytes.clone.rp43-41g5.GenBank:AC099533.36  ------------------------------------------------------------ 20190
human.FAM30B-LOC105372935-GGT2.NCBI.                                            gagtacgccgtccacggcatcgccaacgaggacgccgcccagggcatcgccaacgaggac 16706
human.FAM230E-LOC105377182-GGT3P.NCBI.                                          gaggacgctgcccacggcattgctaacgaggatgccgtgcacggcatcgctaatgaggac 15776
human.FAM230J-LOC105372942-GGTLC5P.NCBI.rev.compl                               gaggatgctgcccacggcattgctaacgaggatgccgtgcacggcatcgctaatgaggac 16151
human.FAM230A.ncRNA.18487127.18500594-GGTLC3.18516335.18518165.rev.compl.REF    gaggacgccgcccacggcatcgccaacgaggacgctgcccacggcattgctaacgaggat 5197

chimp.LOC112206744-LOC107973052-GGT2.revcompl                                   ------------------------------------------------------------ 18677
chimp54546-135457.revcompl.Pan.troglodytes.clone.rp43-41g5.GenBank:AC099533.36  ------------------------------------------------------------ 20190
human.FAM30B-LOC105372935-GGT2.NCBI.                                            gccgcccagggcatcgccaacgaggacgccgcccagggcatcgccaaggaggacgccgcc 16766
human.FAM230E-LOC105377182-GGT3P.NCBI.                                          tccgtatacgacatcgctaatgaggatgccatatatgacatcgcta---atgacaccgta 15833
human.FAM230J-LOC105372942-GGTLC5P.NCBI.rev.compl                               tccgtatacgacatcgctaatgaggatgccatatatgacatcgcta---atgacaccgta 16208
human.FAM230A.ncRNA.18487127.18500594-GGTLC3.18516335.18518165.rev.compl.REF    gcc--------------------------------------------------------- 5200

chimp.LOC112206744-LOC107973052-GGT2.revcompl                                   ------------------------------------------------------------ 18677
chimp54546-135457.revcompl.Pan.troglodytes.clone.rp43-41g5.GenBank:AC099533.36  ------------------------------------------------------------ 20190
human.FAM30B-LOC105372935-GGT2.NCBI.                                            cagggcatcgccaacgagggcgccgcccagggcatcgccaaagaggacgccgcccagggc 16826
human.FAM230E-LOC105377182-GGT3P.NCBI.                                          caaggca-cgctaacgaggatgctgtacacgacatcgctaatgaggacaccatacaaggc 15892
human.FAM230J-LOC105372942-GGTLC5P.NCBI.rev.compl                               caaggca-cactaacgaggacgctgtacacgacatcgctaatgaggacaccatacaaggc 16267
human.FAM230A.ncRNA.18487127.18500594-GGTLC3.18516335.18518165.rev.compl.REF    ------------------------------------------------------------ 5200

chimp.LOC112206744-LOC107973052-GGT2.revcompl                                   ------------------------------------------------------------ 18677
chimp54546-135457.revcompl.Pan.troglodytes.clone.rp43-41g5.GenBank:AC099533.36  ------------------------------------------------------------ 20190
human.FAM30B-LOC105372935-GGT2.NCBI.                                            atcgctaacgaggccgccgacaagggcatcgccaacgaggatgccgcccacggcatcgcc 16886
human.FAM230E-LOC105377182-GGT3P.NCBI.                                          atcggtaatgaggacgttgtatatgacatcgctaacaaggacactctacaagccgtcgct 15952
human.FAM230J-LOC105372942-GGTLC5P.NCBI.rev.compl                               attggtaatgaggacgttgtatatgacatcgctaacaaggacactctacaagccgtcgct 16327
human.FAM230A.ncRNA.18487127.18500594-GGTLC3.18516335.18518165.rev.compl.REF    ------------------------------------------------------------ 5200

chimp.LOC112206744-LOC107973052-GGT2.revcompl                                   ------------------------------------------------------------ 18677
chimp54546-135457.revcompl.Pan.troglodytes.clone.rp43-41g5.GenBank:AC099533.36  ------------------------------------------------------------ 20190
human.FAM30B-LOC105372935-GGT2.NCBI.                                            aacgaggacgccgcccacggcatcgccagcga---------------------------- 16918
human.FAM230E-LOC105377182-GGT3P.NCBI.                                          aacaaggacactgtacacaacatcgctaatgac--------------------------- 15985
human.FAM230J-LOC105372942-GGTLC5P.NCBI.rev.compl                               aacaaggacactgtacacaacatcgctaatgacggcaccatacaagacatcaccaatgag 16387
human.FAM230A.ncRNA.18487127.18500594-GGTLC3.18516335.18518165.rev.compl.REF    ------------------------------------------------------------ 5200

chimp.LOC112206744-LOC107973052-GGT2.revcompl                                   ------------------------------------------------------------ 18677
chimp54546-135457.revcompl.Pan.troglodytes.clone.rp43-41g5.GenBank:AC099533.36  ------------------------------------------------------------ 20190
human.FAM30B-LOC105372935-GGT2.NCBI.                                            ------------------------------------------------------------ 16918
human.FAM230E-LOC105377182-GGT3P.NCBI.                                          ------------------------------------------------------------ 15985
human.FAM230J-LOC105372942-GGTLC5P.NCBI.rev.compl                               ggcgctttatacgacattgctaatggcaccgacaaggcacgctaacgtggacactgtaca 16447
human.FAM230A.ncRNA.18487127.18500594-GGTLC3.18516335.18518165.rev.compl.REF    -------------------------------------------------------gtgca 5205

chimp.LOC112206744-LOC107973052-GGT2.revcompl                                   ------------------------------------------------------------ 18677
chimp54546-135457.revcompl.Pan.troglodytes.clone.rp43-41g5.GenBank:AC099533.36  ------------------------------------------------------------ 20190
human.FAM30B-LOC105372935-GGT2.NCBI.                                            ------------------------------------------------------------ 16918
human.FAM230E-LOC105377182-GGT3P.NCBI.                                          ------------------------------------------------------------ 15985
human.FAM230J-LOC105372942-GGTLC5P.NCBI.rev.compl                               cgacattgctaatgaggactccgtatacgacatcgctaatgagggtgccgtatatgacat 16507
human.FAM230A.ncRNA.18487127.18500594-GGTLC3.18516335.18518165.rev.compl.REF    cggcatcgctaatgaggactccgtatacgacatcgctaatgaggatgccatatatgacat 5265

chimp.LOC112206744-LOC107973052-GGT2.revcompl                                   ------------------------------------------------------------ 18677
chimp54546-135457.revcompl.Pan.troglodytes.clone.rp43-41g5.GenBank:AC099533.36  ------------------------------------------------------------ 20190
human.FAM30B-LOC105372935-GGT2.NCBI.                                            ------------------------------------------------------------ 16918
human.FAM230E-LOC105377182-GGT3P.NCBI.                                          ------------------------------------------------------------ 15985
human.FAM230J-LOC105372942-GGTLC5P.NCBI.rev.compl                               cgctaatgacaccgtacaaggcacgctaacgaggacgctgtacacgacatcgctaatgag 16567
human.FAM230A.ncRNA.18487127.18500594-GGTLC3.18516335.18518165.rev.compl.REF    cgctaatgacaccgtacaaggcacgctaaagaggacgctgtacacgacatcgctaatgag 5325

chimp.LOC112206744-LOC107973052-GGT2.revcompl                                   ------------------------------------------------------------ 18677
chimp54546-135457.revcompl.Pan.troglodytes.clone.rp43-41g5.GenBank:AC099533.36  ------------------------------------------------------------ 20190
human.FAM30B-LOC105372935-GGT2.NCBI.                                            ------------------------------------------------------------ 16918
human.FAM230E-LOC105377182-GGT3P.NCBI.                                          ------------------------------------------------------------ 15985
human.FAM230J-LOC105372942-GGTLC5P.NCBI.rev.compl                               gacaccatacaaggcatcggtaatgaggacgctgtatacgacatcgctaacgaggacacc 16627
human.FAM230A.ncRNA.18487127.18500594-GGTLC3.18516335.18518165.rev.compl.REF    gacaccatacaaggcattggtaatgaggacgttgtatatgacatcgctaacaaggacact 5385

chimp.LOC112206744-LOC107973052-GGT2.revcompl                                   ------------------------------------------------------------ 18677
chimp54546-135457.revcompl.Pan.troglodytes.clone.rp43-41g5.GenBank:AC099533.36  ------------------------------------------------------------ 20190
human.FAM30B-LOC105372935-GGT2.NCBI.                                            -----------------------------------------------ggacgccgcccac 16931
human.FAM230E-LOC105377182-GGT3P.NCBI.                                          ------------------------------------------------ggcaccgtacaa 15997
human.FAM230J-LOC105372942-GGTLC5P.NCBI.rev.compl                               atacaagccgtcgctaacaaggacactgtacacaacatcgctaatgatggcaccgtacaa 16687
human.FAM230A.ncRNA.18487127.18500594-GGTLC3.18516335.18518165.rev.compl.REF    ctacaagccgtcgctaacaaggacactgtacacaacatcgctaatgacggcaccgtacaa 5445

chimp.LOC112206744-LOC107973052-GGT2.revcompl                                   ------------------------------------------------------------ 18677
chimp54546-135457.revcompl.Pan.troglodytes.clone.rp43-41g5.GenBank:AC099533.36  -----------CGTGATCGCCGTCCACGGCATCGCCAATGTGGTCGCCGTCCAGGGCATC 20239
human.FAM30B-LOC105372935-GGT2.NCBI.                                            ggcatcgccagcgaggacgccgcccacggcatcgccagcgaggacgccgcccagggcatc 16991
human.FAM230E-LOC105377182-GGT3P.NCBI.                                          gacatcaccaatgagggcgctttatacgacattgctaatgataccgac-----aaggcac 16052
human.FAM230J-LOC105372942-GGTLC5P.NCBI.rev.compl                               gacatcaccaatgagggcgctttatacgacattgctaatgataccgac-----aaggcac 16742
human.FAM230A.ncRNA.18487127.18500594-GGTLC3.18516335.18518165.rev.compl.REF    gacatcaccaatgagggcgctttatacgacattgctaatgataccgac-----aaggcac 5500

chimp.LOC112206744-LOC107973052-GGT2.revcompl                                   ------------------------------------------------------------ 18677
chimp54546-135457.revcompl.Pan.troglodytes.clone.rp43-41g5.GenBank:AC099533.36  GCCAACGAGGACGCCACCCAGGGCATCGCCAAGGAGGACGCCGTCCACGGCATCGCCAAC 20299
human.FAM30B-LOC105372935-GGT2.NCBI.                                            gccaacgaggacgccatccagggcatcgccaaggagtacgccgtccacggcatcgccaac 17051
human.FAM230E-LOC105377182-GGT3P.NCBI.                                          gctaacgcggacactgtacacgacatcgctaatgaggactccgtatacgacatcgctaat 16112
human.FAM230J-LOC105372942-GGTLC5P.NCBI.rev.compl                               gctatcgtggacgctgtacacgacatcgctaatgaggactccgtatacgacatcgctaat 16802
human.FAM230A.ncRNA.18487127.18500594-GGTLC3.18516335.18518165.rev.compl.REF    gctaacgcggacactgtacacgacatcgctaatgaggactccgtataccacatcgctaat 5560

chimp.LOC112206744-LOC107973052-GGT2.revcompl                                   ------------------------------------------------------------ 18677
chimp54546-135457.revcompl.Pan.troglodytes.clone.rp43-41g5.GenBank:AC099533.36  GTGGTCGCCGTCCAGGGCATCGCCAACGTGGTCGCCGTCCAGGGCATCACCAAGGAGGAC 20359
human.FAM30B-LOC105372935-GGT2.NCBI.                                            gaggacgccgcccagggcatcgccaacgaggacgccgcccagggcatcgccaacgaggac 17111
human.FAM230E-LOC105377182-GGT3P.NCBI.                                          gagggtgccgtatatgacatcgcta---atgacaccgtacaaggca-cgctaacgaggac 16168
human.FAM230J-LOC105372942-GGTLC5P.NCBI.rev.compl                               gagggtgccgtatatgacatcgcta---atgacaccgtacaaggca-cgctaacgaggac 16858
human.FAM230A.ncRNA.18487127.18500594-GGTLC3.18516335.18518165.rev.compl.REF    gagggtgccgtatatgacatcgcta---atgacaccgtacaaggca-cgctaacgaggac 5616

chimp.LOC112206744-LOC107973052-GGT2.revcompl                                   ------------------------------------------------------------ 18677
chimp54546-135457.revcompl.Pan.troglodytes.clone.rp43-41g5.GenBank:AC099533.36  GCCGTCCATGGCATCGCCAAGGAGGACGCCGCCCAGGGCATCGCCAACGTGGTCGCCGTC 20419
human.FAM30B-LOC105372935-GGT2.NCBI.                                            gccgcccagggcatcgccaaggaggacgccgcccagggcatcgccaaggaggacgccgcc 17171
human.FAM230E-LOC105377182-GGT3P.NCBI.                                          gctgtacacgacatcgctaatgaggacaccatacaaggcatcggtaatgaggac------ 16222
human.FAM230J-LOC105372942-GGTLC5P.NCBI.rev.compl                               gctgtacacgacatcgctaatgaggacaccatacaaggcatcggtaatgaggac------ 16912
human.FAM230A.ncRNA.18487127.18500594-GGTLC3.18516335.18518165.rev.compl.REF    gctgtacacgacatcgctaatgaggacaccatacaaggcatcggtaatgaggac------ 5670

chimp.LOC112206744-LOC107973052-GGT2.revcompl                                   ------------------------------------------------------------ 18677
chimp54546-135457.revcompl.Pan.troglodytes.clone.rp43-41g5.GenBank:AC099533.36  CAGGGCATCGCCAACGAGGACGCCGCCCAGGGCATCGCCAAGGAGGACGCCGTCCACGGC 20479
human.FAM30B-LOC105372935-GGT2.NCBI.                                            cagggcatcgccaacgagggcgccgcccagggcatcgccaaagaggacgccgcccacggc 17231
human.FAM230E-LOC105377182-GGT3P.NCBI.                                          ------------------------------------------------------------ 16222
human.FAM230J-LOC105372942-GGTLC5P.NCBI.rev.compl                               ------------------------------------------------------------ 16912
human.FAM230A.ncRNA.18487127.18500594-GGTLC3.18516335.18518165.rev.compl.REF    ------------------------------------------------------------ 5670

chimp.LOC112206744-LOC107973052-GGT2.revcompl                                   ------------------------------------------------------------ 18677
chimp54546-135457.revcompl.Pan.troglodytes.clone.rp43-41g5.GenBank:AC099533.36  ATCGCCAACGAGGACGCCGCCCAGGGCATCGCCAACGAGGACGCCGCCCAGGGCATCGC- 20538
human.FAM30B-LOC105372935-GGT2.NCBI.                                            atcgccaacgaggacgccgcccagggcatcgccaacgaggacgccgcccacggaatcgcc 17291
human.FAM230E-LOC105377182-GGT3P.NCBI.                                          ------------------------------------------------------------ 16222
human.FAM230J-LOC105372942-GGTLC5P.NCBI.rev.compl                               ------------------------------------------------------------ 16912
```

```
human.FAM230A.ncRNA.18487127.18500594-GGTLC3.18516335.18518165.rev.compl.REF      ------------------------------------------------------------ 5670

chimp.LOC112206744-LOC107973052-GGT2.revcompl                                     ------------------------------------------------------------ 18677
chimp54546-135457.revcompl.Pan.troglodytes.clone.rp43-41g5.GenBank:AC099533.36    ------------------------------------------------------------ 20538
human.FAM30B-LOC105372935-GGT2.NCBI.                                              agtgaggacgccgcccacggcatcgccagcgaggacgccgtccagggcatcgacaaggag 17351
human.FAM230E-LOC105377182-GGT3P.NCBI.                                            ------------------------------------------------------------ 16222
human.FAM230J-LOC105372942-GGTLC5P.NCBI.rev.compl                                 ------------------------------------------------------------ 16912
human.FAM230A.ncRNA.18487127.18500594-GGTLC3.18516335.18518165.rev.compl.REF      ------------------------------------------------------------ 5670

chimp.LOC112206744-LOC107973052-GGT2.revcompl                                     ------------------------------------------------------------ 18677
chimp54546-135457.revcompl.Pan.troglodytes.clone.rp43-41g5.GenBank:AC099533.36    ------------------------------------------------------------ 20538
human.FAM30B-LOC105372935-GGT2.NCBI.                                              gatgccgcccagggcatcgccaacgaggacgccgcccagggcatcgccaacgaggacgcc 17411
human.FAM230E-LOC105377182-GGT3P.NCBI.                                            ------------------------------------------------------------ 16222
human.FAM230J-LOC105372942-GGTLC5P.NCBI.rev.compl                                 ------------------------------------------------------------ 16912
human.FAM230A.ncRNA.18487127.18500594-GGTLC3.18516335.18518165.rev.compl.REF      ------------------------------------------------------------ 5670

chimp.LOC112206744-LOC107973052-GGT2.revcompl                                     --------catcgctaatgaggatgccatatatgacatcgctaatgacaccgtacaaggc 18729
chimp54546-135457.revcompl.Pan.troglodytes.clone.rp43-41g5.GenBank:AC099533.36    --------CATCGCTAATGAGGATGCCATATATGACATCGCTAATGACACCGTACAAGGC 20590
human.FAM30B-LOC105372935-GGT2.NCBI.                                              gcccagggcatcgccaaagaggacgccgcccacggcatcgccaacgagctgtatacgaca 17471
human.FAM230E-LOC105377182-GGT3P.NCBI.                                            -----------------------------------------------gctgtatacgaca 16235
human.FAM230J-LOC105372942-GGTLC5P.NCBI.rev.compl                                 -----------------------------------------------gctgtatacgaca 16925
human.FAM230A.ncRNA.18487127.18500594-GGTLC3.18516335.18518165.rev.compl.REF      -----------------------------------------------gctgtatacgaca 5683

chimp.LOC112206744-LOC107973052-GGT2.revcompl                                     atgctaacgaagacatcgtacaagccgtcgctaacaagtacactgtacacaacatcgcta 18789
chimp54546-135457.revcompl.Pan.troglodytes.clone.rp43-41g5.GenBank:AC099533.36    ATGCTAACGAGGACATCGTACAAGCCGTCGCTAACAAGTACACTGTACACAACATCGCTA 20650
human.FAM30B-LOC105372935-GGT2.NCBI.                                              tcgctaacgaggacaccctacaagccgtcgctaacaagtacactgtacacaacatcgcta 17531
human.FAM230E-LOC105377182-GGT3P.NCBI.                                            tcgctaacgaggacaccatacaagccgtcgctaacaaggacactgtacacaacatcgcta 16295
human.FAM230J-LOC105372942-GGTLC5P.NCBI.rev.compl                                 tcgctaacgaggacaccatacaagccgtcactaacaaggacactgtacacaacatcgcta 16985
human.FAM230A.ncRNA.18487127.18500594-GGTLC3.18516335.18518165.rev.compl.REF      tcgctaacgaggacaccatacaagccgtcgctaacaaggacactgtacacaacatcgcta 5743
                                                                                    ******** **** * *********** ******** *********************

chimp.LOC112206744-LOC107973052-GGT2.revcompl                                     atgagggcactgtacaagacatcaccaatgaggacgctttatacgacattgctaatggca 18849
chimp54546-135457.revcompl.Pan.troglodytes.clone.rp43-41g5.GenBank:AC099533.36    ATGAGGGCACTGTACAAGACATCACCAATGAGGACGCTTTATACGACATTGCTAATGGCA 20710
human.FAM30B-LOC105372935-GGT2.NCBI.                                              atgagggcactgtacaagacatcaccaatgagggcgctttatacgacattgctaatggca 17591
human.FAM230E-LOC105377182-GGT3P.NCBI.                                            atgacggcaccgtacaagacatcaccaatgagggcgctttatacgacattgctaatgata 16355
human.FAM230J-LOC105372942-GGTLC5P.NCBI.rev.compl                                 atgacggcaccgtacaagacatcaccaatgagggcgctttatacgacattgctaatgata 17045
human.FAM230A.ncRNA.18487127.18500594-GGTLC3.18516335.18518165.rev.compl.REF      atgacggcaccgtacaagacatcaccaatgagggcgctttatacgacattgctaatgata 5803
                                                                                  **** ***** ********************** ***********************  *

chimp.LOC112206744-LOC107973052-GGT2.revcompl                                     ccgacaaggcacgctaacgtggatgctgtacacgacattgctaatgaggacaccgtataa 18909
chimp54546-135457.revcompl.Pan.troglodytes.clone.rp43-41g5.GenBank:AC099533.36    CCGACAAGGCACGCTAACGTGGATGCTGTACACGACATTGCTAATGAGGACACCGTATAA 20770
human.FAM30B-LOC105372935-GGT2.NCBI.                                              ccgacaaggcacgctaacgtggacgctgtacacgacattgctaatgaggacagcgtataa 17651
human.FAM230E-LOC105377182-GGT3P.NCBI.                                            ccgacaaggcacgctaacgtggacgctgtacacgacattgctaatgaggacaccgtataa 16415
human.FAM230J-LOC105372942-GGTLC5P.NCBI.rev.compl                                 ccgacaaggcacgctaacgtggacgctgtacacgacattgctaatgaggacaccgtataa 17105
human.FAM230A.ncRNA.18487127.18500594-GGTLC3.18516335.18518165.rev.compl.REF      ccgacaaggcacgctaacgtggacgctgtacacgacattgctaatgaggacaccgtataa 5863
                                                                                  ********************** ***************************** *******

chimp.LOC112206744-LOC107973052-GGT2.revcompl                                     gacatcgctagtaactatcgcaagaacaaaaaaccaaacaccgcatattctcactcatag 18969
chimp54546-135457.revcompl.Pan.troglodytes.clone.rp43-41g5.GenBank:AC099533.36    GACATCGCTAGTAACTATCGCAAGAACAAAAAACCAAACACCGCATATTCTCACTCATAG 20830
human.FAM30B-LOC105372935-GGT2.NCBI.                                              gacatcgctagtaactatcgcaagaacaaaaaaccaaacaccgcatattctcactcatag 17711
human.FAM230E-LOC105377182-GGT3P.NCBI.                                            gacatcgctagtaactatcgcaagaacaaaaaaccaaacaccgcatattctcactcatag 16475
human.FAM230J-LOC105372942-GGTLC5P.NCBI.rev.compl                                 gacatcgctagtaactatcgcaagaacaaaaaaccaaacaccgcatattctcactcatag 17165
human.FAM230A.ncRNA.18487127.18500594-GGTLC3.18516335.18518165.rev.compl.REF      gacatcgctagtaactatcgcaagaacaaaaaaccaaacaccgcatattctcactcatag 5923
                                                                                  ************************************************************

chimp.LOC112206744-LOC107973052-GGT2.revcompl                                     gtgggaattgaacaatgagatcacatggacacaggaaggggaatatcacactctggggac 19029
chimp54546-135457.revcompl.Pan.troglodytes.clone.rp43-41g5.GenBank:AC099533.36    GTGGGAATTGAACAATGAGATCACATGGACACAGGAAGGGGAATATCACACTCTGGGGAC 20890
human.FAM30B-LOC105372935-GGT2.NCBI.                                              atgggaattgaacaatgagatcacatggacacaggaaggggaatatcacactctggggac 17771
human.FAM230E-LOC105377182-GGT3P.NCBI.                                            gtgggaattgaacaatgagatcacatggacacaggaaggggaatatcacattctggggac 16535
human.FAM230J-LOC105372942-GGTLC5P.NCBI.rev.compl                                 gtgggaattgaacaatgagatcacatggacacaggaaggggaatatcacactctggggac 17225
human.FAM230A.ncRNA.18487127.18500594-GGTLC3.18516335.18518165.rev.compl.REF      gtgggaattgaacaatgagatcacatggacacaggaaggggaatatcacattctggggac 5983
                                                                                   ************************************************* *********

chimp.LOC112206744-LOC107973052-GGT2.revcompl                                     tgttgtggggtgggggggagggggggagggatagcatcgggagatatacctaatgctagatg 19089
chimp54546-135457.revcompl.Pan.troglodytes.clone.rp43-41g5.GenBank:AC099533.36    TGTTGTGG---TGGCGGGGGGGGAAGGGATAGCATCGGGAGATATACCTAATGCTAGATG 20947
human.FAM30B-LOC105372935-GGT2.NCBI.                                              tgttgtggggttgg----gggggagggatagcatcgggagatatacctaatgctagatg 17827
human.FAM230E-LOC105377182-GGT3P.NCBI.                                            tgttgtggggtgggggggagggggggagggatagcatcgggagatatacctaatgctagatg 16595
human.FAM230J-LOC105372942-GGTLC5P.NCBI.rev.compl                                 tcttgtggggtggg----gggggagggatagcatcgggagatatacctaatgctagatg 17281
human.FAM230A.ncRNA.18487127.18500594-GGTLC3.18516335.18518165.rev.compl.REF      tgttgtggggtgggggggagggggggagggatagcatcgggagatatacctaatgctagatg 6043
                                                                                  * ******    **    ***** ************************************

chimp.LOC112206744-LOC107973052-GGT2.revcompl                                     atgagttagtgggtgcagcgcaccagcatggcacatgtatacatatgtaactaacctgca 19149
chimp54546-135457.revcompl.Pan.troglodytes.clone.rp43-41g5.GenBank:AC099533.36    ATGAGTTAGTGGGTGCAGCGCACCAGCATGGCACATGTATACATATGTAACTAACCTGCA 21007
human.FAM30B-LOC105372935-GGT2.NCBI.                                              atgagttagtgggtgcagcgcaccagcacggcacatgtatacatatgtaactaacctgca 17887
human.FAM230E-LOC105377182-GGT3P.NCBI.                                            atgagttagtgggtgcagcgcaccagcgtggcacatgtatacatatgtaactaacctgca 16655
human.FAM230J-LOC105372942-GGTLC5P.NCBI.rev.compl                                 atgagttagtgggtgcagcgcaccagcgtggcacatgtatacatatgtaactaacctgca 17341
human.FAM230A.ncRNA.18487127.18500594-GGTLC3.18516335.18518165.rev.compl.REF      atgagttagtgggtgcagcgcaccagcgtggcacatgtatacatatgtaactaacctgca 6103
                                                                                  ***************************  *******************************

chimp.LOC112206744-LOC107973052-GGT2.revcompl                                     caatgtgcacatgtaccctaaaacttaaagtatatatataaaaaaaaagacatcgctagt 19209
chimp54546-135457.revcompl.Pan.troglodytes.clone.rp43-41g5.GenBank:AC099533.36    CAATGTGCACATGTACCCTAAAACTTAAAG--TATATATAAAAAAAAAGACATCGCTAGT 21065
human.FAM30B-LOC105372935-GGT2.NCBI.                                              caatgtgcacatgtaccctaaaacttaaagtatatatataaaaaaaaagacatcgctagt 17947
human.FAM230E-LOC105377182-GGT3P.NCBI.                                            caatgtgcacatgtaccctaaaacttaaagtatatat--aaaaaaaaagacatcgctagt 16713
human.FAM230J-LOC105372942-GGTLC5P.NCBI.rev.compl                                 caatgtgcacatgtaccctaaaacttaaagtatatataaaaaaaaaaagacatcgctagt 17401
human.FAM230A.ncRNA.18487127.18500594-GGTLC3.18516335.18518165.rev.compl.REF      caatgtgcacatgtaccctaaaacttaaagtatatat-aaaaaaaaaagacatcgctagt 6162
                                                                                  ******************************  *****   ********************

chimp.LOC112206744-LOC107973052-GGT2.revcompl                                     gagcacgctgtatacgacatcactaatgaggatgctatatatgacatcgctgatgaggac 19269
chimp54546-135457.revcompl.Pan.troglodytes.clone.rp43-41g5.GenBank:AC099533.36    GAGCACGCTGTATACGACATCGCTAATGAGGACGCTATATATGACATCGCTGATGAGGAC 21125
human.FAM30B-LOC105372935-GGT2.NCBI.                                              gagcacgctgtatacgacatcg-------------------------------------- 17969
human.FAM230E-LOC105377182-GGT3P.NCBI.                                            gagcacgctgtatacgacatcg-------------------------------------- 16735
human.FAM230J-LOC105372942-GGTLC5P.NCBI.rev.compl                                 gagcacgctgtatacgacatcg-------------------------------------- 17423
human.FAM230A.ncRNA.18487127.18500594-GGTLC3.18516335.18518165.rev.compl.REF      gagcacgctgtatacgacatcg-------------------------------------- 6184
                                                                                  ********************

chimp.LOC112206744-LOC107973052-GGT2.revcompl                                     attgtatacgacatcactaatgaggacaccatacaaggcatcgctaacaatgatgctgta 19329
chimp54546-135457.revcompl.Pan.troglodytes.clone.rp43-41g5.GenBank:AC099533.36    ATTGTATACGACATCACTAATGAGGACACCATACAAGGCATCGCTAACGATGACGCTGTA 21185
human.FAM30B-LOC105372935-GGT2.NCBI.                                              ----------------ctaatgaggacaccatacaaggcatcgctaacgatgacgctgta 18013
human.FAM230E-LOC105377182-GGT3P.NCBI.                                            ----------------ctaatgaggacaccatacaaggcatcgctaacgatgacgctgta 16779
human.FAM230J-LOC105372942-GGTLC5P.NCBI.rev.compl                                 ----------------ctaatgaggacaccatacaaggcatcgctaacgatgacgctgta 17467
human.FAM230A.ncRNA.18487127.18500594-GGTLC3.18516335.18518165.rev.compl.REF      ----------------ctaatgaggacaccatacaaggcatcgctaacgatgacgctgta 6228
                                                                                                  ******************************** **** ******

chimp.LOC112206744-LOC107973052-GGT2.revcompl                                     cacaacatcactaatgatgacaccgtataacacatcgctaattatgacgctgtatacgac 19389
chimp54546-135457.revcompl.Pan.troglodytes.clone.rp43-41g5.GenBank:AC099533.36    CACAACATCGCTAATGATGACACCGTATAAGACATCGCTAATTATGACGCTGTATACGAC 21245
human.FAM30B-LOC105372935-GGT2.NCBI.                                              cacaacatcactaatgatgacaccgtataagacatcgctaattatgacgctgtatacgac 18073
human.FAM230E-LOC105377182-GGT3P.NCBI.                                            cacaacatcactaatgatgacaccgtataagacatcgctaattatgacgctgtatacgac 16839
human.FAM230J-LOC105372942-GGTLC5P.NCBI.rev.compl                                 cacaacatcactaatgatgacaccgtataagacatcgctaattatgacgctgtatacgac 17527
human.FAM230A.ncRNA.18487127.18500594-GGTLC3.18516335.18518165.rev.compl.REF      cacaacatcactaatgatgacaccgtataagacatcgctaattatgacgctgtatacgac 6288
                                                                                  ********* ******************** *****************************

chimp.LOC112206744-LOC107973052-GGT2.revcompl                                     atcgctaatgacaccgtacaaggcacactaatgaggatgctgtacacgacatcactaatg 19449
chimp54546-135457.revcompl.Pan.troglodytes.clone.rp43-41g5.GenBank:AC099533.36    ATCGCTAATGACACCGTACAAGGCACGCTAACGAGGATGCTGTACACGACATCACTAATG 21305
human.FAM30B-LOC105372935-GGT2.NCBI.                                              atcgctaatgacaccgtacaaggcacgctaacgaggatgctgtacacgacatcactaatg 18133
human.FAM230E-LOC105377182-GGT3P.NCBI.                                            atcgctaatgacaccgtacgaggcacgctaacaaggatgctgtacacaacgtcgctaatg 16899
human.FAM230J-LOC105372942-GGTLC5P.NCBI.rev.compl                                 atcgctaatgacaccgtacgaggcacactaacaaggatgctgtacacaacatcgctaatg 17587
human.FAM230A.ncRNA.18487127.18500594-GGTLC3.18516335.18518165.rev.compl.REF      atcgctaatgacaccgtacgaggcacgctaacaaggatgctgtacacaacatcgctaatg 6348
                                                                                  ******************* ****** ****  ************** ** ** ******

chimp.LOC112206744-LOC107973052-GGT2.revcompl                                     aggacagtgtacaagccatcgctaatgaggacactgtatatgacatcgctaacgaggaca 19509
chimp54546-135457.revcompl.Pan.troglodytes.clone.rp43-41g5.GenBank:AC099533.36    AGGACAGTGTACAAGCCATCGCTAATGAGGACACTGTATATGACATCGCTAACGAGGACA 21365
human.FAM30B-LOC105372935-GGT2.NCBI.                                              aggacagtgtacaagccatcactaatgaggacactgtatatggcatcgctaacgaggaca 18193
human.FAM230E-LOC105377182-GGT3P.NCBI.                                            aggacagtgtacaagccatcgctaatgaggacactgtatatgacattgctaacgaggaca 16959
human.FAM230J-LOC105372942-GGTLC5P.NCBI.rev.compl                                 aggacagtgtataagccatcgctaatgaggacactgtatatgacattgctaacgaggaca 17647
human.FAM230A.ncRNA.18487127.18500594-GGTLC3.18516335.18518165.rev.compl.REF      aggacagtgtacaagccatcgctaatgaggacactgtatatgacattgctaacgaggaca 6408
                                                                                  *********** ******** ********************* *** *************

chimp.LOC112206744-LOC107973052-GGT2.revcompl                                     ctgtacaaggcattgctaacgaggacgctgtac---acatcgctaatgaggacaccatat 19566
chimp54546-135457.revcompl.Pan.troglodytes.clone.rp43-41g5.GenBank:AC099533.36    CTGTACAAGGCATTGCTAACGAGGACGCTGTACACAACACCGCTAATGAGGACACCATAT 21425
human.FAM30B-LOC105372935-GGT2.NCBI.                                              ctgtacaagccattgctaacgaggacgctgtacacaacatcgctaatgaggacaccatat 18253
human.FAM230E-LOC105377182-GGT3P.NCBI.                                            ctgtacaaggcattgctaacgaggacgctgtacacaacatcgctaatgaggacaccatat 17019
human.FAM230J-LOC105372942-GGTLC5P.NCBI.rev.compl                                 ctgtacaaggcattgctaacgaggacgctgtacacaacatcgctaat---gacaccatat 17704
human.FAM230A.ncRNA.18487127.18500594-GGTLC3.18516335.18518165.rev.compl.REF      ctgtacaaggcattgctaacgaggacgctgtacacaacatcgctaatgaggacaccatat 6468
                                                                                  ********* ***********************   *** *******   **********

chimp.LOC112206744-LOC107973052-GGT2.revcompl                                     aagacatcaccaatgaggatgctgtatatgacatcactaatgacacccacaaggcatgct 19626
chimp54546-135457.revcompl.Pan.troglodytes.clone.rp43-41g5.GenBank:AC099533.36    AAGACATCACCAATGAGGATGCTGTATATGACATCGCTAATGACACCCACAAGGCATGCT 21485
human.FAM30B-LOC105372935-GGT2.NCBI.                                              aagacatcaccaatgaggatgctgtatatgacatcgctaatggcacccacaaggcatgct 18313
human.FAM230E-LOC105377182-GGT3P.NCBI.                                            aagacatcaccaatgaggatgctgtatatgacatcgctaatgacacccacaaggcatgct 17079
human.FAM230J-LOC105372942-GGTLC5P.NCBI.rev.compl                                 aagacatcaccaatgaggatgctgtatatgacatcgctaatgacacccacaaggcatgct 17764
human.FAM230A.ncRNA.18487127.18500594-GGTLC3.18516335.18518165.rev.compl.REF      aagacatcaccaatgaggatgctctatatgacatcgctaatgacacccacaaggcatgct 6528
```

```
                                                                                ********************** *********** ****** *****************

chimp.LOC112206744-LOC107973052-GGT2.revcompl                                   aacgaggacgctgtagacgacattgcttataaggacaccgtacaagacatcgctaacgag 19686
chimp54546-135457.revcompl.Pan.troglodytes.clone.rp43-41g5.GenBank:AC099533.36  AACGAGGACGCTGTAGACGACATTGCTTATAAGGACACCGTACAAGACATCGCTAACCAG 21545
human.FAM30B-LOC105372935-GGT2.NCBI.                                            aacgaggacgctgtagacgacattgcttataaggacaccgtacaagacatcgctaacgag 18373
human.FAM230E-LOC105377182-GGT3P.NCBI.                                          aacgaggacgctgtagacgacattgcttataaggacaccgtacaagacatcactaacgag 17139
human.FAM230J-LOC105372942-GGTLC5P.NCBI.rev.compl                               aacgaggacgctgtagacgacattgcttataaggacaccgtacaagacatcactaacgag 17824
human.FAM230A.ncRNA.18487127.18500594-GGTLC3.18516335.18518165.rev.compl.REF    aacgaggacgctgtagacgacattgcttataaggacaccgtacaagacatcgctaacgag 6588
                                                                                *************************************************** ***** **

chimp.LOC112206744-LOC107973052-GGT2.revcompl                                   gacgctgtatacgacatcgctaatgaggacgttgtatatgacatcgctaatgaggatgct 19746
chimp54546-135457.revcompl.Pan.troglodytes.clone.rp43-41g5.GenBank:AC099533.36  GACGCTGTATAC------------------------------------------------ 21557
human.FAM30B-LOC105372935-GGT2.NCBI.                                            gacgctgtatacgacatcgctaatgaggacgttgtatatgacatcgctaatgaggatgct 18433
human.FAM230E-LOC105377182-GGT3P.NCBI.                                          gacgctgtatacgacatcgctaatgaggacgttgtatatgacatcgctaatgaggatgct 17199
human.FAM230J-LOC105372942-GGTLC5P.NCBI.rev.compl                               gacgctgtatacgacatcgctaatgaggacgttgtatatgacatcgctaatgaggatgct 17884
human.FAM230A.ncRNA.18487127.18500594-GGTLC3.18516335.18518165.rev.compl.REF    gacgctgtatacgacatcgctaatgaggacgttgtatatgacatcgctaatgaggatgct 6648
                                                                                ************

chimp.LOC112206744-LOC107973052-GGT2.revcompl                                   ttacaagacatagctaatgaggttgctgtatatgatatcgctaatgaggacattgtatat 19806
chimp54546-135457.revcompl.Pan.troglodytes.clone.rp43-41g5.GenBank:AC099533.36  ---------------------------------GACATCGCTAATGAGGACACTGTATAT 21584
human.FAM30B-LOC105372935-GGT2.NCBI.                                            ttacaagacatagctaatgaggttgctgtatatgacatcgctaatgaggacattgtatat 18493
human.FAM230E-LOC105377182-GGT3P.NCBI.                                          ttacaagacatagcgaatgaggttgctgtatatgacatcgctaatgaggacattgtatat 17259
human.FAM230J-LOC105372942-GGTLC5P.NCBI.rev.compl                               ttacaagacatagcgaatgaggttgctgtatatgacatcgctaatgaggacattgtatat 17944
human.FAM230A.ncRNA.18487127.18500594-GGTLC3.18516335.18518165.rev.compl.REF    ttacaagacatagcgaatgaggttgctgtatatgacatcgctaatgaggacattgtatat 6708
                                                                                                                 ** **************** *******

chimp.LOC112206744-LOC107973052-GGT2.revcompl                                   gacatcgctaatgaggacgctctatacgacatcactaatgaggatgctgtatacaacatc 19866
chimp54546-135457.revcompl.Pan.troglodytes.clone.rp43-41g5.GenBank:AC099533.36  GACATCGCTAATGAGGACGCTCTATACGACATCACTAACGAGGACGCTGTATACAACATT 21644
human.FAM30B-LOC105372935-GGT2.NCBI.                                            gacatcgctaatgaggacgctctatacgacatcactaatgaggacgctgtatacaacatc 18553
human.FAM230E-LOC105377182-GGT3P.NCBI.                                          gacatcgctaatgaggacgctctatacgacatcactaatgaggacgctgtatacaacatc 17319
human.FAM230J-LOC105372942-GGTLC5P.NCBI.rev.compl                               gacatcgctaatgaggacgctctatacgacatcactaatgaggacgctgtatacaacatc 18004
human.FAM230A.ncRNA.18487127.18500594-GGTLC3.18516335.18518165.rev.compl.REF    gacatcgctaatgaggacgctctatacgacatcactaatgaggacgctgtatacaacatc 6768
                                                                                ************************************** ***** **************

chimp.LOC112206744-LOC107973052-GGT2.revcompl                                   gctaatgaggacgctgtatatggcatcgctaatgaggatgctgtatacgaattcgctaat 19926
chimp54546-135457.revcompl.Pan.troglodytes.clone.rp43-41g5.GenBank:AC099533.36  GCTAATGAGGACGCTGTATATGGCATCGCTAATGAGGATGCTGTATACAAATTCGCTAAT 21704
human.FAM30B-LOC105372935-GGT2.NCBI.                                            gctaatgaggacgctgtatatggcatcgctaatgaggatgctgtatacgaattcgctaat 18613
human.FAM230E-LOC105377182-GGT3P.NCBI.                                          gctaatgaggacgctgtatatggcatcgctaatgaggatgctgtatacgaattcgctaat 17379
human.FAM230J-LOC105372942-GGTLC5P.NCBI.rev.compl                               gctaatgaggacgctgtatatggcatcgctaatgaggatgctgtatacgaattcgctaat 18064
human.FAM230A.ncRNA.18487127.18500594-GGTLC3.18516335.18518165.rev.compl.REF    gctaatgaggacgctgtatatggcatcgctaatgaggatgctgtatacgaattcgctaat 6828
                                                                                ************************************************ ***********

chimp.LOC112206744-LOC107973052-GGT2.revcompl                                   aaggacgctgtatatgacattgctaatgaggacactatacaagacatctgtaaaaaagaa 19986
chimp54546-135457.revcompl.Pan.troglodytes.clone.rp43-41g5.GenBank:AC099533.36  AAGGACGCTGTATATGACATTGCTAATGAGGACACTGTACAAGACATCTGTAAAAAAGAA 21764
human.FAM30B-LOC105372935-GGT2.NCBI.                                            aaggacgctgtatatgacattgctaatgaggacactgtacaagacatctgtaaaaaagaa 18673
human.FAM230E-LOC105377182-GGT3P.NCBI.                                          aaggacgctgtatatgacattgctaatgaggacactgtacaagacatctgtaaaaaagaa 17439
human.FAM230J-LOC105372942-GGTLC5P.NCBI.rev.compl                               aaggacgctgtatatgacattgctaatgaggacactgtacaagacatctgtaaaaaagaa 18124
human.FAM230A.ncRNA.18487127.18500594-GGTLC3.18516335.18518165.rev.compl.REF    aaggacgctgtatatgacattgctaatgaggacactgtacaagacatctgtaaaaaagaa 6888
                                                                                *********************************** ************************

chimp.LOC112206744-LOC107973052-GGT2.revcompl                                   gatgctgccaatgtaagacacttttctttgtcttgaacagaaatgttactttcctggctt 20046
chimp54546-135457.revcompl.Pan.troglodytes.clone.rp43-41g5.GenBank:AC099533.36  GATGTTGCCAATGTAAGACACTTTTCTTTGTCTTGAACAGAAATGTTACTTTCCTGGCTT 21824
human.FAM30B-LOC105372935-GGT2.NCBI.                                            gatgctgccaatgtaagacacttttctttgtcttgaacagaaatgttactttcctggctt 18733
human.FAM230E-LOC105377182-GGT3P.NCBI.                                          gatgctgccaatgtaagacacttttctttgtcttgaacagaaatgttactttcctggctt 17499
human.FAM230J-LOC105372942-GGTLC5P.NCBI.rev.compl                               gatgctgccaatgtaagacacttttctttgtcttgaacagaaatgttactttcctggctt 18184
human.FAM230A.ncRNA.18487127.18500594-GGTLC3.18516335.18518165.rev.compl.REF    gatgctgccaatgtaagacacttttctttgtcttgaacagaaatgttactttcctggctt 6948
                                                                                **** *******************************************************

chimp.LOC112206744-LOC107973052-GGT2.revcompl                                   ctttccaatcagatgtagacatgaacatctgccagtgtgcattattgatgtcatctgcag 20106
chimp54546-135457.revcompl.Pan.troglodytes.clone.rp43-41g5.GenBank:AC099533.36  CTTTCCAATCAGACGTAGACATGAACATCTGCCAGTGTGAATTATCGATGTCATCTGCAG 21884
human.FAM30B-LOC105372935-GGT2.NCBI.                                            ctttccaatcagatgtagacatgaacatctgccagtgtgcattatcgatgtcatctgcag 18793
human.FAM230E-LOC105377182-GGT3P.NCBI.                                          ctttccaatcagatgtagacatgaacatctgccagtgtgcattatcgatgtcatctgcag 17559
human.FAM230J-LOC105372942-GGTLC5P.NCBI.rev.compl                               ctttccaatcagatgtagacatgaacatctgccagtgtgcattatggatgtcatctgcag 18244
human.FAM230A.ncRNA.18487127.18500594-GGTLC3.18516335.18518165.rev.compl.REF    ctttccaatcagatgtagacatgaacatctgccagtgtgcattatcgatgtcatctgcag 7008
                                                                                ************* ************************* ***** **************

chimp.LOC112206744-LOC107973052-GGT2.revcompl                                   tttaatcaaatgtagacatgaacatctgccaatgtggactatttatgacatctgcaattc 20166
chimp54546-135457.revcompl.Pan.troglodytes.clone.rp43-41g5.GenBank:AC099533.36  TTTAATCAAATGTAGACATGAACATCTGCCAATGTGGACTATTTATGACATCTGCAATTC 21944
human.FAM30B-LOC105372935-GGT2.NCBI.                                            tttaatcaaatgtagacatgaacatctgccaatgtggactatttatgacatctgcaattc 18853
human.FAM230E-LOC105377182-GGT3P.NCBI.                                          tttaatcaaatgtagacatgaacatctgccaatgtggactatttatgacatctgcaattc 17619
human.FAM230J-LOC105372942-GGTLC5P.NCBI.rev.compl                               tttaatcaaatgtagacatgaacatctgccaatgtggactatttatgacatctgcaattc 18304
human.FAM230A.ncRNA.18487127.18500594-GGTLC3.18516335.18518165.rev.compl.REF    tttaatcaaatgtagacatgaacatctgccaatgtggactatttatgacatctgcaattc 7068
                                                                                ************************************************************

chimp.LOC112206744-LOC107973052-GGT2.revcompl                                   ccttggtgtggtgctattgattggcagcctctcaccaacccatgccaggcacactggggt 20226
chimp54546-135457.revcompl.Pan.troglodytes.clone.rp43-41g5.GenBank:AC099533.36  CCTTGGTGTGGTGCTATTGATTGGCAGCCTCTCACCAACCCATGCCAGGCACACTGGGGT 22004
human.FAM30B-LOC105372935-GGT2.NCBI.                                            ccttggtgtggtgctattgattggcagcctctcaccaacccatgccaggcacactggggt 18913
human.FAM230E-LOC105377182-GGT3P.NCBI.                                          ccttggtgtggtgctattgattggcggcctctcaccaacccatgccaggcacactggggt 17679
human.FAM230J-LOC105372942-GGTLC5P.NCBI.rev.compl                               ccttggtgtggtgctattgattggcagcctctcaccaacccatgccaggcacactggggt 18364
human.FAM230A.ncRNA.18487127.18500594-GGTLC3.18516335.18518165.rev.compl.REF    ccttggtgtggtgctattgattggcggcctctcaccaacccatgccaggcacactggggt 7128
                                                                                ************************* **********************************

chimp.LOC112206744-LOC107973052-GGT2.revcompl                                   gtggtagatggcagcatccacgatccactgcaatgcagaggtgtttccctccacagcagt 20286
chimp54546-135457.revcompl.Pan.troglodytes.clone.rp43-41g5.GenBank:AC099533.36  GTGGTAGATGGCAGCATCCACGATCCACTGCAATGCAGAGGTGTTTCCCTCCACAGCAGT 22064
human.FAM30B-LOC105372935-GGT2.NCBI.                                            gtggtagatggcagcatccacgatccactgcaatgcagaggtgtttccctccacagcagt 18973
human.FAM230E-LOC105377182-GGT3P.NCBI.                                          gtggtagatggcagcatccacgatccactgcaatgcagaggtgtttccctccacagcagt 17739
human.FAM230J-LOC105372942-GGTLC5P.NCBI.rev.compl                               gtggtagatggcagcatccacgatccactgcaatgcagaggtgtttccctccacagcagt 18424
human.FAM230A.ncRNA.18487127.18500594-GGTLC3.18516335.18518165.rev.compl.REF    gtggtagatggcagcatccacgatccactgcaatgcagaggtgtttccctccacagcagt 7188
                                                                                ************************************************************

chimp.LOC112206744-LOC107973052-GGT2.revcompl                                   tttccccatggattaagagttgtgaaactgccaatctagatacactttaaagataaatt 20346
chimp54546-135457.revcompl.Pan.troglodytes.clone.rp43-41g5.GenBank:AC099533.36  TTTCCCCCATGGATTAAGAGTTGTGAAACTGCCAATCTAGATACACTTTAAAGATAAATT 22124
human.FAM30B-LOC105372935-GGT2.NCBI.                                            tttccccatggattaagagttgtgaaactgccaatctagatacactttaaagataaatt 19033
human.FAM230E-LOC105377182-GGT3P.NCBI.                                          tttccccatggattaagagttgtgaaactgccaatctagatacactttaaagataaatt 17799
human.FAM230J-LOC105372942-GGTLC5P.NCBI.rev.compl                               tttccccatggattaagagttgtgaaactgccaatctagatacactttaaagataaatt 18484
human.FAM230A.ncRNA.18487127.18500594-GGTLC3.18516335.18518165.rev.compl.REF    tttccccatggattaagagttgtgaaactgccaatctagatacactttaaagataaatt 7248
                                                                                ************************************************************

chimp.LOC112206744-LOC107973052-GGT2.revcompl                                   ctgtgggaaaaggtcttgtcttttccacaggtgtcttccgtgccagttttggggacttc 20406
chimp54546-135457.revcompl.Pan.troglodytes.clone.rp43-41g5.GenBank:AC099533.36  CTGTGGGAAAAGGTCTTGTCTTTTCCACAGGTGTCTTCCGTGCCAGTTTTGGGGACTTC 22184
human.FAM30B-LOC105372935-GGT2.NCBI.                                            ctgtgggaaaaggtcttgtcttttccacaggtgtcttccgtgccagttttggggacttc 19093
human.FAM230E-LOC105377182-GGT3P.NCBI.                                          ctgtgggaaaaggtcttgtcttttccacaggtgtcttccgtgccagttttggggga---- 17855
human.FAM230J-LOC105372942-GGTLC5P.NCBI.rev.compl                               ctgtgggaaaaggtcttgtcttttccacaggtgtcttccgtgccagttttggggacttt 18544
human.FAM230A.ncRNA.18487127.18500594-GGTLC3.18516335.18518165.rev.compl.REF    ctgtgggaaaaggtcttgtcttttccacaggtgtcttccgtgccagttttggggacttt 7308
                                                                                ********************************************************

chimp.LOC112206744-LOC107973052-GGT2.revcompl                                   gacctttgactcaatcactataccccttcttatttctctctcaagttgtcgagagacta 20466
chimp54546-135457.revcompl.Pan.troglodytes.clone.rp43-41g5.GenBank:AC099533.36  GACCTTTGACTCAATCACTATACCCCTTCTTATTTCTCTCTCAAGTTATCGAGAGACTA 22244
human.FAM30B-LOC105372935-GGT2.NCBI.                                            gacctttgactcaatcactataccccttcctatttctctctcaagttgtcgagagacta 19153
human.FAM230E-LOC105377182-GGT3P.NCBI.                                          -------------------------cttcatatttctctctcaagttgtcgagagacta 17890
human.FAM230J-LOC105372942-GGTLC5P.NCBI.rev.compl                               gacctttgactcaatcactataccccttcttatttctctctcaagttgtcgagagacta 18604
human.FAM230A.ncRNA.18487127.18500594-GGTLC3.18516335.18518165.rev.compl.REF    gacctttgactcaatcactataccccttcttatttctctctcaagttgtcgagagacta 7368
                                                                                                         **** ****************** ***********

chimp.LOC112206744-LOC107973052-GGT2.revcompl                                   tcagatctgtgtgacgtgtatggcatcatttcaccctcctaatgttttcttttctataat 20526
chimp54546-135457.revcompl.Pan.troglodytes.clone.rp43-41g5.GenBank:AC099533.36  TCAGATCTGTGTGACATGTATGGCATAATTTCACCCTCCTAATGTTTTCTTTTCTATAAT 22304
human.FAM30B-LOC105372935-GGT2.NCBI.                                            tcagatctgtgtgacgtgtatggcatcatttcaccctcctaatgttttcttttctataat 19213
human.FAM230E-LOC105377182-GGT3P.NCBI.                                          tcagatctgtgtgacgtgtatggcatcatttcaccctcctaatgttttcttttctataat 17950
human.FAM230J-LOC105372942-GGTLC5P.NCBI.rev.compl                               tcagatctgtgtgacgtgtatggcatcatttcaccctcctaatgttttcttttctataat 18664
human.FAM230A.ncRNA.18487127.18500594-GGTLC3.18516335.18518165.rev.compl.REF    tcagatctgtgtgacgtgtatggcatcatttcaccctcctaatgttttcttttctataat 7428
                                                                                *************** ********** *********************************

chimp.LOC112206744-LOC107973052-GGT2.revcompl                                   tgcaggagccattgacattggagaatgatacgtaccctgaaataactcacttcctgagga 20586
chimp54546-135457.revcompl.Pan.troglodytes.clone.rp43-41g5.GenBank:AC099533.36  TGCAGGAGCCATTGACACTGGAGAATGATACGTACCCTGAAATAACTCACTTCCTGAGGA 22364
human.FAM30B-LOC105372935-GGT2.NCBI.                                            tgcaggagccattgacactggagaatgatacgtaccctgaaataactcacttcctgagga 19273
human.FAM230E-LOC105377182-GGT3P.NCBI.                                          tgcaggagccattgacactggagaatgatacctaccctgaaataactcacttcctgagaa 18010
human.FAM230J-LOC105372942-GGTLC5P.NCBI.rev.compl                               tgcaggagccattgacactggagaatgatacctaccctgaaataactcacttcctgagga 18724
human.FAM230A.ncRNA.18487127.18500594-GGTLC3.18516335.18518165.rev.compl.REF    tgcaggagccattgacactggagaatgatacctaccctgaaataactcacttcctgagga 7488
                                                                                **************** ************** ************************** *

chimp.LOC112206744-LOC107973052-GGT2.revcompl                                   aaaagcgccatctctagggtacagaaacctgattctgggctccttttgggaaggaggatt 20646
chimp54546-135457.revcompl.Pan.troglodytes.clone.rp43-41g5.GenBank:AC099533.36  AAAAGTGCCATCTCTAGGGTACAGAAACCTGATTCTGGGCTCCTTTTGGGAAGGAGGATT 22424
human.FAM30B-LOC105372935-GGT2.NCBI.                                            aaaagcgccatctctagggtacagaaacctgattctgggctccttttgggaaggaggatt 19333
human.FAM230E-LOC105377182-GGT3P.NCBI.                                          aaaagcgccatctctagggtacagaaacctgattctgggctccttttgggaaggaggatt 18070
human.FAM230J-LOC105372942-GGTLC5P.NCBI.rev.compl                               aaaaacgccatctctagggtacagaaacctgattctgggctccttttgggaaggaggatt 18784
human.FAM230A.ncRNA.18487127.18500594-GGTLC3.18516335.18518165.rev.compl.REF    aaaaacgccatctctagggtacagaaacctgattctgggctccttttgggaaggaggatt 7548
                                                                                ****  ******************************************************

chimp.LOC112206744-LOC107973052-GGT2.revcompl                                   tggggtctggtgagagcaaatgattttgcaagtataaaacaatgtccagagaggctgtag 20706
chimp54546-135457.revcompl.Pan.troglodytes.clone.rp43-41g5.GenBank:AC099533.36  TGGGGTCTGGTGAGAGCAAATGATTTTGCAAGTATAAAACAATGTCCAGAGAGGCTGTAG 22484
human.FAM30B-LOC105372935-GGT2.NCBI.                                            tggggtctggtgagagcaaatgattttgcaagtataaaaccatgtccagagaggctgtag 19393
human.FAM230E-LOC105377182-GGT3P.NCBI.                                          tggggtctggtgagagcaaatgattttgcaagtataaaaccatgtccagagaggctgtag 18130
human.FAM230J-LOC105372942-GGTLC5P.NCBI.rev.compl                               tggagtctggtgagagcaaatgattttgcaagtataaaacaatgtccagagaggctgtag 18844
human.FAM230A.ncRNA.18487127.18500594-GGTLC3.18516335.18518165.rev.compl.REF    tggagtctggtgagagcaaatgattttgcaagtataaaacaatgtccagagaggctgtag 7608
                                                                                *** ************************************ *******************
```

```
chimp.LOC112206744-LOC107973052-GGT2.revcompl                                      ggatatctgtgagcccagaggaaacaccaggggatcctgtgcgaagcaccatggcttcag 20766
chimp54546-135457.revcompl.Pan.troglodytes.clone.rp43-41g5.GenBank:AC099533.36     GGATATCTGTGAGCCCAGAGGAAACACCAGGGGATCCTGTGCGAAGCACCATGGCTTCAG 22544
human.FAM30B-LOC105372935-GGT2.NCBI.                                               ggatatctgtgagcccagaggaaacaccaggggatcctgtgcgaagcaccatggcttcag 19453
human.FAM230E-LOC105377182-GGT3P.NCBI.                                             ggatatctgtgagcccagaggaaacaccaggggatcctgtgcgaagcaccatggcttcag 18190
human.FAM230J-LOC105372942-GGTLC5P.NCBI.rev.compl                                  ggatatctgtgagcccagaggaaacaccaggggatcctgtgcgaagcaccatggcttcag 18904
human.FAM230A.ncRNA.18487127.18500594-GGTLC3.18516335.18518165.rev.compl.REF       ggatatctgtgagcccagaggaaacaccaggggatcctgtgcgaagcaccatggcttcag 7668
                                                                                   ************************************************************

chimp.LOC112206744-LOC107973052-GGT2.revcompl                                      ctagggtgggaggagtgggtgggcctctctctaatgacttatcctggtgtttgtgtttct 20826
chimp54546-135457.revcompl.Pan.troglodytes.clone.rp43-41g5.GenBank:AC099533.36     CTAGGGTGGGAGGAGTGGGTGGGCCTCTCTCTAATGACTTATCCTGGTGTTTGTGTTTCT 22604
human.FAM30B-LOC105372935-GGT2.NCBI.                                               ctagggtgggaggagtgggtgggcctctctctaatgacttatcctggtgtttgtgtttct 19513
human.FAM230E-LOC105377182-GGT3P.NCBI.                                             ctagggtgggaggagtgggtgggcctctctctaatgacttatcctggtgtttgtgtttct 18250
human.FAM230J-LOC105372942-GGTLC5P.NCBI.rev.compl                                  ctagggtgggaggagtgggtgggcctctctctaatgacttatcctggtgtttgtgtttct 18964
human.FAM230A.ncRNA.18487127.18500594-GGTLC3.18516335.18518165.rev.compl.REF       ctagggtgggaggagtgggtgggcctctctctaatgacttatcctggtgtttgtgtttct 7728
                                                                                   ************************************************************

chimp.LOC112206744-LOC107973052-GGT2.revcompl                                      aaagatttgattgtggagagcatatctgatgatggggatttgtaggtaggtaactacttt 20886
chimp54546-135457.revcompl.Pan.troglodytes.clone.rp43-41g5.GenBank:AC099533.36     AAAGATTTGATTGTGGAGAGCATATCTGATGATGGGGATTTGTAGGTAGGTAACTACTTT 22664
human.FAM30B-LOC105372935-GGT2.NCBI.                                               aaagatttgattgtggagagcatatctgatgatggggatttgtaggtaggtaactgcttt 19573
human.FAM230E-LOC105377182-GGT3P.NCBI.                                             aaagatttgattgtggagagcatatctgatgatggggatttgtaggtaggtaactacttt 18310
human.FAM230J-LOC105372942-GGTLC5P.NCBI.rev.compl                                  aaagatttgattgtggagagcatatctgatgatggggatttgtaggtaggtaactacttt 19024
human.FAM230A.ncRNA.18487127.18500594-GGTLC3.18516335.18518165.rev.compl.REF       aaagatttgattgtggagagcatatctgatgatggggatttgtaggtaggtaactacttt 7788
                                                                                   ******************************************************* ****

chimp.LOC112206744-LOC107973052-GGT2.revcompl                                      ccacgtaagatccaattggagagagttcccaggggccttcggggtatccatgctgcttgg 20946
chimp54546-135457.revcompl.Pan.troglodytes.clone.rp43-41g5.GenBank:AC099533.36     CCACGTAAGATCCAATTGGAGAGAGTTCCCAGGGGCCTTCGGGGTATCCATGCTGCTTGG 22724
human.FAM30B-LOC105372935-GGT2.NCBI.                                               ccacgtaagatccaattggagagagttcccaggggccttcggggtatccatgctgcttgg 19633
human.FAM230E-LOC105377182-GGT3P.NCBI.                                             ccacgtaagatccaattggagagagttcccaggggccttcggggtatccatgctgcttgg 18370
human.FAM230J-LOC105372942-GGTLC5P.NCBI.rev.compl                                  ccacgtaagatccaattggagagagttcccaggggccttcggggtatccatgctgcttgg 19084
human.FAM230A.ncRNA.18487127.18500594-GGTLC3.18516335.18518165.rev.compl.REF       ccacgtaagatccaattggagagagttcccaggggccttcggggtatccatgctgcttgg 7848
                                                                                   ************************************************************

chimp.LOC112206744-LOC107973052-GGT2.revcompl                                      gaggttaagggagggggcatgaaatcaaaacgaaacaggaaatatgtgtcatattggatt 21006
chimp54546-135457.revcompl.Pan.troglodytes.clone.rp43-41g5.GenBank:AC099533.36     GAGGTTAAGGGAGGGGGCATGAAATCAAAACGAAACAGGAAATATGTGTCATATTGGATT 22784
human.FAM30B-LOC105372935-GGT2.NCBI.                                               gaggttaagggagggggcatgaaatcaaaacgaaacaggaaatatgtgtcatatcggatt 19693
human.FAM230E-LOC105377182-GGT3P.NCBI.                                             gaggttaagggagggggcatgaaatcaaaacaaaacaggaaatatgtgtcatattggact 18430
human.FAM230J-LOC105372942-GGTLC5P.NCBI.rev.compl                                  gaggttaagggagggggcatgaaatcaaaaggaaacaggaaatatgtgtcatattggatt 19144
human.FAM230A.ncRNA.18487127.18500594-GGTLC3.18516335.18518165.rev.compl.REF       gaggttaagggagggggcatgaaatcaaaaggaaacaggaaatatgtgtcatattggatt 7908
                                                                                   ******************************  ********************** *** *

chimp.LOC112206744-LOC107973052-GGT2.revcompl                                      tggtcttttccgggtttattggcataatagtaagaactgtctctctgggctatgagggtg 21066
chimp54546-135457.revcompl.Pan.troglodytes.clone.rp43-41g5.GenBank:AC099533.36     TGGTCTTTTCCGGGTTTATTGGCATAATAGTTAGAACTGTCTCTCTGGGCTATGAGGGTG 22844
human.FAM30B-LOC105372935-GGT2.NCBI.                                               tggtcttttccgggtttattggcataatagttagaactgtctctctgggctatgagggtg 19753
human.FAM230E-LOC105377182-GGT3P.NCBI.                                             tggtcttttccgggtttattggcataatagttagaactgtctctctgggctatgagggtg 18490
human.FAM230J-LOC105372942-GGTLC5P.NCBI.rev.compl                                  tggtcttttccgggtttattggcataatagttagaactgtctctctgggctatgagggtg 19204
human.FAM230A.ncRNA.18487127.18500594-GGTLC3.18516335.18518165.rev.compl.REF       tggtcttttccgggtttattggcataatagttagaactgtctctctgggctatgagggtg 7968
                                                                                   ******************************* ****************************

chimp.LOC112206744-LOC107973052-GGT2.revcompl                                      ctgtgttatttaaaggtggtctttcccagaacacctggccttttcttttccgcctctgcc 21126
chimp54546-135457.revcompl.Pan.troglodytes.clone.rp43-41g5.GenBank:AC099533.36     CTGTGTTATTTAAAGGTGGTCTTTCCCAGAGCACCTGGCCTTTTCTTTTCTGCCTCTGCC 22904
human.FAM30B-LOC105372935-GGT2.NCBI.                                               ctgtgttatttaaaggtggtctttcccagaacacctggccttttcttttctgcctctgcc 19813
human.FAM230E-LOC105377182-GGT3P.NCBI.                                             ctgtgttatttaaaggtggtctttcccagaacacctggccttttcttttctgcctctgcc 18550
human.FAM230J-LOC105372942-GGTLC5P.NCBI.rev.compl                                  ctgtgatatttaaaggtggtctttcccagaacacctggccttttcttttctgcctctgcc 19264
human.FAM230A.ncRNA.18487127.18500594-GGTLC3.18516335.18518165.rev.compl.REF       ctgtgatatttaaaggtggtctttcccagaacacctggccttttcttttctgcctctgcc 8028
                                                                                   ***** ************************ ******************* *********

chimp.LOC112206744-LOC107973052-GGT2.revcompl                                      aaacatcacagactttgggttggattagtcagcaccccttgggattgtgcagaagaggtt 21186
chimp54546-135457.revcompl.Pan.troglodytes.clone.rp43-41g5.GenBank:AC099533.36     AAACATCACAGCCTTTGGGTTGGATTAGTCAGCACCCCTTGGGATTGTGCAGAAGAGGTT 22964
human.FAM30B-LOC105372935-GGT2.NCBI.                                               aaacatcacagcctttgggttggattagtcagcaccccttgggattgtgcagaagaggtt 19873
human.FAM230E-LOC105377182-GGT3P.NCBI.                                             aaacatcacagcctttgggttggattagtcagcaccccttgggattgtgcagaagaggtt 18610
human.FAM230J-LOC105372942-GGTLC5P.NCBI.rev.compl                                  aaacatcacagcctttgggttggattagtcagcaccccttgggattgtgcagaagaggtt 19324
human.FAM230A.ncRNA.18487127.18500594-GGTLC3.18516335.18518165.rev.compl.REF       aaacatcacagcctttgggttggattagtcagcaccccttgggattgtgcagaagaggtt 8088
                                                                                   *********** ************************************************

chimp.LOC112206744-LOC107973052-GGT2.revcompl                                      tggggttgcatcgagtgtcacctgtggtgaacagaatctgagggacacaactctctcaca 21246
chimp54546-135457.revcompl.Pan.troglodytes.clone.rp43-41g5.GenBank:AC099533.36     TGGGGTTGCACCGAGTGTCACCTGTGGTGAACAGAATCTGAGGGACACAACTCTCTCACA 23024
human.FAM30B-LOC105372935-GGT2.NCBI.                                               tggggttgcattgagtgtcacctgtggtgaacagaatctgagggacacaactctctcaca 19933
human.FAM230E-LOC105377182-GGT3P.NCBI.                                             tggggttgcatcgagtgtcacctgtggtgaacagaatctgagggacacaactctctcaca 18670
human.FAM230J-LOC105372942-GGTLC5P.NCBI.rev.compl                                  tggggttgcatcgagtgtcacctgtggtgaacagaatctgagggacacaactctctcaca 19384
human.FAM230A.ncRNA.18487127.18500594-GGTLC3.18516335.18518165.rev.compl.REF       tggggttgcatcgagtgtcacctgtggtgaacagaatctgagggacacaactctctcaca 8148
                                                                                   **********  ************************************************

chimp.LOC112206744-LOC107973052-GGT2.revcompl                                      ggcacttccttcaacctggagacagagttctcctggtgtgtgcccaggggtgcaggagaa 21306
chimp54546-135457.revcompl.Pan.troglodytes.clone.rp43-41g5.GenBank:AC099533.36     GGCACTTCCTTCAACCTGGAGACAGAGTTCTCCTGGTGTGTGCCCAGGGGTGGAGGAGAA 23084
human.FAM30B-LOC105372935-GGT2.NCBI.                                               ggcacttcctccaacctggagacagagttctcctggtgtgtgcccaggggtggaggagaa 19993
human.FAM230E-LOC105377182-GGT3P.NCBI.                                             ggcacttcctccaacctggagacagaattctcctggtgtgtgcccaggggtggaggagaa 18730
human.FAM230J-LOC105372942-GGTLC5P.NCBI.rev.compl                                  ggcacttccttcaacctggagacagagttctcctggtgtgtgcccaggggtggaggagaa 19444
human.FAM230A.ncRNA.18487127.18500594-GGTLC3.18516335.18518165.rev.compl.REF       ggcacttccttcaacctggagacagagttctcctggtgtgtgcccaggggtggaggagaa 8208
                                                                                   ********** *************** ************************* *******

chimp.LOC112206744-LOC107973052-GGT2.revcompl                                      attgacagtctgcctctgaactttcaggactttaaaaagcactcatgtttccatcctcgc 21366
chimp54546-135457.revcompl.Pan.troglodytes.clone.rp43-41g5.GenBank:AC099533.36     ATTGACAGTCTGCCTCTGAACTTTCAGGACTTTAAAAAGCACTCATGTTTCCATCCTCAC 23144
human.FAM30B-LOC105372935-GGT2.NCBI.                                               attgacagtctgcctctgaactttcaggactttaaaaagcactcatgtttccatcctcac 20053
human.FAM230E-LOC105377182-GGT3P.NCBI.                                             attgacagtctgcctctgaactttcaggactttaaaaagcactcatgtttccatcctcac 18790
human.FAM230J-LOC105372942-GGTLC5P.NCBI.rev.compl                                  attgacagtctgcctctgaactttcaggactttaaaaagcactcatgtttccatcctcac 19504
human.FAM230A.ncRNA.18487127.18500594-GGTLC3.18516335.18518165.rev.compl.REF       attgacagtctgcctctgaactttcaggactttaaaaagcactcatgtttccatcctcac 8268
                                                                                   ********************************************************** *

chimp.LOC112206744-LOC107973052-GGT2.revcompl                                      tgttgactcctggcttaaagggatctccaggggtgagtgaggaggagggatcggaccctg 21426
chimp54546-135457.revcompl.Pan.troglodytes.clone.rp43-41g5.GenBank:AC099533.36     TGTTGACTCCTGGCTTAAAGGGATCTCCCGGGGTGAGTGAGGAGGCGGGATCGGACCCTG 23204
human.FAM30B-LOC105372935-GGT2.NCBI.                                               tgttgactcctggcttaaagggatctcccggggtgagtgaggaggcgggatcggaccctg 20113
human.FAM230E-LOC105377182-GGT3P.NCBI.                                             tgttgactcctggcttaaagggatctcccggggtgagtgaggaggcgggatcggacccta 18850
human.FAM230J-LOC105372942-GGTLC5P.NCBI.rev.compl                                  tgttgactcctggcttaaagggatctcccggggtgagtgaggaggcgggattggaccctg 19564
human.FAM230A.ncRNA.18487127.18500594-GGTLC3.18516335.18518165.rev.compl.REF       tgttgactcctggcttaaagggatctcccggggtgagtgaggaggcgggatcggaccctg 8328
                                                                                   **************************** **************** ***** *******

chimp.LOC112206744-LOC107973052-GGT2.revcompl                                      gcagtctgacggcagcacctgtgttcctctgcactgggccgtggatgacattacacacct 21486
chimp54546-135457.revcompl.Pan.troglodytes.clone.rp43-41g5.GenBank:AC099533.36     GCAGTCTGACGGCAGCACCTGTGTTCCTCTGCACTGGGCCGTGGATGACATTACACACCT 23264
human.FAM30B-LOC105372935-GGT2.NCBI.                                               gcagtctgacggcagcacctgtgttcctctgcactgggccgtggatgacattacatacct 20173
human.FAM230E-LOC105377182-GGT3P.NCBI.                                             gcagtctgacggcagcacctgtgttcctctgcactgggccgtggatgacattacacacct 18910
human.FAM230J-LOC105372942-GGTLC5P.NCBI.rev.compl                                  gcagtctgacggcagcacctgtgttcctctgcactgggccgtggatgacattacacacct 19624
human.FAM230A.ncRNA.18487127.18500594-GGTLC3.18516335.18518165.rev.compl.REF       gcagtctgacggcagcacctgtgttcctctgcactgggccgtggatgacattacacacct 8388
                                                                                   ******************************************************* ****

chimp.LOC112206744-LOC107973052-GGT2.revcompl                                      tggtgagaatcaggaattgaggctaaccacatctgaaattgagatgggccttgagtcata 21546
chimp54546-135457.revcompl.Pan.troglodytes.clone.rp43-41g5.GenBank:AC099533.36     TGGTGAGAATCAGGAATTGAGGCTAACCACATCTGAAATTGAGATGGGCCTTGAGTCATA 23324
human.FAM30B-LOC105372935-GGT2.NCBI.                                               tggtgagaatcaggaattgaggctaaccacatctgaaattgagatgggccttgagtcata 20233
human.FAM230E-LOC105377182-GGT3P.NCBI.                                             tggtgagaatcaggaattgaggctaaccacatctgaaattgagatgggccttgagtcata 18970
human.FAM230J-LOC105372942-GGTLC5P.NCBI.rev.compl                                  tggtgagaatcaggaattgaggctaaccacatctgaaattgagatgggccttgagtcata 19684
human.FAM230A.ncRNA.18487127.18500594-GGTLC3.18516335.18518165.rev.compl.REF       tggtgagaatcaggaattgaggctaaccacatctgaaattgagatgggccttgagtcata 8448
                                                                                   ************************************************************

chimp.LOC112206744-LOC107973052-GGT2.revcompl                                      taaatagtttggaaaagatgcattttactacgctattgaaagaaaccatttatttctcac 21606
chimp54546-135457.revcompl.Pan.troglodytes.clone.rp43-41g5.GenBank:AC099533.36     TAAATAGTTTGGAAAAGATGCATTTTACTACGCTATTGAAAGAAACCATTTATTTCTCAC 23384
human.FAM30B-LOC105372935-GGT2.NCBI.                                               taaatagtttggaaaagatgcattttactacgctattgaaagaaaccatttatttctcac 20293
human.FAM230E-LOC105377182-GGT3P.NCBI.                                             taaatagtttggaaaagatgcattttactacgctattgaaagaaaccatttatttctcac 19030
human.FAM230J-LOC105372942-GGTLC5P.NCBI.rev.compl                                  taaatagtttggaaaagatgcattttactacgctattgaaagaaaccatttatttctcac 19744
human.FAM230A.ncRNA.18487127.18500594-GGTLC3.18516335.18518165.rev.compl.REF       taaatagtttggaaaagatgcattttactacgctattgaaagaaaccatttatttctcac 8508
                                                                                   ************************************************************

chimp.LOC112206744-LOC107973052-GGT2.revcompl                                      tccagcaggataaatggttttcagtatccatttaactgctcattgactcttactgtagat 21666
chimp54546-135457.revcompl.Pan.troglodytes.clone.rp43-41g5.GenBank:AC099533.36     TCCAGCAGGATAAATGGTTTTCAGTATCCATTTAACTGCTCATTGACTCTTACTGTGGAT 23444
human.FAM30B-LOC105372935-GGT2.NCBI.                                               tccagcaggataaatggttttcagtatccatttaactgctcattgactcttactgtagat 20353
human.FAM230E-LOC105377182-GGT3P.NCBI.                                             tccagcaggataaatggttttcagtatccatttaactgctcattgactcttactgtagat 19090
human.FAM230J-LOC105372942-GGTLC5P.NCBI.rev.compl                                  tccagcaggataaatggttttcagtatccacttaactgctcattgactcttactgtagat 19804
human.FAM230A.ncRNA.18487127.18500594-GGTLC3.18516335.18518165.rev.compl.REF       tccagcaggataaatggttttcagtatccacttaactgctcattgactcttactgtagat 8568
                                                                                   ****************************** ************************* ***

chimp.LOC112206744-LOC107973052-GGT2.revcompl                                      gaggaggtggccagcagcccctgccctcccccagtt-gtaggcccaaggtaaccagcaat 21725
chimp54546-135457.revcompl.Pan.troglodytes.clone.rp43-41g5.GenBank:AC099533.36     GAGGAGGTAGCCAGCAGCCCCTGCCCTCCCCCAGTT-GTAGGCCCAAGGTAACCAGCAAT 23503
human.FAM30B-LOC105372935-GGT2.NCBI.                                               gaggaggtggccagcagcccctgccctcccccagttggtaggcccaaggtaaccagcaat 20413
human.FAM230E-LOC105377182-GGT3P.NCBI.                                             gaggaggtggccagcagcccctgccctcccccagttggtaggcccaaggtaaccagcaat 19150
human.FAM230J-LOC105372942-GGTLC5P.NCBI.rev.compl                                  gaggaggtggccagcagcccctgccctcccccagtt-gtaggcccaaggtaaccagcaat 19863
human.FAM230A.ncRNA.18487127.18500594-GGTLC3.18516335.18518165.rev.compl.REF       gaggaggtggccagcagcccctgccctcccccagtt-gtaggcccaaggtaaccagcaat 8627
                                                                                   ******** *************************** ***********************

chimp.LOC112206744-LOC107973052-GGT2.revcompl                                      ggactggatataatggaagagtggtgcattcggaggtatctgtattaatgggacccacat 21785
chimp54546-135457.revcompl.Pan.troglodytes.clone.rp43-41g5.GenBank:AC099533.36     GGACTGGATATAATGGAAGGGTGGTGCATTCGGAGGTATCTGTATTAATGGGACCCACAT 23563
human.FAM30B-LOC105372935-GGT2.NCBI.                                               tgactggatataatggaagagtggtgcattcgggggtatctgtattaatgggacccacat 20473
human.FAM230E-LOC105377182-GGT3P.NCBI.                                             tgactggatataatggaagagtggtgcattcggaggtatctgtattaatgggacccacat 19210
human.FAM230J-LOC105372942-GGTLC5P.NCBI.rev.compl                                  tgactggatataatggaagagtggtgcattcggaggtatctgtattaatgggacccacat 19923
human.FAM230A.ncRNA.18487127.18500594-GGTLC3.18516335.18518165.rev.compl.REF       tgactggatataatggaagagtggtgcattcggaggtatctgtattaatgggacccacat 8687
                                                                                    ****************** ************* **************************
```

```
chimp.LOC112206744-LOC107973052-GGT2.revcompl                                     gatatggatgagagctattagggtgagaaaaagcctgggagcacaatgaaatatttaaat 21845
chimp54546-135457.revcompl.Pan.troglodytes.clone.rp43-41g5.GenBank:AC099533.36    GATATGGATGAGAGCTATTAGGGTGAGAAAAAGCCTGGGAGCACAATGAAATATTTAAAT 23623
human.FAM30B-LOC105372935-GGT2.NCBI.                                              gatatggatgagagctattagggtgagaaaaagcctgggagcacaatgaaatatttaaat 20533
human.FAM230E-LOC105377182-GGT3P.NCBI.                                            gatatggatgagagctattagggtgagaaaaagcctgggagcacaatgaaatatttaaat 19270
human.FAM230J-LOC105372942-GGTLC5P.NCBI.rev.compl                                 gatatggatgagagctattagggtgagaaaaagcctgggagcacaatgaaatatttaaat 19983
human.FAM230A.ncRNA.18487127.18500594-GGTLC3.18516335.18518165.rev.compl.REF      gatatggatgagagctattagggtgagaaaaagcctgggagcacaatgaaatatttaaat 8747
                                                                                  ************************************************************

chimp.LOC112206744-LOC107973052-GGT2.revcompl                                     attaaacaaaacattgttgaaatctccattgtactttagtagttgaagtcattcttgtgg 21905
chimp54546-135457.revcompl.Pan.troglodytes.clone.rp43-41g5.GenBank:AC099533.36    ATTAAACAAAACATTGTTGAAATCTCCATTGTACTTTAGTAGTTGAAGTCATTCTTGTGG 23683
human.FAM30B-LOC105372935-GGT2.NCBI.                                              attaaacaaaacattgttgaaatctccattgtactttagtagttgaagtcattcttgtgg 20593
human.FAM230E-LOC105377182-GGT3P.NCBI.                                            attaaacaaaacattgttgaaatctccattgtactttagtagttgaagtcattcttgtgg 19330
human.FAM230J-LOC105372942-GGTLC5P.NCBI.rev.compl                                 attaaacaaaacattgttgaaatctccattgtactttagtagttgaagtcattcttgtgg 20043
human.FAM230A.ncRNA.18487127.18500594-GGTLC3.18516335.18518165.rev.compl.REF      attaaacaaaacattgttgaaatctccattgtactttagtagttgaagtcattcttgtgg 8807
                                                                                  ************************************************************

chimp.LOC112206744-LOC107973052-GGT2.revcompl                                     tcatcactgcctttcccaagcataacaagctacttaatatcacatagacccgtgccatga 21965
chimp54546-135457.revcompl.Pan.troglodytes.clone.rp43-41g5.GenBank:AC099533.36    TCATCACTGCCTTTCCCAAGCATAACAAGCTACTTAATATCACATAGACCCATGCCATGA 23743
human.FAM30B-LOC105372935-GGT2.NCBI.                                              tcatcactgcctttcccaagcataacaagctacttaatatcacatggacccgtgccatga 20653
human.FAM230E-LOC105377182-GGT3P.NCBI.                                            tcatcactgcctttcccaagcataacaagctacttaatatcacatggacccgtgccatga 19390
human.FAM230J-LOC105372942-GGTLC5P.NCBI.rev.compl                                 tcatcactgcctttcccaagcataacaagctacttaatatcacatggacccgtgccatga 20103
human.FAM230A.ncRNA.18487127.18500594-GGTLC3.18516335.18518165.rev.compl.REF      tcatcactgcctttcccaagcataacaagctacttaatatcacatggacccgtgccatga 8867
                                                                                  ********************************************* ***** ********

chimp.LOC112206744-LOC107973052-GGT2.revcompl                                     ggaatgatgatcagtttgtaaaatgccaataaaacaattgcctatagaagccacaatgtt 22025
chimp54546-135457.revcompl.Pan.troglodytes.clone.rp43-41g5.GenBank:AC099533.36    GGAATGATGATCAGTTTGTAAAATGCCAATAAAACAATTGCCTATATAAGCCACAATGTT 23803
human.FAM30B-LOC105372935-GGT2.NCBI.                                              ggaatgatgatcagtttgtaaaatgccaataaaacaattgcctatataagccacaatgtt 20713
human.FAM230E-LOC105377182-GGT3P.NCBI.                                            ggaatgatgatcagtttgtaaaatgccaataaaacaattgcctatataagccacaatgtt 19450
human.FAM230J-LOC105372942-GGTLC5P.NCBI.rev.compl                                 ggaatgatgatcagtttgtaaaatgccaataaaacaattgcctatataagccacaatgtt 20163
human.FAM230A.ncRNA.18487127.18500594-GGTLC3.18516335.18518165.rev.compl.REF      ggaatgatgatcagtttgtcaaatgccaataaaacaattgcctctataagccacaatgtt 8927
                                                                                  ******************* *********************** ** *************

chimp.LOC112206744-LOC107973052-GGT2.revcompl                                     tcatccatatatttcaatttccatgtgtaagtatagttcaaatttcagaaatttattatt 22085
chimp54546-135457.revcompl.Pan.troglodytes.clone.rp43-41g5.GenBank:AC099533.36    TCATCCATATATTTCAATTTCCATGTGTAAGTATAGTTCAAATTTCAGAAAGTTATTATT 23863
human.FAM30B-LOC105372935-GGT2.NCBI.                                              tcatccatatatttcaatttccatgtgtaagtatagttcaaatttcagaaatttattatt 20773
human.FAM230E-LOC105377182-GGT3P.NCBI.                                            tcatccatatatttcaatttccatgtataagtatagttcaaatttcagaaatttattatt 19510
human.FAM230J-LOC105372942-GGTLC5P.NCBI.rev.compl                                 tcatccatatatttcaatttccatgtgtaagtatagttcaaatttcagaaatttattatt 20223
human.FAM230A.ncRNA.18487127.18500594-GGTLC3.18516335.18518165.rev.compl.REF      tcatccatatatttcaatttccatgtgtaagtatagttcaaatttcagaaatttattatt 8987
                                                                                  ************************** ************************ ********

chimp.LOC112206744-LOC107973052-GGT2.revcompl                                     atctaatagaatatgcatggtatatcaatgagcaattatcatactgtttctattaacaat 22145
chimp54546-135457.revcompl.Pan.troglodytes.clone.rp43-41g5.GenBank:AC099533.36    ATCTAATAGAATATGCATGGTATATCAATGAGAAATTATCATACTGTTTCTATTAACAAT 23923
human.FAM30B-LOC105372935-GGT2.NCBI.                                              atctaatagaatatgcatggtatatcaatgagcaattatcatactgtttctattaacaat 20833
human.FAM230E-LOC105377182-GGT3P.NCBI.                                            atctaatagaatatgcatggtatatcaatgagcaattatcatactgtttctattaacaat 19570
human.FAM230J-LOC105372942-GGTLC5P.NCBI.rev.compl                                 atctaatagaatatgcatggtatatcaatgagcaattatcatactgtttctattaacaat 20283
human.FAM230A.ncRNA.18487127.18500594-GGTLC3.18516335.18518165.rev.compl.REF      atctaatagaatatgcatggtatatcaatgagcaattatcatactgtttctattaacaat 9047
                                                                                  ******************************** ***************************

chimp.LOC112206744-LOC107973052-GGT2.revcompl                                     tatttgtatgatgaaaaaagcagactcccattcttggatttttctcagtttgcacacatt 22205
chimp54546-135457.revcompl.Pan.troglodytes.clone.rp43-41g5.GenBank:AC099533.36    TATTTGTATGATGAAAAAAGCAGACTCCCATTCTTGGATTTTTCTCAGTTTGCACACATT 23983
human.FAM30B-LOC105372935-GGT2.NCBI.                                              tatttgtatgatgaaaaaagcagactcccattcttggatttttctcagtttgcacacatt 20893
human.FAM230E-LOC105377182-GGT3P.NCBI.                                            tatttgtatgatgaaaaaagcagactcccattcttggatttttctcagtttgcacacatt 19630
human.FAM230J-LOC105372942-GGTLC5P.NCBI.rev.compl                                 tatttgtatgatgaaaaaagcagactcccattcttggatttttctcagtttgcacacatt 20343
human.FAM230A.ncRNA.18487127.18500594-GGTLC3.18516335.18518165.rev.compl.REF      tatttgtatgatgaaaaaagcagactcccattcttggatttttctcagtttgcacacatt 9107
                                                                                  ************************************************************

chimp.LOC112206744-LOC107973052-GGT2.revcompl                                     agcatgacagccccatttccacctgacatgtgccagcaagaggccaggaacagaggcttt 22265
chimp54546-135457.revcompl.Pan.troglodytes.clone.rp43-41g5.GenBank:AC099533.36    AGCATGACAGCCCCATTTCCACCTGACATGTGCCAGCAAGAGGCCAGGAACAGAGGCTTT 24043
human.FAM30B-LOC105372935-GGT2.NCBI.                                              agcatgacagccccatttccacctgacatgtgccagcaagaggccaggaacagaggcttt 20953
human.FAM230E-LOC105377182-GGT3P.NCBI.                                            agcatgacagccccatttccacctgacatgtgccagcaagaggccaggaacagaggcttt 19690
human.FAM230J-LOC105372942-GGTLC5P.NCBI.rev.compl                                 agcatgacagccccatttccacctgacatgtgccagcaagaggccaggaacagaggcttt 20403
human.FAM230A.ncRNA.18487127.18500594-GGTLC3.18516335.18518165.rev.compl.REF      agcatgacagccccatttccacctgacatgtgccagcaagaggccaggaacagaggcttt 9167
                                                                                  ************************************************************

chimp.LOC112206744-LOC107973052-GGT2.revcompl                                     tcttat----taagatttctaaatgtattatgtattcacatttagaaactctaaatgtca 22321
chimp54546-135457.revcompl.Pan.troglodytes.clone.rp43-41g5.GenBank:AC099533.36    TCTTATTAACTAAGATTTCTAAATGTATTATGTATTCACATTTAGAAACTCTAAATATCA 24103
human.FAM30B-LOC105372935-GGT2.NCBI.                                              tcttattaactaagatttctaaatgtattacgtattcacatttagaaactctaaatatca 21013
human.FAM230E-LOC105377182-GGT3P.NCBI.                                            tcttattaactaagatttctaaatgtattacgtattcacatttagaaactctaaatatca 19750
human.FAM230J-LOC105372942-GGTLC5P.NCBI.rev.compl                                 tcttattaactaagatttctaaatgtattacgtattcacatttagaaactctaaatatca 20463
human.FAM230A.ncRNA.18487127.18500594-GGTLC3.18516335.18518165.rev.compl.REF      tcttattaactaagatttctaaatgtattacgtattcacatttagaaactctaaatatca 9227
                                                                                  ******    ******************** ************************* ***

chimp.LOC112206744-LOC107973052-GGT2.revcompl                                     taaaaggttagcaaggaagtttcccttccactctgaacttccaaacaccaagtcaacatt 22381
chimp54546-135457.revcompl.Pan.troglodytes.clone.rp43-41g5.GenBank:AC099533.36    TAAAAGGTTAGCAAGGAAGTTTCCCTTCCACTCTGAACTTCCAAACACCAAGTCAACATT 24163
human.FAM30B-LOC105372935-GGT2.NCBI.                                              taaaaggttagcaaggaagtttcccttccactctgaacttccaaacaccaagtcaacatt 21073
human.FAM230E-LOC105377182-GGT3P.NCBI.                                            taaaaggttagcaaggaagtttcccttccactctgaacttccaaacaccaagtcaacatt 19810
human.FAM230J-LOC105372942-GGTLC5P.NCBI.rev.compl                                 taaaaggttagcaaggaagtttcccttccactctgaacttccaaacaccaagtcaacatt 20523
human.FAM230A.ncRNA.18487127.18500594-GGTLC3.18516335.18518165.rev.compl.REF      taaaaggttagcaaggaagtttcccttccactctgaacttccaaacaccaagtcaacgtt 9287
                                                                                  ********************************************************* **

chimp.LOC112206744-LOC107973052-GGT2.revcompl                                     tttgtttgcatatcatccctgcaatctatgtgcaaatagaagcatgcacctggaatgcag 22441
chimp54546-135457.revcompl.Pan.troglodytes.clone.rp43-41g5.GenBank:AC099533.36    TTTGTTTACATATCATCCCTGCAATCTATGTGCAAATAGAAGCATGCACCTGGAATGCAG 24223
human.FAM30B-LOC105372935-GGT2.NCBI.                                              tttgtttgcatatcatccctgcaatctatgtgcaaatagaagcatgcacctggaatgcag 21133
human.FAM230E-LOC105377182-GGT3P.NCBI.                                            tttgtttgcatatcatccctgcaatctatgtgcaaatagaagcatgcacctggaatgcag 19870
human.FAM230J-LOC105372942-GGTLC5P.NCBI.rev.compl                                 tttgtttgcatatcatccctgcaatctatgtgcaaatagaagcatgcacctggaatgcag 20583
human.FAM230A.ncRNA.18487127.18500594-GGTLC3.18516335.18518165.rev.compl.REF      tttgtttgcatatcatccctgcaatctatgtgcaaatagaagcatgcacctggaatgcag 9347
                                                                                  ******* ****************************************************

chimp.LOC112206744-LOC107973052-GGT2.revcompl                                     gctgatgtgtgatcgtgtttacacaaagtcctctgcacctctgcatatatcactgggcaa 22501
chimp54546-135457.revcompl.Pan.troglodytes.clone.rp43-41g5.GenBank:AC099533.36    GCTGATGTGTGATCGTGTTTACACAAAGTCCTCTGCACCTCTGCATGTATCACTGGGCAA 24283
human.FAM30B-LOC105372935-GGT2.NCBI.                                              gctgatgtgtgatcatgtttacacaaagtcctctgcacctctgcatatatcactgggcaa 21193
human.FAM230E-LOC105377182-GGT3P.NCBI.                                            gctgatgtgtgatcgtgtttacacaaagtcctctgcacctctgcatatatcactgggcaa 19930
human.FAM230J-LOC105372942-GGTLC5P.NCBI.rev.compl                                 gctgatgtgtgatcgtgtttacacaaagtcctctgcacctctgcatatatcactgggcaa 20643
human.FAM230A.ncRNA.18487127.18500594-GGTLC3.18516335.18518165.rev.compl.REF      gctgatgtgtgatcgtgtttacacaaagtcctctgcacctctgcatatatcactgggcaa 9407
                                                                                  ************** ******************************* *************

chimp.LOC112206744-LOC107973052-GGT2.revcompl                                     tgcaccttagttatcattccacatttcaaatgtaaatccattgtattgtttcagagctat 22561
chimp54546-135457.revcompl.Pan.troglodytes.clone.rp43-41g5.GenBank:AC099533.36    TGCACCTTAGTTATCATTCCACATTTCAAATGTAAATCCATTGTATTGTTTCAGAGCTAT 24343
human.FAM30B-LOC105372935-GGT2.NCBI.                                              tgcaccttagttatcattccacatttcaaatgtaaatccattgtattgtttcagagctat 21253
human.FAM230E-LOC105377182-GGT3P.NCBI.                                            tgcaccttagttatcattccacatttcaaatgtaaatccattgtattgtttcagagctat 19990
human.FAM230J-LOC105372942-GGTLC5P.NCBI.rev.compl                                 tgcaccttagttatcattccacatttcaaatgtaaatccattgtattgtttcagagctat 20703
human.FAM230A.ncRNA.18487127.18500594-GGTLC3.18516335.18518165.rev.compl.REF      tgcaccttagttatcattccacatttcaaatgtaaatccattgtattgtttcagagctat 9467
                                                                                  ************************************************************

chimp.LOC112206744-LOC107973052-GGT2.revcompl                                     gaagtactgcaccccatgactattcccaaaattacttaagcaccccgctatgggtatcca 22621
chimp54546-135457.revcompl.Pan.troglodytes.clone.rp43-41g5.GenBank:AC099533.36    AAAGTACTGCACCCCATGACTATTCCCAAAATTACTTAAGCACCCCGCTATGGGTATCCA 24403
human.FAM30B-LOC105372935-GGT2.NCBI.                                              aaagtactgcaccccatgactattcccaaaattacttaagcaccccgctatgggtatcca 21313
human.FAM230E-LOC105377182-GGT3P.NCBI.                                            aaagtactgcaccccatgactattcccaaaattacttaagcaccccgctatgggtatcca 20050
human.FAM230J-LOC105372942-GGTLC5P.NCBI.rev.compl                                 aaagtactgcaccccatgactattcccaaaattacttaagcaccccgctatgggtatcca 20763
human.FAM230A.ncRNA.18487127.18500594-GGTLC3.18516335.18518165.rev.compl.REF      aaagtactgcaccccatgactattcccaaaattacttaagcaccccgctatgggtatccg 9527
                                                                                   **********************************************************

chimp.LOC112206744-LOC107973052-GGT2.revcompl                                     tttgttctgtttccagtcttgctcttataaccaatgctgtagtgaacagcactgtgttga 22681
chimp54546-135457.revcompl.Pan.troglodytes.clone.rp43-41g5.GenBank:AC099533.36    TTTGTTCTGTTTCCAGTCTTGCTCTTATAACCAATGCTGTAGTGAACAGCACTGTGTTGA 24463
human.FAM30B-LOC105372935-GGT2.NCBI.                                              tttgttctgtttccagtcttgctcttataaccaatgctgtagtgaacagcactgtgttga 21373
human.FAM230E-LOC105377182-GGT3P.NCBI.                                            tttgttctgtttccagtcttgctcttataaccaatgctgtagtgaacagcactgtgttga 20110
human.FAM230J-LOC105372942-GGTLC5P.NCBI.rev.compl                                 tttgttctgtttccagtcttgctcttataaccaatgctgtagtgaacagcactgtgttga 20823
human.FAM230A.ncRNA.18487127.18500594-GGTLC3.18516335.18518165.rev.compl.REF      tttgttctgtttccagtcttgctcttataaccaatgctgtagtgaacagcactgtgttga 9587
                                                                                  ************************************************************

chimp.LOC112206744-LOC107973052-GGT2.revcompl                                     gaagtggtgaacgtgggcatctttgtcttgttcccgtcctcagggggaatgctttcaact 22741
chimp54546-135457.revcompl.Pan.troglodytes.clone.rp43-41g5.GenBank:AC099533.36    GAAGTGGTGAACGTGGGCATCTTTGTCTTGTTCCCTTCCTCAGGGGGAATGCTTTCAACT 24523
human.FAM30B-LOC105372935-GGT2.NCBI.                                              gaagcggtgaacgtgggcatctttgtcttgttcccttcctcaggcggaatgctttcaact 21433
human.FAM230E-LOC105377182-GGT3P.NCBI.                                            gaagtggtgaacgtgggcatctttgtcttgttcccttcctcagggggaatgctttcaact 20170
human.FAM230J-LOC105372942-GGTLC5P.NCBI.rev.compl                                 gaagcggtgaacgtgggcatctttgtcttgttcccttcctcagggggaatgctttcaact 20883
human.FAM230A.ncRNA.18487127.18500594-GGTLC3.18516335.18518165.rev.compl.REF      gaagcggtgaacgtgggcatctttgtcttgttcccttcctcagggggaatgctttcaact 9647
                                                                                  **** ****************************** ******** ***************

chimp.LOC112206744-LOC107973052-GGT2.revcompl                                     ttcccccattcaggaaactgttggctgtgggtttgtcatagatagcttttattaccttaa 22801
chimp54546-135457.revcompl.Pan.troglodytes.clone.rp43-41g5.GenBank:AC099533.36    CTCCCCCATTCAGGAAAATGTTGGCTGTGGGTTTGTCATAGATAGCTTTTATTACCTTAA 24583
human.FAM30B-LOC105372935-GGT2.NCBI.                                              ctcccccattcaggaaaatgttggctgtgggtttgtcatagatagcttttattaccttaa 21493
human.FAM230E-LOC105377182-GGT3P.NCBI.                                            ctcccccattcaggaaaatgttggctgtgggtttgtcatagatagcttttattaccttaa 20230
human.FAM230J-LOC105372942-GGTLC5P.NCBI.rev.compl                                 ctcccccattcaggaaaatgttggctgtgggtttgtcatagatagcttttattaccttaa 20943
human.FAM230A.ncRNA.18487127.18500594-GGTLC3.18516335.18518165.rev.compl.REF      ctcccccattcaggaaaatgttggctgtgggtttgtcatagatagcttttattaccttaa 9707
                                                                                   ************** ********************************************

chimp.LOC112206744-LOC107973052-GGT2.revcompl                                     ggtatgtccattctatgctgattttgatgaacggttttaatcataaagaaatgctggatt 22861
chimp54546-135457.revcompl.Pan.troglodytes.clone.rp43-41g5.GenBank:AC099533.36    GGTATGTCCGTTCTATGCTGATTTTGATGAACGGTTTTAATCGTAAAGAAATGCTGGATT 24643
human.FAM30B-LOC105372935-GGT2.NCBI.                                              ggtatgtccgttctacgctgattttgatgaacggttttaatcataaagaaatgctggatt 21553
human.FAM230E-LOC105377182-GGT3P.NCBI.                                            ggtatgtccgttctatgctgattttgacgaacggttttaatcataaagaaatgctggatt 20290
human.FAM230J-LOC105372942-GGTLC5P.NCBI.rev.compl                                 ggtatgtccgttctatgctgattgtgacgaacggttttaatcataaagaaatgctggatt 21003
human.FAM230A.ncRNA.18487127.18500594-GGTLC3.18516335.18518165.rev.compl.REF      ggtatgtccgttctatgctgattttgacgaacggttttaatcataaagaaatgctggatt 9767
                                                                                  ********* ***** ******* *** ************** *****************

chimp.LOC112206744-LOC107973052-GGT2.revcompl                                     ttgtcaaaggctttttctgcatctattcagattatcatgtgatttttgtttttagtttta 22921
```

```
chimp54546-135457.revcompl.Pan.troglodytes.clone.rp43-41g5.GenBank:AC099533.36      TTGTCAAAGGCTTTTTCTGCATCTATTCAGATTATCATGTGATTTTTGTTTTTAGTTTTA 24703
human.FAM30B-LOC105372935-GGT2.NCBI.                                                ttgtcaaaggctttttctgcatctattcagattatcatgtgatttttgtttttagtttta 21613
human.FAM230E-LOC105377182-GGT3P.NCBI.                                              ttgtcaaaggctttttctgcatctattcagattatcatgtgatttttgtttttagtttta 20350
human.FAM230J-LOC105372942-GGTLC5P.NCBI.rev.compl                                   ttgtcaaaggctttttctgcatctattcagattatcatgtgatttttgtttttagtttta 21063
human.FAM230A.ncRNA.18487127.18500594-GGTLC3.18516335.18518165.rev.compl.REF        ttgtcaaaggctttttctgcatctattcagattatcatgtgatttttgtttttagtttta 9827
                                                                                    ************************************************************

chimp.LOC112206744-LOC107973052-GGT2.revcompl                                       tttatgtgatgtatcacatttattgacttgcgtatgttaaaccatccctgcatccctagt 22981
chimp54546-135457.revcompl.Pan.troglodytes.clone.rp43-41g5.GenBank:AC099533.36      TTTATGTGATGTATCACATTTATTGACTTGCGTATGTTAAACCATCCCTGCATCCCTAGT 24763
human.FAM30B-LOC105372935-GGT2.NCBI.                                                ttgatgtgatgtatcacatttattgacttgcgtatgttaaaccatccctgcatccctagt 21673
human.FAM230E-LOC105377182-GGT3P.NCBI.                                              ttgatgtgatgtatcacatttattgacttgcgtatgttaaaccatccctgcatccctagt 20410
human.FAM230J-LOC105372942-GGTLC5P.NCBI.rev.compl                                   ttgatgtgatgtatcacatttattgacttgcgtatgttaaaccatccctgcatccctagt 21123
human.FAM230A.ncRNA.18487127.18500594-GGTLC3.18516335.18518165.rev.compl.REF        ttgatgtgatgtatcacatttattgacttgcgtatgttaaaccatccctgcatccctagt 9887
                                                                                    ** *********************************************************

chimp.LOC112206744-LOC107973052-GGT2.revcompl                                       atgaaacccacttgaatcatggtggattatctttttgatatgctgttggattcagttagc 23041
chimp54546-135457.revcompl.Pan.troglodytes.clone.rp43-41g5.GenBank:AC099533.36      ATGAAACCCACTTGAATCATGGTGGATTATCTTTTTGATATGCTGTTGGATTCAGTTAGC 24823
human.FAM30B-LOC105372935-GGT2.NCBI.                                                atgaaacccacttgaatcatggtggattatctttttgatatgctgttggattcagttagc 21733
human.FAM230E-LOC105377182-GGT3P.NCBI.                                              atgaaacccacttgaatcatggtggattatctttttgatatgctgttggattcagttagc 20470
human.FAM230J-LOC105372942-GGTLC5P.NCBI.rev.compl                                   atgaaacccacttgaatcatggtggattatctttttgatatgctgttggattcagttagc 21183
human.FAM230A.ncRNA.18487127.18500594-GGTLC3.18516335.18518165.rev.compl.REF        atgaaacccacttgaatcatggtggattatctttttgatatgctgttggattcagttagc 9947
                                                                                    ************************************************************

chimp.LOC112206744-LOC107973052-GGT2.revcompl                                       ttggttgtagcatttctttttattccatctgtggaatgtattggtttaaataatgaaaac 23101
chimp54546-135457.revcompl.Pan.troglodytes.clone.rp43-41g5.GenBank:AC099533.36      TTGGTTGTAGCATTTCTTATTATTCCATCTGTGGAATGTATTGGTTTAAATAATGAAAAC 24883
human.FAM30B-LOC105372935-GGT2.NCBI.                                                ttggttgtagcatttcttattattccatctgtggaatgtattggttgaaataatgaaaac 21793
human.FAM230E-LOC105377182-GGT3P.NCBI.                                              ttggttgtagcatttcttattattccatctgtggaatgtattggttgaaataatgaaaac 20530
human.FAM230J-LOC105372942-GGTLC5P.NCBI.rev.compl                                   ttggttgtagcatttcttattattccatctgtggaatgtattggttgaaataatgaaaac 21243
human.FAM230A.ncRNA.18487127.18500594-GGTLC3.18516335.18518165.rev.compl.REF        ttggttgtagcatttcttattattccatctgtggaatgtattggttgaaataatgaaaac 10007
                                                                                    ****************** *************************** *************

chimp.LOC112206744-LOC107973052-GGT2.revcompl                                       atgttctatcctcactgcttagcactttgtgtttctttaatagccttcccaacagggcaa 23161
chimp54546-135457.revcompl.Pan.troglodytes.clone.rp43-41g5.GenBank:AC099533.36      ATGTTCTATCCTCACTGCTTAGCACTTTGTGTTTCTTTAATAGCCTTCCCAACAGGGCAA 24943
human.FAM30B-LOC105372935-GGT2.NCBI.                                                atgttctatcctcactgcttagcactttgtgtttctttaatagccttcccaacagggcaa 21853
human.FAM230E-LOC105377182-GGT3P.NCBI.                                              atgttctatcctcactgcttagcactttgtgtttctttaatagccttcccaacagggcaa 20590
human.FAM230J-LOC105372942-GGTLC5P.NCBI.rev.compl                                   atgttctatcctcactgcttagcactttgtgtttctttaatagccttcccaacagggcaa 21303
human.FAM230A.ncRNA.18487127.18500594-GGTLC3.18516335.18518165.rev.compl.REF        atgttctatcctcactgcttagcactttgtgtttctttaatagccttcccaacagggcaa 10067
                                                                                    ************************************************************

chimp.LOC112206744-LOC107973052-GGT2.revcompl                                       cataaaagcaggagccctgctagtcaccccttaacccggaatcccccccttctccacagct 23221
chimp54546-135457.revcompl.Pan.troglodytes.clone.rp43-41g5.GenBank:AC099533.36      CATAAAAGCAGGAGCCCTGCTAGTCACCCCTTAACCCGGAATCCCCCCTTCTCCACAGCT 25003
human.FAM30B-LOC105372935-GGT2.NCBI.                                                cataaaagcaggagccctgctagtctccccttaacccggaatccccccttctccacagct 21913
human.FAM230E-LOC105377182-GGT3P.NCBI.                                              cataaaagcaggagccctgctagtctccccttaacccggaatccccccttctccacagct 20650
human.FAM230J-LOC105372942-GGTLC5P.NCBI.rev.compl                                   cataaaagcaggagccctgctagtctccccttaacccggaatccccccttctccacagct 21363
human.FAM230A.ncRNA.18487127.18500594-GGTLC3.18516335.18518165.rev.compl.REF        cataaaagcaggagccctgctagtctccccttaacccggaatccccccttctccacagct 10127
                                                                                    ************************* **********************************

chimp.LOC112206744-LOC107973052-GGT2.revcompl                                       cgcttattggacaggatagactgggagcccaggcctcaaggtaaggacgtgctctgtcac 23281
chimp54546-135457.revcompl.Pan.troglodytes.clone.rp43-41g5.GenBank:AC099533.36      CGCTCATTGGACAGGATAGACTGGGCGCCCAGGCTTCAAGGTAAGGACGTGCTCTGTCAC 25063
human.FAM30B-LOC105372935-GGT2.NCBI.                                                cgctcattggacaggatagactgggcgcccaggcttcaaggtaaggacgtgctctgtcac 21973
human.FAM230E-LOC105377182-GGT3P.NCBI.                                              cgctcattggacaggatagactgggcgcccaggcttcaaggtaaggacatgctctgtcac 20710
human.FAM230J-LOC105372942-GGTLC5P.NCBI.rev.compl                                   cgctcattggacaggatagactgggcgcccaggcttcaaggtaaggacgtgctctgtcac 21423
human.FAM230A.ncRNA.18487127.18500594-GGTLC3.18516335.18518165.rev.compl.REF        cgctcattggacaggatagactgggcgcccaggcttcaaggtaaggacgtgctctgtcac 10187
                                                                                    **** ******************** ******** ************* ***********

chimp.LOC112206744-LOC107973052-GGT2.revcompl                                       ctagaggtgcagtgcttgggaaggccaaccttggagggttgcctgccagctttacagtga 23341
chimp54546-135457.revcompl.Pan.troglodytes.clone.rp43-41g5.GenBank:AC099533.36      CTAGAGGTGCAGTGCTTGGGAAGGCCAACCTTGGAGGGTTGCCTGCCAGCTTTACAGTGA 25123
human.FAM30B-LOC105372935-GGT2.NCBI.                                                ctagaggtgcagtgcttgggaaggccaaccttggagggttgcctgccagctttacagtga 22033
human.FAM230E-LOC105377182-GGT3P.NCBI.                                              ctagaggtgcagtgcttgggaaggccaaccttggagggttgcctgccagctttacagtga 20770
human.FAM230J-LOC105372942-GGTLC5P.NCBI.rev.compl                                   ctagaggtgcagtgcttgggaaggccaaccttggagggttgcctgccagctttacagtga 21483
human.FAM230A.ncRNA.18487127.18500594-GGTLC3.18516335.18518165.rev.compl.REF        ctagaggtgcagtgcttgggaaggccaaccttggagggttgcctgccagctttacagtga 10247
                                                                                    ************************************************************

chimp.LOC112206744-LOC107973052-GGT2.revcompl                                       cagaggtgttgggagggactgaccaccagtgcataaggctgtgctttgttggtgacataa 23401
chimp54546-135457.revcompl.Pan.troglodytes.clone.rp43-41g5.GenBank:AC099533.36      CAGAGGTGTTGGGAGGGACTGACCACCAGTGCATAAGGCTGTGCTTTGTTGGTGACATAA 25183
human.FAM30B-LOC105372935-GGT2.NCBI.                                                cagaggtgttgagagggactgaccaccagtgcataaggctgtgctttgttggtgacataa 22093
human.FAM230E-LOC105377182-GGT3P.NCBI.                                              cagaggtgttgagagggactgaccaccagtgcagaaggctgtgctttgttggtgacataa 20830
human.FAM230J-LOC105372942-GGTLC5P.NCBI.rev.compl                                   cagaggtgttgagagggactgaccaccagtgcagaaggctgtgctttgttggtgacataa 21543
human.FAM230A.ncRNA.18487127.18500594-GGTLC3.18516335.18518165.rev.compl.REF        cagaggtgttgagagggactgaccaccagtgcataaggctgtgctttgttggtgacataa 10307
                                                                                    *********** ********************* **************************

chimp.LOC112206744-LOC107973052-GGT2.revcompl                                       aggattgtttcacaggttgttggggagggacaatcccaaggcctcccctggccctggtgc 23461
chimp54546-135457.revcompl.Pan.troglodytes.clone.rp43-41g5.GenBank:AC099533.36      AGGATTGTTTCACAGGTTGTTGGGGAGGGACAATCCCAAGGCCTCCCCTGGCCCTGGTGC 25243
human.FAM30B-LOC105372935-GGT2.NCBI.                                                aggattgtttcacagattgttggggagggacaatcccaaggcctcccctggccctggtgc 22153
human.FAM230E-LOC105377182-GGT3P.NCBI.                                              aggattgtttcacagattgttggggagggacaatcccaaggcctcccctggccctggtgc 20890
human.FAM230J-LOC105372942-GGTLC5P.NCBI.rev.compl                                   aggattgtttcacagattgttggggagggacaatcccaaggcctcccctggccctggtgc 21603
human.FAM230A.ncRNA.18487127.18500594-GGTLC3.18516335.18518165.rev.compl.REF        aggattgtttcacagattgttggggagggacaatcccaaggcctcccctggccctggtgc 10367
                                                                                    ************** *********************************************

chimp.LOC112206744-LOC107973052-GGT2.revcompl                                       tggctctgcacaaaggcaataagagagggatgctggtaagggctgacctgttgctgtgct 23521
chimp54546-135457.revcompl.Pan.troglodytes.clone.rp43-41g5.GenBank:AC099533.36      TGGCTCTGCACAAAGGCAATAAGAGAGGGATGCTGGTAAGGGCTGACCTGTTGCTGTGCT 25303
human.FAM30B-LOC105372935-GGT2.NCBI.                                                tggctctgcacaaaggcaataagagagggatgctggtaagggctgacctgttgctgtgct 22213
human.FAM230E-LOC105377182-GGT3P.NCBI.                                              tggctctgcacaaaggcaataagagagggatgctggtaagggctgacctgttgctgtgct 20950
human.FAM230J-LOC105372942-GGTLC5P.NCBI.rev.compl                                   tggctctgcacaaaggcaataagagagggatgctggtaagggctgacctgttgctgtgct 21663
human.FAM230A.ncRNA.18487127.18500594-GGTLC3.18516335.18518165.rev.compl.REF        tggctctgcacaaaggcaataagagagggatgctggtaagggctgacctgttgctgtgct 10427
                                                                                    ************************************************************

chimp.LOC112206744-LOC107973052-GGT2.revcompl                                       ggggaggaaggtgctgggctgaaattcaggaggctgaggatgcagcagtcccataggagg 23581
chimp54546-135457.revcompl.Pan.troglodytes.clone.rp43-41g5.GenBank:AC099533.36      GGGGAGGAAGGTGCTGGGCTGAAATTCAGGAGGCTGAGGATGCAGCAATCCCATAGGAGG 25363
human.FAM30B-LOC105372935-GGT2.NCBI.                                                ggggaggaaggtgctgggctgaaattcaggaggctgaggatgcagcagtcccataggagg 22273
human.FAM230E-LOC105377182-GGT3P.NCBI.                                              ggggaggaaggtgctgggctgaaattcaggaggctgaggatgcagcagtcccataggagg 21010
human.FAM230J-LOC105372942-GGTLC5P.NCBI.rev.compl                                   ggggaggaaggtgctgggctgaaattcaggaggctgaggatgcagcagtcccataggagg 21723
human.FAM230A.ncRNA.18487127.18500594-GGTLC3.18516335.18518165.rev.compl.REF        ggggaggaaggtgctgggctgaaattcaggaggctgaggatgcagcagtcccataggagg 10487
                                                                                    *********************************************** ************

chimp.LOC112206744-LOC107973052-GGT2.revcompl                                       tacgtgaccttcaggatacatttcttcattgatgatcaatggaaatgagaaatcactga 23641
chimp54546-135457.revcompl.Pan.troglodytes.clone.rp43-41g5.GenBank:AC099533.36      TACGTGACCTTCAGGGTACATTTTCTTCATTGATGATCAATGGAAATGAGAAATCACTGA 25423
human.FAM30B-LOC105372935-GGT2.NCBI.                                                tacatgaccttcaggatacattttcttcattgatgatcaatggaaatgagaaatcactga 22333
human.FAM230E-LOC105377182-GGT3P.NCBI.                                              tacatgaccttcaggatacattttcttcattgatgatcaatggaaatgagaaatcactga 21070
human.FAM230J-LOC105372942-GGTLC5P.NCBI.rev.compl                                   tacatgaccttcaggatacattttcttcattgatgatcaatggaaatgagaaatcactga 21783
human.FAM230A.ncRNA.18487127.18500594-GGTLC3.18516335.18518165.rev.compl.REF        tacatgaccttcaggatacattttcttcattgatgatcaatggaaatgagaaatcactga 10547
                                                                                    *** *********** ********************************************

chimp.LOC112206744-LOC107973052-GGT2.revcompl                                       ctatttttctatcattggaatctactctctactgctcgtgctgttcctgtcttttgggg 23701
chimp54546-135457.revcompl.Pan.troglodytes.clone.rp43-41g5.GenBank:AC099533.36      CTATTTTTTCTATCATTGGAATCTACTCTCTACTGCTCATGCTGTTCCTGTCTTTTGGGG 25483
human.FAM30B-LOC105372935-GGT2.NCBI.                                                ctattttttctatcattggaatctactctccactgctcatgctgttcctgtcttttgggg 22393
human.FAM230E-LOC105377182-GGT3P.NCBI.                                              ctattttttctatcattggaatctactctccactgctcatgctgttcctgtcttttgggg 21130
human.FAM230J-LOC105372942-GGTLC5P.NCBI.rev.compl                                   ctattttttctatcattggaatctactctccactgctcatgctgttcctgtcttttgggg 21843
human.FAM230A.ncRNA.18487127.18500594-GGTLC3.18516335.18518165.rev.compl.REF        ctattttttctatcattggaatctactctccactgctcatgctgttcctgtcttttgggg 10607
                                                                                    ****************************** ******* *********************

chimp.LOC112206744-LOC107973052-GGT2.revcompl                                       aagatggaggatcaatcagtgtgcgctgcactgagtggaaggaaggagaactgcgacaaa 23761
chimp54546-135457.revcompl.Pan.troglodytes.clone.rp43-41g5.GenBank:AC099533.36      AAGATGGAGGATCAATCAGTGTGCGCTGCACTGAGTGGAAGGAAGGAGAACTGTGACAAA 25543
human.FAM30B-LOC105372935-GGT2.NCBI.                                                aagatggaggatcaatcagtgtgcactgcactgagtggaaggaaggagaactgtgacaaa 22453
human.FAM230E-LOC105377182-GGT3P.NCBI.                                              aagatggaggatcaatcagtgtgcgctgccctgagtggaaggaaggagaactgtgacaaa 21190
human.FAM230J-LOC105372942-GGTLC5P.NCBI.rev.compl                                   aagatggaggatcaatcagtgtgcgctgcactgagtggaaggaaggagaactgtgacaaa 21903
human.FAM230A.ncRNA.18487127.18500594-GGTLC3.18516335.18518165.rev.compl.REF        aagatggaggatcaatcagtgtgcgctgcactgagtggaaggaaggagaactgtgacaaa 10667
                                                                                    ************************ **** *********************** ******

chimp.LOC112206744-LOC107973052-GGT2.revcompl                                       aattaaggaaggatgagagacgggagggcccttcatccagctgcttgcagagtcctcctg 23821
chimp54546-135457.revcompl.Pan.troglodytes.clone.rp43-41g5.GenBank:AC099533.36      AATTAAGGAAGGATGAGAGACGGGAGGGCCCTTCATCCAGCTGCTTGCAGAGTCCTCCTG 25603
human.FAM30B-LOC105372935-GGT2.NCBI.                                                aattaaggaaggatgagagacgggagggcccttcatccagctgcttgcagagtcctcctg 22513
human.FAM230E-LOC105377182-GGT3P.NCBI.                                              aattaaggaaggatgagagacgggagggcccttcatccagctgcttgcagagtcctcctg 21250
human.FAM230J-LOC105372942-GGTLC5P.NCBI.rev.compl                                   aattaaggaaggatgagagacgggagggcccttcatccagctgcttgcagagtcctcctg 21963
human.FAM230A.ncRNA.18487127.18500594-GGTLC3.18516335.18518165.rev.compl.REF        aattaaggaaggatgagagacgggagggcccttcatccagctgcttgcagagtcctcctg 10727
                                                                                    ************************************************************

chimp.LOC112206744-LOC107973052-GGT2.revcompl                                       aggaggaaagccccgtggctccctggcgaaggagcagtgagggctgcgtgactcccacag 23881
chimp54546-135457.revcompl.Pan.troglodytes.clone.rp43-41g5.GenBank:AC099533.36      AGGAGGAAAGCCCCGTGGCTCCCTGGCGAAGGAGCAGTGAGGGCTGCGTGACTCCCACAG 25663
human.FAM30B-LOC105372935-GGT2.NCBI.                                                aggaggaaagccccgtggctccctggagaaggagcattgagggctgcgtgactcccacag 22573
human.FAM230E-LOC105377182-GGT3P.NCBI.                                              aggaggaaagccccgtggctccctggcgaaggagcagtgagggctgcgtgactcccacag 21310
human.FAM230J-LOC105372942-GGTLC5P.NCBI.rev.compl                                   aggaggaaagccccgtggctccctggcgaaggagcagtgagggctgcgtgactcccacag 22023
human.FAM230A.ncRNA.18487127.18500594-GGTLC3.18516335.18518165.rev.compl.REF        aggaggaaagccccgtggctccctggcgaaggagcagtgagggctgcgtgactcccacag 10787
                                                                                    ************************** ********* ***********************

chimp.LOC112206744-LOC107973052-GGT2.revcompl                                       tgaagtgtgtggtatgtctgaggacacccaggctggtggtccatgaggagccagtggcag 23941
chimp54546-135457.revcompl.Pan.troglodytes.clone.rp43-41g5.GenBank:AC099533.36      TGAAGTGTGTGGTATGTCTGAGGACACCCAGGCTGGTGGTCCATGAGGAGCCAGTGGCAG 25723
human.FAM30B-LOC105372935-GGT2.NCBI.                                                tgaagtgtgtggtatgtctgaggacacccaggctggtggtccatgaggagccagtggcag 22633
human.FAM230E-LOC105377182-GGT3P.NCBI.                                              tgaagtgtgtggtatgtctgaggacacccaggctggtggtccatgaggagccagtggcag 21370
human.FAM230J-LOC105372942-GGTLC5P.NCBI.rev.compl                                   tgaagtgtgtggtatgtctgaggacacccaggctggtggtccatgaggagccagtggcag 22083
human.FAM230A.ncRNA.18487127.18500594-GGTLC3.18516335.18518165.rev.compl.REF        tgaagtgtgtggtatgtctgaggacacccaggctggtggtccatgaggagccagtggcag 10847
                                                                                    ************************************************************

chimp.LOC112206744-LOC107973052-GGT2.revcompl                                       agtgagaagcagaaaggccaggagggtggctggaggccaggctctgagtcattctccatg 24001
chimp54546-135457.revcompl.Pan.troglodytes.clone.rp43-41g5.GenBank:AC099533.36      AGTGAGAAGCAGAAAGGCCAGGAGGGTGGCTGGAGGCCAGGCTCTGAGTCATTCTCCATG 25783
```

```
human.FAM30B-LOC105372935-GGT2.NCBI.                                               agtgagaagaagaaaggccaggagggtggctggaggccaggctctgagtcattctccatg 22693
human.FAM230E-LOC105377182-GGT3P.NCBI.                                             agtgagaagcagaaaggccaggagggtggctggaggccaggctctgagtcattctccatg 21430
human.FAM230J-LOC105372942-GGTLC5P.NCBI.rev.compl                                  agtgagaagcagaaaggccaggagggtggctggaggccaggctctgagtcattctccatg 22143
human.FAM230A.ncRNA.18487127.18500594-GGTLC3.18516335.18518165.rev.compl.REF       agtgagaagaagaaaggccaggagggtggctggaggccaggctctgagtcattctccatg 10907
                                                                                   ********* **************************************************

chimp.LOC112206744-LOC107973052-GGT2.revcompl                                      tgatggaaacagccggagcccagtgggcttggaggtacaggatgcggtggctgatgacag 24061
chimp54546-135457.revcompl.Pan.troglodytes.clone.rp43-41g5.GenBank:AC099533.36     TGATGGAAACAGCCGGAGCCCAGTGGGCTTGGAGGTACAGGATGCAGTGGCTGATGACAG 25843
human.FAM30B-LOC105372935-GGT2.NCBI.                                               tgatggaaacagccggagcccagtgggcttggaggtacaggatgcggtggctgatgacag 22753
human.FAM230E-LOC105377182-GGT3P.NCBI.                                             tgatggaaacagccggagcccagtgggcttggaggtacaggatgcggtggctgatgacag 21490
human.FAM230J-LOC105372942-GGTLC5P.NCBI.rev.compl                                  tgatggaaacagccggagcccagtggacttggaggtacaggatgcggtggctgatgacag 22203
human.FAM230A.ncRNA.18487127.18500594-GGTLC3.18516335.18518165.rev.compl.REF       tgatggaaacagccggagcccagtgggcttggaggtacaggatgcggtggctgatgacag 10967
                                                                                   ************************** ****************** **************

chimp.LOC112206744-LOC107973052-GGT2.revcompl                                      aacaatgtggagagaggcgtcatttgtcaaatccttactttgttctgggcattgtgctaa 24121
chimp54546-135457.revcompl.Pan.troglodytes.clone.rp43-41g5.GenBank:AC099533.36     AACAATGTGGAGAGAGGCGTCATTTGTCAAATCCTTACTTTGTTCTGGGCATTGTGCTAA 25903
human.FAM30B-LOC105372935-GGT2.NCBI.                                               aacaatgtggagagaggcgtcatttgtcaaatccttactttgttctgggcattgtgctaa 22813
human.FAM230E-LOC105377182-GGT3P.NCBI.                                             aacaatgtggagagaggtgtcatttgtcaaatccttactttgttctgggcattgtgctaa 21550
human.FAM230J-LOC105372942-GGTLC5P.NCBI.rev.compl                                  aacaatgtggagagaggcgtcatttgtcaaatccttactttgttctgggcattgtgctaa 22263
human.FAM230A.ncRNA.18487127.18500594-GGTLC3.18516335.18518165.rev.compl.REF       aacaatgtggagagaggcgtcatttgtcaaatccttactttgttctgggcattgtactaa 11027
                                                                                   ***************** ************************************** ****

chimp.LOC112206744-LOC107973052-GGT2.revcompl                                      aaattctgatggctcatcccatttaggggctgaaagttgcagaggtttaggaagctcacc 24181
chimp54546-135457.revcompl.Pan.troglodytes.clone.rp43-41g5.GenBank:AC099533.36     A------CGTGGCTCATCCCATTTAGGGGCTGAAAGTTGCAGAGGTTTAGGAAGCTCACC 25957
human.FAM30B-LOC105372935-GGT2.NCBI.                                               aaattctgatggctcatcccatttaggggctgaaagttgcagaggtttaggaagctcacc 22873
human.FAM230E-LOC105377182-GGT3P.NCBI.                                             aaattctgatggctcatcccatttaggggctgaaagttgcagaggtttaggaagctcacc 21610
human.FAM230J-LOC105372942-GGTLC5P.NCBI.rev.compl                                  aaattctgatggctcatcccatttaggggctgaaagttgcagaggtttaggaagctcacc 22323
human.FAM230A.ncRNA.18487127.18500594-GGTLC3.18516335.18518165.rev.compl.REF       aaattctaatggctcatcccatttaggggctgaaagttgcagaggtttaggaagctcacc 11087
                                                                                   *       ****************************************************

chimp.LOC112206744-LOC107973052-GGT2.revcompl                                      catgatactggagcccccatctcctgccctagtgccgtccaccttctcacccagccacca 24241
chimp54546-135457.revcompl.Pan.troglodytes.clone.rp43-41g5.GenBank:AC099533.36     CACGATACTGGAGCCCCCATCTCCTGCCCTAGTGCCGTCCACCTTCTCACCCAGCCACCA 26017
human.FAM30B-LOC105372935-GGT2.NCBI.                                               cacgatactggagcccccatctcctgccctagtgctgtccaccttctcacccagccacca 22933
human.FAM230E-LOC105377182-GGT3P.NCBI.                                             cacgatactggagcccccatctcctgccctagtgctgtccaccttctcacccagccacca 21670
human.FAM230J-LOC105372942-GGTLC5P.NCBI.rev.compl                                  cacgatactggagcccccatctcctgccctagtgctgtccaccttctcacccagccacca 22383
human.FAM230A.ncRNA.18487127.18500594-GGTLC3.18516335.18518165.rev.compl.REF       cacgatactggagcccccatctcttgccctagtgctgtccaccttctcacccagccacca 11147
                                                                                   ** ******************** *********** ************************

chimp.LOC112206744-LOC107973052-GGT2.revcompl                                      cctgtttcaggggaacacacagaagtggtaacctcttatggagaggcaagtaaattctgc 24301
chimp54546-135457.revcompl.Pan.troglodytes.clone.rp43-41g5.GenBank:AC099533.36     CCTATTTCAGGGGAACACACAGAAGTGGTAACCTCTTATGGAGAGGCAAGTAAATTCTGC 26077
human.FAM30B-LOC105372935-GGT2.NCBI.                                               cctgtttcggggaacacacagaagtggtaacctcttatggagaggcaagtaaattctgc 22993
human.FAM230E-LOC105377182-GGT3P.NCBI.                                             cctgtttcggggaacacacagaagtggtaacctcttatggagaggcaagtaaattctgc 21730
human.FAM230J-LOC105372942-GGTLC5P.NCBI.rev.compl                                  cctgtttcggggaacacacagaagtggtaacctcttatggtgaggcaagtaaattctgc 22443
human.FAM230A.ncRNA.18487127.18500594-GGTLC3.18516335.18518165.rev.compl.REF       cctgtttcggggaacacacagaagtggtaacctcttatggagaggcaagtaaattctgc 11207
                                                                                   *** **** ******************************* *******************

chimp.LOC112206744-LOC107973052-GGT2.revcompl                                      tgtttttgttattcacagaaaaacactggctcatgtgggttgggaaggtgaaataccaga 24361
chimp54546-135457.revcompl.Pan.troglodytes.clone.rp43-41g5.GenBank:AC099533.36     TGTTTTTGTTATTCACAGAAAAACACTGGCTCTTGTGGGTTGGGAAGGTGAAATACCAGA 26137
human.FAM30B-LOC105372935-GGT2.NCBI.                                               tgtttttgttattcacagaaaaacactggctcgtgtgggttgggaaggtgaaataccaga 23053
human.FAM230E-LOC105377182-GGT3P.NCBI.                                             tgtttttgttattcacagaaaaacactggctcgtgtgggttgggaaggtgaaataccaga 21790
human.FAM230J-LOC105372942-GGTLC5P.NCBI.rev.compl                                  tgtttttgttattcacagaaaaacactggctcgtgtgggttgggaaggtgaaataccaga 22503
human.FAM230A.ncRNA.18487127.18500594-GGTLC3.18516335.18518165.rev.compl.REF       tgtttttgttattcacagaaaaacactggctcgtgtgggttgggaaggtgaaataccaga 11267
                                                                                   ******************************** ***************************

chimp.LOC112206744-LOC107973052-GGT2.revcompl                                      agtatttcatctagttatttctacccatgcaactcctatagtattgaaatgcataggtta 24421
chimp54546-135457.revcompl.Pan.troglodytes.clone.rp43-41g5.GenBank:AC099533.36     AGTATTTCATCTAGTTATTTCTACCCATGCAACTCCTATAGTATTGAAATGCATAGGTTA 26197
human.FAM30B-LOC105372935-GGT2.NCBI.                                               agtatttcatctggttatttctacccatgcgactcctatagtattgaaatgcataggtta 23113
human.FAM230E-LOC105377182-GGT3P.NCBI.                                             agtatttcatctggttatttctacccatgcgactcctatagtattgaaatgcataggtta 21850
human.FAM230J-LOC105372942-GGTLC5P.NCBI.rev.compl                                  agtatttcatctggttatttctacccatgcgactcctatagtattgaaatgcataggtta 22563
human.FAM230A.ncRNA.18487127.18500594-GGTLC3.18516335.18518165.rev.compl.REF       agtatttcatctggttatttctacccatgcaactcctatagtattgaaatgcataggtta 11327
                                                                                   ************ ***************** *****************************

chimp.LOC112206744-LOC107973052-GGT2.revcompl                                      gcatttttggccaatttactcagcattctgggttaaaggct-------------tttatt 24468
chimp54546-135457.revcompl.Pan.troglodytes.clone.rp43-41g5.GenBank:AC099533.36     GCATTTTTGGCCAATTTACTCAGCATTCTGGGTTAAAGGCT-------------TTTATT 26244
human.FAM30B-LOC105372935-GGT2.NCBI.                                               gcatttttggccaatttactcagcattctgggttaaaggcttttatttattttatttatt 23173
human.FAM230E-LOC105377182-GGT3P.NCBI.                                             gcatttttggccaatttactcagcattctgggttaaaggcttttatttat----tttatt 21906
human.FAM230J-LOC105372942-GGTLC5P.NCBI.rev.compl                                  gcatttttggccaatttactcagcattctgggttaaaggcttttatttattttatttatt 22623
human.FAM230A.ncRNA.18487127.18500594-GGTLC3.18516335.18518165.rev.compl.REF       gcatttttggccaatttactcagcattctgggttaaaggcttttatttat----tttatt 11383
                                                                                   *****************************************             ******

chimp.LOC112206744-LOC107973052-GGT2.revcompl                                      tatttatttatttattttcgagatggagtttcgctcttgttgcccaagctggagtgcaat 24528
chimp54546-135457.revcompl.Pan.troglodytes.clone.rp43-41g5.GenBank:AC099533.36     TATTTATTTATTTATTTTTGAGATGGAGTTTCGCTCTTGTTGCCCAAGCTGGAGTGCAAT 26304
human.FAM30B-LOC105372935-GGT2.NCBI.                                               tatttatttatttatttttgagatggagtttcactcttgttgcccaagctggagtgcaat 23233
human.FAM230E-LOC105377182-GGT3P.NCBI.                                             tatttatttatttacttttgagatggagtttcactcttgttgcccaagctggagggcaat 21966
human.FAM230J-LOC105372942-GGTLC5P.NCBI.rev.compl                                  tatttatttatttatttttgagatggagtttcactcttgttgcccaagctggagtgcaat 22683
human.FAM230A.ncRNA.18487127.18500594-GGTLC3.18516335.18518165.rev.compl.REF       tatttatttatttatttttgagatggagtttcactcttgttgcccaagctggagtgcaat 11443
                                                                                   ************** *** ************* ********************* *****

chimp.LOC112206744-LOC107973052-GGT2.revcompl                                      ggtgcgatcctggttcactgcaacctccgcctcccaggttcaaactattctcctgtctca 24588
chimp54546-135457.revcompl.Pan.troglodytes.clone.rp43-41g5.GenBank:AC099533.36     GGTGCGATCCTGGTTCACTGCAACCTCCGCCTCCCAGATTCAAACTATTCTCCTTTCTCA 26364
human.FAM30B-LOC105372935-GGT2.NCBI.                                               ggtgcgatcctggttcattgcaacctccgcctcccaggttcaaactattctcctgtctca 23293
human.FAM230E-LOC105377182-GGT3P.NCBI.                                             ggtgcgatcctggttcattgcaacctccgcctcccaggttcaaactattctcctgtctca 22026
human.FAM230J-LOC105372942-GGTLC5P.NCBI.rev.compl                                  ggtgcgatcctggttcattgcaacctccgcctcccaggttcaaactattctcctgtctca 22743
human.FAM230A.ncRNA.18487127.18500594-GGTLC3.18516335.18518165.rev.compl.REF       ggtgcgatcctggttcattgcaacctccgcctcccaggttcaaactattctcctgtctca 11503
                                                                                   ***************** ****************** **************** *****

chimp.LOC112206744-LOC107973052-GGT2.revcompl                                      gcctcccaagtagctgagattataggcacgtgccaccacactgggctaatttttttgtat 24648
chimp54546-135457.revcompl.Pan.troglodytes.clone.rp43-41g5.GenBank:AC099533.36     GCCTCCCAAGTAGCTGAGATTATAGGCATGTGCCACCACCCTGGGCTAATTTTTTTGTAT 26424
human.FAM30B-LOC105372935-GGT2.NCBI.                                               gcctcccaagtagctgagattataggcacatgccaccacactgggctaatttttttgtat 23353
human.FAM230E-LOC105377182-GGT3P.NCBI.                                             gcctcccaagtagctgagattataggcacatgccaccacactgggctaatttttttgtat 22086
human.FAM230J-LOC105372942-GGTLC5P.NCBI.rev.compl                                  gcctcccaagtagctgagattataggcacatgccaccacactgggctaatttttttgtat 22803
human.FAM230A.ncRNA.18487127.18500594-GGTLC3.18516335.18518165.rev.compl.REF       gcctcccaagtagctgagattataggcacatgccaccacactgggctaatttttttgtat 11563
                                                                                   ****************************  ********* ********************

chimp.LOC112206744-LOC107973052-GGT2.revcompl                                      ttttagtagaaatgagatttcaccatgttggtcaggctggtttcaaaatcctgacctcag 24708
chimp54546-135457.revcompl.Pan.troglodytes.clone.rp43-41g5.GenBank:AC099533.36     TTTTAGTAGAAATGAGATTTCACCATGTTGGTCAGGCTGGTTTCAAACTCCTGACCTCAG 26484
human.FAM30B-LOC105372935-GGT2.NCBI.                                               ttttagtagaaatgagatttcaccatgttggtcaggctggtttcgaactcctgacctcag 23413
human.FAM230E-LOC105377182-GGT3P.NCBI.                                             ttttagtagaaatgagatttcaccatgttggtcaggctggtttcgaactcctgacctcag 22146
human.FAM230J-LOC105372942-GGTLC5P.NCBI.rev.compl                                  ttttagtagaaatgagatttcaccatgttggtcaggctggtttcgaactcctgacctcag 22863
human.FAM230A.ncRNA.18487127.18500594-GGTLC3.18516335.18518165.rev.compl.REF       ttttagtagaaatgagatttcaccatgttggtcaggctggtttcgaactcctgacctcag 11623
                                                                                   ******************************************** ** ************

chimp.LOC112206744-LOC107973052-GGT2.revcompl                                      gtgatctgccctcctcggcctcttaaagtgctgggattacaggcgtgagccaccatgccc 24768
chimp54546-135457.revcompl.Pan.troglodytes.clone.rp43-41g5.GenBank:AC099533.36     GTGATCTGCCCTCCTCGGCCTCTTAAAGTGCTGGAATTACAGGCGTGAGCCACCATGCCC 26544
human.FAM30B-LOC105372935-GGT2.NCBI.                                               gtgatctgcccccctcggcctcttaaagtgctgggattacaggcgtgagccaccacgcct 23473
human.FAM230E-LOC105377182-GGT3P.NCBI.                                             gtgatctgccctcctcggcctcttaaagtgctgggattacaggcgtgagccaccacgcct 22206
human.FAM230J-LOC105372942-GGTLC5P.NCBI.rev.compl                                  gtgatctgccctcctcggcctcttaaagtgctgggattacaggtgtgagccaccacgcct 22923
human.FAM230A.ncRNA.18487127.18500594-GGTLC3.18516335.18518165.rev.compl.REF       gtgatctgccctcctcggcctcttaaagtgctgggattacaggcgtgagccaccacgcct 11683
                                                                                   *********** ********************** ******** *********** ***

chimp.LOC112206744-LOC107973052-GGT2.revcompl                                      accctaaagtcttttaaaattcacttgtataagttgacttagtttctttttttttttctt 24828
chimp54546-135457.revcompl.Pan.troglodytes.clone.rp43-41g5.GenBank:AC099533.36     GCCCTAAAGTCTTTTAAAATTCACTTGTATAAGTT------------------------- 26579
human.FAM30B-LOC105372935-GGT2.NCBI.                                               gccctaaagtcttttaaaattcacttgtataagtt------------------------- 23508
human.FAM230E-LOC105377182-GGT3P.NCBI.                                             gccctaaagtcttttaaaattcacttgtataagtt------------------------- 22241
human.FAM230J-LOC105372942-GGTLC5P.NCBI.rev.compl                                  gccctaaagtcttttaaaattcacttgtataagtt------------------------- 22958
human.FAM230A.ncRNA.18487127.18500594-GGTLC3.18516335.18518165.rev.compl.REF       gccctaaagtcttttaaaattcacttgtataagtt------------------------- 11718
                                                                                    **********************************

chimp.LOC112206744-LOC107973052-GGT2.revcompl                                      tttctttttcttttttttttttttattgatcattcttggacttagtttctttaaccttgt 24888
chimp54546-135457.revcompl.Pan.troglodytes.clone.rp43-41g5.GenBank:AC099533.36     --------------------------------------GACTTAGTTTCTTTAACCTTGT 26602
human.FAM30B-LOC105372935-GGT2.NCBI.                                               --------------------------------------gacttagtttctttaaccttgt 23531
human.FAM230E-LOC105377182-GGT3P.NCBI.                                             --------------------------------------gacttagtttctttaaccttgt 22264
human.FAM230J-LOC105372942-GGTLC5P.NCBI.rev.compl                                  --------------------------------------gacttagtttctttaaccttgt 22981
human.FAM230A.ncRNA.18487127.18500594-GGTLC3.18516335.18518165.rev.compl.REF       --------------------------------------gacttagtttctttaaccttgt 11741
                                                                                                                         **********************

chimp.LOC112206744-LOC107973052-GGT2.revcompl                                      agaaaaat-caaaaatggcaatctgttttatcacacaaataatgtctttttaatggagtg 24947
chimp54546-135457.revcompl.Pan.troglodytes.clone.rp43-41g5.GenBank:AC099533.36     AGAAAAATACAAAAATGGCAATCTCTTTTATCACACAAATAATGTCTTTTTAATGGAGTG 26662
human.FAM30B-LOC105372935-GGT2.NCBI.                                               agaaaaatacaaaaatggcaatctcttttatcacacaaataatgtctttttaatggagtg 23591
human.FAM230E-LOC105377182-GGT3P.NCBI.                                             agaaaaatacaaaaatggcaatctcttttatcacacaaataatgtctttttaatggagtg 22324
human.FAM230J-LOC105372942-GGTLC5P.NCBI.rev.compl                                  agaaaaatacaaaaatagcaatctcttttatcacacaaataatgtctttttaatggagtg 23041
human.FAM230A.ncRNA.18487127.18500594-GGTLC3.18516335.18518165.rev.compl.REF       agaaaaatacaaaaatggcaatctcttttatcacacaaataatgtctttttaatggagtg 11801
                                                                                   ******** ******* ******* ***********************************

chimp.LOC112206744-LOC107973052-GGT2.revcompl                                      a-tttttttctaattgaggtattatgtacttttcatttactaattattgtttacatttga 25006
chimp54546-135457.revcompl.Pan.troglodytes.clone.rp43-41g5.GenBank:AC099533.36     A-TTTTTTTCTAATTGAGGTATTATGTACTTTTCATTTACTAATTATTGTTTACATTTGA 26721
human.FAM30B-LOC105372935-GGT2.NCBI.                                               a-tttttttctaattgaggtattatgtacttttcatttactaattattgtttacatttga 23650
human.FAM230E-LOC105377182-GGT3P.NCBI.                                             attttttttctaattgaggtattatgtacttttcatttactaattattgtttacatttga 22384
human.FAM230J-LOC105372942-GGTLC5P.NCBI.rev.compl                                  a-tttttttctaattgaggtattatgtacttttcatttactaattattgtttacatttga 23100
human.FAM230A.ncRNA.18487127.18500594-GGTLC3.18516335.18518165.rev.compl.REF       a-tttttttctaattgaggtattatgtacttttcatttacaaattattgtttacatttga 11860
                                                                                   * ************************************* ********************

chimp.LOC112206744-LOC107973052-GGT2.revcompl                                      agtgttttatgaattaatatttaattgcatagatgaagattactagttataggcatttta 25066
chimp54546-135457.revcompl.Pan.troglodytes.clone.rp43-41g5.GenBank:AC099533.36     AGTGTTTTATGAATTAATATTTAATTGCATAGATGAAGATTACTAGTTATAGGCATTTTA 26781
human.FAM30B-LOC105372935-GGT2.NCBI.                                               agtgttttatgaattaatatttaattgcatagatgaagattactagttataggcatttta 23710
```

```
human.FAM230E-LOC105377182-GGT3P.NCBI.                                           agtgttttatgaattaatatttaattgcatagatgaagattactagttataggcatttta 22444
human.FAM230J-LOC105372942-GGTLC5P.NCBI.rev.compl                                agtgttttatgaattaatatttaattgcatagatgaagattactagttataggcatttta 23160
human.FAM230A.ncRNA.18487127.18500594-GGTLC3.18516335.18518165.rev.compl.REF     agtgttttatgaattaatatttaattgcatagatgaagattactagttataggcatttta 11920
                                                                                 ************************************************************

chimp.LOC112206744-LOC107973052-GGT2.revcompl                                    gtaaccaatactcattaagcatagcgtggattcatatgacatcaaggagctattttattt 25126
chimp54546-135457.revcompl.Pan.troglodytes.clone.rp43-41g5.GenBank:AC099533.36   CTAACCAATACTCATTAAGCATAGCGTGGATTCATATGACATCAAGGAGCTATTTTATTT 26841
human.FAM30B-LOC105372935-GGT2.NCBI.                                             ctaaccaatactcattaagcatagcgtggattcctatgacatcaaggagctattttattt 23770
human.FAM230E-LOC105377182-GGT3P.NCBI.                                           ctaaccaatactcattaagcatagcgtggattcatatgacatcaaggagctattttattt 22504
human.FAM230J-LOC105372942-GGTLC5P.NCBI.rev.compl                                ctaaccaatactcattaagcatagcgtggattcctatgacatcaaggagctattttattt 23220
human.FAM230A.ncRNA.18487127.18500594-GGTLC3.18516335.18518165.rev.compl.REF     ctaaccaatactcattaagcatagcatggattcatatgacatcaaggagctattttattt 11980
                                                                                  ************************ ******* **************************

chimp.LOC112206744-LOC107973052-GGT2.revcompl                                    ggtaaaacgaaaaagcacaagaatgaacgaacgcaagaactgaaacggtggagacaccta 25186
chimp54546-135457.revcompl.Pan.troglodytes.clone.rp43-41g5.GenBank:AC099533.36   GGTAGAACGAAAAAGCACAAGAATGAACGAACGCAAGAACTGAAACAGTGGAGACACCTA 26901
human.FAM30B-LOC105372935-GGT2.NCBI.                                             ggtaaaacgaaaaagcacaagaatgaacgaacgcaagaactgaaacagtggagacaccta 23830
human.FAM230E-LOC105377182-GGT3P.NCBI.                                           ggtaaaacgaaaaagcacaagaatgaacgaacgcaagaactgaaacagtggagacaccta 22564
human.FAM230J-LOC105372942-GGTLC5P.NCBI.rev.compl                                ggtaaaacgaaaaagcacaagaatgaacgaacgcaagaactgaaacagtggagacaccta 23280
human.FAM230A.ncRNA.18487127.18500594-GGTLC3.18516335.18518165.rev.compl.REF     ggtaaaacgaaaaagcacaagaatgaacgaatgcaagaactgaaacagtggagacaccta 12040
                                                                                 **** ************************** ************** *************

chimp.LOC112206744-LOC107973052-GGT2.revcompl                                    gaatgacttgtctaagatctaaatcattttgttgtcttcccagcgtacttattatcctga 25246
chimp54546-135457.revcompl.Pan.troglodytes.clone.rp43-41g5.GenBank:AC099533.36   GAATGACTTATCTAAGATCTAAATCATTTTGTTGTCTTCCCAGCGTACTTATTATCCTGA 26961
human.FAM30B-LOC105372935-GGT2.NCBI.                                             gaatgacttgtctaagatctaaatcattttgttgtcttcccagcgtacttattatcctga 23890
human.FAM230E-LOC105377182-GGT3P.NCBI.                                           gaatgacttgtctaagatctaaatcattttgttgtcttcccagcgtacttattatcctga 22624
human.FAM230J-LOC105372942-GGTLC5P.NCBI.rev.compl                                gaatgacttgtctaagatctaaatcattttgttgtcttcccagcgtacttattatcctga 23340
human.FAM230A.ncRNA.18487127.18500594-GGTLC3.18516335.18518165.rev.compl.REF     gaatgacttgtctaagatctaaatcattttgttgtcttcccagcatacttattatcctga 12100
                                                                                 ********* ********************************** ***************

chimp.LOC112206744-LOC107973052-GGT2.revcompl                                    tcattgtcatcagcattgtttgggtccttttagcacagatttctcaaaatgggtaactcc 25306
chimp54546-135457.revcompl.Pan.troglodytes.clone.rp43-41g5.GenBank:AC099533.36   TCATTGTCATCAGCATTGTTTGGGTCCTTTTAGCACAGATTTCTCAAAATGGGTAACTCC 27021
human.FAM30B-LOC105372935-GGT2.NCBI.                                             tcattgtcatcagcattgtttgggtccttttagcacagatttctcaaaatgggtaactcc 23950
human.FAM230E-LOC105377182-GGT3P.NCBI.                                           tcattgtcatcagcattgtttgggtccttttagcacagatttctcaaaatgggtaactcc 22684
human.FAM230J-LOC105372942-GGTLC5P.NCBI.rev.compl                                tcattgtcatcagcattgtttgggtccttttagcacagatttctcaaaatgggtaactcc 23400
human.FAM230A.ncRNA.18487127.18500594-GGTLC3.18516335.18518165.rev.compl.REF     tcattgtcatcagcattgtttgggtccttttagcacagatttctcaaaatgggtaactcc 12160
                                                                                 ************************************************************

chimp.LOC112206744-LOC107973052-GGT2.revcompl                                    ataacagttggaagcttacgaattcatataatttgtaagaggtcaatttggaagtaccta 25366
chimp54546-135457.revcompl.Pan.troglodytes.clone.rp43-41g5.GenBank:AC099533.36   ATAACAGTTGGAAGCTTATGAATTCATATAATTTGTAAGAGGTCAATTTGGAAGTACCTA 27081
human.FAM30B-LOC105372935-GGT2.NCBI.                                             ataacagttggaagcttacgaattcatataatttgtaagaggtcaatttgggagtaccta 24010
human.FAM230E-LOC105377182-GGT3P.NCBI.                                           ataacagttggaagcttacgaattcatataatttgtaagaggtcaatttggaagtaccta 22744
human.FAM230J-LOC105372942-GGTLC5P.NCBI.rev.compl                                ataacagttggaagcttacgaattcatataatttgtaagaggtcaatttggaagtaccta 23460
human.FAM230A.ncRNA.18487127.18500594-GGTLC3.18516335.18518165.rev.compl.REF     ataacagttggaagcttacgaattcatataatttgtaagaggtcaatttggaagtaccta 12220
                                                                                 ****************** ******************************** ********

chimp.LOC112206744-LOC107973052-GGT2.revcompl                                    tctattttaaaattccaataacctgggaatttcatcccatgtctagagtcttccatgtaa 25426
chimp54546-135457.revcompl.Pan.troglodytes.clone.rp43-41g5.GenBank:AC099533.36   TGTATTTTAAAATTCCAATAACCTGGGAATTTCATCCCATGTCTAGAGTCTTCTATGTAA 27141
human.FAM30B-LOC105372935-GGT2.NCBI.                                             tctattttaaaattccaataacctgggaatttcatcccatgtctagagtcttttatgtaa 24070
human.FAM230E-LOC105377182-GGT3P.NCBI.                                           tctattttaaaattccaataacctgggaatttcatcccatgtctagagtcttttatgtaa 22804
human.FAM230J-LOC105372942-GGTLC5P.NCBI.rev.compl                                tctattttaaaattccaataacctgggaatttcatcccatgtctagagtcttttatgtaa 23520
human.FAM230A.ncRNA.18487127.18500594-GGTLC3.18516335.18518165.rev.compl.REF     tctattttaaaattccaataacctgggaatttcatcccatgtctagagtcttttatgtaa 12280
                                                                                 * **************************************************  ******

chimp.LOC112206744-LOC107973052-GGT2.revcompl                                    aatatttccacaattaggagaaatatgtgcatggggattttctatgtagcggtgttttga 25486
chimp54546-135457.revcompl.Pan.troglodytes.clone.rp43-41g5.GenBank:AC099533.36   AATATTTCCACAATTAGGAGAAATATGTGCATGGGGATTTTCTATGTAGCGGTGTTTTGA 27201
human.FAM30B-LOC105372935-GGT2.NCBI.                                             aatatttccacaattaggagaaatatgtgcatggggattttctatgtagcggtgttttga 24130
human.FAM230E-LOC105377182-GGT3P.NCBI.                                           aatatttccacaattaggagaaatatgtgcatggggattttctatgtagcggtgttttga 22864
human.FAM230J-LOC105372942-GGTLC5P.NCBI.rev.compl                                aatatttccacaattaggagaaatatgtgcatggggattttctatgtagcggtgttttga 23580
human.FAM230A.ncRNA.18487127.18500594-GGTLC3.18516335.18518165.rev.compl.REF     aatatttccacaattaggagaaatatgtgcatggggattttctatgtagcggtgttttga 12340
                                                                                 ************************************************************

chimp.LOC112206744-LOC107973052-GGT2.revcompl                                    tagaatagaaaaatgggataaaccaaatttccatcacgaaggaaatagtaatatgctgaa 25546
chimp54546-135457.revcompl.Pan.troglodytes.clone.rp43-41g5.GenBank:AC099533.36   TAGAATAGAAAAATGGGATAAACCAAATTTCCATCACGAAGGAAATAGTAATATGCTGAA 27261
human.FAM30B-LOC105372935-GGT2.NCBI.                                             tagaatagaaaattgggataaaccaaatttccatcacgaaggaaatagtaatatgctgaa 24190
human.FAM230E-LOC105377182-GGT3P.NCBI.                                           tagaatagaaaattgggataaaccaaatttccatcacgaaggaaatagtaatatgctgaa 22924
human.FAM230J-LOC105372942-GGTLC5P.NCBI.rev.compl                                tggaatagaaaattgggataaaccaaatttccatcacgaaggaaatagtaatatgctgaa 23640
human.FAM230A.ncRNA.18487127.18500594-GGTLC3.18516335.18518165.rev.compl.REF     tagaatagaaaattgggataaatcaaatttccatcacgaaggaaatagtaatatgctgaa 12400
                                                                                 * ********** ********* *************************************

chimp.LOC112206744-LOC107973052-GGT2.revcompl                                    taataatacagcgaatattatgcaggctttaaacatcaaaaaagagttcaacttctgact 25606
chimp54546-135457.revcompl.Pan.troglodytes.clone.rp43-41g5.GenBank:AC099533.36   TAATAATACAGCGAATATTATGCAGGCTTTAAACATCAAAAAAGAGTTCAACTTCTGACT 27321
human.FAM30B-LOC105372935-GGT2.NCBI.                                             taataatacagcgaatattatgcaggctttaaacatcaaaaaagagttcaacttctgact 24250
human.FAM230E-LOC105377182-GGT3P.NCBI.                                           taataatacagcgaatattatgcaggctttaaacatcaaaaaagagttcaacttctgact 22984
human.FAM230J-LOC105372942-GGTLC5P.NCBI.rev.compl                                taataatacagcgaatattatgcaggctttaaacatcaaaaaagagttcaacttctgact 23700
human.FAM230A.ncRNA.18487127.18500594-GGTLC3.18516335.18518165.rev.compl.REF     taataatacagcgaatattatgcaggctttaaacatcaaaaaagagttcaacttctgact 12460
                                                                                 ************************************************************

chimp.LOC112206744-LOC107973052-GGT2.revcompl                                    tccgatgatggtgttgaagcaggtcactgctggtttacatttgattttcatgtgggaact 25666
chimp54546-135457.revcompl.Pan.troglodytes.clone.rp43-41g5.GenBank:AC099533.36   TCTGATGATGGTGTTGAAGCAGGTCACTGCTGGTTTACATTTGATTTTCATGTGGGAACT 27381
human.FAM30B-LOC105372935-GGT2.NCBI.                                             tccgatgatggtgttgaagcaggtcactgctggtttacatttgattttcatgtgggaact 24310
human.FAM230E-LOC105377182-GGT3P.NCBI.                                           tccgatgatggtgttgaagcaggtcactgctggtttacatttgattttcatgtgggaact 23044
human.FAM230J-LOC105372942-GGTLC5P.NCBI.rev.compl                                tccgatgatggtgttgaagcaggtcactgctggtttacatttgattttcatgtgggaact 23760
human.FAM230A.ncRNA.18487127.18500594-GGTLC3.18516335.18518165.rev.compl.REF     tccgatgatggtgttgaagcaggtcactgctggtttacatttgattttcatgtgggaact 12520
                                                                                 ** *********************************************************

chimp.LOC112206744-LOC107973052-GGT2.revcompl                                    ctggaagtctgccttagtgattttacatgtggctaaattgagctaatgacaagctgttcg 25726
chimp54546-135457.revcompl.Pan.troglodytes.clone.rp43-41g5.GenBank:AC099533.36   CTGGAAGTCTGCCTTAGTGATTTTACATGTGGCTAAATTGAGCTAATGACAAGCTGTTCG 27441
human.FAM30B-LOC105372935-GGT2.NCBI.                                             ctggaagtccgccttagtgattttacatgtggctaaattgagctaatgacaagctgtttg 24370
human.FAM230E-LOC105377182-GGT3P.NCBI.                                           ctggaagtccgccttagtgattttacatgtggctaaattgagctaatgacaagctgttcg 23104
human.FAM230J-LOC105372942-GGTLC5P.NCBI.rev.compl                                ctggaagtccgccttagtgattttacatgtggctaaattgagctaatgacaagctgttcg 23820
human.FAM230A.ncRNA.18487127.18500594-GGTLC3.18516335.18518165.rev.compl.REF     ctggaagtccgccttagtgattttacatgtggctaaattgagctaatgacaagccgttcg 12580
                                                                                 ********* ******************************************** *** *

chimp.LOC112206744-LOC107973052-GGT2.revcompl                                    aagtatggcaaaatggaactttaaaacagtatcttgtcagcaaccaagtggacctgtttc 25786
chimp54546-135457.revcompl.Pan.troglodytes.clone.rp43-41g5.GenBank:AC099533.36   AAGTATGGCAAAATGGAACTTTAAAACAGTATCTTGTCAGCAACCAAGTGGACCTGTTTC 27501
human.FAM30B-LOC105372935-GGT2.NCBI.                                             cagtatggcaaaatggaactttaaaacagtatcttgtcaacaaccaagaggacctgtttc 24430
human.FAM230E-LOC105377182-GGT3P.NCBI.                                           aagtatggcaaaatggaactttaaaacagtatcttgtcaacaaccaagaggacctgtttc 23164
human.FAM230J-LOC105372942-GGTLC5P.NCBI.rev.compl                                aagtatggcaaaatggaactttaaaacagtatcttgtcaacaaccaagaggacctgtttc 23880
human.FAM230A.ncRNA.18487127.18500594-GGTLC3.18516335.18518165.rev.compl.REF     aagtatggcaaaatggaactttaaaacagtatcttgtcaacaaccaagaggacctgtttc 12640
                                                                                  ************************************** ******** ***********

chimp.LOC112206744-LOC107973052-GGT2.revcompl                                    acgtaaagcccatgcattcatctgcctgcccatcattctgtctgtccatcattctgtctg 25846
chimp54546-135457.revcompl.Pan.troglodytes.clone.rp43-41g5.GenBank:AC099533.36   ACGTAAAGCCCATGCATTCATCTGCCTGCCCATCATTCTGTCTGTCCATCATTCTGTCTG 27561
human.FAM30B-LOC105372935-GGT2.NCBI.                                             acataaagcccacgcattcatctgc----------------ctgtccatcattctgtctg 24474
human.FAM230E-LOC105377182-GGT3P.NCBI.                                           acataaagcccacgcattcatctgc----------------ctgtccatcattctgtctg 23208
human.FAM230J-LOC105372942-GGTLC5P.NCBI.rev.compl                                acataaagcccacgcattcatctgc----------------ctgtccatcattctgtctg 23924
human.FAM230A.ncRNA.18487127.18500594-GGTLC3.18516335.18518165.rev.compl.REF     acataaagcccatgcattcatctgc----------------ctgtccatcattctgtctg 12684
                                                                                 ** ********* ************                *******************

chimp.LOC112206744-LOC107973052-GGT2.revcompl                                    tccacacgggcatcatttgttagtggaactgagtgcccactgtcgagctgacaagcccat 25906
chimp54546-135457.revcompl.Pan.troglodytes.clone.rp43-41g5.GenBank:AC099533.36   CCCACACGGGCATCATTTGTTAGTGGAACTGAGTGCCCACTGTCGAGCTGACAAGCCCAT 27621
human.FAM30B-LOC105372935-GGT2.NCBI.                                             tccacacgggcatcattcgttagtggaactgagtgcccgctgtcgagctgacaagcccat 24534
human.FAM230E-LOC105377182-GGT3P.NCBI.                                           tccacacgggcatcattcgttagtggaactgagtgcccgctgtcgagctgacaagcccat 23268
human.FAM230J-LOC105372942-GGTLC5P.NCBI.rev.compl                                tccacacgggcatcattcgttagtggaactgagtgcccgctgtcgagctgacaagcccat 23984
human.FAM230A.ncRNA.18487127.18500594-GGTLC3.18516335.18518165.rev.compl.REF     tccacacgggcatcattcgttagtggaactgagtgcccactgtcgagctgacaagcccat 12744
                                                                                  **************** ********************* ********************

chimp.LOC112206744-LOC107973052-GGT2.revcompl                                    aacctccctgttcctagtcacacattaattcttcaacaagttccttttgatagattgtga 25966
chimp54546-135457.revcompl.Pan.troglodytes.clone.rp43-41g5.GenBank:AC099533.36   AACCTCCCTGTTCCTAGTCACACATTAATTCTTCAACAAGTCCCTTTTGATAGATTGTGA 27681
human.FAM30B-LOC105372935-GGT2.NCBI.                                             aacctccctgttcctagtcacacattaattcttcaacaagtcccttttgatagattgtga 24594
human.FAM230E-LOC105377182-GGT3P.NCBI.                                           aacctccctgttcctagtcacacattaattcttcaacaagtcccttttgatagattgtga 23328
human.FAM230J-LOC105372942-GGTLC5P.NCBI.rev.compl                                aacctccctgttcctagtcacacattaattcttcaacaagtcccttttgatagattgtga 24044
human.FAM230A.ncRNA.18487127.18500594-GGTLC3.18516335.18518165.rev.compl.REF     aacctccctgttcctagtcacacattaattcttcaacaagtcccttttgatagattgtga 12804
                                                                                 ***************************************** ******************

chimp.LOC112206744-LOC107973052-GGT2.revcompl                                    ttaagcttagctgctatttccaattgcttccccaaacgtacttctcactgttctcccatc 26026
chimp54546-135457.revcompl.Pan.troglodytes.clone.rp43-41g5.GenBank:AC099533.36   TTAAGCTTAGCTACTATTTCCAATTGCTTCCCCAAACGTACTTCTCACTGTTCTCCCATC 27741
human.FAM30B-LOC105372935-GGT2.NCBI.                                             ttaagcttagctactatttccaattgcttccccaaacgtacttctcactgttctcccatc 24654
human.FAM230E-LOC105377182-GGT3P.NCBI.                                           ttaagcttagctactatttccaattgcttccccaaacgtacttctcactgttctcccatc 23388
human.FAM230J-LOC105372942-GGTLC5P.NCBI.rev.compl                                ttaagcttagctactatttccaattgcttccccaaacgtacttctcactgttctcccatc 24104
human.FAM230A.ncRNA.18487127.18500594-GGTLC3.18516335.18518165.rev.compl.REF     ttaagcttagctactatttccaattgcttccccaaacgtacttctcactgttctcccatc 12864
                                                                                 ************ ***********************************************

chimp.LOC112206744-LOC107973052-GGT2.revcompl                                    acacccttcagcccatccatgcggggttcctttgcttttcccaccttacaccaaactccc 26086
chimp54546-135457.revcompl.Pan.troglodytes.clone.rp43-41g5.GenBank:AC099533.36   ACACCCTTCAGCCCATCCATGCGGGGTTCCTTTGCTTTTCCCACCTTACACCAAACTCCC 27801
human.FAM30B-LOC105372935-GGT2.NCBI.                                             acacccttcagcccatccatgcggggttcctttgcttttcccaccttacaccaaactccc 24714
human.FAM230E-LOC105377182-GGT3P.NCBI.                                           acacccttcagcccatccatgcggggttcctttgcttttcccaccttacaccaaactccc 23448
human.FAM230J-LOC105372942-GGTLC5P.NCBI.rev.compl                                acacccttcagcccatccatgcggggttcctttgcttttcccaccttacaccaaactccc 24164
human.FAM230A.ncRNA.18487127.18500594-GGTLC3.18516335.18518165.rev.compl.REF     acacccttcagcccatccatgcggggttcctttgcttttcccaccttacaccaaactccc 12924
                                                                                 ************************************************************

chimp.LOC112206744-LOC107973052-GGT2.revcompl                                    tatttttactcccactttacctcctctccaagacaaaacaaacaaaactagcattttaa 26146
chimp54546-135457.revcompl.Pan.troglodytes.clone.rp43-41g5.GenBank:AC099533.36   TATTTTTACTCCCACTTTTACCTCCTCTTCAAGACAAAACAAACAAAACTAGCATTTTAA 27861
human.FAM30B-LOC105372935-GGT2.NCBI.                                             tatttttactcccacttttacctcctctccaagacacaacaaacaaaactagcattttaa 24774
human.FAM230E-LOC105377182-GGT3P.NCBI.                                           tatttttactcccacttttacctcctctccaagacaaaacaaacaaaactagcattttaa 23508
```

```
human.FAM230J-LOC105372942-GGTLC5P.NCBI.rev.compl                                 tatttttactcccacttttacctcctctccaagacaaaacaaacaaaactagcattttaa 24224
human.FAM230A.ncRNA.18487127.18500594-GGTLC3.18516335.18518165.rev.compl.REF      tatttttactcccacttttacctcctctccaagacaaaacaaacaaaactagcattttaa 12984
                                                                                  **************************** ******* ***********************

chimp.LOC112206744-LOC107973052-GGT2.revcompl                                     aacttagttgtaatctttcttccttcatgaaaatttctccaacagccactcccacggtcc 26206
chimp54546-135457.revcompl.Pan.troglodytes.clone.rp43-41g5.GenBank:AC099533.36    AACTTAGTTGTAATCTTTCTTCCTTCATGAAAATTTCTCCAACAGCCACTCCCACGGTCC 27921
human.FAM30B-LOC105372935-GGT2.NCBI.                                              aacttagttgtaatctttcttccttcatgaaaatttctccaacagccactcccacggtcc 24834
human.FAM230E-LOC105377182-GGT3P.NCBI.                                            aacttagttgtaatctttcttccttcatgaaaatttctccaacagccactcccacggtcc 23568
human.FAM230J-LOC105372942-GGTLC5P.NCBI.rev.compl                                 aacttagttgtaatctttcttccttcatgaaaatttctccaacagccactcccacggtcc 24284
human.FAM230A.ncRNA.18487127.18500594-GGTLC3.18516335.18518165.rev.compl.REF      aacttagttgtaatctttcttccttcatgaaaatttctccaacagccactcccacggtcc 13044
                                                                                  ************************************************************

chimp.LOC112206744-LOC107973052-GGT2.revcompl                                     tgtgtgttccggatattttaaaataatggctataaggttgagcacttcaggatatgctgt 26266
chimp54546-135457.revcompl.Pan.troglodytes.clone.rp43-41g5.GenBank:AC099533.36    TGTGTCTTCCGGATATTTTAAAATAATGGCTATAAGGTTGAGCACTTCAGGATATGCTGT 27981
human.FAM30B-LOC105372935-GGT2.NCBI.                                              tgtgtgttccggatattttaaaataatggctataaggttgagcacttcaggatacgctgt 24894
human.FAM230E-LOC105377182-GGT3P.NCBI.                                            tgtgtgttccggatattttaaaataatggctataaggctgagcacttcaggatatgctgt 23628
human.FAM230J-LOC105372942-GGTLC5P.NCBI.rev.compl                                 tgtgtgttccggatattttaaaataatggctataaggttgagcacttcaggatacgctgt 24344
human.FAM230A.ncRNA.18487127.18500594-GGTLC3.18516335.18518165.rev.compl.REF      tgtgtgttccagatattttaaaataatggctataaggctgagcacttcaggatatgctgt 13104
                                                                                  ***** **** ************************** **************** *****

chimp.LOC112206744-LOC107973052-GGT2.revcompl                                     tttgctgtgtgcagatggaggcagtggctggagtgaatgaacggcaacacttgctggcaa 26326
chimp54546-135457.revcompl.Pan.troglodytes.clone.rp43-41g5.GenBank:AC099533.36    TTTGCTGTGTGCAGATGGAGGCAGTGGCTGGAGTGAATGAACGGCAACACTTGCTGGCAA 28041
human.FAM30B-LOC105372935-GGT2.NCBI.                                              tttgctgtgtgcagatggaggcagtggctggagtgaatgaacggcaacacttgctggcaa 24954
human.FAM230E-LOC105377182-GGT3P.NCBI.                                            tttgctgtgtgcagatggaggcagtggctggagtgaatgaacggcaacacttgctggcaa 23688
human.FAM230J-LOC105372942-GGTLC5P.NCBI.rev.compl                                 tttgctgtgtgcagatggaggcagtggctggagtgaatgaacggcaacacttgctggcaa 24404
human.FAM230A.ncRNA.18487127.18500594-GGTLC3.18516335.18518165.rev.compl.REF      tttgctgtgtgcagatggaggcagtggctggagtgaatgaacggcaacacttgctggcaa 13164
                                                                                  ************************************************************

chimp.LOC112206744-LOC107973052-GGT2.revcompl                                     ccggcagaagctgagagacagggaacaggctctcctccagagcctccaggagccaggcct 26386
chimp54546-135457.revcompl.Pan.troglodytes.clone.rp43-41g5.GenBank:AC099533.36    CCGGCAGAAGCTGAGAGACAGGGAACAGGCTCTCCTCCAGAGCCTCCAGGAGCCAGGCCT 28101
human.FAM30B-LOC105372935-GGT2.NCBI.                                              ccggcagaagctgagagacagggaacaggctctcctccagagcctccaggagccaggcct 25014
human.FAM230E-LOC105377182-GGT3P.NCBI.                                            ccggcagaagctgagagacagggaacaggctctcctccagagcctccaggagccaggcct 23748
human.FAM230J-LOC105372942-GGTLC5P.NCBI.rev.compl                                 ccggcagaagctgagagacagggaacaggctctcctccagagcctccaggagccaggcct 24464
human.FAM230A.ncRNA.18487127.18500594-GGTLC3.18516335.18518165.rev.compl.REF      ccggcagaagctgagagacagggaacaggctctcctccagagcctccaggagccaggcct 13224
                                                                                  ************************************************************

chimp.LOC112206744-LOC107973052-GGT2.revcompl                                     ttggacaccttgaatgtgggcttctgggagaccatgcgtttctgttataagcagcccagt 26446
chimp54546-135457.revcompl.Pan.troglodytes.clone.rp43-41g5.GenBank:AC099533.36    TTGGACACCTTGAATGTGGGCTTCTGGGAGACCATGCGTTTCTGTTATAAGCAGCCCAGT 28161
human.FAM30B-LOC105372935-GGT2.NCBI.                                              ttggacaacttgaatgtgggcttctgggagaccatgcgtttctgttataagcagcccagt 25074
human.FAM230E-LOC105377182-GGT3P.NCBI.                                            ttggacaccttgaatgtgggcttctgggagaccatgcgtttctgttataagcagcccagt 23808
human.FAM230J-LOC105372942-GGTLC5P.NCBI.rev.compl                                 ttggacaccttgaatgtgggcttctgggagaccatgcgtttctgttataagcagcccagt 24524
human.FAM230A.ncRNA.18487127.18500594-GGTLC3.18516335.18518165.rev.compl.REF      ttggacaccttgaatgtgggcttctgggagaccatgcgtttctgttataagcagcccagt 13284
                                                                                  ******* ****************************************************

chimp.LOC112206744-LOC107973052-GGT2.revcompl                                     cgctggcagtttttacggctgcccccgaacactcatctatacctgtctgacaaggtcaag 26506
chimp54546-135457.revcompl.Pan.troglodytes.clone.rp43-41g5.GenBank:AC099533.36    CTCTGGCAGTTTTTACGGCTGCCCCCGAACACTCATCTATACCTGTCTGACCAGGTCAAG 28221
human.FAM30B-LOC105372935-GGT2.NCBI.                                              ctctggcagtttttacggctgccccggaacactcatctatacctgtctgacaaggtcaag 25134
human.FAM230E-LOC105377182-GGT3P.NCBI.                                            ctctggcagtttttacggctgccccggaacactcatctatacctgtctgacaaggtcaag 23868
human.FAM230J-LOC105372942-GGTLC5P.NCBI.rev.compl                                 ctctggcagtttttacggctgccccggaacactcatctatacctgtctgacaaggtcaag 24584
human.FAM230A.ncRNA.18487127.18500594-GGTLC3.18516335.18518165.rev.compl.REF      ctctggcagtttttacggctgccccggaacactcatctatacctgtctgacaaggtcaag 13344
                                                                                  * *********************** ************************* ********

chimp.LOC112206744-LOC107973052-GGT2.revcompl                                     ctccaaggaagggattctctacatatctacattgtttgcagattttacaataatcattta 26566
chimp54546-135457.revcompl.Pan.troglodytes.clone.rp43-41g5.GenBank:AC099533.36    CTCCAAGGAAGGGATTCTCTACATATCTACATTGTTTGCAGATTTTACAATAATCATTTA 28281
human.FAM30B-LOC105372935-GGT2.NCBI.                                              ctccaaggaagggactctctacatatctacattgtttgcagattttacaataatcattta 25194
human.FAM230E-LOC105377182-GGT3P.NCBI.                                            ctccaaggaagggattctttacatatctacattgtttgcagattttacaataatcattta 23928
human.FAM230J-LOC105372942-GGTLC5P.NCBI.rev.compl                                 ctccaaggaagggactctctacatatctacattgtttgcagattttacaataatcattta 24644
human.FAM230A.ncRNA.18487127.18500594-GGTLC3.18516335.18518165.rev.compl.REF      ctccaaggaagggattctctacatatctacattgtttgcagattttacaataatcattta 13404
                                                                                  ************** *** *****************************************

chimp.LOC112206744-LOC107973052-GGT2.revcompl                                     ttcttgcatggctgatcattgttaaccaatacaaataaaataataaagaaatgacccaca 26626
chimp54546-135457.revcompl.Pan.troglodytes.clone.rp43-41g5.GenBank:AC099533.36    TTCTTGCATGGCTGATCATTGTTAACCAATACAAATAAAATAATAAAGAAATGACCCACA 28341
human.FAM30B-LOC105372935-GGT2.NCBI.                                              ttcttgcatggctgatcattgttaaccaatacaaataaaataataaagaaatgacccaca 25254
human.FAM230E-LOC105377182-GGT3P.NCBI.                                            ttcttgcatggctgatcattgttaaccaatacaaataaaataataaagaaatgacccaca 23988
human.FAM230J-LOC105372942-GGTLC5P.NCBI.rev.compl                                 ttcttgcatggctgatcattgttaaccaatacaaataaaataataaagaaatgacccaca 24704
human.FAM230A.ncRNA.18487127.18500594-GGTLC3.18516335.18518165.rev.compl.REF      ttcttgcatggctgatcattgttaaccaatacaaataaaataataaagaaatgacccaca 13464
                                                                                  ************************************************************

chimp.LOC112206744-LOC107973052-GGT2.revcompl                                     ttttatgttgggagtttgatctgccatttatcaagtatggaatcttgaacaaggggttaa 26686
chimp54546-135457.revcompl.Pan.troglodytes.clone.rp43-41g5.GenBank:AC099533.36    TTTTATGTTGGGAGTTTGATCTGCCATTTATCAAGTATGGAATCTTGAACAAGGGGTTAA 28401
human.FAM30B-LOC105372935-GGT2.NCBI.                                              ttttatgttgggagtttgatctgccatttatcaagtatggaatcttgaacaaggggttaa 25314
human.FAM230E-LOC105377182-GGT3P.NCBI.                                            ttttatgttgggagtttgatctgccatttatcaagtatggaatcttgaacaaggggttaa 24048
human.FAM230J-LOC105372942-GGTLC5P.NCBI.rev.compl                                 ttttatgttgggagtttgatctgccatttatcaagtatggaatcttgaacaaggggttaa 24764
human.FAM230A.ncRNA.18487127.18500594-GGTLC3.18516335.18518165.rev.compl.REF      ttttatgttgggagtttgatctgccatttatcaagtatggaatcttgaacaaggggttaa 13524
                                                                                  ************************************************************

chimp.LOC112206744-LOC107973052-GGT2.revcompl                                     acatctgaatgtctccatcacttcatctctaaagtggggggtgctcacacccactgggctc 26746
chimp54546-135457.revcompl.Pan.troglodytes.clone.rp43-41g5.GenBank:AC099533.36    ACATCTGAATGTCTCCATCACTTCATCTCTAAAGTGGGGGTGCTCACACCCACTGGGCTC 28461
human.FAM30B-LOC105372935-GGT2.NCBI.                                              acatctgaatgtctccatcacttcatctctaaagtgggggtgctcacacccactgggctc 25374
human.FAM230E-LOC105377182-GGT3P.NCBI.                                            acatctgaatgtctccatcacttcatctctaaagtgggggtgctcacacccactgggctc 24108
human.FAM230J-LOC105372942-GGTLC5P.NCBI.rev.compl                                 acatctgaatgtctccatcacttcatctctaaagtgggggtgctcacacccactgggctc 24824
human.FAM230A.ncRNA.18487127.18500594-GGTLC3.18516335.18518165.rev.compl.REF      acatctgaatgtctccatcacttcatctctaaagtgggggtgctcacacccactgggctc 13584
                                                                                  ************************************************************

chimp.LOC112206744-LOC107973052-GGT2.revcompl                                     cccct-ccaggttggtgccggactctccctgggccccccctgttctctcaccagccacatc 26805
chimp54546-135457.revcompl.Pan.troglodytes.clone.rp43-41g5.GenBank:AC099533.36    CCC--CCCAGGTTGGTGCCGGACTCTCCCTGGGCCCCCCTGTTCTCTCACCAGCCACATC 28519
human.FAM30B-LOC105372935-GGT2.NCBI.                                              ccccgcccaggttggtgccggactctccctgggcccccctgttctctcaccagccacatc 25434
human.FAM230E-LOC105377182-GGT3P.NCBI.                                            ccccgcccaggttggtgccggactctccctgggcccccctgttctctcaccagccacatc 24168
human.FAM230J-LOC105372942-GGTLC5P.NCBI.rev.compl                                 ccccgcccaggttggtgccggactctccctgggcccccctgttctctcaccagccacatc 24884
human.FAM230A.ncRNA.18487127.18500594-GGTLC3.18516335.18518165.rev.compl.REF      ccccacccaggttggtgccggactctccctgggcccccctgttctctcaccagccacatc 13644
                                                                                  ***   ******************************************************

chimp.LOC112206744-LOC107973052-GGT2.revcompl                                     cattctccccccagaggcgctagtgactgtgcgtggctttccattcccaccacgtttgtc 26865
chimp54546-135457.revcompl.Pan.troglodytes.clone.rp43-41g5.GenBank:AC099533.36    CATTCTCCCCCCAGAGGTGCTAGTGACTGTGCGTGGCTTTCCATTCCCACCACGTTTGTC 28579
human.FAM30B-LOC105372935-GGT2.NCBI.                                              cattctccccccagaggcgctagtgactgtgcgtggctttccattcccaccacgtttgtc 25494
human.FAM230E-LOC105377182-GGT3P.NCBI.                                            cattctccccccagaggcgctagtgactgtgcgtggctttccattcccaccacgtttgtc 24228
human.FAM230J-LOC105372942-GGTLC5P.NCBI.rev.compl                                 cattctccccccagaggcgctagtgactgtgcgtggctttccattcccaccacgtttgtc 24944
human.FAM230A.ncRNA.18487127.18500594-GGTLC3.18516335.18518165.rev.compl.REF      cattctccccccagaggcgctagtgactgtgcgtggctttccattcccaccatgtttgtc 13704
                                                                                  ***************** ********************************** *******

chimp.LOC112206744-LOC107973052-GGT2.revcompl                                     tctaaccccagtggcagatcagtgtaagaacacagctgagtgctcctcgcctccttgccc 26925
chimp54546-135457.revcompl.Pan.troglodytes.clone.rp43-41g5.GenBank:AC099533.36    TCTAACCCCAGTGGCAGATCAGTGTAAGAACACAGCTGAGTGCTCCTCGCCTCCTTGCCC 28639
human.FAM30B-LOC105372935-GGT2.NCBI.                                              tctaaccccagtggcagatcagtgtaagaacacagctgagtgctcctcgcctccttgccc 25554
human.FAM230E-LOC105377182-GGT3P.NCBI.                                            tctaaccccagtggcagatcagtgtaagaacacagctgagtgctcctcgcctccttgccc 24288
human.FAM230J-LOC105372942-GGTLC5P.NCBI.rev.compl                                 tctaaccccagtggcagatcagtgtaagaacacagctgagtgctcctcgcctccttgccc 25004
human.FAM230A.ncRNA.18487127.18500594-GGTLC3.18516335.18518165.rev.compl.REF      tctaaccccagtggcagatcagtgtaagaacacagctgagtgctcctcgcctccttgccc 13764
                                                                                  ************************************************************

chimp.LOC112206744-LOC107973052-GGT2.revcompl                                     cttcaagggctcctcaccacccaccagatcaggtgcaaacttccaagccttactggatcc 26985
chimp54546-135457.revcompl.Pan.troglodytes.clone.rp43-41g5.GenBank:AC099533.36    CTTCAAGGGCTCCTCACCACCCACCAGATCAGGTGCAAACTTCCAAGCCTTACTGGATCC 28699
human.FAM30B-LOC105372935-GGT2.NCBI.                                              cttcaagggcccctcaccacccaccagatcaggtgcaaacttccaagccttactggatcc 25614
human.FAM230E-LOC105377182-GGT3P.NCBI.                                            cttcaagggctcctcaccacccaccagatcaggtgcaaacttccaagccttactggatcc 24348
human.FAM230J-LOC105372942-GGTLC5P.NCBI.rev.compl                                 cttcaagggctcctcaccacccaccagatcaggtgcaaacttccaagccttactggatcc 25064
human.FAM230A.ncRNA.18487127.18500594-GGTLC3.18516335.18518165.rev.compl.REF      cttcaagggctcctcaccacccaccagatcaggtgcaaacttccaagccttactggatcc 13824
                                                                                  ********** *************************************************

chimp.LOC112206744-LOC107973052-GGT2.revcompl                                     ccttccacattctgagctccgcctgccttcccatcgctatccttccccacctgcctccct 27045
chimp54546-135457.revcompl.Pan.troglodytes.clone.rp43-41g5.GenBank:AC099533.36    CCTTCCACATTCTGAACTCCGCCTGCCTTCCCATCGCTATCCTTCCCCACCTGCCTCCCT 28759
human.FAM30B-LOC105372935-GGT2.NCBI.                                              ccttccacattctgagctccgcctgccttcccatcgctatccttccccacctgcctccct 25674
human.FAM230E-LOC105377182-GGT3P.NCBI.                                            ctttccacattctgagctccgcctgccttcccatcgctatccttccccacctgcctccct 24408
human.FAM230J-LOC105372942-GGTLC5P.NCBI.rev.compl                                 ccttccacattctgagctccgcctgccttcccatcgctatccttccccacctgcctccct 25124
human.FAM230A.ncRNA.18487127.18500594-GGTLC3.18516335.18518165.rev.compl.REF      ccttccacattctgagctccgcctgccttcccatcgctatccttccccacctgcctccct 13884
                                                                                  * ************* ********************************************

chimp.LOC112206744-LOC107973052-GGT2.revcompl                                     ggtagagaaaagcagagtgtgtgatgctgtctgaatgctgagcacggccttttgcagcca 27105
chimp54546-135457.revcompl.Pan.troglodytes.clone.rp43-41g5.GenBank:AC099533.36    GGTAGAGAAAAGCAGAGTGTGTGATGCTGTCTGAATGCTGAGCACGGCCTTTTGCAGCCA 28819
human.FAM30B-LOC105372935-GGT2.NCBI.                                              ggtagagaaaagcggagtgtgtgatgctgtctgaatgctgagcacggccttttgcagcca 25734
human.FAM230E-LOC105377182-GGT3P.NCBI.                                            ggtagagaaaagcagagtgtgtgatgctgtctgaatgctgagcacggccttttgcagcca 24468
human.FAM230J-LOC105372942-GGTLC5P.NCBI.rev.compl                                 ggtagagaaaagcggagtgtgtgatgctgtctgaatgctgagcacggccttttgcagcca 25184
human.FAM230A.ncRNA.18487127.18500594-GGTLC3.18516335.18518165.rev.compl.REF      ggtagagaaaagcagagtgtgtgatgctgtctgaatgctgagcacggccttttgcagcca 13944
                                                                                  ************* **********************************************

chimp.LOC112206744-LOC107973052-GGT2.revcompl                                     gtccactgtgtacgctgcccctatcggagacctccaccttaaccctttccagcctggagg 27165
chimp54546-135457.revcompl.Pan.troglodytes.clone.rp43-41g5.GenBank:AC099533.36    GTCCACTGTGGACGCTGCCCCTATCGGAGACCTCCACCTTAACCCTTTCCAGCCTGGAGG 28879
human.FAM30B-LOC105372935-GGT2.NCBI.                                              gtccactgtgtacgctgcccctatcggagacctccaccttaaccctttccagcctggagg 25794
human.FAM230E-LOC105377182-GGT3P.NCBI.                                            gtccactgtgtacgctgcccctatcggagacctccaccttaaccctttccagcctgtagg 24528
human.FAM230J-LOC105372942-GGTLC5P.NCBI.rev.compl                                 gtccactgtgtacgctgcccctatcggagacctccaccttaagcctttccagcctggagg 25244
human.FAM230A.ncRNA.18487127.18500594-GGTLC3.18516335.18518165.rev.compl.REF      gtccactgtgtatgctgcccctatcggagacctccaccttaaccctttccagcctggagg 14004
                                                                                  ********** * ***************************** ************* ***

chimp.LOC112206744-LOC107973052-GGT2.revcompl                                     ctcctcccagggccccacaacagaagtgacttcccttgcctttgaatttctatagcacaa 27225
chimp54546-135457.revcompl.Pan.troglodytes.clone.rp43-41g5.GenBank:AC099533.36    CTCCTCCCAGGGCCCCACAACAGAAGTGACTTCCCTTGCCTTTGAATTTCTATAGCACAA 28939
human.FAM30B-LOC105372935-GGT2.NCBI.                                              ctcctcccagggccccacaacagaagtgacttcccttgcctttgaatttctatagcacaa 25854
human.FAM230E-LOC105377182-GGT3P.NCBI.                                            ctcctcccagggccccacaacagaagtgacttcccttgcctttgaatttctatagcacaa 24588
human.FAM230J-LOC105372942-GGTLC5P.NCBI.rev.compl                                 ctcctcccagggccccacaacagaagtgacttcccttgcctttgaatttctatagcacaa 25304
```

```
human.FAM230A.ncRNA.18487127.18500594-GGTLC3.18516335.18518165.rev.compl.REF    ctcctcccagggccccacaacagaagtgacttcccttgcctttgaatttctatagcacaa 14064
                                                                                ************************************************************

chimp.LOC112206744-LOC107973052-GGT2.revcompl                                   tccctactgcccccgttaaaactgcaaagtccttttgtggaaaataactttattcatga 27285
chimp54546-135457.revcompl.Pan.troglodytes.clone.rp43-41g5.GenBank:AC099533.36  TCCCTACTGCCCCCGTTAAAACTGCAAAGTCCTTTTGTGGAAAATAACTTTATTCATGA 28999
human.FAM30B-LOC105372935-GGT2.NCBI.                                            tccctactgcccccgttaaaactgcaaagtccttttgtggaaaataactttattcatga 25914
human.FAM230E-LOC105377182-GGT3P.NCBI.                                          gccctactgcccccgttaaaactgcaaagtccttttgtggaaaataactttattcatga 24648
human.FAM230J-LOC105372942-GGTLC5P.NCBI.rev.compl                               gccctactgcccccgttaaaactgcaaagtccttttgtggaaaataactttattcatga 25364
human.FAM230A.ncRNA.18487127.18500594-GGTLC3.18516335.18518165.rev.compl.REF    gccctactgcccccgttaaaactgcaaagtccttttgtggaaaataactttattcatga 14124
                                                                                 ***********************************************************

chimp.LOC112206744-LOC107973052-GGT2.revcompl                                   ctgtgtttatcacactatcttatggagaagagatgatcaataaatatttgcggaataaat 27345
chimp54546-135457.revcompl.Pan.troglodytes.clone.rp43-41g5.GenBank:AC099533.36  CTGTGTTTATCACACTATCTTATGGAGAAGAGATGATCAATAAATATTTGCTGAATAAAT 29059
human.FAM30B-LOC105372935-GGT2.NCBI.                                            ctgtgtttatcacactatcttatggagaagagatgatcaataaatatttgctgaataaat 25974
human.FAM230E-LOC105377182-GGT3P.NCBI.                                          ctgtgtttatcacactatcttatggagaagagatgatcaataaatatttgctgaataaat 24708
human.FAM230J-LOC105372942-GGTLC5P.NCBI.rev.compl                               ctgtgtttatcacactatcttatggagaagagatgatcaataaatatttgctgaataaat 25424
human.FAM230A.ncRNA.18487127.18500594-GGTLC3.18516335.18518165.rev.compl.REF    ctgtgtttatcacactatcttatggagaagagatgatcaataaatatttgttgaataaat 14184
                                                                                **************************************************  ********

chimp.LOC112206744-LOC107973052-GGT2.revcompl                                   gaatagcagttacaaaacacttgattcatatggaattaatgttggttctcaaagtgaaaa 27405
chimp54546-135457.revcompl.Pan.troglodytes.clone.rp43-41g5.GenBank:AC099533.36  GAATAGCAGTTACAAAACACTTGATTCATATGGAATTAATGTTGGTTCTCAAAGTGAAAA 29119
human.FAM30B-LOC105372935-GGT2.NCBI.                                            gaatagcagttacaaaacacttgattcatatggaattaatgttggttctcaaagtgaaaa 26034
human.FAM230E-LOC105377182-GGT3P.NCBI.                                          gaatagcagttacaaaacactagattcatatggaattaatgttggttctcaaagtgaaaa 24768
human.FAM230J-LOC105372942-GGTLC5P.NCBI.rev.compl                               gaatagcagttacaaaacacttgattcatatggaattaatgttggttctcaaagtgaaaa 25484
human.FAM230A.ncRNA.18487127.18500594-GGTLC3.18516335.18518165.rev.compl.REF    gaatagcagttacaaaacacttgattcatatggaattaatgttggttctcaaagtgaaaa 14244
                                                                                ******************** ***************************************

chimp.LOC112206744-LOC107973052-GGT2.revcompl                                   attacaaacagcactgatattcagccagtatacaagtctggtcacagcagttgtataata 27465
chimp54546-135457.revcompl.Pan.troglodytes.clone.rp43-41g5.GenBank:AC099533.36  ATTACAAACAGCACTGATATTCAGCCAGTATACAAGTCTGGTAACAGCAGTTGTATAATA 29179
human.FAM30B-LOC105372935-GGT2.NCBI.                                            attacaaacagcactgatattcagccagtatacaagtctggtcacagcagttgtataata 26094
human.FAM230E-LOC105377182-GGT3P.NCBI.                                          attacaaacagcactgatattcagccagtatacaagtctggtcacagcagttgtataata 24828
human.FAM230J-LOC105372942-GGTLC5P.NCBI.rev.compl                               attacaaacagcactgatattcagccagtatacaagtctggtcacagcagttgtataata 25544
human.FAM230A.ncRNA.18487127.18500594-GGTLC3.18516335.18518165.rev.compl.REF    attacaaacagcactgatattcagccagtatacaagtctggtcacagcagttgtataata 14304
                                                                                ****************************************** *****************

chimp.LOC112206744-LOC107973052-GGT2.revcompl                                   ctgaaatacccctgccactgacctttggcccccagatgcctcccactgccactgctctc 27525
chimp54546-135457.revcompl.Pan.troglodytes.clone.rp43-41g5.GenBank:AC099533.36  CTGAAATACCCCTGCCACTGACCTTTGGCCCCCAGATGCCTCCCACTGCCACTGCTCTC 29239
human.FAM30B-LOC105372935-GGT2.NCBI.                                            ctgaaatacccctgccactgacctttggcccccagatgcctcccactgccactgctctc 26154
human.FAM230E-LOC105377182-GGT3P.NCBI.                                          ctgaaatacccctgccactgacctttggcccccagatgcctcccactgccactgctctc 24888
human.FAM230J-LOC105372942-GGTLC5P.NCBI.rev.compl                               ctgaaatacccccgccactgacctttggcccccagatgcctcccactgccactgctctc 25604
human.FAM230A.ncRNA.18487127.18500594-GGTLC3.18516335.18518165.rev.compl.REF    ctgaaatacccctgccactgacctttggcccccagatgcctcccactgccactgctctc 14364
                                                                                ************ ***********************************************

chimp.LOC112206744-LOC107973052-GGT2.revcompl                                   cccactgggaacccctgaagttcccacaggctcataactaaagggctaatgtcctgcaca 27585
chimp54546-135457.revcompl.Pan.troglodytes.clone.rp43-41g5.GenBank:AC099533.36  CCCACTGGGAACCCCTGAAGTTCCCACAGGCTCATAACTAAAGGGCTAATGTCTTGCACA 29299
human.FAM30B-LOC105372935-GGT2.NCBI.                                            cccactgggaacccctgaagttcccacaggctcataactaaagggctaatgtcttgcaca 26214
human.FAM230E-LOC105377182-GGT3P.NCBI.                                          cccactgggaacccctgaagttcccacaggctcataactaaagggctaatgtctcgcaca 24948
human.FAM230J-LOC105372942-GGTLC5P.NCBI.rev.compl                               cccactgggaacccctgaagttcccacaggctcataactaaagggctaatgtctcgcaca 25664
human.FAM230A.ncRNA.18487127.18500594-GGTLC3.18516335.18518165.rev.compl.REF    cccactgggaacccctgaagttcccacaggctcataactaaagggctaatgtctcgcaca 14424
                                                                                *****************************************************  *****

chimp.LOC112206744-LOC107973052-GGT2.revcompl                                   gcagcgagcacccaggaccgagcagccacatggccgggtctgctggtgaaagcatccatt 27645
chimp54546-135457.revcompl.Pan.troglodytes.clone.rp43-41g5.GenBank:AC099533.36  GCAGCGAGCACCCAGGACCGAGCAGCCACATGGCTGGGTCTGCTGGTGAAAGCATCCATT 29359
human.FAM30B-LOC105372935-GGT2.NCBI.                                            gcagcgagcacccaggaccgagcagccacatggccgggtctgctggtgaaagcatccatt 26274
human.FAM230E-LOC105377182-GGT3P.NCBI.                                          gcagcgagcacccaggaccgagcagccacatggccgggtctgctggtgaaagcatccatt 25008
human.FAM230J-LOC105372942-GGTLC5P.NCBI.rev.compl                               gcagcgagcacccaggaccgagcagccacatggccgggtctgctggtgaaagcatccatt 25724
human.FAM230A.ncRNA.18487127.18500594-GGTLC3.18516335.18518165.rev.compl.REF    gcagcgagcacccaggaccgagcagccacatggccgggtctgctggtgaaagcatccatt 14484
                                                                                ********************************** *************************

chimp.LOC112206744-LOC107973052-GGT2.revcompl                                   ctgactgatcaggacctgaggggcctcatggttacatattttgataatatccctaattat 27705
chimp54546-135457.revcompl.Pan.troglodytes.clone.rp43-41g5.GenBank:AC099533.36  CTGACTGATCAGGACCTGAGGGGCCTCATGGTTACATATTTTGATAATATCCCTAATTAT 29419
human.FAM30B-LOC105372935-GGT2.NCBI.                                            ctgactgatcaggacctgaggggcctcatggttacatattttgataatatccctaattat 26334
human.FAM230E-LOC105377182-GGT3P.NCBI.                                          ctgactgatcaggacctgaggggcctcatggttacatattttgataatatccctaattat 25068
human.FAM230J-LOC105372942-GGTLC5P.NCBI.rev.compl                               ctgactgatcaggacctgaggggcctcatggttacatattttgataatatccctaattat 25784
human.FAM230A.ncRNA.18487127.18500594-GGTLC3.18516335.18518165.rev.compl.REF    ctgactgatcaggacctgaggggcctcatggttacatattttgataatatccctaattat 14544
                                                                                ************************************************************

chimp.LOC112206744-LOC107973052-GGT2.revcompl                                   aaataaggctcagttatatagtttgaaaacaatgcttctcctcattgcaaaatctcttag 27765
chimp54546-135457.revcompl.Pan.troglodytes.clone.rp43-41g5.GenBank:AC099533.36  AAATAAGGCTCAGTTATATAGTTTGAAAACAATGCTTCTCCTCATTGCAAAATCTCTTAG 29479
human.FAM30B-LOC105372935-GGT2.NCBI.                                            aaataaggctcagttatatagtttgaaaacagtgcttctcctcattgcaaaatctcttag 26394
human.FAM230E-LOC105377182-GGT3P.NCBI.                                          aaataaggctcagttatatagtttgaaaacaatgcttctcctcattgcaaaatctcttag 25128
human.FAM230J-LOC105372942-GGTLC5P.NCBI.rev.compl                               aaataaggctcagttatatagtttgaaaacaatgcttctcctcattgcaaaatctcttag 25844
human.FAM230A.ncRNA.18487127.18500594-GGTLC3.18516335.18518165.rev.compl.REF    aaataaggctcagttatatagtttgaaaacaatgcttctcctcatcgcaaaatctcttag 14604
                                                                                ******************************* ************* **************

chimp.LOC112206744-LOC107973052-GGT2.revcompl                                   aagactccgtagatccaggaacggaaatggaaaatgacagcatgtcaatctctgaaggtt 27825
chimp54546-135457.revcompl.Pan.troglodytes.clone.rp43-41g5.GenBank:AC099533.36  AAGACTCCGTAGATCCAGGAACGGAAATGGAAAATGACAGCGTGTCAATCTCTGAAGGTT 29539
human.FAM30B-LOC105372935-GGT2.NCBI.                                            aagactccgtagatccaggaacggaaatggaaaatgacagcgtgtcaatctctgaaggtt 26454
human.FAM230E-LOC105377182-GGT3P.NCBI.                                          aagactccgtagatccaggaacggaaatggaaaatgacagcgtgtcaatctctgaaggtt 25188
human.FAM230J-LOC105372942-GGTLC5P.NCBI.rev.compl                               aagactccgtagatccaggaacggaaatggaaaatgacagcgtgtcaatctctgaaggtt 25904
human.FAM230A.ncRNA.18487127.18500594-GGTLC3.18516335.18518165.rev.compl.REF    aagactccgtagatccaggaacggaaatggaaaatgacagcgtgtcaatctctgaaggtt 14664
                                                                                ***************************************** ******************

chimp.LOC112206744-LOC107973052-GGT2.revcompl                                   ttgggcatttccattagcactccatcttcatgtaaaccagaagatatgcagtttcctgcc 27885
chimp54546-135457.revcompl.Pan.troglodytes.clone.rp43-41g5.GenBank:AC099533.36  TTGAGCATTTCCATTAGCACTCCATCTTCATGTAAACCAGAAGATACGCAGTTTCCTGCC 29599
human.FAM30B-LOC105372935-GGT2.NCBI.                                            ttgggcatttccattagcactccatcttcatgtaaaccagaagatatgcagtttcctgcc 26514
human.FAM230E-LOC105377182-GGT3P.NCBI.                                          ttgggcatttccattagcactccatcttcatgtaaaccagaagatatgcagtttcctgcc 25248
human.FAM230J-LOC105372942-GGTLC5P.NCBI.rev.compl                               ttgggcatttccattagcactccatcttcatgtaaaccagaagatatgcagtttcctgcc 25964
human.FAM230A.ncRNA.18487127.18500594-GGTLC3.18516335.18518165.rev.compl.REF    ttgggcatttccattagcactccatcttcatgtaaaccagaagatatgcagtttcctgcc 14724
                                                                                *** ***************************************** **************

chimp.LOC112206744-LOC107973052-GGT2.revcompl                                   tagagagaagagaagacacatcagcacagcggcatgaaaccttcatcagaaaacaatgct 27945
chimp54546-135457.revcompl.Pan.troglodytes.clone.rp43-41g5.GenBank:AC099533.36  TAGAGAGAAGAGAAGACACATCAGCACAGTGGCATGAAATCTTCATCAGAAAACAATGCT 29659
human.FAM30B-LOC105372935-GGT2.NCBI.                                            tagagagaagagaagacacatcagcacagcggcatgaaaccttcatcagaaaacaatgct 26574
human.FAM230E-LOC105377182-GGT3P.NCBI.                                          tagagagaagagaagacacatcagcacagcggcatgaaaccttcatcagaaaacaatgct 25308
human.FAM230J-LOC105372942-GGTLC5P.NCBI.rev.compl                               tagagagaagagaagacacatcagcacagcggcatgaaaccttcatcagaaaacaatgct 26024
human.FAM230A.ncRNA.18487127.18500594-GGTLC3.18516335.18518165.rev.compl.REF    tagagagaagagaagacacatcagcacagcggcatgaaagcttcatcagaaaacaatgct 14784
                                                                                ***************************** ********* ********************

chimp.LOC112206744-LOC107973052-GGT2.revcompl                                   tcattaatccttgacaggacaagcgtcagcaaacttccaggccgctggattaagccttca 28005
chimp54546-135457.revcompl.Pan.troglodytes.clone.rp43-41g5.GenBank:AC099533.36  TCATTAATCCGTGACAGGACAAGTGTCAGCAAACTTCCAGGCCGCTGGATTAAGCCTTCA 29719
human.FAM30B-LOC105372935-GGT2.NCBI.                                            tcattaatccgtgacaggacaagcgtcagcaaacttccaggcggctggattaggccttca 26634
human.FAM230E-LOC105377182-GGT3P.NCBI.                                          tcattaatccgtgacaggacaagcgtcagcaaacttccaggcggctggattaggccttca 25368
human.FAM230J-LOC105372942-GGTLC5P.NCBI.rev.compl                               tcattaatccgtgacaggacaagcgtcagcaaacttccaggcggctggataaggccttca 26084
human.FAM230A.ncRNA.18487127.18500594-GGTLC3.18516335.18518165.rev.compl.REF    tcattaatccgtgacaggacaagcgtcagcaaacttccaggcggctggattaggccttca 14844
                                                                                ********** ************ ****************** ******* * *******

chimp.LOC112206744-LOC107973052-GGT2.revcompl                                   tctatccatcaccttggagaggaacaaaataggtggcctgggaagataagcactatgttt 28065
chimp54546-135457.revcompl.Pan.troglodytes.clone.rp43-41g5.GenBank:AC099533.36  TCTATCCATCACCTTGGAGAGGAACAAAATAGGTGGCCTGGGAAGATAAGCACTATGTTT 29779
human.FAM30B-LOC105372935-GGT2.NCBI.                                            tctatccatcaccttggagaggaacaaaataggtggcctgggaagataagcactatgttt 26694
human.FAM230E-LOC105377182-GGT3P.NCBI.                                          tctatccatcaccttggagaggaacaaaataggtggcctgggaagttaagcactatgttt 25428
human.FAM230J-LOC105372942-GGTLC5P.NCBI.rev.compl                               tctatccatcaccttggagaggaacaaaataggtggcctgggaagttaagcactatgttt 26144
human.FAM230A.ncRNA.18487127.18500594-GGTLC3.18516335.18518165.rev.compl.REF    tctatccatcaccttggagaggaacaaaataggtggcctgggaagataagcactatgttt 14904
                                                                                ********************************************* **************

chimp.LOC112206744-LOC107973052-GGT2.revcompl                                   ctattagttaatatctaaagcggaggttaacaagctatggacacacaagccaaacccagc 28125
chimp54546-135457.revcompl.Pan.troglodytes.clone.rp43-41g5.GenBank:AC099533.36  CTATTAGTTAATATCTAAAGCGGAGGTTAACAAGCTATGGACACACAAGCCAAACCCAGC 29839
human.FAM30B-LOC105372935-GGT2.NCBI.                                            ctattagttaatatctaaagcggaggttaacaagctatggacacacaagccaaacccagc 26754
human.FAM230E-LOC105377182-GGT3P.NCBI.                                          ctattagttaatatctaaagcggaggttaacaagctatggacacacaagccaaacccagc 25488
human.FAM230J-LOC105372942-GGTLC5P.NCBI.rev.compl                               ctattcgttaatatctaaagcggaggttaacaagctatggacacacaagccaaacccagc 26204
human.FAM230A.ncRNA.18487127.18500594-GGTLC3.18516335.18518165.rev.compl.REF    ctattccttaatatctaaagcggaggttaacaagctatggacacacaagccaaacccagc 14964
                                                                                *****  *****************************************************

chimp.LOC112206744-LOC107973052-GGT2.revcompl                                   cctcttggggtttttaaatctactttcaacttttattttagattcagcgggcacatgtg 28185
chimp54546-135457.revcompl.Pan.troglodytes.clone.rp43-41g5.GenBank:AC099533.36  CCTCTTGGGGTTTTTAAATCTACTTTCAACTTTTATTTTAGATTCAGCGGGCACATGTG 29899
human.FAM30B-LOC105372935-GGT2.NCBI.                                            cctcttggggtttttaaatctactttcaacttttattttagattcagcgggcacatgtg 26814
human.FAM230E-LOC105377182-GGT3P.NCBI.                                          cctcttggggtttttaaatctactttcaacttttattttagattcagcgggcacatgtg 25548
human.FAM230J-LOC105372942-GGTLC5P.NCBI.rev.compl                               cctcttggggtttttaaatctactttcaacttttattttagattcagcgggcacatgtg 26264
human.FAM230A.ncRNA.18487127.18500594-GGTLC3.18516335.18518165.rev.compl.REF    cctcttggggtttttaaatctactttcaacttttattttagattcagcgggcacatgtg 15024
                                                                                ************************************************************

chimp.LOC112206744-LOC107973052-GGT2.revcompl                                   caggtttgtcacgtggatatgagcatactccccaacagttggcctttcacccctcccctc 28245
chimp54546-135457.revcompl.Pan.troglodytes.clone.rp43-41g5.GenBank:AC099533.36  CAGGTTTGTCACGTGGATATGAGCATACTCCCCAACAGTTGGCCTTTTGCCCCTCCCCTC 29959
human.FAM30B-LOC105372935-GGT2.NCBI.                                            caggtttgtcacatggatatgagcatactccccaacagttggcctttcacccctcccctc 26874
human.FAM230E-LOC105377182-GGT3P.NCBI.                                          caggtttgtcacgtggatatgagcatactccccaacagttggcctttcacccctcccctc 25608
human.FAM230J-LOC105372942-GGTLC5P.NCBI.rev.compl                               caggtttgtcacgtggatatgagcatactccccaacagttggcctttcacccctcccctc 26324
human.FAM230A.ncRNA.18487127.18500594-GGTLC3.18516335.18518165.rev.compl.REF    caggtttgtcacgtggatatgagcatactccccaacagttggcctttcacccctcccctc 15084
                                                                                ************ **********************************  ***********

chimp.LOC112206744-LOC107973052-GGT2.revcompl                                   cctccccatccagcagttcccagttgttgccatctttaagtcaatgactgcccatgttta 28305
chimp54546-135457.revcompl.Pan.troglodytes.clone.rp43-41g5.GenBank:AC099533.36  CCTCCCCATCCAGCAGTTCCCAGTTGTTGCCATCTTTAAGTCAATGAGTCCCCATGTTTA 30019
human.FAM30B-LOC105372935-GGT2.NCBI.                                            cctccccatccagcagttcccagttgttgccatctttaagtcaatgagtccccatgttta 26934
human.FAM230E-LOC105377182-GGT3P.NCBI.                                          cctccccatccagcagttcccagttgttgccatctttaagtcaatgagtccccatgttta 25668
human.FAM230J-LOC105372942-GGTLC5P.NCBI.rev.compl                               cctccccatccagcagttcccagttgttgccatctttaagtcaatgagtccccatgttta 26384
human.FAM230A.ncRNA.18487127.18500594-GGTLC3.18516335.18518165.rev.compl.REF    cctccccatccagcagttcccagttgttgccatctttaagtcaatgagtccccatgttta 15144
```

```
                                                                                  *********************************************** * **********

chimp.LOC112206744-LOC107973052-GGT2.revcompl                                     gctcccatttataagagagaatatgcattatgttttgtttggtttttgctg------gtt 28359
chimp54546-135457.revcompl.Pan.troglodytes.clone.rp43-41g5.GenBank:AC099533.36    GCTCCCATTTATAAGAGAGAACATGCATTATGTTTTGTTTGGTTTTTGCTG------TTT 30073
human.FAM30B-LOC105372935-GGT2.NCBI.                                              gctcccatttataagagagaacatgcattatgttttgtttggtttttgctgg-ttttttt 26993
human.FAM230E-LOC105377182-GGT3P.NCBI.                                            gctcccatttataagagagaacatgcattatgttttgtttggtttttgctgg-ttttttt 25727
human.FAM230J-LOC105372942-GGTLC5P.NCBI.rev.compl                                 gctcccatttataagagagaacatgcattatgttttgtttggtttttgctggtttttttt 26444
human.FAM230A.ncRNA.18487127.18500594-GGTLC3.18516335.18518165.rev.compl.REF      gctcccatttataagagagaacatgcattatgttttgtttggtttttgctgg-ttttttt 15203
                                                                                  ********************* *****************************       **

chimp.LOC112206744-LOC107973052-GGT2.revcompl                                     tttttttttaatggagtcttgccctgtagcccaggctagagtgcagtggcacaatcttg 28419
chimp54546-135457.revcompl.Pan.troglodytes.clone.rp43-41g5.GenBank:AC099533.36    TTTTTTTTTAATGGAGTCTTGCCCTGTAGCCCAGGCTAGAGTGCAGTGGCACAATCTTG 30133
human.FAM30B-LOC105372935-GGT2.NCBI.                                              tttttttttaatggagtcttgccctgtagcccaggctagagtgcagtggcacaaccttg 27053
human.FAM230E-LOC105377182-GGT3P.NCBI.                                            tttttttttaatggagtcttgccctgtagcccaggctagagtgcagtggcacaatcttg 25787
human.FAM230J-LOC105372942-GGTLC5P.NCBI.rev.compl                                 tttttttttaatggagtcttgccctgtagcccaggctagagtgcagtggcacaatcttg 26504
human.FAM230A.ncRNA.18487127.18500594-GGTLC3.18516335.18518165.rev.compl.REF      tttttttttaatggagtcttgccctgtagcccaggctagagtgcagtggcacaatcttg 15263
                                                                                  ******************************************************* ****

chimp.LOC112206744-LOC107973052-GGT2.revcompl                                     gctcactgcaacctccgcctcccaggttcaaacaattctccctcctcagcctcccgagta 28479
chimp54546-135457.revcompl.Pan.troglodytes.clone.rp43-41g5.GenBank:AC099533.36    GCTCACTGCAACCTCCGCCTCCCAGGTTCAAACAATTCTCCCTCCTCAGCCTCCCGAGTA 30193
human.FAM30B-LOC105372935-GGT2.NCBI.                                              gctcactgcaacctccgcctcccaggttcaaacgattctccctcctcagcctcccgagtg 27113
human.FAM230E-LOC105377182-GGT3P.NCBI.                                            gctcactgcaacctccgcctcccaggttcaaacgattctccctcctcagcctcccgagtg 25847
human.FAM230J-LOC105372942-GGTLC5P.NCBI.rev.compl                                 gctcactgcaacctccgcctcccaggttcaaacgattctccctcctcagcctcccgagtg 26564
human.FAM230A.ncRNA.18487127.18500594-GGTLC3.18516335.18518165.rev.compl.REF      gctcactgcaacctccgcctcccaggttcaaacgattctccctcctcagcctcccgagtg 15323
                                                                                  ********************************* *************************

chimp.LOC112206744-LOC107973052-GGT2.revcompl                                     gctgggactacaggtgcctgccaccacgcccagctaactttttgtatttttagtagagac 28539
chimp54546-135457.revcompl.Pan.troglodytes.clone.rp43-41g5.GenBank:AC099533.36    GCTGGGACTACAGGTGCCCGCCACCATGCCCAGCTAACTTTTTGTATTTTTAGTAGAGAC 30253
human.FAM30B-LOC105372935-GGT2.NCBI.                                              gctgggactacaggcgcccaccaccacgcccggctaactttttgtatttttagtagagac 27173
human.FAM230E-LOC105377182-GGT3P.NCBI.                                            gctgggactacaggcgcccgccaccacgcccggctaactttttgtatttttagtagagac 25907
human.FAM230J-LOC105372942-GGTLC5P.NCBI.rev.compl                                 gctgggactacaggcgcccgccaccacgcccggctaactttttgtatttttagtagagac 26624
human.FAM230A.ncRNA.18487127.18500594-GGTLC3.18516335.18518165.rev.compl.REF      gctgggactacaggcgcccgccaccacgcccggctaactttttgtatttttagtagagac 15383
                                                                                  ************** ***  ****** **** ****************************

chimp.LOC112206744-LOC107973052-GGT2.revcompl                                     agggtttcactgtgttagccaggatggtctcaatctcctgacctcatgatctgcccacct 28599
chimp54546-135457.revcompl.Pan.troglodytes.clone.rp43-41g5.GenBank:AC099533.36    AGGGTTTCACCGTGTTAGCCAGGATGGTCTCAATCTCCTGACCTCATGATCTGCCCACCT 30313
human.FAM30B-LOC105372935-GGT2.NCBI.                                              agggtttcaccgtgttagccaggatggtctcaatctcctgacctcatgatctgcccacct 27233
human.FAM230E-LOC105377182-GGT3P.NCBI.                                            agggtttcaccgtgttagccaggatggtctcaatctcctgacctcatgatctgcccacct 25967
human.FAM230J-LOC105372942-GGTLC5P.NCBI.rev.compl                                 agggtttcaccgtgttagccaggatggtctcaatctcctgacctcatgatctgcccacct 26684
human.FAM230A.ncRNA.18487127.18500594-GGTLC3.18516335.18518165.rev.compl.REF      agggtttcaccgtgttagccaggatggtctcaatctcctgacctcatgatctgcccacct 15443
                                                                                  ********** *************************************************

chimp.LOC112206744-LOC107973052-GGT2.revcompl                                     cagtctcccaaagtgcagggattacaggcatgagccaccgtgcccagtcttttgtttatt 28659
chimp54546-135457.revcompl.Pan.troglodytes.clone.rp43-41g5.GenBank:AC099533.36    CACTCTCCCAAAGTGCAGGGATTACAGGCGTGAGCCACCGTGCCCAGCCTTTTGTTTATT 30373
human.FAM30B-LOC105372935-GGT2.NCBI.                                              tagtctcccaaagtgcagggattacaggcgtgagccaccgtgcccagccttttgtttatt 27293
human.FAM230E-LOC105377182-GGT3P.NCBI.                                            cagtctcccaaagtgcagggattacaggcgtgagccaccgtgcccagccttttgtttatt 26027
human.FAM230J-LOC105372942-GGTLC5P.NCBI.rev.compl                                 cagtctcccaaagtgcagggattacaggcgggagccaccgtgcccagccttttgtttatt 26744
human.FAM230A.ncRNA.18487127.18500594-GGTLC3.18516335.18518165.rev.compl.REF      cagtctcccaaagtgcagggattacaggcgtgagccaccgtgcccagccttttgtttatt 15503
                                                                                   * **************************  **************** ************

chimp.LOC112206744-LOC107973052-GGT2.revcompl                                     ttttgacgagaccgttcttgctctgtcaccaggctggagtgcactggcacagtaatagct 28719
chimp54546-135457.revcompl.Pan.troglodytes.clone.rp43-41g5.GenBank:AC099533.36    TTTTGACGAGACCGTTCTTGCTCTGTCACCAGGCTGGAGTGCACTGGCACAGTAATAGCT 30433
human.FAM30B-LOC105372935-GGT2.NCBI.                                              ttttgatgagacggttcttgctctgtcaccaggctggagtgcactggcacaataatagct 27353
human.FAM230E-LOC105377182-GGT3P.NCBI.                                            ttttgacgagaccgttcttgctctgtcaccaggctggagtgcactggcacaataatagct 26087
human.FAM230J-LOC105372942-GGTLC5P.NCBI.rev.compl                                 ttttgacgagaccgttcttgctctgtcaccaggctggagtgcactggcacaataatagct 26804
human.FAM230A.ncRNA.18487127.18500594-GGTLC3.18516335.18518165.rev.compl.REF      ttttgacgagacggttcttgctctgtcaccaggctggagtgcactggcacaataatagct 15563
                                                                                  ****** ***** ************************************** ********

chimp.LOC112206744-LOC107973052-GGT2.revcompl                                     caccacagccttgtgctcctgggctcaactgaccctcctgcctcagttttagcttcctga 28779
chimp54546-135457.revcompl.Pan.troglodytes.clone.rp43-41g5.GenBank:AC099533.36    CACCACAGCCTCGTGCTCCTGGGCTCAACTGACCCTCCTGCCTCAGTTTTAGCTTCCTGA 30493
human.FAM30B-LOC105372935-GGT2.NCBI.                                              caccacagcctcgtgctcctgggctcaactgaccctcctgcctcagttttagcttcctga 27413
human.FAM230E-LOC105377182-GGT3P.NCBI.                                            caccacagcctcgtgctcctgggctcaactgaccctcctgcctcagttttagcttcctga 26147
human.FAM230J-LOC105372942-GGTLC5P.NCBI.rev.compl                                 caccacagcctcgtgctcctgggctcaactgaccctcctgcctcagttttagcttcctga 26864
human.FAM230A.ncRNA.18487127.18500594-GGTLC3.18516335.18518165.rev.compl.REF      caccacagcctcgtgctcctgggctcaactgaccctcctgcctcagttttagcttcctga 15623
                                                                                  *********** ************************************************

chimp.LOC112206744-LOC107973052-GGT2.revcompl                                     gtagctaggactacgggtgtgtaccaccatgcctagctataataattttatttttttgt 28839
chimp54546-135457.revcompl.Pan.troglodytes.clone.rp43-41g5.GenBank:AC099533.36    GTAGCTAGGACTACGGGTGTGTACCACCATGCCTAGCTATAATAATTTTATTTTTTTGT 30553
human.FAM30B-LOC105372935-GGT2.NCBI.                                              gtagctaggactacgggtgtgtaccaccatgcctagctataataattttatttttttgt 27473
human.FAM230E-LOC105377182-GGT3P.NCBI.                                            gtagctaggactacgggtgtgtaccaccatgcctagctataataattttatttttttgt 26207
human.FAM230J-LOC105372942-GGTLC5P.NCBI.rev.compl                                 gtagctaggactacgggtgtgtaccaccatgcctagctataataattttatttttttgt 26924
human.FAM230A.ncRNA.18487127.18500594-GGTLC3.18516335.18518165.rev.compl.REF      gtagctaggactacaggtgtgtaccaccatgcctagctataataattttatttttttgt 15683
                                                                                  ************** *********************************************

chimp.LOC112206744-LOC107973052-GGT2.revcompl                                     agagatggagtcttgctttgttgcccaggctggtcttgaactcctggcttaaagtgatcc 28899
chimp54546-135457.revcompl.Pan.troglodytes.clone.rp43-41g5.GenBank:AC099533.36    AGAGATGGAGACTTGCTTTGTTGCCCAGGCTGGTCTTGAACTCCTGGCTTAAAGTGATCC 30613
human.FAM30B-LOC105372935-GGT2.NCBI.                                              agagatggagtcttgctttgttgcccaggctggtcttgaactcctggcttgaagtgatcc 27533
human.FAM230E-LOC105377182-GGT3P.NCBI.                                            agagatggagtcttgctttgttgcccaggctggtcttgaactcctggcttgaagtgatcc 26267
human.FAM230J-LOC105372942-GGTLC5P.NCBI.rev.compl                                 agagatggagtcttgctttgttgcccaggctggtcttgaactcctggcttgaagtgatcc 26984
human.FAM230A.ncRNA.18487127.18500594-GGTLC3.18516335.18518165.rev.compl.REF      agagatggagtcttgctttgttgcccaggctggtcttgaagtcctggcttgaagtgatcc 15743
                                                                                  ********** ***************************** ********* *********

chimp.LOC112206744-LOC107973052-GGT2.revcompl                                     tcctgcctcggcctcccaaagtgctgggattaaaggtgtgagatcacacccagtctccaa 28959
chimp54546-135457.revcompl.Pan.troglodytes.clone.rp43-41g5.GenBank:AC099533.36    TCCTGCCTCGGCCTCCCAAAGTGCTGGGATTAAAGGTATGAGATCACACCCAGTCTCCAA 30673
human.FAM30B-LOC105372935-GGT2.NCBI.                                              tcctgcctcggcctcccaaagtgccgggattaaaggtgggagatcgcacccagtctccaa 27593
human.FAM230E-LOC105377182-GGT3P.NCBI.                                            tcctgcctcggcctcccaaattgccgggattaaaggtgggagatcgcacccagtctccaa 26327
human.FAM230J-LOC105372942-GGTLC5P.NCBI.rev.compl                                 tcctgcctcggcctcccaaagtgccgggattaaaggtgggagatcgcacccagtctccaa 27044
human.FAM230A.ncRNA.18487127.18500594-GGTLC3.18516335.18518165.rev.compl.REF      tcctgcctcggcctcccaaattgccgggattaaaggtgggagatcgcacccagtctccaa 15803
                                                                                  ******************** *** ************  ****** **************

chimp.LOC112206744-LOC107973052-GGT2.revcompl                                     ccctcttttgcaagtaaatgtaactggaccccagccatgctcatctgcccatgtactgt 29019
chimp54546-135457.revcompl.Pan.troglodytes.clone.rp43-41g5.GenBank:AC099533.36    CCCTCTTTTGCAAGTAAATGTAACTGGACCCCAGCCATGCTCATCTGCCCATGTACTGT 30733
human.FAM30B-LOC105372935-GGT2.NCBI.                                              ccctcttttgcaagtaaatgtaactggaccccagccatgctcatctgcccatgtactgt 27653
human.FAM230E-LOC105377182-GGT3P.NCBI.                                            ccctcttttgcaagtaaatgtaactggaccccagccatgctcatctgcccatgtactgt 26387
human.FAM230J-LOC105372942-GGTLC5P.NCBI.rev.compl                                 ccctcttttgcaagtaaatgtaactggaccccagccatgctcatctgcccatgtactgt 27104
human.FAM230A.ncRNA.18487127.18500594-GGTLC3.18516335.18518165.rev.compl.REF      ccctcttttgcaagtaaatgtaactggaccccagccatgctcatctgcccatgtactgt 15863
                                                                                  ************************************************************

chimp.LOC112206744-LOC107973052-GGT2.revcompl                                     ctacggctgcttttgctctatagggcagagttaagtggttgcaacagacactgcacagac 29079
chimp54546-135457.revcompl.Pan.troglodytes.clone.rp43-41g5.GenBank:AC099533.36    CTACGGCTGCTTTTGCTCTATAGGGCAGAGTTAAGTGGTTGCAACAGACACTGCACAGAC 30793
human.FAM30B-LOC105372935-GGT2.NCBI.                                              ctacggctgcttttgctctacagggcagagttaagtggttgcaacagacactgcacagac 27713
human.FAM230E-LOC105377182-GGT3P.NCBI.                                            ctgcggctgcttttgctctacagggcagagttaagtggttgcaacagacaccgcacagac 26447
human.FAM230J-LOC105372942-GGTLC5P.NCBI.rev.compl                                 ctgcggctgcttttgctctacagggcagagttaagtggttgcaacagacaccgcacagac 27164
human.FAM230A.ncRNA.18487127.18500594-GGTLC3.18516335.18518165.rev.compl.REF      ctacggctgcttttgctctacagggcagagttaagtggttgcaacagacaccgcacagac 15923
                                                                                  ** ***************** ****************************** ********

chimp.LOC112206744-LOC107973052-GGT2.revcompl                                     cacaaagtctggaagtactttctctccagccctttacagagaaagtctgccaacctctaa 29139
chimp54546-135457.revcompl.Pan.troglodytes.clone.rp43-41g5.GenBank:AC099533.36    CACAAAGTCT-GAAGTACTTTCTCTCCAGCCCTTTACAGAGAAAGTCTGCCAACCTCTAA 30852
human.FAM30B-LOC105372935-GGT2.NCBI.                                              cacaaagtct-gaagtactttctctccagccctttacagagaaagtctgccaacctctaa 27772
human.FAM230E-LOC105377182-GGT3P.NCBI.                                            cacaaagtct-gaagtactttctctccagccctttacagagaaagtctgccaacctctaa 26506
human.FAM230J-LOC105372942-GGTLC5P.NCBI.rev.compl                                 cacaaagtct-gaagtactttctctccagccctttacagagaaagtctgccaacctctaa 27223
human.FAM230A.ncRNA.18487127.18500594-GGTLC3.18516335.18518165.rev.compl.REF      cacaaagtct-gaagtactttctctccagccctttacagagaaagtctgccaacctctaa 15982
                                                                                  ********** *************************************************

chimp.LOC112206744-LOC107973052-GGT2.revcompl                                     tctcaataacagggaaatcaatgacaaccacaaagtgacaaagattgggtgtctaagatg 29199
chimp54546-135457.revcompl.Pan.troglodytes.clone.rp43-41g5.GenBank:AC099533.36    TCTCAATAACAGGGAAATCAATGACAACCACAAAGTGACAAAGATTGGGTGTCTAAGATG 30912
human.FAM30B-LOC105372935-GGT2.NCBI.                                              tctcaataacagggaaatcaatgacaaccacaaagtgacaaagattgggtgtctaagatg 27832
human.FAM230E-LOC105377182-GGT3P.NCBI.                                            tctcaataacagggaaatcaatgacaaccacaaagtgacaaagattgggtgtctaagatg 26566
human.FAM230J-LOC105372942-GGTLC5P.NCBI.rev.compl                                 tctcaataacagggaaatcaatgacaaccacaaagtgacaaagattgggtgtctaagatg 27283
human.FAM230A.ncRNA.18487127.18500594-GGTLC3.18516335.18518165.rev.compl.REF      tctcaataacagggaaatcaatgacaaccacaaagtgacaaagactgggtgtctaagatg 16042
                                                                                  ******************************************** ***************

chimp.LOC112206744-LOC107973052-GGT2.revcompl                                     gatggtcagaataaacaagagagaaagatgaaaagtagaaggaggatttcaagcgcaagc 29259
chimp54546-135457.revcompl.Pan.troglodytes.clone.rp43-41g5.GenBank:AC099533.36    GATGGTCAGAATAAACAAGAGAGAAAGATGAAAAGTAGAAGGAGGATTTCAAACGCAAGC 30972
human.FAM30B-LOC105372935-GGT2.NCBI.                                              gatgttcagaataaacaagagagaaagatgagaagtagaaggaggatttcaaacgcaagc 27892
human.FAM230E-LOC105377182-GGT3P.NCBI.                                            gatgctcagaataaacaagagagaaagatgaaaagtagaaggaggatttcaaacgcaagc 26626
human.FAM230J-LOC105372942-GGTLC5P.NCBI.rev.compl                                 gatgctcagaataaacaagagagaaagatgaaaagtagaaggaggatttcaaacgcaagc 27343
human.FAM230A.ncRNA.18487127.18500594-GGTLC3.18516335.18518165.rev.compl.REF      gatgctcagaataaacaagagagaaagatgaaaagtagaaggaggatttcaaacgcaagc 16102
                                                                                  **** ************************** ******************** *******

chimp.LOC112206744-LOC107973052-GGT2.revcompl                                     ttcacctaatccgttatttttcaaatgaccaggcctatctctgtagctgaaaatcacctc 29319
chimp54546-135457.revcompl.Pan.troglodytes.clone.rp43-41g5.GenBank:AC099533.36    TTCACCTAATCCGTTATTTTTCAAATGACCAGGCCTATCTCTGTAGCTGAAAATCACCTC 31032
human.FAM30B-LOC105372935-GGT2.NCBI.                                              ttcacctaatccgttatttttcaaatgaccaggcctatctctgtagccgaaaatcacctc 27952
human.FAM230E-LOC105377182-GGT3P.NCBI.                                            ttcacctaatccgttatttttcaaatgaccaggcctatctctgtagctgaaaatcacctc 26686
human.FAM230J-LOC105372942-GGTLC5P.NCBI.rev.compl                                 ttcacctaatccgttatttttcaaatgaccaggcctatctctgtagctgaaaatcacctc 27403
human.FAM230A.ncRNA.18487127.18500594-GGTLC3.18516335.18518165.rev.compl.REF      ttcacctaatccgttatttttcaaatgaccaggcctatctctgtagctgaaaatcacctc 16162
                                                                                  *********************************************** ************

chimp.LOC112206744-LOC107973052-GGT2.revcompl                                     aaataggatctctgatatacagtctccaaaagctcagctaagaaacttac--agtctctc 29377
chimp54546-135457.revcompl.Pan.troglodytes.clone.rp43-41g5.GenBank:AC099533.36    AAATAGGATCTCTGATATACAGTCTCCAAAAGCTCAGCCAAGAAACTTACAAAGTCTCTC 31092
human.FAM30B-LOC105372935-GGT2.NCBI.                                              aaataggatctctgatatacagtctccaaaagctcagctaagaaacttacaaagtctctc 28012
human.FAM230E-LOC105377182-GGT3P.NCBI.                                            aaataagatctctgatatacagtctccaaaagctcagctaagaaacttacaaagtctctc 26746
human.FAM230J-LOC105372942-GGTLC5P.NCBI.rev.compl                                 aaataggatctctgatatacagtctccaaaagctcagccaagaaacttacaaagtctctc 27463
human.FAM230A.ncRNA.18487127.18500594-GGTLC3.18516335.18518165.rev.compl.REF      aaataagatctctgatatacagtctccaaaagctcagccaagaaacttacaaagtctctc 16222
                                                                                  ***** ******************************** ***********  ********
```

```
chimp.LOC112206744-LOC107973052-GGT2.revcompl                                    tgccttaacttcatccaccttttttctctccagcttctcctcggtagttaatgattataa 29437
chimp54546-135457.revcompl.Pan.troglodytes.clone.rp43-41g5.GenBank:AC099533.36   TGCCTTAACTTCATCCACCTTTTTTCTCTCCAGCTTCTCCTCGGTAGTTAATGATTATAA 31152
human.FAM30B-LOC105372935-GGT2.NCBI.                                             tgccttaacttcatccaccttttttctctccagcttctcctcggtagttaatgattataa 28072
human.FAM230E-LOC105377182-GGT3P.NCBI.                                           tgccttaacttcatccaccttttttctctccagcttttcctcggtagttaatgattataa 26806
human.FAM230J-LOC105372942-GGTLC5P.NCBI.rev.compl                                tgccttaacttcatccaccttttttctctccagcttctcctcggtagttaatgattataa 27523
human.FAM230A.ncRNA.18487127.18500594-GGTLC3.18516335.18518165.rev.compl.REF     tgccttaacttcatccaccttttttctctccagcttctcctcggtagttaatgattataa 16282
                                                                                 ************************************ ***********************

chimp.LOC112206744-LOC107973052-GGT2.revcompl                                    aaatatttattggctcatgcctgtaatcccagcactttgagaggccaaggcgggcagatc 29497
chimp54546-135457.revcompl.Pan.troglodytes.clone.rp43-41g5.GenBank:AC099533.36   AAATATTTATTGGCTCATGCCTGTAATCCCAGCACTTTGAGAGGCCAAGGCGGGCAGATC 31212
human.FAM30B-LOC105372935-GGT2.NCBI.                                             aaatatttattggctcatgcctgtaatcccagcactttgagaggccgaggcgggcagatc 28132
human.FAM230E-LOC105377182-GGT3P.NCBI.                                           aaatatttattggctcatgcctgtaatcccagcactttgagaggccgaggcgggcagatc 26866
human.FAM230J-LOC105372942-GGTLC5P.NCBI.rev.compl                                aaatatttattggctcatgcctgtaatcccagcactttgagaggccgaggcgggcagatc 27583
human.FAM230A.ncRNA.18487127.18500594-GGTLC3.18516335.18518165.rev.compl.REF     aaatatttattggctcatgcctgtaatcccagcactttgagaggccgaggcgggcagatc 16342
                                                                                 *********************************************** ************

chimp.LOC112206744-LOC107973052-GGT2.revcompl                                    acgaggtcaggagagcgagaccatcctggctaacacggtgaaatcccgtctctactaaaa 29557
chimp54546-135457.revcompl.Pan.troglodytes.clone.rp43-41g5.GenBank:AC099533.36   ACGAGGTCAGGAGATCGAGACCATCCTGGCTAACACGGTGAAATCCCATCTCTACTAAAA 31272
human.FAM30B-LOC105372935-GGT2.NCBI.                                             ac--ggtcaggagatcgagaccatcctggctaacacggtgaaatcccgtctctactaaaa 28190
human.FAM230E-LOC105377182-GGT3P.NCBI.                                           acgaggtcaggagattgagaccatcctggctaacacggtgaaatcccgtctctactaaaa 26926
human.FAM230J-LOC105372942-GGTLC5P.NCBI.rev.compl                                acgaggtcaggagatcgagaccatcctggctaacacggtgaaatcccgtctctactaaaa 27643
human.FAM230A.ncRNA.18487127.18500594-GGTLC3.18516335.18518165.rev.compl.REF     acgaggtcaggagatcgagaccatcctggctaacacggtgaaatcccgtctctactaaaa 16402
                                                                                 **  **********  ******************************* ************

chimp.LOC112206744-LOC107973052-GGT2.revcompl                                    atacaaaaaattagccaggcgtggtggcgggcacctgtaatcccagctactcgggaggct 29617
chimp54546-135457.revcompl.Pan.troglodytes.clone.rp43-41g5.GenBank:AC099533.36   ATACAAAAAATTAGCCAGGCGTGGTGGCGGGCGCCTGTAATCCCAGCTACTCGGGAGGCT 31332
human.FAM30B-LOC105372935-GGT2.NCBI.                                             atacaaaaaattagccaggcgtggtggcgggcgcctgtaatcccagctactcaggaggct 28250
human.FAM230E-LOC105377182-GGT3P.NCBI.                                           atacaaaaaattagccaggcgtggtggcgggcgcctgtaatcccagctactcaggaggct 26986
human.FAM230J-LOC105372942-GGTLC5P.NCBI.rev.compl                                atacaaaaaattagccaggcgtggtggcgggcgcctgtaatcccagctactcaggaggct 27703
human.FAM230A.ncRNA.18487127.18500594-GGTLC3.18516335.18518165.rev.compl.REF     atacaaaaaattagccaggcgtggtggcgggcgcctgtaatcccagctactcaggaggct 16462
                                                                                 ******************************** ******************* *******

chimp.LOC112206744-LOC107973052-GGT2.revcompl                                    gaggcaggagaatggcgtgaacccacaaggcggagcttgcaatgaggtgagatcccacta 29677
chimp54546-135457.revcompl.Pan.troglodytes.clone.rp43-41g5.GenBank:AC099533.36   GAGGCAGGAGAATGGCGTGAACCCACAAGGCGGAGCTTGCAATGAGGTGAGATCCCACTA 31392
human.FAM30B-LOC105372935-GGT2.NCBI.                                             gaggcaggagaatggcgtgaacccacaaggcggagcttgcaatgaggtgagatcccacta 28310
human.FAM230E-LOC105377182-GGT3P.NCBI.                                           gaggcaggagaatggcatgtacccacaaggcggagcttacaatgaggtgagatcccacta 27046
human.FAM230J-LOC105372942-GGTLC5P.NCBI.rev.compl                                gaggcaggagaatggcgtgaacccacaaggcggagcttgcaatgaggtgagatcccacta 27763
human.FAM230A.ncRNA.18487127.18500594-GGTLC3.18516335.18518165.rev.compl.REF     gaggcaggagaatggcatgtacccacaaggcggagcttgcaacgaggtgagatcccacta 16522
                                                                                 **************** ** ****************** *** *****************

chimp.LOC112206744-LOC107973052-GGT2.revcompl                                    ctgcactccagcctgggcgacagagcaagactccatctca----------------aaac 29721
chimp54546-135457.revcompl.Pan.troglodytes.clone.rp43-41g5.GenBank:AC099533.36   CTGCACTCCAGCCTGGGCGACAGAGCAAGACTCCATCTCAAAACAAACAAACAAACAAAC 31452
human.FAM30B-LOC105372935-GGT2.NCBI.                                             ctgcactccagcctgggcgacagagcaagactccatctca--------aaacaaacaaac 28362
human.FAM230E-LOC105377182-GGT3P.NCBI.                                           ctgcactccagcctgggcgacagagcaagactccatctca--------aaacaaacaaac 27098
human.FAM230J-LOC105372942-GGTLC5P.NCBI.rev.compl                                ctgcactccagcctgagcgacagagcaagactccatctca--------aaacaaacaaac 27815
human.FAM230A.ncRNA.18487127.18500594-GGTLC3.18516335.18518165.rev.compl.REF     ctgcactccagcctgggcgacagagcaagactccatctca--------aaacaaacaaac 16574
                                                                                 *************** ************************                ****

chimp.LOC112206744-LOC107973052-GGT2.revcompl                                    aaacaaacaaacaaaaaaacagtgtgatggccaggcgcggtgctcaagcctataatccaa 29781
chimp54546-135457.revcompl.Pan.troglodytes.clone.rp43-41g5.GenBank:AC099533.36   AAACAAACAAACAAAAAAACAGTGTGATGGCCAGGCGCGGTGCTCATGCCTATAATCCAA 31512
human.FAM30B-LOC105372935-GGT2.NCBI.                                             aaacaaa----caaaaaaacagtgtgatggccaggcgcagtgctcatgcctataatccaa 28418
human.FAM230E-LOC105377182-GGT3P.NCBI.                                           aaacaaa----caaaaaaacagtgtgatggccaggcgcagtgctcatgcctataatccaa 27154
human.FAM230J-LOC105372942-GGTLC5P.NCBI.rev.compl                                aaacaaacaaccaaaaaaacagtgtgatggccaggcgcagtgctcatgcctataatccaa 27875
human.FAM230A.ncRNA.18487127.18500594-GGTLC3.18516335.18518165.rev.compl.REF     aaacaaa----caaaaaaacagtgtgatggccaggcgcagtgctcatgcctataatccaa 16630
                                                                                 *******    *************************** ******* *************

chimp.LOC112206744-LOC107973052-GGT2.revcompl                                    gcactttgggaggctgaaatggatcgatggcttgagcccagtagtttgagacaagcctgg 29841
chimp54546-135457.revcompl.Pan.troglodytes.clone.rp43-41g5.GenBank:AC099533.36   GCACTTTGGGAGGCTGAAATGGATGGATGGCTTGAGCCCAGTAGTTTGAGACAAGCCTGG 31572
human.FAM30B-LOC105372935-GGT2.NCBI.                                             gcactttgggaggctgaaatggatggatggcttgagcccagtagtttgagacaagcctgg 28478
human.FAM230E-LOC105377182-GGT3P.NCBI.                                           gcactttgggaggctgaaatggatggatggcttgagcccagtagtttgagacaagcctgg 27214
human.FAM230J-LOC105372942-GGTLC5P.NCBI.rev.compl                                gcactttgggaggctgaaatggatggatggcttgagcccagtagtttgagacaagcctgg 27935
human.FAM230A.ncRNA.18487127.18500594-GGTLC3.18516335.18518165.rev.compl.REF     gcactttgggaggctgaaatggatggatggcttgagcccagtagtttgagacaagcctgg 16690
                                                                                 ************************ ***********************************

chimp.LOC112206744-LOC107973052-GGT2.revcompl                                    caacatagcgagacctcatctctacaaacatttttaaaatatgccaggcatggtgtgcag 29901
chimp54546-135457.revcompl.Pan.troglodytes.clone.rp43-41g5.GenBank:AC099533.36   CAACATAGCGAGACCTCATCTCTACAAACATTTTTAAAATATGCCAGGCATGGTG----- 31627
human.FAM30B-LOC105372935-GGT2.NCBI.                                             caacatagcgagacctcatctctacaaacatctttaaaatatgccaggcatggtg----- 28533
human.FAM230E-LOC105377182-GGT3P.NCBI.                                           caacatagcgagacctcatctctacaaacatctttaaaatatgccaggcatggtg----- 27269
human.FAM230J-LOC105372942-GGTLC5P.NCBI.rev.compl                                caacatagcgagacctcatgtctacaaacatctttaaaatatgccaggcatggtg----- 27990
human.FAM230A.ncRNA.18487127.18500594-GGTLC3.18516335.18518165.rev.compl.REF     caacatagcgagacctcatctctacaaacatctttaaaatatgccaggcatggtg----- 16745
                                                                                 ******************* *********** ***********************

chimp.LOC112206744-LOC107973052-GGT2.revcompl                                    actgcatgcctgtagtcccagctattcgggaggctgaggtgggaggatcacctgtgcccg 29961
chimp54546-135457.revcompl.Pan.troglodytes.clone.rp43-41g5.GenBank:AC099533.36   -GTGCATGCCTGTAGTCCCAGCTATTCAGGAGGCTGAGGTGGGAGGATCACCTGTGCCTG 31686
human.FAM30B-LOC105372935-GGT2.NCBI.                                             -gtgcatgcctgtagtcccagctattcaggaggctgaggtgggaggatcacctgtgcccg 28592
human.FAM230E-LOC105377182-GGT3P.NCBI.                                           -gtgcatgcctgtagtcccagctattcaggaggctgaggtgggaggatcacctgtgcccg 27328
human.FAM230J-LOC105372942-GGTLC5P.NCBI.rev.compl                                -gtgcatgcctgtagtcccagctattcaggaggctgaggtgggaggatcacctgtgcccg 28049
human.FAM230A.ncRNA.18487127.18500594-GGTLC3.18516335.18518165.rev.compl.REF     -gtgcatgcctgtagtcccagctattcaggaagctgaggtgggaggatcacctgtgcccg 16804
                                                                                   ************************* *** ************************** *

chimp.LOC112206744-LOC107973052-GGT2.revcompl                                    ggagttcaaggctgcagtgagctatgatcacaccacagtgctccagcctgggcaacaaag 30021
chimp54546-135457.revcompl.Pan.troglodytes.clone.rp43-41g5.GenBank:AC099533.36   GGAGTTCAAGGCTGCAGTGAGCTATGATCACACCACAGTGCTCCAGCCTGGGCAACAAAG 31746
human.FAM30B-LOC105372935-GGT2.NCBI.                                             ggagttcaaggctgcagtgagctatgatcacaccacagtgctccagcctgggcaacaaag 28652
human.FAM230E-LOC105377182-GGT3P.NCBI.                                           ggagttcaaggctgcagtgagctatgatcacaccacagtgctccagcctgggcaacaaag 27388
human.FAM230J-LOC105372942-GGTLC5P.NCBI.rev.compl                                ggagttcaaggctgcagtgagctatgatcacaccacagtgctccagcctgggcaacaaag 28109
human.FAM230A.ncRNA.18487127.18500594-GGTLC3.18516335.18518165.rev.compl.REF     ggagttcaaggctgcagtgagctatgatcacaccacagtgctccagcctgggcaacaaag 16864
                                                                                 ************************************************************

chimp.LOC112206744-LOC107973052-GGT2.revcompl                                    caagactccatctctaaaaataaaataaaatt--aaagaaaagatctttgctgtaaaaga 30079
chimp54546-135457.revcompl.Pan.troglodytes.clone.rp43-41g5.GenBank:AC099533.36   CAAGACTCCATCTCTAAAAATAAAATAAAATT--AAAAAAAAGATCTTCGCTGTAAAAGA 31804
human.FAM30B-LOC105372935-GGT2.NCBI.                                             caagactccatctctaaaaataaaataaaattt-aaaaaaaagatcttcgctgtaaaaga 28711
human.FAM230E-LOC105377182-GGT3P.NCBI.                                           caagactccatctctaaaaataaaataaaatt-aaaaaaaaagatcttcgctgtaaaaga 27447
human.FAM230J-LOC105372942-GGTLC5P.NCBI.rev.compl                                caagactccatctctaaaaataaaataaaatt-aaaaaaaaagatcttcgctgtaaaaga 28168
human.FAM230A.ncRNA.18487127.18500594-GGTLC3.18516335.18518165.rev.compl.REF     caagactccatctctaaaaataaaataaaattaaaaaaaaaaagatcttcgctgtaaaaga 16924
                                                                                 ********************************  *** ********** ***********

chimp.LOC112206744-LOC107973052-GGT2.revcompl                                    ggtacgctcaaatgcaataaaagcatataagaaggccgggtgtggtggctcatgcctgta 30139
chimp54546-135457.revcompl.Pan.troglodytes.clone.rp43-41g5.GenBank:AC099533.36   GGTACGCTCAAATGCAATAAAAGCATATAAGAAGGCCGGGTGTAGTGGCTCATGCCTGTA 31864
human.FAM30B-LOC105372935-GGT2.NCBI.                                             ggtacgctcaaatgcaataaaagcatataagaaggccgggtgtggtggctcatgcctgta 28771
human.FAM230E-LOC105377182-GGT3P.NCBI.                                           ggtacgctcaaatgcaataaaagcatataagaaggccgggtgtggtggctcatgcctgta 27507
human.FAM230J-LOC105372942-GGTLC5P.NCBI.rev.compl                                ggtacgctcaaatgcaataaaagcatataagaaggccgggtgtggtggctcatgcctgta 28228
human.FAM230A.ncRNA.18487127.18500594-GGTLC3.18516335.18518165.rev.compl.REF     ggtacactcaaatgcaataaaagcatataagaaggccgggtgtggtggctcatgcctgta 16984
                                                                                 ***** ************************************* ****************

chimp.LOC112206744-LOC107973052-GGT2.revcompl                                    atctcagcactttgggaggccaagacgggcggatcacgaggtcaggagatcgagactatc 30199
chimp54546-135457.revcompl.Pan.troglodytes.clone.rp43-41g5.GenBank:AC099533.36   ATCTCAGCACTTTGGGAGGCCGAGACGGGCGGATCACGAGGTCAGGAGATCGAGACTATC 31924
human.FAM30B-LOC105372935-GGT2.NCBI.                                             atcccagcactttgggaggccgagacgggcggatcacgaggtcaggagattgagactatc 28831
human.FAM230E-LOC105377182-GGT3P.NCBI.                                           atcccagcactttgggaggccgagacgggcggatcacgaggtcaggagattgagactatc 27567
human.FAM230J-LOC105372942-GGTLC5P.NCBI.rev.compl                                atcccagcactttgggaggccgagacgggcggatcacgaggtcaggagattgagactatc 28288
human.FAM230A.ncRNA.18487127.18500594-GGTLC3.18516335.18518165.rev.compl.REF     atcccagcactttgggaggccgagacgggcggatcacgaggtcaggagattgagactatc 17044
                                                                                 *** ***************** **************************** *********

chimp.LOC112206744-LOC107973052-GGT2.revcompl                                    ctggctaacgcggagaaaccccatctcctctaaaagtacaaaaaaattagttgggctagg 30259
chimp54546-135457.revcompl.Pan.troglodytes.clone.rp43-41g5.GenBank:AC099533.36   CTGGCTAACACGGTGAAACCCCATCTCCTCTAAAAGTACAAAAAAATTAGTTGGGCTAGG 31984
human.FAM30B-LOC105372935-GGT2.NCBI.                                             ctggctaacgcggtgaaaccccatctcctctaaaagtacaaaaaaattagctgggctagg 28891
human.FAM230E-LOC105377182-GGT3P.NCBI.                                           ctggctaacgcggtgaaaccccatctcctctaaaagtacaaaaaaattagctgggctagg 27627
human.FAM230J-LOC105372942-GGTLC5P.NCBI.rev.compl                                ctggctaacgcggtgaaaccccatctcctctaaaagtacaaaaaaattagctgggctagg 28348
human.FAM230A.ncRNA.18487127.18500594-GGTLC3.18516335.18518165.rev.compl.REF     ctggctaacgcggtgaaaccccatctcctctaaaagtacaaaaaaattagctgggctagg 17104
                                                                                 ********* *** ************************************ *********

chimp.LOC112206744-LOC107973052-GGT2.revcompl                                    tggcaggcgcctgtagtcccagctactcaggaggctgaggcaggagaatggcataaaccc 30319
chimp54546-135457.revcompl.Pan.troglodytes.clone.rp43-41g5.GenBank:AC099533.36   TGGCAGGCGCCTGTAGTCCCAGCTACTCAGGAGGCTGAGGCAGGAGAATGGCATAAACCC 32044
human.FAM30B-LOC105372935-GGT2.NCBI.                                             tggcaggcgcctgtagtcccagctactgaggaggctgaggcaggagaatggcataaacca 28951
human.FAM230E-LOC105377182-GGT3P.NCBI.                                           tggcaggcgcctgtagtcccagctactcaggaggctgaggcaggagaatggcataaaccc 27687
human.FAM230J-LOC105372942-GGTLC5P.NCBI.rev.compl                                tggcaggcgcctgtagtcccagctactcaggaggctgaggcaggagaatggcataaaccc 28408
human.FAM230A.ncRNA.18487127.18500594-GGTLC3.18516335.18518165.rev.compl.REF     tggcaggcgcctgtagtcccagctactcaggaggctgaggcaggagaatggcataaaccc 17164
                                                                                 *************************** *******************************

chimp.LOC112206744-LOC107973052-GGT2.revcompl                                    gggaggcagagcttgcagtgagccaagatcgcaccactgcactccagcctgggtgacaga 30379
chimp54546-135457.revcompl.Pan.troglodytes.clone.rp43-41g5.GenBank:AC099533.36   GGGAGGCAGAGCTTGCAGTGAGCCAAGATCGCACCACTGCACTCCACCCTGGGTGACAGA 32104
human.FAM30B-LOC105372935-GGT2.NCBI.                                             gggaggcagagcttgcagtgagcctagatcacaccactgcactccagcctgggtgacaga 29011
human.FAM230E-LOC105377182-GGT3P.NCBI.                                           gggaggcagagcttgcaatgagcctagatcgcaccactgcactccagcctgggtgacaga 27747
human.FAM230J-LOC105372942-GGTLC5P.NCBI.rev.compl                                gggaggcagagcttgcagtgagcctagatcgcaccactgcactccagcctgggtgacaga 28468
human.FAM230A.ncRNA.18487127.18500594-GGTLC3.18516335.18518165.rev.compl.REF     gggaggcagagcttgcagtgagcctagatagcaccactgccctccagcctgggtgacaga 17224
                                                                                 ***************** ****** ****  ********* ***** *************

chimp.LOC112206744-LOC107973052-GGT2.revcompl                                    gcgagactccgtctcaaaaaaaaaaaaa--aaaagaaaaagaaaagaaaagttcttgtga 30437
chimp54546-135457.revcompl.Pan.troglodytes.clone.rp43-41g5.GenBank:AC099533.36   GCGAGACTCCGTCTCAAAAAAA--------AAAAGAAAAAGAAAAGAAAAGTTCTTGTGA 32156
human.FAM30B-LOC105372935-GGT2.NCBI.                                             gcgagactccgtctcaaaaaaaaaaaaaaaaaaagaaaaagaaaagaaaagttcttctga 29071
human.FAM230E-LOC105377182-GGT3P.NCBI.                                           gtgagactccgtctc-aaaaaaaaaaaaa-aaaagaaaaagaaaagaaaagttcttctga 27805
human.FAM230J-LOC105372942-GGTLC5P.NCBI.rev.compl                                gtgagactccgtctc---aaaaaaaaaaa-aaaagaaaaagaaaagaaaagttcttgtga 28524
human.FAM230A.ncRNA.18487127.18500594-GGTLC3.18516335.18518165.rev.compl.REF     gcgagactccgtctcaaaaaaaaaaaaaa-aaaagaaaaagaaaagaaaagttcttgtga 17283
                                                                                 * *************   ****        ************************** ***
```

```
chimp.LOC112206744-LOC107973052-GGT2.revcompl                                     catttgtgtatgaaatcagccttcactacatggataggaccagcacgcttccgcggcacg 30497
chimp54546-135457.revcompl.Pan.troglodytes.clone.rp43-41g5.GenBank:AC099533.36    CATTTGTGTATGAAATCAGCCTTCACTACATGGATAGGACCAGCACGCTTCCATGGCACG 32216
human.FAM30B-LOC105372935-GGT2.NCBI.                                              catttgtgtatgaaatcagccttcactacatggataggaccagcacgcttctgcggcacg 29131
human.FAM230E-LOC105377182-GGT3P.NCBI.                                            catttgtgtatgaaatcagccttcactacatggataggaccagcacgcttctgcggcacg 27865
human.FAM230J-LOC105372942-GGTLC5P.NCBI.rev.compl                                 catttgtgtatgaaatcagccttcactacatggataggaccagcacgcttctgcggcacg 28584
human.FAM230A.ncRNA.18487127.18500594-GGTLC3.18516335.18518165.rev.compl.REF      catttgtgtatgaaatcagccttcactacatggataggaccagcacgcttctgcggcacg 17343
                                                                                  ***************************************************   ******

chimp.LOC112206744-LOC107973052-GGT2.revcompl                                     actctgcaatcttactacatttttttttactttgtattttatttattccttttgagacag 30557
chimp54546-135457.revcompl.Pan.troglodytes.clone.rp43-41g5.GenBank:AC099533.36    ACTCTGCAATCTTACTACATTTTTTTTTACTTTGTATTTTATTTATTCCTTTTGAGACAG 32276
human.FAM30B-LOC105372935-GGT2.NCBI.                                              actctgcaatcttactacatttttttttactttgtattttatttattccttttgagacag 29191
human.FAM230E-LOC105377182-GGT3P.NCBI.                                            actctgcaatcttactacatttttttttactttgtattttatttattccttttgagacag 27925
human.FAM230J-LOC105372942-GGTLC5P.NCBI.rev.compl                                 actctgcaatcttactacatttttttttactttgtattttatttattccttttgagacag 28644
human.FAM230A.ncRNA.18487127.18500594-GGTLC3.18516335.18518165.rev.compl.REF      actctgcaatcttactacatttttttttactttgtattttatttattccttttgagacag 17403
                                                                                  ************************************************************

chimp.LOC112206744-LOC107973052-GGT2.revcompl                                     agtctcactctgtcacccaggctgaagtgcagccgagatctcagctcactgcaacctcca 30617
chimp54546-135457.revcompl.Pan.troglodytes.clone.rp43-41g5.GenBank:AC099533.36    AGTCTCACTCTGTCACCCAGGCTGAAGTGCAGCCAAGATCTCGGCTCACTGCAACCTCCA 32336
human.FAM30B-LOC105372935-GGT2.NCBI.                                              agtctcactctgtcacccaggctgaagtgcagccgagatctcggctcactgcaacctcca 29251
human.FAM230E-LOC105377182-GGT3P.NCBI.                                            agtctcactctgtcacccaggctgaagtgcagccgagatctcggctcactgcaacctcca 27985
human.FAM230J-LOC105372942-GGTLC5P.NCBI.rev.compl                                 agtctcactctgtcacccaggctgaagtgcagccgagatctcggctcactgcaacctcca 28704
human.FAM230A.ncRNA.18487127.18500594-GGTLC3.18516335.18518165.rev.compl.REF      agtctcactctgtcacccaggctgaagtgcagccgagatctcggctcactgcaacctcca 17463
                                                                                  ********************************** ******* *****************

chimp.LOC112206744-LOC107973052-GGT2.revcompl                                     cctcctgggttcaagcaattctcttgtctcagcctcccaagtagctgggactacaggcac 30677
chimp54546-135457.revcompl.Pan.troglodytes.clone.rp43-41g5.GenBank:AC099533.36    CCTCCTGGGTTCAAGCAATTCTCTTGTCTCAGCCTCCCAAGTAGCTGGGACTACAGGCAC 32396
human.FAM30B-LOC105372935-GGT2.NCBI.                                              cctcttgggttcaagcaattctcttgtctcagcctcccaagtagctgggactacaggcac 29311
human.FAM230E-LOC105377182-GGT3P.NCBI.                                            cctcttgggttcaagcaattctcttgtctcagcctcccaagtagctgggactacaggcac 28045
human.FAM230J-LOC105372942-GGTLC5P.NCBI.rev.compl                                 cctcttgggttcaagcaattctcttgtctcagcctcccaagtagctgggactacaggcac 28764
human.FAM230A.ncRNA.18487127.18500594-GGTLC3.18516335.18518165.rev.compl.REF      cctcttgggttcaagcaattctcttgtctcagcctcccaagtagctgggactacaggcac 17523
                                                                                  **** *******************************************************

chimp.LOC112206744-LOC107973052-GGT2.revcompl                                     acgtcaaaacgcccggctaatttttgtatttttagtagagatggagtttagccatattgg 30737
chimp54546-135457.revcompl.Pan.troglodytes.clone.rp43-41g5.GenBank:AC099533.36    ACGTCAAAACGCCCGGCTAATTTTTGTATTTTTAGTAGAGATGGAGTTTTGCCATATTGG 32456
human.FAM30B-LOC105372935-GGT2.NCBI.                                              acgtcaaaacgcccggctaatttttgtatttttagtagagatggagttttgccatattgg 29371
human.FAM230E-LOC105377182-GGT3P.NCBI.                                            acgtcaaaacgcccggctaatttttgtatttttagtagagatggagttttgccctattgg 28105
human.FAM230J-LOC105372942-GGTLC5P.NCBI.rev.compl                                 acgtcaaaacgcccagctaatttttgtatttttagtagagatggagttttgccatattgg 28824
human.FAM230A.ncRNA.18487127.18500594-GGTLC3.18516335.18518165.rev.compl.REF      acgtcaaaacgcccggctaatttttgtatttttagtagagatggagttttgccatattgg 17583
                                                                                  ************** ********************************** *** ******

chimp.LOC112206744-LOC107973052-GGT2.revcompl                                     tcaggctggtctcgaactcctgacctcaggtgatcgacctgtcttagcctcccaaagtgc 30797
chimp54546-135457.revcompl.Pan.troglodytes.clone.rp43-41g5.GenBank:AC099533.36    TCAGGCTGGTCTCGAATTCCTGACCTCAGGTGATTGACCTGTCTTAGCCTCCCAAAGTGC 32516
human.FAM30B-LOC105372935-GGT2.NCBI.                                              tcaggctggtctcgaactcctgacctcaggtgatcgacctgtcttagcctcccaaagtgc 29431
human.FAM230E-LOC105377182-GGT3P.NCBI.                                            tcaggctggtctcaaactcctgacctcaggtgatcgacctgtcttagcctcccaaagtgc 28165
human.FAM230J-LOC105372942-GGTLC5P.NCBI.rev.compl                                 tcaggctggtctcgaactcctgacgtcaggtgatcgacctgtcttagcctcccaaagtgc 28884
human.FAM230A.ncRNA.18487127.18500594-GGTLC3.18516335.18518165.rev.compl.REF      tcaagctggtctcgaactcctgacgtcaggtgatcgacctgtcttagcctcccaaagtgc 17643
                                                                                  *** ********* ** ******* ********* *************************

chimp.LOC112206744-LOC107973052-GGT2.revcompl                                     taggattacaggtgtacatttatttatttatttgagatggaatcctgctctgtatttatt 30857
chimp54546-135457.revcompl.Pan.troglodytes.clone.rp43-41g5.GenBank:AC099533.36    TAGGATTACAGGTGTACATTTATTTATTTATTTGAGATAGAATCTTGCTCTGTATTTATT 32576
human.FAM30B-LOC105372935-GGT2.NCBI.                                              taggattacaggtgtacatttatttatttatttgagatggaatcttgctctgtatttatt 29491
human.FAM230E-LOC105377182-GGT3P.NCBI.                                            taggattacaggtgtacatttatttatttatttgagatggaatcttgctctgtatttatt 28225
human.FAM230J-LOC105372942-GGTLC5P.NCBI.rev.compl                                 taggattacaggtgtacatttatttatttatttgagatggaatcttgctctgtatttatt 28944
human.FAM230A.ncRNA.18487127.18500594-GGTLC3.18516335.18518165.rev.compl.REF      taggattacaggtgtacatttatttatttatttgagatggaatcttgctctgtatttatt 17703
                                                                                  ************************************** ***** ***************

chimp.LOC112206744-LOC107973052-GGT2.revcompl                                     aatttatttatttgagatggagtcttgctccattgcccaggctagagtgcagtggtgcaa 30917
chimp54546-135457.revcompl.Pan.troglodytes.clone.rp43-41g5.GenBank:AC099533.36    AATTTATTTATTTGAGATGGAGTCTTGCTCCATCGCCCAGGCTAGAGTGCAGTGGTGCAA 32636
human.FAM30B-LOC105372935-GGT2.NCBI.                                              aatttatttatttgagatggagtcttgctccatcgcccaggctagagtgcagcggtgcaa 29551
human.FAM230E-LOC105377182-GGT3P.NCBI.                                            aatttatttatttgagatggagtcttgctccatcgcccaggctagagtgcagcagtgcaa 28285
human.FAM230J-LOC105372942-GGTLC5P.NCBI.rev.compl                                 aatttatttagttgagatggagtcttgctccatcgcccaggctagagtgcagcggtgcaa 29004
human.FAM230A.ncRNA.18487127.18500594-GGTLC3.18516335.18518165.rev.compl.REF      aatttatttatttgagatggagtcttgctccatcgcccaggctagagtgcagcggtgcaa 17763
                                                                                  ********** ********************** ******************  ******

chimp.LOC112206744-LOC107973052-GGT2.revcompl                                     tctcggctcattgcaacctctgacttccaggttcaagcgattctcctgcctcagtgtccc 30977
chimp54546-135457.revcompl.Pan.troglodytes.clone.rp43-41g5.GenBank:AC099533.36    TCTCAGCTCATTGCAACCTCTGCCTTCCAGGTTCAAGCGATTCTCCTGCCTCAGTGTCCC 32696
human.FAM30B-LOC105372935-GGT2.NCBI.                                              tctcggctcattgcaacctctgccttccaggttcaagcgattctcctgcctcagtgtccc 29611
human.FAM230E-LOC105377182-GGT3P.NCBI.                                            tctcggctcattgcaacctctgccttccaggttcaagcgattctcctgcctcagtgtccc 28345
human.FAM230J-LOC105372942-GGTLC5P.NCBI.rev.compl                                 tctcggctcattgcaacctctgccttccaggttcaagcgattctcctgcctcagtgtccc 29064
human.FAM230A.ncRNA.18487127.18500594-GGTLC3.18516335.18518165.rev.compl.REF      tctcggctcattgcaacctctgccttccaggttcaagcgattctcctgcctcagtgtccc 17823
                                                                                  **** ***************** *************************************

chimp.LOC112206744-LOC107973052-GGT2.revcompl                                     aagtagctgggattacaggtgcctgccaccacagctggttaatttttgtatttttagtag 31037
chimp54546-135457.revcompl.Pan.troglodytes.clone.rp43-41g5.GenBank:AC099533.36    AAGTAGCTGGGATTACAGGTGCCTGCCACCACAGCTGGCTAATTTTTGTATTTTTAGTAG 32756
human.FAM30B-LOC105372935-GGT2.NCBI.                                              aagtagctgggattacaggtgcctgccaccacagctggctaatttttgtatttttagtag 29671
human.FAM230E-LOC105377182-GGT3P.NCBI.                                            aagtagctgggattacaggtgcctgccaccacagctggctaatttttgtatttttagtag 28405
human.FAM230J-LOC105372942-GGTLC5P.NCBI.rev.compl                                 aagtagctgggattacaggtgcctgccaccacagctggctaatttttgtatttttagtag 29124
human.FAM230A.ncRNA.18487127.18500594-GGTLC3.18516335.18518165.rev.compl.REF      aagtagctgggattacaggtgcctgccaccacagctggctaatttttgtatttttagtag 17883
                                                                                  ************************************** *********************

chimp.LOC112206744-LOC107973052-GGT2.revcompl                                     agacagtgtttcaccatcttggccaggctggtctcgggctcctgacctcatgaaccacct 31097
chimp54546-135457.revcompl.Pan.troglodytes.clone.rp43-41g5.GenBank:AC099533.36    AGACAGTGTTTCACCATCTTGGCCAGGCTGGTCTCAGGCTCCTGACCTCATGAACCACCT 32816
human.FAM30B-LOC105372935-GGT2.NCBI.                                              agacagtgtttcaccatcttggccaggctggtctcgggctcctgacctcatgaaccacct 29731
human.FAM230E-LOC105377182-GGT3P.NCBI.                                            agacagtgtttcaccatcttggccaggctggtctcgggctcctgacctcatgaaccacct 28465
human.FAM230J-LOC105372942-GGTLC5P.NCBI.rev.compl                                 agacagtgtttcaccatcttggccaggctggtctcgggctcctgacctcatgaaccacct 29184
human.FAM230A.ncRNA.18487127.18500594-GGTLC3.18516335.18518165.rev.compl.REF      agacagtgtttcaccatcttggccaggctggtctcgggctcctgacctcatgaaccacct 17943
                                                                                  *********************************** ************************

chimp.LOC112206744-LOC107973052-GGT2.revcompl                                     gcctcagcctcccaaagtgttgggattacaggcctaaggcaccatgttcggccatattta 31157
chimp54546-135457.revcompl.Pan.troglodytes.clone.rp43-41g5.GenBank:AC099533.36    GCCTCAGCCTCCCAAAGTGTTGGGATTACAGGCCTAAGGCACCATGCTCGGCCATATTTA 32876
human.FAM30B-LOC105372935-GGT2.NCBI.                                              gcctcagcctcccaaagtgttgggattacaggcctaaggcaccatgctcggccatattta 29791
human.FAM230E-LOC105377182-GGT3P.NCBI.                                            gcctcagcctcccaaagtgttgggattacaggcctaaggcaccatgctcggccatattta 28525
human.FAM230J-LOC105372942-GGTLC5P.NCBI.rev.compl                                 gcctcagcctcccaaaatgttgggattacaggcctaaggcaccatgctcggccatattta 29244
human.FAM230A.ncRNA.18487127.18500594-GGTLC3.18516335.18518165.rev.compl.REF      gcctcagcctcccaaagtgttgggattacaggcctaaggcaccatgctcggccatattta 18003
                                                                                  **************** ***************************** *************

chimp.LOC112206744-LOC107973052-GGT2.revcompl                                     tttatttaattatttagagacaaagtcttgctctgtcacccaggctggagtgcagtggcg 31217
chimp54546-135457.revcompl.Pan.troglodytes.clone.rp43-41g5.GenBank:AC099533.36    TTTATTTAATTATTTAGAGACAAAGTCTTGCTCTGTCACCCAGGCTGGAGTGCAGTGGCG 32936
human.FAM30B-LOC105372935-GGT2.NCBI.                                              tttatttaattatttagagacaaagtcttgctctgtcacccaggctggagtgcagtggcg 29851
human.FAM230E-LOC105377182-GGT3P.NCBI.                                            tttatttaattatttagagacaaagtcttgctctgtcacccaggctggagtgcagtggcg 28585
human.FAM230J-LOC105372942-GGTLC5P.NCBI.rev.compl                                 tttatttaattatttagagacaaagtcttgctctgtcacccaggctggagtgcagtggcg 29304
human.FAM230A.ncRNA.18487127.18500594-GGTLC3.18516335.18518165.rev.compl.REF      tttatttaattatttagagacaaagtcttgctctgtcacccaggctggagtgcagtggcg 18063
                                                                                  ************************************************************

chimp.LOC112206744-LOC107973052-GGT2.revcompl                                     ccatctcagctcactgcagcctccgcctctgaggtttaagcgattctcatgcctcagcct 31277
chimp54546-135457.revcompl.Pan.troglodytes.clone.rp43-41g5.GenBank:AC099533.36    CCATCTCAGCTCACTGCAGCCTCCGCCTCTGAGGTTTAAGCGATTCTCATGCCTCAGCCT 32996
human.FAM30B-LOC105372935-GGT2.NCBI.                                              ccatctcagctcactgcagcctccgcctccgaggtttaagcaattctcatgcctcagcct 29911
human.FAM230E-LOC105377182-GGT3P.NCBI.                                            ccatctcagctcactgcagcctccgcctccaaggtttaagcaattctcatgcctcagact 28645
human.FAM230J-LOC105372942-GGTLC5P.NCBI.rev.compl                                 ccatctcagctcactgcagcctccgcctccgaggtttaagcaattctcatgcctcagact 29364
human.FAM230A.ncRNA.18487127.18500594-GGTLC3.18516335.18518165.rev.compl.REF      ccatctcagctcactgcagcctccgcctccgaggtttaagcaattctcatgcctcagcct 18123
                                                                                  *****************************  ********** *************** **

chimp.LOC112206744-LOC107973052-GGT2.revcompl                                     cctgaataactaggactacagatactcgccaccacgcagggatttttttttttctatttt 31337
chimp54546-135457.revcompl.Pan.troglodytes.clone.rp43-41g5.GenBank:AC099533.36    CCTGAGTAACTAGGACTACAGATACTCGCCACCACACAGGGA-TTTTTTTTTCTATTTT 33055
human.FAM30B-LOC105372935-GGT2.NCBI.                                              cctgagtaactgggactacagatacttgccaccacgcaggga--ttttttttctatttt 29969
human.FAM230E-LOC105377182-GGT3P.NCBI.                                            cctgagtaactgggactacagaaacttgccaccacgcaggga--ttttttttctatttt 28703
human.FAM230J-LOC105372942-GGTLC5P.NCBI.rev.compl                                 cctgagtaactgggactacagatacttgccaccacgcaggga-tttttttttctatttt 29423
human.FAM230A.ncRNA.18487127.18500594-GGTLC3.18516335.18518165.rev.compl.REF      cctgagtaactgggactacagatacttgccaccacgcaggga-tttttttttctattttt 18182
                                                                                  ***** ***** ********** *** ******** ******  ********    ****

chimp.LOC112206744-LOC107973052-GGT2.revcompl                                     tttgtagagacacagtttca-ccatgttggccaggctggtctcgaactcctgaccttagg 31396
chimp54546-135457.revcompl.Pan.troglodytes.clone.rp43-41g5.GenBank:AC099533.36    TTTGTAGAGACACAGTTTCACCCATGTTGGCCAGGCTGGTCTCGAACTCCTGACCTTAGG 33115
human.FAM30B-LOC105372935-GGT2.NCBI.                                              tttgtagagacacagtttca-ccatgttggccaggctggtctcgaactcctgaccttagg 30028
human.FAM230E-LOC105377182-GGT3P.NCBI.                                            tttgtagagacacagtttca-ccatgttggccaggctggtctcgaactcctgactttagg 28762
human.FAM230J-LOC105372942-GGTLC5P.NCBI.rev.compl                                 tttgtagagacacagtttca-ccatgttggccaggctggtctcgaactcctgaccttagg 29482
human.FAM230A.ncRNA.18487127.18500594-GGTLC3.18516335.18518165.rev.compl.REF      tttgtagagacacagtttca-ccatgttggccaggctggtctcgaactcctgaccttagg 18241
                                                                                  ******************** ********************************* *****

chimp.LOC112206744-LOC107973052-GGT2.revcompl                                     tgatctgacagcctcgtcctctctaagtactgggattacaggcatgagccccttgcccgg 31456
chimp54546-135457.revcompl.Pan.troglodytes.clone.rp43-41g5.GenBank:AC099533.36    TGATCTGACAGCCTCGTCCTCTCTAAGTACTGGGATTACAGGCATGAGCCCCTTGCCCGG 33175
human.FAM30B-LOC105372935-GGT2.NCBI.                                              tgatctgacagcctcgtcctctcaaagcactgggattacaggcatgagccccttgcccgg 30088
human.FAM230E-LOC105377182-GGT3P.NCBI.                                            tgatctgacagcctcgtcctctcaaagcactgggattacaggcatgagccccttgcccgg 28822
human.FAM230J-LOC105372942-GGTLC5P.NCBI.rev.compl                                 tgatctgacagcctcgtcctctcaaagcactgggattacaggcatgagccccttgcccgg 29542
human.FAM230A.ncRNA.18487127.18500594-GGTLC3.18516335.18518165.rev.compl.REF      tgatctgacagcctcgtcctctcaaagcactgggattacaggcatgagccccttgcccgg 18301
                                                                                  *********************** *** ********************************

chimp.LOC112206744-LOC107973052-GGT2.revcompl                                     cctctcactacatttaagtgacgccatggctcatgcctgtaatcctagcactttgggagg 31516
chimp54546-135457.revcompl.Pan.troglodytes.clone.rp43-41g5.GenBank:AC099533.36    CCTCTCACTACATTTAAGTGACGCCATGGCTCATGCCTGTAATCCTAGCACTTTGGGAGG 33235
human.FAM30B-LOC105372935-GGT2.NCBI.                                              cctctcactacatttaagtgacgccatggctcatgcctgtaatcctagcactttgggagg 30148
human.FAM230E-LOC105377182-GGT3P.NCBI.                                            cctctcactacatttaagtgacgccatggctcatgcctgtaatcctagcactttgggagg 28882
human.FAM230J-LOC105372942-GGTLC5P.NCBI.rev.compl                                 cctctcactacgtttaagtgatgccatggctcatgcctgtaatcctagcactttgggagg 29602
human.FAM230A.ncRNA.18487127.18500594-GGTLC3.18516335.18518165.rev.compl.REF      cctctcactacatttaagtgacgccatggctcatgcctgtaatcctagcactttgggagg 18361
                                                                                  *********** ********* **************************************

chimp.LOC112206744-LOC107973052-GGT2.revcompl                                     ccaaggcaggtggatcacctgaggtcaggagttcgacacgagcctggccaacatggggaa 31576
```

```
chimp54546-135457.revcompl.Pan.troglodytes.clone.rp43-41g5.GenBank:AC099533.36      CCAAGGCAGGTGGATCACCTGAGGTCAGGAGTTCGACACAAGCCTGGCCAACATGGGGAA 33295
human.FAM30B-LOC105372935-GGT2.NCBI.                                                 ccaaggcaggtggatcacctgaggtcaggagttcgacacgagcctggccaacatggggaa 30208
human.FAM230E-LOC105377182-GGT3P.NCBI.                                               ccaaggcaggtggatcacctgaggtcaggagttcgacatgagcctggccaacatggggaa 28942
human.FAM230J-LOC105372942-GGTLC5P.NCBI.rev.compl                                    ccaaggcaggtggatcacctgaggtcaggagttcgacacgagcctggccaacatggggaa 29662
human.FAM230A.ncRNA.18487127.18500594-GGTLC3.18516335.18518165.rev.compl.REF         ccaaggcaggtggatcacctgaggtcaggagttcgacatgagcctggccaacatggggaa 18421
                                                                                     **************************************  ********************

chimp.LOC112206744-LOC107973052-GGT2.revcompl                                        accccgtctctagtaaaaatacaaaaattagtcacgcgtggtggtacaagcctgtaggcc 31636
chimp54546-135457.revcompl.Pan.troglodytes.clone.rp43-41g5.GenBank:AC099533.36      ACCCCGTCTCTAGTAAAAATACAAAAATTAGTCACGCGTGGTGGTACAAGCCTGTAGGCC 33355
human.FAM30B-LOC105372935-GGT2.NCBI.                                                 accccgtctctagtaaaaatacaaaaattagtcaggtgtggtggtacaagcctgtaggcc 30268
human.FAM230E-LOC105377182-GGT3P.NCBI.                                               accccgtctctagtaaaaatacaaaaattagtcaggtgtggtggtacaagcctgtaggcc 29002
human.FAM230J-LOC105372942-GGTLC5P.NCBI.rev.compl                                    accctgtctctagtaaaaatacaaaaattagtcaggcgtggtggtacaagcctgtaggcc 29722
human.FAM230A.ncRNA.18487127.18500594-GGTLC3.18516335.18518165.rev.compl.REF         accccgtctctagtaaaaatacaaaaattagtcaggtgtggtggtacaagcctgtaggcc 18481
                                                                                     **** ***************************** * ***********************

chimp.LOC112206744-LOC107973052-GGT2.revcompl                                        cagctacttggaagactgaggcaggagaatcactttaagcgggaggcagaggttgcagtg 31696
chimp54546-135457.revcompl.Pan.troglodytes.clone.rp43-41g5.GenBank:AC099533.36      CAGCTACTTGGAAGACTGAGGCAGGAGAATCACTTTAACCGGGAGGCAGAGGTTGCAGTG 33415
human.FAM30B-LOC105372935-GGT2.NCBI.                                                 cagctacttggaagactgaggcaggagaatcactttaagcgggaggcagaggttgcagtg 30328
human.FAM230E-LOC105377182-GGT3P.NCBI.                                               cagctacttggaagactgaggcaggagaatcactttaagcgggaggcagaggttgcagtg 29062
human.FAM230J-LOC105372942-GGTLC5P.NCBI.rev.compl                                    cagctacttggaagactgaggcaggagaatcactttaagcgggaggcagaggttgcagtg 29782
human.FAM230A.ncRNA.18487127.18500594-GGTLC3.18516335.18518165.rev.compl.REF         cagctacttggaagactgaggcaggagaatcactttaagcgggaggcagaggttgcagtg 18541
                                                                                     ************************************** *********************

chimp.LOC112206744-LOC107973052-GGT2.revcompl                                        agccaatatcatgccactgcactccagcttgggtgacggagtgagacactgtctc----- 31751
chimp54546-135457.revcompl.Pan.troglodytes.clone.rp43-41g5.GenBank:AC099533.36      AGCCAATATCATGCCACTGCACTCCAGCTTGGGTGACAGAGTGAGACACTGTCTCAAAAA 33475
human.FAM30B-LOC105372935-GGT2.NCBI.                                                 agccaatatcatgccactgcactccagcttgggtgacagagtgagatactgtctc----a 30384
human.FAM230E-LOC105377182-GGT3P.NCBI.                                               agccaatatcatgccactgcactccagcttgggtgacagagtgagatactgtctc----a 29118
human.FAM230J-LOC105372942-GGTLC5P.NCBI.rev.compl                                    agccaatatcatgccactgcactccagcttgggtgacagagtgagatactgtctc----a 29838
human.FAM230A.ncRNA.18487127.18500594-GGTLC3.18516335.18518165.rev.compl.REF         agccaatatcatgccactgcactccagcttgggtgacagagtgagatactgtctc----a 18597
                                                                                     ************************************* ******** ********

chimp.LOC112206744-LOC107973052-GGT2.revcompl                                        ------aaaaaaaaaaaagagaaaaaaaatatgatgccggggcatctcggcctcaatacct 31805
chimp54546-135457.revcompl.Pan.troglodytes.clone.rp43-41g5.GenBank:AC099533.36      AAAAAAAGAAAAAAAAAGAGAAAAAAATATGATGCCGGGGCATCACGGCCTCAATACCT 33535
human.FAM30B-LOC105372935-GGT2.NCBI.                                                 aaaaaaaagaaaaaaagagagaaaaacatatgatgccggggcatctcggcctcaatacct 30444
human.FAM230E-LOC105377182-GGT3P.NCBI.                                               aaaaaaaagaaaaaaagagagaaaaacatatgatgccggggcatctcggcctcaatacct 29178
human.FAM230J-LOC105372942-GGTLC5P.NCBI.rev.compl                                    aaaaaaaagaaaaaataagagaaaaacatatgatgccggggcatctcggcctcaatacct 29898
human.FAM230A.ncRNA.18487127.18500594-GGTLC3.18516335.18518165.rev.compl.REF         aaaaaaaagaaaaaaagagagaaaaacatatgatgccagggcatctcggcctcaatacct 18657
                                                                                           ** ******  ********* ********** ******* **************

chimp.LOC112206744-LOC107973052-GGT2.revcompl                                        gcgtgagcacagtcatgtccaggccagggctgctggtcgaggtccggtcccatctcttcc 31865
chimp54546-135457.revcompl.Pan.troglodytes.clone.rp43-41g5.GenBank:AC099533.36      GCGTGAGCACAGTCATGTCCAGGCCAGGGCTGCTGGTCGAGGTCCGGCCCCATCTCTTCC 33595
human.FAM30B-LOC105372935-GGT2.NCBI.                                                 gggtgagcacagtcatgtccaggccagggctgctggtcgaggtccggccccatctcttcc 30504
human.FAM230E-LOC105377182-GGT3P.NCBI.                                               gggtgagcacagtcatgtccaggccagggctgctggtcgaggtccggccccatctcttcc 29238
human.FAM230J-LOC105372942-GGTLC5P.NCBI.rev.compl                                    gggtgagcacagtcatgtccaggccagggctgctggttgaggtccggccccatctcttcc 29958
human.FAM230A.ncRNA.18487127.18500594-GGTLC3.18516335.18518165.rev.compl.REF         gggtgagcacagtcatgtccaggccagggctgctggtcgaggtccggccccatctcttcc 18717
                                                                                     * *********************************** ******** *************

chimp.LOC112206744-LOC107973052-GGT2.revcompl                                        agcagaaagggagtaagcttgcagggaggctggggacaagatcccaggatctcggcctc 31925
chimp54546-135457.revcompl.Pan.troglodytes.clone.rp43-41g5.GenBank:AC099533.36      AGCAGAAAGGGAGTAAGCTTGCGGGGCGGCTGGGGACAAGATCCCAGGATCTCGGCCTC 33655
human.FAM30B-LOC105372935-GGT2.NCBI.                                                 agcagaaagggagtaagcttgcagggcggctggggacaagatcccaggatctcagcctc 30564
human.FAM230E-LOC105377182-GGT3P.NCBI.                                               agcagaaagggagtaagcttgcagggcggctggggacaagatcccaggatctcagcctc 29298
human.FAM230J-LOC105372942-GGTLC5P.NCBI.rev.compl                                    agcagaaagggagtaagcttgcagggcggctggggacaagatcccaggatctcagcctc 30018
human.FAM230A.ncRNA.18487127.18500594-GGTLC3.18516335.18518165.rev.compl.REF         agcagaaagggagtaagcttgcagggaggctggggacaagatcccaggatctcagcctc 18777
                                                                                     ********************** *** *************************** *****

chimp.LOC112206744-LOC107973052-GGT2.revcompl                                        cgctcatggatcagctctgataccccgagtgagctggggtgctctgtgcgcattggttt 31985
chimp54546-135457.revcompl.Pan.troglodytes.clone.rp43-41g5.GenBank:AC099533.36      TGCTCATGGATCAGCTCTGAGACCCCGAGTGAGCTGGGGTGCTCTGTGCGCATCGGTTT 33715
human.FAM30B-LOC105372935-GGT2.NCBI.                                                 tgctcatggatcagctctgagaccccgagtgagctggggtgctctgtgcgcattggttt 30624
human.FAM230E-LOC105377182-GGT3P.NCBI.                                               tgctcatggatcagctctgagaccccgagtgagctggggtgctctgtgcgcattggttt 29358
human.FAM230J-LOC105372942-GGTLC5P.NCBI.rev.compl                                    tgctcatggatcagctctgagaccccgagtgagctggggtgctctgtgcgcgttggttt 30078
human.FAM230A.ncRNA.18487127.18500594-GGTLC3.18516335.18518165.rev.compl.REF         tgctcatggatcagctctgagaccccgagtgagctggggtgctctgtgcgcattggttt 18837
                                                                                      ******************* ******************************* * *****

chimp.LOC112206744-LOC107973052-GGT2.revcompl                                        ccccagctgtcaagtaaagggattgtatgaggaagtcttgtcaaggtggaatgatctcag 32045
chimp54546-135457.revcompl.Pan.troglodytes.clone.rp43-41g5.GenBank:AC099533.36      CCCCAGCTGTCAAGTAAAGGGATTGGATGAGGAAGTCTTGTCAAGGTGGAATGATCTCAG 33775
human.FAM30B-LOC105372935-GGT2.NCBI.                                                 ccccagctgtcaagtaaagggattggatgaggaagtcttgtcaaggtggaatgatctcag 30684
human.FAM230E-LOC105377182-GGT3P.NCBI.                                               ccccagctgtcaagtaaagggattggatgaggaagtcttgtcaaggtggaatgatctcag 29418
human.FAM230J-LOC105372942-GGTLC5P.NCBI.rev.compl                                    ccccagctgtcaagtaaagggattggatgaggaagtcttgtcaaggtggaatgatgtcag 30138
human.FAM230A.ncRNA.18487127.18500594-GGTLC3.18516335.18518165.rev.compl.REF         ccccagctgtcaagtaaagagattggatgaggaagtcttgtcaaggtggaatgatctcag 18897
                                                                                     ******************* ***** ***************************** ****

chimp.LOC112206744-LOC107973052-GGT2.revcompl                                        atttggggcagcagtgaatgatcccgctccctgggccatgccagtggcccggcctcggct 32105
chimp54546-135457.revcompl.Pan.troglodytes.clone.rp43-41g5.GenBank:AC099533.36      CTTTGGGGCAGCAGTGAATGATCCCGCTTCCTGGGCCATGCCAGTGGCCCGGCCTCGGCT 33835
human.FAM30B-LOC105372935-GGT2.NCBI.                                                 atttggggcagcagtgaatgatcccgctccctgggccatgccagtggcccggcctcggct 30744
human.FAM230E-LOC105377182-GGT3P.NCBI.                                               atttggggcagcagtgaatgatcccgctccctgggccatgccagtggcccggcctcggct 29478
human.FAM230J-LOC105372942-GGTLC5P.NCBI.rev.compl                                    atttggggcagcagtgaatgatcccgttccttgggccatgccagtggcctggcctcggct 30198
human.FAM230A.ncRNA.18487127.18500594-GGTLC3.18516335.18518165.rev.compl.REF         atttggggcagcagtgaatgatcccgctccctgggccatgccagtggcccggcctcggct 18957
                                                                                      ************************* * * ****************** **********

chimp.LOC112206744-LOC107973052-GGT2.revcompl                                        caacacagccccaacactctggaatggggatgagggggcagtcagctcttgctcctagta 32165
chimp54546-135457.revcompl.Pan.troglodytes.clone.rp43-41g5.GenBank:AC099533.36      CAACACAGCCCCAACACTCTGGAATGGGGATGAGGGGGCAGTCAGCTCTTGCTCCTAGTA 33895
human.FAM30B-LOC105372935-GGT2.NCBI.                                                 gaacacagccccaacactctggaatggggatgagggggcagtcagctcttgctcctagta 30804
human.FAM230E-LOC105377182-GGT3P.NCBI.                                               gaacacagccccaacactctggaatggggatgagggggcagtcagctcttgctcctagta 29538
human.FAM230J-LOC105372942-GGTLC5P.NCBI.rev.compl                                    caacacagccccaacactctggaatggggatgagggggcagtcagctcttgctcctagta 30258
human.FAM230A.ncRNA.18487127.18500594-GGTLC3.18516335.18518165.rev.compl.REF         gaacacagccccaacactctggaatggggatgagggggcagtcagctcttgctcctagta 19017
                                                                                      ***********************************************************

chimp.LOC112206744-LOC107973052-GGT2.revcompl                                        agagagatgcaacggggctctgtggctgagctgggtgccttgcctcacacctgtaatccc 32225
chimp54546-135457.revcompl.Pan.troglodytes.clone.rp43-41g5.GenBank:AC099533.36      AGAGAGATGCAACAGGGCTCTGTGGCTGAGCTGGGTGCCTTGCCTCACACCTGTAATCCC 33955
human.FAM30B-LOC105372935-GGT2.NCBI.                                                 agagagatgcaacagggctctgtggctgagctgggtgccttgcctcacacctgtaatccc 30864
human.FAM230E-LOC105377182-GGT3P.NCBI.                                               agagagatgcaacagggctctgtggctgagctgggtgccttgcctcacacctgtaatccc 29598
human.FAM230J-LOC105372942-GGTLC5P.NCBI.rev.compl                                    agagagatgcaacagggctctgtggctgagctgggtgccttgcctcacacctgtaatccc 30318
human.FAM230A.ncRNA.18487127.18500594-GGTLC3.18516335.18518165.rev.compl.REF         agagagatgcaacagggctctgtggctgagctgggtgccttgcctcacacctgtaatccc 19077
                                                                                     ************* **********************************************

chimp.LOC112206744-LOC107973052-GGT2.revcompl                                        aacctttgagaggctgaggcaggaggattgctcgaggccgggaattttgagaatagcctg 32285
chimp54546-135457.revcompl.Pan.troglodytes.clone.rp43-41g5.GenBank:AC099533.36      AACCTTTGAGAGGCCGAGGCAGGAGGATTGCTCGAGGCCGGGAATTTTGAGAATAGCCTG 34015
human.FAM30B-LOC105372935-GGT2.NCBI.                                                 aacctttgagaggccgaggcaggaggattgctcgaggccgggaattttgagaatagcctg 30924
human.FAM230E-LOC105377182-GGT3P.NCBI.                                               aacctttgagaggccgaggcaggaggattgctcgagaccgggaattttgagaatagcctg 29658
human.FAM230J-LOC105372942-GGTLC5P.NCBI.rev.compl                                    aacctttgagaggccgaggcaggaggattgctcgaggccgggaattttgagaatagcctg 30378
human.FAM230A.ncRNA.18487127.18500594-GGTLC3.18516335.18518165.rev.compl.REF         aacctttgagaggccgaggcaggaggattgctcgaggccgggaattttgagaatagcctg 19137
                                                                                     ************** ********************* ***********************

chimp.LOC112206744-LOC107973052-GGT2.revcompl                                        gacaacatagccagaccccatgtctacaaaataataaaacatacagctatagtccag 32345
chimp54546-135457.revcompl.Pan.troglodytes.clone.rp43-41g5.GenBank:AC099533.36      GACAACATAGCCAGACCCCATGTCTACAAAATAATAAAACATACAGCTATAGTCCAA 34075
human.FAM30B-LOC105372935-GGT2.NCBI.                                                 gacaacatagccagaccccatgtctacaaaataataagaaaacacacagctatagtccaa 30984
human.FAM230E-LOC105377182-GGT3P.NCBI.                                               gacaacatagccagaccccatgtctacaaaataataaaacacacagctatagtccaa 29718
human.FAM230J-LOC105372942-GGTLC5P.NCBI.rev.compl                                    gacaacatagccagaccccatgtctacaaaataataaaacatacagctatagtccaa 30438
human.FAM230A.ncRNA.18487127.18500594-GGTLC3.18516335.18518165.rev.compl.REF         gacaacatagccagaccccatgtctacaaaataataaaacacacagctatagtccaa 19197
                                                                                     ************************************* ****** **************

chimp.LOC112206744-LOC107973052-GGT2.revcompl                                        gctacttggcaggctgaggcaggagggtcccttgagtccaggaattggaggctgcattga 32405
chimp54546-135457.revcompl.Pan.troglodytes.clone.rp43-41g5.GenBank:AC099533.36      GCTACTTGGCAGGCTGAGGCAGGAGGGTCCCTTGAGTCCAGGAATTGGAGGCTGCATTGA 34135
human.FAM30B-LOC105372935-GGT2.NCBI.                                                 gctacttggcaggctgaggcaggagggtcccttgagtccaggaattggaggctgcattga 31044
human.FAM230E-LOC105377182-GGT3P.NCBI.                                               gctacttggcaggctgaggcaggagggtcccttgagtccaggaattggaggctgcattga 29778
human.FAM230J-LOC105372942-GGTLC5P.NCBI.rev.compl                                    gctacttggcaggctgaggcaggagggtcccttgagtccaggaattggaggctgcattga 30498
human.FAM230A.ncRNA.18487127.18500594-GGTLC3.18516335.18518165.rev.compl.REF         gctacttggcaggctgaggcaggagggtcccttgagtccaggaattggaggctgcattga 19257
                                                                                     ************************************************************

chimp.LOC112206744-LOC107973052-GGT2.revcompl                                        gctataatcgcaccactgcactccagcttgggtgacaaagtgagaccctgtctctaaaag 32465
chimp54546-135457.revcompl.Pan.troglodytes.clone.rp43-41g5.GenBank:AC099533.36      GCTATAATCGTACCACTGCACTCCAGCTTGGGTGACAAAGTGAGACCCTGTCTCTAAAAG 34195
human.FAM30B-LOC105372935-GGT2.NCBI.                                                 gctataatcgcaccactgcactccagcttgggtgacaaagtgagaccctgtctctaaaag 31104
human.FAM230E-LOC105377182-GGT3P.NCBI.                                               gctataatcgcaccactgcactccagcttgggtgacaaagtgagaccctgtctctaaaag 29838
human.FAM230J-LOC105372942-GGTLC5P.NCBI.rev.compl                                    gctataatcgcaccactgcactccagcttgggtgacaaagtgagaccctgtctctaaaag 30558
human.FAM230A.ncRNA.18487127.18500594-GGTLC3.18516335.18518165.rev.compl.REF         gctataatcgcaccactgcactccagcttgggtgacaaagtgagaccctgtctctaaaag 19317
                                                                                     ********** *************************************************

chimp.LOC112206744-LOC107973052-GGT2.revcompl                                        -aaaaaacattggcctgtgagcatgggtttgagtttcaaacaggagccggagggtagggt 32524
chimp54546-135457.revcompl.Pan.troglodytes.clone.rp43-41g5.GenBank:AC099533.36      AAAAAAAAATTGGCCTGTGAGCATGGGTTTGATTTTTAAACAGGACCCAGAGGGTAGGGT 34255
human.FAM30B-LOC105372935-GGT2.NCBI.                                                 -aaaaaaaattggcctgtgagcatgggcttgattttcaaacaggacccggagggtagggt 31163
human.FAM230E-LOC105377182-GGT3P.NCBI.                                               aaaaaaaaattggcctgtgagcatgggcttgattttcaaacaggacccggagggtagggt 29898
human.FAM230J-LOC105372942-GGTLC5P.NCBI.rev.compl                                    aaaaaaaaattggcctgtgagcatgggcttgattttcaaacaggacccggagggtagggt 30618
human.FAM230A.ncRNA.18487127.18500594-GGTLC3.18516335.18518165.rev.compl.REF         aaaaaaaaattggcctgtgagcatgggcttgattttcaaacaggacccggagggtagggt 19377
                                                                                      ****** ******************* **** *** ******** ** ***********

chimp.LOC112206744-LOC107973052-GGT2.revcompl                                        aaacttgtcggtaaatctaaatgaatgttattggtataaaattacaatagtatagaaaat 32584
chimp54546-135457.revcompl.Pan.troglodytes.clone.rp43-41g5.GenBank:AC099533.36      AAACGTGTGGGTAAATCTAAATGAATGCTATTGGTATAAAATTACAGTAGTATAGAAAAT 34315
human.FAM30B-LOC105372935-GGT2.NCBI.                                                 aaacgtgtgggtaaatctaaatgaatgttattggtataaaattacagtagtatagaaaat 31223
human.FAM230E-LOC105377182-GGT3P.NCBI.                                               aaacgtgtgggtaaatctaaatgaatgttattggtataaaattacagtagtatagaaaat 29958
human.FAM230J-LOC105372942-GGTLC5P.NCBI.rev.compl                                    aaacgtgtgggtaaatctaaatgaatgttattggtataaaattacagtagtatagaaaat 30678
human.FAM230A.ncRNA.18487127.18500594-GGTLC3.18516335.18518165.rev.compl.REF         aaacgtgtgggtaaatctaaatgaatgttattggtataaaattacagtagtatagaaaat 19437
                                                                                     **** *** ****************** ****************** *************

chimp.LOC112206744-LOC107973052-GGT2.revcompl                                        gatatcttgtggggtttaaaataaataaaacattctgaaatatgtatgggtacagttata 32644
chimp54546-135457.revcompl.Pan.troglodytes.clone.rp43-41g5.GenBank:AC099533.36      GATATCTTGTGGGGTTTAAAATAAATGAAACATACTGAAATATGTATGGGTACAGTTATA 34375
```

```
human.FAM30B-LOC105372935-GGT2.NCBI.                                                gatatcttgtggggtttaaaataaataaaacatactgaaatatgtattggtacagttata 31283
human.FAM230E-LOC105377182-GGT3P.NCBI.                                              gatatcttgtggggtttaaaagaaataaaacatactgaaatatgtatgggtacagttata 30018
human.FAM230J-LOC105372942-GGTLC5P.NCBI.rev.compl                                   gatatcttgtggggtttaaaagaaataaaacatactgaaatatgtatgggtacagttata 30738
human.FAM230A.ncRNA.18487127.18500594-GGTLC3.18516335.18518165.rev.compl.REF        gatatcttgtggggtttaaaataaataaaacatactgaaatatgtatgggtacagttata 19497
                                                                                    ******************** **** ****** ************* ************

chimp.LOC112206744-LOC107973052-GGT2.revcompl                                       tatctgggatttgcactgaaataatgtggggtagagggaagcaggaaagaggatacatga 32704
chimp54546-135457.revcompl.Pan.troglodytes.clone.rp43-41g5.GenBank:AC099533.36      TATCTGGGATTTGCACTGAAATAATGTGGGGTAGAGGGAAGCAGGAAAGAGGATACATGA 34435
human.FAM30B-LOC105372935-GGT2.NCBI.                                                tatctgggatttgcactgaaataatgtggggtagagggaagcaggaaagagtatacatga 31343
human.FAM230E-LOC105377182-GGT3P.NCBI.                                              tatctgggatttgcactgaaataatgtggggtagagggaagcaggaaagagtatacatga 30078
human.FAM230J-LOC105372942-GGTLC5P.NCBI.rev.compl                                   tatctgggatttgcacggaaataatgtggggtagagggaagcaggaaagagtatacatga 30798
human.FAM230A.ncRNA.18487127.18500594-GGTLC3.18516335.18518165.rev.compl.REF        tatctgggatttgcactgaaataatgtggggtagagggaagcaggaaagagtatacatga 19557
                                                                                    *************** ********************************** ********

chimp.LOC112206744-LOC107973052-GGT2.revcompl                                       aattagcttggccataagattgttgttgaaattgaatggatactcggggcttcattacac 32764
chimp54546-135457.revcompl.Pan.troglodytes.clone.rp43-41g5.GenBank:AC099533.36      AATTAGCTTGGCCATAAGATTGTTGTTGAAATTGAATGGATACTCGGGGCTTCATTACAC 34495
human.FAM30B-LOC105372935-GGT2.NCBI.                                                aatgagcttggccataagattgttgttgaaattgaatggatactcggggcttcattacac 31403
human.FAM230E-LOC105377182-GGT3P.NCBI.                                              aatgagcttggccataagattgttgttgaaattgaatggatactcggggcttcattacac 30138
human.FAM230J-LOC105372942-GGTLC5P.NCBI.rev.compl                                   aatgagcttggccataagattgttgttgaaattgaatggatactcggggcttcattacac 30858
human.FAM230A.ncRNA.18487127.18500594-GGTLC3.18516335.18518165.rev.compl.REF        aatgagcttggccataagattgttgttgaaattgaatggatactctgggcttcattacac 19617
                                                                                    *** ***************************************** *************

chimp.LOC112206744-LOC107973052-GGT2.revcompl                                       aattctttttactcttacatagctctacactctcaacataaataagaataaaaacacaaa 32824
chimp54546-135457.revcompl.Pan.troglodytes.clone.rp43-41g5.GenBank:AC099533.36      AATTCTCTTTACTCTTACATAGCTCTACACTCTCAACATAAATAAGAATAAAAACACAAA 34555
human.FAM30B-LOC105372935-GGT2.NCBI.                                                aattctctttactcttacatagctctacactctcaacataaataagaataaaaacacaaa 31463
human.FAM230E-LOC105377182-GGT3P.NCBI.                                              aattctctttactcttacatagctctacactctcaacataaataagaataaaaacacaaa 30198
human.FAM230J-LOC105372942-GGTLC5P.NCBI.rev.compl                                   aattctctttactcttacatagctctacactctcaacataaataagaataaaaacacaaa 30918
human.FAM230A.ncRNA.18487127.18500594-GGTLC3.18516335.18518165.rev.compl.REF        aattctctttactcttacatagctctacactctcaacataaataagaataaaaacacaaa 19677
                                                                                    ****** *****************************************************

chimp.LOC112206744-LOC107973052-GGT2.revcompl                                       aaacacacagatacatctatgcacacacatgtattttaaatacacaaaaatattagcata 32884
chimp54546-135457.revcompl.Pan.troglodytes.clone.rp43-41g5.GenBank:AC099533.36      AAACACACAGATACATCTATGCACACACATATATTTAAAATACACAAAAATATTAGCATA 34615
human.FAM30B-LOC105372935-GGT2.NCBI.                                                aaacacacagatacatctatgcacacacacatatttaaaatacacaaaaatattagcata 31523
human.FAM230E-LOC105377182-GGT3P.NCBI.                                              aaacacacagatacatctatgcacacacacatatttaaaatacacaaaaatattagcata 30258
human.FAM230J-LOC105372942-GGTLC5P.NCBI.rev.compl                                   aaacacacagatacatctatgcacacacacatatttaaaatacacaaaaatattagcata 30978
human.FAM230A.ncRNA.18487127.18500594-GGTLC3.18516335.18518165.rev.compl.REF        aaacacacagatacatctatgcacacacacatatttaaaatacacaaaaatattagcata 19737
                                                                                    *****************************  ***** ***********************

chimp.LOC112206744-LOC107973052-GGT2.revcompl                                       taagtcactggggggtaaatttagttcctgttccaaggttcttgtactgactaggaagagg 32944
chimp54546-135457.revcompl.Pan.troglodytes.clone.rp43-41g5.GenBank:AC099533.36      TAAGTCACTGGGGGTAAATTTAGTTCCTGTTCCAAGGTTCTTGTACTGACTAGGAAGAGG 34675
human.FAM30B-LOC105372935-GGT2.NCBI.                                                taagtcactgggggtaaatttagttcctgttccaaggttcttgtactgactaggaagagg 31583
human.FAM230E-LOC105377182-GGT3P.NCBI.                                              taagtcactgggggtaaatttagttcctgttccaaggttcttgtactgactaggaagagg 30318
human.FAM230J-LOC105372942-GGTLC5P.NCBI.rev.compl                                   taagtcactgggggtaaatttagttcctgttccaaggttcttgtactgactaggaagagg 31038
human.FAM230A.ncRNA.18487127.18500594-GGTLC3.18516335.18518165.rev.compl.REF        taagtcactgggggtaaatttagttcctgttccaaggttcttgtactgactaggaagagg 19797
                                                                                    ************************************************************

chimp.LOC112206744-LOC107973052-GGT2.revcompl                                       atagaagtactaactcataggctgggcgtggtggctcacgcctgtaatcccaacacttta 33004
chimp54546-135457.revcompl.Pan.troglodytes.clone.rp43-41g5.GenBank:AC099533.36      ATAGAAGTACTAACTCATAGGCTGGGCGTGGTGGCTCACGCCTGTAATCCCAACACTTTA 34735
human.FAM30B-LOC105372935-GGT2.NCBI.                                                atagaagtactaactcataggctgggcgcggtggctcacgcctgtaatcccaacacttta 31643
human.FAM230E-LOC105377182-GGT3P.NCBI.                                              atagaagtactaactcataggctgggtgcggtggctcacgcctgtaatcccaacacttta 30378
human.FAM230J-LOC105372942-GGTLC5P.NCBI.rev.compl                                   atagaagtactaactcataggctgggtgcggtggctcacgcctgtaatcccaacacttta 31098
human.FAM230A.ncRNA.18487127.18500594-GGTLC3.18516335.18518165.rev.compl.REF        atagaagtactaactcataggctgggcgcggtggctcacgcctgtaatcccaacacttta 19857
                                                                                    ************************** * ********************************

chimp.LOC112206744-LOC107973052-GGT2.revcompl                                       ggacgccgaggtaagcagatctcttaaggtcaggagttcaagaccagcctggccaacatg 33064
chimp54546-135457.revcompl.Pan.troglodytes.clone.rp43-41g5.GenBank:AC099533.36      GGACGCCGAGGTAAGCAGATCTCTTAAGGTCAGGAGTTCAAGACCAGCCTGGCCAACATG 34795
human.FAM30B-LOC105372935-GGT2.NCBI.                                                ggatgccgaggtaggcagatctcttaaggtccggagttcaagaccagcctggccaacatg 31703
human.FAM230E-LOC105377182-GGT3P.NCBI.                                              ggatgccgaggtaggcagatctcttaaggtccggagttcaagaccagcctggccaacatg 30438
human.FAM230J-LOC105372942-GGTLC5P.NCBI.rev.compl                                   ggatgccgaggtaggcagatctcttaaggtccggagttcaagaccagcctggccaacatg 31158
human.FAM230A.ncRNA.18487127.18500594-GGTLC3.18516335.18518165.rev.compl.REF        ggatgccgaggtaggcagatctcttaaggtccggagttcaagaccagcctggccaacatg 19917
                                                                                    *** ********* ***************** *****************************

chimp.LOC112206744-LOC107973052-GGT2.revcompl                                       gtgaaaccctgtctctactaaaaaagaatacaaaaattggccgggcatagtggtgcacac 33124
chimp54546-135457.revcompl.Pan.troglodytes.clone.rp43-41g5.GenBank:AC099533.36      GTGAAACCCTGTCTCTACTAAAAAAGAATACAAAAATTGGCCGGGCATAGTGGTGCACAC 34855
human.FAM30B-LOC105372935-GGT2.NCBI.                                                gtgaaaccctgtctctactgaaaaagaatacaaaaattggccgggcatagtggtgcacac 31763
human.FAM230E-LOC105377182-GGT3P.NCBI.                                              gtgaaaccctgtctctactgaaaaagaatacaaaaattggccgggcatagtggtgcacac 30498
human.FAM230J-LOC105372942-GGTLC5P.NCBI.rev.compl                                   gtgaaaccctgtctctactgaaaaagaatacaaaaattggccgggcatagtggtgcacac 31218
human.FAM230A.ncRNA.18487127.18500594-GGTLC3.18516335.18518165.rev.compl.REF        gtgaaaccctgtctctactgaaaaagaatacaaaaattggccgggcatagtggtgcacac 19977
                                                                                    ******************* ****************************************

chimp.LOC112206744-LOC107973052-GGT2.revcompl                                       ctgtggtcccagctactcaggtgactgaggcaggagaattgcttgaacccaagaagtgga 33184
chimp54546-135457.revcompl.Pan.troglodytes.clone.rp43-41g5.GenBank:AC099533.36      CTGTGGTCCCAGCTACTCAGGTGACTGAGGCAGGAGAATTGCTTGAACCCAAGAAGTGGA 34915
human.FAM30B-LOC105372935-GGT2.NCBI.                                                ctgtggtcccagctactcaggtgactgaggcaggagaattgcttgaacccaagaagtgga 31823
human.FAM230E-LOC105377182-GGT3P.NCBI.                                              ctgtggtcccagctactcaggtgactgaggcaggagaattgcttgaacccaagaagtgga 30558
human.FAM230J-LOC105372942-GGTLC5P.NCBI.rev.compl                                   ctgtggtcccagctactcaggtgactgaggcaggagaattgcttgaacccaagaagtgga 31278
human.FAM230A.ncRNA.18487127.18500594-GGTLC3.18516335.18518165.rev.compl.REF        ctgtggtcccagctactcaggtgactgaggcaggagaattgcttgaacccaagaagtgga 20037
                                                                                    ************************************************************

chimp.LOC112206744-LOC107973052-GGT2.revcompl                                       ggttgcagtgaaccaagattgctccactgcactccagcctgggcagcagaggaagactct 33244
chimp54546-135457.revcompl.Pan.troglodytes.clone.rp43-41g5.GenBank:AC099533.36      GGTTGCAGTGAACCAAGATTGCTCCACTGCACTCCAGCCTGGGCAGCAGAGGAAGACTCT 34975
human.FAM30B-LOC105372935-GGT2.NCBI.                                                ggttgcagtgaaccaagattgctccactgcactccagcctgggcagcagaggaagactct 31883
human.FAM230E-LOC105377182-GGT3P.NCBI.                                              ggttgcagtgaaccaagattgctccactgcactccagcctgggcagcagaggaagactct 30618
human.FAM230J-LOC105372942-GGTLC5P.NCBI.rev.compl                                   ggttgcagtgaaccaagattgctccactgcactccagcctgggcagcagaggaagactct 31338
human.FAM230A.ncRNA.18487127.18500594-GGTLC3.18516335.18518165.rev.compl.REF        ggttgcagtgaaccaagattgctccactgcactccagcctgggcagcagaggaagactct 20097
                                                                                    ************************************************************

chimp.LOC112206744-LOC107973052-GGT2.revcompl                                       ctctgtctcaacaacaacaaaaagtactagctcatgttagactttgataagtgaaggatg 33304
chimp54546-135457.revcompl.Pan.troglodytes.clone.rp43-41g5.GenBank:AC099533.36      CTCTATCTCAACAACAACAAAAAGTACTAGCTCATGTTAGACTTTGATAAGTGAAGGATG 35035
human.FAM30B-LOC105372935-GGT2.NCBI.                                                ctctatctcaaccacaacaaaaagtactagctcatgttagactttgataagggaaggatg 31943
human.FAM230E-LOC105377182-GGT3P.NCBI.                                              ctctatctcaaccacaacaaaaagtactagctcatgttagactttgataagtgaaggatg 30678
human.FAM230J-LOC105372942-GGTLC5P.NCBI.rev.compl                                   ctctatctcaaccacaacaaaaagtactagctcatgttagactttgataagggaaggatg 31398
human.FAM230A.ncRNA.18487127.18500594-GGTLC3.18516335.18518165.rev.compl.REF        ctctatctcaaccacaacaaaaagtactagctcatgttagactttgataagggaaggatg 20157
                                                                                    **** ******* *************************************** ********

chimp.LOC112206744-LOC107973052-GGT2.revcompl                                       catgttgtaagctctaaaataatccactgtcatcttttaaaataactctaagactgcaca 33364
chimp54546-135457.revcompl.Pan.troglodytes.clone.rp43-41g5.GenBank:AC099533.36      CATGTTGTAAGCTCTAAAATAATCCA--GTCATCTTTTAAAATAACTCTAAGACTGCACA 35093
human.FAM30B-LOC105372935-GGT2.NCBI.                                                catgttgtaagctctaaaataatcca--gtcatcttttaaaataactctaagactgcaca 32001
human.FAM230E-LOC105377182-GGT3P.NCBI.                                              catgttgtaagctctaaaataatcca--gtcatcttttaaaataactctaagactgcaca 30736
human.FAM230J-LOC105372942-GGTLC5P.NCBI.rev.compl                                   catgttgtaagctctaaaataatcca--gtcatcttttaaaagaactctaagactgcaca 31456
human.FAM230A.ncRNA.18487127.18500594-GGTLC3.18516335.18518165.rev.compl.REF        catgttgtaagctctaaaataatcca--gtcatcttttaaaataactctaagactgcaca 20215
                                                                                    **************************  ************** *****************

chimp.LOC112206744-LOC107973052-GGT2.revcompl                                       gttatgaaactaatag---aggaggaaattaaataataaaaataataaatccaaaacaag 33421
chimp54546-135457.revcompl.Pan.troglodytes.clone.rp43-41g5.GenBank:AC099533.36      GTTATGAAACTAATAGAGAAGGAGGAAATTAAATCATAAAAATAATAAATCCAAAACAAG 35153
human.FAM30B-LOC105372935-GGT2.NCBI.                                                gttatgaaactaatagagaaggaggaaattaaataataaaaataataaatccaaaacaag 32061
human.FAM230E-LOC105377182-GGT3P.NCBI.                                              gttatgaaactaatagagaaggaggaaattaaataataaaaataataaatccaaaacaag 30796
human.FAM230J-LOC105372942-GGTLC5P.NCBI.rev.compl                                   gttatgaaactaatagagaaggaggaaattaaataataaaaataataaatccaaaacaag 31516
human.FAM230A.ncRNA.18487127.18500594-GGTLC3.18516335.18518165.rev.compl.REF        gttatgaaactaatagagaaggaggaaattaaataataaaactaataaatccaaaacaag 20275
                                                                                    ****************   *************** ****** ******************

chimp.LOC112206744-LOC107973052-GGT2.revcompl                                       atgtgagaggagataagaagaaatagaataggcatggaaaacaaattggtggtgggtttc 33481
chimp54546-135457.revcompl.Pan.troglodytes.clone.rp43-41g5.GenBank:AC099533.36      ATGTGAGAGAAGATAAGAAGAAATAGAATAGGCATGGAAAACAAATTGGTGGTGGGTTTC 35213
human.FAM30B-LOC105372935-GGT2.NCBI.                                                atgtgagaggagataagaagaaatagaataggcatggaaaacaaattggtggtgggtttc 32121
human.FAM230E-LOC105377182-GGT3P.NCBI.                                              atgtgagaggagataagaagaaatagaataggcatggaaaacaaattggtggtgggtttc 30856
human.FAM230J-LOC105372942-GGTLC5P.NCBI.rev.compl                                   atgtgagaggagataagaagaaatagaataggcatggaaaacaaattggtggtgggtttc 31576
human.FAM230A.ncRNA.18487127.18500594-GGTLC3.18516335.18518165.rev.compl.REF        atgtgagaggagataagaagaaatagaataggcatggaaaacaaattggtggtgggtttc 20335
                                                                                    ********* **************************************************

chimp.LOC112206744-LOC107973052-GGT2.revcompl                                       aacccaaataaatcattagttacatttaaaaggacaataaaaattaaaataatcgaaaat 33541
chimp54546-135457.revcompl.Pan.troglodytes.clone.rp43-41g5.GenBank:AC099533.36      AACGTAAATAAATCATTAGTTACATTTAAAAGGACAATAAAAATTAAAATAATTGAAAAT 35273
human.FAM30B-LOC105372935-GGT2.NCBI.                                                aacccaaataaatcattagttacatttaaaaggacaataaaaattaaaataattgaaaat 32181
human.FAM230E-LOC105377182-GGT3P.NCBI.                                              aacccaaataaatcattagttacatttaaaaggacaataaaaattaaaataattgaaaat 30916
human.FAM230J-LOC105372942-GGTLC5P.NCBI.rev.compl                                   aacccaaataaatcattagttacatttaaaaggacaataaaaattaaaataattgaaaat 31636
human.FAM230A.ncRNA.18487127.18500594-GGTLC3.18516335.18518165.rev.compl.REF        aacccaaataaatcattagttacatttaaaaggacaataaaaattaaaataattgaaaat 20395
                                                                                    ***  ***************************************** ******

chimp.LOC112206744-LOC107973052-GGT2.revcompl                                       aaagtaaaacccaactaatgccttttatataagaatacagagaggtggaagataatgaaa 33601
chimp54546-135457.revcompl.Pan.troglodytes.clone.rp43-41g5.GenBank:AC099533.36      AAAATAAAACCCAACTAATGCCTTTTATATAAGAATACAGAGAGGTGGAAGATAATGAAA 35333
human.FAM30B-LOC105372935-GGT2.NCBI.                                                aaagtaaaacccaactaatgccttttatataagggtacagagaggtggaagatcatgaaa 32241
human.FAM230E-LOC105377182-GGT3P.NCBI.                                              aaagtaaaacccaactaatgccttttatataaggatacagagaggtggaagatcatgaaa 30976
human.FAM230J-LOC105372942-GGTLC5P.NCBI.rev.compl                                   aaagtaaaacccaactaatgccttttatataagggtacagagaggtggaagatcatgaaa 31696
human.FAM230A.ncRNA.18487127.18500594-GGTLC3.18516335.18518165.rev.compl.REF        aaagtaaaacccaactaatgccttttatataagggtacagagaggtggaagatcatgaaa 20455
                                                                                    *** *****************************  ***************** ******

chimp.LOC112206744-LOC107973052-GGT2.revcompl                                       aatatgtcatgcatgcactaaccaagaaagctgtataacttttttttttttttt----- 33656
chimp54546-135457.revcompl.Pan.troglodytes.clone.rp43-41g5.GenBank:AC099533.36      AATATGTCATGCATGCACTAACCAAGAAAGCTGTATCGCTTTTTTTTTTTTTTTT---- 35389
human.FAM30B-LOC105372935-GGT2.NCBI.                                                aatatgtcatgcatgtactaaccaagaaagctgtataacttttttttttttttttttttt 32301
human.FAM230E-LOC105377182-GGT3P.NCBI.                                              aatatgtcatgcatgtactaaccaagaaagctgtataacttttttttttttttttttttt 31036
human.FAM230J-LOC105372942-GGTLC5P.NCBI.rev.compl                                   aatatgtcatgcatgtactaaccaagaaagctgtataacttttttttttttttttttttt 31756
human.FAM230A.ncRNA.18487127.18500594-GGTLC3.18516335.18518165.rev.compl.REF        aatatgtcatgcatgtactaaccaagaaagctgtataac--ttttttttttttttttttt 20513
                                                                                    *************** ********************  *   **************

chimp.LOC112206744-LOC107973052-GGT2.revcompl                                       --------ggagatagagcctcactctgtctcccaggctggagtgcagtgatgtgatctt 33708
chimp54546-135457.revcompl.Pan.troglodytes.clone.rp43-41g5.GenBank:AC099533.36      --------GGAGATAGAGCCTCACTCTGTCTCCCAGGCTGGAGTGCAGTGATGTGATCTT 35441
human.FAM30B-LOC105372935-GGT2.NCBI.                                                tttttt--ggagatagagcctcactctgtctcccaggctggagtgcagtgatgtgatctt 32359
```

```
human.FAM230E-LOC105377182-GGT3P.NCBI.                                          tttttttgggagatagagcctcactctgtctcccaggctggagtgcagtgatgtgatctt 31096
human.FAM230J-LOC105372942-GGTLC5P.NCBI.rev.compl                               ttttttttggagatagagcctcactctgtctcccaggctggagtgcagtgatgtgatctt 31816
human.FAM230A.ncRNA.18487127.18500594-GGTLC3.18516335.18518165.rev.compl.REF    ttttttttggagatagagcctcactctgtctcccaggctggagtgcagtgatgtgatctt 20573
                                                                                        ****************************************************

chimp.LOC112206744-LOC107973052-GGT2.revcompl                                   ggcttacagcaatctctcccttctaggctcaagcgattctcccacctcagcatcccaagt 33768
chimp54546-135457.revcompl.Pan.troglodytes.clone.rp43-41g5.GenBank:AC099533.36  GGGTTACAGCAATCTCTCCCTTCTAGGCTCAAGCGATTCTCCCACCTCAGCATCCCAAGT 35501
human.FAM30B-LOC105372935-GGT2.NCBI.                                            ggcttacagcaatctctcccttctaggctcaagcgattctcccacctcagcatcccaagt 32419
human.FAM230E-LOC105377182-GGT3P.NCBI.                                          ggcttacagcaatctctcccttctaggctcaagcgattctcccacctcagcatcccaagt 31156
human.FAM230J-LOC105372942-GGTLC5P.NCBI.rev.compl                               ggcttacagcaatctctcccttctaggctcaagcgattctcccacctcagcatcccaagt 31876
human.FAM230A.ncRNA.18487127.18500594-GGTLC3.18516335.18518165.rev.compl.REF    ggcttacagcaatctctcccttctaggctcaagcgattctcccacctcagcatcccaagt 20633
                                                                                ** *********************************************************

chimp.LOC112206744-LOC107973052-GGT2.revcompl                                   agctgggactacaagtgtgccaacttagaattatattagccacacccagctaattttgt 33828
chimp54546-135457.revcompl.Pan.troglodytes.clone.rp43-41g5.GenBank:AC099533.36  AGCTGGGACTACAAGTGTGCCAACTTAGAATTATATTAGCCACACCCAGCTAATTTTTGT 35561
human.FAM30B-LOC105372935-GGT2.NCBI.                                            agctgggactacaagtgtgccaacttagaattatattagccacacccagctaatttttgt 32479
human.FAM230E-LOC105377182-GGT3P.NCBI.                                          agctgggactacaagtgtgccaacttagaattatattagccacacccagctaatttttgt 31216
human.FAM230J-LOC105372942-GGTLC5P.NCBI.rev.compl                               agctgggactacaagtgtgccaacttagaattatactagccacacccagctaatttttgt 31936
human.FAM230A.ncRNA.18487127.18500594-GGTLC3.18516335.18518165.rev.compl.REF    agctgggactacaagtgtgccaacttagaattatattagccacacccagctaatttttgt 20693
                                                                                *********************************** ************************

chimp.LOC112206744-LOC107973052-GGT2.revcompl                                   atttttgtagaggcagggtctcgccatgttgcccaggctggtcttgaactcctgggctt 33888
chimp54546-135457.revcompl.Pan.troglodytes.clone.rp43-41g5.GenBank:AC099533.36  ATTTTTGTAGAGGCAGGGTCTCGCCATGTTGCCCAGGTTGGTCTTGAACTCCTGGGCTT 35621
human.FAM30B-LOC105372935-GGT2.NCBI.                                            atttttgtagaggcagggtctcgccatgttgcccaggttggtcttgaactcctgggctt 32539
human.FAM230E-LOC105377182-GGT3P.NCBI.                                          atttttgtagaggcagggtctcgccatgttgcccaggttggtcttgaactcctgggctt 31276
human.FAM230J-LOC105372942-GGTLC5P.NCBI.rev.compl                               atttttgtagaggcagggtctcgccatgttgcccaggttggtcttgaactcctgggctt 31996
human.FAM230A.ncRNA.18487127.18500594-GGTLC3.18516335.18518165.rev.compl.REF    atttttgtagaggcagggtctcgccatgttgcccaggttggtcttgaactcctgggctt 20753
                                                                                ************************************* **********************

chimp.LOC112206744-LOC107973052-GGT2.revcompl                                   cagtgatccacccacctcgacatccagcaaagtgccaagattacagccatgagccaccat 33948
chimp54546-135457.revcompl.Pan.troglodytes.clone.rp43-41g5.GenBank:AC099533.36  CAGTGATCCACCCACCTCGACCTCCAACAAAGTGCCGAGATTACAGCCATGAGCCACCAT 35681
human.FAM30B-LOC105372935-GGT2.NCBI.                                            cagtgatccacccacctcgacctccagcaaagtgccaagattacagccatgagccaccat 32599
human.FAM230E-LOC105377182-GGT3P.NCBI.                                          cagtgatccacccacctcgacctccagcaaagtgccaagattacagccatgagccaccat 31336
human.FAM230J-LOC105372942-GGTLC5P.NCBI.rev.compl                               cagtgatccacccacctcgacctccagcaaagtgccaagattacagccatgagccaccat 32056
human.FAM230A.ncRNA.18487127.18500594-GGTLC3.18516335.18518165.rev.compl.REF    cagtgatccacccacctcgacctccagcaaagtgccaagattacagccatgagccaccat 20813
                                                                                ********************* **** ********* ***********************

chimp.LOC112206744-LOC107973052-GGT2.revcompl                                   gcccagcataaccattttaatgaagtagactttaagaagaaaagtattattagaggtaa 34008
chimp54546-135457.revcompl.Pan.troglodytes.clone.rp43-41g5.GenBank:AC099533.36  GCCCAGCATAACTATTTTTAATGAAGTAGACTTTAAGAAGAAAAGTATTATTAGAAGTAA 35741
human.FAM30B-LOC105372935-GGT2.NCBI.                                            gcccagcataactatttttaatgaagtagactttaagaagaaaagtattattagaggtaa 32659
human.FAM230E-LOC105377182-GGT3P.NCBI.                                          gcccagcataactatttttaatgaagtagactttaagaagaaaagtattattagaggtaa 31396
human.FAM230J-LOC105372942-GGTLC5P.NCBI.rev.compl                               gcccagcataactatttttaatgaagtagactttaagaagaaaagtattattagaggtaa 32116
human.FAM230A.ncRNA.18487127.18500594-GGTLC3.18516335.18518165.rev.compl.REF    gcccagcataactatttttaatgaagtagactttaagaagaaaagtattattagaggtaa 20873
                                                                                ************ ****************************************** ****

chimp.LOC112206744-LOC107973052-GGT2.revcompl                                   gagacacatcacggaaaagaagaatttactaggagccaggcgcagtggctcatgcctgta 34068
chimp54546-135457.revcompl.Pan.troglodytes.clone.rp43-41g5.GenBank:AC099533.36  GAGACACATCACGGAAAAGAAGAATTTACTAGGAGCCAGGCGCAGTGGCTCGTGCCTGTA 35801
human.FAM30B-LOC105372935-GGT2.NCBI.                                            gagacacatcacagaaaagaagaatttactaggagccaggcgcaatggctcgtgcctgta 32719
human.FAM230E-LOC105377182-GGT3P.NCBI.                                          gagacacatcacagaaaagaagaatttactaggagccaggcgcaatggctcgtgcctgta 31456
human.FAM230J-LOC105372942-GGTLC5P.NCBI.rev.compl                               gggacacatcacagaaaagaagaatttactaggagccaggcgcaatggctcgtgcctgta 32176
human.FAM230A.ncRNA.18487127.18500594-GGTLC3.18516335.18518165.rev.compl.REF    gggacacatcacagaaaagaagaatttactaggagccaggcgcaatggctcgtgcctgta 20933
                                                                                * ********** ******************************* ****** ********

chimp.LOC112206744-LOC107973052-GGT2.revcompl                                   attccagcactttgtgaggccaaggcggcggatcacctgaggttgggagttcaagaccag 34128
chimp54546-135457.revcompl.Pan.troglodytes.clone.rp43-41g5.GenBank:AC099533.36  ATTCCAGCACTTTGTGAGGCCAAGGCGGCGGATCACCTGAGGTTGGGAGTTCAAGACCAG 35861
human.FAM30B-LOC105372935-GGT2.NCBI.                                            attccagcactttgtgaggccaaggcggcggatcacctgaggttgggagttcaagaccag 32779
human.FAM230E-LOC105377182-GGT3P.NCBI.                                          attccagcactttgtgaggccaaggcggcggatcacctgaggttgggagttcaagaccag 31516
human.FAM230J-LOC105372942-GGTLC5P.NCBI.rev.compl                               attccagcactttgtgaggccaaggcggcggatcacctgaggttgggagttcaagaccag 32236
human.FAM230A.ncRNA.18487127.18500594-GGTLC3.18516335.18518165.rev.compl.REF    attccagcactttgtgaggccaaggcggcggatcacctgaggttgggagttcaagaccag 20993
                                                                                ************************************************************

chimp.LOC112206744-LOC107973052-GGT2.revcompl                                   cctgaccaacatggagaagccctgtctctactaaaaatacaaaaattagccaagcatggt 34188
chimp54546-135457.revcompl.Pan.troglodytes.clone.rp43-41g5.GenBank:AC099533.36  CCTGACCAACATGGAGAAGCCCTGTCTCTACTAAAAATACAAAAATTAGCCAAGCATGGT 35921
human.FAM30B-LOC105372935-GGT2.NCBI.                                            cctgaccaacatggagaagccctgtctctactaaaaatacaaaaattagccaagcatggt 32839
human.FAM230E-LOC105377182-GGT3P.NCBI.                                          cctgaccaacatggagaagccctgtctctactaaaaatacaaaaattagccaagcatggt 31576
human.FAM230J-LOC105372942-GGTLC5P.NCBI.rev.compl                               cctgaccaacatggagaagccctgtctctactaaaaatacaaaaattagccaagcatggt 32296
human.FAM230A.ncRNA.18487127.18500594-GGTLC3.18516335.18518165.rev.compl.REF    cctgaccaacatggagaagccctgtctctactaaaaatacaaaaattagccaagcatggt 21053
                                                                                ************************************************************

chimp.LOC112206744-LOC107973052-GGT2.revcompl                                   ggcacatgcctgtaatcccagctactcaggaggctgagggaggagaattgcttggaccca 34248
chimp54546-135457.revcompl.Pan.troglodytes.clone.rp43-41g5.GenBank:AC099533.36  GGCACATGCCTGTAATCCCAGCTACTCAGGAGGCTGAGGGAGGAGAATTGCTTGGACCCA 35981
human.FAM30B-LOC105372935-GGT2.NCBI.                                            ggcacatgcctgtaatcccagctactcaggaggctgagggaggagaattgcttggaccca 32899
human.FAM230E-LOC105377182-GGT3P.NCBI.                                          ggcacatgcctgtaatcccagctactcaggaggctgagggaggagaattgcttggaccca 31636
human.FAM230J-LOC105372942-GGTLC5P.NCBI.rev.compl                               ggcacatgcctgtaatcccagctactcaggaggctgagggaggagaattgcttggaccca 32356
human.FAM230A.ncRNA.18487127.18500594-GGTLC3.18516335.18518165.rev.compl.REF    ggcacatgcctgtaatcccagctactcaggaggctgagggaggagaattgcttggaccca 21113
                                                                                ************************************************************

chimp.LOC112206744-LOC107973052-GGT2.revcompl                                   ggaagtggaggttgcggtgagctgagattgtgccattgcattccagcctgggcaacaaga 34308
chimp54546-135457.revcompl.Pan.troglodytes.clone.rp43-41g5.GenBank:AC099533.36  GGAAGTGGAGGTTGCGGTGAGCTGAGATTGTGCCATTGCATTCCAGCCTGGGCAACAAGA 36041
human.FAM30B-LOC105372935-GGT2.NCBI.                                            ggaagtggaggttgcggtgagctgagattgtgccattgcattccagcctgggcaacaaga 32959
human.FAM230E-LOC105377182-GGT3P.NCBI.                                          ggaagtggaggttgcggtgagctgagactgtgccattgcattccagcctgggcaacaaga 31696
human.FAM230J-LOC105372942-GGTLC5P.NCBI.rev.compl                               ggaagtggaggttgcggtgagctgagattgtgccattgcattccagcctgggcaacaaga 32416
human.FAM230A.ncRNA.18487127.18500594-GGTLC3.18516335.18518165.rev.compl.REF    ggaagtggaggttgcggtgagctgagattgtgccattgcattccagcctgggcaacaaga 21173
                                                                                *************************** ********************************

chimp.LOC112206744-LOC107973052-GGT2.revcompl                                   gcaaaactctgtctc-------------aaaaaaaaagaagttactagctagttacggta 34355
chimp54546-135457.revcompl.Pan.troglodytes.clone.rp43-41g5.GenBank:AC099533.36  GCAAAACTCTGTCTC---------AAAAAAAAAAAAAGAAGTTACTAGCTAGTTTTGGTA 36092
human.FAM30B-LOC105372935-GGT2.NCBI.                                            gcaaaactctgtctc---aaaaaaaaaaaaaaaaaaaagaagttactagctagtttcggta 33016
human.FAM230E-LOC105377182-GGT3P.NCBI.                                          gcaaaactctgtctcaaaaaaaaaaaaaaaaaaaaaaagaagttactagctagtttcggta 31756
human.FAM230J-LOC105372942-GGTLC5P.NCBI.rev.compl                               gcaaaactctgtctc-----aaaaaaaaaaaaaaaaaagaagttactagctagtttcggta 32471
human.FAM230A.ncRNA.18487127.18500594-GGTLC3.18516335.18518165.rev.compl.REF    gcaaaactctgtctc----------aaaaaaaaaaaaagaagttactagctagtttcggta 21223
                                                                                ***************             **************************  ****

chimp.LOC112206744-LOC107973052-GGT2.revcompl                                   attcttaacatccaggaaactgg-ttgtgaaagtttttcagagaaactaaaccaatagat 34414
chimp54546-135457.revcompl.Pan.troglodytes.clone.rp43-41g5.GenBank:AC099533.36  ATTCTTAACATCCAGGAAACTGG-ATGTGAAAGTTTTTCAGAGAAACTAAACCAATAGAT 36151
human.FAM30B-LOC105372935-GGT2.NCBI.                                            attcttaacaaccaggaaactgg-atgtgaaagtttttcagagaaactaaaccaatagat 33075
human.FAM230E-LOC105377182-GGT3P.NCBI.                                          attcttaacaaccaggaaactggaatgtgaaagtttttcagagaaactaaaccaatagat 31816
human.FAM230J-LOC105372942-GGTLC5P.NCBI.rev.compl                               attcttaacatccaggaaactggaatgtgaaagtttttcagagaaactaaaccaatagat 32531
human.FAM230A.ncRNA.18487127.18500594-GGTLC3.18516335.18518165.rev.compl.REF    attcttaacatccaggaaactgg-atgtgaaagtttttcagagaaactaaaccaatagat 21282
                                                                                ********** ***********  ************************************

chimp.LOC112206744-LOC107973052-GGT2.revcompl                                   tatacatagagagagatttattgaggaattggctcacatgattgtggggactagcaag-t 34473
chimp54546-135457.revcompl.Pan.troglodytes.clone.rp43-41g5.GenBank:AC099533.36  TATACATAGAGAGAGATTTATTGAGGAATTGGCTCACATGATTGTGGGGACTAGCAAG-T 36210
human.FAM30B-LOC105372935-GGT2.NCBI.                                            tatacatagagagagatttatttaggaattggctcacatgattgtggggactagcaagtt 33135
human.FAM230E-LOC105377182-GGT3P.NCBI.                                          tatacatagagagagatttatttaggaattggctcacatgattgtggggactagcaagtt 31876
human.FAM230J-LOC105372942-GGTLC5P.NCBI.rev.compl                               tatacatagagagagacttatttaggaattggctcacatgattgtggggactagcaagtt 32591
human.FAM230A.ncRNA.18487127.18500594-GGTLC3.18516335.18518165.rev.compl.REF    tatacatagagagagacttatttaggaattggctcacatgattgtggggactagcaagtt 21342
                                                                                **************** ***** *********************************** *

chimp.LOC112206744-LOC107973052-GGT2.revcompl                                   ttaaaatctgtagggcaagccagcaggctataaattcaggtaagagttgatctcgaagtc 34533
chimp54546-135457.revcompl.Pan.troglodytes.clone.rp43-41g5.GenBank:AC099533.36  TTAAAATCTGTAGGGCAAGCCAGCAGGCTATAGATTCAGGTAAGAGTTGATCTCGAAGTC 36270
human.FAM30B-LOC105372935-GGT2.NCBI.                                            ttaaaatctgtagggcaagccagcaggctataaattcaggtaagagttgatctcgaagtc 33195
human.FAM230E-LOC105377182-GGT3P.NCBI.                                          ttaaaatctgtagggcaagccagcaggctataaattcaggtaagagttgatctcgaagtc 31936
human.FAM230J-LOC105372942-GGTLC5P.NCBI.rev.compl                               ttaaaatctgtagggcaagccagcaggctataaattcaggtaagagttgatctcgaagtc 32651
human.FAM230A.ncRNA.18487127.18500594-GGTLC3.18516335.18518165.rev.compl.REF    ttaaaatctgtagggcaagccagcaggctataaattcaggtaagagttgatctcgaagtc 21402
                                                                                ******************************** ***************************

chimp.LOC112206744-LOC107973052-GGT2.revcompl                                   tggaacctaaaatctgtagagcagtcagcaggccagaaactcaggcagggtttgtgtgtt 34593
chimp54546-135457.revcompl.Pan.troglodytes.clone.rp43-41g5.GenBank:AC099533.36  TTGAACCTAAAATCTGTAGAGCAGTCAGCAGGCCAGAAACTCAGGCAGGGTTTGTGTGTT 36330
human.FAM30B-LOC105372935-GGT2.NCBI.                                            tggaacctaaaatctgtagagcagtcagcaggccagaaactcaggcagggtttgtgtgtt 33255
human.FAM230E-LOC105377182-GGT3P.NCBI.                                          tggaacctaaaatctgtagagcagtcagcaggccagaaactcaggcagggtttgtgtgtt 31996
human.FAM230J-LOC105372942-GGTLC5P.NCBI.rev.compl                               tggaacctaaaatctgtagagcagtcagcaggccagaaactcaggtagggtttgtgtgtt 32711
human.FAM230A.ncRNA.18487127.18500594-GGTLC3.18516335.18518165.rev.compl.REF    tggaacctaaaatctgtagagcagtcagcaggccagaaactcaggcagggtttgtgtgtt 21462
                                                                                * ******************************************* **************

chimp.LOC112206744-LOC107973052-GGT2.revcompl                                   acagtcttgaagcagaattcctgcttctttgggaaacct--gtttttgttcttaaggcct 34651
chimp54546-135457.revcompl.Pan.troglodytes.clone.rp43-41g5.GenBank:AC099533.36  ATAGTCTTGAAGCAGAATTCCTGCTTCTCTGGGAAACCTCAGTTTTTGTTCTTAAGGCCT 36390
human.FAM30B-LOC105372935-GGT2.NCBI.                                            acagtcttgaagcagaattcctgcttctctgggaaacctcagtttttgttcttaaggcct 33315
human.FAM230E-LOC105377182-GGT3P.NCBI.                                          acagtcttgaagcagaattcctgcttctctgggaaacctcagtttttgttcttaaggcct 32056
human.FAM230J-LOC105372942-GGTLC5P.NCBI.rev.compl                               acagtcttgaagcagaattcctgcttctctgggaaacctcagtttttgttcttaaggcct 32771
human.FAM230A.ncRNA.18487127.18500594-GGTLC3.18516335.18518165.rev.compl.REF    acagtcttgaagcagaattcctgcttctctgggaaacctcagtttttgttcttaaggcct 21522
                                                                                * ************************** **********  *******************

chimp.LOC112206744-LOC107973052-GGT2.revcompl                                   tcaagtgattggaggtggcccacccatattatggtgggtaatctgttttacttaaagtca 34711
chimp54546-135457.revcompl.Pan.troglodytes.clone.rp43-41g5.GenBank:AC099533.36  TCAACTGATTGGAGGTGGCCCACCCATATTATGGTGGGTAATCTGTTTTACTTAAAGTCA 36450
human.FAM30B-LOC105372935-GGT2.NCBI.                                            tcaactgattggaggtggcccacccatattatggtgggtaatctgttttacttaaagtca 33375
human.FAM230E-LOC105377182-GGT3P.NCBI.                                          tcaactgattggaggtggcccacccatattatggtgggtaatctgttttacttaaagtca 32116
human.FAM230J-LOC105372942-GGTLC5P.NCBI.rev.compl                               tcaactgattggaggtggcccacccatattatggtgggtaatctgttttacttaaagtca 32831
human.FAM230A.ncRNA.18487127.18500594-GGTLC3.18516335.18518165.rev.compl.REF    tcaactgattggaggtggcccacccatattatggtgggtaatctgttttacttaaagtca 21582
                                                                                **** *******************************************************

chimp.LOC112206744-LOC107973052-GGT2.revcompl                                   attgactgtcagtgttaaccacatctatgaaataacctcccagcaagatattgacaagta 34771
chimp54546-135457.revcompl.Pan.troglodytes.clone.rp43-41g5.GenBank:AC099533.36  ACTGACTGTCAGTGTTAACCACATCTATGAAATAACCTCCCAGCAAGATATTGACAAGTA 36510
human.FAM30B-LOC105372935-GGT2.NCBI.                                            attgactgtcagtgttaatcacatctatgaaataacctcccagcaagatattgacaagta 33435
human.FAM230E-LOC105377182-GGT3P.NCBI.                                          attgactgtcagtgttaatcacatctatgaaataacctcccagcaagatattgacaagta 32176
```

```
human.FAM230J-LOC105372942-GGTLC5P.NCBI.rev.compl                          attgactgtcagtgttaatcacatctatgaaataacctcccagcaagatattgacaagta 32891
human.FAM230A.ncRNA.18487127.18500594-GGTLC3.18516335.18518165.rev.compl.REF attgactgtcagtgttaatcacatctatgaaataacctcccagcaagatattgacaagta 21642
                                                                           * *************** *******************************************

chimp.LOC112206744-LOC107973052-GGT2.revcompl                              tttgaccaaacaacggggcaccatagcttagccaagttgacacataaattaaccatcagg 34831
chimp54546-135457.revcompl.Pan.troglodytes.clone.rp43-41g5.GenBank:AC099533.36 TTTGACCAAACAACGGGGCACCATAGCTTAGCCAAGTTGACACATAAATTAACCATCAGG 36570
human.FAM30B-LOC105372935-GGT2.NCBI.                                       tttgaccaaacaacaggacaccatagcttagccaagttgacacataaattaaccatcagg 33495
human.FAM230E-LOC105377182-GGT3P.NCBI.                                     ttcgaccaaacaacaggacaccatagcttagccaagttgacacataaattaaccatcagg 32236
human.FAM230J-LOC105372942-GGTLC5P.NCBI.rev.compl                          tttgaccaaacaacaggacaccatagcttagccaagttgacacataaattaaccatcagg 32951
human.FAM230A.ncRNA.18487127.18500594-GGTLC3.18516335.18518165.rev.compl.REF tttgaccaaacaacaggacaccatagcttagccaagttgacacataaattaaccatcagg 21702
                                                                           ** *********** ** *****************************************

chimp.LOC112206744-LOC107973052-GGT2.revcompl                              agcgagtagaatatccaaaaaacaacatactaggggtattatatcttatatagc-----t 34886
chimp54546-135457.revcompl.Pan.troglodytes.clone.rp43-41g5.GenBank:AC099533.36 AGCGAGTAGAATATCCAAAAAACAACATACTAGGGGTATTATATCTTATATAGC-----T 36625
human.FAM30B-LOC105372935-GGT2.NCBI.                                       agcaagtagaatatccaaaaaacaacatactaggggtattatatcttatatagctattat 33555
human.FAM230E-LOC105377182-GGT3P.NCBI.                                     agcaagtagaatatccaaaaaacaacatactaggggtattatatcttatatagctattat 32296
human.FAM230J-LOC105372942-GGTLC5P.NCBI.rev.compl                          agcaagtagaatatccaaaaaacaacatactaggggtattatatcttatatagctattat 33011
human.FAM230A.ncRNA.18487127.18500594-GGTLC3.18516335.18518165.rev.compl.REF agcaagtagaatatccaaaaaacaacatactaggggtattatatcttatatagctattat 21762
                                                                           *** **************************************************     *

chimp.LOC112206744-LOC107973052-GGT2.revcompl                              aattatataaaacatataattatagaatgaagatattaagataaccattagaacaaaaat 34946
chimp54546-135457.revcompl.Pan.troglodytes.clone.rp43-41g5.GenBank:AC099533.36 AATTATATAAAACATATAATTATAGAATGAAGATATTAAGATAACCATTAGAACAAAAAT 36685
human.FAM30B-LOC105372935-GGT2.NCBI.                                       aattatataaaacatataattatagaatgacgatattaagataaccattagaacaaaaat 33615
human.FAM230E-LOC105377182-GGT3P.NCBI.                                     aattatataaaacatataattatagaatgacgatattaagataaccattagaacaaaaat 32356
human.FAM230J-LOC105372942-GGTLC5P.NCBI.rev.compl                          aattatataaaacatataattatagaatgacgatattaagataagcattagaacaaaaat 33071
human.FAM230A.ncRNA.18487127.18500594-GGTLC3.18516335.18518165.rev.compl.REF aattatataaaacatataattatagaatgacgatattaagataaccattagaacaaaaat 21822
                                                                           ***************************** ************ **************

chimp.LOC112206744-LOC107973052-GGT2.revcompl                              ataaactttcttt-------ttctttttttttttgagaccaagtcttgctctgtcaccca 34999
chimp54546-135457.revcompl.Pan.troglodytes.clone.rp43-41g5.GenBank:AC099533.36 ATAAACTTTCTT-------TTTTTTTTTTTTTGAGACCAAGTCTTGCTCTGTCACCCA 36738
human.FAM30B-LOC105372935-GGT2.NCBI.                                       ataaactttctttt-ctttctttttttttttttttgagaccaagtcttgctctgtcaccca 33674
human.FAM230E-LOC105377182-GGT3P.NCBI.                                     ataaactttctttcc----ttttttttttttttgagaccaagtcttgctctgtcaccca 32412
human.FAM230J-LOC105372942-GGTLC5P.NCBI.rev.compl                          ataaactttctttc-ttttttttttttttttttgagaccaagtcttgctctgtcaccca 33130
human.FAM230A.ncRNA.18487127.18500594-GGTLC3.18516335.18518165.rev.compl.REF ataaactttctttcttttttttttttttttttttgagaccaagtcttgctctgtcaccca 21882
                                                                           ************       ** ************************************

chimp.LOC112206744-LOC107973052-GGT2.revcompl                              ggctggagtgcagtggtgcaatcttggcttactgcaacctttgcctcctgagttcaagtg 35059
chimp54546-135457.revcompl.Pan.troglodytes.clone.rp43-41g5.GenBank:AC099533.36 GGCTGGAGTGCAGTGGTGCAATCTTGGCTTACTGCAACCTTTGCCTCCTGGGTTCAAGTG 36798
human.FAM30B-LOC105372935-GGT2.NCBI.                                       ggctggagtgcagtggtgcaatcttggcttactgaaacctttgcctcctgggttcaagtg 33734
human.FAM230E-LOC105377182-GGT3P.NCBI.                                     ggctggagtgcagtggtgcaatcttggcttactgcaacctttgcctcctgcgttcaagtg 32472
human.FAM230J-LOC105372942-GGTLC5P.NCBI.rev.compl                          ggctggagtgcagtggtgcaatcttggcttactgcaacctttgcctcctgcgttcaagtg 33190
human.FAM230A.ncRNA.18487127.18500594-GGTLC3.18516335.18518165.rev.compl.REF ggctggagtgcagtggtgcaatcttggcttactgcaacctttgcctcctgcgttcaagtg 21942
                                                                           ********************************* *************** *********

chimp.LOC112206744-LOC107973052-GGT2.revcompl                              atcctcctgtctcagcctcccaagtagctgggattacaggcacctgctaccatgcccagc 35119
chimp54546-135457.revcompl.Pan.troglodytes.clone.rp43-41g5.GenBank:AC099533.36 ATTCTCCTGTCTCAGCCTCCCAAGTATCTGGGATTACAGGCATCCGCTACCATGCCCAGC 36858
human.FAM30B-LOC105372935-GGT2.NCBI.                                       attctcctgtctcagcctcccaagtatctgggattacaggcacctgctaccatgcccagc 33794
human.FAM230E-LOC105377182-GGT3P.NCBI.                                     attctcctgtctcagcctcccaagtatctgggattacaggcacctgctaccatgcccagc 32532
human.FAM230J-LOC105372942-GGTLC5P.NCBI.rev.compl                          attctcctgtctcagcctcccaagtatctgggattacaggcacctgctaccatgcccagc 33250
human.FAM230A.ncRNA.18487127.18500594-GGTLC3.18516335.18518165.rev.compl.REF attctcctgtctcagcctcccaagtatctgggattacaggcacctgctaccatgcccagc 22002
                                                                           ** ********************** ************** * ***************

chimp.LOC112206744-LOC107973052-GGT2.revcompl                              taatttttgtatttttagtagagacatggtttcaccatgttgcccaggctggtctccaac 35179
chimp54546-135457.revcompl.Pan.troglodytes.clone.rp43-41g5.GenBank:AC099533.36 TAATTTTTGTATTTTTAGTAGAGACATGGTTTCACCATGTTGCCCAGGCTGGTCTCCAAC 36918
human.FAM30B-LOC105372935-GGT2.NCBI.                                       taatttttgtatttttagtagagacatggtttcaccatgttgcccaggctggtctccaac 33854
human.FAM230E-LOC105377182-GGT3P.NCBI.                                     taatttttgtatttttagtagagacatggtttcaccatgttgcccaggctggtctccaac 32592
human.FAM230J-LOC105372942-GGTLC5P.NCBI.rev.compl                          taatttttgtatttttagtagagacatggtttcaccatgttgcccaggctggtctccaac 33310
human.FAM230A.ncRNA.18487127.18500594-GGTLC3.18516335.18518165.rev.compl.REF taatttttgtatttttagtagagacatggtttcaccatgttgcccaggctggtctccaac 22062
                                                                           ************************************************************

chimp.LOC112206744-LOC107973052-GGT2.revcompl                              tcctgacctcaagtgagccagccccctcggcctcccaaagtgctgggattacaggtgtga 35239
chimp54546-135457.revcompl.Pan.troglodytes.clone.rp43-41g5.GenBank:AC099533.36 TCCTGACCTCAAGTGAGCCACCCCCTCGGCCTCCCAAAGTGCTGGAATTACAGGTGTGA 36978
human.FAM30B-LOC105372935-GGT2.NCBI.                                       tcctgacctcaagtgagccaccccctcggcctcccaaagtgctgggattacaggtgtga 33914
human.FAM230E-LOC105377182-GGT3P.NCBI.                                     tcctgacctcaagtgagccaccccctcggcctcccaaagtgctgggattacaggtgtga 32652
human.FAM230J-LOC105372942-GGTLC5P.NCBI.rev.compl                          tcctgacctcaagtgagccaccccctcggcctcccaaagtgctgggattacaggtgtga 33370
human.FAM230A.ncRNA.18487127.18500594-GGTLC3.18516335.18518165.rev.compl.REF tcctgacctcaagtgagccaccccctcggcctcccaaagtgctgggattacaggtgtga 22122
                                                                           ******************* ****** ****************** ************

chimp.LOC112206744-LOC107973052-GGT2.revcompl                              gccaccacacccagccaaaaatcaccttttttcacaaggatcaaaacagtcattacgctgg 35299
chimp54546-135457.revcompl.Pan.troglodytes.clone.rp43-41g5.GenBank:AC099533.36 GCCACCACACCCAGCCAAAAATCACCTTTTTCACAAGGATCAAAACAGTCATTACGCTGG 37038
human.FAM30B-LOC105372935-GGT2.NCBI.                                       gccaccacacccagccaaaaatcacctttttttacaaggatcaaaacagtcattatgctgg 33974
human.FAM230E-LOC105377182-GGT3P.NCBI.                                     gccaccacacccagccaaaaatcacctttttttacaaggatcaaaacagtcattatgctgg 32712
human.FAM230J-LOC105372942-GGTLC5P.NCBI.rev.compl                          gccaccacacccagccaaaaatcacctttttttacaaggatcaaaacagtcattatgctgg 33430
human.FAM230A.ncRNA.18487127.18500594-GGTLC3.18516335.18518165.rev.compl.REF gccaccacacccagccaaaaatcacctttttttacaaggatcaaaacagtcattatgctgg 22182
                                                                           ******************************* ********************** *****

chimp.LOC112206744-LOC107973052-GGT2.revcompl                              agatgacagacctcactgtcaccatgctcccttttgtatgtctactaggcatggtgctggg 35359
chimp54546-135457.revcompl.Pan.troglodytes.clone.rp43-41g5.GenBank:AC099533.36 AGATGACAGACCTCACTGTCACCATGCTCCCTTTTGTATGTCTACTAGGCACGGTGCTGGG 37098
human.FAM30B-LOC105372935-GGT2.NCBI.                                       agatgacagacctcactgtcaccatgctcccttttgtatgtctactaggcacggtgctggg 34034
human.FAM230E-LOC105377182-GGT3P.NCBI.                                     agatgacagacctcactgtcaccatgctcccttttgtatgtctactaggcacggtgctggg 32772
human.FAM230J-LOC105372942-GGTLC5P.NCBI.rev.compl                          agatgacagacctcactgtcaccatgctcccttttgtatgtctactaggcacggtgctggg 33490
human.FAM230A.ncRNA.18487127.18500594-GGTLC3.18516335.18518165.rev.compl.REF agatgacagacctcactgtcaccatgctcccttttgtatgtctactaggcacggtgctggg 22242
                                                                           *************************************************** *********

chimp.LOC112206744-LOC107973052-GGT2.revcompl                              tccacactcacagaaagcttaggagctcgcacccaggggctccggctgtagcagaatccc 35419
chimp54546-135457.revcompl.Pan.troglodytes.clone.rp43-41g5.GenBank:AC099533.36 TCCACACTCACAGAAAGCTTAGGAGCTCGCACCCAGGGGCTCCGGCTGTAGCAGAATCCC 37158
human.FAM30B-LOC105372935-GGT2.NCBI.                                       tccacactcacagaaaccttaggaactcgcacccaggggctccggctgtagcagaatccc 34094
human.FAM230E-LOC105377182-GGT3P.NCBI.                                     tccacactcacagaaaccttaggaactcgcacccaggggctccggctgtagcagaatccc 32832
human.FAM230J-LOC105372942-GGTLC5P.NCBI.rev.compl                          tccacactcacagaaaccttaggaactcgcacccaggggctccggctgtagcagaatccc 33550
human.FAM230A.ncRNA.18487127.18500594-GGTLC3.18516335.18518165.rev.compl.REF tccacactcacagaaaccttaggaactcgcacccaggggctccggctgtagcagaatccc 22302
                                                                           **************** ******* ***********************************

chimp.LOC112206744-LOC107973052-GGT2.revcompl                              aagaataaaacctggtgctgaaagagtaggagatgaggccggatgccatgactcactcct 35479
chimp54546-135457.revcompl.Pan.troglodytes.clone.rp43-41g5.GenBank:AC099533.36 AAGAATAAAACCTGGTGCTGAAAGAGTAGGAGATGAGGCCGGATGCCATGACTCACTCCT 37218
human.FAM30B-LOC105372935-GGT2.NCBI.                                       aagaataaaacctggtgctgaaagagtaggagatgaggccaggtgccatgactcactcct 34154
human.FAM230E-LOC105377182-GGT3P.NCBI.                                     aagaataaaacctggtgctgaaagagtaggagatgaggccgggtgccatgactcactcct 32892
human.FAM230J-LOC105372942-GGTLC5P.NCBI.rev.compl                          aagaataaaacctggtgctgaaagagtaggagatgaggccgggtgccatgactcactcct 33610
human.FAM230A.ncRNA.18487127.18500594-GGTLC3.18516335.18518165.rev.compl.REF aagaataaaacctggtgctgaaagagtaggagatgaggccgggtgccatgactcactcct 22362
                                                                           **************************************** * ****************

chimp.LOC112206744-LOC107973052-GGT2.revcompl                              gtaatgccagcactttgggtggccaaggcgggtgaatcaagagatagagaccatcct-gg 35538
chimp54546-135457.revcompl.Pan.troglodytes.clone.rp43-41g5.GenBank:AC099533.36 GTAATGCCAGCACTTTGGGTGGCCAAGGCGGGTGAATCAAGAGATAGAGACCATCCT-GG 37277
human.FAM30B-LOC105372935-GGT2.NCBI.                                       gtaatgccagcactttgggtggccaaggcgggtgaatcaagagatagagaccatcct-gg 34213
human.FAM230E-LOC105377182-GGT3P.NCBI.                                     gtaatgccagcactttgggtggccaaggcgggtgaatcaagagatagagaccatcct-gg 32951
human.FAM230J-LOC105372942-GGTLC5P.NCBI.rev.compl                          gtaatgccagcactttgggtggccaaggcgggtgaatcaagagatagagaccatcctggg 33670
human.FAM230A.ncRNA.18487127.18500594-GGTLC3.18516335.18518165.rev.compl.REF gtaatgccagcactttgggtggccaaggcgggtgaatcaagagatagagaccatcct-gg 22421
                                                                           ********************************************************* **

chimp.LOC112206744-LOC107973052-GGT2.revcompl                              ccaacatggtgaaaccccgtctctactaaaaatacaaaaattagctgggcgtggtggctg 35598
chimp54546-135457.revcompl.Pan.troglodytes.clone.rp43-41g5.GenBank:AC099533.36 CCAACATGGTGAAACCCCGTCTCTACTAAAAATACAAAAATTAGCTGGGCGTGGTGGCTG 37337
human.FAM30B-LOC105372935-GGT2.NCBI.                                       ccaacacggtgaaaccccgtctctactaaaaatacagaaattagcggggcgtggtggctg 34273
human.FAM230E-LOC105377182-GGT3P.NCBI.                                     ccaacacggtgaaaccccgtctctactaaaaatacagaaattagcggggcgtggtggctg 33011
human.FAM230J-LOC105372942-GGTLC5P.NCBI.rev.compl                          ccaacatggtgaaaccctgtctctactaaaaatacagaaattagcggggcgtggtggctg 33730
human.FAM230A.ncRNA.18487127.18500594-GGTLC3.18516335.18518165.rev.compl.REF ccaacacggtgaaaccccgtctctactaaaaatacagaaattagcggggcgtggtggctg 22481
                                                                           ****** ********** ****************** ******** **************

chimp.LOC112206744-LOC107973052-GGT2.revcompl                              gcacctgtagtcccagctactcaggaggctgaggcaggagaatcatttgaaccgaggaag 35658
chimp54546-135457.revcompl.Pan.troglodytes.clone.rp43-41g5.GenBank:AC099533.36 GCACCTGTAGTCCCAGCTACTCAGGAGGCTGAGGCAGGAGAATCATTTGAACCGAGGAAG 37397
human.FAM30B-LOC105372935-GGT2.NCBI.                                       gcacctgtagtcccagctactcaggaggctgaggcaggagaatcatttgaaccgaggaag 34333
human.FAM230E-LOC105377182-GGT3P.NCBI.                                     gcacctgtagtcccagctactcaggaggctgaggcaggagaatcatttgaaccgaggaag 33071
human.FAM230J-LOC105372942-GGTLC5P.NCBI.rev.compl                          gcacctgtagtcccagctactcaggaggctgaggcaggagaatcatttgaaccgaggaag 33790
human.FAM230A.ncRNA.18487127.18500594-GGTLC3.18516335.18518165.rev.compl.REF gcacctgtagtcccagctactcaggaggctgaggcaggagaatcatttgaaccgaggaag 22541
                                                                           ************************************************************

chimp.LOC112206744-LOC107973052-GGT2.revcompl                              cagaggttgcagtaagctgagatcgtgccactgcactccagcctggtgacagagtgagac 35718
chimp54546-135457.revcompl.Pan.troglodytes.clone.rp43-41g5.GenBank:AC099533.36 CAGAGGTTGCAGTAAGCTGAGATCACGCCACTGCACTCCAGCCTGGTGACAGAGTGAGAC 37457
human.FAM30B-LOC105372935-GGT2.NCBI.                                       cagaggttgcagtaagctgagatcgcgccactgcactccagcctggtgacagagtgagac 34393
human.FAM230E-LOC105377182-GGT3P.NCBI.                                     cagaggttgcagtaagctgagatcgcgccactgcactccagcctggtgacagagtgagac 33131
human.FAM230J-LOC105372942-GGTLC5P.NCBI.rev.compl                          cagaggttgcagtaagctgagatcgcgccactgcactccagcctggtgacagagtgagac 33850
human.FAM230A.ncRNA.18487127.18500594-GGTLC3.18516335.18518165.rev.compl.REF cagaggttgcagtaagctgagatcgcgccactgcactccagcctggtgacagagtgagac 22601
                                                                           ************************  **********************************

chimp.LOC112206744-LOC107973052-GGT2.revcompl                              accgtctc--aaaaaaaaaaaaaaaaaaaaaagcaggacactgaactctgggagggcc 35776
chimp54546-135457.revcompl.Pan.troglodytes.clone.rp43-41g5.GenBank:AC099533.36 ACCGTCTC---AAAAAGAAAAAAAAAAAAAAAAGCAGGACACTGAACTCTGGGAGGGCC 37514
human.FAM30B-LOC105372935-GGT2.NCBI.                                       accgtcac---------aaaaaaaaaaaaaaaaagcaggacactgaactctgggagggcc 34444
human.FAM230E-LOC105377182-GGT3P.NCBI.                                     accgtcacaaaaaaaaaaaaaaaaaaaaaaaagcaggacactgaactctgggagggcc 33191
human.FAM230J-LOC105372942-GGTLC5P.NCBI.rev.compl                          accgtcacaaaaaaaaaaaaaaaaaaaaaaaaaaagcaggacactgaactctgggagggcc 33910
human.FAM230A.ncRNA.18487127.18500594-GGTLC3.18516335.18518165.rev.compl.REF accgtccc---aaaaaaaaaaaaaaaaaaaaaaagcaggacactgaactctgggagggcc 22658
                                                                           ****** *         ******************************************

chimp.LOC112206744-LOC107973052-GGT2.revcompl                              tcctggtgagaggtgagcacagaggggagagatggaggcaggagcatgggcttctggtgg 35836
chimp54546-135457.revcompl.Pan.troglodytes.clone.rp43-41g5.GenBank:AC099533.36 TCCTGGTGAGAGGTGAGCACAGAGGGGAGAGATGGAGGCAGGAGCATGGGCTTCTGGTGG 37574
human.FAM30B-LOC105372935-GGT2.NCBI.                                       tcctggtgagaggtgagcacagaggggagagatggaggcaggagcatgggcttctggtgg 34504
human.FAM230E-LOC105377182-GGT3P.NCBI.                                     tcctggtgagaggtgagcacagaggggagagatggaggcaggagcatgggcttctggtgg 33251
human.FAM230J-LOC105372942-GGTLC5P.NCBI.rev.compl                          tcctggtgagaggtgagcacagaggggagagatggaggcaggagcatgggcttctggtgg 33970
```

```
human.FAM230A.ncRNA.18487127.18500594-GGTLC3.18516335.18518165.rev.compl.REF        tcctggtgagaggtgagcacagaggggagagatggaggcaggagcatgggcttctggtgg 22718
                                                                                    ************************************************************

chimp.LOC112206744-LOC107973052-GGT2.revcompl                                       ccccagcagaccctgtggcagcgtggccagggtcctctgcagggaggaatcttggccagg 35896
chimp54546-135457.revcompl.Pan.troglodytes.clone.rp43-41g5.GenBank:AC099533.36      CCCCAGCAGACCCTGTGGCAATGTGGCCAGGGGCCTCTGCAGGGAGGAATCTTGGCCAGG 37634
human.FAM30B-LOC105372935-GGT2.NCBI.                                                ccccagcagaccctgtggcagcgtggccagggtcctctgcagggaggaatcttggccagg 34564
human.FAM230E-LOC105377182-GGT3P.NCBI.                                              ccccagcagaccctgtggcagcgtggccagggtcctctgcagggaggaatcttggccagg 33311
human.FAM230J-LOC105372942-GGTLC5P.NCBI.rev.compl                                   ccccagcagaccctgtggcagcgtggccagggtcctctgcagggaggaatcttggccagg 34030
human.FAM230A.ncRNA.18487127.18500594-GGTLC3.18516335.18518165.rev.compl.REF        ccccagcagaccctgtggcagcgtggccagggtcctctgcagggaggaatcttggccagg 22778
                                                                                    ********************  ********** ***************************

chimp.LOC112206744-LOC107973052-GGT2.revcompl                                       atgacgctgtagcaggcctcttcctgaggcctccagccagcccggccagggacccagcgt 35956
chimp54546-135457.revcompl.Pan.troglodytes.clone.rp43-41g5.GenBank:AC099533.36      ATGACGCTGTAGCAGGCCTCTTCCTGAGGCCTCCAGCCAGCCCGGCCAGGGTCCCAGCGT 37694
human.FAM30B-LOC105372935-GGT2.NCBI.                                                atgacgctgtagcaggcctcttcctgaggcctccagccagcccggccagggtcccagcgt 34624
human.FAM230E-LOC105377182-GGT3P.NCBI.                                              atgacgctgtagcaggcctcttcctgaggcctccagccagcccggccagggtcccagcgt 33371
human.FAM230J-LOC105372942-GGTLC5P.NCBI.rev.compl                                   atgacgctgtagcaggcctcttcctgaggcctccagccagcccggccagggtcccagcgt 34090
human.FAM230A.ncRNA.18487127.18500594-GGTLC3.18516335.18518165.rev.compl.REF        atgacgctgtagcaggcctcttcctgaggcctccagccagcccggccagggtcccagcgt 22838
                                                                                    *************************************************** ********

chimp.LOC112206744-LOC107973052-GGT2.revcompl                                       ccagtgacccctgtttcacagcagcagctggggccagccccaggctctcttccactccca 36016
chimp54546-135457.revcompl.Pan.troglodytes.clone.rp43-41g5.GenBank:AC099533.36      CCAGTGACCCCTGTTTCGCAGCAGCAGCTGGCGCCAGCCCCAGGCTCTCTTCCACTCCCA 37754
human.FAM30B-LOC105372935-GGT2.NCBI.                                                ccagtgacccctgtttcacagcagcagctggggccagccccaggctctcttccactccca 34684
human.FAM230E-LOC105377182-GGT3P.NCBI.                                              ccagtgacccctgtttcacagcagcagctggggccagccccaggctctcttccactccca 33431
human.FAM230J-LOC105372942-GGTLC5P.NCBI.rev.compl                                   ccagtgacccctgtttcacagcagcagctggggccagccccaggctctcttccactccca 34150
human.FAM230A.ncRNA.18487127.18500594-GGTLC3.18516335.18518165.rev.compl.REF        ccagtgacccctgtttcacagcagcagctggggccagccccaggctctcttccactccca 22898
                                                                                    ***************** ************* ****************************

chimp.LOC112206744-LOC107973052-GGT2.revcompl                                       gcttcttaaaactggaagtggagagagttgtttgataaaacactggggcaaaccacatcc 36076
chimp54546-135457.revcompl.Pan.troglodytes.clone.rp43-41g5.GenBank:AC099533.36      GCTTCTTAAAACTGGAAGTGGAGAGAGTAGTTTGATAAAACAGTGGGGCAAACCACATCC 37814
human.FAM30B-LOC105372935-GGT2.NCBI.                                                gcttcttaaaactggaagtggagagagttgtttgataaaacactggggcaaaccacatcc 34744
human.FAM230E-LOC105377182-GGT3P.NCBI.                                              gcttcttaaaactggaagtggagagagttgtttgataaaacactggggcaaaccacatcc 33491
human.FAM230J-LOC105372942-GGTLC5P.NCBI.rev.compl                                   gcttcttaaaactggaagtggagagagttgtttgataaaacactggggcaaaccacatcc 34210
human.FAM230A.ncRNA.18487127.18500594-GGTLC3.18516335.18518165.rev.compl.REF        gcttcttaaaactggaagtggagagagttgtttgataaaacactggggcaaaccacatcc 22958
                                                                                    **************************** ************* *****************

chimp.LOC112206744-LOC107973052-GGT2.revcompl                                       tctcttcaccaagggagagttcgaggggatgccagcagagggcgctttagagtagagacc 36136
chimp54546-135457.revcompl.Pan.troglodytes.clone.rp43-41g5.GenBank:AC099533.36      TCTCTTCACCAAGGGAGAGTTCGAGGGGATGCCGGCAGAGGGAGCTTTAGAGTAGAGACC 37874
human.FAM30B-LOC105372935-GGT2.NCBI.                                                tctcttcaccaagggagagttggaggggatgccggcagagggagctttagagtagagacc 34804
human.FAM230E-LOC105377182-GGT3P.NCBI.                                              tctcttcaccaagggagagttcgaggggatgccggcagagggagctttagagtagagacc 33551
human.FAM230J-LOC105372942-GGTLC5P.NCBI.rev.compl                                   tctcttcaccaagggagagttcgaggggatgccggcagagggagctttagagtagagacc 34270
human.FAM230A.ncRNA.18487127.18500594-GGTLC3.18516335.18518165.rev.compl.REF        tctcttcaccaagggagagttcgaggggatgccggcagagggagctttagagtagagacc 23018
                                                                                    ********************** ********** ******** *****************

chimp.LOC112206744-LOC107973052-GGT2.revcompl                                       cctacccaaccagtgaccgtcacgcacacagcagggcatgctatggagacccccagacag 36196
chimp54546-135457.revcompl.Pan.troglodytes.clone.rp43-41g5.GenBank:AC099533.36      CCTACCCAACCAGTGACCGTCACGCACACAGCAGGGTATGCTATGGAGACCCCCAGACAG 37934
human.FAM30B-LOC105372935-GGT2.NCBI.                                                cctacccaaccagtgaccgtcacgcacacagcagggcatgctatggagacccccagacag 34864
human.FAM230E-LOC105377182-GGT3P.NCBI.                                              cctacccaaccagtgaccgtcacgcacacagcagggcatgctatggagacccccagacag 33611
human.FAM230J-LOC105372942-GGTLC5P.NCBI.rev.compl                                   cctacccaaccagtgaccgtcacgcacacagcagggcatgctatggagacccccagacag 34330
human.FAM230A.ncRNA.18487127.18500594-GGTLC3.18516335.18518165.rev.compl.REF        cctacccaaccagtgaccgtcacgcacacagcagggcatgctatggagacccccagacag 23078
                                                                                    ************************************ ***********************

chimp.LOC112206744-LOC107973052-GGT2.revcompl                                       tcactcggggagacccagcaggtccagactcttcagagatctgtggcagcaggtccccac 36256
chimp54546-135457.revcompl.Pan.troglodytes.clone.rp43-41g5.GenBank:AC099533.36      TCACTCTGGGAGACCCAGCAGGTCCAGACTCTTCAGAGATCTGTGGCAGCAGGTCCCCAC 37994
human.FAM30B-LOC105372935-GGT2.NCBI.                                                tcactcggggagacccagcaggtccagactcttcagagatctgtggcagcaggtccccac 34924
human.FAM230E-LOC105377182-GGT3P.NCBI.                                              tcactcggggagacccagcaggtccagactcttcagagatctgtggcagcaggtccccac 33671
human.FAM230J-LOC105372942-GGTLC5P.NCBI.rev.compl                                   tcactcggggagacccagcaggtccagactcttcagagatctgtggcagcaggtccccac 34390
human.FAM230A.ncRNA.18487127.18500594-GGTLC3.18516335.18518165.rev.compl.REF        tcactcggggagacccagcaggtccagactcttcagagatctgtggcagcaggtccccac 23138
                                                                                    ****** *****************************************************

chimp.LOC112206744-LOC107973052-GGT2.revcompl                                       tcccaaaagccacgtgcccacgggtggtctctggtgcctgagaccccagtctcatttgca 36316
chimp54546-135457.revcompl.Pan.troglodytes.clone.rp43-41g5.GenBank:AC099533.36      TCCCAAAAGCCACGTGCTCACGGGTGGTCTCTGGTGCCTGAGACCCCAGTCTCATTTGCA 38054
human.FAM30B-LOC105372935-GGT2.NCBI.                                                tcccaaaagccacgtgcccacgggtggtctctggtgcctgagaccccagtctcatttgca 34984
human.FAM230E-LOC105377182-GGT3P.NCBI.                                              tcccaaaagccacgtgcccacgggtggtctctggtgcctgagaccccagtctcatttgca 33731
human.FAM230J-LOC105372942-GGTLC5P.NCBI.rev.compl                                   tcccaaaagccacgtgcccacgggtggtctctggtgcctgagaccccagtctcatttgca 34450
human.FAM230A.ncRNA.18487127.18500594-GGTLC3.18516335.18518165.rev.compl.REF        tcccaaaagccacgtgcccacgggtggtctctggtgcctgagaccccagtctcatttgca 23198
                                                                                    ***************** ******************************************

chimp.LOC112206744-LOC107973052-GGT2.revcompl                                       tctttgcaacttcgagtttaagtgggtgtcgcatctctgtatgtcctcctgagcagagga 36376
chimp54546-135457.revcompl.Pan.troglodytes.clone.rp43-41g5.GenBank:AC099533.36      TCTTTGCAACTTTGAGTTTAAGTGGGTGTCGCATCTCTGTATGTCCCCCTGAGCAGAGGA 38114
human.FAM30B-LOC105372935-GGT2.NCBI.                                                tctttgcaacttcgagtttaagtgggtgtcgcatctctgtatgtcctcccgagcagagga 35044
human.FAM230E-LOC105377182-GGT3P.NCBI.                                              tctttgcaacttcgagtttaagtgggtgtcgcatctctgtatgtcctcccgagcagagga 33791
human.FAM230J-LOC105372942-GGTLC5P.NCBI.rev.compl                                   tctttgcaacttcgagtttaagtgggtgtcgcatctctgtatgtcctcccgagcagagga 34510
human.FAM230A.ncRNA.18487127.18500594-GGTLC3.18516335.18518165.rev.compl.REF        tctttgcaacttcgagtttaagtgggtgtcgcatctctgtatgtcctcccgagcagagga 23258
                                                                                    ************ ********************************* ** **********

chimp.LOC112206744-LOC107973052-GGT2.revcompl                                       ggggcacagcctggggtggcagctggcgtcaaaccctcaaatcccctgagagccactggg 36436
chimp54546-135457.revcompl.Pan.troglodytes.clone.rp43-41g5.GenBank:AC099533.36      GGGGCACAGCCTGGGGTGGCGGCTGGCGTCAAACCCTCAAATCCCCTGAGAGCCACTGGG 38174
human.FAM30B-LOC105372935-GGT2.NCBI.                                                ggggcacagcctggggtggcagctggcgtcaaaccctcaaatcccctgagagccactggg 35104
human.FAM230E-LOC105377182-GGT3P.NCBI.                                              ggggcacagcctggggtggcagctgacgtcaaaccctcaaatcccctgagagccactggg 33851
human.FAM230J-LOC105372942-GGTLC5P.NCBI.rev.compl                                   ggggcacagcctggggtggcagctgacgtcaaaccctcaaatcccctgagagccactggg 34570
human.FAM230A.ncRNA.18487127.18500594-GGTLC3.18516335.18518165.rev.compl.REF        ggggcacagcctggggtggcagctggcgtcaaaccctcaaatcccctgagagccactggg 23318
                                                                                    ******************** **** **********************************

chimp.LOC112206744-LOC107973052-GGT2.revcompl                                       gagactaagcagtccccagcccccacttgtccctgagctgccattctcagccctgtggga 36496
chimp54546-135457.revcompl.Pan.troglodytes.clone.rp43-41g5.GenBank:AC099533.36      GAGACTAAGCAGTCTCCAGCCCCCACTTGTCCCTGAGCTGCCATTCTCAGCCCTGTGGGA 38234
human.FAM30B-LOC105372935-GGT2.NCBI.                                                gagactaagcagtccccagcccctacttgtccctgagctgccattctcagccctgtggga 35164
human.FAM230E-LOC105377182-GGT3P.NCBI.                                              gagactaagcagtccccagcccccacttgtccctgagctgccattctcagccctgtggga 33911
human.FAM230J-LOC105372942-GGTLC5P.NCBI.rev.compl                                   gagactaagcagtccccagcccccacttgtccctgagctgccattctcagccctgtggga 34630
human.FAM230A.ncRNA.18487127.18500594-GGTLC3.18516335.18518165.rev.compl.REF        gagactaagcagtccccagcccccacttgtccctgagctgccattctcagccctgtggga 23378
                                                                                    ************** ******** ************************************

chimp.LOC112206744-LOC107973052-GGT2.revcompl                                       ggagacagaaagccctgaagagaaaccaaaggaccaggtcaggaggggct--gggggggt 36554
chimp54546-135457.revcompl.Pan.troglodytes.clone.rp43-41g5.GenBank:AC099533.36      GGAGACAGAAAGCCCTGAAGAGAAACCAAAGGACCAGGTCAGGAGGGGCT--GGGGGGGT 38292
human.FAM30B-LOC105372935-GGT2.NCBI.                                                ggagacagaaagccctgaagagaaaccaaaggaccaggtcaggaggggctgggggggggt 35224
human.FAM230E-LOC105377182-GGT3P.NCBI.                                              ggagacagaaagccctgaagagaaaccaaaggaccaggtcaggaggggct--gggggggt 33969
human.FAM230J-LOC105372942-GGTLC5P.NCBI.rev.compl                                   ggagacagaaagccctgaagagaaaccaaaggaccaggtcaggaggggct--gggggggt 34688
human.FAM230A.ncRNA.18487127.18500594-GGTLC3.18516335.18518165.rev.compl.REF        ggagacagaaagccctgaagagaaaccaaaggaccaggtcaggaggggct--gggggggt 23436
                                                                                    **************************************************  ********

chimp.LOC112206744-LOC107973052-GGT2.revcompl                                       ggcatgagcaatcagggcagggaaggatggacagatgggggaatggagggaagaaggaat 36614
chimp54546-135457.revcompl.Pan.troglodytes.clone.rp43-41g5.GenBank:AC099533.36      GGCATGAGCAATCAGGGCAGGGAAGGATGGACAGATGGGGGAATGGAGGGAAGAAGGAAT 38352
human.FAM30B-LOC105372935-GGT2.NCBI.                                                ggcatgagcaatcagggcagggaaggatgcacagatgggggaatggagggaagaaggaat 35284
human.FAM230E-LOC105377182-GGT3P.NCBI.                                              ggcatgagcaatcagggcagggaaggatggacagatgggggaatggagggaagaaggaat 34029
human.FAM230J-LOC105372942-GGTLC5P.NCBI.rev.compl                                   ggcatgagcaatcagggcagggaaggatggacagatgggggaatggagggaagaaggaat 34748
human.FAM230A.ncRNA.18487127.18500594-GGTLC3.18516335.18518165.rev.compl.REF        ggcatgagcaatcagggcagggaaggatggaccgatgggggaatggagggaagaaggaat 23496
                                                                                    ***************************** ** ***************************

chimp.LOC112206744-LOC107973052-GGT2.revcompl                                       gaatgaaacggtgaatgaatgaacaaagagagagaacggccattcctcccttgctttagt 36674
chimp54546-135457.revcompl.Pan.troglodytes.clone.rp43-41g5.GenBank:AC099533.36      GAATGAAAAGTTGAATGAATGAACAAAGAGAGAGAATGGCCATTCCTCCCTTGCTTTAGT 38412
human.FAM30B-LOC105372935-GGT2.NCBI.                                                gaatgaaaaggtgaatgaatgaacaaagagagagaacggccactcctcccttgctttagt 35344
human.FAM230E-LOC105377182-GGT3P.NCBI.                                              gaatgaaaaggtgaatgaatgaacaaagagagagaacggccactcctcccttgctttagt 34089
human.FAM230J-LOC105372942-GGTLC5P.NCBI.rev.compl                                   gaatgaaaaggtgaatgaatgaacaaagagagagaacggccactcctcccttgctttagt 34808
human.FAM230A.ncRNA.18487127.18500594-GGTLC3.18516335.18518165.rev.compl.REF        gaatgaaaaggtgaatgaatgaacaaagagagagaacggccactcctcccttgctttagt 23556
                                                                                    ******** * ************************* ***** *****************

chimp.LOC112206744-LOC107973052-GGT2.revcompl                                       ttacaaagtactgggatcctcccaacagcctgcaagacagaatttctgggaagcagacca 36734
chimp54546-135457.revcompl.Pan.troglodytes.clone.rp43-41g5.GenBank:AC099533.36      TTACAAAGTACTGGGATCCTCCCAACAGCCTGCAAGACAGAATTTCTGGGAAGCAGACCA 38472
human.FAM30B-LOC105372935-GGT2.NCBI.                                                ttacaaagtactgggatcctcccaacagcctgcaagacagaatttctgggaagcagacca 35404
human.FAM230E-LOC105377182-GGT3P.NCBI.                                              ttacaaagtactgggatcctcccaacagcctgcaagacagaatttctgggaagcagacca 34149
human.FAM230J-LOC105372942-GGTLC5P.NCBI.rev.compl                                   ttacaaagtactgggatcctcccaacagcctgcaagacagaatttctgggaagcagacca 34868
human.FAM230A.ncRNA.18487127.18500594-GGTLC3.18516335.18518165.rev.compl.REF        ttacaaagtactgggatcctcccaacagcctgcaagacagaatttctgggaagcagacca 23616
                                                                                    ************************************************************

chimp.LOC112206744-LOC107973052-GGT2.revcompl                                       ggtggctggcagggaggggaggcttgccctggcttttgtgggcccaatgggaggcagggg 36794
chimp54546-135457.revcompl.Pan.troglodytes.clone.rp43-41g5.GenBank:AC099533.36      GGTGGCTGGCAGGGAGGGGAGGCTTGCCCTGGCTTTTGTGGGCCCAATGGGAGGCAGGGG 38532
human.FAM30B-LOC105372935-GGT2.NCBI.                                                ggtggctggcagggaggggaggcttgccctggcttttgtgggcccaatgggaggcagggg 35464
human.FAM230E-LOC105377182-GGT3P.NCBI.                                              ggtggctggcagggaggggaggcttgccctggcttttgtgggcccaatgggaggcagggg 34209
human.FAM230J-LOC105372942-GGTLC5P.NCBI.rev.compl                                   ggtggctggcagggaggggaggcttgccctggcttttgtgggcccaatgggaggcagggg 34928
human.FAM230A.ncRNA.18487127.18500594-GGTLC3.18516335.18518165.rev.compl.REF        ggtggctggcagggaggggaggcttgccctggcttttgtgggcccaatgggaggcagggg 23676
                                                                                    ************************************************************

chimp.LOC112206744-LOC107973052-GGT2.revcompl                                       gcaagaaggggcatcctgtgtgtgtcctccctgcagcggcagcagcaccttcctggaaga 36854
chimp54546-135457.revcompl.Pan.troglodytes.clone.rp43-41g5.GenBank:AC099533.36      GCAAGAAGGGGCATCCTGTGTGTGTCCTCCCTGCAGCGGCAGCAGCACCTTCCTGGAAGA 38592
human.FAM30B-LOC105372935-GGT2.NCBI.                                                gcaagaaggggcatcctgtgtgtgtcctccctgcagcggcagcagcaccttcctggaaga 35524
human.FAM230E-LOC105377182-GGT3P.NCBI.                                              gcaagaaggggcatcctgtgtgtgtcctccctgcagcggcagcagcaccttcctggaaga 34269
human.FAM230J-LOC105372942-GGTLC5P.NCBI.rev.compl                                   gcaagaaggggcatcctgtgtgtgtcctccctgcagcggcagcagcaccttcctggaaga 34988
human.FAM230A.ncRNA.18487127.18500594-GGTLC3.18516335.18518165.rev.compl.REF        gcaagaaggggcatcctgtgtgtgtcctccctgcagtggcagcagcaccttcctggaaga 23736
                                                                                    ************************************ ***********************

chimp.LOC112206744-LOC107973052-GGT2.revcompl                                       gggtcaggaaacactcactgtggcccctctccatcacgacctcatccaggacaccaaata 36914
chimp54546-135457.revcompl.Pan.troglodytes.clone.rp43-41g5.GenBank:AC099533.36      GGGTCAGGAAACACCCACTGTGGCCCCTCTCCATCACGCCCTCATCCAGGACACCAAGTA 38652
human.FAM30B-LOC105372935-GGT2.NCBI.                                                gggtcaggaaacacccgctgtggcccctctccaccacgccctcatccaggacaccaagta 35584
human.FAM230E-LOC105377182-GGT3P.NCBI.                                              gggtcaggaaacacccgctgtggcccctctccaccacgccctcatccaggacaccaagta 34329
human.FAM230J-LOC105372942-GGTLC5P.NCBI.rev.compl                                   gggtcaggaaacacccgctgtggcccctctccaccacgccctcatccaggacaccaagta 35048
human.FAM230A.ncRNA.18487127.18500594-GGTLC3.18516335.18518165.rev.compl.REF        gggtcaggaaacacccgctgtggcccctctccaccacgccctcatccaggacaccaagta 23796
```

```
                                                                                ************** * **************** **** ****************** **

chimp.LOC112206744-LOC107973052-GGT2.revcompl                                   tcaagtcactcagctcacgagacccaggccctgactcagggagagaggatgtgaggggtg 36974
chimp54546-135457.revcompl.Pan.troglodytes.clone.rp43-41g5.GenBank:AC099533.36  TC-AGTCACTCGGCTCACGAGATCCAGGCCCTGACTCAGGGAGAGAGGATGTGAGGGGTG 38711
human.FAM30B-LOC105372935-GGT2.NCBI.                                            tc-agtcactcagctcacgagacccaggccctgactcagggagagaggatgtgaggggtg 35643
human.FAM230E-LOC105377182-GGT3P.NCBI.                                          tc-agtcactcagctcacgagacccaggccctgactcagggagagaggatgtgaggggtg 34388
human.FAM230J-LOC105372942-GGTLC5P.NCBI.rev.compl                               tc-agtcactcagctcacgagacccaggccctgactcagggagagaggatgtgaggggtg 35107
human.FAM230A.ncRNA.18487127.18500594-GGTLC3.18516335.18518165.rev.compl.REF    tc-agtcactcagctcacgagacccaggccctgactcagggagagaggatgtgaggggtg 23855
                                                                                ** ******** ********** *************************************

chimp.LOC112206744-LOC107973052-GGT2.revcompl                                   gggcaccgggctcctcaggactgagagacctgagatgtggccccgggctgggtgttgggg 37034
chimp54546-135457.revcompl.Pan.troglodytes.clone.rp43-41g5.GenBank:AC099533.36  GGGCACCGGGCTCCTCAGGACTGAGAGACCTGAGATGTGGCCCCGGGCTGGGTGTTGGGG 38771
human.FAM30B-LOC105372935-GGT2.NCBI.                                            gggcaccgggctcctcaggactgagagacctgagatgtggccccgggctgggtgtagggg 35703
human.FAM230E-LOC105377182-GGT3P.NCBI.                                          gggcaccgggctcctcaggactgagagacctgagatgtggccccgggctgggtgtagggg 34448
human.FAM230J-LOC105372942-GGTLC5P.NCBI.rev.compl                               gggcaccgggctcctcaggactgagagacctgagatgtggccccgggctgggtgtagggg 35167
human.FAM230A.ncRNA.18487127.18500594-GGTLC3.18516335.18518165.rev.compl.REF    gggcaccgggctcctcaggactgagagacctgggatgtggccccgggctgggtgtagggg 23915
                                                                                ******************************** ********************** ****

chimp.LOC112206744-LOC107973052-GGT2.revcompl                                   cagactggctatggcagcattgtgtgtaccccagcaggccagtacccatgcagggagcct 37094
chimp54546-135457.revcompl.Pan.troglodytes.clone.rp43-41g5.GenBank:AC099533.36  CAGACTGGCTATGGCAGCATTGTGTGTACCCCAGCAGGCCAGTACCCACGCAGGGAGCCT 38831
human.FAM30B-LOC105372935-GGT2.NCBI.                                            cagactggctatggcagcattgtgtgtaccccagcaggccagtacccacgcagggagcct 35763
human.FAM230E-LOC105377182-GGT3P.NCBI.                                          cagactggctatggcagcattgtgtgtaccccagcaggccagtacccacgcagggagcct 34508
human.FAM230J-LOC105372942-GGTLC5P.NCBI.rev.compl                               cagactggctatggcagcattgtgtgtaccccagcaggccagtacccacgcagggagcct 35227
human.FAM230A.ncRNA.18487127.18500594-GGTLC3.18516335.18518165.rev.compl.REF    cagactggctatggcagcattgtgtgtaccccagcaggccagtacccacgcagggagcct 23975
                                                                                ************************************************ ***********

chimp.LOC112206744-LOC107973052-GGT2.revcompl                                   ccaaaccccttcacccatgaccctgggagaagaccgcaggcttggagaattggcctcact 37154
chimp54546-135457.revcompl.Pan.troglodytes.clone.rp43-41g5.GenBank:AC099533.36  CCAAACCCCTTCACCCATGACCCTGGGAGAAGACCCCAGCCTTGGAGAATT-GCCTCACT 38890
human.FAM30B-LOC105372935-GGT2.NCBI.                                            ccaaaccccttcacccatgaccctgggagaagaccgcagccttggagaattggcctcact 35823
human.FAM230E-LOC105377182-GGT3P.NCBI.                                          ccaaaccccttcacccatgaccctgggagaagaccgcagccttggagaattggcctcact 34568
human.FAM230J-LOC105372942-GGTLC5P.NCBI.rev.compl                               ccaaaccccttcacccatgaccctgggagaagaccgcagccttggagaattggcctcact 35287
human.FAM230A.ncRNA.18487127.18500594-GGTLC3.18516335.18518165.rev.compl.REF    ccaaaccccttcacccatgaccctgggagaagaccgcagccttggagaattggcctcact 24035
                                                                                *********************************** *** *********** ********

chimp.LOC112206744-LOC107973052-GGT2.revcompl                                   gaagggccctgcaccggccagcagggtcaggcggggccagacaggttcccacctgggata 37214
chimp54546-135457.revcompl.Pan.troglodytes.clone.rp43-41g5.GenBank:AC099533.36  GAAGGGACCTGCACTGGCCAGCAGGGTCAGGCGGGGCCAGACAGGTTCCCACCTGGGATA 38950
human.FAM30B-LOC105372935-GGT2.NCBI.                                            gaaggggcctgcgccggccagcagggtcaggcggggccagacaggttcccacctgggata 35883
human.FAM230E-LOC105377182-GGT3P.NCBI.                                          gaaggggcctgcaccggccagcagggtcaggcggggccagacaggttcccacctgggata 34628
human.FAM230J-LOC105372942-GGTLC5P.NCBI.rev.compl                               gaaggggcctgcaccggccagcagggtcaggcggggccagacaggttcccacctgggata 35347
human.FAM230A.ncRNA.18487127.18500594-GGTLC3.18516335.18518165.rev.compl.REF    gaaggggcctgcaccggccagcagggtcaggcggggccagacaggttcccacctgggata 24095
                                                                                ****** ***** * *********************************************

chimp.LOC112206744-LOC107973052-GGT2.revcompl                                   tgcaaatgggcctcctgaatcctggagccaggtatggactcacacaccaccattgtcccc 37274
chimp54546-135457.revcompl.Pan.troglodytes.clone.rp43-41g5.GenBank:AC099533.36  TGCAAATGGGCCTCCTGAATCCTGGAGCCAGGTATGGACTCACACACCACCATTGTCCCC 39010
human.FAM30B-LOC105372935-GGT2.NCBI.                                            tgcaaatgggcctcctgaatcctggagccaggtatggactcacacaccaccattgtcccc 35943
human.FAM230E-LOC105377182-GGT3P.NCBI.                                          tgcaaatgggcctcctgaatcctggagccaggtatggactcacacaccaccattgtcccc 34688
human.FAM230J-LOC105372942-GGTLC5P.NCBI.rev.compl                               tgcaaatgggcctcctgaatcctggagccaggtatggactcacacaccaccattgtcccc 35407
human.FAM230A.ncRNA.18487127.18500594-GGTLC3.18516335.18518165.rev.compl.REF    tgcaaatgggcctcctgaatcctggagccaggtatggactcacacaccaccattgtcccc 24155
                                                                                ************************************************************

chimp.LOC112206744-LOC107973052-GGT2.revcompl                                   aagtccccatctgccccacgggcacaccctgccacctgttctgtgcaagagccctgaggc 37334
chimp54546-135457.revcompl.Pan.troglodytes.clone.rp43-41g5.GenBank:AC099533.36  AAGTCCCCATCTGCCCCACGGGCACACCCTGCCACCTGTTCTGTGCAAAGGCCCTGAGGC 39070
human.FAM30B-LOC105372935-GGT2.NCBI.                                            aagtccccatctgccccacgggcacaccctgccgcctgttctgtgcaagggccctgaggc 36003
human.FAM230E-LOC105377182-GGT3P.NCBI.                                          aagtccccatctgccccacgggcacaccctgccgcctgttctgtgcaagggccctgaggc 34748
human.FAM230J-LOC105372942-GGTLC5P.NCBI.rev.compl                               aagtccccatctgccccacgggcacaccctgccgcctgttctgtgcaagggccctgaggc 35467
human.FAM230A.ncRNA.18487127.18500594-GGTLC3.18516335.18518165.rev.compl.REF    aagtccccatctgccccacgggcacaccctgccgcctgttctgtgcaagggccctgaggc 24215
                                                                                ********************************* **************  **********

chimp.LOC112206744-LOC107973052-GGT2.revcompl                                   tgtctccttgcgctcaagccctgcaggtgctgaagcccacacacacagctcctgcttcct 37394
chimp54546-135457.revcompl.Pan.troglodytes.clone.rp43-41g5.GenBank:AC099533.36  TGTCTCCTTGCGCTCAAGCCCTGCAGGTGCTGAAGCCCACACACACGGCTCCTGCTTCCT 39130
human.FAM30B-LOC105372935-GGT2.NCBI.                                            tgtctccttgcgctcaagccctgcaggtgctgaagcccacacacacggctcctgcttcct 36063
human.FAM230E-LOC105377182-GGT3P.NCBI.                                          tgtctccttgcgctcaagccctgcaggtgctgaagcccacacacacggctcctgcttact 34808
human.FAM230J-LOC105372942-GGTLC5P.NCBI.rev.compl                               tgtctccttgcgctcaagccctgcaggtgctgaagcccacacacacggctcctgcttcct 35527
human.FAM230A.ncRNA.18487127.18500594-GGTLC3.18516335.18518165.rev.compl.REF    tgtctccttgcgcccaagacctgcaggtgctgaagcccacacacacggctcctgcttcct 24275
                                                                                ************* **** *************************** ********** **

chimp.LOC112206744-LOC107973052-GGT2.revcompl                                   gggccagtgcacgtgcacacacacacacgtgcacacacacaccc------acaaatatac 37448
chimp54546-135457.revcompl.Pan.troglodytes.clone.rp43-41g5.GenBank:AC099533.36  GGGCCAGTGCACGTGCACACACACACACATGCACACACACACCCACACATACAC--ATAC 39188
human.FAM30B-LOC105372935-GGT2.NCBI.                                            gggccagtgcacgtgcacacacacacacgtgcacacacacacacacccccacaaatatac 36123
human.FAM230E-LOC105377182-GGT3P.NCBI.                                          gggccagtgcacgtgcacacacacacacgtg--cacacacacacacacccacaaatatac 34866
human.FAM230J-LOC105372942-GGTLC5P.NCBI.rev.compl                               gggccagtgcacgtgcacacacacacacgtg--cacacacacacacacccacaaatatac 35585
human.FAM230A.ncRNA.18487127.18500594-GGTLC3.18516335.18518165.rev.compl.REF    gggccagtgcacgtgcacacacacacacgtgcacacacacacacacacccacaaatatac 24335
                                                                                **************************** **  ********* *      ***   ****

chimp.LOC112206744-LOC107973052-GGT2.revcompl                                   ccacacacaatcacacacattcacacatacccaccccca-------tacactcacacatt 37501
chimp54546-135457.revcompl.Pan.troglodytes.clone.rp43-41g5.GenBank:AC099533.36  CCACACACAATCACACACATTCACACATACCCACACCCCCCATACTCACACTCACACACT 39248
human.FAM30B-LOC105372935-GGT2.NCBI.                                            ccacacacaatcacacacattcacacatacccac-ccccccatactcacactcacacatt 36182
human.FAM230E-LOC105377182-GGT3P.NCBI.                                          ccacacacaatcacacacattcacacataccca--ccccccatactcacactcacacatt 34924
human.FAM230J-LOC105372942-GGTLC5P.NCBI.rev.compl                               ccacacacaatcacacacattcacacataccca--ccccccatactcacactcacacatt 35643
human.FAM230A.ncRNA.18487127.18500594-GGTLC3.18516335.18518165.rev.compl.REF    ccacacacaatcacacacattcacacataccca--ccccccatactcaca--cacacatt 24391
                                                                                *********************************  ***         ***  ****** *

chimp.LOC112206744-LOC107973052-GGT2.revcompl                                   cacacccac------acccacacatacacacacacaatcacacacatttacacacaccca 37555
chimp54546-135457.revcompl.Pan.troglodytes.clone.rp43-41g5.GenBank:AC099533.36  CACACACACCCACAAACACCCACACATACACACACAATCACACACATTTACACACACCCA 39308
human.FAM30B-LOC105372935-GGT2.NCBI.                                            tacacacacc----------------------------------------cacacaccca 36202
human.FAM230E-LOC105377182-GGT3P.NCBI.                                          tacacacacc----------------------------------------cacacaccca 34944
human.FAM230J-LOC105372942-GGTLC5P.NCBI.rev.compl                               tacacacacc----------------------------------------cacacaccca 35663
human.FAM230A.ncRNA.18487127.18500594-GGTLC3.18516335.18518165.rev.compl.REF    tacacacacc----------------------------------------cacacaccca 24411
                                                                                 **** ***                                         **********

chimp.LOC112206744-LOC107973052-GGT2.revcompl                                   cacacccacactcacacactcagtcacacacaccctcacacagcgaaacacaat--caca 37613
chimp54546-135457.revcompl.Pan.troglodytes.clone.rp43-41g5.GenBank:AC099533.36  CACTCACACACTCA------CAGTCACACA-ACCCTCACACAGCGAAACGCAATCACACA 39361
human.FAM30B-LOC105372935-GGT2.NCBI.                                            cactcacacactca------cagtcacacacaccctcacacagcgaaacacaatcacaca 36256
human.FAM230E-LOC105377182-GGT3P.NCBI.                                          cactcacacactca------cagtcacacacaccctcacacagcgaaacacaatcacaca 34998
human.FAM230J-LOC105372942-GGTLC5P.NCBI.rev.compl                               cactcacacactca------cagtcacacacaccctcacacagcgaaacacaatcacaca 35717
human.FAM230A.ncRNA.18487127.18500594-GGTLC3.18516335.18518165.rev.compl.REF    cactcacacactca------cagtcacacacaccctcacacagcgaaacacaatcacaca 24465
                                                                                *** * ********      ********** ****************** ****  ****

chimp.LOC112206744-LOC107973052-GGT2.revcompl                                   cattcacacccacccacacccccacacactcacactcacatatact--------cacaca 37665
chimp54546-135457.revcompl.Pan.troglodytes.clone.rp43-41g5.GenBank:AC099533.36  CATTCACACCCACCCACACCCCCACACACTCACACTCACATATACTCACACACCCACACA 39421
human.FAM30B-LOC105372935-GGT2.NCBI.                                            cattcacacccacccacacccccacacactcacactcacatatact--------cacaca 36308
human.FAM230E-LOC105377182-GGT3P.NCBI.                                          cattcacacccacccacacccccacacactcacactcacatatact--------cacaca 35050
human.FAM230J-LOC105372942-GGTLC5P.NCBI.rev.compl                               cattcacacccacccacacccccacacactcacactcacatatact--------cacaca 35769
human.FAM230A.ncRNA.18487127.18500594-GGTLC3.18516335.18518165.rev.compl.REF    cattcacacccacccaca-ccccacacactcacactcacatatact--------cacaca 24516
                                                                                ****************** ***************************        ******

chimp.LOC112206744-LOC107973052-GGT2.revcompl                                   cacccacacacacatacacaaacacaatcacacacattcacacaca-------------- 37711
chimp54546-135457.revcompl.Pan.troglodytes.clone.rp43-41g5.GenBank:AC099533.36  CACCCACACACACATACACAAACACAATCACACACATTCACACACACCCATACTCACACA 39481
human.FAM30B-LOC105372935-GGT2.NCBI.                                            cacccacacacacatacacaaacacaatcacacacattcacacacacccacactcacaca 36368
human.FAM230E-LOC105377182-GGT3P.NCBI.                                          cacccacacacacatacacaaacacaatcacacacattcacacacacccacactcacaca 35110
human.FAM230J-LOC105372942-GGTLC5P.NCBI.rev.compl                               cacccacacacacatacacaaacacaatcacacacattcacacacacccacactcacaca 35829
human.FAM230A.ncRNA.18487127.18500594-GGTLC3.18516335.18518165.rev.compl.REF    cacccacacacacatacacaaacacaatcacacacattcacacacacccacactcacaca 24576
                                                                                **********************************************

chimp.LOC112206744-LOC107973052-GGT2.revcompl                                   ----------------------------------------caccccccacaaacacactc 37731
chimp54546-135457.revcompl.Pan.troglodytes.clone.rp43-41g5.GenBank:AC099533.36  TACACGC------AAACGATCACACACATTCACACACACCCACACCCCACAAACACACTC 39535
human.FAM30B-LOC105372935-GGT2.NCBI.                                            tacacacacccaaacacgatcacacacattcacacacacccacaccccacaaacacactc 36428
human.FAM230E-LOC105377182-GGT3P.NCBI.                                          tacacacacccaaacacgatcacacacattcacacacacccacaccccacaaacacactc 35170
human.FAM230J-LOC105372942-GGTLC5P.NCBI.rev.compl                               tacacacacccaaacacgatcacacacattcacacacacccacaccccacaaacacactc 35889
human.FAM230A.ncRNA.18487127.18500594-GGTLC3.18516335.18518165.rev.compl.REF    tacacacacccaaacacgatcacacacattcacacacacccacaccccacaaacacactc 24636
                                                                                                                        *** ****************

chimp.LOC112206744-LOC107973052-GGT2.revcompl                                   acacatatacccacacacactcacactcacacataatctcacacacacacacacatgctc 37791
chimp54546-135457.revcompl.Pan.troglodytes.clone.rp43-41g5.GenBank:AC099533.36  ACACATATACCCACACACACTCACACTCATACATAATCTCACACACACACACATATGCTC 39595
human.FAM30B-LOC105372935-GGT2.NCBI.                                            acacatatacccacacacactcac------acataatctctcacacacacac--atgctc 36480
human.FAM230E-LOC105377182-GGT3P.NCBI.                                          acacatatacccacacacactcac------acataatctctcacacacacac--atgctc 35222
human.FAM230J-LOC105372942-GGTLC5P.NCBI.rev.compl                               acacatatacccacacacactcac------acataatctctcacacacacac--atgctc 35941
human.FAM230A.ncRNA.18487127.18500594-GGTLC3.18516335.18518165.rev.compl.REF    acacatatacccacacacactgac------acataatctctcacacacacac--atgctc 24688
                                                                                ********************* **      ********** ***********  ******

chimp.LOC112206744-LOC107973052-GGT2.revcompl                                   acacacacacgccttctccaggaggggctggctgccaaaggccacccagcttcctcccac 37851
chimp54546-135457.revcompl.Pan.troglodytes.clone.rp43-41g5.GenBank:AC099533.36  ACACACACACGCCTTCTCCAGGAGGGGCTGGCTGCCAAAGGCCCCCAGCTTCCTCCCAC 39655
human.FAM30B-LOC105372935-GGT2.NCBI.                                            acacacacacgccttctccaggaggggctggctgccaaaggccacccagcttcctcccac 36540
human.FAM230E-LOC105377182-GGT3P.NCBI.                                          acacacacacgccttctccaggaggggctggctgccaaaggccacccagcttcctcccac 35282
human.FAM230J-LOC105372942-GGTLC5P.NCBI.rev.compl                               acacacacacgccttctccaggaggggctggctgccaaaggccacccagcttcctcccac 36001
human.FAM230A.ncRNA.18487127.18500594-GGTLC3.18516335.18518165.rev.compl.REF    acacacacacgccttctccaggaggggctggctgccaaaggccacccagcttcctcccac 24748
                                                                                ******************************************* ****************

chimp.LOC112206744-LOC107973052-GGT2.revcompl                                   gtctcactcaccgtacaatatttgagcagaccttggagtcagcagcaacagcagcgtggg 37911
chimp54546-135457.revcompl.Pan.troglodytes.clone.rp43-41g5.GenBank:AC099533.36  GTCTGACTCACCGTACAATATTTGAGCAGACCTTGGAGTCAGCAGCAACAGCAGCGTGGG 39715
human.FAM30B-LOC105372935-GGT2.NCBI.                                            gtctcactcaccgtacaatatttgagaagaccttggagtcagcagcaacagcggcgtggg 36600
human.FAM230E-LOC105377182-GGT3P.NCBI.                                          gtctcactcaccgtacaatatttgagaagaccttggagtcagcagcaacagcggtgtggg 35342
human.FAM230J-LOC105372942-GGTLC5P.NCBI.rev.compl                               gtctcactcaccgtacaatatttgagaagaccttggagtcagcagcaacagcggtgtggg 36061
human.FAM230A.ncRNA.18487127.18500594-GGTLC3.18516335.18518165.rev.compl.REF    gtctcactcaccgtacaatatttgagaagaccttggagtcagcagcaacagcggcgtggg 24808
                                                                                **** ******************** ************************** * *****
```

```
chimp.LOC112206744-LOC107973052-GGT2.revcompl                               caaaggcctgggagccacatggggcctggtgtcgagagaggaccacagccagcacaatga 37971
chimp54546-135457.revcompl.Pan.troglodytes.clone.rp43-41g5.GenBank:AC099533.36   CAAAGGCCTGGGAGCCACATGGGGCCTGGTGTCGAGAGAGGACCACAGCCAGCACAATGA 39775
human.FAM30B-LOC105372935-GGT2.NCBI.                                        caaaggcc-gggggtcaaatggggcctggtgtcgagagaggaccacagccagcacaatga 36659
human.FAM230E-LOC105377182-GGT3P.NCBI.                                      caaaggcc-gggggtcaaatggggcctggtgtcgagagaggaccacagccagcacaatga 35401
human.FAM230J-LOC105372942-GGTLC5P.NCBI.rev.compl                           caaaggcc-gggggtcaaatggggcctggtgtcgagagaggaccacagccagcacaatga 36120
human.FAM230A.ncRNA.18487127.18500594-GGTLC3.18516335.18518165.rev.compl.REF caaaggcc-gggggtcaaatggggcctggtgtcgagagaggaccacagccagcacaatga 24867
                                                                            ******** *** * ** ******************************************

chimp.LOC112206744-LOC107973052-GGT2.revcompl                               cagccagcgccagccccagccccagccccagcaggaccaggtcacccatggctctgcagt 38031
chimp54546-135457.revcompl.Pan.troglodytes.clone.rp43-41g5.GenBank:AC099533.36   CAGCCAGCGCCAGCCCCAGCCCCAGCCCCAGCAGGACCAGGTCACCCATGGCTCCGCAGT 39835
human.FAM30B-LOC105372935-GGT2.NCBI.                                        cagccagcgccagccccagccccagccccagcaggaccaggtcacccatggctccgtagt 36719
human.FAM230E-LOC105377182-GGT3P.NCBI.                                      cagccagcgccagccccagccccagccccagcaggaccaggtcacccatggctccgtagt 35461
human.FAM230J-LOC105372942-GGTLC5P.NCBI.rev.compl                           cagccagcgccagccccagccccagccccagcaggaccaggtcacccatggctccgtagt 36180
human.FAM230A.ncRNA.18487127.18500594-GGTLC3.18516335.18518165.rev.compl.REF cagccagcgccagccccagccccagccccagcaggaccaggtcacccatggctccgtagt 24927
                                                                            ****************************************************** * ***

chimp.LOC112206744-LOC107973052-GGT2.revcompl                               cctgggccatggctctgcggcccagaaggagaggggaggccggtgggcagacggagggac 38091
chimp54546-135457.revcompl.Pan.troglodytes.clone.rp43-41g5.GenBank:AC099533.36   CCTGGGCCATGGCTCTGCGGCCCAGAAGGAGAGGGGAGGCCGGTGGGCAGACGGAGGGAC 39895
human.FAM30B-LOC105372935-GGT2.NCBI.                                        cctgggccatggctctgcggcccagaaggagaggggaggccggtgggcagatggagggac 36779
human.FAM230E-LOC105377182-GGT3P.NCBI.                                      cctgggccatggctctgcggcccagaaggagaggggaggccggtgggcagatggagggac 35521
human.FAM230J-LOC105372942-GGTLC5P.NCBI.rev.compl                           cctgggccatggctctgcggcccagaaggagaggggaggccggtgggcagatggagggac 36240
human.FAM230A.ncRNA.18487127.18500594-GGTLC3.18516335.18518165.rev.compl.REF cctgggccatggctctgcggcccagaaggagaggggaggccggtgggcagatggagggac 24987
                                                                            *************************************************** ********

chimp.LOC112206744-LOC107973052-GGT2.revcompl                               agatgggtgggcagatgaatggacaagaagatgcatagatagactcacaggtaattggac 38151
chimp54546-135457.revcompl.Pan.troglodytes.clone.rp43-41g5.GenBank:AC099533.36   AGATGGGTGGGCAGATGAATGGACAAGAAGATGCATAGATAGACTCACAGGTAATTGGAC 39955
human.FAM30B-LOC105372935-GGT2.NCBI.                                        agatgggtgggcagatgaatggacaagaagatgcatagatagactcacaggtaattggac 36839
human.FAM230E-LOC105377182-GGT3P.NCBI.                                      agatgggtgggcagatgaatggacaagaagatgcatagatagactcacaggtaattggac 35581
human.FAM230J-LOC105372942-GGTLC5P.NCBI.rev.compl                           agatgggtgggcagatgaatggacaagaagatgcatagatagactcacaggtaattggac 36300
human.FAM230A.ncRNA.18487127.18500594-GGTLC3.18516335.18518165.rev.compl.REF agatgggtgggcagatgaatggacaagaagatgcatagatagactcacaggtaattggac 25047
                                                                            ************************************************************

chimp.LOC112206744-LOC107973052-GGT2.revcompl                               agatggacaaacaggtgggggctgaagacagacacgaagatggatcgacagacaggccag 38211
chimp54546-135457.revcompl.Pan.troglodytes.clone.rp43-41g5.GenBank:AC099533.36   AGATGGACAAACAGGTGGGGGCTGAAGACAGACACGAAGATGGATCGACAGATAGGCCAG 40015
human.FAM30B-LOC105372935-GGT2.NCBI.                                        agatggacaaacaggtgggggctgaagacagacacgaagatggatcgacagacaggccag 36899
human.FAM230E-LOC105377182-GGT3P.NCBI.                                      agatggacaaacaggtgggggctgaagacagacacgaagatggatcgacagacaggccag 35641
human.FAM230J-LOC105372942-GGTLC5P.NCBI.rev.compl                           agatggacaaacaggtgggggctgaagacagacacgaagatggatcgacagacaggccag 36360
human.FAM230A.ncRNA.18487127.18500594-GGTLC3.18516335.18518165.rev.compl.REF agatggacaaacaggtgggggctgaagacagacacgaagatggatcgacagacaggccag 25107
                                                                            **************************************************** *******

chimp.LOC112206744-LOC107973052-GGT2.revcompl                               atagctagacaaagaggacagtaagagaaagatggtcagatagacaatggaacagagatg 38271
chimp54546-135457.revcompl.Pan.troglodytes.clone.rp43-41g5.GenBank:AC099533.36   ATAGCTAGACAAAGAGGACAGTAAGAGAAAGATGGTCAGATAGACAATGGGACAGAGATG 40075
human.FAM30B-LOC105372935-GGT2.NCBI.                                        atagctagacaaagaggacagtaagagaaagatggtcagatagacaatgggacagagatg 36959
human.FAM230E-LOC105377182-GGT3P.NCBI.                                      atagctagacaaagaggacagtaagagaaagatggtcagatagacaatgggacagagatg 35701
human.FAM230J-LOC105372942-GGTLC5P.NCBI.rev.compl                           atagctagacaaagaggacagtaagagaaagatggtcagatagacaatgggacagagatg 36420
human.FAM230A.ncRNA.18487127.18500594-GGTLC3.18516335.18518165.rev.compl.REF atagctagacaaagaggacagtaagagaaagatggtcagatagacaatgggacagagatg 25167
                                                                            ************************************************** *********

chimp.LOC112206744-LOC107973052-GGT2.revcompl                               ggcttacagatgggcggacagacagacaggtctgaacagcgggctgccagatggacagat 38331
chimp54546-135457.revcompl.Pan.troglodytes.clone.rp43-41g5.GenBank:AC099533.36   GGCTTACAGATGGGCGGACAGACAGACAGGTCTGAACAGCGGGCTGCCAGATGGACAGAT 40135
human.FAM30B-LOC105372935-GGT2.NCBI.                                        ggcttacagatgggcggacagacagacaggtctgaacagcgggctgccagatggacagat 37019
human.FAM230E-LOC105377182-GGT3P.NCBI.                                      ggcttacagatgggcggacagacagacaggtctgaacagcgggctgccagatggacagat 35761
human.FAM230J-LOC105372942-GGTLC5P.NCBI.rev.compl                           ggcttacagatgggcggacagacagacaggtctgaacagcgggctgccagatggacagat 36480
human.FAM230A.ncRNA.18487127.18500594-GGTLC3.18516335.18518165.rev.compl.REF ggcttacagatgggcggacagacagacaggtctgaacagcgggctgccagatggacagat 25227
                                                                            ************************************************************

chimp.LOC112206744-LOC107973052-GGT2.revcompl                               gggtgaatggacagatggctggcagctgtggcgagctgctgccctcaccaagtgcacact 38391
chimp54546-135457.revcompl.Pan.troglodytes.clone.rp43-41g5.GenBank:AC099533.36   GGGTGAATGGACAGATGGCTGGCAGCTGTGGCGAGCTGCTGCCCTCACCAAGTGCACACT 40195
human.FAM30B-LOC105372935-GGT2.NCBI.                                        gggtgaatggacagatggctggcagctgtggcgagctgctgccctcaccaagtgcacact 37079
human.FAM230E-LOC105377182-GGT3P.NCBI.                                      gggtgaatggacagatggctggcagctgtggcgagctgctgccctcaccaagtgcacact 35821
human.FAM230J-LOC105372942-GGTLC5P.NCBI.rev.compl                           gggtgaatggacagatggctggcagctgtggcgagctgctgccctcaccaagtgcacact 36540
human.FAM230A.ncRNA.18487127.18500594-GGTLC3.18516335.18518165.rev.compl.REF gggtgaatggacagatggctggcagctgtggcgagctgctgccctcaccaagtgcacact 25287
                                                                            ************************************************************

chimp.LOC112206744-LOC107973052-GGT2.revcompl                               acggagtggccaaactcatgcctcaacttctagttttcagctcctgcttgttcctggcag 38451
chimp54546-135457.revcompl.Pan.troglodytes.clone.rp43-41g5.GenBank:AC099533.36   ACGGAGTGGCCAAACTCACGCCTCAACTTCTAGTTTTCAGCTCCTGCTTGTTCCTGGCAG 40255
human.FAM30B-LOC105372935-GGT2.NCBI.                                        acggagtggccaaactcatgcctcaacttctagttttcagctcctgcttgttcctggcag 37139
human.FAM230E-LOC105377182-GGT3P.NCBI.                                      acggagtggccaaactcatgcctcaacttctagttttcagctcctgcttgttcctggcag 35881
human.FAM230J-LOC105372942-GGTLC5P.NCBI.rev.compl                           acggagtggccaaactcatgcctcaacttctagttttcagctcctgcttgttcctggcag 36600
human.FAM230A.ncRNA.18487127.18500594-GGTLC3.18516335.18518165.rev.compl.REF acggagtggccaaactcatgcctcaacttctagttttcagctcctgcttgttcctggcag 25347
                                                                            ****************** *****************************************

chimp.LOC112206744-LOC107973052-GGT2.revcompl                               gaggccaggcagcaaagcgtctgaggggagttttctttgcctagagaagtcagctgctgt 38511
chimp54546-135457.revcompl.Pan.troglodytes.clone.rp43-41g5.GenBank:AC099533.36   GAGGCCAGGCAGCAAAGCATCTGAGGGGAGTTTTCTTTGCCTAG---AGTCAGCTGCTGT 40312
human.FAM30B-LOC105372935-GGT2.NCBI.                                        gaggccaggcagcaaagcgtctgaggggagttttctttgcctagagaagtcagctgctgt 37199
human.FAM230E-LOC105377182-GGT3P.NCBI.                                      gaggccaggcagcaaagcgtctgaggggagttttctttgcctagagaagtcagctgctgt 35941
human.FAM230J-LOC105372942-GGTLC5P.NCBI.rev.compl                           gaggccaggcagcaaagcgtctgaggggagttttctttgcctagagaagtcagctgctgt 36660
human.FAM230A.ncRNA.18487127.18500594-GGTLC3.18516335.18518165.rev.compl.REF gaggccaggcagcaaagcgtctgaggggagttttctttgcctagagaagtcagctgctgt 25407
                                                                            ****************** *************************   *************

chimp.LOC112206744-LOC107973052-GGT2.revcompl                               gttaactccctcactgctggtaggtccaaaggccccacctaccgcccgccagagcccatg 38571
chimp54546-135457.revcompl.Pan.troglodytes.clone.rp43-41g5.GenBank:AC099533.36   GTTAACTCCCTCACTGCTGGTAGGTCCAAAGGCCCCACCTACCGCCCGCCAGAGCCCATG 40372
human.FAM30B-LOC105372935-GGT2.NCBI.                                        gttaactccctcactgctggtaggtccaaaggccccacctactgcccgccagagcccatg 37259
human.FAM230E-LOC105377182-GGT3P.NCBI.                                      gttaactccctcactgctggtaggtccaaaggcccgacctaccgcccaccagagcccatg 36001
human.FAM230J-LOC105372942-GGTLC5P.NCBI.rev.compl                           gttaactccctcactgctggtaggtccaaaggcccgacctaccgcccaccagagcccatg 36720
human.FAM230A.ncRNA.18487127.18500594-GGTLC3.18516335.18518165.rev.compl.REF gttaactccctcactgctggtaggtccaaaggccccacctaccgcccgccagagcccatg 25467
                                                                            *********************************** ****** **** ************

chimp.LOC112206744-LOC107973052-GGT2.revcompl                               gtcacactgtcgcaatgtgcaggagaacttggtgcctgctgcactgcgattgccaggtag 38631
chimp54546-135457.revcompl.Pan.troglodytes.clone.rp43-41g5.GenBank:AC099533.36   GTCACACTGTCGCAATGTGCAGGAGAACTTGGTGCCTGCTGCACTGCGGTTGCCAGGTAG 40432
human.FAM30B-LOC105372935-GGT2.NCBI.                                        gtcacactgtcgcaatgtgcaggagaacttggtgcgtgctgcactgcggttgccaggtag 37319
human.FAM230E-LOC105377182-GGT3P.NCBI.                                      gtcacactgtcgcaatgtgcaggagaacttggtgcgtgctgcactgcggttgccaggtag 36061
human.FAM230J-LOC105372942-GGTLC5P.NCBI.rev.compl                           gtcacactgtcgcaatgtgcaggagaacttggtgcgtgctgcactgcggttgccaggtag 36780
human.FAM230A.ncRNA.18487127.18500594-GGTLC3.18516335.18518165.rev.compl.REF gtcacactgtcgcaatgtgcaggagaacttggtgcgtgctgcactgcggttgccaggtag 25527
                                                                            *********************************** ************ ***********

chimp.LOC112206744-LOC107973052-GGT2.revcompl                               gggcagggctccctggaacctccacaccattccccacgttctcagcagctctgggaaagc 38691
chimp54546-135457.revcompl.Pan.troglodytes.clone.rp43-41g5.GenBank:AC099533.36   GGGCAGGGCTCCCTGGAACCTCCACACCATTCCCCAGGTTCTCAGCAGCTCTGGGAAAGC 40492
human.FAM30B-LOC105372935-GGT2.NCBI.                                        gggcagggctccctggaacctccacaccattccccaggttctcagcagctctgggaaagc 37379
human.FAM230E-LOC105377182-GGT3P.NCBI.                                      gggcagggctccctggaacctccacaccattccccaggttctcagcagctctgggaaagc 36121
human.FAM230J-LOC105372942-GGTLC5P.NCBI.rev.compl                           gggcagggctccctggaacctccacaccattccccaggttctcagcagctctgggaaagc 36840
human.FAM230A.ncRNA.18487127.18500594-GGTLC3.18516335.18518165.rev.compl.REF gggcagggctccctggaacctccacaccattccccaggttctcagcagctctgggaaagc 25587
                                                                            ************************************ ***********************

chimp.LOC112206744-LOC107973052-GGT2.revcompl                               agagctggggccgcttaactctgtcctggatccggcaaggctgccccactcccagagtgg 38751
chimp54546-135457.revcompl.Pan.troglodytes.clone.rp43-41g5.GenBank:AC099533.36   AGAGCTGGGGCCGCTTAACTCTGTCCTGGATCCGGCAAGGCTGCCCCCCTCCCAGAGTGG 40552
human.FAM30B-LOC105372935-GGT2.NCBI.                                        agagctggggccgcttaactctgccctggatccggcaaggctgcccacctcccagagtgg 37439
human.FAM230E-LOC105377182-GGT3P.NCBI.                                      agagctggggccgcttaactctgccctggatccggcaaggctgcccccctcccagagtgg 36181
human.FAM230J-LOC105372942-GGTLC5P.NCBI.rev.compl                           agagctggggccgcttaactctgccctggatccggcaaggctgcccccctcccagagtgg 36900
human.FAM230A.ncRNA.18487127.18500594-GGTLC3.18516335.18518165.rev.compl.REF agagctggggccgcttaactctgccctggatccggcaaggctgcccccctcccagagtgg 25647
                                                                            *********************** **********************  ************

chimp.LOC112206744-LOC107973052-GGT2.revcompl                               agccctgctccccagctcccatctctatcccctaaccctctccgcatggcccagcctagt 38811
chimp54546-135457.revcompl.Pan.troglodytes.clone.rp43-41g5.GenBank:AC099533.36   AGCCCTGCTCCCCAGCTCCCATCTCTATCCCCTAACCCTCTCCGCATGGCCCAGCCTAGT 40612
human.FAM30B-LOC105372935-GGT2.NCBI.                                        agccctgctccccagctcccatctctatcccctaaccctctccgcatggcccagcctagt 37499
human.FAM230E-LOC105377182-GGT3P.NCBI.                                      agccctgctccccagctcccatctctatcccctaaccctctccgcatggcccagcctagt 36241
human.FAM230J-LOC105372942-GGTLC5P.NCBI.rev.compl                           agccctgctccccagctcccatctctatcccctaaccctctccgcatggcccagcctagt 36960
human.FAM230A.ncRNA.18487127.18500594-GGTLC3.18516335.18518165.rev.compl.REF agccctgctccccagctcccatctctatcccctaaccctctccgcatggcccagcctagt 25707
                                                                            ************************************************************

chimp.LOC112206744-LOC107973052-GGT2.revcompl                               cagcatcaaggtggagctgaacagaggcagaaggaggaggacccaaggtggtgtcactca 38871
chimp54546-135457.revcompl.Pan.troglodytes.clone.rp43-41g5.GenBank:AC099533.36   CAGCATCAAGGTGGAGCTGAACAGAGGCAGAAGGAGGAGGACCCAAGGTGGTGTCACTCA 40672
human.FAM30B-LOC105372935-GGT2.NCBI.                                        cagcatcaaggtggagctgaacagaggcagaaggaggaggacccaaggtggtgtcactca 37559
human.FAM230E-LOC105377182-GGT3P.NCBI.                                      cagcatcaaggtggagctgaacagaggcagaaggaggaggacccaaggtggtgtcactca 36301
human.FAM230J-LOC105372942-GGTLC5P.NCBI.rev.compl                           cagcatcaaggtggagctgaacagaggcagaaggaggaggacccaaggtggtgtcactca 37020
human.FAM230A.ncRNA.18487127.18500594-GGTLC3.18516335.18518165.rev.compl.REF cagcatcaaggtggagctgaacagaggcagaaggaggaggacccaaggtggtgtcactca 25767
                                                                            ************************************************************

chimp.LOC112206744-LOC107973052-GGT2.revcompl                               ggacccgggttcaagtccttatgcttctgcagcctggcctgggtcccccaatccccccag 38931
chimp54546-135457.revcompl.Pan.troglodytes.clone.rp43-41g5.GenBank:AC099533.36   GGACCCGGGTTCAAGTCCTTATGCTTCTGCAGCCTGGCCTGGGTCCCCCAACCCCCCCAG 40732
human.FAM30B-LOC105372935-GGT2.NCBI.                                        ggacccgggttcaagtccttatgtttctgcagcctggcctgggtcccccaacccccccag 37619
human.FAM230E-LOC105377182-GGT3P.NCBI.                                      ggacccgggttcaagtccttatgcttctgcagcctggcctgggtcccccaactcccccag 36361
human.FAM230J-LOC105372942-GGTLC5P.NCBI.rev.compl                           ggacccgggttcaagtccttatgcttctgcagcctggcctgggtcccccaactcccccag 37080
human.FAM230A.ncRNA.18487127.18500594-GGTLC3.18516335.18518165.rev.compl.REF ggacccgggttcaagtccttatgcttctgcagcctggcctgggtcccccaactcccccag 25827
                                                                            *********************** ***************************  *******

chimp.LOC112206744-LOC107973052-GGT2.revcompl                               ggtgaccaagggcttcccagtctgcacagaggacagggggtcttgacagcatcaaatgct 38991
chimp54546-135457.revcompl.Pan.troglodytes.clone.rp43-41g5.GenBank:AC099533.36   GGTGACCAAGGGCTTCCCAGTCTGCACAGAGGACAGGGGGACTTGACAGCATCAAATGCT 40792
human.FAM30B-LOC105372935-GGT2.NCBI.                                        ggtgaccaagggcttcccagtctgcacagaggacagggggtcttgacagcatcaaatgct 37679
human.FAM230E-LOC105377182-GGT3P.NCBI.                                      ggtgaccaagggcttcccagtctgcacagaggacagggggtcttgacagcatcaaatgct 36421
human.FAM230J-LOC105372942-GGTLC5P.NCBI.rev.compl                           ggtgaccaagggcttcccagtctgcacagaggacagggggtcttgacagcatcaaatgct 37140
human.FAM230A.ncRNA.18487127.18500594-GGTLC3.18516335.18518165.rev.compl.REF ggtgaccaagggctttccagtctgcacagaggacagggggtcttgacagcatcaaattct 25887
                                                                            *************** ************************ **************** **
```

```
chimp.LOC112206744-LOC107973052-GGT2.revcompl                              ggtgactatgagacacttacatgggaaatgcagacagaccatgcctctagcccttggtac 39051
chimp54546-135457.revcompl.Pan.troglodytes.clone.rp43-41g5.GenBank:AC099533.36    GGTGACTATGAGACACTTACGTGGGAAATGCAGACAGACCATGCCTCTAGCCCTTGGTAC 40852
human.FAM30B-LOC105372935-GGT2.NCBI.                                       ggtgactatgagacacttacatgggaaatgcagacagaccatgcctctagcccttggcac 37739
human.FAM230E-LOC105377182-GGT3P.NCBI.                                     agtgactacgagacacttacgtgggaaatgcagacagaccatgcctctagcccttggcac 36481
human.FAM230J-LOC105372942-GGTLC5P.NCBI.rev.compl                          agtgactacgagacacttacgtgggaaatgcagacagaccatgcctctagcccttggcac 37200
human.FAM230A.ncRNA.18487127.18500594-GGTLC3.18516335.18518165.rev.compl.REF ggtgactacgagacacttacgtgggaaatgcagacagaccatgcctctagcccttggcac 25947
                                                                            ******* *********** ************************************ **

chimp.LOC112206744-LOC107973052-GGT2.revcompl                              ccggcaccatccatccctgggacttgctgtcctggaaatgcagcatggacctccagggag 39111
chimp54546-135457.revcompl.Pan.troglodytes.clone.rp43-41g5.GenBank:AC099533.36    CCGGCACCATCCATCCCTGGGACTTGCTGTCCTGGAAATGCAGCATGGACCTCCAGGGAG 40912
human.FAM30B-LOC105372935-GGT2.NCBI.                                       caggcaccatccatccctgggacttgctgtcctggaaatgcagcatggacctccagggag 37799
human.FAM230E-LOC105377182-GGT3P.NCBI.                                     caggcaccatccatccctgggacttgctgtcctggaaatgcagcatggtcctccagggag 36541
human.FAM230J-LOC105372942-GGTLC5P.NCBI.rev.compl                          caggcaccatccatccctgggacttgctgtcctggaaatgcagcatggtcctccagggag 37260
human.FAM230A.ncRNA.18487127.18500594-GGTLC3.18516335.18518165.rev.compl.REF caggcaccatccatccctgggacttgctgtcctggaaatgcagcatggtcctccagggag 26007
                                                                           * ********************************************** ***********

chimp.LOC112206744-LOC107973052-GGT2.revcompl                              gggggctgtgccatgtgggggcctcaccccacctgcagctctttcccaccctggctgcag 39171
chimp54546-135457.revcompl.Pan.troglodytes.clone.rp43-41g5.GenBank:AC099533.36    GGGGGCTGTGCCATGTTGGGGCCCCACCCCACCTGCAGCTCTTTCCCACCCTGGCTGCAG 40972
human.FAM30B-LOC105372935-GGT2.NCBI.                                       gggggctgtgccatgtgggggccccaccccacctgcagctctttcccaccctggctgcag 37859
human.FAM230E-LOC105377182-GGT3P.NCBI.                                     gggggctgtgccatgtgggggccccaccccacctgcagctctttcccaccctggctgcag 36601
human.FAM230J-LOC105372942-GGTLC5P.NCBI.rev.compl                          gggggctgtgccatgtgggggccccaccccacctgcagctctttcccaccctggctgcag 37320
human.FAM230A.ncRNA.18487127.18500594-GGTLC3.18516335.18518165.rev.compl.REF gggggctgtgccatgtgggggccccaccccacctgcagctctttcccaccctggctgcag 26067
                                                                           **************** ****** ************************************

chimp.LOC112206744-LOC107973052-GGT2.revcompl                              gtctgcttccctgaatccaaatccgctactactgtgctggcagcgcagcctctctgggga 39231
chimp54546-135457.revcompl.Pan.troglodytes.clone.rp43-41g5.GenBank:AC099533.36    GTCTGCTTCCCTGAATCCAAATCCGCTACTACTGTGCTGGCAGCGCAGCCTCTCTGGGGA 41032
human.FAM30B-LOC105372935-GGT2.NCBI.                                       gtctgcttctctgaatccaaatccgctactactgtgctggcagcgcagcctctctgggga 37919
human.FAM230E-LOC105377182-GGT3P.NCBI.                                     gtctgcttccctgaatccaaatccgctactactgtgctggcagcgcagcctctctgggga 36661
human.FAM230J-LOC105372942-GGTLC5P.NCBI.rev.compl                          gtctgcttccctgaatccaaatccgctactactgtgctggcagcgcagcctctctgggga 37380
human.FAM230A.ncRNA.18487127.18500594-GGTLC3.18516335.18518165.rev.compl.REF gtctgcttccctgaatccaaatccgctactactgtgctggcagcgcagcctctctgggga 26127
                                                                           ********* **************************************************

chimp.LOC112206744-LOC107973052-GGT2.revcompl                              cactggcctggctctgttctccccaggcctcagggtgcctaaatgggaggcaaccagggg 39291
chimp54546-135457.revcompl.Pan.troglodytes.clone.rp43-41g5.GenBank:AC099533.36    CACTGGCCTGGCTCTGTTCTCCCCAGGCCTCAGGGTGCCTAAATGGGAGGCAGCCAGGGG 41092
human.FAM30B-LOC105372935-GGT2.NCBI.                                       cactggcctggctctgttctccccaggcctcagggtgcctaaatgggaggcaaccagggg 37979
human.FAM230E-LOC105377182-GGT3P.NCBI.                                     cactggcctggctctgttctccccaggcctcagggtgcctaaatgggaggcaaccagggg 36721
human.FAM230J-LOC105372942-GGTLC5P.NCBI.rev.compl                          cactggcctggctctgttctccccaggcctcagggtgcctaaatgggaggcaaccagggg 37440
human.FAM230A.ncRNA.18487127.18500594-GGTLC3.18516335.18518165.rev.compl.REF cactggcctggctctgttctccccaggcctcagggtgcctaaatgggaggcaaccagggg 26187
                                                                           **************************************************** *******

chimp.LOC112206744-LOC107973052-GGT2.revcompl                              agtgaggacccactgaggggctccgttgaccaggctcagcaggggtgcaggtgatgtggg 39351
chimp54546-135457.revcompl.Pan.troglodytes.clone.rp43-41g5.GenBank:AC099533.36    AGTGAGGACCCACTGAGGGGCTCCGTTGACCAGGCTCAGCAGGGGTGCAGGTGATGTGGG 41152
human.FAM30B-LOC105372935-GGT2.NCBI.                                       agtgaggacccactgaggggctccgttgaccaggctcagcaggggtgcaggtgatgtggg 38039
human.FAM230E-LOC105377182-GGT3P.NCBI.                                     agtgaggacccactgaggggctccgttgaccaggctcagcaggggtgcaggtgatgtggg 36781
human.FAM230J-LOC105372942-GGTLC5P.NCBI.rev.compl                          agtgaggacccactgaggggctccgttgaccaggctcagcaggggtgcaggtgatgtggg 37500
human.FAM230A.ncRNA.18487127.18500594-GGTLC3.18516335.18518165.rev.compl.REF agtgaggacccactgaggggctccgttgaccaggctcagcaggggtgcaggtgatgtggg 26247
                                                                           ************************************************************

chimp.LOC112206744-LOC107973052-GGT2.revcompl                              ctggacttcttcccacgtggcccccacagtcctccccgcttcctccccagctgaacactg 39411
chimp54546-135457.revcompl.Pan.troglodytes.clone.rp43-41g5.GenBank:AC099533.36    GTGGAATCCTTCCCACATGGCCCCCACAGTCCTCCCCGCTTCCTCCCCAGCTGAACACTG 41212
human.FAM30B-LOC105372935-GGT2.NCBI.                                       gtggaatccttcccacatggcccccacagtcctccccgcttcctccccaactgaacactg 38099
human.FAM230E-LOC105377182-GGT3P.NCBI.                                     gtggaatccttcccacatggcccccacagtcctccccgcttcctccccaactgaacactg 36841
human.FAM230J-LOC105372942-GGTLC5P.NCBI.rev.compl                          gtggaatccttcccacatggcccccacagtcctccccgcttcctccccaactgaacactg 37560
human.FAM230A.ncRNA.18487127.18500594-GGTLC3.18516335.18518165.rev.compl.REF gtggaatccttcccacatggcccccacagtcctccccgcttcctccccaactgaacactg 26307
                                                                            **** * ******** ******************************** **********

chimp.LOC112206744-LOC107973052-GGT2.revcompl                              cctgctccagatgtctacacctggagtccgggcccctccatctgggcagcagagaaactg 39471
chimp54546-135457.revcompl.Pan.troglodytes.clone.rp43-41g5.GenBank:AC099533.36    CCTGCTCCAGATGTCTACACCTGGAGTCTGGGCCCCTCCATCTGGGCAGCAGAGAAACTG 41272
human.FAM30B-LOC105372935-GGT2.NCBI.                                       cctgctccagatgtgtacacctggagtctgggcccctccatctgggcagcagagaaactg 38159
human.FAM230E-LOC105377182-GGT3P.NCBI.                                     cctgctccagatgtctacacctggagtctgggcccctccatctgggcagcagagaaactg 36901
human.FAM230J-LOC105372942-GGTLC5P.NCBI.rev.compl                          cctgctccagatgtctacacctggagtctgggcccctccatctgggcagcagagaaactg 37620
human.FAM230A.ncRNA.18487127.18500594-GGTLC3.18516335.18518165.rev.compl.REF cctgctccagatgtgtacacctggagtctgggcccctccatctgggcagcagagaaactg 26367
                                                                           ************** ************* *******************************

chimp.LOC112206744-LOC107973052-GGT2.revcompl                              aggcacagagacagactgtgtccttacaggccacacagcctgccaggcccctatgtccgg 39531
chimp54546-135457.revcompl.Pan.troglodytes.clone.rp43-41g5.GenBank:AC099533.36    AGGCACAGAGACAGACTGTGTCCTTACAGGCCACACAGCCTGCCAGGCCCCTATGTCCGG 41332
human.FAM30B-LOC105372935-GGT2.NCBI.                                       aggcacagagacagactgtgtccttacagggcacacagcctgccaggcccctatgtccgg 38219
human.FAM230E-LOC105377182-GGT3P.NCBI.                                     aggcacagagacagactgtgtccttacagggcacacagcctgccaggcccctatgtccgg 36961
human.FAM230J-LOC105372942-GGTLC5P.NCBI.rev.compl                          aggcacagagacagactgtgtccttacagggcacacagcctgccaggcccctatgtccgg 37680
human.FAM230A.ncRNA.18487127.18500594-GGTLC3.18516335.18518165.rev.compl.REF aggcacagagacagactgtgtccttacagggcacacagcctgccaggcccctatgtccgg 26427
                                                                           ****************************** *****************************

chimp.LOC112206744-LOC107973052-GGT2.revcompl                              ccagagcccctggtcagcctgggctgcagtgattgtttagaggtaggctgttcccacggc 39591
chimp54546-135457.revcompl.Pan.troglodytes.clone.rp43-41g5.GenBank:AC099533.36    CCAGAGCCCCTGGTCAGCCTGGGCTGCAGTGATTGTTTAGAGGTAGGCTGTTCCCACGGC 41392
human.FAM30B-LOC105372935-GGT2.NCBI.                                       ccagagcccctggtcagcctgggctgcagtgattgtttagaggtaggctgttcccacggc 38279
human.FAM230E-LOC105377182-GGT3P.NCBI.                                     ccagagcccctggccagcctgggctgcagtgattgtttagaggtaggctgttcccacggc 37021
human.FAM230J-LOC105372942-GGTLC5P.NCBI.rev.compl                          ccagagcccctggccagcctgggctgcagtgattgtttagaggtaggctgttcccacggc 37740
human.FAM230A.ncRNA.18487127.18500594-GGTLC3.18516335.18518165.rev.compl.REF ccagagcccctggtcagcctgggctgcagtgattgtttagaggtaggctgttcccacggc 26487
                                                                           ************ ***********************************************

chimp.LOC112206744-LOC107973052-GGT2.revcompl                              tgcctctcacggtaggggggcctgcggacgcctcctcccgtcccccacccgactcccaagc 39651
chimp54546-135457.revcompl.Pan.troglodytes.clone.rp43-41g5.GenBank:AC099533.36    TGCCTCTCACGGTAGGGGGGCCTGTGGACGCCTCCTCCCACCCCCACCCGACTCCCAAGC 41452
human.FAM30B-LOC105372935-GGT2.NCBI.                                       tgcctctcacggtaggggggcctgcggacgcctcctcccgcccccacccgactcccaagc 38339
human.FAM230E-LOC105377182-GGT3P.NCBI.                                     tgcctctcacggtaggggggcctgcggacgcctcctcccgcccccacccgactcccaagc 37081
human.FAM230J-LOC105372942-GGTLC5P.NCBI.rev.compl                          tgcctctcacggtaggggggcctgcggacgcctcctcccgcccccacccgactcccaagc 37800
human.FAM230A.ncRNA.18487127.18500594-GGTLC3.18516335.18518165.rev.compl.REF tgcctctcacggtaggggggcctgtggacgcctcctcccgcccccacccgactcccaagc 26547
                                                                           ************************ **************  *******************

chimp.LOC112206744-LOC107973052-GGT2.revcompl                              ctcagtgacattgctcaaccaggagctgaagtgcattcctgggctcaggccagcccaccc 39711
chimp54546-135457.revcompl.Pan.troglodytes.clone.rp43-41g5.GenBank:AC099533.36    CTCAGTGACATTGCTCAACCAGGAGCTGAAGTGCATTCCTGGGCTCAGGCCAGCCCACCC 41512
human.FAM30B-LOC105372935-GGT2.NCBI.                                       ctcagtgacattgctcaaccaggaactgaagtgcattcctgggctcaggccagcccaccc 38399
human.FAM230E-LOC105377182-GGT3P.NCBI.                                     ctcagtgacattgctcaaccaggagctgaagtgcattcctgggctcaggccagcccaccc 37141
human.FAM230J-LOC105372942-GGTLC5P.NCBI.rev.compl                          ctcagtgacattgctcaaccaggagctgaagtgcattcctgggctcaggccagcccaccc 37860
human.FAM230A.ncRNA.18487127.18500594-GGTLC3.18516335.18518165.rev.compl.REF ctcagtgacattgctcaaccaggagctgaagtgcattcctgggctccggccagcccaccc 26607
                                                                           ************************ ********************* *************

chimp.LOC112206744-LOC107973052-GGT2.revcompl                              acccacccgctgcagtcctggaagcccagaggcctgggcagcaggaacagtggagacagc 39771
chimp54546-135457.revcompl.Pan.troglodytes.clone.rp43-41g5.GenBank:AC099533.36    ACCCACCCGCTGCAGTCCTGGAAGCTCAGAGGCCTGGGCAGCAGGAACAGTGGAGACAGC 41572
human.FAM30B-LOC105372935-GGT2.NCBI.                                       acccacccgctgcagtcctggaagcccagaggcctgggcagcaggaacagtggagacagc 38459
human.FAM230E-LOC105377182-GGT3P.NCBI.                                     accgacccgctgcagtcctggaagcccagaggcctgggcagcaggaacagtggagacagc 37201
human.FAM230J-LOC105372942-GGTLC5P.NCBI.rev.compl                          accgacccgctgcagtcctggaagcccagaggcctgggcagcaggaacagtggagacagc 37920
human.FAM230A.ncRNA.18487127.18500594-GGTLC3.18516335.18518165.rev.compl.REF acccacccgctgcagtcctggaagcccagaggcctgggcagcaggaacagtggagacagc 26667
                                                                           *** ********************* **********************************

chimp.LOC112206744-LOC107973052-GGT2.revcompl                              agtgtgggggacgtcccccctcctctccccaccatcctcgtcaggcagaggccagggtgc 39831
chimp54546-135457.revcompl.Pan.troglodytes.clone.rp43-41g5.GenBank:AC099533.36    AGTGTGGGGGACATCCCCCCTCCTCTCCCCACCATCCTCGTCAGGCAGAGGCCAGGGTGC 41632
human.FAM30B-LOC105372935-GGT2.NCBI.                                       agtgtgggggacgtcccccctcctctccccaccatcctcgtcaggcagaggccagggtgc 38519
human.FAM230E-LOC105377182-GGT3P.NCBI.                                     agtgtgggggacgt-cccccctcctctccccaccatcctcgtcaggcagaggccagggtgc 37260
human.FAM230J-LOC105372942-GGTLC5P.NCBI.rev.compl                          agtgtgggggacgt-cccccctcctctccccaccatcctcgtcaggcagaggccagggtgc 37979
human.FAM230A.ncRNA.18487127.18500594-GGTLC3.18516335.18518165.rev.compl.REF agtgtgggggacgt-cccccctcctctccccaccatcctcgtcaggcagaggccagggtgc 26726
                                                                           ************ * *********************************************

chimp.LOC112206744-LOC107973052-GGT2.revcompl                              agggaccacccgagcaaaggcccagggaaatgaatgggtgtcattttggtcctgacccga 39891
chimp54546-135457.revcompl.Pan.troglodytes.clone.rp43-41g5.GenBank:AC099533.36    AGGGACCACCCGAGCAAAGGCCCAGGGAAATGAATGGGTGTCATTCTGGTCCTGACCCGA 41692
human.FAM30B-LOC105372935-GGT2.NCBI.                                       agggaccaccggagcaaaggcccagggaaatgaatgggtgtcattctggtcctgacccga 38579
human.FAM230E-LOC105377182-GGT3P.NCBI.                                     agggaccgcccgagcaaaggcccagggaaatgaatgggtgtcattctggtcctgacccga 37320
human.FAM230J-LOC105372942-GGTLC5P.NCBI.rev.compl                          agggaccgcccgagcaaaggcccagggaaatgaatgggtgtcattctggtcctgacccga 38039
human.FAM230A.ncRNA.18487127.18500594-GGTLC3.18516335.18518165.rev.compl.REF agggaccaccggagcaaaggcccagggaaatgaatgggtgtcattctggtcctgacccga 26786
                                                                           ******* ** ********************************** **************

chimp.LOC112206744-LOC107973052-GGT2.revcompl                              ggcacagccaggaaggtccctgtggggaaaagaaacagatatcagactgttactgtgtct 39951
chimp54546-135457.revcompl.Pan.troglodytes.clone.rp43-41g5.GenBank:AC099533.36    GGCACAGCCAGGAAGGTCCCTGTGGGGAAAAGAAAGAGATATCAGACTGTTACTGTGTCT 41752
human.FAM30B-LOC105372935-GGT2.NCBI.                                       ggcacagccaggaaggtccctgtggggaaaagaaagagatatcagactgttactgtgtct 38639
human.FAM230E-LOC105377182-GGT3P.NCBI.                                     ggcacagccaggaaggtccctgtggggaaaagaaagagatatcagactgttactgtgtct 37380
human.FAM230J-LOC105372942-GGTLC5P.NCBI.rev.compl                          ggcacagccaggaaggtccctgtggggaaaagaaagagatatcagactgttactgtgtct 38099
human.FAM230A.ncRNA.18487127.18500594-GGTLC3.18516335.18518165.rev.compl.REF ggcacagccaggaaggtccctgtggggaaaagaaagagatatcagactgttactgtgtct 26846
                                                                           *********************************** ************************

chimp.LOC112206744-LOC107973052-GGT2.revcompl                              atgtagaaagaagtagaggtaagaggctccattttgttgtgtagtaagaaaaattctttt 40011
chimp54546-135457.revcompl.Pan.troglodytes.clone.rp43-41g5.GenBank:AC099533.36    ATGTAGAAAGAAGTAGACGTAAGAGGCTCCATTTTGTTCTGTACTAAGAAAAATTCTTTT 41812
human.FAM30B-LOC105372935-GGT2.NCBI.                                       atgtagaaagaagtagacgtaagaggctccattttgttgtgtagtaagaaaaattctttt 38699
human.FAM230E-LOC105377182-GGT3P.NCBI.                                     atgtagaaagaagtagacgtaagaggctccattttgttgtgtagtaagaaaaattctttt 37440
human.FAM230J-LOC105372942-GGTLC5P.NCBI.rev.compl                          atgtagaaagaagtagacgtaagaggctccattttgttgtgtagtaagaaaaattctttt 38159
human.FAM230A.ncRNA.18487127.18500594-GGTLC3.18516335.18518165.rev.compl.REF atgtagaaagaagtagacgtaagaggctccattttgctgtgtagtaagaaaaattctttt 26906
                                                                           ***************** ***************** * **** *****************

chimp.LOC112206744-LOC107973052-GGT2.revcompl                              gccttgagatactgttaatctgtaaccctagccccaaccctgtgctcacagaaacatgta 40071
chimp54546-135457.revcompl.Pan.troglodytes.clone.rp43-41g5.GenBank:AC099533.36    GCCTTGAGATGCTGTTAATCTGTAACCCTAGCCCCAACCCTGTGCTCACAGAAACATGTA 41872
human.FAM30B-LOC105372935-GGT2.NCBI.                                       gccttgagatgccgttaatctgtaaccctagccccaaccctgtgctcacagaaacatgta 38759
human.FAM230E-LOC105377182-GGT3P.NCBI.                                     gccttgagatgccgttaatctgtaaccctagccccaaccctgtgctcacagaaacatgta 37500
human.FAM230J-LOC105372942-GGTLC5P.NCBI.rev.compl                          gccttgagatgccgttaatctgtaaccctagccccaaccctgtgctcacagaaacatgta 38219
human.FAM230A.ncRNA.18487127.18500594-GGTLC3.18516335.18518165.rev.compl.REF gccttgagatgccgttaatctgtaaccctagccccaaccctgtgctcacagaaacatgta 26966
                                                                           ********** * ***********************************************

chimp.LOC112206744-LOC107973052-GGT2.revcompl                              ctgtgtcgactcaaggtttaatggatttagggctgtgcaggatgtgctttgttaaacaaa 40131
```

```
chimp54546-135457.revcompl.Pan.troglodytes.clone.rp43-41g5.GenBank:AC099533.36      CTGTGTCGACTCAAGGTTTAATGGATTTAGGGCTGTGCAGGATGTGCTTTGTTAAACAAA 41932
human.FAM30B-LOC105372935-GGT2.NCBI.                                                ctgtgtcgactcaaggtttaatggattcagggctgtgcaggatgtgctttgttaaacaaa 38819
human.FAM230E-LOC105377182-GGT3P.NCBI.                                              ctgtgtcgactcaaggtttaatggattcagggctgtgcaggatgtgctttgttaaacaaa 37560
human.FAM230J-LOC105372942-GGTLC5P.NCBI.rev.compl                                   ctgtgtcgactcaaggtttaatggattcagggctgtgcaggatgtgctttgttaaacaaa 38279
human.FAM230A.ncRNA.18487127.18500594-GGTLC3.18516335.18518165.rev.compl.REF        ctgtgtcgactcaaggtttaatggattcagggctgtgcaggatgtgctttgttaaacaaa 27026
                                                                                    *************************** ********************************

chimp.LOC112206744-LOC107973052-GGT2.revcompl                                       tgcttgaaggcagcatgcttgttaaaagtcatcaccactccctaatctcaagtaagcagg 40191
chimp54546-135457.revcompl.Pan.troglodytes.clone.rp43-41g5.GenBank:AC099533.36      TGCTTGAAGGCAGCATGCTTGTTAAAAGTCATCACCACTCCCTAATCTCAAGTAAGCAGG 41992
human.FAM30B-LOC105372935-GGT2.NCBI.                                                tgcttgaaggcagcatgcttgttaagagtcatcaccactccctaatctcaagtaagcagg 38879
human.FAM230E-LOC105377182-GGT3P.NCBI.                                              tgcttgaaggcagcatgcttgttaagagtcatcaccactccctaatctcaagtaagcagg 37620
human.FAM230J-LOC105372942-GGTLC5P.NCBI.rev.compl                                   tgcttgaaggcagcatgcttgttaagagtcatcaccactccctaatctcaagtaagcagg 38339
human.FAM230A.ncRNA.18487127.18500594-GGTLC3.18516335.18518165.rev.compl.REF        tgcttgaaggcagcatgcttgttaagagtcatcaccactccctaatctcaagtaagcagg 27086
                                                                                    ************************* **********************************

chimp.LOC112206744-LOC107973052-GGT2.revcompl                                       gacacaaaacactgcagaaggccgcagggacctctgcctaggaaagccaggtattgtcca 40251
chimp54546-135457.revcompl.Pan.troglodytes.clone.rp43-41g5.GenBank:AC099533.36      GACACAAAACACTGCAGAAGGCCGCAGGGACCTCTGCCTAGGAAAGCCAGGTATTGTCCA 42052
human.FAM30B-LOC105372935-GGT2.NCBI.                                                gacacaaaacactgcagaaggccgcagggacctctgcctaggaaagccaggtatcgtcca 38939
human.FAM230E-LOC105377182-GGT3P.NCBI.                                              gacacaaaacactgcagaaggccgcagggacctctgcctaggaaagccaggtattgtcca 37680
human.FAM230J-LOC105372942-GGTLC5P.NCBI.rev.compl                                   gacacaaaacactgcagaaggccgcagggacctctgcctaggaaagccaggtattgtcca 38399
human.FAM230A.ncRNA.18487127.18500594-GGTLC3.18516335.18518165.rev.compl.REF        gacacaaaacactgcagaaggccgcagggacctctgcctaggaaagccaggtattgtcca 27146
                                                                                    ****************************************************** *****

chimp.LOC112206744-LOC107973052-GGT2.revcompl                                       aggtttctccccatgtgacagtctgaaatatggcctcgtgggaagggaaagacctgaccg 40311
chimp54546-135457.revcompl.Pan.troglodytes.clone.rp43-41g5.GenBank:AC099533.36      AGGTTTCTCCCCATGTGACAGTCTGAAATATGGCCTCGTGGGAAGGGAAAGACCTGACCG 42112
human.FAM30B-LOC105372935-GGT2.NCBI.                                                aggtttctccccaggtgacagtctgaaatatggcctcgtgggaagggaaagacctgaccg 38999
human.FAM230E-LOC105377182-GGT3P.NCBI.                                              aggtttctccccaggtgacagtctgaaatatggcctcgtgggaagggaaagacctgaccg 37740
human.FAM230J-LOC105372942-GGTLC5P.NCBI.rev.compl                                   aggtttctccccaggtgacagtctgaaatatggcctcgtgggaagggaaagacctgaccg 38459
human.FAM230A.ncRNA.18487127.18500594-GGTLC3.18516335.18518165.rev.compl.REF        aggtttctccccaggtgacagtctgaaatatggcctcgtgggaagggaaagacctgaccg 27206
                                                                                    ************* **********************************************

chimp.LOC112206744-LOC107973052-GGT2.revcompl                                       tcccccagcccgacacctgtaaagggtctgtgctgaggaggattagtaaaag----ggga 40367
chimp54546-135457.revcompl.Pan.troglodytes.clone.rp43-41g5.GenBank:AC099533.36      TCCCCCAGCCCGACACCCGTAAAGGGTCTGTGCTGAGGAGGATTAGTATAAGAGGAAGGA 42172
human.FAM30B-LOC105372935-GGT2.NCBI.                                                tcccccagcccgacacctgtaaagggtctgtgctgaggaggattagtaaaag----agga 39055
human.FAM230E-LOC105377182-GGT3P.NCBI.                                              tcccccagcccgacacctgtaaagggtctgtgctgaggaggattagtaaaag----agga 37796
human.FAM230J-LOC105372942-GGTLC5P.NCBI.rev.compl                                   tcccccagcccgacacctgtaaagggtctgtgctgaggaggattagtaaaag----agga 38515
human.FAM230A.ncRNA.18487127.18500594-GGTLC3.18516335.18518165.rev.compl.REF        tcccccagcccgacacctgtaaagggtctgtgctgaggaggattagtaaaag----agga 27262
                                                                                    ***************** ****************************** ***     ***

chimp.LOC112206744-LOC107973052-GGT2.revcompl                                       aggcctctttgcagttgagataagaggaaggcatctctctcctgatcgtccctgggcaaa 40427
chimp54546-135457.revcompl.Pan.troglodytes.clone.rp43-41g5.GenBank:AC099533.36      AGGCCTCTTTGCAGTTGAGATAAGAGGAAGGCATCTGTCTCCTGCCCGTCCCTGGGCAAA 42232
human.FAM30B-LOC105372935-GGT2.NCBI.                                                aggcctctttgcagttgagataagaggaaggcatctctctcctgatcgtccctgggcaaa 39115
human.FAM230E-LOC105377182-GGT3P.NCBI.                                              aggcctctttgcagttgagataagaggaaggcatctctctcctgatcgtccctgggcaaa 37856
human.FAM230J-LOC105372942-GGTLC5P.NCBI.rev.compl                                   aggcctctttgcagttgagataagaggaaggcatctctctcctgatcgtccctgggcaaa 38575
human.FAM230A.ncRNA.18487127.18500594-GGTLC3.18516335.18518165.rev.compl.REF        aggcctctttgcagttgagataagaggaaggcatctctctcctgatcgtccctgggcaaa 27322
                                                                                    ************************************ *******  **************

chimp.LOC112206744-LOC107973052-GGT2.revcompl                                       ggaatgtctcagtgttgatggtatattccatctactgagataggagaaaactgccttagg 40487
chimp54546-135457.revcompl.Pan.troglodytes.clone.rp43-41g5.GenBank:AC099533.36      GGAATGTGTAAAACCCAATTGTATATTCCATCTACTGAGATAGGAGAAAACTGCCTTAGG 42292
human.FAM30B-LOC105372935-GGT2.NCBI.                                                ggaatgtctcagtgttgattgtatattccatctgctgagataggagaaaactgccttagg 39175
human.FAM230E-LOC105377182-GGT3P.NCBI.                                              ggaatgtctcagtgttgattgtatattccatctgctgagataggagaaaactgccttagg 37916
human.FAM230J-LOC105372942-GGTLC5P.NCBI.rev.compl                                   ggaatgtctcagtgttgattgtatattccatctgctgagataggagaaaactgccttagg 38635
human.FAM230A.ncRNA.18487127.18500594-GGTLC3.18516335.18518165.rev.compl.REF        ggaatgtctcagtgttgattgtatattccatctgctgagataggagaaaactgccttagg 27382
                                                                                    ******* * *      ** ************* **************************

chimp.LOC112206744-LOC107973052-GGT2.revcompl                                       gctggaggtgggacatgctggtggcaatactgctatttaatgcattgagatgtttatgta 40547
chimp54546-135457.revcompl.Pan.troglodytes.clone.rp43-41g5.GenBank:AC099533.36      GCTGGAGGTGGGACATGCTGGTGACAATACTGCTCTTTAATGCATTGAGATGTTTATGTA 42352
human.FAM30B-LOC105372935-GGT2.NCBI.                                                gctggagatgggacatgctggtggcaatactgctctttaatgcattgagatgtttatgta 39235
human.FAM230E-LOC105377182-GGT3P.NCBI.                                              gctggaggtgggacatgctggtggcaatactgctctttaatgcattgagatgtttatgta 37976
human.FAM230J-LOC105372942-GGTLC5P.NCBI.rev.compl                                   gctggaggtgggacatgctggtggcaatactgctctttaatgcattgagatgtttatgta 38695
human.FAM230A.ncRNA.18487127.18500594-GGTLC3.18516335.18518165.rev.compl.REF        gctggaggtgggacatgctggtggcaatactgctctttaatgcattgagatgtttatgta 27442
                                                                                    ******* *************** ********** *************************

chimp.LOC112206744-LOC107973052-GGT2.revcompl                                       tatgcacatcaaagcacagcacctttttcttaaccttgtttatgacacagagacatttgt 40607
chimp54546-135457.revcompl.Pan.troglodytes.clone.rp43-41g5.GenBank:AC099533.36      TATGCACATCAAAGCACAGCACCTTTTTCTTAACCTTGTTTATGACACAGAGACATTTGT 42412
human.FAM30B-LOC105372935-GGT2.NCBI.                                                tatgcacatcaaagcatagcacctttttcttaaccttgtttatgacacagagacatttgt 39295
human.FAM230E-LOC105377182-GGT3P.NCBI.                                              tatgcacatcaaagcacagcacctttttcttaaccttgtttatgacacagagacatttgt 38036
human.FAM230J-LOC105372942-GGTLC5P.NCBI.rev.compl                                   tatgcacatcaaagcacagcacctttttcttaaccttgtttatgacacagagacatttgt 38755
human.FAM230A.ncRNA.18487127.18500594-GGTLC3.18516335.18518165.rev.compl.REF        tatgcacatcaaagcacagcacctttttcttaaccttgtttatgacacagagacatttgt 27502
                                                                                    **************** *******************************************

chimp.LOC112206744-LOC107973052-GGT2.revcompl                                       tcacgtgttttcctgctgaccctctccccactattaccctattgtcctgccacatccccc 40667
chimp54546-135457.revcompl.Pan.troglodytes.clone.rp43-41g5.GenBank:AC099533.36      TCACGTGTTTTCCTGCTGACCCTCACCCCACTATTACCATATCGTCCTGCCACATCCCCC 42472
human.FAM30B-LOC105372935-GGT2.NCBI.                                                tcacgtgttttcctgctgaccctctccccactattaccctattgtcctgccacatccccc 39355
human.FAM230E-LOC105377182-GGT3P.NCBI.                                              tcacgtgttttcctgctgaccctctccccactattaccctattgtcctgccacatccccc 38096
human.FAM230J-LOC105372942-GGTLC5P.NCBI.rev.compl                                   tcacgtgttttcctgctgaccctctccccactattaccctattgtcctgccacatccccc 38815
human.FAM230A.ncRNA.18487127.18500594-GGTLC3.18516335.18518165.rev.compl.REF        tcacgtgttttcctgctgaccctctccccactattaccctattgtcctgccacatccccc 27562
                                                                                    ************************ ************* *** *****************

chimp.LOC112206744-LOC107973052-GGT2.revcompl                                       tctctgagatggtagagataatgatcaataaatactagggaactcagagactggtgccag 40727
chimp54546-135457.revcompl.Pan.troglodytes.clone.rp43-41g5.GenBank:AC099533.36      TCTCTGAGATGGTAGAGATAATGATCAATAAATACTAGGGAACTCAGAGACTGGTGCCAG 42532
human.FAM30B-LOC105372935-GGT2.NCBI.                                                tctctgagatggtagagataatgatcaataaatactagggaactcagagactggtgccag 39415
human.FAM230E-LOC105377182-GGT3P.NCBI.                                              tctctgagatggtagagataatgatcaataaatactagggaactcagagactggtgccag 38156
human.FAM230J-LOC105372942-GGTLC5P.NCBI.rev.compl                                   tctctgagatggtagagataatgatcaataaatactagggaactcagagactggtgccag 38875
human.FAM230A.ncRNA.18487127.18500594-GGTLC3.18516335.18518165.rev.compl.REF        tctctgagatggtagagataatgatcaataaatactagggaactcagagactggtgccag 27622
                                                                                    ************************************************************

chimp.LOC112206744-LOC107973052-GGT2.revcompl                                       cgtgggtcctccatatgctgagtgcaggtcccctgggcccacttttctttctctatactt 40787
chimp54546-135457.revcompl.Pan.troglodytes.clone.rp43-41g5.GenBank:AC099533.36      CGTGGGTCCTCCATATGCTGAGTGCAGGTCCCCTGGGCCCACTTTTCTTTCTCTATACTT 42592
human.FAM30B-LOC105372935-GGT2.NCBI.                                                cgtggggcctccgtatgctgagcgcaggtcccctgggcccacttttctttctctatactt 39475
human.FAM230E-LOC105377182-GGT3P.NCBI.                                              cgtggggcctccatatgctgagtgcaggtcccctgggcccacttttctttctctatactt 38216
human.FAM230J-LOC105372942-GGTLC5P.NCBI.rev.compl                                   cgtggggcctccgtatgctgagtgcaggtcccctgggcccacttttctttctctatactt 38935
human.FAM230A.ncRNA.18487127.18500594-GGTLC3.18516335.18518165.rev.compl.REF        cgtggggcctccatatgctgagtgcaggtcccctgggcccacttttctttctctatactt 27682
                                                                                    ****** ***** ********* *************************************

chimp.LOC112206744-LOC107973052-GGT2.revcompl                                       tgtctctgtgtctctttcttttctcagtctcttgtcccacctgatgagaaacacccacag 40847
chimp54546-135457.revcompl.Pan.troglodytes.clone.rp43-41g5.GenBank:AC099533.36      TGTCTCTGTGTCTCTTTCTTTTCTCAGTCTCTTGTCCCACCTGATGAGAAACACCCACAG 42652
human.FAM30B-LOC105372935-GGT2.NCBI.                                                tgtctctgtgtctctttcttttctcagtctcttgtcccagctgatgggaaacacccacag 39535
human.FAM230E-LOC105377182-GGT3P.NCBI.                                              tggctctgtggctctttcttttctcagtctcttgtcccagctgatgagaaacacccacag 38276
human.FAM230J-LOC105372942-GGTLC5P.NCBI.rev.compl                                   tgtctctgtgtctctttcttttctcagtctcttgtcccagctgatgagaaacacccacag 38995
human.FAM230A.ncRNA.18487127.18500594-GGTLC3.18516335.18518165.rev.compl.REF        tgtctctgtgtctctttcttttctcagtctcttgtcccagctgatgagaaacacccacag 27742
                                                                                    ** ******* **************************** ****** *************

chimp.LOC112206744-LOC107973052-GGT2.revcompl                                       atgtggagggcaggccaccccttcaggtccctgaatgtccttcctcaggaaatgatggg 40907
chimp54546-135457.revcompl.Pan.troglodytes.clone.rp43-41g5.GenBank:AC099533.36      ATGTGGAGGGCAGGCCGCTCCTTCAGGTCCCTGAATGTCCTTCCTCAGGAAATGATGGG 42712
human.FAM30B-LOC105372935-GGT2.NCBI.                                                gtgtggagggcaggccaccccttcaggtccctgaatgtccttcctcaggaaatgatggg 39595
human.FAM230E-LOC105377182-GGT3P.NCBI.                                              gtgtggagggcaggccaccccttcaggtccctgaatgtccttcctcaggaaatgatggg 38336
human.FAM230J-LOC105372942-GGTLC5P.NCBI.rev.compl                                   gtggggagggcaggccaccccttcaggtccctgaatgtccttcctcaggaaatgatggg 39055
human.FAM230A.ncRNA.18487127.18500594-GGTLC3.18516335.18518165.rev.compl.REF        gtgtggagggcaggccaccccttcaggtccctgaatgtccttcctcaggaaatgatggg 27802
                                                                                     ** ************ * *****************************************

chimp.LOC112206744-LOC107973052-GGT2.revcompl                                       ggaaggggtgatgagaatgaaggagaggatttaagtccctcacccccccgaggtagtcctg 40967
chimp54546-135457.revcompl.Pan.troglodytes.clone.rp43-41g5.GenBank:AC099533.36      GGAAGGGGTGATGAGAATGAAGGAGAGGATTTAAGTCCCTCACCCCCCGAGGTAGTCCTG 42772
human.FAM30B-LOC105372935-GGT2.NCBI.                                                ggaaggggtgatgagaatgaaggagacgatttaagtccctcacccccccgaggtagtcctg 39655
human.FAM230E-LOC105377182-GGT3P.NCBI.                                              ggaaggggcgatgagaatgaaggagacgatttaagtccctcacccccccgaggtagtcctg 38396
human.FAM230J-LOC105372942-GGTLC5P.NCBI.rev.compl                                   ggaaggggcgatgagaatgaaggagacgatttaagtccctcacccccccgaggtagtcctg 39115
human.FAM230A.ncRNA.18487127.18500594-GGTLC3.18516335.18518165.rev.compl.REF        ggaaggggcgatgagaatgaaggagacgatttaagtccctcacccccccgaggtagtcctg 27862
                                                                                    ******** ***************** *********************************

chimp.LOC112206744-LOC107973052-GGT2.revcompl                                       ggctgagccccatgggacctggagaaccagggtgtaccccaccagcgtgtcgggtccagg 41027
chimp54546-135457.revcompl.Pan.troglodytes.clone.rp43-41g5.GenBank:AC099533.36      GGCTGAGCCCCATGGGACCTGGAGAACCAGGGTGTACCCCACCAGTGTGTCGAGTCGAGG 42832
human.FAM30B-LOC105372935-GGT2.NCBI.                                                ggctgagccccatgggacctagagaaccagggtgtaccccaccagcgtgtcgggtccagg 39715
human.FAM230E-LOC105377182-GGT3P.NCBI.                                              ggctgagccccatgggacctagagaaccagggtgtaccccaccagcatgtcgggtccagg 38456
human.FAM230J-LOC105372942-GGTLC5P.NCBI.rev.compl                                   ggctgagccccatgggacctagagaaccagggtgtaccccaccagcatttcgggtccagg 39175
human.FAM230A.ncRNA.18487127.18500594-GGTLC3.18516335.18518165.rev.compl.REF        ggctgagccccatgggacctagagaaccagggtgtaccccaccagcatgtcgggtccagg 27922
                                                                                    ******************** ************************  * *** *** ***

chimp.LOC112206744-LOC107973052-GGT2.revcompl                                       aagcctcgtggccagctcccacttctcttgctgctgtgcaacccagagcaaggcctgccc 41087
chimp54546-135457.revcompl.Pan.troglodytes.clone.rp43-41g5.GenBank:AC099533.36      AAGCCTCGTGGCCAGCTCCCACTTCTCTTGCTGCTGTGCAACCCAGAGCAAGGCCTGCCC 42892
human.FAM30B-LOC105372935-GGT2.NCBI.                                                aagcctcgtggccagctcccacttctcttcctgctgtgcaacccagagcaaggcctgccc 39775
human.FAM230E-LOC105377182-GGT3P.NCBI.                                              aagcctcgtggccagctcccacttctcttcctgctgtgcaacccagagcaaggcctgccc 38516
human.FAM230J-LOC105372942-GGTLC5P.NCBI.rev.compl                                   aagcctcgtggccagctcccacttctcttcctgctgtgcaacccagagcaaggcctgccc 39235
human.FAM230A.ncRNA.18487127.18500594-GGTLC3.18516335.18518165.rev.compl.REF        aagcctcgtggccagctcccacttctcttcctgctgtgcaacccagagcaaggcctgccc 27982
                                                                                    ***************************** ******************************

chimp.LOC112206744-LOC107973052-GGT2.revcompl                                       ctccagcttcagtcttctcccctgcaaatggggccacggcctttcctctcaggccaaaat 41147
chimp54546-135457.revcompl.Pan.troglodytes.clone.rp43-41g5.GenBank:AC099533.36      CTCCAGCTTCAGTCTTCTCCCCTGCAAATGGGGCCACGGCCTTTCCTCTCAGGCCAAAAT 42952
human.FAM30B-LOC105372935-GGT2.NCBI.                                                ctccagcttcagtcttctcccctgcaaatggggccacggcctttcctctcaggccaatat 39835
human.FAM230E-LOC105377182-GGT3P.NCBI.                                              ctccagcttcagtcttctcccctgcaaatggggccacggcctttcctctcaggccaatat 38576
human.FAM230J-LOC105372942-GGTLC5P.NCBI.rev.compl                                   ctccagcttcagtcttctcccctgcaaatggggccacggcctttcctctcaggccaatat 39295
human.FAM230A.ncRNA.18487127.18500594-GGTLC3.18516335.18518165.rev.compl.REF        ctccagcttcagtcttctcccctgcaaatggggccacggcctttcctctcaggccaatat 28042
                                                                                    ********************************************************* **

chimp.LOC112206744-LOC107973052-GGT2.revcompl                                       aaggattgaggccgggtgcagtggctcacccctgtaatcctagcactttgggagactgag 41207
chimp54546-135457.revcompl.Pan.troglodytes.clone.rp43-41g5.GenBank:AC099533.36      AAGGATTGAGGCCGGGTGCAGTGGCTCACCCCTGTAATTTAGCACTTTGGGAGACTGAG 43012
```

```
human.FAM30B-LOC105372935-GGT2.NCBI.                                              aaggattgaggccgggtgcagtggct-acccctgtaatcctagcactttgggagactgag 39894
human.FAM230E-LOC105377182-GGT3P.NCBI.                                            aaggattgaggccgggtgcagtggct-acccctgtaatcctagcactttgggagactgag 38635
human.FAM230J-LOC105372942-GGTLC5P.NCBI.rev.compl                                 aaggattgaggccgggtgcagtggct-acccctgtaatcctagcactttgggagactgag 39354
human.FAM230A.ncRNA.18487127.18500594-GGTLC3.18516335.18518165.rev.compl.REF      aaggattgaggccgggtgcagtggct-acccctgtaatcctagcactttgggagactgag 28101
                                                                                  ************************** ***********  ********************

chimp.LOC112206744-LOC107973052-GGT2.revcompl                                     atgggggactgcttgaagtcaggagttaagaccagcctggtcaacatagtgagacccca 41267
chimp54546-135457.revcompl.Pan.troglodytes.clone.rp43-41g5.GenBank:AC099533.36     ATGGGGGACTGCTTGAAGTCAGGAGTTAAGACCAGCCTGGTCAACATAGTGAGACCCCA 43072
human.FAM30B-LOC105372935-GGT2.NCBI.                                              atgggggactgcttgaagtcaggagttaagaccagcctggtcaacatagtgagacccca 39954
human.FAM230E-LOC105377182-GGT3P.NCBI.                                            atgggggactgcttgaagtcaggagttaagaccagcctggtcaacatagtgagacccca 38695
human.FAM230J-LOC105372942-GGTLC5P.NCBI.rev.compl                                 atgggggactgcttgaagtcaggagttaagaccagcctggtcaacatagtgagacccca 39414
human.FAM230A.ncRNA.18487127.18500594-GGTLC3.18516335.18518165.rev.compl.REF      atgggggactgcttgaagtcaggagttaagaccagcctggtcaacatagtgagacccca 28161
                                                                                  ************************************************************

chimp.LOC112206744-LOC107973052-GGT2.revcompl                                     tctctattggtttaaattttgttt--tttaattaaataaataaaataaggattgaagagt 41325
chimp54546-135457.revcompl.Pan.troglodytes.clone.rp43-41g5.GenBank:AC099533.36     TCTCTATTGGTTTAAATTTTTTTT---TTAATTAAATAAATAAAATAAGGATTGAAGAGT 43129
human.FAM30B-LOC105372935-GGT2.NCBI.                                              tctctattggtttaaattttttttaaaaaaattaaataaataaaataaggattgaagagt 40014
human.FAM230E-LOC105377182-GGT3P.NCBI.                                            tctctattggtttaaattttttttaaaaaaattaaataaataaaataaggattgaagagt 38755
human.FAM230J-LOC105372942-GGTLC5P.NCBI.rev.compl                                 tctctattggtttaaattttttttaaaaaaattaaataaataaaataaggattgaagagt 39474
human.FAM230A.ncRNA.18487127.18500594-GGTLC3.18516335.18518165.rev.compl.REF      tctctattggtttaaattttttttaaaaaaattaaataaataaaataaggattgaagagt 28221
                                                                                  ******************** **      *******************************

chimp.LOC112206744-LOC107973052-GGT2.revcompl                                     gacttgtacaccagttgagcccacctccatctcacccttgcagagccccagagacacagc 41385
chimp54546-135457.revcompl.Pan.troglodytes.clone.rp43-41g5.GenBank:AC099533.36     GACTTGTACACCATTTGAGCCCACCTCCATCTCACCCTTGCAGAGCCCCAGAGACACAGC 43189
human.FAM30B-LOC105372935-GGT2.NCBI.                                              gacttgtacaccagttgagcccacctccatctcacccttgcagagccccagagacacagc 40074
human.FAM230E-LOC105377182-GGT3P.NCBI.                                            gacttgtacaccagttgagcccacctccatctcacccttgcagagccccagagacacagc 38815
human.FAM230J-LOC105372942-GGTLC5P.NCBI.rev.compl                                 gacttgtacaccagttgagcccacctccatctcacccttgcagagccccagagacacagc 39534
human.FAM230A.ncRNA.18487127.18500594-GGTLC3.18516335.18518165.rev.compl.REF      gacttgtacaccagttgagcccacctccatctcacccttgcagagccccagagacacagc 28281
                                                                                  ************* **********************************************

chimp.LOC112206744-LOC107973052-GGT2.revcompl                                     cctccagggctcagacccagtgggacttgactccacaggcataaaccctgtttgtctat 41445
chimp54546-135457.revcompl.Pan.troglodytes.clone.rp43-41g5.GenBank:AC099533.36     CCTCCAGAGCTCAGACCCAGTGGGACTTGACTCCACAGGCATAAAACCCTGTTTGTCTAT 43249
human.FAM30B-LOC105372935-GGT2.NCBI.                                              cctccagagctcagacccagtgggacttgactccacaggcataaaaccctgtttgtctat 40134
human.FAM230E-LOC105377182-GGT3P.NCBI.                                            cctccagagctcagacccagtgggacttgactccacaggcataaaaccctgtttgtctat 38875
human.FAM230J-LOC105372942-GGTLC5P.NCBI.rev.compl                                 cctccagagctcagacccagtgggacttgactccacaggcataaaaccctgtttgtctat 39594
human.FAM230A.ncRNA.18487127.18500594-GGTLC3.18516335.18518165.rev.compl.REF      cctccagagctcagacccagtgggacttgactccacaggcataaaaccctgtttgtctat 28341
                                                                                  ******* ****************************************************

chimp.LOC112206744-LOC107973052-GGT2.revcompl                                     gggccctttggaatcaccaggttttcggggatcctgaaggatagccccgacctggcctca 41505
chimp54546-135457.revcompl.Pan.troglodytes.clone.rp43-41g5.GenBank:AC099533.36     GGGCCCTTTGGAATCACCAGGTTTTCGGGGCTCCTGAAGGATAGCCCCGACCTGGCCTCA 43309
human.FAM30B-LOC105372935-GGT2.NCBI.                                              gggccctttggaatcaccaggttttcggggctcctgaaggatagccccgacctggcctca 40194
human.FAM230E-LOC105377182-GGT3P.NCBI.                                            gggccctttggaatcaccaggttttcggggctcctgaaggatagccccgacctggcctca 38935
human.FAM230J-LOC105372942-GGTLC5P.NCBI.rev.compl                                 gggccctttggaatcaccaggttttcggggctcctgaaggatagccccgacctggcctca 39654
human.FAM230A.ncRNA.18487127.18500594-GGTLC3.18516335.18518165.rev.compl.REF      gggccctttggaatcaccaggttttcggggctcctgaaggatagccccgacctggcctca 28401
                                                                                  ****************************** *****************************

chimp.LOC112206744-LOC107973052-GGT2.revcompl                                     cctggcccctggccccagtgcccctggtgatatccaggtgc------------------- 41546
chimp54546-135457.revcompl.Pan.troglodytes.clone.rp43-41g5.GenBank:AC099533.36     CCTGGCCCCTGGCCCCAGTGCCCCTGGTGATATCCAGGTGC------------------- 43350
human.FAM30B-LOC105372935-GGT2.NCBI.                                              cctggcccctggccccagtgcccctggtgatatccaggtgctgggctgtgatcaccgcct 40254
human.FAM230E-LOC105377182-GGT3P.NCBI.                                            cctggcccctggccccagtgcccctggtgatatccaggtgc------------------- 38976
human.FAM230J-LOC105372942-GGTLC5P.NCBI.rev.compl                                 cctggcccctggccccagtgcccctggtgatatccaggtgc------------------- 39695
human.FAM230A.ncRNA.18487127.18500594-GGTLC3.18516335.18518165.rev.compl.REF      cctggcccctggccccagtgcccctggtgatatccaggtgc------------------- 28442
                                                                                  *****************************************

chimp.LOC112206744-LOC107973052-GGT2.revcompl                                     -------------------------------------------------tgggctgtgat 41557
chimp54546-135457.revcompl.Pan.troglodytes.clone.rp43-41g5.GenBank:AC099533.36     -------------------------------------------------TGGGCTGTGAT 43361
human.FAM30B-LOC105372935-GGT2.NCBI.                                              cccaccagcccacctccaccagcccttcccagaaccctgctccaggtgttgggctgtgat 40314
human.FAM230E-LOC105377182-GGT3P.NCBI.                                            -------------------------------------------------tgggctgtgat 38987
human.FAM230J-LOC105372942-GGTLC5P.NCBI.rev.compl                                 -------------------------------------------------tgggctgtgat 39706
human.FAM230A.ncRNA.18487127.18500594-GGTLC3.18516335.18518165.rev.compl.REF      -------------------------------------------------tgggctgtgat 28453
                                                                                                                                   ***********

chimp.LOC112206744-LOC107973052-GGT2.revcompl                                     ctccgcctcccaccagcccacctccaccagcccttcccagaaccctgccccaggtgttgg 41617
chimp54546-135457.revcompl.Pan.troglodytes.clone.rp43-41g5.GenBank:AC099533.36     CACCGCCTCCCACCAGCCCACCTCCACCAGCCCTTCCCAGAACCCTGCTCCAGGTGTTGG 43421
human.FAM30B-LOC105372935-GGT2.NCBI.                                              caccgcctcccaccagcccacctccaccagcccttcccagaaccctgctccaggtgttgg 40374
human.FAM230E-LOC105377182-GGT3P.NCBI.                                            caccgcctcccaccagcccacctccaccagcccttcccagaaccctgctccaggtgttgg 39047
human.FAM230J-LOC105372942-GGTLC5P.NCBI.rev.compl                                 caccgcctcccaccagcccacctccaccagcccttcccagaaccctgctccaggtgttgg 39766
human.FAM230A.ncRNA.18487127.18500594-GGTLC3.18516335.18518165.rev.compl.REF      caccgcctcccaccagcccacctccaccagcccttcccagaaccctgctccaggtgttgg 28513
                                                                                  * ********************************************** ***********

chimp.LOC112206744-LOC107973052-GGT2.revcompl                                     aactgtgcacagaggagggagcaggccctgagggaggcctggaggggctgcggatggtga 41677
chimp54546-135457.revcompl.Pan.troglodytes.clone.rp43-41g5.GenBank:AC099533.36     AACTGTGCACAGAGGAGGGAGCAGGCCCCGAGGGAGGCCTGGAGGGACTGCGGATGGTGA 43481
human.FAM30B-LOC105372935-GGT2.NCBI.                                              aactgtgcacagaggagggagcaggccccgagggaggcctggaggggctgccaatggtga 40434
human.FAM230E-LOC105377182-GGT3P.NCBI.                                            aactgtgcacagaggagggagcaggccccgagggaggcctggaggggctgccaatggtga 39107
human.FAM230J-LOC105372942-GGTLC5P.NCBI.rev.compl                                 aactgtgcacagaggagggagcaggccccgagggaggcctggaggggctgccgatggtga 39826
human.FAM230A.ncRNA.18487127.18500594-GGTLC3.18516335.18518165.rev.compl.REF      aactgtgcacagaggagggagcaggccccgagggaggcctggaggggctgccaatggtga 28573
                                                                                  **************************** ***************** ****  *******

chimp.LOC112206744-LOC107973052-GGT2.revcompl                                     aggctgctgtgtctagctgtttccttctggacccactccctctgggctgcgtccccggct 41737
chimp54546-135457.revcompl.Pan.troglodytes.clone.rp43-41g5.GenBank:AC099533.36     AGGCTGCTGTGTCTAGCTGTTTCCTTCTGGACCCACTCCCTCTGGGCTGCGTCCCCGGCT 43541
human.FAM30B-LOC105372935-GGT2.NCBI.                                              aggctgctgtgtcgagctgtttccttccggacccactccctctgggctgcgtccccggct 40494
human.FAM230E-LOC105377182-GGT3P.NCBI.                                            aggctgctgtgtcgagctgtttccttccggacccactccctctgggctgcgtccccggct 39167
human.FAM230J-LOC105372942-GGTLC5P.NCBI.rev.compl                                 aggctgctgtgtcgagctgtttccttccggacccactccctctgggctgcgtccccggct 39886
human.FAM230A.ncRNA.18487127.18500594-GGTLC3.18516335.18518165.rev.compl.REF      aggctgctgtgtcgagctgtttccttccggacccactccctctgggctgcgtccccggct 28633
                                                                                  ************* ************* ********************************

chimp.LOC112206744-LOC107973052-GGT2.revcompl                                     ggtccaagccctgatccctgggatctggggacatcttcccgtttgttgttccctgagaac 41797
chimp54546-135457.revcompl.Pan.troglodytes.clone.rp43-41g5.GenBank:AC099533.36     GGTCCAAGCCCTGATCCCTGGGATCTGGGGACATCTTCCCGTTTGTTGTTCCCTGAGAAC 43601
human.FAM30B-LOC105372935-GGT2.NCBI.                                              ggtccaagccctgatccctgggatctggggacatcttcccgtttgctgttccctgagaac 40554
human.FAM230E-LOC105377182-GGT3P.NCBI.                                            ggtccaagccctgatccctgggatctggggacatcttcccgtttgctgttccctgagaac 39227
human.FAM230J-LOC105372942-GGTLC5P.NCBI.rev.compl                                 ggtccaagccctgatccctgggatctggggacatcttcccgtttgctgttccctgagaac 39946
human.FAM230A.ncRNA.18487127.18500594-GGTLC3.18516335.18518165.rev.compl.REF      ggtccaagccctgatccctgggatctggggacatcttcccgtttgctgttccctgagaac 28693
                                                                                  ********************************************* **************

chimp.LOC112206744-LOC107973052-GGT2.revcompl                                     caggcctccctctggagaggatcacaagcttgggtttcactctgggcttgctcttgggaa 41857
chimp54546-135457.revcompl.Pan.troglodytes.clone.rp43-41g5.GenBank:AC099533.36     CAGGCCTCCCTCTGGAGAGGATCACAAGCTTGGGTTTCACTCTGGGCTTGCTCTTGGGAA 43661
human.FAM30B-LOC105372935-GGT2.NCBI.                                              caggcctccctctggagaggatcacaagcttgggtttcactctgggcttgctcttgggaa 40614
human.FAM230E-LOC105377182-GGT3P.NCBI.                                            caggcctccctctggagaggatcacaagcttgggtttcactctgggcttgctcttgggaa 39287
human.FAM230J-LOC105372942-GGTLC5P.NCBI.rev.compl                                 caggcctccctctggagaggatcacaagcttgggtttcactctgggcttgctcttgggaa 40006
human.FAM230A.ncRNA.18487127.18500594-GGTLC3.18516335.18518165.rev.compl.REF      caggcctccctgtggagaggatcacaagcttgggtttcactctgggcttgctcttgggaa 28753
                                                                                  *********** ************************************************

chimp.LOC112206744-LOC107973052-GGT2.revcompl                                     cccccaggggcgtggctctgaccgagatgttttcctccagcctgttgcctagtccccat 41917
chimp54546-135457.revcompl.Pan.troglodytes.clone.rp43-41g5.GenBank:AC099533.36     CCCCCCAGGGGCGTGGCTCTGACCGAGATGTTTTCCTCCAGCCTGTTGCCCAGTCCCCAT 43721
human.FAM30B-LOC105372935-GGT2.NCBI.                                              ccccccaggggcatggctctgaccgagatgttttcctccagcctgttgcccagtccccat 40674
human.FAM230E-LOC105377182-GGT3P.NCBI.                                            ccccccaggggcatggctctgaccgagatgttttcctccagcctgttgcccagtccccat 39347
human.FAM230J-LOC105372942-GGTLC5P.NCBI.rev.compl                                 ccccccaggggcatggctctgaccgagatgttttcctccagcctgttgcccagtccccat 40066
human.FAM230A.ncRNA.18487127.18500594-GGTLC3.18516335.18518165.rev.compl.REF      ccccccaggggcatggctctgaccgagatgttttcctccagcctgttgcccagtccccat 28813
                                                                                  ************ ************************************* *********

chimp.LOC112206744-LOC107973052-GGT2.revcompl                                     tcctcgga-cctcagcttcacctccagtgtcatcggcagggtgagctggacgcctacggg 41976
chimp54546-135457.revcompl.Pan.troglodytes.clone.rp43-41g5.GenBank:AC099533.36     TCCTCGGA-CCTCAGCTTCACCTCCAGTGTCATCGGCAGGGTGAGCTGGACGCCTACGGG 43780
human.FAM30B-LOC105372935-GGT2.NCBI.                                              tcctcggaccctcagcttcacctccagtgtcatcggcagggtgagctggacgcctacggg 40734
human.FAM230E-LOC105377182-GGT3P.NCBI.                                            tcctcggaccctcagcttcacctccagtgtcatcggcagggtgagctggacgcctacggg 39407
human.FAM230J-LOC105372942-GGTLC5P.NCBI.rev.compl                                 tcctcggaccctcagcttcacctccagtgtcatcggcagggtgagctggacgcctacggg 40126
human.FAM230A.ncRNA.18487127.18500594-GGTLC3.18516335.18518165.rev.compl.REF      tcctcggaccctcagcttcacctccagtgtcatcggcagggtgagctggacgcctacggg 28873
                                                                                  ******** ***************************************************

chimp.LOC112206744-LOC107973052-GGT2.revcompl                                     tctgagaaggcacccgggttcccagcatcagctggccacc-------------------- 42016
chimp54546-135457.revcompl.Pan.troglodytes.clone.rp43-41g5.GenBank:AC099533.36     TCTGAGAAGGCGCCCGGGTTCCCAGCATCAGCTGGCCACCCTCTGCCTAAGAAAGTGCCA 43840
human.FAM30B-LOC105372935-GGT2.NCBI.                                              tctgagaaggcgcccgggttcccagcatcggctggccaccctctgcctaagaaagcgcca 40794
human.FAM230E-LOC105377182-GGT3P.NCBI.                                            tctgagaaggcgcccgggttcccagcatcggctggccaccctctgcctaagaaagcgcca 39467
human.FAM230J-LOC105372942-GGTLC5P.NCBI.rev.compl                                 tctgagaaggcgcccgggttcccagcatcggctggccaccctctgcctaagaaagcgcca 40186
human.FAM230A.ncRNA.18487127.18500594-GGTLC3.18516335.18518165.rev.compl.REF      tctgagaaggcgcccgggttcccagcatcggctggccaccctctgcctaagaaagcgcca 28933
                                                                                  *********** ***************** **********

chimp.LOC112206744-LOC107973052-GGT2.revcompl                                     ------------------------------------------------------------ 42016
chimp54546-135457.revcompl.Pan.troglodytes.clone.rp43-41g5.GenBank:AC099533.36     GGGTCGTGGCACCCCCTGGTGGCTGATCCTAGGTAGTGTCACTGCCCAGCCCCAGTAAGG 43900
human.FAM30B-LOC105372935-GGT2.NCBI.                                              gggtcgtgacacccccctggtggctgatcctaggtagtgtcactgcccagccccagtaagg 40854
human.FAM230E-LOC105377182-GGT3P.NCBI.                                            gggtcgtgacacccccctggtggctgatcctaggtagtgtcactgcccagccccagtaagg 39527
human.FAM230J-LOC105372942-GGTLC5P.NCBI.rev.compl                                 gggtcgtgacacccccctggtggctgatcctaggtagtgtcactgcccagccccagtaagg 40246
human.FAM230A.ncRNA.18487127.18500594-GGTLC3.18516335.18518165.rev.compl.REF      gggtcgtgacacccccctggtggctgatcctaggtagtgtcactgcccagccccagtaagg 28993

chimp.LOC112206744-LOC107973052-GGT2.revcompl                                     ------------------------------------------------------------ 42016
chimp54546-135457.revcompl.Pan.troglodytes.clone.rp43-41g5.GenBank:AC099533.36     GAGGGCCTGGCCCCAAAGTCCGAGGGATCAGGGTGGAAAGGGGCAGGCTTGGTGTGAAT 43960
human.FAM30B-LOC105372935-GGT2.NCBI.                                              gagggcctggccccaaagtctgagggatcagggtgggaaggggcagggtttggtgtgaac 40914
human.FAM230E-LOC105377182-GGT3P.NCBI.                                            gagggcctggccccaaagtctgagggatcagggtgggaaggggcagggtttggtgtgaac 39587
human.FAM230J-LOC105372942-GGTLC5P.NCBI.rev.compl                                 gagggcctggccccaaagtctgagggatcagggtgggaaggggcagggtttggtgtgaac 40306
human.FAM230A.ncRNA.18487127.18500594-GGTLC3.18516335.18518165.rev.compl.REF      gagggcctggccccaaagtctgagggatcagggtgggaaggggcagggtttggtgtgaac 29053

chimp.LOC112206744-LOC107973052-GGT2.revcompl                                     ------------------------------------------------------------ 42016
chimp54546-135457.revcompl.Pan.troglodytes.clone.rp43-41g5.GenBank:AC099533.36     CTTCCCCTGGCCCCCAGCCATGTGCCTGGCTCTCCCCATGCTGAAGATGCTGAGGCTAGT 44020
human.FAM30B-LOC105372935-GGT2.NCBI.                                              cttcccctggcccccagccatgtgccttgctctccccatgctgaagatgctgaggctagt 40974
```

```
human.FAM230E-LOC105377182-GGT3P.NCBI.                                          cttcccctggcccccagccatgtgccttgctctccccatgctgaagatgctgaggctagt 39647
human.FAM230J-LOC105372942-GGTLC5P.NCBI.rev.compl                               cttcccctggcccccagccatgtgcctggctctccccatgctgaagatgctgaggctagt 40366
human.FAM230A.ncRNA.18487127.18500594-GGTLC3.18516335.18518165.rev.compl.REF    cttcccctggcccccagccatgtgccttgctctccccatgctgaagatgctgaggctagt 29113

chimp.LOC112206744-LOC107973052-GGT2.revcompl                                   --------------------------------------ctctgttcctccccagccagat 42038
chimp54546-135457.revcompl.Pan.troglodytes.clone.rp43-41g5.GenBank:AC099533.36  TCCAGTGTCTGCATTGTGAAGATCTCCGAATCCCACCTCTCTGTTCCTCCCCAGCCAGAT 44080
human.FAM30B-LOC105372935-GGT2.NCBI.                                            tccagtgtccgcattgtgaagatctccgaatcccacctctctgttcctccccagccagat 41034
human.FAM230E-LOC105377182-GGT3P.NCBI.                                          tccagtgcccgcattgtgaagatctccgaatcccacctctctgttcctccccagccagat 39707
human.FAM230J-LOC105372942-GGTLC5P.NCBI.rev.compl                               tccagtgtccgcattgtgaagatctccgaatcccacctctctgttcctccccagccagat 40426
human.FAM230A.ncRNA.18487127.18500594-GGTLC3.18516335.18518165.rev.compl.REF    tccagtgcccgcattgtgaagatctccgaatcccacctctctgttcctccccagccagat 29173
                                                                                                                      **********************

chimp.LOC112206744-LOC107973052-GGT2.revcompl                                   ggctccatttcacacacaatacactgaggcccagagagtggggagacaggccagggaggc 42098
chimp54546-135457.revcompl.Pan.troglodytes.clone.rp43-41g5.GenBank:AC099533.36  GGCTCCATTTCACACACAATACACTGAGGCCCAGAGAGTGGGGAGACAGGCCAGGGAGGC 44140
human.FAM30B-LOC105372935-GGT2.NCBI.                                            ggctccatttcacacacaatacactgaggcccagagagtggggagacaggccagggaggc 41094
human.FAM230E-LOC105377182-GGT3P.NCBI.                                          ggctccatttcacacacaatacactgaggcccagagagtggggagacaggccagggaggc 39767
human.FAM230J-LOC105372942-GGTLC5P.NCBI.rev.compl                               ggctccatttcacacacaaaacactgaggcccagagagtggggagacaggccagggaggc 40486
human.FAM230A.ncRNA.18487127.18500594-GGTLC3.18516335.18518165.rev.compl.REF    ggctccatttcacacacaatacactgaggcccagagagtggggagacaggccagggaggc 29233
                                                                                ******************* ****************************************

chimp.LOC112206744-LOC107973052-GGT2.revcompl                                   cacctggagcctggcacagtggcctcatttattatgctgctctgctgctcacaggggaag 42158
chimp54546-135457.revcompl.Pan.troglodytes.clone.rp43-41g5.GenBank:AC099533.36  CACCTGGAGCCTGGCACAGTGGCCTCATTTATTGTGCTGCTCTGCTGCTCACAGGGGAAG 44200
human.FAM30B-LOC105372935-GGT2.NCBI.                                            cacctggagcctggcacagtggcctcatttattatgctgctctgctgctcacaggggaag 41154
human.FAM230E-LOC105377182-GGT3P.NCBI.                                          cacctggagcctggcacagtggcctcatttattatgctgctctgctgctcacaggggaag 39827
human.FAM230J-LOC105372942-GGTLC5P.NCBI.rev.compl                               cacctggagcctggcacagtggcctcatttattatgctgctctgctgctcacaggggaag 40546
human.FAM230A.ncRNA.18487127.18500594-GGTLC3.18516335.18518165.rev.compl.REF    cacctggagcctggcacagtggcctcatttattatgctgctctgctgctcacaggggaag 29293
                                                                                ********************************* **************************

chimp.LOC112206744-LOC107973052-GGT2.revcompl                                   cccgtcccccaaagtcctcttcctcatcctggtgagtatcttgtccctggattgcttgtc 42218
chimp54546-135457.revcompl.Pan.troglodytes.clone.rp43-41g5.GenBank:AC099533.36  CTGGTCCCCCGGAGTCCCCTTCCTGGTCCTGGTGAGTATCTTGTCCCTGGATTGCTTGTC 44260
human.FAM30B-LOC105372935-GGT2.NCBI.                                            cccgtcccccaaagtcctcttcctcatcctggtgagtatcttgtccctggattgcttgtc 41214
human.FAM230E-LOC105377182-GGT3P.NCBI.                                          cccgtcccccaaagtcctcttcctcatcctggtgagtatcttgtccctggattgcttgtc 39887
human.FAM230J-LOC105372942-GGTLC5P.NCBI.rev.compl                               cccgtcccccaaagtcctcttcctcatcctggtgagtatcttgtccctggattgcttgtc 40606
human.FAM230A.ncRNA.18487127.18500594-GGTLC3.18516335.18518165.rev.compl.REF    cccgtcccccaaagtcctcttcctcatcctggtgagtatcttgtccctggattgcttgtc 29353
                                                                                *  *******  ***** ******  **********************************

chimp.LOC112206744-LOC107973052-GGT2.revcompl                                   agccttgtctgcctggagcaatcagtagccagcaggttccccgcctttcctggagtccga 42278
chimp54546-135457.revcompl.Pan.troglodytes.clone.rp43-41g5.GenBank:AC099533.36  AGCCTTGTCCGCCTGGAGCACTCAGTAGCCGGCAGGTTCCCCGCCTTTCCTGGAGTCCGA 44320
human.FAM30B-LOC105372935-GGT2.NCBI.                                            agccttgtctgcctggagcaatcagtagccagcaggttccccgcctttcctggagtccga 41274
human.FAM230E-LOC105377182-GGT3P.NCBI.                                          agccttgtctgcctggagcactcagtagccagcaggttccccgcctttcctggagtccga 39947
human.FAM230J-LOC105372942-GGTLC5P.NCBI.rev.compl                               agccttgtctgcctggagcactcagtagccagcaggttccccgcctttcctggagtccga 40666
human.FAM230A.ncRNA.18487127.18500594-GGTLC3.18516335.18518165.rev.compl.REF    agccttgtctgcctggagcaatcagtagccagcaggttccccgcctttcctggagtccga 29413
                                                                                ********* ********** ********* *****************************

chimp.LOC112206744-LOC107973052-GGT2.revcompl                                   ggcagctgcccagccaccagccatgcggacgatggcttgcaccacagcgatgaaggtgga 42338
chimp54546-135457.revcompl.Pan.troglodytes.clone.rp43-41g5.GenBank:AC099533.36  GGCAGCTGCCCAGCCACCAGCCATGCGGACGATGGCTTGCACCACAGCGATGAAGGTGGA 44380
human.FAM30B-LOC105372935-GGT2.NCBI.                                            ggcagctgcccagccaccagccgtgcggacgatggcttgcaccacagcgatgaaggtgga 41334
human.FAM230E-LOC105377182-GGT3P.NCBI.                                          ggcagctgcccagccaccagccgtgcggacgatggcttgcaccacagcgatgaaggtgga 40007
human.FAM230J-LOC105372942-GGTLC5P.NCBI.rev.compl                               ggcagctgcccagccaccagccgtgcggacgatggcttgcaccacagcgatgaaggtgga 40726
human.FAM230A.ncRNA.18487127.18500594-GGTLC3.18516335.18518165.rev.compl.REF    ggcagctgcccagccaccagccgtgcggacgatggcttgcaccacagcgatgaaggtgga 29473
                                                                                ********************** *************************************

chimp.LOC112206744-LOC107973052-GGT2.revcompl                                   cgcgatctgggtgtgatggtgccgggtctccagggctgcagtcactgcctgggggtgga 42398
chimp54546-135457.revcompl.Pan.troglodytes.clone.rp43-41g5.GenBank:AC099533.36  CGCGATCTGGGTGTGATGGTGCCGGGTCTCCAGGGCTGCAGTCACTGCCTGGGGGTGGA 44440
human.FAM30B-LOC105372935-GGT2.NCBI.                                            cgcgatctgggtgtgatggtgccgggtctccagggctgcagtcactgcctgggggtgga 41394
human.FAM230E-LOC105377182-GGT3P.NCBI.                                          cgcgatctgggtgtgatggtgccgggtctccagggctgcagtcactgcctgggggtgga 40067
human.FAM230J-LOC105372942-GGTLC5P.NCBI.rev.compl                               cgcgatctgggtgtgatggtgccgggtctccagggctgcagtcactgcctgggggtgga 40786
human.FAM230A.ncRNA.18487127.18500594-GGTLC3.18516335.18518165.rev.compl.REF    tgcgatctgggtgtgatggtgccgggtctccagggctgcagtcactgcctgggggtgga 29533
                                                                                 ***********************************************************

chimp.LOC112206744-LOC107973052-GGT2.revcompl                                   ggagaggggaagcctgagcagggctccagatgccacctgaaccacgcctgtgtggtcaca 42458
chimp54546-135457.revcompl.Pan.troglodytes.clone.rp43-41g5.GenBank:AC099533.36  GGAGAGGGGAAACCTGAGCAAGGCTCCAGATGCCACCTGAACCACACCTGTGTGGTCACA 44500
human.FAM30B-LOC105372935-GGT2.NCBI.                                            ggagaggggaagcctgagcagggctccagatgccacctgaaccacacctgtgtggtcaca 41454
human.FAM230E-LOC105377182-GGT3P.NCBI.                                          ggagaggggaagcctgagcagggctccagatgccacctgaaccacacctgtgtggtcaca 40127
human.FAM230J-LOC105372942-GGTLC5P.NCBI.rev.compl                               ggagaggggaagcctgagcagggctccagatgccacctgaaccacacctgtgtggtcaca 40846
human.FAM230A.ncRNA.18487127.18500594-GGTLC3.18516335.18518165.rev.compl.REF    ggagaggggaagcctgagcagggctccagatgccacctgaaccacacctgtgtggtcaca 29593
                                                                                *********** ******** ************************ **************

chimp.LOC112206744-LOC107973052-GGT2.revcompl                                   ggcctcagcccaggtggtgccatttcaggccaggtcatcaggaagagcaggttggggcct 42518
chimp54546-135457.revcompl.Pan.troglodytes.clone.rp43-41g5.GenBank:AC099533.36  GGCCTCAGCCCAGGTGGTGCCATTTCGGGCCAGGTCATCAGGAAGAGCAGGTTGGGGCCT 44560
human.FAM30B-LOC105372935-GGT2.NCBI.                                            ggcctcagcccaggtggtgccatttcaggccaggtcatcaggaagagcaggttggggcct 41514
human.FAM230E-LOC105377182-GGT3P.NCBI.                                          ggcctcagcccaggtggtgccatttcaggccaggtcatcaggaagagcaggttggggcct 40187
human.FAM230J-LOC105372942-GGTLC5P.NCBI.rev.compl                               ggcctcagcccaggtggtgccatttcaggccaggtcatcaggaagagcaggttggggcct 40906
human.FAM230A.ncRNA.18487127.18500594-GGTLC3.18516335.18518165.rev.compl.REF    ggcctcagcccaggtggtgccatttcaggccaggtcatcaggaagagcaggttggggcct 29653
                                                                                ************************** *********************************

chimp.LOC112206744-LOC107973052-GGT2.revcompl                                   gctgggtctcactggagcagggggcttggccctcatggcacaggggctccagatggccca 42578
chimp54546-135457.revcompl.Pan.troglodytes.clone.rp43-41g5.GenBank:AC099533.36  GCTGGGTCTCACTGGAGCAGGGGGCTTGGCCCTCATGGCACAGGGGCTCCAGATGGCCCA 44620
human.FAM30B-LOC105372935-GGT2.NCBI.                                            gctgggtctcactggagcagggggcttggctctcatgtcacaggggctccagatggccca 41574
human.FAM230E-LOC105377182-GGT3P.NCBI.                                          gctgggtctcactggagcagggggcttggccctcatgtcacaggggctccagatggccca 40247
human.FAM230J-LOC105372942-GGTLC5P.NCBI.rev.compl                               gctgggtctcactggagcagggggcttggccctcatgtcacaggggctccagatggccca 40966
human.FAM230A.ncRNA.18487127.18500594-GGTLC3.18516335.18518165.rev.compl.REF    gctgggtctcactggagcagggggcttggctctcatgtcacaggggctccagatggccca 29713
                                                                                ****************************** ****** **********************

chimp.LOC112206744-LOC107973052-GGT2.revcompl                                   ggcactagagagaggacaccaaccattgtccactctgtgatgatccaggcctccagccca 42638
chimp54546-135457.revcompl.Pan.troglodytes.clone.rp43-41g5.GenBank:AC099533.36  GGCACTAGAGAGAGGACACCAACCATTGTCCACTCTGTGATGATCCAGGCCTCCAGCCCA 44680
human.FAM30B-LOC105372935-GGT2.NCBI.                                            ggcactagagagaggacaccaaccattgtccactctgtgatgatccaggcctccagccca 41634
human.FAM230E-LOC105377182-GGT3P.NCBI.                                          ggcactagagagaggacaccaaccattgtccactctgtgatgatccaggcctccagccca 40307
human.FAM230J-LOC105372942-GGTLC5P.NCBI.rev.compl                               ggcactagagagaggacaccaaccattgtccactctgtgatgatccaggcctccagccca 41026
human.FAM230A.ncRNA.18487127.18500594-GGTLC3.18516335.18518165.rev.compl.REF    ggcactagagagaggacaccaaccattgtccactctgtgatgatccaggcctccagccca 29773
                                                                                ************************************************************

chimp.LOC112206744-LOC107973052-GGT2.revcompl                                   ggatgccctggggccccacaccgtgactcagtttctccaacccccggcccacctggtcaa 42698
chimp54546-135457.revcompl.Pan.troglodytes.clone.rp43-41g5.GenBank:AC099533.36  GGATGCCCTGGGGCCCCACACCGTGACTCAGTTTCTCCAACCCCCGGCCCACCTGGTCAA 44740
human.FAM30B-LOC105372935-GGT2.NCBI.                                            ggatgccctgggaccccacaccgtgactcagtttctccaacccctggcccacctggtcaa 41694
human.FAM230E-LOC105377182-GGT3P.NCBI.                                          ggatgccctgggaccccacaccgtgactcagtttctccaacccccggcccacctggtcaa 40367
human.FAM230J-LOC105372942-GGTLC5P.NCBI.rev.compl                               ggatgccctgggaccccacaccgtgactcagtttctccaacccccggcccacctggtcaa 41086
human.FAM230A.ncRNA.18487127.18500594-GGTLC3.18516335.18518165.rev.compl.REF    ggatgccctgggaccccacaccgtgactcagtttctccaacccctggcccacctggtcaa 29833
                                                                                ************ ******************************* ***************

chimp.LOC112206744-LOC107973052-GGT2.revcompl                                   tgtttctctccactgtcgtgacgttgggcagaagcttgttgtgcagccggggctcctcca 42758
chimp54546-135457.revcompl.Pan.troglodytes.clone.rp43-41g5.GenBank:AC099533.36  TGTTTCTCTCCACTGTCGTGACGTTGGGCAGAAGCTGGTTGTGCAGCCGGGGCTCCTCCA 44800
human.FAM30B-LOC105372935-GGT2.NCBI.                                            tgtttctctccactgtcgtgacgttgggcagaagcttgttgtgcagccggggctcctcca 41754
human.FAM230E-LOC105377182-GGT3P.NCBI.                                          tgtttctctccactgtcgtgacgttgggcagaagcttgttgtgcagccggggctcctcca 40427
human.FAM230J-LOC105372942-GGTLC5P.NCBI.rev.compl                               tgtttctctccactgtcgtgacgttgggcagaagcttgttgtgcagccggggctcctcca 41146
human.FAM230A.ncRNA.18487127.18500594-GGTLC3.18516335.18518165.rev.compl.REF    tgtttctctccactgtcgtgacgttgggcagaagcttgttgtgcagccggggctcctcca 29893
                                                                                ************************************ ***********************

chimp.LOC112206744-LOC107973052-GGT2.revcompl                                   cggccttcttcacgtcatagccgaaccagaggttgtagatgatggcctggggcatgggag 42818
chimp54546-135457.revcompl.Pan.troglodytes.clone.rp43-41g5.GenBank:AC099533.36  CGGCCCGCTTCACGTCATAGCCGAACCAGAGGTTGTAGATGATGGCCTGGGGCATGGGAG 44860
human.FAM30B-LOC105372935-GGT2.NCBI.                                            cggccctcttcacgtcatagccgaaccagaggttgtagatgatggcctggggcatgggag 41814
human.FAM230E-LOC105377182-GGT3P.NCBI.                                          cggccctcttcacgtcatagccgaaccagaggttgtagatgatggcctggggcatgggag 40487
human.FAM230J-LOC105372942-GGTLC5P.NCBI.rev.compl                               cggccctcttcacgtcatagccgaaccagaggttgtagatgatggcctggggcatgggag 41206
human.FAM230A.ncRNA.18487127.18500594-GGTLC3.18516335.18518165.rev.compl.REF    cggccctcttcacgtcatagccgaaccagaggttgtagatgatggcctggggcatgggag 29953
                                                                                *****  *****************************************************

chimp.LOC112206744-LOC107973052-GGT2.revcompl                                   tgtgatcagcgtggcttgggggctgtgcagagtgggcagggccagggagaaaaggggtga 42878
chimp54546-135457.revcompl.Pan.troglodytes.clone.rp43-41g5.GenBank:AC099533.36  TGTGATCAGCGTGGCTTGGGGGCTGTGGAGGGTGGGCACGGCCAGGGAGAAAAGGTGTGA 44920
human.FAM30B-LOC105372935-GGT2.NCBI.                                            tgtgatcagggtggcttgggggctgtgcagagtgggcagggccagggagaaaaggggtga 41874
human.FAM230E-LOC105377182-GGT3P.NCBI.                                          tgtgatcagcgtggcttgggggctgtgcagagtgggcagggccagggagaaaaggggtga 40547
human.FAM230J-LOC105372942-GGTLC5P.NCBI.rev.compl                               tgtgatcagcgtggcttgggggctgtgcagagtgggcagggccagggagaaaaggggtga 41266
human.FAM230A.ncRNA.18487127.18500594-GGTLC3.18516335.18518165.rev.compl.REF    tgtgatcagcgtggcttgggggctgtgcagagtgggcagggccagggagaaaaggggtga 30013
                                                                                ********* ***************** ** ******* **************** ****

chimp.LOC112206744-LOC107973052-GGT2.revcompl                                   cacataccagtgcagtgtctgtggtgatctgcgtgcccccagcagctcccaccaccaacc 42938
chimp54546-135457.revcompl.Pan.troglodytes.clone.rp43-41g5.GenBank:AC099533.36  CACATACCAGTGCAGTGGCTGTGGTGATCTGTGTGCCCCCAGCAGCTCCCACAACCATCT 44980
human.FAM30B-LOC105372935-GGT2.NCBI.                                            cacataccagtgcagtgtctgtggtgatctgcgtgcccccagcagctcccaccaccatcc 41934
human.FAM230E-LOC105377182-GGT3P.NCBI.                                          cacataccagtgcagtgtctgtggtgatctgcgtgcccccagcagctcccaccaccatcc 40607
human.FAM230J-LOC105372942-GGTLC5P.NCBI.rev.compl                               cacataccagtgcagtgtctgtggtgatctgcgtgcccccagcagctcccaccaccatcc 41326
human.FAM230A.ncRNA.18487127.18500594-GGTLC3.18516335.18518165.rev.compl.REF    cacataccagtgcagtgtctgtggtgatctgcgtgcccccagcagctcccaccaccatcc 30073
                                                                                ***************** ************* ******************** **** *

chimp.LOC112206744-LOC107973052-GGT2.revcompl                                   ggacctggccgtcctggcccaccatgatcgtcgggcacatggacgagagcggctgcttcc 42998
chimp54546-135457.revcompl.Pan.troglodytes.clone.rp43-41g5.GenBank:AC099533.36  GGACCTGGCCGTCCTGGCCCACCATGATCGTCGGGCACATGGACGAGAGCGGCTGCTTCC 45040
human.FAM30B-LOC105372935-GGT2.NCBI.                                            ggacctggccgtcctggcccaccatgatcgtcaggcacatggacaagagcggctgcttcc 41994
human.FAM230E-LOC105377182-GGT3P.NCBI.                                          ggacctggccgtcctggcccaccatgatcgtcgggcacatggacaagagcggctgcttcc 40667
human.FAM230J-LOC105372942-GGTLC5P.NCBI.rev.compl                               ggacctggccgtcctggcccaccatgatcgtcgggcacatggacaagagcggctgcttcc 41386
human.FAM230A.ncRNA.18487127.18500594-GGTLC3.18516335.18518165.rev.compl.REF    ggacctggccgtcctggcccaccatgatcgtcgggcacatggacaagagcggctgcttcc 30133
                                                                                ******************************** *********** ***************

chimp.LOC112206744-LOC107973052-GGT2.revcompl                                   ctgcggctgatgggagaagacagggatgcccgtcagctgcctgcccaggacacccgcccc 43058
chimp54546-135457.revcompl.Pan.troglodytes.clone.rp43-41g5.GenBank:AC099533.36  CTGTGGCCGATGGGAGAAGACAGGGATGCCCGTCAGCTGCCTGCCCAGGACACCCGCCCC 45100
human.FAM30B-LOC105372935-GGT2.NCBI.                                            ctgcggccgatgggagaagacagggatgcccgtcagctgcctgcccaggacacccgcccc 42054
human.FAM230E-LOC105377182-GGT3P.NCBI.                                          ctgcggccgatgggagaagacagggatgcccgtcagctgcctgcccaggacacccgcccc 40727
```

```
human.FAM230J-LOC105372942-GGTLC5P.NCBI.rev.compl                               ctgcggccgatgggagaagacagggatgcccgtcagctgcctgcccaggacacccgcccc 41446
human.FAM230A.ncRNA.18487127.18500594-GGTLC3.18516335.18518165.rev.compl.REF    ctgcggccgatgggagaagacagggatgcccgtcagctgcctgcccaggacacccgcccc 30193
                                                                                *** *** ****************************************************

chimp.LOC112206744-LOC107973052-GGT2.revcompl                                   tctccaccccagtccccca-cccccagacctccaccccatacctggctggatgaaattgg 43117
chimp54546-135457.revcompl.Pan.troglodytes.clone.rp43-41g5.GenBank:AC099533.36  TCTCCACCCCAGTCCCCCA-TCCCCGGACCCCCACCCCATACCTGGCTGGATGAAATTGG 45159
human.FAM30B-LOC105372935-GGT2.NCBI.                                            tctccaccccagtcccccacccccccggacctccaccccatacctggctggatgaaattgg 42114
human.FAM230E-LOC105377182-GGT3P.NCBI.                                          tctccaccccagtcccccacccccccggacctccaccccatacctggctggatgaaattgg 40787
human.FAM230J-LOC105372942-GGTLC5P.NCBI.rev.compl                               tctccaccccagtcccccacccccccggacctccaccccatacctggctggatgaaattgg 41506
human.FAM230A.ncRNA.18487127.18500594-GGTLC3.18516335.18518165.rev.compl.REF    tctccaccccagtcccccacccccccagacctccaccccatacctggctggatgaaattgg 30253
                                                                                *******************  **** **** *****************************

chimp.LOC112206744-LOC107973052-GGT2.revcompl                                   caggtgaggggggcaccccaaactcattggtg-atgctgggagagctgaagtcgtcca-t 43175
chimp54546-135457.revcompl.Pan.troglodytes.clone.rp43-41g5.GenBank:AC099533.36  CAGGTGAGGGGGGCACCCCAAACTCGTTGGTG-ATGTTGGGAGAGCTGAAGTCGTCCATT 45218
human.FAM30B-LOC105372935-GGT2.NCBI.                                            caggtgaggggggtgccccaaactcattggtgaatgctgggagagctgaagtcgtcca-t 42173
human.FAM230E-LOC105377182-GGT3P.NCBI.                                          caggtgaggggggtgccccaaactcattggtg-atgctgggagagctgaagtcgtcca-t 40845
human.FAM230J-LOC105372942-GGTLC5P.NCBI.rev.compl                               caggtgaggggggtgccccaaactcattggtg-atgctgggagagctgaagtcgtcca-t 41564
human.FAM230A.ncRNA.18487127.18500594-GGTLC3.18516335.18518165.rev.compl.REF    caggtgaggggggtgccccaaactcattggtgaatgctgggagagctgaagtcgtcca-t 30312
                                                                                *************  ********** ****** *** ********************* *

chimp.LOC112206744-LOC107973052-GGT2.revcompl                                   tcattattgaacaggatcccactgaccggggagcagaccttggagccaaagctaccgccc 43235
chimp54546-135457.revcompl.Pan.troglodytes.clone.rp43-41g5.GenBank:AC099533.36  TCATCATTGAACAGGATCCCGCTGACCGGGCAGCGGACCTTGGAGCCAAAGCTACCGCCC 45278
human.FAM30B-LOC105372935-GGT2.NCBI.                                            tcattattgaacaggatcccactgaccggggagcagaccttggagccaaagctaccgccc 42233
human.FAM230E-LOC105377182-GGT3P.NCBI.                                          tcattattgaacaggatcccactgaccggggagcagaccttggagccaaagctaccgccc 40905
human.FAM230J-LOC105372942-GGTLC5P.NCBI.rev.compl                               tcattattgaacaggatcccactgaccggggagcagaccttggagccaaagctaccgccc 41624
human.FAM230A.ncRNA.18487127.18500594-GGTLC3.18516335.18518165.rev.compl.REF    tcattattgaacaggatcccactgaccggggagcagaccttggagccaaagctaccgccc 30372
                                                                                **** *************** ********* *** *************************

chimp.LOC112206744-LOC107973052-GGT2.revcompl                                   agccaggtcagacagcacctgaccttgcctggcccagcctggtccctatccacccactga 43295
chimp54546-135457.revcompl.Pan.troglodytes.clone.rp43-41g5.GenBank:AC099533.36  AGCCAGGTTAGACAGTGCCCGACCTTGCCTGGCCCAGCCTGGTCCCTATCCACCCACTGA 45338
human.FAM30B-LOC105372935-GGT2.NCBI.                                            agccaggtcagacagcacccgaccttgcctggcccagcctggtccctatccacccactga 42293
human.FAM230E-LOC105377182-GGT3P.NCBI.                                          agccaggtcagacagcacccgaccttgcctggcccagcctggtccctatccacccactga 40965
human.FAM230J-LOC105372942-GGTLC5P.NCBI.rev.compl                               agccaggtcagacagcacccgaccttgcctggcccagcctggtccctatccacccactga 41684
human.FAM230A.ncRNA.18487127.18500594-GGTLC3.18516335.18518165.rev.compl.REF    agccaggtcagacagcacccgaccttgcctggcccagcctggtccctatccacccactga 30432
                                                                                ******** ******  ** ****************************************

chimp.LOC112206744-LOC107973052-GGT2.revcompl                                   ggctcaaacatactcactgagaggcccaggataagctaccaaggttgggcctcagtttcc 43355
chimp54546-135457.revcompl.Pan.troglodytes.clone.rp43-41g5.GenBank:AC099533.36  GGCTCAAACATACTCACTGAGAGGCCCAGGATAAGCTACCAAGTTTGGGCCTCAGTTTCC 45398
human.FAM30B-LOC105372935-GGT2.NCBI.                                            ggctcaaacatactcactgagaggcccaggataagctaccaaggttgggcctcagtttcc 42353
human.FAM230E-LOC105377182-GGT3P.NCBI.                                          ggctcaaacatactcactgagaggcccaggataagctaccaaggttgggcctcagtttcc 41025
human.FAM230J-LOC105372942-GGTLC5P.NCBI.rev.compl                               ggctcaaacatactcactgagaggcccaggataagctaccaaggttgggcctcagtttcc 41744
human.FAM230A.ncRNA.18487127.18500594-GGTLC3.18516335.18518165.rev.compl.REF    ggctcaaacatactcactgagaggcccaggataagctaccaaggttgggcctcagtttcc 30492
                                                                                ******************************************* ****************

chimp.LOC112206744-LOC107973052-GGT2.revcompl                                   caccaggaaaagaggtgatggagccaccttactggataagtgggcagtccctgggccacc 43415
chimp54546-135457.revcompl.Pan.troglodytes.clone.rp43-41g5.GenBank:AC099533.36  CACCAGGAAAAGAGGTGATGGAGCCACCTTACTGGATAAGTGGGCAGTCCCTGGGCCACC 45458
human.FAM30B-LOC105372935-GGT2.NCBI.                                            caccaggaaaagaggtgatggagccaccttactggataagtgggcagtccctgggccacc 42413
human.FAM230E-LOC105377182-GGT3P.NCBI.                                          caccaggaaaagaggtgatggagccaccttactggataagtgggcagtccctgggccacc 41085
human.FAM230J-LOC105372942-GGTLC5P.NCBI.rev.compl                               caccaggaaaagaggtgatggagccaccttactggataagtgggcagtccctgggccacc 41804
human.FAM230A.ncRNA.18487127.18500594-GGTLC3.18516335.18518165.rev.compl.REF    caccaggaaaagaggtgatggagccaccttactggataagtgggcagtccctgggccacc 30552
                                                                                ************************************************************

chimp.LOC112206744-LOC107973052-GGT2.revcompl                                   cgcccctggccctttcccacccaggcggcccagcagcccctactagaggttgatggtgct 43475
chimp54546-135457.revcompl.Pan.troglodytes.clone.rp43-41g5.GenBank:AC099533.36  CGCCCCTGGCCCTTTCCCACCCAGGCAGCCCAGCAGCCCCTACTAGAGGTTCATGGTGCT 45518
human.FAM30B-LOC105372935-GGT2.NCBI.                                            cgcccctggccctttcccacccaggcggcccagcagcccctactagaggttgatggtgct 42473
human.FAM230E-LOC105377182-GGT3P.NCBI.                                          cgcccctggccctttcccacccaggcggcccagcagcccctactagaggttgatggtgct 41145
human.FAM230J-LOC105372942-GGTLC5P.NCBI.rev.compl                               cgcccctggccctttcccacccaggcggcccagcagcccctactagaggttgatggtgct 41864
human.FAM230A.ncRNA.18487127.18500594-GGTLC3.18516335.18518165.rev.compl.REF    cgcccctggccctttcccacccaggcggcccagcagcccctactagaggttgatggtgct 30612
                                                                                ************************** ************************ ********

chimp.LOC112206744-LOC107973052-GGT2.revcompl                                   ggtggccgacacagcactgccgtcctctgcgacgacagacaggtgagcagtgcccccgtc 43535
chimp54546-135457.revcompl.Pan.troglodytes.clone.rp43-41g5.GenBank:AC099533.36  GGTGGCAGACACAGCACTGCTGTCCTCTGCGACGACAGACAGGTGAGCAGTGCCCCCATC 45578
human.FAM30B-LOC105372935-GGT2.NCBI.                                            ggtggcggacacagcactgccgtcctctgcgacgacagacaggtgagcagtgcccccgtc 42533
human.FAM230E-LOC105377182-GGT3P.NCBI.                                          ggtggcggacacagcactgccgtcctctgcgacgacagacaggtgagcagtgcccccgtc 41205
human.FAM230J-LOC105372942-GGTLC5P.NCBI.rev.compl                               ggtggcggacacagcactgccgtcctctgcgacgacagacaggtgagcagtgcccccgtc 41924
human.FAM230A.ncRNA.18487127.18500594-GGTLC3.18516335.18518165.rev.compl.REF    ggtggcggacacagcactgccgtcctctgcgacgacagacaggtgagcagtgcccccgtc 30672
                                                                                ****** ************* ************************************ **

chimp.LOC112206744-LOC107973052-GGT2.revcompl                                   atccggcgtgtagaactcgggcttgtagtaggagatcgggtgagtggtgtggtcagagat 43595
chimp54546-135457.revcompl.Pan.troglodytes.clone.rp43-41g5.GenBank:AC099533.36  AACCGGCGTGTAGAACTCGGGCTTGTAGTAGGAGATCGGGTGAGTGGTGTCGTCAGAGAT 45638
human.FAM30B-LOC105372935-GGT2.NCBI.                                            atccggcgtgtagaactcgggcttgtagtaggagatcgggtgagtggtgtggtcagagat 42593
human.FAM230E-LOC105377182-GGT3P.NCBI.                                          atccggcgtgtagaactcgggcttgtagtaggagatcgggtgagtggtgtggtcagagat 41265
human.FAM230J-LOC105372942-GGTLC5P.NCBI.rev.compl                               atccggcgtgtagaactcgggcttgtcgtaggagatcgggtgagtggtgtggtcagagat 41984
human.FAM230A.ncRNA.18487127.18500594-GGTLC3.18516335.18518165.rev.compl.REF    atccggcgtgtagaactcgggcttgtagtaggagatcgggtgagtggtgtggtcagagat 30732
                                                                                * ************************ *********************** *********

chimp.LOC112206744-LOC107973052-GGT2.revcompl                                   ctgggcctggagctgggcagcgaagaactcggaggtcatgttgcggaccacctgccgaga 43655
chimp54546-135457.revcompl.Pan.troglodytes.clone.rp43-41g5.GenBank:AC099533.36  CTGGGCCCGGAGCTGGGCAGCGAAGAACTCAGAGGTCATGTTGCGGACCACCTGCCGAGA 45698
human.FAM30B-LOC105372935-GGT2.NCBI.                                            ctgggaccggagctgggcagcgaagaactcagaggtcatgttgcggaccacctgctgaga 42653
human.FAM230E-LOC105377182-GGT3P.NCBI.                                          ctgggaccggagctgggcagcgaagaactcagaggtcatgttgcggaccacctgctgaga 41325
human.FAM230J-LOC105372942-GGTLC5P.NCBI.rev.compl                               ctgggaccggagctgggcagcgaagaactcagaggtcatgttgcggaccacctgctgaga 42044
human.FAM230A.ncRNA.18487127.18500594-GGTLC3.18516335.18518165.rev.compl.REF    ctgggaccggagctgggcagcgaagaactcagaggtcatgttgcggaccacctgctgaga 30792
                                                                                ***** * ********************** ************************ ****

chimp.LOC112206744-LOC107973052-GGT2.revcompl                                   ccccagagctggcctgaggaggtggggagggagggtggggagggggcacaggtctcagaa 43715
chimp54546-135457.revcompl.Pan.troglodytes.clone.rp43-41g5.GenBank:AC099533.36  CCCCAGAGCTGGCCTGAGGAGGTGGGGAGGGAGGGTGGGGAGGGGGCACAGGTCTCAGAA 45758
human.FAM30B-LOC105372935-GGT2.NCBI.                                            ccccagagctggcctgaggaggtggggagggagggtggggagggggcacaggtctcagaa 42713
human.FAM230E-LOC105377182-GGT3P.NCBI.                                          ccccagagctggcctgagga-------------ggtggggagggggcacaggtctcagaa 41372
human.FAM230J-LOC105372942-GGTLC5P.NCBI.rev.compl                               ccccagagctggcctgagga-------------ggtggggagggggcacaggtctcagaa 42091
human.FAM230A.ncRNA.18487127.18500594-GGTLC3.18516335.18518165.rev.compl.REF    ccccagagctggcctgaggaggtggggagggagggtggggagggggcacaggtctcagaa 30852
                                                                                ********************             ***************************

chimp.LOC112206744-LOC107973052-GGT2.revcompl                                   ggcccctgactgtgactctgaccgcaaccctctggcacccacaaccttccgtggctcccc 43775
chimp54546-135457.revcompl.Pan.troglodytes.clone.rp43-41g5.GenBank:AC099533.36  GGCCCCTGACTGTGGCTCTGACCACAACCCTCTGGCACCCACAACCTTCCGTGGCTCCCC 45818
human.FAM30B-LOC105372935-GGT2.NCBI.                                            ggcccttgactgtgactctgaccgcaaccctctggcacccacaaccttccgtggctcccc 42773
human.FAM230E-LOC105377182-GGT3P.NCBI.                                          ggcccttgactgtgactctgaccgcaaccctctggcacccacaaccttccgtggctcccc 41432
human.FAM230J-LOC105372942-GGTLC5P.NCBI.rev.compl                               ggcccttgactgtgactctgaccgcaaccctctggcacccacaaccttccgtggctcccc 42151
human.FAM230A.ncRNA.18487127.18500594-GGTLC3.18516335.18518165.rev.compl.REF    ggcccttgactgtgactctgaccgcaaccctctggcacccacaaccttccgtggctcccc 30912
                                                                                ***** ******** ******** ************************************

chimp.LOC112206744-LOC107973052-GGT2.revcompl                                   aggacccaagggcaggcccaggaccttgcacaaccagtctgactccctgtctctgtcgcg 43835
chimp54546-135457.revcompl.Pan.troglodytes.clone.rp43-41g5.GenBank:AC099533.36  AGGACCCAAGGGCAGGCCCAGGATCTTGCATGACCAGTCTGACTCCCTGTCTCTGTCGCG 45878
human.FAM30B-LOC105372935-GGT2.NCBI.                                            aggacccaagggcaggcccaagaccttgcatgaccagtctgactccctgtctctgtcgcg 42833
human.FAM230E-LOC105377182-GGT3P.NCBI.                                          aggacccaagggcaggcccaggaccttgcatgaccagtctgactccctgtctctgtcgcg 41492
human.FAM230J-LOC105372942-GGTLC5P.NCBI.rev.compl                               aggacccaagggcaggcccaggaccttgcatgaccagtctgactccctgtctctgtcgcg 42211
human.FAM230A.ncRNA.18487127.18500594-GGTLC3.18516335.18518165.rev.compl.REF    aggacccaagggcaggcccaagaccttgcatgaccagtctgactccctgtctctgtcgcg 30972
                                                                                ******************** ** ******  ****************************

chimp.LOC112206744-LOC107973052-GGT2.revcompl                                   ttccagcaactctgaatgtctgtctgcgtggtcctcagcctccagacccttgccacattc 43895
chimp54546-135457.revcompl.Pan.troglodytes.clone.rp43-41g5.GenBank:AC099533.36  TTCCAGCAACTCTGAATGTCTGTCTGCCTGGTCCTCAGCCTCCAGACCCTTGCTGCATTC 45938
human.FAM30B-LOC105372935-GGT2.NCBI.                                            ttccagcaactctgaatgtctgtctgcctggtcctcagcctccagacccttgccgcattc 42893
human.FAM230E-LOC105377182-GGT3P.NCBI.                                          ttccagcaactctgaatgtctgtctgcctggtcctcagcctccagacccttgccacattc 41552
human.FAM230J-LOC105372942-GGTLC5P.NCBI.rev.compl                               ttccagcaactctgaatgtctgtctgcctggtcctcagcctccagacccttgccgcattc 42271
human.FAM230A.ncRNA.18487127.18500594-GGTLC3.18516335.18518165.rev.compl.REF    ttccagcaactctgaatgtctgtctgcctggtcctcagcctccagacccttgccgcattc 31032
                                                                                *************************** *************************  *****

chimp.LOC112206744-LOC107973052-GGT2.revcompl                                   aatcactcattctttcatgcaaaaaatatttctagaatttgcactgcatgcctggcactg 43955
chimp54546-135457.revcompl.Pan.troglodytes.clone.rp43-41g5.GenBank:AC099533.36  AATCACTCATTCCTTCATGCAAAAAATATTTCTAGAGTTTGCACTGCATGCCTGGAACTG 45998
human.FAM30B-LOC105372935-GGT2.NCBI.                                            aatcactcattccttcatgcaaaaaatatttctagaatttgcactgcatgcctggcactg 42953
human.FAM230E-LOC105377182-GGT3P.NCBI.                                          aatcactcattctttcatgcaaaaaatatttctagaatttgcactgcatgcctggcactg 41612
human.FAM230J-LOC105372942-GGTLC5P.NCBI.rev.compl                               aatcactcattctttcatgcaaaaaatatttctagaatttgcactgcatgcctggcactg 42331
human.FAM230A.ncRNA.18487127.18500594-GGTLC3.18516335.18518165.rev.compl.REF    aatcact----------------------------------------------------- 31039
                                                                                *******

chimp.LOC112206744-LOC107973052-GGT2.revcompl                                   gggaatcaacagggaacagacatttaggtcctgccctcatgccaagaaaaacaaacacac 44015
chimp54546-135457.revcompl.Pan.troglodytes.clone.rp43-41g5.GenBank:AC099533.36  GGGAATCAACAGGGAACAGACACTTAGGTCCTGCCCTCACGCCAAGAAAAACAAACACAC 46058
human.FAM30B-LOC105372935-GGT2.NCBI.                                            gggaatcaacagggaacagacacttaagtcctgccctcatgccaagaaaaacaaacacac 43013
human.FAM230E-LOC105377182-GGT3P.NCBI.                                          gggaatcaacagggaacagacacttaagtcctgccctcatgccaagaaaaacaaacacac 41672
human.FAM230J-LOC105372942-GGTLC5P.NCBI.rev.compl                               gggaatcaacagggaacagacacttaagtcctgccctcatgccaagaaaaacaaacacac 42391
human.FAM230A.ncRNA.18487127.18500594-GGTLC3.18516335.18518165.rev.compl.REF    ------------------------------------------------------------ 31039

chimp.LOC112206744-LOC107973052-GGT2.revcompl                                   acagggaaagtgctgaaaccacaagccaggtaaggggaatcaagaggcatgaggtatggg 44075
chimp54546-135457.revcompl.Pan.troglodytes.clone.rp43-41g5.GenBank:AC099533.36  ACAGGGAAAGTGCTGAAACCACAAGCCAGGTAAGGGGAATCAAGAGGCATGAGGTATGGG 46118
human.FAM30B-LOC105372935-GGT2.NCBI.                                            ccagggaaagtgctgaaaccacaggccaggtaaggggaatcaagaggcatgaggtatggg 43073
human.FAM230E-LOC105377182-GGT3P.NCBI.                                          acagggaaagtgctgaaaccacaggccaggtaaggggaatcaagaggcatgaggtatggg 41732
human.FAM230J-LOC105372942-GGTLC5P.NCBI.rev.compl                               acagggaaagtgctgaaaccacaggccaggtaaggggaatcaagaggcatgaggtatggg 42451
human.FAM230A.ncRNA.18487127.18500594-GGTLC3.18516335.18518165.rev.compl.REF    ------------------------------------------------------------ 31039

chimp.LOC112206744-LOC107973052-GGT2.revcompl                                   cagagtggtcagggagggcttctcagaggaggcagcgtgtgaaaagagcctggaatgtgg 44135
chimp54546-135457.revcompl.Pan.troglodytes.clone.rp43-41g5.GenBank:AC099533.36  CAGAGTGGTCAGGGAGGGCTTCTCAGAGGAGGCAACATGTGAAAAGAGCCTGGAATGTGG 46178
human.FAM30B-LOC105372935-GGT2.NCBI.                                            cagagtggtcagggagggcttctcagaggaggcaacgtgtgaaaagagcctggaatgtgg 43133
human.FAM230E-LOC105377182-GGT3P.NCBI.                                          cagagtggtcagggagggcttctcagaggaggcaacgtgtgaaaagagcctggaaagtgg 41792
human.FAM230J-LOC105372942-GGTLC5P.NCBI.rev.compl                               cagagtggtcagggagggcttctcagaggaggcaacgtgtgaaaagagcctggaatgttg 42511
```

```
human.FAM230A.ncRNA.18487127.18500594-GGTLC3.18516335.18518165.rev.compl.REF        ------------------------------------------------------------ 31039

chimp.LOC112206744-LOC107973052-GGT2.revcompl                                       cctaaatggtcagtgcaaaggccctgaggcaggtggtataggctggtgagcgataggcag 44195
chimp54546-135457.revcompl.Pan.troglodytes.clone.rp43-41g5.GenBank:AC099533.36      CCTAAATGGTCAGTGCAAAGGCCCTGAGGCAGGTGGTATAGGCTGGTGAGCGATAGGCAG 46238
human.FAM30B-LOC105372935-GGT2.NCBI.                                                cctaaatggtcagtgcaaaggccctgaggcaggtggtataggctggtgagcgataggcag 43193
human.FAM230E-LOC105377182-GGT3P.NCBI.                                              cctaaatggtcagtgcaaaggccctgaggcaggtggcataggctggtgagcgataggcag 41852
human.FAM230J-LOC105372942-GGTLC5P.NCBI.rev.compl                                   cctaaatggtcagtgcaaaggccctgaggcaggtggtataggctggtgagcgataggcag 42571
human.FAM230A.ncRNA.18487127.18500594-GGTLC3.18516335.18518165.rev.compl.REF        ------------------------------------------------------------ 31039

chimp.LOC112206744-LOC107973052-GGT2.revcompl                                       agagtgaatggagtaggatggggagaagaggatgaagatgcaggctggggcccatcccac 44255
chimp54546-135457.revcompl.Pan.troglodytes.clone.rp43-41g5.GenBank:AC099533.36      AGAGTGAATGGAGTGGGGTGGGGAGAAGAGGATGAAGATGCAGGCTGGGGCCCATCCCAC 46298
human.FAM30B-LOC105372935-GGT2.NCBI.                                                agagtgaatggagtagggtggggagaagaggatgaagatgcaggctggggcccatcccac 43253
human.FAM230E-LOC105377182-GGT3P.NCBI.                                              agagtgaatggagtagggtggggagaagaggatgaagatgcaggctggggcccatcccac 41912
human.FAM230J-LOC105372942-GGTLC5P.NCBI.rev.compl                                   agagtgaatggagtggggtggggagaagaggatgaagatgcaggctggggcccatcccac 42631
human.FAM230A.ncRNA.18487127.18500594-GGTLC3.18516335.18518165.rev.compl.REF        ------------------------------------------------------------ 31039

chimp.LOC112206744-LOC107973052-GGT2.revcompl                                       aggacctcctaggtcccataacaactggcttttgctctgtgccatgcaggcttagggcag 44315
chimp54546-135457.revcompl.Pan.troglodytes.clone.rp43-41g5.GenBank:AC099533.36      AGGACCTCCTAGGTCCCATAACAACTGGCTTTTGCTCTGTGCCATGCAGGCTTAGGGCAG 46358
human.FAM30B-LOC105372935-GGT2.NCBI.                                                aggacctcctaggtcccataacaactggctttttctctgtgccatgcaggcttagggcag 43313
human.FAM230E-LOC105377182-GGT3P.NCBI.                                              aggacctcctaggtcccataacaactggcttttgctctgtgccatgcaggcttagggcag 41972
human.FAM230J-LOC105372942-GGTLC5P.NCBI.rev.compl                                   aggacctcctaggtcccataacaactggcttttgctctgtgccatgcaggcttagggcag 42691
human.FAM230A.ncRNA.18487127.18500594-GGTLC3.18516335.18518165.rev.compl.REF        ------------------------------------------------------------ 31039

chimp.LOC112206744-LOC107973052-GGT2.revcompl                                       aggaatgaggagggtggggagtgttttcacagggtccctctggcagctatgacggggata 44375
chimp54546-135457.revcompl.Pan.troglodytes.clone.rp43-41g5.GenBank:AC099533.36      AGGAATGAGCAGGCTGGGGAGTGTTTTCACAGGGTCCCTCTGGCAGCTATGACGGGGATA 46418
human.FAM30B-LOC105372935-GGT2.NCBI.                                                aggaatgaggaggctggggagtgttttcacagggtccctctggcagctatgacggggata 43373
human.FAM230E-LOC105377182-GGT3P.NCBI.                                              aggaatgaggaggctggggagtgttttcacagggtccctctggcagctatgacggggata 42032
human.FAM230J-LOC105372942-GGTLC5P.NCBI.rev.compl                                   aagaatgaggaggctggggagtgttttcacagggtccctctggcagctatgacggggata 42751
human.FAM230A.ncRNA.18487127.18500594-GGTLC3.18516335.18518165.rev.compl.REF        ------------------------------------------------------------ 31039

chimp.LOC112206744-LOC107973052-GGT2.revcompl                                       aggataaagcccaaaggggaggctgtgggtatcaaccaggcaagagatgatggcctgggt 44435
chimp54546-135457.revcompl.Pan.troglodytes.clone.rp43-41g5.GenBank:AC099533.36      AGGATAAAGCCAAAAGGGGAGGCTGTGGGTATCAACCAGGCAAGAGATGATGGCCTGGGT 46478
human.FAM30B-LOC105372935-GGT2.NCBI.                                                aggataaagcccaaaggggaggctgtgggtatcaaccaggcaagagatgatggcctgggt 43433
human.FAM230E-LOC105377182-GGT3P.NCBI.                                              aggataaagcccaaaggggaggctgtgggtatcaaccaggcaagagatgatggcctgggt 42092
human.FAM230J-LOC105372942-GGTLC5P.NCBI.rev.compl                                   aggataaagcccaaaggggaggctgtgggtatcaaccaggcaagagatgatggcctgggt 42811
human.FAM230A.ncRNA.18487127.18500594-GGTLC3.18516335.18518165.rev.compl.REF        ------------------------------------------------------------ 31039

chimp.LOC112206744-LOC107973052-GGT2.revcompl                                       gggagaaagagaagaatcaaggatggtgccgactagcgaggtaaaccctgcagaaggggc 44495
chimp54546-135457.revcompl.Pan.troglodytes.clone.rp43-41g5.GenBank:AC099533.36      GGGAGAAAGAGAAGAATCAAGGATGGTGCCGACTAGCGAGGTAAACCCTGCAGAAGGGGC 46538
human.FAM30B-LOC105372935-GGT2.NCBI.                                                gggagaaagagaagaatcaaggatggtgccgactagcgagg-----ccggcagaaggggc 43488
human.FAM230E-LOC105377182-GGT3P.NCBI.                                              gggagaaagagaagaatcaagcatggtgccgactagcgagg-----ccggcagaaggggc 42147
human.FAM230J-LOC105372942-GGTLC5P.NCBI.rev.compl                                   gggagaaagagaagaatcaagcatggtgccgactagcgagg-----ccggcagaaggggc 42866
human.FAM230A.ncRNA.18487127.18500594-GGTLC3.18516335.18518165.rev.compl.REF        ------------------------------------------------------------ 31039

chimp.LOC112206744-LOC107973052-GGT2.revcompl                                       aggtttggggatggtcaggagcttgattttggatacttcatcagacctgaagagcatggg 44555
chimp54546-135457.revcompl.Pan.troglodytes.clone.rp43-41g5.GenBank:AC099533.36      AGGTTTGGGGATGGTCAGGAGCTTGATTTTGGATACTTCATCAGACCCGAAGAGCATGGG 46598
human.FAM30B-LOC105372935-GGT2.NCBI.                                                cggtttggggatggtcaggagcttgattttggatacttcatcagacccaaagagcatggg 43548
human.FAM230E-LOC105377182-GGT3P.NCBI.                                              cggtttggggatggtcaggagcttgattttggatacttcatcagacccaaagagcatggg 42207
human.FAM230J-LOC105372942-GGTLC5P.NCBI.rev.compl                                   cggtttggggatggtcaggagcttgattttggatacttcatcagacccaaagagcatggg 42926
human.FAM230A.ncRNA.18487127.18500594-GGTLC3.18516335.18518165.rev.compl.REF        ------------------------------------------------------------ 31039

chimp.LOC112206744-LOC107973052-GGT2.revcompl                                       tgcacgtataaaaaaaataaataaataagcatgggttcacgggcaagggcgggctgagag 44615
chimp54546-135457.revcompl.Pan.troglodytes.clone.rp43-41g5.GenBank:AC099533.36      TGCAAGTATAAAAAAAATAAATAAATAAGCATGGGTTCACGGGCAAGGGCGGGCTGAGAG 46658
human.FAM30B-LOC105372935-GGT2.NCBI.                                                tgcacgtataaaaaaaataaataaataagcatgggttcacgggcaagggcgggctgagag 43608
human.FAM230E-LOC105377182-GGT3P.NCBI.                                              tgcacgtataaaaaaaataaataaataagcatgggttcacgggcaagggcgggctgagag 42267
human.FAM230J-LOC105372942-GGTLC5P.NCBI.rev.compl                                   tgcacgtataaaaaaaataaataaataagcatgggttcacgggcaagggcgggctgagag 42986
human.FAM230A.ncRNA.18487127.18500594-GGTLC3.18516335.18518165.rev.compl.REF        ------------------------------------------------------------ 31039

chimp.LOC112206744-LOC107973052-GGT2.revcompl                                       atgaacatggaggtattgacattgagtggctgctggatgccatgagcctggccaaggtcc 44675
chimp54546-135457.revcompl.Pan.troglodytes.clone.rp43-41g5.GenBank:AC099533.36      ATGAACACGGAGGTATTGACATTGAGTGGCTGCTGGATGCCATGAGCCTGGCCAAGGTCC 46718
human.FAM30B-LOC105372935-GGT2.NCBI.                                                atgaacatggaggtattgacattgagtggctgctggatgccatgagcctggccaaggtcc 43668
human.FAM230E-LOC105377182-GGT3P.NCBI.                                              atgaacatggaggtattgacattgagtggctgctggatgccatgagcctggccaaggtcc 42327
human.FAM230J-LOC105372942-GGTLC5P.NCBI.rev.compl                                   atgaacatggaggtattgacattgagtggctgctggatgccatgagcctggccaaggtcc 43046
human.FAM230A.ncRNA.18487127.18500594-GGTLC3.18516335.18518165.rev.compl.REF        ------------------------------------------------------------ 31039

chimp.LOC112206744-LOC107973052-GGT2.revcompl                                       ccaaggcagtggcgaggaggagatgaggaggtcaaggaggagac--agaggatggacccg 44733
chimp54546-135457.revcompl.Pan.troglodytes.clone.rp43-41g5.GenBank:AC099533.36      CCAAGGCAGTGGCGAGGAGGAGATGAGGAGGTCAAGGAGGAGAC--AGAGGATGGACCCG 46776
human.FAM30B-LOC105372935-GGT2.NCBI.                                                ccaaggcagtggcgaggaggagatgaggaggtcaaagaggagacagagaggatggacccg 43728
human.FAM230E-LOC105377182-GGT3P.NCBI.                                              ccaaggcagtggcgaggaggagatgaggaggtcaaagaggagacagagaggatggacccg 42387
human.FAM230J-LOC105372942-GGTLC5P.NCBI.rev.compl                                   ccaaggcagtggcgaggaggagatgaggaggtcaaagaggagacagagaggatggacccg 43106
human.FAM230A.ncRNA.18487127.18500594-GGTLC3.18516335.18518165.rev.compl.REF        ------------------------------------------------------------ 31039

chimp.LOC112206744-LOC107973052-GGT2.revcompl                                       aaggccaaagaaaatgcctcaagagagtttcaacaccgggcgcggtggctcacgcctgta 44793
chimp54546-135457.revcompl.Pan.troglodytes.clone.rp43-41g5.GenBank:AC099533.36      AAGGCCGAAGAAAATGCCTCAAGAGAGTTTCAACACCGGGCGCGGTGGCTCACGCCTGTA 46836
human.FAM30B-LOC105372935-GGT2.NCBI.                                                aaggccgaagaaaatgcctcaagagagtttcaacaccgggcgcggtggctcacgcctgta 43788
human.FAM230E-LOC105377182-GGT3P.NCBI.                                              aaggccgaagaaaatgcctcaagagagtttcaacaccgggcgcggtggctcacgcctgta 42447
human.FAM230J-LOC105372942-GGTLC5P.NCBI.rev.compl                                   aaggccgaagaaaatgcctcaagagagtttcaacaccgggcgcggtggctcacgcttgta 43166
human.FAM230A.ncRNA.18487127.18500594-GGTLC3.18516335.18518165.rev.compl.REF        ------------------------------------------------------------ 31039

chimp.LOC112206744-LOC107973052-GGT2.revcompl                                       atcccagcactttgggaggccgaggcctgtaatcccagcactttgggagggcggatcacg 44853
chimp54546-135457.revcompl.Pan.troglodytes.clone.rp43-41g5.GenBank:AC099533.36      ATCCCAGCACTTTGGGAGGCCGAGGCCTGTAATCCCAGCACTTTGGGAGGG-GGATCACG 46895
human.FAM30B-LOC105372935-GGT2.NCBI.                                                atcccagcactttgggaggccgaggcctgtaatcccagcactttgggagggcggatcatg 43848
human.FAM230E-LOC105377182-GGT3P.NCBI.                                              atcccagcactttgggaggccgaggcctgtaatcccagcactttgggagggcggatcatg 42507
human.FAM230J-LOC105372942-GGTLC5P.NCBI.rev.compl                                   atcccagcactttgggaggccgaggcctgtaatcccagcactttgggagggcggatcatg 43226
human.FAM230A.ncRNA.18487127.18500594-GGTLC3.18516335.18518165.rev.compl.REF        ------------------------------------------------------------ 31039

chimp.LOC112206744-LOC107973052-GGT2.revcompl                                       acgtcaggagatcaagaccatcctggctaacacagtgaaaccccgtctctactaaaaata 44913
chimp54546-135457.revcompl.Pan.troglodytes.clone.rp43-41g5.GenBank:AC099533.36      ACGTCAGGAGATGGAGATCATCCTGGCTAACAGGGTGAAACTCCGTCTCTACTAAAAATA 46955
human.FAM30B-LOC105372935-GGT2.NCBI.                                                acgtcaggagatcgagaccatcctggctaacacagtgaaaccccgtctctactaaaaata 43908
human.FAM230E-LOC105377182-GGT3P.NCBI.                                              acgtcaggagatcgagaccatcctggctaacacagtgaaaccccgtctctactaaaaata 42567
human.FAM230J-LOC105372942-GGTLC5P.NCBI.rev.compl                                   acgtcaggagatcgagaccatcctggctaacacagtgaaaccccgtctctactaaaaata 43286
human.FAM230A.ncRNA.18487127.18500594-GGTLC3.18516335.18518165.rev.compl.REF        ------------------------------------------------------------ 31039

chimp.LOC112206744-LOC107973052-GGT2.revcompl                                       caaaaaattagctgggcgcggtggtgggcacctgtagtcccagctacttgggggggctga 44973
chimp54546-135457.revcompl.Pan.troglodytes.clone.rp43-41g5.GenBank:AC099533.36      CAAAAAATTAGCCGGGTGTGGTGGCGGGCGCCTGTAGTCCCAACTACTC-CAGAGGCTGA 47014
human.FAM30B-LOC105372935-GGT2.NCBI.                                                caaaaaattagctgggcgcggtggtgggcacctgtagtcccagctactt-ggggggctga 43967
human.FAM230E-LOC105377182-GGT3P.NCBI.                                              caaaaaattagctgggcgcggtggtgggcacctgtagtcccagctactt-ggggggctga 42626
human.FAM230J-LOC105372942-GGTLC5P.NCBI.rev.compl                                   caaaaaattagctgggcgcggtggtgggcacctgtagtcccagctactt-ggggggctga 43345
human.FAM230A.ncRNA.18487127.18500594-GGTLC3.18516335.18518165.rev.compl.REF        ------------------------------------------------------------ 31039

chimp.LOC112206744-LOC107973052-GGT2.revcompl                                       ggcaggagaatggcatgaacctgggaggcggagcttgcagtgagccgagatcccgccact 45033
chimp54546-135457.revcompl.Pan.troglodytes.clone.rp43-41g5.GenBank:AC099533.36      GGCAGGAGAATGGCGTGAACCCGGTAGGCGGAGCTTGCGGTGAGCCGAGATCAGGCCACT 47074
human.FAM30B-LOC105372935-GGT2.NCBI.                                                ggcaggagaatggcgtgaacctgggaggcggagcttgcagtgagcggagatcctgccact 44027
human.FAM230E-LOC105377182-GGT3P.NCBI.                                              ggcaggagaatggcgtgaacctgggaggcagagcttgcagtgagccgagatcccgccact 42686
human.FAM230J-LOC105372942-GGTLC5P.NCBI.rev.compl                                   ggcaggagaatggcgtgaacctgggaggcggagcttgcagtgagccgagatcccgccact 43405
human.FAM230A.ncRNA.18487127.18500594-GGTLC3.18516335.18518165.rev.compl.REF        ------------------------------------------------------------ 31039

chimp.LOC112206744-LOC107973052-GGT2.revcompl                                       gcactccagcctgggggacagcctgggggacagagcgagactccgtctca---------- 45083
chimp54546-135457.revcompl.Pan.troglodytes.clone.rp43-41g5.GenBank:AC099533.36      GGAATC------------CAGCCTGGGCGATAGAGGGAGACTCCATCTCA---------A 47113
human.FAM30B-LOC105372935-GGT2.NCBI.                                                gcactccagcctgggggacagcctgggggacagagcgagactccgtctca---------- 44077
human.FAM230E-LOC105377182-GGT3P.NCBI.                                              gcactccagcctgggggacagcctgggggacagagcgagactccgtctca---------- 42736
human.FAM230J-LOC105372942-GGTLC5P.NCBI.rev.compl                                   gcactccagcctgggggacagcctgggggacagagcgagactccgtctcaaaaataaaat 43465
human.FAM230A.ncRNA.18487127.18500594-GGTLC3.18516335.18518165.rev.compl.REF        ------------------------------------------------------------ 31039

chimp.LOC112206744-LOC107973052-GGT2.revcompl                                       aaaataaaataaaataaaataaaataaagtttcagcaacaccccacggatt--agttgac 45141
chimp54546-135457.revcompl.Pan.troglodytes.clone.rp43-41g5.GenBank:AC099533.36      AAAAAAAAAAAAAAAAAAAAAGAGTCTGGCTCTGTTGCCCAGGCTGGAGTGCAGTGGCG 47173
human.FAM30B-LOC105372935-GGT2.NCBI.                                                aaaataaaataaaataaaataaaataaagtttcagcaacaccccacggatt--agttgac 44135
human.FAM230E-LOC105377182-GGT3P.NCBI.                                              -----aaaataaaataaaataaaataaagtttcagcaacaccccacggatt--agctgac 42789
human.FAM230J-LOC105372942-GGTLC5P.NCBI.rev.compl                                   aaaataaaataaaataaaataaaataaagtttcagcaacaccccacggatt--agctgac 43523
human.FAM230A.ncRNA.18487127.18500594-GGTLC3.18516335.18518165.rev.compl.REF        ------------------------------------------------------------ 31039

chimp.LOC112206744-LOC107973052-GGT2.revcompl                                       caatcccagggaaaggtgttctgtctgaatctgccctcaaggaaacagaaaggcaaatcc 45201
chimp54546-135457.revcompl.Pan.troglodytes.clone.rp43-41g5.GenBank:AC099533.36      CGATCTCGGGGCATCACAATCCCTTCCCCGCCCGCCCCCGGGTTCAAGTGATTCTCCTGT 47233
human.FAM30B-LOC105372935-GGT2.NCBI.                                                caatcccagggaaaggtgttctgtctgaatctgccctcaaggaaacagaaaggcaaatcc 44195
human.FAM230E-LOC105377182-GGT3P.NCBI.                                              caatcccagggaaaggtgttctgtctgaatctgccctcaaggaaacagaaaggcaaatcc 42849
human.FAM230J-LOC105372942-GGTLC5P.NCBI.rev.compl                                   caatcccagggaaaggtgttctgtctgaatctgccctcaaggaaacagaaaggcaaatcc 43583
human.FAM230A.ncRNA.18487127.18500594-GGTLC3.18516335.18518165.rev.compl.REF        ------------------------------------------------------------ 31039
```

```
chimp.LOC112206744-LOC107973052-GGT2.revcompl                                   acgatgtgggacattttccaagactactgccctgggctttaaaaatcaacaaaacagg-- 45259
chimp54546-135457.revcompl.Pan.troglodytes.clone.rp43-41g5.GenBank:AC099533.36  CTCAGCCGCCGGAGTAGCTGGGACTACAGGCGCGTGCCACCATGTCTGACTAAATTTGTA 47293
human.FAM30B-LOC105372935-GGT2.NCBI.                                            acgatgtgggacattttccaagactactgccctgggctttaaaaatcaacaaaacagg-- 44253
human.FAM230E-LOC105377182-GGT3P.NCBI.                                          acgatgtgggacattttccaagactactgccctgggctttaaaaatcaacaaaacagg-- 42907
human.FAM230J-LOC105372942-GGTLC5P.NCBI.rev.compl                               acgatgtgggacattttccaagactactgccctgggctttaaaaatcaacaaaacagg-- 43641
human.FAM230A.ncRNA.18487127.18500594-GGTLC3.18516335.18518165.rev.compl.REF    ------------------------------------------------------------ 31039

chimp.LOC112206744-LOC107973052-GGT2.revcompl                                   ------ccaggcacggtggctcatgcctgtaatcccagcactttgggaggccgaggcagg 45313
chimp54546-135457.revcompl.Pan.troglodytes.clone.rp43-41g5.GenBank:AC099533.36  TTTTTACTAGAGACGGGGTTTCACTATGTT-----------------------GGCCAGG 47330
human.FAM30B-LOC105372935-GGT2.NCBI.                                            ------ccaggcacggtggttcatgcctgtaatcccagcactttgggaggccgaggcagg 44307
human.FAM230E-LOC105377182-GGT3P.NCBI.                                          ------ccaggcacggtggctcatgcctgtaatcccagcactttgggaggccgaggcagg 42961
human.FAM230J-LOC105372942-GGTLC5P.NCBI.rev.compl                               ------ccaggcacggtggctcatgcctgtaatcccagcactttgggaggccgaggcagg 43695
human.FAM230A.ncRNA.18487127.18500594-GGTLC3.18516335.18518165.rev.compl.REF    ------------------------------------------------------------ 31039

chimp.LOC112206744-LOC107973052-GGT2.revcompl                                   cggatcacgaggtcaggagatcgcgatcacggtgaaaccccgtctctactaaaaatacaa 45373
chimp54546-135457.revcompl.Pan.troglodytes.clone.rp43-41g5.GenBank:AC099533.36  CTGGTCTCCAACTCCTGATCTCGTGATCCGTCTGCCCCGACCTCCCAAAGTGCTAGGATT 47390
human.FAM30B-LOC105372935-GGT2.NCBI.                                            cggatcacgaggtcaggagatcgcgatcacggtgaaaccccgtctctactaaaaatacaa 44367
human.FAM230E-LOC105377182-GGT3P.NCBI.                                          cggatcacgaggtcaggagatcgcgatcacggtgaaaccccgtctctactaaaaatacaa 43021
human.FAM230J-LOC105372942-GGTLC5P.NCBI.rev.compl                               cggatcacgaggtcaggagatcgcgatcacggtgaaaccccgtctctactaaaaatacaa 43755
human.FAM230A.ncRNA.18487127.18500594-GGTLC3.18516335.18518165.rev.compl.REF    ------------------------------------------------------------ 31039

chimp.LOC112206744-LOC107973052-GGT2.revcompl                                   aaaattagctgggcgcagtggcgggtgcctgtagtcccagcta--------ctcgggagg 45425
chimp54546-135457.revcompl.Pan.troglodytes.clone.rp43-41g5.GenBank:AC099533.36  ATAGGCATAAGCCGCCACGCCCGGCCTCTTTTTTTTCTTTTTCTTTTTTTTATCTGGAGA 47450
human.FAM30B-LOC105372935-GGT2.NCBI.                                            aaaattagctgggcgcagtgtcgggcgcctgtagtcccagcta--------ctcgggagg 44419
human.FAM230E-LOC105377182-GGT3P.NCBI.                                          aaaattagctgggcgcagtgtcgggcgcctgtagtcccagcta--------ctcgggagg 43073
human.FAM230J-LOC105372942-GGTLC5P.NCBI.rev.compl                               aaaattagctgggcgcagtgtcgggcgcctgtagtcccagcta--------ctcgggagg 43807
human.FAM230A.ncRNA.18487127.18500594-GGTLC3.18516335.18518165.rev.compl.REF    ------------------------------------------------------------ 31039

chimp.LOC112206744-LOC107973052-GGT2.revcompl                                   ctgaggcaggagaatggcgtgaacccaggaggcggagcttgcagtgagccgagatagcac 45485
chimp54546-135457.revcompl.Pan.troglodytes.clone.rp43-41g5.GenBank:AC099533.36  CTGAGTTTTGCACTTGTTGCCCAGGCTGGAG--------TGCAATGGTGCGATCTCAGCT 47502
human.FAM30B-LOC105372935-GGT2.NCBI.                                            ctgaggcaggagaatggcgtgaacccaggaggcggagcttgcagtgagccgagatagcgc 44479
human.FAM230E-LOC105377182-GGT3P.NCBI.                                          ctgaggcaggagaatggcgtgaacccaggaggcagagcttgcagtgagccgagatagcac 43133
human.FAM230J-LOC105372942-GGTLC5P.NCBI.rev.compl                               ctgaggcaggagaatggcgtgaacccaggaggcggagcttgcagtgagccgagatagcgc 43867
human.FAM230A.ncRNA.18487127.18500594-GGTLC3.18516335.18518165.rev.compl.REF    ------------------------------------------------------------ 31039

chimp.LOC112206744-LOC107973052-GGT2.revcompl                                   cactgcactc-cagcctgggcgacagagcgacaccctgtctcacaaaaaagccacacctg 45544
chimp54546-135457.revcompl.Pan.troglodytes.clone.rp43-41g5.GenBank:AC099533.36  CACTGCAATCTCCACCTCAGCAGGAGAGCAGGAATCTTCAGTGATCCACCGGCAGATCTG 47562
human.FAM30B-LOC105372935-GGT2.NCBI.                                            cactgcactc-cagcctgggcaacagagcgagactccgtctcaaagaaaaaacacacctg 44538
human.FAM230E-LOC105377182-GGT3P.NCBI.                                          cactgcactc-cagcctgggcaacagagcgagactccgtctcaaagaaaaaacacacctg 43192
human.FAM230J-LOC105372942-GGTLC5P.NCBI.rev.compl                               cactgcactc-cagcctgggcaacagagcgagactccgtctcaaagaaaaaacacacctg 43926
human.FAM230A.ncRNA.18487127.18500594-GGTLC3.18516335.18518165.rev.compl.REF    ------------------------------------------------------------ 31039

chimp.LOC112206744-LOC107973052-GGT2.revcompl                                   taatcccagcgctttgggaggctgaggtgggaagatagtttgatcccaggagtttgagac 45604
chimp54546-135457.revcompl.Pan.troglodytes.clone.rp43-41g5.GenBank:AC099533.36  -----CCGCCATTGTGGGCACCTGTTCCTCCCGCGACCTTTGTGCCCGCCTCTCTCCTTC 47617
human.FAM30B-LOC105372935-GGT2.NCBI.                                            taatcccagcgctttgggaggctgaggtgggaagacagtttgatcccaggagtttgagac 44598
human.FAM230E-LOC105377182-GGT3P.NCBI.                                          taatcccagcgctttgggaggctgaggtgggaagacagtttgatcccaggagtttgagac 43252
human.FAM230J-LOC105372942-GGTLC5P.NCBI.rev.compl                               taatcccagcgctttgggaggctgaggtgggaagacagtttgatcccaggagtttgagac 43986
human.FAM230A.ncRNA.18487127.18500594-GGTLC3.18516335.18518165.rev.compl.REF    ------------------------------------------------------------ 31039

chimp.LOC112206744-LOC107973052-GGT2.revcompl                                   cagtctgggcaagaccctgtctctaaaaaaatatataaaaattatccaggtttggtggca 45664
chimp54546-135457.revcompl.Pan.troglodytes.clone.rp43-41g5.GenBank:AC099533.36  CAGT----------ACCTATTGCATGACCCC-CCACGTCCGCCTCCCGCCATTGCAGCA 47666
human.FAM30B-LOC105372935-GGT2.NCBI.                                            cagtctgggcaagaccctgtctctaaaaaaa-atacaaaaattatccaggtttggtggca 44657
human.FAM230E-LOC105377182-GGT3P.NCBI.                                          cagtctgggcaagaccctgtctctaaaaaaa-atacaaaaattatccaggtttggtggca 43311
human.FAM230J-LOC105372942-GGTLC5P.NCBI.rev.compl                               cagtctgggcaagaccctgtctctaaaaaaa-atacaaaaattatccaggtttggtggca 44045
human.FAM230A.ncRNA.18487127.18500594-GGTLC3.18516335.18518165.rev.compl.REF    ------------------------------------------------------------ 31039

chimp.LOC112206744-LOC107973052-GGT2.revcompl                                   cgtgcctctggt------------------------------------------------ 45676
chimp54546-135457.revcompl.Pan.troglodytes.clone.rp43-41g5.GenBank:AC099533.36  AGTGCCTCGCGCGGGTACCTGGCTGCGCTTATTAATCCGTTAAGCTCGCTCTGTCACGGG 47726
human.FAM30B-LOC105372935-GGT2.NCBI.                                            cgtgcctctggt------------------------------------------------ 44669
human.FAM230E-LOC105377182-GGT3P.NCBI.                                          cctgcctctggt------------------------------------------------ 43323
human.FAM230J-LOC105372942-GGTLC5P.NCBI.rev.compl                               cgtgcctctggt------------------------------------------------ 44057
human.FAM230A.ncRNA.18487127.18500594-GGTLC3.18516335.18518165.rev.compl.REF    ------------------------------------------------------------ 31039

chimp.LOC112206744-LOC107973052-GGT2.revcompl                                   ------------------------------------------------------------ 45676
chimp54546-135457.revcompl.Pan.troglodytes.clone.rp43-41g5.GenBank:AC099533.36  CGCCATGATGTGCTCACGCGCCCGCTCCCTCAGGTTTAAAAGGCGCGTTGCCCGGCAACA 47786
human.FAM30B-LOC105372935-GGT2.NCBI.                                            ------------------------------------------------------------ 44669
human.FAM230E-LOC105377182-GGT3P.NCBI.                                          ------------------------------------------------------------ 43323
human.FAM230J-LOC105372942-GGTLC5P.NCBI.rev.compl                               ------------------------------------------------------------ 44057
human.FAM230A.ncRNA.18487127.18500594-GGTLC3.18516335.18518165.rev.compl.REF    ------------------------------------------------------------ 31039

chimp.LOC112206744-LOC107973052-GGT2.revcompl                                   ------------------cccagctgctcaggggctgaagtgggaggattgcttgagcc 45718
chimp54546-135457.revcompl.Pan.troglodytes.clone.rp43-41g5.GenBank:AC099533.36  GAAGAAACTGCTGGCTTAGCCTTTGGCCGAGTTGGCGGCTGGACGAGGACGCTCAGAGCC 47846
human.FAM30B-LOC105372935-GGT2.NCBI.                                            ------------------cccagctgctcaggggctgaagtgggaggattgcttgagcc 44711
human.FAM230E-LOC105377182-GGT3P.NCBI.                                          ------------------cccagctgctcaggggctgaagtgggaggattgcttgagcc 43365
human.FAM230J-LOC105372942-GGTLC5P.NCBI.rev.compl                               ------------------cccagctgctcaggggctgaagtgggaggattgcttgagcc 44099
human.FAM230A.ncRNA.18487127.18500594-GGTLC3.18516335.18518165.rev.compl.REF    ------------------------------------------------------------ 31039

chimp.LOC112206744-LOC107973052-GGT2.revcompl                                   ctggaggttgaggctgcagtgagccaagatcacaccactgcactccaacctggatgacag 45778
chimp54546-135457.revcompl.Pan.troglodytes.clone.rp43-41g5.GenBank:AC099533.36  C-AGCTCTCGAGAGTTCAAGCAACCGACGGTTCCCCACTGCTCCCAGGAGCGGTTACCTG 47905
human.FAM30B-LOC105372935-GGT2.NCBI.                                            ctggaggttgaggctgcagtgagccaagatcacaccactgcactccaacctggatgacag 44771
human.FAM230E-LOC105377182-GGT3P.NCBI.                                          ctggaggttgaggctgcagtgagccaagatcacaccactgcactccaacctggatgacag 43425
human.FAM230J-LOC105372942-GGTLC5P.NCBI.rev.compl                               ctggaggttgaggctgcagtgagccaagatcacaccactgcactccaacctggatgacag 44159
human.FAM230A.ncRNA.18487127.18500594-GGTLC3.18516335.18518165.rev.compl.REF    ------------------------------------------------------------ 31039

chimp.LOC112206744-LOC107973052-GGT2.revcompl                                   ag-actctgtgtccaaacaaaaacacaacaaaaacaaaacagccaagagagcctactgta 45837
chimp54546-135457.revcompl.Pan.troglodytes.clone.rp43-41g5.GenBank:AC099533.36  GGCACTCTGTGCCC-------CTCCTTCCTGTTCGGCCCAGGCCGAGGACCTGCCAGTA 47958
human.FAM30B-LOC105372935-GGT2.NCBI.                                            ag-actctgtgtacaaacaaaaacacaacaaaaacaaaacagccaagggagcctactgta 44830
human.FAM230E-LOC105377182-GGT3P.NCBI.                                          ag-actctgtgtacaaacaaaaacacaacaaaaacaaaacagccaagggagcctactgta 43484
human.FAM230J-LOC105372942-GGTLC5P.NCBI.rev.compl                               ag-actctgtgtacaaacaaaaacacaacaaaaacaaaacagccaagggagcctactgta 44218
human.FAM230A.ncRNA.18487127.18500594-GGTLC3.18516335.18518165.rev.compl.REF    ------------------------------------------------------------ 31039

chimp.LOC112206744-LOC107973052-GGT2.revcompl                                   ggtaaagagaagggacaacagcaggttgtgtcaccccgacatcctgctgtgctatgttca 45897
chimp54546-135457.revcompl.Pan.troglodytes.clone.rp43-41g5.GenBank:AC099533.36  GGGCTCAGTTGCCT---GGAGCCCGTTCAGCCATCCCCCAGTTCACTTTGC-------- 48007
human.FAM30B-LOC105372935-GGT2.NCBI.                                            ggtaaagagaaggg---acagcaggttgtgtcaccccgacatcctgctgtgctatgttca 44887
human.FAM230E-LOC105377182-GGT3P.NCBI.                                          ggtaaagagaaggg---acagcaggttgtgtcaccccgacatcctgctgtgctatgttca 43541
human.FAM230J-LOC105372942-GGTLC5P.NCBI.rev.compl                               ggtaaagagaaggg---acagcaggttgtgtcaccccgacatcctgctgtgctatgttca 44275
human.FAM230A.ncRNA.18487127.18500594-GGTLC3.18516335.18518165.rev.compl.REF    ------------------------------------------------------------ 31039

chimp.LOC112206744-LOC107973052-GGT2.revcompl                                   agtctcacttttgagacacaccctgaggtgcgacatcagtaacctactgtggaatccctc 45957
chimp54546-135457.revcompl.Pan.troglodytes.clone.rp43-41g5.GenBank:AC099533.36  ------------------------------------------------------------ 48007
human.FAM30B-LOC105372935-GGT2.NCBI.                                            agtctcacttttgagacacaccctgaggtgcgacatcagtaacctactgtggaatccctc 44947
human.FAM230E-LOC105377182-GGT3P.NCBI.                                          agtctcacttttgagacacaccctgaggtgcgacatcagtaacctactgtggaatccctc 43601
human.FAM230J-LOC105372942-GGTLC5P.NCBI.rev.compl                               agtctcacttttgagacacaccctgaggtgcgacatcagtaacctactgtggaatccctc 44335
human.FAM230A.ncRNA.18487127.18500594-GGTLC3.18516335.18518165.rev.compl.REF    ------------------------------------------------------------ 31039

chimp.LOC112206744-LOC107973052-GGT2.revcompl                                   agaaaaacacgaatcccaatagacgtgggtggagacggagagagttaggaaatccggcag 46017
chimp54546-135457.revcompl.Pan.troglodytes.clone.rp43-41g5.GenBank:AC099533.36  ------------------------------------------------------------ 48007
human.FAM30B-LOC105372935-GGT2.NCBI.                                            agaaaaacacgaatcccaatagatgtgggtggagacggagagagttaggaaatccggcag 45007
human.FAM230E-LOC105377182-GGT3P.NCBI.                                          agaaaaacacgaatcccaatagatgtgggtggagacggagagagttaggaaatccggcag 43661
human.FAM230J-LOC105372942-GGTLC5P.NCBI.rev.compl                               agaaaaacacgaatcccaatagatgtgggtggagacggagagagttaggaaatccggcag 44395
human.FAM230A.ncRNA.18487127.18500594-GGTLC3.18516335.18518165.rev.compl.REF    ------------------------------------------------------------ 31039

chimp.LOC112206744-LOC107973052-GGT2.revcompl                                   aaacgtccacactgcagactccaggaaaagggaacattgatgcttgggcagttttggggt 46077
chimp54546-135457.revcompl.Pan.troglodytes.clone.rp43-41g5.GenBank:AC099533.36  ------------------------------------------------------------ 48007
human.FAM30B-LOC105372935-GGT2.NCBI.                                            aaatgtccacactgcagaatccaggaaaagggaacattgatgcttgggcagttttgggtt 45067
human.FAM230E-LOC105377182-GGT3P.NCBI.                                          aaatgtccacactgcagaatccaggaaaagggaacattgatgcttgggcagttttgggtt 43721
human.FAM230J-LOC105372942-GGTLC5P.NCBI.rev.compl                               aaatgtccacactgcagaatccaggaaaagggaacattgatgcttgggcagttttgggtt 44455
human.FAM230A.ncRNA.18487127.18500594-GGTLC3.18516335.18518165.rev.compl.REF    ------------------------------------------------------------ 31039

chimp.LOC112206744-LOC107973052-GGT2.revcompl                                   atttttacattttgtaggtgtgaaaatttgcaaaatgaaaactcgag--gagtgtggtg 46135
chimp54546-135457.revcompl.Pan.troglodytes.clone.rp43-41g5.GenBank:AC099533.36  -------------------------------------------------------TTGTG 48012
human.FAM30B-LOC105372935-GGT2.NCBI.                                            tttttacattttgtaggtgcgaaaatttgcaaaatgaaaactcgaggagagtgtggtg 45127
human.FAM230E-LOC105377182-GGT3P.NCBI.                                          tttttacattttgtaggtgcgaaaatttgcaaaatgaaaactcgaggagagtgtggtg 43781
human.FAM230J-LOC105372942-GGTLC5P.NCBI.rev.compl                               tttttacattttgtaggtgcgaaaatttgcaaaatgaaaactcgaggagagtgtggtg 44515
human.FAM230A.ncRNA.18487127.18500594-GGTLC3.18516335.18518165.rev.compl.REF    ------------------------------------------------------------ 31039
```

```
chimp.LOC112206744-LOC107973052-GGT2.revcompl                                      agctgtgtgagatgctgctgagtggggcctgatggggaaactgaggctggacatggcgat 46195
chimp54546-135457.revcompl.Pan.troglodytes.clone.rp43-41g5.GenBank:AC099533.36     GGATCTCCCCGTTGCTCCTGCCCGTGGACTGAGTGGCAGGCCATCCTACAAGCACCCGGA 48072
human.FAM30B-LOC105372935-GGT2.NCBI.                                               agctgtgtgagatgctgctgagtggggcctgatggggaaactgaggctggacatggcgat 45187
human.FAM230E-LOC105377182-GGT3P.NCBI.                                             agctgtgtgagatgctgctgagtggggcctgatggggaaactgaggctggacatggcgat 43841
human.FAM230J-LOC105372942-GGTLC5P.NCBI.rev.compl                                  agctgtgtgagatgctgctgagtggggcctgatggggaaactgaggctggacatggcgat 44575
human.FAM230A.ncRNA.18487127.18500594-GGTLC3.18516335.18518165.rev.compl.REF       ------------------------------------------------------------ 31039

chimp.LOC112206744-LOC107973052-GGT2.revcompl                                      ctggtggcatggggatagagcaggggaggggataccctgaagggagagaggacatagccc 46255
chimp54546-135457.revcompl.Pan.troglodytes.clone.rp43-41g5.GenBank:AC099533.36     CACTTGACATCAGTGGTGTCAAGACAACGCTA---------------------------- 48104
human.FAM30B-LOC105372935-GGT2.NCBI.                                               ctggtggcatggggatagagcaggggaggggataccctgaagggagagaggacatagccc 45247
human.FAM230E-LOC105377182-GGT3P.NCBI.                                             ctggtggcatggggatagagcaggggaggggataccctgaagggagagaggacatagccc 43901
human.FAM230J-LOC105372942-GGTLC5P.NCBI.rev.compl                                  ctggtggcatggggatagagcaggggaggggataccctgaagggagagaggacatagccc 44635
human.FAM230A.ncRNA.18487127.18500594-GGTLC3.18516335.18518165.rev.compl.REF       ------------------------------------------------------------ 31039

chimp.LOC112206744-LOC107973052-GGT2.revcompl                                      agccatgtttgctctaagaggagcaaaggacagaaggaggcagcagatagaaatttctag 46315
chimp54546-135457.revcompl.Pan.troglodytes.clone.rp43-41g5.GenBank:AC099533.36     --------------------------------------------AGAAGGTTTTCCGTGA 48120
human.FAM30B-LOC105372935-GGT2.NCBI.                                               agccatgtttgctctaagaggagcaaaggacagaaggaggcagcagatagaagtttctag 45307
human.FAM230E-LOC105377182-GGT3P.NCBI.                                             agccatgtttgctctaagaggagcaaaggacagaaggaggcagcagatagaagtttctag 43961
human.FAM230J-LOC105372942-GGTLC5P.NCBI.rev.compl                                  agccatgtttgctctaagaggagcaaaggacagaaggaggcagcagatagaagtctctag 44695
human.FAM230A.ncRNA.18487127.18500594-GGTLC3.18516335.18518165.rev.compl.REF       ------------------------------------------------------------ 31039

chimp.LOC112206744-LOC107973052-GGT2.revcompl                                      agcaacaacagctgcctttcttttgggaataatccgtgataaagaagtaaatcgtcagag 46375
chimp54546-135457.revcompl.Pan.troglodytes.clone.rp43-41g5.GenBank:AC099533.36     TCCTGCAAGCCCTGCGTTCCTTCCTGGGATCCTGCCTT---------------------- 48158
human.FAM30B-LOC105372935-GGT2.NCBI.                                               agcaacaacagctgcctttctttcgggaataatccgtgataaagaaataaatcatcagag 45367
human.FAM230E-LOC105377182-GGT3P.NCBI.                                             agcaacaacagctgccttttttttgggaataatccgtgataaagaaataaatcatcagag 44021
human.FAM230J-LOC105372942-GGTLC5P.NCBI.rev.compl                                  agcaataacagctgcctttttttttgggaataatccgtgataaagaaataaatcatcagag 44755
human.FAM230A.ncRNA.18487127.18500594-GGTLC3.18516335.18518165.rev.compl.REF       ------------------------------------------------------------ 31039

chimp.LOC112206744-LOC107973052-GGT2.revcompl                                      gcggaaaggagcattgtaggaacagcaccccgagccagcgagtagatgaaggagctggcc 46435
chimp54546-135457.revcompl.Pan.troglodytes.clone.rp43-41g5.GenBank:AC099533.36     -----CAATTTGATTGCACAGGTACCACAGCAAGCCAGTGCTGTGTGCTCCGAGTTCCAG 48213
human.FAM30B-LOC105372935-GGT2.NCBI.                                               gcagacaggagcattgtaggaacagcaccccgagccagtgagtagatgaaggagctggcc 45427
human.FAM230E-LOC105377182-GGT3P.NCBI.                                             gcagacaggagcattgtaggaacagcaccccgagccagcgagtagatgaaggagctggcc 44081
human.FAM230J-LOC105372942-GGTLC5P.NCBI.rev.compl                                  gcagacaggagcattgtaggaacagcaccccgagccagcgagtagatgaaggacctggcc 44815
human.FAM230A.ncRNA.18487127.18500594-GGTLC3.18516335.18518165.rev.compl.REF       ------------------------------------------------------------ 31039

chimp.LOC112206744-LOC107973052-GGT2.revcompl                                      ttagccaaaagggagggcagagggacacgctgcagtggctctgtcccctcagagagacaa 46495
chimp54546-135457.revcompl.Pan.troglodytes.clone.rp43-41g5.GenBank:AC099533.36     GGCGTCCTCCAGCTCAGCCACTGCGCTGAGAACATGGACTCTCT---------------- 48257
human.FAM30B-LOC105372935-GGT2.NCBI.                                               ttagccaaaaaggagggcagagggacacgctgcagtggctctgtcccctcagagagacaa 45487
human.FAM230E-LOC105377182-GGT3P.NCBI.                                             ttagccaaaaaggagggcagagggacacgctgcagtggctctgtcccctcagagagacaa 44141
human.FAM230J-LOC105372942-GGTLC5P.NCBI.rev.compl                                  ttagccaaaaaggagggcagagggacacgctgcagtggctctgtccccttagagagacaa 44875
human.FAM230A.ncRNA.18487127.18500594-GGTLC3.18516335.18518165.rev.compl.REF       ------------------------------------------------------------ 31039

chimp.LOC112206744-LOC107973052-GGT2.revcompl                                      gacaccaggtcactggctgcagcgggagtcagaggtgcagaatgctcacggggaaagaga 46555
chimp54546-135457.revcompl.Pan.troglodytes.clone.rp43-41g5.GenBank:AC099533.36     ---------------------GTGGGGCCCAGGAGCCGGGAGTCACCCCTTTGGGGTCCA 48296
human.FAM30B-LOC105372935-GGT2.NCBI.                                               gacaccaggtcactggctgcagcgggagtcagaggtgcagaatgctcacagggaaagaga 45547
human.FAM230E-LOC105377182-GGT3P.NCBI.                                             gacaccaggtcactggctgcagcgggagtcagaggtgcagaatgctcacagggaaagaga 44201
human.FAM230J-LOC105372942-GGTLC5P.NCBI.rev.compl                                  gacaccaggtcactggctgcagcgggagtcagaggtgcagaatgctcacagggaaagaga 44935
human.FAM230A.ncRNA.18487127.18500594-GGTLC3.18516335.18518165.rev.compl.REF       ------------------------------------------------------------ 31039

chimp.LOC112206744-LOC107973052-GGT2.revcompl                                      agacaccacccgggcagctgacgcccctcctgggaggtcactggtcagtgt------ggg 46609
chimp54546-135457.revcompl.Pan.troglodytes.clone.rp43-41g5.GenBank:AC099533.36     CAACACCCGGCTGTCCCCAGACTTGTGTCCAGGGAAGATAGTGTTGAGGGCCCTCAAGGA 48356
human.FAM30B-LOC105372935-GGT2.NCBI.                                               agacaccacccgggcagctgacgcccctcctgggaggtcactggtcagtgt------ggg 45601
human.FAM230E-LOC105377182-GGT3P.NCBI.                                             agacaccacccgggcagctgacgcccctcctgggaggtcactggtcagtgt------ggg 44255
human.FAM230J-LOC105372942-GGTLC5P.NCBI.rev.compl                                  agacaccacccgggcagctgacgcccctcctgggaggtcactggtcagtgt------ggg 44989
human.FAM230A.ncRNA.18487127.18500594-GGTLC3.18516335.18518165.rev.compl.REF       ------------------------------------------------------------ 31039

chimp.LOC112206744-LOC107973052-GGT2.revcompl                                      ggggtctgcagatccgcccaggaatgccaaggacccaagtaggtaaggagggatgtgagg 46669
chimp54546-135457.revcompl.Pan.troglodytes.clone.rp43-41g5.GenBank:AC099533.36     GAGCGGGGCAGGGATGCCTGAGCAGGACAAGGACCCTAGAGTCCAAGAGAATCCTGGTGA 48416
human.FAM30B-LOC105372935-GGT2.NCBI.                                               ggggtctgcagatccgcccaggaatgccaaggacccaagtaggtaaggagggatgtgagg 45661
human.FAM230E-LOC105377182-GGT3P.NCBI.                                             ggggtctgcagatccgcccaggaatgccaaggacccaagtaggtaaggagggatgtgagg 44315
human.FAM230J-LOC105372942-GGTLC5P.NCBI.rev.compl                                  ggggtctgcagatccgcccaggaatgccaaggacccaagtaggtaaggagggatgtgagg 45049
human.FAM230A.ncRNA.18487127.18500594-GGTLC3.18516335.18518165.rev.compl.REF       ------------------------------------------------------------ 31039

chimp.LOC112206744-LOC107973052-GGT2.revcompl                                      atcctcagcaggaagggatatgacagggtcttatagggacccagcgtggagctggggcaa 46729
chimp54546-135457.revcompl.Pan.troglodytes.clone.rp43-41g5.GenBank:AC099533.36     TCAGAGAAGGGTCCCCGAGGTCACCGGGGATGCACGGTCTGCATTTCGGCCCCTGCGGGA 48476
human.FAM30B-LOC105372935-GGT2.NCBI.                                               atcctcagtgggaagggatatgacagggtcttatagggacccagcatggagctggggcaa 45721
human.FAM230E-LOC105377182-GGT3P.NCBI.                                             atcctcagcgggaagggatatgacagggtcttatagggacccagcatggagctggggcaa 44375
human.FAM230J-LOC105372942-GGTLC5P.NCBI.rev.compl                                  atcctcagcgggaagggatatgacagggtcttatagggacccagcatggagctggggcaa 45109
human.FAM230A.ncRNA.18487127.18500594-GGTLC3.18516335.18518165.rev.compl.REF       ------------------------------------------------------------ 31039

chimp.LOC112206744-LOC107973052-GGT2.revcompl                                      ctactgagtgagtgggtcaggtggtgcatggctgagggtggcatctgggaagcgttagtt 46789
chimp54546-135457.revcompl.Pan.troglodytes.clone.rp43-41g5.GenBank:AC099533.36     C-AATGGAGGCCTCTCTCCCTTTGTGCCCAGGCCCGGGCCTCTGCAGACAGACCTCCATG 48535
human.FAM30B-LOC105372935-GGT2.NCBI.                                               ctactgggtgagtgggtcaggtggtgcagggctgagggtggcatctgggaagcattagtt 45781
human.FAM230E-LOC105377182-GGT3P.NCBI.                                             ctactgggtgagtgggtcaggtggtgcagggctgagggtggcatctgggaagcgttagtt 44435
human.FAM230J-LOC105372942-GGTLC5P.NCBI.rev.compl                                  ctactgggtgagtgggtcaggtggtgcagggctgagggtggcatctgggaagcgttagtt 45169
human.FAM230A.ncRNA.18487127.18500594-GGTLC3.18516335.18518165.rev.compl.REF       ------------------------------------------------------------ 31039

chimp.LOC112206744-LOC107973052-GGT2.revcompl                                      tggagtgacagggagtggg----tggccaaggtctctgttcacctggccctctccctttc 46845
chimp54546-135457.revcompl.Pan.troglodytes.clone.rp43-41g5.GenBank:AC099533.36     CCCAGAGGTCAGAAATCAGATATAAGCAGACATCCCAGACCTCCTGGACGAGCTCCT--- 48592
human.FAM30B-LOC105372935-GGT2.NCBI.                                               tggagtgacagggagtggg----tggccgaggtctctgttcacctggccctctccctttc 45837
human.FAM230E-LOC105377182-GGT3P.NCBI.                                             tggagtgacagggagtggg----tggccgaggtctctgttcacctggccctctccctttc 44491
human.FAM230J-LOC105372942-GGTLC5P.NCBI.rev.compl                                  tggagtgacagggagtggg----tggccgaggtctctgttcacctggccctctccctttc 45225
human.FAM230A.ncRNA.18487127.18500594-GGTLC3.18516335.18518165.rev.compl.REF       ------------------------------------------------------------ 31039

chimp.LOC112206744-LOC107973052-GGT2.revcompl                                      acccatccatctcaccatgcccgagggctgcaatgcccccatgcattcctcatctcagca 46905
chimp54546-135457.revcompl.Pan.troglodytes.clone.rp43-41g5.GenBank:AC099533.36     ---------------------------------------GCACCAACCGAAATGCCATCT 48613
human.FAM30B-LOC105372935-GGT2.NCBI.                                               acccatccatctcaccatgcccgagggctgcaatgcccccatgcattcctcatctcagca 45897
human.FAM230E-LOC105377182-GGT3P.NCBI.                                             acccatccatctcaccatgcccgagggctgcaatgcccccatgcattcctcatctcagca 44551
human.FAM230J-LOC105372942-GGTLC5P.NCBI.rev.compl                                  acccatccatctcaccatgcccgagggctgcaatgcccccatgcattcctcatctcagca 45285
human.FAM230A.ncRNA.18487127.18500594-GGTLC3.18516335.18518165.rev.compl.REF       ------------------------------------------------------------ 31039

chimp.LOC112206744-LOC107973052-GGT2.revcompl                                      ctgagcactcgagaaccacagcatgtgcaaaggacctgaggtaggagggtggccactaat 46965
chimp54546-135457.revcompl.Pan.troglodytes.clone.rp43-41g5.GenBank:AC099533.36     CCAGCTCCTACAGCTCCACGGGAGGCTTGCCGGGGCTAAAG-CGGAGGAGGGGGCCAGCC 48672
human.FAM30B-LOC105372935-GGT2.NCBI.                                               ctgagcactcaagaaccacagcatgtgcaaaggacctgaggtaggagggtggccactaat 45957
human.FAM230E-LOC105377182-GGT3P.NCBI.                                             ctgagcactcaagaaccacagcatgtgcaaaggacctgaggtaggagggtggccactaat 44611
human.FAM230J-LOC105372942-GGTLC5P.NCBI.rev.compl                                  ctgagcactcaagaaccacagcatgtgcaaaggacctgaggtaggagggtggccactaat 45345
human.FAM230A.ncRNA.18487127.18500594-GGTLC3.18516335.18518165.rev.compl.REF       ------------------------------------------------------------ 31039

chimp.LOC112206744-LOC107973052-GGT2.revcompl                                      tccccacactgtcagttctgtgcggcagaagccaagactgggggtcacccacgagtccat 47025
chimp54546-135457.revcompl.Pan.troglodytes.clone.rp43-41g5.GenBank:AC099533.36     TCATCCCACTGCCAGCTGACCCTCAGTTCCTCAAAGACAGTGAGTGA------------- 48719
human.FAM30B-LOC105372935-GGT2.NCBI.                                               tccccgcactgtcagttctgtggggcagaagccaagactgggggtcacccacgagtccat 46017
human.FAM230E-LOC105377182-GGT3P.NCBI.                                             tccccacactgtcagttctgtggggcagaagccaagactgggggtcacccacaagtccat 44671
human.FAM230J-LOC105372942-GGTLC5P.NCBI.rev.compl                                  tccccacactgtcagttctgtggggcagaagccaagactgggggtcacccacgagtccat 45405
human.FAM230A.ncRNA.18487127.18500594-GGTLC3.18516335.18518165.rev.compl.REF       ------------------------------------------------------------ 31039

chimp.LOC112206744-LOC107973052-GGT2.revcompl                                      ccgagcacacagtaggcccgcaatcaaagtctgtggcaggaatggattcataaagcatat 47085
chimp54546-135457.revcompl.Pan.troglodytes.clone.rp43-41g5.GenBank:AC099533.36     ------------------------------------------------------------ 48719
human.FAM30B-LOC105372935-GGT2.NCBI.                                               ccgagcacacagtaggcccgcaatcgaattctgtggcaggaatggattcataaagcatat 46077
human.FAM230E-LOC105377182-GGT3P.NCBI.                                             ccgagcacacagtaggcccgcaatcaaattctgtggcaggaatggattcataaagcatat 44731
human.FAM230J-LOC105372942-GGTLC5P.NCBI.rev.compl                                  ccgagcacacagtaggcccgcaatcaaattctgtggcaggaatggattcataaagcatat 45465
human.FAM230A.ncRNA.18487127.18500594-GGTLC3.18516335.18518165.rev.compl.REF       ------------------------------------------------------------ 31039

chimp.LOC112206744-LOC107973052-GGT2.revcompl                                      gtgaggctgtagccaccctggggagcccacctgaggcctctacagcaggc-cccacaccc 47144
chimp54546-135457.revcompl.Pan.troglodytes.clone.rp43-41g5.GenBank:AC099533.36     -------------------GGACAGGCCTCAGGCTGTCTCTTCGGGTCACACCCAGTGTG 48760
human.FAM30B-LOC105372935-GGT2.NCBI.                                               gtgaggctgtagccaccctggggagcccacctgatgcctctacggcaggc-cccacaccc 46136
human.FAM230E-LOC105377182-GGT3P.NCBI.                                             gtgaggctgtagccaccctggggagcccacctgatgcctctacggcaggc-cccacaccc 44790
human.FAM230J-LOC105372942-GGTLC5P.NCBI.rev.compl                                  gtgaggctgtagccaccctggggagcccacctgatgcctctacggcaggc-cccacaccc 45524
human.FAM230A.ncRNA.18487127.18500594-GGTLC3.18516335.18518165.rev.compl.REF       ------------------------------------------------------------ 31039

chimp.LOC112206744-LOC107973052-GGT2.revcompl                                      acagtgggccagcccctgccccttaccccagtcacatccacaaacttggggtcccca-ag 47203
chimp54546-135457.revcompl.Pan.troglodytes.clone.rp43-41g5.GenBank:AC099533.36     AAAAGGCAGCAGATATAGCACCAGGGCAGACACTCGCCCTCAGGAATGACTCCTCCACAT 48820
human.FAM30B-LOC105372935-GGT2.NCBI.                                               acagtgagccagcccctgccccttacctcagtcacatccacaaacttggggtcccca-ag 46195
human.FAM230E-LOC105377182-GGT3P.NCBI.                                             acagtgggccagcccctgccccttacctcagtcacatccacaaacttggggtcccca-ag 44849
human.FAM230J-LOC105372942-GGTLC5P.NCBI.rev.compl                                  acagtgagccagcccctgccccttacctcagtcacatccacaaacttggggtcccca-ag 45583
human.FAM230A.ncRNA.18487127.18500594-GGTLC3.18516335.18518165.rev.compl.REF       ------------------------------------------------------------ 31039
```

```
chimp.LOC112206744-LOC107973052-GGT2.revcompl                                  cagggtcctcttggcgtaggcaaaccggaaaggcctctacgatgtggtggtacgtcaggc 47263
chimp54546-135457.revcompl.Pan.troglodytes.clone.rp43-41g5.GenBank:AC099533.36 CCGAGGCCTCTAGGCCCAGTACACGCAAGTT-TCCCCTGCTGCCACGCAGGCGAGGGGAG 48879
human.FAM30B-LOC105372935-GGT2.NCBI.                                           cagggtcctcttggcgtaggcaaaccggaaa-gcctctacgatgcggtggtacgtcaggc 46254
human.FAM230E-LOC105377182-GGT3P.NCBI.                                         cagggtcctcttggcgtaggcaaaccggaaa-gcctctacgatgcggtggtacgtcaggc 44908
human.FAM230J-LOC105372942-GGTLC5P.NCBI.rev.compl                              cagggtcctcttggcgtaggcaaaccggaaa-gcctctacgatgcggtggtacgtcaggc 45642
human.FAM230A.ncRNA.18487127.18500594-GGTLC3.18516335.18518165.rev.compl.REF   ------------------------------------------------------------ 31039

chimp.LOC112206744-LOC107973052-GGT2.revcompl                                  ccttctgcttgggggtctccacgctctcctgggagaagttgtaccctggttgatcagaac 47323
chimp54546-135457.revcompl.Pan.troglodytes.clone.rp43-41g5.GenBank:AC099533.36 CCTTTGATGCTGCCACCTCCCGTAGAGCTGGGGTACCGGGTCACTGTTGAAGACCTGGAC 48939
human.FAM30B-LOC105372935-GGT2.NCBI.                                           ccttctgctcgggggtctccacgctctcccgggagaagttgtaccctggttgatcagagc 46314
human.FAM230E-LOC105377182-GGT3P.NCBI.                                         ccttctgctcgggggtctccacgctctcccgggagaagttgtaccctggttgatcagagc 44968
human.FAM230J-LOC105372942-GGTLC5P.NCBI.rev.compl                              ccttctgctcgggggtctccacgctctcccgggagaagttgtaccctggttgatcagagc 45702
human.FAM230A.ncRNA.18487127.18500594-GGTLC3.18516335.18518165.rev.compl.REF   ------------------------------------------------------------ 31039

chimp.LOC112206744-LOC107973052-GGT2.revcompl                                  caggtgcatgttgctgagccccagaggctctgaggggctcagagggtt-aacacctgcct 47382
chimp54546-135457.revcompl.Pan.troglodytes.clone.rp43-41g5.GenBank:AC099533.36 CGGGAGAAGGAGGCGGCCTTCCAGCGCATCAACAGTGCACTGCAGGTTGAGGACAAGGCC 48999
human.FAM30B-LOC105372935-GGT2.NCBI.                                           caggtgcatgttgctgagccccagaggctctgaggggctcagagggtt-aacacctgcct 46373
human.FAM230E-LOC105377182-GGT3P.NCBI.                                         caggtgcatgttgctgagccccagaggctctgaggggctcagagggtt-aacacctgcct 45027
human.FAM230J-LOC105372942-GGTLC5P.NCBI.rev.compl                              caggtgcatgttgctgagccccagaggctctgaggggctcagagggtt-aacacctgcct 45761
human.FAM230A.ncRNA.18487127.18500594-GGTLC3.18516335.18518165.rev.compl.REF   ------------------------------------------------------------ 31039

chimp.LOC112206744-LOC107973052-GGT2.revcompl                                  gagccactttgcccacctcaaggagcgtttaataaccaatagcagcagctgcttcagaag 47442
chimp54546-135457.revcompl.Pan.troglodytes.clone.rp43-41g5.GenBank:AC099533.36 ATCTCGGACTGCAGACCCTCACGGCC--TTCCCACACTTTGTCCTCACTTGCAACAGGGG 49057
human.FAM30B-LOC105372935-GGT2.NCBI.                                           gagccactttgcccacctcaaggagcgtttaataaccaatagcagcagctgcttcagaag 46433
human.FAM230E-LOC105377182-GGT3P.NCBI.                                         gagccactttgcccacctcaaggagcgtttaataatcaatagcagcagctgcttcagaag 45087
human.FAM230J-LOC105372942-GGTLC5P.NCBI.rev.compl                              gagccactttgcccacctcaaggagcgtttaataaccaatagcagcagctgcttcagaag 45821
human.FAM230A.ncRNA.18487127.18500594-GGTLC3.18516335.18518165.rev.compl.REF   ------------------------------------------------------------ 31039

chimp.LOC112206744-LOC107973052-GGT2.revcompl                                  gctgtggtgagagtgaagtaaggtgagggctcccga-gcctgggcctcacgtcatgcatc 47501
chimp54546-135457.revcompl.Pan.troglodytes.clone.rp43-41g5.GenBank:AC099533.36 CTTCTGGTCTGCCTGCCGTTTCTAAAGCACCCAGTATGGATGCACAGCAGGAGACACACA 49117
human.FAM30B-LOC105372935-GGT2.NCBI.                                           gctgtggtgagagtgaagtaaggtgagggctcccga-gcctggacctcacgtcacgcatc 46492
human.FAM230E-LOC105377182-GGT3P.NCBI.                                         gctgtggtgagagtgaagtaaggtgagggctcccga-gcctggacctcacgtcacgcatc 45146
human.FAM230J-LOC105372942-GGTLC5P.NCBI.rev.compl                              gctgtggtgagagtgaagtaaggtgagggctcctga-gcctggacctcacgtcacgcatc 45880
human.FAM230A.ncRNA.18487127.18500594-GGTLC3.18516335.18518165.rev.compl.REF   ------------------------------------------------------------ 31039

chimp.LOC112206744-LOC107973052-GGT2.revcompl                                  agctctggccattcagtgaccaagtggcagggtcacccactgg-accagggtaccctctg 47560
chimp54546-135457.revcompl.Pan.troglodytes.clone.rp43-41g5.GenBank:AC099533.36 AGTCCCAAGACTGCGTGGGCCTAGTGGCCCCCTTAGCATCTGCTGCAGAGGTCCCCTCTA 49177
human.FAM30B-LOC105372935-GGT2.NCBI.                                           agctctggccattcaatgaccaagtggcagggtcacccactgg-accggggtaccctctg 46551
human.FAM230E-LOC105377182-GGT3P.NCBI.                                         agctctggccattcaatgaccaagtggcagggtcacccactgg-actggggtaccctctg 45205
human.FAM230J-LOC105372942-GGTLC5P.NCBI.rev.compl                              agctctggccattcaatgaccaagtggcagggtcacccactgg-accggggtaccctctg 45939
human.FAM230A.ncRNA.18487127.18500594-GGTLC3.18516335.18518165.rev.compl.REF   ------------------------------------------------------------ 31039

chimp.LOC112206744-LOC107973052-GGT2.revcompl                                  gaccagccctg----------------------cagccctgagctcctgcacctctctcc 47598
chimp54546-135457.revcompl.Pan.troglodytes.clone.rp43-41g5.GenBank:AC099533.36 CAGCTCCCATGTCTGGGAAGAAGCACAGACCACCAGGCCCCCTGTTCTCCTCCTCAGGTC 49237
human.FAM30B-LOC105372935-GGT2.NCBI.                                           gaccggccctg----------------------cagccctgagctcctgcacctctctcc 46589
human.FAM230E-LOC105377182-GGT3P.NCBI.                                         gaccggccctg----------------------cagccctgagctcctgcacctctctcc 45243
human.FAM230J-LOC105372942-GGTLC5P.NCBI.rev.compl                              gaccggccctg----------------------cagccctgagctcctgcacctctctcc 45977
human.FAM230A.ncRNA.18487127.18500594-GGTLC3.18516335.18518165.rev.compl.REF   ------------------------------------------------------------ 31039

chimp.LOC112206744-LOC107973052-GGT2.revcompl                                  ctcctgatgactcctgttcctcctccaacccttgagcattgcccgcttcagccctgtcgc 47658
chimp54546-135457.revcompl.Pan.troglodytes.clone.rp43-41g5.GenBank:AC099533.36 CCCTTCCTGCCACCTCTTCCCATTCCCAGGACTCAGCCCAGGTCACCTC--GCTGATTCC 49295
human.FAM30B-LOC105372935-GGT2.NCBI.                                           ctcctgatgactcctgttcctcctccaacccttgagcattgcccgcttcagccctgttgc 46649
human.FAM230E-LOC105377182-GGT3P.NCBI.                                         ctcctgatgactcctgttcctcctccaacccttgagcattgcccgcttcagccctgttgc 45303
human.FAM230J-LOC105372942-GGTLC5P.NCBI.rev.compl                              ctcctgatgactcctgttcctcctccaacccttgagcattgcccgcttcagccctgttgc 46037
human.FAM230A.ncRNA.18487127.18500594-GGTLC3.18516335.18518165.rev.compl.REF   ------------------------------------------------------------ 31039

chimp.LOC112206744-LOC107973052-GGT2.revcompl                                  tctgcactgcctccttcaggacatgg---------------------------------- 47684
chimp54546-135457.revcompl.Pan.troglodytes.clone.rp43-41g5.GenBank:AC099533.36 TGCCCCCTTCCCAGCTGCAAGCATGGATGCGGGCATGAGAAGAACAAGGCCTGGCACTTC 49355
human.FAM30B-LOC105372935-GGT2.NCBI.                                           tctgcactgcctccttcaggacatgg---------------------------------- 46675
human.FAM230E-LOC105377182-GGT3P.NCBI.                                         tctgcactgcctccttcaggacatgg---------------------------------- 45329
human.FAM230J-LOC105372942-GGTLC5P.NCBI.rev.compl                              tctgcactgcctccttcaggacatgg---------------------------------- 46063
human.FAM230A.ncRNA.18487127.18500594-GGTLC3.18516335.18518165.rev.compl.REF   ------------------------------------------------------------ 31039

chimp.LOC112206744-LOC107973052-GGT2.revcompl                                  ------------------------------------------------------------ 47684
chimp54546-135457.revcompl.Pan.troglodytes.clone.rp43-41g5.GenBank:AC099533.36 TGCTCCTGCAGCTGCCGCAGCAGCCCCTCCCCCCTCCACATTGAACCCCACGTTGGGGTC 49415
human.FAM30B-LOC105372935-GGT2.NCBI.                                           ------------------------------------------------------------ 46675
human.FAM230E-LOC105377182-GGT3P.NCBI.                                         ------------------------------------------------------------ 45329
human.FAM230J-LOC105372942-GGTLC5P.NCBI.rev.compl                              ------------------------------------------------------------ 46063
human.FAM230A.ncRNA.18487127.18500594-GGTLC3.18516335.18518165.rev.compl.REF   ------------------------------------------------------------ 31039

chimp.LOC112206744-LOC107973052-GGT2.revcompl                                  ------------------------------------------------------------ 47684
chimp54546-135457.revcompl.Pan.troglodytes.clone.rp43-41g5.GenBank:AC099533.36 ACTACTGGAGTGGATGGAGGCCCTTCACATTTCTGGGCCTCAGCCACAGCTGCAGCAGGT 49475
human.FAM30B-LOC105372935-GGT2.NCBI.                                           ------------------------------------------------------------ 46675
human.FAM230E-LOC105377182-GGT3P.NCBI.                                         ------------------------------------------------------------ 45329
human.FAM230J-LOC105372942-GGTLC5P.NCBI.rev.compl                              ------------------------------------------------------------ 46063
human.FAM230A.ncRNA.18487127.18500594-GGTLC3.18516335.18518165.rev.compl.REF   ------------------------------------------------------------ 31039

chimp.LOC112206744-LOC107973052-GGT2.revcompl                                  -----------tgagctgtgatgcagggacacgcctcgggagctcagtgatggaaagacg 47733
chimp54546-135457.revcompl.Pan.troglodytes.clone.rp43-41g5.GenBank:AC099533.36 GCCCAGAGGTCAGAACCAGAGATCCCAGACCTCCTGGACCAGCTCGTGCCCCAAACGAAA 49535
human.FAM30B-LOC105372935-GGT2.NCBI.                                           -----------tgagctgtgacgcagggacacacctcaggagctcagtgatggaaagacg 46724
human.FAM230E-LOC105377182-GGT3P.NCBI.                                         -----------tgagctgtgacgcagggacacacctcaggagctcagtgatggaaagacg 45378
human.FAM230J-LOC105372942-GGTLC5P.NCBI.rev.compl                              -----------tgagctgtgacgcagggacacacctcaggagctcagtgatggaaagacg 46112
human.FAM230A.ncRNA.18487127.18500594-GGTLC3.18516335.18518165.rev.compl.REF   ------------------------------------------------------------ 31039

chimp.LOC112206744-LOC107973052-GGT2.revcompl                                  tggcatgggggcgagcagagatgcagcaggggtggggcgtggggagagagaggcagtgt 47793
chimp54546-135457.revcompl.Pan.troglodytes.clone.rp43-41g5.GenBank:AC099533.36 TGCCATCTCGAGCTCCTGCAGCTCTACGGGAGGCCTCCCGGGACGAAAGCGGAAGAGGGG 49595
human.FAM30B-LOC105372935-GGT2.NCBI.                                           tggcatgggggcgagcagagatgcaggaggggtggggcgtggggagagagaggcagtgt 46784
human.FAM230E-LOC105377182-GGT3P.NCBI.                                         tggcatgggggcgagcagagatgcaggaggggtggggcgtggggagagagaggcagtgt 45438
human.FAM230J-LOC105372942-GGTLC5P.NCBI.rev.compl                              tggcatgggggcgagcagagatgcaggaggggtggggcgtggggagagagaggcagtgt 46172
human.FAM230A.ncRNA.18487127.18500594-GGTLC3.18516335.18518165.rev.compl.REF   ------------------------------------------------------------ 31039

chimp.LOC112206744-LOC107973052-GGT2.revcompl                                  catgggtcctaccacacggctgtggtgcgaccacccacctttgaggatgttgaggatgag 47853
chimp54546-135457.revcompl.Pan.troglodytes.clone.rp43-41g5.GenBank:AC099533.36 CCAGCCTCATCCCACTGCCA---GCTGACCCTCAGTTCCTCAAACACAGTGAGTGAGGAC 49652
human.FAM30B-LOC105372935-GGT2.NCBI.                                           catgggtcctaccacacggctgtggtgcgaccactcacctttgaggatgttgaggatgag 46844
human.FAM230E-LOC105377182-GGT3P.NCBI.                                         catgggtcctaccacacggctgtggtgcgaccactcacctttgaggatgttgaggatgag 45498
human.FAM230J-LOC105372942-GGTLC5P.NCBI.rev.compl                              catgggtcctaccacacggctgtggtgcgaccactcacctttgaggatgttgaggatgag 46232
human.FAM230A.ncRNA.18487127.18500594-GGTLC3.18516335.18518165.rev.compl.REF   ------------------------------------------------------------ 31039

chimp.LOC112206744-LOC107973052-GGT2.revcompl                                  ggccagcacgggcccgctgagcggcgcact-------------gggcatgtacagcaccg 47900
chimp54546-135457.revcompl.Pan.troglodytes.clone.rp43-41g5.GenBank:AC099533.36 GGACCTCAGGCTGTCTCTTCGGGTCACACCCAGTGTGAAAAGACGGCAGATACAGCACCA 49712
human.FAM30B-LOC105372935-GGT2.NCBI.                                           ggccagcacgggcccgctgagccgcgcact-------------gggcatgtacagcaccg 46891
human.FAM230E-LOC105377182-GGT3P.NCBI.                                         ggccagcacgggcccgctgagccgcgcact-------------gggcatgtacagcaccg 45545
human.FAM230J-LOC105372942-GGTLC5P.NCBI.rev.compl                              ggccagcacgggcccgctgagccgcgcact-------------gggcatgtacagcaccg 46279
human.FAM230A.ncRNA.18487127.18500594-GGTLC3.18516335.18518165.rev.compl.REF   ------------------------------------------------------------ 31039

chimp.LOC112206744-LOC107973052-GGT2.revcompl                                  cgtc---------tcccaggctgatgttcagcgggtgctcgatcagctcagcacggtagt 47951
chimp54546-135457.revcompl.Pan.troglodytes.clone.rp43-41g5.GenBank:AC099533.36 GGGCAGACACTCGCCCCCAGGGGTGGCTCCCCCAGATCCCAGGCCTCTAGGCCCTGTAGA 49772
human.FAM30B-LOC105372935-GGT2.NCBI.                                           cgtc---------tcccaggctgatgttcagcgggtgctcgatcagctcagcacggtagt 46942
human.FAM230E-LOC105377182-GGT3P.NCBI.                                         cgtc---------tcccaggctgatgttcagcgggtgctcgatcagctcagcacagtagt 45596
human.FAM230J-LOC105372942-GGTLC5P.NCBI.rev.compl                              cgtc---------tcccaggctgatgttcagcgggtgctcgatcagctcagcacggtagt 46330
human.FAM230A.ncRNA.18487127.18500594-GGTLC3.18516335.18518165.rev.compl.REF   ------------------------------------------------------------ 31039

chimp.LOC112206744-LOC107973052-GGT2.revcompl                                  tgttcaggtcctcagctgtcacaatgccccctgcaatgggacagcagctcgaatgggcgc 48011
chimp54546-135457.revcompl.Pan.troglodytes.clone.rp43-41g5.GenBank:AC099533.36 TGCAAGTTTCCCCTGCTGCCACG------------------------------------- 49795
human.FAM30B-LOC105372935-GGT2.NCBI.                                           tgttcaggtcctcagctgtcacaatgccccctgcaatgggacagcagctcgaatgggtgc 47002
human.FAM230E-LOC105377182-GGT3P.NCBI.                                         tgttcaggtcctcagctgtcacaatgccccctgcaatgggacagcagctcgaatgggcgc 45656
human.FAM230J-LOC105372942-GGTLC5P.NCBI.rev.compl                              tgttcaggtcctcagctgtcacaatgccccctgc-------------------------- 46364
human.FAM230A.ncRNA.18487127.18500594-GGTLC3.18516335.18518165.rev.compl.REF   ------------------------------------------------------------ 31039

chimp.LOC112206744-LOC107973052-GGT2.revcompl                                  tgggatggggctgcaccactgcgtggaggatggagctgcaccagtggtgttgggggcagg 48071
chimp54546-135457.revcompl.Pan.troglodytes.clone.rp43-41g5.GenBank:AC099533.36 ------CAGGCGAGGGGAGCCTTTGATGCTGCCACCTCCCTTAGAGCTGGGGTACCGGGT 49849
human.FAM30B-LOC105372935-GGT2.NCBI.                                           tgggatggggctgcaccactgcgtggaggatggaactgcaccagtggtgttgggggcagg 47062
human.FAM230E-LOC105377182-GGT3P.NCBI.                                         tgggatggggctgcaccactgcgtggaggatggagctgcaccagtggtgttgggggcagg 45716
human.FAM230J-LOC105372942-GGTLC5P.NCBI.rev.compl                              ------------------------------------------------------------ 46364
human.FAM230A.ncRNA.18487127.18500594-GGTLC3.18516335.18518165.rev.compl.REF   ------------------------------------------------------------ 31039

chimp.LOC112206744-LOC107973052-GGT2.revcompl                                  catggctgcaccatggtggtggggaaaagcctgtacctaccagggaggacagagtgcact 48131
```

chimp54546-135457.revcompl.Pan.troglodytes.clone.rp43-41g5.GenBank:AC099533.36 CACTGCTGAAGACCTGGACCCGGAGAAGGAGGCGGCATTCCAGCGCATCAACAGTGCACT 49909
human.FAM30B-LOC105372935-GGT2.NCBI. catggctgcaccatggtggtggggaaaagcctgtacctaccagggaggacagagtgcact 47122
human.FAM230E-LOC105377182-GGT3P.NCBI. catggctgcaccatggtggtggggaaaagcctgtacctaccagggaggacagagtgcact 45776
human.FAM230J-LOC105372942-GGTLC5P.NCBI.rev.compl -- 46364
human.FAM230A.ncRNA.18487127.18500594-GGTLC3.18516335.18518165.rev.compl.REF -- 31039

chimp.LOC112206744-LOC107973052-GGT2.revcompl actggaggggtgggactgtgccctgggaggggccacaggcaacctcacctc-cttggga 48190
chimp54546-135457.revcompl.Pan.troglodytes.clone.rp43-41g5.GenBank:AC099533.36 GCAGGTTGAGGACAAGGCCATCTCGGACTGCAGACCCTCACGGCCTTCCCACACTTTGTC 49969
human.FAM30B-LOC105372935-GGT2.NCBI. actggaggggtgggactgtgccctgggaggggccacaggcaacctcacctc-cttggga 47181
human.FAM230E-LOC105377182-GGT3P.NCBI. actggaggggtgggactgtgccctgggaggggccacaggcaacctcacctc-cttggga 45835
human.FAM230J-LOC105372942-GGTLC5P.NCBI.rev.compl -- 46364
human.FAM230A.ncRNA.18487127.18500594-GGTLC3.18516335.18518165.rev.compl.REF -- 31039

chimp.LOC112206744-LOC107973052-GGT2.revcompl acctcaccagctccggcactcctgtctccctgacactgctcaccaccc------------ 48238
chimp54546-135457.revcompl.Pan.troglodytes.clone.rp43-41g5.GenBank:AC099533.36 CTCACTTGCAACAGGGGCTTCTGGTCTGCCTGCCGTTTCTAAAGCACCCAGTATGGATGC 50029
human.FAM30B-LOC105372935-GGT2.NCBI. acctcaccagctccggcactcctgtctccctgacactgctcaccaccc------------ 47229
human.FAM230E-LOC105377182-GGT3P.NCBI. acctcaccagctccggcactcctgtctccctgacactgctcaccaccc------------ 45883
human.FAM230J-LOC105372942-GGTLC5P.NCBI.rev.compl -- 46364
human.FAM230A.ncRNA.18487127.18500594-GGTLC3.18516335.18518165.rev.compl.REF -- 31039

chimp.LOC112206744-LOC107973052-GGT2.revcompl -- 48238
chimp54546-135457.revcompl.Pan.troglodytes.clone.rp43-41g5.GenBank:AC099533.36 ACAGCAGGAGACACACAAGTCCCAAGACTGCCTGGGCCTAGTGGCCCCCCTGCATCTGCT 50089
human.FAM30B-LOC105372935-GGT2.NCBI. -- 47229
human.FAM230E-LOC105377182-GGT3P.NCBI. -- 45883
human.FAM230J-LOC105372942-GGTLC5P.NCBI.rev.compl -- 46364
human.FAM230A.ncRNA.18487127.18500594-GGTLC3.18516335.18518165.rev.compl.REF -- 31039

chimp.LOC112206744-LOC107973052-GGT2.revcompl -- 48238
chimp54546-135457.revcompl.Pan.troglodytes.clone.rp43-41g5.GenBank:AC099533.36 GCACAGGCCTGTAGTCCCAGCTACTTGGGAGGCTGAGGCAGGAGAACGGCATAAACCCGG 50149
human.FAM30B-LOC105372935-GGT2.NCBI. -- 47229
human.FAM230E-LOC105377182-GGT3P.NCBI. -- 45883
human.FAM230J-LOC105372942-GGTLC5P.NCBI.rev.compl -- 46364
human.FAM230A.ncRNA.18487127.18500594-GGTLC3.18516335.18518165.rev.compl.REF -- 31039

chimp.LOC112206744-LOC107973052-GGT2.revcompl ---------------gacagctgggctggggccacctgccctctgcctgcttggcttact 48283
chimp54546-135457.revcompl.Pan.troglodytes.clone.rp43-41g5.GenBank:AC099533.36 GAGGCAGAGCTTGCAGTGAGCTGAGATCGCGCCACTGCACTCCAGCCTGGGTGACAGAGC 50209
human.FAM30B-LOC105372935-GGT2.NCBI. ---------------gacagctgggctggggccacctgccctctgcctgcttggcttact 47274
human.FAM230E-LOC105377182-GGT3P.NCBI. ---------------gacagctgggctggggccacctgccctctgcctgcttggcttact 45928
human.FAM230J-LOC105372942-GGTLC5P.NCBI.rev.compl -- 46364
human.FAM230A.ncRNA.18487127.18500594-GGTLC3.18516335.18518165.rev.compl.REF -- 31039

chimp.LOC112206744-LOC107973052-GGT2.revcompl ggcttcctgtctg--- 48296
chimp54546-135457.revcompl.Pan.troglodytes.clone.rp43-41g5.GenBank:AC099533.36 GAGACTCCGTCTCAAAAAAAAAGAAAAAGAAAAAAAGTTCTTGTGACATTTGTGTATGAA 50269
human.FAM30B-LOC105372935-GGT2.NCBI. ggcttcctgtctg--- 47287
human.FAM230E-LOC105377182-GGT3P.NCBI. ggcttcctgtctg--- 45941
human.FAM230J-LOC105372942-GGTLC5P.NCBI.rev.compl -- 46364
human.FAM230A.ncRNA.18487127.18500594-GGTLC3.18516335.18518165.rev.compl.REF -- 31039

chimp.LOC112206744-LOC107973052-GGT2.revcompl -------------------------ccttctctcatctgtggccggagagtg-------- 48323
chimp54546-135457.revcompl.Pan.troglodytes.clone.rp43-41g5.GenBank:AC099533.36 ATCAGCCTTCACTACATGGATAGGACCAGCACGCTTCTGCGGCACGACTCTGCCATCTTA 50329
human.FAM30B-LOC105372935-GGT2.NCBI. -------------------------ccttctctcatctgtggccagagagtg-------- 47314
human.FAM230E-LOC105377182-GGT3P.NCBI. -------------------------ccttctctcatctgtggccagagagtg-------- 45968
human.FAM230J-LOC105372942-GGTLC5P.NCBI.rev.compl -- 46364
human.FAM230A.ncRNA.18487127.18500594-GGTLC3.18516335.18518165.rev.compl.REF -- 31039

chimp.LOC112206744-LOC107973052-GGT2.revcompl -----------tttcctttttctttttttttttttagagatacgatcttgctctgttacc 48372
chimp54546-135457.revcompl.Pan.troglodytes.clone.rp43-41g5.GenBank:AC099533.36 CTACATTTTTTTTATTTTGTATTTTTTTTATTCCTTTTGAGACAGTCTCACTCTGTCACC 50389
human.FAM30B-LOC105372935-GGT2.NCBI. -----------ttttctttttctttttttttttttagagatacgatcttgctctgttacc 47363
human.FAM230E-LOC105377182-GGT3P.NCBI. -----------ttttctttttc-ttttttttttttagagatacgatcttgctctgttacc 46016
human.FAM230J-LOC105372942-GGTLC5P.NCBI.rev.compl -- 46364
human.FAM230A.ncRNA.18487127.18500594-GGTLC3.18516335.18518165.rev.compl.REF -- 31039

chimp.LOC112206744-LOC107973052-GGT2.revcompl caggctggagtgcag--------tggcttaatcacagccttgaactcccgggctcaagtg 48424
chimp54546-135457.revcompl.Pan.troglodytes.clone.rp43-41g5.GenBank:AC099533.36 CAGGCTGAAGTGCAGCCGAGATCTCGGCTCACTGCAACCTCCACCTCTTGGGTTCAAGCA 50449
human.FAM30B-LOC105372935-GGT2.NCBI. caggctggagtgcag--------tggcttaatcacagccttgaacccctgggctcaagtg 47415
human.FAM230E-LOC105377182-GGT3P.NCBI. caggctggagtgcag--------tggcttaatcacagccttgaacccctgggctcaagtg 46068
human.FAM230J-LOC105372942-GGTLC5P.NCBI.rev.compl -- 46364
human.FAM230A.ncRNA.18487127.18500594-GGTLC3.18516335.18518165.rev.compl.REF -- 31039

chimp.LOC112206744-LOC107973052-GGT2.revcompl atcctccag-cagaccctccccagtagctgagactaaaggcacaact----acacccagc 48479
chimp54546-135457.revcompl.Pan.troglodytes.clone.rp43-41g5.GenBank:AC099533.36 ATTCTCCTGTCTCAGCCTCCCAAGTAGCTGGGACTACAGGCACACATCAAAACGCCTGGC 50509
human.FAM30B-LOC105372935-GGT2.NCBI. atcctccag-cagaccctccccagtagctgagactaaaggcacaact----acacccagc 47470
human.FAM230E-LOC105377182-GGT3P.NCBI. atcctccag-cagaccctccccagtagctgagactaaaggcacaact----acacccagc 46123
human.FAM230J-LOC105372942-GGTLC5P.NCBI.rev.compl -- 46364
human.FAM230A.ncRNA.18487127.18500594-GGTLC3.18516335.18518165.rev.compl.REF -- 31039

chimp.LOC112206744-LOC107973052-GGT2.revcompl aaattttaattttttt-------gttgtgttttgctatatttct---------------- 48516
chimp54546-135457.revcompl.Pan.troglodytes.clone.rp43-41g5.GenBank:AC099533.36 TAATTTTTGTATTTTTAGTAGAGATGGAGTTTTGCCATATTGGTCAGGCTGGTCTCGAAC 50569
human.FAM30B-LOC105372935-GGT2.NCBI. aaattttaattttttt-------gttgtgttttgctatatttct---------------- 47507
human.FAM230E-LOC105377182-GGT3P.NCBI. aaattttaattttttt-------gttgtgttttgctatatttct---------------- 46160
human.FAM230J-LOC105372942-GGTLC5P.NCBI.rev.compl -- 46364
human.FAM230A.ncRNA.18487127.18500594-GGTLC3.18516335.18518165.rev.compl.REF -- 31039

chimp.LOC112206744-LOC107973052-GGT2.revcompl -- 48516
chimp54546-135457.revcompl.Pan.troglodytes.clone.rp43-41g5.GenBank:AC099533.36 TCCTGACCTCAGGTGATCGACCTGTCTTAGCCTCCCGAACTGCTAGGATTACAGGTGTAC 50629
human.FAM30B-LOC105372935-GGT2.NCBI. -- 47507
human.FAM230E-LOC105377182-GGT3P.NCBI. -- 46160
human.FAM230J-LOC105372942-GGTLC5P.NCBI.rev.compl -- 46364
human.FAM230A.ncRNA.18487127.18500594-GGTLC3.18516335.18518165.rev.compl.REF -- 31039

chimp.LOC112206744-LOC107973052-GGT2.revcompl --------------------------ttctttctttctttcttttttttttgtgagacgg 48550
chimp54546-135457.revcompl.Pan.troglodytes.clone.rp43-41g5.GenBank:AC099533.36 GTTTATTTATTTGAGATGGAATCTTGCTCTGTATTT-ATTAATTTTTTTATCTGAGATGG 50688
human.FAM30B-LOC105372935-GGT2.NCBI. --------------------------ttctttcttt-ttttttttttttttgtgagacgg 47540
human.FAM230E-LOC105377182-GGT3P.NCBI. --------------------------ttctttc-tt-ttttttttttttttgtgagacgg 46192
human.FAM230J-LOC105372942-GGTLC5P.NCBI.rev.compl -- 46364
human.FAM230A.ncRNA.18487127.18500594-GGTLC3.18516335.18518165.rev.compl.REF -- 31039

chimp.LOC112206744-LOC107973052-GGT2.revcompl agtctcactctatcgcccaggctggagtgcagtggcgcgatatcggctcactgcaagctc 48610
chimp54546-135457.revcompl.Pan.troglodytes.clone.rp43-41g5.GenBank:AC099533.36 AGTCTTTCTCCATCGCCCAGGCTAGAGTGCAGTGGTGCAATCTCGGCTCCCTGCAACCTC 50748
human.FAM30B-LOC105372935-GGT2.NCBI. agtctcgctctatcgcccaggctggagtgcagtggcgcgatatcggctcactgcaagctc 47600
human.FAM230E-LOC105377182-GGT3P.NCBI. agtctcgctctatcgcccaggctggagtgcagtggcgcgatatcggctcactgcaagctc 46252
human.FAM230J-LOC105372942-GGTLC5P.NCBI.rev.compl -- 46364
human.FAM230A.ncRNA.18487127.18500594-GGTLC3.18516335.18518165.rev.compl.REF -- 31039

chimp.LOC112206744-LOC107973052-GGT2.revcompl cgcctcccaggttcacgccattctcctgcctcagcctcccaagtagctgggactgcaggc 48670
chimp54546-135457.revcompl.Pan.troglodytes.clone.rp43-41g5.GenBank:AC099533.36 TGACTTCCAGTTTCAAGTGATTCTCCTGCCTCAGTGTCCCAAGTAGCTGGGATTACAGGT 50808
human.FAM30B-LOC105372935-GGT2.NCBI. cgcctcccaggttcacgccattctcctgcctcagcctcccaagtagctgggactgcaggc 47660
human.FAM230E-LOC105377182-GGT3P.NCBI. cgcctcccaggttcacgccattctcctgcctcagcctcccaagtagctgggactgcaggc 46312
human.FAM230J-LOC105372942-GGTLC5P.NCBI.rev.compl -- 46364
human.FAM230A.ncRNA.18487127.18500594-GGTLC3.18516335.18518165.rev.compl.REF -- 31039

chimp.LOC112206744-LOC107973052-GGT2.revcompl acccgccaccacacctggctaatttttttaaatttttagtagagacag-gtttcactgtg 48729
chimp54546-135457.revcompl.Pan.troglodytes.clone.rp43-41g5.GenBank:AC099533.36 GCCTGCCACCACAGCTGGCTAATTTT--TGTATTTTTAGTAGAGACAGTGTTTCACCATC 50866
human.FAM30B-LOC105372935-GGT2.NCBI. acccgccaccacatgtggctaatttttctgtatttttagtagagacag-gtttcactgtg 47719
human.FAM230E-LOC105377182-GGT3P.NCBI. acccgccaccacatgtggctaatttttctgtatttttagtagagacag-gtttcactgtg 46371
human.FAM230J-LOC105372942-GGTLC5P.NCBI.rev.compl -- 46364
human.FAM230A.ncRNA.18487127.18500594-GGTLC3.18516335.18518165.rev.compl.REF -- 31039

chimp.LOC112206744-LOC107973052-GGT2.revcompl ttagccaggatggtctcgatctcctgaccctgtgatccacacgcctcggcctctcaaagt 48789
chimp54546-135457.revcompl.Pan.troglodytes.clone.rp43-41g5.GenBank:AC099533.36 TTGGCCAGGCTGGTCTCGGGCTCCTGACCTCATGAACCACCTGCCTCAGCCTCCCAAAGT 50926
human.FAM30B-LOC105372935-GGT2.NCBI. ttagccaggatggtctcgatctcctgaccccgtgatccacacgcctcggcctctcaaagt 47779
human.FAM230E-LOC105377182-GGT3P.NCBI. ttagccaggatggtctcgatctcctgaccccgtgatccacacgcctcggcctctcaaagt 46431
human.FAM230J-LOC105372942-GGTLC5P.NCBI.rev.compl -- 46364
human.FAM230A.ncRNA.18487127.18500594-GGTLC3.18516335.18518165.rev.compl.REF -- 31039

chimp.LOC112206744-LOC107973052-GGT2.revcompl gctgggattacaggcgtgaggcaccatgcccggccgggttttgctatatttcttttcact 48849
chimp54546-135457.revcompl.Pan.troglodytes.clone.rp43-41g5.GenBank:AC099533.36 GTTGGGATTACAGGCCTAAGGCACCATGCTCGGCCATATTTATTTAT------------- 50973

```
human.FAM30B-LOC105372935-GGT2.NCBI.                                      gctgagattacaggcgtgaggcaccatgcccggccgggttttgctatatttcttttcact 47839
human.FAM230E-LOC105377182-GGT3P.NCBI.                                    gctgagattacaggcgtgaggcaccatgcccggccgggttttgctatatttcttttcact 46491
human.FAM230J-LOC105372942-GGTLC5P.NCBI.rev.compl                         ------------------------------------------------------------ 46364
human.FAM230A.ncRNA.18487127.18500594-GGTLC3.18516335.18518165.rev.compl.REF ------------------------------------------------------------ 31039

chimp.LOC112206744-LOC107973052-GGT2.revcompl                             atgctttgaatttttttgttttcttgttccccacccccacccccactatatttatgtagat 48909
chimp54546-135457.revcompl.Pan.troglodytes.clone.rp43-41g5.GenBank:AC099533.36 ------------------------------------------------------------ 50973
human.FAM30B-LOC105372935-GGT2.NCBI.                                      atgctttgaatttttttgttttcttgttccccacccccacccccactatatttatgtagat 47899
human.FAM230E-LOC105377182-GGT3P.NCBI.                                    atgctttgaatttttttgttttcttgttccccacccccacccccactatatttatgtagat 46551
human.FAM230J-LOC105372942-GGTLC5P.NCBI.rev.compl                         ------------------------------------------------------------ 46364
human.FAM230A.ncRNA.18487127.18500594-GGTLC3.18516335.18518165.rev.compl.REF ------------------------------------------------------------ 31039

chimp.LOC112206744-LOC107973052-GGT2.revcompl                             tctcaattttttttttgtagactcactatgttgcccaggcttgtcttggacctcctgacc 48969
chimp54546-135457.revcompl.Pan.troglodytes.clone.rp43-41g5.GenBank:AC099533.36 ------------------------------------------------------------ 50973
human.FAM30B-LOC105372935-GGT2.NCBI.                                      tctcaatattttttttgtagactcactatgttgcccaggcttgtcttggacccccctgacc 47959
human.FAM230E-LOC105377182-GGT3P.NCBI.                                    tctcaatattttttttgtagactcactatgttgcccaggcttgtcttggacctcctgacc 46611
human.FAM230J-LOC105372942-GGTLC5P.NCBI.rev.compl                         ------------------------------------------------------------ 46364
human.FAM230A.ncRNA.18487127.18500594-GGTLC3.18516335.18518165.rev.compl.REF ------------------------------------------------------------ 31039

chimp.LOC112206744-LOC107973052-GGT2.revcompl                             tcaacttctacctcagcctcccaaagtgttgggattacaggcatgagacaccaggctttg 49029
chimp54546-135457.revcompl.Pan.troglodytes.clone.rp43-41g5.GenBank:AC099533.36 ------------------------------------------------------------ 50973
human.FAM30B-LOC105372935-GGT2.NCBI.                                      tcaacttctacctcagcctcccaaagtgttgggattacaggcatgagacaccaagcttgg 48019
human.FAM230E-LOC105377182-GGT3P.NCBI.                                    tcaacttctacctcaggctcccaaagtgttgggattacaggcatgagacaccaagcttgg 46671
human.FAM230J-LOC105372942-GGTLC5P.NCBI.rev.compl                         ------------------------------------------------------------ 46364
human.FAM230A.ncRNA.18487127.18500594-GGTLC3.18516335.18518165.rev.compl.REF ------------------------------------------------------------ 31039

chimp.LOC112206744-LOC107973052-GGT2.revcompl                             cctcagacggccttttctttctttttttttttttgagatggagtctcactctg------- 49082
chimp54546-135457.revcompl.Pan.troglodytes.clone.rp43-41g5.GenBank:AC099533.36 -----------------------TTAATTATTTAGAGACAAAGTCTTGCTCTGTCACCCA 51010
human.FAM30B-LOC105372935-GGT2.NCBI.                                      cctcagagggccttttctctc--tttttttttttgagatggagtctcactctg------- 48070
human.FAM230E-LOC105377182-GGT3P.NCBI.                                    cctcagagggccttttctctc-tttttttttttgagatggagtctcactctg------- 46723
human.FAM230J-LOC105372942-GGTLC5P.NCBI.rev.compl                         ------------------------------------------------------------ 46364
human.FAM230A.ncRNA.18487127.18500594-GGTLC3.18516335.18518165.rev.compl.REF ------------------------------------------------------------ 31039

chimp.LOC112206744-LOC107973052-GGT2.revcompl                             ---tggagtgcagtggtgcaatctcggctcactgcaacctcagccccccaggttctagcg 49139
chimp54546-135457.revcompl.Pan.troglodytes.clone.rp43-41g5.GenBank:AC099533.36 GGCTGGAGTGCAGTGGCGCCATCTCAGCTCACTGCAGCCTCCGCCTCCAAGGTTTAAGCG 51070
human.FAM30B-LOC105372935-GGT2.NCBI.                                      ---tggagtgcagtggtgcaatctcggctcactgcaacctcagccccacaggttctagcg 48127
human.FAM230E-LOC105377182-GGT3P.NCBI.                                    ---tggagtgcagtggtgcaatctcggctcactgcaacctcagccccacaggttctagcg 46780
human.FAM230J-LOC105372942-GGTLC5P.NCBI.rev.compl                         ------------------------------------------------------------ 46364
human.FAM230A.ncRNA.18487127.18500594-GGTLC3.18516335.18518165.rev.compl.REF ------------------------------------------------------------ 31039

chimp.LOC112206744-LOC107973052-GGT2.revcompl                             attctcctgccacagcctcccacgtagctgggattacaggcacaacccaccatgcctgac 49199
chimp54546-135457.revcompl.Pan.troglodytes.clone.rp43-41g5.GenBank:AC099533.36 ATTCTCATGCCTCAGCCTCCTGACTAACTGGGACTACAGGTACTCACCACCATGCAGGGA 51130
human.FAM30B-LOC105372935-GGT2.NCBI.                                      attctcctgccacagcctcccaagtagctgggattacaggcacaacccaccatgcctgac 48187
human.FAM230E-LOC105377182-GGT3P.NCBI.                                    attctcctgccacagcctcccaagtagctgggattacaggcacaacccaccatgcctgac 46840
human.FAM230J-LOC105372942-GGTLC5P.NCBI.rev.compl                         ------------------------------------------------------------ 46364
human.FAM230A.ncRNA.18487127.18500594-GGTLC3.18516335.18518165.rev.compl.REF ------------------------------------------------------------ 31039

chimp.LOC112206744-LOC107973052-GGT2.revcompl                             ta--attttgcatttttagtagagacagggtttcaccatgttggccaggctggtcttgaa 49257
chimp54546-135457.revcompl.Pan.troglodytes.clone.rp43-41g5.GenBank:AC099533.36 TATTTTTTTCTATTTTTTTTACAGACACAGTTTCACCATGTTGGCCAGGCTGGTCTCGAA 51190
human.FAM30B-LOC105372935-GGT2.NCBI.                                      ta--attttgcatttttagtagagacagggtttcaccatgttggccaggctggtcttgaa 48245
human.FAM230E-LOC105377182-GGT3P.NCBI.                                    ta--attttgcatttttagtagagacagggtttcaccatgttggccaggctggtcttgaa 46898
human.FAM230J-LOC105372942-GGTLC5P.NCBI.rev.compl                         ------------------------------------------------------------ 46364
human.FAM230A.ncRNA.18487127.18500594-GGTLC3.18516335.18518165.rev.compl.REF ------------------------------------------------------------ 31039

chimp.LOC112206744-LOC107973052-GGT2.revcompl                             ctcctgagctcaggtgttccacccgcctcggcctcccaaagtgctgggattacagacgtg 49317
chimp54546-135457.revcompl.Pan.troglodytes.clone.rp43-41g5.GenBank:AC099533.36 CTCCTGACCTTAGGTGATCTGACAGCCTCGTCCCCTCAAAGTACTGGGATTACAGGCATG 51250
human.FAM30B-LOC105372935-GGT2.NCBI.                                      ctcctgacctcaggtgatccacccgcctcggcctcccaaagtgctgggattacagacgtg 48305
human.FAM230E-LOC105377182-GGT3P.NCBI.                                    ctcctgacctcaggtgatccacccgcctcggcctcccaaagtgctgggattacagacgtg 46958
human.FAM230J-LOC105372942-GGTLC5P.NCBI.rev.compl                         ------------------------------------------------------------ 46364
human.FAM230A.ncRNA.18487127.18500594-GGTLC3.18516335.18518165.rev.compl.REF ------------------------------------------------------------ 31039

chimp.LOC112206744-LOC107973052-GGT2.revcompl                             agccactgcgcctggctcagagggcctttt--ctaactggagaattcctgccggtgtccc 49375
chimp54546-135457.revcompl.Pan.troglodytes.clone.rp43-41g5.GenBank:AC099533.36 AGCCACTGTGCCCGGCCTCTCACTACATTTAAGTGACGCCATGGCTCATGCCTGTAATCC 51310
human.FAM30B-LOC105372935-GGT2.NCBI.                                      agccaccgcacctggctcagagggcctttt--ctaactggagaattcctgccggtgtccc 48363
human.FAM230E-LOC105377182-GGT3P.NCBI.                                    agccaccgcacctggctcagagggcctttt--ctaactggagaattcctgccggtgtccc 47016
human.FAM230J-LOC105372942-GGTLC5P.NCBI.rev.compl                         ------------------------------------------------------------ 46364
human.FAM230A.ncRNA.18487127.18500594-GGTLC3.18516335.18518165.rev.compl.REF ------------------------------------------------------------ 31039

chimp.LOC112206744-LOC107973052-GGT2.revcompl                             tgctgct----------------------------------------------------- 49382
chimp54546-135457.revcompl.Pan.troglodytes.clone.rp43-41g5.GenBank:AC099533.36 TAGCACTTTGGGAGGCCAAGGCAGGTGGATCACTTCAGGTCAGAAGTTCGACACGAGCCT 51370
human.FAM30B-LOC105372935-GGT2.NCBI.                                      tgctgct----------------------------------------------------- 48370
human.FAM230E-LOC105377182-GGT3P.NCBI.                                    tgctgct----------------------------------------------------- 47023
human.FAM230J-LOC105372942-GGTLC5P.NCBI.rev.compl                         ------------------------------------------------------------ 46364
human.FAM230A.ncRNA.18487127.18500594-GGTLC3.18516335.18518165.rev.compl.REF ------------------------------------------------------------ 31039

chimp.LOC112206744-LOC107973052-GGT2.revcompl                             ------------------------------------------------------------ 49382
chimp54546-135457.revcompl.Pan.troglodytes.clone.rp43-41g5.GenBank:AC099533.36 GGCCAACATGGGGAAACCCCGTCTCTAGTAAAAATGCACAAATTAGTCAGGCGTGGTGGT 51430
human.FAM30B-LOC105372935-GGT2.NCBI.                                      ------------------------------------------------------------ 48370
human.FAM230E-LOC105377182-GGT3P.NCBI.                                    ------------------------------------------------------------ 47023
human.FAM230J-LOC105372942-GGTLC5P.NCBI.rev.compl                         ------------------------------------------------------------ 46364
human.FAM230A.ncRNA.18487127.18500594-GGTLC3.18516335.18518165.rev.compl.REF ------------------------------------------------------------ 31039

chimp.LOC112206744-LOC107973052-GGT2.revcompl                             ------------------------------------------------------------ 49382
chimp54546-135457.revcompl.Pan.troglodytes.clone.rp43-41g5.GenBank:AC099533.36 ACAAGCCTGTAGGCCCAGCTACTTGGAAGGCTGAGGCAGGAGAATCACTTTAACCGGGAG 51490
human.FAM30B-LOC105372935-GGT2.NCBI.                                      ------------------------------------------------------------ 48370
human.FAM230E-LOC105377182-GGT3P.NCBI.                                    ------------------------------------------------------------ 47023
human.FAM230J-LOC105372942-GGTLC5P.NCBI.rev.compl                         ------------------------------------------------------------ 46364
human.FAM230A.ncRNA.18487127.18500594-GGTLC3.18516335.18518165.rev.compl.REF ------------------------------------------------------------ 31039

chimp.LOC112206744-LOC107973052-GGT2.revcompl                             ------------------------------------------------------------ 49382
chimp54546-135457.revcompl.Pan.troglodytes.clone.rp43-41g5.GenBank:AC099533.36 GCAGAGGTTGCAGTGAGCCAATATCATGCCACTGCACTCCAGCTTGGGTGACAGAGTGAG 51550
human.FAM30B-LOC105372935-GGT2.NCBI.                                      ------------------------------------------------------------ 48370
human.FAM230E-LOC105377182-GGT3P.NCBI.                                    ------------------------------------------------------------ 47023
human.FAM230J-LOC105372942-GGTLC5P.NCBI.rev.compl                         ------------------------------------------------------------ 46364
human.FAM230A.ncRNA.18487127.18500594-GGTLC3.18516335.18518165.rev.compl.REF ------------------------------------------------------------ 31039

chimp.LOC112206744-LOC107973052-GGT2.revcompl                             ------------------------------------------------------------ 49382
chimp54546-135457.revcompl.Pan.troglodytes.clone.rp43-41g5.GenBank:AC099533.36 ACACCGTCTCAAAAAAAAAAAAAAAAAAAGGAAAGAAAAACATATGATGCCGGGGCATCTC 51610
human.FAM30B-LOC105372935-GGT2.NCBI.                                      ------------------------------------------------------------ 48370
human.FAM230E-LOC105377182-GGT3P.NCBI.                                    ------------------------------------------------------------ 47023
human.FAM230J-LOC105372942-GGTLC5P.NCBI.rev.compl                         ------------------------------------------------------------ 46364
human.FAM230A.ncRNA.18487127.18500594-GGTLC3.18516335.18518165.rev.compl.REF ------------------------------------------------------------ 31039

chimp.LOC112206744-LOC107973052-GGT2.revcompl                             ---------------------------------------------------------tgg 49385
chimp54546-135457.revcompl.Pan.troglodytes.clone.rp43-41g5.GenBank:AC099533.36 GGCCTCAATACCTGCGTGAGCACAGTGATGTCCAGGCCAGGGCTGCTGGTCGAGGTCTGG 51670
human.FAM30B-LOC105372935-GGT2.NCBI.                                      ---------------------------------------------------------tgg 48373
human.FAM230E-LOC105377182-GGT3P.NCBI.                                    ---------------------------------------------------------tgg 47026
human.FAM230J-LOC105372942-GGTLC5P.NCBI.rev.compl                         ------------------------------------------------------------ 46364
human.FAM230A.ncRNA.18487127.18500594-GGTLC3.18516335.18518165.rev.compl.REF ------------------------------------------------------------ 31039

chimp.LOC112206744-LOC107973052-GGT2.revcompl                             cctcttctcctcatgaggaatggatagagggagggagggaggct---------------- 49429
chimp54546-135457.revcompl.Pan.troglodytes.clone.rp43-41g5.GenBank:AC099533.36 CCCCATCTCTTCCAGCAGAAAGGGAGTAAGCTTGCAGGGAGGCTGGGGGACAAGATCCCA 51730
human.FAM30B-LOC105372935-GGT2.NCBI.                                      cctcttctcctcacgatgaatggatagagggagggagggaggct---------------- 48417
human.FAM230E-LOC105377182-GGT3P.NCBI.                                    cctcttctcctcacgatgaatggatagagggagggagggaggct---------------- 47070
human.FAM230J-LOC105372942-GGTLC5P.NCBI.rev.compl                         ------------------------------------------------------------ 46364
human.FAM230A.ncRNA.18487127.18500594-GGTLC3.18516335.18518165.rev.compl.REF ------------------------------------------------------------ 31039

chimp.LOC112206744-LOC107973052-GGT2.revcompl                             ------------------------------------------------------------ 49429
chimp54546-135457.revcompl.Pan.troglodytes.clone.rp43-41g5.GenBank:AC099533.36 GGATCTCAGCCTCTGCTCATGGATCAGCTCTGAGACCCCGAGTGAGCTGGGGGTGCTCTG 51790
human.FAM30B-LOC105372935-GGT2.NCBI.                                      ------------------------------------------------------------ 48417
human.FAM230E-LOC105377182-GGT3P.NCBI.                                    ------------------------------------------------------------ 47070
human.FAM230J-LOC105372942-GGTLC5P.NCBI.rev.compl                         ------------------------------------------------------------ 46364
human.FAM230A.ncRNA.18487127.18500594-GGTLC3.18516335.18518165.rev.compl.REF ------------------------------------------------------------ 31039

chimp.LOC112206744-LOC107973052-GGT2.revcompl                             ------------------------------------------------------------ 49429
chimp54546-135457.revcompl.Pan.troglodytes.clone.rp43-41g5.GenBank:AC099533.36 TGCGCATTGGTTTCCCCAGCTGTCAAGTAAAGGGATTGGATGAGGAAGTCTTGTCAAGGT 51850
human.FAM30B-LOC105372935-GGT2.NCBI.                                      ------------------------------------------------------------ 48417
```

```
human.FAM230E-LOC105377182-GGT3P.NCBI.                                               ------------------------------------------------------------ 47070
human.FAM230J-LOC105372942-GGTLC5P.NCBI.rev.compl                                    ------------------------------------------------------------ 46364
human.FAM230A.ncRNA.18487127.18500594-GGTLC3.18516335.18518165.rev.compl.REF         ------------------------------------------------------------ 31039


chimp.LOC112206744-LOC107973052-GGT2.revcompl                                        ------------------------------------------------------------ 49429
chimp54546-135457.revcompl.Pan.troglodytes.clone.rp43-41g5.GenBank:AC099533.36       GGAATGATCTCAGATTTGGGGCAGCAGTGAATGATCCCGCTCCCTGGGCCATGCCAGTGG 51910
human.FAM30B-LOC105372935-GGT2.NCBI.                                                 ------------------------------------------------------------ 48417
human.FAM230E-LOC105377182-GGT3P.NCBI.                                               ------------------------------------------------------------ 47070
human.FAM230J-LOC105372942-GGTLC5P.NCBI.rev.compl                                    ------------------------------------------------------------ 46364
human.FAM230A.ncRNA.18487127.18500594-GGTLC3.18516335.18518165.rev.compl.REF         ------------------------------------------------------------ 31039


chimp.LOC112206744-LOC107973052-GGT2.revcompl                                        ------------------------------------------------------------ 49429
chimp54546-135457.revcompl.Pan.troglodytes.clone.rp43-41g5.GenBank:AC099533.36       CCCGGCCTCGGCTCAACACAGCCCCAACACTCTGGAATGGGGATGAGGGGGCAGTCAGCT 51970
human.FAM30B-LOC105372935-GGT2.NCBI.                                                 ------------------------------------------------------------ 48417
human.FAM230E-LOC105377182-GGT3P.NCBI.                                               ------------------------------------------------------------ 47070
human.FAM230J-LOC105372942-GGTLC5P.NCBI.rev.compl                                    ------------------------------------------------------------ 46364
human.FAM230A.ncRNA.18487127.18500594-GGTLC3.18516335.18518165.rev.compl.REF         ------------------------------------------------------------ 31039


chimp.LOC112206744-LOC107973052-GGT2.revcompl                                        ------------------------------------------------------------ 49429
chimp54546-135457.revcompl.Pan.troglodytes.clone.rp43-41g5.GenBank:AC099533.36       CTTGCTCCTAGTAAGAGAGATGCAATAGGGCTCTGTGGCTGAGCTGGGTGCCTTGCCTCA 52030
human.FAM30B-LOC105372935-GGT2.NCBI.                                                 ------------------------------------------------------------ 48417
human.FAM230E-LOC105377182-GGT3P.NCBI.                                               ------------------------------------------------------------ 47070
human.FAM230J-LOC105372942-GGTLC5P.NCBI.rev.compl                                    ------------------------------------------------------------ 46364
human.FAM230A.ncRNA.18487127.18500594-GGTLC3.18516335.18518165.rev.compl.REF         ------------------------------------------------------------ 31039


chimp.LOC112206744-LOC107973052-GGT2.revcompl                                        ------------------------------------------------------------ 49429
chimp54546-135457.revcompl.Pan.troglodytes.clone.rp43-41g5.GenBank:AC099533.36       CACCTGTAATCCCAACCTTTGAGAGGCCGAGGCGGGAGGATTGCTTGAGGCCGGGAATTT 52090
human.FAM30B-LOC105372935-GGT2.NCBI.                                                 ------------------------------------------------------------ 48417
human.FAM230E-LOC105377182-GGT3P.NCBI.                                               ------------------------------------------------------------ 47070
human.FAM230J-LOC105372942-GGTLC5P.NCBI.rev.compl                                    ------------------------------------------------------------ 46364
human.FAM230A.ncRNA.18487127.18500594-GGTLC3.18516335.18518165.rev.compl.REF         ------------------------------------------------------------ 31039


chimp.LOC112206744-LOC107973052-GGT2.revcompl                                        ------------------------------------------------------------ 49429
chimp54546-135457.revcompl.Pan.troglodytes.clone.rp43-41g5.GenBank:AC099533.36       TGAGAATAGCCTGGACAACATAGCCAGACCCCATGTCTGCAAAATAATAAGAAAACACAC 52150
human.FAM30B-LOC105372935-GGT2.NCBI.                                                 ------------------------------------------------------------ 48417
human.FAM230E-LOC105377182-GGT3P.NCBI.                                               ------------------------------------------------------------ 47070
human.FAM230J-LOC105372942-GGTLC5P.NCBI.rev.compl                                    ------------------------------------------------------------ 46364
human.FAM230A.ncRNA.18487127.18500594-GGTLC3.18516335.18518165.rev.compl.REF         ------------------------------------------------------------ 31039


chimp.LOC112206744-LOC107973052-GGT2.revcompl                                        ------------------------------------------------------------ 49429
chimp54546-135457.revcompl.Pan.troglodytes.clone.rp43-41g5.GenBank:AC099533.36       AGCTATAGTCCAAGCTACTTGGCAGGCTGAGGCAGGAAGGTCCCTTGAGTCCGGGAATTG 52210
human.FAM30B-LOC105372935-GGT2.NCBI.                                                 ------------------------------------------------------------ 48417
human.FAM230E-LOC105377182-GGT3P.NCBI.                                               ------------------------------------------------------------ 47070
human.FAM230J-LOC105372942-GGTLC5P.NCBI.rev.compl                                    ------------------------------------------------------------ 46364
human.FAM230A.ncRNA.18487127.18500594-GGTLC3.18516335.18518165.rev.compl.REF         ------------------------------------------------------------ 31039


chimp.LOC112206744-LOC107973052-GGT2.revcompl                                        ------------------------------------------------------------ 49429
chimp54546-135457.revcompl.Pan.troglodytes.clone.rp43-41g5.GenBank:AC099533.36       GAGGCTGCATTGAGCTATAATCACACCACTGCACTCCAGCTTGGGTGACAAAGTGAGACC 52270
human.FAM30B-LOC105372935-GGT2.NCBI.                                                 ------------------------------------------------------------ 48417
human.FAM230E-LOC105377182-GGT3P.NCBI.                                               ------------------------------------------------------------ 47070
human.FAM230J-LOC105372942-GGTLC5P.NCBI.rev.compl                                    ------------------------------------------------------------ 46364
human.FAM230A.ncRNA.18487127.18500594-GGTLC3.18516335.18518165.rev.compl.REF         ------------------------------------------------------------ 31039


chimp.LOC112206744-LOC107973052-GGT2.revcompl                                        ------------------------------------------------------------ 49429
chimp54546-135457.revcompl.Pan.troglodytes.clone.rp43-41g5.GenBank:AC099533.36       CTGTCTCTAAAAGAAAAAAAAATTGGCCTGTGAGCATGGGTTTGATTTTCAAACAGGACC 52330
human.FAM30B-LOC105372935-GGT2.NCBI.                                                 ------------------------------------------------------------ 48417
human.FAM230E-LOC105377182-GGT3P.NCBI.                                               ------------------------------------------------------------ 47070
human.FAM230J-LOC105372942-GGTLC5P.NCBI.rev.compl                                    ------------------------------------------------------------ 46364
human.FAM230A.ncRNA.18487127.18500594-GGTLC3.18516335.18518165.rev.compl.REF         ------------------------------------------------------------ 31039


chimp.LOC112206744-LOC107973052-GGT2.revcompl                                        ------------------------------------------------------------ 49429
chimp54546-135457.revcompl.Pan.troglodytes.clone.rp43-41g5.GenBank:AC099533.36       CGGAGGGTAGGGACAGACAGTGCTGTCACCCTTAGGTGCTGAACACTCAGAAACGGGCCA 52390
human.FAM30B-LOC105372935-GGT2.NCBI.                                                 ------------------------------------------------------------ 48417
human.FAM230E-LOC105377182-GGT3P.NCBI.                                               ------------------------------------------------------------ 47070
human.FAM230J-LOC105372942-GGTLC5P.NCBI.rev.compl                                    ------------------------------------------------------------ 46364
human.FAM230A.ncRNA.18487127.18500594-GGTLC3.18516335.18518165.rev.compl.REF         ------------------------------------------------------------ 31039


chimp.LOC112206744-LOC107973052-GGT2.revcompl                                        ---------cttaattctcctggagtcagctccagacagacagggtattggcatgccaat 49480
chimp54546-135457.revcompl.Pan.troglodytes.clone.rp43-41g5.GenBank:AC099533.36       GCGGCAGCCCTTTCCTCACCTGCAGACACCAG----ATTGGGCAGAACAGCACGTGGCAC 52446
human.FAM30B-LOC105372935-GGT2.NCBI.                                                 ---------cttaattctcctggagtcagctc----cagacagggtattggcatgccaat 48464
human.FAM230E-LOC105377182-GGT3P.NCBI.                                               ---------cttaattctcctggagtcagctc----cagacagggtattggcatgccaat 47117
human.FAM230J-LOC105372942-GGTLC5P.NCBI.rev.compl                                    ------------------------------------------------------------ 46364
human.FAM230A.ncRNA.18487127.18500594-GGTLC3.18516335.18518165.rev.compl.REF         ------------------------------------------------------------ 31039


chimp.LOC112206744-LOC107973052-GGT2.revcompl                                        ttccagcctcagtggtaaaggtcg-acacgctaatcaccctcctccatgaagcagtgaca 49539
chimp54546-135457.revcompl.Pan.troglodytes.clone.rp43-41g5.GenBank:AC099533.36       TTGCAGCTCTTGCAGTGAGGACAGAACCCAGTGTCAACCCTTCTGCCTGTGGGAGGGGCT 52506
human.FAM30B-LOC105372935-GGT2.NCBI.                                                 ttccagcctcagtggtaaaggtcg-acacgctaatcaccctcctccatgaaacagtgaca 48523
human.FAM230E-LOC105377182-GGT3P.NCBI.                                               ttccagcctcagtggtaaaggtcg-acacgctaatcaccctcctccatgaaacagtgaca 47176
human.FAM230J-LOC105372942-GGTLC5P.NCBI.rev.compl                                    ------------------------------------------------------------ 46364
human.FAM230A.ncRNA.18487127.18500594-GGTLC3.18516335.18518165.rev.compl.REF         ------------------------------------------------------------ 31039


chimp.LOC112206744-LOC107973052-GGT2.revcompl                                        aaaattacctgaagaaagccacagccaagctccaggcccctgccccacaaatccccttcc 49599
chimp54546-135457.revcompl.Pan.troglodytes.clone.rp43-41g5.GenBank:AC099533.36       GCTGAGGCCTGCAGAGAG-----GCCAGGGTGGAGGCTCATCCCCTTGTCCAGCCCTTGG 52561
human.FAM30B-LOC105372935-GGT2.NCBI.                                                 aaaattacctgaagaaagccacagccaagctccaggcccctgccccacaaatccccttcc 48583
human.FAM230E-LOC105377182-GGT3P.NCBI.                                               aaaattacctgaagaaagccacagccaagctccaggcccctgccccacaaatccccttcc 47236
human.FAM230J-LOC105372942-GGTLC5P.NCBI.rev.compl                                    ------------------------------------------------------------ 46364
human.FAM230A.ncRNA.18487127.18500594-GGTLC3.18516335.18518165.rev.compl.REF         ------------------------------------------------------------ 31039


chimp.LOC112206744-LOC107973052-GGT2.revcompl                                        ccatgcctctctcaaggcgaccctcatcccttgtaaccctcttggtgaatcaaagcgccc 49659
chimp54546-135457.revcompl.Pan.troglodytes.clone.rp43-41g5.GenBank:AC099533.36       CGTGGTCTCCACCAGGTCCCCAGCCCACCAGTGTAGGGC---------------GCCCCT 52606
human.FAM30B-LOC105372935-GGT2.NCBI.                                                 ccatgcctctctcaaggcgaccctcatcccttgtaaccctcttggtgaatcaaagcgccc 48643
human.FAM230E-LOC105377182-GGT3P.NCBI.                                               ccatgcctctctcaaggcgaccctcatcccttgtaaccctcttggtgaatcaaagcgccc 47296
human.FAM230J-LOC105372942-GGTLC5P.NCBI.rev.compl                                    ------------------------------------------------------------ 46364
human.FAM230A.ncRNA.18487127.18500594-GGTLC3.18516335.18518165.rev.compl.REF         ------------------------------------------------------------ 31039


chimp.LOC112206744-LOC107973052-GGT2.revcompl                                        tctactgggccttagcccagctgttcctct-gctaggaatcccttcctctctctgcctaa 49718
chimp54546-135457.revcompl.Pan.troglodytes.clone.rp43-41g5.GenBank:AC099533.36       GAACCTGCTGCTGCCACAGGCCCTGTCTCTACCCAGGACGTCCCCCCACCCTCGCAGTGT 52666
human.FAM30B-LOC105372935-GGT2.NCBI.                                                 tctactgggccttagcccagctgttcctct-gctaggaatcccttcctctctctgcctaa 48702
human.FAM230E-LOC105377182-GGT3P.NCBI.                                               tctactgggccttagcccagctgttcctct-gctaggaatcccttcctctctctgcctaa 47355
human.FAM230J-LOC105372942-GGTLC5P.NCBI.rev.compl                                    ------------------------------------------------------------ 46364
human.FAM230A.ncRNA.18487127.18500594-GGTLC3.18516335.18518165.rev.compl.REF         ------------------------------------------------------------ 31039


chimp.LOC112206744-LOC107973052-GGT2.revcompl                                        cgaagttatctgcagcccagccgccacctcctccaagaagtcctcct------------- 49765
chimp54546-135457.revcompl.Pan.troglodytes.clone.rp43-41g5.GenBank:AC099533.36       CAGGGAAATGATCATGGTGGCGGTGACACTCCGCAGGCAGGGCTGCTGAGAGAAGCTGAG 52726
human.FAM30B-LOC105372935-GGT2.NCBI.                                                 cgaagttatctgcagcccagccgccacctcctccaagaagtcctcct------------- 48749
human.FAM230E-LOC105377182-GGT3P.NCBI.                                               cgaagttatctgcagcccagccgccacctcctccaagaagtcctcct------------- 47402
human.FAM230J-LOC105372942-GGTLC5P.NCBI.rev.compl                                    ------------------------------------------------------------ 46364
human.FAM230A.ncRNA.18487127.18500594-GGTLC3.18516335.18518165.rev.compl.REF         ------------------------------------------------------------ 31039


chimp.LOC112206744-LOC107973052-GGT2.revcompl                                        ------------------------------------------------------------ 49765
chimp54546-135457.revcompl.Pan.troglodytes.clone.rp43-41g5.GenBank:AC099533.36       AAGGGTCACACTGCAGGCAGGGGCCCGTGTGACAAGCCCCTCTCACCCCGAGAGAGCTGA 52786
human.FAM30B-LOC105372935-GGT2.NCBI.                                                 ------------------------------------------------------------ 48749
human.FAM230E-LOC105377182-GGT3P.NCBI.                                               ------------------------------------------------------------ 47402
human.FAM230J-LOC105372942-GGTLC5P.NCBI.rev.compl                                    ------------------------------------------------------------ 46364
human.FAM230A.ncRNA.18487127.18500594-GGTLC3.18516335.18518165.rev.compl.REF         ------------------------------------------------------------ 31039


chimp.LOC112206744-LOC107973052-GGT2.revcompl                                        ------------------------------------------------------------ 49765
chimp54546-135457.revcompl.Pan.troglodytes.clone.rp43-41g5.GenBank:AC099533.36       CCAGGCAGCTCACGAGCAGAGCCACATCCCGGGAGTCCGAGAAAGGTCCTGGCTGGGCTC 52846
human.FAM30B-LOC105372935-GGT2.NCBI.                                                 ------------------------------------------------------------ 48749
human.FAM230E-LOC105377182-GGT3P.NCBI.                                               ------------------------------------------------------------ 47402
human.FAM230J-LOC105372942-GGTLC5P.NCBI.rev.compl                                    ------------------------------------------------------------ 46364
human.FAM230A.ncRNA.18487127.18500594-GGTLC3.18516335.18518165.rev.compl.REF         ------------------------------------------------------------ 31039


chimp.LOC112206744-LOC107973052-GGT2.revcompl                                        -----------------------------------------------------ggatctt 49772
chimp54546-135457.revcompl.Pan.troglodytes.clone.rp43-41g5.GenBank:AC099533.36       AGCCACCTCATTGGCCACGGGCAGCCTTTGTCGTGTGAGCCTTGCTCTCCTGGGGAGGCT 52906
human.FAM30B-LOC105372935-GGT2.NCBI.                                                 -----------------------------------------------------ggatctt 48756
human.FAM230E-LOC105377182-GGT3P.NCBI.                                               -----------------------------------------------------ggatctt 47409
```

```
human.FAM230J-LOC105372942-GGTLC5P.NCBI.rev.compl                           ------------------------------------------------------------ 46364
human.FAM230A.ncRNA.18487127.18500594-GGTLC3.18516335.18518165.rev.compl.REF ------------------------------------------------------------ 31039

chimp.LOC112206744-LOC107973052-GGT2.revcompl                               caggctgtattctagtgcttccctagccctggctcttgcctacacctgcattta------ 49826
chimp54546-135457.revcompl.Pan.troglodytes.clone.rp43-41g5.GenBank:AC099533.36 CAGGCTGATGGCTGATGTGGGCATTGCCGAGGGTAACCTGTGGCCCAGTGTATATGGCCG 52966
human.FAM30B-LOC105372935-GGT2.NCBI.                                        caggctgtattctagtgcttccctagccctggctcttgcctacacctgcattta------ 48810
human.FAM230E-LOC105377182-GGT3P.NCBI.                                      caggctgtattctagtgcttccctagccctggctcttgcctacacctgcattta------ 47463
human.FAM230J-LOC105372942-GGTLC5P.NCBI.rev.compl                           ------------------------------------------------------------ 46364
human.FAM230A.ncRNA.18487127.18500594-GGTLC3.18516335.18518165.rev.compl.REF ------------------------------------------------------------ 31039

chimp.LOC112206744-LOC107973052-GGT2.revcompl                               --------------------------------------------------ccccaacagg 49836
chimp54546-135457.revcompl.Pan.troglodytes.clone.rp43-41g5.GenBank:AC099533.36 GCTCTCCTCAAGCTGCATTCATTCAAGTAGGACCCAGGGTGCATGCCCATCTCCAGCCCA 53026
human.FAM30B-LOC105372935-GGT2.NCBI.                                        --------------------------------------------------ccccaacagg 48820
human.FAM230E-LOC105377182-GGT3P.NCBI.                                      --------------------------------------------------ccccaacagg 47473
human.FAM230J-LOC105372942-GGTLC5P.NCBI.rev.compl                           ------------------------------------------------------------ 46364
human.FAM230A.ncRNA.18487127.18500594-GGTLC3.18516335.18518165.rev.compl.REF ------------------------------------------------------------ 31039

chimp.LOC112206744-LOC107973052-GGT2.revcompl                               gacttgctctcctggactgtgtggcctctcttggttttgatataagcaggagctgtgga- 49895
chimp54546-135457.revcompl.Pan.troglodytes.clone.rp43-41g5.GenBank:AC099533.36 GGGCAGCTCCCCTGTAAGCTGGGTGAGCTACTGAAGCCAAGGTGGGAGGCAGCTGACAAC 53086
human.FAM30B-LOC105372935-GGT2.NCBI.                                        gacttgctctcctggactgtgtggcctctcttggttttgatataagcaggagctgtgga- 48879
human.FAM230E-LOC105377182-GGT3P.NCBI.                                      gacttgctctcctggactgtgtggcctctcttggttttgatataagcaggagctgtgga- 47532
human.FAM230J-LOC105372942-GGTLC5P.NCBI.rev.compl                           ------------------------------------------------------------ 46364
human.FAM230A.ncRNA.18487127.18500594-GGTLC3.18516335.18518165.rev.compl.REF ------------------------------------------------------------ 31039

chimp.LOC112206744-LOC107973052-GGT2.revcompl                               --cccacatggcc--------agtcactgaccctcctccaccaggaacttcctgcaggct 49945
chimp54546-135457.revcompl.Pan.troglodytes.clone.rp43-41g5.GenBank:AC099533.36 ACCACCCATGGCCCATGCGGAGGTGGCAGAAAGGCTGGACTCAGCAGGAACACCAAATCC 53146
human.FAM30B-LOC105372935-GGT2.NCBI.                                        --cccacatggcc--------agtcactgaccctcctccaccaggaacttcctgcaggct 48929
human.FAM230E-LOC105377182-GGT3P.NCBI.                                      --cccacatggcc--------agtcactgaccctcctccaccaggaacttcctgcaggct 47582
human.FAM230J-LOC105372942-GGTLC5P.NCBI.rev.compl                           ------------------------------------------------------------ 46364
human.FAM230A.ncRNA.18487127.18500594-GGTLC3.18516335.18518165.rev.compl.REF ------------------------------------------------------------ 31039

chimp.LOC112206744-LOC107973052-GGT2.revcompl                               caggcaagacaggaggaccccatggctctgggctacagctcagggtttccactgcagagt 50005
chimp54546-135457.revcompl.Pan.troglodytes.clone.rp43-41g5.GenBank:AC099533.36 CGGACCAGGCAGAAACCACCCAAGACTGAGGGGCTCGTGCCAGAGCGGTGGC--CACAGG 53204
human.FAM30B-LOC105372935-GGT2.NCBI.                                        caggcaagacaggaggaccccatggctctgggctacagctcagggtttccactgcagagt 48989
human.FAM230E-LOC105377182-GGT3P.NCBI.                                      caggcaagacaggaggaccccatggctctgggctacagctcagggtttccactgcagagt 47642
human.FAM230J-LOC105372942-GGTLC5P.NCBI.rev.compl                           ------------------------------------------------------------ 46364
human.FAM230A.ncRNA.18487127.18500594-GGTLC3.18516335.18518165.rev.compl.REF ------------------------------------------------------------ 31039

chimp.LOC112206744-LOC107973052-GGT2.revcompl                               tcctcacccaggtccttgaggttacccactcaccagccgcctggatgtccttcacaatct 50065
chimp54546-135457.revcompl.Pan.troglodytes.clone.rp43-41g5.GenBank:AC099533.36 TAAGAACCCGGGCCCAGGTTGT--------------GTGTGGCGGAATCCTCCATGTCCC 53251
human.FAM30B-LOC105372935-GGT2.NCBI.                                        tcctcacccaggtccttgaggttacccactcaccagccgcctggatgtccttcacaatct 49049
human.FAM230E-LOC105377182-GGT3P.NCBI.                                      tcctcacccaggtccttgaggttacccactcaccagccgcctggatgtccttcacaatct 47702
human.FAM230J-LOC105372942-GGTLC5P.NCBI.rev.compl                           ------------------------------------------------------------ 46364
human.FAM230A.ncRNA.18487127.18500594-GGTLC3.18516335.18518165.rev.compl.REF ------------------------------------------------------------ 31039

chimp.LOC112206744-LOC107973052-GGT2.revcompl                               gggccatgaggctgccgttgtagaaggcctgggcgccctcgatggccagcatctcgtagg 50125
chimp54546-135457.revcompl.Pan.troglodytes.clone.rp43-41g5.GenBank:AC099533.36 AGGGCTTAGCGTAGCAAAGGAAGACCAGCCGGGTCACCCTGGTGGCCATC----TGTCCC 53307
human.FAM30B-LOC105372935-GGT2.NCBI.                                        gggccatgaggctgccgttgtagaaggcctgggcaccctcgatggccagcatctcatagg 49109
human.FAM230E-LOC105377182-GGT3P.NCBI.                                      gggccatgaggctgccgttgtagaaggcctgggcaccctcgatggccagcatctcgtagg 47762
human.FAM230J-LOC105372942-GGTLC5P.NCBI.rev.compl                           ------------------------------------------------------------ 46364
human.FAM230A.ncRNA.18487127.18500594-GGTLC3.18516335.18518165.rev.compl.REF ------------------------------------------------------------ 31039

chimp.LOC112206744-LOC107973052-GGT2.revcompl                               tgtcagccagccgcggcagggtcagtctctcccccctcccgaagcacctttctatcccggc 50185
chimp54546-135457.revcompl.Pan.troglodytes.clone.rp43-41g5.GenBank:AC099533.36 TGTCCCACCTGCAGAGTCAGAACAGCCTCTCCCCAGTGGGGATCATCTCTCTCTGCCAAA 53367
human.FAM30B-LOC105372935-GGT2.NCBI.                                        tgtcagccagccgcggcagggtcagtctctcccccctcccgaagcacctttctatcccggc 49169
human.FAM230E-LOC105377182-GGT3P.NCBI.                                      tgtcagccagccgcggcagggtcagtctctcccccctcccgaagcacctttctatcccggc 47822
human.FAM230J-LOC105372942-GGTLC5P.NCBI.rev.compl                           ------------------------------------------------------------ 46364
human.FAM230A.ncRNA.18487127.18500594-GGTLC3.18516335.18518165.rev.compl.REF ------------------------------------------------------------ 31039

chimp.LOC112206744-LOC107973052-GGT2.revcompl                               agaacacctcactggggcagaggggg---------------------------------- 50211
chimp54546-135457.revcompl.Pan.troglodytes.clone.rp43-41g5.GenBank:AC099533.36 GCAATAGCGGTCCCTGCCCCAACCAGACTACCCCACTCAGTGGAGTTACGGATGCTGCTC 53427
human.FAM30B-LOC105372935-GGT2.NCBI.                                        agaacacctcaccggggcagaggggg---------------------------------- 49195
human.FAM230E-LOC105377182-GGT3P.NCBI.                                      agaacacctcactggggcagaggggg---------------------------------- 47848
human.FAM230J-LOC105372942-GGTLC5P.NCBI.rev.compl                           ------------------------------------------------------------ 46364
human.FAM230A.ncRNA.18487127.18500594-GGTLC3.18516335.18518165.rev.compl.REF ------------------------------------------------------------ 31039

chimp.LOC112206744-LOC107973052-GGT2.revcompl                               -------ctcatgtgaggcagcaggtggggtggactcagctagaccacccccacacccg 50264
chimp54546-135457.revcompl.Pan.troglodytes.clone.rp43-41g5.GenBank:AC099533.36 CAGCATCCTAACACTGCCCAGCTGGTGCCTGCCTGTGCTCACCCACACCCCCCAGGCCAG 53487
human.FAM30B-LOC105372935-GGT2.NCBI.                                        -------ctcatgtgaggcagcaggtggggtggactcagctagaccacccccacacctg 49248
human.FAM230E-LOC105377182-GGT3P.NCBI.                                      -------ctcatgtgaggcagcaggtggggtggactcagctagaccacccccacacctg 47901
human.FAM230J-LOC105372942-GGTLC5P.NCBI.rev.compl                           ------------------------------------------------------------ 46364
human.FAM230A.ncRNA.18487127.18500594-GGTLC3.18516335.18518165.rev.compl.REF ------------------------------------------------------------ 31039

chimp.LOC112206744-LOC107973052-GGT2.revcompl                               tccatacaggagaccagcacaaagcaggggcagcgcctgtcacaggtgggtggccctgtc 50324
chimp54546-135457.revcompl.Pan.troglodytes.clone.rp43-41g5.GenBank:AC099533.36 CCTTCCCTGCAGCCTGGGCTTGGCCACCTTGGCCTGATTGAGCACTGAGGACTCCTGGGC 53547
human.FAM30B-LOC105372935-GGT2.NCBI.                                        cccacacaggagaccagcacaaagcaggggcagcacctgtcacaggtgggtggccctgtc 49308
human.FAM230E-LOC105377182-GGT3P.NCBI.                                      cccacacaggagaacggagcagaacaggggcagcgcctgtcacaggtgggtggccctgtc 47961
human.FAM230J-LOC105372942-GGTLC5P.NCBI.rev.compl                           ------------------------------------------------------------ 46364
human.FAM230A.ncRNA.18487127.18500594-GGTLC3.18516335.18518165.rev.compl.REF ------------------------------------------------------------ 31039

chimp.LOC112206744-LOC107973052-GGT2.revcompl                               actcagcgctcatcctcctagtgtcccttccgggaacctcctagtgtc------------ 50372
chimp54546-135457.revcompl.Pan.troglodytes.clone.rp43-41g5.GenBank:AC099533.36 ACCCAGCCCCATCACTGCACCTGCTGCTTCCAGCCCCACCCCACCGGCTCTGGGGTTCTT 53607
human.FAM30B-LOC105372935-GGT2.NCBI.                                        actcagcgctcatcctcctagtgtcccttccgggagcctcctagtgtc------------ 49356
human.FAM230E-LOC105377182-GGT3P.NCBI.                                      actcagcgctcgtcctcctagtgtcccttccgggagcctcctagtgtc------------ 48009
human.FAM230J-LOC105372942-GGTLC5P.NCBI.rev.compl                           ------------------------------------------------------------ 46364
human.FAM230A.ncRNA.18487127.18500594-GGTLC3.18516335.18518165.rev.compl.REF ------------------------------------------------------------ 31039

chimp.LOC112206744-LOC107973052-GGT2.revcompl                               ------------------------------------------------------------ 50372
chimp54546-135457.revcompl.Pan.troglodytes.clone.rp43-41g5.GenBank:AC099533.36 CCCAGTGGCGCTGATCATGAAATCAACATGCACACAAGTCGTCTCAGGAAACTTTTTAAT 53667
human.FAM30B-LOC105372935-GGT2.NCBI.                                        ------------------------------------------------------------ 49356
human.FAM230E-LOC105377182-GGT3P.NCBI.                                      ------------------------------------------------------------ 48009
human.FAM230J-LOC105372942-GGTLC5P.NCBI.rev.compl                           ------------------------------------------------------------ 46364
human.FAM230A.ncRNA.18487127.18500594-GGTLC3.18516335.18518165.rev.compl.REF ------------------------------------------------------------ 31039

chimp.LOC112206744-LOC107973052-GGT2.revcompl                               ------------------------------------------------------------ 50372
chimp54546-135457.revcompl.Pan.troglodytes.clone.rp43-41g5.GenBank:AC099533.36 GAAAGTGTCGGCCATGGTGGTGTGTAGGTGGCTGAGCTCAGATTGCAGCTGCTAAGACAC 53727
human.FAM30B-LOC105372935-GGT2.NCBI.                                        ------------------------------------------------------------ 49356
human.FAM230E-LOC105377182-GGT3P.NCBI.                                      ------------------------------------------------------------ 48009
human.FAM230J-LOC105372942-GGTLC5P.NCBI.rev.compl                           ------------------------------------------------------------ 46364
human.FAM230A.ncRNA.18487127.18500594-GGTLC3.18516335.18518165.rev.compl.REF ------------------------------------------------------------ 31039

chimp.LOC112206744-LOC107973052-GGT2.revcompl                               ----------------------------ccttgccactcaggacacatggccagccacag 50404
chimp54546-135457.revcompl.Pan.troglodytes.clone.rp43-41g5.GenBank:AC099533.36 CAGCCACTTACCAAGAGAAAGCCAGGCTGCTTCAAACTCAGGGCCCGAGGCAAAAAAGCA 53787
human.FAM30B-LOC105372935-GGT2.NCBI.                                        ----------------------------ccttgccactcaggacacgtggccagccacag 49388
human.FAM230E-LOC105377182-GGT3P.NCBI.                                      ----------------------------ccttgccactcaggacacgtggccagccacag 48041
human.FAM230J-LOC105372942-GGTLC5P.NCBI.rev.compl                           ------------------------------------------------------------ 46364
human.FAM230A.ncRNA.18487127.18500594-GGTLC3.18516335.18518165.rev.compl.REF ------------------------------------------------------------ 31039

chimp.LOC112206744-LOC107973052-GGT2.revcompl                               tggccactgggaccccacgctcagaatgtgtccccacacgtggtgggaagggtctgtatc 50464
chimp54546-135457.revcompl.Pan.troglodytes.clone.rp43-41g5.GenBank:AC099533.36 TCACTTCTGGTCGGGGAGTCTGGAAGCCATGCCTTGTGGGAGGTCACACTGGCATCTAGG 53847
human.FAM30B-LOC105372935-GGT2.NCBI.                                        tggccactgggaccccatgctcagaatgtgtccccacacgtggtgggaagggtctgtatc 49448
human.FAM230E-LOC105377182-GGT3P.NCBI.                                      tggccactgggaccccacgctcagaatgtgtccccacacgtggtgggaagggtctgtatc 48101
human.FAM230J-LOC105372942-GGTLC5P.NCBI.rev.compl                           ------------------------------------------------------------ 46364
human.FAM230A.ncRNA.18487127.18500594-GGTLC3.18516335.18518165.rev.compl.REF ------------------------------------------------------------ 31039

chimp.LOC112206744-LOC107973052-GGT2.revcompl                               tcctcatcccattatcagcacagg------------gtcctgaaggcagagggccactcc 50512
chimp54546-135457.revcompl.Pan.troglodytes.clone.rp43-41g5.GenBank:AC099533.36 CCTTCGCCTGCATTGCAGAAGGAGAGCCGGGTCCCCCTCCTGGAGAACGCTGCGTTCCCC 53907
human.FAM30B-LOC105372935-GGT2.NCBI.                                        tcctcatcccattatcagcacagg------------gtcctgaaggcagagggccgctcc 49496
human.FAM230E-LOC105377182-GGT3P.NCBI.                                      tcctcatcccattatcagcacagg------------gtcctgaaggcagagggccgctcc 48149
human.FAM230J-LOC105372942-GGTLC5P.NCBI.rev.compl                           ------------------------------------------------------------ 46364
human.FAM230A.ncRNA.18487127.18500594-GGTLC3.18516335.18518165.rev.compl.REF ------------------------------------------------------------ 31039

chimp.LOC112206744-LOC107973052-GGT2.revcompl                               actgctgctacggcctgcaaggtccttgggctgtgcctgccctgcctgtgtcagggg--g 50570
chimp54546-135457.revcompl.Pan.troglodytes.clone.rp43-41g5.GenBank:AC099533.36 AGCCCCACACCGGCTTTGCCACCACACAGGCTTTTGAGGCAGGAGGTGGGTAAGACGTAG 53967
human.FAM30B-LOC105372935-GGT2.NCBI.                                        actgctgctacggcctgcaaggtccttgggctgtgcctgccctgcctgtgtcagggg--g 49554
human.FAM230E-LOC105377182-GGT3P.NCBI.                                      actgctgctacggcctgcaaggtccttgggctgtgcctgccctgcctgtgtcagggg--g 48207
human.FAM230J-LOC105372942-GGTLC5P.NCBI.rev.compl                           ------------------------------------------------------------ 46364
```

```
human.FAM230A.ncRNA.18487127.18500594-GGTLC3.18516335.18518165.rev.compl.REF        ------------------------------------------------------------ 31039

chimp.LOC112206744-LOC107973052-GGT2.revcompl                                       ccgcacccacagacatgccacaagacaggc------------------------------ 50600
chimp54546-135457.revcompl.Pan.troglodytes.clone.rp43-41g5.GenBank:AC099533.36      CTGTAGACCCAAAGCAACCACCAGCCCTGGGACCCTGCGGGAGAGGAGCACTTTTAGAAA 54027
human.FAM30B-LOC105372935-GGT2.NCBI.                                                ccgcacccacagacataccacaagacaggc------------------------------ 49584
human.FAM230E-LOC105377182-GGT3P.NCBI.                                              ccgcacccacagacataccacaagacaggc------------------------------ 48237
human.FAM230J-LOC105372942-GGTLC5P.NCBI.rev.compl                                   ------------------------------------------------------------ 46364
human.FAM230A.ncRNA.18487127.18500594-GGTLC3.18516335.18518165.rev.compl.REF        ------------------------------------------------------------ 31039

chimp.LOC112206744-LOC107973052-GGT2.revcompl                                       ------------------------------------------------------------ 50600
chimp54546-135457.revcompl.Pan.troglodytes.clone.rp43-41g5.GenBank:AC099533.36      ATGGAAAAGTATGGTCATCCCATCATTAGACAGCACACATCCTACATAAATAAAAAGTCG 54087
human.FAM30B-LOC105372935-GGT2.NCBI.                                                ------------------------------------------------------------ 49584
human.FAM230E-LOC105377182-GGT3P.NCBI.                                              ------------------------------------------------------------ 48237
human.FAM230J-LOC105372942-GGTLC5P.NCBI.rev.compl                                   ------------------------------------------------------------ 46364
human.FAM230A.ncRNA.18487127.18500594-GGTLC3.18516335.18518165.rev.compl.REF        ------------------------------------------------------------ 31039

chimp.LOC112206744-LOC107973052-GGT2.revcompl                                       ------------------------------------------------------------ 50600
chimp54546-135457.revcompl.Pan.troglodytes.clone.rp43-41g5.GenBank:AC099533.36      TATGGGGAAGGAGGTTGGGGAGGGAATAAAAAATTGGCACAGACATTGATAGACTGGTTT 54147
human.FAM30B-LOC105372935-GGT2.NCBI.                                                ------------------------------------------------------------ 49584
human.FAM230E-LOC105377182-GGT3P.NCBI.                                              ------------------------------------------------------------ 48237
human.FAM230J-LOC105372942-GGTLC5P.NCBI.rev.compl                                   ------------------------------------------------------------ 46364
human.FAM230A.ncRNA.18487127.18500594-GGTLC3.18516335.18518165.rev.compl.REF        ------------------------------------------------------------ 31039

chimp.LOC112206744-LOC107973052-GGT2.revcompl                                       ------------------------------------------------------------ 50600
chimp54546-135457.revcompl.Pan.troglodytes.clone.rp43-41g5.GenBank:AC099533.36      CCAGTTTCAAGGTAACAGATGCACATCATGAGACCAGAGGAGGCAGAGACAAGGCTGGAT 54207
human.FAM30B-LOC105372935-GGT2.NCBI.                                                ------------------------------------------------------------ 49584
human.FAM230E-LOC105377182-GGT3P.NCBI.                                              ------------------------------------------------------------ 48237
human.FAM230J-LOC105372942-GGTLC5P.NCBI.rev.compl                                   ------------------------------------------------------------ 46364
human.FAM230A.ncRNA.18487127.18500594-GGTLC3.18516335.18518165.rev.compl.REF        ------------------------------------------------------------ 31039

chimp.LOC112206744-LOC107973052-GGT2.revcompl                                       --tgctgctcgatgacggtccgcttgttttccagggctgccgccaagcccttgcccacag 50658
chimp54546-135457.revcompl.Pan.troglodytes.clone.rp43-41g5.GenBank:AC099533.36      TTGGCTTTTCTAAGCAACATGTGTTCCTGTGCAGGGCTGAATGGTCGCTGAGACAGAGAT 54267
human.FAM30B-LOC105372935-GGT2.NCBI.                                                --tgctgctcgatgacagtccgcttgttttccaggactgccgccaagcccttgcccacag 49642
human.FAM230E-LOC105377182-GGT3P.NCBI.                                              --tgctgctcgatgacggtccgcttgttttccaggactgccgccaagcccttgcccacgg 48295
human.FAM230J-LOC105372942-GGTLC5P.NCBI.rev.compl                                   ------------------------------------------------------------ 46364
human.FAM230A.ncRNA.18487127.18500594-GGTLC3.18516335.18518165.rev.compl.REF        ------------------------------------------------------------ 31039

chimp.LOC112206744-LOC107973052-GGT2.revcompl                                       ggaagccctggcgggccagctg-------------------------------------- 50680
chimp54546-135457.revcompl.Pan.troglodytes.clone.rp43-41g5.GenBank:AC099533.36      GGAAGCCAGGACAAGGGAGCCCACCGGGCCCAGATAGGTACAGAGAGCAGAGGCTCCTGT 54327
human.FAM30B-LOC105372935-GGT2.NCBI.                                                ggaagccctggcgggccagctg-------------------------------------- 49664
human.FAM230E-LOC105377182-GGT3P.NCBI.                                              ggaagccctggcgggccagctg-------------------------------------- 48317
human.FAM230J-LOC105372942-GGTLC5P.NCBI.rev.compl                                   ------------------------------------------------------------ 46364
human.FAM230A.ncRNA.18487127.18500594-GGTLC3.18516335.18518165.rev.compl.REF        ------------------------------------------------------------ 31039

chimp.LOC112206744-LOC107973052-GGT2.revcompl                                       ------------------------------------------------------------ 50680
chimp54546-135457.revcompl.Pan.troglodytes.clone.rp43-41g5.GenBank:AC099533.36      TCTGTCCTCGCCACCCACGAGGGTGACACTGCTTGTAAATGGTGGCTGTACTCTCCCAGC 54387
human.FAM30B-LOC105372935-GGT2.NCBI.                                                ------------------------------------------------------------ 49664
human.FAM230E-LOC105377182-GGT3P.NCBI.                                              ------------------------------------------------------------ 48317
human.FAM230J-LOC105372942-GGTLC5P.NCBI.rev.compl                                   ------------------------------------------------------------ 46364
human.FAM230A.ncRNA.18487127.18500594-GGTLC3.18516335.18518165.rev.compl.REF        ------------------------------------------------------------ 31039

chimp.LOC112206744-LOC107973052-GGT2.revcompl                                       ------------------------------------------------------------ 50680
chimp54546-135457.revcompl.Pan.troglodytes.clone.rp43-41g5.GenBank:AC099533.36      AAGAAAAAAGCACAACTAAATCCACACTGCACACAGACGCAGACAGAAAGCCTTCAAGTG 54447
human.FAM30B-LOC105372935-GGT2.NCBI.                                                ------------------------------------------------------------ 49664
human.FAM230E-LOC105377182-GGT3P.NCBI.                                              ------------------------------------------------------------ 48317
human.FAM230J-LOC105372942-GGTLC5P.NCBI.rev.compl                                   ------------------------------------------------------------ 46364
human.FAM230A.ncRNA.18487127.18500594-GGTLC3.18516335.18518165.rev.compl.REF        ------------------------------------------------------------ 31039

chimp.LOC112206744-LOC107973052-GGT2.revcompl                                       ------------------------------------------gatgctgggctggaagag 50698
chimp54546-135457.revcompl.Pan.troglodytes.clone.rp43-41g5.GenBank:AC099533.36      GCTCTGTTTTCTGCTCCCTGCCTTGCCAGGTCCACAAGCAGAGAGGAGTGTCAGGCGCAT 54507
human.FAM30B-LOC105372935-GGT2.NCBI.                                                ------------------------------------------gatgctgggctggaagag 49682
human.FAM230E-LOC105377182-GGT3P.NCBI.                                              ------------------------------------------gatgctgggctggaagag 48335
human.FAM230J-LOC105372942-GGTLC5P.NCBI.rev.compl                                   ------------------------------------------------------------ 46364
human.FAM230A.ncRNA.18487127.18500594-GGTLC3.18516335.18518165.rev.compl.REF        ------------------------------------------------------------ 31039

chimp.LOC112206744-LOC107973052-GGT2.revcompl                                       gcgagcccagggcagctgcccatgccactgatgtgccagctcatagcctcggatctcccc 50758
chimp54546-135457.revcompl.Pan.troglodytes.clone.rp43-41g5.GenBank:AC099533.36      GGCCCCACTGTCAGGCTCCCCAGTGAGCTGCGGGTTCAGCAGGAGCTGCCCACTGACACA 54567
human.FAM30B-LOC105372935-GGT2.NCBI.                                                gcgagcccagggcagccgcccatgccgctggtgtgccagctcatagcctcggatctcccc 49742
human.FAM230E-LOC105377182-GGT3P.NCBI.                                              gcgagcccagggcagccgcccatgccgctggtgtgccagctcatagcctcggatctcccc 48395
human.FAM230J-LOC105372942-GGTLC5P.NCBI.rev.compl                                   ------------------------------------------------------------ 46364
human.FAM230A.ncRNA.18487127.18500594-GGTLC3.18516335.18518165.rev.compl.REF        ------------------------------------------------------------ 31039

chimp.LOC112206744-LOC107973052-GGT2.revcompl                                       aggcaccgccaccgacagccctcctggggagagagagccacagttagtgaccctgagtgg 50818
chimp54546-135457.revcompl.Pan.troglodytes.clone.rp43-41g5.GenBank:AC099533.36      CAGGGGACACCCACTCCTGCCACCTTGGGAGCGGTTGCCAGACAGAGCCGCACTGGGTGC 54627
human.FAM30B-LOC105372935-GGT2.NCBI.                                                aggcaccgccaccgacagccctcctggggagagagagccacagttagtgaccctgagtgg 49802
human.FAM230E-LOC105377182-GGT3P.NCBI.                                              aggcaccgccaccgacagccctcctggggagagagagccacagttagtgaccctgagtgg 48455
human.FAM230J-LOC105372942-GGTLC5P.NCBI.rev.compl                                   ------------------------------------------------------------ 46364
human.FAM230A.ncRNA.18487127.18500594-GGTLC3.18516335.18518165.rev.compl.REF        ------------------------------------------------------------ 31039

chimp.LOC112206744-LOC107973052-GGT2.revcompl                                       gggacatccggatctctcgcaggcagcatcccaggcacagtccctgactcgctttacaga 50878
chimp54546-135457.revcompl.Pan.troglodytes.clone.rp43-41g5.GenBank:AC099533.36      TG---------------------------------------------------------- 54629
human.FAM30B-LOC105372935-GGT2.NCBI.                                                gggacatcgggatctctagcaggcagcatcccaggcacagtccctgactcatttacaga 49862
human.FAM230E-LOC105377182-GGT3P.NCBI.                                              gggacatcgggatctctcgcaggcagcatcccaggcacagtccctgactcgctttacaga 48515
human.FAM230J-LOC105372942-GGTLC5P.NCBI.rev.compl                                   ------------------------------------------------------------ 46364
human.FAM230A.ncRNA.18487127.18500594-GGTLC3.18516335.18518165.rev.compl.REF        ------------------------------------------------------------ 31039

chimp.LOC112206744-LOC107973052-GGT2.revcompl                                       tggggcaatgaggcttaggaggaaagatttttttttctttttttgagttggggtcttgcca 50938
chimp54546-135457.revcompl.Pan.troglodytes.clone.rp43-41g5.GenBank:AC099533.36      ---------GTGTCATCCAGGGACCCCACACACTTCCTTAAATGTGATCCTGCTTCCCTC 54680
human.FAM30B-LOC105372935-GGT2.NCBI.                                                tggggcaatgaggcttaggaggaaagatttttttttctttttttgagttggggtcttgcca 49922
human.FAM230E-LOC105377182-GGT3P.NCBI.                                              tggggcaatgaggcttaggaggaaagatttttttttctttttttgagttggggtcttgcca 48575
human.FAM230J-LOC105372942-GGTLC5P.NCBI.rev.compl                                   ------------------------------------------------------------ 46364
human.FAM230A.ncRNA.18487127.18500594-GGTLC3.18516335.18518165.rev.compl.REF        ------------------------------------------------------------ 31039

chimp.LOC112206744-LOC107973052-GGT2.revcompl                                       tcttgcccagcctgatctcgaactcctggattcaagcaatcctcccacctcagcctcctg 50998
chimp54546-135457.revcompl.Pan.troglodytes.clone.rp43-41g5.GenBank:AC099533.36      TGGGCAGCTGCATCCTCTCCTCCTGCAGGACTGTCTGGAAACTTGGCTCTCAGTTTGCT- 54739
human.FAM30B-LOC105372935-GGT2.NCBI.                                                tcttgcccagcctgatctcgaactcctggattcaagcaatcctcccacctcagcctcctg 49982
human.FAM230E-LOC105377182-GGT3P.NCBI.                                              tcttgcccagcccaatctcgaactcctggattcaagcaatcctcccacctcagcctcctg 48635
human.FAM230J-LOC105372942-GGTLC5P.NCBI.rev.compl                                   ------------------------------------------------------------ 46364
human.FAM230A.ncRNA.18487127.18500594-GGTLC3.18516335.18518165.rev.compl.REF        ------------------------------------------------------------ 31039

chimp.LOC112206744-LOC107973052-GGT2.revcompl                                       agtagctgagattacaggcgtgaacaccacac---ccagcagaaggggatttttaatttt 51055
chimp54546-135457.revcompl.Pan.troglodytes.clone.rp43-41g5.GenBank:AC099533.36      -CTCCCTTCTCTCCTCTGCCTGCCCAAGCCCCTCTTTCTAAAAAAGTGATGCCATGTTCA 54798
human.FAM30B-LOC105372935-GGT2.NCBI.                                                agtcgctgagattacaggcgtgaacagcacac---ctagcagaaggggatttttaatttt 50039
human.FAM230E-LOC105377182-GGT3P.NCBI.                                              agtcgctgagattacaggtgtgaacagcacac---ctagcagaaggggatttttaatttt 48692
human.FAM230J-LOC105372942-GGTLC5P.NCBI.rev.compl                                   ------------------------------------------------------------ 46364
human.FAM230A.ncRNA.18487127.18500594-GGTLC3.18516335.18518165.rev.compl.REF        ------------------------------------------------------------ 31039

chimp.LOC112206744-LOC107973052-GGT2.revcompl                                       ttaatttatttttaattttaatttttttttttttaggaggggatgtttaatttttttt- 51114
chimp54546-135457.revcompl.Pan.troglodytes.clone.rp43-41g5.GenBank:AC099533.36      TGGGGTTATTTCTTGAAAATAC--------TTGGCGGCCTCCATGCTTCTGTTTTCTTTG 54850
human.FAM30B-LOC105372935-GGT2.NCBI.                                                taaatttatttttttaattttaatttttttttttttaggaggggatgtttaatttttttttt 50099
human.FAM230E-LOC105377182-GGT3P.NCBI.                                              taaatttatttttttaattttaattgtttttttttaggaggggatgtttaatttttttttt 48752
human.FAM230J-LOC105372942-GGTLC5P.NCBI.rev.compl                                   ------------------------------------------------------------ 46364
human.FAM230A.ncRNA.18487127.18500594-GGTLC3.18516335.18518165.rev.compl.REF        ------------------------------------------------------------ 31039

chimp.LOC112206744-LOC107973052-GGT2.revcompl                                       -aggaggggctcagca-gtaggagtgtacatggacaagggatgtctgaggagggcacagg 51172
chimp54546-135457.revcompl.Pan.troglodytes.clone.rp43-41g5.GenBank:AC099533.36      AGCCAGGTGGTCAGGAGGGCTTACAAAGAATGCCTGGGCTCCCCCGCAGGTGTCAGCAGA 54910
human.FAM30B-LOC105372935-GGT2.NCBI.                                                taggaggggctcagcaggtaggagtgtacatggaccagggatgtctgaggagggcacagc 50159
human.FAM230E-LOC105377182-GGT3P.NCBI.                                              taggaggggctcagcaggtaggagtgtacatggaccagggatgtctgaggagggcacagc 48812
human.FAM230J-LOC105372942-GGTLC5P.NCBI.rev.compl                                   ------------------------------------------------------------ 46364
human.FAM230A.ncRNA.18487127.18500594-GGTLC3.18516335.18518165.rev.compl.REF        ------------------------------------------------------------ 31039

chimp.LOC112206744-LOC107973052-GGT2.revcompl                                       aggggaagcagtagcatgcggctgggttttgctgtcccaggatgaggtgtctgtctgtgc 51232
chimp54546-135457.revcompl.Pan.troglodytes.clone.rp43-41g5.GenBank:AC099533.36      TGGGGTAGC----GAATGGTCCTGTGCCTCCACCTGCTCCGGGAGGGAGTCT-------- 54958
human.FAM30B-LOC105372935-GGT2.NCBI.                                                aggggaagcagtagcatgcggctgggttttgctgtcccaggatgaggtgtctgtctgtac 50219
human.FAM230E-LOC105377182-GGT3P.NCBI.                                              aggggaagcagtagcatgcggctgggttttgctgtcccaggatgaggtgtctgtctgtgc 48872
human.FAM230J-LOC105372942-GGTLC5P.NCBI.rev.compl                                   ------------------------------------------------------------ 46364
human.FAM230A.ncRNA.18487127.18500594-GGTLC3.18516335.18518165.rev.compl.REF        ------------------------------------------------------------ 31039
```

```
chimp.LOC112206744-LOC107973052-GGT2.revcompl                                      aggtgcctgcatgtctaaaatcctgtgccaggccagacccctcccatctcgctgaccac 51292
chimp54546-135457.revcompl.Pan.troglodytes.clone.rp43-41g5.GenBank:AC099533.36     ---------CCTGTCTCTAGGCCTGGCCCCTTCCTAACCCTCCACGTATCCTGTTCTCCA 55009
human.FAM30B-LOC105372935-GGT2.NCBI.                                               aggtgcctgcatgtctaaaatcctgtgccaggccagacccctcccatctcgctgaccac 50279
human.FAM230E-LOC105377182-GGT3P.NCBI.                                             aggtgcctgcatgtctaaaatcctgtgccaggccagacccctcccatctcgctgaccac 48932
human.FAM230J-LOC105372942-GGTLC5P.NCBI.rev.compl                                  ------------------------------------------------------------ 46364
human.FAM230A.ncRNA.18487127.18500594-GGTLC3.18516335.18518165.rev.compl.REF       ------------------------------------------------------------ 31039

chimp.LOC112206744-LOC107973052-GGT2.revcompl                                      aaggccttatcctgtaagactcatgggctccaccagaatgtgccaaaacaagagcagatc 51352
chimp54546-135457.revcompl.Pan.troglodytes.clone.rp43-41g5.GenBank:AC099533.36     GAGACTTCAGAATCCACTCCTGAGAACAGCGGAGCCAGGCGCTTAGAGGAAGACCAAATG 55069
human.FAM30B-LOC105372935-GGT2.NCBI.                                               aaggccttatcctgtaagactcatgggctccaccagaatgtgccaaaacaagagcagatc 50339
human.FAM230E-LOC105377182-GGT3P.NCBI.                                             aaggccttatcctgtaagactcatgggctccaccagaatgtgccaaaacaagagcagatc 48992
human.FAM230J-LOC105372942-GGTLC5P.NCBI.rev.compl                                  ------------------------------------------------------------ 46364
human.FAM230A.ncRNA.18487127.18500594-GGTLC3.18516335.18518165.rev.compl.REF       ------------------------------------------------------------ 31039

chimp.LOC112206744-LOC107973052-GGT2.revcompl                                      ccaccctgacccaggtcaagcacaggccaccttcaagacac------------------- 51393
chimp54546-135457.revcompl.Pan.troglodytes.clone.rp43-41g5.GenBank:AC099533.36     CTGCCAGGACACGGATTGTCCAGGGATTACATTCCAGCATCTTATTAGGTATCTGGATCT 55129
human.FAM30B-LOC105372935-GGT2.NCBI.                                               ccaccctgacccaggtcaagcacaggccaccttcaagacac------------------- 50380
human.FAM230E-LOC105377182-GGT3P.NCBI.                                             ccaccttgaccgaggtcaagcacaggccaccttcaagacac------------------- 49033
human.FAM230J-LOC105372942-GGTLC5P.NCBI.rev.compl                                  ------------------------------------------------------------ 46364
human.FAM230A.ncRNA.18487127.18500594-GGTLC3.18516335.18518165.rev.compl.REF       ------------------------------------------------------------ 31039

chimp.LOC112206744-LOC107973052-GGT2.revcompl                                      ------------------------------------------------------------ 51393
chimp54546-135457.revcompl.Pan.troglodytes.clone.rp43-41g5.GenBank:AC099533.36     GTTGGGGAAAAAATTAGAAACTACGTATAAAACTTAAAAATATTCAAGCATCAAAAGGTT 55189
human.FAM30B-LOC105372935-GGT2.NCBI.                                               ------------------------------------------------------------ 50380
human.FAM230E-LOC105377182-GGT3P.NCBI.                                             ------------------------------------------------------------ 49033
human.FAM230J-LOC105372942-GGTLC5P.NCBI.rev.compl                                  ------------------------------------------------------------ 46364
human.FAM230A.ncRNA.18487127.18500594-GGTLC3.18516335.18518165.rev.compl.REF       ------------------------------------------------------------ 31039

chimp.LOC112206744-LOC107973052-GGT2.revcompl                                      ------------------------------------------------------------ 51393
chimp54546-135457.revcompl.Pan.troglodytes.clone.rp43-41g5.GenBank:AC099533.36     ATTTAGGATGAAAGTTTTAAAACAAGTCATCAGCAAGCTGCTGCCACCAAGTGGAGACTT 55249
human.FAM30B-LOC105372935-GGT2.NCBI.                                               ------------------------------------------------------------ 50380
human.FAM230E-LOC105377182-GGT3P.NCBI.                                             ------------------------------------------------------------ 49033
human.FAM230J-LOC105372942-GGTLC5P.NCBI.rev.compl                                  ------------------------------------------------------------ 46364
human.FAM230A.ncRNA.18487127.18500594-GGTLC3.18516335.18518165.rev.compl.REF       ------------------------------------------------------------ 31039

chimp.LOC112206744-LOC107973052-GGT2.revcompl                                      ------------------------------------------------------------ 51393
chimp54546-135457.revcompl.Pan.troglodytes.clone.rp43-41g5.GenBank:AC099533.36     ATACAAAAGTTGAGCGAGTCCACTGAGCTGAGAGGACAGAAATGAAGTCACCTGTGCTGG 55309
human.FAM30B-LOC105372935-GGT2.NCBI.                                               ------------------------------------------------------------ 50380
human.FAM230E-LOC105377182-GGT3P.NCBI.                                             ------------------------------------------------------------ 49033
human.FAM230J-LOC105372942-GGTLC5P.NCBI.rev.compl                                  ------------------------------------------------------------ 46364
human.FAM230A.ncRNA.18487127.18500594-GGTLC3.18516335.18518165.rev.compl.REF       ------------------------------------------------------------ 31039

chimp.LOC112206744-LOC107973052-GGT2.revcompl                                      ------------------------------------------------------------ 51393
chimp54546-135457.revcompl.Pan.troglodytes.clone.rp43-41g5.GenBank:AC099533.36     GGCAGGGGCAGGGACACTGGGAGCAGGGAGTGTGTGGGCAGAGAAGCCAGAGAAGTCCAG 55369
human.FAM30B-LOC105372935-GGT2.NCBI.                                               ------------------------------------------------------------ 50380
human.FAM230E-LOC105377182-GGT3P.NCBI.                                             ------------------------------------------------------------ 49033
human.FAM230J-LOC105372942-GGTLC5P.NCBI.rev.compl                                  ------------------------------------------------------------ 46364
human.FAM230A.ncRNA.18487127.18500594-GGTLC3.18516335.18518165.rev.compl.REF       ------------------------------------------------------------ 31039

chimp.LOC112206744-LOC107973052-GGT2.revcompl                                      ------------------------------------------------------------ 51393
chimp54546-135457.revcompl.Pan.troglodytes.clone.rp43-41g5.GenBank:AC099533.36     GCCTGTGGAAGCCAAACAGGAGAGCGTGGGCCGGAAGGGCGGTCAGAATCGGGGGATGAG 55429
human.FAM30B-LOC105372935-GGT2.NCBI.                                               ------------------------------------------------------------ 50380
human.FAM230E-LOC105377182-GGT3P.NCBI.                                             ------------------------------------------------------------ 49033
human.FAM230J-LOC105372942-GGTLC5P.NCBI.rev.compl                                  ------------------------------------------------------------ 46364
human.FAM230A.ncRNA.18487127.18500594-GGTLC3.18516335.18518165.rev.compl.REF       ------------------------------------------------------------ 31039

chimp.LOC112206744-LOC107973052-GGT2.revcompl                                      ------------------------------------------------------------ 51393
chimp54546-135457.revcompl.Pan.troglodytes.clone.rp43-41g5.GenBank:AC099533.36     GTCGCTCTCCCTGGAGAATGAACCCTAAAGTGCGTAGCCTGGGATTCCCTCCCTGGGGGT 55489
human.FAM30B-LOC105372935-GGT2.NCBI.                                               ------------------------------------------------------------ 50380
human.FAM230E-LOC105377182-GGT3P.NCBI.                                             ------------------------------------------------------------ 49033
human.FAM230J-LOC105372942-GGTLC5P.NCBI.rev.compl                                  ------------------------------------------------------------ 46364
human.FAM230A.ncRNA.18487127.18500594-GGTLC3.18516335.18518165.rev.compl.REF       ------------------------------------------------------------ 31039

chimp.LOC112206744-LOC107973052-GGT2.revcompl                                      ------------------------------------------------------------ 51393
chimp54546-135457.revcompl.Pan.troglodytes.clone.rp43-41g5.GenBank:AC099533.36     CCTGTCCCCCGACATTTCACGGGCCTTCTGAGCTGCTTTCCAAGGAGGACTAACACAGCA 55549
human.FAM30B-LOC105372935-GGT2.NCBI.                                               ------------------------------------------------------------ 50380
human.FAM230E-LOC105377182-GGT3P.NCBI.                                             ------------------------------------------------------------ 49033
human.FAM230J-LOC105372942-GGTLC5P.NCBI.rev.compl                                  ------------------------------------------------------------ 46364
human.FAM230A.ncRNA.18487127.18500594-GGTLC3.18516335.18518165.rev.compl.REF       ------------------------------------------------------------ 31039

chimp.LOC112206744-LOC107973052-GGT2.revcompl                                      ------------------------------------------------------------ 51393
chimp54546-135457.revcompl.Pan.troglodytes.clone.rp43-41g5.GenBank:AC099533.36     ACAAAACATCCATTTCTGCACAAAAATCCTTCTGGAAAGAAAAAGAAGCAAGCCAAGAAT 55609
human.FAM30B-LOC105372935-GGT2.NCBI.                                               ------------------------------------------------------------ 50380
human.FAM230E-LOC105377182-GGT3P.NCBI.                                             ------------------------------------------------------------ 49033
human.FAM230J-LOC105372942-GGTLC5P.NCBI.rev.compl                                  ------------------------------------------------------------ 46364
human.FAM230A.ncRNA.18487127.18500594-GGTLC3.18516335.18518165.rev.compl.REF       ------------------------------------------------------------ 31039

chimp.LOC112206744-LOC107973052-GGT2.revcompl                                      ------------------------------------------------------------ 51393
chimp54546-135457.revcompl.Pan.troglodytes.clone.rp43-41g5.GenBank:AC099533.36     GGAGTGAAAACGCTACCCAGTGCTGACTAAGCCTCTCAAACCCTGTTCTAAGTGGAGTGT 55669
human.FAM30B-LOC105372935-GGT2.NCBI.                                               ------------------------------------------------------------ 50380
human.FAM230E-LOC105377182-GGT3P.NCBI.                                             ------------------------------------------------------------ 49033
human.FAM230J-LOC105372942-GGTLC5P.NCBI.rev.compl                                  ------------------------------------------------------------ 46364
human.FAM230A.ncRNA.18487127.18500594-GGTLC3.18516335.18518165.rev.compl.REF       ------------------------------------------------------------ 31039

chimp.LOC112206744-LOC107973052-GGT2.revcompl                                      ------------------------------------------------------------ 51393
chimp54546-135457.revcompl.Pan.troglodytes.clone.rp43-41g5.GenBank:AC099533.36     GGTTTCTAAGTCAGGGAAATGGAAGAGGCCCCACCCACACAGGGACAGGGCCACGGCCCC 55729
human.FAM30B-LOC105372935-GGT2.NCBI.                                               ------------------------------------------------------------ 50380
human.FAM230E-LOC105377182-GGT3P.NCBI.                                             ------------------------------------------------------------ 49033
human.FAM230J-LOC105372942-GGTLC5P.NCBI.rev.compl                                  ------------------------------------------------------------ 46364
human.FAM230A.ncRNA.18487127.18500594-GGTLC3.18516335.18518165.rev.compl.REF       ------------------------------------------------------------ 31039

chimp.LOC112206744-LOC107973052-GGT2.revcompl                                      ------------------------agccagccccaagaaagggctcccttcctcttttct 51429
chimp54546-135457.revcompl.Pan.troglodytes.clone.rp43-41g5.GenBank:AC099533.36     CACAGGATGAAGCAGCAGCGTTTATTCAAGATACAAGTGAGGGAATCCGGTCACGTTCCC 55789
human.FAM30B-LOC105372935-GGT2.NCBI.                                               ------------------------agccagccccaagaaagggctcccttcctcttttct 50416
human.FAM230E-LOC105377182-GGT3P.NCBI.                                             ------------------------agccagccccaagaaagggctccctgcctcttttct 49069
human.FAM230J-LOC105372942-GGTLC5P.NCBI.rev.compl                                  ------------------------------------------------------------ 46364
human.FAM230A.ncRNA.18487127.18500594-GGTLC3.18516335.18518165.rev.compl.REF       ------------------------------------------------------------ 31039

chimp.LOC112206744-LOC107973052-GGT2.revcompl                                      actgccccagagaggcaagactgagccttaacctccg----------------------- 51466
chimp54546-135457.revcompl.Pan.troglodytes.clone.rp43-41g5.GenBank:AC099533.36     TTCTCCCCAGAGAGGGCACTTCTTGACAAGTGATTCAGTAGAAATCTTTTAGACTCTATA 55849
human.FAM30B-LOC105372935-GGT2.NCBI.                                               actgccccagagaggcaagactgagccttaacctcca----------------------- 50453
human.FAM230E-LOC105377182-GGT3P.NCBI.                                             actgccccagagaggcaagactgagccttaatctccg----------------------- 49106
human.FAM230J-LOC105372942-GGTLC5P.NCBI.rev.compl                                  ------------------------------------------------------------ 46364
human.FAM230A.ncRNA.18487127.18500594-GGTLC3.18516335.18518165.rev.compl.REF       ------------------------------------------------------------ 31039

chimp.LOC112206744-LOC107973052-GGT2.revcompl                                      ------------------------tcctgtcccctctcccagcctcagtttctccatcca 51502
chimp54546-135457.revcompl.Pan.troglodytes.clone.rp43-41g5.GenBank:AC099533.36     AGTTAAGTTCATAAAAACCACTGCTTTCACCCTGTCTCCCAGGGCCAGGCCTGGACTCCA 55909
human.FAM30B-LOC105372935-GGT2.NCBI.                                               ------------------------tcctgtcccctctcccagcctcagtttctccaacca 50489
human.FAM230E-LOC105377182-GGT3P.NCBI.                                             ------------------------tcctgtcccctctcccagcctcagtttctgcatcca 49142
human.FAM230J-LOC105372942-GGTLC5P.NCBI.rev.compl                                  ------------------------------------------------------------ 46364
human.FAM230A.ncRNA.18487127.18500594-GGTLC3.18516335.18518165.rev.compl.REF       ------------------------------------------------------------ 31039

chimp.LOC112206744-LOC107973052-GGT2.revcompl                                      actataagggtttttgtttg----tctgttttgaga-cagggtctcattctgttgtccca 51557
chimp54546-135457.revcompl.Pan.troglodytes.clone.rp43-41g5.GenBank:AC099533.36     AGATGAACTGGCTTGGGGTGCCCTCGGGTGGCCACATAAAAAACTCACAGTCTGAGGCCA 55969
human.FAM30B-LOC105372935-GGT2.NCBI.                                               actataagggtttttgtttgtttgtctgttttgaga-cagggtctcactctgttgtccca 50548
human.FAM230E-LOC105377182-GGT3P.NCBI.                                             actataagggtttttgtttgtttgtctgttttgagaccagggtctcattctgttgtccca 49202
human.FAM230J-LOC105372942-GGTLC5P.NCBI.rev.compl                                  ------------------------------------------------------------ 46364
human.FAM230A.ncRNA.18487127.18500594-GGTLC3.18516335.18518165.rev.compl.REF       ------------------------------------------------------------ 31039

chimp.LOC112206744-LOC107973052-GGT2.revcompl                                      gctggaa----------------------------------------------------- 51564
chimp54546-135457.revcompl.Pan.troglodytes.clone.rp43-41g5.GenBank:AC099533.36     GCCTGGGGCTTTCAGACCTGGGCGGGATTTGCCCAGGCCACCTGTCCTTCTGCTTTGGGC 56029
human.FAM30B-LOC105372935-GGT2.NCBI.                                               gctggag----------------------------------------------------- 50555
human.FAM230E-LOC105377182-GGT3P.NCBI.                                             gctggag----------------------------------------------------- 49209
human.FAM230J-LOC105372942-GGTLC5P.NCBI.rev.compl                                  ------------------------------------------------------------ 46364
human.FAM230A.ncRNA.18487127.18500594-GGTLC3.18516335.18518165.rev.compl.REF       ------------------------------------------------------------ 31039
```

```
chimp.LOC112206744-LOC107973052-GGT2.revcompl    ------------------------------------------------------------ 51564
chimp54546-135457.revcompl.Pan.troglodytes.clone.rp43-41g5.GenBank:AC099533.36    CGCTGTCTCTTGGCAGATGGCCTGACACCTGGGGGTGGCCCAAGGATGCCTCAGAAAATC 56089
human.FAM30B-LOC105372935-GGT2.NCBI.    ------------------------------------------------------------ 50555
human.FAM230E-LOC105377182-GGT3P.NCBI.    ------------------------------------------------------------ 49209
human.FAM230J-LOC105372942-GGTLC5P.NCBI.rev.compl    ------------------------------------------------------------ 46364
human.FAM230A.ncRNA.18487127.18500594-GGTLC3.18516335.18518165.rev.compl.REF    ------------------------------------------------------------ 31039

chimp.LOC112206744-LOC107973052-GGT2.revcompl    ------------------------------------------------------------ 51564
chimp54546-135457.revcompl.Pan.troglodytes.clone.rp43-41g5.GenBank:AC099533.36    TTGATTCCCACTCTACAGATGGCCTGATTAGCCAGAGGTTTCCAGGCCGTCTGTCCGCCT 56149
human.FAM30B-LOC105372935-GGT2.NCBI.    ------------------------------------------------------------ 50555
human.FAM230E-LOC105377182-GGT3P.NCBI.    ------------------------------------------------------------ 49209
human.FAM230J-LOC105372942-GGTLC5P.NCBI.rev.compl    ------------------------------------------------------------ 46364
human.FAM230A.ncRNA.18487127.18500594-GGTLC3.18516335.18518165.rev.compl.REF    ------------------------------------------------------------ 31039

chimp.LOC112206744-LOC107973052-GGT2.revcompl    --------tgcagtggtgcaatcatggctcactgcagccttggcttcccaggctcaagcg 51616
chimp54546-135457.revcompl.Pan.troglodytes.clone.rp43-41g5.GenBank:AC099533.36    CCAGGAGATGGACTGGGACCTTTAGACATCGGTGGAGAACAGGATGCTCTGTCCCTTGCT 56209
human.FAM30B-LOC105372935-GGT2.NCBI.    --------tgcagtggtgcaatcatggctcactgcagccttggcttcccaggctcaagcg 50607
human.FAM230E-LOC105377182-GGT3P.NCBI.    --------tgcagtggtgcaatcatggctcactgcagccttggcttcccaggctcaagcg 49261
human.FAM230J-LOC105372942-GGTLC5P.NCBI.rev.compl    ------------------------------------------------------------ 46364
human.FAM230A.ncRNA.18487127.18500594-GGTLC3.18516335.18518165.rev.compl.REF    ------------------------------------------------------------ 31039

chimp.LOC112206744-LOC107973052-GGT2.revcompl    atcctcccacctcagcctctgaagtacctaagactacagacacaccccac---------- 51666
chimp54546-135457.revcompl.Pan.troglodytes.clone.rp43-41g5.GenBank:AC099533.36    GTCCAGGGCAGGGATGGCCTCCAGCCGCAAGAAGTACAGCAGCACCTCGACCTGCCCTCG 56269
human.FAM30B-LOC105372935-GGT2.NCBI.    atcctcccacttcagcctctgaagtacctaagactacagacataccccac---------- 50657
human.FAM230E-LOC105377182-GGT3P.NCBI.    atcctcccacctcagcctctgaagtacctaagactacagacataccccac---------- 49311
human.FAM230J-LOC105372942-GGTLC5P.NCBI.rev.compl    ------------------------------------------------------------ 46364
human.FAM230A.ncRNA.18487127.18500594-GGTLC3.18516335.18518165.rev.compl.REF    ------------------------------------------------------------ 31039

chimp.LOC112206744-LOC107973052-GGT2.revcompl    ------------------------------------------------------------ 51666
chimp54546-135457.revcompl.Pan.troglodytes.clone.rp43-41g5.GenBank:AC099533.36    CAGAGTGGGGAAGAGGAGAGTGGCTCGGAGGGGGGCTCACAGCTGCTGGTGGGGAGGTCT 56329
human.FAM30B-LOC105372935-GGT2.NCBI.    ------------------------------------------------------------ 50657
human.FAM230E-LOC105377182-GGT3P.NCBI.    ------------------------------------------------------------ 49311
human.FAM230J-LOC105372942-GGTLC5P.NCBI.rev.compl    ------------------------------------------------------------ 46364
human.FAM230A.ncRNA.18487127.18500594-GGTLC3.18516335.18518165.rev.compl.REF    ------------------------------------------------------------ 31039

chimp.LOC112206744-LOC107973052-GGT2.revcompl    ------------------------------------------------------------ 51666
chimp54546-135457.revcompl.Pan.troglodytes.clone.rp43-41g5.GenBank:AC099533.36    TTGGGGCCCAAGCCCCCAAGTCCACCTCAGGTGCTAGAAACCCCTGCTGGTGTCATCAAC 56389
human.FAM30B-LOC105372935-GGT2.NCBI.    ------------------------------------------------------------ 50657
human.FAM230E-LOC105377182-GGT3P.NCBI.    ------------------------------------------------------------ 49311
human.FAM230J-LOC105372942-GGTLC5P.NCBI.rev.compl    ------------------------------------------------------------ 46364
human.FAM230A.ncRNA.18487127.18500594-GGTLC3.18516335.18518165.rev.compl.REF    ------------------------------------------------------------ 31039

chimp.LOC112206744-LOC107973052-GGT2.revcompl    ------------------------------------------------tacacgtggctt 51678
chimp54546-135457.revcompl.Pan.troglodytes.clone.rp43-41g5.GenBank:AC099533.36    CCCTTACAGTGAGACGGGGGTGGGGTGGGGTCCTGACAAGGCATGACTTGTTGGGTGGGG 56449
human.FAM30B-LOC105372935-GGT2.NCBI.    ------------------------------------------------tgcacgtggctt 50669
human.FAM230E-LOC105377182-GGT3P.NCBI.    ------------------------------------------------tgcacgtggc-t 49322
human.FAM230J-LOC105372942-GGTLC5P.NCBI.rev.compl    ------------------------------------------------------------ 46364
human.FAM230A.ncRNA.18487127.18500594-GGTLC3.18516335.18518165.rev.compl.REF    ------------------------------------------------------------ 31039

chimp.LOC112206744-LOC107973052-GGT2.revcompl    tttttttttttttttt--tgagatggagtttcactcttgttgcccaggctggagtgcaat 51736
chimp54546-135457.revcompl.Pan.troglodytes.clone.rp43-41g5.GenBank:AC099533.36    GGTGGTTATTTATTTTAGAGATGCACAGGGCCTTGCTCTGTCCCCAGGCTGGAGTACAGT 56509
human.FAM30B-LOC105372935-GGT2.NCBI.    ttttttttttttttttttgagatggagtttcactcttgttgcccaggctggagtgcaat 50729
human.FAM230E-LOC105377182-GGT3P.NCBI.    tttttttttttttttttctgagatggagtttcactcttgttgcccaggctggagtgcaat 49382
human.FAM230J-LOC105372942-GGTLC5P.NCBI.rev.compl    ------------------------------------------------------------ 46364
human.FAM230A.ncRNA.18487127.18500594-GGTLC3.18516335.18518165.rev.compl.REF    ------------------------------------------------------------ 31039

chimp.LOC112206744-LOC107973052-GGT2.revcompl    ggcacaatcttggctcactccaacctccacctcccgagttcaagcaattctcctgcctca 51796
chimp54546-135457.revcompl.Pan.troglodytes.clone.rp43-41g5.GenBank:AC099533.36    GGCTCCATCATGGATCACTGCAGCCTCTAACTCCTGGGCTCAAGCAATCCTCCTGTGTCA 56569
human.FAM30B-LOC105372935-GGT2.NCBI.    ggcacaatcttggctcactccaacctccacctcccgagttcaagtaattctcctgcctca 50789
human.FAM230E-LOC105377182-GGT3P.NCBI.    ggcacaatcttggctcactccaacctccacctcccgagttcaagtaattctcctgcctca 49442
human.FAM230J-LOC105372942-GGTLC5P.NCBI.rev.compl    ------------------------------------------------------------ 46364
human.FAM230A.ncRNA.18487127.18500594-GGTLC3.18516335.18518165.rev.compl.REF    ------------------------------------------------------------ 31039

chimp.LOC112206744-LOC107973052-GGT2.revcompl    gcctcccaagtagctgggattacaggcatgcaccaccacgccttgctaatt----tttgt 51852
chimp54546-135457.revcompl.Pan.troglodytes.clone.rp43-41g5.GenBank:AC099533.36    GCCTCCCAGATACCTAGGATTACAGATGTGTGCCACAATGCCTGCCTAATTTTTCTTTGT 56629
human.FAM30B-LOC105372935-GGT2.NCBI.    gcctcccaagtacctgggattacaggcatgtaccaccacgccttgctaatt----tttgt 50845
human.FAM230E-LOC105377182-GGT3P.NCBI.    gcctcccaagtagctgggattacaggcatgtaccaccacgccttgctaatt----tttgt 49498
human.FAM230J-LOC105372942-GGTLC5P.NCBI.rev.compl    ------------------------------------------------------------ 46364
human.FAM230A.ncRNA.18487127.18500594-GGTLC3.18516335.18518165.rev.compl.REF    ------------------------------------------------------------ 31039

chimp.LOC112206744-LOC107973052-GGT2.revcompl    atttttagtggagacggggtttcaccaggttggtcaggctggtcttgaactcctaacatc 51912
chimp54546-135457.revcompl.Pan.troglodytes.clone.rp43-41g5.GenBank:AC099533.36    ATTTTTTCTGGAGATGGGGTTTG-CTGCATTGCCCAGACTGATCTCAAACACTTGGGTTC 56688
human.FAM30B-LOC105372935-GGT2.NCBI.    atttttagtagagacggggtttcaccaggttggtcaggctggtcttgaactcctaacatc 50905
human.FAM230E-LOC105377182-GGT3P.NCBI.    atttttagtagagacggggtttcaccaggttggtcaggctggtcttgaactcctaacatc 49558
human.FAM230J-LOC105372942-GGTLC5P.NCBI.rev.compl    ------------------------------------------------------------ 46364
human.FAM230A.ncRNA.18487127.18500594-GGTLC3.18516335.18518165.rev.compl.REF    ------------------------------------------------------------ 31039

chimp.LOC112206744-LOC107973052-GGT2.revcompl    aggtgatccatcctcctcagcctctcagagttctgggattacaggcgtgagccaccactc 51972
chimp54546-135457.revcompl.Pan.troglodytes.clone.rp43-41g5.GenBank:AC099533.36    GGTTGTCCTG----CCTCGGCCTCCCAAAGTGCTGGGATTACAGGCATGAGCCACCACAC 56744
human.FAM30B-LOC105372935-GGT2.NCBI.    aggtgatccatcctcctcagcttctcagagttctgggattacaggcgtgagccaccactc 50965
human.FAM230E-LOC105377182-GGT3P.NCBI.    aggtgatccatcctcctcagcctctcagagttctgggattacaggcgtgagccaccactc 49618
human.FAM230J-LOC105372942-GGTLC5P.NCBI.rev.compl    ------------------------------------------------------------ 46364
human.FAM230A.ncRNA.18487127.18500594-GGTLC3.18516335.18518165.rev.compl.REF    ------------------------------------------------------------ 31039

chimp.LOC112206744-LOC107973052-GGT2.revcompl    ccagcctaattttttattttta-------------------------------------- 51994
chimp54546-135457.revcompl.Pan.troglodytes.clone.rp43-41g5.GenBank:AC099533.36    CCGAACACTTGGGTTGGTTTTAAGCCCCCAGCAAGGTGCACCAGCAGGACCAGGAGGTGG 56804
human.FAM30B-LOC105372935-GGT2.NCBI.    ccagcctaattttttattttta-------------------------------------- 50987
human.FAM230E-LOC105377182-GGT3P.NCBI.    ccagcctaattttttattttta-------------------------------------- 49640
human.FAM230J-LOC105372942-GGTLC5P.NCBI.rev.compl    ------------------------------------------------------------ 46364
human.FAM230A.ncRNA.18487127.18500594-GGTLC3.18516335.18518165.rev.compl.REF    ------------------------------------------------------------ 31039

chimp.LOC112206744-LOC107973052-GGT2.revcompl    ------------------------------------------------------------ 51994
chimp54546-135457.revcompl.Pan.troglodytes.clone.rp43-41g5.GenBank:AC099533.36    CCTGGGCACCCCCTATCACTCCCATCCATGCAAACCTAGGCAAGTCCCTGTCTCTGAATC 56864
human.FAM30B-LOC105372935-GGT2.NCBI.    ------------------------------------------------------------ 50987
human.FAM230E-LOC105377182-GGT3P.NCBI.    ------------------------------------------------------------ 49640
human.FAM230J-LOC105372942-GGTLC5P.NCBI.rev.compl    ------------------------------------------------------------ 46364
human.FAM230A.ncRNA.18487127.18500594-GGTLC3.18516335.18518165.rev.compl.REF    ------------------------------------------------------------ 31039

chimp.LOC112206744-LOC107973052-GGT2.revcompl    ------------------------------------------------------------ 51994
chimp54546-135457.revcompl.Pan.troglodytes.clone.rp43-41g5.GenBank:AC099533.36    TCAGCCACCACCACATACAATGCAAGTCGGAAGATGGGCAGGACTGGGGGTGGGGCAGGC 56924
human.FAM30B-LOC105372935-GGT2.NCBI.    ------------------------------------------------------------ 50987
human.FAM230E-LOC105377182-GGT3P.NCBI.    ------------------------------------------------------------ 49640
human.FAM230J-LOC105372942-GGTLC5P.NCBI.rev.compl    ------------------------------------------------------------ 46364
human.FAM230A.ncRNA.18487127.18500594-GGTLC3.18516335.18518165.rev.compl.REF    ------------------------------------------------------------ 31039

chimp.LOC112206744-LOC107973052-GGT2.revcompl    ------------------------------------------------------------ 51994
chimp54546-135457.revcompl.Pan.troglodytes.clone.rp43-41g5.GenBank:AC099533.36    AGAGGCCACCTCTGTCAGGCTGGGGTTGCATGGGCTGGAGACTGTCTTCCCATACCTGGG 56984
human.FAM30B-LOC105372935-GGT2.NCBI.    ------------------------------------------------------------ 50987
human.FAM230E-LOC105377182-GGT3P.NCBI.    ------------------------------------------------------------ 49640
human.FAM230J-LOC105372942-GGTLC5P.NCBI.rev.compl    ------------------------------------------------------------ 46364
human.FAM230A.ncRNA.18487127.18500594-GGTLC3.18516335.18518165.rev.compl.REF    ------------------------------------------------------------ 31039

chimp.LOC112206744-LOC107973052-GGT2.revcompl    -----------------------------------------ttttttgtagagacagggg 52013
chimp54546-135457.revcompl.Pan.troglodytes.clone.rp43-41g5.GenBank:AC099533.36    ACATGACCTCCAAGGACCTGCTGTCAGTCATGGTGATGGGCTGGTTGGGGTTGGCAGGGA 57044
human.FAM30B-LOC105372935-GGT2.NCBI.    -----------------------------------------ttttttgtagagacagggg 51006
human.FAM230E-LOC105377182-GGT3P.NCBI.    -----------------------------------------ttttttgtagagacagggg 49659
human.FAM230J-LOC105372942-GGTLC5P.NCBI.rev.compl    ------------------------------------------------------------ 46364
human.FAM230A.ncRNA.18487127.18500594-GGTLC3.18516335.18518165.rev.compl.REF    ------------------------------------------------------------ 31039

chimp.LOC112206744-LOC107973052-GGT2.revcompl    tcttgctacgttgccaaga----------------------------------------- 52032
chimp54546-135457.revcompl.Pan.troglodytes.clone.rp43-41g5.GenBank:AC099533.36    GCTTGCTCTCCTTCTCGGAGGGCCAGAGCAGCGTGGGGCCAAAGACAGTGGCGAGGTTGT 57104
human.FAM30B-LOC105372935-GGT2.NCBI.    tcttgctacattgccaaga----------------------------------------- 51025
human.FAM230E-LOC105377182-GGT3P.NCBI.    tcttgctacgttgccaaga----------------------------------------- 49678
human.FAM230J-LOC105372942-GGTLC5P.NCBI.rev.compl    ------------------------------------------------------------ 46364
human.FAM230A.ncRNA.18487127.18500594-GGTLC3.18516335.18518165.rev.compl.REF    ------------------------------------------------------------ 31039
```

```
chimp.LOC112206744-LOC107973052-GGT2.revcompl                                     ------------------------------------------------------------ 52032
chimp54546-135457.revcompl.Pan.troglodytes.clone.rp43-41g5.GenBank:AC099533.36    GCAGGGACATCTTATTGACTGCCTCCTTCTCTGCCACCCTGTAGAGGACCGAAGCAGAGG 57164
human.FAM30B-LOC105372935-GGT2.NCBI.                                              ------------------------------------------------------------ 51025
human.FAM230E-LOC105377182-GGT3P.NCBI.                                            ------------------------------------------------------------ 49678
human.FAM230J-LOC105372942-GGTLC5P.NCBI.rev.compl                                 ------------------------------------------------------------ 46364
human.FAM230A.ncRNA.18487127.18500594-GGTLC3.18516335.18518165.rev.compl.REF      ------------------------------------------------------------ 31039

chimp.LOC112206744-LOC107973052-GGT2.revcompl                                     ------------------------------------------------------------ 52032
chimp54546-135457.revcompl.Pan.troglodytes.clone.rp43-41g5.GenBank:AC099533.36    GTGCTGTTTCAATGCCACCACCAGGAGAGAGGCAGAGGGGCTGTGCCGTGCTAGAGTCCT 57224
human.FAM30B-LOC105372935-GGT2.NCBI.                                              ------------------------------------------------------------ 51025
human.FAM230E-LOC105377182-GGT3P.NCBI.                                            ------------------------------------------------------------ 49678
human.FAM230J-LOC105372942-GGTLC5P.NCBI.rev.compl                                 ------------------------------------------------------------ 46364
human.FAM230A.ncRNA.18487127.18500594-GGTLC3.18516335.18518165.rev.compl.REF      ------------------------------------------------------------ 31039

chimp.LOC112206744-LOC107973052-GGT2.revcompl                                     ------------------------------------------------------------ 52032
chimp54546-135457.revcompl.Pan.troglodytes.clone.rp43-41g5.GenBank:AC099533.36    CAGGGAGGGAGTGACCTCGACCCTGGCTGTGCTGCAAGCTGACTCCAGCCTTGGTACTTC 57284
human.FAM30B-LOC105372935-GGT2.NCBI.                                              ------------------------------------------------------------ 51025
human.FAM230E-LOC105377182-GGT3P.NCBI.                                            ------------------------------------------------------------ 49678
human.FAM230J-LOC105372942-GGTLC5P.NCBI.rev.compl                                 ------------------------------------------------------------ 46364
human.FAM230A.ncRNA.18487127.18500594-GGTLC3.18516335.18518165.rev.compl.REF      ------------------------------------------------------------ 31039

chimp.LOC112206744-LOC107973052-GGT2.revcompl                                     ------------------------------------------------------------ 52032
chimp54546-135457.revcompl.Pan.troglodytes.clone.rp43-41g5.GenBank:AC099533.36    TGGGTCTCAGTGGCCCGGGACAAGGGGCCAGCTCTGGGCTGATGGGGAGGTCTTCATGAT 57344
human.FAM30B-LOC105372935-GGT2.NCBI.                                              ------------------------------------------------------------ 51025
human.FAM230E-LOC105377182-GGT3P.NCBI.                                            ------------------------------------------------------------ 49678
human.FAM230J-LOC105372942-GGTLC5P.NCBI.rev.compl                                 ------------------------------------------------------------ 46364
human.FAM230A.ncRNA.18487127.18500594-GGTLC3.18516335.18518165.rev.compl.REF      ------------------------------------------------------------ 31039

chimp.LOC112206744-LOC107973052-GGT2.revcompl                                     ------------------------------------------------------------ 52032
chimp54546-135457.revcompl.Pan.troglodytes.clone.rp43-41g5.GenBank:AC099533.36    GTGCTTGGGAGGGAAGAGGCGGGTCCAAGTGCACTGCTGGCCACGGCCAAAGCTCTGAGC 57404
human.FAM30B-LOC105372935-GGT2.NCBI.                                              ------------------------------------------------------------ 51025
human.FAM230E-LOC105377182-GGT3P.NCBI.                                            ------------------------------------------------------------ 49678
human.FAM230J-LOC105372942-GGTLC5P.NCBI.rev.compl                                 ------------------------------------------------------------ 46364
human.FAM230A.ncRNA.18487127.18500594-GGTLC3.18516335.18518165.rev.compl.REF      ------------------------------------------------------------ 31039

chimp.LOC112206744-LOC107973052-GGT2.revcompl                                     ------------------------------------------------------------ 52032
chimp54546-135457.revcompl.Pan.troglodytes.clone.rp43-41g5.GenBank:AC099533.36    TCTTTGTTAAGGCCACAGTGCAGAGGGAGGAGGGTGGCAAAGAGGAGAGGCAGTGGTGGG 57464
human.FAM30B-LOC105372935-GGT2.NCBI.                                              ------------------------------------------------------------ 51025
human.FAM230E-LOC105377182-GGT3P.NCBI.                                            ------------------------------------------------------------ 49678
human.FAM230J-LOC105372942-GGTLC5P.NCBI.rev.compl                                 ------------------------------------------------------------ 46364
human.FAM230A.ncRNA.18487127.18500594-GGTLC3.18516335.18518165.rev.compl.REF      ------------------------------------------------------------ 31039

chimp.LOC112206744-LOC107973052-GGT2.revcompl                                     ------------------------------------------------------------ 52032
chimp54546-135457.revcompl.Pan.troglodytes.clone.rp43-41g5.GenBank:AC099533.36    GGTGGCAGTGGTGCTAGTCCTTAGAAGCAGTGAGTTACTGCAGACAGGGGTCGGGGGAAA 57524
human.FAM30B-LOC105372935-GGT2.NCBI.                                              ------------------------------------------------------------ 51025
human.FAM230E-LOC105377182-GGT3P.NCBI.                                            ------------------------------------------------------------ 49678
human.FAM230J-LOC105372942-GGTLC5P.NCBI.rev.compl                                 ------------------------------------------------------------ 46364
human.FAM230A.ncRNA.18487127.18500594-GGTLC3.18516335.18518165.rev.compl.REF      ------------------------------------------------------------ 31039

chimp.LOC112206744-LOC107973052-GGT2.revcompl                                     ------------------------------------------------------------ 52032
chimp54546-135457.revcompl.Pan.troglodytes.clone.rp43-41g5.GenBank:AC099533.36    AGGTCCTTGGTGCTGGGGGTCTGGTGGGAGCAAAGCGGCACCCCAACGGCCTGGAGACCT 57584
human.FAM30B-LOC105372935-GGT2.NCBI.                                              ------------------------------------------------------------ 51025
human.FAM230E-LOC105377182-GGT3P.NCBI.                                            ------------------------------------------------------------ 49678
human.FAM230J-LOC105372942-GGTLC5P.NCBI.rev.compl                                 ------------------------------------------------------------ 46364
human.FAM230A.ncRNA.18487127.18500594-GGTLC3.18516335.18518165.rev.compl.REF      ------------------------------------------------------------ 31039

chimp.LOC112206744-LOC107973052-GGT2.revcompl                                     ------------------------------------------------------------ 52032
chimp54546-135457.revcompl.Pan.troglodytes.clone.rp43-41g5.GenBank:AC099533.36    GGAGTCCTGGGCAGCCACAAGAGAGCTGGGCTACCTTTCAAGGTGGTCTAGAAGGAAAAG 57644
human.FAM30B-LOC105372935-GGT2.NCBI.                                              ------------------------------------------------------------ 51025
human.FAM230E-LOC105377182-GGT3P.NCBI.                                            ------------------------------------------------------------ 49678
human.FAM230J-LOC105372942-GGTLC5P.NCBI.rev.compl                                 ------------------------------------------------------------ 46364
human.FAM230A.ncRNA.18487127.18500594-GGTLC3.18516335.18518165.rev.compl.REF      ------------------------------------------------------------ 31039

chimp.LOC112206744-LOC107973052-GGT2.revcompl                                     ------------------------------------------------------------ 52032
chimp54546-135457.revcompl.Pan.troglodytes.clone.rp43-41g5.GenBank:AC099533.36    GAAGGTGAGCAGGTTGGCCTCCGGCAGGGACAACAACAGGTTGAACATGCAGCTCTTCTT 57704
human.FAM30B-LOC105372935-GGT2.NCBI.                                              ------------------------------------------------------------ 51025
human.FAM230E-LOC105377182-GGT3P.NCBI.                                            ------------------------------------------------------------ 49678
human.FAM230J-LOC105372942-GGTLC5P.NCBI.rev.compl                                 ------------------------------------------------------------ 46364
human.FAM230A.ncRNA.18487127.18500594-GGTLC3.18516335.18518165.rev.compl.REF      ------------------------------------------------------------ 31039

chimp.LOC112206744-LOC107973052-GGT2.revcompl                                     ------------------------------------------------------------ 52032
chimp54546-135457.revcompl.Pan.troglodytes.clone.rp43-41g5.GenBank:AC099533.36    TGCAACTGGGTCTGAAAGAGCTGCAGGAGGCAGTGGGTTACTCCTCTGGGTTACGACAAG 57764
human.FAM30B-LOC105372935-GGT2.NCBI.                                              ------------------------------------------------------------ 51025
human.FAM230E-LOC105377182-GGT3P.NCBI.                                            ------------------------------------------------------------ 49678
human.FAM230J-LOC105372942-GGTLC5P.NCBI.rev.compl                                 ------------------------------------------------------------ 46364
human.FAM230A.ncRNA.18487127.18500594-GGTLC3.18516335.18518165.rev.compl.REF      ------------------------------------------------------------ 31039

chimp.LOC112206744-LOC107973052-GGT2.revcompl                                     ------------------------------------------------------------ 52032
chimp54546-135457.revcompl.Pan.troglodytes.clone.rp43-41g5.GenBank:AC099533.36    CCAGAGACCTCTCCCGAGGTGGTCACATGGAGCACCTAGGACACGAGTCCTTGCGCAGTT 57824
human.FAM30B-LOC105372935-GGT2.NCBI.                                              ------------------------------------------------------------ 51025
human.FAM230E-LOC105377182-GGT3P.NCBI.                                            ------------------------------------------------------------ 49678
human.FAM230J-LOC105372942-GGTLC5P.NCBI.rev.compl                                 ------------------------------------------------------------ 46364
human.FAM230A.ncRNA.18487127.18500594-GGTLC3.18516335.18518165.rev.compl.REF      ------------------------------------------------------------ 31039

chimp.LOC112206744-LOC107973052-GGT2.revcompl                                     ------------------------------------------------------------ 52032
chimp54546-135457.revcompl.Pan.troglodytes.clone.rp43-41g5.GenBank:AC099533.36    TGGGCTTGTCATCATCGTCACACCCACAGCGCTGGCCACCAGTGAGGACCCTGTGAGGGG 57884
human.FAM30B-LOC105372935-GGT2.NCBI.                                              ------------------------------------------------------------ 51025
human.FAM230E-LOC105377182-GGT3P.NCBI.                                            ------------------------------------------------------------ 49678
human.FAM230J-LOC105372942-GGTLC5P.NCBI.rev.compl                                 ------------------------------------------------------------ 46364
human.FAM230A.ncRNA.18487127.18500594-GGTLC3.18516335.18518165.rev.compl.REF      ------------------------------------------------------------ 31039

chimp.LOC112206744-LOC107973052-GGT2.revcompl                                     ------------------------------------------------------------ 52032
chimp54546-135457.revcompl.Pan.troglodytes.clone.rp43-41g5.GenBank:AC099533.36    CACCTGTGTGGGGTGTGAACCACCTGAATGCCTTTTCTCTACCTCACAGGGGTCAGCAGC 57944
human.FAM30B-LOC105372935-GGT2.NCBI.                                              ------------------------------------------------------------ 51025
human.FAM230E-LOC105377182-GGT3P.NCBI.                                            ------------------------------------------------------------ 49678
human.FAM230J-LOC105372942-GGTLC5P.NCBI.rev.compl                                 ------------------------------------------------------------ 46364
human.FAM230A.ncRNA.18487127.18500594-GGTLC3.18516335.18518165.rev.compl.REF      ------------------------------------------------------------ 31039

chimp.LOC112206744-LOC107973052-GGT2.revcompl                                     ----------------------------------------ctggtctcaaactctggcct 52052
chimp54546-135457.revcompl.Pan.troglodytes.clone.rp43-41g5.GenBank:AC099533.36    ACCCGGTGAACAGCAGCAGGAGGAGCCGCTAGAGCAGCTGCTGATGGGCAGAGCTGCCCT 58004
human.FAM30B-LOC105372935-GGT2.NCBI.                                              ----------------------------------------ctggtctcaaactctggcct 51045
human.FAM230E-LOC105377182-GGT3P.NCBI.                                            ----------------------------------------ctggtctcaaactctggcct 49698
human.FAM230J-LOC105372942-GGTLC5P.NCBI.rev.compl                                 ------------------------------------------------------------ 46364
human.FAM230A.ncRNA.18487127.18500594-GGTLC3.18516335.18518165.rev.compl.REF      ------------------------------------------------------------ 31039

chimp.LOC112206744-LOC107973052-GGT2.revcompl                                     caagcaa-tcctcccacctcagcctcccaacatgctgggattacaggtgcactcagccta 52111
chimp54546-135457.revcompl.Pan.troglodytes.clone.rp43-41g5.GenBank:AC099533.36    TGGACAACTCCTGCCACCACCCCCTCCCCAGGGAGCCCAAGGCAGGGGAGGCTCAGCATG 58064
human.FAM30B-LOC105372935-GGT2.NCBI.                                              caagcaa-tcctcccacctcagcctcccaacatgctgggattacaggtgcactcagccta 51104
human.FAM230E-LOC105377182-GGT3P.NCBI.                                            caagcaa-tcctcccacctcagcctcccaacatgctgggattacaggtgcactcagccta 49757
human.FAM230J-LOC105372942-GGTLC5P.NCBI.rev.compl                                 ------------------------------------------------------------ 46364
human.FAM230A.ncRNA.18487127.18500594-GGTLC3.18516335.18518165.rev.compl.REF      ------------------------------------------------------------ 31039

chimp.LOC112206744-LOC107973052-GGT2.revcompl                                     taagg------------------------------------------------------- 52116
chimp54546-135457.revcompl.Pan.troglodytes.clone.rp43-41g5.GenBank:AC099533.36    GAATGAAACAGGGGAGTGAGGGACACAAGGAGGTGGGAAGTGGGAGGGTCCCAGCCCCAC 58124
human.FAM30B-LOC105372935-GGT2.NCBI.                                              taagg------------------------------------------------------- 51109
human.FAM230E-LOC105377182-GGT3P.NCBI.                                            taagg------------------------------------------------------- 49762
human.FAM230J-LOC105372942-GGTLC5P.NCBI.rev.compl                                 ------------------------------------------------------------ 46364
human.FAM230A.ncRNA.18487127.18500594-GGTLC3.18516335.18518165.rev.compl.REF      ------------------------------------------------------------ 31039

chimp.LOC112206744-LOC107973052-GGT2.revcompl                                     ------------------------------------------------------------ 52116
chimp54546-135457.revcompl.Pan.troglodytes.clone.rp43-41g5.GenBank:AC099533.36    CAAGTACGCAGAGACCCCCTCGTCGTCCTGGACACCACAGGGGCACCTGCAGGCTGGGAG 58184
human.FAM30B-LOC105372935-GGT2.NCBI.                                              ------------------------------------------------------------ 51109
human.FAM230E-LOC105377182-GGT3P.NCBI.                                            ------------------------------------------------------------ 49762
human.FAM230J-LOC105372942-GGTLC5P.NCBI.rev.compl                                 ------------------------------------------------------------ 46364
human.FAM230A.ncRNA.18487127.18500594-GGTLC3.18516335.18518165.rev.compl.REF      ------------------------------------------------------------ 31039

chimp.LOC112206744-LOC107973052-GGT2.revcompl                                     ------------------------------------------------------------ 52116
```

```
chimp54546-135457.revcompl.Pan.troglodytes.clone.rp43-41g5.GenBank:AC099533.36      ACCAGGTCCTCTGTGCATGGGCCCGGGAGGCAGACCTGCCCTAAGGGTGATGCAGAGGCC 58244
human.FAM30B-LOC105372935-GGT2.NCBI.                                                 ------------------------------------------------------------ 51109
human.FAM230E-LOC105377182-GGT3P.NCBI.                                               ------------------------------------------------------------ 49762
human.FAM230J-LOC105372942-GGTLC5P.NCBI.rev.compl                                    ------------------------------------------------------------ 46364
human.FAM230A.ncRNA.18487127.18500594-GGTLC3.18516335.18518165.rev.compl.REF         ------------------------------------------------------------ 31039

chimp.LOC112206744-LOC107973052-GGT2.revcompl                                        ------------------------------------------------------------ 52116
chimp54546-135457.revcompl.Pan.troglodytes.clone.rp43-41g5.GenBank:AC099533.36      ACAGGTGCTGCATGCATCAGCACCCACTCTAGACATCAGCCTCCAGGTTGACTAAGGGTC 58304
human.FAM30B-LOC105372935-GGT2.NCBI.                                                 ------------------------------------------------------------ 51109
human.FAM230E-LOC105377182-GGT3P.NCBI.                                               ------------------------------------------------------------ 49762
human.FAM230J-LOC105372942-GGTLC5P.NCBI.rev.compl                                    ------------------------------------------------------------ 46364
human.FAM230A.ncRNA.18487127.18500594-GGTLC3.18516335.18518165.rev.compl.REF         ------------------------------------------------------------ 31039

chimp.LOC112206744-LOC107973052-GGT2.revcompl                                        ------------------------------------------------------------ 52116
chimp54546-135457.revcompl.Pan.troglodytes.clone.rp43-41g5.GenBank:AC099533.36      AGGTCATGTTTGAAACCATGCTTGGCTGGACCAGGACCCATGGCAAGAGCACCTGGGCAC 58364
human.FAM30B-LOC105372935-GGT2.NCBI.                                                 ------------------------------------------------------------ 51109
human.FAM230E-LOC105377182-GGT3P.NCBI.                                               ------------------------------------------------------------ 49762
human.FAM230J-LOC105372942-GGTLC5P.NCBI.rev.compl                                    ------------------------------------------------------------ 46364
human.FAM230A.ncRNA.18487127.18500594-GGTLC3.18516335.18518165.rev.compl.REF         ------------------------------------------------------------ 31039

chimp.LOC112206744-LOC107973052-GGT2.revcompl                                        ------------------------------------------------------------ 52116
chimp54546-135457.revcompl.Pan.troglodytes.clone.rp43-41g5.GenBank:AC099533.36      CAGTGTTTAGCCCTGGTCTGCAGGAAGGAGGACAGCAGACTTTAGGACCCCACAGCACGG 58424
human.FAM30B-LOC105372935-GGT2.NCBI.                                                 ------------------------------------------------------------ 51109
human.FAM230E-LOC105377182-GGT3P.NCBI.                                               ------------------------------------------------------------ 49762
human.FAM230J-LOC105372942-GGTLC5P.NCBI.rev.compl                                    ------------------------------------------------------------ 46364
human.FAM230A.ncRNA.18487127.18500594-GGTLC3.18516335.18518165.rev.compl.REF         ------------------------------------------------------------ 31039

chimp.LOC112206744-LOC107973052-GGT2.revcompl                                        ------------------------------------------------------------ 52116
chimp54546-135457.revcompl.Pan.troglodytes.clone.rp43-41g5.GenBank:AC099533.36      CAGTGCTGACCATTTCACCCACTTGGCCTCCTTGAGGAATAGGGATGGGGAGCCCTCTGG 58484
human.FAM30B-LOC105372935-GGT2.NCBI.                                                 ------------------------------------------------------------ 51109
human.FAM230E-LOC105377182-GGT3P.NCBI.                                               ------------------------------------------------------------ 49762
human.FAM230J-LOC105372942-GGTLC5P.NCBI.rev.compl                                    ------------------------------------------------------------ 46364
human.FAM230A.ncRNA.18487127.18500594-GGTLC3.18516335.18518165.rev.compl.REF         ------------------------------------------------------------ 31039

chimp.LOC112206744-LOC107973052-GGT2.revcompl                                        --------------------------------------------------ggttttgcct 52126
chimp54546-135457.revcompl.Pan.troglodytes.clone.rp43-41g5.GenBank:AC099533.36      GGATGGGCAAGGCCTTCCAGGACAGGCTCGGTTCTGGTCCCCTGCTTTTTGAGGTTGGGT 58544
human.FAM30B-LOC105372935-GGT2.NCBI.                                                 --------------------------------------------------ggttttgcct 51119
human.FAM230E-LOC105377182-GGT3P.NCBI.                                               --------------------------------------------------ggttttgcct 49772
human.FAM230J-LOC105372942-GGTLC5P.NCBI.rev.compl                                    ------------------------------------------------------------ 46364
human.FAM230A.ncRNA.18487127.18500594-GGTLC3.18516335.18518165.rev.compl.REF         ------------------------------------------------------------ 31039

chimp.LOC112206744-LOC107973052-GGT2.revcompl                                        tccagttctgacttttgaggaggtcattggaaacagacccctgggcctgctt-------- 52178
chimp54546-135457.revcompl.Pan.troglodytes.clone.rp43-41g5.GenBank:AC099533.36      TAAAATGCCGACCATGGCAGAGGGGTTACAGCTCAGGTTCCCACACCTCGCTTTTCACAG 58604
human.FAM30B-LOC105372935-GGT2.NCBI.                                                 tccagttctgacttttgaggaggtcattggaaacagacccctgggcctgctt-------- 51171
human.FAM230E-LOC105377182-GGT3P.NCBI.                                               tccagttctgacttttgaggaggtcattggaaacagacccctgggcctgctt-------- 49824
human.FAM230J-LOC105372942-GGTLC5P.NCBI.rev.compl                                    ------------------------------------------------------------ 46364
human.FAM230A.ncRNA.18487127.18500594-GGTLC3.18516335.18518165.rev.compl.REF         ------------------------------------------------------------ 31039

chimp.LOC112206744-LOC107973052-GGT2.revcompl                                        tctccctgagcctcactgcccatatggacactacagacactgaccctttgcccagaaagg 52238
chimp54546-135457.revcompl.Pan.troglodytes.clone.rp43-41g5.GenBank:AC099533.36      CCTCCGAGGGCAGCAGTGCACATGGAGGAGACGTCTCCCACGAGGCCAAGGCCTCCAGTG 58664
human.FAM30B-LOC105372935-GGT2.NCBI.                                                 ccccccctgagccccactgcccatatggacactacagacactgaccctttgcccagaaagg 51231
human.FAM230E-LOC105377182-GGT3P.NCBI.                                               cccccc-gagccccactgcccatatggacactacagacactgaccctttgcccagaaagg 49883
human.FAM230J-LOC105372942-GGTLC5P.NCBI.rev.compl                                    ------------------------------------------------------------ 46364
human.FAM230A.ncRNA.18487127.18500594-GGTLC3.18516335.18518165.rev.compl.REF         ------------------------------------------------------------ 31039

chimp.LOC112206744-LOC107973052-GGT2.revcompl                                        tacaactatggcctctgcc----------------------------------------- 52257
chimp54546-135457.revcompl.Pan.troglodytes.clone.rp43-41g5.GenBank:AC099533.36      CTCACCGATGCCCTCTGCGAAGTTGGGGTAGAACTCGTCAGTGAAGAGGGGCTCGGGCAG 58724
human.FAM30B-LOC105372935-GGT2.NCBI.                                                 tacaactatggcctctgcc----------------------------------------- 51250
human.FAM230E-LOC105377182-GGT3P.NCBI.                                               tacaactatggcctctgcc----------------------------------------- 49902
human.FAM230J-LOC105372942-GGTLC5P.NCBI.rev.compl                                    ------------------------------------------------------------ 46364
human.FAM230A.ncRNA.18487127.18500594-GGTLC3.18516335.18518165.rev.compl.REF         ------------------------------------------------------------ 31039

chimp.LOC112206744-LOC107973052-GGT2.revcompl                                        ------------------------------------------------------------ 52257
chimp54546-135457.revcompl.Pan.troglodytes.clone.rp43-41g5.GenBank:AC099533.36      CTCACGGAAGTACAGCTTCAGCGTGCCTGCGATGGCGTTCACGTCCATCTCACTCATCAT 58784
human.FAM30B-LOC105372935-GGT2.NCBI.                                                 ------------------------------------------------------------ 51250
human.FAM230E-LOC105377182-GGT3P.NCBI.                                               ------------------------------------------------------------ 49902
human.FAM230J-LOC105372942-GGTLC5P.NCBI.rev.compl                                    ------------------------------------------------------------ 46364
human.FAM230A.ncRNA.18487127.18500594-GGTLC3.18516335.18518165.rev.compl.REF         ------------------------------------------------------------ 31039

chimp.LOC112206744-LOC107973052-GGT2.revcompl                                        ------------------------------------------------------------ 52257
chimp54546-135457.revcompl.Pan.troglodytes.clone.rp43-41g5.GenBank:AC099533.36      CACCGACATGTCCTTGTTATCTGGAAAGAGCATGGAAATGCAGCGGCCTCCTTGAAGATC 58844
human.FAM30B-LOC105372935-GGT2.NCBI.                                                 ------------------------------------------------------------ 51250
human.FAM230E-LOC105377182-GGT3P.NCBI.                                               ------------------------------------------------------------ 49902
human.FAM230J-LOC105372942-GGTLC5P.NCBI.rev.compl                                    ------------------------------------------------------------ 46364
human.FAM230A.ncRNA.18487127.18500594-GGTLC3.18516335.18518165.rev.compl.REF         ------------------------------------------------------------ 31039

chimp.LOC112206744-LOC107973052-GGT2.revcompl                                        ------------------------------------------------------------ 52257
chimp54546-135457.revcompl.Pan.troglodytes.clone.rp43-41g5.GenBank:AC099533.36      CTGAGTGAGTCACCCGCCACCCCTGCCTTGGCTAAAGCACCGTCCCTGCCATGCTGACCA 58904
human.FAM30B-LOC105372935-GGT2.NCBI.                                                 ------------------------------------------------------------ 51250
human.FAM230E-LOC105377182-GGT3P.NCBI.                                               ------------------------------------------------------------ 49902
human.FAM230J-LOC105372942-GGTLC5P.NCBI.rev.compl                                    ------------------------------------------------------------ 46364
human.FAM230A.ncRNA.18487127.18500594-GGTLC3.18516335.18518165.rev.compl.REF         ------------------------------------------------------------ 31039

chimp.LOC112206744-LOC107973052-GGT2.revcompl                                        ------------------------------------------------------------ 52257
chimp54546-135457.revcompl.Pan.troglodytes.clone.rp43-41g5.GenBank:AC099533.36      CTGTATGGGTCCCTCCTGGGCTTTGAGCAGCTCATCTGACTCCTCCCAAGAGCTGTGCAT 58964
human.FAM30B-LOC105372935-GGT2.NCBI.                                                 ------------------------------------------------------------ 51250
human.FAM230E-LOC105377182-GGT3P.NCBI.                                               ------------------------------------------------------------ 49902
human.FAM230J-LOC105372942-GGTLC5P.NCBI.rev.compl                                    ------------------------------------------------------------ 46364
human.FAM230A.ncRNA.18487127.18500594-GGTLC3.18516335.18518165.rev.compl.REF         ------------------------------------------------------------ 31039

chimp.LOC112206744-LOC107973052-GGT2.revcompl                                        ------------------------------------------------------------ 52257
chimp54546-135457.revcompl.Pan.troglodytes.clone.rp43-41g5.GenBank:AC099533.36      GGTTCTGTGTCTGCAGAGTTGATAGGGGTGCGTGGGCATTCCCATTCCTCTCCCCTGCTT 59024
human.FAM30B-LOC105372935-GGT2.NCBI.                                                 ------------------------------------------------------------ 51250
human.FAM230E-LOC105377182-GGT3P.NCBI.                                               ------------------------------------------------------------ 49902
human.FAM230J-LOC105372942-GGTLC5P.NCBI.rev.compl                                    ------------------------------------------------------------ 46364
human.FAM230A.ncRNA.18487127.18500594-GGTLC3.18516335.18518165.rev.compl.REF         ------------------------------------------------------------ 31039

chimp.LOC112206744-LOC107973052-GGT2.revcompl                                        ------------------------------------------------------------ 52257
chimp54546-135457.revcompl.Pan.troglodytes.clone.rp43-41g5.GenBank:AC099533.36      GGCCTGATGTGATGGCCAGGAGGAGGCCAGCATGGCAGGACACAGCACCTGCGTGGGGAT 59084
human.FAM30B-LOC105372935-GGT2.NCBI.                                                 ------------------------------------------------------------ 51250
human.FAM230E-LOC105377182-GGT3P.NCBI.                                               ------------------------------------------------------------ 49902
human.FAM230J-LOC105372942-GGTLC5P.NCBI.rev.compl                                    ------------------------------------------------------------ 46364
human.FAM230A.ncRNA.18487127.18500594-GGTLC3.18516335.18518165.rev.compl.REF         ------------------------------------------------------------ 31039

chimp.LOC112206744-LOC107973052-GGT2.revcompl                                        ------------------------------------------------------------ 52257
chimp54546-135457.revcompl.Pan.troglodytes.clone.rp43-41g5.GenBank:AC099533.36      TGGGTGGCTCTGCCCTGTACATAGCAACCACCCCTGCACCAGTGTCGTCTGATAGCAGGA 59144
human.FAM30B-LOC105372935-GGT2.NCBI.                                                 ------------------------------------------------------------ 51250
human.FAM230E-LOC105377182-GGT3P.NCBI.                                               ------------------------------------------------------------ 49902
human.FAM230J-LOC105372942-GGTLC5P.NCBI.rev.compl                                    ------------------------------------------------------------ 46364
human.FAM230A.ncRNA.18487127.18500594-GGTLC3.18516335.18518165.rev.compl.REF         ------------------------------------------------------------ 31039

chimp.LOC112206744-LOC107973052-GGT2.revcompl                                        ------------------------------------------------------------ 52257
chimp54546-135457.revcompl.Pan.troglodytes.clone.rp43-41g5.GenBank:AC099533.36      AGGCCGTGGGAGAATCTGATTGGTTTCAGTGTTTGAACTGGTGTCTTCATTTGGACCCAA 59204
human.FAM30B-LOC105372935-GGT2.NCBI.                                                 ------------------------------------------------------------ 51250
human.FAM230E-LOC105377182-GGT3P.NCBI.                                               ------------------------------------------------------------ 49902
human.FAM230J-LOC105372942-GGTLC5P.NCBI.rev.compl                                    ------------------------------------------------------------ 46364
human.FAM230A.ncRNA.18487127.18500594-GGTLC3.18516335.18518165.rev.compl.REF         ------------------------------------------------------------ 31039

chimp.LOC112206744-LOC107973052-GGT2.revcompl                                        ------------------------------------------------------------ 52257
chimp54546-135457.revcompl.Pan.troglodytes.clone.rp43-41g5.GenBank:AC099533.36      TTGGCCACTGGTGCTTACATCCTCACCACAGGCCAGGTTCATTCTGGGCCCCCAGAGGGA 59264
human.FAM30B-LOC105372935-GGT2.NCBI.                                                 ------------------------------------------------------------ 51250
human.FAM230E-LOC105377182-GGT3P.NCBI.                                               ------------------------------------------------------------ 49902
human.FAM230J-LOC105372942-GGTLC5P.NCBI.rev.compl                                    ------------------------------------------------------------ 46364
human.FAM230A.ncRNA.18487127.18500594-GGTLC3.18516335.18518165.rev.compl.REF         ------------------------------------------------------------ 31039

chimp.LOC112206744-LOC107973052-GGT2.revcompl                                        ----------cccagggactctcctgctcttgcgagagatgatggggccatttgccttgg 52307
chimp54546-135457.revcompl.Pan.troglodytes.clone.rp43-41g5.GenBank:AC099533.36      GCTGAAACTACCACAGGGCCCTCCCAGGGATGCTGGGCATTCTAGGGGTCCTGGTCAGGG 59324
```

```
human.FAM30B-LOC105372935-GGT2.NCBI.                                            ----------cccagggactctcctgctcttgcgagagatgatggggccatttgccttgg 51300
human.FAM230E-LOC105377182-GGT3P.NCBI.                                          ----------cccagggactctcctgctcttgcgagagatgatggggccatttgccttgg 49952
human.FAM230J-LOC105372942-GGTLC5P.NCBI.rev.compl                               ------------------------------------------------------------ 46364
human.FAM230A.ncRNA.18487127.18500594-GGTLC3.18516335.18518165.rev.compl.REF    ------------------------------------------------------------ 31039

chimp.LOC112206744-LOC107973052-GGT2.revcompl                                   cttggcggctgtggctctagaactgcctctcccaccctgaagcctggcac---------- 52357
chimp54546-135457.revcompl.Pan.troglodytes.clone.rp43-41g5.GenBank:AC099533.36  TGGGTGGTGTGTGCTGCAAAGAAGGGTCTGCAGGCACAAAATCCTGTTGCTTTGAAGATG 59384
human.FAM30B-LOC105372935-GGT2.NCBI.                                            cttggcggctgtggctctagaactgcctctcccaccctgaagcctggcac---------- 51350
human.FAM230E-LOC105377182-GGT3P.NCBI.                                          cttggcggctgtggctctagaactgcctctcccaccctgaagcctggcac---------- 50002
human.FAM230J-LOC105372942-GGTLC5P.NCBI.rev.compl                               ------------------------------------------------------------ 46364
human.FAM230A.ncRNA.18487127.18500594-GGTLC3.18516335.18518165.rev.compl.REF    ------------------------------------------------------------ 31039

chimp.LOC112206744-LOC107973052-GGT2.revcompl                                   ------------------------------------------------------------ 52357
chimp54546-135457.revcompl.Pan.troglodytes.clone.rp43-41g5.GenBank:AC099533.36  CTGGGAAGGACCCTCTGGGGTCTCAGTGCCCTCCCCTGGCATTTGAGGCAGGTCTGGGTC 59444
human.FAM30B-LOC105372935-GGT2.NCBI.                                            ------------------------------------------------------------ 51350
human.FAM230E-LOC105377182-GGT3P.NCBI.                                          ------------------------------------------------------------ 50002
human.FAM230J-LOC105372942-GGTLC5P.NCBI.rev.compl                               ------------------------------------------------------------ 46364
human.FAM230A.ncRNA.18487127.18500594-GGTLC3.18516335.18518165.rev.compl.REF    ------------------------------------------------------------ 31039

chimp.LOC112206744-LOC107973052-GGT2.revcompl                                   -----aagtttccaagagctggtggtttcaattcctagaagctgcacgtacatcccggaa 52412
chimp54546-135457.revcompl.Pan.troglodytes.clone.rp43-41g5.GenBank:AC099533.36  CTTCAAAGCCTGTGAGGGTTGGTGAGATGGAGGCGGAGATGCTGCAG----CCCCGGCCT 59500
human.FAM30B-LOC105372935-GGT2.NCBI.                                            -----aagtttccaagagctggtggtttcaattcctagaagctgcacatatatcccggaa 51405
human.FAM230E-LOC105377182-GGT3P.NCBI.                                          -----aagtttccaagagctggtggtttcaattcctagaagctgcacatatatcccggaa 50057
human.FAM230J-LOC105372942-GGTLC5P.NCBI.rev.compl                               ------------------------------------------------------------ 46364
human.FAM230A.ncRNA.18487127.18500594-GGTLC3.18516335.18518165.rev.compl.REF    ------------------------------------------------------------ 31039

chimp.LOC112206744-LOC107973052-GGT2.revcompl                                   ggtctgacacccagcacatgattccttccaccttgtagttagacag-------------- 52458
chimp54546-135457.revcompl.Pan.troglodytes.clone.rp43-41g5.GenBank:AC099533.36  GCGCTGAATTTCATCAGTGCCCTCTGCCCACCACATCCTCATACAGGGCAGTGGACAGAC 59560
human.FAM30B-LOC105372935-GGT2.NCBI.                                            ggtctgacacccagcatatgattccttccaccttgtagttagacag-------------- 51451
human.FAM230E-LOC105377182-GGT3P.NCBI.                                          ggtctgacacccagcatatgattccttccaccttgtagttagacag-------------- 50103
human.FAM230J-LOC105372942-GGTLC5P.NCBI.rev.compl                               ------------------------------------------------------------ 46364
human.FAM230A.ncRNA.18487127.18500594-GGTLC3.18516335.18518165.rev.compl.REF    ------------------------------------------------------------ 31039

chimp.LOC112206744-LOC107973052-GGT2.revcompl                                   ------------------------------------------------------------ 52458
chimp54546-135457.revcompl.Pan.troglodytes.clone.rp43-41g5.GenBank:AC099533.36  CGCACTGAGTCCTGGGCTTCCACCTCCTGTCCACCCCCAAGGCAGGAAGGTCAAGTCCCC 59620
human.FAM30B-LOC105372935-GGT2.NCBI.                                            ------------------------------------------------------------ 51451
human.FAM230E-LOC105377182-GGT3P.NCBI.                                          ------------------------------------------------------------ 50103
human.FAM230J-LOC105372942-GGTLC5P.NCBI.rev.compl                               ------------------------------------------------------------ 46364
human.FAM230A.ncRNA.18487127.18500594-GGTLC3.18516335.18518165.rev.compl.REF    ------------------------------------------------------------ 31039

chimp.LOC112206744-LOC107973052-GGT2.revcompl                                   ------------------------------------------------------------ 52458
chimp54546-135457.revcompl.Pan.troglodytes.clone.rp43-41g5.GenBank:AC099533.36  ACAGAAGCCCCTGGTCCACTCCACCAAGTGGCATGAGTGGGTACGATGGTGTAAAAACTG 59680
human.FAM30B-LOC105372935-GGT2.NCBI.                                            ------------------------------------------------------------ 51451
human.FAM230E-LOC105377182-GGT3P.NCBI.                                          ------------------------------------------------------------ 50103
human.FAM230J-LOC105372942-GGTLC5P.NCBI.rev.compl                               ------------------------------------------------------------ 46364
human.FAM230A.ncRNA.18487127.18500594-GGTLC3.18516335.18518165.rev.compl.REF    ------------------------------------------------------------ 31039

chimp.LOC112206744-LOC107973052-GGT2.revcompl                                   ------------------------------------------------------------ 52458
chimp54546-135457.revcompl.Pan.troglodytes.clone.rp43-41g5.GenBank:AC099533.36  GCTTCTATAGAAGCTGTTTGTACAACTCTTGTTTTCTCTTTTTTAAAAATAATAAAACAG 59740
human.FAM30B-LOC105372935-GGT2.NCBI.                                            ------------------------------------------------------------ 51451
human.FAM230E-LOC105377182-GGT3P.NCBI.                                          ------------------------------------------------------------ 50103
human.FAM230J-LOC105372942-GGTLC5P.NCBI.rev.compl                               ------------------------------------------------------------ 46364
human.FAM230A.ncRNA.18487127.18500594-GGTLC3.18516335.18518165.rev.compl.REF    ------------------------------------------------------------ 31039

chimp.LOC112206744-LOC107973052-GGT2.revcompl                                   ------------------------------------------------------------ 52458
chimp54546-135457.revcompl.Pan.troglodytes.clone.rp43-41g5.GenBank:AC099533.36  TAAATGAAGAAAAGACACAGAGAAGGATGTGACATGCCTGGGCCATGGAGCACTCTGAGA 59800
human.FAM30B-LOC105372935-GGT2.NCBI.                                            ------------------------------------------------------------ 51451
human.FAM230E-LOC105377182-GGT3P.NCBI.                                          ------------------------------------------------------------ 50103
human.FAM230J-LOC105372942-GGTLC5P.NCBI.rev.compl                               ------------------------------------------------------------ 46364
human.FAM230A.ncRNA.18487127.18500594-GGTLC3.18516335.18518165.rev.compl.REF    ------------------------------------------------------------ 31039

chimp.LOC112206744-LOC107973052-GGT2.revcompl                                   ------------------------------------------------------------ 52458
chimp54546-135457.revcompl.Pan.troglodytes.clone.rp43-41g5.GenBank:AC099533.36  TCTCATCGCGGACACCACTGCCCACACCTCCATCCCGTCCTGCGCAGGCCGACACTCACT 59860
human.FAM30B-LOC105372935-GGT2.NCBI.                                            ------------------------------------------------------------ 51451
human.FAM230E-LOC105377182-GGT3P.NCBI.                                          ------------------------------------------------------------ 50103
human.FAM230J-LOC105372942-GGTLC5P.NCBI.rev.compl                               ------------------------------------------------------------ 46364
human.FAM230A.ncRNA.18487127.18500594-GGTLC3.18516335.18518165.rev.compl.REF    ------------------------------------------------------------ 31039

chimp.LOC112206744-LOC107973052-GGT2.revcompl                                   ------------------------------------------------------------ 52458
chimp54546-135457.revcompl.Pan.troglodytes.clone.rp43-41g5.GenBank:AC099533.36  GACGTGGAAGCCTGCCTTCAGTGCCTGGATGTCTGCAGCCACACCGGACACGCGGTAGAT 59920
human.FAM30B-LOC105372935-GGT2.NCBI.                                            ------------------------------------------------------------ 51451
human.FAM230E-LOC105377182-GGT3P.NCBI.                                          ------------------------------------------------------------ 50103
human.FAM230J-LOC105372942-GGTLC5P.NCBI.rev.compl                               ------------------------------------------------------------ 46364
human.FAM230A.ncRNA.18487127.18500594-GGTLC3.18516335.18518165.rev.compl.REF    ------------------------------------------------------------ 31039

chimp.LOC112206744-LOC107973052-GGT2.revcompl                                   ------------------------------------------------------------ 52458
chimp54546-135457.revcompl.Pan.troglodytes.clone.rp43-41g5.GenBank:AC099533.36  GCCCACCTCCTCCATGCCTCGGTGCTCGATCTCCTCCACGCACTGGCGCACGATGTAGGG 59980
human.FAM30B-LOC105372935-GGT2.NCBI.                                            ------------------------------------------------------------ 51451
human.FAM230E-LOC105377182-GGT3P.NCBI.                                          ------------------------------------------------------------ 50103
human.FAM230J-LOC105372942-GGTLC5P.NCBI.rev.compl                               ------------------------------------------------------------ 46364
human.FAM230A.ncRNA.18487127.18500594-GGTLC3.18516335.18518165.rev.compl.REF    ------------------------------------------------------------ 31039

chimp.LOC112206744-LOC107973052-GGT2.revcompl                                   ------------------------------------------------------------ 52458
chimp54546-135457.revcompl.Pan.troglodytes.clone.rp43-41g5.GenBank:AC099533.36  CACCTTGGACCTCTCTCTCCTGCGGGAGGAGGGAATGCTCTCAGTGTCCTAAGAGCCCTG 60040
human.FAM30B-LOC105372935-GGT2.NCBI.                                            ------------------------------------------------------------ 51451
human.FAM230E-LOC105377182-GGT3P.NCBI.                                          ------------------------------------------------------------ 50103
human.FAM230J-LOC105372942-GGTLC5P.NCBI.rev.compl                               ------------------------------------------------------------ 46364
human.FAM230A.ncRNA.18487127.18500594-GGTLC3.18516335.18518165.rev.compl.REF    ------------------------------------------------------------ 31039

chimp.LOC112206744-LOC107973052-GGT2.revcompl                                   ------------------------------------------------------------ 52458
chimp54546-135457.revcompl.Pan.troglodytes.clone.rp43-41g5.GenBank:AC099533.36  CTTGGGCCATAACACAGGAGACCTGCTCCCTATCTGCACACCCGGAGGTGGGGTGAGGAC 60100
human.FAM30B-LOC105372935-GGT2.NCBI.                                            ------------------------------------------------------------ 51451
human.FAM230E-LOC105377182-GGT3P.NCBI.                                          ------------------------------------------------------------ 50103
human.FAM230J-LOC105372942-GGTLC5P.NCBI.rev.compl                               ------------------------------------------------------------ 46364
human.FAM230A.ncRNA.18487127.18500594-GGTLC3.18516335.18518165.rev.compl.REF    ------------------------------------------------------------ 31039

chimp.LOC112206744-LOC107973052-GGT2.revcompl                                   ------------------------------------------------------------ 52458
chimp54546-135457.revcompl.Pan.troglodytes.clone.rp43-41g5.GenBank:AC099533.36  GGTGACGAAGGTACCCAGGTCTGGGGCTGCACACAGAGCCTGCTGCATGCCTGTCCTCCC 60160
human.FAM30B-LOC105372935-GGT2.NCBI.                                            ------------------------------------------------------------ 51451
human.FAM230E-LOC105377182-GGT3P.NCBI.                                          ------------------------------------------------------------ 50103
human.FAM230J-LOC105372942-GGTLC5P.NCBI.rev.compl                               ------------------------------------------------------------ 46364
human.FAM230A.ncRNA.18487127.18500594-GGTLC3.18516335.18518165.rev.compl.REF    ------------------------------------------------------------ 31039

chimp.LOC112206744-LOC107973052-GGT2.revcompl                                   ------------------------------------------------------------ 52458
chimp54546-135457.revcompl.Pan.troglodytes.clone.rp43-41g5.GenBank:AC099533.36  TCTGCAAGCTCTGTCCTCATTGCATGTACTTTCTCAGGAACCTTTCAAGCGGCCAGAACC 60220
human.FAM30B-LOC105372935-GGT2.NCBI.                                            ------------------------------------------------------------ 51451
human.FAM230E-LOC105377182-GGT3P.NCBI.                                          ------------------------------------------------------------ 50103
human.FAM230J-LOC105372942-GGTLC5P.NCBI.rev.compl                               ------------------------------------------------------------ 46364
human.FAM230A.ncRNA.18487127.18500594-GGTLC3.18516335.18518165.rev.compl.REF    ------------------------------------------------------------ 31039

chimp.LOC112206744-LOC107973052-GGT2.revcompl                                   ------------------------------------------------------------ 52458
chimp54546-135457.revcompl.Pan.troglodytes.clone.rp43-41g5.GenBank:AC099533.36  CCTGCGAATCACACATGATCTTTGCGGGAAGGTCAGGAGGCCTTTCTAAGTCAAGTCAGC 60280
human.FAM30B-LOC105372935-GGT2.NCBI.                                            ------------------------------------------------------------ 51451
human.FAM230E-LOC105377182-GGT3P.NCBI.                                          ------------------------------------------------------------ 50103
human.FAM230J-LOC105372942-GGTLC5P.NCBI.rev.compl                               ------------------------------------------------------------ 46364
human.FAM230A.ncRNA.18487127.18500594-GGTLC3.18516335.18518165.rev.compl.REF    ------------------------------------------------------------ 31039

chimp.LOC112206744-LOC107973052-GGT2.revcompl                                   ------------------------------------------------------------ 52458
chimp54546-135457.revcompl.Pan.troglodytes.clone.rp43-41g5.GenBank:AC099533.36  ACAGGAAGGGCATCTCACAGATTCCAGGCCTGGGGTTAGCAGCCTGTGCCCCCGGCTGGG 60340
human.FAM30B-LOC105372935-GGT2.NCBI.                                            ------------------------------------------------------------ 51451
human.FAM230E-LOC105377182-GGT3P.NCBI.                                          ------------------------------------------------------------ 50103
human.FAM230J-LOC105372942-GGTLC5P.NCBI.rev.compl                               ------------------------------------------------------------ 46364
human.FAM230A.ncRNA.18487127.18500594-GGTLC3.18516335.18518165.rev.compl.REF    ------------------------------------------------------------ 31039

chimp.LOC112206744-LOC107973052-GGT2.revcompl                                   ------------------------------------------------------------ 52458
chimp54546-135457.revcompl.Pan.troglodytes.clone.rp43-41g5.GenBank:AC099533.36  AGATCAGACCCAGTGTTGGTCCTGCCACCCACGTGCTGTGTGAGAGGAGAATCCCTGACC 60400
human.FAM30B-LOC105372935-GGT2.NCBI.                                            ------------------------------------------------------------ 51451
```

```
human.FAM230E-LOC105377182-GGT3P.NCBI.                                       ------------------------------------------------------------ 50103
human.FAM230J-LOC105372942-GGTLC5P.NCBI.rev.compl                            ------------------------------------------------------------ 46364
human.FAM230A.ncRNA.18487127.18500594-GGTLC3.18516335.18518165.rev.compl.REF ------------------------------------------------------------ 31039

chimp.LOC112206744-LOC107973052-GGT2.revcompl                                ------------------------------------------------------------ 52458
chimp54546-135457.revcompl.Pan.troglodytes.clone.rp43-41g5.GenBank:AC099533.36 CCTGCGCTGGGCCTTAACACACATCCGACGAATGAATGAAGGGTTGCCTCAGCACCGGTG 60460
human.FAM30B-LOC105372935-GGT2.NCBI.                                         ------------------------------------------------------------ 51451
human.FAM230E-LOC105377182-GGT3P.NCBI.                                       ------------------------------------------------------------ 50103
human.FAM230J-LOC105372942-GGTLC5P.NCBI.rev.compl                            ------------------------------------------------------------ 46364
human.FAM230A.ncRNA.18487127.18500594-GGTLC3.18516335.18518165.rev.compl.REF ------------------------------------------------------------ 31039

chimp.LOC112206744-LOC107973052-GGT2.revcompl                                ----aagttcttttttgttttgttttgtt-ttctttgtttgtttttgagatggagtcttg 52513
chimp54546-135457.revcompl.Pan.troglodytes.clone.rp43-41g5.GenBank:AC099533.36 CTCCAAGTCCTGCGATGCTAAGTGCTTTTCTCCTCTG--AGTCTTAGCAATGGAAAATTC 60518
human.FAM30B-LOC105372935-GGT2.NCBI.                                         ----aagttcttttttgttttgttttgtttttctttgtttgtttttgagatggagtcttg 51507
human.FAM230E-LOC105377182-GGT3P.NCBI.                                       ----aagttcttttttgttttgttttttttttctttgtttgtttttgagatggagtcttg 50159
human.FAM230J-LOC105372942-GGTLC5P.NCBI.rev.compl                            ------------------------------------------------------------ 46364
human.FAM230A.ncRNA.18487127.18500594-GGTLC3.18516335.18518165.rev.compl.REF ------------------------------------------------------------ 31039

chimp.LOC112206744-LOC107973052-GGT2.revcompl                                ctctgtctcc--------------------------------------cagactgcagtg 52535
chimp54546-135457.revcompl.Pan.troglodytes.clone.rp43-41g5.GenBank:AC099533.36 CAATACCTCCACACAGGACACTAGAGTAAGAATCCTTCACAGTTAGAACGCAGTGCTGTG 60578
human.FAM30B-LOC105372935-GGT2.NCBI.                                         ctctgtctcc--------------------------------------cagactgcagtg 51529
human.FAM230E-LOC105377182-GGT3P.NCBI.                                       ctctgtctcc--------------------------------------cagactgcagtg 50181
human.FAM230J-LOC105372942-GGTLC5P.NCBI.rev.compl                            ------------------------------------------------------------ 46364
human.FAM230A.ncRNA.18487127.18500594-GGTLC3.18516335.18518165.rev.compl.REF ------------------------------------------------------------ 31039

chimp.LOC112206744-LOC107973052-GGT2.revcompl                                cagtggcatgatc-tcagctcactataacctccgcctcccaggttcaagcgattctcctg 52594
chimp54546-135457.revcompl.Pan.troglodytes.clone.rp43-41g5.GenBank:AC099533.36 CGGAGGCCTTAACTTGAGTTCTGTTTTGCACCTGGATTTACCAGCACATCAAAGCTGCTT 60638
human.FAM30B-LOC105372935-GGT2.NCBI.                                         cagtggcatgatc-tcagctcactataacctccgcctcccaggttcaagcgattctcctg 51588
human.FAM230E-LOC105377182-GGT3P.NCBI.                                       cagtggcatgatc-tcagctcactataacctccgcctcccaggttcaagcgattctcctg 50240
human.FAM230J-LOC105372942-GGTLC5P.NCBI.rev.compl                            ------------------------------------------------------------ 46364
human.FAM230A.ncRNA.18487127.18500594-GGTLC3.18516335.18518165.rev.compl.REF ------------------------------------------------------------ 31039

chimp.LOC112206744-LOC107973052-GGT2.revcompl                                cctcagcctcccgagtagctgggatta--------------------------------- 52621
chimp54546-135457.revcompl.Pan.troglodytes.clone.rp43-41g5.GenBank:AC099533.36 CGCAAGCCCCCTCATCAGCAGGGCTTATCTGGGGGAGCTGCTGATGGAGTCCTCGCTGCT 60698
human.FAM30B-LOC105372935-GGT2.NCBI.                                         cctcagcctcccgagtagctgggatta--------------------------------- 51615
human.FAM230E-LOC105377182-GGT3P.NCBI.                                       cctcagcctcccgagtagctgggatta--------------------------------- 50267
human.FAM230J-LOC105372942-GGTLC5P.NCBI.rev.compl                            ------------------------------------------------------------ 46364
human.FAM230A.ncRNA.18487127.18500594-GGTLC3.18516335.18518165.rev.compl.REF ------------------------------------------------------------ 31039

chimp.LOC112206744-LOC107973052-GGT2.revcompl                                ------------------------------------------------------------ 52621
chimp54546-135457.revcompl.Pan.troglodytes.clone.rp43-41g5.GenBank:AC099533.36 CATGCCCACAGCCCTCCCAGAGTGCTATGCGAGTGGCTGCCATGCAGTTGGGGGTGGGGT 60758
human.FAM30B-LOC105372935-GGT2.NCBI.                                         ------------------------------------------------------------ 51615
human.FAM230E-LOC105377182-GGT3P.NCBI.                                       ------------------------------------------------------------ 50267
human.FAM230J-LOC105372942-GGTLC5P.NCBI.rev.compl                            ------------------------------------------------------------ 46364
human.FAM230A.ncRNA.18487127.18500594-GGTLC3.18516335.18518165.rev.compl.REF ------------------------------------------------------------ 31039

chimp.LOC112206744-LOC107973052-GGT2.revcompl                                ------------------------------------------caggcacaggccagcacg 52639
chimp54546-135457.revcompl.Pan.troglodytes.clone.rp43-41g5.GenBank:AC099533.36 GTGGTGTTTAGACACAGATGGGAGTCCAGGGTATGACTGATGGAGGCCCCGGCCCACGTG 60818
human.FAM30B-LOC105372935-GGT2.NCBI.                                         ------------------------------------------caggcacaggccagcacg 51633
human.FAM230E-LOC105377182-GGT3P.NCBI.                                       ------------------------------------------caggcacaggccagcacg 50285
human.FAM230J-LOC105372942-GGTLC5P.NCBI.rev.compl                            ------------------------------------------------------------ 46364
human.FAM230A.ncRNA.18487127.18500594-GGTLC3.18516335.18518165.rev.compl.REF ------------------------------------------------------------ 31039

chimp.LOC112206744-LOC107973052-GGT2.revcompl                                ccaggctaa--------------------------------------------------- 52648
chimp54546-135457.revcompl.Pan.troglodytes.clone.rp43-41g5.GenBank:AC099533.36 ACCAGCAAGGTCAGAGGCCCAGCCAGATTCCATCCTAGGGAAGCAAATGAATTCTCAGAG 60878
human.FAM30B-LOC105372935-GGT2.NCBI.                                         ccaggctaa--------------------------------------------------- 51642
human.FAM230E-LOC105377182-GGT3P.NCBI.                                       ccaggctaa--------------------------------------------------- 50294
human.FAM230J-LOC105372942-GGTLC5P.NCBI.rev.compl                            ------------------------------------------------------------ 46364
human.FAM230A.ncRNA.18487127.18500594-GGTLC3.18516335.18518165.rev.compl.REF ------------------------------------------------------------ 31039

chimp.LOC112206744-LOC107973052-GGT2.revcompl                                ------------------------------------------------------------ 52648
chimp54546-135457.revcompl.Pan.troglodytes.clone.rp43-41g5.GenBank:AC099533.36 GAAGTGGTCTGTGTCTGCATGAACTGCTCTCAAACCAACAAACAGGCTTCTCTTGGCAAC 60938
human.FAM30B-LOC105372935-GGT2.NCBI.                                         ------------------------------------------------------------ 51642
human.FAM230E-LOC105377182-GGT3P.NCBI.                                       ------------------------------------------------------------ 50294
human.FAM230J-LOC105372942-GGTLC5P.NCBI.rev.compl                            ------------------------------------------------------------ 46364
human.FAM230A.ncRNA.18487127.18500594-GGTLC3.18516335.18518165.rev.compl.REF ------------------------------------------------------------ 31039

chimp.LOC112206744-LOC107973052-GGT2.revcompl                                ------------------------------------------------------------ 52648
chimp54546-135457.revcompl.Pan.troglodytes.clone.rp43-41g5.GenBank:AC099533.36 TGACTCATGACAAAGGGGCAGGGGTTACAAACATTCAACAGGAATCTGGGACCGTGACTA 60998
human.FAM30B-LOC105372935-GGT2.NCBI.                                         ------------------------------------------------------------ 51642
human.FAM230E-LOC105377182-GGT3P.NCBI.                                       ------------------------------------------------------------ 50294
human.FAM230J-LOC105372942-GGTLC5P.NCBI.rev.compl                            ------------------------------------------------------------ 46364
human.FAM230A.ncRNA.18487127.18500594-GGTLC3.18516335.18518165.rev.compl.REF ------------------------------------------------------------ 31039

chimp.LOC112206744-LOC107973052-GGT2.revcompl                                ------------------------------------------------------------ 52648
chimp54546-135457.revcompl.Pan.troglodytes.clone.rp43-41g5.GenBank:AC099533.36 ATTCCAAGCAAGCGCAGAAAACCCTGCACTTGGGCAGTTCCTGGAGCCAGCCTACTGCTT 61058
human.FAM30B-LOC105372935-GGT2.NCBI.                                         ------------------------------------------------------------ 51642
human.FAM230E-LOC105377182-GGT3P.NCBI.                                       ------------------------------------------------------------ 50294
human.FAM230J-LOC105372942-GGTLC5P.NCBI.rev.compl                            ------------------------------------------------------------ 46364
human.FAM230A.ncRNA.18487127.18500594-GGTLC3.18516335.18518165.rev.compl.REF ------------------------------------------------------------ 31039

chimp.LOC112206744-LOC107973052-GGT2.revcompl                                ------------------------------------------------------------ 52648
chimp54546-135457.revcompl.Pan.troglodytes.clone.rp43-41g5.GenBank:AC099533.36 CCAAGGAAGGTGGCAAGGACATTCCTGAGCTGGTTTGCAGGAAACACATGAGGTAGCTGA 61118
human.FAM30B-LOC105372935-GGT2.NCBI.                                         ------------------------------------------------------------ 51642
human.FAM230E-LOC105377182-GGT3P.NCBI.                                       ------------------------------------------------------------ 50294
human.FAM230J-LOC105372942-GGTLC5P.NCBI.rev.compl                            ------------------------------------------------------------ 46364
human.FAM230A.ncRNA.18487127.18500594-GGTLC3.18516335.18518165.rev.compl.REF ------------------------------------------------------------ 31039

chimp.LOC112206744-LOC107973052-GGT2.revcompl                                ------------------------------------------------------------ 52648
chimp54546-135457.revcompl.Pan.troglodytes.clone.rp43-41g5.GenBank:AC099533.36 ACACGCCGTGTTCCCTGGGGCCTGACCTCACCGTGCAGTGGCCACACACAGAGAGGAGAG 61178
human.FAM30B-LOC105372935-GGT2.NCBI.                                         ------------------------------------------------------------ 51642
human.FAM230E-LOC105377182-GGT3P.NCBI.                                       ------------------------------------------------------------ 50294
human.FAM230J-LOC105372942-GGTLC5P.NCBI.rev.compl                            ------------------------------------------------------------ 46364
human.FAM230A.ncRNA.18487127.18500594-GGTLC3.18516335.18518165.rev.compl.REF ------------------------------------------------------------ 31039

chimp.LOC112206744-LOC107973052-GGT2.revcompl                                ------------------------------------------------------------ 52648
chimp54546-135457.revcompl.Pan.troglodytes.clone.rp43-41g5.GenBank:AC099533.36 ATCCACACATGCTCTTTTCTGCAGAAACAGCTGAAGGGACAGGCACCCTCAGTGCGTGAG 61238
human.FAM30B-LOC105372935-GGT2.NCBI.                                         ------------------------------------------------------------ 51642
human.FAM230E-LOC105377182-GGT3P.NCBI.                                       ------------------------------------------------------------ 50294
human.FAM230J-LOC105372942-GGTLC5P.NCBI.rev.compl                            ------------------------------------------------------------ 46364
human.FAM230A.ncRNA.18487127.18500594-GGTLC3.18516335.18518165.rev.compl.REF ------------------------------------------------------------ 31039

chimp.LOC112206744-LOC107973052-GGT2.revcompl                                ------------------------------------------------------------ 52648
chimp54546-135457.revcompl.Pan.troglodytes.clone.rp43-41g5.GenBank:AC099533.36 CCCAAGCCCCTCCCCACTCACTTGGTGACCACAGCAATCTTGACTCTGAAGACCCCTGTC 61298
human.FAM30B-LOC105372935-GGT2.NCBI.                                         ------------------------------------------------------------ 51642
human.FAM230E-LOC105377182-GGT3P.NCBI.                                       ------------------------------------------------------------ 50294
human.FAM230J-LOC105372942-GGTLC5P.NCBI.rev.compl                            ------------------------------------------------------------ 46364
human.FAM230A.ncRNA.18487127.18500594-GGTLC3.18516335.18518165.rev.compl.REF ------------------------------------------------------------ 31039

chimp.LOC112206744-LOC107973052-GGT2.revcompl                                ------------------------------------------------------------ 52648
chimp54546-135457.revcompl.Pan.troglodytes.clone.rp43-41g5.GenBank:AC099533.36 TGTTTCTGGGATGGCATCCTCTTCAAGCTGAACTCCCTGCTGTTGAACTTGACCGAGAGC 61358
human.FAM30B-LOC105372935-GGT2.NCBI.                                         ------------------------------------------------------------ 51642
human.FAM230E-LOC105377182-GGT3P.NCBI.                                       ------------------------------------------------------------ 50294
human.FAM230J-LOC105372942-GGTLC5P.NCBI.rev.compl                            ------------------------------------------------------------ 46364
human.FAM230A.ncRNA.18487127.18500594-GGTLC3.18516335.18518165.rev.compl.REF ------------------------------------------------------------ 31039

chimp.LOC112206744-LOC107973052-GGT2.revcompl                                ------------------------------------------------------------ 52648
chimp54546-135457.revcompl.Pan.troglodytes.clone.rp43-41g5.GenBank:AC099533.36 TTTACTTCAATCTACAGTGGGGAGAAGGGGCACAGTCCTCTAATCCTTCTGGTCCTCATA 61418
human.FAM30B-LOC105372935-GGT2.NCBI.                                         ------------------------------------------------------------ 51642
human.FAM230E-LOC105377182-GGT3P.NCBI.                                       ------------------------------------------------------------ 50294
human.FAM230J-LOC105372942-GGTLC5P.NCBI.rev.compl                            ------------------------------------------------------------ 46364
human.FAM230A.ncRNA.18487127.18500594-GGTLC3.18516335.18518165.rev.compl.REF ------------------------------------------------------------ 31039

chimp.LOC112206744-LOC107973052-GGT2.revcompl                                ------------------------------------------------------------ 52648
chimp54546-135457.revcompl.Pan.troglodytes.clone.rp43-41g5.GenBank:AC099533.36 GCCCAAGGTCCCAAGGACCCCTGAGAGTCTCATCAAATATTTCCACCCCCAAGGCATGCA 61478
human.FAM30B-LOC105372935-GGT2.NCBI.                                         ------------------------------------------------------------ 51642
human.FAM230E-LOC105377182-GGT3P.NCBI.                                       ------------------------------------------------------------ 50294
```

```
human.FAM230J-LOC105372942-GGTLC5P.NCBI.rev.compl                          ------------------------------------------------------------ 46364
human.FAM230A.ncRNA.18487127.18500594-GGTLC3.18516335.18518165.rev.compl.REF ------------------------------------------------------------ 31039

chimp.LOC112206744-LOC107973052-GGT2.revcompl                              ------------------------------------------------------------ 52648
chimp54546-135457.revcompl.Pan.troglodytes.clone.rp43-41g5.GenBank:AC099533.36 GAGGTCTCAGCATGAGGTGGAGCCGCCCCTCGGGGAAGCCCCTAGTCTACTTCAGCAAGA 61538
human.FAM30B-LOC105372935-GGT2.NCBI.                                       ------------------------------------------------------------ 51642
human.FAM230E-LOC105377182-GGT3P.NCBI.                                     ------------------------------------------------------------ 50294
human.FAM230J-LOC105372942-GGTLC5P.NCBI.rev.compl                          ------------------------------------------------------------ 46364
human.FAM230A.ncRNA.18487127.18500594-GGTLC3.18516335.18518165.rev.compl.REF ------------------------------------------------------------ 31039

chimp.LOC112206744-LOC107973052-GGT2.revcompl                              ------------------------------------------------------------ 52648
chimp54546-135457.revcompl.Pan.troglodytes.clone.rp43-41g5.GenBank:AC099533.36 GCCAGGATTGTTGAAGATGATGTAAACGACTGGTTTCTGCAGAACCATCTCCTAAAATCC 61598
human.FAM30B-LOC105372935-GGT2.NCBI.                                       ------------------------------------------------------------ 51642
human.FAM230E-LOC105377182-GGT3P.NCBI.                                     ------------------------------------------------------------ 50294
human.FAM230J-LOC105372942-GGTLC5P.NCBI.rev.compl                          ------------------------------------------------------------ 46364
human.FAM230A.ncRNA.18487127.18500594-GGTLC3.18516335.18518165.rev.compl.REF ------------------------------------------------------------ 31039

chimp.LOC112206744-LOC107973052-GGT2.revcompl                              -----------------tttttgtatttttagtacagatggtgttttgccatgttggcca 52691
chimp54546-135457.revcompl.Pan.troglodytes.clone.rp43-41g5.GenBank:AC099533.36 CCGCTGCTTCTCTTGCTTGCTTACTTTTTTTAAGATATGGGATCTTGCAATGTTGCCCA 61658
human.FAM30B-LOC105372935-GGT2.NCBI.                                       -----------------tttttgtatttttagtacagatggggttttgccatgttggcca 51685
human.FAM230E-LOC105377182-GGT3P.NCBI.                                     -----------------tttttgtatttttagtacagatggggttttgccatgttggcca 50337
human.FAM230J-LOC105372942-GGTLC5P.NCBI.rev.compl                          ------------------------------------------------------------ 46364
human.FAM230A.ncRNA.18487127.18500594-GGTLC3.18516335.18518165.rev.compl.REF ------------------------------------------------------------ 31039

chimp.LOC112206744-LOC107973052-GGT2.revcompl                              ggctgttttcaaactcttgacctcaggtcatccacccacctcagccttccaaggtgctgg 52751
chimp54546-135457.revcompl.Pan.troglodytes.clone.rp43-41g5.GenBank:AC099533.36 GGCTGTAATCAAACTCTTGGGCTCCAGTGATTCTCCTGGCTCAGCCTCCAGAGTAGCTGA 61718
human.FAM30B-LOC105372935-GGT2.NCBI.                                       ggctgttttcaaactcctgacctcaggtcatccacccacctcagccttccaaggtgctgg 51745
human.FAM230E-LOC105377182-GGT3P.NCBI.                                     ggctgttttcagactcctgacctcaggtcatccacccacctcagccttccaaggtgctgg 50397
human.FAM230J-LOC105372942-GGTLC5P.NCBI.rev.compl                          ------------------------------------------------------------ 46364
human.FAM230A.ncRNA.18487127.18500594-GGTLC3.18516335.18518165.rev.compl.REF ------------------------------------------------------------ 31039

chimp.LOC112206744-LOC107973052-GGT2.revcompl                              gatgacaggcgtgagccaccgtgcccagccaagacaggagaagttctaatctttgatagc 52811
chimp54546-135457.revcompl.Pan.troglodytes.clone.rp43-41g5.GenBank:AC099533.36 GATTATAGGTGT-----ACTGTGACCAGCTTTCTCTTTCTTAGTAACAATTAAAAT--- 61770
human.FAM30B-LOC105372935-GGT2.NCBI.                                       gatgacaggcgtgagccaccgtgcccagccaagacaggagaagttctaatctttgatagc 51805
human.FAM230E-LOC105377182-GGT3P.NCBI.                                     gatgacaggcgtgagccaccgtgcccagccaagacaggagaagttctaatctttgatagc 50457
human.FAM230J-LOC105372942-GGTLC5P.NCBI.rev.compl                          ------------------------------------------------------------ 46364
human.FAM230A.ncRNA.18487127.18500594-GGTLC3.18516335.18518165.rev.compl.REF ------------------------------------------------------------ 31039

chimp.LOC112206744-LOC107973052-GGT2.revcompl                              agaccagggtgacgatgcttagcaacagtattttgtatatttcaaagtaacgaagagagg 52871
chimp54546-135457.revcompl.Pan.troglodytes.clone.rp43-41g5.GenBank:AC099533.36 -------------------------------------------TAAGTAGGAAAGACAGA 61787
human.FAM30B-LOC105372935-GGT2.NCBI.                                       agaccagggtgacgatgcttagcaacagtattttgtatatttcaaagtaacgaagagagg 51865
human.FAM230E-LOC105377182-GGT3P.NCBI.                                     agaccagggtgacgatgcttagcaacagtattttgtatatttcaaagtaacgaagagagc 50517
human.FAM230J-LOC105372942-GGTLC5P.NCBI.rev.compl                          ------------------------------------------------------------ 46364
human.FAM230A.ncRNA.18487127.18500594-GGTLC3.18516335.18518165.rev.compl.REF ------------------------------------------------------------ 31039

chimp.LOC112206744-LOC107973052-GGT2.revcompl                              actatggtgctaacacccagaaatgaaaaatattcaaggt-------------------- 52911
chimp54546-135457.revcompl.Pan.troglodytes.clone.rp43-41g5.GenBank:AC099533.36 ATAATATAAGAAAAAGCCAAGAATCTCAAATATTAACATTTTACCATTTATACCCGGGCC 61847
human.FAM30B-LOC105372935-GGT2.NCBI.                                       actatggtgctaacacccggaaatgaaaaatattcaaggt-------------------- 51905
human.FAM230E-LOC105377182-GGT3P.NCBI.                                     actatggtgctaacacccagaaatgaaaaatattcaaggt-------------------- 50557
human.FAM230J-LOC105372942-GGTLC5P.NCBI.rev.compl                          ------------------------------------------------------------ 46364
human.FAM230A.ncRNA.18487127.18500594-GGTLC3.18516335.18518165.rev.compl.REF ------------------------------------------------------------ 31039

chimp.LOC112206744-LOC107973052-GGT2.revcompl                              ------------------------------------------------------------ 52911
chimp54546-135457.revcompl.Pan.troglodytes.clone.rp43-41g5.GenBank:AC099533.36 TTTTTGGGTGTGTTATTGCTTTTAAGGTCTAAACCTTTCTGGGGTACCTAAAGACAAATC 61907
human.FAM30B-LOC105372935-GGT2.NCBI.                                       ------------------------------------------------------------ 51905
human.FAM230E-LOC105377182-GGT3P.NCBI.                                     ------------------------------------------------------------ 50557
human.FAM230J-LOC105372942-GGTLC5P.NCBI.rev.compl                          ------------------------------------------------------------ 46364
human.FAM230A.ncRNA.18487127.18500594-GGTLC3.18516335.18518165.rev.compl.REF ------------------------------------------------------------ 31039

chimp.LOC112206744-LOC107973052-GGT2.revcompl                              --------------------------------------gacggagactccaaataccctg 52933
chimp54546-135457.revcompl.Pan.troglodytes.clone.rp43-41g5.GenBank:AC099533.36 AGGGGTCTCCATTTCTATGGCAGCCCCCAGGTCCCTGAGGGGAGGCCCCACCTGTCCTG 61967
human.FAM30B-LOC105372935-GGT2.NCBI.                                       --------------------------------------gacggagactccaaataccctg 51927
human.FAM230E-LOC105377182-GGT3P.NCBI.                                     --------------------------------------gacggagactccaaataccctg 50579
human.FAM230J-LOC105372942-GGTLC5P.NCBI.rev.compl                          ------------------------------------------------------------ 46364
human.FAM230A.ncRNA.18487127.18500594-GGTLC3.18516335.18518165.rev.compl.REF ------------------------------------------------------------ 31039

chimp.LOC112206744-LOC107973052-GGT2.revcompl                              ccttgatcattatacactatatgcatggaacaagcactcacatgtacccataaatataga 52993
chimp54546-135457.revcompl.Pan.troglodytes.clone.rp43-41g5.GenBank:AC099533.36 GTGGTGCCATGAGACCTTTGTTTGAGGAGGGATGCTCAGGGCCTGGCTGGCAACTGCATT 62027
human.FAM30B-LOC105372935-GGT2.NCBI.                                       ccttgatcattatacactctatgcatggaacaagcactcacatgtacccataaatataga 51987
human.FAM230E-LOC105377182-GGT3P.NCBI.                                     ccttgatcattatacactctatgcatggaacaagcactcacatgtacccataaatataga 50639
human.FAM230J-LOC105372942-GGTLC5P.NCBI.rev.compl                          ------------------------------------------------------------ 46364
human.FAM230A.ncRNA.18487127.18500594-GGTLC3.18516335.18518165.rev.compl.REF ------------------------------------------------------------ 31039

chimp.LOC112206744-LOC107973052-GGT2.revcompl                              aaatatcatgtatcaatatcagaaaaaaatctcctcctgacctcagcccaatcaggctct 53053
chimp54546-135457.revcompl.Pan.troglodytes.clone.rp43-41g5.GenBank:AC099533.36 CCAGCCAATGAATGACAATTCCGGTGCATGGTGATTCTGCTCTTGGGGGCCCTCCCTTCT 62087
human.FAM30B-LOC105372935-GGT2.NCBI.                                       aaatatcatgtatcaatatcagaaaaaaatctcctcctgacctcagcccaatcaggctct 52047
human.FAM230E-LOC105377182-GGT3P.NCBI.                                     aaatatcatgtatcaatatcagaaaaaaatctcctcctgacctcagcccaatcaggctct 50699
human.FAM230J-LOC105372942-GGTLC5P.NCBI.rev.compl                          ------------------------------------------------------------ 46364
human.FAM230A.ncRNA.18487127.18500594-GGTLC3.18516335.18518165.rev.compl.REF ------------------------------------------------------------ 31039

chimp.LOC112206744-LOC107973052-GGT2.revcompl                              catgccaccacacttgccaagttctctg-------------------------------- 53081
chimp54546-135457.revcompl.Pan.troglodytes.clone.rp43-41g5.GenBank:AC099533.36 CCAACCAGGCTCCCTCTCAGATGCTGCCTCAGGCTCAGCCACCTCTGACTGATTTGAAAT 62147
human.FAM30B-LOC105372935-GGT2.NCBI.                                       catgccaccacacttgccaagttctctg-------------------------------- 52075
human.FAM230E-LOC105377182-GGT3P.NCBI.                                     catgccaccacacttgccaagttctctg-------------------------------- 50727
human.FAM230J-LOC105372942-GGTLC5P.NCBI.rev.compl                          ------------------------------------------------------------ 46364
human.FAM230A.ncRNA.18487127.18500594-GGTLC3.18516335.18518165.rev.compl.REF ------------------------------------------------------------ 31039

chimp.LOC112206744-LOC107973052-GGT2.revcompl                              ------------------------------------------------------------ 53081
chimp54546-135457.revcompl.Pan.troglodytes.clone.rp43-41g5.GenBank:AC099533.36 GAACAGAGGCCACCCTATCCTTCTGGGGGAAGTGGGCACCAGGAGAGTCCCACGGACACG 62207
human.FAM30B-LOC105372935-GGT2.NCBI.                                       ------------------------------------------------------------ 52075
human.FAM230E-LOC105377182-GGT3P.NCBI.                                     ------------------------------------------------------------ 50727
human.FAM230J-LOC105372942-GGTLC5P.NCBI.rev.compl                          ------------------------------------------------------------ 46364
human.FAM230A.ncRNA.18487127.18500594-GGTLC3.18516335.18518165.rev.compl.REF ------------------------------------------------------------ 31039

chimp.LOC112206744-LOC107973052-GGT2.revcompl                              ------------------------------------------------------------ 53081
chimp54546-135457.revcompl.Pan.troglodytes.clone.rp43-41g5.GenBank:AC099533.36 TACCCCATTCATGGCGATGACGGTGCGCTGCCAGTCTCTGTCCTGCAGGGCCTGCGGGTC 62267
human.FAM30B-LOC105372935-GGT2.NCBI.                                       ------------------------------------------------------------ 52075
human.FAM230E-LOC105377182-GGT3P.NCBI.                                     ------------------------------------------------------------ 50727
human.FAM230J-LOC105372942-GGTLC5P.NCBI.rev.compl                          ------------------------------------------------------------ 46364
human.FAM230A.ncRNA.18487127.18500594-GGTLC3.18516335.18518165.rev.compl.REF ------------------------------------------------------------ 31039

chimp.LOC112206744-LOC107973052-GGT2.revcompl                              ------------------------------------------------------------ 53081
chimp54546-135457.revcompl.Pan.troglodytes.clone.rp43-41g5.GenBank:AC099533.36 CAGCTGAAAGCAAGGAGTCATGATTGGCAGAGGCCCTGGGGTCTGTTCAGAGGCCCCACG 62327
human.FAM30B-LOC105372935-GGT2.NCBI.                                       ------------------------------------------------------------ 52075
human.FAM230E-LOC105377182-GGT3P.NCBI.                                     ------------------------------------------------------------ 50727
human.FAM230J-LOC105372942-GGTLC5P.NCBI.rev.compl                          ------------------------------------------------------------ 46364
human.FAM230A.ncRNA.18487127.18500594-GGTLC3.18516335.18518165.rev.compl.REF ------------------------------------------------------------ 31039

chimp.LOC112206744-LOC107973052-GGT2.revcompl                              -----------------------------------------------gtgacccccacac 53094
chimp54546-135457.revcompl.Pan.troglodytes.clone.rp43-41g5.GenBank:AC099533.36 GGGCAGCAGCCCACATCCGACACTTCCCCAACCCTTAGTCAGAGGCAGAGGCCCGACTGC 62387
human.FAM30B-LOC105372935-GGT2.NCBI.                                       -----------------------------------------------gtgacccccacac 52088
human.FAM230E-LOC105377182-GGT3P.NCBI.                                     -----------------------------------------------gtgacccccacac 50740
human.FAM230J-LOC105372942-GGTLC5P.NCBI.rev.compl                          ------------------------------------------------------------ 46364
human.FAM230A.ncRNA.18487127.18500594-GGTLC3.18516335.18518165.rev.compl.REF ------------------------------------------------------------ 31039

chimp.LOC112206744-LOC107973052-GGT2.revcompl                              tgccagacccagtgccccctctcagctttactgggctcatcactctccctgagagccgcc 53154
chimp54546-135457.revcompl.Pan.troglodytes.clone.rp43-41g5.GenBank:AC099533.36 GGCCAGCACCTCCATCCAGAGGCCGAGAGAGGTAGAGTAAGGAGGGGCCTGCCAGACACC 62447
human.FAM30B-LOC105372935-GGT2.NCBI.                                       tgccagacccagtgccccctctcagctttactgggctcatcactctccctgagagccgcc 52148
human.FAM230E-LOC105377182-GGT3P.NCBI.                                     tgccagacccagtgccccctctcagctttactgggctcatcactctccctgagagccgcc 50800
human.FAM230J-LOC105372942-GGTLC5P.NCBI.rev.compl                          ------------------------------------------------------------ 46364
human.FAM230A.ncRNA.18487127.18500594-GGTLC3.18516335.18518165.rev.compl.REF ------------------------------------------------------------ 31039

chimp.LOC112206744-LOC107973052-GGT2.revcompl                              cctgcatcccagcacctggctccacccgagtctcccccgcctgccatcctagctcctact 53214
chimp54546-135457.revcompl.Pan.troglodytes.clone.rp43-41g5.GenBank:AC099533.36 CCTGGAACCCAGGGCCCCACTGCTCCCAGAGCCCTGCTGCCTGGGGTCC--------CCA 62499
human.FAM30B-LOC105372935-GGT2.NCBI.                                       cctgcatcccagcacctggctccacccgagtctcccccgcctgccatcctagctcctact 52208
human.FAM230E-LOC105377182-GGT3P.NCBI.                                     cctgcatcccagcacctggctccacccgagtctcccccgcctgccatcctagctcctact 50860
human.FAM230J-LOC105372942-GGTLC5P.NCBI.rev.compl                          ------------------------------------------------------------ 46364
```

```
human.FAM230A.ncRNA.18487127.18500594-GGTLC3.18516335.18518165.rev.compl.REF         ------------------------------------------------------------ 31039

chimp.LOC112206744-LOC107973052-GGT2.revcompl                                        ctcccctctgtctttgctctctctcctggtggtctgcttgacatctgagcttcagcctcc 53274
chimp54546-135457.revcompl.Pan.troglodytes.clone.rp43-41g5.GenBank:AC099533.36      CCCCCTCCTCCCTCTGCCCTCCCAGTTGGGAGCTGATGTGCAGGCTGGCAGGCTCTCTCC 62559
human.FAM30B-LOC105372935-GGT2.NCBI.                                                 ctcccctctgtctttgctctctctcctggtggtctgcttgacatctgagcttcagcctcc 52268
human.FAM230E-LOC105377182-GGT3P.NCBI.                                               ctcccctctgtctttgctctctctcctggtggtctgcttgacatctgagcttcagcctcc 50920
human.FAM230J-LOC105372942-GGTLC5P.NCBI.rev.compl                                    ------------------------------------------------------------ 46364
human.FAM230A.ncRNA.18487127.18500594-GGTLC3.18516335.18518165.rev.compl.REF         ------------------------------------------------------------ 31039

chimp.LOC112206744-LOC107973052-GGT2.revcompl                                        atttatgcactgacaactcccaaattgacctgctggcctggactgctcctctgatcacca 53334
chimp54546-135457.revcompl.Pan.troglodytes.clone.rp43-41g5.GenBank:AC099533.36      ATCCGGCTCCCATCACCACGGGTGCTATGTGGAGCTCAGTGAAAGGTGAGCTGTCCAGCA 62619
human.FAM30B-LOC105372935-GGT2.NCBI.                                                 atttatgcactgacaactctcaaattgacctgctggcctggactgctcctccgatcacca 52328
human.FAM230E-LOC105377182-GGT3P.NCBI.                                               atttatgcactgacaactctcaaattgacctgctggcctggactgctcctccgatcacca 50980
human.FAM230J-LOC105372942-GGTLC5P.NCBI.rev.compl                                    ------------------------------------------------------------ 46364
human.FAM230A.ncRNA.18487127.18500594-GGTLC3.18516335.18518165.rev.compl.REF         ------------------------------------------------------------ 31039

chimp.LOC112206744-LOC107973052-GGT2.revcompl                                        g----------------------------------------------------------- 53335
chimp54546-135457.revcompl.Pan.troglodytes.clone.rp43-41g5.GenBank:AC099533.36      GCAACTTCAGTAACAACTCCTGGGGGCCTGGCATCCACAGCTGCTGTCTGGGGAACTGTG 62679
human.FAM30B-LOC105372935-GGT2.NCBI.                                                 g----------------------------------------------------------- 52329
human.FAM230E-LOC105377182-GGT3P.NCBI.                                               g----------------------------------------------------------- 50981
human.FAM230J-LOC105372942-GGTLC5P.NCBI.rev.compl                                    ------------------------------------------------------------ 46364
human.FAM230A.ncRNA.18487127.18500594-GGTLC3.18516335.18518165.rev.compl.REF         ------------------------------------------------------------ 31039

chimp.LOC112206744-LOC107973052-GGT2.revcompl                                        ------------------------------------------------------------ 53335
chimp54546-135457.revcompl.Pan.troglodytes.clone.rp43-41g5.GenBank:AC099533.36      GACACTGTTCTCCCCCTGCACCATGAACAGCTTGTGGCGGGGGGAGCCTGCAGGCCACAC 62739
human.FAM30B-LOC105372935-GGT2.NCBI.                                                 ------------------------------------------------------------ 52329
human.FAM230E-LOC105377182-GGT3P.NCBI.                                               ------------------------------------------------------------ 50981
human.FAM230J-LOC105372942-GGTLC5P.NCBI.rev.compl                                    ------------------------------------------------------------ 46364
human.FAM230A.ncRNA.18487127.18500594-GGTLC3.18516335.18518165.rev.compl.REF         ------------------------------------------------------------ 31039

chimp.LOC112206744-LOC107973052-GGT2.revcompl                                        ------------------------------------------------------------ 53335
chimp54546-135457.revcompl.Pan.troglodytes.clone.rp43-41g5.GenBank:AC099533.36      CAGTCCCATGGTTCAGAATCAGGCCCAGAAGGACCCAGAAACAAGCCCTAAATCAAGCAG 62799
human.FAM30B-LOC105372935-GGT2.NCBI.                                                 ------------------------------------------------------------ 52329
human.FAM230E-LOC105377182-GGT3P.NCBI.                                               ------------------------------------------------------------ 50981
human.FAM230J-LOC105372942-GGTLC5P.NCBI.rev.compl                                    ------------------------------------------------------------ 46364
human.FAM230A.ncRNA.18487127.18500594-GGTLC3.18516335.18518165.rev.compl.REF         ------------------------------------------------------------ 31039

chimp.LOC112206744-LOC107973052-GGT2.revcompl                                        ------------------------------------------------------------ 53335
chimp54546-135457.revcompl.Pan.troglodytes.clone.rp43-41g5.GenBank:AC099533.36      CTGATGAGGGGGCCGGGAGGAAGCCCCTCTGTGACCCCTCTAACTTCTACCCCCAGCCAG 62859
human.FAM30B-LOC105372935-GGT2.NCBI.                                                 ------------------------------------------------------------ 52329
human.FAM230E-LOC105377182-GGT3P.NCBI.                                               ------------------------------------------------------------ 50981
human.FAM230J-LOC105372942-GGTLC5P.NCBI.rev.compl                                    ------------------------------------------------------------ 46364
human.FAM230A.ncRNA.18487127.18500594-GGTLC3.18516335.18518165.rev.compl.REF         ------------------------------------------------------------ 31039

chimp.LOC112206744-LOC107973052-GGT2.revcompl                                        ------------------------------------------------------------ 53335
chimp54546-135457.revcompl.Pan.troglodytes.clone.rp43-41g5.GenBank:AC099533.36      CACTATCCAGGCAGCTAGCCTGCAGGAGCTTGTCCTTAATGTCACCCCAACACAGTCTGT 62919
human.FAM30B-LOC105372935-GGT2.NCBI.                                                 ------------------------------------------------------------ 52329
human.FAM230E-LOC105377182-GGT3P.NCBI.                                               ------------------------------------------------------------ 50981
human.FAM230J-LOC105372942-GGTLC5P.NCBI.rev.compl                                    ------------------------------------------------------------ 46364
human.FAM230A.ncRNA.18487127.18500594-GGTLC3.18516335.18518165.rev.compl.REF         ------------------------------------------------------------ 31039

chimp.LOC112206744-LOC107973052-GGT2.revcompl                                        ------------------------------------------------------------ 53335
chimp54546-135457.revcompl.Pan.troglodytes.clone.rp43-41g5.GenBank:AC099533.36      CCTGGTATGTAACACCCTGACTGTCATGACCAATCCACATGTCCCCCACCCAGGAACGCT 62979
human.FAM30B-LOC105372935-GGT2.NCBI.                                                 ------------------------------------------------------------ 52329
human.FAM230E-LOC105377182-GGT3P.NCBI.                                               ------------------------------------------------------------ 50981
human.FAM230J-LOC105372942-GGTLC5P.NCBI.rev.compl                                    ------------------------------------------------------------ 46364
human.FAM230A.ncRNA.18487127.18500594-GGTLC3.18516335.18518165.rev.compl.REF         ------------------------------------------------------------ 31039

chimp.LOC112206744-LOC107973052-GGT2.revcompl                                        ------------------------------------------------------------ 53335
chimp54546-135457.revcompl.Pan.troglodytes.clone.rp43-41g5.GenBank:AC099533.36      CTGTTTCCCACTTTATGGGGAGCACAAGCTCTATGTCAGCGGTTCCTGGGTATGCCAAGA 63039
human.FAM30B-LOC105372935-GGT2.NCBI.                                                 ------------------------------------------------------------ 52329
human.FAM230E-LOC105377182-GGT3P.NCBI.                                               ------------------------------------------------------------ 50981
human.FAM230J-LOC105372942-GGTLC5P.NCBI.rev.compl                                    ------------------------------------------------------------ 46364
human.FAM230A.ncRNA.18487127.18500594-GGTLC3.18516335.18518165.rev.compl.REF         ------------------------------------------------------------ 31039

chimp.LOC112206744-LOC107973052-GGT2.revcompl                                        --acctgagtatctacctgcctgcttgaagaaagcatctcaaacttcaacgtgcccaaaa 53393
chimp54546-135457.revcompl.Pan.troglodytes.clone.rp43-41g5.GenBank:AC099533.36      CCACTGGCTGGGCTGGGTGAATGAGCAGTATCATCTTGTTAAAAATACACCTTCCTGGGT 63099
human.FAM30B-LOC105372935-GGT2.NCBI.                                                 --acctgaggatctacctgcctgctcgaagaaagcatctcaaacttcaacgtgcccaaaa 52387
human.FAM230E-LOC105377182-GGT3P.NCBI.                                               --acctgaggatctacctgcctgcttgaagaaagcatctcaaacttcaacgtgcccaaaa 51039
human.FAM230J-LOC105372942-GGTLC5P.NCBI.rev.compl                                    ------------------------------------------------------------ 46364
human.FAM230A.ncRNA.18487127.18500594-GGTLC3.18516335.18518165.rev.compl.REF         ------------------------------------------------------------ 31039

chimp.LOC112206744-LOC107973052-GGT2.revcompl                                        ccgagctcctgagtgtctgctcaacctgcttcctgagaaccctgcctgtctccattaggg 53453
chimp54546-135457.revcompl.Pan.troglodytes.clone.rp43-41g5.GenBank:AC099533.36      TCCACCTGCAGAGAACTGGACTCATAATCTGAGTGGGACCTGGGATTTACACAGCTAGAA 63159
human.FAM30B-LOC105372935-GGT2.NCBI.                                                 ccgagctcctgagtgtctgctcaacctgcttcctgagaaccctgcctgtctccattaggg 52447
human.FAM230E-LOC105377182-GGT3P.NCBI.                                               ccgagctcctgagtgtctgctcaacctgcttcctgagaaccctgcctgtctccattaggg 51099
human.FAM230J-LOC105372942-GGTLC5P.NCBI.rev.compl                                    ------------------------------------------------------------ 46364
human.FAM230A.ncRNA.18487127.18500594-GGTLC3.18516335.18518165.rev.compl.REF         ------------------------------------------------------------ 31039

chimp.LOC112206744-LOC107973052-GGT2.revcompl                                        tcaccccatccttccaggtacagacaaaagatcaggggtccccggggactccctacacaa 53513
chimp54546-135457.revcompl.Pan.troglodytes.clone.rp43-41g5.GenBank:AC099533.36      ACCCCAGGTAGTTCTGGAGTGAAGCCAGGTCACAGAGCCACCGTCTGACTCCATAGAAGG 63219
human.FAM30B-LOC105372935-GGT2.NCBI.                                                 tcaccccatccttccaggtacagacaaaagatcaggggtccccggggactccctacacaa 52507
human.FAM230E-LOC105377182-GGT3P.NCBI.                                               tcaccccatccttccaggtacagacaaaagatcaggggtccccggggactccctacacaa 51159
human.FAM230J-LOC105372942-GGTLC5P.NCBI.rev.compl                                    ------------------------------------------------------------ 46364
human.FAM230A.ncRNA.18487127.18500594-GGTLC3.18516335.18518165.rev.compl.REF         ------------------------------------------------------------ 31039

chimp.LOC112206744-LOC107973052-GGT2.revcompl                                        gcgtcacacccaacccatcctcaaatccgcaggctccacttccaagtgtgtctgtccagc 53573
chimp54546-135457.revcompl.Pan.troglodytes.clone.rp43-41g5.GenBank:AC099533.36      CCACTGCATCTGGCCTTGTGTTTCCATCCCTTGATTCAGGTGATGGTTCACTTCAGCCAC 63279
human.FAM30B-LOC105372935-GGT2.NCBI.                                                 gcgtcacacccaacccatcctcaaatccacaggctccacttccaagtgtgtctgtgcagc 52567
human.FAM230E-LOC105377182-GGT3P.NCBI.                                               gcgtcacacccaacccatcctcaaatccacaggctccacttccaagtgtgtctgtgcagc 51219
human.FAM230J-LOC105372942-GGTLC5P.NCBI.rev.compl                                    ------------------------------------------------------------ 46364
human.FAM230A.ncRNA.18487127.18500594-GGTLC3.18516335.18518165.rev.compl.REF         ------------------------------------------------------------ 31039

chimp.LOC112206744-LOC107973052-GGT2.revcompl                                        gtcagccacttcccagcac-----------tctctccacgaattactgcagtgacctccg 53622
chimp54546-135457.revcompl.Pan.troglodytes.clone.rp43-41g5.GenBank:AC099533.36      AAGGCAAGCTGCCTAACAGCAGGGACCACACCTCATGATGTCTGCACACATTCACATGCG 63339
human.FAM30B-LOC105372935-GGT2.NCBI.                                                 gtcagccacttcccagcac-----------cctctccacgaattactgcagtgacctccg 52616
human.FAM230E-LOC105377182-GGT3P.NCBI.                                               gtcagccacttcccagcac-----------cctctccacgaattactgcagtgacctccg 51268
human.FAM230J-LOC105372942-GGTLC5P.NCBI.rev.compl                                    ------------------------------------------------------------ 46364
human.FAM230A.ncRNA.18487127.18500594-GGTLC3.18516335.18518165.rev.compl.REF         ------------------------------------------------------------ 31039

chimp.LOC112206744-LOC107973052-GGT2.revcompl                                        gacaggcccccacatgctccctgccccttacacagcaacccaaggggtccatagaccaga 53682
chimp54546-135457.revcompl.Pan.troglodytes.clone.rp43-41g5.GenBank:AC099533.36      TGCACATATACATACACACACACGACGGTGCTGAGCACAGCGCCAGGCCTAGAGAGGGGC 63399
human.FAM30B-LOC105372935-GGT2.NCBI.                                                 gacaggtccccacatgctccctgccccttacacagcaatccaaggggtccatagaccaga 52676
human.FAM230E-LOC105377182-GGT3P.NCBI.                                               gacaggtccccacatgctccctgccccttacacagcaatccaaggggtccatagaccaga 51328
human.FAM230J-LOC105372942-GGTLC5P.NCBI.rev.compl                                    ------------------------------------------------------------ 46364
human.FAM230A.ncRNA.18487127.18500594-GGTLC3.18516335.18518165.rev.compl.REF         ------------------------------------------------------------ 31039

chimp.LOC112206744-LOC107973052-GGT2.revcompl                                        t--------ccatcgccttccactcacatactccacgagcccccacttccctcagacagg 53734
chimp54546-135457.revcompl.Pan.troglodytes.clone.rp43-41g5.GenBank:AC099533.36      TGAGGACAGGCATCATTTCCACTGCAAGGGACCCTAGCGGCCACAGCCACCTGGGCCTTC 63459
human.FAM30B-LOC105372935-GGT2.NCBI.                                                 t--------ccatccccttccagtcacacactccacgagcccccacttccctcagacagg 52728
human.FAM230E-LOC105377182-GGT3P.NCBI.                                               t--------ccatccccttccagtcacacactccacgagcccccacttccctcagacagg 51380
human.FAM230J-LOC105372942-GGTLC5P.NCBI.rev.compl                                    ------------------------------------------------------------ 46364
human.FAM230A.ncRNA.18487127.18500594-GGTLC3.18516335.18518165.rev.compl.REF         ------------------------------------------------------------ 31039

chimp.LOC112206744-LOC107973052-GGT2.revcompl                                        aagcagaggcttcaccataacctaagagatcccacacaacct----gggccattcccctt 53790
chimp54546-135457.revcompl.Pan.troglodytes.clone.rp43-41g5.GenBank:AC099533.36      TGCAGGTGAAGTGGCCAGAAAATAAGAAGCTCTGGAGCAGGTCAAGGAGCCCAGCCAGCT 63519
human.FAM30B-LOC105372935-GGT2.NCBI.                                                 aagcagaggcttcaccataacctaagagatcccgcacaacct----gggccattcccctt 52784
human.FAM230E-LOC105377182-GGT3P.NCBI.                                               aagcagaggcttcaccataacctaagagatcccgcacaacct----gggccattcccctt 51436
human.FAM230J-LOC105372942-GGTLC5P.NCBI.rev.compl                                    ------------------------------------------------------------ 46364
human.FAM230A.ncRNA.18487127.18500594-GGTLC3.18516335.18518165.rev.compl.REF         ------------------------------------------------------------ 31039

chimp.LOC112206744-LOC107973052-GGT2.revcompl                                        gggtcacttgctgcagcctccccagctccccacagggctctgtccctgc----catcaca 53846
chimp54546-135457.revcompl.Pan.troglodytes.clone.rp43-41g5.GenBank:AC099533.36      GCCCCTACTTCACAGGTGTGTGCACCTGGGCCTCCCTCCCTGCACCTGCTCAGCATGAAG 63579
human.FAM30B-LOC105372935-GGT2.NCBI.                                                 ggatcacttgctgcagcctccccagctccccacagggctctgtccctgc----catcaca 52840
human.FAM230E-LOC105377182-GGT3P.NCBI.                                               ggatcacttgctgcagcctccccagctccccacagggctctgtccctgc----catcaca 51492
human.FAM230J-LOC105372942-GGTLC5P.NCBI.rev.compl                                    ------------------------------------------------------------ 46364
human.FAM230A.ncRNA.18487127.18500594-GGTLC3.18516335.18518165.rev.compl.REF         ------------------------------------------------------------ 31039
```

```
chimp.LOC112206744-LOC107973052-GGT2.revcompl                                        cctggatagcagaccaggagatagctcccctgaccccatctctgcctctgggtctt---- 53902
chimp54546-135457.revcompl.Pan.troglodytes.clone.rp43-41g5.GenBank:AC099533.36       GCTCAGCACAGGCACACGTGGGGGGTGACCCCTTCTCACCTCCCCTTCTAGGTCCTACTG 63639
human.FAM30B-LOC105372935-GGT2.NCBI.                                                 cctggatagcagaccaggagataactcccctgaccccatctctgcctctgggtctt---- 52896
human.FAM230E-LOC105377182-GGT3P.NCBI.                                               cctggatagcagaccaggagataactcccctgaccccatctctgcctctgggtctt---- 51548
human.FAM230J-LOC105372942-GGTLC5P.NCBI.rev.compl                                    ------------------------------------------------------------ 46364
human.FAM230A.ncRNA.18487127.18500594-GGTLC3.18516335.18518165.rev.compl.REF         ------------------------------------------------------------ 31039

chimp.LOC112206744-LOC107973052-GGT2.revcompl                                        ------------------------------------------------------------ 53902
chimp54546-135457.revcompl.Pan.troglodytes.clone.rp43-41g5.GenBank:AC099533.36       GTTAACATTTCCCAAGGCTCTGAACTGTGCAGCCAGAGTGGCACAGAGAAGCAAAGGGCG 63699
human.FAM30B-LOC105372935-GGT2.NCBI.                                                 ------------------------------------------------------------ 52896
human.FAM230E-LOC105377182-GGT3P.NCBI.                                               ------------------------------------------------------------ 51548
human.FAM230J-LOC105372942-GGTLC5P.NCBI.rev.compl                                    ------------------------------------------------------------ 46364
human.FAM230A.ncRNA.18487127.18500594-GGTLC3.18516335.18518165.rev.compl.REF         ------------------------------------------------------------ 31039

chimp.LOC112206744-LOC107973052-GGT2.revcompl                                        ------------------------------------------------------------ 53902
chimp54546-135457.revcompl.Pan.troglodytes.clone.rp43-41g5.GenBank:AC099533.36       GCGGGACGTCTCCCACCAGGGGCCATGTGGCCACAGGCTCTCATTCCGCACCTGGCCCTG 63759
human.FAM30B-LOC105372935-GGT2.NCBI.                                                 ------------------------------------------------------------ 52896
human.FAM230E-LOC105377182-GGT3P.NCBI.                                               ------------------------------------------------------------ 51548
human.FAM230J-LOC105372942-GGTLC5P.NCBI.rev.compl                                    ------------------------------------------------------------ 46364
human.FAM230A.ncRNA.18487127.18500594-GGTLC3.18516335.18518165.rev.compl.REF         ------------------------------------------------------------ 31039

chimp.LOC112206744-LOC107973052-GGT2.revcompl                                        ----tgctcagatgtccccttccctgactaggtcaccctccatagagtcccagattttga 53958
chimp54546-135457.revcompl.Pan.troglodytes.clone.rp43-41g5.GenBank:AC099533.36       AAGGTTGTCAGAGGCCACACTCCAGGCCTCAGGGACCCCACCCTGAGAAGCAGCATCTGT 63819
human.FAM30B-LOC105372935-GGT2.NCBI.                                                 ----tgctcagatgtccccttccctgactaggtcaccctccatagagtcccagattttga 52952
human.FAM230E-LOC105377182-GGT3P.NCBI.                                               ----tgctcagatgtccccttccctgactaggtcaccctccatagagtcccagattttga 51604
human.FAM230J-LOC105372942-GGTLC5P.NCBI.rev.compl                                    ------------------------------------------------------------ 46364
human.FAM230A.ncRNA.18487127.18500594-GGTLC3.18516335.18518165.rev.compl.REF         ------------------------------------------------------------ 31039

chimp.LOC112206744-LOC107973052-GGT2.revcompl                                        ggccctccaggtctgtttttctacagcctgtaacacacccgcacctgcctagtttctttt 54018
chimp54546-135457.revcompl.Pan.troglodytes.clone.rp43-41g5.GenBank:AC099533.36       GAGCAGGGAGCGGTCAGTCAGAACCACACATAAAAAGGAAACTCTCAAGTGGTACTTTCC 63879
human.FAM30B-LOC105372935-GGT2.NCBI.                                                 ggccctccaggtctgtttttctacagcccgtaacacacccgcacttgcctagtttctcct 53012
human.FAM230E-LOC105377182-GGT3P.NCBI.                                               ggccctccaggtctgtttttctacagcccgtaacacacccgcacttgcctagtttctcct 51664
human.FAM230J-LOC105372942-GGTLC5P.NCBI.rev.compl                                    ------------------------------------------------------------ 46364
human.FAM230A.ncRNA.18487127.18500594-GGTLC3.18516335.18518165.rev.compl.REF         ------------------------------------------------------------ 31039

chimp.LOC112206744-LOC107973052-GGT2.revcompl                                        cccacctggagtgtcacagatttcatctgccgtctttgtttttcaccccagcttcaggaa 54078
chimp54546-135457.revcompl.Pan.troglodytes.clone.rp43-41g5.GenBank:AC099533.36       CCCGGAGTG--------------------------------------------------- 63888
human.FAM30B-LOC105372935-GGT2.NCBI.                                                 cccacctggagtgtcacagatttcatctgccgtctttgtttttcaccccagcttcaggaa 53072
human.FAM230E-LOC105377182-GGT3P.NCBI.                                               cccacctggagtgtcacagatttcatctgccgtctttgtttttcaccccagcttcaggaa 51724
human.FAM230J-LOC105372942-GGTLC5P.NCBI.rev.compl                                    ------------------------------------------------------------ 46364
human.FAM230A.ncRNA.18487127.18500594-GGTLC3.18516335.18518165.rev.compl.REF         ------------------------------------------------------------ 31039

chimp.LOC112206744-LOC107973052-GGT2.revcompl                                        caacagctgattctttaagacaatgctcaatacattctagttaaataaatgattctaagc 54138
chimp54546-135457.revcompl.Pan.troglodytes.clone.rp43-41g5.GenBank:AC099533.36       --------------------------------------AACCCTAAGCAGAGGCAGGAGA 63910
human.FAM30B-LOC105372935-GGT2.NCBI.                                                 caacagctgattctttaagacaatgctcaatacattctagtcaaataaatggttttaagc 53132
human.FAM230E-LOC105377182-GGT3P.NCBI.                                               caacagctgattctttaagacaatgctcaatacattctagtcaaataaatggttttaagc 51784
human.FAM230J-LOC105372942-GGTLC5P.NCBI.rev.compl                                    ------------------------------------------------------------ 46364
human.FAM230A.ncRNA.18487127.18500594-GGTLC3.18516335.18518165.rev.compl.REF         ------------------------------------------------------------ 31039

chimp.LOC112206744-LOC107973052-GGT2.revcompl                                        gtccacaaggtgccaagcctgtgattcccgcattgtcttaccctgagcaacttcacatct 54198
chimp54546-135457.revcompl.Pan.troglodytes.clone.rp43-41g5.GenBank:AC099533.36       GGACAACATGAGGGATGCGGACAGAGTGCGCTTTCTCTGGAAAGGAGGAGTTTAATACCA 63970
human.FAM30B-LOC105372935-GGT2.NCBI.                                                 gtccacaaggtgccaagcctatggttcccgcattctcttaccctcagcaacttcatgtct 53192
human.FAM230E-LOC105377182-GGT3P.NCBI.                                               gtccacaaggtgccaagcctatgattcccgcattctcttaccctcagcaacttcatgtct 51844
human.FAM230J-LOC105372942-GGTLC5P.NCBI.rev.compl                                    ------------------------------------------------------------ 46364
human.FAM230A.ncRNA.18487127.18500594-GGTLC3.18516335.18518165.rev.compl.REF         ------------------------------------------------------------ 31039

chimp.LOC112206744-LOC107973052-GGT2.revcompl                                        acagatgctgagtttctcaatgagtattaaaaacaaatgaaagattggtcgggcacagtg 54258
chimp54546-135457.revcompl.Pan.troglodytes.clone.rp43-41g5.GenBank:AC099533.36       CTGCTGGGAGCTTTAAAAAATGGAAA---------------------------------- 63996
human.FAM30B-LOC105372935-GGT2.NCBI.                                                 acagatgctgagtttctcaatgagtattaaaaacaaatgaaagattggtcgggcacagtg 53252
human.FAM230E-LOC105377182-GGT3P.NCBI.                                               acagatgctgagtttctcagtgagtattaaaaacaaatgaaagattggtcgggcacagtg 51904
human.FAM230J-LOC105372942-GGTLC5P.NCBI.rev.compl                                    ------------------------------------------------------------ 46364
human.FAM230A.ncRNA.18487127.18500594-GGTLC3.18516335.18518165.rev.compl.REF         ------------------------------------------------------------ 31039

chimp.LOC112206744-LOC107973052-GGT2.revcompl                                        gctcacgcctgtaattccagcactttgggaggctgaggcaggggatcacgaggtcagga 54318
chimp54546-135457.revcompl.Pan.troglodytes.clone.rp43-41g5.GenBank:AC099533.36       --CCCTGCCTGTGATATCAGCACTTTGAAAGGCTGAGGCAGGAGGATCACTTGAGCCCAT 64054
human.FAM30B-LOC105372935-GGT2.NCBI.                                                 gctcacgcctgtaattccagcactttgggaggctgaggcaggggatcacgaggtcagga 53312
human.FAM230E-LOC105377182-GGT3P.NCBI.                                               gctcacgcctgtaattccagcactttgggaggctgaggcaggggatcacgaggtcagga 51964
human.FAM230J-LOC105372942-GGTLC5P.NCBI.rev.compl                                    ------------------------------------------------------------ 46364
human.FAM230A.ncRNA.18487127.18500594-GGTLC3.18516335.18518165.rev.compl.REF         ------------------------------------------------------------ 31039

chimp.LOC112206744-LOC107973052-GGT2.revcompl                                        gatcgagggaccagcctgggcaacatagtgaaaacccgtctctactaaaaaatacaaaaa 54378
chimp54546-135457.revcompl.Pan.troglodytes.clone.rp43-41g5.GenBank:AC099533.36       GAGTTTAAGACCATCCTGGGAAATATAGTGAGACCTTGTCTGTATTAAAAAAAAAAAAAA 64114
human.FAM30B-LOC105372935-GGT2.NCBI.                                                 gatcgagggaccagcctgggcaacatagtgaaaacccgtctctactaaaaaatacaaaaa 53372
human.FAM230E-LOC105377182-GGT3P.NCBI.                                               gatcgagggaccagcctgggcaacacagtgaaaacccgtctctactaaaaaatacaaaaa 52024
human.FAM230J-LOC105372942-GGTLC5P.NCBI.rev.compl                                    ------------------------------------------------------------ 46364
human.FAM230A.ncRNA.18487127.18500594-GGTLC3.18516335.18518165.rev.compl.REF         ------------------------------------------------------------ 31039

chimp.LOC112206744-LOC107973052-GGT2.revcompl                                        att--------agccgggtgtggtggcaggcgcctgtagtcccagctactcaggaggctg 54430
chimp54546-135457.revcompl.Pan.troglodytes.clone.rp43-41g5.GenBank:AC099533.36       TTTAAAAAGGCAGCCAAGCATGGTGGCACATACCTGCAGTCCCAGCTGCTTGAGAGGCTG 64174
human.FAM30B-LOC105372935-GGT2.NCBI.                                                 att--------agccgggcgcggtggcgggcgcctgtagtcccagctactcgggaggctg 53424
human.FAM230E-LOC105377182-GGT3P.NCBI.                                               att--------agccgggtgtggtggcgggcgcctgtagtcccagctactcgggaggctg 52076
human.FAM230J-LOC105372942-GGTLC5P.NCBI.rev.compl                                    ------------------------------------------------------------ 46364
human.FAM230A.ncRNA.18487127.18500594-GGTLC3.18516335.18518165.rev.compl.REF         ------------------------------------------------------------ 31039

chimp.LOC112206744-LOC107973052-GGT2.revcompl                                        aggcaggagaatggcgtgaactcaggaggcagagcttgcagtgagccgagatcgcaccac 54490
chimp54546-135457.revcompl.Pan.troglodytes.clone.rp43-41g5.GenBank:AC099533.36       AGGTGGGAGCATTGCTCGAGCCTGGAAGGTGGAGGCTGCTGTGAGCCACGATCATGCCAT 64234
human.FAM30B-LOC105372935-GGT2.NCBI.                                                 aggcaggagaatggcgcgaactcaggaggcagagcttgcagtgagccgagatcgcaccac 53484
human.FAM230E-LOC105377182-GGT3P.NCBI.                                               aggcaggagaatggcgtgaactcaggaggcggaacttgcagtgagctgagatcgcaccac 52136
human.FAM230J-LOC105372942-GGTLC5P.NCBI.rev.compl                                    ------------------------------------------------------------ 46364
human.FAM230A.ncRNA.18487127.18500594-GGTLC3.18516335.18518165.rev.compl.REF         ------------------------------------------------------------ 31039

chimp.LOC112206744-LOC107973052-GGT2.revcompl                                        tgcactccaacctgggcgacagagtaagactccgtctcaaaaaaaaaaaaaagaaaaaaa 54550
chimp54546-135457.revcompl.Pan.troglodytes.clone.rp43-41g5.GenBank:AC099533.36       TGTACTCCAGCCTGCGCAACAGAGCGAGAGACCCTGGCTCTAACTAACTAACCAAGTAAC 64294
human.FAM30B-LOC105372935-GGT2.NCBI.                                                 tgcactccaacctgggcgacagagtgagactctgtctcaaaaaaaaaaaaaagaaaaaaaa 53544
human.FAM230E-LOC105377182-GGT3P.NCBI.                                               tgcactccaacctgggcgacagagtgagactccgtctcaaaaaaaaaaaaaagaaaaaaaa 52196
human.FAM230J-LOC105372942-GGTLC5P.NCBI.rev.compl                                    ------------------------------------------------------------ 46364
human.FAM230A.ncRNA.18487127.18500594-GGTLC3.18516335.18518165.rev.compl.REF         ------------------------------------------------------------ 31039

chimp.LOC112206744-LOC107973052-GGT2.revcompl                                        aatcaaagattgagtatgttgcagaagactccaaagggcaccacccaggacccccacctg 54610
chimp54546-135457.revcompl.Pan.troglodytes.clone.rp43-41g5.GenBank:AC099533.36       TGGAAACATAGAAACATGTT---------------------------------------- 64314
human.FAM30B-LOC105372935-GGT2.NCBI.                                                 aatcaaagattgagtatgttgcagaagactccaaagggcaccacccaggacccccacctg 53604
human.FAM230E-LOC105377182-GGT3P.NCBI.                                               aatcaaagattgagtatgttgcagaagactccaaagggcaccacccaggacccccacctg 52256
human.FAM230J-LOC105372942-GGTLC5P.NCBI.rev.compl                                    ------------------------------------------------------------ 46364
human.FAM230A.ncRNA.18487127.18500594-GGTLC3.18516335.18518165.rev.compl.REF         ------------------------------------------------------------ 31039

chimp.LOC112206744-LOC107973052-GGT2.revcompl                                        aagtctaagacctgctgtggtaagtgtgtcctgcccctccatcctccaac---ttttttt 54667
chimp54546-135457.revcompl.Pan.troglodytes.clone.rp43-41g5.GenBank:AC099533.36       ------------------------------------------------------------ 64314
human.FAM30B-LOC105372935-GGT2.NCBI.                                                 aagtctaagacctgctatggtgagtgtgtcctgcccctccatcctccaactttttttttt 53664
human.FAM230E-LOC105377182-GGT3P.NCBI.                                               aagtctaagacctgctatggtgagtgtgtcctgcccctccatcctccaactttttttttt 52316
human.FAM230J-LOC105372942-GGTLC5P.NCBI.rev.compl                                    ------------------------------------------------------------ 46364
human.FAM230A.ncRNA.18487127.18500594-GGTLC3.18516335.18518165.rev.compl.REF         ------------------------------------------------------------ 31039

chimp.LOC112206744-LOC107973052-GGT2.revcompl                                        tttttttttttttgagacggagcttcgttcttgttgcccaggctggagtgcagtggcatg 54727
chimp54546-135457.revcompl.Pan.troglodytes.clone.rp43-41g5.GenBank:AC099533.36       ------------------------------------------------------------ 64314
human.FAM30B-LOC105372935-GGT2.NCBI.                                                 tttttttttttttgagacggagcttcgttcttgttgcccaggctggagtgcagtggcatg 53724
human.FAM230E-LOC105377182-GGT3P.NCBI.                                               tttttttttttttgagacggagcttcgttcttgttgcccaggctggagtgcagtggcatg 52376
human.FAM230J-LOC105372942-GGTLC5P.NCBI.rev.compl                                    ------------------------------------------------------------ 46364
human.FAM230A.ncRNA.18487127.18500594-GGTLC3.18516335.18518165.rev.compl.REF         ------------------------------------------------------------ 31039

chimp.LOC112206744-LOC107973052-GGT2.revcompl                                        atctcggctcactgcaatctccacctcgtgggttcaagcgattctcctgcctcagcctcc 54787
chimp54546-135457.revcompl.Pan.troglodytes.clone.rp43-41g5.GenBank:AC099533.36       ------------------------------------------------------------ 64314
human.FAM30B-LOC105372935-GGT2.NCBI.                                                 atctcggctcactgcaatctctacctcgtgggttcaagcgattctcctgccttagcctcc 53784
human.FAM230E-LOC105377182-GGT3P.NCBI.                                               atctcggctcacagcaatctctacctcgtgggttcaagcgattctcctgccttagcctcc 52436
human.FAM230J-LOC105372942-GGTLC5P.NCBI.rev.compl                                    ------------------------------------------------------------ 46364
human.FAM230A.ncRNA.18487127.18500594-GGTLC3.18516335.18518165.rev.compl.REF         ------------------------------------------------------------ 31039
```

```
chimp.LOC112206744-LOC107973052-GGT2.revcompl                                    tgagtagctgggatattacaggcctgtgccaccacgcccaactaattattatacttttag 54847
chimp54546-135457.revcompl.Pan.troglodytes.clone.rp43-41g5.GenBank:AC099533.36   ---------------------------------AAGCAGGAGACATTCTTCCCTTTCCTT 64341
human.FAM30B-LOC105372935-GGT2.NCBI.                                             tgagtagctgggatattacaggcctgtgccaccacgcccgactaattattgtacttttag 53844
human.FAM230E-LOC105377182-GGT3P.NCBI.                                           tgagtagctgggatattacaggcctgtgccaccacgcccgactaattattgtacttttag 52496
human.FAM230J-LOC105372942-GGTLC5P.NCBI.rev.compl                                ------------------------------------------------------------ 46364
human.FAM230A.ncRNA.18487127.18500594-GGTLC3.18516335.18518165.rev.compl.REF     ------------------------------------------------------------ 31039

chimp.LOC112206744-LOC107973052-GGT2.revcompl                                    tagagaaagggtttcactatgttggccaggctggtcttgaactc-ctgacctaggtgatc 54906
chimp54546-135457.revcompl.Pan.troglodytes.clone.rp43-41g5.GenBank:AC099533.36   CAAAGAACAGCCATCACTG----GGCCCCTCTCCCCTTGAAAACTCAGAACTGGCAGAAG 64397
human.FAM30B-LOC105372935-GGT2.NCBI.                                             tagagaaagggtttcactatgttggccaggctggtcttggactc-ctgacctaggtgatc 53903
human.FAM230E-LOC105377182-GGT3P.NCBI.                                           tagagaaagggtttcactatgttggccaggctggtcttggactc-ctgacctaggtgatc 52555
human.FAM230J-LOC105372942-GGTLC5P.NCBI.rev.compl                                ------------------------------------------------------------ 46364
human.FAM230A.ncRNA.18487127.18500594-GGTLC3.18516335.18518165.rev.compl.REF     ------------------------------------------------------------ 31039

chimp.LOC112206744-LOC107973052-GGT2.revcompl                                    catctgcctcagcctcccaaagtgctgggattacaggcgtgagcctgtgaaaaaaaggcc 54966
chimp54546-135457.revcompl.Pan.troglodytes.clone.rp43-41g5.GenBank:AC099533.36   CAGCTAAC-AAGCAACCCAGAGTCCAGGTAAGAAAA------------------------ 64432
human.FAM30B-LOC105372935-GGT2.NCBI.                                             catctgcctcagcctcccaaagtgctgggattacaggcgtgagcctgtgaaaaaaaggcc 53963
human.FAM230E-LOC105377182-GGT3P.NCBI.                                           catctgcctcagcctcccaaagtgctgggattacaggcgtgagcctgtgaaaaaaaggcc 52615
human.FAM230J-LOC105372942-GGTLC5P.NCBI.rev.compl                                ------------------------------------------------------------ 46364
human.FAM230A.ncRNA.18487127.18500594-GGTLC3.18516335.18518165.rev.compl.REF     ------------------------------------------------------------ 31039

chimp.LOC112206744-LOC107973052-GGT2.revcompl                                    cagccttttttttttttttttttttgacagcgtctcactttgttgcccaagctagagt 55026
chimp54546-135457.revcompl.Pan.troglodytes.clone.rp43-41g5.GenBank:AC099533.36   ------------------------------------------------------------ 64432
human.FAM30B-LOC105372935-GGT2.NCBI.                                             cagcc----ttttttttttttttttttgacagggtctcactttgttgcccaagctagagt 54019
human.FAM230E-LOC105377182-GGT3P.NCBI.                                           cagcc---tttttttttttttttttttgacagggtctcactttgttgcccaagctagagt 52672
human.FAM230J-LOC105372942-GGTLC5P.NCBI.rev.compl                                ------------------------------------------------------------ 46364
human.FAM230A.ncRNA.18487127.18500594-GGTLC3.18516335.18518165.rev.compl.REF     ------------------------------------------------------------ 31039

chimp.LOC112206744-LOC107973052-GGT2.revcompl                                    gtagtggtataatcatggctgactgcagcctcaacctcctgggctcaagtgatcctccca 55086
chimp54546-135457.revcompl.Pan.troglodytes.clone.rp43-41g5.GenBank:AC099533.36   --------------------GACAGAAAGAGAAGCTGTCCAGACATCAGGCAGCTGCCCC 64472
human.FAM30B-LOC105372935-GGT2.NCBI.                                             gtagtggtataatcatggctgactgcagcctcaacctcctgggctcaagtgatcctccca 54079
human.FAM230E-LOC105377182-GGT3P.NCBI.                                           gtagtggtataatcatggctgactgcagcctcaacctcctgggctcaagtgatcctccca 52732
human.FAM230J-LOC105372942-GGTLC5P.NCBI.rev.compl                                ------------------------------------------------------------ 46364
human.FAM230A.ncRNA.18487127.18500594-GGTLC3.18516335.18518165.rev.compl.REF     ------------------------------------------------------------ 31039

chimp.LOC112206744-LOC107973052-GGT2.revcompl                                    ccttagcctcccgagtagctgggaccataagcacacaccgccatacctagctaa-ttttt 55145
chimp54546-135457.revcompl.Pan.troglodytes.clone.rp43-41g5.GenBank:AC099533.36   TCTGAGCCCGCCTGGAACCATGGCCCACATGCCAGCCCATTAATAAAATAATAA------ 64526
human.FAM30B-LOC105372935-GGT2.NCBI.                                             ccttagcctcccgagtaactgggaccataagcacacaccgccatacct-gctaatttttt 54138
human.FAM230E-LOC105377182-GGT3P.NCBI.                                           ccttagcctcccgagtaactgggaccataagcacacaccgccatacctagctaatttttt 52792
human.FAM230J-LOC105372942-GGTLC5P.NCBI.rev.compl                                ------------------------------------------------------------ 46364
human.FAM230A.ncRNA.18487127.18500594-GGTLC3.18516335.18518165.rev.compl.REF     ------------------------------------------------------------ 31039

chimp.LOC112206744-LOC107973052-GGT2.revcompl                                    tttccatttttgtagagatggagtcttgctatgttgtccaggcaggtctcctgggctca 55205
chimp54546-135457.revcompl.Pan.troglodytes.clone.rp43-41g5.GenBank:AC099533.36   ------------------------------------------------------------ 64526
human.FAM30B-LOC105372935-GGT2.NCBI.                                             tttccatttttgtagagatggagtcttgctatgttgtccaggcaggtctcctgggctca 54198
human.FAM230E-LOC105377182-GGT3P.NCBI.                                           tttccatttttgtagagatggagtcttgctatgttgtccaggcaggtctcctgggctca 52852
human.FAM230J-LOC105372942-GGTLC5P.NCBI.rev.compl                                ------------------------------------------------------------ 46364
human.FAM230A.ncRNA.18487127.18500594-GGTLC3.18516335.18518165.rev.compl.REF     ------------------------------------------------------------ 31039

chimp.LOC112206744-LOC107973052-GGT2.revcompl                                    tgcgctcctcctgacttggcctcctaaagtgctaggattagaggcgtgagttgttgagac 55265
chimp54546-135457.revcompl.Pan.troglodytes.clone.rp43-41g5.GenBank:AC099533.36   ------------------------------------------------------------ 64526
human.FAM30B-LOC105372935-GGT2.NCBI.                                             tgcgctcctcctgacttggcctcctaaagtgctaggattagaggcgtgagttgttgagac 54258
human.FAM230E-LOC105377182-GGT3P.NCBI.                                           tgcgctcctcctgacttggcctcctaaagtgctaggattagaggtgtgagttgttgagac 52912
human.FAM230J-LOC105372942-GGTLC5P.NCBI.rev.compl                                ------------------------------------------------------------ 46364
human.FAM230A.ncRNA.18487127.18500594-GGTLC3.18516335.18518165.rev.compl.REF     ------------------------------------------------------------ 31039

chimp.LOC112206744-LOC107973052-GGT2.revcompl                                    cctcccatcctccaactttatctcacaatctattgtgcctcctttggggacagacagtg 55325
chimp54546-135457.revcompl.Pan.troglodytes.clone.rp43-41g5.GenBank:AC099533.36   ------------------------------------------------------------ 64526
human.FAM30B-LOC105372935-GGT2.NCBI.                                             cctcccatcctccaactttatctcacaatctattgtgcctcctttggggacagacagtg 54318
human.FAM230E-LOC105377182-GGT3P.NCBI.                                           cctcccatcctccaactttatctcacactctattgtgcctcctttggggacagacagtg 52972
human.FAM230J-LOC105372942-GGTLC5P.NCBI.rev.compl                                ------------------------------------------------------------ 46364
human.FAM230A.ncRNA.18487127.18500594-GGTLC3.18516335.18518165.rev.compl.REF     ------------------------------------------------------------ 31039

chimp.LOC112206744-LOC107973052-GGT2.revcompl                                    gcttcctggatggacagtggcttcccttcaggtacctggggaatttgggggcctcctctc 55385
chimp54546-135457.revcompl.Pan.troglodytes.clone.rp43-41g5.GenBank:AC099533.36   ------------------------------------------------------------ 64526
human.FAM30B-LOC105372935-GGT2.NCBI.                                             gcttcctggatggacagtggcttcccctcaggtacctggggaatttgggggcctcctccc 54378
human.FAM230E-LOC105377182-GGT3P.NCBI.                                           gcttcctggatggacagtggcttcccctcaggtacctggggaatttgggggcctcctccc 53032
human.FAM230J-LOC105372942-GGTLC5P.NCBI.rev.compl                                ------------------------------------------------------------ 46364
human.FAM230A.ncRNA.18487127.18500594-GGTLC3.18516335.18518165.rev.compl.REF     ------------------------------------------------------------ 31039

chimp.LOC112206744-LOC107973052-GGT2.revcompl                                    cacttaagaccagattagaaaagagggactccacctcacattctagagcaccatccccac 55445
chimp54546-135457.revcompl.Pan.troglodytes.clone.rp43-41g5.GenBank:AC099533.36   ------------------------------------------------------------ 64526
human.FAM30B-LOC105372935-GGT2.NCBI.                                             cacttaagaccagattagaaaagagagactccacctcacattctagagcgccatccccac 54438
human.FAM230E-LOC105377182-GGT3P.NCBI.                                           cacttaagaccagattagaaaagagagactccacctcacattctagagcgccatccccac 53092
human.FAM230J-LOC105372942-GGTLC5P.NCBI.rev.compl                                ------------------------------------------------------------ 46364
human.FAM230A.ncRNA.18487127.18500594-GGTLC3.18516335.18518165.rev.compl.REF     ------------------------------------------------------------ 31039

chimp.LOC112206744-LOC107973052-GGT2.revcompl                                    aaatgaacaaatgagtgaatgggatgcctgttgaaaaagcaggatatagacagcctgggt 55505
chimp54546-135457.revcompl.Pan.troglodytes.clone.rp43-41g5.GenBank:AC099533.36   ------------------------------------------------------------ 64526
human.FAM30B-LOC105372935-GGT2.NCBI.                                             aaatgaacaaatgagtgaatgggatgcctgttgaaaaggcaggatatagacagcctgggt 54498
human.FAM230E-LOC105377182-GGT3P.NCBI.                                           aaatgaacaaatgagtgaatgggatgcctgttgaaaaggcaggatatagacagcctgggt 53152
human.FAM230J-LOC105372942-GGTLC5P.NCBI.rev.compl                                ------------------------------------------------------------ 46364
human.FAM230A.ncRNA.18487127.18500594-GGTLC3.18516335.18518165.rev.compl.REF     ------------------------------------------------------------ 31039

chimp.LOC112206744-LOC107973052-GGT2.revcompl                                    tcaattttagctttaccacctcccagctgtgtgacctcaactgatttgcatgacctgtct 55565
chimp54546-135457.revcompl.Pan.troglodytes.clone.rp43-41g5.GenBank:AC099533.36   ------------------------------------------------------------ 64526
human.FAM30B-LOC105372935-GGT2.NCBI.                                             tcaattttagcttcaccacctcccagctgtgtgacctcagctgatttgcatgacctgtct 54558
human.FAM230E-LOC105377182-GGT3P.NCBI.                                           tcaattttagcttcaccacctcccagctgtgtgacctcagctgatttgcatgacctgtct 53212
human.FAM230J-LOC105372942-GGTLC5P.NCBI.rev.compl                                ------------------------------------------------------------ 46364
human.FAM230A.ncRNA.18487127.18500594-GGTLC3.18516335.18518165.rev.compl.REF     ------------------------------------------------------------ 31039

chimp.LOC112206744-LOC107973052-GGT2.revcompl                                    gagcctcagcatccccaccctgtaaaatgggaatccacacagcatcccctagcccaaagg 55625
chimp54546-135457.revcompl.Pan.troglodytes.clone.rp43-41g5.GenBank:AC099533.36   ------------------------------------------------------------ 64526
human.FAM30B-LOC105372935-GGT2.NCBI.                                             gagcctcagcatccccaccctgtaaaatgggaatccacacagcatcccctagcccaaagg 54618
human.FAM230E-LOC105377182-GGT3P.NCBI.                                           gagcctcagcatccccaccctgtaaaatgggaatccacacagcatccccttgcccaaagg 53272
human.FAM230J-LOC105372942-GGTLC5P.NCBI.rev.compl                                ------------------------------------------------------------ 46364
human.FAM230A.ncRNA.18487127.18500594-GGTLC3.18516335.18518165.rev.compl.REF     ------------------------------------------------------------ 31039

chimp.LOC112206744-LOC107973052-GGT2.revcompl                                    agcagggagggttgtgagaggctcgtgggtgaaaagcacagagcagagcgtgggccccag 55685
chimp54546-135457.revcompl.Pan.troglodytes.clone.rp43-41g5.GenBank:AC099533.36   ------------------------------------------------------------ 64526
human.FAM30B-LOC105372935-GGT2.NCBI.                                             agcagggagggttgtgagaggctcgtgggtgaaaagcacagagcagagcatgggccccag 54678
human.FAM230E-LOC105377182-GGT3P.NCBI.                                           agcagggagggttgtgagaggctcgtgggtgaaaagcacagagcagagcatgggccccag 53332
human.FAM230J-LOC105372942-GGTLC5P.NCBI.rev.compl                                ------------------------------------------------------------ 46364
human.FAM230A.ncRNA.18487127.18500594-GGTLC3.18516335.18518165.rev.compl.REF     ------------------------------------------------------------ 31039

chimp.LOC112206744-LOC107973052-GGT2.revcompl                                    tgagccctggtccatgaggtctgctagcataataattattctttccatgtgctgcacaga 55745
chimp54546-135457.revcompl.Pan.troglodytes.clone.rp43-41g5.GenBank:AC099533.36   ------------------------------------------------------------ 64526
human.FAM30B-LOC105372935-GGT2.NCBI.                                             tgagccctgatccatgaggtctgctagcataataattattctttccatgtgctgcacaga 54738
human.FAM230E-LOC105377182-GGT3P.NCBI.                                           tgagccctgatccatgaggtctgctagcataataattattctttccatgtgctgcacaga 53392
human.FAM230J-LOC105372942-GGTLC5P.NCBI.rev.compl                                ------------------------------------------------------------ 46364
human.FAM230A.ncRNA.18487127.18500594-GGTLC3.18516335.18518165.rev.compl.REF     ------------------------------------------------------------ 31039

chimp.LOC112206744-LOC107973052-GGT2.revcompl                                    gtggccccggaggccttagcagaaataacagaagctcccggccctttaccgtggtgatga 55805
chimp54546-135457.revcompl.Pan.troglodytes.clone.rp43-41g5.GenBank:AC099533.36   ------------------------------------------------------------ 64526
human.FAM30B-LOC105372935-GGT2.NCBI.                                             gtggccccggaggccttagcagaaataacagaagctcccggccctttaccgtggtgatga 54798
human.FAM230E-LOC105377182-GGT3P.NCBI.                                           gtggccccggaggccttagcagaaataacagaagctcccggccctttaccatggtgatga 53452
human.FAM230J-LOC105372942-GGTLC5P.NCBI.rev.compl                                ------------------------------------------------------------ 46364
human.FAM230A.ncRNA.18487127.18500594-GGTLC3.18516335.18518165.rev.compl.REF     ------------------------------------------------------------ 31039

chimp.LOC112206744-LOC107973052-GGT2.revcompl                                    tggtcctgaccactcattgtgggagggtgctatggggcaaggaagggatggggggtgcta 55865
chimp54546-135457.revcompl.Pan.troglodytes.clone.rp43-41g5.GenBank:AC099533.36   ------------------------------------------------------------ 64526
human.FAM30B-LOC105372935-GGT2.NCBI.                                             tggtcctgaccactcattgtgggagggtgctatggggccaggaagggatggggggtgaca 54858
human.FAM230E-LOC105377182-GGT3P.NCBI.                                           tggtcctgaccactcactgtgggagggtgctatggggacaggaagggatggggggtgcta 53512
human.FAM230J-LOC105372942-GGTLC5P.NCBI.rev.compl                                ------------------------------------------------------------ 46364
human.FAM230A.ncRNA.18487127.18500594-GGTLC3.18516335.18518165.rev.compl.REF     ------------------------------------------------------------ 31039
```

```
chimp.LOC112206744-LOC107973052-GGT2.revcompl                                     gaactgcccctgaaccctgacgggagcaggctcctgtgggcaaggccccttcccggtggc 55925
chimp54546-135457.revcompl.Pan.troglodytes.clone.rp43-41g5.GenBank:AC099533.36    ----------TGAAAACTACTCACATCTAACAAGAGATTAGACATCCAGATAGAGGAGGC 64576
human.FAM30B-LOC105372935-GGT2.NCBI.                                              gaactgcccctgaaccctgacgggagcaggcttctgtgggcaaggccccttcccggtggc 54918
human.FAM230E-LOC105377182-GGT3P.NCBI.                                            gaactgcccctgaaccctgacgggagcaggctcctgtgggcaaggccccttcccggtggc 53572
human.FAM230J-LOC105372942-GGTLC5P.NCBI.rev.compl                                 ------------------------------------------------------------ 46364
human.FAM230A.ncRNA.18487127.18500594-GGTLC3.18516335.18518165.rev.compl.REF      ------------------------------------------------------------ 31039

chimp.LOC112206744-LOC107973052-GGT2.revcompl                                     tcagccagctctgcac----ccatgccccaagtctgcagcatggcttacccttctgggac 55981
chimp54546-135457.revcompl.Pan.troglodytes.clone.rp43-41g5.GenBank:AC099533.36    TCAAATATCCCCAAACAATGCAATGCAAAGGGTCTTTGCCGTGGCAAACTATAATCAGAC 64636
human.FAM30B-LOC105372935-GGT2.NCBI.                                              tcagccagctctgcac----ccatgccccaagtctgcagcatggcttacccttctgggac 54974
human.FAM230E-LOC105377182-GGT3P.NCBI.                                            tcagccagctctgcac----ccacgccccaagtctgccgcatggcttacccttctgggac 53628
human.FAM230J-LOC105372942-GGTLC5P.NCBI.rev.compl                                 ------------------------------------------------------------ 46364
human.FAM230A.ncRNA.18487127.18500594-GGTLC3.18516335.18518165.rev.compl.REF      ------------------------------------------------------------ 31039

chimp.LOC112206744-LOC107973052-GGT2.revcompl                                     tgctccgagctgttg----aacatgctggcaaaggccagcctggggggccacctcgcggc 56037
chimp54546-135457.revcompl.Pan.troglodytes.clone.rp43-41g5.GenBank:AC099533.36    TATTTAAGGTCAATGTGAAAATGCGAATTCTAAAACCACCAAGAGAAAAGAATCTAGTCA 64696
human.FAM30B-LOC105372935-GGT2.NCBI.                                              tgctccgagctgttg----aacatgctggcaaaggccagcctggggggccacctcgcggc 55030
human.FAM230E-LOC105377182-GGT3P.NCBI.                                            tgctccgagctgttg----aacatgctggcaaaggccagcctggggggccacctcgcggc 53684
human.FAM230J-LOC105372942-GGTLC5P.NCBI.rev.compl                                 ------------------------------------------------------------ 46364
human.FAM230A.ncRNA.18487127.18500594-GGTLC3.18516335.18518165.rev.compl.REF      ------------------------------------------------------------ 31039

chimp.LOC112206744-LOC107973052-GGT2.revcompl                                     gttgatgacctcagcttttcctagaaggagaagcaggtaggcaggcccacccacccaaac 56097
chimp54546-135457.revcompl.Pan.troglodytes.clone.rp43-41g5.GenBank:AC099533.36    CCTATAAAGCAAACCCCATCAGATG---AAGAGCAGATTTCACAGCCCAGCAAATAGAAA 64753
human.FAM30B-LOC105372935-GGT2.NCBI.                                              attgatgacctcagcttttcctaga---agaagcaggtaggcaggcccacccaccaaaac 55087
human.FAM230E-LOC105377182-GGT3P.NCBI.                                            attgatgacctcagcttttcctaga---agaagcaggtaggcaggcccacccaccaaaac 53741
human.FAM230J-LOC105372942-GGTLC5P.NCBI.rev.compl                                 ------------------------------------------------------------ 46364
human.FAM230A.ncRNA.18487127.18500594-GGTLC3.18516335.18518165.rev.compl.REF      ------------------------------------------------------------ 31039

chimp.LOC112206744-LOC107973052-GGT2.revcompl                                     ------cctttatgccacgtgagcctgggggccacccagctgtgcctcggcccaacccac 56151
chimp54546-135457.revcompl.Pan.troglodytes.clone.rp43-41g5.GenBank:AC099533.36    ACATGTGCTTAAAGGCCTAGGAGGCAGAGAGGTACAGGGCTGGAGACAGGCAAGGCCAGC 64813
human.FAM30B-LOC105372935-GGT2.NCBI.                                              ------cctttatgccacgtgagcctgggggccacccagctgtgcctcggcccaacccac 55141
human.FAM230E-LOC105377182-GGT3P.NCBI.                                            ------cctttatgccacgtgagcctgggggccacccagctgtgcctcggcccaacccac 53795
human.FAM230J-LOC105372942-GGTLC5P.NCBI.rev.compl                                 ------------------------------------------------------------ 46364
human.FAM230A.ncRNA.18487127.18500594-GGTLC3.18516335.18518165.rev.compl.REF      ------------------------------------------------------------ 31039

chimp.LOC112206744-LOC107973052-GGT2.revcompl                                     accccctgcccctctccctctcctcttccgaggcactcatgagtggtgctgttgtagatg 56211
chimp54546-135457.revcompl.Pan.troglodytes.clone.rp43-41g5.GenBank:AC099533.36    ATTCCCCTCAAGGCAACGGCCAATCCTGGAAGAGGAGGCTGAGAGACAGAGCAGT----- 64868
human.FAM30B-LOC105372935-GGT2.NCBI.                                              accccctgcccctctccctctcctcttctgaggcactcatgagtggtgctgttgtagatg 55201
human.FAM230E-LOC105377182-GGT3P.NCBI.                                            accccctgcccctctccctctccgcttccgaggcactcatgagtggtgctgttgtagatg 53855
human.FAM230J-LOC105372942-GGTLC5P.NCBI.rev.compl                                 ------------------------------------------------------------ 46364
human.FAM230A.ncRNA.18487127.18500594-GGTLC3.18516335.18518165.rev.compl.REF      ------------------------------------------------------------ 31039

chimp.LOC112206744-LOC107973052-GGT2.revcompl                                     gtgaggaagaggccacccccgatgcccatgctgtgggcattcatgagccccacacacaac 56271
chimp54546-135457.revcompl.Pan.troglodytes.clone.rp43-41g5.GenBank:AC099533.36    ------------------------------------------------------------ 64868
human.FAM30B-LOC105372935-GGT2.NCBI.                                              gtgaggaagaggccaaccccgatgcccatgctgtgggcattcatgagccccacacacaac 55261
human.FAM230E-LOC105377182-GGT3P.NCBI.                                            gtgaggaagaggccaaccccgatgcccatgctgtgggcattcatgagccccacacacaac 53915
human.FAM230J-LOC105372942-GGTLC5P.NCBI.rev.compl                                 ------------------------------------------------------------ 46364
human.FAM230A.ncRNA.18487127.18500594-GGTLC3.18516335.18518165.rev.compl.REF      ------------------------------------------------------------ 31039

chimp.LOC112206744-LOC107973052-GGT2.revcompl                                     agggctgcaatggctgcatccaccgcagagccaccgtcccgcagtgcgtccctgccatgt 56331
chimp54546-135457.revcompl.Pan.troglodytes.clone.rp43-41g5.GenBank:AC099533.36    ------------------------------------------------------------ 64868
human.FAM30B-LOC105372935-GGT2.NCBI.                                              agggctgcaatggctgcatccactgcagagccaccgtcccgcagtgtgtccctgccatgt 55321
human.FAM230E-LOC105377182-GGT3P.NCBI.                                            agggctgcaatggctgcatccactgcagagccaccgtcccgcagtgtgtccctgccatgt 53975
human.FAM230J-LOC105372942-GGTLC5P.NCBI.rev.compl                                 ------------------------------------------------------------ 46364
human.FAM230A.ncRNA.18487127.18500594-GGTLC3.18516335.18518165.rev.compl.REF      ------------------------------------------------------------ 31039

chimp.LOC112206744-LOC107973052-GGT2.revcompl                                     ggcacataaaggcatgagaacctgcaggcttccaccctggccccgcatacacaccctgct 56391
chimp54546-135457.revcompl.Pan.troglodytes.clone.rp43-41g5.GenBank:AC099533.36    ------------------------------------------------------------ 64868
human.FAM30B-LOC105372935-GGT2.NCBI.                                              ggcacataaaggcatgagaacctgcaggcttccaccctggccccgcatacacaccctgct 55381
human.FAM230E-LOC105377182-GGT3P.NCBI.                                            ggcacataaaggcatgagaacctgcaggcttccaccctggccccgcatacacaccctgct 54035
human.FAM230J-LOC105372942-GGTLC5P.NCBI.rev.compl                                 ------------------------------------------------------------ 46364
human.FAM230A.ncRNA.18487127.18500594-GGTLC3.18516335.18518165.rev.compl.REF      ------------------------------------------------------------ 31039

chimp.LOC112206744-LOC107973052-GGT2.revcompl                                     gcccacctgcccaaaggaggatggaagagaagtccattcgagttttggggtttttgtttt 56451
chimp54546-135457.revcompl.Pan.troglodytes.clone.rp43-41g5.GenBank:AC099533.36    ------------------------------------------------------------ 64868
human.FAM30B-LOC105372935-GGT2.NCBI.                                              gcccacctgcccaaaggaggatggaagagaagtccattcgagttttggggtttttgtttt 55441
human.FAM230E-LOC105377182-GGT3P.NCBI.                                            gcccacctgcccaaaggaggatggaagagaagtccattcgagttttggggtttttgtttt 54095
human.FAM230J-LOC105372942-GGTLC5P.NCBI.rev.compl                                 ------------------------------------------------------------ 46364
human.FAM230A.ncRNA.18487127.18500594-GGTLC3.18516335.18518165.rev.compl.REF      ------------------------------------------------------------ 31039

chimp.LOC112206744-LOC107973052-GGT2.revcompl                                     tagtttctttctcttttttttgagatggagtcttgctctatt-gccaggctggagtgcag 56510
chimp54546-135457.revcompl.Pan.troglodytes.clone.rp43-41g5.GenBank:AC099533.36    --GATTCTTTTTTTTTTTTTTGAGACGGAGTCTCACTCTGTCACCCAGGCTGGAGTGCAG 64926
human.FAM30B-LOC105372935-GGT2.NCBI.                                              tagtttctttctcttttttttgagatggagtcttgctctatt-gccaggctggagtgcag 55500
human.FAM230E-LOC105377182-GGT3P.NCBI.                                            tagtttctttctcttttttttgagatggagtcttgctctatt-gccaggctggagtgcag 54154
human.FAM230J-LOC105372942-GGTLC5P.NCBI.rev.compl                                 ------------------------------------------------------------ 46364
human.FAM230A.ncRNA.18487127.18500594-GGTLC3.18516335.18518165.rev.compl.REF      ------------------------------------------------------------ 31039

chimp.LOC112206744-LOC107973052-GGT2.revcompl                                     tggcacgatctcagctcactgcaacctctgcctcccgggttcaagccattctcctgcctc 56570
chimp54546-135457.revcompl.Pan.troglodytes.clone.rp43-41g5.GenBank:AC099533.36    TGGCATGATCTCGGCTCACTGCAAGCTCCACCTCCTGGGTTCATGCCATTCTCCTGCCTC 64986
human.FAM30B-LOC105372935-GGT2.NCBI.                                              tggcacgatctcagctcactgcaacctctgcctcccgggttcaagccattctcctgcctc 55560
human.FAM230E-LOC105377182-GGT3P.NCBI.                                            tggcacgatctcagctcactgcaacctctgcctcccgggttcaagccattctcctgcctc 54214
human.FAM230J-LOC105372942-GGTLC5P.NCBI.rev.compl                                 ------------------------------------------------------------ 46364
human.FAM230A.ncRNA.18487127.18500594-GGTLC3.18516335.18518165.rev.compl.REF      ------------------------------------------------------------ 31039

chimp.LOC112206744-LOC107973052-GGT2.revcompl                                     agcctcctgagtagctgggactacaggcgcacaccaccacgcccggctaa-tttttgtat 56629
chimp54546-135457.revcompl.Pan.troglodytes.clone.rp43-41g5.GenBank:AC099533.36    AGCCTCCTGAGTAGCTGGGACTACAGGCGCCTGCCACCACGCCCAGCTAATTTTTTGTAT 65046
human.FAM30B-LOC105372935-GGT2.NCBI.                                              agcctcctgagtagctgggactacaggtgcatgccaccacgcccagctaa-tttttgtat 55619
human.FAM230E-LOC105377182-GGT3P.NCBI.                                            agcctcctgagtagctgggactacaggtgcatgccaccacgcccagctaa-tttttgtat 54273
human.FAM230J-LOC105372942-GGTLC5P.NCBI.rev.compl                                 ------------------------------------------------------------ 46364
human.FAM230A.ncRNA.18487127.18500594-GGTLC3.18516335.18518165.rev.compl.REF      ------------------------------------------------------------ 31039

chimp.LOC112206744-LOC107973052-GGT2.revcompl                                     ttttagtagagacggggtttcaccatgttggccaggatggtctctatctcttgacttcat 56689
chimp54546-135457.revcompl.Pan.troglodytes.clone.rp43-41g5.GenBank:AC099533.36    TTTTAGTAGAAATGGGGTTTCACCGTGTTAGCCAGGATGGTCTCGATCTCCTGATCTCAT 65106
human.FAM30B-LOC105372935-GGT2.NCBI.                                              ttttagtagagatggggtttcaccatgttggccaggatggtctctatctcttgacttcat 55679
human.FAM230E-LOC105377182-GGT3P.NCBI.                                            ttttagtagagacggggtttcaccatgttggccaggatggtctctatctcttgacttcat 54333
human.FAM230J-LOC105372942-GGTLC5P.NCBI.rev.compl                                 ------------------------------------------------------------ 46364
human.FAM230A.ncRNA.18487127.18500594-GGTLC3.18516335.18518165.rev.compl.REF      ------------------------------------------------------------ 31039

chimp.LOC112206744-LOC107973052-GGT2.revcompl                                     gatccacctgccttggcctc--------tttttttttttttttggagacaaagtctctct 56741
chimp54546-135457.revcompl.Pan.troglodytes.clone.rp43-41g5.GenBank:AC099533.36    GATCTGCCCGCCTTG--------------------------------------------- 65121
human.FAM30B-LOC105372935-GGT2.NCBI.                                              gatccgcctgccttggcctcttttttttttttttttttttttggagacaaagtctctct 55739
human.FAM230E-LOC105377182-GGT3P.NCBI.                                            gatccacctgccttggcctcttttttttttttttttttttttggagacaaagtctctct 54393
human.FAM230J-LOC105372942-GGTLC5P.NCBI.rev.compl                                 ------------------------------------------------------------ 46364
human.FAM230A.ncRNA.18487127.18500594-GGTLC3.18516335.18518165.rev.compl.REF      ------------------------------------------------------------ 31039

chimp.LOC112206744-LOC107973052-GGT2.revcompl                                     ttgttgctcaggctggagtgcagtggtgtgatctcggatcactgcagcctcagtctcctg 56801
chimp54546-135457.revcompl.Pan.troglodytes.clone.rp43-41g5.GenBank:AC099533.36    ------------------------------------------------------------ 65121
human.FAM30B-LOC105372935-GGT2.NCBI.                                              ttgttgctcaggctggagtgcagtggtgtgatcttggatcactgcagcctcagtctcctg 55799
human.FAM230E-LOC105377182-GGT3P.NCBI.                                            ttgttgctcaggctggagtgcagtggtgtgatcttggatcactgcagcctcagtctcctg 54453
human.FAM230J-LOC105372942-GGTLC5P.NCBI.rev.compl                                 ------------------------------------------------------------ 46364
human.FAM230A.ncRNA.18487127.18500594-GGTLC3.18516335.18518165.rev.compl.REF      ------------------------------------------------------------ 31039

chimp.LOC112206744-LOC107973052-GGT2.revcompl                                     ggctcaattgaccctcccatctcagcctcccaagtagctgggactatgggcacatgccac 56861
chimp54546-135457.revcompl.Pan.troglodytes.clone.rp43-41g5.GenBank:AC099533.36    ------------------------GCCTCCCAAAGTGCTGGGATTAGAGGCGTGAGCCAC 65157
human.FAM30B-LOC105372935-GGT2.NCBI.                                              ggctcaattgaccctcccatctcagcctcccaagtagctgggactatgggcacatgccac 55859
human.FAM230E-LOC105377182-GGT3P.NCBI.                                            ggctcaattgaccctcccatctcagcctcccaagtagctgggactatgggcacatgccac 54513
human.FAM230J-LOC105372942-GGTLC5P.NCBI.rev.compl                                 ------------------------------------------------------------ 46364
human.FAM230A.ncRNA.18487127.18500594-GGTLC3.18516335.18518165.rev.compl.REF      ------------------------------------------------------------ 31039

chimp.LOC112206744-LOC107973052-GGT2.revcompl                                     catgcccagccaatt---------------------ttgtttgtgtgttttgtagagatg 56900
chimp54546-135457.revcompl.Pan.troglodytes.clone.rp43-41g5.GenBank:AC099533.36    CAAGCCCAGCTGATTCTTTTTTTTAATTCTTCCTTCCTTTTTTTTTTTTTTTTTTTTTTT 65217
human.FAM30B-LOC105372935-GGT2.NCBI.                                              catgcccagccaatt-----------------ttgtttgtttgtgtattttgtagagatg 55902
human.FAM230E-LOC105377182-GGT3P.NCBI.                                            catgcccagccaatt-----------------ttgtttgtttgtgtattttgtagagatg 54556
human.FAM230J-LOC105372942-GGTLC5P.NCBI.rev.compl                                 ------------------------------------------------------------ 46364
human.FAM230A.ncRNA.18487127.18500594-GGTLC3.18516335.18518165.rev.compl.REF      ------------------------------------------------------------ 31039

chimp.LOC112206744-LOC107973052-GGT2.revcompl                                     gggtttcatcatgttgcccaggctggtcaagaactcctgtgctcaagtgatccacccacc 56960
```

```
chimp54546-135457.revcompl.Pan.troglodytes.clone.rp43-41g5.GenBank:AC099533.36      GCTTTTTATTTTGCTGAAACAGGGTTGCCATTACTTTTAAAGGCAAAAACCGCAATTACT 65277
human.FAM30B-LOC105372935-GGT2.NCBI.                                                 gggtttcatcatgttgcccaggctggtcaagaactcctgtgctcaagtaatccacccacc 55962
human.FAM230E-LOC105377182-GGT3P.NCBI.                                               gggtttcatcatgttgcccaggctggtcaagaactcctgtgctcaagtaatccacccacc 54616
human.FAM230J-LOC105372942-GGTLC5P.NCBI.rev.compl                                    ------------------------------------------------------------ 46364
human.FAM230A.ncRNA.18487127.18500594-GGTLC3.18516335.18518165.rev.compl.REF         ------------------------------------------------------------ 31039


chimp.LOC112206744-LOC107973052-GGT2.revcompl                                        ttggcctcccaaagtgctggtattacaggcatgagccactgtgctcagcctttgttttat 57020
chimp54546-135457.revcompl.Pan.troglodytes.clone.rp43-41g5.GenBank:AC099533.36      TTTG--TACCAACCTAATAATAT------------------------------------- 65298
human.FAM30B-LOC105372935-GGT2.NCBI.                                                 ttggcctcccaaagtgctggtattacaggcatgagccactgtgcccagcctttgttttat 56022
human.FAM230E-LOC105377182-GGT3P.NCBI.                                               ttggcctcccaaagtgctggtattacaggcatgagccactgtgcccagcctttgttttat 54676
human.FAM230J-LOC105372942-GGTLC5P.NCBI.rev.compl                                    ------------------------------------------------------------ 46364
human.FAM230A.ncRNA.18487127.18500594-GGTLC3.18516335.18518165.rev.compl.REF         ------------------------------------------------------------ 31039


chimp.LOC112206744-LOC107973052-GGT2.revcompl                                        gagacagggtctcactttgtcacccaggatgaagtgcagtggcacagtcttggctcaatg 57080
chimp54546-135457.revcompl.Pan.troglodytes.clone.rp43-41g5.GenBank:AC099533.36      -------------------------------------------------ATTGCTCTGGC 65309
human.FAM30B-LOC105372935-GGT2.NCBI.                                                 gagacagggtctcactttgtcacccaggatgaagtgcagtggcacagtcttggctcaatg 56082
human.FAM230E-LOC105377182-GGT3P.NCBI.                                               gagacagggtctcactttgtcacccaggatgaagtgcagtggcacagtcttggctcaatg 54736
human.FAM230J-LOC105372942-GGTLC5P.NCBI.rev.compl                                    ------------------------------------------------------------ 46364
human.FAM230A.ncRNA.18487127.18500594-GGTLC3.18516335.18518165.rev.compl.REF         ------------------------------------------------------------ 31039


chimp.LOC112206744-LOC107973052-GGT2.revcompl                                        cagctttgacctcctgggctcaagcaatcctcccacttcagtctcctgagtagctgggac 57140
chimp54546-135457.revcompl.Pan.troglodytes.clone.rp43-41g5.GenBank:AC099533.36      TGGTTTCAAATTCCTGGCCTGAAGCAATCCTCCTGCCTCAGTCTCCCGAGTAGCTGGGAC 65369
human.FAM30B-LOC105372935-GGT2.NCBI.                                                 cagctttgacctcctgggctcaagcaatcctcccacttcagtctcctgagtagctgggac 56142
human.FAM230E-LOC105377182-GGT3P.NCBI.                                               cagctttgacctcctgggctcaagcaatcctcccacttcagtctcctgagtagctgggac 54796
human.FAM230J-LOC105372942-GGTLC5P.NCBI.rev.compl                                    ------------------------------------------------------------ 46364
human.FAM230A.ncRNA.18487127.18500594-GGTLC3.18516335.18518165.rev.compl.REF         ------------------------------------------------------------ 31039


chimp.LOC112206744-LOC107973052-GGT2.revcompl                                        tacaggtaagaaccaccacaccgggcaatttttgtctttttgtagagatagggtcttt 57200
chimp54546-135457.revcompl.Pan.troglodytes.clone.rp43-41g5.GenBank:AC099533.36      TATAGGCGTGAGCCACCACACCTGGTCTAGAGCAGT------------------------ 65405
human.FAM30B-LOC105372935-GGT2.NCBI.                                                 tacaggtaagaaccaccacaccgggcaatttttgtctttttgtagagatagggtcttt 56202
human.FAM230E-LOC105377182-GGT3P.NCBI.                                               tacaggtaagaaccaccacaccgggcaatttttgtcttttcgtagagatagggtcttt 54856
human.FAM230J-LOC105372942-GGTLC5P.NCBI.rev.compl                                    ------------------------------------------------------------ 46364
human.FAM230A.ncRNA.18487127.18500594-GGTLC3.18516335.18518165.rev.compl.REF         ------------------------------------------------------------ 31039


chimp.LOC112206744-LOC107973052-GGT2.revcompl                                        ctatgttgcccaggctggtctcaaactcatggtctaaagcaatcttatctcctcaacctc 57260
chimp54546-135457.revcompl.Pan.troglodytes.clone.rp43-41g5.GenBank:AC099533.36      -------------GCTGTTGACTTACTCATGTGGCTGAGGGAGGGGATGCCATAATGCCC 65452
human.FAM30B-LOC105372935-GGT2.NCBI.                                                 ctatgttgcccaggctggtctcaaactcatggtctaaagcaatcctatcgcctcaacctc 56262
human.FAM230E-LOC105377182-GGT3P.NCBI.                                               ctatgttgcccaggctggtctcaaactcatggtctaaagcaatcctatcgcctcaacctc 54916
human.FAM230J-LOC105372942-GGTLC5P.NCBI.rev.compl                                    ------------------------------------------------------------ 46364
human.FAM230A.ncRNA.18487127.18500594-GGTLC3.18516335.18518165.rev.compl.REF         ------------------------------------------------------------ 31039


chimp.LOC112206744-LOC107973052-GGT2.revcompl                                        ccaaagtgctgggattacagtttcttctttt----------------------------- 57291
chimp54546-135457.revcompl.Pan.troglodytes.clone.rp43-41g5.GenBank:AC099533.36      CACACG---TGGGGTGCAGGTTTCTTACTCTAGGAGTCACAGTCAACCAGAAATAAAGCA 65509
human.FAM30B-LOC105372935-GGT2.NCBI.                                                 ccaaagtgctgggattacagtttcttctttt----------------------------- 56293
human.FAM230E-LOC105377182-GGT3P.NCBI.                                               ccaaagtgctgggattacagtttcttctttt----------------------------- 54947
human.FAM230J-LOC105372942-GGTLC5P.NCBI.rev.compl                                    ------------------------------------------------------------ 46364
human.FAM230A.ncRNA.18487127.18500594-GGTLC3.18516335.18518165.rev.compl.REF         ------------------------------------------------------------ 31039


chimp.LOC112206744-LOC107973052-GGT2.revcompl                                        ------------------------------------------------------------ 57291
chimp54546-135457.revcompl.Pan.troglodytes.clone.rp43-41g5.GenBank:AC099533.36      GCCCTCAGGAGCACTGGATCCAGCTTCAGATCATCTCAGTCACTCAAATGCATAAAGGAG 65569
human.FAM30B-LOC105372935-GGT2.NCBI.                                                 ------------------------------------------------------------ 56293
human.FAM230E-LOC105377182-GGT3P.NCBI.                                               ------------------------------------------------------------ 54947
human.FAM230J-LOC105372942-GGTLC5P.NCBI.rev.compl                                    ------------------------------------------------------------ 46364
human.FAM230A.ncRNA.18487127.18500594-GGTLC3.18516335.18518165.rev.compl.REF         ------------------------------------------------------------ 31039


chimp.LOC112206744-LOC107973052-GGT2.revcompl                                        -----------------------------------------tcttttcttttttttttct 57310
chimp54546-135457.revcompl.Pan.troglodytes.clone.rp43-41g5.GenBank:AC099533.36      ATCTCAGAATTTCAGACCCCTAGGCCTCAAATTATTTATTTTTATTTATTTATTTATTT 65629
human.FAM30B-LOC105372935-GGT2.NCBI.                                                 -------------------------------tcttttcttttttctttttttttttttct 56322
human.FAM230E-LOC105377182-GGT3P.NCBI.                                               -------------------------------tcttttcttttttctttttttttttttct 54976
human.FAM230J-LOC105372942-GGTLC5P.NCBI.rev.compl                                    ------------------------------------------------------------ 46364
human.FAM230A.ncRNA.18487127.18500594-GGTLC3.18516335.18518165.rev.compl.REF         ------------------------------------------------------------ 31039


chimp.LOC112206744-LOC107973052-GGT2.revcompl                                        gagacagagtttcactcagttgcccaggctggagtgcagtggcatgatcttagctcactg 57370
chimp54546-135457.revcompl.Pan.troglodytes.clone.rp43-41g5.GenBank:AC099533.36      GAGACAGCATCTCACTCTGTTGCCCAGGCTGGAGTACAGTCTCAGGATCATGGCTCACTG 65689
human.FAM30B-LOC105372935-GGT2.NCBI.                                                 gagacagagtttcactcagttgcccaggctggagtgcagtggcatgatcttagctcactg 56382
human.FAM230E-LOC105377182-GGT3P.NCBI.                                               gagacagagtttcactcagttgcccaggctggagtgcagtggcatgatcttagctcactg 55036
human.FAM230J-LOC105372942-GGTLC5P.NCBI.rev.compl                                    ------------------------------------------------------------ 46364
human.FAM230A.ncRNA.18487127.18500594-GGTLC3.18516335.18518165.rev.compl.REF         ------------------------------------------------------------ 31039


chimp.LOC112206744-LOC107973052-GGT2.revcompl                                        caacctctgcctcctgggttcaagcgattctcctgcctcagcctcctgagtagctgggat 57430
chimp54546-135457.revcompl.Pan.troglodytes.clone.rp43-41g5.GenBank:AC099533.36      CAGCCTCGATCACCCAGGCTCAAGTGAGCCTCCCACCTCAGCGTCCCAAACAGCTGGGAC 65749
human.FAM30B-LOC105372935-GGT2.NCBI.                                                 caacctctgcctcctgggttcaagcgattctcctgcctcagcctcctgagtagctgggat 56442
human.FAM230E-LOC105377182-GGT3P.NCBI.                                               caacctctgcctcctgggttcaagcgattctcctgcctcagcctcctgagtagctgggat 55096
human.FAM230J-LOC105372942-GGTLC5P.NCBI.rev.compl                                    ------------------------------------------------------------ 46364
human.FAM230A.ncRNA.18487127.18500594-GGTLC3.18516335.18518165.rev.compl.REF         ------------------------------------------------------------ 31039


chimp.LOC112206744-LOC107973052-GGT2.revcompl                                        tacaggcgcacaccaccatg---cccggctaacttttatattttagtagggacagag- 57486
chimp54546-135457.revcompl.Pan.troglodytes.clone.rp43-41g5.GenBank:AC099533.36      TACAGGCACACACCACCACCACACCCAGCTAATTTTT--TATTTTTTGTAGAGATGGGGT 65807
human.FAM30B-LOC105372935-GGT2.NCBI.                                                 tacaggcgcacaccaccatg---cccggctaacttttatattttagtagggacagag- 56498
human.FAM230E-LOC105377182-GGT3P.NCBI.                                               tacaggcgcacaccaccatg---cccggctaacttttatattttagtagggacagag- 55152
human.FAM230J-LOC105372942-GGTLC5P.NCBI.rev.compl                                    ------------------------------------------------------------ 46364
human.FAM230A.ncRNA.18487127.18500594-GGTLC3.18516335.18518165.rev.compl.REF         ------------------------------------------------------------ 31039


chimp.LOC112206744-LOC107973052-GGT2.revcompl                                        tgcaccatgttggccaggctggtctcgaactcctgacctcgggtggtctgcccgcctcaa 57546
chimp54546-135457.revcompl.Pan.troglodytes.clone.rp43-41g5.GenBank:AC099533.36      TTCACCATGTTGCGCAGGCTGATCTTGAACTCCTAGGCTCAAGCAATCTGCCTGCCTCAG 65867
human.FAM30B-LOC105372935-GGT2.NCBI.                                                 tgcaccatgttggccaggctgatctcgaactcctgacctc-agtggtctgcccgcctcaa 56557
human.FAM230E-LOC105377182-GGT3P.NCBI.                                               tgcaccatgttggccaggctggtctcgaactcctgacctc-agtggtctgcccgcctcaa 55211
human.FAM230J-LOC105372942-GGTLC5P.NCBI.rev.compl                                    ------------------------------------------------------------ 46364
human.FAM230A.ncRNA.18487127.18500594-GGTLC3.18516335.18518165.rev.compl.REF         ------------------------------------------------------------ 31039


chimp.LOC112206744-LOC107973052-GGT2.revcompl                                        ccccccaaagtgctgggattactggtgtggaccaccttgcccagacagtttcctctttat 57606
chimp54546-135457.revcompl.Pan.troglodytes.clone.rp43-41g5.GenBank:AC099533.36      CCTCCCAAAGTGATGGGATTACAAGCATGAGCCATCATGCTTGG---------------- 65911
human.FAM30B-LOC105372935-GGT2.NCBI.                                                 ccccccaaagtgctgggattacaggtgtggaccaccttgcccagacagtttcctctttat 56617
human.FAM230E-LOC105377182-GGT3P.NCBI.                                               ccccccaaagtgctgggattacaggtgtggaccaccttgcccagacagtttcctctttat 55271
human.FAM230J-LOC105372942-GGTLC5P.NCBI.rev.compl                                    ------------------------------------------------------------ 46364
human.FAM230A.ncRNA.18487127.18500594-GGTLC3.18516335.18518165.rev.compl.REF         ------------------------------------------------------------ 31039


chimp.LOC112206744-LOC107973052-GGT2.revcompl                                        caagcaaacaaatgtacatgacttttataattgggacaaaaagggaaattgctatacttt 57666
chimp54546-135457.revcompl.Pan.troglodytes.clone.rp43-41g5.GenBank:AC099533.36      --------------------------------------------------GCTCGAAATT 65921
human.FAM30B-LOC105372935-GGT2.NCBI.                                                 taagcaaacaaatgtacatgacttttataattgggacaaaaagggaaattgctatacttt 56677
human.FAM230E-LOC105377182-GGT3P.NCBI.                                               taagcaaacaaatgtacatgacttttataattgggacaaaaagggaaattgctatacttt 55331
human.FAM230J-LOC105372942-GGTLC5P.NCBI.rev.compl                                    ------------------------------------------------------------ 46364
human.FAM230A.ncRNA.18487127.18500594-GGTLC3.18516335.18518165.rev.compl.REF         ------------------------------------------------------------ 31039


chimp.LOC112206744-LOC107973052-GGT2.revcompl                                        attaatacattttttttttttccttgctagagatggtggcttacacctgtaatctcagcac 57726
chimp54546-135457.revcompl.Pan.troglodytes.clone.rp43-41g5.GenBank:AC099533.36      ATTTTTAAAAATTATTTCT----GGCTGGGCGCAGTGGCTCACGCCTGTAATCCCAGCAC 65977
human.FAM30B-LOC105372935-GGT2.NCBI.                                                 attaatacattttttttttttccctgctagagatggtggcttacacctgtaatctcagcac 56737
human.FAM230E-LOC105377182-GGT3P.NCBI.                                               attaatacattttttttttttccctgctagagatggtggcttacacctgtaatctcagcac 55391
human.FAM230J-LOC105372942-GGTLC5P.NCBI.rev.compl                                    ------------------------------------------------------------ 46364
human.FAM230A.ncRNA.18487127.18500594-GGTLC3.18516335.18518165.rev.compl.REF         ------------------------------------------------------------ 31039


chimp.LOC112206744-LOC107973052-GGT2.revcompl                                        tctgggaggccaaggtggaggatcacttgaggccaggagttcaagaccagcctgggcaat 57786
chimp54546-135457.revcompl.Pan.troglodytes.clone.rp43-41g5.GenBank:AC099533.36      TTTGGGAAGCTGAGGTGGA-CAGATCACGAGGTCAGGAGTCTGAGACCAGCCTGACCAAC 66036
human.FAM30B-LOC105372935-GGT2.NCBI.                                                 tttgggaggccaaggtggaggatcacttgaggccaggagttcaagaccagcctgggcaat 56797
human.FAM230E-LOC105377182-GGT3P.NCBI.                                               tttgggaggccaaggtggaggatcacttgaggccaggagttcaagaccagcctgggcaat 55451
human.FAM230J-LOC105372942-GGTLC5P.NCBI.rev.compl                                    ------------------------------------------------------------ 46364
human.FAM230A.ncRNA.18487127.18500594-GGTLC3.18516335.18518165.rev.compl.REF         ------------------------------------------------------------ 31039


chimp.LOC112206744-LOC107973052-GGT2.revcompl                                        agagtgaga--ccatctctagaaaacagtctttttttaattagtcaggtgtgatgcacgcc 57844
chimp54546-135457.revcompl.Pan.troglodytes.clone.rp43-41g5.GenBank:AC099533.36      ATGGTGAAAACCCATCTCTACTAAAAATACAAAAATTAGCCAGGCGTGCTGGCACCTGCC 66096
human.FAM30B-LOC105372935-GGT2.NCBI.                                                 agagtgaga--ccatctctacaaaacagtctttttttaattagtcaggtatgatgcacgcc 56855
human.FAM230E-LOC105377182-GGT3P.NCBI.                                               agagtgaga--ccatctctacaaaacagtctttttttaattagtcaggtatgatgcacgcc 55509
human.FAM230J-LOC105372942-GGTLC5P.NCBI.rev.compl                                    ------------------------------------------------------------ 46364
human.FAM230A.ncRNA.18487127.18500594-GGTLC3.18516335.18518165.rev.compl.REF         ------------------------------------------------------------ 31039


chimp.LOC112206744-LOC107973052-GGT2.revcompl                                        tgtagtcctagctactcaggggggctgaggtgagaggatcgcttaagcccaagagttcaag 57904
chimp54546-135457.revcompl.Pan.troglodytes.clone.rp43-41g5.GenBank:AC099533.36      TGCAATCCCAGCTACTTGGGAGGCTGAGATGGGAGAAT---TGAACCCTGGGATGCAGAG 66153
```

```
human.FAM30B-LOC105372935-GGT2.NCBI.                                            tgtagtcctagctactcaggggggctgaggtgagaggatcgcttaagcccaagagttcaag 56915
human.FAM230E-LOC105377182-GGT3P.NCBI.                                          tgtagtcctagctactcaggggggctgaggtgagaggatcgcttaagcccaagagttcaag 55569
human.FAM230J-LOC105372942-GGTLC5P.NCBI.rev.compl                               ------------------------------------------------------------ 46364
human.FAM230A.ncRNA.18487127.18500594-GGTLC3.18516335.18518165.rev.compl.REF    ------------------------------------------------------------ 31039

chimp.LOC112206744-LOC107973052-GGT2.revcompl                                   gctgcagtgagctatgatcatgccactgcactccagcctgggtgacagaacaagaccctg 57964
chimp54546-135457.revcompl.Pan.troglodytes.clone.rp43-41g5.GenBank:AC099533.36  GTTGCAGTGAGCCGAGATCACATCATTGCACTCCAGCTTGGGCAACAAAGCGAGACTCCA 66213
human.FAM30B-LOC105372935-GGT2.NCBI.                                            gctgcagtgagctatgatcatgccactgcactccagcctgggtgacagaacaagaccctg 56975
human.FAM230E-LOC105377182-GGT3P.NCBI.                                          gctgcagtgagctatgatcatgccactgcactccagcctgggtgacagaacaagaccctg 55629
human.FAM230J-LOC105372942-GGTLC5P.NCBI.rev.compl                               ------------------------------------------------------------ 46364
human.FAM230A.ncRNA.18487127.18500594-GGTLC3.18516335.18518165.rev.compl.REF    ------------------------------------------------------------ 31039

chimp.LOC112206744-LOC107973052-GGT2.revcompl                                   tctcaaaatatgaaaaacataatatttttctgtttaagtctttaagagggaactgatct 58024
chimp54546-135457.revcompl.Pan.troglodytes.clone.rp43-41g5.GenBank:AC099533.36  TCTCAAAAAAAAAATTATTTGTAACAATTTTTATACACAACTTTCATTGCCAAATTAAAA 66273
human.FAM30B-LOC105372935-GGT2.NCBI.                                            tctcaaaatatgaaaaacataatatttttctgtttaagtctttaggagggaacttatct 57035
human.FAM230E-LOC105377182-GGT3P.NCBI.                                          tctcaaaatatgaaaaacataatatttttctgtttaagtctttaggagggaacttatct 55689
human.FAM230J-LOC105372942-GGTLC5P.NCBI.rev.compl                               ------------------------------------------------------------ 46364
human.FAM230A.ncRNA.18487127.18500594-GGTLC3.18516335.18518165.rev.compl.REF    ------------------------------------------------------------ 31039

chimp.LOC112206744-LOC107973052-GGT2.revcompl                                   ttatatataacatgagataagagtctaaaatagaataaaacagtagaagccaggcaccat 58084
chimp54546-135457.revcompl.Pan.troglodytes.clone.rp43-41g5.GenBank:AC099533.36  ACAAGCAAACAAGGAAATAAGA--CAGCAGGAACAAAACTCAAGAGAAAGCACAC----- 66326
human.FAM30B-LOC105372935-GGT2.NCBI.                                            ttatatataacatgagataagagtctaaaatagaataacacagtagaagccaggcaccat 57095
human.FAM230E-LOC105377182-GGT3P.NCBI.                                          ttatatataacatgagataagagtctaaaatagaataacacagtagaagccaggcaccat 55749
human.FAM230J-LOC105372942-GGTLC5P.NCBI.rev.compl                               ------------------------------------------------------------ 46364
human.FAM230A.ncRNA.18487127.18500594-GGTLC3.18516335.18518165.rev.compl.REF    ------------------------------------------------------------ 31039

chimp.LOC112206744-LOC107973052-GGT2.revcompl                                   ggctcacacctgtaatcccagcactttaggaagctgaggcgggaggatcacttaaaccca 58144
chimp54546-135457.revcompl.Pan.troglodytes.clone.rp43-41g5.GenBank:AC099533.36  ------------------------------------------------------------ 66326
human.FAM30B-LOC105372935-GGT2.NCBI.                                            ggctcacacctgtaatcccagcactttaggaagctgaggcgggaggatcacttaaaccca 57155
human.FAM230E-LOC105377182-GGT3P.NCBI.                                          ggctcacacctgtaatcccagcactttaggaagctgaggcgggaggatcacttaaaccca 55809
human.FAM230J-LOC105372942-GGTLC5P.NCBI.rev.compl                               ------------------------------------------------------------ 46364
human.FAM230A.ncRNA.18487127.18500594-GGTLC3.18516335.18518165.rev.compl.REF    ------------------------------------------------------------ 31039

chimp.LOC112206744-LOC107973052-GGT2.revcompl                                   agagtttgaggctgcagtgggctatgatcgctcccctatactccagcctgggtgacagag 58204
chimp54546-135457.revcompl.Pan.troglodytes.clone.rp43-41g5.GenBank:AC099533.36  ------------------------------------------------------------ 66326
human.FAM30B-LOC105372935-GGT2.NCBI.                                            agagtttgaggctgcagtgggctatgatcgctcccctatactccagcctgggtgacagag 57215
human.FAM230E-LOC105377182-GGT3P.NCBI.                                          agagtttgaggctgcagtgggctatgatcgctcccctatactccagcctgggtgacagag 55869
human.FAM230J-LOC105372942-GGTLC5P.NCBI.rev.compl                               ------------------------------------------------------------ 46364
human.FAM230A.ncRNA.18487127.18500594-GGTLC3.18516335.18518165.rev.compl.REF    ------------------------------------------------------------ 31039

chimp.LOC112206744-LOC107973052-GGT2.revcompl                                   taagactccaccttaaaaaaagaataagcccttcatgtccctgtttgggcaacagcgttc 58264
chimp54546-135457.revcompl.Pan.troglodytes.clone.rp43-41g5.GenBank:AC099533.36  ------------------------------------------------------------ 66326
human.FAM30B-LOC105372935-GGT2.NCBI.                                            taagactccaccttaaaaaaagaataagcccttcatgtccctgtttgggcaacagcgttc 57275
human.FAM230E-LOC105377182-GGT3P.NCBI.                                          taagactccaccttaaaaaaagaataagcccttcatgtccctgtttgggcaacagcgttc 55929
human.FAM230J-LOC105372942-GGTLC5P.NCBI.rev.compl                               ------------------------------------------------------------ 46364
human.FAM230A.ncRNA.18487127.18500594-GGTLC3.18516335.18518165.rev.compl.REF    ------------------------------------------------------------ 31039

chimp.LOC112206744-LOC107973052-GGT2.revcompl                                   ctt-------ggggggatgggggagggatggcattgggagatatacataatgctaaatga 58317
chimp54546-135457.revcompl.Pan.troglodytes.clone.rp43-41g5.GenBank:AC099533.36  ------------------------------------------------------------ 66326
human.FAM30B-LOC105372935-GGT2.NCBI.                                            cttggggggaggggggaggggggagggatggcattgggagatatacctaatgctaaatga 57335
human.FAM230E-LOC105377182-GGT3P.NCBI.                                          cttggggggaggggggaggggggagggatggcattgggagatatacctaatgctaaatga 55989
human.FAM230J-LOC105372942-GGTLC5P.NCBI.rev.compl                               ------------------------------------------------------------ 46364
human.FAM230A.ncRNA.18487127.18500594-GGTLC3.18516335.18518165.rev.compl.REF    ------------------------------------------------------------ 31039

chimp.LOC112206744-LOC107973052-GGT2.revcompl                                   tgagttggtgggtgcagcacaccaacatggcacatgtgtacatatgtaacaaacctgcac 58377
chimp54546-135457.revcompl.Pan.troglodytes.clone.rp43-41g5.GenBank:AC099533.36  ------------------------------------------------ACAACTTTGAAA 66338
human.FAM30B-LOC105372935-GGT2.NCBI.                                            cgagttggtgggtgcagcacaccaacatggcacatgtgtacatatgtaacaaacctgcac 57395
human.FAM230E-LOC105377182-GGT3P.NCBI.                                          cgagttggtgggtgcagcacaccaacatggcacatgtgtacatatgtaacaaacctgcac 56049
human.FAM230J-LOC105372942-GGTLC5P.NCBI.rev.compl                               ------------------------------------------------------------ 46364
human.FAM230A.ncRNA.18487127.18500594-GGTLC3.18516335.18518165.rev.compl.REF    ------------------------------------------------------------ 31039

chimp.LOC112206744-LOC107973052-GGT2.revcompl                                   attgtgcacatgtaccctaaaacttaaagtataataataataaaataaaataaaaaaaag 58437
chimp54546-135457.revcompl.Pan.troglodytes.clone.rp43-41g5.GenBank:AC099533.36  AAGGCCTATAGGATCTCCAGAATATAAAAT---CATCAGACCATGTACTTTAAAATAAAT 66395
human.FAM30B-LOC105372935-GGT2.NCBI.                                            attgtgcacatgtaccctaaaacttaaagt---ataataataaaataaaataaaaaaaag 57452
human.FAM230E-LOC105377182-GGT3P.NCBI.                                          attgtgcacatgtaccctaaaacttaaagt---ataataataaaataaaataaaaaaaag 56106
human.FAM230J-LOC105372942-GGTLC5P.NCBI.rev.compl                               ------------------------------------------------------------ 46364
human.FAM230A.ncRNA.18487127.18500594-GGTLC3.18516335.18518165.rev.compl.REF    ------------------------------------------------------------ 31039

chimp.LOC112206744-LOC107973052-GGT2.revcompl                                   aaagaaagaaattgtctcctaaccaaaaaaaaaaaaaaaaaaaagaaaagaaaag----- 58492
chimp54546-135457.revcompl.Pan.troglodytes.clone.rp43-41g5.GenBank:AC099533.36  TCTTTACATGTGTATCAAGGATATAAAGAAATAATTAAGAATTTCATCAGAAAACTGGCA 66455
human.FAM30B-LOC105372935-GGT2.NCBI.                                            aaagaaagaaattgtctcctaaccaaaaaaaaaaaaaaaaaaaagaaaagaaaag----- 57507
human.FAM230E-LOC105377182-GGT3P.NCBI.                                          aaagaaagaaattgtctcctaacc-------aaaaaaaaaaaaagaaaagaaaag----- 56154
human.FAM230J-LOC105372942-GGTLC5P.NCBI.rev.compl                               ------------------------------------------------------------ 46364
human.FAM230A.ncRNA.18487127.18500594-GGTLC3.18516335.18518165.rev.compl.REF    ------------------------------------------------------------ 31039

chimp.LOC112206744-LOC107973052-GGT2.revcompl                                   -------------aataagccgggcgtggtggctcacacctgtaatcccaacacttcggg 58539
chimp54546-135457.revcompl.Pan.troglodytes.clone.rp43-41g5.GenBank:AC099533.36  GCTACAAAAAATGGACGCACTAGGTGTGGTGACTCACGCCTGTAATCCCAGCACTATGGG 66515
human.FAM30B-LOC105372935-GGT2.NCBI.                                            --------aaaagaataagccgggcgtggtggctcatacctgtaatcccaacactttggg 57559
human.FAM230E-LOC105377182-GGT3P.NCBI.                                          --------aaaagaataagccgggcgtggtggctcatacctgtaatcccaacactttggg 56206
human.FAM230J-LOC105372942-GGTLC5P.NCBI.rev.compl                               ------------------------------------------------------------ 46364
human.FAM230A.ncRNA.18487127.18500594-GGTLC3.18516335.18518165.rev.compl.REF    ------------------------------------------------------------ 31039

chimp.LOC112206744-LOC107973052-GGT2.revcompl                                   aggcccaggtgggtggattacctgaggtcaggagttcgagaccagcctgaccaacatggt 58599
chimp54546-135457.revcompl.Pan.troglodytes.clone.rp43-41g5.GenBank:AC099533.36  AGGCTGAGGTGGGTGGATCACCTGAGGTCAGAAGTTCAAGACCAGCCTGACCAATATGGT 66575
human.FAM30B-LOC105372935-GGT2.NCBI.                                            aggcccaggtgggtggattacctgaggtcaggagttcgagaccagcctgaccaacatggt 57619
human.FAM230E-LOC105377182-GGT3P.NCBI.                                          aggcccaggtgggtggattacctgaggtcaggagttcgagaccagcctgaccaacatggt 56266
human.FAM230J-LOC105372942-GGTLC5P.NCBI.rev.compl                               ------------------------------------------------------------ 46364
human.FAM230A.ncRNA.18487127.18500594-GGTLC3.18516335.18518165.rev.compl.REF    ------------------------------------------------------------ 31039

chimp.LOC112206744-LOC107973052-GGT2.revcompl                                   gaaaccccatctctactaaaaacaccataattaggccaggcgcagtggctcacacctgta 58659
chimp54546-135457.revcompl.Pan.troglodytes.clone.rp43-41g5.GenBank:AC099533.36  GAAACCCCA--------------------------------------------------- 66584
human.FAM30B-LOC105372935-GGT2.NCBI.                                            gaaaccccatctctactaaaaacaccataattaggccaggcgcagtggctcacacctgta 57679
human.FAM230E-LOC105377182-GGT3P.NCBI.                                          gaaaccccatctctactaaaaacaccataattaggccaggcgcagtggctcacacctgta 56326
human.FAM230J-LOC105372942-GGTLC5P.NCBI.rev.compl                               ------------------------------------------------------------ 46364
human.FAM230A.ncRNA.18487127.18500594-GGTLC3.18516335.18518165.rev.compl.REF    ------------------------------------------------------------ 31039

chimp.LOC112206744-LOC107973052-GGT2.revcompl                                   atcccagcactttgagaggcagaggtgggaggatcacaaggtcaggagttcaagaccagc 58719
chimp54546-135457.revcompl.Pan.troglodytes.clone.rp43-41g5.GenBank:AC099533.36  ------------------------------------------------------------ 66584
human.FAM30B-LOC105372935-GGT2.NCBI.                                            atcccagcactttgagaggcagaggtgggaggatcacaaggtcaggagttcaagaccagc 57739
human.FAM230E-LOC105377182-GGT3P.NCBI.                                          atcccagcactttgagaggcagaggtgggaggatcacaaggtcaggagttcaagaccagc 56386
human.FAM230J-LOC105372942-GGTLC5P.NCBI.rev.compl                               ------------------------------------------------------------ 46364
human.FAM230A.ncRNA.18487127.18500594-GGTLC3.18516335.18518165.rev.compl.REF    ------------------------------------------------------------ 31039

chimp.LOC112206744-LOC107973052-GGT2.revcompl                                   ctggccaacatagcaaaaccctgtctctactaaaaatacaaaaattagctgggcatggtg 58779
chimp54546-135457.revcompl.Pan.troglodytes.clone.rp43-41g5.GenBank:AC099533.36  -----------------------TCTCTACTAAAATTACAAAAATTAGCCAGGCATGGTG 66621
human.FAM30B-LOC105372935-GGT2.NCBI.                                            ctggccaacatagcaaaaccctgtctctactaaaaatacaaaaattagctgggcatggtg 57799
human.FAM230E-LOC105377182-GGT3P.NCBI.                                          ctggccaacatagcaaaaccctgtctctactaaaaatacaaaaattagctgggcatggtg 56446
human.FAM230J-LOC105372942-GGTLC5P.NCBI.rev.compl                               ------------------------------------------------------------ 46364
human.FAM230A.ncRNA.18487127.18500594-GGTLC3.18516335.18518165.rev.compl.REF    ------------------------------------------------------------ 31039

chimp.LOC112206744-LOC107973052-GGT2.revcompl                                   gcacatgcctgtagtctcagctactgtggaggctgaggcaggagaatcacttgaacctgg 58839
chimp54546-135457.revcompl.Pan.troglodytes.clone.rp43-41g5.GenBank:AC099533.36  GCATGCTCCTGTAGTCCCAGCTACTCGGGAGGCTGAGGCAGGAGAATCACTTGAA-CTGG 66680
human.FAM30B-LOC105372935-GGT2.NCBI.                                            gcacatgcctgtagtctcagctactgtggaggctgaggcaggagaatcacttgaacctgg 57859
human.FAM230E-LOC105377182-GGT3P.NCBI.                                          gcacatgcctgtagtctcagctactgtggaggctgaggcaggagaatcacttgaacctgg 56506
human.FAM230J-LOC105372942-GGTLC5P.NCBI.rev.compl                               ------------------------------------------------------------ 46364
human.FAM230A.ncRNA.18487127.18500594-GGTLC3.18516335.18518165.rev.compl.REF    ------------------------------------------------------------ 31039

chimp.LOC112206744-LOC107973052-GGT2.revcompl                                   gaggtggaggctgcagtaagccaagattgcaccactgccctccagcctgggtaacagagc 58899
chimp54546-135457.revcompl.Pan.troglodytes.clone.rp43-41g5.GenBank:AC099533.36  GAAGGGGAGGTTGCAGTGAGCCGAGATCGCACCACTCTACTCCAGCCTGGGTGACAGAGC 66740
human.FAM30B-LOC105372935-GGT2.NCBI.                                            gaggtggaggctgcagtaagccaagattgcaccactgccctccagcctgggtaacagagc 57919
human.FAM230E-LOC105377182-GGT3P.NCBI.                                          gaggtggaggctgcagtaagccaagattgcaccactgccctccagcctgggtaacagagc 56566
human.FAM230J-LOC105372942-GGTLC5P.NCBI.rev.compl                               ------------------------------------------------------------ 46364
human.FAM230A.ncRNA.18487127.18500594-GGTLC3.18516335.18518165.rev.compl.REF    ------------------------------------------------------------ 31039

chimp.LOC112206744-LOC107973052-GGT2.revcompl                                   gatactctgtctcagaaaacacacaca--------------cacacacacacacacatat 58945
chimp54546-135457.revcompl.Pan.troglodytes.clone.rp43-41g5.GenBank:AC099533.36  GAGACTCCGTCTCAAAAAAAAAAAAAAAAAAAAAAAAAATGGA----------------- 66783
human.FAM30B-LOC105372935-GGT2.NCBI.                                            gatactctgtctcagaaaacacacacacacacacacacacacacacacacacacacatat 57979
```

```
human.FAM230E-LOC105377182-GGT3P.NCBI.                                       gatactctgtctcagaaa----------acacacacacacacacacacacacacacat 56616
human.FAM230J-LOC105372942-GGTLC5P.NCBI.rev.compl                            ------------------------------------------------------------ 46364
human.FAM230A.ncRNA.18487127.18500594-GGTLC3.18516335.18518165.rev.compl.REF ------------------------------------------------------------ 31039

chimp.LOC112206744-LOC107973052-GGT2.revcompl                                acacacacacaaatttgttgggcgtggtggcacactcctgtaatcccagctacttgggag 59005
chimp54546-135457.revcompl.Pan.troglodytes.clone.rp43-41g5.GenBank:AC099533.36 ------------------------------------------------------------ 66783
human.FAM30B-LOC105372935-GGT2.NCBI.                                         acacacacacaaatttgttgggcgtggtggcgcactcctataatcccagctacttgggag 58039
human.FAM230E-LOC105377182-GGT3P.NCBI.                                       acacacacacaaatttgttgggcgtggtggcgcactcctataatcccagctacttgggag 56676
human.FAM230J-LOC105372942-GGTLC5P.NCBI.rev.compl                            ------------------------------------------------------------ 46364
human.FAM230A.ncRNA.18487127.18500594-GGTLC3.18516335.18518165.rev.compl.REF ------------------------------------------------------------ 31039

chimp.LOC112206744-LOC107973052-GGT2.revcompl                                gcttaggcatgagaatcacttgaacttgggaggcggaggttgcagttaactgagattgca 59065
chimp54546-135457.revcompl.Pan.troglodytes.clone.rp43-41g5.GenBank:AC099533.36 ------------------------------------------------------------ 66783
human.FAM30B-LOC105372935-GGT2.NCBI.                                         gcttaggcatgagaatcgcttgaacttgggaggcggaggttgcagttaactgagattgca 58099
human.FAM230E-LOC105377182-GGT3P.NCBI.                                       gcttaggcatgagaatcgctttaacttgggaggcggaggttgcagttaactgagattgca 56736
human.FAM230J-LOC105372942-GGTLC5P.NCBI.rev.compl                            ------------------------------------------------------------ 46364
human.FAM230A.ncRNA.18487127.18500594-GGTLC3.18516335.18518165.rev.compl.REF ------------------------------------------------------------ 31039

chimp.LOC112206744-LOC107973052-GGT2.revcompl                                ccactgcactccagcctggcaacagagcaaaactccatctctaaatagat-tagatagat 59124
chimp54546-135457.revcompl.Pan.troglodytes.clone.rp43-41g5.GenBank:AC099533.36 ------------------------------------------------------------ 66783
human.FAM30B-LOC105372935-GGT2.NCBI.                                         ccactgcactccagcctggcaacagagcaaaactccatctctaaatagatatagatagat 58159
human.FAM230E-LOC105377182-GGT3P.NCBI.                                       ccactgcactccagcctggcaacagagcaaaactccatctctaaatagatatagatagat 56796
human.FAM230J-LOC105372942-GGTLC5P.NCBI.rev.compl                            ------------------------------------------------------------ 46364
human.FAM230A.ncRNA.18487127.18500594-GGTLC3.18516335.18518165.rev.compl.REF ------------------------------------------------------------ 31039

chimp.LOC112206744-LOC107973052-GGT2.revcompl                                agatagatagatagata-atagata-------------------------------gata 59152
chimp54546-135457.revcompl.Pan.troglodytes.clone.rp43-41g5.GenBank:AC099533.36 ------------------------------------------------------------ 66783
human.FAM30B-LOC105372935-GGT2.NCBI.                                         agatagatagatagatagatagatagatagatagat--------------------gata 58199
human.FAM230E-LOC105377182-GGT3P.NCBI.                                       agatagatagatagatagatagatagatagatagatagataatagatagatagatagata 56856
human.FAM230J-LOC105372942-GGTLC5P.NCBI.rev.compl                            ------------------------------------------------------------ 46364
human.FAM230A.ncRNA.18487127.18500594-GGTLC3.18516335.18518165.rev.compl.REF ------------------------------------------------------------ 31039

chimp.LOC112206744-LOC107973052-GGT2.revcompl                                gatagatagatagttagataggaagaaataagtaaaaataacaaccaaacaacaaaacag 59212
chimp54546-135457.revcompl.Pan.troglodytes.clone.rp43-41g5.GenBank:AC099533.36 ------------------------------------------------------------ 66783
human.FAM30B-LOC105372935-GGT2.NCBI.                                         gatagatagatagatagataggaagaaataagtaaaaataacaaccaaacaacaaaacag 58259
human.FAM230E-LOC105377182-GGT3P.NCBI.                                       gatagatagatagatagataggaagaaataagtaaaaataacaaccaaacaacaaaacag 56916
human.FAM230J-LOC105372942-GGTLC5P.NCBI.rev.compl                            ------------------------------------------------------------ 46364
human.FAM230A.ncRNA.18487127.18500594-GGTLC3.18516335.18518165.rev.compl.REF ------------------------------------------------------------ 31039

chimp.LOC112206744-LOC107973052-GGT2.revcompl                                tggagtttatccaaagagacagagactcttagaactgagaaaaggggccctgtttggctc 59272
chimp54546-135457.revcompl.Pan.troglodytes.clone.rp43-41g5.GenBank:AC099533.36 ------------------------------------------------------------ 66783
human.FAM30B-LOC105372935-GGT2.NCBI.                                         tggagtttatccaaagaaacagagactcttagaactgagaaaaggggccctgtttggctc 58319
human.FAM230E-LOC105377182-GGT3P.NCBI.                                       tggagtttatccaaagaaacagagactcttagaactgagaaaaggggccctgtttggctc 56976
human.FAM230J-LOC105372942-GGTLC5P.NCBI.rev.compl                            ------------------------------------------------------------ 46364
human.FAM230A.ncRNA.18487127.18500594-GGTLC3.18516335.18518165.rev.compl.REF ------------------------------------------------------------ 31039

chimp.LOC112206744-LOC107973052-GGT2.revcompl                                taggagacccacaccctgctctcggagtcaccgtcccttcccaaaggctactgagagag 59332
chimp54546-135457.revcompl.Pan.troglodytes.clone.rp43-41g5.GenBank:AC099533.36 ------------------------------------------------------------ 66783
human.FAM30B-LOC105372935-GGT2.NCBI.                                         ta-gagacccacaccctgctctcggagtcaccgtcccttcccaaaggctactgagagag 58378
human.FAM230E-LOC105377182-GGT3P.NCBI.                                       ta-gagacccacaccctgctctcggagtcaccgtcccttcccaaaggctactgagagag 57035
human.FAM230J-LOC105372942-GGTLC5P.NCBI.rev.compl                            ------------------------------------------------------------ 46364
human.FAM230A.ncRNA.18487127.18500594-GGTLC3.18516335.18518165.rev.compl.REF ------------------------------------------------------------ 31039

chimp.LOC112206744-LOC107973052-GGT2.revcompl                                tccaagcaaagcttacatgtggggaaactgagtctcagaggggtgaaggtattgctcagg 59392
chimp54546-135457.revcompl.Pan.troglodytes.clone.rp43-41g5.GenBank:AC099533.36 ------------------------------------------------------------ 66783
human.FAM30B-LOC105372935-GGT2.NCBI.                                         tccaagcgaagcttgcatgtggggaaactgagtctcagaggggtgaaggtattgctcagg 58438
human.FAM230E-LOC105377182-GGT3P.NCBI.                                       tccaagcgaagcttacatgtggggaaactgagtctcagaggggtgaaggtattgctcagg 57095
human.FAM230J-LOC105372942-GGTLC5P.NCBI.rev.compl                            ------------------------------------------------------------ 46364
human.FAM230A.ncRNA.18487127.18500594-GGTLC3.18516335.18518165.rev.compl.REF ------------------------------------------------------------ 31039

chimp.LOC112206744-LOC107973052-GGT2.revcompl                                tccacttgcccagttttcagggcccatgtcccatgccctgccccgctcacctcccaatct 59452
chimp54546-135457.revcompl.Pan.troglodytes.clone.rp43-41g5.GenBank:AC099533.36 ------------------------------------------------------------ 66783
human.FAM30B-LOC105372935-GGT2.NCBI.                                         tccacttgcccagttttcagggcccatgtcccatgccctgccccgctcacctcccaatct 58498
human.FAM230E-LOC105377182-GGT3P.NCBI.                                       tccacttgcccagttttcagggcccatgtcccatgccctgccccgctcacctcccaatct 57155
human.FAM230J-LOC105372942-GGTLC5P.NCBI.rev.compl                            ------------------------------------------------------------ 46364
human.FAM230A.ncRNA.18487127.18500594-GGTLC3.18516335.18518165.rev.compl.REF ------------------------------------------------------------ 31039

chimp.LOC112206744-LOC107973052-GGT2.revcompl                                ccgagcactgcttggcatccgcgcccacggcagccctggtgtacacatggttgtcaggtt 59512
chimp54546-135457.revcompl.Pan.troglodytes.clone.rp43-41g5.GenBank:AC099533.36 ------------------------------------------------------------ 66783
human.FAM30B-LOC105372935-GGT2.NCBI.                                         ccaagcactgcttggcatccgcggccatggcagccctggtgtacacatggttgtcaggtt 58558
human.FAM230E-LOC105377182-GGT3P.NCBI.                                       ccaagcactgcttggcatccgcggccacggcagccctggtgtacacatggttgtcaggtt 57215
human.FAM230J-LOC105372942-GGTLC5P.NCBI.rev.compl                            ------------------------------------------------------------ 46364
human.FAM230A.ncRNA.18487127.18500594-GGTLC3.18516335.18518165.rev.compl.REF ------------------------------------------------------------ 31039

chimp.LOC112206744-LOC107973052-GGT2.revcompl                                ccttggaggctgagggcagccagagacagaggccgacaatgaccagcacgaggaccacgg 59572
chimp54546-135457.revcompl.Pan.troglodytes.clone.rp43-41g5.GenBank:AC099533.36 ------------------------------------------------------------ 66783
human.FAM30B-LOC105372935-GGT2.NCBI.                                         ccttggaggccgagggcagccagagacagaggccgacaatgaccagcaccaggaccacgg 58618
human.FAM230E-LOC105377182-GGT3P.NCBI.                                       ccttggaggccgagggcagccagagacagaggccgacaatgaccagcaccaggaccacgg 57275
human.FAM230J-LOC105372942-GGTLC5P.NCBI.rev.compl                            ------------------------------------------------------------ 46364
human.FAM230A.ncRNA.18487127.18500594-GGTLC3.18516335.18518165.rev.compl.REF ------------------------------------------------------------ 31039

chimp.LOC112206744-LOC107973052-GGT2.revcompl                                ccagcaggcccagcaccactaacttcttcttcatggctctgctgcacccacggggtaagg 59632
chimp54546-135457.revcompl.Pan.troglodytes.clone.rp43-41g5.GenBank:AC099533.36 ------------------------------------------------------------ 66783
human.FAM30B-LOC105372935-GGT2.NCBI.                                         ccagcaggcccagcaccactaacttcttcttcatggctctgctgcacccacggggtaagg 58678
human.FAM230E-LOC105377182-GGT3P.NCBI.                                       ccagcaggcccagcaccactaacttcttcttcatg------------------------- 57310
human.FAM230J-LOC105372942-GGTLC5P.NCBI.rev.compl                            ------------------------------------------------------------ 46364
human.FAM230A.ncRNA.18487127.18500594-GGTLC3.18516335.18518165.rev.compl.REF ------------------------------------------------------------ 31039

chimp.LOC112206744-LOC107973052-GGT2.revcompl                                agcagggtcaggcccagcctcagacattccctggcccctccccaacagggcacagtctaa 59692
chimp54546-135457.revcompl.Pan.troglodytes.clone.rp43-41g5.GenBank:AC099533.36 ------------------------------------------------------------ 66783
human.FAM30B-LOC105372935-GGT2.NCBI.                                         agcagggtcaggcccagcctcagacatgcctggcccctccccaacagggcacagtctaa 58738
human.FAM230E-LOC105377182-GGT3P.NCBI.                                       ------------------------------------------------------------ 57310
human.FAM230J-LOC105372942-GGTLC5P.NCBI.rev.compl                            ------------------------------------------------------------ 46364
human.FAM230A.ncRNA.18487127.18500594-GGTLC3.18516335.18518165.rev.compl.REF ------------------------------------------------------------ 31039

chimp.LOC112206744-LOC107973052-GGT2.revcompl                                agtcaggcctcagaaacacaaggcctgtgtctccttcccgcttcccagaatacgtgcagg 59752
chimp54546-135457.revcompl.Pan.troglodytes.clone.rp43-41g5.GenBank:AC099533.36 ------------------------------------------------------------ 66783
human.FAM30B-LOC105372935-GGT2.NCBI.                                         agtcgggcctcagaaacacaaggccggagtctccttcccgcttcccagaatacgtgcagg 58798
human.FAM230E-LOC105377182-GGT3P.NCBI.                                       ------------------------------------------------------------ 57310
human.FAM230J-LOC105372942-GGTLC5P.NCBI.rev.compl                            ------------------------------------------------------------ 46364
human.FAM230A.ncRNA.18487127.18500594-GGTLC3.18516335.18518165.rev.compl.REF ------------------------------------------------------------ 31039

chimp.LOC112206744-LOC107973052-GGT2.revcompl                                ctgtccggcccccagacctttgcgcaggccatgccctctgccagaagctctgggcctcat 59812
chimp54546-135457.revcompl.Pan.troglodytes.clone.rp43-41g5.GenBank:AC099533.36 ------------------------------------------------------------ 66783
human.FAM30B-LOC105372935-GGT2.NCBI.                                         ctgtccggcccccagacctttgcgcaggccatgccctctgccagaagctctgggcctcat 58858
human.FAM230E-LOC105377182-GGT3P.NCBI.                                       ------------------------------------------------------------ 57310
human.FAM230J-LOC105372942-GGTLC5P.NCBI.rev.compl                            ------------------------------------------------------------ 46364
human.FAM230A.ncRNA.18487127.18500594-GGTLC3.18516335.18518165.rev.compl.REF ------------------------------------------------------------ 31039

chimp.LOC112206744-LOC107973052-GGT2.revcompl                                ctctgccctcccaaatcctccctgcttatcttcagagcccatcctggtaagaaccccatc 59872
chimp54546-135457.revcompl.Pan.troglodytes.clone.rp43-41g5.GenBank:AC099533.36 ------------------------------------------------------------ 66783
human.FAM30B-LOC105372935-GGT2.NCBI.                                         ctctgccctcccaaatcctccctgcttatcttcagagcccatcctggtaagaaccccatc 58918
human.FAM230E-LOC105377182-GGT3P.NCBI.                                       ------------------------------------------------------------ 57310
human.FAM230J-LOC105372942-GGTLC5P.NCBI.rev.compl                            ------------------------------------------------------------ 46364
human.FAM230A.ncRNA.18487127.18500594-GGTLC3.18516335.18518165.rev.compl.REF ------------------------------------------------------------ 31039

chimp.LOC112206744-LOC107973052-GGT2.revcompl                                tccaccagcggcccttcctgggagcccccagatttccacacccctctttctgcagggcct 59932
chimp54546-135457.revcompl.Pan.troglodytes.clone.rp43-41g5.GenBank:AC099533.36 ------------------------------------------------------------ 66783
human.FAM30B-LOC105372935-GGT2.NCBI.                                         tccagcagcggcccttcctgggagcccccagatttccacacccctctttctgcagggcct 58978
human.FAM230E-LOC105377182-GGT3P.NCBI.                                       ------------------------------------------------------------ 57310
human.FAM230J-LOC105372942-GGTLC5P.NCBI.rev.compl                            ------------------------------------------------------------ 46364
human.FAM230A.ncRNA.18487127.18500594-GGTLC3.18516335.18518165.rev.compl.REF ------------------------------------------------------------ 31039

chimp.LOC112206744-LOC107973052-GGT2.revcompl                                ggcctacctcctcacagtggctgagcctccactgcttagggagaagctccagtagggatg 59992
chimp54546-135457.revcompl.Pan.troglodytes.clone.rp43-41g5.GenBank:AC099533.36 ------------------------------------------------------------ 66783
human.FAM30B-LOC105372935-GGT2.NCBI.                                         ggcctacctcctcacagtggctgagcctccactgcttagggagaagctccagcagggatg 59038
human.FAM230E-LOC105377182-GGT3P.NCBI.                                       ------------------------------------------------------------ 57310
```

```
human.FAM230J-LOC105372942-GGTLC5P.NCBI.rev.compl                                  ------------------------------------------------------------ 46364
human.FAM230A.ncRNA.18487127.18500594-GGTLC3.18516335.18518165.rev.compl.REF        ------------------------------------------------------------ 31039

chimp.LOC112206744-LOC107973052-GGT2.revcompl                                       ggcctggcctggtttctcctgtgtcccccacctcagcctagagcctggcactgtccagga 60052
chimp54546-135457.revcompl.Pan.troglodytes.clone.rp43-41g5.GenBank:AC099533.36      ------------------------------------------------------------ 66783
human.FAM30B-LOC105372935-GGT2.NCBI.                                                ggcctggcctggtttctcctgcgtcccccaccccagcctagagcctggcactgtccagga 59098
human.FAM230E-LOC105377182-GGT3P.NCBI.                                              ------------------------------------------------------------ 57310
human.FAM230J-LOC105372942-GGTLC5P.NCBI.rev.compl                                   ------------------------------------------------------------ 46364
human.FAM230A.ncRNA.18487127.18500594-GGTLC3.18516335.18518165.rev.compl.REF        ------------------------------------------------------------ 31039

chimp.LOC112206744-LOC107973052-GGT2.revcompl                                       gtcctctgaagaccctccaccccacctggagcatggggtttagcttccatagtgcccaca 60112
chimp54546-135457.revcompl.Pan.troglodytes.clone.rp43-41g5.GenBank:AC099533.36      ------------------------------------------------------------ 66783
human.FAM30B-LOC105372935-GGT2.NCBI.                                                gtcctctgaagaccctccaccccacctggagcatggggtttagcttccatagtgcccaca 59158
human.FAM230E-LOC105377182-GGT3P.NCBI.                                              ------------------------------------------------------------ 57310
human.FAM230J-LOC105372942-GGTLC5P.NCBI.rev.compl                                   ------------------------------------------------------------ 46364
human.FAM230A.ncRNA.18487127.18500594-GGTLC3.18516335.18518165.rev.compl.REF        ------------------------------------------------------------ 31039

chimp.LOC112206744-LOC107973052-GGT2.revcompl                                       atcagagcgccccacagattcactgccacggggccaggacttaccgtccagcagcagaca 60172
chimp54546-135457.revcompl.Pan.troglodytes.clone.rp43-41g5.GenBank:AC099533.36      ------------------------------------------------------------ 66783
human.FAM30B-LOC105372935-GGT2.NCBI.                                                atcagagcgccccacagattcactgccacggggccaggacttaccgtccagcagcagacg 59218
human.FAM230E-LOC105377182-GGT3P.NCBI.                                              ------------------------------------------------------------ 57310
human.FAM230J-LOC105372942-GGTLC5P.NCBI.rev.compl                                   ------------------------------------------------------------ 46364
human.FAM230A.ncRNA.18487127.18500594-GGTLC3.18516335.18518165.rev.compl.REF        ------------------------------------------------------------ 31039

chimp.LOC112206744-LOC107973052-GGT2.revcompl                                       ggggccccaagccttgcctggggtgttggccacgaaagacaggaggatttggtggaaaca 60232
chimp54546-135457.revcompl.Pan.troglodytes.clone.rp43-41g5.GenBank:AC099533.36      ------------------------------------------------------------ 66783
human.FAM30B-LOC105372935-GGT2.NCBI.                                                ggggccccaagccttgcctggggtgttggccacgaaagacaggaggatttggtggaaaca 59278
human.FAM230E-LOC105377182-GGT3P.NCBI.                                              ------------------------------------------------------------ 57310
human.FAM230J-LOC105372942-GGTLC5P.NCBI.rev.compl                                   ------------------------------------------------------------ 46364
human.FAM230A.ncRNA.18487127.18500594-GGTLC3.18516335.18518165.rev.compl.REF        ------------------------------------------------------------ 31039

chimp.LOC112206744-LOC107973052-GGT2.revcompl                                       gctgaggaaataaccggggtctccctcacactctgctgaagcctgtagccacagaatctt 60292
chimp54546-135457.revcompl.Pan.troglodytes.clone.rp43-41g5.GenBank:AC099533.36      ------------------------------------------------------------ 66783
human.FAM30B-LOC105372935-GGT2.NCBI.                                                gctgaggaaataaccggggtctccctcacactctgctgaagcctgtagccacagaatctt 59338
human.FAM230E-LOC105377182-GGT3P.NCBI.                                              ------------------------------------------------------------ 57310
human.FAM230J-LOC105372942-GGTLC5P.NCBI.rev.compl                                   ------------------------------------------------------------ 46364
human.FAM230A.ncRNA.18487127.18500594-GGTLC3.18516335.18518165.rev.compl.REF        ------------------------------------------------------------ 31039

chimp.LOC112206744-LOC107973052-GGT2.revcompl                                       cttcagagactccctgatcaggcagccttctcgttctcctgaaggtcaaaggaggttacc 60352
chimp54546-135457.revcompl.Pan.troglodytes.clone.rp43-41g5.GenBank:AC099533.36      ------------------------------------------------------------ 66783
human.FAM30B-LOC105372935-GGT2.NCBI.                                                cttcagagactctctgatcaggcagccttctcgttctcctgaaggtcaagggaggttacc 59398
human.FAM230E-LOC105377182-GGT3P.NCBI.                                              ------------------------------------------------------------ 57310
human.FAM230J-LOC105372942-GGTLC5P.NCBI.rev.compl                                   ------------------------------------------------------------ 46364
human.FAM230A.ncRNA.18487127.18500594-GGTLC3.18516335.18518165.rev.compl.REF        ------------------------------------------------------------ 31039

chimp.LOC112206744-LOC107973052-GGT2.revcompl                                       tgaagcacgcacagctcagacctttctggggtactccgtgttacctccctctgcctctag 60412
chimp54546-135457.revcompl.Pan.troglodytes.clone.rp43-41g5.GenBank:AC099533.36      ------------------------------------------------------------ 66783
human.FAM30B-LOC105372935-GGT2.NCBI.                                                tgaagcacgcacagcccagacctttctggggactccgtgttacctccctctgcctctag 59458
human.FAM230E-LOC105377182-GGT3P.NCBI.                                              ------------------------------------------------------------ 57310
human.FAM230J-LOC105372942-GGTLC5P.NCBI.rev.compl                                   ------------------------------------------------------------ 46364
human.FAM230A.ncRNA.18487127.18500594-GGTLC3.18516335.18518165.rev.compl.REF        ------------------------------------------------------------ 31039

chimp.LOC112206744-LOC107973052-GGT2.revcompl                                       ctggtttctctgtctccagttgaactctggaggcaaagaggctgtcagtaacacatttgt 60472
chimp54546-135457.revcompl.Pan.troglodytes.clone.rp43-41g5.GenBank:AC099533.36      ------------------------------------------------------------ 66783
human.FAM30B-LOC105372935-GGT2.NCBI.                                                ctggtttctctgtctccagttgaactctggaggcaaagaggctgtcagtaacacatttgt 59518
human.FAM230E-LOC105377182-GGT3P.NCBI.                                              ------------------------------------------------------------ 57310
human.FAM230J-LOC105372942-GGTLC5P.NCBI.rev.compl                                   ------------------------------------------------------------ 46364
human.FAM230A.ncRNA.18487127.18500594-GGTLC3.18516335.18518165.rev.compl.REF        ------------------------------------------------------------ 31039

chimp.LOC112206744-LOC107973052-GGT2.revcompl                                       ttccatgaattctctcagcatgtctcccaggcacagctcaaagagggttttgcacggagc 60532
chimp54546-135457.revcompl.Pan.troglodytes.clone.rp43-41g5.GenBank:AC099533.36      ------------------------------------------------------------ 66783
human.FAM30B-LOC105372935-GGT2.NCBI.                                                ttccatgaattctctcagcatgtctcccaggcac---------agggttttgcacggagc 59569
human.FAM230E-LOC105377182-GGT3P.NCBI.                                              ------------------------------------------------------------ 57310
human.FAM230J-LOC105372942-GGTLC5P.NCBI.rev.compl                                   ------------------------------------------------------------ 46364
human.FAM230A.ncRNA.18487127.18500594-GGTLC3.18516335.18518165.rev.compl.REF        ------------------------------------------------------------ 31039

chimp.LOC112206744-LOC107973052-GGT2.revcompl                                       agggcaggtaggggacagggcattcctgcacaagcccaggatgtccatgcggtaagcatg 60592
chimp54546-135457.revcompl.Pan.troglodytes.clone.rp43-41g5.GenBank:AC099533.36      ------------------------------------------------------------ 66783
human.FAM30B-LOC105372935-GGT2.NCBI.                                                agggcaggtaggggacagggcattcctgcacaagcccaggatgtgcatgcggtaagcatg 59629
human.FAM230E-LOC105377182-GGT3P.NCBI.                                              ------------------------------------------------------------ 57310
human.FAM230J-LOC105372942-GGTLC5P.NCBI.rev.compl                                   ------------------------------------------------------------ 46364
human.FAM230A.ncRNA.18487127.18500594-GGTLC3.18516335.18518165.rev.compl.REF        ------------------------------------------------------------ 31039

chimp.LOC112206744-LOC107973052-GGT2.revcompl                                       gcaaaggggggctcaggggggcaccgccagcctgccctgctctgacgctggacttgccactc 60652
chimp54546-135457.revcompl.Pan.troglodytes.clone.rp43-41g5.GenBank:AC099533.36      ------------------------------------------------------------ 66783
human.FAM30B-LOC105372935-GGT2.NCBI.                                                gcaaaggggggctcaggaggcaccgccagcctgccctgctctgacgctggacttgccactc 59689
human.FAM230E-LOC105377182-GGT3P.NCBI.                                              ------------------------------------------------------------ 57310
human.FAM230J-LOC105372942-GGTLC5P.NCBI.rev.compl                                   ------------------------------------------------------------ 46364
human.FAM230A.ncRNA.18487127.18500594-GGTLC3.18516335.18518165.rev.compl.REF        ------------------------------------------------------------ 31039

chimp.LOC112206744-LOC107973052-GGT2.revcompl                                       acctgctgtggggcctcaggcaaatcactgaactgtccagcctggatgacggcagcacct 60712
chimp54546-135457.revcompl.Pan.troglodytes.clone.rp43-41g5.GenBank:AC099533.36      ------------------------------------------------------------ 66783
human.FAM30B-LOC105372935-GGT2.NCBI.                                                acctgctgtggggcctcaggcaaatcactgaactgtccagcctggatgacggcagcacct 59749
human.FAM230E-LOC105377182-GGT3P.NCBI.                                              ------------------------------------------------------------ 57310
human.FAM230J-LOC105372942-GGTLC5P.NCBI.rev.compl                                   ------------------------------------------------------------ 46364
human.FAM230A.ncRNA.18487127.18500594-GGTLC3.18516335.18518165.rev.compl.REF        ------------------------------------------------------------ 31039

chimp.LOC112206744-LOC107973052-GGT2.revcompl                                       cacttgccttgctgctgggagtgttgtgaatagagtaggttagactgtgggcagggcttg 60772
chimp54546-135457.revcompl.Pan.troglodytes.clone.rp43-41g5.GenBank:AC099533.36      ------------------------------------------------------------ 66783
human.FAM30B-LOC105372935-GGT2.NCBI.                                                cacttgccttgctgctgggagtgttgtgaatagagtaggttagactgtgggcagggcttg 59809
human.FAM230E-LOC105377182-GGT3P.NCBI.                                              ------------------------------------------------------------ 57310
human.FAM230J-LOC105372942-GGTLC5P.NCBI.rev.compl                                   ------------------------------------------------------------ 46364
human.FAM230A.ncRNA.18487127.18500594-GGTLC3.18516335.18518165.rev.compl.REF        ------------------------------------------------------------ 31039

chimp.LOC112206744-LOC107973052-GGT2.revcompl                                       gtgaatggtagctgtgattatcatcatggctgcaccggggacacccccaggaggcctgag 60832
chimp54546-135457.revcompl.Pan.troglodytes.clone.rp43-41g5.GenBank:AC099533.36      ------------------------------------------------------------ 66783
human.FAM30B-LOC105372935-GGT2.NCBI.                                                gtgaatggtagctgtgattatcatcatggctgcactggggacacccccaggaggcctgag 59869
human.FAM230E-LOC105377182-GGT3P.NCBI.                                              ------------------------------------------------------------ 57310
human.FAM230J-LOC105372942-GGTLC5P.NCBI.rev.compl                                   ------------------------------------------------------------ 46364
human.FAM230A.ncRNA.18487127.18500594-GGTLC3.18516335.18518165.rev.compl.REF        ------------------------------------------------------------ 31039

chimp.LOC112206744-LOC107973052-GGT2.revcompl                                       tggcacaggtctcttgctcactgtatgtcccctgtggactcccttccaggctgtgcagtg 60892
chimp54546-135457.revcompl.Pan.troglodytes.clone.rp43-41g5.GenBank:AC099533.36      ------------------------------------------------------------ 66783
human.FAM30B-LOC105372935-GGT2.NCBI.                                                tggcacaggtctcttgctcactgtatgtcccctgtggactcccttccaggctgtgcagtg 59929
human.FAM230E-LOC105377182-GGT3P.NCBI.                                              ------------------------------------------------------------ 57310
human.FAM230J-LOC105372942-GGTLC5P.NCBI.rev.compl                                   ------------------------------------------------------------ 46364
human.FAM230A.ncRNA.18487127.18500594-GGTLC3.18516335.18518165.rev.compl.REF        ------------------------------------------------------------ 31039

chimp.LOC112206744-LOC107973052-GGT2.revcompl                                       agtggcagcagtgacccttgggaagcctcatggctatggcagcaggtgacaggtgtgaca 60952
chimp54546-135457.revcompl.Pan.troglodytes.clone.rp43-41g5.GenBank:AC099533.36      ------------------------------------------------------------ 66783
human.FAM30B-LOC105372935-GGT2.NCBI.                                                agtggcagcagtgacccttgggaagtctcatggctacggcagcaggtgacaggtgtgaca 59989
human.FAM230E-LOC105377182-GGT3P.NCBI.                                              ------------------------------------------------------------ 57310
human.FAM230J-LOC105372942-GGTLC5P.NCBI.rev.compl                                   ------------------------------------------------------------ 46364
human.FAM230A.ncRNA.18487127.18500594-GGTLC3.18516335.18518165.rev.compl.REF        ------------------------------------------------------------ 31039

chimp.LOC112206744-LOC107973052-GGT2.revcompl                                       acagggaagaaggatgtggtgacagaggttggggttcccctctcccacagtcagtttccc 61012
chimp54546-135457.revcompl.Pan.troglodytes.clone.rp43-41g5.GenBank:AC099533.36      ------------------------------------------------------------ 66783
human.FAM30B-LOC105372935-GGT2.NCBI.                                                acagggaagagggatgtggtgacagaggttggggttcccctctcccacagtcagtttccc 60049
human.FAM230E-LOC105377182-GGT3P.NCBI.                                              ------------------------------------------------------------ 57310
human.FAM230J-LOC105372942-GGTLC5P.NCBI.rev.compl                                   ------------------------------------------------------------ 46364
human.FAM230A.ncRNA.18487127.18500594-GGTLC3.18516335.18518165.rev.compl.REF        ------------------------------------------------------------ 31039

chimp.LOC112206744-LOC107973052-GGT2.revcompl                                       acaaagggcggtgtctgccagcaagcccctccaatgagccccaagctgggtttccctcca 61072
chimp54546-135457.revcompl.Pan.troglodytes.clone.rp43-41g5.GenBank:AC099533.36      ------------------------------------------------------------ 66783
human.FAM30B-LOC105372935-GGT2.NCBI.                                                acaaagggcggtgtctgccagcaagcccctccaatgagccccaagcttggtttccctcca 60109
human.FAM230E-LOC105377182-GGT3P.NCBI.                                              ------------------------------------------------------------ 57310
human.FAM230J-LOC105372942-GGTLC5P.NCBI.rev.compl                                   ------------------------------------------------------------ 46364
```

```
human.FAM230A.ncRNA.18487127.18500594-GGTLC3.18516335.18518165.rev.compl.REF        ------------------------------------------------------------ 31039

chimp.LOC112206744-LOC107973052-GGT2.revcompl                                       ctccaccctgtcccagtgcagagcgtctgacctcagaggcagacacactgtcccagaggt 61132
chimp54546-135457.revcompl.Pan.troglodytes.clone.rp43-41g5.GenBank:AC099533.36      ------------------------------------------------------------ 66783
human.FAM30B-LOC105372935-GGT2.NCBI.                                                ctccacgctgtcccagtgcagagcatctgacctcagaggcagacacactgtcccagaggt 60169
human.FAM230E-LOC105377182-GGT3P.NCBI.                                              ------------------------------------------------------------ 57310
human.FAM230J-LOC105372942-GGTLC5P.NCBI.rev.compl                                   ------------------------------------------------------------ 46364
human.FAM230A.ncRNA.18487127.18500594-GGTLC3.18516335.18518165.rev.compl.REF        ------------------------------------------------------------ 31039

chimp.LOC112206744-LOC107973052-GGT2.revcompl                                       ggtctacgaatggagtccctgtgccctccccacacacagggaacatccaaatgccatcgt 61192
chimp54546-135457.revcompl.Pan.troglodytes.clone.rp43-41g5.GenBank:AC099533.36      ------------------------------------------------------------ 66783
human.FAM30B-LOC105372935-GGT2.NCBI.                                                ggtctacgaatggagtccccgtgccctccccacacacagggaacatccaaatgccatcgt 60229
human.FAM230E-LOC105377182-GGT3P.NCBI.                                              ------------------------------------------------------------ 57310
human.FAM230J-LOC105372942-GGTLC5P.NCBI.rev.compl                                   ------------------------------------------------------------ 46364
human.FAM230A.ncRNA.18487127.18500594-GGTLC3.18516335.18518165.rev.compl.REF        ------------------------------------------------------------ 31039

chimp.LOC112206744-LOC107973052-GGT2.revcompl                                       ggaagggtgg-cacctccccaggcttggtgggcctggggccgatagtgtgatacatttga 61251
chimp54546-135457.revcompl.Pan.troglodytes.clone.rp43-41g5.GenBank:AC099533.36      ------------------------------------------------------------ 66783
human.FAM30B-LOC105372935-GGT2.NCBI.                                                ggaagggtggccacctccccaggcttggtgggcctggggccgatagtgtgatacatttga 60289
human.FAM230E-LOC105377182-GGT3P.NCBI.                                              ------------------------------------------------------------ 57310
human.FAM230J-LOC105372942-GGTLC5P.NCBI.rev.compl                                   ------------------------------------------------------------ 46364
human.FAM230A.ncRNA.18487127.18500594-GGTLC3.18516335.18518165.rev.compl.REF        ------------------------------------------------------------ 31039

chimp.LOC112206744-LOC107973052-GGT2.revcompl                                       ccccctcccagccctggatgcagacaccaagagcagagagacctggcagtagtcatgcag 61311
chimp54546-135457.revcompl.Pan.troglodytes.clone.rp43-41g5.GenBank:AC099533.36      ------------------------------------------------------------ 66783
human.FAM30B-LOC105372935-GGT2.NCBI.                                                ccccctcccagccctggatgcagacaccaagagcagagagacctggcagtagccatgcag 60349
human.FAM230E-LOC105377182-GGT3P.NCBI.                                              ------------------------------------------------------------ 57310
human.FAM230J-LOC105372942-GGTLC5P.NCBI.rev.compl                                   ------------------------------------------------------------ 46364
human.FAM230A.ncRNA.18487127.18500594-GGTLC3.18516335.18518165.rev.compl.REF        ------------------------------------------------------------ 31039

chimp.LOC112206744-LOC107973052-GGT2.revcompl                                       cagcgcaccaccccacatccccagcacaatccaaagcagcccgtcatccccaccgtgacc 61371
chimp54546-135457.revcompl.Pan.troglodytes.clone.rp43-41g5.GenBank:AC099533.36      ------------------------------------------------------------ 66783
human.FAM30B-LOC105372935-GGT2.NCBI.                                                cagcgcaccaccccacatccccagcacaatccaaagcagcccctcatccccaccgtgacc 60409
human.FAM230E-LOC105377182-GGT3P.NCBI.                                              ------------------------------------------------------------ 57310
human.FAM230J-LOC105372942-GGTLC5P.NCBI.rev.compl                                   ------------------------------------------------------------ 46364
human.FAM230A.ncRNA.18487127.18500594-GGTLC3.18516335.18518165.rev.compl.REF        ------------------------------------------------------------ 31039

chimp.LOC112206744-LOC107973052-GGT2.revcompl                                       accacagcctgaatccaggtgccacctgtttctgacctgaactccctcacagcccctgcc 61431
chimp54546-135457.revcompl.Pan.troglodytes.clone.rp43-41g5.GenBank:AC099533.36      ------------------------------------------------------------ 66783
human.FAM30B-LOC105372935-GGT2.NCBI.                                                accacagcctgaatccaggcgccacctgtttctgacctgaactccctcacagcccctgcc 60469
human.FAM230E-LOC105377182-GGT3P.NCBI.                                              ------------------------------------------------------------ 57310
human.FAM230J-LOC105372942-GGTLC5P.NCBI.rev.compl                                   ------------------------------------------------------------ 46364
human.FAM230A.ncRNA.18487127.18500594-GGTLC3.18516335.18518165.rev.compl.REF        ------------------------------------------------------------ 31039

chimp.LOC112206744-LOC107973052-GGT2.revcompl                                       tgcactccctccctccaacatcgcctgcccttcagtcttccagaaagcagctagagggct 61491
chimp54546-135457.revcompl.Pan.troglodytes.clone.rp43-41g5.GenBank:AC099533.36      ------------------------------------------------------------ 66783
human.FAM30B-LOC105372935-GGT2.NCBI.                                                tgcactccctccctccaacatcacctgcccttcagtcttccagaaagcagctagagggct 60529
human.FAM230E-LOC105377182-GGT3P.NCBI.                                              ------------------------------------------------------------ 57310
human.FAM230J-LOC105372942-GGTLC5P.NCBI.rev.compl                                   ------------------------------------------------------------ 46364
human.FAM230A.ncRNA.18487127.18500594-GGTLC3.18516335.18518165.rev.compl.REF        ------------------------------------------------------------ 31039

chimp.LOC112206744-LOC107973052-GGT2.revcompl                                       ctgtctgtccaactgcagaacaggccctgcctcctccctgtcccgttggacagctcacac 61551
chimp54546-135457.revcompl.Pan.troglodytes.clone.rp43-41g5.GenBank:AC099533.36      ------------------------------------------------------------ 66783
human.FAM30B-LOC105372935-GGT2.NCBI.                                                ctgtctgtccaactgcagaacaggccctgcctcctccctgccctgttggacagctcacac 60589
human.FAM230E-LOC105377182-GGT3P.NCBI.                                              ------------------------------------------------------------ 57310
human.FAM230J-LOC105372942-GGTLC5P.NCBI.rev.compl                                   ------------------------------------------------------------ 46364
human.FAM230A.ncRNA.18487127.18500594-GGTLC3.18516335.18518165.rev.compl.REF        ------------------------------------------------------------ 31039

chimp.LOC112206744-LOC107973052-GGT2.revcompl                                       ccttcaccaggcctgacagcactcttgccactccaacaccctgggtcccagccgggagtc 61611
chimp54546-135457.revcompl.Pan.troglodytes.clone.rp43-41g5.GenBank:AC099533.36      ------------------------------------------------------------ 66783
human.FAM30B-LOC105372935-GGT2.NCBI.                                                ccttcaccaggcctgacagcgctcttgccactccaacaccccgggtcccagccgggagtc 60649
human.FAM230E-LOC105377182-GGT3P.NCBI.                                              ------------------------------------------------------------ 57310
human.FAM230J-LOC105372942-GGTLC5P.NCBI.rev.compl                                   ------------------------------------------------------------ 46364
human.FAM230A.ncRNA.18487127.18500594-GGTLC3.18516335.18518165.rev.compl.REF        ------------------------------------------------------------ 31039

chimp.LOC112206744-LOC107973052-GGT2.revcompl                                       caggtcagggttaagggttcctgatagagacaccgattcctggaggtccaaagagcctca 61671
chimp54546-135457.revcompl.Pan.troglodytes.clone.rp43-41g5.GenBank:AC099533.36      ------------------------------------------------------------ 66783
human.FAM30B-LOC105372935-GGT2.NCBI.                                                cgggtcagggttaagggttcctggtagagacaccgattcctggaggtccaaagagcctca 60709
human.FAM230E-LOC105377182-GGT3P.NCBI.                                              ------------------------------------------------------------ 57310
human.FAM230J-LOC105372942-GGTLC5P.NCBI.rev.compl                                   ------------------------------------------------------------ 46364
human.FAM230A.ncRNA.18487127.18500594-GGTLC3.18516335.18518165.rev.compl.REF        ------------------------------------------------------------ 31039

chimp.LOC112206744-LOC107973052-GGT2.revcompl                                       ggagctgggccagcaatatgcagcatctattatggacacagaacattcccatcacatggc 61731
chimp54546-135457.revcompl.Pan.troglodytes.clone.rp43-41g5.GenBank:AC099533.36      ---------------------------------------------------------GGC 66786
human.FAM30B-LOC105372935-GGT2.NCBI.                                                ggagctgggccagcaatatgcagcatctattatggacacagaacattcccatcacatggc 60769
human.FAM230E-LOC105377182-GGT3P.NCBI.                                              ------------------------------------------------------------ 57310
human.FAM230J-LOC105372942-GGTLC5P.NCBI.rev.compl                                   ------------------------------------------------------------ 46364
human.FAM230A.ncRNA.18487127.18500594-GGTLC3.18516335.18518165.rev.compl.REF        ------------------------------------------------------------ 31039

chimp.LOC112206744-LOC107973052-GGT2.revcompl                                       cgggtgcagtggctcacgcctataatcccag----------------------------- 61762
chimp54546-135457.revcompl.Pan.troglodytes.clone.rp43-41g5.GenBank:AC099533.36      TGGGAGTGGTGGCTTAGGCCTATAATCCCAGCACTTTGGGAGACAGAGGTAGGCCAATCA 66846
human.FAM30B-LOC105372935-GGT2.NCBI.                                                cgggtgcagtggctcacgcctttaatcccagcactttgggaggcagaggcaggtggatca 60829
human.FAM230E-LOC105377182-GGT3P.NCBI.                                              ------------------------------------------------------------ 57310
human.FAM230J-LOC105372942-GGTLC5P.NCBI.rev.compl                                   ------------------------------------------------------------ 46364
human.FAM230A.ncRNA.18487127.18500594-GGTLC3.18516335.18518165.rev.compl.REF        ------------------------------------------------------------ 31039

chimp.LOC112206744-LOC107973052-GGT2.revcompl                                       ------------------------------------------------------------ 61762
chimp54546-135457.revcompl.Pan.troglodytes.clone.rp43-41g5.GenBank:AC099533.36      CATGAGGCCAGGAGTTCAAGACCAGCCTGGCCAACATGG-CAGAACCCCATCTCTACTAA 66905
human.FAM30B-LOC105372935-GGT2.NCBI.                                                cctgaggtgaagagttcgagaccagtctggccaacatggtgaaaccccatctccactaa 60889
human.FAM230E-LOC105377182-GGT3P.NCBI.                                              ------------------------------------------------------------ 57310
human.FAM230J-LOC105372942-GGTLC5P.NCBI.rev.compl                                   ------------------------------------------------------------ 46364
human.FAM230A.ncRNA.18487127.18500594-GGTLC3.18516335.18518165.rev.compl.REF        ------------------------------------------------------------ 31039

chimp.LOC112206744-LOC107973052-GGT2.revcompl                                       ------------------------------------------------------------ 61762
chimp54546-135457.revcompl.Pan.troglodytes.clone.rp43-41g5.GenBank:AC099533.36      AAATAC-AAAAATGAGTTAGGCGTGGTG----------GTAATCCCAGTTACTCGAGAGG 66954
human.FAM30B-LOC105372935-GGT2.NCBI.                                                aaatacaaaaaattagccaggcatggtggcaggtgcctgtaatcccagctactc-aggag 60948
human.FAM230E-LOC105377182-GGT3P.NCBI.                                              ------------------------------------------------------------ 57310
human.FAM230J-LOC105372942-GGTLC5P.NCBI.rev.compl                                   ------------------------------------------------------------ 46364
human.FAM230A.ncRNA.18487127.18500594-GGTLC3.18516335.18518165.rev.compl.REF        ------------------------------------------------------------ 31039

chimp.LOC112206744-LOC107973052-GGT2.revcompl                                       ------------------------------------------------------------ 61762
chimp54546-135457.revcompl.Pan.troglodytes.clone.rp43-41g5.GenBank:AC099533.36      CTGAGGCATGAGAATCGCTTGAACCTGGGAGGTGGAGGCTACAGTGAGTTGAGATTGCAC 67014
human.FAM30B-LOC105372935-GGT2.NCBI.                                                ctgaggcaggagaattgcttgaacccgggaggtggaggttgcagtgaggcaagattgcac 61008
human.FAM230E-LOC105377182-GGT3P.NCBI.                                              ------------------------------------------------------------ 57310
human.FAM230J-LOC105372942-GGTLC5P.NCBI.rev.compl                                   ------------------------------------------------------------ 46364
human.FAM230A.ncRNA.18487127.18500594-GGTLC3.18516335.18518165.rev.compl.REF        ------------------------------------------------------------ 31039

chimp.LOC112206744-LOC107973052-GGT2.revcompl                                       ------------------------------------------------------------ 61762
chimp54546-135457.revcompl.Pan.troglodytes.clone.rp43-41g5.GenBank:AC099533.36      CACTGAACTCCAGCCTGGGC-------------------AACAGATAGAGACTCAAAAAA 67055
human.FAM30B-LOC105372935-GGT2.NCBI.                                                cactgcactccagcctgggccacaagagtgaaactccgtcacacacacacacacacacac 61068
human.FAM230E-LOC105377182-GGT3P.NCBI.                                              ------------------------------------------------------------ 57310
human.FAM230J-LOC105372942-GGTLC5P.NCBI.rev.compl                                   ------------------------------------------------------------ 46364
human.FAM230A.ncRNA.18487127.18500594-GGTLC3.18516335.18518165.rev.compl.REF        ------------------------------------------------------------ 31039

chimp.LOC112206744-LOC107973052-GGT2.revcompl                                       ------------------------------------------------------------ 61762
chimp54546-135457.revcompl.Pan.troglodytes.clone.rp43-41g5.GenBank:AC099533.36      AAAAAGTCTCAAAAAAAAATGGAATTAGGAACTCAATTTGAACAGCACACTAGACACAAA 67115
human.FAM30B-LOC105372935-GGT2.NCBI.                                                acacaaaaaaaaaaaaaaagaaaaaaaaggtctcctgctggaacacagactagttagaga 61128
human.FAM230E-LOC105377182-GGT3P.NCBI.                                              ------------------------------------------------------------ 57310
human.FAM230J-LOC105372942-GGTLC5P.NCBI.rev.compl                                   ------------------------------------------------------------ 46364
human.FAM230A.ncRNA.18487127.18500594-GGTLC3.18516335.18518165.rev.compl.REF        ------------------------------------------------------------ 31039

chimp.LOC112206744-LOC107973052-GGT2.revcompl                                       ------------------------------------------------------------ 61762
chimp54546-135457.revcompl.Pan.troglodytes.clone.rp43-41g5.GenBank:AC099533.36      CATAAAAAAAAATAATGAACTGGTATTTATTTATTTA----------------------- 67152
human.FAM30B-LOC105372935-GGT2.NCBI.                                                aaggaaaaataaacaaatgatagtatgtgtattaataaaagaactagcaacacccactgc 61188
human.FAM230E-LOC105377182-GGT3P.NCBI.                                              ------------------------------------------------------------ 57310
human.FAM230J-LOC105372942-GGTLC5P.NCBI.rev.compl                                   ------------------------------------------------------------ 46364
human.FAM230A.ncRNA.18487127.18500594-GGTLC3.18516335.18518165.rev.compl.REF        ------------------------------------------------------------ 31039
```

```
chimp.LOC112206744-LOC107973052-GGT2.revcompl                                   ------------------------------------------------------------ 61762
chimp54546-135457.revcompl.Pan.troglodytes.clone.rp43-41g5.GenBank:AC099533.36  ------------------------------------------------------------ 67152
human.FAM30B-LOC105372935-GGT2.NCBI.                                            ttagttgtgataataaaaactggacattaatataggcagaaaaacagaacagccttgata 61248
human.FAM230E-LOC105377182-GGT3P.NCBI.                                          ------------------------------------------------------------ 57310
human.FAM230J-LOC105372942-GGTLC5P.NCBI.rev.compl                               ------------------------------------------------------------ 46364
human.FAM230A.ncRNA.18487127.18500594-GGTLC3.18516335.18518165.rev.compl.REF    ------------------------------------------------------------ 31039

chimp.LOC112206744-LOC107973052-GGT2.revcompl                                   ------------------------------------------------------------ 61762
chimp54546-135457.revcompl.Pan.troglodytes.clone.rp43-41g5.GenBank:AC099533.36  ------------------------------------------------------------ 67152
human.FAM30B-LOC105372935-GGT2.NCBI.                                            tggttaaccctttgacactggcaaacactgtgaccagtgctgcccacactggaagctcct 61308
human.FAM230E-LOC105377182-GGT3P.NCBI.                                          ------------------------------------------------------------ 57310
human.FAM230J-LOC105372942-GGTLC5P.NCBI.rev.compl                               ------------------------------------------------------------ 46364
human.FAM230A.ncRNA.18487127.18500594-GGTLC3.18516335.18518165.rev.compl.REF    ------------------------------------------------------------ 31039

chimp.LOC112206744-LOC107973052-GGT2.revcompl                                   ------------------------------------------------------------ 61762
chimp54546-135457.revcompl.Pan.troglodytes.clone.rp43-41g5.GenBank:AC099533.36  ------------------------------------------------------------ 67152
human.FAM30B-LOC105372935-GGT2.NCBI.                                            gcatccctcttcctcatgtttccttcagcattaaggagcaacaagggagacagccagttc 61368
human.FAM230E-LOC105377182-GGT3P.NCBI.                                          ------------------------------------------------------------ 57310
human.FAM230J-LOC105372942-GGTLC5P.NCBI.rev.compl                               ------------------------------------------------------------ 46364
human.FAM230A.ncRNA.18487127.18500594-GGTLC3.18516335.18518165.rev.compl.REF    ------------------------------------------------------------ 31039

chimp.LOC112206744-LOC107973052-GGT2.revcompl                                   ------------------------------------------------------------ 61762
chimp54546-135457.revcompl.Pan.troglodytes.clone.rp43-41g5.GenBank:AC099533.36  ------------------------------------------------------------ 67152
human.FAM30B-LOC105372935-GGT2.NCBI.                                            atagttccttacgcatggagccaaaggaccttcaatgtacaaggtctgagcaaggacccg 61428
human.FAM230E-LOC105377182-GGT3P.NCBI.                                          ------------------------------------------------------------ 57310
human.FAM230J-LOC105372942-GGTLC5P.NCBI.rev.compl                               ------------------------------------------------------------ 46364
human.FAM230A.ncRNA.18487127.18500594-GGTLC3.18516335.18518165.rev.compl.REF    ------------------------------------------------------------ 31039

chimp.LOC112206744-LOC107973052-GGT2.revcompl                                   ------------------------------------------------------------ 61762
chimp54546-135457.revcompl.Pan.troglodytes.clone.rp43-41g5.GenBank:AC099533.36  ------------------------------------------------------------ 67152
human.FAM30B-LOC105372935-GGT2.NCBI.                                            cagccacatgtgcttcctgcttcagcagtgccccgtgggtctcagagctacccaaaggcc 61488
human.FAM230E-LOC105377182-GGT3P.NCBI.                                          ------------------------------------------------------------ 57310
human.FAM230J-LOC105372942-GGTLC5P.NCBI.rev.compl                               ------------------------------------------------------------ 46364
human.FAM230A.ncRNA.18487127.18500594-GGTLC3.18516335.18518165.rev.compl.REF    ------------------------------------------------------------ 31039

chimp.LOC112206744-LOC107973052-GGT2.revcompl                                   ------------------------------------------------------------ 61762
chimp54546-135457.revcompl.Pan.troglodytes.clone.rp43-41g5.GenBank:AC099533.36  ------------------------------------------------------------ 67152
human.FAM30B-LOC105372935-GGT2.NCBI.                                            tatccttccgagaggtttctcctctctcccaactgatgtcatacgtctcatcttccttgt 61548
human.FAM230E-LOC105377182-GGT3P.NCBI.                                          ------------------------------------------------------------ 57310
human.FAM230J-LOC105372942-GGTLC5P.NCBI.rev.compl                               ------------------------------------------------------------ 46364
human.FAM230A.ncRNA.18487127.18500594-GGTLC3.18516335.18518165.rev.compl.REF    ------------------------------------------------------------ 31039

chimp.LOC112206744-LOC107973052-GGT2.revcompl                                   ------------------------------------------------------------ 61762
chimp54546-135457.revcompl.Pan.troglodytes.clone.rp43-41g5.GenBank:AC099533.36  ------------------------------------------------------------ 67152
human.FAM30B-LOC105372935-GGT2.NCBI.                                            cattcagatcatcaaccacaaacgtccttgccttatattttcatatcccttttcaccagt 61608
human.FAM230E-LOC105377182-GGT3P.NCBI.                                          ------------------------------------------------------------ 57310
human.FAM230J-LOC105372942-GGTLC5P.NCBI.rev.compl                               ------------------------------------------------------------ 46364
human.FAM230A.ncRNA.18487127.18500594-GGTLC3.18516335.18518165.rev.compl.REF    ------------------------------------------------------------ 31039

chimp.LOC112206744-LOC107973052-GGT2.revcompl                                   ------------------------------------------------------------ 61762
chimp54546-135457.revcompl.Pan.troglodytes.clone.rp43-41g5.GenBank:AC099533.36  ------------------------------------------------------------ 67152
human.FAM30B-LOC105372935-GGT2.NCBI.                                            tacagggttagttaacttacgaaattcttaacatctgcattagatctttttaaagtttca 61668
human.FAM230E-LOC105377182-GGT3P.NCBI.                                          ------------------------------------------------------------ 57310
human.FAM230J-LOC105372942-GGTLC5P.NCBI.rev.compl                               ------------------------------------------------------------ 46364
human.FAM230A.ncRNA.18487127.18500594-GGTLC3.18516335.18518165.rev.compl.REF    ------------------------------------------------------------ 31039

chimp.LOC112206744-LOC107973052-GGT2.revcompl                                   ------------------------------------------------------------ 61762
chimp54546-135457.revcompl.Pan.troglodytes.clone.rp43-41g5.GenBank:AC099533.36  ------------------------------------------------------------ 67152
human.FAM30B-LOC105372935-GGT2.NCBI.                                            ccctcaaccacctattatttagaagtgaacacagaaatttagtttccttgtgctatctgt 61728
human.FAM230E-LOC105377182-GGT3P.NCBI.                                          ------------------------------------------------------------ 57310
human.FAM230J-LOC105372942-GGTLC5P.NCBI.rev.compl                               ------------------------------------------------------------ 46364
human.FAM230A.ncRNA.18487127.18500594-GGTLC3.18516335.18518165.rev.compl.REF    ------------------------------------------------------------ 31039

chimp.LOC112206744-LOC107973052-GGT2.revcompl                                   ------------------------------------------------------------ 61762
chimp54546-135457.revcompl.Pan.troglodytes.clone.rp43-41g5.GenBank:AC099533.36  ------------------------------------------------------------ 67152
human.FAM30B-LOC105372935-GGT2.NCBI.                                            tgacccctaaaatatgctgggagttttgggattttttttaaagtcaaatgcatggcataa 61788
human.FAM230E-LOC105377182-GGT3P.NCBI.                                          ------------------------------------------------------------ 57310
human.FAM230J-LOC105372942-GGTLC5P.NCBI.rev.compl                               ------------------------------------------------------------ 46364
human.FAM230A.ncRNA.18487127.18500594-GGTLC3.18516335.18518165.rev.compl.REF    ------------------------------------------------------------ 31039

chimp.LOC112206744-LOC107973052-GGT2.revcompl                                   ------------------------------------------------------------ 61762
chimp54546-135457.revcompl.Pan.troglodytes.clone.rp43-41g5.GenBank:AC099533.36  ------------------------------------------------------------ 67152
human.FAM30B-LOC105372935-GGT2.NCBI.                                            agcaaaattacactactaaagaactgagtcaggccagacgctggcaacgtgaagacagct 61848
human.FAM230E-LOC105377182-GGT3P.NCBI.                                          ------------------------------------------------------------ 57310
human.FAM230J-LOC105372942-GGTLC5P.NCBI.rev.compl                               ------------------------------------------------------------ 46364
human.FAM230A.ncRNA.18487127.18500594-GGTLC3.18516335.18518165.rev.compl.REF    ------------------------------------------------------------ 31039

chimp.LOC112206744-LOC107973052-GGT2.revcompl                                   ------------------------------------------------------------ 61762
chimp54546-135457.revcompl.Pan.troglodytes.clone.rp43-41g5.GenBank:AC099533.36  ------------------------------------------------------------ 67152
human.FAM30B-LOC105372935-GGT2.NCBI.                                            tctccttacccactaggttcatctttgcactgttaactcccaaagtattgcttctcggtg 61908
human.FAM230E-LOC105377182-GGT3P.NCBI.                                          ------------------------------------------------------------ 57310
human.FAM230J-LOC105372942-GGTLC5P.NCBI.rev.compl                               ------------------------------------------------------------ 46364
human.FAM230A.ncRNA.18487127.18500594-GGTLC3.18516335.18518165.rev.compl.REF    ------------------------------------------------------------ 31039

chimp.LOC112206744-LOC107973052-GGT2.revcompl                                   ------------------------------------------------------------ 61762
chimp54546-135457.revcompl.Pan.troglodytes.clone.rp43-41g5.GenBank:AC099533.36  ------------------------------------------------------------ 67152
human.FAM30B-LOC105372935-GGT2.NCBI.                                            ctgggtgtgtggcattcatattccccggcatcccgggcctgaagatctgtgatgtgcaat 61968
human.FAM230E-LOC105377182-GGT3P.NCBI.                                          ------------------------------------------------------------ 57310
human.FAM230J-LOC105372942-GGTLC5P.NCBI.rev.compl                               ------------------------------------------------------------ 46364
human.FAM230A.ncRNA.18487127.18500594-GGTLC3.18516335.18518165.rev.compl.REF    ------------------------------------------------------------ 31039

chimp.LOC112206744-LOC107973052-GGT2.revcompl                                   ------------------------------------------------------------ 61762
chimp54546-135457.revcompl.Pan.troglodytes.clone.rp43-41g5.GenBank:AC099533.36  ------------------------------------------------------------ 67152
human.FAM30B-LOC105372935-GGT2.NCBI.                                            agggttgggttcccctggactctttctatgaagatcttccctccgcggacgcgctgggtg 62028
human.FAM230E-LOC105377182-GGT3P.NCBI.                                          ------------------------------------------------------------ 57310
human.FAM230J-LOC105372942-GGTLC5P.NCBI.rev.compl                               ------------------------------------------------------------ 46364
human.FAM230A.ncRNA.18487127.18500594-GGTLC3.18516335.18518165.rev.compl.REF    ------------------------------------------------------------ 31039

chimp.LOC112206744-LOC107973052-GGT2.revcompl                                   ------------------------------------------------------------ 61762
chimp54546-135457.revcompl.Pan.troglodytes.clone.rp43-41g5.GenBank:AC099533.36  ------------------------------------------------------------ 67152
human.FAM30B-LOC105372935-GGT2.NCBI.                                            tagatggcataggggaaggaagagtccatggtgctgacgatctgcacctctcgctctggc 62088
human.FAM230E-LOC105377182-GGT3P.NCBI.                                          ------------------------------------------------------------ 57310
human.FAM230J-LOC105372942-GGTLC5P.NCBI.rev.compl                               ------------------------------------------------------------ 46364
human.FAM230A.ncRNA.18487127.18500594-GGTLC3.18516335.18518165.rev.compl.REF    ------------------------------------------------------------ 31039

chimp.LOC112206744-LOC107973052-GGT2.revcompl                                   ------------------------------------------------------------ 61762
chimp54546-135457.revcompl.Pan.troglodytes.clone.rp43-41g5.GenBank:AC099533.36  ------------------------------------------------------------ 67152
human.FAM30B-LOC105372935-GGT2.NCBI.                                            gacgaaggcaggtaaatggaccactggaaattctgttcagaaggtccctggtagccactc 62148
human.FAM230E-LOC105377182-GGT3P.NCBI.                                          ------------------------------------------------------------ 57310
human.FAM230J-LOC105372942-GGTLC5P.NCBI.rev.compl                               ------------------------------------------------------------ 46364
human.FAM230A.ncRNA.18487127.18500594-GGTLC3.18516335.18518165.rev.compl.REF    ------------------------------------------------------------ 31039

chimp.LOC112206744-LOC107973052-GGT2.revcompl                                   ------------------------------------------------------------ 61762
chimp54546-135457.revcompl.Pan.troglodytes.clone.rp43-41g5.GenBank:AC099533.36  ------------------------------------------------------------ 67152
human.FAM30B-LOC105372935-GGT2.NCBI.                                            acattgcaccagatagtgatgtgggagccctccgtgcggtacaagggtccttcctgaacg 62208
human.FAM230E-LOC105377182-GGT3P.NCBI.                                          ------------------------------------------------------------ 57310
human.FAM230J-LOC105372942-GGTLC5P.NCBI.rev.compl                               ------------------------------------------------------------ 46364
human.FAM230A.ncRNA.18487127.18500594-GGTLC3.18516335.18518165.rev.compl.REF    ------------------------------------------------------------ 31039

chimp.LOC112206744-LOC107973052-GGT2.revcompl                                   ------------------------------------------------------------ 61762
chimp54546-135457.revcompl.Pan.troglodytes.clone.rp43-41g5.GenBank:AC099533.36  ------------------------------------------------------------ 67152
human.FAM30B-LOC105372935-GGT2.NCBI.                                            gtgacctgccgctgtgctgacaccacacctacgagggagagaaacacacgcaacatgctc 62268
human.FAM230E-LOC105377182-GGT3P.NCBI.                                          ------------------------------------------------------------ 57310
human.FAM230J-LOC105372942-GGTLC5P.NCBI.rev.compl                               ------------------------------------------------------------ 46364
human.FAM230A.ncRNA.18487127.18500594-GGTLC3.18516335.18518165.rev.compl.REF    ------------------------------------------------------------ 31039
```

```
chimp.LOC112206744-LOC107973052-GGT2.revcompl                                     ------------------------------------------------------------ 61762
chimp54546-135457.revcompl.Pan.troglodytes.clone.rp43-41g5.GenBank:AC099533.36    ------------------------------------------------------------ 67152
human.FAM30B-LOC105372935-GGT2.NCBI.                                              acttacttctcagaccaaaatgcaaagtaggcagcaatctccaaagggtttgttatttgt 62328
human.FAM230E-LOC105377182-GGT3P.NCBI.                                            ------------------------------------------------------------ 57310
human.FAM230J-LOC105372942-GGTLC5P.NCBI.rev.compl                                 ------------------------------------------------------------ 46364
human.FAM230A.ncRNA.18487127.18500594-GGTLC3.18516335.18518165.rev.compl.REF      ------------------------------------------------------------ 31039

chimp.LOC112206744-LOC107973052-GGT2.revcompl                                     ------------------------------------------------------------ 61762
chimp54546-135457.revcompl.Pan.troglodytes.clone.rp43-41g5.GenBank:AC099533.36    ------------------------------------------------------------ 67152
human.FAM30B-LOC105372935-GGT2.NCBI.                                              gtgacatgataataatataaacagcttactggccctttcaaaggcactctgtgatttata 62388
human.FAM230E-LOC105377182-GGT3P.NCBI.                                            ------------------------------------------------------------ 57310
human.FAM230J-LOC105372942-GGTLC5P.NCBI.rev.compl                                 ------------------------------------------------------------ 46364
human.FAM230A.ncRNA.18487127.18500594-GGTLC3.18516335.18518165.rev.compl.REF      ------------------------------------------------------------ 31039

chimp.LOC112206744-LOC107973052-GGT2.revcompl                                     ------------------------------------------------------------ 61762
chimp54546-135457.revcompl.Pan.troglodytes.clone.rp43-41g5.GenBank:AC099533.36    ------------------------------------------------------------ 67152
human.FAM30B-LOC105372935-GGT2.NCBI.                                              aatattaattaaaacactctgtggtaaagcactgtccctaatttgcatatatgggaattt 62448
human.FAM230E-LOC105377182-GGT3P.NCBI.                                            ------------------------------------------------------------ 57310
human.FAM230J-LOC105372942-GGTLC5P.NCBI.rev.compl                                 ------------------------------------------------------------ 46364
human.FAM230A.ncRNA.18487127.18500594-GGTLC3.18516335.18518165.rev.compl.REF      ------------------------------------------------------------ 31039

chimp.LOC112206744-LOC107973052-GGT2.revcompl                                     ------------------------------------------------------------ 61762
chimp54546-135457.revcompl.Pan.troglodytes.clone.rp43-41g5.GenBank:AC099533.36    ------------------------------------------------------------ 67152
human.FAM30B-LOC105372935-GGT2.NCBI.                                              gatcatgccaagatgtgcttttgttacttattcaacagctgacccttgggaatttcattt 62508
human.FAM230E-LOC105377182-GGT3P.NCBI.                                            ------------------------------------------------------------ 57310
human.FAM230J-LOC105372942-GGTLC5P.NCBI.rev.compl                                 ------------------------------------------------------------ 46364
human.FAM230A.ncRNA.18487127.18500594-GGTLC3.18516335.18518165.rev.compl.REF      ------------------------------------------------------------ 31039

chimp.LOC112206744-LOC107973052-GGT2.revcompl                                     ------------------------------------------------------------ 61762
chimp54546-135457.revcompl.Pan.troglodytes.clone.rp43-41g5.GenBank:AC099533.36    ------------------------------------------------------------ 67152
human.FAM30B-LOC105372935-GGT2.NCBI.                                              agcccctctgtttgtcatgtatacggcagttttatatctatttattaatgctgtgtataa 62568
human.FAM230E-LOC105377182-GGT3P.NCBI.                                            ------------------------------------------------------------ 57310
human.FAM230J-LOC105372942-GGTLC5P.NCBI.rev.compl                                 ------------------------------------------------------------ 46364
human.FAM230A.ncRNA.18487127.18500594-GGTLC3.18516335.18518165.rev.compl.REF      ------------------------------------------------------------ 31039

chimp.LOC112206744-LOC107973052-GGT2.revcompl                                     ------------------------------------------------------------ 61762
chimp54546-135457.revcompl.Pan.troglodytes.clone.rp43-41g5.GenBank:AC099533.36    ------------------------------------------------------------ 67152
human.FAM30B-LOC105372935-GGT2.NCBI.                                              agtttttaataaaataagcaggaaaagttgaatccttggcacaaattcagatgaaaacaa 62628
human.FAM230E-LOC105377182-GGT3P.NCBI.                                            ------------------------------------------------------------ 57310
human.FAM230J-LOC105372942-GGTLC5P.NCBI.rev.compl                                 ------------------------------------------------------------ 46364
human.FAM230A.ncRNA.18487127.18500594-GGTLC3.18516335.18518165.rev.compl.REF      ------------------------------------------------------------ 31039

chimp.LOC112206744-LOC107973052-GGT2.revcompl                                     ------------------------------------------------------------ 61762
chimp54546-135457.revcompl.Pan.troglodytes.clone.rp43-41g5.GenBank:AC099533.36    ------------------------------------------------------------ 67152
human.FAM30B-LOC105372935-GGT2.NCBI.                                              ataaaagcaaccatctaggaatctggctgaaattaaatgcttcttccttggtccgagtgc 62688
human.FAM230E-LOC105377182-GGT3P.NCBI.                                            ------------------------------------------------------------ 57310
human.FAM230J-LOC105372942-GGTLC5P.NCBI.rev.compl                                 ------------------------------------------------------------ 46364
human.FAM230A.ncRNA.18487127.18500594-GGTLC3.18516335.18518165.rev.compl.REF      ------------------------------------------------------------ 31039

chimp.LOC112206744-LOC107973052-GGT2.revcompl                                     ------------------------------------------------------------ 61762
chimp54546-135457.revcompl.Pan.troglodytes.clone.rp43-41g5.GenBank:AC099533.36    ------------------------------------------------------------ 67152
human.FAM30B-LOC105372935-GGT2.NCBI.                                              tggtgggagggagtcctgggagcaccttttttactggtgctgcacccttcattttcccatg 62748
human.FAM230E-LOC105377182-GGT3P.NCBI.                                            ------------------------------------------------------------ 57310
human.FAM230J-LOC105372942-GGTLC5P.NCBI.rev.compl                                 ------------------------------------------------------------ 46364
human.FAM230A.ncRNA.18487127.18500594-GGTLC3.18516335.18518165.rev.compl.REF      ------------------------------------------------------------ 31039

chimp.LOC112206744-LOC107973052-GGT2.revcompl                                     ------------------------------------------------------------ 61762
chimp54546-135457.revcompl.Pan.troglodytes.clone.rp43-41g5.GenBank:AC099533.36    ------------------------------------------------------------ 67152
human.FAM30B-LOC105372935-GGT2.NCBI.                                              tagctgctccccaacaccaccccaaggagcattccttagtttctcttccctccataaatg 62808
human.FAM230E-LOC105377182-GGT3P.NCBI.                                            ------------------------------------------------------------ 57310
human.FAM230J-LOC105372942-GGTLC5P.NCBI.rev.compl                                 ------------------------------------------------------------ 46364
human.FAM230A.ncRNA.18487127.18500594-GGTLC3.18516335.18518165.rev.compl.REF      ------------------------------------------------------------ 31039

chimp.LOC112206744-LOC107973052-GGT2.revcompl                                     ------------------------------------------------------------ 61762
chimp54546-135457.revcompl.Pan.troglodytes.clone.rp43-41g5.GenBank:AC099533.36    ------------------------------------------------------------ 67152
human.FAM30B-LOC105372935-GGT2.NCBI.                                              gaggcagagctgagctcctgcaggctgctatcgttttaagctgagcacacccaaatacaa 62868
human.FAM230E-LOC105377182-GGT3P.NCBI.                                            ------------------------------------------------------------ 57310
human.FAM230J-LOC105372942-GGTLC5P.NCBI.rev.compl                                 ------------------------------------------------------------ 46364
human.FAM230A.ncRNA.18487127.18500594-GGTLC3.18516335.18518165.rev.compl.REF      ------------------------------------------------------------ 31039

chimp.LOC112206744-LOC107973052-GGT2.revcompl                                     ------------------------------------------------------------ 61762
chimp54546-135457.revcompl.Pan.troglodytes.clone.rp43-41g5.GenBank:AC099533.36    ------------------------------------------------------------ 67152
human.FAM30B-LOC105372935-GGT2.NCBI.                                              atgtgaagggctgaaacagatccctgatgcccagattcccacaaactgacaaagggggga 62928
human.FAM230E-LOC105377182-GGT3P.NCBI.                                            ------------------------------------------------------------ 57310
human.FAM230J-LOC105372942-GGTLC5P.NCBI.rev.compl                                 ------------------------------------------------------------ 46364
human.FAM230A.ncRNA.18487127.18500594-GGTLC3.18516335.18518165.rev.compl.REF      ------------------------------------------------------------ 31039

chimp.LOC112206744-LOC107973052-GGT2.revcompl                                     ------------------------------------------------------------ 61762
chimp54546-135457.revcompl.Pan.troglodytes.clone.rp43-41g5.GenBank:AC099533.36    ------------------------------------------------------------ 67152
human.FAM30B-LOC105372935-GGT2.NCBI.                                              actaacagtgcccagctaaacctgtgacaaggacagtcaggctacccttgagtttcccac 62988
human.FAM230E-LOC105377182-GGT3P.NCBI.                                            ------------------------------------------------------------ 57310
human.FAM230J-LOC105372942-GGTLC5P.NCBI.rev.compl                                 ------------------------------------------------------------ 46364
human.FAM230A.ncRNA.18487127.18500594-GGTLC3.18516335.18518165.rev.compl.REF      ------------------------------------------------------------ 31039

chimp.LOC112206744-LOC107973052-GGT2.revcompl                                     ------------------------------------------------------------ 61762
chimp54546-135457.revcompl.Pan.troglodytes.clone.rp43-41g5.GenBank:AC099533.36    ------------------------------------------------------------ 67152
human.FAM30B-LOC105372935-GGT2.NCBI.                                              tcaagactcccccgctgacctcctgccttctggctcagatactgttctccaaggtcactt 63048
human.FAM230E-LOC105377182-GGT3P.NCBI.                                            ------------------------------------------------------------ 57310
human.FAM230J-LOC105372942-GGTLC5P.NCBI.rev.compl                                 ------------------------------------------------------------ 46364
human.FAM230A.ncRNA.18487127.18500594-GGTLC3.18516335.18518165.rev.compl.REF      ------------------------------------------------------------ 31039

chimp.LOC112206744-LOC107973052-GGT2.revcompl                                     ------------------------------------------------------------ 61762
chimp54546-135457.revcompl.Pan.troglodytes.clone.rp43-41g5.GenBank:AC099533.36    ------------------------------------------------------------ 67152
human.FAM30B-LOC105372935-GGT2.NCBI.                                              ctgcctgccttctccatgttcttcttaaagtggcatggtgagtttaatttctcatttcgt 63108
human.FAM230E-LOC105377182-GGT3P.NCBI.                                            ------------------------------------------------------------ 57310
human.FAM230J-LOC105372942-GGTLC5P.NCBI.rev.compl                                 ------------------------------------------------------------ 46364
human.FAM230A.ncRNA.18487127.18500594-GGTLC3.18516335.18518165.rev.compl.REF      ------------------------------------------------------------ 31039

chimp.LOC112206744-LOC107973052-GGT2.revcompl                                     ------------------------------------------------------------ 61762
chimp54546-135457.revcompl.Pan.troglodytes.clone.rp43-41g5.GenBank:AC099533.36    ------------------------------------------------------------ 67152
human.FAM30B-LOC105372935-GGT2.NCBI.                                              gccaactcaacattgattgtgcctgagccactcagaaggtgggagggggcacacccctgc 63168
human.FAM230E-LOC105377182-GGT3P.NCBI.                                            ------------------------------------------------------------ 57310
human.FAM230J-LOC105372942-GGTLC5P.NCBI.rev.compl                                 ------------------------------------------------------------ 46364
human.FAM230A.ncRNA.18487127.18500594-GGTLC3.18516335.18518165.rev.compl.REF      ------------------------------------------------------------ 31039

chimp.LOC112206744-LOC107973052-GGT2.revcompl                                     ------------------------------------------------------------ 61762
chimp54546-135457.revcompl.Pan.troglodytes.clone.rp43-41g5.GenBank:AC099533.36    ------------------------------------------------------------ 67152
human.FAM30B-LOC105372935-GGT2.NCBI.                                              ctcttcatttcggcagtggtcacgtactgcaggttgtgaaaagcgcaggtgagttatagg 63228
human.FAM230E-LOC105377182-GGT3P.NCBI.                                            ------------------------------------------------------------ 57310
human.FAM230J-LOC105372942-GGTLC5P.NCBI.rev.compl                                 ------------------------------------------------------------ 46364
human.FAM230A.ncRNA.18487127.18500594-GGTLC3.18516335.18518165.rev.compl.REF      ------------------------------------------------------------ 31039

chimp.LOC112206744-LOC107973052-GGT2.revcompl                                     ------------------------------------------------------------ 61762
chimp54546-135457.revcompl.Pan.troglodytes.clone.rp43-41g5.GenBank:AC099533.36    ------------------------------------------------------------ 67152
human.FAM30B-LOC105372935-GGT2.NCBI.                                              cgcagctttgccaccaattagagctggttgtgtcatctcagacaacttgcttttcttctc 63288
human.FAM230E-LOC105377182-GGT3P.NCBI.                                            ------------------------------------------------------------ 57310
human.FAM230J-LOC105372942-GGTLC5P.NCBI.rev.compl                                 ------------------------------------------------------------ 46364
human.FAM230A.ncRNA.18487127.18500594-GGTLC3.18516335.18518165.rev.compl.REF      ------------------------------------------------------------ 31039

chimp.LOC112206744-LOC107973052-GGT2.revcompl                                     ------------------------------------------------------------ 61762
chimp54546-135457.revcompl.Pan.troglodytes.clone.rp43-41g5.GenBank:AC099533.36    ------------------------------------------------------------ 67152
human.FAM30B-LOC105372935-GGT2.NCBI.                                              cagaccttgatcttctcatctatcacatgatttctaagaacgctttagagagcactagca 63348
human.FAM230E-LOC105377182-GGT3P.NCBI.                                            ------------------------------------------------------------ 57310
human.FAM230J-LOC105372942-GGTLC5P.NCBI.rev.compl                                 ------------------------------------------------------------ 46364
human.FAM230A.ncRNA.18487127.18500594-GGTLC3.18516335.18518165.rev.compl.REF      ------------------------------------------------------------ 31039
```

```
chimp.LOC112206744-LOC107973052-GGT2.revcompl                                        ------------------------------------------------------------ 61762
chimp54546-135457.revcompl.Pan.troglodytes.clone.rp43-41g5.GenBank:AC099533.36       ------------------------------------------------------------ 67152
human.FAM30B-LOC105372935-GGT2.NCBI.                                                 tgggttccgatgctggctttgtcacttattaactgtgatcttggataagctgcttaacct 63408
human.FAM230E-LOC105377182-GGT3P.NCBI.                                               ------------------------------------------------------------ 57310
human.FAM230J-LOC105372942-GGTLC5P.NCBI.rev.compl                                    ------------------------------------------------------------ 46364
human.FAM230A.ncRNA.18487127.18500594-GGTLC3.18516335.18518165.rev.compl.REF         ------------------------------------------------------------ 31039

chimp.LOC112206744-LOC107973052-GGT2.revcompl                                        ------------------------------------------------------------ 61762
chimp54546-135457.revcompl.Pan.troglodytes.clone.rp43-41g5.GenBank:AC099533.36       ------------------------------------------------------------ 67152
human.FAM30B-LOC105372935-GGT2.NCBI.                                                 ctctaagcctcagttttctcatcggtaagatggggataataatgcctgtctcatagggtt 63468
human.FAM230E-LOC105377182-GGT3P.NCBI.                                               ------------------------------------------------------------ 57310
human.FAM230J-LOC105372942-GGTLC5P.NCBI.rev.compl                                    ------------------------------------------------------------ 46364
human.FAM230A.ncRNA.18487127.18500594-GGTLC3.18516335.18518165.rev.compl.REF         ------------------------------------------------------------ 31039

chimp.LOC112206744-LOC107973052-GGT2.revcompl                                        ------------------------------------------------------------ 61762
chimp54546-135457.revcompl.Pan.troglodytes.clone.rp43-41g5.GenBank:AC099533.36       ------------------------------------------------------------ 67152
human.FAM30B-LOC105372935-GGT2.NCBI.                                                 attgagaagatttagtaacatctgtaaagaccctagcacagagcctagcccacaatcagt 63528
human.FAM230E-LOC105377182-GGT3P.NCBI.                                               ------------------------------------------------------------ 57310
human.FAM230J-LOC105372942-GGTLC5P.NCBI.rev.compl                                    ------------------------------------------------------------ 46364
human.FAM230A.ncRNA.18487127.18500594-GGTLC3.18516335.18518165.rev.compl.REF         ------------------------------------------------------------ 31039

chimp.LOC112206744-LOC107973052-GGT2.revcompl                                        ------------------------------------------------------------ 61762
chimp54546-135457.revcompl.Pan.troglodytes.clone.rp43-41g5.GenBank:AC099533.36       ------------------------------------------------------------ 67152
human.FAM30B-LOC105372935-GGT2.NCBI.                                                 cctcaataaagagctgctggctaagacatatttctgctggaacattctgtggctctggat 63588
human.FAM230E-LOC105377182-GGT3P.NCBI.                                               ------------------------------------------------------------ 57310
human.FAM230J-LOC105372942-GGTLC5P.NCBI.rev.compl                                    ------------------------------------------------------------ 46364
human.FAM230A.ncRNA.18487127.18500594-GGTLC3.18516335.18518165.rev.compl.REF         ------------------------------------------------------------ 31039

chimp.LOC112206744-LOC107973052-GGT2.revcompl                                        ------------------------------------------------------------ 61762
chimp54546-135457.revcompl.Pan.troglodytes.clone.rp43-41g5.GenBank:AC099533.36       ------------------------------------------------------------ 67152
human.FAM30B-LOC105372935-GGT2.NCBI.                                                 ccatagaggacaggatttggccaatgactgcttaaggacatttcaacggccccttgacaa 63648
human.FAM230E-LOC105377182-GGT3P.NCBI.                                               ------------------------------------------------------------ 57310
human.FAM230J-LOC105372942-GGTLC5P.NCBI.rev.compl                                    ------------------------------------------------------------ 46364
human.FAM230A.ncRNA.18487127.18500594-GGTLC3.18516335.18518165.rev.compl.REF         ------------------------------------------------------------ 31039

chimp.LOC112206744-LOC107973052-GGT2.revcompl                                        ------------------------------------------------------------ 61762
chimp54546-135457.revcompl.Pan.troglodytes.clone.rp43-41g5.GenBank:AC099533.36       ------------------------------------------------------------ 67152
human.FAM30B-LOC105372935-GGT2.NCBI.                                                 tgacaccatcactgggctgcccacctcatcccacagctgcagcccattcttgtgatagct 63708
human.FAM230E-LOC105377182-GGT3P.NCBI.                                               ------------------------------------------------------------ 57310
human.FAM230J-LOC105372942-GGTLC5P.NCBI.rev.compl                                    ------------------------------------------------------------ 46364
human.FAM230A.ncRNA.18487127.18500594-GGTLC3.18516335.18518165.rev.compl.REF         ------------------------------------------------------------ 31039

chimp.LOC112206744-LOC107973052-GGT2.revcompl                                        ------------------------------------------------------------ 61762
chimp54546-135457.revcompl.Pan.troglodytes.clone.rp43-41g5.GenBank:AC099533.36       ------------------------------------------------------------ 67152
human.FAM30B-LOC105372935-GGT2.NCBI.                                                 tccctttttccccacaaatgggaatggcggctgccctggctatgcctatctgaagtggat 63768
human.FAM230E-LOC105377182-GGT3P.NCBI.                                               ------------------------------------------------------------ 57310
human.FAM230J-LOC105372942-GGTLC5P.NCBI.rev.compl                                    ------------------------------------------------------------ 46364
human.FAM230A.ncRNA.18487127.18500594-GGTLC3.18516335.18518165.rev.compl.REF         ------------------------------------------------------------ 31039

chimp.LOC112206744-LOC107973052-GGT2.revcompl                                        ------------------------------------------------------------ 61762
chimp54546-135457.revcompl.Pan.troglodytes.clone.rp43-41g5.GenBank:AC099533.36       ------------------------------------------------------------ 67152
human.FAM30B-LOC105372935-GGT2.NCBI.                                                 ccagctggtgaacagccgggacagctcaaatactagtggagtgcagctctctgcccagcc 63828
human.FAM230E-LOC105377182-GGT3P.NCBI.                                               ------------------------------------------------------------ 57310
human.FAM230J-LOC105372942-GGTLC5P.NCBI.rev.compl                                    ------------------------------------------------------------ 46364
human.FAM230A.ncRNA.18487127.18500594-GGTLC3.18516335.18518165.rev.compl.REF         ------------------------------------------------------------ 31039

chimp.LOC112206744-LOC107973052-GGT2.revcompl                                        ------------------------------------------------------------ 61762
chimp54546-135457.revcompl.Pan.troglodytes.clone.rp43-41g5.GenBank:AC099533.36       ------------------------------------------------------------ 67152
human.FAM30B-LOC105372935-GGT2.NCBI.                                                 cagcaggtgactgtgcctcagtgtaagctaatctaatctgcaagatgacagacctcaaaa 63888
human.FAM230E-LOC105377182-GGT3P.NCBI.                                               ------------------------------------------------------------ 57310
human.FAM230J-LOC105372942-GGTLC5P.NCBI.rev.compl                                    ------------------------------------------------------------ 46364
human.FAM230A.ncRNA.18487127.18500594-GGTLC3.18516335.18518165.rev.compl.REF         ------------------------------------------------------------ 31039

chimp.LOC112206744-LOC107973052-GGT2.revcompl                                        ------------------------------------------------------------ 61762
chimp54546-135457.revcompl.Pan.troglodytes.clone.rp43-41g5.GenBank:AC099533.36       ------------------------------------------------------------ 67152
human.FAM30B-LOC105372935-GGT2.NCBI.                                                 cggcccatacaatgaaatgcccatggcttgcaaaactttaattcatgcagaacctttcct 63948
human.FAM230E-LOC105377182-GGT3P.NCBI.                                               ------------------------------------------------------------ 57310
human.FAM230J-LOC105372942-GGTLC5P.NCBI.rev.compl                                    ------------------------------------------------------------ 46364
human.FAM230A.ncRNA.18487127.18500594-GGTLC3.18516335.18518165.rev.compl.REF         ------------------------------------------------------------ 31039

chimp.LOC112206744-LOC107973052-GGT2.revcompl                                        ------------------------------------------------------------ 61762
chimp54546-135457.revcompl.Pan.troglodytes.clone.rp43-41g5.GenBank:AC099533.36       ------------------------------------------------------------ 67152
human.FAM30B-LOC105372935-GGT2.NCBI.                                                 cacaaacaaaatcttacacagcaccccatcttggcttcagcagaaccaggaaaaataaaa 64008
human.FAM230E-LOC105377182-GGT3P.NCBI.                                               ------------------------------------------------------------ 57310
human.FAM230J-LOC105372942-GGTLC5P.NCBI.rev.compl                                    ------------------------------------------------------------ 46364
human.FAM230A.ncRNA.18487127.18500594-GGTLC3.18516335.18518165.rev.compl.REF         ------------------------------------------------------------ 31039

chimp.LOC112206744-LOC107973052-GGT2.revcompl                                        ------------------------------------------------------------ 61762
chimp54546-135457.revcompl.Pan.troglodytes.clone.rp43-41g5.GenBank:AC099533.36       ------------------------------------------------------------ 67152
human.FAM30B-LOC105372935-GGT2.NCBI.                                                 aattggcaggggaaggaaataaacagaacaagtggtatctgaatttatttgatatattct 64068
human.FAM230E-LOC105377182-GGT3P.NCBI.                                               ------------------------------------------------------------ 57310
human.FAM230J-LOC105372942-GGTLC5P.NCBI.rev.compl                                    ------------------------------------------------------------ 46364
human.FAM230A.ncRNA.18487127.18500594-GGTLC3.18516335.18518165.rev.compl.REF         ------------------------------------------------------------ 31039

chimp.LOC112206744-LOC107973052-GGT2.revcompl                                        ------------------------------------------------------------ 61762
chimp54546-135457.revcompl.Pan.troglodytes.clone.rp43-41g5.GenBank:AC099533.36       ------------------------------------------------------------ 67152
human.FAM30B-LOC105372935-GGT2.NCBI.                                                 ggttgatggcattaattatgacatttaaagtcaccatgaggacaactcattagtaatatc 64128
human.FAM230E-LOC105377182-GGT3P.NCBI.                                               ------------------------------------------------------------ 57310
human.FAM230J-LOC105372942-GGTLC5P.NCBI.rev.compl                                    ------------------------------------------------------------ 46364
human.FAM230A.ncRNA.18487127.18500594-GGTLC3.18516335.18518165.rev.compl.REF         ------------------------------------------------------------ 31039

chimp.LOC112206744-LOC107973052-GGT2.revcompl                                        ------------------------------------------------------------ 61762
chimp54546-135457.revcompl.Pan.troglodytes.clone.rp43-41g5.GenBank:AC099533.36       ------------------------------------------------------------ 67152
human.FAM30B-LOC105372935-GGT2.NCBI.                                                 agtatgttaaaatatggtgcataacatttttaatatatgtacagtcatgcactgaataac 64188
human.FAM230E-LOC105377182-GGT3P.NCBI.                                               ------------------------------------------------------------ 57310
human.FAM230J-LOC105372942-GGTLC5P.NCBI.rev.compl                                    ------------------------------------------------------------ 46364
human.FAM230A.ncRNA.18487127.18500594-GGTLC3.18516335.18518165.rev.compl.REF         ------------------------------------------------------------ 31039

chimp.LOC112206744-LOC107973052-GGT2.revcompl                                        ------------------------------------------------------------ 61762
chimp54546-135457.revcompl.Pan.troglodytes.clone.rp43-41g5.GenBank:AC099533.36       ------------------------------------------------------------ 67152
human.FAM30B-LOC105372935-GGT2.NCBI.                                                 atttcagtcaatgaggaaacacatgtgcaacagtgatcctgtaagattataatggagcat 64248
human.FAM230E-LOC105377182-GGT3P.NCBI.                                               ------------------------------------------------------------ 57310
human.FAM230J-LOC105372942-GGTLC5P.NCBI.rev.compl                                    ------------------------------------------------------------ 46364
human.FAM230A.ncRNA.18487127.18500594-GGTLC3.18516335.18518165.rev.compl.REF         ------------------------------------------------------------ 31039

chimp.LOC112206744-LOC107973052-GGT2.revcompl                                        ------------------------------------------------------------ 61762
chimp54546-135457.revcompl.Pan.troglodytes.clone.rp43-41g5.GenBank:AC099533.36       ------------------------------------------------------------ 67152
human.FAM30B-LOC105372935-GGT2.NCBI.                                                 atatagagatctaatatatggcacttaatgttggcatggcagatcaagtaggggaaatga 64308
human.FAM230E-LOC105377182-GGT3P.NCBI.                                               ------------------------------------------------------------ 57310
human.FAM230J-LOC105372942-GGTLC5P.NCBI.rev.compl                                    ------------------------------------------------------------ 46364
human.FAM230A.ncRNA.18487127.18500594-GGTLC3.18516335.18518165.rev.compl.REF         ------------------------------------------------------------ 31039

chimp.LOC112206744-LOC107973052-GGT2.revcompl                                        ------------------------------------------------------------ 61762
chimp54546-135457.revcompl.Pan.troglodytes.clone.rp43-41g5.GenBank:AC099533.36       ------------------------------------------------------------ 67152
human.FAM30B-LOC105372935-GGT2.NCBI.                                                 ctgatatttagtaacagtgctgggacatttgattttccataataaaatatataaatgaaa 64368
human.FAM230E-LOC105377182-GGT3P.NCBI.                                               ------------------------------------------------------------ 57310
human.FAM230J-LOC105372942-GGTLC5P.NCBI.rev.compl                                    ------------------------------------------------------------ 46364
human.FAM230A.ncRNA.18487127.18500594-GGTLC3.18516335.18518165.rev.compl.REF         ------------------------------------------------------------ 31039

chimp.LOC112206744-LOC107973052-GGT2.revcompl                                        ------------------------------------------------------------ 61762
chimp54546-135457.revcompl.Pan.troglodytes.clone.rp43-41g5.GenBank:AC099533.36       ------------------------------------------------------------ 67152
human.FAM30B-LOC105372935-GGT2.NCBI.                                                 atatatatcccatctaggtttgttgaaatacaccctatgatgttcacacaagaatgaaat 64428
human.FAM230E-LOC105377182-GGT3P.NCBI.                                               ------------------------------------------------------------ 57310
human.FAM230J-LOC105372942-GGTLC5P.NCBI.rev.compl                                    ------------------------------------------------------------ 46364
human.FAM230A.ncRNA.18487127.18500594-GGTLC3.18516335.18518165.rev.compl.REF         ------------------------------------------------------------ 31039

chimp.LOC112206744-LOC107973052-GGT2.revcompl                                        ------------------------------------------------------------ 61762
```

```
chimp54546-135457.revcompl.Pan.troglodytes.clone.rp43-41g5.GenBank:AC099533.36      ------------------------------------------------------------ 67152
human.FAM30B-LOC105372935-GGT2.NCBI.                                                 tgcctaatgatgcatttcttaaaacatgtccccatcattaagtgacccatgactgtatac 64488
human.FAM230E-LOC105377182-GGT3P.NCBI.                                               ------------------------------------------------------------ 57310
human.FAM230J-LOC105372942-GGTLC5P.NCBI.rev.compl                                    ------------------------------------------------------------ 46364
human.FAM230A.ncRNA.18487127.18500594-GGTLC3.18516335.18518165.rev.compl.REF         ------------------------------------------------------------ 31039

chimp.LOC112206744-LOC107973052-GGT2.revcompl                                        ------------------------------------------------------------ 61762
chimp54546-135457.revcompl.Pan.troglodytes.clone.rp43-41g5.GenBank:AC099533.36      ------------------------------------------------------------ 67152
human.FAM30B-LOC105372935-GGT2.NCBI.                                                 acacacacatatggtgacacttaaatcaaatgattgcagtattcctgaaacacagaacat 64548
human.FAM230E-LOC105377182-GGT3P.NCBI.                                               ------------------------------------------------------------ 57310
human.FAM230J-LOC105372942-GGTLC5P.NCBI.rev.compl                                    ------------------------------------------------------------ 46364
human.FAM230A.ncRNA.18487127.18500594-GGTLC3.18516335.18518165.rev.compl.REF         ------------------------------------------------------------ 31039

chimp.LOC112206744-LOC107973052-GGT2.revcompl                                        ------------------------------------------------------------ 61762
chimp54546-135457.revcompl.Pan.troglodytes.clone.rp43-41g5.GenBank:AC099533.36      ------------------------------------------------------------ 67152
human.FAM30B-LOC105372935-GGT2.NCBI.                                                 tttgcaaacaatttacctacattcataacattaggcatccttagaattgcagtgttctat 64608
human.FAM230E-LOC105377182-GGT3P.NCBI.                                               ------------------------------------------------------------ 57310
human.FAM230J-LOC105372942-GGTLC5P.NCBI.rev.compl                                    ------------------------------------------------------------ 46364
human.FAM230A.ncRNA.18487127.18500594-GGTLC3.18516335.18518165.rev.compl.REF         ------------------------------------------------------------ 31039

chimp.LOC112206744-LOC107973052-GGT2.revcompl                                        ------------------------------------------------------------ 61762
chimp54546-135457.revcompl.Pan.troglodytes.clone.rp43-41g5.GenBank:AC099533.36      ------------------------------------------------------------ 67152
human.FAM30B-LOC105372935-GGT2.NCBI.                                                 gacagagcaactacaaccaaccccatccatctcctgcaaagaaggctggaggcaggtca 64668
human.FAM230E-LOC105377182-GGT3P.NCBI.                                               ------------------------------------------------------------ 57310
human.FAM230J-LOC105372942-GGTLC5P.NCBI.rev.compl                                    ------------------------------------------------------------ 46364
human.FAM230A.ncRNA.18487127.18500594-GGTLC3.18516335.18518165.rev.compl.REF         ------------------------------------------------------------ 31039

chimp.LOC112206744-LOC107973052-GGT2.revcompl                                        ------------------------------------------------------------ 61762
chimp54546-135457.revcompl.Pan.troglodytes.clone.rp43-41g5.GenBank:AC099533.36      ------------------------------------------------------------ 67152
human.FAM30B-LOC105372935-GGT2.NCBI.                                                 gggtacaccagcatcttcaagaactgcttctcaatcctggacctacactggaatcacctg 64728
human.FAM230E-LOC105377182-GGT3P.NCBI.                                               ------------------------------------------------------------ 57310
human.FAM230J-LOC105372942-GGTLC5P.NCBI.rev.compl                                    ------------------------------------------------------------ 46364
human.FAM230A.ncRNA.18487127.18500594-GGTLC3.18516335.18518165.rev.compl.REF         ------------------------------------------------------------ 31039

chimp.LOC112206744-LOC107973052-GGT2.revcompl                                        ------------------------------------------------------------ 61762
chimp54546-135457.revcompl.Pan.troglodytes.clone.rp43-41g5.GenBank:AC099533.36      ------------------------------------------------------------ 67152
human.FAM30B-LOC105372935-GGT2.NCBI.                                                 gggagctttaaaaaaataacagtgcctggaccccacctgacataactggtcttaggatt 64788
human.FAM230E-LOC105377182-GGT3P.NCBI.                                               ------------------------------------------------------------ 57310
human.FAM230J-LOC105372942-GGTLC5P.NCBI.rev.compl                                    ------------------------------------------------------------ 46364
human.FAM230A.ncRNA.18487127.18500594-GGTLC3.18516335.18518165.rev.compl.REF         ------------------------------------------------------------ 31039

chimp.LOC112206744-LOC107973052-GGT2.revcompl                                        ------------------------------------------------------------ 61762
chimp54546-135457.revcompl.Pan.troglodytes.clone.rp43-41g5.GenBank:AC099533.36      ------------------------------------------------------------ 67152
human.FAM30B-LOC105372935-GGT2.NCBI.                                                 aattagggtttttttgaagctccacagatgattcaaatcaggtgtagcaaagcactgtt 64848
human.FAM230E-LOC105377182-GGT3P.NCBI.                                               ------------------------------------------------------------ 57310
human.FAM230J-LOC105372942-GGTLC5P.NCBI.rev.compl                                    ------------------------------------------------------------ 46364
human.FAM230A.ncRNA.18487127.18500594-GGTLC3.18516335.18518165.rev.compl.REF         ------------------------------------------------------------ 31039

chimp.LOC112206744-LOC107973052-GGT2.revcompl                                        ------------------------------------------------------------ 61762
chimp54546-135457.revcompl.Pan.troglodytes.clone.rp43-41g5.GenBank:AC099533.36      ------------------------------------------------------------ 67152
human.FAM30B-LOC105372935-GGT2.NCBI.                                                 actttaaagtgtctctacctacatggtgccagccaagtgctcaaatgaaatatcttaagg 64908
human.FAM230E-LOC105377182-GGT3P.NCBI.                                               ------------------------------------------------------------ 57310
human.FAM230J-LOC105372942-GGTLC5P.NCBI.rev.compl                                    ------------------------------------------------------------ 46364
human.FAM230A.ncRNA.18487127.18500594-GGTLC3.18516335.18518165.rev.compl.REF         ------------------------------------------------------------ 31039

chimp.LOC112206744-LOC107973052-GGT2.revcompl                                        ------------------------------------------------------------ 61762
chimp54546-135457.revcompl.Pan.troglodytes.clone.rp43-41g5.GenBank:AC099533.36      ------------------------------------------------------------ 67152
human.FAM30B-LOC105372935-GGT2.NCBI.                                                 ctctcactggctttaagtttctctctttggtagagacccaatcactggttcaaggaagtt 64968
human.FAM230E-LOC105377182-GGT3P.NCBI.                                               ------------------------------------------------------------ 57310
human.FAM230J-LOC105372942-GGTLC5P.NCBI.rev.compl                                    ------------------------------------------------------------ 46364
human.FAM230A.ncRNA.18487127.18500594-GGTLC3.18516335.18518165.rev.compl.REF         ------------------------------------------------------------ 31039

chimp.LOC112206744-LOC107973052-GGT2.revcompl                                        ------------------------------------------------------------ 61762
chimp54546-135457.revcompl.Pan.troglodytes.clone.rp43-41g5.GenBank:AC099533.36      ------------------------------------------------------------ 67152
human.FAM30B-LOC105372935-GGT2.NCBI.                                                 tcattctcctccagtcttccccagtgcaaaagaaaacagctgagacccatcagatactgg 65028
human.FAM230E-LOC105377182-GGT3P.NCBI.                                               ------------------------------------------------------------ 57310
human.FAM230J-LOC105372942-GGTLC5P.NCBI.rev.compl                                    ------------------------------------------------------------ 46364
human.FAM230A.ncRNA.18487127.18500594-GGTLC3.18516335.18518165.rev.compl.REF         ------------------------------------------------------------ 31039

chimp.LOC112206744-LOC107973052-GGT2.revcompl                                        ------------------------------------------------------------ 61762
chimp54546-135457.revcompl.Pan.troglodytes.clone.rp43-41g5.GenBank:AC099533.36      ------------------------------------------------------------ 67152
human.FAM30B-LOC105372935-GGT2.NCBI.                                                 cttttgtgatgcaataatgagtttaaccagaatgcatccgatttacagaccttacaaagg 65088
human.FAM230E-LOC105377182-GGT3P.NCBI.                                               ------------------------------------------------------------ 57310
human.FAM230J-LOC105372942-GGTLC5P.NCBI.rev.compl                                    ------------------------------------------------------------ 46364
human.FAM230A.ncRNA.18487127.18500594-GGTLC3.18516335.18518165.rev.compl.REF         ------------------------------------------------------------ 31039

chimp.LOC112206744-LOC107973052-GGT2.revcompl                                        ------------------------------------------------------------ 61762
chimp54546-135457.revcompl.Pan.troglodytes.clone.rp43-41g5.GenBank:AC099533.36      ------------------------------------------------------------ 67152
human.FAM30B-LOC105372935-GGT2.NCBI.                                                 cactaggccagcggatcatgcacgtcctccccacccaaaggtaaacagtgttaagtggcc 65148
human.FAM230E-LOC105377182-GGT3P.NCBI.                                               ------------------------------------------------------------ 57310
human.FAM230J-LOC105372942-GGTLC5P.NCBI.rev.compl                                    ------------------------------------------------------------ 46364
human.FAM230A.ncRNA.18487127.18500594-GGTLC3.18516335.18518165.rev.compl.REF         ------------------------------------------------------------ 31039

chimp.LOC112206744-LOC107973052-GGT2.revcompl                                        ------------------------------------------------------------ 61762
chimp54546-135457.revcompl.Pan.troglodytes.clone.rp43-41g5.GenBank:AC099533.36      ------------------------------------------------------------ 67152
human.FAM30B-LOC105372935-GGT2.NCBI.                                                 tgaatgagccaggacagcagggtcaaatcaacttgtctaggctggaagcaggttataaac 65208
human.FAM230E-LOC105377182-GGT3P.NCBI.                                               ------------------------------------------------------------ 57310
human.FAM230J-LOC105372942-GGTLC5P.NCBI.rev.compl                                    ------------------------------------------------------------ 46364
human.FAM230A.ncRNA.18487127.18500594-GGTLC3.18516335.18518165.rev.compl.REF         ------------------------------------------------------------ 31039

chimp.LOC112206744-LOC107973052-GGT2.revcompl                                        ------------------------------------------------------------ 61762
chimp54546-135457.revcompl.Pan.troglodytes.clone.rp43-41g5.GenBank:AC099533.36      ------------------------------------------------------------ 67152
human.FAM30B-LOC105372935-GGT2.NCBI.                                                 aatccggacaaataaataactactggattgcactttaaccacacacattcagctgcacct 65268
human.FAM230E-LOC105377182-GGT3P.NCBI.                                               ------------------------------------------------------------ 57310
human.FAM230J-LOC105372942-GGTLC5P.NCBI.rev.compl                                    ------------------------------------------------------------ 46364
human.FAM230A.ncRNA.18487127.18500594-GGTLC3.18516335.18518165.rev.compl.REF         ------------------------------------------------------------ 31039

chimp.LOC112206744-LOC107973052-GGT2.revcompl                                        ------------------------------------------------------------ 61762
chimp54546-135457.revcompl.Pan.troglodytes.clone.rp43-41g5.GenBank:AC099533.36      ------------------------------------------------------------ 67152
human.FAM30B-LOC105372935-GGT2.NCBI.                                                 gtttattaaatacctccttaatttccctctgccccacaaagggcttctgacccctgaaaa 65328
human.FAM230E-LOC105377182-GGT3P.NCBI.                                               ------------------------------------------------------------ 57310
human.FAM230J-LOC105372942-GGTLC5P.NCBI.rev.compl                                    ------------------------------------------------------------ 46364
human.FAM230A.ncRNA.18487127.18500594-GGTLC3.18516335.18518165.rev.compl.REF         ------------------------------------------------------------ 31039

chimp.LOC112206744-LOC107973052-GGT2.revcompl                                        ------------------------------------------------------------ 61762
chimp54546-135457.revcompl.Pan.troglodytes.clone.rp43-41g5.GenBank:AC099533.36      ------------------------------------------------------------ 67152
human.FAM30B-LOC105372935-GGT2.NCBI.                                                 taatgcttctcaacataaaaagaattgttttcttcttggcagtttaattccccttccaca 65388
human.FAM230E-LOC105377182-GGT3P.NCBI.                                               ------------------------------------------------------------ 57310
human.FAM230J-LOC105372942-GGTLC5P.NCBI.rev.compl                                    ------------------------------------------------------------ 46364
human.FAM230A.ncRNA.18487127.18500594-GGTLC3.18516335.18518165.rev.compl.REF         ------------------------------------------------------------ 31039

chimp.LOC112206744-LOC107973052-GGT2.revcompl                                        ------------------------------------------------------------ 61762
chimp54546-135457.revcompl.Pan.troglodytes.clone.rp43-41g5.GenBank:AC099533.36      ------------------------------------------------------------ 67152
human.FAM30B-LOC105372935-GGT2.NCBI.                                                 catcccaccccaccatttccctataatggtaataccccgctgtgcagacctctgctgcct 65448
human.FAM230E-LOC105377182-GGT3P.NCBI.                                               ------------------------------------------------------------ 57310
human.FAM230J-LOC105372942-GGTLC5P.NCBI.rev.compl                                    ------------------------------------------------------------ 46364
human.FAM230A.ncRNA.18487127.18500594-GGTLC3.18516335.18518165.rev.compl.REF         ------------------------------------------------------------ 31039

chimp.LOC112206744-LOC107973052-GGT2.revcompl                                        ------------------------------------------------------------ 61762
chimp54546-135457.revcompl.Pan.troglodytes.clone.rp43-41g5.GenBank:AC099533.36      ------------------------------------------------------------ 67152
human.FAM30B-LOC105372935-GGT2.NCBI.                                                 ccagagagacacaggcgcccaaccctgaccagggcatcctctgacccacagcccctctat 65508
human.FAM230E-LOC105377182-GGT3P.NCBI.                                               ------------------------------------------------------------ 57310
human.FAM230J-LOC105372942-GGTLC5P.NCBI.rev.compl                                    ------------------------------------------------------------ 46364
human.FAM230A.ncRNA.18487127.18500594-GGTLC3.18516335.18518165.rev.compl.REF         ------------------------------------------------------------ 31039

chimp.LOC112206744-LOC107973052-GGT2.revcompl                                        ------------------------------------------------------------ 61762
chimp54546-135457.revcompl.Pan.troglodytes.clone.rp43-41g5.GenBank:AC099533.36      ------------------------------------------------------------ 67152
```

```
human.FAM30B-LOC105372935-GGT2.NCBI.                                       ctcccctctcagcctacaatagaaagttcctcccaggcaaggatcattttttttaaataa 65568
human.FAM230E-LOC105377182-GGT3P.NCBI.                                     ------------------------------------------------------------ 57310
human.FAM230J-LOC105372942-GGTLC5P.NCBI.rev.compl                          ------------------------------------------------------------ 46364
human.FAM230A.ncRNA.18487127.18500594-GGTLC3.18516335.18518165.rev.compl.REF ------------------------------------------------------------ 31039

chimp.LOC112206744-LOC107973052-GGT2.revcompl                              ------------------------------------------------------------ 61762
chimp54546-135457.revcompl.Pan.troglodytes.clone.rp43-41g5.GenBank:AC099533.36 ------------------------------------------------------------ 67152
human.FAM30B-LOC105372935-GGT2.NCBI.                                       ctttttaactttagacattctgtactctttggattactctgcaataagcaaatatgact 65628
human.FAM230E-LOC105377182-GGT3P.NCBI.                                     ------------------------------------------------------------ 57310
human.FAM230J-LOC105372942-GGTLC5P.NCBI.rev.compl                          ------------------------------------------------------------ 46364
human.FAM230A.ncRNA.18487127.18500594-GGTLC3.18516335.18518165.rev.compl.REF ------------------------------------------------------------ 31039

chimp.LOC112206744-LOC107973052-GGT2.revcompl                              ------------------------------------------------------------ 61762
chimp54546-135457.revcompl.Pan.troglodytes.clone.rp43-41g5.GenBank:AC099533.36 ------------------------------------------------------------ 67152
human.FAM30B-LOC105372935-GGT2.NCBI.                                       tctgtaacataaaaagaaagatcaaaatgttatatataactgcatgaaaagaactagaaa 65688
human.FAM230E-LOC105377182-GGT3P.NCBI.                                     ------------------------------------------------------------ 57310
human.FAM230J-LOC105372942-GGTLC5P.NCBI.rev.compl                          ------------------------------------------------------------ 46364
human.FAM230A.ncRNA.18487127.18500594-GGTLC3.18516335.18518165.rev.compl.REF ------------------------------------------------------------ 31039

chimp.LOC112206744-LOC107973052-GGT2.revcompl                              ------------------------------------------------------------ 61762
chimp54546-135457.revcompl.Pan.troglodytes.clone.rp43-41g5.GenBank:AC099533.36 ------------------------------------------------------------ 67152
human.FAM30B-LOC105372935-GGT2.NCBI.                                       gaaaatactaggtgacaggatggcaagtgattttatttttttcttaatattttactcct 65748
human.FAM230E-LOC105377182-GGT3P.NCBI.                                     ------------------------------------------------------------ 57310
human.FAM230J-LOC105372942-GGTLC5P.NCBI.rev.compl                          ------------------------------------------------------------ 46364
human.FAM230A.ncRNA.18487127.18500594-GGTLC3.18516335.18518165.rev.compl.REF ------------------------------------------------------------ 31039

chimp.LOC112206744-LOC107973052-GGT2.revcompl                              ------------------------------------------------------------ 61762
chimp54546-135457.revcompl.Pan.troglodytes.clone.rp43-41g5.GenBank:AC099533.36 ------------------------------------------------------------ 67152
human.FAM30B-LOC105372935-GGT2.NCBI.                                       tcccccagtcctctgcaatgagtatgtaccagttttataactagaaaaaaatttagcca 65808
human.FAM230E-LOC105377182-GGT3P.NCBI.                                     ------------------------------------------------------------ 57310
human.FAM230J-LOC105372942-GGTLC5P.NCBI.rev.compl                          ------------------------------------------------------------ 46364
human.FAM230A.ncRNA.18487127.18500594-GGTLC3.18516335.18518165.rev.compl.REF ------------------------------------------------------------ 31039

chimp.LOC112206744-LOC107973052-GGT2.revcompl                              ------------------------------------------------------------ 61762
chimp54546-135457.revcompl.Pan.troglodytes.clone.rp43-41g5.GenBank:AC099533.36 ------------------------------------------------------------ 67152
human.FAM30B-LOC105372935-GGT2.NCBI.                                       aaaagaaaaataatgcatgtttgctatataaaattcactactgtagtatatatatataca 65868
human.FAM230E-LOC105377182-GGT3P.NCBI.                                     ------------------------------------------------------------ 57310
human.FAM230J-LOC105372942-GGTLC5P.NCBI.rev.compl                          ------------------------------------------------------------ 46364
human.FAM230A.ncRNA.18487127.18500594-GGTLC3.18516335.18518165.rev.compl.REF ------------------------------------------------------------ 31039

chimp.LOC112206744-LOC107973052-GGT2.revcompl                              ------------------------------------------------------------ 61762
chimp54546-135457.revcompl.Pan.troglodytes.clone.rp43-41g5.GenBank:AC099533.36 ------------------------------------------------------------ 67152
human.FAM30B-LOC105372935-GGT2.NCBI.                                       tacatacatatttggtctttgtccccagttcctggcactgagctcctaaaccccttggaa 65928
human.FAM230E-LOC105377182-GGT3P.NCBI.                                     ------------------------------------------------------------ 57310
human.FAM230J-LOC105372942-GGTLC5P.NCBI.rev.compl                          ------------------------------------------------------------ 46364
human.FAM230A.ncRNA.18487127.18500594-GGTLC3.18516335.18518165.rev.compl.REF ------------------------------------------------------------ 31039

chimp.LOC112206744-LOC107973052-GGT2.revcompl                              ------------------------------------------------------------ 61762
chimp54546-135457.revcompl.Pan.troglodytes.clone.rp43-41g5.GenBank:AC099533.36 ------------------------------------------------------------ 67152
human.FAM30B-LOC105372935-GGT2.NCBI.                                       cttcctaagcaatgggagtacctttgttatttataagaagccccttttggccatcccag 65988
human.FAM230E-LOC105377182-GGT3P.NCBI.                                     ------------------------------------------------------------ 57310
human.FAM230J-LOC105372942-GGTLC5P.NCBI.rev.compl                          ------------------------------------------------------------ 46364
human.FAM230A.ncRNA.18487127.18500594-GGTLC3.18516335.18518165.rev.compl.REF ------------------------------------------------------------ 31039

chimp.LOC112206744-LOC107973052-GGT2.revcompl                              ------------------------------------------------------------ 61762
chimp54546-135457.revcompl.Pan.troglodytes.clone.rp43-41g5.GenBank:AC099533.36 ------------------------------------------------------------ 67152
human.FAM30B-LOC105372935-GGT2.NCBI.                                       agtttatgctaacgaggtgactgaaggtgagcacggaggcagtttcaaggaaggagctgg 66048
human.FAM230E-LOC105377182-GGT3P.NCBI.                                     ------------------------------------------------------------ 57310
human.FAM230J-LOC105372942-GGTLC5P.NCBI.rev.compl                          ------------------------------------------------------------ 46364
human.FAM230A.ncRNA.18487127.18500594-GGTLC3.18516335.18518165.rev.compl.REF ------------------------------------------------------------ 31039

chimp.LOC112206744-LOC107973052-GGT2.revcompl                              ------------------------------------------------------------ 61762
chimp54546-135457.revcompl.Pan.troglodytes.clone.rp43-41g5.GenBank:AC099533.36 ------------------------------------------------------------ 67152
human.FAM30B-LOC105372935-GGT2.NCBI.                                       ccacgcttagaattgtggagttttcagccccgcccttagacctccaaggacaagaggggg 66108
human.FAM230E-LOC105377182-GGT3P.NCBI.                                     ------------------------------------------------------------ 57310
human.FAM230J-LOC105372942-GGTLC5P.NCBI.rev.compl                          ------------------------------------------------------------ 46364
human.FAM230A.ncRNA.18487127.18500594-GGTLC3.18516335.18518165.rev.compl.REF ------------------------------------------------------------ 31039

chimp.LOC112206744-LOC107973052-GGT2.revcompl                              ------------------------------------------------------------ 61762
chimp54546-135457.revcompl.Pan.troglodytes.clone.rp43-41g5.GenBank:AC099533.36 ------------------------------------------------------------ 67152
human.FAM30B-LOC105372935-GGT2.NCBI.                                       accgcagattgatccaatcagcattggtcagtgatttgatcagtcatacccacgtaatga 66168
human.FAM230E-LOC105377182-GGT3P.NCBI.                                     ------------------------------------------------------------ 57310
human.FAM230J-LOC105372942-GGTLC5P.NCBI.rev.compl                          ------------------------------------------------------------ 46364
human.FAM230A.ncRNA.18487127.18500594-GGTLC3.18516335.18518165.rev.compl.REF ------------------------------------------------------------ 31039

chimp.LOC112206744-LOC107973052-GGT2.revcompl                              ------------------------------------------------------------ 61762
chimp54546-135457.revcompl.Pan.troglodytes.clone.rp43-41g5.GenBank:AC099533.36 ------------------------------------------------------------ 67152
human.FAM30B-LOC105372935-GGT2.NCBI.                                       aatcccatatgaaaaccctaaataatggagttaggagagcttctgggtgctggaagcggc 66228
human.FAM230E-LOC105377182-GGT3P.NCBI.                                     ------------------------------------------------------------ 57310
human.FAM230J-LOC105372942-GGTLC5P.NCBI.rev.compl                          ------------------------------------------------------------ 46364
human.FAM230A.ncRNA.18487127.18500594-GGTLC3.18516335.18518165.rev.compl.REF ------------------------------------------------------------ 31039

chimp.LOC112206744-LOC107973052-GGT2.revcompl                              ------------------------------------------------------------ 61762
chimp54546-135457.revcompl.Pan.troglodytes.clone.rp43-41g5.GenBank:AC099533.36 ------------------------------------------------------------ 67152
human.FAM30B-LOC105372935-GGT2.NCBI.                                       gtgcaccaggagagggcatgggcagtgagcactactcccctctcagaccttgccctatgc 66288
human.FAM230E-LOC105377182-GGT3P.NCBI.                                     ------------------------------------------------------------ 57310
human.FAM230J-LOC105372942-GGTLC5P.NCBI.rev.compl                          ------------------------------------------------------------ 46364
human.FAM230A.ncRNA.18487127.18500594-GGTLC3.18516335.18518165.rev.compl.REF ------------------------------------------------------------ 31039

chimp.LOC112206744-LOC107973052-GGT2.revcompl                              ------------------------------------------------------------ 61762
chimp54546-135457.revcompl.Pan.troglodytes.clone.rp43-41g5.GenBank:AC099533.36 ------------------------------------------------------------ 67152
human.FAM30B-LOC105372935-GGT2.NCBI.                                       actaaataggactgacctatgtgaccaataggaaatgcacaaatggtggaatgtgacttc 66348
human.FAM230E-LOC105377182-GGT3P.NCBI.                                     ------------------------------------------------------------ 57310
human.FAM230J-LOC105372942-GGTLC5P.NCBI.rev.compl                          ------------------------------------------------------------ 46364
human.FAM230A.ncRNA.18487127.18500594-GGTLC3.18516335.18518165.rev.compl.REF ------------------------------------------------------------ 31039

chimp.LOC112206744-LOC107973052-GGT2.revcompl                              ------------------------------------------------------------ 61762
chimp54546-135457.revcompl.Pan.troglodytes.clone.rp43-41g5.GenBank:AC099533.36 ------------------------------------------------------------ 67152
human.FAM30B-LOC105372935-GGT2.NCBI.                                       cagggctaggtcataaaaagacaagcacgatgttatgaggacatcaaagcagccatatgg 66408
human.FAM230E-LOC105377182-GGT3P.NCBI.                                     ------------------------------------------------------------ 57310
human.FAM230J-LOC105372942-GGTLC5P.NCBI.rev.compl                          ------------------------------------------------------------ 46364
human.FAM230A.ncRNA.18487127.18500594-GGTLC3.18516335.18518165.rev.compl.REF ------------------------------------------------------------ 31039

chimp.LOC112206744-LOC107973052-GGT2.revcompl                              ------------------------------------------------------------ 61762
chimp54546-135457.revcompl.Pan.troglodytes.clone.rp43-41g5.GenBank:AC099533.36 ------------------------------------------------------------ 67152
human.FAM30B-LOC105372935-GGT2.NCBI.                                       ggaggaccccatgaggcctcctgccagcagctagcactaacttggctagcatgtgactgg 66468
human.FAM230E-LOC105377182-GGT3P.NCBI.                                     ------------------------------------------------------------ 57310
human.FAM230J-LOC105372942-GGTLC5P.NCBI.rev.compl                          ------------------------------------------------------------ 46364
human.FAM230A.ncRNA.18487127.18500594-GGTLC3.18516335.18518165.rev.compl.REF ------------------------------------------------------------ 31039

chimp.LOC112206744-LOC107973052-GGT2.revcompl                              ------------------------------------------------------------ 61762
chimp54546-135457.revcompl.Pan.troglodytes.clone.rp43-41g5.GenBank:AC099533.36 -----------------------------------------------------------T 67153
human.FAM30B-LOC105372935-GGT2.NCBI.                                       aagtagattctccagcctcagtcaactcagtcaagccttcagataatgtcaaccccaggc 66528
human.FAM230E-LOC105377182-GGT3P.NCBI.                                     ------------------------------------------------------------ 57310
human.FAM230J-LOC105372942-GGTLC5P.NCBI.rev.compl                          ------------------------------------------------------------ 46364
human.FAM230A.ncRNA.18487127.18500594-GGTLC3.18516335.18518165.rev.compl.REF ------------------------------------------------------------ 31039

chimp.LOC112206744-LOC107973052-GGT2.revcompl                              ------------------------------------------------------------ 61762
chimp54546-135457.revcompl.Pan.troglodytes.clone.rp43-41g5.GenBank:AC099533.36 TTATTTATTTATTTATTGAGACAGGGTCTCACTCTGTTGCCCAGGCTAGAGTGCAGTGGC 67213
human.FAM30B-LOC105372935-GGT2.NCBI.                                       atctttttgttgttgttgagacagggtctcactctgtcacccaggctggagtgcagtggt 66588
human.FAM230E-LOC105377182-GGT3P.NCBI.                                     ------------------------------------------------------------ 57310
human.FAM230J-LOC105372942-GGTLC5P.NCBI.rev.compl                          ------------------------------------------------------------ 46364
human.FAM230A.ncRNA.18487127.18500594-GGTLC3.18516335.18518165.rev.compl.REF ------------------------------------------------------------ 31039

chimp.LOC112206744-LOC107973052-GGT2.revcompl                              ------------------------------------------------------------ 61762
chimp54546-135457.revcompl.Pan.troglodytes.clone.rp43-41g5.GenBank:AC099533.36 ATGATCTCGGCTCACTACAACTTCCACCTCCCGGGTTCAAGCAATTGTCCTGCCTCAGCC 67273
human.FAM30B-LOC105372935-GGT2.NCBI.                                       gcaatcatagctcactgcagccttgacctctgaggctcaagtgaccctcccacatcagcc 66648
```

```
human.FAM230E-LOC105377182-GGT3P.NCBI.                                   ------------------------------------------------------------ 57310
human.FAM230J-LOC105372942-GGTLC5P.NCBI.rev.compl                        ------------------------------------------------------------ 46364
human.FAM230A.ncRNA.18487127.18500594-GGTLC3.18516335.18518165.rev.compl.REF ------------------------------------------------------------ 31039

chimp.LOC112206744-LOC107973052-GGT2.revcompl                            ------------------------------------------------------------ 61762
chimp54546-135457.revcompl.Pan.troglodytes.clone.rp43-41g5.GenBank:AC099533.36 TCCCAAGTAGCTGGGATTACAGGCACCCACCACCACGCCCGGCTAATTGGGAGATAGTTT 67333
human.FAM30B-LOC105372935-GGT2.NCBI.                                     tcctgagtatctgggaccaccagcacacaccaccatgcctggctaactttttatttctt 66708
human.FAM230E-LOC105377182-GGT3P.NCBI.                                   ------------------------------------------------------------ 57310
human.FAM230J-LOC105372942-GGTLC5P.NCBI.rev.compl                        ------------------------------------------------------------ 46364
human.FAM230A.ncRNA.18487127.18500594-GGTLC3.18516335.18518165.rev.compl.REF ------------------------------------------------------------ 31039

chimp.LOC112206744-LOC107973052-GGT2.revcompl                            ------------------------------------------------------------ 61762
chimp54546-135457.revcompl.Pan.troglodytes.clone.rp43-41g5.GenBank:AC099533.36 G----------------------------------------------------------- 67334
human.FAM30B-LOC105372935-GGT2.NCBI.                                     atagagatgggatgatatggttggggtctgtgtacccacctaaatctcacgttcaattat 66768
human.FAM230E-LOC105377182-GGT3P.NCBI.                                   ------------------------------------------------------------ 57310
human.FAM230J-LOC105372942-GGTLC5P.NCBI.rev.compl                        ------------------------------------------------------------ 46364
human.FAM230A.ncRNA.18487127.18500594-GGTLC3.18516335.18518165.rev.compl.REF ------------------------------------------------------------ 31039

chimp.LOC112206744-LOC107973052-GGT2.revcompl                            ------------------------------------------------------------ 61762
chimp54546-135457.revcompl.Pan.troglodytes.clone.rp43-41g5.GenBank:AC099533.36 ------------------------------------------------------------ 67334
human.FAM30B-LOC105372935-GGT2.NCBI.                                     aatccccaatgttagaggtggggcctggtgggaggtgactggatcacaggaatggatctt 66828
human.FAM230E-LOC105377182-GGT3P.NCBI.                                   ------------------------------------------------------------ 57310
human.FAM230J-LOC105372942-GGTLC5P.NCBI.rev.compl                        ------------------------------------------------------------ 46364
human.FAM230A.ncRNA.18487127.18500594-GGTLC3.18516335.18518165.rev.compl.REF ------------------------------------------------------------ 31039

chimp.LOC112206744-LOC107973052-GGT2.revcompl                            ------------------------------------------------------------ 61762
chimp54546-135457.revcompl.Pan.troglodytes.clone.rp43-41g5.GenBank:AC099533.36 ------------------------------------------------------------ 67334
human.FAM30B-LOC105372935-GGT2.NCBI.                                     tcatgaatgatttagcatcaccctttggtgctgtgcttgttagagttctcccagtatct 66888
human.FAM230E-LOC105377182-GGT3P.NCBI.                                   ------------------------------------------------------------ 57310
human.FAM230J-LOC105372942-GGTLC5P.NCBI.rev.compl                        ------------------------------------------------------------ 46364
human.FAM230A.ncRNA.18487127.18500594-GGTLC3.18516335.18518165.rev.compl.REF ------------------------------------------------------------ 31039

chimp.LOC112206744-LOC107973052-GGT2.revcompl                            ------------------------------------------------------------ 61762
chimp54546-135457.revcompl.Pan.troglodytes.clone.rp43-41g5.GenBank:AC099533.36 ------------------------------------------------------------ 67334
human.FAM30B-LOC105372935-GGT2.NCBI.                                     aattgtttaaaagtgtgtggcacctccccctctctctcttgctcctgctttggccatgt 66948
human.FAM230E-LOC105377182-GGT3P.NCBI.                                   ------------------------------------------------------------ 57310
human.FAM230J-LOC105372942-GGTLC5P.NCBI.rev.compl                        ------------------------------------------------------------ 46364
human.FAM230A.ncRNA.18487127.18500594-GGTLC3.18516335.18518165.rev.compl.REF ------------------------------------------------------------ 31039

chimp.LOC112206744-LOC107973052-GGT2.revcompl                            ------------------------------------------------------------ 61762
chimp54546-135457.revcompl.Pan.troglodytes.clone.rp43-41g5.GenBank:AC099533.36 ------------------------------------------------------------ 67334
human.FAM30B-LOC105372935-GGT2.NCBI.                                     aaggcgtgcctgcttccccttcaccttccaccatgattgaaagtttcctgtgcttgcccc 67008
human.FAM230E-LOC105377182-GGT3P.NCBI.                                   ------------------------------------------------------------ 57310
human.FAM230J-LOC105372942-GGTLC5P.NCBI.rev.compl                        ------------------------------------------------------------ 46364
human.FAM230A.ncRNA.18487127.18500594-GGTLC3.18516335.18518165.rev.compl.REF ------------------------------------------------------------ 31039

chimp.LOC112206744-LOC107973052-GGT2.revcompl                            ------------------------------------------------------------ 61762
chimp54546-135457.revcompl.Pan.troglodytes.clone.rp43-41g5.GenBank:AC099533.36 ------------------------------------------------------------ 67334
human.FAM30B-LOC105372935-GGT2.NCBI.                                     agaagctgagcagatgccagcattatgctccctgaacagcctgtggaactgcgagacaat 67068
human.FAM230E-LOC105377182-GGT3P.NCBI.                                   ------------------------------------------------------------ 57310
human.FAM230J-LOC105372942-GGTLC5P.NCBI.rev.compl                        ------------------------------------------------------------ 46364
human.FAM230A.ncRNA.18487127.18500594-GGTLC3.18516335.18518165.rev.compl.REF ------------------------------------------------------------ 31039

chimp.LOC112206744-LOC107973052-GGT2.revcompl                            ------------------------------------------------------------ 61762
chimp54546-135457.revcompl.Pan.troglodytes.clone.rp43-41g5.GenBank:AC099533.36 ------------------------------------------------------------ 67334
human.FAM30B-LOC105372935-GGT2.NCBI.                                     taaacctcttttctttataaattctccagtctcatgtatttatagcaatgtgagaactga 67128
human.FAM230E-LOC105377182-GGT3P.NCBI.                                   ------------------------------------------------------------ 57310
human.FAM230J-LOC105372942-GGTLC5P.NCBI.rev.compl                        ------------------------------------------------------------ 46364
human.FAM230A.ncRNA.18487127.18500594-GGTLC3.18516335.18518165.rev.compl.REF ------------------------------------------------------------ 31039

chimp.LOC112206744-LOC107973052-GGT2.revcompl                            ------------------------------------------------------------ 61762
chimp54546-135457.revcompl.Pan.troglodytes.clone.rp43-41g5.GenBank:AC099533.36 ------------------------------------------------------------ 67334
human.FAM30B-LOC105372935-GGT2.NCBI.                                     ctaatacatagggtctcactgtgtttcccaggctggtctcaaactcctgggctcaagtaa 67188
human.FAM230E-LOC105377182-GGT3P.NCBI.                                   ------------------------------------------------------------ 57310
human.FAM230J-LOC105372942-GGTLC5P.NCBI.rev.compl                        ------------------------------------------------------------ 46364
human.FAM230A.ncRNA.18487127.18500594-GGTLC3.18516335.18518165.rev.compl.REF ------------------------------------------------------------ 31039

chimp.LOC112206744-LOC107973052-GGT2.revcompl                            ------------------------------------------------------------ 61762
chimp54546-135457.revcompl.Pan.troglodytes.clone.rp43-41g5.GenBank:AC099533.36 ------------------------------------------------------------ 67334
human.FAM30B-LOC105372935-GGT2.NCBI.                                     tcctcctggctttgcttcccagagtgtagagattacaggcatgagccactgaacctgccc 67248
human.FAM230E-LOC105377182-GGT3P.NCBI.                                   ------------------------------------------------------------ 57310
human.FAM230J-LOC105372942-GGTLC5P.NCBI.rev.compl                        ------------------------------------------------------------ 46364
human.FAM230A.ncRNA.18487127.18500594-GGTLC3.18516335.18518165.rev.compl.REF ------------------------------------------------------------ 31039

chimp.LOC112206744-LOC107973052-GGT2.revcompl                            ------------------------------------------------------------ 61762
chimp54546-135457.revcompl.Pan.troglodytes.clone.rp43-41g5.GenBank:AC099533.36 ------------------------------------------------------------ 67334
human.FAM30B-LOC105372935-GGT2.NCBI.                                     ctttcccccaacaacatcttgattgcaacctcctaagagactccgagtcagaagtatcc 67308
human.FAM230E-LOC105377182-GGT3P.NCBI.                                   ------------------------------------------------------------ 57310
human.FAM230J-LOC105372942-GGTLC5P.NCBI.rev.compl                        ------------------------------------------------------------ 46364
human.FAM230A.ncRNA.18487127.18500594-GGTLC3.18516335.18518165.rev.compl.REF ------------------------------------------------------------ 31039

chimp.LOC112206744-LOC107973052-GGT2.revcompl                            ------------------------------------------------------------ 61762
chimp54546-135457.revcompl.Pan.troglodytes.clone.rp43-41g5.GenBank:AC099533.36 ------------------------------------------------------------ 67334
human.FAM30B-LOC105372935-GGT2.NCBI.                                     agttaagatgttactgactttctgaactacagaaactgtgagataataaacatctattgt 67368
human.FAM230E-LOC105377182-GGT3P.NCBI.                                   ------------------------------------------------------------ 57310
human.FAM230J-LOC105372942-GGTLC5P.NCBI.rev.compl                        ------------------------------------------------------------ 46364
human.FAM230A.ncRNA.18487127.18500594-GGTLC3.18516335.18518165.rev.compl.REF ------------------------------------------------------------ 31039

chimp.LOC112206744-LOC107973052-GGT2.revcompl                            ------------------------------------------------------------ 61762
chimp54546-135457.revcompl.Pan.troglodytes.clone.rp43-41g5.GenBank:AC099533.36 ------------------------------------------------------------ 67334
human.FAM30B-LOC105372935-GGT2.NCBI.                                     tcctttaagccttaagtttggggcttttttttttacaccaatatataactaatgtagac 67428
human.FAM230E-LOC105377182-GGT3P.NCBI.                                   ------------------------------------------------------------ 57310
human.FAM230J-LOC105372942-GGTLC5P.NCBI.rev.compl                        ------------------------------------------------------------ 46364
human.FAM230A.ncRNA.18487127.18500594-GGTLC3.18516335.18518165.rev.compl.REF ------------------------------------------------------------ 31039

chimp.LOC112206744-LOC107973052-GGT2.revcompl                            ------------------------------------------------------------ 61762
chimp54546-135457.revcompl.Pan.troglodytes.clone.rp43-41g5.GenBank:AC099533.36 ------------------------------------------------------------ 67334
human.FAM30B-LOC105372935-GGT2.NCBI.                                     acctgcagtccctaagattgaatgcaaaattgtgttacatacacatgagtgtgttcttct 67488
human.FAM230E-LOC105377182-GGT3P.NCBI.                                   ------------------------------------------------------------ 57310
human.FAM230J-LOC105372942-GGTLC5P.NCBI.rev.compl                        ------------------------------------------------------------ 46364
human.FAM230A.ncRNA.18487127.18500594-GGTLC3.18516335.18518165.rev.compl.REF ------------------------------------------------------------ 31039

chimp.LOC112206744-LOC107973052-GGT2.revcompl                            ------------------------------------------------------------ 61762
chimp54546-135457.revcompl.Pan.troglodytes.clone.rp43-41g5.GenBank:AC099533.36 ------------------------------------------------------------ 67334
human.FAM30B-LOC105372935-GGT2.NCBI.                                     gggaaaagagtccataaacttcaaaagattctcaaaggaatccaagacccaaaaagatta 67548
human.FAM230E-LOC105377182-GGT3P.NCBI.                                   ------------------------------------------------------------ 57310
human.FAM230J-LOC105372942-GGTLC5P.NCBI.rev.compl                        ------------------------------------------------------------ 46364
human.FAM230A.ncRNA.18487127.18500594-GGTLC3.18516335.18518165.rev.compl.REF ------------------------------------------------------------ 31039

chimp.LOC112206744-LOC107973052-GGT2.revcompl                            ------------------------------------------------------------ 61762
chimp54546-135457.revcompl.Pan.troglodytes.clone.rp43-41g5.GenBank:AC099533.36 ------------------------------------------------------------ 67334
human.FAM30B-LOC105372935-GGT2.NCBI.                                     agaaacattgcatcagacaataaattgtcaatgagcaaggtcctgtccttttagattggg 67608
human.FAM230E-LOC105377182-GGT3P.NCBI.                                   ------------------------------------------------------------ 57310
human.FAM230J-LOC105372942-GGTLC5P.NCBI.rev.compl                        ------------------------------------------------------------ 46364
human.FAM230A.ncRNA.18487127.18500594-GGTLC3.18516335.18518165.rev.compl.REF ------------------------------------------------------------ 31039

chimp.LOC112206744-LOC107973052-GGT2.revcompl                            ------------------------------------------------------------ 61762
chimp54546-135457.revcompl.Pan.troglodytes.clone.rp43-41g5.GenBank:AC099533.36 ------------------------------------------------------------ 67334
human.FAM30B-LOC105372935-GGT2.NCBI.                                     ttttgaaatcacccatcccacaaatatatgataccacttaactagtttttcaagatattc 67668
human.FAM230E-LOC105377182-GGT3P.NCBI.                                   ------------------------------------------------------------ 57310
human.FAM230J-LOC105372942-GGTLC5P.NCBI.rev.compl                        ------------------------------------------------------------ 46364
human.FAM230A.ncRNA.18487127.18500594-GGTLC3.18516335.18518165.rev.compl.REF ------------------------------------------------------------ 31039

chimp.LOC112206744-LOC107973052-GGT2.revcompl                            ------------------------------------------------------------ 61762
chimp54546-135457.revcompl.Pan.troglodytes.clone.rp43-41g5.GenBank:AC099533.36 ------------------------------------------------------------ 67334
human.FAM30B-LOC105372935-GGT2.NCBI.                                     cttgttcaaagatgctcccactttttctttgtgctcaagtcctagataaacttggctagg 67728
human.FAM230E-LOC105377182-GGT3P.NCBI.                                   ------------------------------------------------------------ 57310
```

```
human.FAM230J-LOC105372942-GGTLC5P.NCBI.rev.compl                                  ------------------------------------------------------------ 46364
human.FAM230A.ncRNA.18487127.18500594-GGTLC3.18516335.18518165.rev.compl.REF       ------------------------------------------------------------ 31039

chimp.LOC112206744-LOC107973052-GGT2.revcompl                                      ------------------------------------------------------------ 61762
chimp54546-135457.revcompl.Pan.troglodytes.clone.rp43-41g5.GenBank:AC099533.36     ------------------------------------------------------------ 67334
human.FAM30B-LOC105372935-GGT2.NCBI.                                               ggcaggaggtatgattcaggagttagtgagccgggagggctcactgtgcctgggaaagcc 67788
human.FAM230E-LOC105377182-GGT3P.NCBI.                                             ------------------------------------------------------------ 57310
human.FAM230J-LOC105372942-GGTLC5P.NCBI.rev.compl                                  ------------------------------------------------------------ 46364
human.FAM230A.ncRNA.18487127.18500594-GGTLC3.18516335.18518165.rev.compl.REF       ------------------------------------------------------------ 31039

chimp.LOC112206744-LOC107973052-GGT2.revcompl                                      ------------------------------------------------------------ 61762
chimp54546-135457.revcompl.Pan.troglodytes.clone.rp43-41g5.GenBank:AC099533.36     ------------------------------------------------------------ 67334
human.FAM30B-LOC105372935-GGT2.NCBI.                                               agcttctcatgaattttggaatatgccttggctcagaaacctacctctctaggcacagg 67848
human.FAM230E-LOC105377182-GGT3P.NCBI.                                             ------------------------------------------------------------ 57310
human.FAM230J-LOC105372942-GGTLC5P.NCBI.rev.compl                                  ------------------------------------------------------------ 46364
human.FAM230A.ncRNA.18487127.18500594-GGTLC3.18516335.18518165.rev.compl.REF       ------------------------------------------------------------ 31039

chimp.LOC112206744-LOC107973052-GGT2.revcompl                                      ------------------------------------------------------------ 61762
chimp54546-135457.revcompl.Pan.troglodytes.clone.rp43-41g5.GenBank:AC099533.36     ------------------------------------------------------------ 67334
human.FAM30B-LOC105372935-GGT2.NCBI.                                               atccaacttatacgttcaagatgcgtgactgagcatgtgaaagtgtcagagggatcgacc 67908
human.FAM230E-LOC105377182-GGT3P.NCBI.                                             ------------------------------------------------------------ 57310
human.FAM230J-LOC105372942-GGTLC5P.NCBI.rev.compl                                  ------------------------------------------------------------ 46364
human.FAM230A.ncRNA.18487127.18500594-GGTLC3.18516335.18518165.rev.compl.REF       ------------------------------------------------------------ 31039

chimp.LOC112206744-LOC107973052-GGT2.revcompl                                      ------------------------------------------------------------ 61762
chimp54546-135457.revcompl.Pan.troglodytes.clone.rp43-41g5.GenBank:AC099533.36     ------------------------------------------------------------ 67334
human.FAM30B-LOC105372935-GGT2.NCBI.                                               tcaccattttacatctacttttccagttgtggcccctacaatcatcaaggctcattcggc 67968
human.FAM230E-LOC105377182-GGT3P.NCBI.                                             ------------------------------------------------------------ 57310
human.FAM230J-LOC105372942-GGTLC5P.NCBI.rev.compl                                  ------------------------------------------------------------ 46364
human.FAM230A.ncRNA.18487127.18500594-GGTLC3.18516335.18518165.rev.compl.REF       ------------------------------------------------------------ 31039

chimp.LOC112206744-LOC107973052-GGT2.revcompl                                      ------------------------------------------------------------ 61762
chimp54546-135457.revcompl.Pan.troglodytes.clone.rp43-41g5.GenBank:AC099533.36     ------------------------------------------------------------ 67334
human.FAM30B-LOC105372935-GGT2.NCBI.                                               accatgctcctccaaattctgcatgcgactcagccacactaatatctttcttcatctata 68028
human.FAM230E-LOC105377182-GGT3P.NCBI.                                             ------------------------------------------------------------ 57310
human.FAM230J-LOC105372942-GGTLC5P.NCBI.rev.compl                                  ------------------------------------------------------------ 46364
human.FAM230A.ncRNA.18487127.18500594-GGTLC3.18516335.18518165.rev.compl.REF       ------------------------------------------------------------ 31039

chimp.LOC112206744-LOC107973052-GGT2.revcompl                                      ------------------------------------------------------------ 61762
chimp54546-135457.revcompl.Pan.troglodytes.clone.rp43-41g5.GenBank:AC099533.36     ------------------------------------------------------------ 67334
human.FAM30B-LOC105372935-GGT2.NCBI.                                               agatgaaagtaatacaacgcaccatgtagcaaactcacaggagaccctagagatcaaata 68088
human.FAM230E-LOC105377182-GGT3P.NCBI.                                             ------------------------------------------------------------ 57310
human.FAM230J-LOC105372942-GGTLC5P.NCBI.rev.compl                                  ------------------------------------------------------------ 46364
human.FAM230A.ncRNA.18487127.18500594-GGTLC3.18516335.18518165.rev.compl.REF       ------------------------------------------------------------ 31039

chimp.LOC112206744-LOC107973052-GGT2.revcompl                                      ------------------------------------------------------------ 61762
chimp54546-135457.revcompl.Pan.troglodytes.clone.rp43-41g5.GenBank:AC099533.36     -----------------------------------------------ATACAAACTATAC 67347
human.FAM30B-LOC105372935-GGT2.NCBI.                                               acactgtgaaagccaccaaatgaatattttaaaattttatctcatttctgccaaggtgag 68148
human.FAM230E-LOC105377182-GGT3P.NCBI.                                             ------------------------------------------------------------ 57310
human.FAM230J-LOC105372942-GGTLC5P.NCBI.rev.compl                                  ------------------------------------------------------------ 46364
human.FAM230A.ncRNA.18487127.18500594-GGTLC3.18516335.18518165.rev.compl.REF       ------------------------------------------------------------ 31039

chimp.LOC112206744-LOC107973052-GGT2.revcompl                                      ------------------------------------------------------------ 61762
chimp54546-135457.revcompl.Pan.troglodytes.clone.rp43-41g5.GenBank:AC099533.36     AAATTGAAGTATAAAGAGAAAAATAGATAGAACATACAGGAAATTGATTAAGAG----AC 67403
human.FAM30B-LOC105372935-GGT2.NCBI.                                               atatcaatctaaaaacaggataaatctttgtaagtacaagaacattaaaaagaacattaa 68208
human.FAM230E-LOC105377182-GGT3P.NCBI.                                             ------------------------------------------------------------ 57310
human.FAM230J-LOC105372942-GGTLC5P.NCBI.rev.compl                                  ------------------------------------------------------------ 46364
human.FAM230A.ncRNA.18487127.18500594-GGTLC3.18516335.18518165.rev.compl.REF       ------------------------------------------------------------ 31039

chimp.LOC112206744-LOC107973052-GGT2.revcompl                                      ------------------------------------------------------------ 61762
chimp54546-135457.revcompl.Pan.troglodytes.clone.rp43-41g5.GenBank:AC099533.36     ATGGGGATTAACAAGGCATAAGTC------------------------------------ 67427
human.FAM30B-LOC105372935-GGT2.NCBI.                                               gtttgggttaaaaatgcaaaactgcatatgcaggaaaattacgttaaaaaaaaaaaaatc 68268
human.FAM230E-LOC105377182-GGT3P.NCBI.                                             ------------------------------------------------------------ 57310
human.FAM230J-LOC105372942-GGTLC5P.NCBI.rev.compl                                  ------------------------------------------------------------ 46364
human.FAM230A.ncRNA.18487127.18500594-GGTLC3.18516335.18518165.rev.compl.REF       ------------------------------------------------------------ 31039

chimp.LOC112206744-LOC107973052-GGT2.revcompl                                      ------------------------------------------------------------ 61762
chimp54546-135457.revcompl.Pan.troglodytes.clone.rp43-41g5.GenBank:AC099533.36     ------------------------------------------------------------ 67427
human.FAM30B-LOC105372935-GGT2.NCBI.                                               ctacacctacaaaaacaaccaaacaaaacattgatgatactgggctgattcactgaatgg 68328
human.FAM230E-LOC105377182-GGT3P.NCBI.                                             ------------------------------------------------------------ 57310
human.FAM230J-LOC105372942-GGTLC5P.NCBI.rev.compl                                  ------------------------------------------------------------ 46364
human.FAM230A.ncRNA.18487127.18500594-GGTLC3.18516335.18518165.rev.compl.REF       ------------------------------------------------------------ 31039

chimp.LOC112206744-LOC107973052-GGT2.revcompl                                      ------------------------------------------------------------ 61762
chimp54546-135457.revcompl.Pan.troglodytes.clone.rp43-41g5.GenBank:AC099533.36     ------------------------------------------------------------ 67427
human.FAM30B-LOC105372935-GGT2.NCBI.                                               tgagaatatgagtgattttctcctctcctatttttctcaatacctttttctctattatctc 68388
human.FAM230E-LOC105377182-GGT3P.NCBI.                                             ------------------------------------------------------------ 57310
human.FAM230J-LOC105372942-GGTLC5P.NCBI.rev.compl                                  ------------------------------------------------------------ 46364
human.FAM230A.ncRNA.18487127.18500594-GGTLC3.18516335.18518165.rev.compl.REF       ------------------------------------------------------------ 31039

chimp.LOC112206744-LOC107973052-GGT2.revcompl                                      ------------------------------------------------------------ 61762
chimp54546-135457.revcompl.Pan.troglodytes.clone.rp43-41g5.GenBank:AC099533.36     ------------------------------------------------------------ 67427
human.FAM30B-LOC105372935-GGT2.NCBI.                                               ctttttaaataaacaagtatatattgttctaaaccgaagataaatgatcatgagcagca 68448
human.FAM230E-LOC105377182-GGT3P.NCBI.                                             ------------------------------------------------------------ 57310
human.FAM230J-LOC105372942-GGTLC5P.NCBI.rev.compl                                  ------------------------------------------------------------ 46364
human.FAM230A.ncRNA.18487127.18500594-GGTLC3.18516335.18518165.rev.compl.REF       ------------------------------------------------------------ 31039

chimp.LOC112206744-LOC107973052-GGT2.revcompl                                      ------------------------------------------------------------ 61762
chimp54546-135457.revcompl.Pan.troglodytes.clone.rp43-41g5.GenBank:AC099533.36     ------------------------------------------------------------ 67427
human.FAM30B-LOC105372935-GGT2.NCBI.                                               ctttcccaggcactgtcctagttgctctgcatacatcatcgcatttaatcctcacaacag 68508
human.FAM230E-LOC105377182-GGT3P.NCBI.                                             ------------------------------------------------------------ 57310
human.FAM230J-LOC105372942-GGTLC5P.NCBI.rev.compl                                  ------------------------------------------------------------ 46364
human.FAM230A.ncRNA.18487127.18500594-GGTLC3.18516335.18518165.rev.compl.REF       ------------------------------------------------------------ 31039

chimp.LOC112206744-LOC107973052-GGT2.revcompl                                      ------------------------------------------------------------ 61762
chimp54546-135457.revcompl.Pan.troglodytes.clone.rp43-41g5.GenBank:AC099533.36     ------------------------------------------------------------ 67427
human.FAM30B-LOC105372935-GGT2.NCBI.                                               tgaatggggtcgatttcattacagccacatttaccagacaaggaaatggttttcaaggtt 68568
human.FAM230E-LOC105377182-GGT3P.NCBI.                                             ------------------------------------------------------------ 57310
human.FAM230J-LOC105372942-GGTLC5P.NCBI.rev.compl                                  ------------------------------------------------------------ 46364
human.FAM230A.ncRNA.18487127.18500594-GGTLC3.18516335.18518165.rev.compl.REF       ------------------------------------------------------------ 31039

chimp.LOC112206744-LOC107973052-GGT2.revcompl                                      ------------------------------------------------------------ 61762
chimp54546-135457.revcompl.Pan.troglodytes.clone.rp43-41g5.GenBank:AC099533.36     ------------------------------------------------------------ 67427
human.FAM30B-LOC105372935-GGT2.NCBI.                                               aagtaaccttctcatgacacagaatgtgactaagagttttcttattccatggctcacact 68628
human.FAM230E-LOC105377182-GGT3P.NCBI.                                             ------------------------------------------------------------ 57310
human.FAM230J-LOC105372942-GGTLC5P.NCBI.rev.compl                                  ------------------------------------------------------------ 46364
human.FAM230A.ncRNA.18487127.18500594-GGTLC3.18516335.18518165.rev.compl.REF       ------------------------------------------------------------ 31039

chimp.LOC112206744-LOC107973052-GGT2.revcompl                                      ------------------------------------------------------------ 61762
chimp54546-135457.revcompl.Pan.troglodytes.clone.rp43-41g5.GenBank:AC099533.36     -----------------------------------------ATGTGAAATTGGAGTCTCA 67446
human.FAM30B-LOC105372935-GGT2.NCBI.                                               aaatgtgctctgtgaatatcagtgagaagtaataaagcactgactggattttcttggtca 68688
human.FAM230E-LOC105377182-GGT3P.NCBI.                                             ------------------------------------------------------------ 57310
human.FAM230J-LOC105372942-GGTLC5P.NCBI.rev.compl                                  ------------------------------------------------------------ 46364
human.FAM230A.ncRNA.18487127.18500594-GGTLC3.18516335.18518165.rev.compl.REF       ------------------------------------------------------------ 31039

chimp.LOC112206744-LOC107973052-GGT2.revcompl                                      ------------------------------------------------------------ 61762
chimp54546-135457.revcompl.Pan.troglodytes.clone.rp43-41g5.GenBank:AC099533.36     TAAGGAGAGTAGAGAGAGGATGGGGAAGACGTAATACTTGAAAAGATAAAGCTGACAGTT 67506
human.FAM30B-LOC105372935-GGT2.NCBI.                                               ccgggtcctaaaagaaatgttggggaat--ctatgcagtcccaagttaaaaatgggaact 68746
human.FAM230E-LOC105377182-GGT3P.NCBI.                                             ------------------------------------------------------------ 57310
human.FAM230J-LOC105372942-GGTLC5P.NCBI.rev.compl                                  ------------------------------------------------------------ 46364
human.FAM230A.ncRNA.18487127.18500594-GGTLC3.18516335.18518165.rev.compl.REF       ------------------------------------------------------------ 31039

chimp.LOC112206744-LOC107973052-GGT2.revcompl                                      ------------------------------------------------------------ 61762
chimp54546-135457.revcompl.Pan.troglodytes.clone.rp43-41g5.GenBank:AC099533.36     TTCTAAAATGGACAAAAAAGAATCAAGTCATGGAATGAAGAGCCACCTAAATGCATCAGT 67566
human.FAM30B-LOC105372935-GGT2.NCBI.                                               gaactgagctgccacagcatgctgaataggtggagtccag-gccaactaagtgcttcatg 68805
human.FAM230E-LOC105377182-GGT3P.NCBI.                                             ------------------------------------------------------------ 57310
human.FAM230J-LOC105372942-GGTLC5P.NCBI.rev.compl                                  ------------------------------------------------------------ 46364
```

```
human.FAM230A.ncRNA.18487127.18500594-GGTLC3.18516335.18518165.rev.compl.REF        ------------------------------------------------------------ 31039

chimp.LOC112206744-LOC107973052-GGT2.revcompl                                       ------------------------------------------------------------ 61762
chimp54546-135457.revcompl.Pan.troglodytes.clone.rp43-41g5.GenBank:AC099533.36      G---TAAACTGCTCAAAACAAAGACAAAACTTTTCAAAGCAGCCAGAGGAAAAAAGATAC 67623
human.FAM30B-LOC105372935-GGT2.NCBI.                                                gaacttaaaggctccagagggaaagcaatcgggaaggcacttccacaggcacatcggtga 68865
human.FAM230E-LOC105377182-GGT3P.NCBI.                                              ------------------------------------------------------------ 57310
human.FAM230J-LOC105372942-GGTLC5P.NCBI.rev.compl                                   ------------------------------------------------------------ 46364
human.FAM230A.ncRNA.18487127.18500594-GGTLC3.18516335.18518165.rev.compl.REF        ------------------------------------------------------------ 31039

chimp.LOC112206744-LOC107973052-GGT2.revcompl                                       ------------------------------------------------------------ 61762
chimp54546-135457.revcompl.Pan.troglodytes.clone.rp43-41g5.GenBank:AC099533.36      TGCCTTCAGGCCAGGGTT------------------------------------------ 67641
human.FAM30B-LOC105372935-GGT2.NCBI.                                                caacttctggggattcttacctgtcagccttcagccaaggaaggacaggaagtggcatgg 68925
human.FAM230E-LOC105377182-GGT3P.NCBI.                                              ------------------------------------------------------------ 57310
human.FAM230J-LOC105372942-GGTLC5P.NCBI.rev.compl                                   ------------------------------------------------------------ 46364
human.FAM230A.ncRNA.18487127.18500594-GGTLC3.18516335.18518165.rev.compl.REF        ------------------------------------------------------------ 31039

chimp.LOC112206744-LOC107973052-GGT2.revcompl                                       ------------------------------------------------------------ 61762
chimp54546-135457.revcompl.Pan.troglodytes.clone.rp43-41g5.GenBank:AC099533.36      ------------------------------------------------------------ 67641
human.FAM30B-LOC105372935-GGT2.NCBI.                                                gctaaaagatcctttgtttgacctctttccagatcaggtgacaggaaaacacaacacttg 68985
human.FAM230E-LOC105377182-GGT3P.NCBI.                                              ------------------------------------------------------------ 57310
human.FAM230J-LOC105372942-GGTLC5P.NCBI.rev.compl                                   ------------------------------------------------------------ 46364
human.FAM230A.ncRNA.18487127.18500594-GGTLC3.18516335.18518165.rev.compl.REF        ------------------------------------------------------------ 31039

chimp.LOC112206744-LOC107973052-GGT2.revcompl                                       ------------------------------------------------------------ 61762
chimp54546-135457.revcompl.Pan.troglodytes.clone.rp43-41g5.GenBank:AC099533.36      ------------------------------------------------------------ 67641
human.FAM30B-LOC105372935-GGT2.NCBI.                                                aacctttccaataaggacagccagcacttactgagccccagccaggtaactgacactgtt 69045
human.FAM230E-LOC105377182-GGT3P.NCBI.                                              ------------------------------------------------------------ 57310
human.FAM230J-LOC105372942-GGTLC5P.NCBI.rev.compl                                   ------------------------------------------------------------ 46364
human.FAM230A.ncRNA.18487127.18500594-GGTLC3.18516335.18518165.rev.compl.REF        ------------------------------------------------------------ 31039

chimp.LOC112206744-LOC107973052-GGT2.revcompl                                       ------------------------------------------------------------ 61762
chimp54546-135457.revcompl.Pan.troglodytes.clone.rp43-41g5.GenBank:AC099533.36      ------------------------------------------------------------ 67641
human.FAM30B-LOC105372935-GGT2.NCBI.                                                ccaagtactctgcttgtataaccagctttaaccttgcaacagccctctgaggtaaatact 69105
human.FAM230E-LOC105377182-GGT3P.NCBI.                                              ------------------------------------------------------------ 57310
human.FAM230J-LOC105372942-GGTLC5P.NCBI.rev.compl                                   ------------------------------------------------------------ 46364
human.FAM230A.ncRNA.18487127.18500594-GGTLC3.18516335.18518165.rev.compl.REF        ------------------------------------------------------------ 31039

chimp.LOC112206744-LOC107973052-GGT2.revcompl                                       ------------------------------------------------------------ 61762
chimp54546-135457.revcompl.Pan.troglodytes.clone.rp43-41g5.GenBank:AC099533.36      ------------------------------------------------------------ 67641
human.FAM30B-LOC105372935-GGT2.NCBI.                                                cctagtgacctcactttacggatgcagaacctgaggcaaggagttagggagtaaatgaag 69165
human.FAM230E-LOC105377182-GGT3P.NCBI.                                              ------------------------------------------------------------ 57310
human.FAM230J-LOC105372942-GGTLC5P.NCBI.rev.compl                                   ------------------------------------------------------------ 46364
human.FAM230A.ncRNA.18487127.18500594-GGTLC3.18516335.18518165.rev.compl.REF        ------------------------------------------------------------ 31039

chimp.LOC112206744-LOC107973052-GGT2.revcompl                                       ------------------------------------------------------------ 61762
chimp54546-135457.revcompl.Pan.troglodytes.clone.rp43-41g5.GenBank:AC099533.36      ------------------------------------------------------------ 67641
human.FAM30B-LOC105372935-GGT2.NCBI.                                                ctggctaaaggcacttgcactggccaggcacagtggctcatgcctgtaatcccagcactt 69225
human.FAM230E-LOC105377182-GGT3P.NCBI.                                              ------------------------------------------------------------ 57310
human.FAM230J-LOC105372942-GGTLC5P.NCBI.rev.compl                                   ------------------------------------------------------------ 46364
human.FAM230A.ncRNA.18487127.18500594-GGTLC3.18516335.18518165.rev.compl.REF        ------------------------------------------------------------ 31039

chimp.LOC112206744-LOC107973052-GGT2.revcompl                                       ------------------------------------------------------------ 61762
chimp54546-135457.revcompl.Pan.troglodytes.clone.rp43-41g5.GenBank:AC099533.36      ------------------------------------------------------------ 67641
human.FAM30B-LOC105372935-GGT2.NCBI.                                                ccagatggatcacttgaggttaggggttcgagaccagcctggccaacatggtgaatcccc 69285
human.FAM230E-LOC105377182-GGT3P.NCBI.                                              ------------------------------------------------------------ 57310
human.FAM230J-LOC105372942-GGTLC5P.NCBI.rev.compl                                   ------------------------------------------------------------ 46364
human.FAM230A.ncRNA.18487127.18500594-GGTLC3.18516335.18518165.rev.compl.REF        ------------------------------------------------------------ 31039

chimp.LOC112206744-LOC107973052-GGT2.revcompl                                       ------------------------------------------------------------ 61762
chimp54546-135457.revcompl.Pan.troglodytes.clone.rp43-41g5.GenBank:AC099533.36      ------------------------------GGGGGCTGGCTCATGCCTGTAATCCCAGCA 67671
human.FAM30B-LOC105372935-GGT2.NCBI.                                                agctctactaaaaatacaaaaattagccgggtgtgatggcacaagcctgtaatcccagca 69345
human.FAM230E-LOC105377182-GGT3P.NCBI.                                              ------------------------------------------------------------ 57310
human.FAM230J-LOC105372942-GGTLC5P.NCBI.rev.compl                                   ------------------------------------------------------------ 46364
human.FAM230A.ncRNA.18487127.18500594-GGTLC3.18516335.18518165.rev.compl.REF        ------------------------------------------------------------ 31039

chimp.LOC112206744-LOC107973052-GGT2.revcompl                                       ------------------------------------------------------------ 61762
chimp54546-135457.revcompl.Pan.troglodytes.clone.rp43-41g5.GenBank:AC099533.36      -TTTGGGAGGCTGAGGCGGGCGGATCACCTGAAATGAGGAGTTTGGGATCAGCCTGACCA 67730
human.FAM30B-LOC105372935-GGT2.NCBI.                                                ctttgggaggccaaggcagatgggtcacttgaggttaggggttcgagaccagcctggcca 69405
human.FAM230E-LOC105377182-GGT3P.NCBI.                                              ------------------------------------------------------------ 57310
human.FAM230J-LOC105372942-GGTLC5P.NCBI.rev.compl                                   ------------------------------------------------------------ 46364
human.FAM230A.ncRNA.18487127.18500594-GGTLC3.18516335.18518165.rev.compl.REF        ------------------------------------------------------------ 31039

chimp.LOC112206744-LOC107973052-GGT2.revcompl                                       ------------------------------------------------------------ 61762
chimp54546-135457.revcompl.Pan.troglodytes.clone.rp43-41g5.GenBank:AC099533.36      ACATGGCAAAAGTTCATCTCTATTAAAAATACAACAATTAGCCAGGTGTGATGGTGGACA 67790
human.FAM30B-LOC105372935-GGT2.NCBI.                                                acatggtgaatccccagctctactaaaaatacaaaaattagccgggtgtgatggcacacg 69465
human.FAM230E-LOC105377182-GGT3P.NCBI.                                              ------------------------------------------------------------ 57310
human.FAM230J-LOC105372942-GGTLC5P.NCBI.rev.compl                                   ------------------------------------------------------------ 46364
human.FAM230A.ncRNA.18487127.18500594-GGTLC3.18516335.18518165.rev.compl.REF        ------------------------------------------------------------ 31039

chimp.LOC112206744-LOC107973052-GGT2.revcompl                                       ------------------------------------------------------------ 61762
chimp54546-135457.revcompl.Pan.troglodytes.clone.rp43-41g5.GenBank:AC099533.36      CCTGTAATCCCAGCTACTTGGGAGGCTGAGGCAGAAGAATCACTTGAACCCGGGAGGCAG 67850
human.FAM30B-LOC105372935-GGT2.NCBI.                                                cctgtaatcccagctactcaggaggctgaggcaggagaattgcttgaacctgggagatgg 69525
human.FAM230E-LOC105377182-GGT3P.NCBI.                                              ------------------------------------------------------------ 57310
human.FAM230J-LOC105372942-GGTLC5P.NCBI.rev.compl                                   ------------------------------------------------------------ 46364
human.FAM230A.ncRNA.18487127.18500594-GGTLC3.18516335.18518165.rev.compl.REF        ------------------------------------------------------------ 31039

chimp.LOC112206744-LOC107973052-GGT2.revcompl                                       ------------------------------------------------------------ 61762
chimp54546-135457.revcompl.Pan.troglodytes.clone.rp43-41g5.GenBank:AC099533.36      AGGTTGCAGTGAGCTGAGATTGCGCTACTGCACTCCAGCCTGGGCGACAGAGTAAGACTC 67910
human.FAM30B-LOC105372935-GGT2.NCBI.                                                aggttgcagcaagccgagatcgcaccactgcactccagcccactcggtcaatgtcactgc 69585
human.FAM230E-LOC105377182-GGT3P.NCBI.                                              ------------------------------------------------------------ 57310
human.FAM230J-LOC105372942-GGTLC5P.NCBI.rev.compl                                   ------------------------------------------------------------ 46364
human.FAM230A.ncRNA.18487127.18500594-GGTLC3.18516335.18518165.rev.compl.REF        ------------------------------------------------------------ 31039

chimp.LOC112206744-LOC107973052-GGT2.revcompl                                       ------------------------------------------------------------ 61762
chimp54546-135457.revcompl.Pan.troglodytes.clone.rp43-41g5.GenBank:AC099533.36      CATC-----TCAGAAAACACAACAGCATAAAAAGCCATTGCCTTCAAATGAGCAAAATTA 67965
human.FAM30B-LOC105372935-GGT2.NCBI.                                                catcaagggacagtctaaccaaggtccagatggcacaagtggagccataaaaccaggaca 69645
human.FAM230E-LOC105377182-GGT3P.NCBI.                                              ------------------------------------------------------------ 57310
human.FAM230J-LOC105372942-GGTLC5P.NCBI.rev.compl                                   ------------------------------------------------------------ 46364
human.FAM230A.ncRNA.18487127.18500594-GGTLC3.18516335.18518165.rev.compl.REF        ------------------------------------------------------------ 31039

chimp.LOC112206744-LOC107973052-GGT2.revcompl                                       ------------------------------------------------------------ 61762
chimp54546-135457.revcompl.Pan.troglodytes.clone.rp43-41g5.GenBank:AC099533.36      GATTGAGAATTGGCTTCCCAATAAAAGCAGGAGAAATCAGATACAAGGGAATCATGTTTT 68025
human.FAM30B-LOC105372935-GGT2.NCBI.                                                gcttgaggttcagcttcctaagccca-caggtgcaaggaccttatctggatccatacttt 69704
human.FAM230E-LOC105377182-GGT3P.NCBI.                                              ------------------------------------------------------------ 57310
human.FAM230J-LOC105372942-GGTLC5P.NCBI.rev.compl                                   ------------------------------------------------------------ 46364
human.FAM230A.ncRNA.18487127.18500594-GGTLC3.18516335.18518165.rev.compl.REF        ------------------------------------------------------------ 31039

chimp.LOC112206744-LOC107973052-GGT2.revcompl                                       ------------------------------------------------------------ 61762
chimp54546-135457.revcompl.Pan.troglodytes.clone.rp43-41g5.GenBank:AC099533.36      CAAACTGAGGAAAGAAAATATCTGCCAACCTAGAATTCTATGCTCACTAACAATATCCTT 68085
human.FAM30B-LOC105372935-GGT2.NCBI.                                                ca---------------------------------------------------------t 69707
human.FAM230E-LOC105377182-GGT3P.NCBI.                                              ------------------------------------------------------------ 57310
human.FAM230J-LOC105372942-GGTLC5P.NCBI.rev.compl                                   ------------------------------------------------------------ 46364
human.FAM230A.ncRNA.18487127.18500594-GGTLC3.18516335.18518165.rev.compl.REF        ------------------------------------------------------------ 31039

chimp.LOC112206744-LOC107973052-GGT2.revcompl                                       ------------------------------------------------------------ 61762
chimp54546-135457.revcompl.Pan.troglodytes.clone.rp43-41g5.GenBank:AC099533.36      CAAAAGTGAAAAT---GGCTGGGCATGGTGGCTCATGCCTGTAATCCCAGCACTTTGGAA 68142
human.FAM30B-LOC105372935-GGT2.NCBI.                                                caaaaatgtcccttgaggccaggcacagtggctcatgcctataatcccagcactttggga 69767
human.FAM230E-LOC105377182-GGT3P.NCBI.                                              ------------------------------------------------------------ 57310
human.FAM230J-LOC105372942-GGTLC5P.NCBI.rev.compl                                   ------------------------------------------------------------ 46364
human.FAM230A.ncRNA.18487127.18500594-GGTLC3.18516335.18518165.rev.compl.REF        ------------------------------------------------------------ 31039

chimp.LOC112206744-LOC107973052-GGT2.revcompl                                       ------------------------------------------------------------ 61762
chimp54546-135457.revcompl.Pan.troglodytes.clone.rp43-41g5.GenBank:AC099533.36      GGCCAAGGCAGGTGGATCACCTGAGGTCAGAAGTTCGAGACCAGCCTGGCCAACATGGTG 68202
human.FAM30B-LOC105372935-GGT2.NCBI.                                                gggcgaggcaggtggatcacctgaggtcaggagttcgagaccagcctggccaatatggtg 69827
human.FAM230E-LOC105377182-GGT3P.NCBI.                                              ------------------------------------------------------------ 57310
human.FAM230J-LOC105372942-GGTLC5P.NCBI.rev.compl                                   ------------------------------------------------------------ 46364
human.FAM230A.ncRNA.18487127.18500594-GGTLC3.18516335.18518165.rev.compl.REF        ------------------------------------------------------------ 31039
```

```
chimp.LOC112206744-LOC107973052-GGT2.revcompl                                          ------------------------------------------------------------ 61762
chimp54546-135457.revcompl.Pan.troglodytes.clone.rp43-41g5.GenBank:AC099533.36        AAACCCCATCTCTACCAAAAATACACAAA-TTAGCCAGATGTGGTGGTGGATGCCTGTAA 68261
human.FAM30B-LOC105372935-GGT2.NCBI.                                                   aaaccctgtccctactgaaaatacaaaaatttagcccagcatggtgatgtgcgcctgtaa 69887
human.FAM230E-LOC105377182-GGT3P.NCBI.                                                 ------------------------------------------------------------ 57310
human.FAM230J-LOC105372942-GGTLC5P.NCBI.rev.compl                                      ------------------------------------------------------------ 46364
human.FAM230A.ncRNA.18487127.18500594-GGTLC3.18516335.18518165.rev.compl.REF           ------------------------------------------------------------ 31039

chimp.LOC112206744-LOC107973052-GGT2.revcompl                                          ------------------------------------------------------------ 61762
chimp54546-135457.revcompl.Pan.troglodytes.clone.rp43-41g5.GenBank:AC099533.36        TCCCAGCTACTCAGGAGGCTGAGGCACAAGAATTGCTTGAACCCGAGAGGTGGAGGTTGC 68321
human.FAM30B-LOC105372935-GGT2.NCBI.                                                   tcccagctactccggaggctgaggcaggagaatcgcttgaacccagcaggtggaggttgc 69947
human.FAM230E-LOC105377182-GGT3P.NCBI.                                                 ------------------------------------------------------------ 57310
human.FAM230J-LOC105372942-GGTLC5P.NCBI.rev.compl                                      ------------------------------------------------------------ 46364
human.FAM230A.ncRNA.18487127.18500594-GGTLC3.18516335.18518165.rev.compl.REF           ------------------------------------------------------------ 31039

chimp.LOC112206744-LOC107973052-GGT2.revcompl                                          ------------------------------------------------------------ 61762
chimp54546-135457.revcompl.Pan.troglodytes.clone.rp43-41g5.GenBank:AC099533.36        AGTGAGCCAAGATCTTGCCACTGCACTCCAGCCTGGGTGACAGAGCAAAACTCCATCTCA 68381
human.FAM30B-LOC105372935-GGT2.NCBI.                                                   agtgagccgagatcatgtcattgcactgtgggctgggcaacagagcaacactctatgtaa 70007
human.FAM230E-LOC105377182-GGT3P.NCBI.                                                 ------------------------------------------------------------ 57310
human.FAM230J-LOC105372942-GGTLC5P.NCBI.rev.compl                                      ------------------------------------------------------------ 46364
human.FAM230A.ncRNA.18487127.18500594-GGTLC3.18516335.18518165.rev.compl.REF           ------------------------------------------------------------ 31039

chimp.LOC112206744-LOC107973052-GGT2.revcompl                                          ------------------------------------------------------------ 61762
chimp54546-135457.revcompl.Pan.troglodytes.clone.rp43-41g5.GenBank:AC099533.36        AAAAAAAAAACAGG--CCAGTCACGGTGGCTCATGCCT---------------------- 68417
human.FAM30B-LOC105372935-GGT2.NCBI.                                                   aaaaaaaaaaaaaggtcccttcaggattcctcgagcctgggaggccgaggctgccatgaa 70067
human.FAM230E-LOC105377182-GGT3P.NCBI.                                                 ------------------------------------------------------------ 57310
human.FAM230J-LOC105372942-GGTLC5P.NCBI.rev.compl                                      ------------------------------------------------------------ 46364
human.FAM230A.ncRNA.18487127.18500594-GGTLC3.18516335.18518165.rev.compl.REF           ------------------------------------------------------------ 31039

chimp.LOC112206744-LOC107973052-GGT2.revcompl                                          ------------------------------------------------------------ 61762
chimp54546-135457.revcompl.Pan.troglodytes.clone.rp43-41g5.GenBank:AC099533.36        ------------------------------------------------------------ 68417
human.FAM30B-LOC105372935-GGT2.NCBI.                                                   caatgattgtgccactgcactccagcctgggtgacagagcgagatcttgtctcaaaaaaa 70127
human.FAM230E-LOC105377182-GGT3P.NCBI.                                                 ------------------------------------------------------------ 57310
human.FAM230J-LOC105372942-GGTLC5P.NCBI.rev.compl                                      ------------------------------------------------------------ 46364
human.FAM230A.ncRNA.18487127.18500594-GGTLC3.18516335.18518165.rev.compl.REF           ------------------------------------------------------------ 31039

chimp.LOC112206744-LOC107973052-GGT2.revcompl                                          ------------------------------------------------------------ 61762
chimp54546-135457.revcompl.Pan.troglodytes.clone.rp43-41g5.GenBank:AC099533.36        ------------------------------------------------------------ 68417
human.FAM30B-LOC105372935-GGT2.NCBI.                                                   aaaaaaaaaaaaaaaatggctcttgaaacagccatcaactcccatgtgataaatgcactg 70187
human.FAM230E-LOC105377182-GGT3P.NCBI.                                                 ------------------------------------------------------------ 57310
human.FAM230J-LOC105372942-GGTLC5P.NCBI.rev.compl                                      ------------------------------------------------------------ 46364
human.FAM230A.ncRNA.18487127.18500594-GGTLC3.18516335.18518165.rev.compl.REF           ------------------------------------------------------------ 31039

chimp.LOC112206744-LOC107973052-GGT2.revcompl                                          ------------------------------------------------------------ 61762
chimp54546-135457.revcompl.Pan.troglodytes.clone.rp43-41g5.GenBank:AC099533.36        ------------------------------------------------------------ 68417
human.FAM30B-LOC105372935-GGT2.NCBI.                                                   ccacaatccagcagcaaacctaaagttcggagtgaattcctaagtcttcgccttcactga 70247
human.FAM230E-LOC105377182-GGT3P.NCBI.                                                 ------------------------------------------------------------ 57310
human.FAM230J-LOC105372942-GGTLC5P.NCBI.rev.compl                                      ------------------------------------------------------------ 46364
human.FAM230A.ncRNA.18487127.18500594-GGTLC3.18516335.18518165.rev.compl.REF           ------------------------------------------------------------ 31039

chimp.LOC112206744-LOC107973052-GGT2.revcompl                                          ------------------------------------------------------------ 61762
chimp54546-135457.revcompl.Pan.troglodytes.clone.rp43-41g5.GenBank:AC099533.36        ------------------------------------------------------------ 68417
human.FAM30B-LOC105372935-GGT2.NCBI.                                                   cttcccacagcatgggcagtagctgtgagaccttgggctggttacttaacctctctgcac 70307
human.FAM230E-LOC105377182-GGT3P.NCBI.                                                 ------------------------------------------------------------ 57310
human.FAM230J-LOC105372942-GGTLC5P.NCBI.rev.compl                                      ------------------------------------------------------------ 46364
human.FAM230A.ncRNA.18487127.18500594-GGTLC3.18516335.18518165.rev.compl.REF           ------------------------------------------------------------ 31039

chimp.LOC112206744-LOC107973052-GGT2.revcompl                                          ------------------------------------------------------------ 61762
chimp54546-135457.revcompl.Pan.troglodytes.clone.rp43-41g5.GenBank:AC099533.36        ------------------------------------------------------------ 68417
human.FAM30B-LOC105372935-GGT2.NCBI.                                                   ctcagtttcctcatctgtaaagtggtataatcgtcacagtgttatggaaggatttaatga 70367
human.FAM230E-LOC105377182-GGT3P.NCBI.                                                 ------------------------------------------------------------ 57310
human.FAM230J-LOC105372942-GGTLC5P.NCBI.rev.compl                                      ------------------------------------------------------------ 46364
human.FAM230A.ncRNA.18487127.18500594-GGTLC3.18516335.18518165.rev.compl.REF           ------------------------------------------------------------ 31039

chimp.LOC112206744-LOC107973052-GGT2.revcompl                                          ------------------------------------------------------------ 61762
chimp54546-135457.revcompl.Pan.troglodytes.clone.rp43-41g5.GenBank:AC099533.36        ------------------------------------------------------------ 68417
human.FAM30B-LOC105372935-GGT2.NCBI.                                                   gttgctacatttaaaacttaattcctggcacacaataagtgctcaataaatgtttagccg 70427
human.FAM230E-LOC105377182-GGT3P.NCBI.                                                 ------------------------------------------------------------ 57310
human.FAM230J-LOC105372942-GGTLC5P.NCBI.rev.compl                                      ------------------------------------------------------------ 46364
human.FAM230A.ncRNA.18487127.18500594-GGTLC3.18516335.18518165.rev.compl.REF           ------------------------------------------------------------ 31039

chimp.LOC112206744-LOC107973052-GGT2.revcompl                                          ------------------------------------------------------------ 61762
chimp54546-135457.revcompl.Pan.troglodytes.clone.rp43-41g5.GenBank:AC099533.36        ------------------------------------------------------------ 68417
human.FAM30B-LOC105372935-GGT2.NCBI.                                                   ctagttgcagtagtagcaatagtagtgatattaactctagctgtggaactaatttctccc 70487
human.FAM230E-LOC105377182-GGT3P.NCBI.                                                 ------------------------------------------------------------ 57310
human.FAM230J-LOC105372942-GGTLC5P.NCBI.rev.compl                                      ------------------------------------------------------------ 46364
human.FAM230A.ncRNA.18487127.18500594-GGTLC3.18516335.18518165.rev.compl.REF           ------------------------------------------------------------ 31039

chimp.LOC112206744-LOC107973052-GGT2.revcompl                                          ------------------------------------------------------------ 61762
chimp54546-135457.revcompl.Pan.troglodytes.clone.rp43-41g5.GenBank:AC099533.36        ------------------------------------------------------------ 68417
human.FAM30B-LOC105372935-GGT2.NCBI.                                                   acacaccgtggcaggttgtattctccagacacagcagtaacaatatctccttcccacacg 70547
human.FAM230E-LOC105377182-GGT3P.NCBI.                                                 ------------------------------------------------------------ 57310
human.FAM230J-LOC105372942-GGTLC5P.NCBI.rev.compl                                      ------------------------------------------------------------ 46364
human.FAM230A.ncRNA.18487127.18500594-GGTLC3.18516335.18518165.rev.compl.REF           ------------------------------------------------------------ 31039

chimp.LOC112206744-LOC107973052-GGT2.revcompl                                          ------------------------------------------------------------ 61762
chimp54546-135457.revcompl.Pan.troglodytes.clone.rp43-41g5.GenBank:AC099533.36        ------------------------------------------------------------ 68417
human.FAM30B-LOC105372935-GGT2.NCBI.                                                   ctcttttgcaatgtgaccatgccacacactcccatcaagaggtggagtttagaataattc 70607
human.FAM230E-LOC105377182-GGT3P.NCBI.                                                 ------------------------------------------------------------ 57310
human.FAM230J-LOC105372942-GGTLC5P.NCBI.rev.compl                                      ------------------------------------------------------------ 46364
human.FAM230A.ncRNA.18487127.18500594-GGTLC3.18516335.18518165.rev.compl.REF           ------------------------------------------------------------ 31039

chimp.LOC112206744-LOC107973052-GGT2.revcompl                                          ------------------------------------------------------------ 61762
chimp54546-135457.revcompl.Pan.troglodytes.clone.rp43-41g5.GenBank:AC099533.36        ------------------------------------------------------------ 68417
human.FAM30B-LOC105372935-GGT2.NCBI.                                                   cctcctctgtggatctgggcaggaagtcactgtgtgacttccagtcctaggtcatgacaa 70667
human.FAM230E-LOC105377182-GGT3P.NCBI.                                                 ------------------------------------------------------------ 57310
human.FAM230J-LOC105372942-GGTLC5P.NCBI.rev.compl                                      ------------------------------------------------------------ 46364
human.FAM230A.ncRNA.18487127.18500594-GGTLC3.18516335.18518165.rev.compl.REF           ------------------------------------------------------------ 31039

chimp.LOC112206744-LOC107973052-GGT2.revcompl                                          ------------------------------------------------------------ 61762
chimp54546-135457.revcompl.Pan.troglodytes.clone.rp43-41g5.GenBank:AC099533.36        ------------------------------------------------------------ 68417
human.FAM30B-LOC105372935-GGT2.NCBI.                                                   gtgatgtagcttccatttcactcagggtgccctgaaccaccacataagaaagccaacggt 70727
human.FAM230E-LOC105377182-GGT3P.NCBI.                                                 ------------------------------------------------------------ 57310
human.FAM230J-LOC105372942-GGTLC5P.NCBI.rev.compl                                      ------------------------------------------------------------ 46364
human.FAM230A.ncRNA.18487127.18500594-GGTLC3.18516335.18518165.rev.compl.REF           ------------------------------------------------------------ 31039

chimp.LOC112206744-LOC107973052-GGT2.revcompl                                          ------------------------------------------------------------ 61762
chimp54546-135457.revcompl.Pan.troglodytes.clone.rp43-41g5.GenBank:AC099533.36        ------------------------------------------------------------ 68417
human.FAM30B-LOC105372935-GGT2.NCBI.                                                   cctgaggccatgctgtgagggggcccaaggcacatggagaggcccagggcaggtgctcca 70787
human.FAM230E-LOC105377182-GGT3P.NCBI.                                                 ------------------------------------------------------------ 57310
human.FAM230J-LOC105372942-GGTLC5P.NCBI.rev.compl                                      ------------------------------------------------------------ 46364
human.FAM230A.ncRNA.18487127.18500594-GGTLC3.18516335.18518165.rev.compl.REF           ------------------------------------------------------------ 31039

chimp.LOC112206744-LOC107973052-GGT2.revcompl                                          ------------------------------------------------------------ 61762
chimp54546-135457.revcompl.Pan.troglodytes.clone.rp43-41g5.GenBank:AC099533.36        ------------------------------------------------------------ 68417
human.FAM30B-LOC105372935-GGT2.NCBI.                                                   gtccagagctggctccagccaggtcccctgccaccatgtgagtgaatgagccaatcacc 70847
human.FAM230E-LOC105377182-GGT3P.NCBI.                                                 ------------------------------------------------------------ 57310
human.FAM230J-LOC105372942-GGTLC5P.NCBI.rev.compl                                      ------------------------------------------------------------ 46364
human.FAM230A.ncRNA.18487127.18500594-GGTLC3.18516335.18518165.rev.compl.REF           ------------------------------------------------------------ 31039

chimp.LOC112206744-LOC107973052-GGT2.revcompl                                          ------------------------------------------------------------ 61762
chimp54546-135457.revcompl.Pan.troglodytes.clone.rp43-41g5.GenBank:AC099533.36        ------------------------------------------------------------ 68417
human.FAM30B-LOC105372935-GGT2.NCBI.                                                   acccagctccacctgccaccatgtgagtgaacaagcctcccctgtgagctgcccccagcc 70907
human.FAM230E-LOC105377182-GGT3P.NCBI.                                                 ------------------------------------------------------------ 57310
human.FAM230J-LOC105372942-GGTLC5P.NCBI.rev.compl                                      ------------------------------------------------------------ 46364
human.FAM230A.ncRNA.18487127.18500594-GGTLC3.18516335.18518165.rev.compl.REF           ------------------------------------------------------------ 31039
```

```
chimp.LOC112206744-LOC107973052-GGT2.revcompl                                     ------------------------------------------------------------ 61762
chimp54546-135457.revcompl.Pan.troglodytes.clone.rp43-41g5.GenBank:AC099533.36     ------------------------------------------------------------ 68417
human.FAM30B-LOC105372935-GGT2.NCBI.                                              ttggaacttccagccatgcctgccgtgcctgtaaattctcaacccccagattctgtgagc 70967
human.FAM230E-LOC105377182-GGT3P.NCBI.                                            ------------------------------------------------------------ 57310
human.FAM230J-LOC105372942-GGTLC5P.NCBI.rev.compl                                 ------------------------------------------------------------ 46364
human.FAM230A.ncRNA.18487127.18500594-GGTLC3.18516335.18518165.rev.compl.REF      ------------------------------------------------------------ 31039

chimp.LOC112206744-LOC107973052-GGT2.revcompl                                     ------------------------------------------------------------ 61762
chimp54546-135457.revcompl.Pan.troglodytes.clone.rp43-41g5.GenBank:AC099533.36     ------------------------------------------------------------ 68417
human.FAM30B-LOC105372935-GGT2.NCBI.                                              ataacaaaattgttgttttaagccactgaggttgggaaacatttgttacatagaaaggta 71027
human.FAM230E-LOC105377182-GGT3P.NCBI.                                            ------------------------------------------------------------ 57310
human.FAM230J-LOC105372942-GGTLC5P.NCBI.rev.compl                                 ------------------------------------------------------------ 46364
human.FAM230A.ncRNA.18487127.18500594-GGTLC3.18516335.18518165.rev.compl.REF      ------------------------------------------------------------ 31039

chimp.LOC112206744-LOC107973052-GGT2.revcompl                                     ------------------------------------------------------------ 61762
chimp54546-135457.revcompl.Pan.troglodytes.clone.rp43-41g5.GenBank:AC099533.36     ------------------------------------------------------------ 68417
human.FAM30B-LOC105372935-GGT2.NCBI.                                              acaagaacatatgctatggcttcaatcaacatttttttcagtacctattatgatggacac 71087
human.FAM230E-LOC105377182-GGT3P.NCBI.                                            ------------------------------------------------------------ 57310
human.FAM230J-LOC105372942-GGTLC5P.NCBI.rev.compl                                 ------------------------------------------------------------ 46364
human.FAM230A.ncRNA.18487127.18500594-GGTLC3.18516335.18518165.rev.compl.REF      ------------------------------------------------------------ 31039

chimp.LOC112206744-LOC107973052-GGT2.revcompl                                     ------------------------------------------------------------ 61762
chimp54546-135457.revcompl.Pan.troglodytes.clone.rp43-41g5.GenBank:AC099533.36     ------------------------------------------------------------ 68417
human.FAM30B-LOC105372935-GGT2.NCBI.                                              taggacagaaactgtgagggagagattaataagacatagttgacaagttgtcctaagctg 71147
human.FAM230E-LOC105377182-GGT3P.NCBI.                                            ------------------------------------------------------------ 57310
human.FAM230J-LOC105372942-GGTLC5P.NCBI.rev.compl                                 ------------------------------------------------------------ 46364
human.FAM230A.ncRNA.18487127.18500594-GGTLC3.18516335.18518165.rev.compl.REF      ------------------------------------------------------------ 31039

chimp.LOC112206744-LOC107973052-GGT2.revcompl                                     ------------------------------------------------------------ 61762
chimp54546-135457.revcompl.Pan.troglodytes.clone.rp43-41g5.GenBank:AC099533.36     ------------------------------------------------------------ 68417
human.FAM30B-LOC105372935-GGT2.NCBI.                                              acaatcctaaaataaatacacataactaatcattttaatggtatacgatagcagtgaggt 71207
human.FAM230E-LOC105377182-GGT3P.NCBI.                                            ------------------------------------------------------------ 57310
human.FAM230J-LOC105372942-GGTLC5P.NCBI.rev.compl                                 ------------------------------------------------------------ 46364
human.FAM230A.ncRNA.18487127.18500594-GGTLC3.18516335.18518165.rev.compl.REF      ------------------------------------------------------------ 31039

chimp.LOC112206744-LOC107973052-GGT2.revcompl                                     ------------------------------------------------------------ 61762
chimp54546-135457.revcompl.Pan.troglodytes.clone.rp43-41g5.GenBank:AC099533.36     ------------------------------------------------------------ 68417
human.FAM30B-LOC105372935-GGT2.NCBI.                                              gcaaagtgcaaggttaagccctttcagaaagtgaaagcattcaaaaagccttcaaaaaag 71267
human.FAM230E-LOC105377182-GGT3P.NCBI.                                            ------------------------------------------------------------ 57310
human.FAM230J-LOC105372942-GGTLC5P.NCBI.rev.compl                                 ------------------------------------------------------------ 46364
human.FAM230A.ncRNA.18487127.18500594-GGTLC3.18516335.18518165.rev.compl.REF      ------------------------------------------------------------ 31039

chimp.LOC112206744-LOC107973052-GGT2.revcompl                                     ------------------------------------------------------------ 61762
chimp54546-135457.revcompl.Pan.troglodytes.clone.rp43-41g5.GenBank:AC099533.36     ------------------------------------------------------------ 68417
human.FAM30B-LOC105372935-GGT2.NCBI.                                              tagtgtcatctgaattggatcccaaagcgcaagggaaatatataccacatgaaaacaccg 71327
human.FAM230E-LOC105377182-GGT3P.NCBI.                                            ------------------------------------------------------------ 57310
human.FAM230J-LOC105372942-GGTLC5P.NCBI.rev.compl                                 ------------------------------------------------------------ 46364
human.FAM230A.ncRNA.18487127.18500594-GGTLC3.18516335.18518165.rev.compl.REF      ------------------------------------------------------------ 31039

chimp.LOC112206744-LOC107973052-GGT2.revcompl                                     ------------------------------------------------------------ 61762
chimp54546-135457.revcompl.Pan.troglodytes.clone.rp43-41g5.GenBank:AC099533.36     ------------------------------------------------------------ 68417
human.FAM30B-LOC105372935-GGT2.NCBI.                                              cagtgcattccaggattagcaagaagtttgcggaggtcgttttgtcacatgactggaggg 71387
human.FAM230E-LOC105377182-GGT3P.NCBI.                                            ------------------------------------------------------------ 57310
human.FAM230J-LOC105372942-GGTLC5P.NCBI.rev.compl                                 ------------------------------------------------------------ 46364
human.FAM230A.ncRNA.18487127.18500594-GGTLC3.18516335.18518165.rev.compl.REF      ------------------------------------------------------------ 31039

chimp.LOC112206744-LOC107973052-GGT2.revcompl                                     ------------------------------------------------------------ 61762
chimp54546-135457.revcompl.Pan.troglodytes.clone.rp43-41g5.GenBank:AC099533.36     ------------------------------------------------------------ 68417
human.FAM30B-LOC105372935-GGT2.NCBI.                                              cacatcagtgccaggcggaaaagggcaccgaaaggcacattaataaagtcaaacacctcc 71447
human.FAM230E-LOC105377182-GGT3P.NCBI.                                            ------------------------------------------------------------ 57310
human.FAM230J-LOC105372942-GGTLC5P.NCBI.rev.compl                                 ------------------------------------------------------------ 46364
human.FAM230A.ncRNA.18487127.18500594-GGTLC3.18516335.18518165.rev.compl.REF      ------------------------------------------------------------ 31039

chimp.LOC112206744-LOC107973052-GGT2.revcompl                                     ------------------------------------------------------------ 61762
chimp54546-135457.revcompl.Pan.troglodytes.clone.rp43-41g5.GenBank:AC099533.36     ------------------------------------------------------------ 68417
human.FAM30B-LOC105372935-GGT2.NCBI.                                              tctgggacaggggaagttgagaaagagcctaacagaggtaaggaagaagacggatcagat 71507
human.FAM230E-LOC105377182-GGT3P.NCBI.                                            ------------------------------------------------------------ 57310
human.FAM230J-LOC105372942-GGTLC5P.NCBI.rev.compl                                 ------------------------------------------------------------ 46364
human.FAM230A.ncRNA.18487127.18500594-GGTLC3.18516335.18518165.rev.compl.REF      ------------------------------------------------------------ 31039

chimp.LOC112206744-LOC107973052-GGT2.revcompl                                     ------------------------------------------------------------ 61762
chimp54546-135457.revcompl.Pan.troglodytes.clone.rp43-41g5.GenBank:AC099533.36     ------------------------------------------------------------ 68417
human.FAM30B-LOC105372935-GGT2.NCBI.                                              ttacactttcgaattatcactctggtggcaggtgaaggacagagcagagggcaggactgg 71567
human.FAM230E-LOC105377182-GGT3P.NCBI.                                            ------------------------------------------------------------ 57310
human.FAM230J-LOC105372942-GGTLC5P.NCBI.rev.compl                                 ------------------------------------------------------------ 46364
human.FAM230A.ncRNA.18487127.18500594-GGTLC3.18516335.18518165.rev.compl.REF      ------------------------------------------------------------ 31039

chimp.LOC112206744-LOC107973052-GGT2.revcompl                                     ------------------------------------------------------------ 61762
chimp54546-135457.revcompl.Pan.troglodytes.clone.rp43-41g5.GenBank:AC099533.36     ------------------------------------------------------------ 68417
human.FAM30B-LOC105372935-GGT2.NCBI.                                              aagcagacagaccaaattcacagtcaaatgcgacaagatgagggcctggcctggttttaa 71627
human.FAM230E-LOC105377182-GGT3P.NCBI.                                            ------------------------------------------------------------ 57310
human.FAM230J-LOC105372942-GGTLC5P.NCBI.rev.compl                                 ------------------------------------------------------------ 46364
human.FAM230A.ncRNA.18487127.18500594-GGTLC3.18516335.18518165.rev.compl.REF      ------------------------------------------------------------ 31039

chimp.LOC112206744-LOC107973052-GGT2.revcompl                                     ------------------------------------------------------------ 61762
chimp54546-135457.revcompl.Pan.troglodytes.clone.rp43-41g5.GenBank:AC099533.36     ------------------------------------------------------------ 68417
human.FAM30B-LOC105372935-GGT2.NCBI.                                              atccaaggacaaaaatatcattcattcactctttcattctttctcttatctgaatcccca 71687
human.FAM230E-LOC105377182-GGT3P.NCBI.                                            ------------------------------------------------------------ 57310
human.FAM230J-LOC105372942-GGTLC5P.NCBI.rev.compl                                 ------------------------------------------------------------ 46364
human.FAM230A.ncRNA.18487127.18500594-GGTLC3.18516335.18518165.rev.compl.REF      ------------------------------------------------------------ 31039

chimp.LOC112206744-LOC107973052-GGT2.revcompl                                     ------------------------------------------------------------ 61762
chimp54546-135457.revcompl.Pan.troglodytes.clone.rp43-41g5.GenBank:AC099533.36     ------------------------------------------------------------ 68417
human.FAM30B-LOC105372935-GGT2.NCBI.                                              gtggtacctaaaatagagctggtctaaagatgatgattaatgaatgttaaattattgata 71747
human.FAM230E-LOC105377182-GGT3P.NCBI.                                            ------------------------------------------------------------ 57310
human.FAM230J-LOC105372942-GGTLC5P.NCBI.rev.compl                                 ------------------------------------------------------------ 46364
human.FAM230A.ncRNA.18487127.18500594-GGTLC3.18516335.18518165.rev.compl.REF      ------------------------------------------------------------ 31039

chimp.LOC112206744-LOC107973052-GGT2.revcompl                                     ------------------------------------------------------------ 61762
chimp54546-135457.revcompl.Pan.troglodytes.clone.rp43-41g5.GenBank:AC099533.36     ------------------------------------------------------------ 68417
human.FAM30B-LOC105372935-GGT2.NCBI.                                              ctacttctaagaagatgtaactgccagctacagaaccagccaaaaggactgcaaacccag 71807
human.FAM230E-LOC105377182-GGT3P.NCBI.                                            ------------------------------------------------------------ 57310
human.FAM230J-LOC105372942-GGTLC5P.NCBI.rev.compl                                 ------------------------------------------------------------ 46364
human.FAM230A.ncRNA.18487127.18500594-GGTLC3.18516335.18518165.rev.compl.REF      ------------------------------------------------------------ 31039

chimp.LOC112206744-LOC107973052-GGT2.revcompl                                     ------------------------------------------------------------ 61762
chimp54546-135457.revcompl.Pan.troglodytes.clone.rp43-41g5.GenBank:AC099533.36     ------------------------------------------------------------ 68417
human.FAM30B-LOC105372935-GGT2.NCBI.                                              taattcatttcatcccaacctgactgatctctctgatgtctgcactgctgtgtcactaga 71867
human.FAM230E-LOC105377182-GGT3P.NCBI.                                            ------------------------------------------------------------ 57310
human.FAM230J-LOC105372942-GGTLC5P.NCBI.rev.compl                                 ------------------------------------------------------------ 46364
human.FAM230A.ncRNA.18487127.18500594-GGTLC3.18516335.18518165.rev.compl.REF      ------------------------------------------------------------ 31039

chimp.LOC112206744-LOC107973052-GGT2.revcompl                                     ------------------------------------------------------------ 61762
chimp54546-135457.revcompl.Pan.troglodytes.clone.rp43-41g5.GenBank:AC099533.36     ------------------------------------------------------------ 68417
human.FAM30B-LOC105372935-GGT2.NCBI.                                              ctgcccgattgtcctaggttgagacatttacctactgagccccagggatgggtggctgct 71927
human.FAM230E-LOC105377182-GGT3P.NCBI.                                            ------------------------------------------------------------ 57310
human.FAM230J-LOC105372942-GGTLC5P.NCBI.rev.compl                                 ------------------------------------------------------------ 46364
human.FAM230A.ncRNA.18487127.18500594-GGTLC3.18516335.18518165.rev.compl.REF      ------------------------------------------------------------ 31039

chimp.LOC112206744-LOC107973052-GGT2.revcompl                                     ------------------------------------------------------------ 61762
chimp54546-135457.revcompl.Pan.troglodytes.clone.rp43-41g5.GenBank:AC099533.36     ------------------------------------------------------------ 68417
human.FAM30B-LOC105372935-GGT2.NCBI.                                              tctaagctgctgcctaattccctcattactaaatgttgacgctgcttccttagaggactg 71987
human.FAM230E-LOC105377182-GGT3P.NCBI.                                            ------------------------------------------------------------ 57310
human.FAM230J-LOC105372942-GGTLC5P.NCBI.rev.compl                                 ------------------------------------------------------------ 46364
human.FAM230A.ncRNA.18487127.18500594-GGTLC3.18516335.18518165.rev.compl.REF      ------------------------------------------------------------ 31039
```

```
chimp.LOC112206744-LOC107973052-GGT2.revcompl                                      ------------------------------------------------------------ 61762
chimp54546-135457.revcompl.Pan.troglodytes.clone.rp43-41g5.GenBank:AC099533.36     ------------------------------------------------------------ 68417
human.FAM30B-LOC105372935-GGT2.NCBI.                                               ctaaaccataactcaaaaatgttttaaacatgctactatttacttagattagaatattta 72047
human.FAM230E-LOC105377182-GGT3P.NCBI.                                             ------------------------------------------------------------ 57310
human.FAM230J-LOC105372942-GGTLC5P.NCBI.rev.compl                                  ------------------------------------------------------------ 46364
human.FAM230A.ncRNA.18487127.18500594-GGTLC3.18516335.18518165.rev.compl.REF       ------------------------------------------------------------ 31039

chimp.LOC112206744-LOC107973052-GGT2.revcompl                                      ------------------------------------------------------------ 61762
chimp54546-135457.revcompl.Pan.troglodytes.clone.rp43-41g5.GenBank:AC099533.36     ------------------------------------------------------------ 68417
human.FAM30B-LOC105372935-GGT2.NCBI.                                               cttaaatgttttaaaaaatacagtattttaagtcttttatctcttctgactactaccact 72107
human.FAM230E-LOC105377182-GGT3P.NCBI.                                             ------------------------------------------------------------ 57310
human.FAM230J-LOC105372942-GGTLC5P.NCBI.rev.compl                                  ------------------------------------------------------------ 46364
human.FAM230A.ncRNA.18487127.18500594-GGTLC3.18516335.18518165.rev.compl.REF       ------------------------------------------------------------ 31039

chimp.LOC112206744-LOC107973052-GGT2.revcompl                                      ------------------------------------------------------------ 61762
chimp54546-135457.revcompl.Pan.troglodytes.clone.rp43-41g5.GenBank:AC099533.36     ------------------------------------------------------------ 68417
human.FAM30B-LOC105372935-GGT2.NCBI.                                               aaccctgcttcccctcatttttccagagattagcactactgtgatttaaggatagagctc 72167
human.FAM230E-LOC105377182-GGT3P.NCBI.                                             ------------------------------------------------------------ 57310
human.FAM230J-LOC105372942-GGTLC5P.NCBI.rev.compl                                  ------------------------------------------------------------ 46364
human.FAM230A.ncRNA.18487127.18500594-GGTLC3.18516335.18518165.rev.compl.REF       ------------------------------------------------------------ 31039

chimp.LOC112206744-LOC107973052-GGT2.revcompl                                      ------------------------------------------------------------ 61762
chimp54546-135457.revcompl.Pan.troglodytes.clone.rp43-41g5.GenBank:AC099533.36     ------------------------------------------------------------ 68417
human.FAM30B-LOC105372935-GGT2.NCBI.                                               tccagacttaaaaaaaaaccaaaaaactgcttttatatccagatatatgtaaccactgaa 72227
human.FAM230E-LOC105377182-GGT3P.NCBI.                                             ------------------------------------------------------------ 57310
human.FAM230J-LOC105372942-GGTLC5P.NCBI.rev.compl                                  ------------------------------------------------------------ 46364
human.FAM230A.ncRNA.18487127.18500594-GGTLC3.18516335.18518165.rev.compl.REF       ------------------------------------------------------------ 31039

chimp.LOC112206744-LOC107973052-GGT2.revcompl                                      ------------------------------------------------------------ 61762
chimp54546-135457.revcompl.Pan.troglodytes.clone.rp43-41g5.GenBank:AC099533.36     ------------------------------------------------------------ 68417
human.FAM30B-LOC105372935-GGT2.NCBI.                                               aaatatacattatttttcagcatttttacataaacattatcatccctttcattgagtta 72287
human.FAM230E-LOC105377182-GGT3P.NCBI.                                             ------------------------------------------------------------ 57310
human.FAM230J-LOC105372942-GGTLC5P.NCBI.rev.compl                                  ------------------------------------------------------------ 46364
human.FAM230A.ncRNA.18487127.18500594-GGTLC3.18516335.18518165.rev.compl.REF       ------------------------------------------------------------ 31039

chimp.LOC112206744-LOC107973052-GGT2.revcompl                                      ------------------------------------------------------------ 61762
chimp54546-135457.revcompl.Pan.troglodytes.clone.rp43-41g5.GenBank:AC099533.36     ------------------------------------------------------------ 68417
human.FAM30B-LOC105372935-GGT2.NCBI.                                               tgttcctcagttttcctcctcaaagtttttgagatcaaatatagttcccatttttcttct 72347
human.FAM230E-LOC105377182-GGT3P.NCBI.                                             ------------------------------------------------------------ 57310
human.FAM230J-LOC105372942-GGTLC5P.NCBI.rev.compl                                  ------------------------------------------------------------ 46364
human.FAM230A.ncRNA.18487127.18500594-GGTLC3.18516335.18518165.rev.compl.REF       ------------------------------------------------------------ 31039

chimp.LOC112206744-LOC107973052-GGT2.revcompl                                      ------------------------------------------------------------ 61762
chimp54546-135457.revcompl.Pan.troglodytes.clone.rp43-41g5.GenBank:AC099533.36     ------------------------------------------------------------ 68417
human.FAM30B-LOC105372935-GGT2.NCBI.                                               ttatatagctgcacagtattccatcataggattacaccacatttatttattccaatatt 72407
human.FAM230E-LOC105377182-GGT3P.NCBI.                                             ------------------------------------------------------------ 57310
human.FAM230J-LOC105372942-GGTLC5P.NCBI.rev.compl                                  ------------------------------------------------------------ 46364
human.FAM230A.ncRNA.18487127.18500594-GGTLC3.18516335.18518165.rev.compl.REF       ------------------------------------------------------------ 31039

chimp.LOC112206744-LOC107973052-GGT2.revcompl                                      ------------------------------------------------------------ 61762
chimp54546-135457.revcompl.Pan.troglodytes.clone.rp43-41g5.GenBank:AC099533.36     ------------------------------------------------------------ 68417
human.FAM30B-LOC105372935-GGT2.NCBI.                                               aatgaatatttaggttatttatactttttttctataaccaacaagcccatgccttctac 72467
human.FAM230E-LOC105377182-GGT3P.NCBI.                                             ------------------------------------------------------------ 57310
human.FAM230J-LOC105372942-GGTLC5P.NCBI.rev.compl                                  ------------------------------------------------------------ 46364
human.FAM230A.ncRNA.18487127.18500594-GGTLC3.18516335.18518165.rev.compl.REF       ------------------------------------------------------------ 31039

chimp.LOC112206744-LOC107973052-GGT2.revcompl                                      ------------------------------------------------------------ 61762
chimp54546-135457.revcompl.Pan.troglodytes.clone.rp43-41g5.GenBank:AC099533.36     ------------------------------------------------------------ 68417
human.FAM30B-LOC105372935-GGT2.NCBI.                                               tgcttatcctttccccacctgaatttgtacaatccccagcttctcttatccatgaccact 72527
human.FAM230E-LOC105377182-GGT3P.NCBI.                                             ------------------------------------------------------------ 57310
human.FAM230J-LOC105372942-GGTLC5P.NCBI.rev.compl                                  ------------------------------------------------------------ 46364
human.FAM230A.ncRNA.18487127.18500594-GGTLC3.18516335.18518165.rev.compl.REF       ------------------------------------------------------------ 31039

chimp.LOC112206744-LOC107973052-GGT2.revcompl                                      ------------------------------------------------------------ 61762
chimp54546-135457.revcompl.Pan.troglodytes.clone.rp43-41g5.GenBank:AC099533.36     ------------------------------------------------------------ 68417
human.FAM30B-LOC105372935-GGT2.NCBI.                                               gaaaccccagggaagaccttatcactttcaggatgtccatggctctttcaccctatgcca 72587
human.FAM230E-LOC105377182-GGT3P.NCBI.                                             ------------------------------------------------------------ 57310
human.FAM230J-LOC105372942-GGTLC5P.NCBI.rev.compl                                  ------------------------------------------------------------ 46364
human.FAM230A.ncRNA.18487127.18500594-GGTLC3.18516335.18518165.rev.compl.REF       ------------------------------------------------------------ 31039

chimp.LOC112206744-LOC107973052-GGT2.revcompl                                      ------------------------------------------------------------ 61762
chimp54546-135457.revcompl.Pan.troglodytes.clone.rp43-41g5.GenBank:AC099533.36     ------------------------------------------------------------ 68417
human.FAM30B-LOC105372935-GGT2.NCBI.                                               tgcttggaaccaggaaagcccaaaagaccccacttcctgcccttctattctgctcctcct 72647
human.FAM230E-LOC105377182-GGT3P.NCBI.                                             ------------------------------------------------------------ 57310
human.FAM230J-LOC105372942-GGTLC5P.NCBI.rev.compl                                  ------------------------------------------------------------ 46364
human.FAM230A.ncRNA.18487127.18500594-GGTLC3.18516335.18518165.rev.compl.REF       ------------------------------------------------------------ 31039

chimp.LOC112206744-LOC107973052-GGT2.revcompl                                      ------------------------------------------------------------ 61762
chimp54546-135457.revcompl.Pan.troglodytes.clone.rp43-41g5.GenBank:AC099533.36     ------------------------------------------------------------ 68417
human.FAM30B-LOC105372935-GGT2.NCBI.                                               ctcctatcgctcatttctttgcctctatcctccactcctcccagcaaaggtagggatagc 72707
human.FAM230E-LOC105377182-GGT3P.NCBI.                                             ------------------------------------------------------------ 57310
human.FAM230J-LOC105372942-GGTLC5P.NCBI.rev.compl                                  ------------------------------------------------------------ 46364
human.FAM230A.ncRNA.18487127.18500594-GGTLC3.18516335.18518165.rev.compl.REF       ------------------------------------------------------------ 31039

chimp.LOC112206744-LOC107973052-GGT2.revcompl                                      ------------------------------------------------------------ 61762
chimp54546-135457.revcompl.Pan.troglodytes.clone.rp43-41g5.GenBank:AC099533.36     ------------------------------------------------------------ 68417
human.FAM30B-LOC105372935-GGT2.NCBI.                                               acctgagtcctgccaccctctcacctgcattccccctcactctcatgcgaaggacagcac 72767
human.FAM230E-LOC105377182-GGT3P.NCBI.                                             ------------------------------------------------------------ 57310
human.FAM230J-LOC105372942-GGTLC5P.NCBI.rev.compl                                  ------------------------------------------------------------ 46364
human.FAM230A.ncRNA.18487127.18500594-GGTLC3.18516335.18518165.rev.compl.REF       ------------------------------------------------------------ 31039

chimp.LOC112206744-LOC107973052-GGT2.revcompl                                      ------------------------------------------------------------ 61762
chimp54546-135457.revcompl.Pan.troglodytes.clone.rp43-41g5.GenBank:AC099533.36     ------------------------------------------------------------ 68417
human.FAM30B-LOC105372935-GGT2.NCBI.                                               agaagcagcatcaaaaaagaaaggcagcgagggagccctgcagcctgccctaggccagcc 72827
human.FAM230E-LOC105377182-GGT3P.NCBI.                                             ------------------------------------------------------------ 57310
human.FAM230J-LOC105372942-GGTLC5P.NCBI.rev.compl                                  ------------------------------------------------------------ 46364
human.FAM230A.ncRNA.18487127.18500594-GGTLC3.18516335.18518165.rev.compl.REF       ------------------------------------------------------------ 31039

chimp.LOC112206744-LOC107973052-GGT2.revcompl                                      ------------------------------------------------------------ 61762
chimp54546-135457.revcompl.Pan.troglodytes.clone.rp43-41g5.GenBank:AC099533.36     ------------------------------------------------------------ 68417
human.FAM30B-LOC105372935-GGT2.NCBI.                                               atgcccacagccacaggaatagacactgttttcagccccacctagattcaaggcagcatc 72887
human.FAM230E-LOC105377182-GGT3P.NCBI.                                             ------------------------------------------------------------ 57310
human.FAM230J-LOC105372942-GGTLC5P.NCBI.rev.compl                                  ------------------------------------------------------------ 46364
human.FAM230A.ncRNA.18487127.18500594-GGTLC3.18516335.18518165.rev.compl.REF       ------------------------------------------------------------ 31039

chimp.LOC112206744-LOC107973052-GGT2.revcompl                                      ------------------------------------------------------------ 61762
chimp54546-135457.revcompl.Pan.troglodytes.clone.rp43-41g5.GenBank:AC099533.36     ------------------------------------------------------------ 68417
human.FAM30B-LOC105372935-GGT2.NCBI.                                               taatagaaatgcttataagtaaatataagtgccagtatttgccttctattttctagaggg 72947
human.FAM230E-LOC105377182-GGT3P.NCBI.                                             ------------------------------------------------------------ 57310
human.FAM230J-LOC105372942-GGTLC5P.NCBI.rev.compl                                  ------------------------------------------------------------ 46364
human.FAM230A.ncRNA.18487127.18500594-GGTLC3.18516335.18518165.rev.compl.REF       ------------------------------------------------------------ 31039

chimp.LOC112206744-LOC107973052-GGT2.revcompl                                      ------------------------------------------------------------ 61762
chimp54546-135457.revcompl.Pan.troglodytes.clone.rp43-41g5.GenBank:AC099533.36     ------------------------------------------------------------ 68417
human.FAM30B-LOC105372935-GGT2.NCBI.                                               gaaaaaaattaaaaggcaagatcagattacttgcctggaaatgttctccctgtaattact 73007
human.FAM230E-LOC105377182-GGT3P.NCBI.                                             ------------------------------------------------------------ 57310
human.FAM230J-LOC105372942-GGTLC5P.NCBI.rev.compl                                  ------------------------------------------------------------ 46364
human.FAM230A.ncRNA.18487127.18500594-GGTLC3.18516335.18518165.rev.compl.REF       ------------------------------------------------------------ 31039

chimp.LOC112206744-LOC107973052-GGT2.revcompl                                      ------------------------------------------------------------ 61762
chimp54546-135457.revcompl.Pan.troglodytes.clone.rp43-41g5.GenBank:AC099533.36     ------------------------------------------------------------ 68417
human.FAM30B-LOC105372935-GGT2.NCBI.                                               ctgaccgctctttctttccctccggttttggagataatggaaataggctgagtgttgggc 73067
human.FAM230E-LOC105377182-GGT3P.NCBI.                                             ------------------------------------------------------------ 57310
human.FAM230J-LOC105372942-GGTLC5P.NCBI.rev.compl                                  ------------------------------------------------------------ 46364
human.FAM230A.ncRNA.18487127.18500594-GGTLC3.18516335.18518165.rev.compl.REF       ------------------------------------------------------------ 31039

chimp.LOC112206744-LOC107973052-GGT2.revcompl                                      ------------------------------------------------------------ 61762
```

```
chimp54546-135457.revcompl.Pan.troglodytes.clone.rp43-41g5.GenBank:AC099533.36      ------------------------------------------------------------ 68417
human.FAM30B-LOC105372935-GGT2.NCBI.                                                 atttttttttttaaagagggcagcttcatggcaacactccctctgagaagtccagaggct 73127
human.FAM230E-LOC105377182-GGT3P.NCBI.                                               ------------------------------------------------------------ 57310
human.FAM230J-LOC105372942-GGTLC5P.NCBI.rev.compl                                    ------------------------------------------------------------ 46364
human.FAM230A.ncRNA.18487127.18500594-GGTLC3.18516335.18518165.rev.compl.REF         ------------------------------------------------------------ 31039


chimp.LOC112206744-LOC107973052-GGT2.revcompl                                        ------------------------------------------------------------ 61762
chimp54546-135457.revcompl.Pan.troglodytes.clone.rp43-41g5.GenBank:AC099533.36      ------------------------------------------------------------ 68417
human.FAM30B-LOC105372935-GGT2.NCBI.                                                 cagtgaaccgcagcttagagtgagttccagtaaaaggaccatcacttgaaccagtaagtg 73187
human.FAM230E-LOC105377182-GGT3P.NCBI.                                               ------------------------------------------------------------ 57310
human.FAM230J-LOC105372942-GGTLC5P.NCBI.rev.compl                                    ------------------------------------------------------------ 46364
human.FAM230A.ncRNA.18487127.18500594-GGTLC3.18516335.18518165.rev.compl.REF         ------------------------------------------------------------ 31039


chimp.LOC112206744-LOC107973052-GGT2.revcompl                                        ------------------------------------------------------------ 61762
chimp54546-135457.revcompl.Pan.troglodytes.clone.rp43-41g5.GenBank:AC099533.36      ------------------------------------------------------------ 68417
human.FAM30B-LOC105372935-GGT2.NCBI.                                                 cctcattccttctcacagcatacatttcacagcaattactgttgctattattttacgctt 73247
human.FAM230E-LOC105377182-GGT3P.NCBI.                                               ------------------------------------------------------------ 57310
human.FAM230J-LOC105372942-GGTLC5P.NCBI.rev.compl                                    ------------------------------------------------------------ 46364
human.FAM230A.ncRNA.18487127.18500594-GGTLC3.18516335.18518165.rev.compl.REF         ------------------------------------------------------------ 31039


chimp.LOC112206744-LOC107973052-GGT2.revcompl                                        ------------------------------------------------------------ 61762
chimp54546-135457.revcompl.Pan.troglodytes.clone.rp43-41g5.GenBank:AC099533.36      ------------------------------------------------------------ 68417
human.FAM30B-LOC105372935-GGT2.NCBI.                                                 catttctggacctaggatacctcagtttctccagactctcagtctggtccccaggttttc 73307
human.FAM230E-LOC105377182-GGT3P.NCBI.                                               ------------------------------------------------------------ 57310
human.FAM230J-LOC105372942-GGTLC5P.NCBI.rev.compl                                    ------------------------------------------------------------ 46364
human.FAM230A.ncRNA.18487127.18500594-GGTLC3.18516335.18518165.rev.compl.REF         ------------------------------------------------------------ 31039


chimp.LOC112206744-LOC107973052-GGT2.revcompl                                        ------------------------------------------------------------ 61762
chimp54546-135457.revcompl.Pan.troglodytes.clone.rp43-41g5.GenBank:AC099533.36      ------------------------------------------------------------ 68417
human.FAM30B-LOC105372935-GGT2.NCBI.                                                 agtctgggagaaaggtgtctgctggatttcaaacagtccctctactggccgccaccacca 73367
human.FAM230E-LOC105377182-GGT3P.NCBI.                                               ------------------------------------------------------------ 57310
human.FAM230J-LOC105372942-GGTLC5P.NCBI.rev.compl                                    ------------------------------------------------------------ 46364
human.FAM230A.ncRNA.18487127.18500594-GGTLC3.18516335.18518165.rev.compl.REF         ------------------------------------------------------------ 31039


chimp.LOC112206744-LOC107973052-GGT2.revcompl                                        ------------------------------------------------------------ 61762
chimp54546-135457.revcompl.Pan.troglodytes.clone.rp43-41g5.GenBank:AC099533.36      ------------------------------------------------------------ 68417
human.FAM30B-LOC105372935-GGT2.NCBI.                                                 ccctctcctaaagaggactggaataactcttccacagctcccgccacactcagcactgcc 73427
human.FAM230E-LOC105377182-GGT3P.NCBI.                                               ------------------------------------------------------------ 57310
human.FAM230J-LOC105372942-GGTLC5P.NCBI.rev.compl                                    ------------------------------------------------------------ 46364
human.FAM230A.ncRNA.18487127.18500594-GGTLC3.18516335.18518165.rev.compl.REF         ------------------------------------------------------------ 31039


chimp.LOC112206744-LOC107973052-GGT2.revcompl                                        ------------------------------------------------------------ 61762
chimp54546-135457.revcompl.Pan.troglodytes.clone.rp43-41g5.GenBank:AC099533.36      ------------------------------------------------------------ 68417
human.FAM30B-LOC105372935-GGT2.NCBI.                                                 tgcacctcctcctggagctggcagggccccagctgtgcacccctcaatgctctcacagct 73487
human.FAM230E-LOC105377182-GGT3P.NCBI.                                               ------------------------------------------------------------ 57310
human.FAM230J-LOC105372942-GGTLC5P.NCBI.rev.compl                                    ------------------------------------------------------------ 46364
human.FAM230A.ncRNA.18487127.18500594-GGTLC3.18516335.18518165.rev.compl.REF         ------------------------------------------------------------ 31039


chimp.LOC112206744-LOC107973052-GGT2.revcompl                                        ------------------------------------------------------------ 61762
chimp54546-135457.revcompl.Pan.troglodytes.clone.rp43-41g5.GenBank:AC099533.36      ------------------------------------------------------------ 68417
human.FAM30B-LOC105372935-GGT2.NCBI.                                                 tcaggaagaggactgctaagcccagggccctgctgacaacatgccgggagcgcatctgca 73547
human.FAM230E-LOC105377182-GGT3P.NCBI.                                               ------------------------------------------------------------ 57310
human.FAM230J-LOC105372942-GGTLC5P.NCBI.rev.compl                                    ------------------------------------------------------------ 46364
human.FAM230A.ncRNA.18487127.18500594-GGTLC3.18516335.18518165.rev.compl.REF         ------------------------------------------------------------ 31039


chimp.LOC112206744-LOC107973052-GGT2.revcompl                                        ------------------------------------------------------------ 61762
chimp54546-135457.revcompl.Pan.troglodytes.clone.rp43-41g5.GenBank:AC099533.36      ------------------------------------------------------------ 68417
human.FAM30B-LOC105372935-GGT2.NCBI.                                                 ggcagggtcagaatgtcagctaccaggcagcagagagatgcggggcgccaacagcaaaag 73607
human.FAM230E-LOC105377182-GGT3P.NCBI.                                               ------------------------------------------------------------ 57310
human.FAM230J-LOC105372942-GGTLC5P.NCBI.rev.compl                                    ------------------------------------------------------------ 46364
human.FAM230A.ncRNA.18487127.18500594-GGTLC3.18516335.18518165.rev.compl.REF         ------------------------------------------------------------ 31039


chimp.LOC112206744-LOC107973052-GGT2.revcompl                                        ------------------------------------------------------------ 61762
chimp54546-135457.revcompl.Pan.troglodytes.clone.rp43-41g5.GenBank:AC099533.36      ------------------------------------------------------------ 68417
human.FAM30B-LOC105372935-GGT2.NCBI.                                                 cacttgggggctttttcaagtggcatcagaactagacctggatacaaaggggtggccct 73667
human.FAM230E-LOC105377182-GGT3P.NCBI.                                               ------------------------------------------------------------ 57310
human.FAM230J-LOC105372942-GGTLC5P.NCBI.rev.compl                                    ------------------------------------------------------------ 46364
human.FAM230A.ncRNA.18487127.18500594-GGTLC3.18516335.18518165.rev.compl.REF         ------------------------------------------------------------ 31039


chimp.LOC112206744-LOC107973052-GGT2.revcompl                                        ------------------------------------------------------------ 61762
chimp54546-135457.revcompl.Pan.troglodytes.clone.rp43-41g5.GenBank:AC099533.36      ------------------------------------------------------------ 68417
human.FAM30B-LOC105372935-GGT2.NCBI.                                                 ggcttcaggaagcccatgataagtcagtctgctgtgtccttcaacacagtgtccccacct 73727
human.FAM230E-LOC105377182-GGT3P.NCBI.                                               ------------------------------------------------------------ 57310
human.FAM230J-LOC105372942-GGTLC5P.NCBI.rev.compl                                    ------------------------------------------------------------ 46364
human.FAM230A.ncRNA.18487127.18500594-GGTLC3.18516335.18518165.rev.compl.REF         ------------------------------------------------------------ 31039


chimp.LOC112206744-LOC107973052-GGT2.revcompl                                        ------------------------------------------------------------ 61762
chimp54546-135457.revcompl.Pan.troglodytes.clone.rp43-41g5.GenBank:AC099533.36      ------------------------------------------------------------ 68417
human.FAM30B-LOC105372935-GGT2.NCBI.                                                 tctgtctcccaacactaggccatggagacacaaaactagaaaatggggctgcctagctac 73787
human.FAM230E-LOC105377182-GGT3P.NCBI.                                               ------------------------------------------------------------ 57310
human.FAM230J-LOC105372942-GGTLC5P.NCBI.rev.compl                                    ------------------------------------------------------------ 46364
human.FAM230A.ncRNA.18487127.18500594-GGTLC3.18516335.18518165.rev.compl.REF         ------------------------------------------------------------ 31039


chimp.LOC112206744-LOC107973052-GGT2.revcompl                                        ------------------------------------------------------------ 61762
chimp54546-135457.revcompl.Pan.troglodytes.clone.rp43-41g5.GenBank:AC099533.36      ------------------------------------------------------------ 68417
human.FAM30B-LOC105372935-GGT2.NCBI.                                                 gaatgcccaggctgtgaattggggtctccaaaggaacctcagtgccgtgtcctactcaga 73847
human.FAM230E-LOC105377182-GGT3P.NCBI.                                               ------------------------------------------------------------ 57310
human.FAM230J-LOC105372942-GGTLC5P.NCBI.rev.compl                                    ------------------------------------------------------------ 46364
human.FAM230A.ncRNA.18487127.18500594-GGTLC3.18516335.18518165.rev.compl.REF         ------------------------------------------------------------ 31039


chimp.LOC112206744-LOC107973052-GGT2.revcompl                                        ------------------------------------------------------------ 61762
chimp54546-135457.revcompl.Pan.troglodytes.clone.rp43-41g5.GenBank:AC099533.36      ------------------------------------------------------------ 68417
human.FAM30B-LOC105372935-GGT2.NCBI.                                                 gcagctccttcagctcttcaccctggaaagcactccggtacaaaacaggaatattcctct 73907
human.FAM230E-LOC105377182-GGT3P.NCBI.                                               ------------------------------------------------------------ 57310
human.FAM230J-LOC105372942-GGTLC5P.NCBI.rev.compl                                    ------------------------------------------------------------ 46364
human.FAM230A.ncRNA.18487127.18500594-GGTLC3.18516335.18518165.rev.compl.REF         ------------------------------------------------------------ 31039


chimp.LOC112206744-LOC107973052-GGT2.revcompl                                        ------------------------------------------------------------ 61762
chimp54546-135457.revcompl.Pan.troglodytes.clone.rp43-41g5.GenBank:AC099533.36      ------------------------------------------------------------ 68417
human.FAM30B-LOC105372935-GGT2.NCBI.                                                 tctcctgactctgcccaaggcactgctgtgaccctctcagcagtggaagcactagccaat 73967
human.FAM230E-LOC105377182-GGT3P.NCBI.                                               ------------------------------------------------------------ 57310
human.FAM230J-LOC105372942-GGTLC5P.NCBI.rev.compl                                    ------------------------------------------------------------ 46364
human.FAM230A.ncRNA.18487127.18500594-GGTLC3.18516335.18518165.rev.compl.REF         ------------------------------------------------------------ 31039


chimp.LOC112206744-LOC107973052-GGT2.revcompl                                        ------------------------------------------------------------ 61762
chimp54546-135457.revcompl.Pan.troglodytes.clone.rp43-41g5.GenBank:AC099533.36      ------------------------------------------------------------ 68417
human.FAM30B-LOC105372935-GGT2.NCBI.                                                 gtgccatcctcctggacgatagctggataatcgcaataagcagagccccaacaactagct 74027
human.FAM230E-LOC105377182-GGT3P.NCBI.                                               ------------------------------------------------------------ 57310
human.FAM230J-LOC105372942-GGTLC5P.NCBI.rev.compl                                    ------------------------------------------------------------ 46364
human.FAM230A.ncRNA.18487127.18500594-GGTLC3.18516335.18518165.rev.compl.REF         ------------------------------------------------------------ 31039


chimp.LOC112206744-LOC107973052-GGT2.revcompl                                        ------------------------------------------------------------ 61762
chimp54546-135457.revcompl.Pan.troglodytes.clone.rp43-41g5.GenBank:AC099533.36      ------------------------------------------------------------ 68417
human.FAM30B-LOC105372935-GGT2.NCBI.                                                 ctagtgtgtggggagaagtggggaggatggagaaaggatacaaaaagacactgtgggatt 74087
human.FAM230E-LOC105377182-GGT3P.NCBI.                                               ------------------------------------------------------------ 57310
human.FAM230J-LOC105372942-GGTLC5P.NCBI.rev.compl                                    ------------------------------------------------------------ 46364
human.FAM230A.ncRNA.18487127.18500594-GGTLC3.18516335.18518165.rev.compl.REF         ------------------------------------------------------------ 31039


chimp.LOC112206744-LOC107973052-GGT2.revcompl                                        ------------------------------------------------------------ 61762
chimp54546-135457.revcompl.Pan.troglodytes.clone.rp43-41g5.GenBank:AC099533.36      ------------------------------------------------------------ 68417
human.FAM30B-LOC105372935-GGT2.NCBI.                                                 tcagggcaacccatgagtgaggatcacagcagagctatgccttgcaaggaccagaactgg 74147
human.FAM230E-LOC105377182-GGT3P.NCBI.                                               ------------------------------------------------------------ 57310
human.FAM230J-LOC105372942-GGTLC5P.NCBI.rev.compl                                    ------------------------------------------------------------ 46364
human.FAM230A.ncRNA.18487127.18500594-GGTLC3.18516335.18518165.rev.compl.REF         ------------------------------------------------------------ 31039


chimp.LOC112206744-LOC107973052-GGT2.revcompl                                        ------------------------------------------------------------ 61762
chimp54546-135457.revcompl.Pan.troglodytes.clone.rp43-41g5.GenBank:AC099533.36      ------------------------------------------------------------ 68417
```

```
human.FAM30B-LOC105372935-GGT2.NCBI.                                            aaaacctttgtgacttagaccgccctcctcaggacagctcccgccactcttcagggacaa 74207
human.FAM230E-LOC105377182-GGT3P.NCBI.                                          ------------------------------------------------------------ 57310
human.FAM230J-LOC105372942-GGTLC5P.NCBI.rev.compl                               ------------------------------------------------------------ 46364
human.FAM230A.ncRNA.18487127.18500594-GGTLC3.18516335.18518165.rev.compl.REF    ------------------------------------------------------------ 31039

chimp.LOC112206744-LOC107973052-GGT2.revcompl                                   ------------------------------------------------------------ 61762
chimp54546-135457.revcompl.Pan.troglodytes.clone.rp43-41g5.GenBank:AC099533.36  ------------------------------------------------------------ 68417
human.FAM30B-LOC105372935-GGT2.NCBI.                                            agttcctttactcagccatagtttctgtgctccaaatactcagcttacacatgcctgttg 74267
human.FAM230E-LOC105377182-GGT3P.NCBI.                                          ------------------------------------------------------------ 57310
human.FAM230J-LOC105372942-GGTLC5P.NCBI.rev.compl                               ------------------------------------------------------------ 46364
human.FAM230A.ncRNA.18487127.18500594-GGTLC3.18516335.18518165.rev.compl.REF    ------------------------------------------------------------ 31039

chimp.LOC112206744-LOC107973052-GGT2.revcompl                                   ------------------------------------------------------------ 61762
chimp54546-135457.revcompl.Pan.troglodytes.clone.rp43-41g5.GenBank:AC099533.36  ------------------------------------------------------------ 68417
human.FAM30B-LOC105372935-GGT2.NCBI.                                            ctgctgcactcaccactgtggttctcagtgcaaattccccagaaagcagtctgactggct 74327
human.FAM230E-LOC105377182-GGT3P.NCBI.                                          ------------------------------------------------------------ 57310
human.FAM230J-LOC105372942-GGTLC5P.NCBI.rev.compl                               ------------------------------------------------------------ 46364
human.FAM230A.ncRNA.18487127.18500594-GGTLC3.18516335.18518165.rev.compl.REF    ------------------------------------------------------------ 31039

chimp.LOC112206744-LOC107973052-GGT2.revcompl                                   ------------------------------------------------------------ 61762
chimp54546-135457.revcompl.Pan.troglodytes.clone.rp43-41g5.GenBank:AC099533.36  ------------------------------------------------------------ 68417
human.FAM30B-LOC105372935-GGT2.NCBI.                                            gggagaccttttttcttttaaccaggcccccaaagttgtagattttggggtcatttacaa 74387
human.FAM230E-LOC105377182-GGT3P.NCBI.                                          ------------------------------------------------------------ 57310
human.FAM230J-LOC105372942-GGTLC5P.NCBI.rev.compl                               ------------------------------------------------------------ 46364
human.FAM230A.ncRNA.18487127.18500594-GGTLC3.18516335.18518165.rev.compl.REF    ------------------------------------------------------------ 31039

chimp.LOC112206744-LOC107973052-GGT2.revcompl                                   ------------------------------------------------------------ 61762
chimp54546-135457.revcompl.Pan.troglodytes.clone.rp43-41g5.GenBank:AC099533.36  ------------------------------------------------------------ 68417
human.FAM30B-LOC105372935-GGT2.NCBI.                                            tatgcatttccccaacacactcagatctaggtggacttgaccagatgggcatctgtattc 74447
human.FAM230E-LOC105377182-GGT3P.NCBI.                                          ------------------------------------------------------------ 57310
human.FAM230J-LOC105372942-GGTLC5P.NCBI.rev.compl                               ------------------------------------------------------------ 46364
human.FAM230A.ncRNA.18487127.18500594-GGTLC3.18516335.18518165.rev.compl.REF    ------------------------------------------------------------ 31039

chimp.LOC112206744-LOC107973052-GGT2.revcompl                                   ------------------------------------------------------------ 61762
chimp54546-135457.revcompl.Pan.troglodytes.clone.rp43-41g5.GenBank:AC099533.36  ------------------------------------------------------------ 68417
human.FAM30B-LOC105372935-GGT2.NCBI.                                            cattacccagcatataaccactttgggcaacagggaatgtgaagggccccaaaccgttcc 74507
human.FAM230E-LOC105377182-GGT3P.NCBI.                                          ------------------------------------------------------------ 57310
human.FAM230J-LOC105372942-GGTLC5P.NCBI.rev.compl                               ------------------------------------------------------------ 46364
human.FAM230A.ncRNA.18487127.18500594-GGTLC3.18516335.18518165.rev.compl.REF    ------------------------------------------------------------ 31039

chimp.LOC112206744-LOC107973052-GGT2.revcompl                                   ------------------------------------------------------------ 61762
chimp54546-135457.revcompl.Pan.troglodytes.clone.rp43-41g5.GenBank:AC099533.36  ------------------------------------------------------------ 68417
human.FAM30B-LOC105372935-GGT2.NCBI.                                            ccaccaagagtgtgggtggcaggcaccctgaaatatgtctagcacactcataaagcatga 74567
human.FAM230E-LOC105377182-GGT3P.NCBI.                                          ------------------------------------------------------------ 57310
human.FAM230J-LOC105372942-GGTLC5P.NCBI.rev.compl                               ------------------------------------------------------------ 46364
human.FAM230A.ncRNA.18487127.18500594-GGTLC3.18516335.18518165.rev.compl.REF    ------------------------------------------------------------ 31039

chimp.LOC112206744-LOC107973052-GGT2.revcompl                                   ------------------------------------------------------------ 61762
chimp54546-135457.revcompl.Pan.troglodytes.clone.rp43-41g5.GenBank:AC099533.36  ------------------------------------------------------------ 68417
human.FAM30B-LOC105372935-GGT2.NCBI.                                            acatctacacacaactaaaaataaatagtgagagccctgacactgcttctggggggttttc 74627
human.FAM230E-LOC105377182-GGT3P.NCBI.                                          ------------------------------------------------------------ 57310
human.FAM230J-LOC105372942-GGTLC5P.NCBI.rev.compl                               ------------------------------------------------------------ 46364
human.FAM230A.ncRNA.18487127.18500594-GGTLC3.18516335.18518165.rev.compl.REF    ------------------------------------------------------------ 31039

chimp.LOC112206744-LOC107973052-GGT2.revcompl                                   ------------------------------------------------------------ 61762
chimp54546-135457.revcompl.Pan.troglodytes.clone.rp43-41g5.GenBank:AC099533.36  ------------------------------------------------------------ 68417
human.FAM30B-LOC105372935-GGT2.NCBI.                                            ccaagcacctgttacgaatggtaacaatggatggccgctccttctccctcagctttgact 74687
human.FAM230E-LOC105377182-GGT3P.NCBI.                                          ------------------------------------------------------------ 57310
human.FAM230J-LOC105372942-GGTLC5P.NCBI.rev.compl                               ------------------------------------------------------------ 46364
human.FAM230A.ncRNA.18487127.18500594-GGTLC3.18516335.18518165.rev.compl.REF    ------------------------------------------------------------ 31039

chimp.LOC112206744-LOC107973052-GGT2.revcompl                                   ------------------------------------------------------------ 61762
chimp54546-135457.revcompl.Pan.troglodytes.clone.rp43-41g5.GenBank:AC099533.36  ------------------------------------------------------------ 68417
human.FAM30B-LOC105372935-GGT2.NCBI.                                            ttcattgctggtggctcaggctcctgtgcttggctgtcatcggtgggtagccctggattg 74747
human.FAM230E-LOC105377182-GGT3P.NCBI.                                          ------------------------------------------------------------ 57310
human.FAM230J-LOC105372942-GGTLC5P.NCBI.rev.compl                               ------------------------------------------------------------ 46364
human.FAM230A.ncRNA.18487127.18500594-GGTLC3.18516335.18518165.rev.compl.REF    ------------------------------------------------------------ 31039

chimp.LOC112206744-LOC107973052-GGT2.revcompl                                   ------------------------------------------------------------ 61762
chimp54546-135457.revcompl.Pan.troglodytes.clone.rp43-41g5.GenBank:AC099533.36  ------------------------------------------------------------ 68417
human.FAM30B-LOC105372935-GGT2.NCBI.                                            gacattttccctattcaattcaaggaccatgttcttgagcataactatgaccaggcagta 74807
human.FAM230E-LOC105377182-GGT3P.NCBI.                                          ------------------------------------------------------------ 57310
human.FAM230J-LOC105372942-GGTLC5P.NCBI.rev.compl                               ------------------------------------------------------------ 46364
human.FAM230A.ncRNA.18487127.18500594-GGTLC3.18516335.18518165.rev.compl.REF    ------------------------------------------------------------ 31039

chimp.LOC112206744-LOC107973052-GGT2.revcompl                                   ------------------------------------------------------------ 61762
chimp54546-135457.revcompl.Pan.troglodytes.clone.rp43-41g5.GenBank:AC099533.36  ------------------------------------------------------------ 68417
human.FAM30B-LOC105372935-GGT2.NCBI.                                            ggcagtgggccctgctctttctttccagggaggcacagataagccagcatcacagatgca 74867
human.FAM230E-LOC105377182-GGT3P.NCBI.                                          ------------------------------------------------------------ 57310
human.FAM230J-LOC105372942-GGTLC5P.NCBI.rev.compl                               ------------------------------------------------------------ 46364
human.FAM230A.ncRNA.18487127.18500594-GGTLC3.18516335.18518165.rev.compl.REF    ------------------------------------------------------------ 31039

chimp.LOC112206744-LOC107973052-GGT2.revcompl                                   ------------------------------------------------------------ 61762
chimp54546-135457.revcompl.Pan.troglodytes.clone.rp43-41g5.GenBank:AC099533.36  ------------------------------------------------------------ 68417
human.FAM30B-LOC105372935-GGT2.NCBI.                                            aaaaacagaaccgtggaggtagagactaattcttcagcagcccagagaagagaactggga 74927
human.FAM230E-LOC105377182-GGT3P.NCBI.                                          ------------------------------------------------------------ 57310
human.FAM230J-LOC105372942-GGTLC5P.NCBI.rev.compl                               ------------------------------------------------------------ 46364
human.FAM230A.ncRNA.18487127.18500594-GGTLC3.18516335.18518165.rev.compl.REF    ------------------------------------------------------------ 31039

chimp.LOC112206744-LOC107973052-GGT2.revcompl                                   ------------------------------------------------------------ 61762
chimp54546-135457.revcompl.Pan.troglodytes.clone.rp43-41g5.GenBank:AC099533.36  ------------------------------------------------------------ 68417
human.FAM30B-LOC105372935-GGT2.NCBI.                                            aacagggccctcgcagatacacaagaccaagctggagagttcagggacttccaaaagaag 74987
human.FAM230E-LOC105377182-GGT3P.NCBI.                                          ------------------------------------------------------------ 57310
human.FAM230J-LOC105372942-GGTLC5P.NCBI.rev.compl                               ------------------------------------------------------------ 46364
human.FAM230A.ncRNA.18487127.18500594-GGTLC3.18516335.18518165.rev.compl.REF    ------------------------------------------------------------ 31039

chimp.LOC112206744-LOC107973052-GGT2.revcompl                                   ------------------------------------------------------------ 61762
chimp54546-135457.revcompl.Pan.troglodytes.clone.rp43-41g5.GenBank:AC099533.36  ------------------------------------------------------------ 68417
human.FAM30B-LOC105372935-GGT2.NCBI.                                            gtacaagaatgagcactataccatagtggggtgggtcacaaaagcaggaaaaggtcccca 75047
human.FAM230E-LOC105377182-GGT3P.NCBI.                                          ------------------------------------------------------------ 57310
human.FAM230J-LOC105372942-GGTLC5P.NCBI.rev.compl                               ------------------------------------------------------------ 46364
human.FAM230A.ncRNA.18487127.18500594-GGTLC3.18516335.18518165.rev.compl.REF    ------------------------------------------------------------ 31039

chimp.LOC112206744-LOC107973052-GGT2.revcompl                                   ------------------------------------------------------------ 61762
chimp54546-135457.revcompl.Pan.troglodytes.clone.rp43-41g5.GenBank:AC099533.36  ------------------------------------------------------------ 68417
human.FAM30B-LOC105372935-GGT2.NCBI.                                            aacaaagaatccatcagtcagttacagcactctccacatgtggtgaaacagtatgcaacc 75107
human.FAM230E-LOC105377182-GGT3P.NCBI.                                          ------------------------------------------------------------ 57310
human.FAM230J-LOC105372942-GGTLC5P.NCBI.rev.compl                               ------------------------------------------------------------ 46364
human.FAM230A.ncRNA.18487127.18500594-GGTLC3.18516335.18518165.rev.compl.REF    ------------------------------------------------------------ 31039

chimp.LOC112206744-LOC107973052-GGT2.revcompl                                   ------------------------------------------------------------ 61762
chimp54546-135457.revcompl.Pan.troglodytes.clone.rp43-41g5.GenBank:AC099533.36  ------------------------------------------------------------ 68417
human.FAM30B-LOC105372935-GGT2.NCBI.                                            atttaaaaactgcagagaacaatatttatgaacacctaaaaatgctgaaagcacactact 75167
human.FAM230E-LOC105377182-GGT3P.NCBI.                                          ------------------------------------------------------------ 57310
human.FAM230J-LOC105372942-GGTLC5P.NCBI.rev.compl                               ------------------------------------------------------------ 46364
human.FAM230A.ncRNA.18487127.18500594-GGTLC3.18516335.18518165.rev.compl.REF    ------------------------------------------------------------ 31039

chimp.LOC112206744-LOC107973052-GGT2.revcompl                                   ------------------------------------------------------------ 61762
chimp54546-135457.revcompl.Pan.troglodytes.clone.rp43-41g5.GenBank:AC099533.36  ------------------------------------------------------------ 68417
human.FAM30B-LOC105372935-GGT2.NCBI.                                            aagtaaaaggggagaaaggctataaaaggatttgaaagtattttcgtaaagataaacacc 75227
human.FAM230E-LOC105377182-GGT3P.NCBI.                                          ------------------------------------------------------------ 57310
human.FAM230J-LOC105372942-GGTLC5P.NCBI.rev.compl                               ------------------------------------------------------------ 46364
human.FAM230A.ncRNA.18487127.18500594-GGTLC3.18516335.18518165.rev.compl.REF    ------------------------------------------------------------ 31039

chimp.LOC112206744-LOC107973052-GGT2.revcompl                                   ------------------------------------------------------------ 61762
chimp54546-135457.revcompl.Pan.troglodytes.clone.rp43-41g5.GenBank:AC099533.36  ------------------------------------------------------------ 68417
human.FAM30B-LOC105372935-GGT2.NCBI.                                            cacacacacccacacacctctatatatgtgtggaaaaaggactgggctagtatatttaaa 75287
```

```
human.FAM230E-LOC105377182-GGT3P.NCBI.    ------------------------------------------------------------ 57310
human.FAM230J-LOC105372942-GGTLC5P.NCBI.rev.compl    ------------------------------------------------------------ 46364
human.FAM230A.ncRNA.18487127.18500594-GGTLC3.18516335.18518165.rev.compl.REF    ------------------------------------------------------------ 31039

chimp.LOC112206744-LOC107973052-GGT2.revcompl    ------------------------------------------------------------ 61762
chimp54546-135457.revcompl.Pan.troglodytes.clone.rp43-41g5.GenBank:AC099533.36    ------------------------------------------------------------ 68417
human.FAM30B-LOC105372935-GGT2.NCBI.    aaacgctaaagggaggtcgggtaataagggattgtttttccttgcactttgtgttttct 75347
human.FAM230E-LOC105377182-GGT3P.NCBI.    ------------------------------------------------------------ 57310
human.FAM230J-LOC105372942-GGTLC5P.NCBI.rev.compl    ------------------------------------------------------------ 46364
human.FAM230A.ncRNA.18487127.18500594-GGTLC3.18516335.18518165.rev.compl.REF    ------------------------------------------------------------ 31039

chimp.LOC112206744-LOC107973052-GGT2.revcompl    ------------------------------------------------------------ 61762
chimp54546-135457.revcompl.Pan.troglodytes.clone.rp43-41g5.GenBank:AC099533.36    ------------------------------------------------------------ 68417
human.FAM30B-LOC105372935-GGT2.NCBI.    aagtattctatagtctgaatgaattagattcataattaggaccaacagtcataagcaatt 75407
human.FAM230E-LOC105377182-GGT3P.NCBI.    ------------------------------------------------------------ 57310
human.FAM230J-LOC105372942-GGTLC5P.NCBI.rev.compl    ------------------------------------------------------------ 46364
human.FAM230A.ncRNA.18487127.18500594-GGTLC3.18516335.18518165.rev.compl.REF    ------------------------------------------------------------ 31039

chimp.LOC112206744-LOC107973052-GGT2.revcompl    ------------------------------------------------------------ 61762
chimp54546-135457.revcompl.Pan.troglodytes.clone.rp43-41g5.GenBank:AC099533.36    ------------------------------------------------------------ 68417
human.FAM30B-LOC105372935-GGT2.NCBI.    tctcagaccacctgcctcataaaacccaaaaaaagaagtcagagaggggtgaatattttg 75467
human.FAM230E-LOC105377182-GGT3P.NCBI.    ------------------------------------------------------------ 57310
human.FAM230J-LOC105372942-GGTLC5P.NCBI.rev.compl    ------------------------------------------------------------ 46364
human.FAM230A.ncRNA.18487127.18500594-GGTLC3.18516335.18518165.rev.compl.REF    ------------------------------------------------------------ 31039

chimp.LOC112206744-LOC107973052-GGT2.revcompl    ------------------------------------------------------------ 61762
chimp54546-135457.revcompl.Pan.troglodytes.clone.rp43-41g5.GenBank:AC099533.36    ------------------------------------------------------------ 68417
human.FAM30B-LOC105372935-GGT2.NCBI.    gtacagatgaattcaggttgtgtaagtggagaggagggaggtggccaaggagcctgaagt 75527
human.FAM230E-LOC105377182-GGT3P.NCBI.    ------------------------------------------------------------ 57310
human.FAM230J-LOC105372942-GGTLC5P.NCBI.rev.compl    ------------------------------------------------------------ 46364
human.FAM230A.ncRNA.18487127.18500594-GGTLC3.18516335.18518165.rev.compl.REF    ------------------------------------------------------------ 31039

chimp.LOC112206744-LOC107973052-GGT2.revcompl    ------------------------------------------------------------ 61762
chimp54546-135457.revcompl.Pan.troglodytes.clone.rp43-41g5.GenBank:AC099533.36    ------------------------------------------------------------ 68417
human.FAM30B-LOC105372935-GGT2.NCBI.    gggggagggggggctgatggtgaaatagggaatgggtggagagagaaaatgattgcaaaat 75587
human.FAM230E-LOC105377182-GGT3P.NCBI.    ------------------------------------------------------------ 57310
human.FAM230J-LOC105372942-GGTLC5P.NCBI.rev.compl    ------------------------------------------------------------ 46364
human.FAM230A.ncRNA.18487127.18500594-GGTLC3.18516335.18518165.rev.compl.REF    ------------------------------------------------------------ 31039

chimp.LOC112206744-LOC107973052-GGT2.revcompl    ------------------------------------------------------------ 61762
chimp54546-135457.revcompl.Pan.troglodytes.clone.rp43-41g5.GenBank:AC099533.36    ------------------------------------------------------------ 68417
human.FAM30B-LOC105372935-GGT2.NCBI.    tggctggagccagcctggtcaggtcaactttgcaggtgtgtgaagtgtcaaatggttaat 75647
human.FAM230E-LOC105377182-GGT3P.NCBI.    ------------------------------------------------------------ 57310
human.FAM230J-LOC105372942-GGTLC5P.NCBI.rev.compl    ------------------------------------------------------------ 46364
human.FAM230A.ncRNA.18487127.18500594-GGTLC3.18516335.18518165.rev.compl.REF    ------------------------------------------------------------ 31039

chimp.LOC112206744-LOC107973052-GGT2.revcompl    ------------------------------------------------------------ 61762
chimp54546-135457.revcompl.Pan.troglodytes.clone.rp43-41g5.GenBank:AC099533.36    ------------------------------------------------------------ 68417
human.FAM30B-LOC105372935-GGT2.NCBI.    cagttgccctctgtttgagctgcttaatgcacaagggcctggttgaacaggtttgacctc 75707
human.FAM230E-LOC105377182-GGT3P.NCBI.    ------------------------------------------------------------ 57310
human.FAM230J-LOC105372942-GGTLC5P.NCBI.rev.compl    ------------------------------------------------------------ 46364
human.FAM230A.ncRNA.18487127.18500594-GGTLC3.18516335.18518165.rev.compl.REF    ------------------------------------------------------------ 31039

chimp.LOC112206744-LOC107973052-GGT2.revcompl    ------------------------------------------------------------ 61762
chimp54546-135457.revcompl.Pan.troglodytes.clone.rp43-41g5.GenBank:AC099533.36    ------------------------------------------------------------ 68417
human.FAM30B-LOC105372935-GGT2.NCBI.    atttactttttcttaaagctgtttagccactttcctaaaaactcctattttctgagttat 75767
human.FAM230E-LOC105377182-GGT3P.NCBI.    ------------------------------------------------------------ 57310
human.FAM230J-LOC105372942-GGTLC5P.NCBI.rev.compl    ------------------------------------------------------------ 46364
human.FAM230A.ncRNA.18487127.18500594-GGTLC3.18516335.18518165.rev.compl.REF    ------------------------------------------------------------ 31039

chimp.LOC112206744-LOC107973052-GGT2.revcompl    ------------------------------------------------------------ 61762
chimp54546-135457.revcompl.Pan.troglodytes.clone.rp43-41g5.GenBank:AC099533.36    ------------------------------------------------------------ 68417
human.FAM30B-LOC105372935-GGT2.NCBI.    taaactttccattccatgttgatcccttccgagccctctaacgcatctcttctcttgctt 75827
human.FAM230E-LOC105377182-GGT3P.NCBI.    ------------------------------------------------------------ 57310
human.FAM230J-LOC105372942-GGTLC5P.NCBI.rev.compl    ------------------------------------------------------------ 46364
human.FAM230A.ncRNA.18487127.18500594-GGTLC3.18516335.18518165.rev.compl.REF    ------------------------------------------------------------ 31039

chimp.LOC112206744-LOC107973052-GGT2.revcompl    ------------------------------------------------------------ 61762
chimp54546-135457.revcompl.Pan.troglodytes.clone.rp43-41g5.GenBank:AC099533.36    ------------------------------------------------------------ 68417
human.FAM30B-LOC105372935-GGT2.NCBI.    cttccaactggatgccttcatgaggcattaacaggtactgtgagccctgatgaaaatcat 75887
human.FAM230E-LOC105377182-GGT3P.NCBI.    ------------------------------------------------------------ 57310
human.FAM230J-LOC105372942-GGTLC5P.NCBI.rev.compl    ------------------------------------------------------------ 46364
human.FAM230A.ncRNA.18487127.18500594-GGTLC3.18516335.18518165.rev.compl.REF    ------------------------------------------------------------ 31039

chimp.LOC112206744-LOC107973052-GGT2.revcompl    ------------------------------------------------------------ 61762
chimp54546-135457.revcompl.Pan.troglodytes.clone.rp43-41g5.GenBank:AC099533.36    ------------------------------------------------------------ 68417
human.FAM30B-LOC105372935-GGT2.NCBI.    cctttcagggtacaaggaaggcacctgaatgacagatggttggaggaatcttgagaaagc 75947
human.FAM230E-LOC105377182-GGT3P.NCBI.    ------------------------------------------------------------ 57310
human.FAM230J-LOC105372942-GGTLC5P.NCBI.rev.compl    ------------------------------------------------------------ 46364
human.FAM230A.ncRNA.18487127.18500594-GGTLC3.18516335.18518165.rev.compl.REF    ------------------------------------------------------------ 31039

chimp.LOC112206744-LOC107973052-GGT2.revcompl    ------------------------------------------------------------ 61762
chimp54546-135457.revcompl.Pan.troglodytes.clone.rp43-41g5.GenBank:AC099533.36    ------------------------------------------------------------ 68417
human.FAM30B-LOC105372935-GGT2.NCBI.    agtttaggcattaacatgcaggctgtcgctgacttacacagagccaaggacgtgacatac 76007
human.FAM230E-LOC105377182-GGT3P.NCBI.    ------------------------------------------------------------ 57310
human.FAM230J-LOC105372942-GGTLC5P.NCBI.rev.compl    ------------------------------------------------------------ 46364
human.FAM230A.ncRNA.18487127.18500594-GGTLC3.18516335.18518165.rev.compl.REF    ------------------------------------------------------------ 31039

chimp.LOC112206744-LOC107973052-GGT2.revcompl    ------------------------------------------------------------ 61762
chimp54546-135457.revcompl.Pan.troglodytes.clone.rp43-41g5.GenBank:AC099533.36    ------------------------------------------------------------ 68417
human.FAM30B-LOC105372935-GGT2.NCBI.    agcatggggccatttctaaatccccactgaggacaagggtcctggagggaatgggatttg 76067
human.FAM230E-LOC105377182-GGT3P.NCBI.    ------------------------------------------------------------ 57310
human.FAM230J-LOC105372942-GGTLC5P.NCBI.rev.compl    ------------------------------------------------------------ 46364
human.FAM230A.ncRNA.18487127.18500594-GGTLC3.18516335.18518165.rev.compl.REF    ------------------------------------------------------------ 31039

chimp.LOC112206744-LOC107973052-GGT2.revcompl    ------------------------------------------------------------ 61762
chimp54546-135457.revcompl.Pan.troglodytes.clone.rp43-41g5.GenBank:AC099533.36    ------------------------------------------------------------ 68417
human.FAM30B-LOC105372935-GGT2.NCBI.    gaacaagatttgggctttgaacattcgctacaggctctgacaaagcagtgatggttcaaa 76127
human.FAM230E-LOC105377182-GGT3P.NCBI.    ------------------------------------------------------------ 57310
human.FAM230J-LOC105372942-GGTLC5P.NCBI.rev.compl    ------------------------------------------------------------ 46364
human.FAM230A.ncRNA.18487127.18500594-GGTLC3.18516335.18518165.rev.compl.REF    ------------------------------------------------------------ 31039

chimp.LOC112206744-LOC107973052-GGT2.revcompl    ------------------------------------------------------------ 61762
chimp54546-135457.revcompl.Pan.troglodytes.clone.rp43-41g5.GenBank:AC099533.36    ------------------------------------------------------------ 68417
human.FAM30B-LOC105372935-GGT2.NCBI.    agcctctgccccctggtggcaagccctgcccagatgctgttaaggggctctccaggcacg 76187
human.FAM230E-LOC105377182-GGT3P.NCBI.    ------------------------------------------------------------ 57310
human.FAM230J-LOC105372942-GGTLC5P.NCBI.rev.compl    ------------------------------------------------------------ 46364
human.FAM230A.ncRNA.18487127.18500594-GGTLC3.18516335.18518165.rev.compl.REF    ------------------------------------------------------------ 31039

chimp.LOC112206744-LOC107973052-GGT2.revcompl    ------------------------------------------------------------ 61762
chimp54546-135457.revcompl.Pan.troglodytes.clone.rp43-41g5.GenBank:AC099533.36    ------------------------------------------------------------ 68417
human.FAM30B-LOC105372935-GGT2.NCBI.    ccctcctaattaggggcaaaatcagagaagcctctttggaagaagacaaaaggcacaatg 76247
human.FAM230E-LOC105377182-GGT3P.NCBI.    ------------------------------------------------------------ 57310
human.FAM230J-LOC105372942-GGTLC5P.NCBI.rev.compl    ------------------------------------------------------------ 46364
human.FAM230A.ncRNA.18487127.18500594-GGTLC3.18516335.18518165.rev.compl.REF    ------------------------------------------------------------ 31039

chimp.LOC112206744-LOC107973052-GGT2.revcompl    ------------------------------------------------------------ 61762
chimp54546-135457.revcompl.Pan.troglodytes.clone.rp43-41g5.GenBank:AC099533.36    ------------------------------------------------------------ 68417
human.FAM30B-LOC105372935-GGT2.NCBI.    agatgcggtctcctgaagtagaggctttctctctggttatcacctgaaacaggaaataag 76307
human.FAM230E-LOC105377182-GGT3P.NCBI.    ------------------------------------------------------------ 57310
human.FAM230J-LOC105372942-GGTLC5P.NCBI.rev.compl    ------------------------------------------------------------ 46364
human.FAM230A.ncRNA.18487127.18500594-GGTLC3.18516335.18518165.rev.compl.REF    ------------------------------------------------------------ 31039

chimp.LOC112206744-LOC107973052-GGT2.revcompl    ------------------------------------------------------------ 61762
chimp54546-135457.revcompl.Pan.troglodytes.clone.rp43-41g5.GenBank:AC099533.36    ------------------------------------------------------------ 68417
human.FAM30B-LOC105372935-GGT2.NCBI.    gactgagtacctgctcaattccaggcactagccaaatctgcagcaaactagcatttccaa 76367
human.FAM230E-LOC105377182-GGT3P.NCBI.    ------------------------------------------------------------ 57310
```

```
human.FAM230J-LOC105372942-GGTLC5P.NCBI.rev.compl  ------------------------------------------------------------ 46364
human.FAM230A.ncRNA.18487127.18500594-GGTLC3.18516335.18518165.rev.compl.REF  ------------------------------------------------------------ 31039

chimp.LOC112206744-LOC107973052-GGT2.revcompl  ------------------------------------------------------------ 61762
chimp54546-135457.revcompl.Pan.troglodytes.clone.rp43-41g5.GenBank:AC099533.36  ------------------------------------------------------------ 68417
human.FAM30B-LOC105372935-GGT2.NCBI.  aggtgtcttcttactttaatctcacagccaccctgtgagggaagtgtacattccccattt 76427
human.FAM230E-LOC105377182-GGT3P.NCBI.  ------------------------------------------------------------ 57310
human.FAM230J-LOC105372942-GGTLC5P.NCBI.rev.compl  ------------------------------------------------------------ 46364
human.FAM230A.ncRNA.18487127.18500594-GGTLC3.18516335.18518165.rev.compl.REF  ------------------------------------------------------------ 31039

chimp.LOC112206744-LOC107973052-GGT2.revcompl  ------------------------------------------------------------ 61762
chimp54546-135457.revcompl.Pan.troglodytes.clone.rp43-41g5.GenBank:AC099533.36  ------------------------------------------------------------ 68417
human.FAM30B-LOC105372935-GGT2.NCBI.  cgctgatgagatgagcaaactgaggctcggagaaattgtcagtgggatacagagttggca 76487
human.FAM230E-LOC105377182-GGT3P.NCBI.  ------------------------------------------------------------ 57310
human.FAM230J-LOC105372942-GGTLC5P.NCBI.rev.compl  ------------------------------------------------------------ 46364
human.FAM230A.ncRNA.18487127.18500594-GGTLC3.18516335.18518165.rev.compl.REF  ------------------------------------------------------------ 31039

chimp.LOC112206744-LOC107973052-GGT2.revcompl  ------------------------------------------------------------ 61762
chimp54546-135457.revcompl.Pan.troglodytes.clone.rp43-41g5.GenBank:AC099533.36  ------------------------------------------------------------ 68417
human.FAM30B-LOC105372935-GGT2.NCBI.  tccaaacccacaatctctgactctaagcttctgtttcattcattccacaaatatatatgg 76547
human.FAM230E-LOC105377182-GGT3P.NCBI.  ------------------------------------------------------------ 57310
human.FAM230J-LOC105372942-GGTLC5P.NCBI.rev.compl  ------------------------------------------------------------ 46364
human.FAM230A.ncRNA.18487127.18500594-GGTLC3.18516335.18518165.rev.compl.REF  ------------------------------------------------------------ 31039

chimp.LOC112206744-LOC107973052-GGT2.revcompl  ------------------------------------------------------------ 61762
chimp54546-135457.revcompl.Pan.troglodytes.clone.rp43-41g5.GenBank:AC099533.36  ------------------------------------------------------------ 68417
human.FAM30B-LOC105372935-GGT2.NCBI.  tgtctcctctttgctggacactgtactgggctgatgagctacaagacccaatctttacag 76607
human.FAM230E-LOC105377182-GGT3P.NCBI.  ------------------------------------------------------------ 57310
human.FAM230J-LOC105372942-GGTLC5P.NCBI.rev.compl  ------------------------------------------------------------ 46364
human.FAM230A.ncRNA.18487127.18500594-GGTLC3.18516335.18518165.rev.compl.REF  ------------------------------------------------------------ 31039

chimp.LOC112206744-LOC107973052-GGT2.revcompl  ------------------------------------------------------------ 61762
chimp54546-135457.revcompl.Pan.troglodytes.clone.rp43-41g5.GenBank:AC099533.36  ------------------------------------------------------------ 68417
human.FAM30B-LOC105372935-GGT2.NCBI.  tctaggaggcagagatagataagtaaacagaggcggcaaagcgtggcactgtgaaagatg 76667
human.FAM230E-LOC105377182-GGT3P.NCBI.  ------------------------------------------------------------ 57310
human.FAM230J-LOC105372942-GGTLC5P.NCBI.rev.compl  ------------------------------------------------------------ 46364
human.FAM230A.ncRNA.18487127.18500594-GGTLC3.18516335.18518165.rev.compl.REF  ------------------------------------------------------------ 31039

chimp.LOC112206744-LOC107973052-GGT2.revcompl  ------------------------------------------------------------ 61762
chimp54546-135457.revcompl.Pan.troglodytes.clone.rp43-41g5.GenBank:AC099533.36  ------------------------------------------------------------ 68417
human.FAM30B-LOC105372935-GGT2.NCBI.  cactgaggacaccgagctaggtgttccacaggcgtcaggaaaggcttcacacaagagagg 76727
human.FAM230E-LOC105377182-GGT3P.NCBI.  ------------------------------------------------------------ 57310
human.FAM230J-LOC105372942-GGTLC5P.NCBI.rev.compl  ------------------------------------------------------------ 46364
human.FAM230A.ncRNA.18487127.18500594-GGTLC3.18516335.18518165.rev.compl.REF  ------------------------------------------------------------ 31039

chimp.LOC112206744-LOC107973052-GGT2.revcompl  ------------------------------------------------------------ 61762
chimp54546-135457.revcompl.Pan.troglodytes.clone.rp43-41g5.GenBank:AC099533.36  ------------------------------------------------------------ 68417
human.FAM30B-LOC105372935-GGT2.NCBI.  ttgcttgtgcccagttctaattaggatgagtaggtatttctgctagatcgtggtggggtg 76787
human.FAM230E-LOC105377182-GGT3P.NCBI.  ------------------------------------------------------------ 57310
human.FAM230J-LOC105372942-GGTLC5P.NCBI.rev.compl  ------------------------------------------------------------ 46364
human.FAM230A.ncRNA.18487127.18500594-GGTLC3.18516335.18518165.rev.compl.REF  ------------------------------------------------------------ 31039

chimp.LOC112206744-LOC107973052-GGT2.revcompl  ------------------------------------------------------------ 61762
chimp54546-135457.revcompl.Pan.troglodytes.clone.rp43-41g5.GenBank:AC099533.36  ------------------------------------------------------------ 68417
human.FAM30B-LOC105372935-GGT2.NCBI.  gggaggacatcacataggacctcagcagagagcagctcgaatctatgcagagctgcacga 76847
human.FAM230E-LOC105377182-GGT3P.NCBI.  ------------------------------------------------------------ 57310
human.FAM230J-LOC105372942-GGTLC5P.NCBI.rev.compl  ------------------------------------------------------------ 46364
human.FAM230A.ncRNA.18487127.18500594-GGTLC3.18516335.18518165.rev.compl.REF  ------------------------------------------------------------ 31039

chimp.LOC112206744-LOC107973052-GGT2.revcompl  ------------------------------------------------------------ 61762
chimp54546-135457.revcompl.Pan.troglodytes.clone.rp43-41g5.GenBank:AC099533.36  ------------------------------------------------------------ 68417
human.FAM30B-LOC105372935-GGT2.NCBI.  ggagccggaggtccagagtactgcagcagtttgacattgatgggtggtagggtccatgaa 76907
human.FAM230E-LOC105377182-GGT3P.NCBI.  ------------------------------------------------------------ 57310
human.FAM230J-LOC105372942-GGTLC5P.NCBI.rev.compl  ------------------------------------------------------------ 46364
human.FAM230A.ncRNA.18487127.18500594-GGTLC3.18516335.18518165.rev.compl.REF  ------------------------------------------------------------ 31039

chimp.LOC112206744-LOC107973052-GGT2.revcompl  ------------------------------------------------------------ 61762
chimp54546-135457.revcompl.Pan.troglodytes.clone.rp43-41g5.GenBank:AC099533.36  ------------------------------------------------------------ 68417
human.FAM30B-LOC105372935-GGT2.NCBI.  agtagaggaagaccaagaggaagcagcagacaggggccaggccagggcagggatagggtg 76967
human.FAM230E-LOC105377182-GGT3P.NCBI.  ------------------------------------------------------------ 57310
human.FAM230J-LOC105372942-GGTLC5P.NCBI.rev.compl  ------------------------------------------------------------ 46364
human.FAM230A.ncRNA.18487127.18500594-GGTLC3.18516335.18518165.rev.compl.REF  ------------------------------------------------------------ 31039

chimp.LOC112206744-LOC107973052-GGT2.revcompl  ------------------------------------------------------------ 61762
chimp54546-135457.revcompl.Pan.troglodytes.clone.rp43-41g5.GenBank:AC099533.36  ------------------------------------------------------------ 68417
human.FAM30B-LOC105372935-GGT2.NCBI.  ctctactaaagagcatgggctttgaactatgagcagtggagagccactggggggtgtaaat 77027
human.FAM230E-LOC105377182-GGT3P.NCBI.  ------------------------------------------------------------ 57310
human.FAM230J-LOC105372942-GGTLC5P.NCBI.rev.compl  ------------------------------------------------------------ 46364
human.FAM230A.ncRNA.18487127.18500594-GGTLC3.18516335.18518165.rev.compl.REF  ------------------------------------------------------------ 31039

chimp.LOC112206744-LOC107973052-GGT2.revcompl  ------------------------------------------------------------ 61762
chimp54546-135457.revcompl.Pan.troglodytes.clone.rp43-41g5.GenBank:AC099533.36  ------------------------------------------------------------ 68417
human.FAM30B-LOC105372935-GGT2.NCBI.  aaggaaacagatattccaaatcctgccttgggaagattgttctggcaggagtgatgggaa 77087
human.FAM230E-LOC105377182-GGT3P.NCBI.  ------------------------------------------------------------ 57310
human.FAM230J-LOC105372942-GGTLC5P.NCBI.rev.compl  ------------------------------------------------------------ 46364
human.FAM230A.ncRNA.18487127.18500594-GGTLC3.18516335.18518165.rev.compl.REF  ------------------------------------------------------------ 31039

chimp.LOC112206744-LOC107973052-GGT2.revcompl  ------------------------------------------------------------ 61762
chimp54546-135457.revcompl.Pan.troglodytes.clone.rp43-41g5.GenBank:AC099533.36  ------------------------------------------------------------ 68417
human.FAM30B-LOC105372935-GGT2.NCBI.  aggggccctggcagaagattccttcatgctcctgcggtgatccaggtgagagctgctgag 77147
human.FAM230E-LOC105377182-GGT3P.NCBI.  ------------------------------------------------------------ 57310
human.FAM230J-LOC105372942-GGTLC5P.NCBI.rev.compl  ------------------------------------------------------------ 46364
human.FAM230A.ncRNA.18487127.18500594-GGTLC3.18516335.18518165.rev.compl.REF  ------------------------------------------------------------ 31039

chimp.LOC112206744-LOC107973052-GGT2.revcompl  ------------------------------------------------------------ 61762
chimp54546-135457.revcompl.Pan.troglodytes.clone.rp43-41g5.GenBank:AC099533.36  ------------------------------------------------------------ 68417
human.FAM30B-LOC105372935-GGT2.NCBI.  gaccccaaccaaatcagatcactggacctccactgggagtgaaagggggtggtctggcca 77207
human.FAM230E-LOC105377182-GGT3P.NCBI.  ------------------------------------------------------------ 57310
human.FAM230J-LOC105372942-GGTLC5P.NCBI.rev.compl  ------------------------------------------------------------ 46364
human.FAM230A.ncRNA.18487127.18500594-GGTLC3.18516335.18518165.rev.compl.REF  ------------------------------------------------------------ 31039

chimp.LOC112206744-LOC107973052-GGT2.revcompl  ------------------------------------------------------------ 61762
chimp54546-135457.revcompl.Pan.troglodytes.clone.rp43-41g5.GenBank:AC099533.36  ------------------------------------------------------------ 68417
human.FAM30B-LOC105372935-GGT2.NCBI.  gagagatagtaaagagatgtagttaacaggcaagagaatgtggggatatccagggagctg 77267
human.FAM230E-LOC105377182-GGT3P.NCBI.  ------------------------------------------------------------ 57310
human.FAM230J-LOC105372942-GGTLC5P.NCBI.rev.compl  ------------------------------------------------------------ 46364
human.FAM230A.ncRNA.18487127.18500594-GGTLC3.18516335.18518165.rev.compl.REF  ------------------------------------------------------------ 31039

chimp.LOC112206744-LOC107973052-GGT2.revcompl  ------------------------------------------------------------ 61762
chimp54546-135457.revcompl.Pan.troglodytes.clone.rp43-41g5.GenBank:AC099533.36  ------------------------------------------------------------ 68417
human.FAM30B-LOC105372935-GGT2.NCBI.  tccacaattcgcaggtcttaggcaactgcttgggagaagttgatgaggtcatcactggac 77327
human.FAM230E-LOC105377182-GGT3P.NCBI.  ------------------------------------------------------------ 57310
human.FAM230J-LOC105372942-GGTLC5P.NCBI.rev.compl  ------------------------------------------------------------ 46364
human.FAM230A.ncRNA.18487127.18500594-GGTLC3.18516335.18518165.rev.compl.REF  ------------------------------------------------------------ 31039

chimp.LOC112206744-LOC107973052-GGT2.revcompl  ------------------------------------------------------------ 61762
chimp54546-135457.revcompl.Pan.troglodytes.clone.rp43-41g5.GenBank:AC099533.36  ------------------------------------------------------------ 68417
human.FAM30B-LOC105372935-GGT2.NCBI.  agagaatctattccctgggcccaaccaactctctagaccatcctgccatgtgcctctgag 77387
human.FAM230E-LOC105377182-GGT3P.NCBI.  ------------------------------------------------------------ 57310
human.FAM230J-LOC105372942-GGTLC5P.NCBI.rev.compl  ------------------------------------------------------------ 46364
human.FAM230A.ncRNA.18487127.18500594-GGTLC3.18516335.18518165.rev.compl.REF  ------------------------------------------------------------ 31039

chimp.LOC112206744-LOC107973052-GGT2.revcompl  ------------------------------------------------------------ 61762
chimp54546-135457.revcompl.Pan.troglodytes.clone.rp43-41g5.GenBank:AC099533.36  ------------------------------------------------------------ 68417
human.FAM30B-LOC105372935-GGT2.NCBI.  aagtgagcttccccagccaagcccacaggaggacatatggagcaaaggtcaacagtcact 77447
human.FAM230E-LOC105377182-GGT3P.NCBI.  ------------------------------------------------------------ 57310
human.FAM230J-LOC105372942-GGTLC5P.NCBI.rev.compl  ------------------------------------------------------------ 46364
```

```
human.FAM230A.ncRNA.18487127.18500594-GGTLC3.18516335.18518165.rev.compl.REF        ------------------------------------------------------------ 31039

chimp.LOC112206744-LOC107973052-GGT2.revcompl                                       ------------------------------------------------------------ 61762
chimp54546-135457.revcompl.Pan.troglodytes.clone.rp43-41g5.GenBank:AC099533.36      ------------------------------------------------------------ 68417
human.FAM30B-LOC105372935-GGT2.NCBI.                                                cagctgatggatggcaagcattcagatgggaggtgcctccacaggccatgataacccatg 77507
human.FAM230E-LOC105377182-GGT3P.NCBI.                                              ------------------------------------------------------------ 57310
human.FAM230J-LOC105372942-GGTLC5P.NCBI.rev.compl                                   ------------------------------------------------------------ 46364
human.FAM230A.ncRNA.18487127.18500594-GGTLC3.18516335.18518165.rev.compl.REF        ------------------------------------------------------------ 31039

chimp.LOC112206744-LOC107973052-GGT2.revcompl                                       ------------------------------------------------------------ 61762
chimp54546-135457.revcompl.Pan.troglodytes.clone.rp43-41g5.GenBank:AC099533.36      ------------------------------------------------------------ 68417
human.FAM30B-LOC105372935-GGT2.NCBI.                                                gatccatttgagaaagatgccaaaaaacagttaattcaaggccactttaaaatacctggc 77567
human.FAM230E-LOC105377182-GGT3P.NCBI.                                              ------------------------------------------------------------ 57310
human.FAM230J-LOC105372942-GGTLC5P.NCBI.rev.compl                                   ------------------------------------------------------------ 46364
human.FAM230A.ncRNA.18487127.18500594-GGTLC3.18516335.18518165.rev.compl.REF        ------------------------------------------------------------ 31039

chimp.LOC112206744-LOC107973052-GGT2.revcompl                                       ------------------------------------------------------------ 61762
chimp54546-135457.revcompl.Pan.troglodytes.clone.rp43-41g5.GenBank:AC099533.36      ------------------------------------------------------------ 68417
human.FAM30B-LOC105372935-GGT2.NCBI.                                                attcaatcctcctcaatgtctccatcaactgtagaccctataactaccaaatgagctcca 77627
human.FAM230E-LOC105377182-GGT3P.NCBI.                                              ------------------------------------------------------------ 57310
human.FAM230J-LOC105372942-GGTLC5P.NCBI.rev.compl                                   ------------------------------------------------------------ 46364
human.FAM230A.ncRNA.18487127.18500594-GGTLC3.18516335.18518165.rev.compl.REF        ------------------------------------------------------------ 31039

chimp.LOC112206744-LOC107973052-GGT2.revcompl                                       ------------------------------------------------------------ 61762
chimp54546-135457.revcompl.Pan.troglodytes.clone.rp43-41g5.GenBank:AC099533.36      ------------------------------------------------------------ 68417
human.FAM30B-LOC105372935-GGT2.NCBI.                                                ctcgccacaaaggtcaagacacagaaacggtcagagagcagagcctattcctgaaaagca 77687
human.FAM230E-LOC105377182-GGT3P.NCBI.                                              ------------------------------------------------------------ 57310
human.FAM230J-LOC105372942-GGTLC5P.NCBI.rev.compl                                   ------------------------------------------------------------ 46364
human.FAM230A.ncRNA.18487127.18500594-GGTLC3.18516335.18518165.rev.compl.REF        ------------------------------------------------------------ 31039

chimp.LOC112206744-LOC107973052-GGT2.revcompl                                       ------------------------------------------------------------ 61762
chimp54546-135457.revcompl.Pan.troglodytes.clone.rp43-41g5.GenBank:AC099533.36      ------------------------------------------------------------ 68417
human.FAM30B-LOC105372935-GGT2.NCBI.                                                aaagactccaaactgccacaactcaaccagtgaagggctggggaagactgacaggttcac 77747
human.FAM230E-LOC105377182-GGT3P.NCBI.                                              ------------------------------------------------------------ 57310
human.FAM230J-LOC105372942-GGTLC5P.NCBI.rev.compl                                   ------------------------------------------------------------ 46364
human.FAM230A.ncRNA.18487127.18500594-GGTLC3.18516335.18518165.rev.compl.REF        ------------------------------------------------------------ 31039

chimp.LOC112206744-LOC107973052-GGT2.revcompl                                       ------------------------------------------------------------ 61762
chimp54546-135457.revcompl.Pan.troglodytes.clone.rp43-41g5.GenBank:AC099533.36      ------------------------------------------------------------ 68417
human.FAM30B-LOC105372935-GGT2.NCBI.                                                taagaaatgagaggaaggacaaacaaggtgttcaaattagaaaatgattccattaatgag 77807
human.FAM230E-LOC105377182-GGT3P.NCBI.                                              ------------------------------------------------------------ 57310
human.FAM230J-LOC105372942-GGTLC5P.NCBI.rev.compl                                   ------------------------------------------------------------ 46364
human.FAM230A.ncRNA.18487127.18500594-GGTLC3.18516335.18518165.rev.compl.REF        ------------------------------------------------------------ 31039

chimp.LOC112206744-LOC107973052-GGT2.revcompl                                       ------------------------------------------------------------ 61762
chimp54546-135457.revcompl.Pan.troglodytes.clone.rp43-41g5.GenBank:AC099533.36      ------------------------------------------------------------ 68417
human.FAM30B-LOC105372935-GGT2.NCBI.                                                ggctctgatgatatagttgtggaggttatcagtttgccagagctgcagatttaaaatact 77867
human.FAM230E-LOC105377182-GGT3P.NCBI.                                              ------------------------------------------------------------ 57310
human.FAM230J-LOC105372942-GGTLC5P.NCBI.rev.compl                                   ------------------------------------------------------------ 46364
human.FAM230A.ncRNA.18487127.18500594-GGTLC3.18516335.18518165.rev.compl.REF        ------------------------------------------------------------ 31039

chimp.LOC112206744-LOC107973052-GGT2.revcompl                                       ------------------------------------------------------------ 61762
chimp54546-135457.revcompl.Pan.troglodytes.clone.rp43-41g5.GenBank:AC099533.36      ------------------------------------------------------------ 68417
human.FAM30B-LOC105372935-GGT2.NCBI.                                                tacatggtcatcgaaaccaaaaaatgctgaccatcccccaaaacctcccaccccacctag 77927
human.FAM230E-LOC105377182-GGT3P.NCBI.                                              ------------------------------------------------------------ 57310
human.FAM230J-LOC105372942-GGTLC5P.NCBI.rev.compl                                   ------------------------------------------------------------ 46364
human.FAM230A.ncRNA.18487127.18500594-GGTLC3.18516335.18518165.rev.compl.REF        ------------------------------------------------------------ 31039

chimp.LOC112206744-LOC107973052-GGT2.revcompl                                       ------------------------------------------------------------ 61762
chimp54546-135457.revcompl.Pan.troglodytes.clone.rp43-41g5.GenBank:AC099533.36      ------------------------------------------------------------ 68417
human.FAM30B-LOC105372935-GGT2.NCBI.                                                tgctgataacaggagttatcagttgccttttgtgtacagctagatgagatatggatctgt 77987
human.FAM230E-LOC105377182-GGT3P.NCBI.                                              ------------------------------------------------------------ 57310
human.FAM230J-LOC105372942-GGTLC5P.NCBI.rev.compl                                   ------------------------------------------------------------ 46364
human.FAM230A.ncRNA.18487127.18500594-GGTLC3.18516335.18518165.rev.compl.REF        ------------------------------------------------------------ 31039

chimp.LOC112206744-LOC107973052-GGT2.revcompl                                       ------------------------------------------------------------ 61762
chimp54546-135457.revcompl.Pan.troglodytes.clone.rp43-41g5.GenBank:AC099533.36      ------------------------------------------------------------ 68417
human.FAM30B-LOC105372935-GGT2.NCBI.                                                gggggaagaaggggtgggcgtgctagcatctctcagctagactgtcatcggatggtgac 78047
human.FAM230E-LOC105377182-GGT3P.NCBI.                                              ------------------------------------------------------------ 57310
human.FAM230J-LOC105372942-GGTLC5P.NCBI.rev.compl                                   ------------------------------------------------------------ 46364
human.FAM230A.ncRNA.18487127.18500594-GGTLC3.18516335.18518165.rev.compl.REF        ------------------------------------------------------------ 31039

chimp.LOC112206744-LOC107973052-GGT2.revcompl                                       ------------------------------------------------------------ 61762
chimp54546-135457.revcompl.Pan.troglodytes.clone.rp43-41g5.GenBank:AC099533.36      ------------------------------------------------------------ 68417
human.FAM30B-LOC105372935-GGT2.NCBI.                                                attccccaggactcaagcagcatctgagaagccactttcagtatgactatggaacagcta 78107
human.FAM230E-LOC105377182-GGT3P.NCBI.                                              ------------------------------------------------------------ 57310
human.FAM230J-LOC105372942-GGTLC5P.NCBI.rev.compl                                   ------------------------------------------------------------ 46364
human.FAM230A.ncRNA.18487127.18500594-GGTLC3.18516335.18518165.rev.compl.REF        ------------------------------------------------------------ 31039

chimp.LOC112206744-LOC107973052-GGT2.revcompl                                       ------------------------------------------------------------ 61762
chimp54546-135457.revcompl.Pan.troglodytes.clone.rp43-41g5.GenBank:AC099533.36      ------------------------------------------------------------ 68417
human.FAM30B-LOC105372935-GGT2.NCBI.                                                accacacaacacagcatactgaatgaggagcctcgcaggctgagaagaaagcaaacccgg 78167
human.FAM230E-LOC105377182-GGT3P.NCBI.                                              ------------------------------------------------------------ 57310
human.FAM230J-LOC105372942-GGTLC5P.NCBI.rev.compl                                   ------------------------------------------------------------ 46364
human.FAM230A.ncRNA.18487127.18500594-GGTLC3.18516335.18518165.rev.compl.REF        ------------------------------------------------------------ 31039

chimp.LOC112206744-LOC107973052-GGT2.revcompl                                       ------------------------------------------------------------ 61762
chimp54546-135457.revcompl.Pan.troglodytes.clone.rp43-41g5.GenBank:AC099533.36      ------------------------------------------------------------ 68417
human.FAM30B-LOC105372935-GGT2.NCBI.                                                aaggcaacaaatggatacgtgcatgctgaaggactcgtgtttaattcttcccggtaatcc 78227
human.FAM230E-LOC105377182-GGT3P.NCBI.                                              ------------------------------------------------------------ 57310
human.FAM230J-LOC105372942-GGTLC5P.NCBI.rev.compl                                   ------------------------------------------------------------ 46364
human.FAM230A.ncRNA.18487127.18500594-GGTLC3.18516335.18518165.rev.compl.REF        ------------------------------------------------------------ 31039

chimp.LOC112206744-LOC107973052-GGT2.revcompl                                       ------------------------------------------------------------ 61762
chimp54546-135457.revcompl.Pan.troglodytes.clone.rp43-41g5.GenBank:AC099533.36      ------------------------------------------------------------ 68417
human.FAM30B-LOC105372935-GGT2.NCBI.                                                tttgtcaggcatatatttttgtctcctcaaacttaagaaagaaatccatgctctggaggg 78287
human.FAM230E-LOC105377182-GGT3P.NCBI.                                              ------------------------------------------------------------ 57310
human.FAM230J-LOC105372942-GGTLC5P.NCBI.rev.compl                                   ------------------------------------------------------------ 46364
human.FAM230A.ncRNA.18487127.18500594-GGTLC3.18516335.18518165.rev.compl.REF        ------------------------------------------------------------ 31039

chimp.LOC112206744-LOC107973052-GGT2.revcompl                                       ------------------------------------------------------------ 61762
chimp54546-135457.revcompl.Pan.troglodytes.clone.rp43-41g5.GenBank:AC099533.36      ------------------------------------------------------------ 68417
human.FAM30B-LOC105372935-GGT2.NCBI.                                                gagaaagagaggctgtactcattaactcagagctgacctcgtctggcaggattaactcgc 78347
human.FAM230E-LOC105377182-GGT3P.NCBI.                                              ------------------------------------------------------------ 57310
human.FAM230J-LOC105372942-GGTLC5P.NCBI.rev.compl                                   ------------------------------------------------------------ 46364
human.FAM230A.ncRNA.18487127.18500594-GGTLC3.18516335.18518165.rev.compl.REF        ------------------------------------------------------------ 31039

chimp.LOC112206744-LOC107973052-GGT2.revcompl                                       ------------------------------------------------------------ 61762
chimp54546-135457.revcompl.Pan.troglodytes.clone.rp43-41g5.GenBank:AC099533.36      ------------------------------------------------------------ 68417
human.FAM30B-LOC105372935-GGT2.NCBI.                                                catacctgtccagcccacagaacctggcctggagcatggagggagaaccacagccaactc 78407
human.FAM230E-LOC105377182-GGT3P.NCBI.                                              ------------------------------------------------------------ 57310
human.FAM230J-LOC105372942-GGTLC5P.NCBI.rev.compl                                   ------------------------------------------------------------ 46364
human.FAM230A.ncRNA.18487127.18500594-GGTLC3.18516335.18518165.rev.compl.REF        ------------------------------------------------------------ 31039

chimp.LOC112206744-LOC107973052-GGT2.revcompl                                       ------------------------------------------------------------ 61762
chimp54546-135457.revcompl.Pan.troglodytes.clone.rp43-41g5.GenBank:AC099533.36      ------------------------------------------------------------ 68417
human.FAM30B-LOC105372935-GGT2.NCBI.                                                cagaaaaataaaaggctgcagcgagaaacaagtggggccacagagagaaaggggttttga 78467
human.FAM230E-LOC105377182-GGT3P.NCBI.                                              ------------------------------------------------------------ 57310
human.FAM230J-LOC105372942-GGTLC5P.NCBI.rev.compl                                   ------------------------------------------------------------ 46364
human.FAM230A.ncRNA.18487127.18500594-GGTLC3.18516335.18518165.rev.compl.REF        ------------------------------------------------------------ 31039

chimp.LOC112206744-LOC107973052-GGT2.revcompl                                       ------------------------------------------------------------ 61762
chimp54546-135457.revcompl.Pan.troglodytes.clone.rp43-41g5.GenBank:AC099533.36      ------------------------------------------------------------ 68417
human.FAM30B-LOC105372935-GGT2.NCBI.                                                aaaagttctaaatccccatggctagggacagtgacatcagtgtgtttaccaagtatttga 78527
human.FAM230E-LOC105377182-GGT3P.NCBI.                                              ------------------------------------------------------------ 57310
human.FAM230J-LOC105372942-GGTLC5P.NCBI.rev.compl                                   ------------------------------------------------------------ 46364
human.FAM230A.ncRNA.18487127.18500594-GGTLC3.18516335.18518165.rev.compl.REF        ------------------------------------------------------------ 31039
```

```
chimp.LOC112206744-LOC107973052-GGT2.revcompl                                ------------------------------------------------------------ 61762
chimp54546-135457.revcompl.Pan.troglodytes.clone.rp43-41g5.GenBank:AC099533.36 ------------------------------------------------------------ 68417
human.FAM30B-LOC105372935-GGT2.NCBI.                                         tttacttgctcaggcaaaattccagaagagacactgggacctgtgtggagagtttcttca 78587
human.FAM230E-LOC105377182-GGT3P.NCBI.                                       ------------------------------------------------------------ 57310
human.FAM230J-LOC105372942-GGTLC5P.NCBI.rev.compl                            ------------------------------------------------------------ 46364
human.FAM230A.ncRNA.18487127.18500594-GGTLC3.18516335.18518165.rev.compl.REF ------------------------------------------------------------ 31039

chimp.LOC112206744-LOC107973052-GGT2.revcompl                                ------------------------------------------------------------ 61762
chimp54546-135457.revcompl.Pan.troglodytes.clone.rp43-41g5.GenBank:AC099533.36 ------------------------------------------------------------ 68417
human.FAM30B-LOC105372935-GGT2.NCBI.                                         ctgcagctatcctgccaagctcattgtaaagtgacgaattttgtggacacttacagagaa 78647
human.FAM230E-LOC105377182-GGT3P.NCBI.                                       ------------------------------------------------------------ 57310
human.FAM230J-LOC105372942-GGTLC5P.NCBI.rev.compl                            ------------------------------------------------------------ 46364
human.FAM230A.ncRNA.18487127.18500594-GGTLC3.18516335.18518165.rev.compl.REF ------------------------------------------------------------ 31039

chimp.LOC112206744-LOC107973052-GGT2.revcompl                                ------------------------------------------------------------ 61762
chimp54546-135457.revcompl.Pan.troglodytes.clone.rp43-41g5.GenBank:AC099533.36 ------------------------------------------------------------ 68417
human.FAM30B-LOC105372935-GGT2.NCBI.                                         aaaatagcttcaaatgctatagaccaatgctgcacgctgcagagacacagtctttgggtt 78707
human.FAM230E-LOC105377182-GGT3P.NCBI.                                       ------------------------------------------------------------ 57310
human.FAM230J-LOC105372942-GGTLC5P.NCBI.rev.compl                            ------------------------------------------------------------ 46364
human.FAM230A.ncRNA.18487127.18500594-GGTLC3.18516335.18518165.rev.compl.REF ------------------------------------------------------------ 31039

chimp.LOC112206744-LOC107973052-GGT2.revcompl                                ------------------------------------------------------------ 61762
chimp54546-135457.revcompl.Pan.troglodytes.clone.rp43-41g5.GenBank:AC099533.36 ------------------------------------------------------------ 68417
human.FAM30B-LOC105372935-GGT2.NCBI.                                         ctgtgagctgtatttgggcattacgcagagttcagctgccatctgtccctttgaaggcca 78767
human.FAM230E-LOC105377182-GGT3P.NCBI.                                       ------------------------------------------------------------ 57310
human.FAM230J-LOC105372942-GGTLC5P.NCBI.rev.compl                            ------------------------------------------------------------ 46364
human.FAM230A.ncRNA.18487127.18500594-GGTLC3.18516335.18518165.rev.compl.REF ------------------------------------------------------------ 31039

chimp.LOC112206744-LOC107973052-GGT2.revcompl                                ------------------------------------------------------------ 61762
chimp54546-135457.revcompl.Pan.troglodytes.clone.rp43-41g5.GenBank:AC099533.36 ------------------------------------------------------------ 68417
human.FAM30B-LOC105372935-GGT2.NCBI.                                         gctaagaagttggtcccagcctgggcatggtaccactgtcctacattccagccatttggg 78827
human.FAM230E-LOC105377182-GGT3P.NCBI.                                       ------------------------------------------------------------ 57310
human.FAM230J-LOC105372942-GGTLC5P.NCBI.rev.compl                            ------------------------------------------------------------ 46364
human.FAM230A.ncRNA.18487127.18500594-GGTLC3.18516335.18518165.rev.compl.REF ------------------------------------------------------------ 31039

chimp.LOC112206744-LOC107973052-GGT2.revcompl                                ------------------------------------------------------------ 61762
chimp54546-135457.revcompl.Pan.troglodytes.clone.rp43-41g5.GenBank:AC099533.36 ------------------------------------------------------------ 68417
human.FAM30B-LOC105372935-GGT2.NCBI.                                         atatcctgggttcaagcattccctcccattgctggatcaagcacttcccacctcctgctc 78887
human.FAM230E-LOC105377182-GGT3P.NCBI.                                       ------------------------------------------------------------ 57310
human.FAM230J-LOC105372942-GGTLC5P.NCBI.rev.compl                            ------------------------------------------------------------ 46364
human.FAM230A.ncRNA.18487127.18500594-GGTLC3.18516335.18518165.rev.compl.REF ------------------------------------------------------------ 31039

chimp.LOC112206744-LOC107973052-GGT2.revcompl                                ------------------------------------------------------------ 61762
chimp54546-135457.revcompl.Pan.troglodytes.clone.rp43-41g5.GenBank:AC099533.36 ------------------------------------------------------------ 68417
human.FAM30B-LOC105372935-GGT2.NCBI.                                         tgggaaatcagatctcagtaggtcatcaaccatcccagaaagtccagcagagttggtttt 78947
human.FAM230E-LOC105377182-GGT3P.NCBI.                                       ------------------------------------------------------------ 57310
human.FAM230J-LOC105372942-GGTLC5P.NCBI.rev.compl                            ------------------------------------------------------------ 46364
human.FAM230A.ncRNA.18487127.18500594-GGTLC3.18516335.18518165.rev.compl.REF ------------------------------------------------------------ 31039

chimp.LOC112206744-LOC107973052-GGT2.revcompl                                ------------------------------------------------------------ 61762
chimp54546-135457.revcompl.Pan.troglodytes.clone.rp43-41g5.GenBank:AC099533.36 ------------------------------------------------------------ 68417
human.FAM30B-LOC105372935-GGT2.NCBI.                                         ggtttggggggtgtttttgagttgggtggaagaagagaccttacataatcttttaacttt 79007
human.FAM230E-LOC105377182-GGT3P.NCBI.                                       ------------------------------------------------------------ 57310
human.FAM230J-LOC105372942-GGTLC5P.NCBI.rev.compl                            ------------------------------------------------------------ 46364
human.FAM230A.ncRNA.18487127.18500594-GGTLC3.18516335.18518165.rev.compl.REF ------------------------------------------------------------ 31039

chimp.LOC112206744-LOC107973052-GGT2.revcompl                                ------------------------------------------------------------ 61762
chimp54546-135457.revcompl.Pan.troglodytes.clone.rp43-41g5.GenBank:AC099533.36 ------------------------------------------------------------ 68417
human.FAM30B-LOC105372935-GGT2.NCBI.                                         ttctctaaggctgaattccctataagcaaaatttaaaattttttttaaagaagaaaaac 79067
human.FAM230E-LOC105377182-GGT3P.NCBI.                                       ------------------------------------------------------------ 57310
human.FAM230J-LOC105372942-GGTLC5P.NCBI.rev.compl                            ------------------------------------------------------------ 46364
human.FAM230A.ncRNA.18487127.18500594-GGTLC3.18516335.18518165.rev.compl.REF ------------------------------------------------------------ 31039

chimp.LOC112206744-LOC107973052-GGT2.revcompl                                ------------------------------------------------------------ 61762
chimp54546-135457.revcompl.Pan.troglodytes.clone.rp43-41g5.GenBank:AC099533.36 ------------------------------------------------------------ 68417
human.FAM30B-LOC105372935-GGT2.NCBI.                                         acccagacaatccttaaaatcccctttcagctccaagactgtgtaattctgtgataccag 79127
human.FAM230E-LOC105377182-GGT3P.NCBI.                                       ------------------------------------------------------------ 57310
human.FAM230J-LOC105372942-GGTLC5P.NCBI.rev.compl                            ------------------------------------------------------------ 46364
human.FAM230A.ncRNA.18487127.18500594-GGTLC3.18516335.18518165.rev.compl.REF ------------------------------------------------------------ 31039

chimp.LOC112206744-LOC107973052-GGT2.revcompl                                ------------------------------------------------------------ 61762
chimp54546-135457.revcompl.Pan.troglodytes.clone.rp43-41g5.GenBank:AC099533.36 ------------------------------------------------------------ 68417
human.FAM30B-LOC105372935-GGT2.NCBI.                                         tattgaaagcccctgcatttccctaactatatattcccctatgccagggaaaggtgcatg 79187
human.FAM230E-LOC105377182-GGT3P.NCBI.                                       ------------------------------------------------------------ 57310
human.FAM230J-LOC105372942-GGTLC5P.NCBI.rev.compl                            ------------------------------------------------------------ 46364
human.FAM230A.ncRNA.18487127.18500594-GGTLC3.18516335.18518165.rev.compl.REF ------------------------------------------------------------ 31039

chimp.LOC112206744-LOC107973052-GGT2.revcompl                                ------------------------------------------------------------ 61762
chimp54546-135457.revcompl.Pan.troglodytes.clone.rp43-41g5.GenBank:AC099533.36 ------------------------------------------------------------ 68417
human.FAM30B-LOC105372935-GGT2.NCBI.                                         taagtgagagaacaaaaaatttcacaatcgtggggacctcttctttcggctttggagccc 79247
human.FAM230E-LOC105377182-GGT3P.NCBI.                                       ------------------------------------------------------------ 57310
human.FAM230J-LOC105372942-GGTLC5P.NCBI.rev.compl                            ------------------------------------------------------------ 46364
human.FAM230A.ncRNA.18487127.18500594-GGTLC3.18516335.18518165.rev.compl.REF ------------------------------------------------------------ 31039

chimp.LOC112206744-LOC107973052-GGT2.revcompl                                ------------------------------------------------------------ 61762
chimp54546-135457.revcompl.Pan.troglodytes.clone.rp43-41g5.GenBank:AC099533.36 ------------------------------------------------------------ 68417
human.FAM30B-LOC105372935-GGT2.NCBI.                                         ccttccctctgtctctgtacggggcagctttttccttctgtcttctcccttccttcttgt 79307
human.FAM230E-LOC105377182-GGT3P.NCBI.                                       ------------------------------------------------------------ 57310
human.FAM230J-LOC105372942-GGTLC5P.NCBI.rev.compl                            ------------------------------------------------------------ 46364
human.FAM230A.ncRNA.18487127.18500594-GGTLC3.18516335.18518165.rev.compl.REF ------------------------------------------------------------ 31039

chimp.LOC112206744-LOC107973052-GGT2.revcompl                                ------------------------------------------------------------ 61762
chimp54546-135457.revcompl.Pan.troglodytes.clone.rp43-41g5.GenBank:AC099533.36 ------------------------------------------------------------ 68417
human.FAM30B-LOC105372935-GGT2.NCBI.                                         ctactaaactctctgcgccttagaaccaaaaataaaaattcaaaatcatgatcttagggg 79367
human.FAM230E-LOC105377182-GGT3P.NCBI.                                       ------------------------------------------------------------ 57310
human.FAM230J-LOC105372942-GGTLC5P.NCBI.rev.compl                            ------------------------------------------------------------ 46364
human.FAM230A.ncRNA.18487127.18500594-GGTLC3.18516335.18518165.rev.compl.REF ------------------------------------------------------------ 31039

chimp.LOC112206744-LOC107973052-GGT2.revcompl                                ------------------------------------------------------------ 61762
chimp54546-135457.revcompl.Pan.troglodytes.clone.rp43-41g5.GenBank:AC099533.36 ------------------------------------------------------------ 68417
human.FAM30B-LOC105372935-GGT2.NCBI.                                         tgaaagaacactgagaaaccatagaaagcaaaaacgaaaagccaacatagaaaaagtctg 79427
human.FAM230E-LOC105377182-GGT3P.NCBI.                                       ------------------------------------------------------------ 57310
human.FAM230J-LOC105372942-GGTLC5P.NCBI.rev.compl                            ------------------------------------------------------------ 46364
human.FAM230A.ncRNA.18487127.18500594-GGTLC3.18516335.18518165.rev.compl.REF ------------------------------------------------------------ 31039

chimp.LOC112206744-LOC107973052-GGT2.revcompl                                ------------------------------------------------------------ 61762
chimp54546-135457.revcompl.Pan.troglodytes.clone.rp43-41g5.GenBank:AC099533.36 ------------------------------------------------------------ 68417
human.FAM30B-LOC105372935-GGT2.NCBI.                                         acttgaattagaagcagcacttaaaattacaggttcagaaaaagcttgaacagaatccag 79487
human.FAM230E-LOC105377182-GGT3P.NCBI.                                       ------------------------------------------------------------ 57310
human.FAM230J-LOC105372942-GGTLC5P.NCBI.rev.compl                            ------------------------------------------------------------ 46364
human.FAM230A.ncRNA.18487127.18500594-GGTLC3.18516335.18518165.rev.compl.REF ------------------------------------------------------------ 31039

chimp.LOC112206744-LOC107973052-GGT2.revcompl                                ------------------------------------------------------------ 61762
chimp54546-135457.revcompl.Pan.troglodytes.clone.rp43-41g5.GenBank:AC099533.36 ------------------------------------------------------------ 68417
human.FAM30B-LOC105372935-GGT2.NCBI.                                         ggctgaggtcagccctgagtctgcaggctctgccctctcacaaaggtgagtagatgggtc 79547
human.FAM230E-LOC105377182-GGT3P.NCBI.                                       ------------------------------------------------------------ 57310
human.FAM230J-LOC105372942-GGTLC5P.NCBI.rev.compl                            ------------------------------------------------------------ 46364
human.FAM230A.ncRNA.18487127.18500594-GGTLC3.18516335.18518165.rev.compl.REF ------------------------------------------------------------ 31039

chimp.LOC112206744-LOC107973052-GGT2.revcompl                                ------------------------------------------------------------ 61762
chimp54546-135457.revcompl.Pan.troglodytes.clone.rp43-41g5.GenBank:AC099533.36 ------------------------------------------------------------ 68417
human.FAM30B-LOC105372935-GGT2.NCBI.                                         tggagagggctgttcatgctatggaagggcctctatgccatcgaggctaaaaccaaagca 79607
human.FAM230E-LOC105377182-GGT3P.NCBI.                                       ------------------------------------------------------------ 57310
human.FAM230J-LOC105372942-GGTLC5P.NCBI.rev.compl                            ------------------------------------------------------------ 46364
human.FAM230A.ncRNA.18487127.18500594-GGTLC3.18516335.18518165.rev.compl.REF ------------------------------------------------------------ 31039
```

```
chimp.LOC112206744-LOC107973052-GGT2.revcompl                                    ------------------------------------------------------------ 61762
chimp54546-135457.revcompl.Pan.troglodytes.clone.rp43-41g5.GenBank:AC099533.36   ------------------------------------------------------------ 68417
human.FAM30B-LOC105372935-GGT2.NCBI.                                             ggagcaaatcagccctgctagtgtgtacagtcggcatcaaagaggcaacaacggtagaga 79667
human.FAM230E-LOC105377182-GGT3P.NCBI.                                           ------------------------------------------------------------ 57310
human.FAM230J-LOC105372942-GGTLC5P.NCBI.rev.compl                                ------------------------------------------------------------ 46364
human.FAM230A.ncRNA.18487127.18500594-GGTLC3.18516335.18518165.rev.compl.REF     ------------------------------------------------------------ 31039

chimp.LOC112206744-LOC107973052-GGT2.revcompl                                    ------------------------------------------------------------ 61762
chimp54546-135457.revcompl.Pan.troglodytes.clone.rp43-41g5.GenBank:AC099533.36   ------------------------------------------------------------ 68417
human.FAM30B-LOC105372935-GGT2.NCBI.                                             atattttctgaaagacacacatgaaaccagtaacggcgatggtctctggaagaattaggt 79727
human.FAM230E-LOC105377182-GGT3P.NCBI.                                           ------------------------------------------------------------ 57310
human.FAM230J-LOC105372942-GGTLC5P.NCBI.rev.compl                                ------------------------------------------------------------ 46364
human.FAM230A.ncRNA.18487127.18500594-GGTLC3.18516335.18518165.rev.compl.REF     ------------------------------------------------------------ 31039

chimp.LOC112206744-LOC107973052-GGT2.revcompl                                    ------------------------------------------------------------ 61762
chimp54546-135457.revcompl.Pan.troglodytes.clone.rp43-41g5.GenBank:AC099533.36   ------------------------------------------------------------ 68417
human.FAM30B-LOC105372935-GGT2.NCBI.                                             cagaagcagacttaatttgcactgtgtacattctttggtatcctttttaattgaacgcat 79787
human.FAM230E-LOC105377182-GGT3P.NCBI.                                           ------------------------------------------------------------ 57310
human.FAM230J-LOC105372942-GGTLC5P.NCBI.rev.compl                                ------------------------------------------------------------ 46364
human.FAM230A.ncRNA.18487127.18500594-GGTLC3.18516335.18518165.rev.compl.REF     ------------------------------------------------------------ 31039

chimp.LOC112206744-LOC107973052-GGT2.revcompl                                    ------------------------------------------------------------ 61762
chimp54546-135457.revcompl.Pan.troglodytes.clone.rp43-41g5.GenBank:AC099533.36   ------------------------------------------------------------ 68417
human.FAM30B-LOC105372935-GGT2.NCBI.                                             gtgaatgcattacctattcaaaaataaatgtgtaaaaacacatgccacgacaaagcaaaa 79847
human.FAM230E-LOC105377182-GGT3P.NCBI.                                           ------------------------------------------------------------ 57310
human.FAM230J-LOC105372942-GGTLC5P.NCBI.rev.compl                                ------------------------------------------------------------ 46364
human.FAM230A.ncRNA.18487127.18500594-GGTLC3.18516335.18518165.rev.compl.REF     ------------------------------------------------------------ 31039

chimp.LOC112206744-LOC107973052-GGT2.revcompl                                    ------------------------------------------------------------ 61762
chimp54546-135457.revcompl.Pan.troglodytes.clone.rp43-41g5.GenBank:AC099533.36   ------------------------------------------------------------ 68417
human.FAM30B-LOC105372935-GGT2.NCBI.                                             tgaggagacagtataaaaaagacaaggataattttaaatttctcctaataaaacgctgtt 79907
human.FAM230E-LOC105377182-GGT3P.NCBI.                                           ------------------------------------------------------------ 57310
human.FAM230J-LOC105372942-GGTLC5P.NCBI.rev.compl                                ------------------------------------------------------------ 46364
human.FAM230A.ncRNA.18487127.18500594-GGTLC3.18516335.18518165.rev.compl.REF     ------------------------------------------------------------ 31039

chimp.LOC112206744-LOC107973052-GGT2.revcompl                                    ------------------------------------------------------------ 61762
chimp54546-135457.revcompl.Pan.troglodytes.clone.rp43-41g5.GenBank:AC099533.36   ------------------------------------------------------------ 68417
human.FAM30B-LOC105372935-GGT2.NCBI.                                             taaaaatctaagaggaaaaaagggtaggctccagctagtggagagaacaactgaaaaaca 79967
human.FAM230E-LOC105377182-GGT3P.NCBI.                                           ------------------------------------------------------------ 57310
human.FAM230J-LOC105372942-GGTLC5P.NCBI.rev.compl                                ------------------------------------------------------------ 46364
human.FAM230A.ncRNA.18487127.18500594-GGTLC3.18516335.18518165.rev.compl.REF     ------------------------------------------------------------ 31039

chimp.LOC112206744-LOC107973052-GGT2.revcompl                                    ------------------------------------------------------------ 61762
chimp54546-135457.revcompl.Pan.troglodytes.clone.rp43-41g5.GenBank:AC099533.36   ------------------------------------------------------------ 68417
human.FAM30B-LOC105372935-GGT2.NCBI.                                             agagccacgtgttcccatggtcctggccaaccccacttctttcccatcctcactcgttca 80027
human.FAM230E-LOC105377182-GGT3P.NCBI.                                           ------------------------------------------------------------ 57310
human.FAM230J-LOC105372942-GGTLC5P.NCBI.rev.compl                                ------------------------------------------------------------ 46364
human.FAM230A.ncRNA.18487127.18500594-GGTLC3.18516335.18518165.rev.compl.REF     ------------------------------------------------------------ 31039

chimp.LOC112206744-LOC107973052-GGT2.revcompl                                    ------------------------------------------------------------ 61762
chimp54546-135457.revcompl.Pan.troglodytes.clone.rp43-41g5.GenBank:AC099533.36   ------------------------------------------------------------ 68417
human.FAM30B-LOC105372935-GGT2.NCBI.                                             catctgccttggagcacccacagcaagcctggcttcatgcccagaacatcacctggcttt 80087
human.FAM230E-LOC105377182-GGT3P.NCBI.                                           ------------------------------------------------------------ 57310
human.FAM230J-LOC105372942-GGTLC5P.NCBI.rev.compl                                ------------------------------------------------------------ 46364
human.FAM230A.ncRNA.18487127.18500594-GGTLC3.18516335.18518165.rev.compl.REF     ------------------------------------------------------------ 31039

chimp.LOC112206744-LOC107973052-GGT2.revcompl                                    ------------------------------------------------------------ 61762
chimp54546-135457.revcompl.Pan.troglodytes.clone.rp43-41g5.GenBank:AC099533.36   ------------------------------------------------------------ 68417
human.FAM30B-LOC105372935-GGT2.NCBI.                                             cctcctctctccctgcccacatctcctcagtcactcgctgcctcctctcttgcttctctc 80147
human.FAM230E-LOC105377182-GGT3P.NCBI.                                           ------------------------------------------------------------ 57310
human.FAM230J-LOC105372942-GGTLC5P.NCBI.rev.compl                                ------------------------------------------------------------ 46364
human.FAM230A.ncRNA.18487127.18500594-GGTLC3.18516335.18518165.rev.compl.REF     ------------------------------------------------------------ 31039

chimp.LOC112206744-LOC107973052-GGT2.revcompl                                    ------------------------------------------------------------ 61762
chimp54546-135457.revcompl.Pan.troglodytes.clone.rp43-41g5.GenBank:AC099533.36   ------------------------------------------------------------ 68417
human.FAM30B-LOC105372935-GGT2.NCBI.                                             ccagttctcagtttgcccatcaaatctcaggtggtactctccattctctgctatgaggga 80207
human.FAM230E-LOC105377182-GGT3P.NCBI.                                           ------------------------------------------------------------ 57310
human.FAM230J-LOC105372942-GGTLC5P.NCBI.rev.compl                                ------------------------------------------------------------ 46364
human.FAM230A.ncRNA.18487127.18500594-GGTLC3.18516335.18518165.rev.compl.REF     ------------------------------------------------------------ 31039

chimp.LOC112206744-LOC107973052-GGT2.revcompl                                    ------------------------------------------------------------ 61762
chimp54546-135457.revcompl.Pan.troglodytes.clone.rp43-41g5.GenBank:AC099533.36   ------------------------------------------------------------ 68417
human.FAM30B-LOC105372935-GGT2.NCBI.                                             gttcatttgttcatgcaatttccccaccggagacaaaatgtatttcccaaagttggcttg 80267
human.FAM230E-LOC105377182-GGT3P.NCBI.                                           ------------------------------------------------------------ 57310
human.FAM230J-LOC105372942-GGTLC5P.NCBI.rev.compl                                ------------------------------------------------------------ 46364
human.FAM230A.ncRNA.18487127.18500594-GGTLC3.18516335.18518165.rev.compl.REF     ------------------------------------------------------------ 31039

chimp.LOC112206744-LOC107973052-GGT2.revcompl                                    ------------------------------------------------------------ 61762
chimp54546-135457.revcompl.Pan.troglodytes.clone.rp43-41g5.GenBank:AC099533.36   ------------------------------------------------------------ 68417
human.FAM30B-LOC105372935-GGT2.NCBI.                                             caatgacatctcccatgcctgctctgcttacccatgactttgacactccttccatcgaga 80327
human.FAM230E-LOC105377182-GGT3P.NCBI.                                           ------------------------------------------------------------ 57310
human.FAM230J-LOC105372942-GGTLC5P.NCBI.rev.compl                                ------------------------------------------------------------ 46364
human.FAM230A.ncRNA.18487127.18500594-GGTLC3.18516335.18518165.rev.compl.REF     ------------------------------------------------------------ 31039

chimp.LOC112206744-LOC107973052-GGT2.revcompl                                    ------------------------------------------------------------ 61762
chimp54546-135457.revcompl.Pan.troglodytes.clone.rp43-41g5.GenBank:AC099533.36   ------------------------------------------------------------ 68417
human.FAM30B-LOC105372935-GGT2.NCBI.                                             gggcagaacctgtgtttcctctctctgaatctaggtgggctacagctctggtagaagtga 80387
human.FAM230E-LOC105377182-GGT3P.NCBI.                                           ------------------------------------------------------------ 57310
human.FAM230J-LOC105372942-GGTLC5P.NCBI.rev.compl                                ------------------------------------------------------------ 46364
human.FAM230A.ncRNA.18487127.18500594-GGTLC3.18516335.18518165.rev.compl.REF     ------------------------------------------------------------ 31039

chimp.LOC112206744-LOC107973052-GGT2.revcompl                                    ------------------------------------------------------------ 61762
chimp54546-135457.revcompl.Pan.troglodytes.clone.rp43-41g5.GenBank:AC099533.36   ------------------------------------------------------------ 68417
human.FAM30B-LOC105372935-GGT2.NCBI.                                             cactctgtgaccttaaggctaggtcacaaaaagggaatgcagcttctgccagcaatcctg 80447
human.FAM230E-LOC105377182-GGT3P.NCBI.                                           ------------------------------------------------------------ 57310
human.FAM230J-LOC105372942-GGTLC5P.NCBI.rev.compl                                ------------------------------------------------------------ 46364
human.FAM230A.ncRNA.18487127.18500594-GGTLC3.18516335.18518165.rev.compl.REF     ------------------------------------------------------------ 31039

chimp.LOC112206744-LOC107973052-GGT2.revcompl                                    ------------------------------------------------------------ 61762
chimp54546-135457.revcompl.Pan.troglodytes.clone.rp43-41g5.GenBank:AC099533.36   ------------------------------------------------------------ 68417
human.FAM30B-LOC105372935-GGT2.NCBI.                                             ggacacacacttggagcccgagctgctgtgtaagccatctgcctgctctgagcccaccac 80507
human.FAM230E-LOC105377182-GGT3P.NCBI.                                           ------------------------------------------------------------ 57310
human.FAM230J-LOC105372942-GGTLC5P.NCBI.rev.compl                                ------------------------------------------------------------ 46364
human.FAM230A.ncRNA.18487127.18500594-GGTLC3.18516335.18518165.rev.compl.REF     ------------------------------------------------------------ 31039

chimp.LOC112206744-LOC107973052-GGT2.revcompl                                    ------------------------------------------------------------ 61762
chimp54546-135457.revcompl.Pan.troglodytes.clone.rp43-41g5.GenBank:AC099533.36   ------------------------------------------------------------ 68417
human.FAM30B-LOC105372935-GGT2.NCBI.                                             actataaggaagcccaagctagcccacaggagagacgaggtgaagagaatgagatgccag 80567
human.FAM230E-LOC105377182-GGT3P.NCBI.                                           ------------------------------------------------------------ 57310
human.FAM230J-LOC105372942-GGTLC5P.NCBI.rev.compl                                ------------------------------------------------------------ 46364
human.FAM230A.ncRNA.18487127.18500594-GGTLC3.18516335.18518165.rev.compl.REF     ------------------------------------------------------------ 31039

chimp.LOC112206744-LOC107973052-GGT2.revcompl                                    ------------------------------------------------------------ 61762
chimp54546-135457.revcompl.Pan.troglodytes.clone.rp43-41g5.GenBank:AC099533.36   ------------------------------------------------------------ 68417
human.FAM30B-LOC105372935-GGT2.NCBI.                                             tctccactgctccagctctctagtgtcccagccccagccactatctgactgcaaccccat 80627
human.FAM230E-LOC105377182-GGT3P.NCBI.                                           ------------------------------------------------------------ 57310
human.FAM230J-LOC105372942-GGTLC5P.NCBI.rev.compl                                ------------------------------------------------------------ 46364
human.FAM230A.ncRNA.18487127.18500594-GGTLC3.18516335.18518165.rev.compl.REF     ------------------------------------------------------------ 31039

chimp.LOC112206744-LOC107973052-GGT2.revcompl                                    ------------------------------------------------------------ 61762
chimp54546-135457.revcompl.Pan.troglodytes.clone.rp43-41g5.GenBank:AC099533.36   ------------------------------------------------------------ 68417
human.FAM30B-LOC105372935-GGT2.NCBI.                                             gaaagactgagccagaaccacgagcccagcccttcctgatgttgacatacagacttcatg 80687
human.FAM230E-LOC105377182-GGT3P.NCBI.                                           ------------------------------------------------------------ 57310
human.FAM230J-LOC105372942-GGTLC5P.NCBI.rev.compl                                ------------------------------------------------------------ 46364
human.FAM230A.ncRNA.18487127.18500594-GGTLC3.18516335.18518165.rev.compl.REF     ------------------------------------------------------------ 31039
```

```
chimp.LOC112206744-LOC107973052-GGT2.revcompl                                     ------------------------------------------------------------ 61762
chimp54546-135457.revcompl.Pan.troglodytes.clone.rp43-41g5.GenBank:AC099533.36    ------------------------------------------------------------ 68417
human.FAM30B-LOC105372935-GGT2.NCBI.                                              aaagagaataaactaattttgctttaagccactatgatttgggatgatttgttatgcag 80747
human.FAM230E-LOC105377182-GGT3P.NCBI.                                            ------------------------------------------------------------ 57310
human.FAM230J-LOC105372942-GGTLC5P.NCBI.rev.compl                                 ------------------------------------------------------------ 46364
human.FAM230A.ncRNA.18487127.18500594-GGTLC3.18516335.18518165.rev.compl.REF      ------------------------------------------------------------ 31039

chimp.LOC112206744-LOC107973052-GGT2.revcompl                                     ------------------------------------------------------------ 61762
chimp54546-135457.revcompl.Pan.troglodytes.clone.rp43-41g5.GenBank:AC099533.36    ------------------------------------------------------------ 68417
human.FAM30B-LOC105372935-GGT2.NCBI.                                              cactggataactgcaacacctgcttctaaacagtagaatcccaaacgtctaggccccatc 80807
human.FAM230E-LOC105377182-GGT3P.NCBI.                                            ------------------------------------------------------------ 57310
human.FAM230J-LOC105372942-GGTLC5P.NCBI.rev.compl                                 ------------------------------------------------------------ 46364
human.FAM230A.ncRNA.18487127.18500594-GGTLC3.18516335.18518165.rev.compl.REF      ------------------------------------------------------------ 31039

chimp.LOC112206744-LOC107973052-GGT2.revcompl                                     ------------------------------------------------------------ 61762
chimp54546-135457.revcompl.Pan.troglodytes.clone.rp43-41g5.GenBank:AC099533.36    ------------------------------------------------------------ 68417
human.FAM30B-LOC105372935-GGT2.NCBI.                                              tctaacttacttcatgttcctgttctcactgatttttgccactgagcttgaccaagccc 80867
human.FAM230E-LOC105377182-GGT3P.NCBI.                                            ------------------------------------------------------------ 57310
human.FAM230J-LOC105372942-GGTLC5P.NCBI.rev.compl                                 ------------------------------------------------------------ 46364
human.FAM230A.ncRNA.18487127.18500594-GGTLC3.18516335.18518165.rev.compl.REF      ------------------------------------------------------------ 31039

chimp.LOC112206744-LOC107973052-GGT2.revcompl                                     ------------------------------------------------------------ 61762
chimp54546-135457.revcompl.Pan.troglodytes.clone.rp43-41g5.GenBank:AC099533.36    ------------------------------------------------------------ 68417
human.FAM30B-LOC105372935-GGT2.NCBI.                                              ttgagtcaccaattccagatatttgtttaaacaaattcactgtgactgtctacactatcc 80927
human.FAM230E-LOC105377182-GGT3P.NCBI.                                            ------------------------------------------------------------ 57310
human.FAM230J-LOC105372942-GGTLC5P.NCBI.rev.compl                                 ------------------------------------------------------------ 46364
human.FAM230A.ncRNA.18487127.18500594-GGTLC3.18516335.18518165.rev.compl.REF      ------------------------------------------------------------ 31039

chimp.LOC112206744-LOC107973052-GGT2.revcompl                                     ------------------------------------------------------------ 61762
chimp54546-135457.revcompl.Pan.troglodytes.clone.rp43-41g5.GenBank:AC099533.36    ------------------------------------------------------------ 68417
human.FAM30B-LOC105372935-GGT2.NCBI.                                              attcctccaaaacaatccctgttatagttggccttccccagttctcttcctggcatagag 80987
human.FAM230E-LOC105377182-GGT3P.NCBI.                                            ------------------------------------------------------------ 57310
human.FAM230J-LOC105372942-GGTLC5P.NCBI.rev.compl                                 ------------------------------------------------------------ 46364
human.FAM230A.ncRNA.18487127.18500594-GGTLC3.18516335.18518165.rev.compl.REF      ------------------------------------------------------------ 31039

chimp.LOC112206744-LOC107973052-GGT2.revcompl                                     ------------------------------------------------------------ 61762
chimp54546-135457.revcompl.Pan.troglodytes.clone.rp43-41g5.GenBank:AC099533.36    ------------------------------------------------------------ 68417
human.FAM30B-LOC105372935-GGT2.NCBI.                                              tagtctgtgcccttctctcatttcttaatatgaccctcacccatggagtgttcctagatt 81047
human.FAM230E-LOC105377182-GGT3P.NCBI.                                            ------------------------------------------------------------ 57310
human.FAM230J-LOC105372942-GGTLC5P.NCBI.rev.compl                                 ------------------------------------------------------------ 46364
human.FAM230A.ncRNA.18487127.18500594-GGTLC3.18516335.18518165.rev.compl.REF      ------------------------------------------------------------ 31039

chimp.LOC112206744-LOC107973052-GGT2.revcompl                                     ------------------------------------------------------------ 61762
chimp54546-135457.revcompl.Pan.troglodytes.clone.rp43-41g5.GenBank:AC099533.36    ------------------------------------------------------------ 68417
human.FAM30B-LOC105372935-GGT2.NCBI.                                              ctgctcattcataatgcttcttcacccaccctaacctctccctcctgtagccacagatca 81107
human.FAM230E-LOC105377182-GGT3P.NCBI.                                            ------------------------------------------------------------ 57310
human.FAM230J-LOC105372942-GGTLC5P.NCBI.rev.compl                                 ------------------------------------------------------------ 46364
human.FAM230A.ncRNA.18487127.18500594-GGTLC3.18516335.18518165.rev.compl.REF      ------------------------------------------------------------ 31039

chimp.LOC112206744-LOC107973052-GGT2.revcompl                                     ------------------------------------------------------------ 61762
chimp54546-135457.revcompl.Pan.troglodytes.clone.rp43-41g5.GenBank:AC099533.36    ------------------------------------------------------------ 68417
human.FAM30B-LOC105372935-GGT2.NCBI.                                              tccacttttccaggcctctgcactggaggctgcatcagccacctcccagtacgtcatcct 81167
human.FAM230E-LOC105377182-GGT3P.NCBI.                                            ------------------------------------------------------------ 57310
human.FAM230J-LOC105372942-GGTLC5P.NCBI.rev.compl                                 ------------------------------------------------------------ 46364
human.FAM230A.ncRNA.18487127.18500594-GGTLC3.18516335.18518165.rev.compl.REF      ------------------------------------------------------------ 31039

chimp.LOC112206744-LOC107973052-GGT2.revcompl                                     ------------------------------------------------------------ 61762
chimp54546-135457.revcompl.Pan.troglodytes.clone.rp43-41g5.GenBank:AC099533.36    ------------------------------------------------------------ 68417
human.FAM30B-LOC105372935-GGT2.NCBI.                                              ttccaatgccctacaacacggggcagaaaggactctttttgctgcacatccaggctgtt 81227
human.FAM230E-LOC105377182-GGT3P.NCBI.                                            ------------------------------------------------------------ 57310
human.FAM230J-LOC105372942-GGTLC5P.NCBI.rev.compl                                 ------------------------------------------------------------ 46364
human.FAM230A.ncRNA.18487127.18500594-GGTLC3.18516335.18518165.rev.compl.REF      ------------------------------------------------------------ 31039

chimp.LOC112206744-LOC107973052-GGT2.revcompl                                     ------------------------------------------------------------ 61762
chimp54546-135457.revcompl.Pan.troglodytes.clone.rp43-41g5.GenBank:AC099533.36    ------------------------------------------------------------ 68417
human.FAM30B-LOC105372935-GGT2.NCBI.                                              taagaaaaggcttaaggcagcttaagcacaaagaatgtcatcttcaaacattgtaacggc 81287
human.FAM230E-LOC105377182-GGT3P.NCBI.                                            ------------------------------------------------------------ 57310
human.FAM230J-LOC105372942-GGTLC5P.NCBI.rev.compl                                 ------------------------------------------------------------ 46364
human.FAM230A.ncRNA.18487127.18500594-GGTLC3.18516335.18518165.rev.compl.REF      ------------------------------------------------------------ 31039

chimp.LOC112206744-LOC107973052-GGT2.revcompl                                     ------------------------------------------------------------ 61762
chimp54546-135457.revcompl.Pan.troglodytes.clone.rp43-41g5.GenBank:AC099533.36    ------------------------------------------------------------ 68417
human.FAM30B-LOC105372935-GGT2.NCBI.                                              atcttctttttggggcactgaagtattcccatgattccaagaatcaaatgaacagcatct 81347
human.FAM230E-LOC105377182-GGT3P.NCBI.                                            ------------------------------------------------------------ 57310
human.FAM230J-LOC105372942-GGTLC5P.NCBI.rev.compl                                 ------------------------------------------------------------ 46364
human.FAM230A.ncRNA.18487127.18500594-GGTLC3.18516335.18518165.rev.compl.REF      ------------------------------------------------------------ 31039

chimp.LOC112206744-LOC107973052-GGT2.revcompl                                     ------------------------------------------------------------ 61762
chimp54546-135457.revcompl.Pan.troglodytes.clone.rp43-41g5.GenBank:AC099533.36    ------------------------------------------------------------ 68417
human.FAM30B-LOC105372935-GGT2.NCBI.                                              ggttatggggtctctggtctctgccaaggcaaactcataaagcatttcagagcccttcat 81407
human.FAM230E-LOC105377182-GGT3P.NCBI.                                            ------------------------------------------------------------ 57310
human.FAM230J-LOC105372942-GGTLC5P.NCBI.rev.compl                                 ------------------------------------------------------------ 46364
human.FAM230A.ncRNA.18487127.18500594-GGTLC3.18516335.18518165.rev.compl.REF      ------------------------------------------------------------ 31039

chimp.LOC112206744-LOC107973052-GGT2.revcompl                                     ------------------------------------------------------------ 61762
chimp54546-135457.revcompl.Pan.troglodytes.clone.rp43-41g5.GenBank:AC099533.36    ------------------------------------------------------------ 68417
human.FAM30B-LOC105372935-GGT2.NCBI.                                              ctggaaaggccttttctccaccccacgcccagccaatgcccactcatactttaaggccta 81467
human.FAM230E-LOC105377182-GGT3P.NCBI.                                            ------------------------------------------------------------ 57310
human.FAM230J-LOC105372942-GGTLC5P.NCBI.rev.compl                                 ------------------------------------------------------------ 46364
human.FAM230A.ncRNA.18487127.18500594-GGTLC3.18516335.18518165.rev.compl.REF      ------------------------------------------------------------ 31039

chimp.LOC112206744-LOC107973052-GGT2.revcompl                                     ------------------------------------------------------------ 61762
chimp54546-135457.revcompl.Pan.troglodytes.clone.rp43-41g5.GenBank:AC099533.36    ------------------------------------------------------------ 68417
human.FAM30B-LOC105372935-GGT2.NCBI.                                              gctcatccgtcatctctgctagggaaccttcctcagtgccttatgcaggcaaccattcct 81527
human.FAM230E-LOC105377182-GGT3P.NCBI.                                            ------------------------------------------------------------ 57310
human.FAM230J-LOC105372942-GGTLC5P.NCBI.rev.compl                                 ------------------------------------------------------------ 46364
human.FAM230A.ncRNA.18487127.18500594-GGTLC3.18516335.18518165.rev.compl.REF      ------------------------------------------------------------ 31039

chimp.LOC112206744-LOC107973052-GGT2.revcompl                                     ------------------------------------------------------------ 61762
chimp54546-135457.revcompl.Pan.troglodytes.clone.rp43-41g5.GenBank:AC099533.36    ------------------------------------------------------------ 68417
human.FAM30B-LOC105372935-GGT2.NCBI.                                              gccttgtgttgtgttgtgttgtgttgtgccttaagtcaaatccttaactcatctttgtgt 81587
human.FAM230E-LOC105377182-GGT3P.NCBI.                                            ------------------------------------------------------------ 57310
human.FAM230J-LOC105372942-GGTLC5P.NCBI.rev.compl                                 ------------------------------------------------------------ 46364
human.FAM230A.ncRNA.18487127.18500594-GGTLC3.18516335.18518165.rev.compl.REF      ------------------------------------------------------------ 31039

chimp.LOC112206744-LOC107973052-GGT2.revcompl                                     ------------------------------------------------------------ 61762
chimp54546-135457.revcompl.Pan.troglodytes.clone.rp43-41g5.GenBank:AC099533.36    ------------------------------------------------------------ 68417
human.FAM30B-LOC105372935-GGT2.NCBI.                                              gtccccttgcatttcagacagtaggtgcccaataaatgtttgtgggatgaatcaactggt 81647
human.FAM230E-LOC105377182-GGT3P.NCBI.                                            ------------------------------------------------------------ 57310
human.FAM230J-LOC105372942-GGTLC5P.NCBI.rev.compl                                 ------------------------------------------------------------ 46364
human.FAM230A.ncRNA.18487127.18500594-GGTLC3.18516335.18518165.rev.compl.REF      ------------------------------------------------------------ 31039

chimp.LOC112206744-LOC107973052-GGT2.revcompl                                     ------------------------------------------------------------ 61762
chimp54546-135457.revcompl.Pan.troglodytes.clone.rp43-41g5.GenBank:AC099533.36    ------------------------------------------------------------ 68417
human.FAM30B-LOC105372935-GGT2.NCBI.                                              tattctcctggaacaccctttcccagtacacctccactcgccagaacaactgtcttcctc 81707
human.FAM230E-LOC105377182-GGT3P.NCBI.                                            ------------------------------------------------------------ 57310
human.FAM230J-LOC105372942-GGTLC5P.NCBI.rev.compl                                 ------------------------------------------------------------ 46364
human.FAM230A.ncRNA.18487127.18500594-GGTLC3.18516335.18518165.rev.compl.REF      ------------------------------------------------------------ 31039

chimp.LOC112206744-LOC107973052-GGT2.revcompl                                     ------------------------------------------------------------ 61762
chimp54546-135457.revcompl.Pan.troglodytes.clone.rp43-41g5.GenBank:AC099533.36    ------------------------------------------------------------ 68417
human.FAM30B-LOC105372935-GGT2.NCBI.                                              acaggtgccagccatcaccccgaccacaccctctcaaaaggcctttgccagtcatcaaac 81767
human.FAM230E-LOC105377182-GGT3P.NCBI.                                            ------------------------------------------------------------ 57310
human.FAM230J-LOC105372942-GGTLC5P.NCBI.rev.compl                                 ------------------------------------------------------------ 46364
human.FAM230A.ncRNA.18487127.18500594-GGTLC3.18516335.18518165.rev.compl.REF      ------------------------------------------------------------ 31039

chimp.LOC112206744-LOC107973052-GGT2.revcompl                                     ------------------------------------------------------------ 61762
```

```
chimp54546-135457.revcompl.Pan.troglodytes.clone.rp43-41g5.GenBank:AC099533.36      ------------------------------------------------------------ 68417
human.FAM30B-LOC105372935-GGT2.NCBI.                                                  aaagcctcttgtctcctgagagccgctccacaatcaccttcccaagaagaattccccata 81827
human.FAM230E-LOC105377182-GGT3P.NCBI.                                                ------------------------------------------------------------ 57310
human.FAM230J-LOC105372942-GGTLC5P.NCBI.rev.compl                                     ------------------------------------------------------------ 46364
human.FAM230A.ncRNA.18487127.18500594-GGTLC3.18516335.18518165.rev.compl.REF          ------------------------------------------------------------ 31039

chimp.LOC112206744-LOC107973052-GGT2.revcompl                                         ------------------------------------------------------------ 61762
chimp54546-135457.revcompl.Pan.troglodytes.clone.rp43-41g5.GenBank:AC099533.36      ------------------------------------------------------------ 68417
human.FAM30B-LOC105372935-GGT2.NCBI.                                                  ccgagtccaccattccttccctgatcaaagttgctttcacaggcatcttttcagctatcc 81887
human.FAM230E-LOC105377182-GGT3P.NCBI.                                                ------------------------------------------------------------ 57310
human.FAM230J-LOC105372942-GGTLC5P.NCBI.rev.compl                                     ------------------------------------------------------------ 46364
human.FAM230A.ncRNA.18487127.18500594-GGTLC3.18516335.18518165.rev.compl.REF          ------------------------------------------------------------ 31039

chimp.LOC112206744-LOC107973052-GGT2.revcompl                                         ------------------------------------------------------------ 61762
chimp54546-135457.revcompl.Pan.troglodytes.clone.rp43-41g5.GenBank:AC099533.36      ------------------------------------------------------------ 68417
human.FAM30B-LOC105372935-GGT2.NCBI.                                                  actcttcacggcttggttttcatgcactctgagttggccctatctccccttttggcctc 81947
human.FAM230E-LOC105377182-GGT3P.NCBI.                                                ------------------------------------------------------------ 57310
human.FAM230J-LOC105372942-GGTLC5P.NCBI.rev.compl                                     ------------------------------------------------------------ 46364
human.FAM230A.ncRNA.18487127.18500594-GGTLC3.18516335.18518165.rev.compl.REF          ------------------------------------------------------------ 31039

chimp.LOC112206744-LOC107973052-GGT2.revcompl                                         ------------------------------------------------------------ 61762
chimp54546-135457.revcompl.Pan.troglodytes.clone.rp43-41g5.GenBank:AC099533.36      ------------------------------------------------------------ 68417
human.FAM30B-LOC105372935-GGT2.NCBI.                                                  aagcacgtcaagaggaaggctggtgacagcctttcccgagctcaggacagttttcatgca 82007
human.FAM230E-LOC105377182-GGT3P.NCBI.                                                ------------------------------------------------------------ 57310
human.FAM230J-LOC105372942-GGTLC5P.NCBI.rev.compl                                     ------------------------------------------------------------ 46364
human.FAM230A.ncRNA.18487127.18500594-GGTLC3.18516335.18518165.rev.compl.REF          ------------------------------------------------------------ 31039

chimp.LOC112206744-LOC107973052-GGT2.revcompl                                         ------------------------------------------------------------ 61762
chimp54546-135457.revcompl.Pan.troglodytes.clone.rp43-41g5.GenBank:AC099533.36      ------------------------------------------------------------ 68417
human.FAM30B-LOC105372935-GGT2.NCBI.                                                  aactggttcttccaggttcttatgcctggaagaactgctgctctccaatagcatgaagta 82067
human.FAM230E-LOC105377182-GGT3P.NCBI.                                                ------------------------------------------------------------ 57310
human.FAM230J-LOC105372942-GGTLC5P.NCBI.rev.compl                                     ------------------------------------------------------------ 46364
human.FAM230A.ncRNA.18487127.18500594-GGTLC3.18516335.18518165.rev.compl.REF          ------------------------------------------------------------ 31039

chimp.LOC112206744-LOC107973052-GGT2.revcompl                                         ------------------------------------------------------------ 61762
chimp54546-135457.revcompl.Pan.troglodytes.clone.rp43-41g5.GenBank:AC099533.36      ------------------------------------------------------------ 68417
human.FAM30B-LOC105372935-GGT2.NCBI.                                                  gacaatgatgttcttagcatccagtcttaaaactagaaaaagtattggcactgctggatc 82127
human.FAM230E-LOC105377182-GGT3P.NCBI.                                                ------------------------------------------------------------ 57310
human.FAM230J-LOC105372942-GGTLC5P.NCBI.rev.compl                                     ------------------------------------------------------------ 46364
human.FAM230A.ncRNA.18487127.18500594-GGTLC3.18516335.18518165.rev.compl.REF          ------------------------------------------------------------ 31039

chimp.LOC112206744-LOC107973052-GGT2.revcompl                                         ------------------------------------------------------------ 61762
chimp54546-135457.revcompl.Pan.troglodytes.clone.rp43-41g5.GenBank:AC099533.36      ------------------------------------------------------------ 68417
human.FAM30B-LOC105372935-GGT2.NCBI.                                                  cccagctcctagaatggtgtgtggcacctaaacagagctcaaaaaatattagttgaatgg 82187
human.FAM230E-LOC105377182-GGT3P.NCBI.                                                ------------------------------------------------------------ 57310
human.FAM230J-LOC105372942-GGTLC5P.NCBI.rev.compl                                     ------------------------------------------------------------ 46364
human.FAM230A.ncRNA.18487127.18500594-GGTLC3.18516335.18518165.rev.compl.REF          ------------------------------------------------------------ 31039

chimp.LOC112206744-LOC107973052-GGT2.revcompl                                         ------------------------------------------------------------ 61762
chimp54546-135457.revcompl.Pan.troglodytes.clone.rp43-41g5.GenBank:AC099533.36      ------------------------------------------------------------ 68417
human.FAM30B-LOC105372935-GGT2.NCBI.                                                  aggaaagaatgaacagtcatctggcccagacttggagaagctgtgatctgtctagccctt 82247
human.FAM230E-LOC105377182-GGT3P.NCBI.                                                ------------------------------------------------------------ 57310
human.FAM230J-LOC105372942-GGTLC5P.NCBI.rev.compl                                     ------------------------------------------------------------ 46364
human.FAM230A.ncRNA.18487127.18500594-GGTLC3.18516335.18518165.rev.compl.REF          ------------------------------------------------------------ 31039

chimp.LOC112206744-LOC107973052-GGT2.revcompl                                         ------------------------------------------------------------ 61762
chimp54546-135457.revcompl.Pan.troglodytes.clone.rp43-41g5.GenBank:AC099533.36      ------------------------------------------------------------ 68417
human.FAM30B-LOC105372935-GGT2.NCBI.                                                  ctacaccaggagcgaggcctgaaaagctgcatgactcacccaaggtcacaagcaagccag 82307
human.FAM230E-LOC105377182-GGT3P.NCBI.                                                ------------------------------------------------------------ 57310
human.FAM230J-LOC105372942-GGTLC5P.NCBI.rev.compl                                     ------------------------------------------------------------ 46364
human.FAM230A.ncRNA.18487127.18500594-GGTLC3.18516335.18518165.rev.compl.REF          ------------------------------------------------------------ 31039

chimp.LOC112206744-LOC107973052-GGT2.revcompl                                         ------------------------------------------------------------ 61762
chimp54546-135457.revcompl.Pan.troglodytes.clone.rp43-41g5.GenBank:AC099533.36      ------------------------------------------------------------ 68417
human.FAM30B-LOC105372935-GGT2.NCBI.                                                  tgaagaggggctggaaccctgtcttttgcctcaaagcacagggccctccttctgtcccac 82367
human.FAM230E-LOC105377182-GGT3P.NCBI.                                                ------------------------------------------------------------ 57310
human.FAM230J-LOC105372942-GGTLC5P.NCBI.rev.compl                                     ------------------------------------------------------------ 46364
human.FAM230A.ncRNA.18487127.18500594-GGTLC3.18516335.18518165.rev.compl.REF          ------------------------------------------------------------ 31039

chimp.LOC112206744-LOC107973052-GGT2.revcompl                                         ------------------------------------------------------------ 61762
chimp54546-135457.revcompl.Pan.troglodytes.clone.rp43-41g5.GenBank:AC099533.36      ------------------------------------------------------------ 68417
human.FAM30B-LOC105372935-GGT2.NCBI.                                                  ccagctttctcaccacccaccccaccccttccacactccaaagacagcatctccgtgaga 82427
human.FAM230E-LOC105377182-GGT3P.NCBI.                                                ------------------------------------------------------------ 57310
human.FAM230J-LOC105372942-GGTLC5P.NCBI.rev.compl                                     ------------------------------------------------------------ 46364
human.FAM230A.ncRNA.18487127.18500594-GGTLC3.18516335.18518165.rev.compl.REF          ------------------------------------------------------------ 31039

chimp.LOC112206744-LOC107973052-GGT2.revcompl                                         ------------------------------------------------------------ 61762
chimp54546-135457.revcompl.Pan.troglodytes.clone.rp43-41g5.GenBank:AC099533.36      ------------------------------------------------------------ 68417
human.FAM30B-LOC105372935-GGT2.NCBI.                                                  agtgttgggcagtgtccaggagtgtttttcttcctggctttaaaacaaattgtgacaaat 82487
human.FAM230E-LOC105377182-GGT3P.NCBI.                                                ------------------------------------------------------------ 57310
human.FAM230J-LOC105372942-GGTLC5P.NCBI.rev.compl                                     ------------------------------------------------------------ 46364
human.FAM230A.ncRNA.18487127.18500594-GGTLC3.18516335.18518165.rev.compl.REF          ------------------------------------------------------------ 31039

chimp.LOC112206744-LOC107973052-GGT2.revcompl                                         ------------------------------------------------------------ 61762
chimp54546-135457.revcompl.Pan.troglodytes.clone.rp43-41g5.GenBank:AC099533.36      ------------------------------------------------------------ 68417
human.FAM30B-LOC105372935-GGT2.NCBI.                                                  ggctccacagatctaccgggccatggagctgagcagatgggactgcaattcacgctatta 82547
human.FAM230E-LOC105377182-GGT3P.NCBI.                                                ------------------------------------------------------------ 57310
human.FAM230J-LOC105372942-GGTLC5P.NCBI.rev.compl                                     ------------------------------------------------------------ 46364
human.FAM230A.ncRNA.18487127.18500594-GGTLC3.18516335.18518165.rev.compl.REF          ------------------------------------------------------------ 31039

chimp.LOC112206744-LOC107973052-GGT2.revcompl                                         ------------------------------------------------------------ 61762
chimp54546-135457.revcompl.Pan.troglodytes.clone.rp43-41g5.GenBank:AC099533.36      ------------------------------------------------------------ 68417
human.FAM30B-LOC105372935-GGT2.NCBI.                                                  actcaaattctacttgactttctttcaaatcagggcattttatctagaaattataaaaaa 82607
human.FAM230E-LOC105377182-GGT3P.NCBI.                                                ------------------------------------------------------------ 57310
human.FAM230J-LOC105372942-GGTLC5P.NCBI.rev.compl                                     ------------------------------------------------------------ 46364
human.FAM230A.ncRNA.18487127.18500594-GGTLC3.18516335.18518165.rev.compl.REF          ------------------------------------------------------------ 31039

chimp.LOC112206744-LOC107973052-GGT2.revcompl                                         ------------------------------------------------------------ 61762
chimp54546-135457.revcompl.Pan.troglodytes.clone.rp43-41g5.GenBank:AC099533.36      ------------------------------------------------------------ 68417
human.FAM30B-LOC105372935-GGT2.NCBI.                                                  ttgttggggggagtgcctttgtaaggaagtttgccagcacattcatttacaaatcgacttg 82667
human.FAM230E-LOC105377182-GGT3P.NCBI.                                                ------------------------------------------------------------ 57310
human.FAM230J-LOC105372942-GGTLC5P.NCBI.rev.compl                                     ------------------------------------------------------------ 46364
human.FAM230A.ncRNA.18487127.18500594-GGTLC3.18516335.18518165.rev.compl.REF          ------------------------------------------------------------ 31039

chimp.LOC112206744-LOC107973052-GGT2.revcompl                                         ------------------------------------------------------------ 61762
chimp54546-135457.revcompl.Pan.troglodytes.clone.rp43-41g5.GenBank:AC099533.36      ------------------------------------------------------------ 68417
human.FAM30B-LOC105372935-GGT2.NCBI.                                                  ctcctggccaccctcactgatcacggacttttactcacgctcccttactctctcccatca 82727
human.FAM230E-LOC105377182-GGT3P.NCBI.                                                ------------------------------------------------------------ 57310
human.FAM230J-LOC105372942-GGTLC5P.NCBI.rev.compl                                     ------------------------------------------------------------ 46364
human.FAM230A.ncRNA.18487127.18500594-GGTLC3.18516335.18518165.rev.compl.REF          ------------------------------------------------------------ 31039

chimp.LOC112206744-LOC107973052-GGT2.revcompl                                         ------------------------------------------------------------ 61762
chimp54546-135457.revcompl.Pan.troglodytes.clone.rp43-41g5.GenBank:AC099533.36      ------------------------------------------------------------ 68417
human.FAM30B-LOC105372935-GGT2.NCBI.                                                  aagagtccagcgcatggagagagtgatggaatcttgaggcccaccggtctaaccacccac 82787
human.FAM230E-LOC105377182-GGT3P.NCBI.                                                ------------------------------------------------------------ 57310
human.FAM230J-LOC105372942-GGTLC5P.NCBI.rev.compl                                     ------------------------------------------------------------ 46364
human.FAM230A.ncRNA.18487127.18500594-GGTLC3.18516335.18518165.rev.compl.REF          ------------------------------------------------------------ 31039

chimp.LOC112206744-LOC107973052-GGT2.revcompl                                         ------------------------------------------------------------ 61762
chimp54546-135457.revcompl.Pan.troglodytes.clone.rp43-41g5.GenBank:AC099533.36      ------------------------------------------------------------ 68417
human.FAM30B-LOC105372935-GGT2.NCBI.                                                  ctaagcttgactcccctgtgcctgtggacatcctgcgtgcagatgcacattcccactaat 82847
human.FAM230E-LOC105377182-GGT3P.NCBI.                                                ------------------------------------------------------------ 57310
human.FAM230J-LOC105372942-GGTLC5P.NCBI.rev.compl                                     ------------------------------------------------------------ 46364
human.FAM230A.ncRNA.18487127.18500594-GGTLC3.18516335.18518165.rev.compl.REF          ------------------------------------------------------------ 31039

chimp.LOC112206744-LOC107973052-GGT2.revcompl                                         ------------------------------------------------------------ 61762
chimp54546-135457.revcompl.Pan.troglodytes.clone.rp43-41g5.GenBank:AC099533.36      ------------------------------------------------------------ 68417
```

```
human.FAM30B-LOC105372935-GGT2.NCBI.    ggcagccaggggaaagcactattcgaaagttcttctttactttgaactcaaattgggctc 82907
human.FAM230E-LOC105377182-GGT3P.NCBI.    ------------------------------------------------------------ 57310
human.FAM230J-LOC105372942-GGTLC5P.NCBI.rev.compl    ------------------------------------------------------------ 46364
human.FAM230A.ncRNA.18487127.18500594-GGTLC3.18516335.18518165.rev.compl.REF    ------------------------------------------------------------ 31039

chimp.LOC112206744-LOC107973052-GGT2.revcompl    ------------------------------------------------------------ 61762
chimp54546-135457.revcompl.Pan.troglodytes.clone.rp43-41g5.GenBank:AC099533.36    ------------------------------------------------------------ 68417
human.FAM30B-LOC105372935-GGT2.NCBI.    aggggtcctggatacaggacaaccccaatctcttatttattcaatggacaacaggtgagc 82967
human.FAM230E-LOC105377182-GGT3P.NCBI.    ------------------------------------------------------------ 57310
human.FAM230J-LOC105372942-GGTLC5P.NCBI.rev.compl    ------------------------------------------------------------ 46364
human.FAM230A.ncRNA.18487127.18500594-GGTLC3.18516335.18518165.rev.compl.REF    ------------------------------------------------------------ 31039

chimp.LOC112206744-LOC107973052-GGT2.revcompl    ------------------------------------------------------------ 61762
chimp54546-135457.revcompl.Pan.troglodytes.clone.rp43-41g5.GenBank:AC099533.36    ------------------------------------------------------------ 68417
human.FAM30B-LOC105372935-GGT2.NCBI.    acatactaggcctggacactgatgtaggtaggcactaaagcttcagagagatgcaaatga 83027
human.FAM230E-LOC105377182-GGT3P.NCBI.    ------------------------------------------------------------ 57310
human.FAM230J-LOC105372942-GGTLC5P.NCBI.rev.compl    ------------------------------------------------------------ 46364
human.FAM230A.ncRNA.18487127.18500594-GGTLC3.18516335.18518165.rev.compl.REF    ------------------------------------------------------------ 31039

chimp.LOC112206744-LOC107973052-GGT2.revcompl    ------------------------------------------------------------ 61762
chimp54546-135457.revcompl.Pan.troglodytes.clone.rp43-41g5.GenBank:AC099533.36    ------------------------------------------------------------ 68417
human.FAM30B-LOC105372935-GGT2.NCBI.    acaatctttaccctgaaggagctcatgttctatgggttcatttcctttccctgtgtctta 83087
human.FAM230E-LOC105377182-GGT3P.NCBI.    ------------------------------------------------------------ 57310
human.FAM230J-LOC105372942-GGTLC5P.NCBI.rev.compl    ------------------------------------------------------------ 46364
human.FAM230A.ncRNA.18487127.18500594-GGTLC3.18516335.18518165.rev.compl.REF    ------------------------------------------------------------ 31039

chimp.LOC112206744-LOC107973052-GGT2.revcompl    ------------------------------------------------------------ 61762
chimp54546-135457.revcompl.Pan.troglodytes.clone.rp43-41g5.GenBank:AC099533.36    ------------------------------------------------------------ 68417
human.FAM30B-LOC105372935-GGT2.NCBI.    caaacaggttccagtttgtttccgtccttcagagtgtactgcctagaactgcatgctata 83147
human.FAM230E-LOC105377182-GGT3P.NCBI.    ------------------------------------------------------------ 57310
human.FAM230J-LOC105372942-GGTLC5P.NCBI.rev.compl    ------------------------------------------------------------ 46364
human.FAM230A.ncRNA.18487127.18500594-GGTLC3.18516335.18518165.rev.compl.REF    ------------------------------------------------------------ 31039

chimp.LOC112206744-LOC107973052-GGT2.revcompl    ------------------------------------------------------------ 61762
chimp54546-135457.revcompl.Pan.troglodytes.clone.rp43-41g5.GenBank:AC099533.36    ------------------------------------------------------------ 68417
human.FAM30B-LOC105372935-GGT2.NCBI.    ctccacaagtggcctgaccagcttgtggacaaggatgaagcaagcagaccaccttctttc 83207
human.FAM230E-LOC105377182-GGT3P.NCBI.    ------------------------------------------------------------ 57310
human.FAM230J-LOC105372942-GGTLC5P.NCBI.rev.compl    ------------------------------------------------------------ 46364
human.FAM230A.ncRNA.18487127.18500594-GGTLC3.18516335.18518165.rev.compl.REF    ------------------------------------------------------------ 31039

chimp.LOC112206744-LOC107973052-GGT2.revcompl    ------------------------------------------------------------ 61762
chimp54546-135457.revcompl.Pan.troglodytes.clone.rp43-41g5.GenBank:AC099533.36    ------------------------------------------------------------ 68417
human.FAM30B-LOC105372935-GGT2.NCBI.    agtgcagacaagatggctgtttagaggccacatcacgtggcagacacctgccaactaaaa 83267
human.FAM230E-LOC105377182-GGT3P.NCBI.    ------------------------------------------------------------ 57310
human.FAM230J-LOC105372942-GGTLC5P.NCBI.rev.compl    ------------------------------------------------------------ 46364
human.FAM230A.ncRNA.18487127.18500594-GGTLC3.18516335.18518165.rev.compl.REF    ------------------------------------------------------------ 31039

chimp.LOC112206744-LOC107973052-GGT2.revcompl    ------------------------------------------------------------ 61762
chimp54546-135457.revcompl.Pan.troglodytes.clone.rp43-41g5.GenBank:AC099533.36    ------------------------------------------------------------ 68417
human.FAM30B-LOC105372935-GGT2.NCBI.    ccctccatccttcccacagcctcccctcctacactttgcagctggattcaaaccttgc 83327
human.FAM230E-LOC105377182-GGT3P.NCBI.    ------------------------------------------------------------ 57310
human.FAM230J-LOC105372942-GGTLC5P.NCBI.rev.compl    ------------------------------------------------------------ 46364
human.FAM230A.ncRNA.18487127.18500594-GGTLC3.18516335.18518165.rev.compl.REF    ------------------------------------------------------------ 31039

chimp.LOC112206744-LOC107973052-GGT2.revcompl    ------------------------------------------------------------ 61762
chimp54546-135457.revcompl.Pan.troglodytes.clone.rp43-41g5.GenBank:AC099533.36    ------------------------------------------------------------ 68417
human.FAM30B-LOC105372935-GGT2.NCBI.    attcacccttgttccatcaacagggccaggccatcagtctatctcatccggactgttttt 83387
human.FAM230E-LOC105377182-GGT3P.NCBI.    ------------------------------------------------------------ 57310
human.FAM230J-LOC105372942-GGTLC5P.NCBI.rev.compl    ------------------------------------------------------------ 46364
human.FAM230A.ncRNA.18487127.18500594-GGTLC3.18516335.18518165.rev.compl.REF    ------------------------------------------------------------ 31039

chimp.LOC112206744-LOC107973052-GGT2.revcompl    ------------------------------------------------------------ 61762
chimp54546-135457.revcompl.Pan.troglodytes.clone.rp43-41g5.GenBank:AC099533.36    ------------------------------------------------------------ 68417
human.FAM30B-LOC105372935-GGT2.NCBI.    tatcttacttgccaacctacacacttgctgttccttcttgccttaaagtcatacagatga 83447
human.FAM230E-LOC105377182-GGT3P.NCBI.    ------------------------------------------------------------ 57310
human.FAM230J-LOC105372942-GGTLC5P.NCBI.rev.compl    ------------------------------------------------------------ 46364
human.FAM230A.ncRNA.18487127.18500594-GGTLC3.18516335.18518165.rev.compl.REF    ------------------------------------------------------------ 31039

chimp.LOC112206744-LOC107973052-GGT2.revcompl    ------------------------------------------------------------ 61762
chimp54546-135457.revcompl.Pan.troglodytes.clone.rp43-41g5.GenBank:AC099533.36    ------------------------------------------------------------ 68417
human.FAM30B-LOC105372935-GGT2.NCBI.    ctttaggtgattcagatgagtgtgacctctactttcaccaaacacaatagtcaattcaga 83507
human.FAM230E-LOC105377182-GGT3P.NCBI.    ------------------------------------------------------------ 57310
human.FAM230J-LOC105372942-GGTLC5P.NCBI.rev.compl    ------------------------------------------------------------ 46364
human.FAM230A.ncRNA.18487127.18500594-GGTLC3.18516335.18518165.rev.compl.REF    ------------------------------------------------------------ 31039

chimp.LOC112206744-LOC107973052-GGT2.revcompl    ------------------------------------------------------------ 61762
chimp54546-135457.revcompl.Pan.troglodytes.clone.rp43-41g5.GenBank:AC099533.36    ------------------------------------------------------------ 68417
human.FAM30B-LOC105372935-GGT2.NCBI.    aaggtcaaggacagaaccctccagcatgctaacgcagccaaagaagtccgaaaacaatac 83567
human.FAM230E-LOC105377182-GGT3P.NCBI.    ------------------------------------------------------------ 57310
human.FAM230J-LOC105372942-GGTLC5P.NCBI.rev.compl    ------------------------------------------------------------ 46364
human.FAM230A.ncRNA.18487127.18500594-GGTLC3.18516335.18518165.rev.compl.REF    ------------------------------------------------------------ 31039

chimp.LOC112206744-LOC107973052-GGT2.revcompl    ------------------------------------------------------------ 61762
chimp54546-135457.revcompl.Pan.troglodytes.clone.rp43-41g5.GenBank:AC099533.36    ------------------------------------------------------------ 68417
human.FAM30B-LOC105372935-GGT2.NCBI.    ttggccacactgttttcctgcttacggatgtggactgctcttgtcctgcctgtaaatata 83627
human.FAM230E-LOC105377182-GGT3P.NCBI.    ------------------------------------------------------------ 57310
human.FAM230J-LOC105372942-GGTLC5P.NCBI.rev.compl    ------------------------------------------------------------ 46364
human.FAM230A.ncRNA.18487127.18500594-GGTLC3.18516335.18518165.rev.compl.REF    ------------------------------------------------------------ 31039

chimp.LOC112206744-LOC107973052-GGT2.revcompl    ------------------------------------------------------------ 61762
chimp54546-135457.revcompl.Pan.troglodytes.clone.rp43-41g5.GenBank:AC099533.36    ------------------------------------------------------------ 68417
human.FAM30B-LOC105372935-GGT2.NCBI.    tcggatcgctctccttcttgctggcatccacagtaactatactcaataaacacaccaact 83687
human.FAM230E-LOC105377182-GGT3P.NCBI.    ------------------------------------------------------------ 57310
human.FAM230J-LOC105372942-GGTLC5P.NCBI.rev.compl    ------------------------------------------------------------ 46364
human.FAM230A.ncRNA.18487127.18500594-GGTLC3.18516335.18518165.rev.compl.REF    ------------------------------------------------------------ 31039

chimp.LOC112206744-LOC107973052-GGT2.revcompl    ------------------------------------------------------------ 61762
chimp54546-135457.revcompl.Pan.troglodytes.clone.rp43-41g5.GenBank:AC099533.36    ------------------------------------------------------------ 68417
human.FAM30B-LOC105372935-GGT2.NCBI.    ttgatgccaaatcctaggccatcccacacatcttaaaaataaaacaaaaatatgctattc 83747
human.FAM230E-LOC105377182-GGT3P.NCBI.    ------------------------------------------------------------ 57310
human.FAM230J-LOC105372942-GGTLC5P.NCBI.rev.compl    ------------------------------------------------------------ 46364
human.FAM230A.ncRNA.18487127.18500594-GGTLC3.18516335.18518165.rev.compl.REF    ------------------------------------------------------------ 31039

chimp.LOC112206744-LOC107973052-GGT2.revcompl    ------------------------------------------------------------ 61762
chimp54546-135457.revcompl.Pan.troglodytes.clone.rp43-41g5.GenBank:AC099533.36    ------------------------------------------------------------ 68417
human.FAM30B-LOC105372935-GGT2.NCBI.    cagctaccctcactctttttgtggtttcctgcagtccaactagctacaaatctcaaagcc 83807
human.FAM230E-LOC105377182-GGT3P.NCBI.    ------------------------------------------------------------ 57310
human.FAM230J-LOC105372942-GGTLC5P.NCBI.rev.compl    ------------------------------------------------------------ 46364
human.FAM230A.ncRNA.18487127.18500594-GGTLC3.18516335.18518165.rev.compl.REF    ------------------------------------------------------------ 31039

chimp.LOC112206744-LOC107973052-GGT2.revcompl    ------------------------------------------------------------ 61762
chimp54546-135457.revcompl.Pan.troglodytes.clone.rp43-41g5.GenBank:AC099533.36    ------------------------------------------------------------ 68417
human.FAM30B-LOC105372935-GGT2.NCBI.    aaagattgtagctccctctcttttgttctttcatcaagccagggagtcctgctcctctct 83867
human.FAM230E-LOC105377182-GGT3P.NCBI.    ------------------------------------------------------------ 57310
human.FAM230J-LOC105372942-GGTLC5P.NCBI.rev.compl    ------------------------------------------------------------ 46364
human.FAM230A.ncRNA.18487127.18500594-GGTLC3.18516335.18518165.rev.compl.REF    ------------------------------------------------------------ 31039

chimp.LOC112206744-LOC107973052-GGT2.revcompl    ------------------------------------------------------------ 61762
chimp54546-135457.revcompl.Pan.troglodytes.clone.rp43-41g5.GenBank:AC099533.36    ------------------------------------------------------------ 68417
human.FAM30B-LOC105372935-GGT2.NCBI.    ctctagtctctcctgtgtcagtgccacttgtcaactgcctaatccagccacgctgctctt 83927
human.FAM230E-LOC105377182-GGT3P.NCBI.    ------------------------------------------------------------ 57310
human.FAM230J-LOC105372942-GGTLC5P.NCBI.rev.compl    ------------------------------------------------------------ 46364
human.FAM230A.ncRNA.18487127.18500594-GGTLC3.18516335.18518165.rev.compl.REF    ------------------------------------------------------------ 31039

chimp.LOC112206744-LOC107973052-GGT2.revcompl    ------------------------------------------------------------ 61762
chimp54546-135457.revcompl.Pan.troglodytes.clone.rp43-41g5.GenBank:AC099533.36    ------------------------------------------------------------ 68417
human.FAM30B-LOC105372935-GGT2.NCBI.    caatattaaaggtcttcagcatattttaaagttagtctcctactgccactcttttcccta 83987
```

```
human.FAM230E-LOC105377182-GGT3P.NCBI.                                   ------------------------------------------------------------ 57310
human.FAM230J-LOC105372942-GGTLC5P.NCBI.rev.compl                        ------------------------------------------------------------ 46364
human.FAM230A.ncRNA.18487127.18500594-GGTLC3.18516335.18518165.rev.compl.REF ------------------------------------------------------------ 31039

chimp.LOC112206744-LOC107973052-GGT2.revcompl                            ------------------------------------------------------------ 61762
chimp54546-135457.revcompl.Pan.troglodytes.clone.rp43-41g5.GenBank:AC099533.36 ------------------------------------------------------------ 68417
human.FAM30B-LOC105372935-GGT2.NCBI.                                     ctcttgccagtccactcagtaaactactgccatacaggtctcccacagcagtttctcacc 84047
human.FAM230E-LOC105377182-GGT3P.NCBI.                                   ------------------------------------------------------------ 57310
human.FAM230J-LOC105372942-GGTLC5P.NCBI.rev.compl                        ------------------------------------------------------------ 46364
human.FAM230A.ncRNA.18487127.18500594-GGTLC3.18516335.18518165.rev.compl.REF ------------------------------------------------------------ 31039

chimp.LOC112206744-LOC107973052-GGT2.revcompl                            ------------------------------------------------------------ 61762
chimp54546-135457.revcompl.Pan.troglodytes.clone.rp43-41g5.GenBank:AC099533.36 ------------------------------------------------------------ 68417
human.FAM30B-LOC105372935-GGT2.NCBI.                                     agataaacaatggccggagattccactttgccaatcacatcaagtccaaactccgtgtct 84107
human.FAM230E-LOC105377182-GGT3P.NCBI.                                   ------------------------------------------------------------ 57310
human.FAM230J-LOC105372942-GGTLC5P.NCBI.rev.compl                        ------------------------------------------------------------ 46364
human.FAM230A.ncRNA.18487127.18500594-GGTLC3.18516335.18518165.rev.compl.REF ------------------------------------------------------------ 31039

chimp.LOC112206744-LOC107973052-GGT2.revcompl                            ------------------------------------------------------------ 61762
chimp54546-135457.revcompl.Pan.troglodytes.clone.rp43-41g5.GenBank:AC099533.36 ------------------------------------------------------------ 68417
human.FAM30B-LOC105372935-GGT2.NCBI.                                     tggtatccaaggcttctaaagtctggccccactccacctactcactctgacacccaccac 84167
human.FAM230E-LOC105377182-GGT3P.NCBI.                                   ------------------------------------------------------------ 57310
human.FAM230J-LOC105372942-GGTLC5P.NCBI.rev.compl                        ------------------------------------------------------------ 46364
human.FAM230A.ncRNA.18487127.18500594-GGTLC3.18516335.18518165.rev.compl.REF ------------------------------------------------------------ 31039

chimp.LOC112206744-LOC107973052-GGT2.revcompl                            ------------------------------------------------------------ 61762
chimp54546-135457.revcompl.Pan.troglodytes.clone.rp43-41g5.GenBank:AC099533.36 ------------------------------------------------------------ 68417
human.FAM30B-LOC105372935-GGT2.NCBI.                                     tccctccaaacatccccggtacgtcttactggattttgccatttcctccaagttgaccag 84227
human.FAM230E-LOC105377182-GGT3P.NCBI.                                   ------------------------------------------------------------ 57310
human.FAM230J-LOC105372942-GGTLC5P.NCBI.rev.compl                        ------------------------------------------------------------ 46364
human.FAM230A.ncRNA.18487127.18500594-GGTLC3.18516335.18518165.rev.compl.REF ------------------------------------------------------------ 31039

chimp.LOC112206744-LOC107973052-GGT2.revcompl                            ------------------------------------------------------------ 61762
chimp54546-135457.revcompl.Pan.troglodytes.clone.rp43-41g5.GenBank:AC099533.36 ------------------------------------------------------------ 68417
human.FAM30B-LOC105372935-GGT2.NCBI.                                     gagtcattcacacctaaaggctatgcttacatcgttttccatctcttgtgttggtgagct 84287
human.FAM230E-LOC105377182-GGT3P.NCBI.                                   ------------------------------------------------------------ 57310
human.FAM230J-LOC105372942-GGTLC5P.NCBI.rev.compl                        ------------------------------------------------------------ 46364
human.FAM230A.ncRNA.18487127.18500594-GGTLC3.18516335.18518165.rev.compl.REF ------------------------------------------------------------ 31039

chimp.LOC112206744-LOC107973052-GGT2.revcompl                            ------------------------------------------------------------ 61762
chimp54546-135457.revcompl.Pan.troglodytes.clone.rp43-41g5.GenBank:AC099533.36 ------------------------------------------------------------ 68417
human.FAM30B-LOC105372935-GGT2.NCBI.                                     aaaaacaagatggaaaagaaaaaagaagaggaagagagaaaaagagaaaaggaagaaatg 84347
human.FAM230E-LOC105377182-GGT3P.NCBI.                                   ------------------------------------------------------------ 57310
human.FAM230J-LOC105372942-GGTLC5P.NCBI.rev.compl                        ------------------------------------------------------------ 46364
human.FAM230A.ncRNA.18487127.18500594-GGTLC3.18516335.18518165.rev.compl.REF ------------------------------------------------------------ 31039

chimp.LOC112206744-LOC107973052-GGT2.revcompl                            ------------------------------------------------------------ 61762
chimp54546-135457.revcompl.Pan.troglodytes.clone.rp43-41g5.GenBank:AC099533.36 ------------------------------------------------------------ 68417
human.FAM30B-LOC105372935-GGT2.NCBI.                                     gggaaaacagcaataggaccagctaactcctacagcagagtcttgccaggccctccccta 84407
human.FAM230E-LOC105377182-GGT3P.NCBI.                                   ------------------------------------------------------------ 57310
human.FAM230J-LOC105372942-GGTLC5P.NCBI.rev.compl                        ------------------------------------------------------------ 46364
human.FAM230A.ncRNA.18487127.18500594-GGTLC3.18516335.18518165.rev.compl.REF ------------------------------------------------------------ 31039

chimp.LOC112206744-LOC107973052-GGT2.revcompl                            ------------------------------------------------------------ 61762
chimp54546-135457.revcompl.Pan.troglodytes.clone.rp43-41g5.GenBank:AC099533.36 ------------------------------------------------------------ 68417
human.FAM30B-LOC105372935-GGT2.NCBI.                                     attgctttatatgtgttacaatgtttaaccctcacaactacaacaggagttgggtaccat 84467
human.FAM230E-LOC105377182-GGT3P.NCBI.                                   ------------------------------------------------------------ 57310
human.FAM230J-LOC105372942-GGTLC5P.NCBI.rev.compl                        ------------------------------------------------------------ 46364
human.FAM230A.ncRNA.18487127.18500594-GGTLC3.18516335.18518165.rev.compl.REF ------------------------------------------------------------ 31039

chimp.LOC112206744-LOC107973052-GGT2.revcompl                            ------------------------------------------------------------ 61762
chimp54546-135457.revcompl.Pan.troglodytes.clone.rp43-41g5.GenBank:AC099533.36 ------------------------------------------------------------ 68417
human.FAM30B-LOC105372935-GGT2.NCBI.                                     tccatccccacctgcagatgagtgtggcaccccaagattaagtatctgcccagggtcacg 84527
human.FAM230E-LOC105377182-GGT3P.NCBI.                                   ------------------------------------------------------------ 57310
human.FAM230J-LOC105372942-GGTLC5P.NCBI.rev.compl                        ------------------------------------------------------------ 46364
human.FAM230A.ncRNA.18487127.18500594-GGTLC3.18516335.18518165.rev.compl.REF ------------------------------------------------------------ 31039

chimp.LOC112206744-LOC107973052-GGT2.revcompl                            ------------------------------------------------------------ 61762
chimp54546-135457.revcompl.Pan.troglodytes.clone.rp43-41g5.GenBank:AC099533.36 ------------------------------------------------------------ 68417
human.FAM30B-LOC105372935-GGT2.NCBI.                                     tgaaaagtctgcatgcatgctgaaccattccagctgcaccactgcccaggaaaaaagtca 84587
human.FAM230E-LOC105377182-GGT3P.NCBI.                                   ------------------------------------------------------------ 57310
human.FAM230J-LOC105372942-GGTLC5P.NCBI.rev.compl                        ------------------------------------------------------------ 46364
human.FAM230A.ncRNA.18487127.18500594-GGTLC3.18516335.18518165.rev.compl.REF ------------------------------------------------------------ 31039

chimp.LOC112206744-LOC107973052-GGT2.revcompl                            ------------------------------------------------------------ 61762
chimp54546-135457.revcompl.Pan.troglodytes.clone.rp43-41g5.GenBank:AC099533.36 ------------------------------------------------------------ 68417
human.FAM30B-LOC105372935-GGT2.NCBI.                                     caggtaggaagacaagcatagaattaagaacaaagcaactaactgtaccaggtgctacag 84647
human.FAM230E-LOC105377182-GGT3P.NCBI.                                   ------------------------------------------------------------ 57310
human.FAM230J-LOC105372942-GGTLC5P.NCBI.rev.compl                        ------------------------------------------------------------ 46364
human.FAM230A.ncRNA.18487127.18500594-GGTLC3.18516335.18518165.rev.compl.REF ------------------------------------------------------------ 31039

chimp.LOC112206744-LOC107973052-GGT2.revcompl                            ------------------------------------------------------------ 61762
chimp54546-135457.revcompl.Pan.troglodytes.clone.rp43-41g5.GenBank:AC099533.36 ------------------------------------------------------------ 68417
human.FAM30B-LOC105372935-GGT2.NCBI.                                     aagggtggtcagcctggaaaatcaggaggtgcattccttgggagattgccgcaatcagag 84707
human.FAM230E-LOC105377182-GGT3P.NCBI.                                   ------------------------------------------------------------ 57310
human.FAM230J-LOC105372942-GGTLC5P.NCBI.rev.compl                        ------------------------------------------------------------ 46364
human.FAM230A.ncRNA.18487127.18500594-GGTLC3.18516335.18518165.rev.compl.REF ------------------------------------------------------------ 31039

chimp.LOC112206744-LOC107973052-GGT2.revcompl                            ------------------------------------------------------------ 61762
chimp54546-135457.revcompl.Pan.troglodytes.clone.rp43-41g5.GenBank:AC099533.36 ------------------------------------------------------------ 68417
human.FAM30B-LOC105372935-GGT2.NCBI.                                     tctgggagtatgcaggagactggcaggcagcccagggacaggtgaggcacaggcactcat 84767
human.FAM230E-LOC105377182-GGT3P.NCBI.                                   ------------------------------------------------------------ 57310
human.FAM230J-LOC105372942-GGTLC5P.NCBI.rev.compl                        ------------------------------------------------------------ 46364
human.FAM230A.ncRNA.18487127.18500594-GGTLC3.18516335.18518165.rev.compl.REF ------------------------------------------------------------ 31039

chimp.LOC112206744-LOC107973052-GGT2.revcompl                            ------------------------------------------------------------ 61762
chimp54546-135457.revcompl.Pan.troglodytes.clone.rp43-41g5.GenBank:AC099533.36 ------------------------------------------------------------ 68417
human.FAM30B-LOC105372935-GGT2.NCBI.                                     tcccttccatgtgagaagtcagggcacgtgcaaagccaaggccagaggacaaaggtgagc 84827
human.FAM230E-LOC105377182-GGT3P.NCBI.                                   ------------------------------------------------------------ 57310
human.FAM230J-LOC105372942-GGTLC5P.NCBI.rev.compl                        ------------------------------------------------------------ 46364
human.FAM230A.ncRNA.18487127.18500594-GGTLC3.18516335.18518165.rev.compl.REF ------------------------------------------------------------ 31039

chimp.LOC112206744-LOC107973052-GGT2.revcompl                            ------------------------------------------------------------ 61762
chimp54546-135457.revcompl.Pan.troglodytes.clone.rp43-41g5.GenBank:AC099533.36 ------------------------------------------------------------ 68417
human.FAM30B-LOC105372935-GGT2.NCBI.                                     tcgccaatagggccaatactgggtctggctggaaggggagggtggacagagatgggtact 84887
human.FAM230E-LOC105377182-GGT3P.NCBI.                                   ------------------------------------------------------------ 57310
human.FAM230J-LOC105372942-GGTLC5P.NCBI.rev.compl                        ------------------------------------------------------------ 46364
human.FAM230A.ncRNA.18487127.18500594-GGTLC3.18516335.18518165.rev.compl.REF ------------------------------------------------------------ 31039

chimp.LOC112206744-LOC107973052-GGT2.revcompl                            ------------------------------------------------------------ 61762
chimp54546-135457.revcompl.Pan.troglodytes.clone.rp43-41g5.GenBank:AC099533.36 ------------------------------------------------------------ 68417
human.FAM30B-LOC105372935-GGT2.NCBI.                                     aagaagtgaagatggacaggcaggaccaggtcttaaagggtttgtgtcttgctaaagatg 84947
human.FAM230E-LOC105377182-GGT3P.NCBI.                                   ------------------------------------------------------------ 57310
human.FAM230J-LOC105372942-GGTLC5P.NCBI.rev.compl                        ------------------------------------------------------------ 46364
human.FAM230A.ncRNA.18487127.18500594-GGTLC3.18516335.18518165.rev.compl.REF ------------------------------------------------------------ 31039

chimp.LOC112206744-LOC107973052-GGT2.revcompl                            ------------------------------------------------------------ 61762
chimp54546-135457.revcompl.Pan.troglodytes.clone.rp43-41g5.GenBank:AC099533.36 ------------------------------------------------------------ 68417
human.FAM30B-LOC105372935-GGT2.NCBI.                                     gacccttaattccgtgacacatctattcaaccactcctcattatctacctaaattctact 85007
human.FAM230E-LOC105377182-GGT3P.NCBI.                                   ------------------------------------------------------------ 57310
human.FAM230J-LOC105372942-GGTLC5P.NCBI.rev.compl                        ------------------------------------------------------------ 46364
human.FAM230A.ncRNA.18487127.18500594-GGTLC3.18516335.18518165.rev.compl.REF ------------------------------------------------------------ 31039

chimp.LOC112206744-LOC107973052-GGT2.revcompl                            ------------------------------------------------------------ 61762
chimp54546-135457.revcompl.Pan.troglodytes.clone.rp43-41g5.GenBank:AC099533.36 ------------------------------------------------------------ 68417
human.FAM30B-LOC105372935-GGT2.NCBI.                                     gtgcctttgcaaagcccagctcaagtcctttttcctctcaggcctgtcctccctactcct 85067
human.FAM230E-LOC105377182-GGT3P.NCBI.                                   ------------------------------------------------------------ 57310
```

```
human.FAM230J-LOC105372942-GGTLC5P.NCBI.rev.compl                          ------------------------------------------------------------ 46364
human.FAM230A.ncRNA.18487127.18500594-GGTLC3.18516335.18518165.rev.compl.REF ------------------------------------------------------------ 31039

chimp.LOC112206744-LOC107973052-GGT2.revcompl                              ------------------------------------------------------------ 61762
chimp54546-135457.revcompl.Pan.troglodytes.clone.rp43-41g5.GenBank:AC099533.36 ------------------------------------------------------------ 68417
human.FAM30B-LOC105372935-GGT2.NCBI.                                       gagcaaaccacgggtgtggagacaggtcccaaccacctgcagatgacagaaggaagctgc 85127
human.FAM230E-LOC105377182-GGT3P.NCBI.                                     ------------------------------------------------------------ 57310
human.FAM230J-LOC105372942-GGTLC5P.NCBI.rev.compl                          ------------------------------------------------------------ 46364
human.FAM230A.ncRNA.18487127.18500594-GGTLC3.18516335.18518165.rev.compl.REF ------------------------------------------------------------ 31039

chimp.LOC112206744-LOC107973052-GGT2.revcompl                              ------------------------------------------------------------ 61762
chimp54546-135457.revcompl.Pan.troglodytes.clone.rp43-41g5.GenBank:AC099533.36 ------------------------------------------------------------ 68417
human.FAM30B-LOC105372935-GGT2.NCBI.                                       atctaggatcggtctacacagaggcaagaagggatcctcctcctctgccttctcctttca 85187
human.FAM230E-LOC105377182-GGT3P.NCBI.                                     ------------------------------------------------------------ 57310
human.FAM230J-LOC105372942-GGTLC5P.NCBI.rev.compl                          ------------------------------------------------------------ 46364
human.FAM230A.ncRNA.18487127.18500594-GGTLC3.18516335.18518165.rev.compl.REF ------------------------------------------------------------ 31039

chimp.LOC112206744-LOC107973052-GGT2.revcompl                              ------------------------------------------------------------ 61762
chimp54546-135457.revcompl.Pan.troglodytes.clone.rp43-41g5.GenBank:AC099533.36 ------------------------------------------------------------ 68417
human.FAM30B-LOC105372935-GGT2.NCBI.                                       tgctgttcttactttctctcaaaaagaaagaaaatacagtgtcattgcttcttccctaag 85247
human.FAM230E-LOC105377182-GGT3P.NCBI.                                     ------------------------------------------------------------ 57310
human.FAM230J-LOC105372942-GGTLC5P.NCBI.rev.compl                          ------------------------------------------------------------ 46364
human.FAM230A.ncRNA.18487127.18500594-GGTLC3.18516335.18518165.rev.compl.REF ------------------------------------------------------------ 31039

chimp.LOC112206744-LOC107973052-GGT2.revcompl                              ------------------------------------------------------------ 61762
chimp54546-135457.revcompl.Pan.troglodytes.clone.rp43-41g5.GenBank:AC099533.36 ------------------------------------------------------------ 68417
human.FAM30B-LOC105372935-GGT2.NCBI.                                       cttgttaagtcaaagcaagtcaaagcatcagagcttcattacttggctttttctagctat 85307
human.FAM230E-LOC105377182-GGT3P.NCBI.                                     ------------------------------------------------------------ 57310
human.FAM230J-LOC105372942-GGTLC5P.NCBI.rev.compl                          ------------------------------------------------------------ 46364
human.FAM230A.ncRNA.18487127.18500594-GGTLC3.18516335.18518165.rev.compl.REF ------------------------------------------------------------ 31039

chimp.LOC112206744-LOC107973052-GGT2.revcompl                              ------------------------------------------------------------ 61762
chimp54546-135457.revcompl.Pan.troglodytes.clone.rp43-41g5.GenBank:AC099533.36 ------------------------------------------------------------ 68417
human.FAM30B-LOC105372935-GGT2.NCBI.                                       aagcgtctggaaaatatcaggagtcactggcctctaggaatccattaagttaaaggagct 85367
human.FAM230E-LOC105377182-GGT3P.NCBI.                                     ------------------------------------------------------------ 57310
human.FAM230J-LOC105372942-GGTLC5P.NCBI.rev.compl                          ------------------------------------------------------------ 46364
human.FAM230A.ncRNA.18487127.18500594-GGTLC3.18516335.18518165.rev.compl.REF ------------------------------------------------------------ 31039

chimp.LOC112206744-LOC107973052-GGT2.revcompl                              ------------------------------------------------------------ 61762
chimp54546-135457.revcompl.Pan.troglodytes.clone.rp43-41g5.GenBank:AC099533.36 -------------------------------ATAATCCCAGCACTTTGAGAGGCCGAGGT 68446
human.FAM30B-LOC105372935-GGT2.NCBI.                                       ccatgatctactatcataagatgacaaagaaaatatgcactttctttgggaggtcaagtt 85427
human.FAM230E-LOC105377182-GGT3P.NCBI.                                     ------------------------------------------------------------ 57310
human.FAM230J-LOC105372942-GGTLC5P.NCBI.rev.compl                          ------------------------------------------------------------ 46364
human.FAM230A.ncRNA.18487127.18500594-GGTLC3.18516335.18518165.rev.compl.REF ------------------------------------------------------------ 31039

chimp.LOC112206744-LOC107973052-GGT2.revcompl                              ------------------------------------------------------------ 61762
chimp54546-135457.revcompl.Pan.troglodytes.clone.rp43-41g5.GenBank:AC099533.36 GGGCGGATTGCCTGAAGTCAGAAGTTCAAGACTAGTCTGACCATCATAGGGAAACCCAGT 68506
human.FAM30B-LOC105372935-GGT2.NCBI.                                       gggcgaatcacttgaggtcaggagttcaagaccaatctggccaacatggtgaaaccccgt 85487
human.FAM230E-LOC105377182-GGT3P.NCBI.                                     ------------------------------------------------------------ 57310
human.FAM230J-LOC105372942-GGTLC5P.NCBI.rev.compl                          ------------------------------------------------------------ 46364
human.FAM230A.ncRNA.18487127.18500594-GGTLC3.18516335.18518165.rev.compl.REF ------------------------------------------------------------ 31039

chimp.LOC112206744-LOC107973052-GGT2.revcompl                              ------------------------------------------------------------ 61762
chimp54546-135457.revcompl.Pan.troglodytes.clone.rp43-41g5.GenBank:AC099533.36 CTCTACTACAAATACAAAAAAATTATCCAGGCATGGTGGCGTGCACCTGTAATCCCAGCT 68566
human.FAM30B-LOC105372935-GGT2.NCBI.                                       ctctactgaaa--acacaaaaattagctgggcttggtggcaggcacctgtaatcccagct 85545
human.FAM230E-LOC105377182-GGT3P.NCBI.                                     ------------------------------------------------------------ 57310
human.FAM230J-LOC105372942-GGTLC5P.NCBI.rev.compl                          ------------------------------------------------------------ 46364
human.FAM230A.ncRNA.18487127.18500594-GGTLC3.18516335.18518165.rev.compl.REF ------------------------------------------------------------ 31039

chimp.LOC112206744-LOC107973052-GGT2.revcompl                              ------------------------------------------------------------ 61762
chimp54546-135457.revcompl.Pan.troglodytes.clone.rp43-41g5.GenBank:AC099533.36 CCTTGGAAGGCTGCGGCAGGGGAATTGCTTGA------ACCAGGGAGATGGAGGTTGCAG 68620
human.FAM30B-LOC105372935-GGT2.NCBI.                                       actcaggagactgaggcaggagaatcgattgaacccttacccaggagacagaggttgcaa 85605
human.FAM230E-LOC105377182-GGT3P.NCBI.                                     ------------------------------------------------------------ 57310
human.FAM230J-LOC105372942-GGTLC5P.NCBI.rev.compl                          ------------------------------------------------------------ 46364
human.FAM230A.ncRNA.18487127.18500594-GGTLC3.18516335.18518165.rev.compl.REF ------------------------------------------------------------ 31039

chimp.LOC112206744-LOC107973052-GGT2.revcompl                              ------------------------------------------------------------ 61762
chimp54546-135457.revcompl.Pan.troglodytes.clone.rp43-41g5.GenBank:AC099533.36 TGAGCTGAGATCACGCCACTGCACTCCAGCCTGGGCAACAGAGTGATACTCCGTCTCAAA 68680
human.FAM30B-LOC105372935-GGT2.NCBI.                                       tgagccgagatggcaccacggcactccagcctgagcaacagcgtg--actccatctcaaa 85663
human.FAM230E-LOC105377182-GGT3P.NCBI.                                     ------------------------------------------------------------ 57310
human.FAM230J-LOC105372942-GGTLC5P.NCBI.rev.compl                          ------------------------------------------------------------ 46364
human.FAM230A.ncRNA.18487127.18500594-GGTLC3.18516335.18518165.rev.compl.REF ------------------------------------------------------------ 31039

chimp.LOC112206744-LOC107973052-GGT2.revcompl                              ------------------------------------------------------------ 61762
chimp54546-135457.revcompl.Pan.troglodytes.clone.rp43-41g5.GenBank:AC099533.36 AACAAACAAC-------------------------------------------------- 68691
human.FAM30B-LOC105372935-GGT2.NCBI.                                       aaaaaaaaaaagaaagaaagaaaggaaggaaggaagaaagaagaaaatatgcattttctc 85723
human.FAM230E-LOC105377182-GGT3P.NCBI.                                     ------------------------------------------------------------ 57310
human.FAM230J-LOC105372942-GGTLC5P.NCBI.rev.compl                          ------------------------------------------------------------ 46364
human.FAM230A.ncRNA.18487127.18500594-GGTLC3.18516335.18518165.rev.compl.REF ------------------------------------------------------------ 31039

chimp.LOC112206744-LOC107973052-GGT2.revcompl                              ------------------------------------------------------------ 61762
chimp54546-135457.revcompl.Pan.troglodytes.clone.rp43-41g5.GenBank:AC099533.36 ------------------------------------------------------------ 68691
human.FAM30B-LOC105372935-GGT2.NCBI.                                       aggattaaataacaaaaaatgccaagtttcttagctccagtcaagaaaatgacatcatgt 85783
human.FAM230E-LOC105377182-GGT3P.NCBI.                                     ------------------------------------------------------------ 57310
human.FAM230J-LOC105372942-GGTLC5P.NCBI.rev.compl                          ------------------------------------------------------------ 46364
human.FAM230A.ncRNA.18487127.18500594-GGTLC3.18516335.18518165.rev.compl.REF ------------------------------------------------------------ 31039

chimp.LOC112206744-LOC107973052-GGT2.revcompl                              ------------------------------------------------------------ 61762
chimp54546-135457.revcompl.Pan.troglodytes.clone.rp43-41g5.GenBank:AC099533.36 ------------------------------------------------------------ 68691
human.FAM30B-LOC105372935-GGT2.NCBI.                                       gacaacagcagccattgcaagtttatcagaagatgaacaattacagatttttcatttctg 85843
human.FAM230E-LOC105377182-GGT3P.NCBI.                                     ------------------------------------------------------------ 57310
human.FAM230J-LOC105372942-GGTLC5P.NCBI.rev.compl                          ------------------------------------------------------------ 46364
human.FAM230A.ncRNA.18487127.18500594-GGTLC3.18516335.18518165.rev.compl.REF ------------------------------------------------------------ 31039

chimp.LOC112206744-LOC107973052-GGT2.revcompl                              ------------------------------------------------------------ 61762
chimp54546-135457.revcompl.Pan.troglodytes.clone.rp43-41g5.GenBank:AC099533.36 ------------------------------------------------------------ 68691
human.FAM30B-LOC105372935-GGT2.NCBI.                                       aataaggaaccatttacacagacacatgtataacaaatgtctgtgcacatccaggacttg 85903
human.FAM230E-LOC105377182-GGT3P.NCBI.                                     ------------------------------------------------------------ 57310
human.FAM230J-LOC105372942-GGTLC5P.NCBI.rev.compl                          ------------------------------------------------------------ 46364
human.FAM230A.ncRNA.18487127.18500594-GGTLC3.18516335.18518165.rev.compl.REF ------------------------------------------------------------ 31039

chimp.LOC112206744-LOC107973052-GGT2.revcompl                              ------------------------------------------------------------ 61762
chimp54546-135457.revcompl.Pan.troglodytes.clone.rp43-41g5.GenBank:AC099533.36 ------------------------------------------------------------ 68691
human.FAM30B-LOC105372935-GGT2.NCBI.                                       tcaaatcttaacattttgccacacctggtttagtttttttatttttcctttcaagaaaga 85963
human.FAM230E-LOC105377182-GGT3P.NCBI.                                     ------------------------------------------------------------ 57310
human.FAM230J-LOC105372942-GGTLC5P.NCBI.rev.compl                          ------------------------------------------------------------ 46364
human.FAM230A.ncRNA.18487127.18500594-GGTLC3.18516335.18518165.rev.compl.REF ------------------------------------------------------------ 31039

chimp.LOC112206744-LOC107973052-GGT2.revcompl                              ------------------------------------------------------------ 61762
chimp54546-135457.revcompl.Pan.troglodytes.clone.rp43-41g5.GenBank:AC099533.36 ------------------------------------------------------------ 68691
human.FAM30B-LOC105372935-GGT2.NCBI.                                       aagcattacaggctgtgcttcaatccctatttcacccccttttcaagccccactattccac 86023
human.FAM230E-LOC105377182-GGT3P.NCBI.                                     ------------------------------------------------------------ 57310
human.FAM230J-LOC105372942-GGTLC5P.NCBI.rev.compl                          ------------------------------------------------------------ 46364
human.FAM230A.ncRNA.18487127.18500594-GGTLC3.18516335.18518165.rev.compl.REF ------------------------------------------------------------ 31039

chimp.LOC112206744-LOC107973052-GGT2.revcompl                              ------------------------------------------------------------ 61762
chimp54546-135457.revcompl.Pan.troglodytes.clone.rp43-41g5.GenBank:AC099533.36 ------------------------------------------------------------ 68691
human.FAM30B-LOC105372935-GGT2.NCBI.                                       ccatcccagaaatgacaacatcccgaatttggtatttaccactcttgcacatttattttt 86083
human.FAM230E-LOC105377182-GGT3P.NCBI.                                     ------------------------------------------------------------ 57310
human.FAM230J-LOC105372942-GGTLC5P.NCBI.rev.compl                          ------------------------------------------------------------ 46364
human.FAM230A.ncRNA.18487127.18500594-GGTLC3.18516335.18518165.rev.compl.REF ------------------------------------------------------------ 31039

chimp.LOC112206744-LOC107973052-GGT2.revcompl                              ------------------------------------------------------------ 61762
chimp54546-135457.revcompl.Pan.troglodytes.clone.rp43-41g5.GenBank:AC099533.36 ------------------------------------------------------------ 68691
human.FAM30B-LOC105372935-GGT2.NCBI.                                       atgcttttactacctatgtatgtagccataaacaatatacagagtttttggatgctataa 86143
human.FAM230E-LOC105377182-GGT3P.NCBI.                                     ------------------------------------------------------------ 57310
human.FAM230J-LOC105372942-GGTLC5P.NCBI.rev.compl                          ------------------------------------------------------------ 46364
```

```
human.FAM230A.ncRNA.18487127.18500594-GGTLC3.18516335.18518165.rev.compl.REF        ------------------------------------------------------------ 31039

chimp.LOC112206744-LOC107973052-GGT2.revcompl                                       ------------------------------------------------------------ 61762
chimp54546-135457.revcompl.Pan.troglodytes.clone.rp43-41g5.GenBank:AC099533.36      ------------------------------------------------------------ 68691
human.FAM30B-LOC105372935-GGT2.NCBI.                                                aactgtttaaaatggcatcacactgtatgcacccttttgcatctgttttaattgacaaaa 86203
human.FAM230E-LOC105377182-GGT3P.NCBI.                                              ------------------------------------------------------------ 57310
human.FAM230J-LOC105372942-GGTLC5P.NCBI.rev.compl                                   ------------------------------------------------------------ 46364
human.FAM230A.ncRNA.18487127.18500594-GGTLC3.18516335.18518165.rev.compl.REF        ------------------------------------------------------------ 31039

chimp.LOC112206744-LOC107973052-GGT2.revcompl                                       ------------------------------------------------------------ 61762
chimp54546-135457.revcompl.Pan.troglodytes.clone.rp43-41g5.GenBank:AC099533.36      ------------------------------------------------------------ 68691
human.FAM30B-LOC105372935-GGT2.NCBI.                                                cattttgagggtaatccttgttgacatatgtcactccagggcattcctttcaactgctt 86263
human.FAM230E-LOC105377182-GGT3P.NCBI.                                              ------------------------------------------------------------ 57310
human.FAM230J-LOC105372942-GGTLC5P.NCBI.rev.compl                                   ------------------------------------------------------------ 46364
human.FAM230A.ncRNA.18487127.18500594-GGTLC3.18516335.18518165.rev.compl.REF        ------------------------------------------------------------ 31039

chimp.LOC112206744-LOC107973052-GGT2.revcompl                                       ------------------------------------------------------------ 61762
chimp54546-135457.revcompl.Pan.troglodytes.clone.rp43-41g5.GenBank:AC099533.36      ------------------------------------------------------------ 68691
human.FAM30B-LOC105372935-GGT2.NCBI.                                                tataccatggtccactgtctggataatcattatctatagcacaatgtattcattttccta 86323
human.FAM230E-LOC105377182-GGT3P.NCBI.                                              ------------------------------------------------------------ 57310
human.FAM230J-LOC105372942-GGTLC5P.NCBI.rev.compl                                   ------------------------------------------------------------ 46364
human.FAM230A.ncRNA.18487127.18500594-GGTLC3.18516335.18518165.rev.compl.REF        ------------------------------------------------------------ 31039

chimp.LOC112206744-LOC107973052-GGT2.revcompl                                       ------------------------------------------------------------ 61762
chimp54546-135457.revcompl.Pan.troglodytes.clone.rp43-41g5.GenBank:AC099533.36      ------------------------------------------------------------ 68691
human.FAM30B-LOC105372935-GGT2.NCBI.                                                ctgataaatttttaagtggtttccaacttttcattgcaataaatattcttgtaaatcttc 86383
human.FAM230E-LOC105377182-GGT3P.NCBI.                                              ------------------------------------------------------------ 57310
human.FAM230J-LOC105372942-GGTLC5P.NCBI.rev.compl                                   ------------------------------------------------------------ 46364
human.FAM230A.ncRNA.18487127.18500594-GGTLC3.18516335.18518165.rev.compl.REF        ------------------------------------------------------------ 31039

chimp.LOC112206744-LOC107973052-GGT2.revcompl                                       ------------------------------------------------------------ 61762
chimp54546-135457.revcompl.Pan.troglodytes.clone.rp43-41g5.GenBank:AC099533.36      ------------------------------------------------------------ 68691
human.FAM30B-LOC105372935-GGT2.NCBI.                                                gtccaaggttatcttcaaccacactaggcattgcccaaattgctctgcaacatcagtgca 86443
human.FAM230E-LOC105377182-GGT3P.NCBI.                                              ------------------------------------------------------------ 57310
human.FAM230J-LOC105372942-GGTLC5P.NCBI.rev.compl                                   ------------------------------------------------------------ 46364
human.FAM230A.ncRNA.18487127.18500594-GGTLC3.18516335.18518165.rev.compl.REF        ------------------------------------------------------------ 31039

chimp.LOC112206744-LOC107973052-GGT2.revcompl                                       ------------------------------------------------------------ 61762
chimp54546-135457.revcompl.Pan.troglodytes.clone.rp43-41g5.GenBank:AC099533.36      ------------------------------------------------------------ 68691
human.FAM30B-LOC105372935-GGT2.NCBI.                                                ccaatttacattgccaagatcagtgtttaagtgtttctattctttgatgtctttgtcaac 86503
human.FAM230E-LOC105377182-GGT3P.NCBI.                                              ------------------------------------------------------------ 57310
human.FAM230J-LOC105372942-GGTLC5P.NCBI.rev.compl                                   ------------------------------------------------------------ 46364
human.FAM230A.ncRNA.18487127.18500594-GGTLC3.18516335.18518165.rev.compl.REF        ------------------------------------------------------------ 31039

chimp.LOC112206744-LOC107973052-GGT2.revcompl                                       ------------------------------------------------------------ 61762
chimp54546-135457.revcompl.Pan.troglodytes.clone.rp43-41g5.GenBank:AC099533.36      ------------------------------------------------------------ 68691
human.FAM30B-LOC105372935-GGT2.NCBI.                                                cttggtactatttgactactacacttttcaccaatcagatataaggatgaaatggtgcct 86563
human.FAM230E-LOC105377182-GGT3P.NCBI.                                              ------------------------------------------------------------ 57310
human.FAM230J-LOC105372942-GGTLC5P.NCBI.rev.compl                                   ------------------------------------------------------------ 46364
human.FAM230A.ncRNA.18487127.18500594-GGTLC3.18516335.18518165.rev.compl.REF        ------------------------------------------------------------ 31039

chimp.LOC112206744-LOC107973052-GGT2.revcompl                                       ------------------------------------------------------------ 61762
chimp54546-135457.revcompl.Pan.troglodytes.clone.rp43-41g5.GenBank:AC099533.36      ------------------------------------------------------------ 68691
human.FAM30B-LOC105372935-GGT2.NCBI.                                                tcttggtattctgaaaattaattttaaatgggaaaataaattactaataagtgcagctta 86623
human.FAM230E-LOC105377182-GGT3P.NCBI.                                              ------------------------------------------------------------ 57310
human.FAM230J-LOC105372942-GGTLC5P.NCBI.rev.compl                                   ------------------------------------------------------------ 46364
human.FAM230A.ncRNA.18487127.18500594-GGTLC3.18516335.18518165.rev.compl.REF        ------------------------------------------------------------ 31039

chimp.LOC112206744-LOC107973052-GGT2.revcompl                                       ------------------------------------------------------------ 61762
chimp54546-135457.revcompl.Pan.troglodytes.clone.rp43-41g5.GenBank:AC099533.36      ------------------------------------------------------------ 68691
human.FAM30B-LOC105372935-GGT2.NCBI.                                                atcaacaagagttttcattaacatgtgggaatacaccaggtgttccttggattttcttga 86683
human.FAM230E-LOC105377182-GGT3P.NCBI.                                              ------------------------------------------------------------ 57310
human.FAM230J-LOC105372942-GGTLC5P.NCBI.rev.compl                                   ------------------------------------------------------------ 46364
human.FAM230A.ncRNA.18487127.18500594-GGTLC3.18516335.18518165.rev.compl.REF        ------------------------------------------------------------ 31039

chimp.LOC112206744-LOC107973052-GGT2.revcompl                                       ------------------------------------------------------------ 61762
chimp54546-135457.revcompl.Pan.troglodytes.clone.rp43-41g5.GenBank:AC099533.36      ------------------------------------------------------------ 68691
human.FAM30B-LOC105372935-GGT2.NCBI.                                                aaattctacagggagggtacgggagtgacggtacagataagattggacatgagctgataa 86743
human.FAM230E-LOC105377182-GGT3P.NCBI.                                              ------------------------------------------------------------ 57310
human.FAM230J-LOC105372942-GGTLC5P.NCBI.rev.compl                                   ------------------------------------------------------------ 46364
human.FAM230A.ncRNA.18487127.18500594-GGTLC3.18516335.18518165.rev.compl.REF        ------------------------------------------------------------ 31039

chimp.LOC112206744-LOC107973052-GGT2.revcompl                                       ------------------------------------------------------------ 61762
chimp54546-135457.revcompl.Pan.troglodytes.clone.rp43-41g5.GenBank:AC099533.36      ------------------------------------------------------------ 68691
human.FAM30B-LOC105372935-GGT2.NCBI.                                                ttgttggaactaggtgatgagtgtatgagggttaattattctgctctctccactttggta 86803
human.FAM230E-LOC105377182-GGT3P.NCBI.                                              ------------------------------------------------------------ 57310
human.FAM230J-LOC105372942-GGTLC5P.NCBI.rev.compl                                   ------------------------------------------------------------ 46364
human.FAM230A.ncRNA.18487127.18500594-GGTLC3.18516335.18518165.rev.compl.REF        ------------------------------------------------------------ 31039

chimp.LOC112206744-LOC107973052-GGT2.revcompl                                       ------------------------------------------------------------ 61762
chimp54546-135457.revcompl.Pan.troglodytes.clone.rp43-41g5.GenBank:AC099533.36      ------------------------------------------------------------ 68691
human.FAM30B-LOC105372935-GGT2.NCBI.                                                catatttgaaatttttcacaatagaaattaaaagaagaacggggcgtggtggctcacgcc 86863
human.FAM230E-LOC105377182-GGT3P.NCBI.                                              ------------------------------------------------------------ 57310
human.FAM230J-LOC105372942-GGTLC5P.NCBI.rev.compl                                   ------------------------------------------------------------ 46364
human.FAM230A.ncRNA.18487127.18500594-GGTLC3.18516335.18518165.rev.compl.REF        ------------------------------------------------------------ 31039

chimp.LOC112206744-LOC107973052-GGT2.revcompl                                       ------------------------------------------------------------ 61762
chimp54546-135457.revcompl.Pan.troglodytes.clone.rp43-41g5.GenBank:AC099533.36      ------------------------------------------------------------ 68691
human.FAM30B-LOC105372935-GGT2.NCBI.                                                tgtaattccagcactttggaaggccatggcagaggatcacttgaggtcaggaattaaaga 86923
human.FAM230E-LOC105377182-GGT3P.NCBI.                                              ------------------------------------------------------------ 57310
human.FAM230J-LOC105372942-GGTLC5P.NCBI.rev.compl                                   ------------------------------------------------------------ 46364
human.FAM230A.ncRNA.18487127.18500594-GGTLC3.18516335.18518165.rev.compl.REF        ------------------------------------------------------------ 31039

chimp.LOC112206744-LOC107973052-GGT2.revcompl                                       ------------------------------------------------------------ 61762
chimp54546-135457.revcompl.Pan.troglodytes.clone.rp43-41g5.GenBank:AC099533.36      -----------------------------------AAACAAACAAAAATTAGCCAGACGT 68716
human.FAM30B-LOC105372935-GGT2.NCBI.                                                ccagctggtcaacatggggaaaccccacctctactaaaaatacaaaaattagctgggtat 86983
human.FAM230E-LOC105377182-GGT3P.NCBI.                                              ------------------------------------------------------------ 57310
human.FAM230J-LOC105372942-GGTLC5P.NCBI.rev.compl                                   ------------------------------------------------------------ 46364
human.FAM230A.ncRNA.18487127.18500594-GGTLC3.18516335.18518165.rev.compl.REF        ------------------------------------------------------------ 31039

chimp.LOC112206744-LOC107973052-GGT2.revcompl                                       ------------------------------------------------------------ 61762
chimp54546-135457.revcompl.Pan.troglodytes.clone.rp43-41g5.GenBank:AC099533.36      GATGGCTAACACCTGTAATCCCAGCTACTCAGGAGGCTGAGACGGGAGAATCACCTGAAC 68776
human.FAM30B-LOC105372935-GGT2.NCBI.                                                ggtgg------tgtgtaatcccagctattgaggaggctgaggcacgagaatcgcttggac 87037
human.FAM230E-LOC105377182-GGT3P.NCBI.                                              ------------------------------------------------------------ 57310
human.FAM230J-LOC105372942-GGTLC5P.NCBI.rev.compl                                   ------------------------------------------------------------ 46364
human.FAM230A.ncRNA.18487127.18500594-GGTLC3.18516335.18518165.rev.compl.REF        ------------------------------------------------------------ 31039

chimp.LOC112206744-LOC107973052-GGT2.revcompl                                       ------------------------------------------------------------ 61762
chimp54546-135457.revcompl.Pan.troglodytes.clone.rp43-41g5.GenBank:AC099533.36      CCAGGAGGTAGAAGTTGCAGTGAGCTGAGATCCCACCACCGCACTCCAGCCTGGGCCACA 68836
human.FAM30B-LOC105372935-GGT2.NCBI.                                                ccaggaggcagaggttgcagtgagccaagatcgtgccactgcactctagcctgggcaaca 87097
human.FAM230E-LOC105377182-GGT3P.NCBI.                                              ------------------------------------------------------------ 57310
human.FAM230J-LOC105372942-GGTLC5P.NCBI.rev.compl                                   ------------------------------------------------------------ 46364
human.FAM230A.ncRNA.18487127.18500594-GGTLC3.18516335.18518165.rev.compl.REF        ------------------------------------------------------------ 31039

chimp.LOC112206744-LOC107973052-GGT2.revcompl                                       ------------------------------------------------------------ 61762
chimp54546-135457.revcompl.Pan.troglodytes.clone.rp43-41g5.GenBank:AC099533.36      GAGCAAGACTCGGTCTCAAAAAACAAACAAACAAAAAAGTGAAAGTGGAATATATATA-- 68894
human.FAM30B-LOC105372935-GGT2.NCBI.                                                gggcaagcctctgtctcaaaaaaaaaaaaaaaaaaaaaagaagaagaagaagaaagaaa 87157
human.FAM230E-LOC105377182-GGT3P.NCBI.                                              ------------------------------------------------------------ 57310
human.FAM230J-LOC105372942-GGTLC5P.NCBI.rev.compl                                   ------------------------------------------------------------ 46364
human.FAM230A.ncRNA.18487127.18500594-GGTLC3.18516335.18518165.rev.compl.REF        ------------------------------------------------------------ 31039

chimp.LOC112206744-LOC107973052-GGT2.revcompl                                       ------------------------------------------------------------ 61762
chimp54546-135457.revcompl.Pan.troglodytes.clone.rp43-41g5.GenBank:AC099533.36      ------------------------------------------------------------ 68894
human.FAM30B-LOC105372935-GGT2.NCBI.                                                acaaagaagaaagaaagaaagaaaaagaaagaaagaaagaaaattaaaagaaggaaatgc 87217
human.FAM230E-LOC105377182-GGT3P.NCBI.                                              ------------------------------------------------------------ 57310
human.FAM230J-LOC105372942-GGTLC5P.NCBI.rev.compl                                   ------------------------------------------------------------ 46364
human.FAM230A.ncRNA.18487127.18500594-GGTLC3.18516335.18518165.rev.compl.REF        ------------------------------------------------------------ 31039
```

```
chimp.LOC112206744-LOC107973052-GGT2.revcompl                                       ------------------------------------------------------------ 61762
chimp54546-135457.revcompl.Pan.troglodytes.clone.rp43-41g5.GenBank:AC099533.36      ------------------------------------------------------------ 68894
human.FAM30B-LOC105372935-GGT2.NCBI.                                                cataaagcatctggagcaaagaacatccaaagaagagcactgatcactgcagcagacagc 87277
human.FAM230E-LOC105377182-GGT3P.NCBI.                                              ------------------------------------------------------------ 57310
human.FAM230J-LOC105372942-GGTLC5P.NCBI.rev.compl                                   ------------------------------------------------------------ 46364
human.FAM230A.ncRNA.18487127.18500594-GGTLC3.18516335.18518165.rev.compl.REF        ------------------------------------------------------------ 31039

chimp.LOC112206744-LOC107973052-GGT2.revcompl                                       ------------------------------------------------------------ 61762
chimp54546-135457.revcompl.Pan.troglodytes.clone.rp43-41g5.GenBank:AC099533.36      ------------------------------------------------------------ 68894
human.FAM30B-LOC105372935-GGT2.NCBI.                                                cattcagaatgggcagagagcagagctggcaggaagagaggacaaaggaagaccaggaa 87337
human.FAM230E-LOC105377182-GGT3P.NCBI.                                              ------------------------------------------------------------ 57310
human.FAM230J-LOC105372942-GGTLC5P.NCBI.rev.compl                                   ------------------------------------------------------------ 46364
human.FAM230A.ncRNA.18487127.18500594-GGTLC3.18516335.18518165.rev.compl.REF        ------------------------------------------------------------ 31039

chimp.LOC112206744-LOC107973052-GGT2.revcompl                                       ------------------------------------------------------------ 61762
chimp54546-135457.revcompl.Pan.troglodytes.clone.rp43-41g5.GenBank:AC099533.36      ------------------------------------------------------------ 68894
human.FAM30B-LOC105372935-GGT2.NCBI.                                                gggaagccaggaaaagagattttgcattgtgttattatttcaatcagaagacttaggaac 87397
human.FAM230E-LOC105377182-GGT3P.NCBI.                                              ------------------------------------------------------------ 57310
human.FAM230J-LOC105372942-GGTLC5P.NCBI.rev.compl                                   ------------------------------------------------------------ 46364
human.FAM230A.ncRNA.18487127.18500594-GGTLC3.18516335.18518165.rev.compl.REF        ------------------------------------------------------------ 31039

chimp.LOC112206744-LOC107973052-GGT2.revcompl                                       ------------------------------------------------------------ 61762
chimp54546-135457.revcompl.Pan.troglodytes.clone.rp43-41g5.GenBank:AC099533.36      ------------------------------------------------------------ 68894
human.FAM30B-LOC105372935-GGT2.NCBI.                                                attatttaattatcaaatttgtgtgtctgatgatcctttacaacactgcagcatcaaata 87457
human.FAM230E-LOC105377182-GGT3P.NCBI.                                              ------------------------------------------------------------ 57310
human.FAM230J-LOC105372942-GGTLC5P.NCBI.rev.compl                                   ------------------------------------------------------------ 46364
human.FAM230A.ncRNA.18487127.18500594-GGTLC3.18516335.18518165.rev.compl.REF        ------------------------------------------------------------ 31039

chimp.LOC112206744-LOC107973052-GGT2.revcompl                                       ------------------------------------------------------------ 61762
chimp54546-135457.revcompl.Pan.troglodytes.clone.rp43-41g5.GenBank:AC099533.36      ------------------------------------------------------------ 68894
human.FAM30B-LOC105372935-GGT2.NCBI.                                                aagtgttatcagatttctttcttttttttttttttgagactgggtctgattctgtagccc 87517
human.FAM230E-LOC105377182-GGT3P.NCBI.                                              ------------------------------------------------------------ 57310
human.FAM230J-LOC105372942-GGTLC5P.NCBI.rev.compl                                   ------------------------------------------------------------ 46364
human.FAM230A.ncRNA.18487127.18500594-GGTLC3.18516335.18518165.rev.compl.REF        ------------------------------------------------------------ 31039

chimp.LOC112206744-LOC107973052-GGT2.revcompl                                       ------------------------------------------------------------ 61762
chimp54546-135457.revcompl.Pan.troglodytes.clone.rp43-41g5.GenBank:AC099533.36      -------------------------------------------------TGTGTGTGTGT 68905
human.FAM30B-LOC105372935-GGT2.NCBI.                                                aggctggagtgcaggggtataataatagctcactgcagcttcaaactcctgagtttgttt 87577
human.FAM230E-LOC105377182-GGT3P.NCBI.                                              ------------------------------------------------------------ 57310
human.FAM230J-LOC105372942-GGTLC5P.NCBI.rev.compl                                   ------------------------------------------------------------ 46364
human.FAM230A.ncRNA.18487127.18500594-GGTLC3.18516335.18518165.rev.compl.REF        ------------------------------------------------------------ 31039

chimp.LOC112206744-LOC107973052-GGT2.revcompl                                       ------------------------------------------------------------ 61762
chimp54546-135457.revcompl.Pan.troglodytes.clone.rp43-41g5.GenBank:AC099533.36      GTATATGTGTATATGTGTGTGTGTATATG------------------------------- 68934
human.FAM30B-LOC105372935-GGT2.NCBI.                                                gtttgtttgtctgtttgtttgtttgtttggagagacagcgtctcgaactcctgggctcca 87637
human.FAM230E-LOC105377182-GGT3P.NCBI.                                              ------------------------------------------------------------ 57310
human.FAM230J-LOC105372942-GGTLC5P.NCBI.rev.compl                                   ------------------------------------------------------------ 46364
human.FAM230A.ncRNA.18487127.18500594-GGTLC3.18516335.18518165.rev.compl.REF        ------------------------------------------------------------ 31039

chimp.LOC112206744-LOC107973052-GGT2.revcompl                                       ------------------------------------------------------------ 61762
chimp54546-135457.revcompl.Pan.troglodytes.clone.rp43-41g5.GenBank:AC099533.36      ------------------------------------------------------------ 68934
human.FAM30B-LOC105372935-GGT2.NCBI.                                                gtgatcctcccacctcagcctcccaaagtgctgggattacaagcatgagccactgtgccc 87697
human.FAM230E-LOC105377182-GGT3P.NCBI.                                              ------------------------------------------------------------ 57310
human.FAM230J-LOC105372942-GGTLC5P.NCBI.rev.compl                                   ------------------------------------------------------------ 46364
human.FAM230A.ncRNA.18487127.18500594-GGTLC3.18516335.18518165.rev.compl.REF        ------------------------------------------------------------ 31039

chimp.LOC112206744-LOC107973052-GGT2.revcompl                                       ------------------------------------------------------------ 61762
chimp54546-135457.revcompl.Pan.troglodytes.clone.rp43-41g5.GenBank:AC099533.36      ------------------------------------------------------------ 68934
human.FAM30B-LOC105372935-GGT2.NCBI.                                                cagcccaacatctgatttcaaaagtactattgatattataatcaccattaaaatcactg 87757
human.FAM230E-LOC105377182-GGT3P.NCBI.                                              ------------------------------------------------------------ 57310
human.FAM230J-LOC105372942-GGTLC5P.NCBI.rev.compl                                   ------------------------------------------------------------ 46364
human.FAM230A.ncRNA.18487127.18500594-GGTLC3.18516335.18518165.rev.compl.REF        ------------------------------------------------------------ 31039

chimp.LOC112206744-LOC107973052-GGT2.revcompl                                       ------------------------------------------------------------ 61762
chimp54546-135457.revcompl.Pan.troglodytes.clone.rp43-41g5.GenBank:AC099533.36      ------------------------------------------------------------ 68934
human.FAM30B-LOC105372935-GGT2.NCBI.                                                aagtttttgtcacccaaagttttacgttgaaatatagaacatttcatttttttcccacac 87817
human.FAM230E-LOC105377182-GGT3P.NCBI.                                              ------------------------------------------------------------ 57310
human.FAM230J-LOC105372942-GGTLC5P.NCBI.rev.compl                                   ------------------------------------------------------------ 46364
human.FAM230A.ncRNA.18487127.18500594-GGTLC3.18516335.18518165.rev.compl.REF        ------------------------------------------------------------ 31039

chimp.LOC112206744-LOC107973052-GGT2.revcompl                                       ------------------------------------------------------------ 61762
chimp54546-135457.revcompl.Pan.troglodytes.clone.rp43-41g5.GenBank:AC099533.36      ------------------------------------------------------------ 68934
human.FAM30B-LOC105372935-GGT2.NCBI.                                                acctcacatctgcttttgatttttaacatgtgcatgaattacctcttattagaaaaaaaa 87877
human.FAM230E-LOC105377182-GGT3P.NCBI.                                              ------------------------------------------------------------ 57310
human.FAM230J-LOC105372942-GGTLC5P.NCBI.rev.compl                                   ------------------------------------------------------------ 46364
human.FAM230A.ncRNA.18487127.18500594-GGTLC3.18516335.18518165.rev.compl.REF        ------------------------------------------------------------ 31039

chimp.LOC112206744-LOC107973052-GGT2.revcompl                                       ------------------------------------------------------------ 61762
chimp54546-135457.revcompl.Pan.troglodytes.clone.rp43-41g5.GenBank:AC099533.36      ------------------------------------------------------------ 68934
human.FAM30B-LOC105372935-GGT2.NCBI.                                                acacaaaatttttttggtaaaagaattttccttaagtgggtatttttttccctaagtctgaa 87937
human.FAM230E-LOC105377182-GGT3P.NCBI.                                              ------------------------------------------------------------ 57310
human.FAM230J-LOC105372942-GGTLC5P.NCBI.rev.compl                                   ------------------------------------------------------------ 46364
human.FAM230A.ncRNA.18487127.18500594-GGTLC3.18516335.18518165.rev.compl.REF        ------------------------------------------------------------ 31039

chimp.LOC112206744-LOC107973052-GGT2.revcompl                                       ------------------------------------------------------------ 61762
chimp54546-135457.revcompl.Pan.troglodytes.clone.rp43-41g5.GenBank:AC099533.36      ------------------------------------------------------------ 68934
human.FAM30B-LOC105372935-GGT2.NCBI.                                                atgacttagtaaggataagaggtcaaaccatatgctttgtagtgaaactgcaactatgat 87997
human.FAM230E-LOC105377182-GGT3P.NCBI.                                              ------------------------------------------------------------ 57310
human.FAM230J-LOC105372942-GGTLC5P.NCBI.rev.compl                                   ------------------------------------------------------------ 46364
human.FAM230A.ncRNA.18487127.18500594-GGTLC3.18516335.18518165.rev.compl.REF        ------------------------------------------------------------ 31039

chimp.LOC112206744-LOC107973052-GGT2.revcompl                                       ------------------------------------------------------------ 61762
chimp54546-135457.revcompl.Pan.troglodytes.clone.rp43-41g5.GenBank:AC099533.36      ------------------------------------------------------------ 68934
human.FAM30B-LOC105372935-GGT2.NCBI.                                                gaataacattagaattctggatgccttccagcagctggaacagggcagacctgaggcggg 88057
human.FAM230E-LOC105377182-GGT3P.NCBI.                                              ------------------------------------------------------------ 57310
human.FAM230J-LOC105372942-GGTLC5P.NCBI.rev.compl                                   ------------------------------------------------------------ 46364
human.FAM230A.ncRNA.18487127.18500594-GGTLC3.18516335.18518165.rev.compl.REF        ------------------------------------------------------------ 31039

chimp.LOC112206744-LOC107973052-GGT2.revcompl                                       ------------------------------------------------------------ 61762
chimp54546-135457.revcompl.Pan.troglodytes.clone.rp43-41g5.GenBank:AC099533.36      ------------------------------------------------------------ 68934
human.FAM30B-LOC105372935-GGT2.NCBI.                                                agtcctcatgcttaggggaaggggctcaggcactgggagtcggtcagggctcccaggatg 88117
human.FAM230E-LOC105377182-GGT3P.NCBI.                                              ------------------------------------------------------------ 57310
human.FAM230J-LOC105372942-GGTLC5P.NCBI.rev.compl                                   ------------------------------------------------------------ 46364
human.FAM230A.ncRNA.18487127.18500594-GGTLC3.18516335.18518165.rev.compl.REF        ------------------------------------------------------------ 31039

chimp.LOC112206744-LOC107973052-GGT2.revcompl                                       ------------------------------------------------------------ 61762
chimp54546-135457.revcompl.Pan.troglodytes.clone.rp43-41g5.GenBank:AC099533.36      ------------------------------------------------------------ 68934
human.FAM30B-LOC105372935-GGT2.NCBI.                                                taaaagatacacatgaaagaaacaaacagtgggactcgggccagtgcaaaatttggcctg 88177
human.FAM230E-LOC105377182-GGT3P.NCBI.                                              ------------------------------------------------------------ 57310
human.FAM230J-LOC105372942-GGTLC5P.NCBI.rev.compl                                   ------------------------------------------------------------ 46364
human.FAM230A.ncRNA.18487127.18500594-GGTLC3.18516335.18518165.rev.compl.REF        ------------------------------------------------------------ 31039

chimp.LOC112206744-LOC107973052-GGT2.revcompl                                       ------------------------------------------------------------ 61762
chimp54546-135457.revcompl.Pan.troglodytes.clone.rp43-41g5.GenBank:AC099533.36      ------------------------------------------------------------ 68934
human.FAM30B-LOC105372935-GGT2.NCBI.                                                gaactgagctaaaaactgtcatactggtgggaagaagcagattggttcaacagggtaacc 88237
human.FAM230E-LOC105377182-GGT3P.NCBI.                                              ------------------------------------------------------------ 57310
human.FAM230J-LOC105372942-GGTLC5P.NCBI.rev.compl                                   ------------------------------------------------------------ 46364
human.FAM230A.ncRNA.18487127.18500594-GGTLC3.18516335.18518165.rev.compl.REF        ------------------------------------------------------------ 31039

chimp.LOC112206744-LOC107973052-GGT2.revcompl                                       ------------------------------------------------------------ 61762
chimp54546-135457.revcompl.Pan.troglodytes.clone.rp43-41g5.GenBank:AC099533.36      ------------------------------------------------------------ 68934
human.FAM30B-LOC105372935-GGT2.NCBI.                                                aaatgtgaaaaagaaaagcctcacaagaattaaaattgatggttggaactgggcgctccg 88297
human.FAM230E-LOC105377182-GGT3P.NCBI.                                              ------------------------------------------------------------ 57310
human.FAM230J-LOC105372942-GGTLC5P.NCBI.rev.compl                                   ------------------------------------------------------------ 46364
human.FAM230A.ncRNA.18487127.18500594-GGTLC3.18516335.18518165.rev.compl.REF        ------------------------------------------------------------ 31039
```

```
chimp.LOC112206744-LOC107973052-GGT2.revcompl                               ------------------------------------------------------------ 61762
chimp54546-135457.revcompl.Pan.troglodytes.clone.rp43-41g5.GenBank:AC099533.36 ------------------------------------------------------------ 68934
human.FAM30B-LOC105372935-GGT2.NCBI.                                        aggctgtgggccggtgtcctgccccgaagggtttggctgccagcaggtgcagcgctgccc 88357
human.FAM230E-LOC105377182-GGT3P.NCBI.                                      ------------------------------------------------------------ 57310
human.FAM230J-LOC105372942-GGTLC5P.NCBI.rev.compl                           ------------------------------------------------------------ 46364
human.FAM230A.ncRNA.18487127.18500594-GGTLC3.18516335.18518165.rev.compl.REF ------------------------------------------------------------ 31039

chimp.LOC112206744-LOC107973052-GGT2.revcompl                               ------------------------------------------------------------ 61762
chimp54546-135457.revcompl.Pan.troglodytes.clone.rp43-41g5.GenBank:AC099533.36 ------------------------------------------------------------ 68934
human.FAM30B-LOC105372935-GGT2.NCBI.                                        tgggataccagctcctgggatgcgctgtagtatcatcaatcaccggtgctgctcgctggg 88417
human.FAM230E-LOC105377182-GGT3P.NCBI.                                      ------------------------------------------------------------ 57310
human.FAM230J-LOC105372942-GGTLC5P.NCBI.rev.compl                           ------------------------------------------------------------ 46364
human.FAM230A.ncRNA.18487127.18500594-GGTLC3.18516335.18518165.rev.compl.REF ------------------------------------------------------------ 31039

chimp.LOC112206744-LOC107973052-GGT2.revcompl                               ------------------------------------------------------------ 61762
chimp54546-135457.revcompl.Pan.troglodytes.clone.rp43-41g5.GenBank:AC099533.36 ------------------------------------------------------------ 68934
human.FAM30B-LOC105372935-GGT2.NCBI.                                        ccttggaagcacacagtagtagcagatgtcagcaggggccaccagggccacctgccagt 88477
human.FAM230E-LOC105377182-GGT3P.NCBI.                                      ------------------------------------------------------------ 57310
human.FAM230J-LOC105372942-GGTLC5P.NCBI.rev.compl                           ------------------------------------------------------------ 46364
human.FAM230A.ncRNA.18487127.18500594-GGTLC3.18516335.18518165.rev.compl.REF ------------------------------------------------------------ 31039

chimp.LOC112206744-LOC107973052-GGT2.revcompl                               ------------------------------------------------------------ 61762
chimp54546-135457.revcompl.Pan.troglodytes.clone.rp43-41g5.GenBank:AC099533.36 ------------------------------------------------------------ 68934
human.FAM30B-LOC105372935-GGT2.NCBI.                                        gagcccactgcttagctgagtcatggtcaagtttctctgcagattgttgcctgaaaatat 88537
human.FAM230E-LOC105377182-GGT3P.NCBI.                                      ------------------------------------------------------------ 57310
human.FAM230J-LOC105372942-GGTLC5P.NCBI.rev.compl                           ------------------------------------------------------------ 46364
human.FAM230A.ncRNA.18487127.18500594-GGTLC3.18516335.18518165.rev.compl.REF ------------------------------------------------------------ 31039

chimp.LOC112206744-LOC107973052-GGT2.revcompl                               ------------------------------------------------------------ 61762
chimp54546-135457.revcompl.Pan.troglodytes.clone.rp43-41g5.GenBank:AC099533.36 ------------------------------------------------------------ 68934
human.FAM30B-LOC105372935-GGT2.NCBI.                                        ttcaccacctggaacctccctgtcccacccatcttcacttgcaatactcactcccctcac 88597
human.FAM230E-LOC105377182-GGT3P.NCBI.                                      ------------------------------------------------------------ 57310
human.FAM230J-LOC105372942-GGTLC5P.NCBI.rev.compl                           ------------------------------------------------------------ 46364
human.FAM230A.ncRNA.18487127.18500594-GGTLC3.18516335.18518165.rev.compl.REF ------------------------------------------------------------ 31039

chimp.LOC112206744-LOC107973052-GGT2.revcompl                               ------------------------------------------------------------ 61762
chimp54546-135457.revcompl.Pan.troglodytes.clone.rp43-41g5.GenBank:AC099533.36 ------------------------------------------------------------ 68934
human.FAM30B-LOC105372935-GGT2.NCBI.                                        ctctgcccctaaagcaattattcattaaccacaggatccaccatccaggaacaaaagag 88657
human.FAM230E-LOC105377182-GGT3P.NCBI.                                      ------------------------------------------------------------ 57310
human.FAM230J-LOC105372942-GGTLC5P.NCBI.rev.compl                           ------------------------------------------------------------ 46364
human.FAM230A.ncRNA.18487127.18500594-GGTLC3.18516335.18518165.rev.compl.REF ------------------------------------------------------------ 31039

chimp.LOC112206744-LOC107973052-GGT2.revcompl                               ------------------------------------------------------------ 61762
chimp54546-135457.revcompl.Pan.troglodytes.clone.rp43-41g5.GenBank:AC099533.36 ------------------------------------------------------------ 68934
human.FAM30B-LOC105372935-GGT2.NCBI.                                        gcttgaaagctctatctgtgcctgcacttcttggtccaaaaggcgcaggggggatgggga 88717
human.FAM230E-LOC105377182-GGT3P.NCBI.                                      ------------------------------------------------------------ 57310
human.FAM230J-LOC105372942-GGTLC5P.NCBI.rev.compl                           ------------------------------------------------------------ 46364
human.FAM230A.ncRNA.18487127.18500594-GGTLC3.18516335.18518165.rev.compl.REF ------------------------------------------------------------ 31039

chimp.LOC112206744-LOC107973052-GGT2.revcompl                               ------------------------------------------------------------ 61762
chimp54546-135457.revcompl.Pan.troglodytes.clone.rp43-41g5.GenBank:AC099533.36 ------------------------------------------------------------ 68934
human.FAM30B-LOC105372935-GGT2.NCBI.                                        gaggcaccggggtcatggtgcaggctcggctgttgagcctcattccccaaacacaatgtg 88777
human.FAM230E-LOC105377182-GGT3P.NCBI.                                      ------------------------------------------------------------ 57310
human.FAM230J-LOC105372942-GGTLC5P.NCBI.rev.compl                           ------------------------------------------------------------ 46364
human.FAM230A.ncRNA.18487127.18500594-GGTLC3.18516335.18518165.rev.compl.REF ------------------------------------------------------------ 31039

chimp.LOC112206744-LOC107973052-GGT2.revcompl                               ------------------------------------------------------------ 61762
chimp54546-135457.revcompl.Pan.troglodytes.clone.rp43-41g5.GenBank:AC099533.36 ------------------------------------------------------------ 68934
human.FAM30B-LOC105372935-GGT2.NCBI.                                        acaggtatcaatagttctggagttgatcatttcacagaaaggaaactgctgtcaacctgc 88837
human.FAM230E-LOC105377182-GGT3P.NCBI.                                      ------------------------------------------------------------ 57310
human.FAM230J-LOC105372942-GGTLC5P.NCBI.rev.compl                           ------------------------------------------------------------ 46364
human.FAM230A.ncRNA.18487127.18500594-GGTLC3.18516335.18518165.rev.compl.REF ------------------------------------------------------------ 31039

chimp.LOC112206744-LOC107973052-GGT2.revcompl                               ------------------------------------------------------------ 61762
chimp54546-135457.revcompl.Pan.troglodytes.clone.rp43-41g5.GenBank:AC099533.36 ------------------------------------------------------------ 68934
human.FAM30B-LOC105372935-GGT2.NCBI.                                        ccaaggccacacagctagtcctggataaattgacttaaagaacccaggtctctgattccc 88897
human.FAM230E-LOC105377182-GGT3P.NCBI.                                      ------------------------------------------------------------ 57310
human.FAM230J-LOC105372942-GGTLC5P.NCBI.rev.compl                           ------------------------------------------------------------ 46364
human.FAM230A.ncRNA.18487127.18500594-GGTLC3.18516335.18518165.rev.compl.REF ------------------------------------------------------------ 31039

chimp.LOC112206744-LOC107973052-GGT2.revcompl                               ------------------------------------------------------------ 61762
chimp54546-135457.revcompl.Pan.troglodytes.clone.rp43-41g5.GenBank:AC099533.36 ------------------------------------------------------------ 68934
human.FAM30B-LOC105372935-GGT2.NCBI.                                        aaatcagtgctcttacccctcccacgccacctctctcttctaaagatccacccaatggaa 88957
human.FAM230E-LOC105377182-GGT3P.NCBI.                                      ------------------------------------------------------------ 57310
human.FAM230J-LOC105372942-GGTLC5P.NCBI.rev.compl                           ------------------------------------------------------------ 46364
human.FAM230A.ncRNA.18487127.18500594-GGTLC3.18516335.18518165.rev.compl.REF ------------------------------------------------------------ 31039

chimp.LOC112206744-LOC107973052-GGT2.revcompl                               ------------------------------------------------------------ 61762
chimp54546-135457.revcompl.Pan.troglodytes.clone.rp43-41g5.GenBank:AC099533.36 ------------------------------------------------------------ 68934
human.FAM30B-LOC105372935-GGT2.NCBI.                                        ctgccccagggctggtatcccagctacttctccattcatgccgcaggtcaatgattcagg 89017
human.FAM230E-LOC105377182-GGT3P.NCBI.                                      ------------------------------------------------------------ 57310
human.FAM230J-LOC105372942-GGTLC5P.NCBI.rev.compl                           ------------------------------------------------------------ 46364
human.FAM230A.ncRNA.18487127.18500594-GGTLC3.18516335.18518165.rev.compl.REF ------------------------------------------------------------ 31039

chimp.LOC112206744-LOC107973052-GGT2.revcompl                               ------------------------------------------------------------ 61762
chimp54546-135457.revcompl.Pan.troglodytes.clone.rp43-41g5.GenBank:AC099533.36 ------------------------------------------------------------ 68934
human.FAM30B-LOC105372935-GGT2.NCBI.                                        gcggacactcctcaagtgaccttcagaggccctgactcactcctccgacagtcctttgtg 89077
human.FAM230E-LOC105377182-GGT3P.NCBI.                                      ------------------------------------------------------------ 57310
human.FAM230J-LOC105372942-GGTLC5P.NCBI.rev.compl                           ------------------------------------------------------------ 46364
human.FAM230A.ncRNA.18487127.18500594-GGTLC3.18516335.18518165.rev.compl.REF ------------------------------------------------------------ 31039

chimp.LOC112206744-LOC107973052-GGT2.revcompl                               ------------------------------------------------------------ 61762
chimp54546-135457.revcompl.Pan.troglodytes.clone.rp43-41g5.GenBank:AC099533.36 ------------------------------------------------------------ 68934
human.FAM30B-LOC105372935-GGT2.NCBI.                                        tctggactcccagccgctcctcagcagctctgtcctcttttgtacatttattactgtcag 89137
human.FAM230E-LOC105377182-GGT3P.NCBI.                                      ------------------------------------------------------------ 57310
human.FAM230J-LOC105372942-GGTLC5P.NCBI.rev.compl                           ------------------------------------------------------------ 46364
human.FAM230A.ncRNA.18487127.18500594-GGTLC3.18516335.18518165.rev.compl.REF ------------------------------------------------------------ 31039

chimp.LOC112206744-LOC107973052-GGT2.revcompl                               ------------------------------------------------------------ 61762
chimp54546-135457.revcompl.Pan.troglodytes.clone.rp43-41g5.GenBank:AC099533.36 ------------------------------------------------------------ 68934
human.FAM30B-LOC105372935-GGT2.NCBI.                                        cctgaggctagcaggaggtctcaatgcctggtgatctgaggccagcctgtcccaggccct 89197
human.FAM230E-LOC105377182-GGT3P.NCBI.                                      ------------------------------------------------------------ 57310
human.FAM230J-LOC105372942-GGTLC5P.NCBI.rev.compl                           ------------------------------------------------------------ 46364
human.FAM230A.ncRNA.18487127.18500594-GGTLC3.18516335.18518165.rev.compl.REF ------------------------------------------------------------ 31039

chimp.LOC112206744-LOC107973052-GGT2.revcompl                               ------------------------------------------------------------ 61762
chimp54546-135457.revcompl.Pan.troglodytes.clone.rp43-41g5.GenBank:AC099533.36 ------------------------------------------------------------ 68934
human.FAM30B-LOC105372935-GGT2.NCBI.                                        cccagtcccgtgaggtcctcctggggatgcaggcacagcgactgacaaggataaggagca 89257
human.FAM230E-LOC105377182-GGT3P.NCBI.                                      ------------------------------------------------------------ 57310
human.FAM230J-LOC105372942-GGTLC5P.NCBI.rev.compl                           ------------------------------------------------------------ 46364
human.FAM230A.ncRNA.18487127.18500594-GGTLC3.18516335.18518165.rev.compl.REF ------------------------------------------------------------ 31039

chimp.LOC112206744-LOC107973052-GGT2.revcompl                               ------------------------------------------------------------ 61762
chimp54546-135457.revcompl.Pan.troglodytes.clone.rp43-41g5.GenBank:AC099533.36 ------------------------------------------------------------ 68934
human.FAM30B-LOC105372935-GGT2.NCBI.                                        gaagggcaggtggctttctgggtgtgagcttggccttctgccacacccatgttcagaaag 89317
human.FAM230E-LOC105377182-GGT3P.NCBI.                                      ------------------------------------------------------------ 57310
human.FAM230J-LOC105372942-GGTLC5P.NCBI.rev.compl                           ------------------------------------------------------------ 46364
human.FAM230A.ncRNA.18487127.18500594-GGTLC3.18516335.18518165.rev.compl.REF ------------------------------------------------------------ 31039

chimp.LOC112206744-LOC107973052-GGT2.revcompl                               ------------------------------------------------------------ 61762
chimp54546-135457.revcompl.Pan.troglodytes.clone.rp43-41g5.GenBank:AC099533.36 ------------------------------------------------------------ 68934
human.FAM30B-LOC105372935-GGT2.NCBI.                                        gtgtctacgggcacatcttgtgttgtcacaggtgtagaaagggctttgacacgcggcaga 89377
human.FAM230E-LOC105377182-GGT3P.NCBI.                                      ------------------------------------------------------------ 57310
human.FAM230J-LOC105372942-GGTLC5P.NCBI.rev.compl                           ------------------------------------------------------------ 46364
human.FAM230A.ncRNA.18487127.18500594-GGTLC3.18516335.18518165.rev.compl.REF ------------------------------------------------------------ 31039
```

```
chimp.LOC112206744-LOC107973052-GGT2.revcompl                                         ------------------------------------------------------------ 61762
chimp54546-135457.revcompl.Pan.troglodytes.clone.rp43-41g5.GenBank:AC099533.36        ------------------------------------------------------------ 68934
human.FAM30B-LOC105372935-GGT2.NCBI.                                                  tcctccaatccctgtgaaagagggatcattctttctattatacagtgaggacgctgaagt 89437
human.FAM230E-LOC105377182-GGT3P.NCBI.                                                ------------------------------------------------------------ 57310
human.FAM230J-LOC105372942-GGTLC5P.NCBI.rev.compl                                     ------------------------------------------------------------ 46364
human.FAM230A.ncRNA.18487127.18500594-GGTLC3.18516335.18518165.rev.compl.REF          ------------------------------------------------------------ 31039

chimp.LOC112206744-LOC107973052-GGT2.revcompl                                         ------------------------------------------------------------ 61762
chimp54546-135457.revcompl.Pan.troglodytes.clone.rp43-41g5.GenBank:AC099533.36        ------------------------------------------------------------ 68934
human.FAM30B-LOC105372935-GGT2.NCBI.                                                  ccagaaaggtcaggtgactgatgaaaggtcataaaattagcaggaatcagggaaagggca 89497
human.FAM230E-LOC105377182-GGT3P.NCBI.                                                ------------------------------------------------------------ 57310
human.FAM230J-LOC105372942-GGTLC5P.NCBI.rev.compl                                     ------------------------------------------------------------ 46364
human.FAM230A.ncRNA.18487127.18500594-GGTLC3.18516335.18518165.rev.compl.REF          ------------------------------------------------------------ 31039

chimp.LOC112206744-LOC107973052-GGT2.revcompl                                         ------------------------------------------------------------ 61762
chimp54546-135457.revcompl.Pan.troglodytes.clone.rp43-41g5.GenBank:AC099533.36        ------------------------------------------------------------ 68934
human.FAM30B-LOC105372935-GGT2.NCBI.                                                  ggaaagcagagcttccagttgcaaggtcaggactcttcagcccagcaggcgagtttgcca 89557
human.FAM230E-LOC105377182-GGT3P.NCBI.                                                ------------------------------------------------------------ 57310
human.FAM230J-LOC105372942-GGTLC5P.NCBI.rev.compl                                     ------------------------------------------------------------ 46364
human.FAM230A.ncRNA.18487127.18500594-GGTLC3.18516335.18518165.rev.compl.REF          ------------------------------------------------------------ 31039

chimp.LOC112206744-LOC107973052-GGT2.revcompl                                         ------------------------------------------------------------ 61762
chimp54546-135457.revcompl.Pan.troglodytes.clone.rp43-41g5.GenBank:AC099533.36        ------------------------------------------------------------ 68934
human.FAM30B-LOC105372935-GGT2.NCBI.                                                  gccagtctgtgccctggtctctgccctccccaaacacgtgcaccaatttggaggggctgc 89617
human.FAM230E-LOC105377182-GGT3P.NCBI.                                                ------------------------------------------------------------ 57310
human.FAM230J-LOC105372942-GGTLC5P.NCBI.rev.compl                                     ------------------------------------------------------------ 46364
human.FAM230A.ncRNA.18487127.18500594-GGTLC3.18516335.18518165.rev.compl.REF          ------------------------------------------------------------ 31039

chimp.LOC112206744-LOC107973052-GGT2.revcompl                                         ------------------------------------------------------------ 61762
chimp54546-135457.revcompl.Pan.troglodytes.clone.rp43-41g5.GenBank:AC099533.36        ------------------------------------------------------------ 68934
human.FAM30B-LOC105372935-GGT2.NCBI.                                                  tcccagcacgcatgctgcaagataaacttcagtgctcagccctctcactgtgacaacaat 89677
human.FAM230E-LOC105377182-GGT3P.NCBI.                                                ------------------------------------------------------------ 57310
human.FAM230J-LOC105372942-GGTLC5P.NCBI.rev.compl                                     ------------------------------------------------------------ 46364
human.FAM230A.ncRNA.18487127.18500594-GGTLC3.18516335.18518165.rev.compl.REF          ------------------------------------------------------------ 31039

chimp.LOC112206744-LOC107973052-GGT2.revcompl                                         ------------------------------------------------------------ 61762
chimp54546-135457.revcompl.Pan.troglodytes.clone.rp43-41g5.GenBank:AC099533.36        ------------------------------------------------------------ 68934
human.FAM30B-LOC105372935-GGT2.NCBI.                                                  cccttagaaaggggtccacgctgtccatctgggcccctacaccaaagaacacctaattat 89737
human.FAM230E-LOC105377182-GGT3P.NCBI.                                                ------------------------------------------------------------ 57310
human.FAM230J-LOC105372942-GGTLC5P.NCBI.rev.compl                                     ------------------------------------------------------------ 46364
human.FAM230A.ncRNA.18487127.18500594-GGTLC3.18516335.18518165.rev.compl.REF          ------------------------------------------------------------ 31039

chimp.LOC112206744-LOC107973052-GGT2.revcompl                                         ------------------------------------------------------------ 61762
chimp54546-135457.revcompl.Pan.troglodytes.clone.rp43-41g5.GenBank:AC099533.36        ------------------------------------------------------------ 68934
human.FAM30B-LOC105372935-GGT2.NCBI.                                                  cagtggtcctcactcaggaaacatgcccacaaataaagacaaaagtaaacccaactacac 89797
human.FAM230E-LOC105377182-GGT3P.NCBI.                                                ------------------------------------------------------------ 57310
human.FAM230J-LOC105372942-GGTLC5P.NCBI.rev.compl                                     ------------------------------------------------------------ 46364
human.FAM230A.ncRNA.18487127.18500594-GGTLC3.18516335.18518165.rev.compl.REF          ------------------------------------------------------------ 31039

chimp.LOC112206744-LOC107973052-GGT2.revcompl                                         ------------------------------------------------------------ 61762
chimp54546-135457.revcompl.Pan.troglodytes.clone.rp43-41g5.GenBank:AC099533.36        ------------------------------------------------------------ 68934
human.FAM30B-LOC105372935-GGT2.NCBI.                                                  attcctcaaaatcctcctctccatttcacagaaacaccacaacagagcagaactgcaacc 89857
human.FAM230E-LOC105377182-GGT3P.NCBI.                                                ------------------------------------------------------------ 57310
human.FAM230J-LOC105372942-GGTLC5P.NCBI.rev.compl                                     ------------------------------------------------------------ 46364
human.FAM230A.ncRNA.18487127.18500594-GGTLC3.18516335.18518165.rev.compl.REF          ------------------------------------------------------------ 31039

chimp.LOC112206744-LOC107973052-GGT2.revcompl                                         ------------------------------------------------------------ 61762
chimp54546-135457.revcompl.Pan.troglodytes.clone.rp43-41g5.GenBank:AC099533.36        ------------------------------------------------------------ 68934
human.FAM30B-LOC105372935-GGT2.NCBI.                                                  tgatagcatccctgtctggttttggctctttcaaaacatcagggacctcacaggaaagca 89917
human.FAM230E-LOC105377182-GGT3P.NCBI.                                                ------------------------------------------------------------ 57310
human.FAM230J-LOC105372942-GGTLC5P.NCBI.rev.compl                                     ------------------------------------------------------------ 46364
human.FAM230A.ncRNA.18487127.18500594-GGTLC3.18516335.18518165.rev.compl.REF          ------------------------------------------------------------ 31039

chimp.LOC112206744-LOC107973052-GGT2.revcompl                                         ------------------------------------------------------------ 61762
chimp54546-135457.revcompl.Pan.troglodytes.clone.rp43-41g5.GenBank:AC099533.36        ------------------------------------------------------------ 68934
human.FAM30B-LOC105372935-GGT2.NCBI.                                                  atgcagaaaactgcagtcatttggccgcttgttaaggagagccctgagcagcaagctcca 89977
human.FAM230E-LOC105377182-GGT3P.NCBI.                                                ------------------------------------------------------------ 57310
human.FAM230J-LOC105372942-GGTLC5P.NCBI.rev.compl                                     ------------------------------------------------------------ 46364
human.FAM230A.ncRNA.18487127.18500594-GGTLC3.18516335.18518165.rev.compl.REF          ------------------------------------------------------------ 31039

chimp.LOC112206744-LOC107973052-GGT2.revcompl                                         ------------------------------------------------------------ 61762
chimp54546-135457.revcompl.Pan.troglodytes.clone.rp43-41g5.GenBank:AC099533.36        ------------------------------------------------------------ 68934
human.FAM30B-LOC105372935-GGT2.NCBI.                                                  gaccggagatgcgcatataacagcagcagttccacacagccctgcacagcagcaattcag 90037
human.FAM230E-LOC105377182-GGT3P.NCBI.                                                ------------------------------------------------------------ 57310
human.FAM230J-LOC105372942-GGTLC5P.NCBI.rev.compl                                     ------------------------------------------------------------ 46364
human.FAM230A.ncRNA.18487127.18500594-GGTLC3.18516335.18518165.rev.compl.REF          ------------------------------------------------------------ 31039

chimp.LOC112206744-LOC107973052-GGT2.revcompl                                         ------------------------------------------------------------ 61762
chimp54546-135457.revcompl.Pan.troglodytes.clone.rp43-41g5.GenBank:AC099533.36        ------------------------------------------------------------ 68934
human.FAM30B-LOC105372935-GGT2.NCBI.                                                  cctctggcagctgtgatgatgctgaggcagtgtccccactgccccagtggcttctgcatg 90097
human.FAM230E-LOC105377182-GGT3P.NCBI.                                                ------------------------------------------------------------ 57310
human.FAM230J-LOC105372942-GGTLC5P.NCBI.rev.compl                                     ------------------------------------------------------------ 46364
human.FAM230A.ncRNA.18487127.18500594-GGTLC3.18516335.18518165.rev.compl.REF          ------------------------------------------------------------ 31039

chimp.LOC112206744-LOC107973052-GGT2.revcompl                                         ------------------------------------------------------------ 61762
chimp54546-135457.revcompl.Pan.troglodytes.clone.rp43-41g5.GenBank:AC099533.36        ------------------------------------------------------------ 68934
human.FAM30B-LOC105372935-GGT2.NCBI.                                                  caggttcatttgggacaggacacctctaactccatctctggccaggagccagcatagtag 90157
human.FAM230E-LOC105377182-GGT3P.NCBI.                                                ------------------------------------------------------------ 57310
human.FAM230J-LOC105372942-GGTLC5P.NCBI.rev.compl                                     ------------------------------------------------------------ 46364
human.FAM230A.ncRNA.18487127.18500594-GGTLC3.18516335.18518165.rev.compl.REF          ------------------------------------------------------------ 31039

chimp.LOC112206744-LOC107973052-GGT2.revcompl                                         ------------------------------------------------------------ 61762
chimp54546-135457.revcompl.Pan.troglodytes.clone.rp43-41g5.GenBank:AC099533.36        ------------------------------------------------------------ 68934
human.FAM30B-LOC105372935-GGT2.NCBI.                                                  gtgccgtgaaagccaggaagtgaactgcacagaatggactgttccagtgtccatgggctg 90217
human.FAM230E-LOC105377182-GGT3P.NCBI.                                                ------------------------------------------------------------ 57310
human.FAM230J-LOC105372942-GGTLC5P.NCBI.rev.compl                                     ------------------------------------------------------------ 46364
human.FAM230A.ncRNA.18487127.18500594-GGTLC3.18516335.18518165.rev.compl.REF          ------------------------------------------------------------ 31039

chimp.LOC112206744-LOC107973052-GGT2.revcompl                                         ------------------------------------------------------------ 61762
chimp54546-135457.revcompl.Pan.troglodytes.clone.rp43-41g5.GenBank:AC099533.36        ------------------------------------------------------------ 68934
human.FAM30B-LOC105372935-GGT2.NCBI.                                                  caagtgggtcccacgtcacccacaggtgaatcttaattatgaaccaaggtgacggcagaa 90277
human.FAM230E-LOC105377182-GGT3P.NCBI.                                                ------------------------------------------------------------ 57310
human.FAM230J-LOC105372942-GGTLC5P.NCBI.rev.compl                                     ------------------------------------------------------------ 46364
human.FAM230A.ncRNA.18487127.18500594-GGTLC3.18516335.18518165.rev.compl.REF          ------------------------------------------------------------ 31039

chimp.LOC112206744-LOC107973052-GGT2.revcompl                                         ------------------------------------------------------------ 61762
chimp54546-135457.revcompl.Pan.troglodytes.clone.rp43-41g5.GenBank:AC099533.36        ------------------------------------------------------------ 68934
human.FAM30B-LOC105372935-GGT2.NCBI.                                                  aggatgggcagcaaaggagggtgtgtgtggtgatcagccatcagaggagacggccctgtg 90337
human.FAM230E-LOC105377182-GGT3P.NCBI.                                                ------------------------------------------------------------ 57310
human.FAM230J-LOC105372942-GGTLC5P.NCBI.rev.compl                                     ------------------------------------------------------------ 46364
human.FAM230A.ncRNA.18487127.18500594-GGTLC3.18516335.18518165.rev.compl.REF          ------------------------------------------------------------ 31039

chimp.LOC112206744-LOC107973052-GGT2.revcompl                                         ------------------------------------------------------------ 61762
chimp54546-135457.revcompl.Pan.troglodytes.clone.rp43-41g5.GenBank:AC099533.36        ------------------------------------------------------------ 68934
human.FAM30B-LOC105372935-GGT2.NCBI.                                                  tgatgaaaggacctcctcaggaaacctccccaccagcaactggctccaaatggtcagact 90397
human.FAM230E-LOC105377182-GGT3P.NCBI.                                                ------------------------------------------------------------ 57310
human.FAM230J-LOC105372942-GGTLC5P.NCBI.rev.compl                                     ------------------------------------------------------------ 46364
human.FAM230A.ncRNA.18487127.18500594-GGTLC3.18516335.18518165.rev.compl.REF          ------------------------------------------------------------ 31039

chimp.LOC112206744-LOC107973052-GGT2.revcompl                                         ------------------------------------------------------------ 61762
chimp54546-135457.revcompl.Pan.troglodytes.clone.rp43-41g5.GenBank:AC099533.36        ------------------------------------------------------------ 68934
human.FAM30B-LOC105372935-GGT2.NCBI.                                                  ttccaagaaattcctgtgaaaaggacaggctcagggcatgtgaatgtgatagtcagcaat 90457
human.FAM230E-LOC105377182-GGT3P.NCBI.                                                ------------------------------------------------------------ 57310
human.FAM230J-LOC105372942-GGTLC5P.NCBI.rev.compl                                     ------------------------------------------------------------ 46364
human.FAM230A.ncRNA.18487127.18500594-GGTLC3.18516335.18518165.rev.compl.REF          ------------------------------------------------------------ 31039

chimp.LOC112206744-LOC107973052-GGT2.revcompl                                         ------------------------------------------------------------ 61762
```

```
chimp54546-135457.revcompl.Pan.troglodytes.clone.rp43-41g5.GenBank:AC099533.36      ------------------------------------------------------------ 68934
human.FAM30B-LOC105372935-GGT2.NCBI.                                                tcgcacctcagcagccagtgacactatggtatagggaaaagaacttagattaggcatcaa 90517
human.FAM230E-LOC105377182-GGT3P.NCBI.                                              ------------------------------------------------------------ 57310
human.FAM230J-LOC105372942-GGTLC5P.NCBI.rev.compl                                   ------------------------------------------------------------ 46364
human.FAM230A.ncRNA.18487127.18500594-GGTLC3.18516335.18518165.rev.compl.REF        ------------------------------------------------------------ 31039

chimp.LOC112206744-LOC107973052-GGT2.revcompl                                       ------------------------------------------------------------ 61762
chimp54546-135457.revcompl.Pan.troglodytes.clone.rp43-41g5.GenBank:AC099533.36      ---------------------------------------------------------TGT 68937
human.FAM30B-LOC105372935-GGT2.NCBI.                                                aagtacccggtccccttcatacatggctggtggggaggtaggatggtgcagccactttgg 90577
human.FAM230E-LOC105377182-GGT3P.NCBI.                                              ------------------------------------------------------------ 57310
human.FAM230J-LOC105372942-GGTLC5P.NCBI.rev.compl                                   ------------------------------------------------------------ 46364
human.FAM230A.ncRNA.18487127.18500594-GGTLC3.18516335.18518165.rev.compl.REF        ------------------------------------------------------------ 31039

chimp.LOC112206744-LOC107973052-GGT2.revcompl                                       ------------------------------------------------------------ 61762
chimp54546-135457.revcompl.Pan.troglodytes.clone.rp43-41g5.GenBank:AC099533.36      GTATATATATGGAATATATATGTATATTTTGCCCATGACATAGCCCTCAGG-----AGAT 68992
human.FAM30B-LOC105372935-GGT2.NCBI.                                                aaaacagtctggtagtttctttcaaagttaccaaatgacccagcaatcccactcctaagt 90637
human.FAM230E-LOC105377182-GGT3P.NCBI.                                              ------------------------------------------------------------ 57310
human.FAM230J-LOC105372942-GGTLC5P.NCBI.rev.compl                                   ------------------------------------------------------------ 46364
human.FAM230A.ncRNA.18487127.18500594-GGTLC3.18516335.18518165.rev.compl.REF        ------------------------------------------------------------ 31039

chimp.LOC112206744-LOC107973052-GGT2.revcompl                                       ------------------------------------------------------------ 61762
chimp54546-135457.revcompl.Pan.troglodytes.clone.rp43-41g5.GenBank:AC099533.36      CCTTGACCATGTGCTGAAGGATATTTTTTCCCAAAGATATTTTTATAAATAAAAATAAAG 69052
human.FAM30B-LOC105372935-GGT2.NCBI.                                                atttacccaagagaaataaaacctgtgtccacacaaagacttgtacacatcctaatagct 90697
human.FAM230E-LOC105377182-GGT3P.NCBI.                                              ------------------------------------------------------------ 57310
human.FAM230J-LOC105372942-GGTLC5P.NCBI.rev.compl                                   ------------------------------------------------------------ 46364
human.FAM230A.ncRNA.18487127.18500594-GGTLC3.18516335.18518165.rev.compl.REF        ------------------------------------------------------------ 31039

chimp.LOC112206744-LOC107973052-GGT2.revcompl                                       ------------------------------------------------------------ 61762
chimp54546-135457.revcompl.Pan.troglodytes.clone.rp43-41g5.GenBank:AC099533.36      GGTAACAGAAGAGGTAGATGTGTGGGTAAATCTAAATGAATGTTATTGGTATAAAA---- 69108
human.FAM30B-LOC105372935-GGT2.NCBI.                                                gtattcatagtagccccaaactggaaacaacccaaatgcccatcagtttgtgaagagatg 90757
human.FAM230E-LOC105377182-GGT3P.NCBI.                                              ------------------------------------------------------------ 57310
human.FAM230J-LOC105372942-GGTLC5P.NCBI.rev.compl                                   ------------------------------------------------------------ 46364
human.FAM230A.ncRNA.18487127.18500594-GGTLC3.18516335.18518165.rev.compl.REF        ------------------------------------------------------------ 31039

chimp.LOC112206744-LOC107973052-GGT2.revcompl                                       ------------------------------------------------------------ 61762
chimp54546-135457.revcompl.Pan.troglodytes.clone.rp43-41g5.GenBank:AC099533.36      ------------------------------------------------------------ 69108
human.FAM30B-LOC105372935-GGT2.NCBI.                                                gacaaaatgtgctatatccataaaacggaaccctactcagcaataaaaaggtgtgaacta 90817
human.FAM230E-LOC105377182-GGT3P.NCBI.                                              ------------------------------------------------------------ 57310
human.FAM230J-LOC105372942-GGTLC5P.NCBI.rev.compl                                   ------------------------------------------------------------ 46364
human.FAM230A.ncRNA.18487127.18500594-GGTLC3.18516335.18518165.rev.compl.REF        ------------------------------------------------------------ 31039

chimp.LOC112206744-LOC107973052-GGT2.revcompl                                       ------------------------------------------------------------ 61762
chimp54546-135457.revcompl.Pan.troglodytes.clone.rp43-41g5.GenBank:AC099533.36      ------------------------------------------------------------ 69108
human.FAM30B-LOC105372935-GGT2.NCBI.                                                gatacacacaagaacatggagggatctcaaaagcactgtgctgagcgagagaagccagat 90877
human.FAM230E-LOC105377182-GGT3P.NCBI.                                              ------------------------------------------------------------ 57310
human.FAM230J-LOC105372942-GGTLC5P.NCBI.rev.compl                                   ------------------------------------------------------------ 46364
human.FAM230A.ncRNA.18487127.18500594-GGTLC3.18516335.18518165.rev.compl.REF        ------------------------------------------------------------ 31039

chimp.LOC112206744-LOC107973052-GGT2.revcompl                                       ------------------------------------------------------------ 61762
chimp54546-135457.revcompl.Pan.troglodytes.clone.rp43-41g5.GenBank:AC099533.36      ------------------------------------------------------------ 69108
human.FAM30B-LOC105372935-GGT2.NCBI.                                                tcaaagggctacacactgtaggatgccatgtgcatcacattctgtacagtgcaaaactgt 90937
human.FAM230E-LOC105377182-GGT3P.NCBI.                                              ------------------------------------------------------------ 57310
human.FAM230J-LOC105372942-GGTLC5P.NCBI.rev.compl                                   ------------------------------------------------------------ 46364
human.FAM230A.ncRNA.18487127.18500594-GGTLC3.18516335.18518165.rev.compl.REF        ------------------------------------------------------------ 31039

chimp.LOC112206744-LOC107973052-GGT2.revcompl                                       ------------------------------------------------------------ 61762
chimp54546-135457.revcompl.Pan.troglodytes.clone.rp43-41g5.GenBank:AC099533.36      ------------------------------------------------------------ 69108
human.FAM30B-LOC105372935-GGT2.NCBI.                                                agtggcaggattcaggtcccctgaggctggggtcctggaggcaggagaggggctggtagc 90997
human.FAM230E-LOC105377182-GGT3P.NCBI.                                              ------------------------------------------------------------ 57310
human.FAM230J-LOC105372942-GGTLC5P.NCBI.rev.compl                                   ------------------------------------------------------------ 46364
human.FAM230A.ncRNA.18487127.18500594-GGTLC3.18516335.18518165.rev.compl.REF        ------------------------------------------------------------ 31039

chimp.LOC112206744-LOC107973052-GGT2.revcompl                                       ------------------------------------------------------------ 61762
chimp54546-135457.revcompl.Pan.troglodytes.clone.rp43-41g5.GenBank:AC099533.36      ------------------------------------------------------------ 69108
human.FAM30B-LOC105372935-GGT2.NCBI.                                                aaggaggcacaagggaacttttggggacagaggagtggtccatatttgataggtggtgg 91057
human.FAM230E-LOC105377182-GGT3P.NCBI.                                              ------------------------------------------------------------ 57310
human.FAM230J-LOC105372942-GGTLC5P.NCBI.rev.compl                                   ------------------------------------------------------------ 46364
human.FAM230A.ncRNA.18487127.18500594-GGTLC3.18516335.18518165.rev.compl.REF        ------------------------------------------------------------ 31039

chimp.LOC112206744-LOC107973052-GGT2.revcompl                                       ------------------------------------------------------------ 61762
chimp54546-135457.revcompl.Pan.troglodytes.clone.rp43-41g5.GenBank:AC099533.36      ------------------------------------------------------------ 69108
human.FAM30B-LOC105372935-GGT2.NCBI.                                                ttgtgtggctgctcatgtttgtcaacaaagaattacatattttaaactggtgagttccgt 91117
human.FAM230E-LOC105377182-GGT3P.NCBI.                                              ------------------------------------------------------------ 57310
human.FAM230J-LOC105372942-GGTLC5P.NCBI.rev.compl                                   ------------------------------------------------------------ 46364
human.FAM230A.ncRNA.18487127.18500594-GGTLC3.18516335.18518165.rev.compl.REF        ------------------------------------------------------------ 31039

chimp.LOC112206744-LOC107973052-GGT2.revcompl                                       ------------------------------------------------------------ 61762
chimp54546-135457.revcompl.Pan.troglodytes.clone.rp43-41g5.GenBank:AC099533.36      ------------------------------------------------------------ 69108
human.FAM30B-LOC105372935-GGT2.NCBI.                                                tgtatgcaagcaaattataccacaatgaagcaaatctttaaaacactaggttccttttct 91177
human.FAM230E-LOC105377182-GGT3P.NCBI.                                              ------------------------------------------------------------ 57310
human.FAM230J-LOC105372942-GGTLC5P.NCBI.rev.compl                                   ------------------------------------------------------------ 46364
human.FAM230A.ncRNA.18487127.18500594-GGTLC3.18516335.18518165.rev.compl.REF        ------------------------------------------------------------ 31039

chimp.LOC112206744-LOC107973052-GGT2.revcompl                                       ------------------------------------------------------------ 61762
chimp54546-135457.revcompl.Pan.troglodytes.clone.rp43-41g5.GenBank:AC099533.36      ------------------------------------------------------------ 69108
human.FAM30B-LOC105372935-GGT2.NCBI.                                                ttatctagagctcaaagtagtggcccatgctggctatgacttggagaaatcacacaagct 91237
human.FAM230E-LOC105377182-GGT3P.NCBI.                                              ------------------------------------------------------------ 57310
human.FAM230J-LOC105372942-GGTLC5P.NCBI.rev.compl                                   ------------------------------------------------------------ 46364
human.FAM230A.ncRNA.18487127.18500594-GGTLC3.18516335.18518165.rev.compl.REF        ------------------------------------------------------------ 31039

chimp.LOC112206744-LOC107973052-GGT2.revcompl                                       ------------------------------------------------------------ 61762
chimp54546-135457.revcompl.Pan.troglodytes.clone.rp43-41g5.GenBank:AC099533.36      ------------------------------------------------------------ 69108
human.FAM30B-LOC105372935-GGT2.NCBI.                                                tactgggtctcaactgcttcatttgtacaggtggctgcataacactccccgggcaggga 91297
human.FAM230E-LOC105377182-GGT3P.NCBI.                                              ------------------------------------------------------------ 57310
human.FAM230J-LOC105372942-GGTLC5P.NCBI.rev.compl                                   ------------------------------------------------------------ 46364
human.FAM230A.ncRNA.18487127.18500594-GGTLC3.18516335.18518165.rev.compl.REF        ------------------------------------------------------------ 31039

chimp.LOC112206744-LOC107973052-GGT2.revcompl                                       ------------------------------------------------------------ 61762
chimp54546-135457.revcompl.Pan.troglodytes.clone.rp43-41g5.GenBank:AC099533.36      ------------------------------------------------------------ 69108
human.FAM30B-LOC105372935-GGT2.NCBI.                                                actgtagggatgaaatcagcaaatgtgagtgaacacacagaggaagcaacacataggcct 91357
human.FAM230E-LOC105377182-GGT3P.NCBI.                                              ------------------------------------------------------------ 57310
human.FAM230J-LOC105372942-GGTLC5P.NCBI.rev.compl                                   ------------------------------------------------------------ 46364
human.FAM230A.ncRNA.18487127.18500594-GGTLC3.18516335.18518165.rev.compl.REF        ------------------------------------------------------------ 31039

chimp.LOC112206744-LOC107973052-GGT2.revcompl                                       ------------------------------------------------------------ 61762
chimp54546-135457.revcompl.Pan.troglodytes.clone.rp43-41g5.GenBank:AC099533.36      ------------------------------------------------------------ 69108
human.FAM30B-LOC105372935-GGT2.NCBI.                                                tccccaaatacccacttctgttttctttcctcagtgtgagtaagtggctctaatgatcca 91417
human.FAM230E-LOC105377182-GGT3P.NCBI.                                              ------------------------------------------------------------ 57310
human.FAM230J-LOC105372942-GGTLC5P.NCBI.rev.compl                                   ------------------------------------------------------------ 46364
human.FAM230A.ncRNA.18487127.18500594-GGTLC3.18516335.18518165.rev.compl.REF        ------------------------------------------------------------ 31039

chimp.LOC112206744-LOC107973052-GGT2.revcompl                                       ------------------------------------------------------------ 61762
chimp54546-135457.revcompl.Pan.troglodytes.clone.rp43-41g5.GenBank:AC099533.36      ------------------------------------------------------------ 69108
human.FAM30B-LOC105372935-GGT2.NCBI.                                                caaaccttccaaccagccaagtcaaggaccagcaggaggactcaggaaagtactgaaatg 91477
human.FAM230E-LOC105377182-GGT3P.NCBI.                                              ------------------------------------------------------------ 57310
human.FAM230J-LOC105372942-GGTLC5P.NCBI.rev.compl                                   ------------------------------------------------------------ 46364
human.FAM230A.ncRNA.18487127.18500594-GGTLC3.18516335.18518165.rev.compl.REF        ------------------------------------------------------------ 31039

chimp.LOC112206744-LOC107973052-GGT2.revcompl                                       ------------------------------------------------------------ 61762
chimp54546-135457.revcompl.Pan.troglodytes.clone.rp43-41g5.GenBank:AC099533.36      ------------------------------------------------------------ 69108
human.FAM30B-LOC105372935-GGT2.NCBI.                                                ctggggagaaccaggacagcggctgagcacagccagacagggagggcagcctggtggggc 91537
human.FAM230E-LOC105377182-GGT3P.NCBI.                                              ------------------------------------------------------------ 57310
human.FAM230J-LOC105372942-GGTLC5P.NCBI.rev.compl                                   ------------------------------------------------------------ 46364
human.FAM230A.ncRNA.18487127.18500594-GGTLC3.18516335.18518165.rev.compl.REF        ------------------------------------------------------------ 31039

chimp.LOC112206744-LOC107973052-GGT2.revcompl                                       ------------------------------------------------------------ 61762
chimp54546-135457.revcompl.Pan.troglodytes.clone.rp43-41g5.GenBank:AC099533.36      ------------------------------------------------------------ 69108
```

```
human.FAM30B-LOC105372935-GGT2.NCBI.                                      cctgggagccatggacgcgactgcgctgggagtggggatgggagatagcaggagttggtg 91597
human.FAM230E-LOC105377182-GGT3P.NCBI.                                    ------------------------------------------------------------ 57310
human.FAM230J-LOC105372942-GGTLC5P.NCBI.rev.compl                         ------------------------------------------------------------ 46364
human.FAM230A.ncRNA.18487127.18500594-GGTLC3.18516335.18518165.rev.compl.REF ------------------------------------------------------------ 31039

chimp.LOC112206744-LOC107973052-GGT2.revcompl                             ------------------------------------------------------------ 61762
chimp54546-135457.revcompl.Pan.troglodytes.clone.rp43-41g5.GenBank:AC099533.36 ------------------------------------------------------------ 69108
human.FAM30B-LOC105372935-GGT2.NCBI.                                      ggtggggcacaaagtgggagccagggccaaattgctgctgatttgaccattttgcaaaag 91657
human.FAM230E-LOC105377182-GGT3P.NCBI.                                    ------------------------------------------------------------ 57310
human.FAM230J-LOC105372942-GGTLC5P.NCBI.rev.compl                         ------------------------------------------------------------ 46364
human.FAM230A.ncRNA.18487127.18500594-GGTLC3.18516335.18518165.rev.compl.REF ------------------------------------------------------------ 31039

chimp.LOC112206744-LOC107973052-GGT2.revcompl                             ------------------------------------------------------------ 61762
chimp54546-135457.revcompl.Pan.troglodytes.clone.rp43-41g5.GenBank:AC099533.36 -------------------------------TTACAGTAGTATAGGAAATGATATCTTGT 69137
human.FAM30B-LOC105372935-GGT2.NCBI.                                      gccagcctggctgtggcctcaggcctctcatttcctctaccataatgaggggtcacggaa 91717
human.FAM230E-LOC105377182-GGT3P.NCBI.                                    ------------------------------------------------------------ 57310
human.FAM230J-LOC105372942-GGTLC5P.NCBI.rev.compl                         ------------------------------------------------------------ 46364
human.FAM230A.ncRNA.18487127.18500594-GGTLC3.18516335.18518165.rev.compl.REF ------------------------------------------------------------ 31039

chimp.LOC112206744-LOC107973052-GGT2.revcompl                             ------------------------------------------------------------ 61762
chimp54546-135457.revcompl.Pan.troglodytes.clone.rp43-41g5.GenBank:AC099533.36 GGGGTTTAAAATAAATGAAACATACTGAAATATGTATGGGTACAGTTATATATCTGGGAT 69197
human.FAM30B-LOC105372935-GGT2.NCBI.                                      gtgccttcccacaactgcagcatctccctccacatttgggctcaaatacagagtggggcc 91777
human.FAM230E-LOC105377182-GGT3P.NCBI.                                    ------------------------------------------------------------ 57310
human.FAM230J-LOC105372942-GGTLC5P.NCBI.rev.compl                         ------------------------------------------------------------ 46364
human.FAM230A.ncRNA.18487127.18500594-GGTLC3.18516335.18518165.rev.compl.REF ------------------------------------------------------------ 31039

chimp.LOC112206744-LOC107973052-GGT2.revcompl                             ------------------------------------------------------------ 61762
chimp54546-135457.revcompl.Pan.troglodytes.clone.rp43-41g5.GenBank:AC099533.36 TTGCACTGAAATAGTGTGGG---------------------------------------- 69217
human.FAM30B-LOC105372935-GGT2.NCBI.                                      ggagacagaaaaagtctccgtgacacactgtgatttgtttctgcactgtgtgaacccttg 91837
human.FAM230E-LOC105377182-GGT3P.NCBI.                                    ------------------------------------------------------------ 57310
human.FAM230J-LOC105372942-GGTLC5P.NCBI.rev.compl                         ------------------------------------------------------------ 46364
human.FAM230A.ncRNA.18487127.18500594-GGTLC3.18516335.18518165.rev.compl.REF ------------------------------------------------------------ 31039

chimp.LOC112206744-LOC107973052-GGT2.revcompl                             ------------------------------------------------------------ 61762
chimp54546-135457.revcompl.Pan.troglodytes.clone.rp43-41g5.GenBank:AC099533.36 ------------------------------------------------------------ 69217
human.FAM30B-LOC105372935-GGT2.NCBI.                                      gctctgtgacacagaacacaggtttcagcagttcctgtcacttcccacaggcgaaacaga 91897
human.FAM230E-LOC105377182-GGT3P.NCBI.                                    ------------------------------------------------------------ 57310
human.FAM230J-LOC105372942-GGTLC5P.NCBI.rev.compl                         ------------------------------------------------------------ 46364
human.FAM230A.ncRNA.18487127.18500594-GGTLC3.18516335.18518165.rev.compl.REF ------------------------------------------------------------ 31039

chimp.LOC112206744-LOC107973052-GGT2.revcompl                             ------------------------------------------------------------ 61762
chimp54546-135457.revcompl.Pan.troglodytes.clone.rp43-41g5.GenBank:AC099533.36 ------------------------------------------------------------ 69217
human.FAM30B-LOC105372935-GGT2.NCBI.                                      cagagcattcttttgtccggcttctggtgaaacgccattatcgttatttttaggaggaag 91957
human.FAM230E-LOC105377182-GGT3P.NCBI.                                    ------------------------------------------------------------ 57310
human.FAM230J-LOC105372942-GGTLC5P.NCBI.rev.compl                         ------------------------------------------------------------ 46364
human.FAM230A.ncRNA.18487127.18500594-GGTLC3.18516335.18518165.rev.compl.REF ------------------------------------------------------------ 31039

chimp.LOC112206744-LOC107973052-GGT2.revcompl                             ------------------------------------------------------------ 61762
chimp54546-135457.revcompl.Pan.troglodytes.clone.rp43-41g5.GenBank:AC099533.36 ----------GTAGAGGGAAGCAGGAAAGAGGATACATGAAATTAGCTTGGCCATAAGAT 69267
human.FAM30B-LOC105372935-GGT2.NCBI.                                      atggagaattggggtgggtccccacaaagaggatggattcagaagacttccaggaacc-- 92015
human.FAM230E-LOC105377182-GGT3P.NCBI.                                    ------------------------------------------------------------ 57310
human.FAM230J-LOC105372942-GGTLC5P.NCBI.rev.compl                         ------------------------------------------------------------ 46364
human.FAM230A.ncRNA.18487127.18500594-GGTLC3.18516335.18518165.rev.compl.REF ------------------------------------------------------------ 31039

chimp.LOC112206744-LOC107973052-GGT2.revcompl                             ------------------------------------------------------------ 61762
chimp54546-135457.revcompl.Pan.troglodytes.clone.rp43-41g5.GenBank:AC099533.36 TGTTGTTGAAATTGAATGGATACTCGGGGCTTCATTACACAATTCTCTTTACTCTTACAT 69327
human.FAM30B-LOC105372935-GGT2.NCBI.                                      ------ttaaacggcttggaaactaattgtgccagttctttttgatgtctaattttattc 92069
human.FAM230E-LOC105377182-GGT3P.NCBI.                                    ------------------------------------------------------------ 57310
human.FAM230J-LOC105372942-GGTLC5P.NCBI.rev.compl                         ------------------------------------------------------------ 46364
human.FAM230A.ncRNA.18487127.18500594-GGTLC3.18516335.18518165.rev.compl.REF ------------------------------------------------------------ 31039

chimp.LOC112206744-LOC107973052-GGT2.revcompl                             ------------------------------------------------------------ 61762
chimp54546-135457.revcompl.Pan.troglodytes.clone.rp43-41g5.GenBank:AC099533.36 AGCTCTACACTCTCAACATAAATAAGAATAAAAACACAAAAAACACACAGATACATCTAT 69387
human.FAM30B-LOC105372935-GGT2.NCBI.                                      taaaatttcctctcagcacaatatattaatactgatcagccaagagaaggttaggttttg 92129
human.FAM230E-LOC105377182-GGT3P.NCBI.                                    ------------------------------------------------------------ 57310
human.FAM230J-LOC105372942-GGTLC5P.NCBI.rev.compl                         ------------------------------------------------------------ 46364
human.FAM230A.ncRNA.18487127.18500594-GGTLC3.18516335.18518165.rev.compl.REF ------------------------------------------------------------ 31039

chimp.LOC112206744-LOC107973052-GGT2.revcompl                             ------------------------------------------------------------ 61762
chimp54546-135457.revcompl.Pan.troglodytes.clone.rp43-41g5.GenBank:AC099533.36 GCACACACATATATTTAAAATACACAAAAATATTAGCATATAAGTCACTGGGGGTAAATT 69447
human.FAM30B-LOC105372935-GGT2.NCBI.                                      gctcacaaata-gtcaaggagagaatctccttcattcattgagaaggcagaagatggcgt 92188
human.FAM230E-LOC105377182-GGT3P.NCBI.                                    ------------------------------------------------------------ 57310
human.FAM230J-LOC105372942-GGTLC5P.NCBI.rev.compl                         ------------------------------------------------------------ 46364
human.FAM230A.ncRNA.18487127.18500594-GGTLC3.18516335.18518165.rev.compl.REF ------------------------------------------------------------ 31039

chimp.LOC112206744-LOC107973052-GGT2.revcompl                             ------------------------------------------------------------ 61762
chimp54546-135457.revcompl.Pan.troglodytes.clone.rp43-41g5.GenBank:AC099533.36 TAGTTCCTGTTCCAAGGTTCTTGTACTGACTAGGAAGAGGATAGAAGTACT--------- 69498
human.FAM30B-LOC105372935-GGT2.NCBI.                                      taaagagtattcacaggtaactaagattgccagagggagaatcgagtgagttgtcagtgg 92248
human.FAM230E-LOC105377182-GGT3P.NCBI.                                    ------------------------------------------------------------ 57310
human.FAM230J-LOC105372942-GGTLC5P.NCBI.rev.compl                         ------------------------------------------------------------ 46364
human.FAM230A.ncRNA.18487127.18500594-GGTLC3.18516335.18518165.rev.compl.REF ------------------------------------------------------------ 31039

chimp.LOC112206744-LOC107973052-GGT2.revcompl                             ------------------------------------------------------------ 61762
chimp54546-135457.revcompl.Pan.troglodytes.clone.rp43-41g5.GenBank:AC099533.36 ------------------------------------------------------------ 69498
human.FAM30B-LOC105372935-GGT2.NCBI.                                      tgaaatacagaggaggtagagagagctacactgagacaacaaaggcagcttaagggaaca 92308
human.FAM230E-LOC105377182-GGT3P.NCBI.                                    ------------------------------------------------------------ 57310
human.FAM230J-LOC105372942-GGTLC5P.NCBI.rev.compl                         ------------------------------------------------------------ 46364
human.FAM230A.ncRNA.18487127.18500594-GGTLC3.18516335.18518165.rev.compl.REF ------------------------------------------------------------ 31039

chimp.LOC112206744-LOC107973052-GGT2.revcompl                             ------------------------------------------------------------ 61762
chimp54546-135457.revcompl.Pan.troglodytes.clone.rp43-41g5.GenBank:AC099533.36 ------------------------------------------------------------ 69498
human.FAM30B-LOC105372935-GGT2.NCBI.                                      aagccagctaagggaacaaagccggggtccctcaggtctgtggccaccggctccagccag 92368
human.FAM230E-LOC105377182-GGT3P.NCBI.                                    ------------------------------------------------------------ 57310
human.FAM230J-LOC105372942-GGTLC5P.NCBI.rev.compl                         ------------------------------------------------------------ 46364
human.FAM230A.ncRNA.18487127.18500594-GGTLC3.18516335.18518165.rev.compl.REF ------------------------------------------------------------ 31039

chimp.LOC112206744-LOC107973052-GGT2.revcompl                             ------------------------------------------------------------ 61762
chimp54546-135457.revcompl.Pan.troglodytes.clone.rp43-41g5.GenBank:AC099533.36 ------------------------------------------------------------ 69498
human.FAM30B-LOC105372935-GGT2.NCBI.                                      gacagcctggtagctgaagagcacatccttgcccgttacatgcctgtgtttcaagtgatg 92428
human.FAM230E-LOC105377182-GGT3P.NCBI.                                    ------------------------------------------------------------ 57310
human.FAM230J-LOC105372942-GGTLC5P.NCBI.rev.compl                         ------------------------------------------------------------ 46364
human.FAM230A.ncRNA.18487127.18500594-GGTLC3.18516335.18518165.rev.compl.REF ------------------------------------------------------------ 31039

chimp.LOC112206744-LOC107973052-GGT2.revcompl                             ------------------------------------------------------------ 61762
chimp54546-135457.revcompl.Pan.troglodytes.clone.rp43-41g5.GenBank:AC099533.36 ------------------------------------------------------------ 69498
human.FAM30B-LOC105372935-GGT2.NCBI.                                      ctaacttgctaatgcagatatctccccaaaccctgcccctggctcaccacccagtgagca 92488
human.FAM230E-LOC105377182-GGT3P.NCBI.                                    ------------------------------------------------------------ 57310
human.FAM230J-LOC105372942-GGTLC5P.NCBI.rev.compl                         ------------------------------------------------------------ 46364
human.FAM230A.ncRNA.18487127.18500594-GGTLC3.18516335.18518165.rev.compl.REF ------------------------------------------------------------ 31039

chimp.LOC112206744-LOC107973052-GGT2.revcompl                             ------------------------------------------------------------ 61762
chimp54546-135457.revcompl.Pan.troglodytes.clone.rp43-41g5.GenBank:AC099533.36 ------------------------------------------------------------ 69498
human.FAM30B-LOC105372935-GGT2.NCBI.                                      atcactcactttccccaggacaccttcagggaggtactctgccagcaccgcacacttaaa 92548
human.FAM230E-LOC105377182-GGT3P.NCBI.                                    ------------------------------------------------------------ 57310
human.FAM230J-LOC105372942-GGTLC5P.NCBI.rev.compl                         ------------------------------------------------------------ 46364
human.FAM230A.ncRNA.18487127.18500594-GGTLC3.18516335.18518165.rev.compl.REF ------------------------------------------------------------ 31039

chimp.LOC112206744-LOC107973052-GGT2.revcompl                             ------------------------------------------------------------ 61762
chimp54546-135457.revcompl.Pan.troglodytes.clone.rp43-41g5.GenBank:AC099533.36 ------------------------------------------------------------ 69498
human.FAM30B-LOC105372935-GGT2.NCBI.                                      cagctactgcaggttcttctctcagacgcagcacggttcctgagatgacgtcagcgctgc 92608
human.FAM230E-LOC105377182-GGT3P.NCBI.                                    ------------------------------------------------------------ 57310
human.FAM230J-LOC105372942-GGTLC5P.NCBI.rev.compl                         ------------------------------------------------------------ 46364
human.FAM230A.ncRNA.18487127.18500594-GGTLC3.18516335.18518165.rev.compl.REF ------------------------------------------------------------ 31039

chimp.LOC112206744-LOC107973052-GGT2.revcompl                             ------------------------------------------------------------ 61762
chimp54546-135457.revcompl.Pan.troglodytes.clone.rp43-41g5.GenBank:AC099533.36 ------------------------------------------------------------ 69498
human.FAM30B-LOC105372935-GGT2.NCBI.                                      tcgctgaaagcaccaggcaaggaggcaaaacaaaggaaatcctaaagagcaggcgagaga 92668
```

```
human.FAM230E-LOC105377182-GGT3P.NCBI.  ------------------------------------------------------------ 57310
human.FAM230J-LOC105372942-GGTLC5P.NCBI.rev.compl  ------------------------------------------------------------ 46364
human.FAM230A.ncRNA.18487127.18500594-GGTLC3.18516335.18518165.rev.compl.REF  ------------------------------------------------------------ 31039

chimp.LOC112206744-LOC107973052-GGT2.revcompl  ------------------------------------------------------------ 61762
chimp54546-135457.revcompl.Pan.troglodytes.clone.rp43-41g5.GenBank:AC099533.36  ------------------------------------------------------------ 69498
human.FAM30B-LOC105372935-GGT2.NCBI.  acagtggaactggcagagagaggcactggtgcggatggagagggcagcaataaaaactgg 92728
human.FAM230E-LOC105377182-GGT3P.NCBI.  ------------------------------------------------------------ 57310
human.FAM230J-LOC105372942-GGTLC5P.NCBI.rev.compl  ------------------------------------------------------------ 46364
human.FAM230A.ncRNA.18487127.18500594-GGTLC3.18516335.18518165.rev.compl.REF  ------------------------------------------------------------ 31039

chimp.LOC112206744-LOC107973052-GGT2.revcompl  ------------------------------------------------------------ 61762
chimp54546-135457.revcompl.Pan.troglodytes.clone.rp43-41g5.GenBank:AC099533.36  ------------------------------------------------------------ 69498
human.FAM30B-LOC105372935-GGT2.NCBI.  gggacacttagaggcaaaggtatggcaggtgacaaaaaagatgagctgaagctcaggaga 92788
human.FAM230E-LOC105377182-GGT3P.NCBI.  ------------------------------------------------------------ 57310
human.FAM230J-LOC105372942-GGTLC5P.NCBI.rev.compl  ------------------------------------------------------------ 46364
human.FAM230A.ncRNA.18487127.18500594-GGTLC3.18516335.18518165.rev.compl.REF  ------------------------------------------------------------ 31039

chimp.LOC112206744-LOC107973052-GGT2.revcompl  ------------------------------------------------------------ 61762
chimp54546-135457.revcompl.Pan.troglodytes.clone.rp43-41g5.GenBank:AC099533.36  ------------------------------------------------------------ 69498
human.FAM30B-LOC105372935-GGT2.NCBI.  gacccaggcagcgatgcccaaatgaccggctgaggaatcacctcactgctgctgatgccc 92848
human.FAM230E-LOC105377182-GGT3P.NCBI.  ------------------------------------------------------------ 57310
human.FAM230J-LOC105372942-GGTLC5P.NCBI.rev.compl  ------------------------------------------------------------ 46364
human.FAM230A.ncRNA.18487127.18500594-GGTLC3.18516335.18518165.rev.compl.REF  ------------------------------------------------------------ 31039

chimp.LOC112206744-LOC107973052-GGT2.revcompl  ------------------------------------------------------------ 61762
chimp54546-135457.revcompl.Pan.troglodytes.clone.rp43-41g5.GenBank:AC099533.36  ------------------------------------------------------------ 69498
human.FAM30B-LOC105372935-GGT2.NCBI.  ttgacagcgatgtctgaggctggcgtgagtgctccatgctcagagatacctagaacccag 92908
human.FAM230E-LOC105377182-GGT3P.NCBI.  ------------------------------------------------------------ 57310
human.FAM230J-LOC105372942-GGTLC5P.NCBI.rev.compl  ------------------------------------------------------------ 46364
human.FAM230A.ncRNA.18487127.18500594-GGTLC3.18516335.18518165.rev.compl.REF  ------------------------------------------------------------ 31039

chimp.LOC112206744-LOC107973052-GGT2.revcompl  ------------------------------------------------------------ 61762
chimp54546-135457.revcompl.Pan.troglodytes.clone.rp43-41g5.GenBank:AC099533.36  ------------------------------------------------------------ 69498
human.FAM30B-LOC105372935-GGT2.NCBI.  ctacaatggccttatcagactgaaacaagaacaccaagatattccaagggaggtgacaat 92968
human.FAM230E-LOC105377182-GGT3P.NCBI.  ------------------------------------------------------------ 57310
human.FAM230J-LOC105372942-GGTLC5P.NCBI.rev.compl  ------------------------------------------------------------ 46364
human.FAM230A.ncRNA.18487127.18500594-GGTLC3.18516335.18518165.rev.compl.REF  ------------------------------------------------------------ 31039

chimp.LOC112206744-LOC107973052-GGT2.revcompl  ------------------------------------------------------------ 61762
chimp54546-135457.revcompl.Pan.troglodytes.clone.rp43-41g5.GenBank:AC099533.36  ------------------------------------------------------------ 69498
human.FAM30B-LOC105372935-GGT2.NCBI.  ggggtccaaaggttgtgcatttatggatcacaactatgtgctgagtgtggcccaaatccc 93028
human.FAM230E-LOC105377182-GGT3P.NCBI.  ------------------------------------------------------------ 57310
human.FAM230J-LOC105372942-GGTLC5P.NCBI.rev.compl  ------------------------------------------------------------ 46364
human.FAM230A.ncRNA.18487127.18500594-GGTLC3.18516335.18518165.rev.compl.REF  ------------------------------------------------------------ 31039

chimp.LOC112206744-LOC107973052-GGT2.revcompl  ------------------------------------------------------------ 61762
chimp54546-135457.revcompl.Pan.troglodytes.clone.rp43-41g5.GenBank:AC099533.36  ------------------------------------------------------------ 69498
human.FAM30B-LOC105372935-GGT2.NCBI.  agctccatatggtcacaccatgtctacagccaatcagctgaccggggtggcaacccctgg 93088
human.FAM230E-LOC105377182-GGT3P.NCBI.  ------------------------------------------------------------ 57310
human.FAM230J-LOC105372942-GGTLC5P.NCBI.rev.compl  ------------------------------------------------------------ 46364
human.FAM230A.ncRNA.18487127.18500594-GGTLC3.18516335.18518165.rev.compl.REF  ------------------------------------------------------------ 31039

chimp.LOC112206744-LOC107973052-GGT2.revcompl  ------------------------------------------------------------ 61762
chimp54546-135457.revcompl.Pan.troglodytes.clone.rp43-41g5.GenBank:AC099533.36  ------------------------------------------------------------ 69498
human.FAM30B-LOC105372935-GGT2.NCBI.  ctctgctcaacaataatgacaaccatcatttactgagcacttactatgtgccagaccgat 93148
human.FAM230E-LOC105377182-GGT3P.NCBI.  ------------------------------------------------------------ 57310
human.FAM230J-LOC105372942-GGTLC5P.NCBI.rev.compl  ------------------------------------------------------------ 46364
human.FAM230A.ncRNA.18487127.18500594-GGTLC3.18516335.18518165.rev.compl.REF  ------------------------------------------------------------ 31039

chimp.LOC112206744-LOC107973052-GGT2.revcompl  ------------------------------------------------------------ 61762
chimp54546-135457.revcompl.Pan.troglodytes.clone.rp43-41g5.GenBank:AC099533.36  ------------------------------------------------------------ 69498
human.FAM30B-LOC105372935-GGT2.NCBI.  gctaagtgctttcctcagatgtttccatttaactcttagaatcaccctaatgagtatctc 93208
human.FAM230E-LOC105377182-GGT3P.NCBI.  ------------------------------------------------------------ 57310
human.FAM230J-LOC105372942-GGTLC5P.NCBI.rev.compl  ------------------------------------------------------------ 46364
human.FAM230A.ncRNA.18487127.18500594-GGTLC3.18516335.18518165.rev.compl.REF  ------------------------------------------------------------ 31039

chimp.LOC112206744-LOC107973052-GGT2.revcompl  ------------------------------------------------------------ 61762
chimp54546-135457.revcompl.Pan.troglodytes.clone.rp43-41g5.GenBank:AC099533.36  ------------------------------------------------------------ 69498
human.FAM30B-LOC105372935-GGT2.NCBI.  ctattattttacagatgaggaaatgagagtttagaaaagtaacttgcaaaatcatactgc 93268
human.FAM230E-LOC105377182-GGT3P.NCBI.  ------------------------------------------------------------ 57310
human.FAM230J-LOC105372942-GGTLC5P.NCBI.rev.compl  ------------------------------------------------------------ 46364
human.FAM230A.ncRNA.18487127.18500594-GGTLC3.18516335.18518165.rev.compl.REF  ------------------------------------------------------------ 31039

chimp.LOC112206744-LOC107973052-GGT2.revcompl  ------------------------------------------------------------ 61762
chimp54546-135457.revcompl.Pan.troglodytes.clone.rp43-41g5.GenBank:AC099533.36  ------------------------------------------------------------ 69498
human.FAM30B-LOC105372935-GGT2.NCBI.  tggctccataacaaatgttgattgaatgttgtctgggtggacagaggcaaggatgaccgg 93328
human.FAM230E-LOC105377182-GGT3P.NCBI.  ------------------------------------------------------------ 57310
human.FAM230J-LOC105372942-GGTLC5P.NCBI.rev.compl  ------------------------------------------------------------ 46364
human.FAM230A.ncRNA.18487127.18500594-GGTLC3.18516335.18518165.rev.compl.REF  ------------------------------------------------------------ 31039

chimp.LOC112206744-LOC107973052-GGT2.revcompl  ------------------------------------------------------------ 61762
chimp54546-135457.revcompl.Pan.troglodytes.clone.rp43-41g5.GenBank:AC099533.36  ------------------------------------------------------------ 69498
human.FAM30B-LOC105372935-GGT2.NCBI.  aacgagacaaacaaacaaaaggctgactgacagaccattcgggtagcacttttcctggga 93388
human.FAM230E-LOC105377182-GGT3P.NCBI.  ------------------------------------------------------------ 57310
human.FAM230J-LOC105372942-GGTLC5P.NCBI.rev.compl  ------------------------------------------------------------ 46364
human.FAM230A.ncRNA.18487127.18500594-GGTLC3.18516335.18518165.rev.compl.REF  ------------------------------------------------------------ 31039

chimp.LOC112206744-LOC107973052-GGT2.revcompl  ------------------------------------------------------------ 61762
chimp54546-135457.revcompl.Pan.troglodytes.clone.rp43-41g5.GenBank:AC099533.36  --------------------------------------------------AACTCATAGG 69508
human.FAM30B-LOC105372935-GGT2.NCBI.  ttaagcttagaccttgtaggcctatcagccagaagcagtgtgtaaaataagagtggctga 93448
human.FAM230E-LOC105377182-GGT3P.NCBI.  ------------------------------------------------------------ 57310
human.FAM230J-LOC105372942-GGTLC5P.NCBI.rev.compl  ------------------------------------------------------------ 46364
human.FAM230A.ncRNA.18487127.18500594-GGTLC3.18516335.18518165.rev.compl.REF  ------------------------------------------------------------ 31039

chimp.LOC112206744-LOC107973052-GGT2.revcompl  ------------------------------------------------------------ 61762
chimp54546-135457.revcompl.Pan.troglodytes.clone.rp43-41g5.GenBank:AC099533.36  CTGGGCGTGGTGGCTCACGCCTGTAATCCCAACACTTTAGGACGCCGAGGTAAGCAGATC 69568
human.FAM30B-LOC105372935-GGT2.NCBI.  ctgggcacggtggctcaagcctgtaatccaagcactttgggaggccaaggcgggcggatc 93508
human.FAM230E-LOC105377182-GGT3P.NCBI.  ------------------------------------------------------------ 57310
human.FAM230J-LOC105372942-GGTLC5P.NCBI.rev.compl  ------------------------------------------------------------ 46364
human.FAM230A.ncRNA.18487127.18500594-GGTLC3.18516335.18518165.rev.compl.REF  ------------------------------------------------------------ 31039

chimp.LOC112206744-LOC107973052-GGT2.revcompl  ------------------------------------------------------------ 61762
chimp54546-135457.revcompl.Pan.troglodytes.clone.rp43-41g5.GenBank:AC099533.36  TCTTAAGGTCAGGAGTTCAAGACCAGCCTGGCCAACATGGTGAAACCCTGTCTCTACTAA 69628
human.FAM30B-LOC105372935-GGT2.NCBI.  acctgaggtcaggagttcaagaccagcctggccaacatgatgaaaccccgtctctactac 93568
human.FAM230E-LOC105377182-GGT3P.NCBI.  ------------------------------------------------------------ 57310
human.FAM230J-LOC105372942-GGTLC5P.NCBI.rev.compl  ------------------------------------------------------------ 46364
human.FAM230A.ncRNA.18487127.18500594-GGTLC3.18516335.18518165.rev.compl.REF  ------------------------------------------------------------ 31039

chimp.LOC112206744-LOC107973052-GGT2.revcompl  ------------------------------------------------------------ 61762
chimp54546-135457.revcompl.Pan.troglodytes.clone.rp43-41g5.GenBank:AC099533.36  AAAAGAATACAAAAATTGGCCGGGCATAGTGGTGCACACCTGTGGTCCCAGCTACTCAGG 69688
human.FAM30B-LOC105372935-GGT2.NCBI.  a----aatacaaaaattagccgggtgtgatggcacacgcctgtaatcctagttactcggg 93624
human.FAM230E-LOC105377182-GGT3P.NCBI.  ------------------------------------------------------------ 57310
human.FAM230J-LOC105372942-GGTLC5P.NCBI.rev.compl  ------------------------------------------------------------ 46364
human.FAM230A.ncRNA.18487127.18500594-GGTLC3.18516335.18518165.rev.compl.REF  ------------------------------------------------------------ 31039

chimp.LOC112206744-LOC107973052-GGT2.revcompl  ------------------------------------------------------------ 61762
chimp54546-135457.revcompl.Pan.troglodytes.clone.rp43-41g5.GenBank:AC099533.36  TGACTGAAGCAGGAGAATTGCTTGAACCCAAGAAGTGGAGGTTGCAGTGAACCAAGATTG 69748
human.FAM30B-LOC105372935-GGT2.NCBI.  aggctgaggcacgagaatcgcttgaacctgggaggcagaggttgcgataagctgagattg 93684
human.FAM230E-LOC105377182-GGT3P.NCBI.  ------------------------------------------------------------ 57310
human.FAM230J-LOC105372942-GGTLC5P.NCBI.rev.compl  ------------------------------------------------------------ 46364
human.FAM230A.ncRNA.18487127.18500594-GGTLC3.18516335.18518165.rev.compl.REF  ------------------------------------------------------------ 31039

chimp.LOC112206744-LOC107973052-GGT2.revcompl  ------------------------------------------------------------ 61762
chimp54546-135457.revcompl.Pan.troglodytes.clone.rp43-41g5.GenBank:AC099533.36  CTCCACTGCACTCCAGCCTGGGCAGGAGAGGAAGACTCTCTCTGTCTCAACAACAACAAA 69808
human.FAM30B-LOC105372935-GGT2.NCBI.  ggccactgcactccagcctgggcgacagagcaagactccctctgaaaaaaaaaaaaaaaa 93744
human.FAM230E-LOC105377182-GGT3P.NCBI.  ------------------------------------------------------------ 57310
```

```
human.FAM230J-LOC105372942-GGTLC5P.NCBI.rev.compl                           ------------------------------------------------------------ 46364
human.FAM230A.ncRNA.18487127.18500594-GGTLC3.18516335.18518165.rev.compl.REF ------------------------------------------------------------ 31039

chimp.LOC112206744-LOC107973052-GGT2.revcompl                               ------------------------------------------------------------ 61762
chimp54546-135457.revcompl.Pan.troglodytes.clone.rp43-41g5.GenBank:AC099533.36 A----------------------------------------------------------- 69809
human.FAM30B-LOC105372935-GGT2.NCBI.                                        agagtggctacccaagaacgcaacagcagcagcaaacactgatctgatgacaccccagtt 93804
human.FAM230E-LOC105377182-GGT3P.NCBI.                                      ------------------------------------------------------------ 57310
human.FAM230J-LOC105372942-GGTLC5P.NCBI.rev.compl                           ------------------------------------------------------------ 46364
human.FAM230A.ncRNA.18487127.18500594-GGTLC3.18516335.18518165.rev.compl.REF ------------------------------------------------------------ 31039

chimp.LOC112206744-LOC107973052-GGT2.revcompl                               ------------------------------------------------------------ 61762
chimp54546-135457.revcompl.Pan.troglodytes.clone.rp43-41g5.GenBank:AC099533.36 ------------------------------------------------------------ 69809
human.FAM30B-LOC105372935-GGT2.NCBI.                                        ttgcagaagaggaaggaaaaataagaatgtcaactctccattcaacagagaaacagtgcc 93864
human.FAM230E-LOC105377182-GGT3P.NCBI.                                      ------------------------------------------------------------ 57310
human.FAM230J-LOC105372942-GGTLC5P.NCBI.rev.compl                           ------------------------------------------------------------ 46364
human.FAM230A.ncRNA.18487127.18500594-GGTLC3.18516335.18518165.rev.compl.REF ------------------------------------------------------------ 31039

chimp.LOC112206744-LOC107973052-GGT2.revcompl                               ------------------------------------------------------------ 61762
chimp54546-135457.revcompl.Pan.troglodytes.clone.rp43-41g5.GenBank:AC099533.36 ------------------------------------------------------------ 69809
human.FAM30B-LOC105372935-GGT2.NCBI.                                        accactgtctttaaattttcaaacttatcgagaaaagctccagagaggccagtgccagca 93924
human.FAM230E-LOC105377182-GGT3P.NCBI.                                      ------------------------------------------------------------ 57310
human.FAM230J-LOC105372942-GGTLC5P.NCBI.rev.compl                           ------------------------------------------------------------ 46364
human.FAM230A.ncRNA.18487127.18500594-GGTLC3.18516335.18518165.rev.compl.REF ------------------------------------------------------------ 31039

chimp.LOC112206744-LOC107973052-GGT2.revcompl                               ------------------------------------------------------------ 61762
chimp54546-135457.revcompl.Pan.troglodytes.clone.rp43-41g5.GenBank:AC099533.36 ------------------------------------------------------------ 69809
human.FAM30B-LOC105372935-GGT2.NCBI.                                        atgccggctgtctcccaagtctgaagggttttcctctgggtattaggagttgaggtgttt 93984
human.FAM230E-LOC105377182-GGT3P.NCBI.                                      ------------------------------------------------------------ 57310
human.FAM230J-LOC105372942-GGTLC5P.NCBI.rev.compl                           ------------------------------------------------------------ 46364
human.FAM230A.ncRNA.18487127.18500594-GGTLC3.18516335.18518165.rev.compl.REF ------------------------------------------------------------ 31039

chimp.LOC112206744-LOC107973052-GGT2.revcompl                               ------------------------------------------------------------ 61762
chimp54546-135457.revcompl.Pan.troglodytes.clone.rp43-41g5.GenBank:AC099533.36 ------------------------------------------------------------ 69809
human.FAM30B-LOC105372935-GGT2.NCBI.                                        ggttacagaaggaaacaggatgcacgggagatcacacacagagtaagaggtgcctctcaa 94044
human.FAM230E-LOC105377182-GGT3P.NCBI.                                      ------------------------------------------------------------ 57310
human.FAM230J-LOC105372942-GGTLC5P.NCBI.rev.compl                           ------------------------------------------------------------ 46364
human.FAM230A.ncRNA.18487127.18500594-GGTLC3.18516335.18518165.rev.compl.REF ------------------------------------------------------------ 31039

chimp.LOC112206744-LOC107973052-GGT2.revcompl                               ------------------------------------------------------------ 61762
chimp54546-135457.revcompl.Pan.troglodytes.clone.rp43-41g5.GenBank:AC099533.36 ------------------------------------------------------------ 69809
human.FAM30B-LOC105372935-GGT2.NCBI.                                        atgcctttcccacgtttctaactctgctttttttaaagcacgatcagacccgccatctgc 94104
human.FAM230E-LOC105377182-GGT3P.NCBI.                                      ------------------------------------------------------------ 57310
human.FAM230J-LOC105372942-GGTLC5P.NCBI.rev.compl                           ------------------------------------------------------------ 46364
human.FAM230A.ncRNA.18487127.18500594-GGTLC3.18516335.18518165.rev.compl.REF ------------------------------------------------------------ 31039

chimp.LOC112206744-LOC107973052-GGT2.revcompl                               ------------------------------------------------------------ 61762
chimp54546-135457.revcompl.Pan.troglodytes.clone.rp43-41g5.GenBank:AC099533.36 ------------------------------------------------------------ 69809
human.FAM30B-LOC105372935-GGT2.NCBI.                                        acttttacctgctgcctgacgaaaaccagatgccaaaggcctcccagcactcacggggcc 94164
human.FAM230E-LOC105377182-GGT3P.NCBI.                                      ------------------------------------------------------------ 57310
human.FAM230J-LOC105372942-GGTLC5P.NCBI.rev.compl                           ------------------------------------------------------------ 46364
human.FAM230A.ncRNA.18487127.18500594-GGTLC3.18516335.18518165.rev.compl.REF ------------------------------------------------------------ 31039

chimp.LOC112206744-LOC107973052-GGT2.revcompl                               ------------------------------------------------------------ 61762
chimp54546-135457.revcompl.Pan.troglodytes.clone.rp43-41g5.GenBank:AC099533.36 ------------------------------------------------------------ 69809
human.FAM30B-LOC105372935-GGT2.NCBI.                                        ctttacccttaggaaaacaccgtgattcagccaggcgagattgatgcaacattgtaacaa 94224
human.FAM230E-LOC105377182-GGT3P.NCBI.                                      ------------------------------------------------------------ 57310
human.FAM230J-LOC105372942-GGTLC5P.NCBI.rev.compl                           ------------------------------------------------------------ 46364
human.FAM230A.ncRNA.18487127.18500594-GGTLC3.18516335.18518165.rev.compl.REF ------------------------------------------------------------ 31039

chimp.LOC112206744-LOC107973052-GGT2.revcompl                               ------------------------------------------------------------ 61762
chimp54546-135457.revcompl.Pan.troglodytes.clone.rp43-41g5.GenBank:AC099533.36 ------------------------------------------------------------ 69809
human.FAM30B-LOC105372935-GGT2.NCBI.                                        agcgtatgcaaagtgcctagctccggactgcttgcagaaaaggcacattttgactcttct 94284
human.FAM230E-LOC105377182-GGT3P.NCBI.                                      ------------------------------------------------------------ 57310
human.FAM230J-LOC105372942-GGTLC5P.NCBI.rev.compl                           ------------------------------------------------------------ 46364
human.FAM230A.ncRNA.18487127.18500594-GGTLC3.18516335.18518165.rev.compl.REF ------------------------------------------------------------ 31039

chimp.LOC112206744-LOC107973052-GGT2.revcompl                               ------------------------------------------------------------ 61762
chimp54546-135457.revcompl.Pan.troglodytes.clone.rp43-41g5.GenBank:AC099533.36 ------------------------------------------------------------ 69809
human.FAM30B-LOC105372935-GGT2.NCBI.                                        gagagtgggaaagatttgaaagatgggatttgcaccttattcttattcaagtctggagat 94344
human.FAM230E-LOC105377182-GGT3P.NCBI.                                      ------------------------------------------------------------ 57310
human.FAM230J-LOC105372942-GGTLC5P.NCBI.rev.compl                           ------------------------------------------------------------ 46364
human.FAM230A.ncRNA.18487127.18500594-GGTLC3.18516335.18518165.rev.compl.REF ------------------------------------------------------------ 31039

chimp.LOC112206744-LOC107973052-GGT2.revcompl                               ------------------------------------------------------------ 61762
chimp54546-135457.revcompl.Pan.troglodytes.clone.rp43-41g5.GenBank:AC099533.36 ------------------------------------------------------------ 69809
human.FAM30B-LOC105372935-GGT2.NCBI.                                        ctggaacttcttcctgaagctccatgtttagccagataattgagacgctccacctggtca 94404
human.FAM230E-LOC105377182-GGT3P.NCBI.                                      ------------------------------------------------------------ 57310
human.FAM230J-LOC105372942-GGTLC5P.NCBI.rev.compl                           ------------------------------------------------------------ 46364
human.FAM230A.ncRNA.18487127.18500594-GGTLC3.18516335.18518165.rev.compl.REF ------------------------------------------------------------ 31039

chimp.LOC112206744-LOC107973052-GGT2.revcompl                               ------------------------------------------------------------ 61762
chimp54546-135457.revcompl.Pan.troglodytes.clone.rp43-41g5.GenBank:AC099533.36 ------------------------------------------------------------ 69809
human.FAM30B-LOC105372935-GGT2.NCBI.                                        ccactggacaccagccacccaaacattctcctctgcaaggcacttggcattctggtgcct 94464
human.FAM230E-LOC105377182-GGT3P.NCBI.                                      ------------------------------------------------------------ 57310
human.FAM230J-LOC105372942-GGTLC5P.NCBI.rev.compl                           ------------------------------------------------------------ 46364
human.FAM230A.ncRNA.18487127.18500594-GGTLC3.18516335.18518165.rev.compl.REF ------------------------------------------------------------ 31039

chimp.LOC112206744-LOC107973052-GGT2.revcompl                               ------------------------------------------------------------ 61762
chimp54546-135457.revcompl.Pan.troglodytes.clone.rp43-41g5.GenBank:AC099533.36 ------------------------------------------------------------ 69809
human.FAM30B-LOC105372935-GGT2.NCBI.                                        gtggagagagcagccttctcactagcacagctcatgaacaaggatccccttccctggaca 94524
human.FAM230E-LOC105377182-GGT3P.NCBI.                                      ------------------------------------------------------------ 57310
human.FAM230J-LOC105372942-GGTLC5P.NCBI.rev.compl                           ------------------------------------------------------------ 46364
human.FAM230A.ncRNA.18487127.18500594-GGTLC3.18516335.18518165.rev.compl.REF ------------------------------------------------------------ 31039

chimp.LOC112206744-LOC107973052-GGT2.revcompl                               ------------------------------------------------------------ 61762
chimp54546-135457.revcompl.Pan.troglodytes.clone.rp43-41g5.GenBank:AC099533.36 ------------------------------------------------------------ 69809
human.FAM30B-LOC105372935-GGT2.NCBI.                                        gccaagatgtaaacctgcccccagaaacactgagagtcctagaggtagaatgagtcggcc 94584
human.FAM230E-LOC105377182-GGT3P.NCBI.                                      ------------------------------------------------------------ 57310
human.FAM230J-LOC105372942-GGTLC5P.NCBI.rev.compl                           ------------------------------------------------------------ 46364
human.FAM230A.ncRNA.18487127.18500594-GGTLC3.18516335.18518165.rev.compl.REF ------------------------------------------------------------ 31039

chimp.LOC112206744-LOC107973052-GGT2.revcompl                               ------------------------------------------------------------ 61762
chimp54546-135457.revcompl.Pan.troglodytes.clone.rp43-41g5.GenBank:AC099533.36 ------------------------------------------------------------ 69809
human.FAM30B-LOC105372935-GGT2.NCBI.                                        agcaagctccagcccattgaatgcagacccctgagcaaatcaagtcaggagaaggtggac 94644
human.FAM230E-LOC105377182-GGT3P.NCBI.                                      ------------------------------------------------------------ 57310
human.FAM230J-LOC105372942-GGTLC5P.NCBI.rev.compl                           ------------------------------------------------------------ 46364
human.FAM230A.ncRNA.18487127.18500594-GGTLC3.18516335.18518165.rev.compl.REF ------------------------------------------------------------ 31039

chimp.LOC112206744-LOC107973052-GGT2.revcompl                               ------------------------------------------------------------ 61762
chimp54546-135457.revcompl.Pan.troglodytes.clone.rp43-41g5.GenBank:AC099533.36 ------------------------------------------------------------ 69809
human.FAM30B-LOC105372935-GGT2.NCBI.                                        aactgtaccctgaagaccacataccacccagacccaacacaaatagctgtcagcactgcc 94704
human.FAM230E-LOC105377182-GGT3P.NCBI.                                      ------------------------------------------------------------ 57310
human.FAM230J-LOC105372942-GGTLC5P.NCBI.rev.compl                           ------------------------------------------------------------ 46364
human.FAM230A.ncRNA.18487127.18500594-GGTLC3.18516335.18518165.rev.compl.REF ------------------------------------------------------------ 31039

chimp.LOC112206744-LOC107973052-GGT2.revcompl                               ------------------------------------------------------------ 61762
chimp54546-135457.revcompl.Pan.troglodytes.clone.rp43-41g5.GenBank:AC099533.36 ------------------------------------------------------------ 69809
human.FAM30B-LOC105372935-GGT2.NCBI.                                        acccattacagcttcagatctcgcttctaccagcatttcccagctgagcaacaaccgtca 94764
human.FAM230E-LOC105377182-GGT3P.NCBI.                                      ------------------------------------------------------------ 57310
human.FAM230J-LOC105372942-GGTLC5P.NCBI.rev.compl                           ------------------------------------------------------------ 46364
human.FAM230A.ncRNA.18487127.18500594-GGTLC3.18516335.18518165.rev.compl.REF ------------------------------------------------------------ 31039

chimp.LOC112206744-LOC107973052-GGT2.revcompl                               ------------------------------------------------------------ 61762
chimp54546-135457.revcompl.Pan.troglodytes.clone.rp43-41g5.GenBank:AC099533.36 ------------------------------------------------------------ 69809
human.FAM30B-LOC105372935-GGT2.NCBI.                                        aagtctggtcctggaaggacagccctccccgtcttggcagccacactttctggccctagc 94824
human.FAM230E-LOC105377182-GGT3P.NCBI.                                      ------------------------------------------------------------ 57310
human.FAM230J-LOC105372942-GGTLC5P.NCBI.rev.compl                           ------------------------------------------------------------ 46364
```

```
human.FAM230A.ncRNA.18487127.18500594-GGTLC3.18516335.18518165.rev.compl.REF        ------------------------------------------------------------ 31039

chimp.LOC112206744-LOC107973052-GGT2.revcompl                                       ------------------------------------------------------------ 61762
chimp54546-135457.revcompl.Pan.troglodytes.clone.rp43-41g5.GenBank:AC099533.36      ------------------------------------------------------------ 69809
human.FAM30B-LOC105372935-GGT2.NCBI.                                                ccaaggctgctgtggcatgggaggaatcaatggaggaatacatggctcttccctggttcc 94884
human.FAM230E-LOC105377182-GGT3P.NCBI.                                              ------------------------------------------------------------ 57310
human.FAM230J-LOC105372942-GGTLC5P.NCBI.rev.compl                                   ------------------------------------------------------------ 46364
human.FAM230A.ncRNA.18487127.18500594-GGTLC3.18516335.18518165.rev.compl.REF        ------------------------------------------------------------ 31039

chimp.LOC112206744-LOC107973052-GGT2.revcompl                                       ------------------------------------------------------------ 61762
chimp54546-135457.revcompl.Pan.troglodytes.clone.rp43-41g5.GenBank:AC099533.36      ------------------------------------------------------------ 69809
human.FAM30B-LOC105372935-GGT2.NCBI.                                                ttcctctcccacttcattgactaacactagtcacatggccccaactgaactgtgagggag 94944
human.FAM230E-LOC105377182-GGT3P.NCBI.                                              ------------------------------------------------------------ 57310
human.FAM230J-LOC105372942-GGTLC5P.NCBI.rev.compl                                   ------------------------------------------------------------ 46364
human.FAM230A.ncRNA.18487127.18500594-GGTLC3.18516335.18518165.rev.compl.REF        ------------------------------------------------------------ 31039

chimp.LOC112206744-LOC107973052-GGT2.revcompl                                       ------------------------------------------------------------ 61762
chimp54546-135457.revcompl.Pan.troglodytes.clone.rp43-41g5.GenBank:AC099533.36      ------------------------------------------------------------ 69809
human.FAM30B-LOC105372935-GGT2.NCBI.                                                gctggaaaatctggagtatgtgcatgcctagtgagttgcctctaccacatcctcttaacc 95004
human.FAM230E-LOC105377182-GGT3P.NCBI.                                              ------------------------------------------------------------ 57310
human.FAM230J-LOC105372942-GGTLC5P.NCBI.rev.compl                                   ------------------------------------------------------------ 46364
human.FAM230A.ncRNA.18487127.18500594-GGTLC3.18516335.18518165.rev.compl.REF        ------------------------------------------------------------ 31039

chimp.LOC112206744-LOC107973052-GGT2.revcompl                                       ------------------------------------------------------------ 61762
chimp54546-135457.revcompl.Pan.troglodytes.clone.rp43-41g5.GenBank:AC099533.36      ------------------------------------------------------------ 69809
human.FAM30B-LOC105372935-GGT2.NCBI.                                                cttggcctctcattcccaaagtctagccctattttcaaatgaattcaacgcctcattact 95064
human.FAM230E-LOC105377182-GGT3P.NCBI.                                              ------------------------------------------------------------ 57310
human.FAM230J-LOC105372942-GGTLC5P.NCBI.rev.compl                                   ------------------------------------------------------------ 46364
human.FAM230A.ncRNA.18487127.18500594-GGTLC3.18516335.18518165.rev.compl.REF        ------------------------------------------------------------ 31039

chimp.LOC112206744-LOC107973052-GGT2.revcompl                                       ------------------------------------------------------------ 61762
chimp54546-135457.revcompl.Pan.troglodytes.clone.rp43-41g5.GenBank:AC099533.36      ------------------------------------------------------------ 69809
human.FAM30B-LOC105372935-GGT2.NCBI.                                                tagatctttcctcattacctcaaagccatagtggcaaaaagcaaacagcatcttcctaaa 95124
human.FAM230E-LOC105377182-GGT3P.NCBI.                                              ------------------------------------------------------------ 57310
human.FAM230J-LOC105372942-GGTLC5P.NCBI.rev.compl                                   ------------------------------------------------------------ 46364
human.FAM230A.ncRNA.18487127.18500594-GGTLC3.18516335.18518165.rev.compl.REF        ------------------------------------------------------------ 31039

chimp.LOC112206744-LOC107973052-GGT2.revcompl                                       ------------------------------------------------------------ 61762
chimp54546-135457.revcompl.Pan.troglodytes.clone.rp43-41g5.GenBank:AC099533.36      ------------------------------------------------------------ 69809
human.FAM30B-LOC105372935-GGT2.NCBI.                                                atcttcctcttccatcttcctctcctagatatccatcttacccttctttgccagttaatt 95184
human.FAM230E-LOC105377182-GGT3P.NCBI.                                              ------------------------------------------------------------ 57310
human.FAM230J-LOC105372942-GGTLC5P.NCBI.rev.compl                                   ------------------------------------------------------------ 46364
human.FAM230A.ncRNA.18487127.18500594-GGTLC3.18516335.18518165.rev.compl.REF        ------------------------------------------------------------ 31039

chimp.LOC112206744-LOC107973052-GGT2.revcompl                                       ------------------------------------------------------------ 61762
chimp54546-135457.revcompl.Pan.troglodytes.clone.rp43-41g5.GenBank:AC099533.36      ------------------------------------------------------------ 69809
human.FAM30B-LOC105372935-GGT2.NCBI.                                                tattacaatcttcctttgacagcttcactccctccccgcactgctatcaactcaaatgtg 95244
human.FAM230E-LOC105377182-GGT3P.NCBI.                                              ------------------------------------------------------------ 57310
human.FAM230J-LOC105372942-GGTLC5P.NCBI.rev.compl                                   ------------------------------------------------------------ 46364
human.FAM230A.ncRNA.18487127.18500594-GGTLC3.18516335.18518165.rev.compl.REF        ------------------------------------------------------------ 31039

chimp.LOC112206744-LOC107973052-GGT2.revcompl                                       ------------------------------------------------------------ 61762
chimp54546-135457.revcompl.Pan.troglodytes.clone.rp43-41g5.GenBank:AC099533.36      ------------------------------------------------------------ 69809
human.FAM30B-LOC105372935-GGT2.NCBI.                                                agttatttcagtagcttgagcttgtcactggccttttactttttccccttccaaagaac 95304
human.FAM230E-LOC105377182-GGT3P.NCBI.                                              ------------------------------------------------------------ 57310
human.FAM230J-LOC105372942-GGTLC5P.NCBI.rev.compl                                   ------------------------------------------------------------ 46364
human.FAM230A.ncRNA.18487127.18500594-GGTLC3.18516335.18518165.rev.compl.REF        ------------------------------------------------------------ 31039

chimp.LOC112206744-LOC107973052-GGT2.revcompl                                       ------------------------------------------------------------ 61762
chimp54546-135457.revcompl.Pan.troglodytes.clone.rp43-41g5.GenBank:AC099533.36      ------------------------------------------------------------ 69809
human.FAM30B-LOC105372935-GGT2.NCBI.                                                ctggtacccagctacttaatccttatccttcttcccaaggcccactttcattcctttctc 95364
human.FAM230E-LOC105377182-GGT3P.NCBI.                                              ------------------------------------------------------------ 57310
human.FAM230J-LOC105372942-GGTLC5P.NCBI.rev.compl                                   ------------------------------------------------------------ 46364
human.FAM230A.ncRNA.18487127.18500594-GGTLC3.18516335.18518165.rev.compl.REF        ------------------------------------------------------------ 31039

chimp.LOC112206744-LOC107973052-GGT2.revcompl                                       ------------------------------------------------------------ 61762
chimp54546-135457.revcompl.Pan.troglodytes.clone.rp43-41g5.GenBank:AC099533.36      ------------------------------------------------------------ 69809
human.FAM30B-LOC105372935-GGT2.NCBI.                                                ccccattcatgagcctgcaatacttttcccgctgcataagtcacgtgtcaccatataaac 95424
human.FAM230E-LOC105377182-GGT3P.NCBI.                                              ------------------------------------------------------------ 57310
human.FAM230J-LOC105372942-GGTLC5P.NCBI.rev.compl                                   ------------------------------------------------------------ 46364
human.FAM230A.ncRNA.18487127.18500594-GGTLC3.18516335.18518165.rev.compl.REF        ------------------------------------------------------------ 31039

chimp.LOC112206744-LOC107973052-GGT2.revcompl                                       ------------------------------------------------------------ 61762
chimp54546-135457.revcompl.Pan.troglodytes.clone.rp43-41g5.GenBank:AC099533.36      ------------------------------------------------------------ 69809
human.FAM30B-LOC105372935-GGT2.NCBI.                                                tcacaatttgaggtgctctagaatctgtccacggaattagtcaggacataatctccctgt 95484
human.FAM230E-LOC105377182-GGT3P.NCBI.                                              ------------------------------------------------------------ 57310
human.FAM230J-LOC105372942-GGTLC5P.NCBI.rev.compl                                   ------------------------------------------------------------ 46364
human.FAM230A.ncRNA.18487127.18500594-GGTLC3.18516335.18518165.rev.compl.REF        ------------------------------------------------------------ 31039

chimp.LOC112206744-LOC107973052-GGT2.revcompl                                       ------------------------------------------------------------ 61762
chimp54546-135457.revcompl.Pan.troglodytes.clone.rp43-41g5.GenBank:AC099533.36      ------------------------------------------------------------ 69809
human.FAM30B-LOC105372935-GGT2.NCBI.                                                ttttcggcagtgccacatccatacctgtcttcacaggtttgctcatgccaccattccttt 95544
human.FAM230E-LOC105377182-GGT3P.NCBI.                                              ------------------------------------------------------------ 57310
human.FAM230J-LOC105372942-GGTLC5P.NCBI.rev.compl                                   ------------------------------------------------------------ 46364
human.FAM230A.ncRNA.18487127.18500594-GGTLC3.18516335.18518165.rev.compl.REF        ------------------------------------------------------------ 31039

chimp.LOC112206744-LOC107973052-GGT2.revcompl                                       ------------------------------------------------------------ 61762
chimp54546-135457.revcompl.Pan.troglodytes.clone.rp43-41g5.GenBank:AC099533.36      ------------------------------------------------------------ 69809
human.FAM30B-LOC105372935-GGT2.NCBI.                                                ccccagaacgcccttctgctcctcccacacaaatcctaatcctccctcaggacccattta 95604
human.FAM230E-LOC105377182-GGT3P.NCBI.                                              ------------------------------------------------------------ 57310
human.FAM230J-LOC105372942-GGTLC5P.NCBI.rev.compl                                   ------------------------------------------------------------ 46364
human.FAM230A.ncRNA.18487127.18500594-GGTLC3.18516335.18518165.rev.compl.REF        ------------------------------------------------------------ 31039

chimp.LOC112206744-LOC107973052-GGT2.revcompl                                       ------------------------------------------------------------ 61762
chimp54546-135457.revcompl.Pan.troglodytes.clone.rp43-41g5.GenBank:AC099533.36      ------------------------------------------------------------ 69809
human.FAM30B-LOC105372935-GGT2.NCBI.                                                aaatacaatctcctcactcaactctttcctaccaacttgctttcccctctcctttctgcc 95664
human.FAM230E-LOC105377182-GGT3P.NCBI.                                              ------------------------------------------------------------ 57310
human.FAM230J-LOC105372942-GGTLC5P.NCBI.rev.compl                                   ------------------------------------------------------------ 46364
human.FAM230A.ncRNA.18487127.18500594-GGTLC3.18516335.18518165.rev.compl.REF        ------------------------------------------------------------ 31039

chimp.LOC112206744-LOC107973052-GGT2.revcompl                                       ------------------------------------------------------------ 61762
chimp54546-135457.revcompl.Pan.troglodytes.clone.rp43-41g5.GenBank:AC099533.36      ------------------------------------------------------------ 69809
human.FAM30B-LOC105372935-GGT2.NCBI.                                                ttaccacaaagctaagctctgtctctgaataatgtgctttttgatgataatctgaatggt 95724
human.FAM230E-LOC105377182-GGT3P.NCBI.                                              ------------------------------------------------------------ 57310
human.FAM230J-LOC105372942-GGTLC5P.NCBI.rev.compl                                   ------------------------------------------------------------ 46364
human.FAM230A.ncRNA.18487127.18500594-GGTLC3.18516335.18518165.rev.compl.REF        ------------------------------------------------------------ 31039

chimp.LOC112206744-LOC107973052-GGT2.revcompl                                       ------------------------------------------------------------ 61762
chimp54546-135457.revcompl.Pan.troglodytes.clone.rp43-41g5.GenBank:AC099533.36      ---------------AGTACTAGCTCATGTTAGACTTTGATAAGTGAAGGATGCA----- 69849
human.FAM30B-LOC105372935-GGT2.NCBI.                                                acaagaagaaatcctatttcatgctcctttgagggagtgtctaatataggttgccttcga 95784
human.FAM230E-LOC105377182-GGT3P.NCBI.                                              ------------------------------------------------------------ 57310
human.FAM230J-LOC105372942-GGTLC5P.NCBI.rev.compl                                   ------------------------------------------------------------ 46364
human.FAM230A.ncRNA.18487127.18500594-GGTLC3.18516335.18518165.rev.compl.REF        ------------------------------------------------------------ 31039

chimp.LOC112206744-LOC107973052-GGT2.revcompl                                       ------------------------------------------------------------ 61762
chimp54546-135457.revcompl.Pan.troglodytes.clone.rp43-41g5.GenBank:AC099533.36      ------------------------------------------------------------ 69849
human.FAM30B-LOC105372935-GGT2.NCBI.                                                ctataagtagtagaaaattcccactcaactggcttcaacagtgaggacaaaatgtatgga 95844
human.FAM230E-LOC105377182-GGT3P.NCBI.                                              ------------------------------------------------------------ 57310
human.FAM230J-LOC105372942-GGTLC5P.NCBI.rev.compl                                   ------------------------------------------------------------ 46364
human.FAM230A.ncRNA.18487127.18500594-GGTLC3.18516335.18518165.rev.compl.REF        ------------------------------------------------------------ 31039

chimp.LOC112206744-LOC107973052-GGT2.revcompl                                       ------------------------------------------------------------ 61762
chimp54546-135457.revcompl.Pan.troglodytes.clone.rp43-41g5.GenBank:AC099533.36      ------------------------------------------------------------ 69849
human.FAM30B-LOC105372935-GGT2.NCBI.                                                gattggacgcttcagtcttcaggggcttggccctcagggtttttcccatctcttcccgtc 95904
human.FAM230E-LOC105377182-GGT3P.NCBI.                                              ------------------------------------------------------------ 57310
human.FAM230J-LOC105372942-GGTLC5P.NCBI.rev.compl                                   ------------------------------------------------------------ 46364
human.FAM230A.ncRNA.18487127.18500594-GGTLC3.18516335.18518165.rev.compl.REF        ------------------------------------------------------------ 31039
```

```
chimp.LOC112206744-LOC107973052-GGT2.revcompl                                  ------------------------------------------------------------ 61762
chimp54546-135457.revcompl.Pan.troglodytes.clone.rp43-41g5.GenBank:AC099533.36 ------------------------------------------------------------ 69849
human.FAM30B-LOC105372935-GGT2.NCBI.                                           tgctggcctcaacaaacctgctttcctcattgcaacaagatggctgcttcacgtccgaga 95964
human.FAM230E-LOC105377182-GGT3P.NCBI.                                         ------------------------------------------------------------ 57310
human.FAM230J-LOC105372942-GGTLC5P.NCBI.rev.compl                              ------------------------------------------------------------ 46364
human.FAM230A.ncRNA.18487127.18500594-GGTLC3.18516335.18518165.rev.compl.REF   ------------------------------------------------------------ 31039

chimp.LOC112206744-LOC107973052-GGT2.revcompl                                  ------------------------------------------------------------ 61762
chimp54546-135457.revcompl.Pan.troglodytes.clone.rp43-41g5.GenBank:AC099533.36 ------------------------------------------------------------ 69849
human.FAM30B-LOC105372935-GGT2.NCBI.                                           atctagtgaaagaagctgtcccttctgcatttccttttacaaggaaaaacatctttccca 96024
human.FAM230E-LOC105377182-GGT3P.NCBI.                                         ------------------------------------------------------------ 57310
human.FAM230J-LOC105372942-GGTLC5P.NCBI.rev.compl                              ------------------------------------------------------------ 46364
human.FAM230A.ncRNA.18487127.18500594-GGTLC3.18516335.18518165.rev.compl.REF   ------------------------------------------------------------ 31039

chimp.LOC112206744-LOC107973052-GGT2.revcompl                                  ------------------------------------------------------------ 61762
chimp54546-135457.revcompl.Pan.troglodytes.clone.rp43-41g5.GenBank:AC099533.36 ------------------------------------------------------------ 69849
human.FAM30B-LOC105372935-GGT2.NCBI.                                           gcatctcccagcagacttctcatacctcagtggccaaaaccatgtctaaactacgggcct 96084
human.FAM230E-LOC105377182-GGT3P.NCBI.                                         ------------------------------------------------------------ 57310
human.FAM230J-LOC105372942-GGTLC5P.NCBI.rev.compl                              ------------------------------------------------------------ 46364
human.FAM230A.ncRNA.18487127.18500594-GGTLC3.18516335.18518165.rev.compl.REF   ------------------------------------------------------------ 31039

chimp.LOC112206744-LOC107973052-GGT2.revcompl                                  ------------------------------------------------------------ 61762
chimp54546-135457.revcompl.Pan.troglodytes.clone.rp43-41g5.GenBank:AC099533.36 ------------------------------------------------------------ 69849
human.FAM30B-LOC105372935-GGT2.NCBI.                                           ccaggatgggctcagctcaatcagcacataccctagagctggaggcagggtcacctttcc 96144
human.FAM230E-LOC105377182-GGT3P.NCBI.                                         ------------------------------------------------------------ 57310
human.FAM230J-LOC105372942-GGTLC5P.NCBI.rev.compl                              ------------------------------------------------------------ 46364
human.FAM230A.ncRNA.18487127.18500594-GGTLC3.18516335.18518165.rev.compl.REF   ------------------------------------------------------------ 31039

chimp.LOC112206744-LOC107973052-GGT2.revcompl                                  ------------------------------------------------------------ 61762
chimp54546-135457.revcompl.Pan.troglodytes.clone.rp43-41g5.GenBank:AC099533.36 ------------------------------------------------------------ 69849
human.FAM30B-LOC105372935-GGT2.NCBI.                                           ttgagtggcgtgtgggagaggtgtggcatccaacccaactgggtcctgccagcaggaaga 96204
human.FAM230E-LOC105377182-GGT3P.NCBI.                                         ------------------------------------------------------------ 57310
human.FAM230J-LOC105372942-GGTLC5P.NCBI.rev.compl                              ------------------------------------------------------------ 46364
human.FAM230A.ncRNA.18487127.18500594-GGTLC3.18516335.18518165.rev.compl.REF   ------------------------------------------------------------ 31039

chimp.LOC112206744-LOC107973052-GGT2.revcompl                                  ------------------------------------------------------------ 61762
chimp54546-135457.revcompl.Pan.troglodytes.clone.rp43-41g5.GenBank:AC099533.36 ------------------------------------------------------------ 69849
human.FAM30B-LOC105372935-GGT2.NCBI.                                           gaaaagaagagccactgtaggtaaccaatcacgtctaccacaggcagggaccaataccac 96264
human.FAM230E-LOC105377182-GGT3P.NCBI.                                         ------------------------------------------------------------ 57310
human.FAM230J-LOC105372942-GGTLC5P.NCBI.rev.compl                              ------------------------------------------------------------ 46364
human.FAM230A.ncRNA.18487127.18500594-GGTLC3.18516335.18518165.rev.compl.REF   ------------------------------------------------------------ 31039

chimp.LOC112206744-LOC107973052-GGT2.revcompl                                  ------------------------------------------------------------ 61762
chimp54546-135457.revcompl.Pan.troglodytes.clone.rp43-41g5.GenBank:AC099533.36 ------------------------------------------------------------ 69849
human.FAM30B-LOC105372935-GGT2.NCBI.                                           atatctgtgtttcccctcacttgccacccccatcctgtgctggcagatgaggatgctcaa 96324
human.FAM230E-LOC105377182-GGT3P.NCBI.                                         ------------------------------------------------------------ 57310
human.FAM230J-LOC105372942-GGTLC5P.NCBI.rev.compl                              ------------------------------------------------------------ 46364
human.FAM230A.ncRNA.18487127.18500594-GGTLC3.18516335.18518165.rev.compl.REF   ------------------------------------------------------------ 31039

chimp.LOC112206744-LOC107973052-GGT2.revcompl                                  ------------------------------------------------------------ 61762
chimp54546-135457.revcompl.Pan.troglodytes.clone.rp43-41g5.GenBank:AC099533.36 ------------------------------------------------------------ 69849
human.FAM30B-LOC105372935-GGT2.NCBI.                                           caaatagctgtggaactacaaattatcctgtgggtgtgagaaaagagtgtgcattataat 96384
human.FAM230E-LOC105377182-GGT3P.NCBI.                                         ------------------------------------------------------------ 57310
human.FAM230J-LOC105372942-GGTLC5P.NCBI.rev.compl                              ------------------------------------------------------------ 46364
human.FAM230A.ncRNA.18487127.18500594-GGTLC3.18516335.18518165.rev.compl.REF   ------------------------------------------------------------ 31039

chimp.LOC112206744-LOC107973052-GGT2.revcompl                                  ------------------------------------------------------------ 61762
chimp54546-135457.revcompl.Pan.troglodytes.clone.rp43-41g5.GenBank:AC099533.36 ------------------------------------------------------------ 69849
human.FAM30B-LOC105372935-GGT2.NCBI.                                           agggtggtacaattggacatgtgtgcacggaaaatggaaaaacagccatgaaatccaaac 96444
human.FAM230E-LOC105377182-GGT3P.NCBI.                                         ------------------------------------------------------------ 57310
human.FAM230J-LOC105372942-GGTLC5P.NCBI.rev.compl                              ------------------------------------------------------------ 46364
human.FAM230A.ncRNA.18487127.18500594-GGTLC3.18516335.18518165.rev.compl.REF   ------------------------------------------------------------ 31039

chimp.LOC112206744-LOC107973052-GGT2.revcompl                                  ------------------------------------------------------------ 61762
chimp54546-135457.revcompl.Pan.troglodytes.clone.rp43-41g5.GenBank:AC099533.36 ------------------------------------------------------------ 69849
human.FAM30B-LOC105372935-GGT2.NCBI.                                           agatgcaaaaacgttcttaacacagaaggtttgggaagctctgatctaagccctagcctg 96504
human.FAM230E-LOC105377182-GGT3P.NCBI.                                         ------------------------------------------------------------ 57310
human.FAM230J-LOC105372942-GGTLC5P.NCBI.rev.compl                              ------------------------------------------------------------ 46364
human.FAM230A.ncRNA.18487127.18500594-GGTLC3.18516335.18518165.rev.compl.REF   ------------------------------------------------------------ 31039

chimp.LOC112206744-LOC107973052-GGT2.revcompl                                  ------------------------------------------------------------ 61762
chimp54546-135457.revcompl.Pan.troglodytes.clone.rp43-41g5.GenBank:AC099533.36 ------------------------------------------------------------ 69849
human.FAM30B-LOC105372935-GGT2.NCBI.                                           ggccctactcccagttccacagatagggagcaggccccaggccagagtgctgggcttctc 96564
human.FAM230E-LOC105377182-GGT3P.NCBI.                                         ------------------------------------------------------------ 57310
human.FAM230J-LOC105372942-GGTLC5P.NCBI.rev.compl                              ------------------------------------------------------------ 46364
human.FAM230A.ncRNA.18487127.18500594-GGTLC3.18516335.18518165.rev.compl.REF   ------------------------------------------------------------ 31039

chimp.LOC112206744-LOC107973052-GGT2.revcompl                                  ------------------------------------------------------------ 61762
chimp54546-135457.revcompl.Pan.troglodytes.clone.rp43-41g5.GenBank:AC099533.36 ------------------------------------------------------------ 69849
human.FAM30B-LOC105372935-GGT2.NCBI.                                           ccagcaggatccagctggggctttctcctcagttcaacccttgcctctgaggaccagtgg 96624
human.FAM230E-LOC105377182-GGT3P.NCBI.                                         ------------------------------------------------------------ 57310
human.FAM230J-LOC105372942-GGTLC5P.NCBI.rev.compl                              ------------------------------------------------------------ 46364
human.FAM230A.ncRNA.18487127.18500594-GGTLC3.18516335.18518165.rev.compl.REF   ------------------------------------------------------------ 31039

chimp.LOC112206744-LOC107973052-GGT2.revcompl                                  ------------------------------------------------------------ 61762
chimp54546-135457.revcompl.Pan.troglodytes.clone.rp43-41g5.GenBank:AC099533.36 ------------------------------------------------------------ 69849
human.FAM30B-LOC105372935-GGT2.NCBI.                                           tctacagtccagcccaccacccttcagacagaagttccaggcacaaaaaggaagctcaca 96684
human.FAM230E-LOC105377182-GGT3P.NCBI.                                         ------------------------------------------------------------ 57310
human.FAM230J-LOC105372942-GGTLC5P.NCBI.rev.compl                              ------------------------------------------------------------ 46364
human.FAM230A.ncRNA.18487127.18500594-GGTLC3.18516335.18518165.rev.compl.REF   ------------------------------------------------------------ 31039

chimp.LOC112206744-LOC107973052-GGT2.revcompl                                  ------------------------------------------------------------ 61762
chimp54546-135457.revcompl.Pan.troglodytes.clone.rp43-41g5.GenBank:AC099533.36 ------------------------------------------------------------ 69849
human.FAM30B-LOC105372935-GGT2.NCBI.                                           ataaggaacgcccatgcctagccataactgagcgcatcacagactcacacaaatctgtgg 96744
human.FAM230E-LOC105377182-GGT3P.NCBI.                                         ------------------------------------------------------------ 57310
human.FAM230J-LOC105372942-GGTLC5P.NCBI.rev.compl                              ------------------------------------------------------------ 46364
human.FAM230A.ncRNA.18487127.18500594-GGTLC3.18516335.18518165.rev.compl.REF   ------------------------------------------------------------ 31039

chimp.LOC112206744-LOC107973052-GGT2.revcompl                                  ------------------------------------------------------------ 61762
chimp54546-135457.revcompl.Pan.troglodytes.clone.rp43-41g5.GenBank:AC099533.36 ------------------------------------------------------------ 69849
human.FAM30B-LOC105372935-GGT2.NCBI.                                           aatgagtgcatgatctcatgagctctggtctggaaaagtaagtctccctacactaggact 96804
human.FAM230E-LOC105377182-GGT3P.NCBI.                                         ------------------------------------------------------------ 57310
human.FAM230J-LOC105372942-GGTLC5P.NCBI.rev.compl                              ------------------------------------------------------------ 46364
human.FAM230A.ncRNA.18487127.18500594-GGTLC3.18516335.18518165.rev.compl.REF   ------------------------------------------------------------ 31039

chimp.LOC112206744-LOC107973052-GGT2.revcompl                                  ------------------------------------------------------------ 61762
chimp54546-135457.revcompl.Pan.troglodytes.clone.rp43-41g5.GenBank:AC099533.36 ------------------------------------------------------------ 69849
human.FAM30B-LOC105372935-GGT2.NCBI.                                           taaagggaaggcttctttggctatgccaatatctttcctgccactttcttcttcaggcca 96864
human.FAM230E-LOC105377182-GGT3P.NCBI.                                         ------------------------------------------------------------ 57310
human.FAM230J-LOC105372942-GGTLC5P.NCBI.rev.compl                              ------------------------------------------------------------ 46364
human.FAM230A.ncRNA.18487127.18500594-GGTLC3.18516335.18518165.rev.compl.REF   ------------------------------------------------------------ 31039

chimp.LOC112206744-LOC107973052-GGT2.revcompl                                  ------------------------------------------------------------ 61762
chimp54546-135457.revcompl.Pan.troglodytes.clone.rp43-41g5.GenBank:AC099533.36 ------------------------------------------------------------ 69849
human.FAM30B-LOC105372935-GGT2.NCBI.                                           aacagctcctatccctgccttccatcttagctttccagcatactcttcccaggttctttg 96924
human.FAM230E-LOC105377182-GGT3P.NCBI.                                         ------------------------------------------------------------ 57310
human.FAM230J-LOC105372942-GGTLC5P.NCBI.rev.compl                              ------------------------------------------------------------ 46364
human.FAM230A.ncRNA.18487127.18500594-GGTLC3.18516335.18518165.rev.compl.REF   ------------------------------------------------------------ 31039

chimp.LOC112206744-LOC107973052-GGT2.revcompl                                  ------------------------------------------------------------ 61762
chimp54546-135457.revcompl.Pan.troglodytes.clone.rp43-41g5.GenBank:AC099533.36 ------------------------------------------------------------ 69849
human.FAM30B-LOC105372935-GGT2.NCBI.                                           ggctactgacaagcactccagacccaaatacacatgctcccacctgaggctgggaaatgt 96984
human.FAM230E-LOC105377182-GGT3P.NCBI.                                         ------------------------------------------------------------ 57310
human.FAM230J-LOC105372942-GGTLC5P.NCBI.rev.compl                              ------------------------------------------------------------ 46364
human.FAM230A.ncRNA.18487127.18500594-GGTLC3.18516335.18518165.rev.compl.REF   ------------------------------------------------------------ 31039
```

```
chimp.LOC112206744-LOC107973052-GGT2.revcompl                                 ------------------------------------------------------------ 61762
chimp54546-135457.revcompl.Pan.troglodytes.clone.rp43-41g5.GenBank:AC099533.36 ------------------------------------------------------------ 69849
human.FAM30B-LOC105372935-GGT2.NCBI.                                          accaagaactgcatagaaaatatactggacaaaaggcactccagtctcacagatctccag 97044
human.FAM230E-LOC105377182-GGT3P.NCBI.                                        ------------------------------------------------------------ 57310
human.FAM230J-LOC105372942-GGTLC5P.NCBI.rev.compl                             ------------------------------------------------------------ 46364
human.FAM230A.ncRNA.18487127.18500594-GGTLC3.18516335.18518165.rev.compl.REF  ------------------------------------------------------------ 31039

chimp.LOC112206744-LOC107973052-GGT2.revcompl                                 ------------------------------------------------------------ 61762
chimp54546-135457.revcompl.Pan.troglodytes.clone.rp43-41g5.GenBank:AC099533.36 ------------------------------------------------------------ 69849
human.FAM30B-LOC105372935-GGT2.NCBI.                                          tcccacagtctatatcaccagttttagctgtttgaagttttagaattccttttcaaaacc 97104
human.FAM230E-LOC105377182-GGT3P.NCBI.                                        ------------------------------------------------------------ 57310
human.FAM230J-LOC105372942-GGTLC5P.NCBI.rev.compl                             ------------------------------------------------------------ 46364
human.FAM230A.ncRNA.18487127.18500594-GGTLC3.18516335.18518165.rev.compl.REF  ------------------------------------------------------------ 31039

chimp.LOC112206744-LOC107973052-GGT2.revcompl                                 ------------------------------------------------------------ 61762
chimp54546-135457.revcompl.Pan.troglodytes.clone.rp43-41g5.GenBank:AC099533.36 ------------------------------------------------------------ 69849
human.FAM30B-LOC105372935-GGT2.NCBI.                                          atagctagcatttctttggctagagaaaactgtgtgaaatgcagcctaccataggatcac 97164
human.FAM230E-LOC105377182-GGT3P.NCBI.                                        ------------------------------------------------------------ 57310
human.FAM230J-LOC105372942-GGTLC5P.NCBI.rev.compl                             ------------------------------------------------------------ 46364
human.FAM230A.ncRNA.18487127.18500594-GGTLC3.18516335.18518165.rev.compl.REF  ------------------------------------------------------------ 31039

chimp.LOC112206744-LOC107973052-GGT2.revcompl                                 ------------------------------------------------------------ 61762
chimp54546-135457.revcompl.Pan.troglodytes.clone.rp43-41g5.GenBank:AC099533.36 ------------------------------------------------------------ 69849
human.FAM30B-LOC105372935-GGT2.NCBI.                                          tgattacaacatgcattcaacattcaatacatattgactgagcaactattaggttgaact 97224
human.FAM230E-LOC105377182-GGT3P.NCBI.                                        ------------------------------------------------------------ 57310
human.FAM230J-LOC105372942-GGTLC5P.NCBI.rev.compl                             ------------------------------------------------------------ 46364
human.FAM230A.ncRNA.18487127.18500594-GGTLC3.18516335.18518165.rev.compl.REF  ------------------------------------------------------------ 31039

chimp.LOC112206744-LOC107973052-GGT2.revcompl                                 ------------------------------------------------------------ 61762
chimp54546-135457.revcompl.Pan.troglodytes.clone.rp43-41g5.GenBank:AC099533.36 ------------------------------------------------------------ 69849
human.FAM30B-LOC105372935-GGT2.NCBI.                                          atgtgacattattgacattcgactgcttctgacctatcaaaatggcaataaagctcaact 97284
human.FAM230E-LOC105377182-GGT3P.NCBI.                                        ------------------------------------------------------------ 57310
human.FAM230J-LOC105372942-GGTLC5P.NCBI.rev.compl                             ------------------------------------------------------------ 46364
human.FAM230A.ncRNA.18487127.18500594-GGTLC3.18516335.18518165.rev.compl.REF  ------------------------------------------------------------ 31039

chimp.LOC112206744-LOC107973052-GGT2.revcompl                                 ------------------------------------------------------------ 61762
chimp54546-135457.revcompl.Pan.troglodytes.clone.rp43-41g5.GenBank:AC099533.36 ------------------------------------------------------------ 69849
human.FAM30B-LOC105372935-GGT2.NCBI.                                          taatatttactctatgccaggcagtaggctaaggaaggatttgggacaccatgacaaaca 97344
human.FAM230E-LOC105377182-GGT3P.NCBI.                                        ------------------------------------------------------------ 57310
human.FAM230J-LOC105372942-GGTLC5P.NCBI.rev.compl                             ------------------------------------------------------------ 46364
human.FAM230A.ncRNA.18487127.18500594-GGTLC3.18516335.18518165.rev.compl.REF  ------------------------------------------------------------ 31039

chimp.LOC112206744-LOC107973052-GGT2.revcompl                                 ------------------------------------------------------------ 61762
chimp54546-135457.revcompl.Pan.troglodytes.clone.rp43-41g5.GenBank:AC099533.36 ------------------------------------------------------------ 69849
human.FAM30B-LOC105372935-GGT2.NCBI.                                          acacagaagtaacccctatcttaatggaacttaaaatctagttgattaaaccattactta 97404
human.FAM230E-LOC105377182-GGT3P.NCBI.                                        ------------------------------------------------------------ 57310
human.FAM230J-LOC105372942-GGTLC5P.NCBI.rev.compl                             ------------------------------------------------------------ 46364
human.FAM230A.ncRNA.18487127.18500594-GGTLC3.18516335.18518165.rev.compl.REF  ------------------------------------------------------------ 31039

chimp.LOC112206744-LOC107973052-GGT2.revcompl                                 ------------------------------------------------------------ 61762
chimp54546-135457.revcompl.Pan.troglodytes.clone.rp43-41g5.GenBank:AC099533.36 ------------------------------------------------------------ 69849
human.FAM30B-LOC105372935-GGT2.NCBI.                                          caaaagtacaatctaacttcaggattgtaattaattataaatgcatttatttgtttactg 97464
human.FAM230E-LOC105377182-GGT3P.NCBI.                                        ------------------------------------------------------------ 57310
human.FAM230J-LOC105372942-GGTLC5P.NCBI.rev.compl                             ------------------------------------------------------------ 46364
human.FAM230A.ncRNA.18487127.18500594-GGTLC3.18516335.18518165.rev.compl.REF  ------------------------------------------------------------ 31039

chimp.LOC112206744-LOC107973052-GGT2.revcompl                                 ------------------------------------------------------------ 61762
chimp54546-135457.revcompl.Pan.troglodytes.clone.rp43-41g5.GenBank:AC099533.36 ------------------------------------------------------------ 69849
human.FAM30B-LOC105372935-GGT2.NCBI.                                          cctgacttctttgctagcctataaactacacaaagatgagggttatgtctcctccatttc 97524
human.FAM230E-LOC105377182-GGT3P.NCBI.                                        ------------------------------------------------------------ 57310
human.FAM230J-LOC105372942-GGTLC5P.NCBI.rev.compl                             ------------------------------------------------------------ 46364
human.FAM230A.ncRNA.18487127.18500594-GGTLC3.18516335.18518165.rev.compl.REF  ------------------------------------------------------------ 31039

chimp.LOC112206744-LOC107973052-GGT2.revcompl                                 ------------------------------------------------------------ 61762
chimp54546-135457.revcompl.Pan.troglodytes.clone.rp43-41g5.GenBank:AC099533.36 ------------------------------------------------------------ 69849
human.FAM30B-LOC105372935-GGT2.NCBI.                                          acacactggcaaagcatatagcagaatacatcatatatttgttaaatgtattgattagtg 97584
human.FAM230E-LOC105377182-GGT3P.NCBI.                                        ------------------------------------------------------------ 57310
human.FAM230J-LOC105372942-GGTLC5P.NCBI.rev.compl                             ------------------------------------------------------------ 46364
human.FAM230A.ncRNA.18487127.18500594-GGTLC3.18516335.18518165.rev.compl.REF  ------------------------------------------------------------ 31039

chimp.LOC112206744-LOC107973052-GGT2.revcompl                                 ------------------------------------------------------------ 61762
chimp54546-135457.revcompl.Pan.troglodytes.clone.rp43-41g5.GenBank:AC099533.36 ------------------------------------------------------------ 69849
human.FAM30B-LOC105372935-GGT2.NCBI.                                          ttacaacagacaattgcatgtgctatggaagcgtttggcagagagatctaatctaatctt 97644
human.FAM230E-LOC105377182-GGT3P.NCBI.                                        ------------------------------------------------------------ 57310
human.FAM230J-LOC105372942-GGTLC5P.NCBI.rev.compl                             ------------------------------------------------------------ 46364
human.FAM230A.ncRNA.18487127.18500594-GGTLC3.18516335.18518165.rev.compl.REF  ------------------------------------------------------------ 31039

chimp.LOC112206744-LOC107973052-GGT2.revcompl                                 ------------------------------------------------------------ 61762
chimp54546-135457.revcompl.Pan.troglodytes.clone.rp43-41g5.GenBank:AC099533.36 ------------------------------------------------------------ 69849
human.FAM30B-LOC105372935-GGT2.NCBI.                                          ggaaggtgaggatcctggccatcactaacttggaatttctgatactgatgatataaggtt 97704
human.FAM230E-LOC105377182-GGT3P.NCBI.                                        ------------------------------------------------------------ 57310
human.FAM230J-LOC105372942-GGTLC5P.NCBI.rev.compl                             ------------------------------------------------------------ 46364
human.FAM230A.ncRNA.18487127.18500594-GGTLC3.18516335.18518165.rev.compl.REF  ------------------------------------------------------------ 31039

chimp.LOC112206744-LOC107973052-GGT2.revcompl                                 ------------------------------------------------------------ 61762
chimp54546-135457.revcompl.Pan.troglodytes.clone.rp43-41g5.GenBank:AC099533.36 ------------------------------------------------------------ 69849
human.FAM30B-LOC105372935-GGT2.NCBI.                                          gagttatgtcttcgtgaaacaggtctctatacagcatataaacaactaccagcatgcaca 97764
human.FAM230E-LOC105377182-GGT3P.NCBI.                                        ------------------------------------------------------------ 57310
human.FAM230J-LOC105372942-GGTLC5P.NCBI.rev.compl                             ------------------------------------------------------------ 46364
human.FAM230A.ncRNA.18487127.18500594-GGTLC3.18516335.18518165.rev.compl.REF  ------------------------------------------------------------ 31039

chimp.LOC112206744-LOC107973052-GGT2.revcompl                                 ------------------------------------------------------------ 61762
chimp54546-135457.revcompl.Pan.troglodytes.clone.rp43-41g5.GenBank:AC099533.36 ------------------------------------------------------------ 69849
human.FAM30B-LOC105372935-GGT2.NCBI.                                          ggggttccgtagggactatttaaaatacttaagggaacaaacggttttcaagctcagtct 97824
human.FAM230E-LOC105377182-GGT3P.NCBI.                                        ------------------------------------------------------------ 57310
human.FAM230J-LOC105372942-GGTLC5P.NCBI.rev.compl                             ------------------------------------------------------------ 46364
human.FAM230A.ncRNA.18487127.18500594-GGTLC3.18516335.18518165.rev.compl.REF  ------------------------------------------------------------ 31039

chimp.LOC112206744-LOC107973052-GGT2.revcompl                                 ------------------------------------------------------------ 61762
chimp54546-135457.revcompl.Pan.troglodytes.clone.rp43-41g5.GenBank:AC099533.36 ------------------------------------------------------------ 69849
human.FAM30B-LOC105372935-GGT2.NCBI.                                          agtatttcagaaattccatttacataaacaattggtgcactaattgcaaatcattcttat 97884
human.FAM230E-LOC105377182-GGT3P.NCBI.                                        ------------------------------------------------------------ 57310
human.FAM230J-LOC105372942-GGTLC5P.NCBI.rev.compl                             ------------------------------------------------------------ 46364
human.FAM230A.ncRNA.18487127.18500594-GGTLC3.18516335.18518165.rev.compl.REF  ------------------------------------------------------------ 31039

chimp.LOC112206744-LOC107973052-GGT2.revcompl                                 ------------------------------------------------------------ 61762
chimp54546-135457.revcompl.Pan.troglodytes.clone.rp43-41g5.GenBank:AC099533.36 ------------------------------------------------------------ 69849
human.FAM30B-LOC105372935-GGT2.NCBI.                                          ttctctcctaaagcaggatcccctaaggatctttattaggcattctattagcctagtttg 97944
human.FAM230E-LOC105377182-GGT3P.NCBI.                                        ------------------------------------------------------------ 57310
human.FAM230J-LOC105372942-GGTLC5P.NCBI.rev.compl                             ------------------------------------------------------------ 46364
human.FAM230A.ncRNA.18487127.18500594-GGTLC3.18516335.18518165.rev.compl.REF  ------------------------------------------------------------ 31039

chimp.LOC112206744-LOC107973052-GGT2.revcompl                                 ------------------------------------------------------------ 61762
chimp54546-135457.revcompl.Pan.troglodytes.clone.rp43-41g5.GenBank:AC099533.36 -------------------------------------TGTTGCAAGCTCTAAAATAATCC 69872
human.FAM30B-LOC105372935-GGT2.NCBI.                                          agttacagtatttacttaaaagtaataacagtgaagttcttgaaaaatatttggtaattc 98004
human.FAM230E-LOC105377182-GGT3P.NCBI.                                        ------------------------------------------------------------ 57310
human.FAM230J-LOC105372942-GGTLC5P.NCBI.rev.compl                             ------------------------------------------------------------ 46364
human.FAM230A.ncRNA.18487127.18500594-GGTLC3.18516335.18518165.rev.compl.REF  ------------------------------------------------------------ 31039

chimp.LOC112206744-LOC107973052-GGT2.revcompl                                 ------------------------------------------------------------ 61762
chimp54546-135457.revcompl.Pan.troglodytes.clone.rp43-41g5.GenBank:AC099533.36 AGTCATCTTTTAAAATAACTCTAAGACTGCACAGTTATGAAACTAATAGAGAAGGAGGAA 69932
human.FAM30B-LOC105372935-GGT2.NCBI.                                          agacattttactaattcaaactaatcttcctcattagtctacgttgt--------tagag 98056
human.FAM230E-LOC105377182-GGT3P.NCBI.                                        ------------------------------------------------------------ 57310
human.FAM230J-LOC105372942-GGTLC5P.NCBI.rev.compl                             ------------------------------------------------------------ 46364
human.FAM230A.ncRNA.18487127.18500594-GGTLC3.18516335.18518165.rev.compl.REF  ------------------------------------------------------------ 31039
```

```
chimp.LOC112206744-LOC107973052-GGT2.revcompl                                        ------------------------------------------------------------ 61762
chimp54546-135457.revcompl.Pan.troglodytes.clone.rp43-41g5.GenBank:AC099533.36       ATTAAATCATAAAAATAATAAATCCAAAACAAGATGTGAGAGGAGATAAGAAGAAATAGA 69992
human.FAM30B-LOC105372935-GGT2.NCBI.                                                 gtttaaccattgtattaagagaaagtatcaaaggcttcac-ttagagtaatagtcaccaa 98115
human.FAM230E-LOC105377182-GGT3P.NCBI.                                               ------------------------------------------------------------ 57310
human.FAM230J-LOC105372942-GGTLC5P.NCBI.rev.compl                                    ------------------------------------------------------------ 46364
human.FAM230A.ncRNA.18487127.18500594-GGTLC3.18516335.18518165.rev.compl.REF         ------------------------------------------------------------ 31039

chimp.LOC112206744-LOC107973052-GGT2.revcompl                                        ------------------------------------------------------------ 61762
chimp54546-135457.revcompl.Pan.troglodytes.clone.rp43-41g5.GenBank:AC099533.36       ATAGGCATGGAAAACAAATTGGTGGTGGGTTTCAACCCAAATAAATCATTAGTTACATTT 70052
human.FAM30B-LOC105372935-GGT2.NCBI.                                                 atttttttgcaagacaccttggtagcaggaacctgagcta--acatcaggaggtaggtag 98173
human.FAM230E-LOC105377182-GGT3P.NCBI.                                               ------------------------------------------------------------ 57310
human.FAM230J-LOC105372942-GGTLC5P.NCBI.rev.compl                                    ------------------------------------------------------------ 46364
human.FAM230A.ncRNA.18487127.18500594-GGTLC3.18516335.18518165.rev.compl.REF         ------------------------------------------------------------ 31039

chimp.LOC112206744-LOC107973052-GGT2.revcompl                                        ------------------------------------------------------------ 61762
chimp54546-135457.revcompl.Pan.troglodytes.clone.rp43-41g5.GenBank:AC099533.36       AAAAGGACAATAAAAGTTAAAATAATTGAAAATAAAGTAAAACCCAACTAATGCC----- 70107
human.FAM30B-LOC105372935-GGT2.NCBI.                                                 aaatagaaaataaaggaatgacaatttgaagagcaactgcaatccaatttagtccaacag 98233
human.FAM230E-LOC105377182-GGT3P.NCBI.                                               ------------------------------------------------------------ 57310
human.FAM230J-LOC105372942-GGTLC5P.NCBI.rev.compl                                    ------------------------------------------------------------ 46364
human.FAM230A.ncRNA.18487127.18500594-GGTLC3.18516335.18518165.rev.compl.REF         ------------------------------------------------------------ 31039

chimp.LOC112206744-LOC107973052-GGT2.revcompl                                        ------------------------------------------------------------ 61762
chimp54546-135457.revcompl.Pan.troglodytes.clone.rp43-41g5.GenBank:AC099533.36       ------------------------------------------------------------ 70107
human.FAM30B-LOC105372935-GGT2.NCBI.                                                 atgacacacatatgaagtcacattcaaattataagcagagcacaaaaatattttagatcc 98293
human.FAM230E-LOC105377182-GGT3P.NCBI.                                               ------------------------------------------------------------ 57310
human.FAM230J-LOC105372942-GGTLC5P.NCBI.rev.compl                                    ------------------------------------------------------------ 46364
human.FAM230A.ncRNA.18487127.18500594-GGTLC3.18516335.18518165.rev.compl.REF         ------------------------------------------------------------ 31039

chimp.LOC112206744-LOC107973052-GGT2.revcompl                                        ------------------------------------------------------------ 61762
chimp54546-135457.revcompl.Pan.troglodytes.clone.rp43-41g5.GenBank:AC099533.36       ------------------------------------------------------------ 70107
human.FAM30B-LOC105372935-GGT2.NCBI.                                                 ttatcttttgattaacctggtatcattcagggaaaaatgtcagggaacaccactgaccag 98353
human.FAM230E-LOC105377182-GGT3P.NCBI.                                               ------------------------------------------------------------ 57310
human.FAM230J-LOC105372942-GGTLC5P.NCBI.rev.compl                                    ------------------------------------------------------------ 46364
human.FAM230A.ncRNA.18487127.18500594-GGTLC3.18516335.18518165.rev.compl.REF         ------------------------------------------------------------ 31039

chimp.LOC112206744-LOC107973052-GGT2.revcompl                                        ------------------------------------------------------------ 61762
chimp54546-135457.revcompl.Pan.troglodytes.clone.rp43-41g5.GenBank:AC099533.36       ------------------------------------------------------------ 70107
human.FAM30B-LOC105372935-GGT2.NCBI.                                                 cagtcctctcttatctattttgcagatcttatctatttttctccctgagaaccagccctg 98413
human.FAM230E-LOC105377182-GGT3P.NCBI.                                               ------------------------------------------------------------ 57310
human.FAM230J-LOC105372942-GGTLC5P.NCBI.rev.compl                                    ------------------------------------------------------------ 46364
human.FAM230A.ncRNA.18487127.18500594-GGTLC3.18516335.18518165.rev.compl.REF         ------------------------------------------------------------ 31039

chimp.LOC112206744-LOC107973052-GGT2.revcompl                                        ------------------------------------------------------------ 61762
chimp54546-135457.revcompl.Pan.troglodytes.clone.rp43-41g5.GenBank:AC099533.36       ------------------------------------------------------------ 70107
human.FAM30B-LOC105372935-GGT2.NCBI.                                                 gacacaaactgcataaacatcaatgagcctagacagggtcttcatgtgtgtttggcctgc 98473
human.FAM230E-LOC105377182-GGT3P.NCBI.                                               ------------------------------------------------------------ 57310
human.FAM230J-LOC105372942-GGTLC5P.NCBI.rev.compl                                    ------------------------------------------------------------ 46364
human.FAM230A.ncRNA.18487127.18500594-GGTLC3.18516335.18518165.rev.compl.REF         ------------------------------------------------------------ 31039

chimp.LOC112206744-LOC107973052-GGT2.revcompl                                        ------------------------------------------------------------ 61762
chimp54546-135457.revcompl.Pan.troglodytes.clone.rp43-41g5.GenBank:AC099533.36       ------------------------------------------------------------ 70107
human.FAM30B-LOC105372935-GGT2.NCBI.                                                 cattgttctctgtaccagtacggagatttagtaacacactggtcacattcatggggcatc 98533
human.FAM230E-LOC105377182-GGT3P.NCBI.                                               ------------------------------------------------------------ 57310
human.FAM230J-LOC105372942-GGTLC5P.NCBI.rev.compl                                    ------------------------------------------------------------ 46364
human.FAM230A.ncRNA.18487127.18500594-GGTLC3.18516335.18518165.rev.compl.REF         ------------------------------------------------------------ 31039

chimp.LOC112206744-LOC107973052-GGT2.revcompl                                        ------------------------------------------------------------ 61762
chimp54546-135457.revcompl.Pan.troglodytes.clone.rp43-41g5.GenBank:AC099533.36       ------------------------------------------------------------ 70107
human.FAM30B-LOC105372935-GGT2.NCBI.                                                 ttgaactctcttgacatggtaaatgaacagcaggtcctgttcccatgccaaaagcaaaca 98593
human.FAM230E-LOC105377182-GGT3P.NCBI.                                               ------------------------------------------------------------ 57310
human.FAM230J-LOC105372942-GGTLC5P.NCBI.rev.compl                                    ------------------------------------------------------------ 46364
human.FAM230A.ncRNA.18487127.18500594-GGTLC3.18516335.18518165.rev.compl.REF         ------------------------------------------------------------ 31039

chimp.LOC112206744-LOC107973052-GGT2.revcompl                                        ------------------------------------------------------------ 61762
chimp54546-135457.revcompl.Pan.troglodytes.clone.rp43-41g5.GenBank:AC099533.36       ------------------------------------------------------------ 70107
human.FAM30B-LOC105372935-GGT2.NCBI.                                                 aaaacctgtcaccagcaaggaagacacccagtctctccaaaggatgccaaagtccacatc 98653
human.FAM230E-LOC105377182-GGT3P.NCBI.                                               ------------------------------------------------------------ 57310
human.FAM230J-LOC105372942-GGTLC5P.NCBI.rev.compl                                    ------------------------------------------------------------ 46364
human.FAM230A.ncRNA.18487127.18500594-GGTLC3.18516335.18518165.rev.compl.REF         ------------------------------------------------------------ 31039

chimp.LOC112206744-LOC107973052-GGT2.revcompl                                        ------------------------------------------------------------ 61762
chimp54546-135457.revcompl.Pan.troglodytes.clone.rp43-41g5.GenBank:AC099533.36       ------------------------------------------------------------ 70107
human.FAM30B-LOC105372935-GGT2.NCBI.                                                 cagcctcctttgcagagcctcacaaatctctcggatgcaggcaggatgatcacgggctag 98713
human.FAM230E-LOC105377182-GGT3P.NCBI.                                               ------------------------------------------------------------ 57310
human.FAM230J-LOC105372942-GGTLC5P.NCBI.rev.compl                                    ------------------------------------------------------------ 46364
human.FAM230A.ncRNA.18487127.18500594-GGTLC3.18516335.18518165.rev.compl.REF         ------------------------------------------------------------ 31039

chimp.LOC112206744-LOC107973052-GGT2.revcompl                                        ------------------------------------------------------------ 61762
chimp54546-135457.revcompl.Pan.troglodytes.clone.rp43-41g5.GenBank:AC099533.36       ------------------------------------------------------------ 70107
human.FAM30B-LOC105372935-GGT2.NCBI.                                                 ggaagattatgctctgaatgtcttaagagattagttacttaaatcctccacatgacttc 98773
human.FAM230E-LOC105377182-GGT3P.NCBI.                                               ------------------------------------------------------------ 57310
human.FAM230J-LOC105372942-GGTLC5P.NCBI.rev.compl                                    ------------------------------------------------------------ 46364
human.FAM230A.ncRNA.18487127.18500594-GGTLC3.18516335.18518165.rev.compl.REF         ------------------------------------------------------------ 31039

chimp.LOC112206744-LOC107973052-GGT2.revcompl                                        ------------------------------------------------------------ 61762
chimp54546-135457.revcompl.Pan.troglodytes.clone.rp43-41g5.GenBank:AC099533.36       ------------------------------------------------------------ 70107
human.FAM30B-LOC105372935-GGT2.NCBI.                                                 agtcatccccgttgcagatcacagagtaaaaagcctacctgattaaagctgaatgcctat 98833
human.FAM230E-LOC105377182-GGT3P.NCBI.                                               ------------------------------------------------------------ 57310
human.FAM230J-LOC105372942-GGTLC5P.NCBI.rev.compl                                    ------------------------------------------------------------ 46364
human.FAM230A.ncRNA.18487127.18500594-GGTLC3.18516335.18518165.rev.compl.REF         ------------------------------------------------------------ 31039

chimp.LOC112206744-LOC107973052-GGT2.revcompl                                        ------------------------------------------------------------ 61762
chimp54546-135457.revcompl.Pan.troglodytes.clone.rp43-41g5.GenBank:AC099533.36       ------------------------------------------------------------ 70107
human.FAM30B-LOC105372935-GGT2.NCBI.                                                 acatttgcaaaagggaaaatatgcagaagaaaacgagagtcagatactgatgggcatagc 98893
human.FAM230E-LOC105377182-GGT3P.NCBI.                                               ------------------------------------------------------------ 57310
human.FAM230J-LOC105372942-GGTLC5P.NCBI.rev.compl                                    ------------------------------------------------------------ 46364
human.FAM230A.ncRNA.18487127.18500594-GGTLC3.18516335.18518165.rev.compl.REF         ------------------------------------------------------------ 31039

chimp.LOC112206744-LOC107973052-GGT2.revcompl                                        ------------------------------------------------------------ 61762
chimp54546-135457.revcompl.Pan.troglodytes.clone.rp43-41g5.GenBank:AC099533.36       ------------------------------------------------------------ 70107
human.FAM30B-LOC105372935-GGT2.NCBI.                                                 accagctctaggacatcagaggtgttgaataaatgatgacaaggaggcacacatagttct 98953
human.FAM230E-LOC105377182-GGT3P.NCBI.                                               ------------------------------------------------------------ 57310
human.FAM230J-LOC105372942-GGTLC5P.NCBI.rev.compl                                    ------------------------------------------------------------ 46364
human.FAM230A.ncRNA.18487127.18500594-GGTLC3.18516335.18518165.rev.compl.REF         ------------------------------------------------------------ 31039

chimp.LOC112206744-LOC107973052-GGT2.revcompl                                        ------------------------------------------------------------ 61762
chimp54546-135457.revcompl.Pan.troglodytes.clone.rp43-41g5.GenBank:AC099533.36       ------------------------------------------------------------ 70107
human.FAM30B-LOC105372935-GGT2.NCBI.                                                 gatatattcaccataaataccacagtcctcaaggctctctcaattacttacttcccccca 99013
human.FAM230E-LOC105377182-GGT3P.NCBI.                                               ------------------------------------------------------------ 57310
human.FAM230J-LOC105372942-GGTLC5P.NCBI.rev.compl                                    ------------------------------------------------------------ 46364
human.FAM230A.ncRNA.18487127.18500594-GGTLC3.18516335.18518165.rev.compl.REF         ------------------------------------------------------------ 31039

chimp.LOC112206744-LOC107973052-GGT2.revcompl                                        ------------------------------------------------------------ 61762
chimp54546-135457.revcompl.Pan.troglodytes.clone.rp43-41g5.GenBank:AC099533.36       ------------------------------------------------------------ 70107
human.FAM30B-LOC105372935-GGT2.NCBI.                                                 aatcagagactgaatctggccttatcttcaaatgaggcccagctctccccaaattccaaa 99073
human.FAM230E-LOC105377182-GGT3P.NCBI.                                               ------------------------------------------------------------ 57310
human.FAM230J-LOC105372942-GGTLC5P.NCBI.rev.compl                                    ------------------------------------------------------------ 46364
human.FAM230A.ncRNA.18487127.18500594-GGTLC3.18516335.18518165.rev.compl.REF         ------------------------------------------------------------ 31039

chimp.LOC112206744-LOC107973052-GGT2.revcompl                                        ------------------------------------------------------------ 61762
chimp54546-135457.revcompl.Pan.troglodytes.clone.rp43-41g5.GenBank:AC099533.36       ------------------------------------------------------------ 70107
human.FAM30B-LOC105372935-GGT2.NCBI.                                                 tatgggaaattacagctcctctgtggtagggggaagagtgggaaggcgaatgtttgtatt 99133
human.FAM230E-LOC105377182-GGT3P.NCBI.                                               ------------------------------------------------------------ 57310
human.FAM230J-LOC105372942-GGTLC5P.NCBI.rev.compl                                    ------------------------------------------------------------ 46364
human.FAM230A.ncRNA.18487127.18500594-GGTLC3.18516335.18518165.rev.compl.REF         ------------------------------------------------------------ 31039

chimp.LOC112206744-LOC107973052-GGT2.revcompl                                        ------------------------------------------------------------ 61762
```

```
chimp54546-135457.revcompl.Pan.troglodytes.clone.rp43-41g5.GenBank:AC099533.36      ------------------------------------------------------------ 70107
human.FAM30B-LOC105372935-GGT2.NCBI.                                                gcataacgttttaattaaaacaaagatgacgtgcaccagagtctctttctcatctcctgt 99193
human.FAM230E-LOC105377182-GGT3P.NCBI.                                              ------------------------------------------------------------ 57310
human.FAM230J-LOC105372942-GGTLC5P.NCBI.rev.compl                                   ------------------------------------------------------------ 46364
human.FAM230A.ncRNA.18487127.18500594-GGTLC3.18516335.18518165.rev.compl.REF        ------------------------------------------------------------ 31039

chimp.LOC112206744-LOC107973052-GGT2.revcompl                                       ------------------------------------------------------------ 61762
chimp54546-135457.revcompl.Pan.troglodytes.clone.rp43-41g5.GenBank:AC099533.36      ------------------------------------------------------------ 70107
human.FAM30B-LOC105372935-GGT2.NCBI.                                                taggggcgttttccagtactacttccaccctttacccacccaggtcaccatggtcttttt 99253
human.FAM230E-LOC105377182-GGT3P.NCBI.                                              ------------------------------------------------------------ 57310
human.FAM230J-LOC105372942-GGTLC5P.NCBI.rev.compl                                   ------------------------------------------------------------ 46364
human.FAM230A.ncRNA.18487127.18500594-GGTLC3.18516335.18518165.rev.compl.REF        ------------------------------------------------------------ 31039

chimp.LOC112206744-LOC107973052-GGT2.revcompl                                       ------------------------------------------------------------ 61762
chimp54546-135457.revcompl.Pan.troglodytes.clone.rp43-41g5.GenBank:AC099533.36      ------------------------------------------------------------ 70107
human.FAM30B-LOC105372935-GGT2.NCBI.                                                cttccctctaggacaattcacctttaaaataagaggattgggcaagaaatctacaaagat 99313
human.FAM230E-LOC105377182-GGT3P.NCBI.                                              ------------------------------------------------------------ 57310
human.FAM230J-LOC105372942-GGTLC5P.NCBI.rev.compl                                   ------------------------------------------------------------ 46364
human.FAM230A.ncRNA.18487127.18500594-GGTLC3.18516335.18518165.rev.compl.REF        ------------------------------------------------------------ 31039

chimp.LOC112206744-LOC107973052-GGT2.revcompl                                       ------------------------------------------------------------ 61762
chimp54546-135457.revcompl.Pan.troglodytes.clone.rp43-41g5.GenBank:AC099533.36      ------------------------------------------------------------ 70107
human.FAM30B-LOC105372935-GGT2.NCBI.                                                ttttccaaaatgaactcaggcctagaggcatattagttctctatcttgttgctcccttct 99373
human.FAM230E-LOC105377182-GGT3P.NCBI.                                              ------------------------------------------------------------ 57310
human.FAM230J-LOC105372942-GGTLC5P.NCBI.rev.compl                                   ------------------------------------------------------------ 46364
human.FAM230A.ncRNA.18487127.18500594-GGTLC3.18516335.18518165.rev.compl.REF        ------------------------------------------------------------ 31039

chimp.LOC112206744-LOC107973052-GGT2.revcompl                                       ------------------------------------------------------------ 61762
chimp54546-135457.revcompl.Pan.troglodytes.clone.rp43-41g5.GenBank:AC099533.36      ------------------------------------------------------------ 70107
human.FAM30B-LOC105372935-GGT2.NCBI.                                                agaagtgcacagttcagagggatgcccagccacagaggtgccaaaacgcagagctggaac 99433
human.FAM230E-LOC105377182-GGT3P.NCBI.                                              ------------------------------------------------------------ 57310
human.FAM230J-LOC105372942-GGTLC5P.NCBI.rev.compl                                   ------------------------------------------------------------ 46364
human.FAM230A.ncRNA.18487127.18500594-GGTLC3.18516335.18518165.rev.compl.REF        ------------------------------------------------------------ 31039

chimp.LOC112206744-LOC107973052-GGT2.revcompl                                       ------------------------------------------------------------ 61762
chimp54546-135457.revcompl.Pan.troglodytes.clone.rp43-41g5.GenBank:AC099533.36      ------------------------------------------------------------ 70107
human.FAM30B-LOC105372935-GGT2.NCBI.                                                caatcacctgctccaacagagacaaggtgaaagcactgggcagggtgctcagaatgtgct 99493
human.FAM230E-LOC105377182-GGT3P.NCBI.                                              ------------------------------------------------------------ 57310
human.FAM230J-LOC105372942-GGTLC5P.NCBI.rev.compl                                   ------------------------------------------------------------ 46364
human.FAM230A.ncRNA.18487127.18500594-GGTLC3.18516335.18518165.rev.compl.REF        ------------------------------------------------------------ 31039

chimp.LOC112206744-LOC107973052-GGT2.revcompl                                       ------------------------------------------------------------ 61762
chimp54546-135457.revcompl.Pan.troglodytes.clone.rp43-41g5.GenBank:AC099533.36      ------------------------------------------------------------ 70107
human.FAM30B-LOC105372935-GGT2.NCBI.                                                cttgcttctttgtaatgaaatatcagaagacagattgtaacattgttcagcaagctattt 99553
human.FAM230E-LOC105377182-GGT3P.NCBI.                                              ------------------------------------------------------------ 57310
human.FAM230J-LOC105372942-GGTLC5P.NCBI.rev.compl                                   ------------------------------------------------------------ 46364
human.FAM230A.ncRNA.18487127.18500594-GGTLC3.18516335.18518165.rev.compl.REF        ------------------------------------------------------------ 31039

chimp.LOC112206744-LOC107973052-GGT2.revcompl                                       ------------------------------------------------------------ 61762
chimp54546-135457.revcompl.Pan.troglodytes.clone.rp43-41g5.GenBank:AC099533.36      ------------------------------------------------------------ 70107
human.FAM30B-LOC105372935-GGT2.NCBI.                                                tttgtactgaaacatcagaaggcattatgaaactgttcagcaaatcatctctaaacaatg 99613
human.FAM230E-LOC105377182-GGT3P.NCBI.                                              ------------------------------------------------------------ 57310
human.FAM230J-LOC105372942-GGTLC5P.NCBI.rev.compl                                   ------------------------------------------------------------ 46364
human.FAM230A.ncRNA.18487127.18500594-GGTLC3.18516335.18518165.rev.compl.REF        ------------------------------------------------------------ 31039

chimp.LOC112206744-LOC107973052-GGT2.revcompl                                       ------------------------------------------------------------ 61762
chimp54546-135457.revcompl.Pan.troglodytes.clone.rp43-41g5.GenBank:AC099533.36      ------------------------------------------------------------ 70107
human.FAM30B-LOC105372935-GGT2.NCBI.                                                tcataaagggttacctagtttggggtaagcccggaatgaatatctgcaccatcattcctc 99673
human.FAM230E-LOC105377182-GGT3P.NCBI.                                              ------------------------------------------------------------ 57310
human.FAM230J-LOC105372942-GGTLC5P.NCBI.rev.compl                                   ------------------------------------------------------------ 46364
human.FAM230A.ncRNA.18487127.18500594-GGTLC3.18516335.18518165.rev.compl.REF        ------------------------------------------------------------ 31039

chimp.LOC112206744-LOC107973052-GGT2.revcompl                                       ------------------------------------------------------------ 61762
chimp54546-135457.revcompl.Pan.troglodytes.clone.rp43-41g5.GenBank:AC099533.36      ------------------------------------------------------------ 70107
human.FAM30B-LOC105372935-GGT2.NCBI.                                                ccgctaacaaacatgtccatctatgtgaagcataattcccaacacaccaccatctccaaa 99733
human.FAM230E-LOC105377182-GGT3P.NCBI.                                              ------------------------------------------------------------ 57310
human.FAM230J-LOC105372942-GGTLC5P.NCBI.rev.compl                                   ------------------------------------------------------------ 46364
human.FAM230A.ncRNA.18487127.18500594-GGTLC3.18516335.18518165.rev.compl.REF        ------------------------------------------------------------ 31039

chimp.LOC112206744-LOC107973052-GGT2.revcompl                                       ------------------------------------------------------------ 61762
chimp54546-135457.revcompl.Pan.troglodytes.clone.rp43-41g5.GenBank:AC099533.36      ------------------------------------------------------------ 70107
human.FAM30B-LOC105372935-GGT2.NCBI.                                                ccaacaccaaaccactgccaaacaaagctgaagaggaaaaccagaaaggtctccgagaag 99793
human.FAM230E-LOC105377182-GGT3P.NCBI.                                              ------------------------------------------------------------ 57310
human.FAM230J-LOC105372942-GGTLC5P.NCBI.rev.compl                                   ------------------------------------------------------------ 46364
human.FAM230A.ncRNA.18487127.18500594-GGTLC3.18516335.18518165.rev.compl.REF        ------------------------------------------------------------ 31039

chimp.LOC112206744-LOC107973052-GGT2.revcompl                                       ------------------------------------------------------------ 61762
chimp54546-135457.revcompl.Pan.troglodytes.clone.rp43-41g5.GenBank:AC099533.36      ------------------------------------------------------------ 70107
human.FAM30B-LOC105372935-GGT2.NCBI.                                                tgctgcttgcagggagaagaatggccctgcacagaggctcctgagggcagcaggtgcctg 99853
human.FAM230E-LOC105377182-GGT3P.NCBI.                                              ------------------------------------------------------------ 57310
human.FAM230J-LOC105372942-GGTLC5P.NCBI.rev.compl                                   ------------------------------------------------------------ 46364
human.FAM230A.ncRNA.18487127.18500594-GGTLC3.18516335.18518165.rev.compl.REF        ------------------------------------------------------------ 31039

chimp.LOC112206744-LOC107973052-GGT2.revcompl                                       ------------------------------------------------------------ 61762
chimp54546-135457.revcompl.Pan.troglodytes.clone.rp43-41g5.GenBank:AC099533.36      ------------------------------------------------------------ 70107
human.FAM30B-LOC105372935-GGT2.NCBI.                                                ctttggagccttggcaatttccaccaggcagaatttacaaccgtctgggacctgcagtct 99913
human.FAM230E-LOC105377182-GGT3P.NCBI.                                              ------------------------------------------------------------ 57310
human.FAM230J-LOC105372942-GGTLC5P.NCBI.rev.compl                                   ------------------------------------------------------------ 46364
human.FAM230A.ncRNA.18487127.18500594-GGTLC3.18516335.18518165.rev.compl.REF        ------------------------------------------------------------ 31039

chimp.LOC112206744-LOC107973052-GGT2.revcompl                                       ------------------------------------------------------------ 61762
chimp54546-135457.revcompl.Pan.troglodytes.clone.rp43-41g5.GenBank:AC099533.36      ------------------------------------------------------------ 70107
human.FAM30B-LOC105372935-GGT2.NCBI.                                                cagagggctcacggagaaggggaagctcttggctacccgggaggtcaccagtgactctg 99973
human.FAM230E-LOC105377182-GGT3P.NCBI.                                              ------------------------------------------------------------ 57310
human.FAM230J-LOC105372942-GGTLC5P.NCBI.rev.compl                                   ------------------------------------------------------------ 46364
human.FAM230A.ncRNA.18487127.18500594-GGTLC3.18516335.18518165.rev.compl.REF        ------------------------------------------------------------ 31039

chimp.LOC112206744-LOC107973052-GGT2.revcompl                                       ------------------------------------------------------------ 61762
chimp54546-135457.revcompl.Pan.troglodytes.clone.rp43-41g5.GenBank:AC099533.36      ------------------------------------------------------------ 70107
human.FAM30B-LOC105372935-GGT2.NCBI.                                                agcaaagtgcagatcaatgcctgcttagggttacccccaggctgggagagggcatctggc 100033
human.FAM230E-LOC105377182-GGT3P.NCBI.                                              ------------------------------------------------------------ 57310
human.FAM230J-LOC105372942-GGTLC5P.NCBI.rev.compl                                   ------------------------------------------------------------ 46364
human.FAM230A.ncRNA.18487127.18500594-GGTLC3.18516335.18518165.rev.compl.REF        ------------------------------------------------------------ 31039

chimp.LOC112206744-LOC107973052-GGT2.revcompl                                       ------------------------------------------------------------ 61762
chimp54546-135457.revcompl.Pan.troglodytes.clone.rp43-41g5.GenBank:AC099533.36      ------------------------------------------------------------ 70107
human.FAM30B-LOC105372935-GGT2.NCBI.                                                aaggaacagacctggaagagcctgcccctctttgagctgctacagcagcttctgggaatt 100093
human.FAM230E-LOC105377182-GGT3P.NCBI.                                              ------------------------------------------------------------ 57310
human.FAM230J-LOC105372942-GGTLC5P.NCBI.rev.compl                                   ------------------------------------------------------------ 46364
human.FAM230A.ncRNA.18487127.18500594-GGTLC3.18516335.18518165.rev.compl.REF        ------------------------------------------------------------ 31039

chimp.LOC112206744-LOC107973052-GGT2.revcompl                                       ------------------------------------------------------------ 61762
chimp54546-135457.revcompl.Pan.troglodytes.clone.rp43-41g5.GenBank:AC099533.36      ------------------------------------------------------------ 70107
human.FAM30B-LOC105372935-GGT2.NCBI.                                                tgtaagagccatactgatgagggtcccacaccctcccttgccagggcgccctcagggagg 100153
human.FAM230E-LOC105377182-GGT3P.NCBI.                                              ------------------------------------------------------------ 57310
human.FAM230J-LOC105372942-GGTLC5P.NCBI.rev.compl                                   ------------------------------------------------------------ 46364
human.FAM230A.ncRNA.18487127.18500594-GGTLC3.18516335.18518165.rev.compl.REF        ------------------------------------------------------------ 31039

chimp.LOC112206744-LOC107973052-GGT2.revcompl                                       ------------------------------------------------------------ 61762
chimp54546-135457.revcompl.Pan.troglodytes.clone.rp43-41g5.GenBank:AC099533.36      ------------------------------------------------------------ 70107
human.FAM30B-LOC105372935-GGT2.NCBI.                                                ccacactgcaggaggcagagtatgaggtggggaagatggatcggccttatcctcaccacc 100213
human.FAM230E-LOC105377182-GGT3P.NCBI.                                              ------------------------------------------------------------ 57310
human.FAM230J-LOC105372942-GGTLC5P.NCBI.rev.compl                                   ------------------------------------------------------------ 46364
human.FAM230A.ncRNA.18487127.18500594-GGTLC3.18516335.18518165.rev.compl.REF        ------------------------------------------------------------ 31039

chimp.LOC112206744-LOC107973052-GGT2.revcompl                                       ------------------------------------------------------------ 61762
chimp54546-135457.revcompl.Pan.troglodytes.clone.rp43-41g5.GenBank:AC099533.36      ------------------------------------------------------------ 70107
```

```
human.FAM30B-LOC105372935-GGT2.NCBI.    ccccatacgagtctcctctggtatggatggtgtctcctctctggtgggaagtatgtgatt 100273
human.FAM230E-LOC105377182-GGT3P.NCBI.    ------------------------------------------------------------ 57310
human.FAM230J-LOC105372942-GGTLC5P.NCBI.rev.compl    ------------------------------------------------------------ 46364
human.FAM230A.ncRNA.18487127.18500594-GGTLC3.18516335.18518165.rev.compl.REF    ------------------------------------------------------------ 31039

chimp.LOC112206744-LOC107973052-GGT2.revcompl    ------------------------------------------------------------ 61762
chimp54546-135457.revcompl.Pan.troglodytes.clone.rp43-41g5.GenBank:AC099533.36    ------------------------------------------------------------ 70107
human.FAM30B-LOC105372935-GGT2.NCBI.    tctctatttctatttctcttttgttcgggcttcctagaaattcagagctaggtgtgaaat 100333
human.FAM230E-LOC105377182-GGT3P.NCBI.    ------------------------------------------------------------ 57310
human.FAM230J-LOC105372942-GGTLC5P.NCBI.rev.compl    ------------------------------------------------------------ 46364
human.FAM230A.ncRNA.18487127.18500594-GGTLC3.18516335.18518165.rev.compl.REF    ------------------------------------------------------------ 31039

chimp.LOC112206744-LOC107973052-GGT2.revcompl    ------------------------------------------------------------ 61762
chimp54546-135457.revcompl.Pan.troglodytes.clone.rp43-41g5.GenBank:AC099533.36    ------------------------------------------------------------ 70107
human.FAM30B-LOC105372935-GGT2.NCBI.    tcccaattcccacagacaagcaacatccccagcagtaataaggacgttccacacacccag 100393
human.FAM230E-LOC105377182-GGT3P.NCBI.    ------------------------------------------------------------ 57310
human.FAM230J-LOC105372942-GGTLC5P.NCBI.rev.compl    ------------------------------------------------------------ 46364
human.FAM230A.ncRNA.18487127.18500594-GGTLC3.18516335.18518165.rev.compl.REF    ------------------------------------------------------------ 31039

chimp.LOC112206744-LOC107973052-GGT2.revcompl    ------------------------------------------------------------ 61762
chimp54546-135457.revcompl.Pan.troglodytes.clone.rp43-41g5.GenBank:AC099533.36    ------------------------------------------------------------ 70107
human.FAM30B-LOC105372935-GGT2.NCBI.    gacttgggatatttgtgtattttcaagaataaaagtttgccctttagcctcctcagagcc 100453
human.FAM230E-LOC105377182-GGT3P.NCBI.    ------------------------------------------------------------ 57310
human.FAM230J-LOC105372942-GGTLC5P.NCBI.rev.compl    ------------------------------------------------------------ 46364
human.FAM230A.ncRNA.18487127.18500594-GGTLC3.18516335.18518165.rev.compl.REF    ------------------------------------------------------------ 31039

chimp.LOC112206744-LOC107973052-GGT2.revcompl    ------------------------------------------------------------ 61762
chimp54546-135457.revcompl.Pan.troglodytes.clone.rp43-41g5.GenBank:AC099533.36    ------------------------------------------------------------ 70107
human.FAM30B-LOC105372935-GGT2.NCBI.    ccctcccttctcctcgctctccccttaccactgctattcatcatcttactcctacagata 100513
human.FAM230E-LOC105377182-GGT3P.NCBI.    ------------------------------------------------------------ 57310
human.FAM230J-LOC105372942-GGTLC5P.NCBI.rev.compl    ------------------------------------------------------------ 46364
human.FAM230A.ncRNA.18487127.18500594-GGTLC3.18516335.18518165.rev.compl.REF    ------------------------------------------------------------ 31039

chimp.LOC112206744-LOC107973052-GGT2.revcompl    ------------------------------------------------------------ 61762
chimp54546-135457.revcompl.Pan.troglodytes.clone.rp43-41g5.GenBank:AC099533.36    ------------------------------------------------------------ 70107
human.FAM30B-LOC105372935-GGT2.NCBI.    ccccctcttcgtggtggcaccgggcactagtaactcatggctgtaaagaagagctgagaa 100573
human.FAM230E-LOC105377182-GGT3P.NCBI.    ------------------------------------------------------------ 57310
human.FAM230J-LOC105372942-GGTLC5P.NCBI.rev.compl    ------------------------------------------------------------ 46364
human.FAM230A.ncRNA.18487127.18500594-GGTLC3.18516335.18518165.rev.compl.REF    ------------------------------------------------------------ 31039

chimp.LOC112206744-LOC107973052-GGT2.revcompl    ------------------------------------------------------------ 61762
chimp54546-135457.revcompl.Pan.troglodytes.clone.rp43-41g5.GenBank:AC099533.36    ------------------------------------------------------------ 70107
human.FAM30B-LOC105372935-GGT2.NCBI.    caaaacaggaagaaaagaggttaagtaatggcaagaaaaaagatgtaccttaggaataaa 100633
human.FAM230E-LOC105377182-GGT3P.NCBI.    ------------------------------------------------------------ 57310
human.FAM230J-LOC105372942-GGTLC5P.NCBI.rev.compl    ------------------------------------------------------------ 46364
human.FAM230A.ncRNA.18487127.18500594-GGTLC3.18516335.18518165.rev.compl.REF    ------------------------------------------------------------ 31039

chimp.LOC112206744-LOC107973052-GGT2.revcompl    ------------------------------------------------------------ 61762
chimp54546-135457.revcompl.Pan.troglodytes.clone.rp43-41g5.GenBank:AC099533.36    ------------------------------------------------------------ 70107
human.FAM30B-LOC105372935-GGT2.NCBI.    aaagagttcccgaatgtcacagtgtatttaattaatgcttctatttattaaagattaaaa 100693
human.FAM230E-LOC105377182-GGT3P.NCBI.    ------------------------------------------------------------ 57310
human.FAM230J-LOC105372942-GGTLC5P.NCBI.rev.compl    ------------------------------------------------------------ 46364
human.FAM230A.ncRNA.18487127.18500594-GGTLC3.18516335.18518165.rev.compl.REF    ------------------------------------------------------------ 31039

chimp.LOC112206744-LOC107973052-GGT2.revcompl    ------------------------------------------------------------ 61762
chimp54546-135457.revcompl.Pan.troglodytes.clone.rp43-41g5.GenBank:AC099533.36    ------------------------------------------------------------ 70107
human.FAM30B-LOC105372935-GGT2.NCBI.    atacatatcaggtgacgacttaaaatgttagtcatgattttaactgtgaattctgtggca 100753
human.FAM230E-LOC105377182-GGT3P.NCBI.    ------------------------------------------------------------ 57310
human.FAM230J-LOC105372942-GGTLC5P.NCBI.rev.compl    ------------------------------------------------------------ 46364
human.FAM230A.ncRNA.18487127.18500594-GGTLC3.18516335.18518165.rev.compl.REF    ------------------------------------------------------------ 31039

chimp.LOC112206744-LOC107973052-GGT2.revcompl    ------------------------------------------------------------ 61762
chimp54546-135457.revcompl.Pan.troglodytes.clone.rp43-41g5.GenBank:AC099533.36    ------------------------------------------------------------ 70107
human.FAM30B-LOC105372935-GGT2.NCBI.    ccggagataacagaacaatgagtgcttgtagaaggcactcggttatttgttgaatgaact 100813
human.FAM230E-LOC105377182-GGT3P.NCBI.    ------------------------------------------------------------ 57310
human.FAM230J-LOC105372942-GGTLC5P.NCBI.rev.compl    ------------------------------------------------------------ 46364
human.FAM230A.ncRNA.18487127.18500594-GGTLC3.18516335.18518165.rev.compl.REF    ------------------------------------------------------------ 31039

chimp.LOC112206744-LOC107973052-GGT2.revcompl    ------------------------------------------------------------ 61762
chimp54546-135457.revcompl.Pan.troglodytes.clone.rp43-41g5.GenBank:AC099533.36    ------------------------------------------------------------ 70107
human.FAM30B-LOC105372935-GGT2.NCBI.    tcttgggaaagccttcttaggaaagtattagaagtagattctgctaaaacaggggctacc 100873
human.FAM230E-LOC105377182-GGT3P.NCBI.    ------------------------------------------------------------ 57310
human.FAM230J-LOC105372942-GGTLC5P.NCBI.rev.compl    ------------------------------------------------------------ 46364
human.FAM230A.ncRNA.18487127.18500594-GGTLC3.18516335.18518165.rev.compl.REF    ------------------------------------------------------------ 31039

chimp.LOC112206744-LOC107973052-GGT2.revcompl    ------------------------------------------------------------ 61762
chimp54546-135457.revcompl.Pan.troglodytes.clone.rp43-41g5.GenBank:AC099533.36    ------------------------------------------------------------ 70107
human.FAM30B-LOC105372935-GGT2.NCBI.    ctcagaggtctcaggacaagggatagaaagtcaaacactgatgtttaagaagggatgcct 100933
human.FAM230E-LOC105377182-GGT3P.NCBI.    ------------------------------------------------------------ 57310
human.FAM230J-LOC105372942-GGTLC5P.NCBI.rev.compl    ------------------------------------------------------------ 46364
human.FAM230A.ncRNA.18487127.18500594-GGTLC3.18516335.18518165.rev.compl.REF    ------------------------------------------------------------ 31039

chimp.LOC112206744-LOC107973052-GGT2.revcompl    ------------------------------------------------------------ 61762
chimp54546-135457.revcompl.Pan.troglodytes.clone.rp43-41g5.GenBank:AC099533.36    ------------------------------------------------------------ 70107
human.FAM30B-LOC105372935-GGT2.NCBI.    atgacacatgaatcccacagccatctctccagaagagcaactatttgactgcgcatgcg 100993
human.FAM230E-LOC105377182-GGT3P.NCBI.    ------------------------------------------------------------ 57310
human.FAM230J-LOC105372942-GGTLC5P.NCBI.rev.compl    ------------------------------------------------------------ 46364
human.FAM230A.ncRNA.18487127.18500594-GGTLC3.18516335.18518165.rev.compl.REF    ------------------------------------------------------------ 31039

chimp.LOC112206744-LOC107973052-GGT2.revcompl    ------------------------------------------------------------ 61762
chimp54546-135457.revcompl.Pan.troglodytes.clone.rp43-41g5.GenBank:AC099533.36    ------------------------------------------------------------ 70107
human.FAM30B-LOC105372935-GGT2.NCBI.    ggaagatggcgggccgggcgacttgagatccgcgggtctccctgctccttttccgtctgc 101053
human.FAM230E-LOC105377182-GGT3P.NCBI.    ------------------------------------------------------------ 57310
human.FAM230J-LOC105372942-GGTLC5P.NCBI.rev.compl    ------------------------------------------------------------ 46364
human.FAM230A.ncRNA.18487127.18500594-GGTLC3.18516335.18518165.rev.compl.REF    ------------------------------------------------------------ 31039

chimp.LOC112206744-LOC107973052-GGT2.revcompl    ------------------------------------------------------------ 61762
chimp54546-135457.revcompl.Pan.troglodytes.clone.rp43-41g5.GenBank:AC099533.36    ------------------------------------------------------------ 70107
human.FAM30B-LOC105372935-GGT2.NCBI.    gtcgggagctcccgggcacgtgaggccgtgccgcgtttactggcgggagggacggcctag 101113
human.FAM230E-LOC105377182-GGT3P.NCBI.    ------------------------------------------------------------ 57310
human.FAM230J-LOC105372942-GGTLC5P.NCBI.rev.compl    ------------------------------------------------------------ 46364
human.FAM230A.ncRNA.18487127.18500594-GGTLC3.18516335.18518165.rev.compl.REF    ------------------------------------------------------------ 31039

chimp.LOC112206744-LOC107973052-GGT2.revcompl    ------------------------------------------------------------ 61762
chimp54546-135457.revcompl.Pan.troglodytes.clone.rp43-41g5.GenBank:AC099533.36    ------------------------------------------------------------ 70107
human.FAM30B-LOC105372935-GGT2.NCBI.    ccgggcgacgcctcggaggaggccgcggaccccttaggtgctgggcccttggaaatcggc 101173
human.FAM230E-LOC105377182-GGT3P.NCBI.    ------------------------------------------------------------ 57310
human.FAM230J-LOC105372942-GGTLC5P.NCBI.rev.compl    ------------------------------------------------------------ 46364
human.FAM230A.ncRNA.18487127.18500594-GGTLC3.18516335.18518165.rev.compl.REF    ------------------------------------------------------------ 31039

chimp.LOC112206744-LOC107973052-GGT2.revcompl    ------------------------------------------------------------ 61762
chimp54546-135457.revcompl.Pan.troglodytes.clone.rp43-41g5.GenBank:AC099533.36    ------------------------------------------------------------ 70107
human.FAM30B-LOC105372935-GGT2.NCBI.    gcgtggggggcggtgctcgagctgagcgcgagagggcgggagagctcgtggggtgcgagg 101233
human.FAM230E-LOC105377182-GGT3P.NCBI.    ------------------------------------------------------------ 57310
human.FAM230J-LOC105372942-GGTLC5P.NCBI.rev.compl    ------------------------------------------------------------ 46364
human.FAM230A.ncRNA.18487127.18500594-GGTLC3.18516335.18518165.rev.compl.REF    ------------------------------------------------------------ 31039

chimp.LOC112206744-LOC107973052-GGT2.revcompl    ------------------------------------------------------------ 61762
chimp54546-135457.revcompl.Pan.troglodytes.clone.rp43-41g5.GenBank:AC099533.36    ------------------------------------------------------------ 70107
human.FAM30B-LOC105372935-GGT2.NCBI.    ggagcaggacgcccggccgggcagcatgagtcagcagcggccggcgaggagattacccag 101293
human.FAM230E-LOC105377182-GGT3P.NCBI.    ------------------------------------------------------------ 57310
human.FAM230J-LOC105372942-GGTLC5P.NCBI.rev.compl    ------------------------------------------------------------ 46364
human.FAM230A.ncRNA.18487127.18500594-GGTLC3.18516335.18518165.rev.compl.REF    ------------------------------------------------------------ 31039

chimp.LOC112206744-LOC107973052-GGT2.revcompl    ------------------------------------------------------------ 61762
chimp54546-135457.revcompl.Pan.troglodytes.clone.rp43-41g5.GenBank:AC099533.36    ------------------------------------------------------------ 70107
human.FAM30B-LOC105372935-GGT2.NCBI.    tctcctcctgcacccgacggaggagaccccatcaacgtggagggcctgctgccatcaaaa 101353
```

```
human.FAM230E-LOC105377182-GGT3P.NCBI.                                              ------------------------------------------------------------ 57310
human.FAM230J-LOC105372942-GGTLC5P.NCBI.rev.compl                                   ------------------------------------------------------------ 46364
human.FAM230A.ncRNA.18487127.18500594-GGTLC3.18516335.18518165.rev.compl.REF        ------------------------------------------------------------ 31039

chimp.LOC112206744-LOC107973052-GGT2.revcompl                                       ------------------------------------------------------------ 61762
chimp54546-135457.revcompl.Pan.troglodytes.clone.rp43-41g5.GenBank:AC099533.36      ------------------------------------------------------------ 70107
human.FAM30B-LOC105372935-GGT2.NCBI.                                                ataaggattaatttagaagataatgtacaatatgtgtccatgagaaatctgctcccgggg 101413
human.FAM230E-LOC105377182-GGT3P.NCBI.                                              ------------------------------------------------------------ 57310
human.FAM230J-LOC105372942-GGTLC5P.NCBI.rev.compl                                   ------------------------------------------------------------ 46364
human.FAM230A.ncRNA.18487127.18500594-GGTLC3.18516335.18518165.rev.compl.REF        ------------------------------------------------------------ 31039

chimp.LOC112206744-LOC107973052-GGT2.revcompl                                       ------------------------------------------------------------ 61762
chimp54546-135457.revcompl.Pan.troglodytes.clone.rp43-41g5.GenBank:AC099533.36      ------------------------------------------------------------ 70107
human.FAM30B-LOC105372935-GGT2.NCBI.                                                gtattcttgacttaaacaaggttgcaacgaaactgggagtccgaaagcggagagtgtatg 101473
human.FAM230E-LOC105377182-GGT3P.NCBI.                                              ------------------------------------------------------------ 57310
human.FAM230J-LOC105372942-GGTLC5P.NCBI.rev.compl                                   ------------------------------------------------------------ 46364
human.FAM230A.ncRNA.18487127.18500594-GGTLC3.18516335.18518165.rev.compl.REF        ------------------------------------------------------------ 31039

chimp.LOC112206744-LOC107973052-GGT2.revcompl                                       ------------------------------------------------------------ 61762
chimp54546-135457.revcompl.Pan.troglodytes.clone.rp43-41g5.GenBank:AC099533.36      ------------------------------------------------------------ 70107
human.FAM30B-LOC105372935-GGT2.NCBI.                                                acatcaccgatgtcttagatggaatcgacctcgttgaaaagaaatccaagaaccatatta 101533
human.FAM230E-LOC105377182-GGT3P.NCBI.                                              ------------------------------------------------------------ 57310
human.FAM230J-LOC105372942-GGTLC5P.NCBI.rev.compl                                   ------------------------------------------------------------ 46364
human.FAM230A.ncRNA.18487127.18500594-GGTLC3.18516335.18518165.rev.compl.REF        ------------------------------------------------------------ 31039

chimp.LOC112206744-LOC107973052-GGT2.revcompl                                       ------------------------------------------------------------ 61762
chimp54546-135457.revcompl.Pan.troglodytes.clone.rp43-41g5.GenBank:AC099533.36      ------------------------------------------------------------ 70107
human.FAM30B-LOC105372935-GGT2.NCBI.                                                gatggataggatctgatcttagcaattttggagcagttccccaacaaaagaagctacagg 101593
human.FAM230E-LOC105377182-GGT3P.NCBI.                                              ------------------------------------------------------------ 57310
human.FAM230J-LOC105372942-GGTLC5P.NCBI.rev.compl                                   ------------------------------------------------------------ 46364
human.FAM230A.ncRNA.18487127.18500594-GGTLC3.18516335.18518165.rev.compl.REF        ------------------------------------------------------------ 31039

chimp.LOC112206744-LOC107973052-GGT2.revcompl                                       ------------------------------------------------------------ 61762
chimp54546-135457.revcompl.Pan.troglodytes.clone.rp43-41g5.GenBank:AC099533.36      ------------------------------------------------------------ 70107
human.FAM30B-LOC105372935-GGT2.NCBI.                                                aggaactttctgacttaccagcaatggaagatgctttggatgagttaattaaggattgtg 101653
human.FAM230E-LOC105377182-GGT3P.NCBI.                                              ------------------------------------------------------------ 57310
human.FAM230J-LOC105372942-GGTLC5P.NCBI.rev.compl                                   ------------------------------------------------------------ 46364
human.FAM230A.ncRNA.18487127.18500594-GGTLC3.18516335.18518165.rev.compl.REF        ------------------------------------------------------------ 31039

chimp.LOC112206744-LOC107973052-GGT2.revcompl                                       ------------------------------------------------------------ 61762
chimp54546-135457.revcompl.Pan.troglodytes.clone.rp43-41g5.GenBank:AC099533.36      ------------------------------------------------------------ 70107
human.FAM30B-LOC105372935-GGT2.NCBI.                                                ctcagcagctgtttgagttaacagatgacaaagaaaatgaaagactagcatatgtgacct 101713
human.FAM230E-LOC105377182-GGT3P.NCBI.                                              ------------------------------------------------------------ 57310
human.FAM230J-LOC105372942-GGTLC5P.NCBI.rev.compl                                   ------------------------------------------------------------ 46364
human.FAM230A.ncRNA.18487127.18500594-GGTLC3.18516335.18518165.rev.compl.REF        ------------------------------------------------------------ 31039

chimp.LOC112206744-LOC107973052-GGT2.revcompl                                       ------------------------------------------------------------ 61762
chimp54546-135457.revcompl.Pan.troglodytes.clone.rp43-41g5.GenBank:AC099533.36      ------------------------------------------------------------ 70107
human.FAM30B-LOC105372935-GGT2.NCBI.                                                atcaagacattcatagcattcaggccttccatgaacagatcgtcattgcagttaaagctc 101773
human.FAM230E-LOC105377182-GGT3P.NCBI.                                              ------------------------------------------------------------ 57310
human.FAM230J-LOC105372942-GGTLC5P.NCBI.rev.compl                                   ------------------------------------------------------------ 46364
human.FAM230A.ncRNA.18487127.18500594-GGTLC3.18516335.18518165.rev.compl.REF        ------------------------------------------------------------ 31039

chimp.LOC112206744-LOC107973052-GGT2.revcompl                                       ------------------------------------------------------------ 61762
chimp54546-135457.revcompl.Pan.troglodytes.clone.rp43-41g5.GenBank:AC099533.36      ------------------------------------------------------------ 70107
human.FAM30B-LOC105372935-GGT2.NCBI.                                                cagcagaaaccagattggatgttccagctcccagagaagactctatcacagtgcacataa 101833
human.FAM230E-LOC105377182-GGT3P.NCBI.                                              ------------------------------------------------------------ 57310
human.FAM230J-LOC105372942-GGTLC5P.NCBI.rev.compl                                   ------------------------------------------------------------ 46364
human.FAM230A.ncRNA.18487127.18500594-GGTLC3.18516335.18518165.rev.compl.REF        ------------------------------------------------------------ 31039

chimp.LOC112206744-LOC107973052-GGT2.revcompl                                       ------------------------------------------------------------ 61762
chimp54546-135457.revcompl.Pan.troglodytes.clone.rp43-41g5.GenBank:AC099533.36      ------------------------------------------------------------ 70107
human.FAM30B-LOC105372935-GGT2.NCBI.                                                ggagcaccaacggacctatcgatgtctatttgtgagaagtggagcagggtcagaccagta 101893
human.FAM230E-LOC105377182-GGT3P.NCBI.                                              ------------------------------------------------------------ 57310
human.FAM230J-LOC105372942-GGTLC5P.NCBI.rev.compl                                   ------------------------------------------------------------ 46364
human.FAM230A.ncRNA.18487127.18500594-GGTLC3.18516335.18518165.rev.compl.REF        ------------------------------------------------------------ 31039

chimp.LOC112206744-LOC107973052-GGT2.revcompl                                       ------------------------------------------------------------ 61762
chimp54546-135457.revcompl.Pan.troglodytes.clone.rp43-41g5.GenBank:AC099533.36      ------------------------------------------------------------ 70107
human.FAM30B-LOC105372935-GGT2.NCBI.                                                acaaaaggtctgaaggtgtcaggacctcttcatctgagagcactcatccagaaggccctg 101953
human.FAM230E-LOC105377182-GGT3P.NCBI.                                              ------------------------------------------------------------ 57310
human.FAM230J-LOC105372942-GGTLC5P.NCBI.rev.compl                                   ------------------------------------------------------------ 46364
human.FAM230A.ncRNA.18487127.18500594-GGTLC3.18516335.18518165.rev.compl.REF        ------------------------------------------------------------ 31039

chimp.LOC112206744-LOC107973052-GGT2.revcompl                                       ------------------------------------------------------------ 61762
chimp54546-135457.revcompl.Pan.troglodytes.clone.rp43-41g5.GenBank:AC099533.36      ------------------------------------------------------------ 70107
human.FAM30B-LOC105372935-GGT2.NCBI.                                                aggaagaagaaaatcctcagcaaagtgaagaattgcttgaagtaagcaactgatggcatt 102013
human.FAM230E-LOC105377182-GGT3P.NCBI.                                              ------------------------------------------------------------ 57310
human.FAM230J-LOC105372942-GGTLC5P.NCBI.rev.compl                                   ------------------------------------------------------------ 46364
human.FAM230A.ncRNA.18487127.18500594-GGTLC3.18516335.18518165.rev.compl.REF        ------------------------------------------------------------ 31039

chimp.LOC112206744-LOC107973052-GGT2.revcompl                                       ------------------------------------------------------------ 61762
chimp54546-135457.revcompl.Pan.troglodytes.clone.rp43-41g5.GenBank:AC099533.36      ------------------------------------------------------------ 70107
human.FAM30B-LOC105372935-GGT2.NCBI.                                                tgagaatttatgtatcactgagtttttgggaatatcttcctggagaattacgcatcaaa 102073
human.FAM230E-LOC105377182-GGT3P.NCBI.                                              ------------------------------------------------------------ 57310
human.FAM230J-LOC105372942-GGTLC5P.NCBI.rev.compl                                   ------------------------------------------------------------ 46364
human.FAM230A.ncRNA.18487127.18500594-GGTLC3.18516335.18518165.rev.compl.REF        ------------------------------------------------------------ 31039

chimp.LOC112206744-LOC107973052-GGT2.revcompl                                       ------------------------------------------------------------ 61762
chimp54546-135457.revcompl.Pan.troglodytes.clone.rp43-41g5.GenBank:AC099533.36      ------------------------------------------------------------ 70107
human.FAM30B-LOC105372935-GGT2.NCBI.                                                tttgattctcagagcaataaattatccatgaagtgctctcgttctcagtagcggcatcat 102133
human.FAM230E-LOC105377182-GGT3P.NCBI.                                              ------------------------------------------------------------ 57310
human.FAM230J-LOC105372942-GGTLC5P.NCBI.rev.compl                                   ------------------------------------------------------------ 46364
human.FAM230A.ncRNA.18487127.18500594-GGTLC3.18516335.18518165.rev.compl.REF        ------------------------------------------------------------ 31039

chimp.LOC112206744-LOC107973052-GGT2.revcompl                                       ------------------------------------------------------------ 61762
chimp54546-135457.revcompl.Pan.troglodytes.clone.rp43-41g5.GenBank:AC099533.36      ------------------------------------------------------------ 70107
human.FAM30B-LOC105372935-GGT2.NCBI.                                                ggccagtagtgtctttgaggagttcaccacttagattactgagtaattgtggtttccaca 102193
human.FAM230E-LOC105377182-GGT3P.NCBI.                                              ------------------------------------------------------------ 57310
human.FAM230J-LOC105372942-GGTLC5P.NCBI.rev.compl                                   ------------------------------------------------------------ 46364
human.FAM230A.ncRNA.18487127.18500594-GGTLC3.18516335.18518165.rev.compl.REF        ------------------------------------------------------------ 31039

chimp.LOC112206744-LOC107973052-GGT2.revcompl                                       ------------------------------------------------------------ 61762
chimp54546-135457.revcompl.Pan.troglodytes.clone.rp43-41g5.GenBank:AC099533.36      ------------------------------------------------------------ 70107
human.FAM30B-LOC105372935-GGT2.NCBI.                                                tttgaaaacaactccttttataattattcactgctttttgtcagtgaaatagacatcttg 102253
human.FAM230E-LOC105377182-GGT3P.NCBI.                                              ------------------------------------------------------------ 57310
human.FAM230J-LOC105372942-GGTLC5P.NCBI.rev.compl                                   ------------------------------------------------------------ 46364
human.FAM230A.ncRNA.18487127.18500594-GGTLC3.18516335.18518165.rev.compl.REF        ------------------------------------------------------------ 31039

chimp.LOC112206744-LOC107973052-GGT2.revcompl                                       ------------------------------------------------------------ 61762
chimp54546-135457.revcompl.Pan.troglodytes.clone.rp43-41g5.GenBank:AC099533.36      ------------------------------------------------------------ 70107
human.FAM30B-LOC105372935-GGT2.NCBI.                                                cctcctgaagtagcttcatcacagagtgtcatgaagacagacagtcaggctgaaaaggac 102313
human.FAM230E-LOC105377182-GGT3P.NCBI.                                              ------------------------------------------------------------ 57310
human.FAM230J-LOC105372942-GGTLC5P.NCBI.rev.compl                                   ------------------------------------------------------------ 46364
human.FAM230A.ncRNA.18487127.18500594-GGTLC3.18516335.18518165.rev.compl.REF        ------------------------------------------------------------ 31039

chimp.LOC112206744-LOC107973052-GGT2.revcompl                                       ------------------------------------------------------------ 61762
chimp54546-135457.revcompl.Pan.troglodytes.clone.rp43-41g5.GenBank:AC099533.36      ------------------------------------------------------------ 70107
human.FAM30B-LOC105372935-GGT2.NCBI.                                                agttctttgtggactctacccttcccttcaaggagtatgtcatatgtcacaaaagaaatt 102373
human.FAM230E-LOC105377182-GGT3P.NCBI.                                              ------------------------------------------------------------ 57310
human.FAM230J-LOC105372942-GGTLC5P.NCBI.rev.compl                                   ------------------------------------------------------------ 46364
human.FAM230A.ncRNA.18487127.18500594-GGTLC3.18516335.18518165.rev.compl.REF        ------------------------------------------------------------ 31039

chimp.LOC112206744-LOC107973052-GGT2.revcompl                                       ------------------------------------------------------------ 61762
chimp54546-135457.revcompl.Pan.troglodytes.clone.rp43-41g5.GenBank:AC099533.36      ------------------------------------------------------------ 70107
human.FAM30B-LOC105372935-GGT2.NCBI.                                                gccttacactggttcatgtttgcagttactgttgtacattgcatagatgtacacacgaat 102433
human.FAM230E-LOC105377182-GGT3P.NCBI.                                              ------------------------------------------------------------ 57310
```

```
human.FAM230J-LOC105372942-GGTLC5P.NCBI.rev.compl                              ------------------------------------------------------------ 46364
human.FAM230A.ncRNA.18487127.18500594-GGTLC3.18516335.18518165.rev.compl.REF   ------------------------------------------------------------ 31039

chimp.LOC112206744-LOC107973052-GGT2.revcompl                                  ------------------------------------------------------------ 61762
chimp54546-135457.revcompl.Pan.troglodytes.clone.rp43-41g5.GenBank:AC099533.36 ---------------TTTTATATAAGAATACAGAGAGGTGGAAAATAATGAAAAATATGT 70152
human.FAM30B-LOC105372935-GGT2.NCBI.                                           ttaaatgtgatgtctttgtatatatctgtataatgttgagattacttacgaaatatgtct 102493
human.FAM230E-LOC105377182-GGT3P.NCBI.                                         ------------------------------------------------------------ 57310
human.FAM230J-LOC105372942-GGTLC5P.NCBI.rev.compl                              ------------------------------------------------------------ 46364
human.FAM230A.ncRNA.18487127.18500594-GGTLC3.18516335.18518165.rev.compl.REF   ------------------------------------------------------------ 31039

chimp.LOC112206744-LOC107973052-GGT2.revcompl                                  ------------------------------------------------------------ 61762
chimp54546-135457.revcompl.Pan.troglodytes.clone.rp43-41g5.GenBank:AC099533.36 CA---------------------------------------------------------- 70154
human.FAM30B-LOC105372935-GGT2.NCBI.                                           gagtgacacttttcactctcgtacagccaaaataatgtatatatggaaagtgacagacaa 102553
human.FAM230E-LOC105377182-GGT3P.NCBI.                                         ------------------------------------------------------------ 57310
human.FAM230J-LOC105372942-GGTLC5P.NCBI.rev.compl                              ------------------------------------------------------------ 46364
human.FAM230A.ncRNA.18487127.18500594-GGTLC3.18516335.18518165.rev.compl.REF   ------------------------------------------------------------ 31039

chimp.LOC112206744-LOC107973052-GGT2.revcompl                                  ------------------------------------------------------------ 61762
chimp54546-135457.revcompl.Pan.troglodytes.clone.rp43-41g5.GenBank:AC099533.36 ------------------------------------------------------------ 70154
human.FAM30B-LOC105372935-GGT2.NCBI.                                           attctctaatctctttggtatctataacttattagaatcctctggatgagggttagaaga 102613
human.FAM230E-LOC105377182-GGT3P.NCBI.                                         ------------------------------------------------------------ 57310
human.FAM230J-LOC105372942-GGTLC5P.NCBI.rev.compl                              ------------------------------------------------------------ 46364
human.FAM230A.ncRNA.18487127.18500594-GGTLC3.18516335.18518165.rev.compl.REF   ------------------------------------------------------------ 31039

chimp.LOC112206744-LOC107973052-GGT2.revcompl                                  ------------------------------------------------------------ 61762
chimp54546-135457.revcompl.Pan.troglodytes.clone.rp43-41g5.GenBank:AC099533.36 ------------------------------------------------------------ 70154
human.FAM30B-LOC105372935-GGT2.NCBI.                                           gactttttccaaacttctacatgtagaagtatcataaatgtgctacacatttatgtttgt 102673
human.FAM230E-LOC105377182-GGT3P.NCBI.                                         ------------------------------------------------------------ 57310
human.FAM230J-LOC105372942-GGTLC5P.NCBI.rev.compl                              ------------------------------------------------------------ 46364
human.FAM230A.ncRNA.18487127.18500594-GGTLC3.18516335.18518165.rev.compl.REF   ------------------------------------------------------------ 31039

chimp.LOC112206744-LOC107973052-GGT2.revcompl                                  ------------------------------------------------------------ 61762
chimp54546-135457.revcompl.Pan.troglodytes.clone.rp43-41g5.GenBank:AC099533.36 ------------------------------------------------------------ 70154
human.FAM30B-LOC105372935-GGT2.NCBI.                                           ggatttaattaaagtattttaatatggttttcagtgctaaaattggagtcagatacttct 102733
human.FAM230E-LOC105377182-GGT3P.NCBI.                                         ------------------------------------------------------------ 57310
human.FAM230J-LOC105372942-GGTLC5P.NCBI.rev.compl                              ------------------------------------------------------------ 46364
human.FAM230A.ncRNA.18487127.18500594-GGTLC3.18516335.18518165.rev.compl.REF   ------------------------------------------------------------ 31039

chimp.LOC112206744-LOC107973052-GGT2.revcompl                                  ------------------------------------------------------------ 61762
chimp54546-135457.revcompl.Pan.troglodytes.clone.rp43-41g5.GenBank:AC099533.36 ------------------------------------------------------------ 70154
human.FAM30B-LOC105372935-GGT2.NCBI.                                           tggttttaagctgtctacctaattgctgtctcccagcagatcggtggcatgcccagtggc 102793
human.FAM230E-LOC105377182-GGT3P.NCBI.                                         ------------------------------------------------------------ 57310
human.FAM230J-LOC105372942-GGTLC5P.NCBI.rev.compl                              ------------------------------------------------------------ 46364
human.FAM230A.ncRNA.18487127.18500594-GGTLC3.18516335.18518165.rev.compl.REF   ------------------------------------------------------------ 31039

chimp.LOC112206744-LOC107973052-GGT2.revcompl                                  ------------------------------------------------------------ 61762
chimp54546-135457.revcompl.Pan.troglodytes.clone.rp43-41g5.GenBank:AC099533.36 ------------------------------------------------------------ 70154
human.FAM30B-LOC105372935-GGT2.NCBI.                                           tttgggggcaaggatagaaatgtcatcaggaaatagctgaattcattgtgaaacatgaat 102853
human.FAM230E-LOC105377182-GGT3P.NCBI.                                         ------------------------------------------------------------ 57310
human.FAM230J-LOC105372942-GGTLC5P.NCBI.rev.compl                              ------------------------------------------------------------ 46364
human.FAM230A.ncRNA.18487127.18500594-GGTLC3.18516335.18518165.rev.compl.REF   ------------------------------------------------------------ 31039

chimp.LOC112206744-LOC107973052-GGT2.revcompl                                  ------------------------------------------------------------ 61762
chimp54546-135457.revcompl.Pan.troglodytes.clone.rp43-41g5.GenBank:AC099533.36 ------------------------------------------------------------ 70154
human.FAM30B-LOC105372935-GGT2.NCBI.                                           tcagtcatggtgataattggaaactcctttcaggtttttgcaagtagattttgtaatgtt 102913
human.FAM230E-LOC105377182-GGT3P.NCBI.                                         ------------------------------------------------------------ 57310
human.FAM230J-LOC105372942-GGTLC5P.NCBI.rev.compl                              ------------------------------------------------------------ 46364
human.FAM230A.ncRNA.18487127.18500594-GGTLC3.18516335.18518165.rev.compl.REF   ------------------------------------------------------------ 31039

chimp.LOC112206744-LOC107973052-GGT2.revcompl                                  ------------------------------------------------------------ 61762
chimp54546-135457.revcompl.Pan.troglodytes.clone.rp43-41g5.GenBank:AC099533.36 ------------------------------------------------------------ 70154
human.FAM30B-LOC105372935-GGT2.NCBI.                                           tgtgtatgcagccttgctgttgagtcagtccaaggggttttacttaggacaagttgtacc 102973
human.FAM230E-LOC105377182-GGT3P.NCBI.                                         ------------------------------------------------------------ 57310
human.FAM230J-LOC105372942-GGTLC5P.NCBI.rev.compl                              ------------------------------------------------------------ 46364
human.FAM230A.ncRNA.18487127.18500594-GGTLC3.18516335.18518165.rev.compl.REF   ------------------------------------------------------------ 31039

chimp.LOC112206744-LOC107973052-GGT2.revcompl                                  ------------------------------------------------------------ 61762
chimp54546-135457.revcompl.Pan.troglodytes.clone.rp43-41g5.GenBank:AC099533.36 ------------------------------------------------------------ 70154
human.FAM30B-LOC105372935-GGT2.NCBI.                                           ttgccctctctccagctctgctcccacattttcacatacctagctatttctacctcattg 103033
human.FAM230E-LOC105377182-GGT3P.NCBI.                                         ------------------------------------------------------------ 57310
human.FAM230J-LOC105372942-GGTLC5P.NCBI.rev.compl                              ------------------------------------------------------------ 46364
human.FAM230A.ncRNA.18487127.18500594-GGTLC3.18516335.18518165.rev.compl.REF   ------------------------------------------------------------ 31039

chimp.LOC112206744-LOC107973052-GGT2.revcompl                                  ------------------------------------------------------------ 61762
chimp54546-135457.revcompl.Pan.troglodytes.clone.rp43-41g5.GenBank:AC099533.36 ------------------------------------------------------------ 70154
human.FAM30B-LOC105372935-GGT2.NCBI.                                           ggtaagtcatttaccactctgtgcctcagtttactctgtagtttaccattagactgtgag 103093
human.FAM230E-LOC105377182-GGT3P.NCBI.                                         ------------------------------------------------------------ 57310
human.FAM230J-LOC105372942-GGTLC5P.NCBI.rev.compl                              ------------------------------------------------------------ 46364
human.FAM230A.ncRNA.18487127.18500594-GGTLC3.18516335.18518165.rev.compl.REF   ------------------------------------------------------------ 31039

chimp.LOC112206744-LOC107973052-GGT2.revcompl                                  ------------------------------------------------------------ 61762
chimp54546-135457.revcompl.Pan.troglodytes.clone.rp43-41g5.GenBank:AC099533.36 ------------------------------------------------------------ 70154
human.FAM30B-LOC105372935-GGT2.NCBI.                                           ctccctgagggactttgtcataatcactgttacatcccagtgcctcacaccatgcctggc 103153
human.FAM230E-LOC105377182-GGT3P.NCBI.                                         ------------------------------------------------------------ 57310
human.FAM230J-LOC105372942-GGTLC5P.NCBI.rev.compl                              ------------------------------------------------------------ 46364
human.FAM230A.ncRNA.18487127.18500594-GGTLC3.18516335.18518165.rev.compl.REF   ------------------------------------------------------------ 31039

chimp.LOC112206744-LOC107973052-GGT2.revcompl                                  ------------------------------------------------------------ 61762
chimp54546-135457.revcompl.Pan.troglodytes.clone.rp43-41g5.GenBank:AC099533.36 ------------------------------------------------------------ 70154
human.FAM30B-LOC105372935-GGT2.NCBI.                                           ccttaagaagtgctcaataaatgtctgaacaaaaaaaaaaaaaaaaaaaaaaaaaatcag 103213
human.FAM230E-LOC105377182-GGT3P.NCBI.                                         ------------------------------------------------------------ 57310
human.FAM230J-LOC105372942-GGTLC5P.NCBI.rev.compl                              ------------------------------------------------------------ 46364
human.FAM230A.ncRNA.18487127.18500594-GGTLC3.18516335.18518165.rev.compl.REF   ------------------------------------------------------------ 31039

chimp.LOC112206744-LOC107973052-GGT2.revcompl                                  ------------------------------------------------------------ 61762
chimp54546-135457.revcompl.Pan.troglodytes.clone.rp43-41g5.GenBank:AC099533.36 ------------------------------------------------------------ 70154
human.FAM30B-LOC105372935-GGT2.NCBI.                                           gtctaaagatgagccatccaagaaactgctcttgctcccagaggttttaaagaaagtcat 103273
human.FAM230E-LOC105377182-GGT3P.NCBI.                                         ------------------------------------------------------------ 57310
human.FAM230J-LOC105372942-GGTLC5P.NCBI.rev.compl                              ------------------------------------------------------------ 46364
human.FAM230A.ncRNA.18487127.18500594-GGTLC3.18516335.18518165.rev.compl.REF   ------------------------------------------------------------ 31039

chimp.LOC112206744-LOC107973052-GGT2.revcompl                                  ------------------------------------------------------------ 61762
chimp54546-135457.revcompl.Pan.troglodytes.clone.rp43-41g5.GenBank:AC099533.36 ------------------------------------------------------------ 70154
human.FAM30B-LOC105372935-GGT2.NCBI.                                           tcccatgaacgtgagaagtaacccaacactttgaaatgataatctttttacagcaataac 103333
human.FAM230E-LOC105377182-GGT3P.NCBI.                                         ------------------------------------------------------------ 57310
human.FAM230J-LOC105372942-GGTLC5P.NCBI.rev.compl                              ------------------------------------------------------------ 46364
human.FAM230A.ncRNA.18487127.18500594-GGTLC3.18516335.18518165.rev.compl.REF   ------------------------------------------------------------ 31039

chimp.LOC112206744-LOC107973052-GGT2.revcompl                                  ------------------------------------------------------------ 61762
chimp54546-135457.revcompl.Pan.troglodytes.clone.rp43-41g5.GenBank:AC099533.36 ------TGCATGCACTAACCAAGAAAGCTGTATCGCTTT--------------------- 70187
human.FAM30B-LOC105372935-GGT2.NCBI.                                           tgttgctgcagggcttgagaaaaaaagttctcttcctttgggttaaagtcttaaatgctg 103393
human.FAM230E-LOC105377182-GGT3P.NCBI.                                         ------------------------------------------------------------ 57310
human.FAM230J-LOC105372942-GGTLC5P.NCBI.rev.compl                              ------------------------------------------------------------ 46364
human.FAM230A.ncRNA.18487127.18500594-GGTLC3.18516335.18518165.rev.compl.REF   ------------------------------------------------------------ 31039

chimp.LOC112206744-LOC107973052-GGT2.revcompl                                  ------------------------------------------------------------ 61762
chimp54546-135457.revcompl.Pan.troglodytes.clone.rp43-41g5.GenBank:AC099533.36 ------------------------------------------------------------ 70187
human.FAM30B-LOC105372935-GGT2.NCBI.                                           agcccgtagccagcaaccttaaagactactcgggtccagttcattctatctggaggagtc 103453
human.FAM230E-LOC105377182-GGT3P.NCBI.                                         ------------------------------------------------------------ 57310
human.FAM230J-LOC105372942-GGTLC5P.NCBI.rev.compl                              ------------------------------------------------------------ 46364
human.FAM230A.ncRNA.18487127.18500594-GGTLC3.18516335.18518165.rev.compl.REF   ------------------------------------------------------------ 31039

chimp.LOC112206744-LOC107973052-GGT2.revcompl                                  ------------------------------------------------------------ 61762
chimp54546-135457.revcompl.Pan.troglodytes.clone.rp43-41g5.GenBank:AC099533.36 ------------------------------------------------------------ 70187
human.FAM30B-LOC105372935-GGT2.NCBI.                                           acttgggaattgaaaacacaggaaaactttttttctggtccatgaacaaacaagaacgct 103513
human.FAM230E-LOC105377182-GGT3P.NCBI.                                         ------------------------------------------------------------ 57310
human.FAM230J-LOC105372942-GGTLC5P.NCBI.rev.compl                              ------------------------------------------------------------ 46364
```

```
human.FAM230A.ncRNA.18487127.18500594-GGTLC3.18516335.18518165.rev.compl.REF        ------------------------------------------------------------ 31039

chimp.LOC112206744-LOC107973052-GGT2.revcompl                                       ------------------------------------------------------------ 61762
chimp54546-135457.revcompl.Pan.troglodytes.clone.rp43-41g5.GenBank:AC099533.36      ------------------------------------------------------------ 70187
human.FAM30B-LOC105372935-GGT2.NCBI.                                                taactgaattagacacaaaactaagtatttatggttgtcatcttaacaggtttcaaacag 103573
human.FAM230E-LOC105377182-GGT3P.NCBI.                                              ------------------------------------------------------------ 57310
human.FAM230J-LOC105372942-GGTLC5P.NCBI.rev.compl                                   ------------------------------------------------------------ 46364
human.FAM230A.ncRNA.18487127.18500594-GGTLC3.18516335.18518165.rev.compl.REF        ------------------------------------------------------------ 31039

chimp.LOC112206744-LOC107973052-GGT2.revcompl                                       ------------------------------------------------------------ 61762
chimp54546-135457.revcompl.Pan.troglodytes.clone.rp43-41g5.GenBank:AC099533.36      ------------------------------------------------------------ 70187
human.FAM30B-LOC105372935-GGT2.NCBI.                                                gcttcattttaatttaacataatttcaaaattatagcaaaatagtcaataaaatacgaac 103633
human.FAM230E-LOC105377182-GGT3P.NCBI.                                              ------------------------------------------------------------ 57310
human.FAM230J-LOC105372942-GGTLC5P.NCBI.rev.compl                                   ------------------------------------------------------------ 46364
human.FAM230A.ncRNA.18487127.18500594-GGTLC3.18516335.18518165.rev.compl.REF        ------------------------------------------------------------ 31039

chimp.LOC112206744-LOC107973052-GGT2.revcompl                                       ------------------------------------------------------------ 61762
chimp54546-135457.revcompl.Pan.troglodytes.clone.rp43-41g5.GenBank:AC099533.36      ------------------------------------------------------------ 70187
human.FAM30B-LOC105372935-GGT2.NCBI.                                                gtgcttaatttgatgaatttatgtgtttttttgaagaaaatatgcagaataaacaagtcaa 103693
human.FAM230E-LOC105377182-GGT3P.NCBI.                                              ------------------------------------------------------------ 57310
human.FAM230J-LOC105372942-GGTLC5P.NCBI.rev.compl                                   ------------------------------------------------------------ 46364
human.FAM230A.ncRNA.18487127.18500594-GGTLC3.18516335.18518165.rev.compl.REF        ------------------------------------------------------------ 31039

chimp.LOC112206744-LOC107973052-GGT2.revcompl                                       ------------------------------------------------------------ 61762
chimp54546-135457.revcompl.Pan.troglodytes.clone.rp43-41g5.GenBank:AC099533.36      ------------------------------------------------------------ 70187
human.FAM30B-LOC105372935-GGT2.NCBI.                                                ttattctcactaaatacattaaaatttaagggtaatatttttattatgacttgctctcct 103753
human.FAM230E-LOC105377182-GGT3P.NCBI.                                              ------------------------------------------------------------ 57310
human.FAM230J-LOC105372942-GGTLC5P.NCBI.rev.compl                                   ------------------------------------------------------------ 46364
human.FAM230A.ncRNA.18487127.18500594-GGTLC3.18516335.18518165.rev.compl.REF        ------------------------------------------------------------ 31039

chimp.LOC112206744-LOC107973052-GGT2.revcompl                                       ------------------------------------------------------------ 61762
chimp54546-135457.revcompl.Pan.troglodytes.clone.rp43-41g5.GenBank:AC099533.36      ------------------------------------------------------------ 70187
human.FAM30B-LOC105372935-GGT2.NCBI.                                                gatatagcaaaatttattgttatatagttatggaaggaggctcactactccccaatgatt 103813
human.FAM230E-LOC105377182-GGT3P.NCBI.                                              ------------------------------------------------------------ 57310
human.FAM230J-LOC105372942-GGTLC5P.NCBI.rev.compl                                   ------------------------------------------------------------ 46364
human.FAM230A.ncRNA.18487127.18500594-GGTLC3.18516335.18518165.rev.compl.REF        ------------------------------------------------------------ 31039

chimp.LOC112206744-LOC107973052-GGT2.revcompl                                       ------------------------------------------------------------ 61762
chimp54546-135457.revcompl.Pan.troglodytes.clone.rp43-41g5.GenBank:AC099533.36      ------------------------------------------------------------ 70187
human.FAM30B-LOC105372935-GGT2.NCBI.                                                tcacaaattacagcttcagccagctataaaataaccaaatcagttctgatatagccataa 103873
human.FAM230E-LOC105377182-GGT3P.NCBI.                                              ------------------------------------------------------------ 57310
human.FAM230J-LOC105372942-GGTLC5P.NCBI.rev.compl                                   ------------------------------------------------------------ 46364
human.FAM230A.ncRNA.18487127.18500594-GGTLC3.18516335.18518165.rev.compl.REF        ------------------------------------------------------------ 31039

chimp.LOC112206744-LOC107973052-GGT2.revcompl                                       ------------------------------------------------------------ 61762
chimp54546-135457.revcompl.Pan.troglodytes.clone.rp43-41g5.GenBank:AC099533.36      ------------------------------------------------------------ 70187
human.FAM30B-LOC105372935-GGT2.NCBI.                                                aattaagcctgaaaaaatagtgaagtaaatcatccctttaaaatcaagttgttgaacaat 103933
human.FAM230E-LOC105377182-GGT3P.NCBI.                                              ------------------------------------------------------------ 57310
human.FAM230J-LOC105372942-GGTLC5P.NCBI.rev.compl                                   ------------------------------------------------------------ 46364
human.FAM230A.ncRNA.18487127.18500594-GGTLC3.18516335.18518165.rev.compl.REF        ------------------------------------------------------------ 31039

chimp.LOC112206744-LOC107973052-GGT2.revcompl                                       ------------------------------------------------------------ 61762
chimp54546-135457.revcompl.Pan.troglodytes.clone.rp43-41g5.GenBank:AC099533.36      ------------------------------------------------------------ 70187
human.FAM30B-LOC105372935-GGT2.NCBI.                                                atgttctgcatgattccatttatgtcagaaaaaatatatttgtgtaagtatatacacaga 103993
human.FAM230E-LOC105377182-GGT3P.NCBI.                                              ------------------------------------------------------------ 57310
human.FAM230J-LOC105372942-GGTLC5P.NCBI.rev.compl                                   ------------------------------------------------------------ 46364
human.FAM230A.ncRNA.18487127.18500594-GGTLC3.18516335.18518165.rev.compl.REF        ------------------------------------------------------------ 31039

chimp.LOC112206744-LOC107973052-GGT2.revcompl                                       ------------------------------------------------------------ 61762
chimp54546-135457.revcompl.Pan.troglodytes.clone.rp43-41g5.GenBank:AC099533.36      ------------------------------------------------------------ 70187
human.FAM30B-LOC105372935-GGT2.NCBI.                                                aaagggtctgggagaatatataccaaactataaagaatctatatcaaagtcaataagggg 104053
human.FAM230E-LOC105377182-GGT3P.NCBI.                                              ------------------------------------------------------------ 57310
human.FAM230J-LOC105372942-GGTLC5P.NCBI.rev.compl                                   ------------------------------------------------------------ 46364
human.FAM230A.ncRNA.18487127.18500594-GGTLC3.18516335.18518165.rev.compl.REF        ------------------------------------------------------------ 31039

chimp.LOC112206744-LOC107973052-GGT2.revcompl                                       ------------------------------------------------------------ 61762
chimp54546-135457.revcompl.Pan.troglodytes.clone.rp43-41g5.GenBank:AC099533.36      ----------------------------------------TTTTTTTTTTTTTTTTGGAG 70207
human.FAM30B-LOC105372935-GGT2.NCBI.                                                catagacaaggaggggaactttcacatttactttccacattcttttttttttttttttg 104113
human.FAM230E-LOC105377182-GGT3P.NCBI.                                              ------------------------------------------------------------ 57310
human.FAM230J-LOC105372942-GGTLC5P.NCBI.rev.compl                                   ------------------------------------------------------------ 46364
human.FAM230A.ncRNA.18487127.18500594-GGTLC3.18516335.18518165.rev.compl.REF        ------------------------------------------------------------ 31039

chimp.LOC112206744-LOC107973052-GGT2.revcompl                                       ------------------------------------------------------------ 61762
chimp54546-135457.revcompl.Pan.troglodytes.clone.rp43-41g5.GenBank:AC099533.36      ATAGAGCCTCACTCTGTCTCCCAGGCTGGAGTGCAATGATGTGATCTTGGGTTACAGCAA 70267
human.FAM30B-LOC105372935-GGT2.NCBI.                                                acggagtctcactctgttgcccaggctggagtgcagtggcgcaatcttggctcactgcaa 104173
human.FAM230E-LOC105377182-GGT3P.NCBI.                                              ------------------------------------------------------------ 57310
human.FAM230J-LOC105372942-GGTLC5P.NCBI.rev.compl                                   ------------------------------------------------------------ 46364
human.FAM230A.ncRNA.18487127.18500594-GGTLC3.18516335.18518165.rev.compl.REF        ------------------------------------------------------------ 31039

chimp.LOC112206744-LOC107973052-GGT2.revcompl                                       ------------------------------------------------------------ 61762
chimp54546-135457.revcompl.Pan.troglodytes.clone.rp43-41g5.GenBank:AC099533.36      TCTCTCCCTTCTAGGCTCAAGCGATTCTCCCACCTCAGCATCCCAAGTAGCTGGGACTAC 70327
human.FAM30B-LOC105372935-GGT2.NCBI.                                                gctccgcctcccgggttcacaccattctcctgcctcagcctcccgagtagctgggactac 104233
human.FAM230E-LOC105377182-GGT3P.NCBI.                                              ------------------------------------------------------------ 57310
human.FAM230J-LOC105372942-GGTLC5P.NCBI.rev.compl                                   ------------------------------------------------------------ 46364
human.FAM230A.ncRNA.18487127.18500594-GGTLC3.18516335.18518165.rev.compl.REF        ------------------------------------------------------------ 31039

chimp.LOC112206744-LOC107973052-GGT2.revcompl                                       ------------------------------------------------------------ 61762
chimp54546-135457.revcompl.Pan.troglodytes.clone.rp43-41g5.GenBank:AC099533.36      AAGTGTGCCAACTTAGAATTATATTAGCCACACCCAGCTAATTTTTGTATTTTTTGTAGA 70387
human.FAM30B-LOC105372935-GGT2.NCBI.                                                aggcacccgc---------------caccacgcccagctaatttttttatattt------ 104272
human.FAM230E-LOC105377182-GGT3P.NCBI.                                              ------------------------------------------------------------ 57310
human.FAM230J-LOC105372942-GGTLC5P.NCBI.rev.compl                                   ------------------------------------------------------------ 46364
human.FAM230A.ncRNA.18487127.18500594-GGTLC3.18516335.18518165.rev.compl.REF        ------------------------------------------------------------ 31039

chimp.LOC112206744-LOC107973052-GGT2.revcompl                                       ------------------------------------------------------------ 61762
chimp54546-135457.revcompl.Pan.troglodytes.clone.rp43-41g5.GenBank:AC099533.36      GGCAGGGTCTCGCCATGTTGCCCAGGCTGGTCTTGAACTCCTGGGCTTCAGTGATCCACC 70447
human.FAM30B-LOC105372935-GGT2.NCBI.                                                ---ggggtttcactgcgttagccaggatggtctcaatctcctgacctt--gtgatctgcc 104327
human.FAM230E-LOC105377182-GGT3P.NCBI.                                              ------------------------------------------------------------ 57310
human.FAM230J-LOC105372942-GGTLC5P.NCBI.rev.compl                                   ------------------------------------------------------------ 46364
human.FAM230A.ncRNA.18487127.18500594-GGTLC3.18516335.18518165.rev.compl.REF        ------------------------------------------------------------ 31039

chimp.LOC112206744-LOC107973052-GGT2.revcompl                                       ------------------------------------------------------------ 61762
chimp54546-135457.revcompl.Pan.troglodytes.clone.rp43-41g5.GenBank:AC099533.36      CACCTCAACCTCC-------AACAAAGTGCCAAGATTACAGCCATGAGCCACCATGCCCA 70500
human.FAM30B-LOC105372935-GGT2.NCBI.                                                cacctcacccacctcagcctcccaaagtgctgggattacaggcgtgactttctacattct 104387
human.FAM230E-LOC105377182-GGT3P.NCBI.                                              ------------------------------------------------------------ 57310
human.FAM230J-LOC105372942-GGTLC5P.NCBI.rev.compl                                   ------------------------------------------------------------ 46364
human.FAM230A.ncRNA.18487127.18500594-GGTLC3.18516335.18518165.rev.compl.REF        ------------------------------------------------------------ 31039

chimp.LOC112206744-LOC107973052-GGT2.revcompl                                       ------------------------------------------------------------ 61762
chimp54546-135457.revcompl.Pan.troglodytes.clone.rp43-41g5.GenBank:AC099533.36      GCATAACTATTTTTAATGAAGTAGACTTTAAGAAGAAAAGTATTATTAGAGGTAAGAGAC 70560
human.FAM30B-LOC105372935-GGT2.NCBI.                                                tgtaacagaattatagtcaccgttacctccatcaaaaattaaaattcaa----------- 104437
human.FAM230E-LOC105377182-GGT3P.NCBI.                                              ------------------------------------------------------------ 57310
human.FAM230J-LOC105372942-GGTLC5P.NCBI.rev.compl                                   ------------------------------------------------------------ 46364
human.FAM230A.ncRNA.18487127.18500594-GGTLC3.18516335.18518165.rev.compl.REF        ------------------------------------------------------------ 31039

chimp.LOC112206744-LOC107973052-GGT2.revcompl                                       ------------------------------------------------------------ 61762
chimp54546-135457.revcompl.Pan.troglodytes.clone.rp43-41g5.GenBank:AC099533.36      ACATCATGGAAAAGAAGAATTTACTAGGAGCCAGGCGCGGTGGCTCATGCCTGTAATTCC 70620
human.FAM30B-LOC105372935-GGT2.NCBI.                                                ----------------------aaattaggccaggcacggtggctcacgcctgtaatccc 104475
human.FAM230E-LOC105377182-GGT3P.NCBI.                                              ------------------------------------------------------------ 57310
human.FAM230J-LOC105372942-GGTLC5P.NCBI.rev.compl                                   ------------------------------------------------------------ 46364
human.FAM230A.ncRNA.18487127.18500594-GGTLC3.18516335.18518165.rev.compl.REF        ------------------------------------------------------------ 31039

chimp.LOC112206744-LOC107973052-GGT2.revcompl                                       ------------------------------------------------------------ 61762
chimp54546-135457.revcompl.Pan.troglodytes.clone.rp43-41g5.GenBank:AC099533.36      AGCACTTTGTGAGGCCAAGGCGGCGGATCACCTGAGGTTGGGAGTTCAACACCAGCCTGA 70680
human.FAM30B-LOC105372935-GGT2.NCBI.                                                agcactttgggagtctggagcagcagatcacctgaggtcaggagttggagagcagcctga 104535
human.FAM230E-LOC105377182-GGT3P.NCBI.                                              ------------------------------------------------------------ 57310
human.FAM230J-LOC105372942-GGTLC5P.NCBI.rev.compl                                   ------------------------------------------------------------ 46364
human.FAM230A.ncRNA.18487127.18500594-GGTLC3.18516335.18518165.rev.compl.REF        ------------------------------------------------------------ 31039
```

```
chimp.LOC112206744-LOC107973052-GGT2.revcompl                            ------------------------------------------------------------ 61762
chimp54546-135457.revcompl.Pan.troglodytes.clone.rp43-41g5.GenBank:AC099533.36    ----CCAACATGGAGAAGCCCTGTCTCTACTAAAAATACAAAAACTAGCCAAGCATGGTG 70736
human.FAM30B-LOC105372935-GGT2.NCBI.                                     ctggccaacatggtgaaaccccatctctactaaaaatacaaaaattagccgggcgtggtg 104595
human.FAM230E-LOC105377182-GGT3P.NCBI.                                   ------------------------------------------------------------ 57310
human.FAM230J-LOC105372942-GGTLC5P.NCBI.rev.compl                        ------------------------------------------------------------ 46364
human.FAM230A.ncRNA.18487127.18500594-GGTLC3.18516335.18518165.rev.compl.REF  ------------------------------------------------------------ 31039

chimp.LOC112206744-LOC107973052-GGT2.revcompl                            ------------------------------------------------------------ 61762
chimp54546-135457.revcompl.Pan.troglodytes.clone.rp43-41g5.GenBank:AC099533.36    GCATATGCCTGTAATCCCAGCTACTCAGGAGGCTGAGGGAAGAGAATTGCTTGGACCCAG 70796
human.FAM30B-LOC105372935-GGT2.NCBI.                                     gagggcacctgtaatcccagctacttgggaggctaaggcaggagaatcacttaaacccgg 104655
human.FAM230E-LOC105377182-GGT3P.NCBI.                                   ------------------------------------------------------------ 57310
human.FAM230J-LOC105372942-GGTLC5P.NCBI.rev.compl                        ------------------------------------------------------------ 46364
human.FAM230A.ncRNA.18487127.18500594-GGTLC3.18516335.18518165.rev.compl.REF  ------------------------------------------------------------ 31039

chimp.LOC112206744-LOC107973052-GGT2.revcompl                            ------------------------------------------------------------ 61762
chimp54546-135457.revcompl.Pan.troglodytes.clone.rp43-41g5.GenBank:AC099533.36    GAAGTGGAGATTGAGGTGAGCTGAGATTGTGCCATTGCATTCCAGCCTGGGCAACAAGAG 70856
human.FAM30B-LOC105372935-GGT2.NCBI.                                     gaggcggaggttgcagtgagccaagattgtgccactgcactccggcccgggtaac-agag 104714
human.FAM230E-LOC105377182-GGT3P.NCBI.                                   ------------------------------------------------------------ 57310
human.FAM230J-LOC105372942-GGTLC5P.NCBI.rev.compl                        ------------------------------------------------------------ 46364
human.FAM230A.ncRNA.18487127.18500594-GGTLC3.18516335.18518165.rev.compl.REF  ------------------------------------------------------------ 31039

chimp.LOC112206744-LOC107973052-GGT2.revcompl                            ------------------------------------------------------------ 61762
chimp54546-135457.revcompl.Pan.troglodytes.clone.rp43-41g5.GenBank:AC099533.36    CAAAACTCTGTCTCAAAAAAAAAAAAAAAAAAAAAAAAAGGAA----------------- 70899
human.FAM30B-LOC105372935-GGT2.NCBI.                                     cgagactccatctcaaaaatgaatgaattaattaattaattaaagcaaacaaatataagc 104774
human.FAM230E-LOC105377182-GGT3P.NCBI.                                   ------------------------------------------------------------ 57310
human.FAM230J-LOC105372942-GGTLC5P.NCBI.rev.compl                        ------------------------------------------------------------ 46364
human.FAM230A.ncRNA.18487127.18500594-GGTLC3.18516335.18518165.rev.compl.REF  ------------------------------------------------------------ 31039

chimp.LOC112206744-LOC107973052-GGT2.revcompl                            ------------------------------------------------------------ 61762
chimp54546-135457.revcompl.Pan.troglodytes.clone.rp43-41g5.GenBank:AC099533.36    ------------------------------------------------------------ 70899
human.FAM30B-LOC105372935-GGT2.NCBI.                                     tacctcctgaagtagaaaccagtatctcttcaccttacctttacataatactttacccta 104834
human.FAM230E-LOC105377182-GGT3P.NCBI.                                   ------------------------------------------------------------ 57310
human.FAM230J-LOC105372942-GGTLC5P.NCBI.rev.compl                        ------------------------------------------------------------ 46364
human.FAM230A.ncRNA.18487127.18500594-GGTLC3.18516335.18518165.rev.compl.REF  ------------------------------------------------------------ 31039

chimp.LOC112206744-LOC107973052-GGT2.revcompl                            ------------------------------------------------------------ 61762
chimp54546-135457.revcompl.Pan.troglodytes.clone.rp43-41g5.GenBank:AC099533.36    ------------------------------------------------------------ 70899
human.FAM30B-LOC105372935-GGT2.NCBI.                                     atgcattttatctgctgttctaaatcatgacttacttcaaatcaaatcttaagtgaaaat 104894
human.FAM230E-LOC105377182-GGT3P.NCBI.                                   ------------------------------------------------------------ 57310
human.FAM230J-LOC105372942-GGTLC5P.NCBI.rev.compl                        ------------------------------------------------------------ 46364
human.FAM230A.ncRNA.18487127.18500594-GGTLC3.18516335.18518165.rev.compl.REF  ------------------------------------------------------------ 31039

chimp.LOC112206744-LOC107973052-GGT2.revcompl                            ------------------------------------------------------------ 61762
chimp54546-135457.revcompl.Pan.troglodytes.clone.rp43-41g5.GenBank:AC099533.36    ------------------------------------------------------------ 70899
human.FAM30B-LOC105372935-GGT2.NCBI.                                     caagatttccttttggggcattatacaaaaaagactgtaaaatttcattctctgaaagt 104954
human.FAM230E-LOC105377182-GGT3P.NCBI.                                   ------------------------------------------------------------ 57310
human.FAM230J-LOC105372942-GGTLC5P.NCBI.rev.compl                        ------------------------------------------------------------ 46364
human.FAM230A.ncRNA.18487127.18500594-GGTLC3.18516335.18518165.rev.compl.REF  ------------------------------------------------------------ 31039

chimp.LOC112206744-LOC107973052-GGT2.revcompl                            ------------------------------------------------------------ 61762
chimp54546-135457.revcompl.Pan.troglodytes.clone.rp43-41g5.GenBank:AC099533.36    ------------------------------------------------------------ 70899
human.FAM30B-LOC105372935-GGT2.NCBI.                                     ctttttaaaaagggatagattgtatctggctgggatagggtaggtgtgatcggacccgga 105014
human.FAM230E-LOC105377182-GGT3P.NCBI.                                   ------------------------------------------------------------ 57310
human.FAM230J-LOC105372942-GGTLC5P.NCBI.rev.compl                        ------------------------------------------------------------ 46364
human.FAM230A.ncRNA.18487127.18500594-GGTLC3.18516335.18518165.rev.compl.REF  ------------------------------------------------------------ 31039

chimp.LOC112206744-LOC107973052-GGT2.revcompl                            ------------------------------------------------------------ 61762
chimp54546-135457.revcompl.Pan.troglodytes.clone.rp43-41g5.GenBank:AC099533.36    ----------------------------------------GTTACTAGCTAGTTTCAGTA 70919
human.FAM30B-LOC105372935-GGT2.NCBI.                                     ggaaatgacggctgctcaactgtgcatctcctttcctcaccctgctgaaaagtgccacct 105074
human.FAM230E-LOC105377182-GGT3P.NCBI.                                   ------------------------------------------------------------ 57310
human.FAM230J-LOC105372942-GGTLC5P.NCBI.rev.compl                        ------------------------------------------------------------ 46364
human.FAM230A.ncRNA.18487127.18500594-GGTLC3.18516335.18518165.rev.compl.REF  ------------------------------------------------------------ 31039

chimp.LOC112206744-LOC107973052-GGT2.revcompl                            ------------------------------------------------------------ 61762
chimp54546-135457.revcompl.Pan.troglodytes.clone.rp43-41g5.GenBank:AC099533.36    ATTCTTAACATCCAGGAAACTGGATGTGAAAGCTTTTCAGAGAAGCTAAACCAATAGGTT 70979
human.FAM30B-LOC105372935-GGT2.NCBI.                                     agcatggtcaatcaaggtcaccaatgcaaagcagtagtggcaaacaccaccc--aggtt 105132
human.FAM230E-LOC105377182-GGT3P.NCBI.                                   ------------------------------------------------------------ 57310
human.FAM230J-LOC105372942-GGTLC5P.NCBI.rev.compl                        ------------------------------------------------------------ 46364
human.FAM230A.ncRNA.18487127.18500594-GGTLC3.18516335.18518165.rev.compl.REF  ------------------------------------------------------------ 31039

chimp.LOC112206744-LOC107973052-GGT2.revcompl                            ------------------------------------------------------------ 61762
chimp54546-135457.revcompl.Pan.troglodytes.clone.rp43-41g5.GenBank:AC099533.36    ATACATAGAGAGAGATTTATTTAGGAATTGGCTTACATGATTGTGGGGACTAGCAAGTTT 71039
human.FAM30B-LOC105372935-GGT2.NCBI.                                     atccaaagcagcacatggggttgggaaactgatgaccttggagatttcaaaatcacctaa 105192
human.FAM230E-LOC105377182-GGT3P.NCBI.                                   ------------------------------------------------------------ 57310
human.FAM230J-LOC105372942-GGTLC5P.NCBI.rev.compl                        ------------------------------------------------------------ 46364
human.FAM230A.ncRNA.18487127.18500594-GGTLC3.18516335.18518165.rev.compl.REF  ------------------------------------------------------------ 31039

chimp.LOC112206744-LOC107973052-GGT2.revcompl                            ------------------------------------------------------------ 61762
chimp54546-135457.revcompl.Pan.troglodytes.clone.rp43-41g5.GenBank:AC099533.36    AAAATCTGTAGGGCAAGCCAGCCGGCTATAAATTCAGGTAAGAGTTGATCTCGAAGTCTT 71099
human.FAM30B-LOC105372935-GGT2.NCBI.                                     caa----------taaccccaccttccctaaccacagggctaag--caacctaatggatg 105240
human.FAM230E-LOC105377182-GGT3P.NCBI.                                   ------------------------------------------------------------ 57310
human.FAM230J-LOC105372942-GGTLC5P.NCBI.rev.compl                        ------------------------------------------------------------ 46364
human.FAM230A.ncRNA.18487127.18500594-GGTLC3.18516335.18518165.rev.compl.REF  ------------------------------------------------------------ 31039

chimp.LOC112206744-LOC107973052-GGT2.revcompl                            ------------------------------------------------------------ 61762
chimp54546-135457.revcompl.Pan.troglodytes.clone.rp43-41g5.GenBank:AC099533.36    GAACCTAAAATCTGTAGAGCAGTCAGCAGGCCAGAAACTCAGGCAGGGTTTG-------- 71151
human.FAM30B-LOC105372935-GGT2.NCBI.                                     gaacatatgtgtgctttggtcatccgcaacccagaccactagactaattttgctttagaa 105300
human.FAM230E-LOC105377182-GGT3P.NCBI.                                   ------------------------------------------------------------ 57310
human.FAM230J-LOC105372942-GGTLC5P.NCBI.rev.compl                        ------------------------------------------------------------ 46364
human.FAM230A.ncRNA.18487127.18500594-GGTLC3.18516335.18518165.rev.compl.REF  ------------------------------------------------------------ 31039

chimp.LOC112206744-LOC107973052-GGT2.revcompl                            ------------------------------------------------------------ 61762
chimp54546-135457.revcompl.Pan.troglodytes.clone.rp43-41g5.GenBank:AC099533.36    ---------------------------------------TGTGTTACAGTCTTGAAGCAG 71172
human.FAM30B-LOC105372935-GGT2.NCBI.                                     ctggactatatcaagtgtgaacgatcatacttgttatcatgtgggaaaaaaggtttaatt 105360
human.FAM230E-LOC105377182-GGT3P.NCBI.                                   ------------------------------------------------------------ 57310
human.FAM230J-LOC105372942-GGTLC5P.NCBI.rev.compl                        ------------------------------------------------------------ 46364
human.FAM230A.ncRNA.18487127.18500594-GGTLC3.18516335.18518165.rev.compl.REF  ------------------------------------------------------------ 31039

chimp.LOC112206744-LOC107973052-GGT2.revcompl                            ------------------------------------------------------------ 61762
chimp54546-135457.revcompl.Pan.troglodytes.clone.rp43-41g5.GenBank:AC099533.36    AATTCCTGCTTCTCTGGGAAACCTCAGTTTT-----------TGTTCTTAAGGCCTTCAA 71221
human.FAM30B-LOC105372935-GGT2.NCBI.                                     actccctaattgtctgggaaacaaaagatttcaaaatatggagctcctggggaaggaga 105420
human.FAM230E-LOC105377182-GGT3P.NCBI.                                   ------------------------------------------------------------ 57310
human.FAM230J-LOC105372942-GGTLC5P.NCBI.rev.compl                        ------------------------------------------------------------ 46364
human.FAM230A.ncRNA.18487127.18500594-GGTLC3.18516335.18518165.rev.compl.REF  ------------------------------------------------------------ 31039

chimp.LOC112206744-LOC107973052-GGT2.revcompl                            ------------------------------------------------------------ 61762
chimp54546-135457.revcompl.Pan.troglodytes.clone.rp43-41g5.GenBank:AC099533.36    CTGATTGGAGGTGGCCCACCCATATTATGGTGGGTAATCTGTTTTACTTAAAGTCAACTG 71281
human.FAM30B-LOC105372935-GGT2.NCBI.                                     gttaataaaatgggtccttgagaagtgaagtgggaagcctgcctcatgccatggcaccag 105480
human.FAM230E-LOC105377182-GGT3P.NCBI.                                   ------------------------------------------------------------ 57310
human.FAM230J-LOC105372942-GGTLC5P.NCBI.rev.compl                        ------------------------------------------------------------ 46364
human.FAM230A.ncRNA.18487127.18500594-GGTLC3.18516335.18518165.rev.compl.REF  ------------------------------------------------------------ 31039

chimp.LOC112206744-LOC107973052-GGT2.revcompl                            ------------------------------------------------------------ 61762
chimp54546-135457.revcompl.Pan.troglodytes.clone.rp43-41g5.GenBank:AC099533.36    AC---------------------------------------------TGTCAGTGTTAAT 71296
human.FAM30B-LOC105372935-GGT2.NCBI.                                     cgatgccacaggtgtacccagtgccctgccgagagcagagacggtgttctcagagttgct 105540
human.FAM230E-LOC105377182-GGT3P.NCBI.                                   ------------------------------------------------------------ 57310
human.FAM230J-LOC105372942-GGTLC5P.NCBI.rev.compl                        ------------------------------------------------------------ 46364
human.FAM230A.ncRNA.18487127.18500594-GGTLC3.18516335.18518165.rev.compl.REF  ------------------------------------------------------------ 31039

chimp.LOC112206744-LOC107973052-GGT2.revcompl                            ------------------------------------------------------------ 61762
chimp54546-135457.revcompl.Pan.troglodytes.clone.rp43-41g5.GenBank:AC099533.36    CACATCTATGAAATAACCTCC------CAGCAAGATATTGACAAGTATTTGACCAAACAA 71350
human.FAM30B-LOC105372935-GGT2.NCBI.                                     atcaaaaggaggcagcagactgcattctgaagcatcagacaatgggtcacatgcggaaa 105600
human.FAM230E-LOC105377182-GGT3P.NCBI.                                   ------------------------------------------------------------ 57310
human.FAM230J-LOC105372942-GGTLC5P.NCBI.rev.compl                        ------------------------------------------------------------ 46364
human.FAM230A.ncRNA.18487127.18500594-GGTLC3.18516335.18518165.rev.compl.REF  ------------------------------------------------------------ 31039
```

```
chimp.LOC112206744-LOC107973052-GGT2.revcompl                                      ------------------------------------------------------------ 61762
chimp54546-135457.revcompl.Pan.troglodytes.clone.rp43-41g5.GenBank:AC099533.36     CGGGACACCATAGCTTAGCCAAGTTGACACATAAATTAACCATCAGGAGCAAGTAGAATA 71410
human.FAM30B-LOC105372935-GGT2.NCBI.                                               cagaattaaatacacaaaaatagcaccaacttaaggcaagcctgggcaaactgcactaca 105660
human.FAM230E-LOC105377182-GGT3P.NCBI.                                             ------------------------------------------------------------ 57310
human.FAM230J-LOC105372942-GGTLC5P.NCBI.rev.compl                                  ------------------------------------------------------------ 46364
human.FAM230A.ncRNA.18487127.18500594-GGTLC3.18516335.18518165.rev.compl.REF       ------------------------------------------------------------ 31039

chimp.LOC112206744-LOC107973052-GGT2.revcompl                                      ------------------------------------------------------------ 61762
chimp54546-135457.revcompl.Pan.troglodytes.clone.rp43-41g5.GenBank:AC099533.36     TCCAAAAAACAACATACTAGGGGTATTATGTCTTATATAGCTATTATAATTATATAAAAC 71470
human.FAM30B-LOC105372935-GGT2.NCBI.                                               tcaaagaaagaacacacaactggtatcagatttttttttctgttttaataaagagaaggc 105720
human.FAM230E-LOC105377182-GGT3P.NCBI.                                             ------------------------------------------------------------ 57310
human.FAM230J-LOC105372942-GGTLC5P.NCBI.rev.compl                                  ------------------------------------------------------------ 46364
human.FAM230A.ncRNA.18487127.18500594-GGTLC3.18516335.18518165.rev.compl.REF       ------------------------------------------------------------ 31039

chimp.LOC112206744-LOC107973052-GGT2.revcompl                                      ------------------------------------------------------------ 61762
chimp54546-135457.revcompl.Pan.troglodytes.clone.rp43-41g5.GenBank:AC099533.36     AT--------------------------------------------------ATAATTAT 71480
human.FAM30B-LOC105372935-GGT2.NCBI.                                               ctggacacctgaaagttgacgggaagcaggcatcactaatttctccgagttcacagtttt 105780
human.FAM230E-LOC105377182-GGT3P.NCBI.                                             ------------------------------------------------------------ 57310
human.FAM230J-LOC105372942-GGTLC5P.NCBI.rev.compl                                  ------------------------------------------------------------ 46364
human.FAM230A.ncRNA.18487127.18500594-GGTLC3.18516335.18518165.rev.compl.REF       ------------------------------------------------------------ 31039

chimp.LOC112206744-LOC107973052-GGT2.revcompl                                      ------------------------------------------------------------ 61762
chimp54546-135457.revcompl.Pan.troglodytes.clone.rp43-41g5.GenBank:AC099533.36     AGAATGAAGATATTAAGATAACCATTAGAACAAAAATATAAACTTTTCTTTCTTTCTTTT 71540
human.FAM30B-LOC105372935-GGT2.NCBI.                                               gttagaatgtctttgctctgattatggttttatttatttatttttttgagatggagtttt 105840
human.FAM230E-LOC105377182-GGT3P.NCBI.                                             ------------------------------------------------------------ 57310
human.FAM230J-LOC105372942-GGTLC5P.NCBI.rev.compl                                  ------------------------------------------------------------ 46364
human.FAM230A.ncRNA.18487127.18500594-GGTLC3.18516335.18518165.rev.compl.REF       ------------------------------------------------------------ 31039

chimp.LOC112206744-LOC107973052-GGT2.revcompl                                      ------------------------------------------------------------ 61762
chimp54546-135457.revcompl.Pan.troglodytes.clone.rp43-41g5.GenBank:AC099533.36     TTTTTTTTTGAGACCAAGTCTTGCTCTGTCACCCAGGCTGGAGTGCAGTGGTGCAATCTT 71600
human.FAM30B-LOC105372935-GGT2.NCBI.                                               ttttgttttgtttt---atttgcttttgttgccctggctggagtgcagtggtgcaatctc 105897
human.FAM230E-LOC105377182-GGT3P.NCBI.                                             ------------------------------------------------------------ 57310
human.FAM230J-LOC105372942-GGTLC5P.NCBI.rev.compl                                  ------------------------------------------------------------ 46364
human.FAM230A.ncRNA.18487127.18500594-GGTLC3.18516335.18518165.rev.compl.REF       ------------------------------------------------------------ 31039

chimp.LOC112206744-LOC107973052-GGT2.revcompl                                      ------------------------------------------------------------ 61762
chimp54546-135457.revcompl.Pan.troglodytes.clone.rp43-41g5.GenBank:AC099533.36     GGCTTACTGCAACCTTTGCCTCCTGGGTTCAAGTGATTCTCCTGTCTCAGCCTCCCAAGT 71660
human.FAM30B-LOC105372935-GGT2.NCBI.                                               ggctcaccgcaaactccgcctcccaggttcaagagattctcctgcctcagcctcccaagt 105957
human.FAM230E-LOC105377182-GGT3P.NCBI.                                             ------------------------------------------------------------ 57310
human.FAM230J-LOC105372942-GGTLC5P.NCBI.rev.compl                                  ------------------------------------------------------------ 46364
human.FAM230A.ncRNA.18487127.18500594-GGTLC3.18516335.18518165.rev.compl.REF       ------------------------------------------------------------ 31039

chimp.LOC112206744-LOC107973052-GGT2.revcompl                                      ------------------------------------------------------------ 61762
chimp54546-135457.revcompl.Pan.troglodytes.clone.rp43-41g5.GenBank:AC099533.36     AGCTGGGATTACAGGCACCCGCTACCATGCCCAAGCATCCACCATGTGGTGGTATTTGGA 71720
human.FAM30B-LOC105372935-GGT2.NCBI.                                               agctaggattacaggcacccactacca--------cgtctggcttctttgtgtttttagt 106009
human.FAM230E-LOC105377182-GGT3P.NCBI.                                             ------------------------------------------------------------ 57310
human.FAM230J-LOC105372942-GGTLC5P.NCBI.rev.compl                                  ------------------------------------------------------------ 46364
human.FAM230A.ncRNA.18487127.18500594-GGTLC3.18516335.18518165.rev.compl.REF       ------------------------------------------------------------ 31039

chimp.LOC112206744-LOC107973052-GGT2.revcompl                                      ------------------------------------------------------------ 61762
chimp54546-135457.revcompl.Pan.troglodytes.clone.rp43-41g5.GenBank:AC099533.36     TAAGATGTGCAAGCA--------------------------------------------- 71735
human.FAM30B-LOC105372935-GGT2.NCBI.                                               agagatggggtttcaccatgttggtcaggctggtcttgaactcttgacctcaagcgatcc 106069
human.FAM230E-LOC105377182-GGT3P.NCBI.                                             ------------------------------------------------------------ 57310
human.FAM230J-LOC105372942-GGTLC5P.NCBI.rev.compl                                  ------------------------------------------------------------ 46364
human.FAM230A.ncRNA.18487127.18500594-GGTLC3.18516335.18518165.rev.compl.REF       ------------------------------------------------------------ 31039

chimp.LOC112206744-LOC107973052-GGT2.revcompl                                      ------------------------------------------------------------ 61762
chimp54546-135457.revcompl.Pan.troglodytes.clone.rp43-41g5.GenBank:AC099533.36     ------------------------------------------------------------ 71735
human.FAM30B-LOC105372935-GGT2.NCBI.                                               acccgcctcagcccccaaagtgctggaattacaggcatgagccaccacgcctggcctga 106129
human.FAM230E-LOC105377182-GGT3P.NCBI.                                             ------------------------------------------------------------ 57310
human.FAM230J-LOC105372942-GGTLC5P.NCBI.rev.compl                                  ------------------------------------------------------------ 46364
human.FAM230A.ncRNA.18487127.18500594-GGTLC3.18516335.18518165.rev.compl.REF       ------------------------------------------------------------ 31039

chimp.LOC112206744-LOC107973052-GGT2.revcompl                                      ------------------------------------------------------------ 61762
chimp54546-135457.revcompl.Pan.troglodytes.clone.rp43-41g5.GenBank:AC099533.36     ------------------------------------------------------------ 71735
human.FAM30B-LOC105372935-GGT2.NCBI.                                               ttatgttttgatggatatacagaagctcttgctttggtacagaaacaggccccgctttgc 106189
human.FAM230E-LOC105377182-GGT3P.NCBI.                                             ------------------------------------------------------------ 57310
human.FAM230J-LOC105372942-GGTLC5P.NCBI.rev.compl                                  ------------------------------------------------------------ 46364
human.FAM230A.ncRNA.18487127.18500594-GGTLC3.18516335.18518165.rev.compl.REF       ------------------------------------------------------------ 31039

chimp.LOC112206744-LOC107973052-GGT2.revcompl                                      ------------------------------------------------------------ 61762
chimp54546-135457.revcompl.Pan.troglodytes.clone.rp43-41g5.GenBank:AC099533.36     ------------------------------------------------------------ 71735
human.FAM30B-LOC105372935-GGT2.NCBI.                                               ttcgacctctgggcccccaccttcagaaatgtctggtgccattcctggctcctcctctc 106249
human.FAM230E-LOC105377182-GGT3P.NCBI.                                             ------------------------------------------------------------ 57310
human.FAM230J-LOC105372942-GGTLC5P.NCBI.rev.compl                                  ------------------------------------------------------------ 46364
human.FAM230A.ncRNA.18487127.18500594-GGTLC3.18516335.18518165.rev.compl.REF       ------------------------------------------------------------ 31039

chimp.LOC112206744-LOC107973052-GGT2.revcompl                                      ------------------------------------------------------------ 61762
chimp54546-135457.revcompl.Pan.troglodytes.clone.rp43-41g5.GenBank:AC099533.36     ------------------------------------------------------------ 71735
human.FAM30B-LOC105372935-GGT2.NCBI.                                               agtggtcttccaggatatccactcacagctttctgatgtcacccacctgtctctagccca 106309
human.FAM230E-LOC105377182-GGT3P.NCBI.                                             ------------------------------------------------------------ 57310
human.FAM230J-LOC105372942-GGTLC5P.NCBI.rev.compl                                  ------------------------------------------------------------ 46364
human.FAM230A.ncRNA.18487127.18500594-GGTLC3.18516335.18518165.rev.compl.REF       ------------------------------------------------------------ 31039

chimp.LOC112206744-LOC107973052-GGT2.revcompl                                      ------------------------------------------------------------ 61762
chimp54546-135457.revcompl.Pan.troglodytes.clone.rp43-41g5.GenBank:AC099533.36     ------------------------------------------------------------ 71735
human.FAM30B-LOC105372935-GGT2.NCBI.                                               gatacccagtcctcatggcaagtgactgcagcaatttttacaaatctacgcccttagggt 106369
human.FAM230E-LOC105377182-GGT3P.NCBI.                                             ------------------------------------------------------------ 57310
human.FAM230J-LOC105372942-GGTLC5P.NCBI.rev.compl                                  ------------------------------------------------------------ 46364
human.FAM230A.ncRNA.18487127.18500594-GGTLC3.18516335.18518165.rev.compl.REF       ------------------------------------------------------------ 31039

chimp.LOC112206744-LOC107973052-GGT2.revcompl                                      ------------------------------------------------------------ 61762
chimp54546-135457.revcompl.Pan.troglodytes.clone.rp43-41g5.GenBank:AC099533.36     ------------------------------------------------------------ 71735
human.FAM30B-LOC105372935-GGT2.NCBI.                                               tcccatcccaggacgttcaggtttctccacccctttagaagtgcccagaatatcccctac 106429
human.FAM230E-LOC105377182-GGT3P.NCBI.                                             ------------------------------------------------------------ 57310
human.FAM230J-LOC105372942-GGTLC5P.NCBI.rev.compl                                  ------------------------------------------------------------ 46364
human.FAM230A.ncRNA.18487127.18500594-GGTLC3.18516335.18518165.rev.compl.REF       ------------------------------------------------------------ 31039

chimp.LOC112206744-LOC107973052-GGT2.revcompl                                      ------------------------------------------------------------ 61762
chimp54546-135457.revcompl.Pan.troglodytes.clone.rp43-41g5.GenBank:AC099533.36     ------------------------------------------------------------ 71735
human.FAM30B-LOC105372935-GGT2.NCBI.                                               cccacgattccaacaggacgtcactctcctcttacttaagaaaaatgcctaaaattccag 106489
human.FAM230E-LOC105377182-GGT3P.NCBI.                                             ------------------------------------------------------------ 57310
human.FAM230J-LOC105372942-GGTLC5P.NCBI.rev.compl                                  ------------------------------------------------------------ 46364
human.FAM230A.ncRNA.18487127.18500594-GGTLC3.18516335.18518165.rev.compl.REF       ------------------------------------------------------------ 31039

chimp.LOC112206744-LOC107973052-GGT2.revcompl                                      ------------------------------------------------------------ 61762
chimp54546-135457.revcompl.Pan.troglodytes.clone.rp43-41g5.GenBank:AC099533.36     ------------------------------------------------------------ 71735
human.FAM30B-LOC105372935-GGT2.NCBI.                                               cccttccaaaaggcctttctagattgaccactcaggagttactctggcctccccttcact 106549
human.FAM230E-LOC105377182-GGT3P.NCBI.                                             ------------------------------------------------------------ 57310
human.FAM230J-LOC105372942-GGTLC5P.NCBI.rev.compl                                  ------------------------------------------------------------ 46364
human.FAM230A.ncRNA.18487127.18500594-GGTLC3.18516335.18518165.rev.compl.REF       ------------------------------------------------------------ 31039

chimp.LOC112206744-LOC107973052-GGT2.revcompl                                      ------------------------------------------------------------ 61762
chimp54546-135457.revcompl.Pan.troglodytes.clone.rp43-41g5.GenBank:AC099533.36     ------------------------------------------------------------ 71735
human.FAM30B-LOC105372935-GGT2.NCBI.                                               cctgctagactgaaaacaaggacagacttgcctggcctctctcccccagtagacacctac 106609
human.FAM230E-LOC105377182-GGT3P.NCBI.                                             ------------------------------------------------------------ 57310
human.FAM230J-LOC105372942-GGTLC5P.NCBI.rev.compl                                  ------------------------------------------------------------ 46364
human.FAM230A.ncRNA.18487127.18500594-GGTLC3.18516335.18518165.rev.compl.REF       ------------------------------------------------------------ 31039

chimp.LOC112206744-LOC107973052-GGT2.revcompl                                      ------------------------------------------------------------ 61762
chimp54546-135457.revcompl.Pan.troglodytes.clone.rp43-41g5.GenBank:AC099533.36     ------------------------------------------------------------ 71735
human.FAM30B-LOC105372935-GGT2.NCBI.                                               ccccagtgaacacatcctgtcagtgtcctcccgtccatttgcaaattgattgactgtttc 106669
human.FAM230E-LOC105377182-GGT3P.NCBI.                                             ------------------------------------------------------------ 57310
human.FAM230J-LOC105372942-GGTLC5P.NCBI.rev.compl                                  ------------------------------------------------------------ 46364
human.FAM230A.ncRNA.18487127.18500594-GGTLC3.18516335.18518165.rev.compl.REF       ------------------------------------------------------------ 31039
```

```
chimp.LOC112206744-LOC107973052-GGT2.revcompl                                    ------------------------------------------------------------ 61762
chimp54546-135457.revcompl.Pan.troglodytes.clone.rp43-41g5.GenBank:AC099533.36   ------------------------------------------------------------ 71735
human.FAM30B-LOC105372935-GGT2.NCBI.                                             attgctggccttggatgacctctcctagagggcagggacggtggtgttcggcaaagcctt 106729
human.FAM230E-LOC105377182-GGT3P.NCBI.                                           ------------------------------------------------------------ 57310
human.FAM230J-LOC105372942-GGTLC5P.NCBI.rev.compl                                ------------------------------------------------------------ 46364
human.FAM230A.ncRNA.18487127.18500594-GGTLC3.18516335.18518165.rev.compl.REF     ------------------------------------------------------------ 31039

chimp.LOC112206744-LOC107973052-GGT2.revcompl                                    ------------------------------------------------------------ 61762
chimp54546-135457.revcompl.Pan.troglodytes.clone.rp43-41g5.GenBank:AC099533.36   --------------------------------GTGAAAGGTCATTGGAAGATTTGGGGAG 71763
human.FAM30B-LOC105372935-GGT2.NCBI.                                             ggtcgtctggataatgggaaatggggtcttctggtgaatttaattacagggttaggtgga 106789
human.FAM230E-LOC105377182-GGT3P.NCBI.                                           ------------------------------------------------------------ 57310
human.FAM230J-LOC105372942-GGTLC5P.NCBI.rev.compl                                ------------------------------------------------------------ 46364
human.FAM230A.ncRNA.18487127.18500594-GGTLC3.18516335.18518165.rev.compl.REF     ------------------------------------------------------------ 31039

chimp.LOC112206744-LOC107973052-GGT2.revcompl                                    ------------------------------------------------------------ 61762
chimp54546-135457.revcompl.Pan.troglodytes.clone.rp43-41g5.GenBank:AC099533.36   ACCCATGCATTCA----------------------------------------------- 71776
human.FAM30B-LOC105372935-GGT2.NCBI.                                             aactttgcttcaactctcattattagaaagtctattgtaaagctttttaaaatacaactg 106849
human.FAM230E-LOC105377182-GGT3P.NCBI.                                           ------------------------------------------------------------ 57310
human.FAM230J-LOC105372942-GGTLC5P.NCBI.rev.compl                                ------------------------------------------------------------ 46364
human.FAM230A.ncRNA.18487127.18500594-GGTLC3.18516335.18518165.rev.compl.REF     ------------------------------------------------------------ 31039

chimp.LOC112206744-LOC107973052-GGT2.revcompl                                    ------------------------------------------------------------ 61762
chimp54546-135457.revcompl.Pan.troglodytes.clone.rp43-41g5.GenBank:AC099533.36   ------------------------------------------------------------ 71776
human.FAM30B-LOC105372935-GGT2.NCBI.                                             accactttccagcgagggtagagttaatatgccttaatctttgatttaggatcttgcagt 106909
human.FAM230E-LOC105377182-GGT3P.NCBI.                                           ------------------------------------------------------------ 57310
human.FAM230J-LOC105372942-GGTLC5P.NCBI.rev.compl                                ------------------------------------------------------------ 46364
human.FAM230A.ncRNA.18487127.18500594-GGTLC3.18516335.18518165.rev.compl.REF     ------------------------------------------------------------ 31039

chimp.LOC112206744-LOC107973052-GGT2.revcompl                                    ------------------------------------------------------------ 61762
chimp54546-135457.revcompl.Pan.troglodytes.clone.rp43-41g5.GenBank:AC099533.36   ------------------------------------------------------------ 71776
human.FAM30B-LOC105372935-GGT2.NCBI.                                             gccccaggctcaccattctcttctgctactataattatagatgcggaagacatacagact 106969
human.FAM230E-LOC105377182-GGT3P.NCBI.                                           ------------------------------------------------------------ 57310
human.FAM230J-LOC105372942-GGTLC5P.NCBI.rev.compl                                ------------------------------------------------------------ 46364
human.FAM230A.ncRNA.18487127.18500594-GGTLC3.18516335.18518165.rev.compl.REF     ------------------------------------------------------------ 31039

chimp.LOC112206744-LOC107973052-GGT2.revcompl                                    ------------------------------------------------------------ 61762
chimp54546-135457.revcompl.Pan.troglodytes.clone.rp43-41g5.GenBank:AC099533.36   ------------------------------------------------------------ 71776
human.FAM30B-LOC105372935-GGT2.NCBI.                                             gggctgacaactgcttggaagctaatttctagaataaacccttgctaccacatcttgtga 107029
human.FAM230E-LOC105377182-GGT3P.NCBI.                                           ------------------------------------------------------------ 57310
human.FAM230J-LOC105372942-GGTLC5P.NCBI.rev.compl                                ------------------------------------------------------------ 46364
human.FAM230A.ncRNA.18487127.18500594-GGTLC3.18516335.18518165.rev.compl.REF     ------------------------------------------------------------ 31039

chimp.LOC112206744-LOC107973052-GGT2.revcompl                                    ------------------------------------------------------------ 61762
chimp54546-135457.revcompl.Pan.troglodytes.clone.rp43-41g5.GenBank:AC099533.36   ------------------------------------------------------------ 71776
human.FAM30B-LOC105372935-GGT2.NCBI.                                             tttttgcccttctgaaggtggagttccaaaataaaaacaaaatcccgcttaactgagggt 107089
human.FAM230E-LOC105377182-GGT3P.NCBI.                                           ------------------------------------------------------------ 57310
human.FAM230J-LOC105372942-GGTLC5P.NCBI.rev.compl                                ------------------------------------------------------------ 46364
human.FAM230A.ncRNA.18487127.18500594-GGTLC3.18516335.18518165.rev.compl.REF     ------------------------------------------------------------ 31039

chimp.LOC112206744-LOC107973052-GGT2.revcompl                                    ------------------------------------------------------------ 61762
chimp54546-135457.revcompl.Pan.troglodytes.clone.rp43-41g5.GenBank:AC099533.36   ------------------------------------------------------------ 71776
human.FAM30B-LOC105372935-GGT2.NCBI.                                             tccagttgagggaggtctgctgccatggtctttgttaagtgcctgccaccatggcccaca 107149
human.FAM230E-LOC105377182-GGT3P.NCBI.                                           ------------------------------------------------------------ 57310
human.FAM230J-LOC105372942-GGTLC5P.NCBI.rev.compl                                ------------------------------------------------------------ 46364
human.FAM230A.ncRNA.18487127.18500594-GGTLC3.18516335.18518165.rev.compl.REF     ------------------------------------------------------------ 31039

chimp.LOC112206744-LOC107973052-GGT2.revcompl                                    ------------------------------------------------------------ 61762
chimp54546-135457.revcompl.Pan.troglodytes.clone.rp43-41g5.GenBank:AC099533.36   -------------------------GAGAATAGGAAAGCAAACCTAGGGAGTCATCCTTC 71811
human.FAM30B-LOC105372935-GGT2.NCBI.                                             attagtcagttaataaagggttcttgatgagagaatttggcaacatgtgatttccacaa 107209
human.FAM230E-LOC105377182-GGT3P.NCBI.                                           ------------------------------------------------------------ 57310
human.FAM230J-LOC105372942-GGTLC5P.NCBI.rev.compl                                ------------------------------------------------------------ 46364
human.FAM230A.ncRNA.18487127.18500594-GGTLC3.18516335.18518165.rev.compl.REF     ------------------------------------------------------------ 31039

chimp.LOC112206744-LOC107973052-GGT2.revcompl                                    ------------------------------------------------------------ 61762
chimp54546-135457.revcompl.Pan.troglodytes.clone.rp43-41g5.GenBank:AC099533.36   ATATGAGCAGAAATGTTTAGGGCCTTC----TTGTTTGGAACAGCTGAGCACCTCCCATA 71867
human.FAM30B-LOC105372935-GGT2.NCBI.                                             atataagcttgattttgatgcaaccactcatttctttgaaaatcaggggcattcacaca 107269
human.FAM230E-LOC105377182-GGT3P.NCBI.                                           ------------------------------------------------------------ 57310
human.FAM230J-LOC105372942-GGTLC5P.NCBI.rev.compl                                ------------------------------------------------------------ 46364
human.FAM230A.ncRNA.18487127.18500594-GGTLC3.18516335.18518165.rev.compl.REF     ------------------------------------------------------------ 31039

chimp.LOC112206744-LOC107973052-GGT2.revcompl                                    ------------------------------------------------------------ 61762
chimp54546-135457.revcompl.Pan.troglodytes.clone.rp43-41g5.GenBank:AC099533.36   GAAAAAAAGGACACATTATTACATCCTAACTTCGCATCAAGAAGAAGTATATATGGTCAT 71927
human.FAM30B-LOC105372935-GGT2.NCBI.                                             caggtgttctagatttccttacagtaaaaccaaccctttacaaggaatagctgtgttcac 107329
human.FAM230E-LOC105377182-GGT3P.NCBI.                                           ------------------------------------------------------------ 57310
human.FAM230J-LOC105372942-GGTLC5P.NCBI.rev.compl                                ------------------------------------------------------------ 46364
human.FAM230A.ncRNA.18487127.18500594-GGTLC3.18516335.18518165.rev.compl.REF     ------------------------------------------------------------ 31039

chimp.LOC112206744-LOC107973052-GGT2.revcompl                                    ------------------------------------------------------------ 61762
chimp54546-135457.revcompl.Pan.troglodytes.clone.rp43-41g5.GenBank:AC099533.36   GATGGATTCTGGAAGAAGCA---------------------------------------- 71947
human.FAM30B-LOC105372935-GGT2.NCBI.                                             tttgaagcagtaaagaagcaggtgtccactcatggcaaatgaggaactccctttttttt 107389
human.FAM230E-LOC105377182-GGT3P.NCBI.                                           ------------------------------------------------------------ 57310
human.FAM230J-LOC105372942-GGTLC5P.NCBI.rev.compl                                ------------------------------------------------------------ 46364
human.FAM230A.ncRNA.18487127.18500594-GGTLC3.18516335.18518165.rev.compl.REF     ------------------------------------------------------------ 31039

chimp.LOC112206744-LOC107973052-GGT2.revcompl                                    ------------------------------------------------------------ 61762
chimp54546-135457.revcompl.Pan.troglodytes.clone.rp43-41g5.GenBank:AC099533.36   -----------------------------------------TTATTCACTCTTGGGATGT 71966
human.FAM30B-LOC105372935-GGT2.NCBI.                                             tttttttttttttttttttttttgagacggggtctcgctctttttttgtgtgtgtgagat 107449
human.FAM230E-LOC105377182-GGT3P.NCBI.                                           ------------------------------------------------------------ 57310
human.FAM230J-LOC105372942-GGTLC5P.NCBI.rev.compl                                ------------------------------------------------------------ 46364
human.FAM230A.ncRNA.18487127.18500594-GGTLC3.18516335.18518165.rev.compl.REF     ------------------------------------------------------------ 31039

chimp.LOC112206744-LOC107973052-GGT2.revcompl                                    ------------------------------------------------------------ 61762
chimp54546-135457.revcompl.Pan.troglodytes.clone.rp43-41g5.GenBank:AC099533.36   TGCTCCAACCCAAATAGCAAAAACAAAGAAAAGTTGCATGTCTGTGGGTCCTAGAGCAGG 72026
human.FAM30B-LOC105372935-GGT2.NCBI.                                             gggggccacccaggctagagtgcagtagtgcaattacagctcactgtagcctcaaccttc 107509
human.FAM230E-LOC105377182-GGT3P.NCBI.                                           ------------------------------------------------------------ 57310
human.FAM230J-LOC105372942-GGTLC5P.NCBI.rev.compl                                ------------------------------------------------------------ 46364
human.FAM230A.ncRNA.18487127.18500594-GGTLC3.18516335.18518165.rev.compl.REF     ------------------------------------------------------------ 31039

chimp.LOC112206744-LOC107973052-GGT2.revcompl                                    ------------------------------------------------------------ 61762
chimp54546-135457.revcompl.Pan.troglodytes.clone.rp43-41g5.GenBank:AC099533.36   AAGGCT------------------------------------------------------ 72032
human.FAM30B-LOC105372935-GGT2.NCBI.                                             tgggctcaaccagttcttcccacctcaacttctgaagcagctgagactacaggtgtgtac 107569
human.FAM230E-LOC105377182-GGT3P.NCBI.                                           ------------------------------------------------------------ 57310
human.FAM230J-LOC105372942-GGTLC5P.NCBI.rev.compl                                ------------------------------------------------------------ 46364
human.FAM230A.ncRNA.18487127.18500594-GGTLC3.18516335.18518165.rev.compl.REF     ------------------------------------------------------------ 31039

chimp.LOC112206744-LOC107973052-GGT2.revcompl                                    ------------------------------------------------------------ 61762
chimp54546-135457.revcompl.Pan.troglodytes.clone.rp43-41g5.GenBank:AC099533.36   ------------------------------------------------------------ 72032
human.FAM30B-LOC105372935-GGT2.NCBI.                                             caccatgcccaggtaatgcttttgatttttagtaagatgaggtctcgttatattgcccag 107629
human.FAM230E-LOC105377182-GGT3P.NCBI.                                           ------------------------------------------------------------ 57310
human.FAM230J-LOC105372942-GGTLC5P.NCBI.rev.compl                                ------------------------------------------------------------ 46364
human.FAM230A.ncRNA.18487127.18500594-GGTLC3.18516335.18518165.rev.compl.REF     ------------------------------------------------------------ 31039

chimp.LOC112206744-LOC107973052-GGT2.revcompl                                    ------------------------------------------------------------ 61762
chimp54546-135457.revcompl.Pan.troglodytes.clone.rp43-41g5.GenBank:AC099533.36   ----------------------------------CTGCAGCAGGTCCACACAGTGGTGGG 72058
human.FAM30B-LOC105372935-GGT2.NCBI.                                             gctggtctcaaactcctgagctcaagtgatcctcctgcctcagcctcccaaagtgctagg 107689
human.FAM230E-LOC105377182-GGT3P.NCBI.                                           ------------------------------------------------------------ 57310
human.FAM230J-LOC105372942-GGTLC5P.NCBI.rev.compl                                ------------------------------------------------------------ 46364
human.FAM230A.ncRNA.18487127.18500594-GGTLC3.18516335.18518165.rev.compl.REF     ------------------------------------------------------------ 31039

chimp.LOC112206744-LOC107973052-GGT2.revcompl                                    ------------------------------------------------------------ 61762
chimp54546-135457.revcompl.Pan.troglodytes.clone.rp43-41g5.GenBank:AC099533.36   AGTGCCCTGCTGGTTGAGCCCTAGAACCCAGCTGGGTCTACTGCAATGCAGGAATTTATG 72118
human.FAM30B-LOC105372935-GGT2.NCBI.                                             attatcggcatgagccactatgcccagccagaagctaaaaatattaatcaaggagtcaca 107749
human.FAM230E-LOC105377182-GGT3P.NCBI.                                           ------------------------------------------------------------ 57310
human.FAM230J-LOC105372942-GGTLC5P.NCBI.rev.compl                                ------------------------------------------------------------ 46364
human.FAM230A.ncRNA.18487127.18500594-GGTLC3.18516335.18518165.rev.compl.REF     ------------------------------------------------------------ 31039

chimp.LOC112206744-LOC107973052-GGT2.revcompl                                    ------------------------------------------------------------ 61762
```

```
chimp54546-135457.revcompl.Pan.troglodytes.clone.rp43-41g5.GenBank:AC099533.36      ATGGGAGAAGAAACCTTGCATTTATGGCAAGACCCAATAGGAG----------------- 72161
human.FAM30B-LOC105372935-GGT2.NCBI.                                                 actaaggatgcactgcacacccgaagccagggaccacaagaggtcagcaactgggactgc 107809
human.FAM230E-LOC105377182-GGT3P.NCBI.                                               ------------------------------------------------------------ 57310
human.FAM230J-LOC105372942-GGTLC5P.NCBI.rev.compl                                    ------------------------------------------------------------ 46364
human.FAM230A.ncRNA.18487127.18500594-GGTLC3.18516335.18518165.rev.compl.REF         ------------------------------------------------------------ 31039

chimp.LOC112206744-LOC107973052-GGT2.revcompl                                        ------------------------------------------------------------ 61762
chimp54546-135457.revcompl.Pan.troglodytes.clone.rp43-41g5.GenBank:AC099533.36      ------------------------------------------------------------ 72161
human.FAM30B-LOC105372935-GGT2.NCBI.                                                 ttcctccagttcccaggctggggctcagcagcagtggcctgggctctggggacttacttt 107869
human.FAM230E-LOC105377182-GGT3P.NCBI.                                               ------------------------------------------------------------ 57310
human.FAM230J-LOC105372942-GGTLC5P.NCBI.rev.compl                                    ------------------------------------------------------------ 46364
human.FAM230A.ncRNA.18487127.18500594-GGTLC3.18516335.18518165.rev.compl.REF         ------------------------------------------------------------ 31039

chimp.LOC112206744-LOC107973052-GGT2.revcompl                                        ------------------------------------------------------------ 61762
chimp54546-135457.revcompl.Pan.troglodytes.clone.rp43-41g5.GenBank:AC099533.36      ------------------------------------------------------------ 72161
human.FAM30B-LOC105372935-GGT2.NCBI.                                                 aagccgaaatcccaaactcattatcattcaaaccaattttctgtgtccagcatcatccca 107929
human.FAM230E-LOC105377182-GGT3P.NCBI.                                               ------------------------------------------------------------ 57310
human.FAM230J-LOC105372942-GGTLC5P.NCBI.rev.compl                                    ------------------------------------------------------------ 46364
human.FAM230A.ncRNA.18487127.18500594-GGTLC3.18516335.18518165.rev.compl.REF         ------------------------------------------------------------ 31039

chimp.LOC112206744-LOC107973052-GGT2.revcompl                                        ------------------------------------------------------------ 61762
chimp54546-135457.revcompl.Pan.troglodytes.clone.rp43-41g5.GenBank:AC099533.36      ------------------------------------------------------------ 72161
human.FAM30B-LOC105372935-GGT2.NCBI.                                                 cctaaactggtccttaaaaccactgacagtcatttctccaatctgtaaaaggcctgctga 107989
human.FAM230E-LOC105377182-GGT3P.NCBI.                                               ------------------------------------------------------------ 57310
human.FAM230J-LOC105372942-GGTLC5P.NCBI.rev.compl                                    ------------------------------------------------------------ 46364
human.FAM230A.ncRNA.18487127.18500594-GGTLC3.18516335.18518165.rev.compl.REF         ------------------------------------------------------------ 31039

chimp.LOC112206744-LOC107973052-GGT2.revcompl                                        ------------------------------------------------------------ 61762
chimp54546-135457.revcompl.Pan.troglodytes.clone.rp43-41g5.GenBank:AC099533.36      ----------------------------------------------ATCCCAGTGCAGAC 72175
human.FAM30B-LOC105372935-GGT2.NCBI.                                                 ggcctttgcattatcaggacttaactgggagtagaatgccctccccatccaaatcctgac 108049
human.FAM230E-LOC105377182-GGT3P.NCBI.                                               ------------------------------------------------------------ 57310
human.FAM230J-LOC105372942-GGTLC5P.NCBI.rev.compl                                    ------------------------------------------------------------ 46364
human.FAM230A.ncRNA.18487127.18500594-GGTLC3.18516335.18518165.rev.compl.REF         ------------------------------------------------------------ 31039

chimp.LOC112206744-LOC107973052-GGT2.revcompl                                        ------------------------------------------------------------ 61762
chimp54546-135457.revcompl.Pan.troglodytes.clone.rp43-41g5.GenBank:AC099533.36      ATGTGGGGTTGTGTAACAAGGCCCTGTCATCTTAATGAAATTTGCCCCT----------- 72224
human.FAM30B-LOC105372935-GGT2.NCBI.                                                 catttc---tgaacactcagcccatgtcctcttcctgagtgtcttccccaacaacaacca 108106
human.FAM230E-LOC105377182-GGT3P.NCBI.                                               ------------------------------------------------------------ 57310
human.FAM230J-LOC105372942-GGTLC5P.NCBI.rev.compl                                    ------------------------------------------------------------ 46364
human.FAM230A.ncRNA.18487127.18500594-GGTLC3.18516335.18518165.rev.compl.REF         ------------------------------------------------------------ 31039

chimp.LOC112206744-LOC107973052-GGT2.revcompl                                        ------------------------------------------------------------ 61762
chimp54546-135457.revcompl.Pan.troglodytes.clone.rp43-41g5.GenBank:AC099533.36      TCTGGAAAGGTTTGTTCTGTCTTTGCTGCTCACACCTGAGAGTCGTGTGTGCACTTCTTT 72284
human.FAM30B-LOC105372935-GGT2.NCBI.                                                 cctacactgaactttactttgatctctgcaggaaactgtagatagagatggggtctcact 108166
human.FAM230E-LOC105377182-GGT3P.NCBI.                                               ------------------------------------------------------------ 57310
human.FAM230J-LOC105372942-GGTLC5P.NCBI.rev.compl                                    ------------------------------------------------------------ 46364
human.FAM230A.ncRNA.18487127.18500594-GGTLC3.18516335.18518165.rev.compl.REF         ------------------------------------------------------------ 31039

chimp.LOC112206744-LOC107973052-GGT2.revcompl                                        ------------------------------------------------------------ 61762
chimp54546-135457.revcompl.Pan.troglodytes.clone.rp43-41g5.GenBank:AC099533.36      CTCATGTGCACGTGCAAGACAACTCCTGTTCTCAGACACCCAGAGAAGAAAAGGCTTGAA 72344
human.FAM30B-LOC105372935-GGT2.NCBI.                                                 atgttgcctagctggtcttgaactgctgagctcaa------------------------- 108201
human.FAM230E-LOC105377182-GGT3P.NCBI.                                               ------------------------------------------------------------ 57310
human.FAM230J-LOC105372942-GGTLC5P.NCBI.rev.compl                                    ------------------------------------------------------------ 46364
human.FAM230A.ncRNA.18487127.18500594-GGTLC3.18516335.18518165.rev.compl.REF         ------------------------------------------------------------ 31039

chimp.LOC112206744-LOC107973052-GGT2.revcompl                                        ------------------------------------------------------------ 61762
chimp54546-135457.revcompl.Pan.troglodytes.clone.rp43-41g5.GenBank:AC099533.36      ACTGAGGAGCTGATCCCTAGGATTCCATTGCACCTGCCTGGCCCAGACAGAGCTCCTGAA 72404
human.FAM30B-LOC105372935-GGT2.NCBI.                                                 ------------------gtgatctccctgcttctgcctcccaaagtgctaggattacag 108243
human.FAM230E-LOC105377182-GGT3P.NCBI.                                               ------------------------------------------------------------ 57310
human.FAM230J-LOC105372942-GGTLC5P.NCBI.rev.compl                                    ------------------------------------------------------------ 46364
human.FAM230A.ncRNA.18487127.18500594-GGTLC3.18516335.18518165.rev.compl.REF         ------------------------------------------------------------ 31039

chimp.LOC112206744-LOC107973052-GGT2.revcompl                                        ------------------------------------------------------------ 61762
chimp54546-135457.revcompl.Pan.troglodytes.clone.rp43-41g5.GenBank:AC099533.36      ATGAAAGGAGCTGGGCTGGGCCAGAGTAGCACATCTCATTCTGCGGGCACAATCCTACTG 72464
human.FAM30B-LOC105372935-GGT2.NCBI.                                                 cagtgagccaccacacctggcctatgtatg-cttttttttttcttttctttttttttttt 108302
human.FAM230E-LOC105377182-GGT3P.NCBI.                                               ------------------------------------------------------------ 57310
human.FAM230J-LOC105372942-GGTLC5P.NCBI.rev.compl                                    ------------------------------------------------------------ 46364
human.FAM230A.ncRNA.18487127.18500594-GGTLC3.18516335.18518165.rev.compl.REF         ------------------------------------------------------------ 31039

chimp.LOC112206744-LOC107973052-GGT2.revcompl                                        ------------------------------------------------------------ 61762
chimp54546-135457.revcompl.Pan.troglodytes.clone.rp43-41g5.GenBank:AC099533.36      GTTCAAAATGCTTTCTTTTTCTCAAGCCCAA-----------------------GTTCTC 72501
human.FAM30B-LOC105372935-GGT2.NCBI.                                                 ttttgagacagagtcttgctctgtggcccagtccgcagtgcagtggtgcaatctcggctc 108362
human.FAM230E-LOC105377182-GGT3P.NCBI.                                               ------------------------------------------------------------ 57310
human.FAM230J-LOC105372942-GGTLC5P.NCBI.rev.compl                                    ------------------------------------------------------------ 46364
human.FAM230A.ncRNA.18487127.18500594-GGTLC3.18516335.18518165.rev.compl.REF         ------------------------------------------------------------ 31039

chimp.LOC112206744-LOC107973052-GGT2.revcompl                                        ------------------------------------------------------------ 61762
chimp54546-135457.revcompl.Pan.troglodytes.clone.rp43-41g5.GenBank:AC099533.36      AGGGTTAGCTCAGAGACCTGGTTCCTTTCCTGCCACACTCTGTGTTGAAAGGGAGGGAAA 72561
human.FAM30B-LOC105372935-GGT2.NCBI.                                                 atggcaacctccaggttcaaggcattttcctgcctcagccttccaaggagctgggattac 108422
human.FAM230E-LOC105377182-GGT3P.NCBI.                                               ------------------------------------------------------------ 57310
human.FAM230J-LOC105372942-GGTLC5P.NCBI.rev.compl                                    ------------------------------------------------------------ 46364
human.FAM230A.ncRNA.18487127.18500594-GGTLC3.18516335.18518165.rev.compl.REF         ------------------------------------------------------------ 31039

chimp.LOC112206744-LOC107973052-GGT2.revcompl                                        ------------------------------------------------------------ 61762
chimp54546-135457.revcompl.Pan.troglodytes.clone.rp43-41g5.GenBank:AC099533.36      CGACTGTCTCTAGTGCAGAAA---------CCAAGCTCTCTGGAGACATTTCTTATCTCA 72612
human.FAM30B-LOC105372935-GGT2.NCBI.                                                 aggcacgcaccatcacgcccagctaattggccaggctggtctcaaactcctgacctcagg 108482
human.FAM230E-LOC105377182-GGT3P.NCBI.                                               ------------------------------------------------------------ 57310
human.FAM230J-LOC105372942-GGTLC5P.NCBI.rev.compl                                    ------------------------------------------------------------ 46364
human.FAM230A.ncRNA.18487127.18500594-GGTLC3.18516335.18518165.rev.compl.REF         ------------------------------------------------------------ 31039

chimp.LOC112206744-LOC107973052-GGT2.revcompl                                        ------------------------------------------------------------ 61762
chimp54546-135457.revcompl.Pan.troglodytes.clone.rp43-41g5.GenBank:AC099533.36      TGAACCTGTGCACTGGTCTCTGTAGGGCCTAGGTAATAAAGGCACAAGATGTTTGGATTT 72672
human.FAM30B-LOC105372935-GGT2.NCBI.                                                 tgatccacccaccttggcctcccaaagtgctgggattacaggtgtaagccaccacaccgg 108542
human.FAM230E-LOC105377182-GGT3P.NCBI.                                               ------------------------------------------------------------ 57310
human.FAM230J-LOC105372942-GGTLC5P.NCBI.rev.compl                                    ------------------------------------------------------------ 46364
human.FAM230A.ncRNA.18487127.18500594-GGTLC3.18516335.18518165.rev.compl.REF         ------------------------------------------------------------ 31039

chimp.LOC112206744-LOC107973052-GGT2.revcompl                                        ------------------------------------------------------------ 61762
chimp54546-135457.revcompl.Pan.troglodytes.clone.rp43-41g5.GenBank:AC099533.36      ACAATGAATGTAATTGTAAAATTCATGGTTAAATAGAA---------------------- 72710
human.FAM30B-LOC105372935-GGT2.NCBI.                                                 gccacgtatgcattttaaaacgttatgctcagacggggtctataggtttcaccagactgc 108602
human.FAM230E-LOC105377182-GGT3P.NCBI.                                               ------------------------------------------------------------ 57310
human.FAM230J-LOC105372942-GGTLC5P.NCBI.rev.compl                                    ------------------------------------------------------------ 46364
human.FAM230A.ncRNA.18487127.18500594-GGTLC3.18516335.18518165.rev.compl.REF         ------------------------------------------------------------ 31039

chimp.LOC112206744-LOC107973052-GGT2.revcompl                                        ------------------------------------------------------------ 61762
chimp54546-135457.revcompl.Pan.troglodytes.clone.rp43-41g5.GenBank:AC099533.36      ------------------------------------------------------------ 72710
human.FAM30B-LOC105372935-GGT2.NCBI.                                                 caaaaggatccatgacacaaaaaaagtactctcagttgcatccttcggaacctttcaaca 108662
human.FAM230E-LOC105377182-GGT3P.NCBI.                                               ------------------------------------------------------------ 57310
human.FAM230J-LOC105372942-GGTLC5P.NCBI.rev.compl                                    ------------------------------------------------------------ 46364
human.FAM230A.ncRNA.18487127.18500594-GGTLC3.18516335.18518165.rev.compl.REF         ------------------------------------------------------------ 31039

chimp.LOC112206744-LOC107973052-GGT2.revcompl                                        ------------------------------------------------------------ 61762
chimp54546-135457.revcompl.Pan.troglodytes.clone.rp43-41g5.GenBank:AC099533.36      ------------------------------------------------------------ 72710
human.FAM30B-LOC105372935-GGT2.NCBI.                                                 catacacatatacctcactcatccttttgtgaagagtgtctattaatcactagattaaga 108722
human.FAM230E-LOC105377182-GGT3P.NCBI.                                               ------------------------------------------------------------ 57310
human.FAM230J-LOC105372942-GGTLC5P.NCBI.rev.compl                                    ------------------------------------------------------------ 46364
human.FAM230A.ncRNA.18487127.18500594-GGTLC3.18516335.18518165.rev.compl.REF         ------------------------------------------------------------ 31039

chimp.LOC112206744-LOC107973052-GGT2.revcompl                                        ------------------------------------------------------------ 61762
chimp54546-135457.revcompl.Pan.troglodytes.clone.rp43-41g5.GenBank:AC099533.36      ------------------------------------------------------------ 72710
human.FAM30B-LOC105372935-GGT2.NCBI.                                                 ggatttggataaaatttataaatgtacaaactttgctaccaaggactgacacatttactt 108782
human.FAM230E-LOC105377182-GGT3P.NCBI.                                               ------------------------------------------------------------ 57310
human.FAM230J-LOC105372942-GGTLC5P.NCBI.rev.compl                                    ------------------------------------------------------------ 46364
human.FAM230A.ncRNA.18487127.18500594-GGTLC3.18516335.18518165.rev.compl.REF         ------------------------------------------------------------ 31039

chimp.LOC112206744-LOC107973052-GGT2.revcompl                                        ------------------------------------------------------------ 61762
chimp54546-135457.revcompl.Pan.troglodytes.clone.rp43-41g5.GenBank:AC099533.36      ------------------------------------------------------------ 72710
```

```
human.FAM30B-LOC105372935-GGT2.NCBI.                                               ttctagctcatcagtttatgtcctagaaaaattccagagccacattaaccagctgtttcc 108842
human.FAM230E-LOC105377182-GGT3P.NCBI.                                             ------------------------------------------------------------ 57310
human.FAM230J-LOC105372942-GGTLC5P.NCBI.rev.compl                                  ------------------------------------------------------------ 46364
human.FAM230A.ncRNA.18487127.18500594-GGTLC3.18516335.18518165.rev.compl.REF       ------------------------------------------------------------ 31039

chimp.LOC112206744-LOC107973052-GGT2.revcompl                                      ------------------------------------------------------------ 61762
chimp54546-135457.revcompl.Pan.troglodytes.clone.rp43-41g5.GenBank:AC099533.36     --------ATCCGACAATTTCTTCTGCTGCTTTGGCTACCATGAGAAACCACCTGAGTGA 72762
human.FAM30B-LOC105372935-GGT2.NCBI.                                               tatttttcatttacgatggtgtttccactttcacctgcaaagtgactaatagtaatacc 108902
human.FAM230E-LOC105377182-GGT3P.NCBI.                                             ------------------------------------------------------------ 57310
human.FAM230J-LOC105372942-GGTLC5P.NCBI.rev.compl                                  ------------------------------------------------------------ 46364
human.FAM230A.ncRNA.18487127.18500594-GGTLC3.18516335.18518165.rev.compl.REF       ------------------------------------------------------------ 31039

chimp.LOC112206744-LOC107973052-GGT2.revcompl                                      ------------------------------------------------------------ 61762
chimp54546-135457.revcompl.Pan.troglodytes.clone.rp43-41g5.GenBank:AC099533.36     ACTGAGACCAAAGTCATGCTCACTGCTTAGAGCAAGCTTGTCCAGCCTGCAGCCTGCAGG 72822
human.FAM30B-LOC105372935-GGT2.NCBI.                                               tattacagtaatttaagtaacaccatttagagtaatttcatctttagtactgtatttata 108962
human.FAM230E-LOC105377182-GGT3P.NCBI.                                             ------------------------------------------------------------ 57310
human.FAM230J-LOC105372942-GGTLC5P.NCBI.rev.compl                                  ------------------------------------------------------------ 46364
human.FAM230A.ncRNA.18487127.18500594-GGTLC3.18516335.18518165.rev.compl.REF       ------------------------------------------------------------ 31039

chimp.LOC112206744-LOC107973052-GGT2.revcompl                                      ------------------------------------------------------------ 61762
chimp54546-135457.revcompl.Pan.troglodytes.clone.rp43-41g5.GenBank:AC099533.36     CCAC-------------------------------------------------------- 72826
human.FAM30B-LOC105372935-GGT2.NCBI.                                               ctactaagaaagccgccaacgattcaagctgcactcactggtgaaaccctatgctatata 109022
human.FAM230E-LOC105377182-GGT3P.NCBI.                                             ------------------------------------------------------------ 57310
human.FAM230J-LOC105372942-GGTLC5P.NCBI.rev.compl                                  ------------------------------------------------------------ 46364
human.FAM230A.ncRNA.18487127.18500594-GGTLC3.18516335.18518165.rev.compl.REF       ------------------------------------------------------------ 31039

chimp.LOC112206744-LOC107973052-GGT2.revcompl                                      ------------------------------------------------------------ 61762
chimp54546-135457.revcompl.Pan.troglodytes.clone.rp43-41g5.GenBank:AC099533.36     ------------------------------------------------------------ 72826
human.FAM30B-LOC105372935-GGT2.NCBI.                                               tgtaattaatttatttcctaaattcttacctccctccacccccctccattggaaaataaac 109082
human.FAM230E-LOC105377182-GGT3P.NCBI.                                             ------------------------------------------------------------ 57310
human.FAM230J-LOC105372942-GGTLC5P.NCBI.rev.compl                                  ------------------------------------------------------------ 46364
human.FAM230A.ncRNA.18487127.18500594-GGTLC3.18516335.18518165.rev.compl.REF       ------------------------------------------------------------ 31039

chimp.LOC112206744-LOC107973052-GGT2.revcompl                                      ------------------------------------------------------------ 61762
chimp54546-135457.revcompl.Pan.troglodytes.clone.rp43-41g5.GenBank:AC099533.36     ------------------------------------------------------------ 72826
human.FAM30B-LOC105372935-GGT2.NCBI.                                               tctaagaatgcaaaagtttttgtgtgtttttcttcaacgcttagagcagtgcctggcacc 109142
human.FAM230E-LOC105377182-GGT3P.NCBI.                                             ------------------------------------------------------------ 57310
human.FAM230J-LOC105372942-GGTLC5P.NCBI.rev.compl                                  ------------------------------------------------------------ 46364
human.FAM230A.ncRNA.18487127.18500594-GGTLC3.18516335.18518165.rev.compl.REF       ------------------------------------------------------------ 31039

chimp.LOC112206744-LOC107973052-GGT2.revcompl                                      ------------------------------------------------------------ 61762
chimp54546-135457.revcompl.Pan.troglodytes.clone.rp43-41g5.GenBank:AC099533.36     ------------------------------------------------------------ 72826
human.FAM30B-LOC105372935-GGT2.NCBI.                                               taacagtcaatatctgctaactttgatgaatgaatggtatatttatattatgcaatatta 109202
human.FAM230E-LOC105377182-GGT3P.NCBI.                                             ------------------------------------------------------------ 57310
human.FAM230J-LOC105372942-GGTLC5P.NCBI.rev.compl                                  ------------------------------------------------------------ 46364
human.FAM230A.ncRNA.18487127.18500594-GGTLC3.18516335.18518165.rev.compl.REF       ------------------------------------------------------------ 31039

chimp.LOC112206744-LOC107973052-GGT2.revcompl                                      ------------------------------------------------------------ 61762
chimp54546-135457.revcompl.Pan.troglodytes.clone.rp43-41g5.GenBank:AC099533.36     --ATGCAGCCTAGGATGGCTTTGAAT----GTGGCCCCACATAAATTTGTAAACTTTCTT 72880
human.FAM30B-LOC105372935-GGT2.NCBI.                                               ctatgcagccatgaaacacaatgagttagagtggtaccagctaatttaatggaatttcca 109262
human.FAM230E-LOC105377182-GGT3P.NCBI.                                             ------------------------------------------------------------ 57310
human.FAM230J-LOC105372942-GGTLC5P.NCBI.rev.compl                                  ------------------------------------------------------------ 46364
human.FAM230A.ncRNA.18487127.18500594-GGTLC3.18516335.18518165.rev.compl.REF       ------------------------------------------------------------ 31039

chimp.LOC112206744-LOC107973052-GGT2.revcompl                                      ------------------------------------------------------------ 61762
chimp54546-135457.revcompl.Pan.troglodytes.clone.rp43-41g5.GenBank:AC099533.36     AAAACGTTATGAGAA--------------------------------------------- 72895
human.FAM30B-LOC105372935-GGT2.NCBI.                                               gaagtatgaaattaagaaaacaagaagaaaagtgtcaaaaatatcatcccactttttttt 109322
human.FAM230E-LOC105377182-GGT3P.NCBI.                                             ------------------------------------------------------------ 57310
human.FAM230J-LOC105372942-GGTLC5P.NCBI.rev.compl                                  ------------------------------------------------------------ 46364
human.FAM230A.ncRNA.18487127.18500594-GGTLC3.18516335.18518165.rev.compl.REF       ------------------------------------------------------------ 31039

chimp.LOC112206744-LOC107973052-GGT2.revcompl                                      ------------------------------------------------------------ 61762
chimp54546-135457.revcompl.Pan.troglodytes.clone.rp43-41g5.GenBank:AC099533.36     ------------------------------------------------------------ 72895
human.FAM30B-LOC105372935-GGT2.NCBI.                                               tttcttccctgaaacggagtcgtgctctgacacccaggctacagtatagtgctgccatca 109382
human.FAM230E-LOC105377182-GGT3P.NCBI.                                             ------------------------------------------------------------ 57310
human.FAM230J-LOC105372942-GGTLC5P.NCBI.rev.compl                                  ------------------------------------------------------------ 46364
human.FAM230A.ncRNA.18487127.18500594-GGTLC3.18516335.18518165.rev.compl.REF       ------------------------------------------------------------ 31039

chimp.LOC112206744-LOC107973052-GGT2.revcompl                                      ------------------------------------------------------------ 61762
chimp54546-135457.revcompl.Pan.troglodytes.clone.rp43-41g5.GenBank:AC099533.36     ---------------------------ATTTTTGCAATTTTTTAAGCTCATCAGCTATTG 72928
human.FAM30B-LOC105372935-GGT2.NCBI.                                               tggctcactgcaacctcaaccttctgggctcagggaatccttccacctcagcctcccatg 109442
human.FAM230E-LOC105377182-GGT3P.NCBI.                                             ------------------------------------------------------------ 57310
human.FAM230J-LOC105372942-GGTLC5P.NCBI.rev.compl                                  ------------------------------------------------------------ 46364
human.FAM230A.ncRNA.18487127.18500594-GGTLC3.18516335.18518165.rev.compl.REF       ------------------------------------------------------------ 31039

chimp.LOC112206744-LOC107973052-GGT2.revcompl                                      ------------------------------------------------------------ 61762
chimp54546-135457.revcompl.Pan.troglodytes.clone.rp43-41g5.GenBank:AC099533.36     TTAGTGTTAATATATTTATGTGTGGCCCAAGACAATTTTTCTTCTTCAAA--------TG 72980
human.FAM30B-LOC105372935-GGT2.NCBI.                                               tagctgggactacaggtgtgtgccacaacgcccagctaatttattttaaattattttttg 109502
human.FAM230E-LOC105377182-GGT3P.NCBI.                                             ------------------------------------------------------------ 57310
human.FAM230J-LOC105372942-GGTLC5P.NCBI.rev.compl                                  ------------------------------------------------------------ 46364
human.FAM230A.ncRNA.18487127.18500594-GGTLC3.18516335.18518165.rev.compl.REF       ------------------------------------------------------------ 31039

chimp.LOC112206744-LOC107973052-GGT2.revcompl                                      ------------------------------------------------------------ 61762
chimp54546-135457.revcompl.Pan.troglodytes.clone.rp43-41g5.GenBank:AC099533.36     TCGCCCAGGGAAGCCAAAATGTTGGACACCCTGGCTTAGAGGGGATGTGAGATATTGAGC 73040
human.FAM30B-LOC105372935-GGT2.NCBI.                                               tagagacagggtctccctatgttgcccaggctggtctcaaactactaggctcaagtgatc 109562
human.FAM230E-LOC105377182-GGT3P.NCBI.                                             ------------------------------------------------------------ 57310
human.FAM230J-LOC105372942-GGTLC5P.NCBI.rev.compl                                  ------------------------------------------------------------ 46364
human.FAM230A.ncRNA.18487127.18500594-GGTLC3.18516335.18518165.rev.compl.REF       ------------------------------------------------------------ 31039

chimp.LOC112206744-LOC107973052-GGT2.revcompl                                      ------------------------------------------------------------ 61762
chimp54546-135457.revcompl.Pan.troglodytes.clone.rp43-41g5.GenBank:AC099533.36     CTAGTTATATTAGAGGTTCCTGTCCCACATCCCCATCTGTCTGTTTCAC---TGTGAGCC 73097
human.FAM30B-LOC105372935-GGT2.NCBI.                                               ctc---------------ccacctcagcttcccaaagtgttgggattacaggtgtgagcc 109607
human.FAM230E-LOC105377182-GGT3P.NCBI.                                             ------------------------------------------------------------ 57310
human.FAM230J-LOC105372942-GGTLC5P.NCBI.rev.compl                                  ------------------------------------------------------------ 46364
human.FAM230A.ncRNA.18487127.18500594-GGTLC3.18516335.18518165.rev.compl.REF       ------------------------------------------------------------ 31039

chimp.LOC112206744-LOC107973052-GGT2.revcompl                                      ------------------------------------------------------------ 61762
chimp54546-135457.revcompl.Pan.troglodytes.clone.rp43-41g5.GenBank:AC099533.36     ACTGCCTGCTGCTCCCTCTGCCATCATAGGTGCTACAG--------TAATAGCCTTGTCC 73149
human.FAM30B-LOC105372935-GGT2.NCBI.                                               actgtgcccagcccaatcccacttttttaaaaagtataccccccaaatccatacactggtga 109667
human.FAM230E-LOC105377182-GGT3P.NCBI.                                             ------------------------------------------------------------ 57310
human.FAM230J-LOC105372942-GGTLC5P.NCBI.rev.compl                                  ------------------------------------------------------------ 46364
human.FAM230A.ncRNA.18487127.18500594-GGTLC3.18516335.18518165.rev.compl.REF       ------------------------------------------------------------ 31039

chimp.LOC112206744-LOC107973052-GGT2.revcompl                                      ------------------------------------------------------------ 61762
chimp54546-135457.revcompl.Pan.troglodytes.clone.rp43-41g5.GenBank:AC099533.36     CTGGCCTGGGCCCCACTGATGGTCAGAGTGGCCGTGTTCCACAAGTTGGAGCCAGAGAAT 73209
human.FAM30B-LOC105372935-GGT2.NCBI.                                               ggaagtggagaacgtggaatcctcacacacagaatgtaaaatggtacagctctggagaac 109727
human.FAM230E-LOC105377182-GGT3P.NCBI.                                             ------------------------------------------------------------ 57310
human.FAM230J-LOC105372942-GGTLC5P.NCBI.rev.compl                                  ------------------------------------------------------------ 46364
human.FAM230A.ncRNA.18487127.18500594-GGTLC3.18516335.18518165.rev.compl.REF       ------------------------------------------------------------ 31039

chimp.LOC112206744-LOC107973052-GGT2.revcompl                                      ------------------------------------------------------------ 61762
chimp54546-135457.revcompl.Pan.troglodytes.clone.rp43-41g5.GenBank:AC099533.36     CACTCAGGGGTCCTTGAGGGCCACTTGCTGTCTTTATAGATGACCGGCACAGGGGTCTGG 73269
human.FAM30B-LOC105372935-GGT2.NCBI.                                               attttagcagtttctcaaaatgtttaacgtagctactacacgacccagcaattcaactcc 109787
human.FAM230E-LOC105377182-GGT3P.NCBI.                                             ------------------------------------------------------------ 57310
human.FAM230J-LOC105372942-GGTLC5P.NCBI.rev.compl                                  ------------------------------------------------------------ 46364
human.FAM230A.ncRNA.18487127.18500594-GGTLC3.18516335.18518165.rev.compl.REF       ------------------------------------------------------------ 31039

chimp.LOC112206744-LOC107973052-GGT2.revcompl                                      ------------------------------------------------------------ 61762
chimp54546-135457.revcompl.Pan.troglodytes.clone.rp43-41g5.GenBank:AC099533.36     CCTGACTTCCGCTGGTACCAATAAACATATCCACCTCCAACAGTATTTCCACAGCAGGTG 73329
human.FAM30B-LOC105372935-GGT2.NCBI.                                               cagatatacacctgagagaactgaaaggatatgtccacacaaaaatttgcagaggaactt 109847
human.FAM230E-LOC105377182-GGT3P.NCBI.                                             ------------------------------------------------------------ 57310
human.FAM230J-LOC105372942-GGTLC5P.NCBI.rev.compl                                  ------------------------------------------------------------ 46364
human.FAM230A.ncRNA.18487127.18500594-GGTLC3.18516335.18518165.rev.compl.REF       ------------------------------------------------------------ 31039

chimp.LOC112206744-LOC107973052-GGT2.revcompl                                      ------------------------------------------------------------ 61762
chimp54546-135457.revcompl.Pan.troglodytes.clone.rp43-41g5.GenBank:AC099533.36     ATCCTGGC---------------------------------------------------- 73337
human.FAM30B-LOC105372935-GGT2.NCBI.                                               gtacacacttgttcacagtagcattattcataatagcccaaagtggcaaaaacgcaaatg 109907
```

```
human.FAM230E-LOC105377182-GGT3P.NCBI.                                             ------------------------------------------------------------ 57310
human.FAM230J-LOC105372942-GGTLC5P.NCBI.rev.compl                                  ------------------------------------------------------------ 46364
human.FAM230A.ncRNA.18487127.18500594-GGTLC3.18516335.18518165.rev.compl.REF       ------------------------------------------------------------ 31039


chimp.LOC112206744-LOC107973052-GGT2.revcompl                                      ------------------------------------------------------------ 61762
chimp54546-135457.revcompl.Pan.troglodytes.clone.rp43-41g5.GenBank:AC099533.36     ------------------------------------------------------------ 73337
human.FAM30B-LOC105372935-GGT2.NCBI.                                               ttcatcaactgatggacaaataaagcatgaacaactgatggacaaataaagtatatcatt 109967
human.FAM230E-LOC105377182-GGT3P.NCBI.                                             ------------------------------------------------------------ 57310
human.FAM230J-LOC105372942-GGTLC5P.NCBI.rev.compl                                  ------------------------------------------------------------ 46364
human.FAM230A.ncRNA.18487127.18500594-GGTLC3.18516335.18518165.rev.compl.REF       ------------------------------------------------------------ 31039


chimp.LOC112206744-LOC107973052-GGT2.revcompl                                      ------------------------------------------------------------ 61762
chimp54546-135457.revcompl.Pan.troglodytes.clone.rp43-41g5.GenBank:AC099533.36     ------------------------------------------------------------ 73337
human.FAM30B-LOC105372935-GGT2.NCBI.                                               cagcatataagaaatgaagtgctaatacatgctacaacaggaatagaccttaataacatt 110027
human.FAM230E-LOC105377182-GGT3P.NCBI.                                             ------------------------------------------------------------ 57310
human.FAM230J-LOC105372942-GGTLC5P.NCBI.rev.compl                                  ------------------------------------------------------------ 46364
human.FAM230A.ncRNA.18487127.18500594-GGTLC3.18516335.18518165.rev.compl.REF       ------------------------------------------------------------ 31039


chimp.LOC112206744-LOC107973052-GGT2.revcompl                                      ------------------------------------------------------------ 61762
chimp54546-135457.revcompl.Pan.troglodytes.clone.rp43-41g5.GenBank:AC099533.36     ------------------------------------------------------------ 73337
human.FAM30B-LOC105372935-GGT2.NCBI.                                               ttgctaaatgaaaaaagccaaacaaaaggtcacatgttgtatgattctatttacataaaa 110087
human.FAM230E-LOC105377182-GGT3P.NCBI.                                             ------------------------------------------------------------ 57310
human.FAM230J-LOC105372942-GGTLC5P.NCBI.rev.compl                                  ------------------------------------------------------------ 46364
human.FAM230A.ncRNA.18487127.18500594-GGTLC3.18516335.18518165.rev.compl.REF       ------------------------------------------------------------ 31039


chimp.LOC112206744-LOC107973052-GGT2.revcompl                                      ------------------------------------------------------------ 61762
chimp54546-135457.revcompl.Pan.troglodytes.clone.rp43-41g5.GenBank:AC099533.36     ------------------------------------------------------------ 73337
human.FAM30B-LOC105372935-GGT2.NCBI.                                               atacctagactaggcaaatccatagagacacaaagatcagtgactgcccagagccagagg 110147
human.FAM230E-LOC105377182-GGT3P.NCBI.                                             ------------------------------------------------------------ 57310
human.FAM230J-LOC105372942-GGTLC5P.NCBI.rev.compl                                  ------------------------------------------------------------ 46364
human.FAM230A.ncRNA.18487127.18500594-GGTLC3.18516335.18518165.rev.compl.REF       ------------------------------------------------------------ 31039


chimp.LOC112206744-LOC107973052-GGT2.revcompl                                      ------------------------------------------------------------ 61762
chimp54546-135457.revcompl.Pan.troglodytes.clone.rp43-41g5.GenBank:AC099533.36     ------------------------------------------------------------ 73337
human.FAM30B-LOC105372935-GGT2.NCBI.                                               gagtggtgaatgtgaaatggctgctaatgagtacagacttctttttgaggtattgaacat 110207
human.FAM230E-LOC105377182-GGT3P.NCBI.                                             ------------------------------------------------------------ 57310
human.FAM230J-LOC105372942-GGTLC5P.NCBI.rev.compl                                  ------------------------------------------------------------ 46364
human.FAM230A.ncRNA.18487127.18500594-GGTLC3.18516335.18518165.rev.compl.REF       ------------------------------------------------------------ 31039


chimp.LOC112206744-LOC107973052-GGT2.revcompl                                      ------------------------------------------------------------ 61762
chimp54546-135457.revcompl.Pan.troglodytes.clone.rp43-41g5.GenBank:AC099533.36     ------------------------------------------------------------ 73337
human.FAM30B-LOC105372935-GGT2.NCBI.                                               gttatggaatcagatggtggtgatcaatgcataactttgtgaatataccaaaaaccacat 110267
human.FAM230E-LOC105377182-GGT3P.NCBI.                                             ------------------------------------------------------------ 57310
human.FAM230J-LOC105372942-GGTLC5P.NCBI.rev.compl                                  ------------------------------------------------------------ 46364
human.FAM230A.ncRNA.18487127.18500594-GGTLC3.18516335.18518165.rev.compl.REF       ------------------------------------------------------------ 31039


chimp.LOC112206744-LOC107973052-GGT2.revcompl                                      ------------------------------------------------------------ 61762
chimp54546-135457.revcompl.Pan.troglodytes.clone.rp43-41g5.GenBank:AC099533.36     ------------------------------------------------------------ 73337
human.FAM30B-LOC105372935-GGT2.NCBI.                                               gaaaggtacactttaaaagcctgaattttaaggtatgtgaattgtatctccataaacctg 110327
human.FAM230E-LOC105377182-GGT3P.NCBI.                                             ------------------------------------------------------------ 57310
human.FAM230J-LOC105372942-GGTLC5P.NCBI.rev.compl                                  ------------------------------------------------------------ 46364
human.FAM230A.ncRNA.18487127.18500594-GGTLC3.18516335.18518165.rev.compl.REF       ------------------------------------------------------------ 31039


chimp.LOC112206744-LOC107973052-GGT2.revcompl                                      ------------------------------------------------------------ 61762
chimp54546-135457.revcompl.Pan.troglodytes.clone.rp43-41g5.GenBank:AC099533.36     ------------------------------------------------------------ 73337
human.FAM30B-LOC105372935-GGT2.NCBI.                                               tgattttgttaatatacgcttatttgcaaatgaatacatggataatgaggaataatatga 110387
human.FAM230E-LOC105377182-GGT3P.NCBI.                                             ------------------------------------------------------------ 57310
human.FAM230J-LOC105372942-GGTLC5P.NCBI.rev.compl                                  ------------------------------------------------------------ 46364
human.FAM230A.ncRNA.18487127.18500594-GGTLC3.18516335.18518165.rev.compl.REF       ------------------------------------------------------------ 31039


chimp.LOC112206744-LOC107973052-GGT2.revcompl                                      ------------------------------------------------------------ 61762
chimp54546-135457.revcompl.Pan.troglodytes.clone.rp43-41g5.GenBank:AC099533.36     ------------------------------------------------------------ 73337
human.FAM30B-LOC105372935-GGT2.NCBI.                                               aaggttaatgacaaccttggtgatagggtacaggagactgggagaggaagataagtgttg 110447
human.FAM230E-LOC105377182-GGT3P.NCBI.                                             ------------------------------------------------------------ 57310
human.FAM230J-LOC105372942-GGTLC5P.NCBI.rev.compl                                  ------------------------------------------------------------ 46364
human.FAM230A.ncRNA.18487127.18500594-GGTLC3.18516335.18518165.rev.compl.REF       ------------------------------------------------------------ 31039


chimp.LOC112206744-LOC107973052-GGT2.revcompl                                      ------------------------------------------------------------ 61762
chimp54546-135457.revcompl.Pan.troglodytes.clone.rp43-41g5.GenBank:AC099533.36     ------------------------------------------------------------ 73337
human.FAM30B-LOC105372935-GGT2.NCBI.                                               aggtagaagaagacaaaaaataaaaggctaaaacaataatccagggccagaggccatgag 110507
human.FAM230E-LOC105377182-GGT3P.NCBI.                                             ------------------------------------------------------------ 57310
human.FAM230J-LOC105372942-GGTLC5P.NCBI.rev.compl                                  ------------------------------------------------------------ 46364
human.FAM230A.ncRNA.18487127.18500594-GGTLC3.18516335.18518165.rev.compl.REF       ------------------------------------------------------------ 31039


chimp.LOC112206744-LOC107973052-GGT2.revcompl                                      ------------------------------------------------------------ 61762
chimp54546-135457.revcompl.Pan.troglodytes.clone.rp43-41g5.GenBank:AC099533.36     ------------------------------------------------------------ 73337
human.FAM30B-LOC105372935-GGT2.NCBI.                                               ttgggagatggagcaagacgaagtgtacaaagaaatgagcataaatcataaaaatgaggg 110567
human.FAM230E-LOC105377182-GGT3P.NCBI.                                             ------------------------------------------------------------ 57310
human.FAM230J-LOC105372942-GGTLC5P.NCBI.rev.compl                                  ------------------------------------------------------------ 46364
human.FAM230A.ncRNA.18487127.18500594-GGTLC3.18516335.18518165.rev.compl.REF       ------------------------------------------------------------ 31039


chimp.LOC112206744-LOC107973052-GGT2.revcompl                                      ------------------------------------------------------------ 61762
chimp54546-135457.revcompl.Pan.troglodytes.clone.rp43-41g5.GenBank:AC099533.36     --------------------------CATCTGTAATGGGGCCACTGACACTGAGGGTGGC 73371
human.FAM30B-LOC105372935-GGT2.NCBI.                                               aactagggttgaaaactccagcaagtagtttgtaatggggagatgaagcaaacctaagt 110627
human.FAM230E-LOC105377182-GGT3P.NCBI.                                             ------------------------------------------------------------ 57310
human.FAM230J-LOC105372942-GGTLC5P.NCBI.rev.compl                                  ------------------------------------------------------------ 46364
human.FAM230A.ncRNA.18487127.18500594-GGTLC3.18516335.18518165.rev.compl.REF       ------------------------------------------------------------ 31039


chimp.LOC112206744-LOC107973052-GGT2.revcompl                                      ------------------------------------------------------------ 61762
chimp54546-135457.revcompl.Pan.troglodytes.clone.rp43-41g5.GenBank:AC099533.36     TGAGACAGCTCATATGTGGCCATGAAACCTGCAGGAGATGAGGGAGAAAAGGCTTGAAGC 73431
human.FAM30B-LOC105372935-GGT2.NCBI.                                               aactgcaatgattcaacggaatttgcagaaaatgcagaagggcgagaaaagcaatgaaaa 110687
human.FAM230E-LOC105377182-GGT3P.NCBI.                                             ------------------------------------------------------------ 57310
human.FAM230J-LOC105372942-GGTLC5P.NCBI.rev.compl                                  ------------------------------------------------------------ 46364
human.FAM230A.ncRNA.18487127.18500594-GGTLC3.18516335.18518165.rev.compl.REF       ------------------------------------------------------------ 31039


chimp.LOC112206744-LOC107973052-GGT2.revcompl                                      ------------------------------------------------------------ 61762
chimp54546-135457.revcompl.Pan.troglodytes.clone.rp43-41g5.GenBank:AC099533.36     TGAGGAGCTGA------------------------------------------------- 73442
human.FAM30B-LOC105372935-GGT2.NCBI.                                               aatggatcagaagatttaaaattggctctaccccaccaaagttttaaaaagcaagcaaga 110747
human.FAM230E-LOC105377182-GGT3P.NCBI.                                             ------------------------------------------------------------ 57310
human.FAM230J-LOC105372942-GGTLC5P.NCBI.rev.compl                                  ------------------------------------------------------------ 46364
human.FAM230A.ncRNA.18487127.18500594-GGTLC3.18516335.18518165.rev.compl.REF       ------------------------------------------------------------ 31039


chimp.LOC112206744-LOC107973052-GGT2.revcompl                                      ------------------------------------------------------------ 61762
chimp54546-135457.revcompl.Pan.troglodytes.clone.rp43-41g5.GenBank:AC099533.36     ------------------------------------------------------------ 73442
human.FAM30B-LOC105372935-GGT2.NCBI.                                               agaaaattcagttgctcctgggcaagaattcttgggcccagtggggtagaaccaaaccat 110807
human.FAM230E-LOC105377182-GGT3P.NCBI.                                             ------------------------------------------------------------ 57310
human.FAM230J-LOC105372942-GGTLC5P.NCBI.rev.compl                                  ------------------------------------------------------------ 46364
human.FAM230A.ncRNA.18487127.18500594-GGTLC3.18516335.18518165.rev.compl.REF       ------------------------------------------------------------ 31039


chimp.LOC112206744-LOC107973052-GGT2.revcompl                                      ------------------------------------------------------------ 61762
chimp54546-135457.revcompl.Pan.troglodytes.clone.rp43-41g5.GenBank:AC099533.36     ------------------------------------------------------------ 73442
human.FAM30B-LOC105372935-GGT2.NCBI.                                               gataaggagacaccagtgttttgtaagcaagagaatatattaaggagagaaaaggggcat 110867
human.FAM230E-LOC105377182-GGT3P.NCBI.                                             ------------------------------------------------------------ 57310
human.FAM230J-LOC105372942-GGTLC5P.NCBI.rev.compl                                  ------------------------------------------------------------ 46364
human.FAM230A.ncRNA.18487127.18500594-GGTLC3.18516335.18518165.rev.compl.REF       ------------------------------------------------------------ 31039


chimp.LOC112206744-LOC107973052-GGT2.revcompl                                      ------------------------------------------------------------ 61762
chimp54546-135457.revcompl.Pan.troglodytes.clone.rp43-41g5.GenBank:AC099533.36     ----------------------------TCCTCAGGATTCCTTTGCACCTGCCTGGCTGG 73474
human.FAM30B-LOC105372935-GGT2.NCBI.                                               ttattgggagaaaaggtctcactgtgattgttctggaataaagtagccccatcggactgc 110927
human.FAM230E-LOC105377182-GGT3P.NCBI.                                             ------------------------------------------------------------ 57310
human.FAM230J-LOC105372942-GGTLC5P.NCBI.rev.compl                                  ------------------------------------------------------------ 46364
human.FAM230A.ncRNA.18487127.18500594-GGTLC3.18516335.18518165.rev.compl.REF       ------------------------------------------------------------ 31039


chimp.LOC112206744-LOC107973052-GGT2.revcompl                                      ------------------------------------------------------------ 61762
chimp54546-135457.revcompl.Pan.troglodytes.clone.rp43-41g5.GenBank:AC099533.36     GACAGAGCTTCTGGGATGAAAGGGAGC--------------------------------- 73501
human.FAM30B-LOC105372935-GGT2.NCBI.                                               ctcaggaactcatggtggaagaggtgccactggaaaatggctaagtcgggatcttcccca 110987
human.FAM230E-LOC105377182-GGT3P.NCBI.                                             ------------------------------------------------------------ 57310
```

```
human.FAM230J-LOC105372942-GGTLC5P.NCBI.rev.compl                                      ------------------------------------------------------------ 46364
human.FAM230A.ncRNA.18487127.18500594-GGTLC3.18516335.18518165.rev.compl.REF           ------------------------------------------------------------ 31039

chimp.LOC112206744-LOC107973052-GGT2.revcompl                                          ------------------------------------------------------------ 61762
chimp54546-135457.revcompl.Pan.troglodytes.clone.rp43-41g5.GenBank:AC099533.36         ------------------------------------------------------------ 73501
human.FAM30B-LOC105372935-GGT2.NCBI.                                                   agaacactagaagagacagaggaagaatagaagagctttaaattttagcagatatttctt 111047
human.FAM230E-LOC105377182-GGT3P.NCBI.                                                 ------------------------------------------------------------ 57310
human.FAM230J-LOC105372942-GGTLC5P.NCBI.rev.compl                                      ------------------------------------------------------------ 46364
human.FAM230A.ncRNA.18487127.18500594-GGTLC3.18516335.18518165.rev.compl.REF           ------------------------------------------------------------ 31039

chimp.LOC112206744-LOC107973052-GGT2.revcompl                                          ------------------------------------------------------------ 61762
chimp54546-135457.revcompl.Pan.troglodytes.clone.rp43-41g5.GenBank:AC099533.36         ------------------------------------------------------CTGACT 73507
human.FAM30B-LOC105372935-GGT2.NCBI.                                                   tttcttgtttaattaaaataaatcttattgacctaatattttgagactgggctcttgca 111107
human.FAM230E-LOC105377182-GGT3P.NCBI.                                                 ------------------------------------------------------------ 57310
human.FAM230J-LOC105372942-GGTLC5P.NCBI.rev.compl                                      ------------------------------------------------------------ 46364
human.FAM230A.ncRNA.18487127.18500594-GGTLC3.18516335.18518165.rev.compl.REF           ------------------------------------------------------------ 31039

chimp.LOC112206744-LOC107973052-GGT2.revcompl                                          ------------------------------------------------------------ 61762
chimp54546-135457.revcompl.Pan.troglodytes.clone.rp43-41g5.GenBank:AC099533.36         GTGCCCCTCAGAGTGCCCATGAGCACAGGAGTACAGGAAGATTTAACCTTACCAGCCCCT 73567
human.FAM30B-LOC105372935-GGT2.NCBI.                                                   atgctccccagactgaactcaaactcctaggctcaagagattcttccacttcagcctcct 111167
human.FAM230E-LOC105377182-GGT3P.NCBI.                                                 ------------------------------------------------------------ 57310
human.FAM230J-LOC105372942-GGTLC5P.NCBI.rev.compl                                      ------------------------------------------------------------ 46364
human.FAM230A.ncRNA.18487127.18500594-GGTLC3.18516335.18518165.rev.compl.REF           ------------------------------------------------------------ 31039

chimp.LOC112206744-LOC107973052-GGT2.revcompl                                          ------------------------------------------------------------ 61762
chimp54546-135457.revcompl.Pan.troglodytes.clone.rp43-41g5.GenBank:AC099533.36         CACCCTGCAG-------------------------------------------------- 73577
human.FAM30B-LOC105372935-GGT2.NCBI.                                                   gaaaagctggcactacaggtgcacaccactgggcccagcataatggctatttcttgacaa 111227
human.FAM230E-LOC105377182-GGT3P.NCBI.                                                 ------------------------------------------------------------ 57310
human.FAM230J-LOC105372942-GGTLC5P.NCBI.rev.compl                                      ------------------------------------------------------------ 46364
human.FAM230A.ncRNA.18487127.18500594-GGTLC3.18516335.18518165.rev.compl.REF           ------------------------------------------------------------ 31039

chimp.LOC112206744-LOC107973052-GGT2.revcompl                                          ------------------------------------------------------------ 61762
chimp54546-135457.revcompl.Pan.troglodytes.clone.rp43-41g5.GenBank:AC099533.36         -------CAAGGTCATGAGCATCCTTTCCACCACAGGCAGAGCCCTGCT---GAGCTCAC 73627
human.FAM30B-LOC105372935-GGT2.NCBI.                                                   atcttgacaatgccaccagtatgtttttctttagagacagagtctcgctttgtagcccag 111287
human.FAM230E-LOC105377182-GGT3P.NCBI.                                                 ------------------------------------------------------------ 57310
human.FAM230J-LOC105372942-GGTLC5P.NCBI.rev.compl                                      ------------------------------------------------------------ 46364
human.FAM230A.ncRNA.18487127.18500594-GGTLC3.18516335.18518165.rev.compl.REF           ------------------------------------------------------------ 31039

chimp.LOC112206744-LOC107973052-GGT2.revcompl                                          ------------------------------------------------------------ 61762
chimp54546-135457.revcompl.Pan.troglodytes.clone.rp43-41g5.GenBank:AC099533.36         AATCAAGGCCAG-GCTGGTCCCACAGCTCGGGGCTGGGCGGGTGGCTGGGACTCTGGAGG 73686
human.FAM30B-LOC105372935-GGT2.NCBI.                                                   gctggagaacagtggtgtgatcctggctcactgcagcctcaaacacctggactcaggaga 111347
human.FAM230E-LOC105377182-GGT3P.NCBI.                                                 ------------------------------------------------------------ 57310
human.FAM230J-LOC105372942-GGTLC5P.NCBI.rev.compl                                      ------------------------------------------------------------ 46364
human.FAM230A.ncRNA.18487127.18500594-GGTLC3.18516335.18518165.rev.compl.REF           ------------------------------------------------------------ 31039

chimp.LOC112206744-LOC107973052-GGT2.revcompl                                          ------------------------------------------------------------ 61762
chimp54546-135457.revcompl.Pan.troglodytes.clone.rp43-41g5.GenBank:AC099533.36         CAGCACCTGTGCAGTGAGCGAGGAGGCTGAGAAAGGTCCAGTCCATGACTGAGACATCCC 73746
human.FAM30B-LOC105372935-GGT2.NCBI.                                                   tccccctgcctcagcctctcaggtagctgaga---------------------------- 111379
human.FAM230E-LOC105377182-GGT3P.NCBI.                                                 ------------------------------------------------------------ 57310
human.FAM230J-LOC105372942-GGTLC5P.NCBI.rev.compl                                      ------------------------------------------------------------ 46364
human.FAM230A.ncRNA.18487127.18500594-GGTLC3.18516335.18518165.rev.compl.REF           ------------------------------------------------------------ 31039

chimp.LOC112206744-LOC107973052-GGT2.revcompl                                          ------------------------------------------------------------ 61762
chimp54546-135457.revcompl.Pan.troglodytes.clone.rp43-41g5.GenBank:AC099533.36         TGATGCTGCTTCCTGTGGCCCAGGGCGGGTAGGTTTCTCCCAGGCCTCTCTTATCCCCTT 73806
human.FAM30B-LOC105372935-GGT2.NCBI.                                                   ------------------------------------------------------------ 111379
human.FAM230E-LOC105377182-GGT3P.NCBI.                                                 ------------------------------------------------------------ 57310
human.FAM230J-LOC105372942-GGTLC5P.NCBI.rev.compl                                      ------------------------------------------------------------ 46364
human.FAM230A.ncRNA.18487127.18500594-GGTLC3.18516335.18518165.rev.compl.REF           ------------------------------------------------------------ 31039

chimp.LOC112206744-LOC107973052-GGT2.revcompl                                          ------------------------------------------------------------ 61762
chimp54546-135457.revcompl.Pan.troglodytes.clone.rp43-41g5.GenBank:AC099533.36         GATGGAGAAAGCGGCCCGTGATGCAAGTCAGCAGAATCAGGGGCTGCTACTGGAGTAGCT 73866
human.FAM30B-LOC105372935-GGT2.NCBI.                                                   --------------ctacaggtgcgcaccaccacacttggcgaattttattttatttttt 111425
human.FAM230E-LOC105377182-GGT3P.NCBI.                                                 ------------------------------------------------------------ 57310
human.FAM230J-LOC105372942-GGTLC5P.NCBI.rev.compl                                      ------------------------------------------------------------ 46364
human.FAM230A.ncRNA.18487127.18500594-GGTLC3.18516335.18518165.rev.compl.REF           ------------------------------------------------------------ 31039

chimp.LOC112206744-LOC107973052-GGT2.revcompl                                          ------------------------------------------------------------ 61762
chimp54546-135457.revcompl.Pan.troglodytes.clone.rp43-41g5.GenBank:AC099533.36         ----TTGTAGTTTCCAGAGAAGGGCTCAGCCCCAAGGGACACAGAGAGGGGAGGGGAGGA 73922
human.FAM30B-LOC105372935-GGT2.NCBI.                                                   tttattatactttaagttctagggtacatacacctggctaattataaaatgtttatctgt 111485
human.FAM230E-LOC105377182-GGT3P.NCBI.                                                 ------------------------------------------------------------ 57310
human.FAM230J-LOC105372942-GGTLC5P.NCBI.rev.compl                                      ------------------------------------------------------------ 46364
human.FAM230A.ncRNA.18487127.18500594-GGTLC3.18516335.18518165.rev.compl.REF           ------------------------------------------------------------ 31039

chimp.LOC112206744-LOC107973052-GGT2.revcompl                                          ------------------------------------------------------------ 61762
chimp54546-135457.revcompl.Pan.troglodytes.clone.rp43-41g5.GenBank:AC099533.36         CCCTACAGATCCACCT-------------------------------------------- 73938
human.FAM30B-LOC105372935-GGT2.NCBI.                                                   acagacaggttctcgttatgttgcccaggctggtctcgaactctcggcctcaagcaatcc 111545
human.FAM230E-LOC105377182-GGT3P.NCBI.                                                 ------------------------------------------------------------ 57310
human.FAM230J-LOC105372942-GGTLC5P.NCBI.rev.compl                                      ------------------------------------------------------------ 46364
human.FAM230A.ncRNA.18487127.18500594-GGTLC3.18516335.18518165.rev.compl.REF           ------------------------------------------------------------ 31039

chimp.LOC112206744-LOC107973052-GGT2.revcompl                                          ------------------------------------------------------------ 61762
chimp54546-135457.revcompl.Pan.troglodytes.clone.rp43-41g5.GenBank:AC099533.36         -----------------------------------------CAGCCCACAGAATTCTCCT 73957
human.FAM30B-LOC105372935-GGT2.NCBI.                                                   tctcctcctcaccttccaaagccccgaaatatgcccacatccagcctgccaccagtttct 111605
human.FAM230E-LOC105377182-GGT3P.NCBI.                                                 ------------------------------------------------------------ 57310
human.FAM230J-LOC105372942-GGTLC5P.NCBI.rev.compl                                      ------------------------------------------------------------ 46364
human.FAM230A.ncRNA.18487127.18500594-GGTLC3.18516335.18518165.rev.compl.REF           ------------------------------------------------------------ 31039

chimp.LOC112206744-LOC107973052-GGT2.revcompl                                          ------------------------------------------------------------ 61762
chimp54546-135457.revcompl.Pan.troglodytes.clone.rp43-41g5.GenBank:AC099533.36         TCACTTGCTGGTCCCAAAGTCTGGGCCAACATCTTTACCTTAAACTATGTCTTGGCCTCG 74017
human.FAM30B-LOC105372935-GGT2.NCBI.                                                   ttgcatacttctttcctaggcttgaccaagacctttactttcttctgtgtacagctcagc 111665
human.FAM230E-LOC105377182-GGT3P.NCBI.                                                 ------------------------------------------------------------ 57310
human.FAM230J-LOC105372942-GGTLC5P.NCBI.rev.compl                                      ------------------------------------------------------------ 46364
human.FAM230A.ncRNA.18487127.18500594-GGTLC3.18516335.18518165.rev.compl.REF           ------------------------------------------------------------ 31039

chimp.LOC112206744-LOC107973052-GGT2.revcompl                                          ------------------------------------------------------------ 61762
chimp54546-135457.revcompl.Pan.troglodytes.clone.rp43-41g5.GenBank:AC099533.36         GTT--------------------------------------------------------- 74020
human.FAM30B-LOC105372935-GGT2.NCBI.                                                   attgaaaaaaaacaaaaaagtttaggctgggcacaatgggtcacgcctgtaatcccaaca 111725
human.FAM230E-LOC105377182-GGT3P.NCBI.                                                 ------------------------------------------------------------ 57310
human.FAM230J-LOC105372942-GGTLC5P.NCBI.rev.compl                                      ------------------------------------------------------------ 46364
human.FAM230A.ncRNA.18487127.18500594-GGTLC3.18516335.18518165.rev.compl.REF           ------------------------------------------------------------ 31039

chimp.LOC112206744-LOC107973052-GGT2.revcompl                                          ------------------------------------------------------------ 61762
chimp54546-135457.revcompl.Pan.troglodytes.clone.rp43-41g5.GenBank:AC099533.36         --------------------------------------------CTCAGCTAGCCAGGCC 74036
human.FAM30B-LOC105372935-GGT2.NCBI.                                                   cttagggtggccaaggtgggaggtttgcttgaacccaggagttccacaccagcctaggca 111785
human.FAM230E-LOC105377182-GGT3P.NCBI.                                                 ------------------------------------------------------------ 57310
human.FAM230J-LOC105372942-GGTLC5P.NCBI.rev.compl                                      ------------------------------------------------------------ 46364
human.FAM230A.ncRNA.18487127.18500594-GGTLC3.18516335.18518165.rev.compl.REF           ------------------------------------------------------------ 31039

chimp.LOC112206744-LOC107973052-GGT2.revcompl                                          ------------------------------------------------------------ 61762
chimp54546-135457.revcompl.Pan.troglodytes.clone.rp43-41g5.GenBank:AC099533.36         AGGTGGAGTTCACAGATCATTCTGTCTGCATTTT-------------------------- 74070
human.FAM30B-LOC105372935-GGT2.NCBI.                                                   agatggcaagaccacatctctccaaaacaatttttgtaaaattagccaggtgtgctggtg 111845
human.FAM230E-LOC105377182-GGT3P.NCBI.                                                 ------------------------------------------------------------ 57310
human.FAM230J-LOC105372942-GGTLC5P.NCBI.rev.compl                                      ------------------------------------------------------------ 46364
human.FAM230A.ncRNA.18487127.18500594-GGTLC3.18516335.18518165.rev.compl.REF           ------------------------------------------------------------ 31039

chimp.LOC112206744-LOC107973052-GGT2.revcompl                                          ------------------------------------------------------------ 61762
chimp54546-135457.revcompl.Pan.troglodytes.clone.rp43-41g5.GenBank:AC099533.36         ------------------------------------GTGAGCCCATTCTATAGTTAACAG 74094
human.FAM30B-LOC105372935-GGT2.NCBI.                                                   cgggcctgcagtctcagctagtgagaagactaaggcaagaggaacccttgaacccaggag 111905
human.FAM230E-LOC105377182-GGT3P.NCBI.                                                 ------------------------------------------------------------ 57310
human.FAM230J-LOC105372942-GGTLC5P.NCBI.rev.compl                                      ------------------------------------------------------------ 46364
human.FAM230A.ncRNA.18487127.18500594-GGTLC3.18516335.18518165.rev.compl.REF           ------------------------------------------------------------ 31039

chimp.LOC112206744-LOC107973052-GGT2.revcompl                                          ------------------------------------------------------------ 61762
chimp54546-135457.revcompl.Pan.troglodytes.clone.rp43-41g5.GenBank:AC099533.36         CAGAAGACTTTGATAGGTTCTGGGTCCTGCCCCAGAGCAGGTG-TGGATTCAGAACCTGC 74153
human.FAM30B-LOC105372935-GGT2.NCBI.                                                   ttcaaggctacggtgagctgtgatcacctctccagcctgggtgatagggtgagacccagt 111965
human.FAM230E-LOC105377182-GGT3P.NCBI.                                                 ------------------------------------------------------------ 57310
human.FAM230J-LOC105372942-GGTLC5P.NCBI.rev.compl                                      ------------------------------------------------------------ 46364
```

```
human.FAM230A.ncRNA.18487127.18500594-GGTLC3.18516335.18518165.rev.compl.REF          ------------------------------------------------------------ 31039

chimp.LOC112206744-LOC107973052-GGT2.revcompl                                          ------------------------------------------------------------ 61762
chimp54546-135457.revcompl.Pan.troglodytes.clone.rp43-41g5.GenBank:AC099533.36         TGCTCAGGTCCCT----------GATTCCCTTGTCCATGGACTGCTGGTTCCAAGAGAGG 74203
human.FAM30B-LOC105372935-GGT2.NCBI.                                                   ctctgaaaacaattaaaaataaaaaaagttgaataatgagtcttcttttagaggcaatg 112025
human.FAM230E-LOC105377182-GGT3P.NCBI.                                                 ------------------------------------------------------------ 57310
human.FAM230J-LOC105372942-GGTLC5P.NCBI.rev.compl                                      ------------------------------------------------------------ 46364
human.FAM230A.ncRNA.18487127.18500594-GGTLC3.18516335.18518165.rev.compl.REF          ------------------------------------------------------------ 31039

chimp.LOC112206744-LOC107973052-GGT2.revcompl                                          ------------------------------------------------------------ 61762
chimp54546-135457.revcompl.Pan.troglodytes.clone.rp43-41g5.GenBank:AC099533.36         AATTCCTCTTCTTTATTTGGCCTTATATGCCTCAATACCAGGCATAAGGGGCTCGGTTAC 74263
human.FAM30B-LOC105372935-GGT2.NCBI.                                                   tatttttcctctttttgttttccttaggctatatgcacaatagacaaagttgaaatttaa 112085
human.FAM230E-LOC105377182-GGT3P.NCBI.                                                 ------------------------------------------------------------ 57310
human.FAM230J-LOC105372942-GGTLC5P.NCBI.rev.compl                                      ------------------------------------------------------------ 46364
human.FAM230A.ncRNA.18487127.18500594-GGTLC3.18516335.18518165.rev.compl.REF          ------------------------------------------------------------ 31039

chimp.LOC112206744-LOC107973052-GGT2.revcompl                                          ------------------------------------------------------------ 61762
chimp54546-135457.revcompl.Pan.troglodytes.clone.rp43-41g5.GenBank:AC099533.36         CCAT-------------------------------------------------------- 74267
human.FAM30B-LOC105372935-GGT2.NCBI.                                                   tcttaatctcgtaaacccttactgtccaaacacatcaaactcaaaaaaaaaaaaagacaa 112145
human.FAM230E-LOC105377182-GGT3P.NCBI.                                                 ------------------------------------------------------------ 57310
human.FAM230J-LOC105372942-GGTLC5P.NCBI.rev.compl                                      ------------------------------------------------------------ 46364
human.FAM230A.ncRNA.18487127.18500594-GGTLC3.18516335.18518165.rev.compl.REF          ------------------------------------------------------------ 31039

chimp.LOC112206744-LOC107973052-GGT2.revcompl                                          ------------------------------------------------------------ 61762
chimp54546-135457.revcompl.Pan.troglodytes.clone.rp43-41g5.GenBank:AC099533.36         ---GAAATTATGAGCACGTCCTATTGGAGGAAAATGGGTAGGGCCC-------------- 74310
human.FAM30B-LOC105372935-GGT2.NCBI.                                                   aaacaaaaaaggtgaaaggcgtacaagagaaaaaagggaagtaatctgtgccaaatgtac 112205
human.FAM230E-LOC105377182-GGT3P.NCBI.                                                 ------------------------------------------------------------ 57310
human.FAM230J-LOC105372942-GGTLC5P.NCBI.rev.compl                                      ------------------------------------------------------------ 46364
human.FAM230A.ncRNA.18487127.18500594-GGTLC3.18516335.18518165.rev.compl.REF          ------------------------------------------------------------ 31039

chimp.LOC112206744-LOC107973052-GGT2.revcompl                                          ------------------------------------------------------------ 61762
chimp54546-135457.revcompl.Pan.troglodytes.clone.rp43-41g5.GenBank:AC099533.36         -------------------ACCTAAGACATCCCTCTACTGTCACTGGGTTGGTAAGTGGT 74351
human.FAM30B-LOC105372935-GGT2.NCBI.                                                   attccttccacaatttgggacaaaaatgatgcctacctggtctctgggtgccaaaataag 112265
human.FAM230E-LOC105377182-GGT3P.NCBI.                                                 ------------------------------------------------------------ 57310
human.FAM230J-LOC105372942-GGTLC5P.NCBI.rev.compl                                      ------------------------------------------------------------ 46364
human.FAM230A.ncRNA.18487127.18500594-GGTLC3.18516335.18518165.rev.compl.REF          ------------------------------------------------------------ 31039

chimp.LOC112206744-LOC107973052-GGT2.revcompl                                          ------------------------------------------------------------ 61762
chimp54546-135457.revcompl.Pan.troglodytes.clone.rp43-41g5.GenBank:AC099533.36         CCAATGCATCTTACCAAGGCTCTGCATATAGTGCCGCAGACCTCAGACACCAAGTTTCAT 74411
human.FAM30B-LOC105372935-GGT2.NCBI.                                                   acattagagacactcccctc-cttcaagaaataaaattggaatcagacactaagatacaa 112324
human.FAM230E-LOC105377182-GGT3P.NCBI.                                                 ------------------------------------------------------------ 57310
human.FAM230J-LOC105372942-GGTLC5P.NCBI.rev.compl                                      ------------------------------------------------------------ 46364
human.FAM230A.ncRNA.18487127.18500594-GGTLC3.18516335.18518165.rev.compl.REF          ------------------------------------------------------------ 31039

chimp.LOC112206744-LOC107973052-GGT2.revcompl                                          ------------------------------------------------------------ 61762
chimp54546-135457.revcompl.Pan.troglodytes.clone.rp43-41g5.GenBank:AC099533.36         GGCTCCAGGTGTAGAAAAACATGAGGCAGTGGTGGCTCCTGCCCCAGAAACAAAGGCAGG 74471
human.FAM30B-LOC105372935-GGT2.NCBI.                                                   ttaaacagactttgaaagttggtatgcactactgggaggtgtctcacagct---ggcagg 112381
human.FAM230E-LOC105377182-GGT3P.NCBI.                                                 ------------------------------------------------------------ 57310
human.FAM230J-LOC105372942-GGTLC5P.NCBI.rev.compl                                      ------------------------------------------------------------ 46364
human.FAM230A.ncRNA.18487127.18500594-GGTLC3.18516335.18518165.rev.compl.REF          ------------------------------------------------------------ 31039

chimp.LOC112206744-LOC107973052-GGT2.revcompl                                          ------------------------------------------------------------ 61762
chimp54546-135457.revcompl.Pan.troglodytes.clone.rp43-41g5.GenBank:AC099533.36         AGCATATCCACCCAGCCAATA--------------------------------------- 74492
human.FAM30B-LOC105372935-GGT2.NCBI.                                                   gtcaactgcaactagaagaaaagctcctaagggcagaaactaagtggctcacttcctctg 112441
human.FAM230E-LOC105377182-GGT3P.NCBI.                                                 ------------------------------------------------------------ 57310
human.FAM230J-LOC105372942-GGTLC5P.NCBI.rev.compl                                      ------------------------------------------------------------ 46364
human.FAM230A.ncRNA.18487127.18500594-GGTLC3.18516335.18518165.rev.compl.REF          ------------------------------------------------------------ 31039

chimp.LOC112206744-LOC107973052-GGT2.revcompl                                          ------------------------------------------------------------ 61762
chimp54546-135457.revcompl.Pan.troglodytes.clone.rp43-41g5.GenBank:AC099533.36         --------------------------------------------------TTTATTGAGA 74502
human.FAM30B-LOC105372935-GGT2.NCBI.                                                   taacactgccgtgcccagcactgtaccagcaccccagcagagtcaacaactttccgtcaa 112501
human.FAM230E-LOC105377182-GGT3P.NCBI.                                                 ------------------------------------------------------------ 57310
human.FAM230J-LOC105372942-GGTLC5P.NCBI.rev.compl                                      ------------------------------------------------------------ 46364
human.FAM230A.ncRNA.18487127.18500594-GGTLC3.18516335.18518165.rev.compl.REF          ------------------------------------------------------------ 31039

chimp.LOC112206744-LOC107973052-GGT2.revcompl                                          ------------------------------------------------------------ 61762
chimp54546-135457.revcompl.Pan.troglodytes.clone.rp43-41g5.GenBank:AC099533.36         CCCACTCTGATCCGGCAATATTCCATCCTGGGAATTCAGCATGAGAAAGACTGTCTCTGA 74562
human.FAM30B-LOC105372935-GGT2.NCBI.                                                   gccacacacttacggtctcatcgtccagtggaggccgcagatgagacatagtgtgccaaa 112561
human.FAM230E-LOC105377182-GGT3P.NCBI.                                                 ------------------------------------------------------------ 57310
human.FAM230J-LOC105372942-GGTLC5P.NCBI.rev.compl                                      ------------------------------------------------------------ 46364
human.FAM230A.ncRNA.18487127.18500594-GGTLC3.18516335.18518165.rev.compl.REF          ------------------------------------------------------------ 31039

chimp.LOC112206744-LOC107973052-GGT2.revcompl                                          ------------------------------------------------------------ 61762
chimp54546-135457.revcompl.Pan.troglodytes.clone.rp43-41g5.GenBank:AC099533.36         CCCCAGGAGTTTCCTACATGGGGGTTGGGGCTGTGTCATAGAAACAGTTTGTAGTAGAAG 74622
human.FAM30B-LOC105372935-GGT2.NCBI.                                                   tcccaaatg--------------------------------------------------- 112570
human.FAM230E-LOC105377182-GGT3P.NCBI.                                                 ------------------------------------------------------------ 57310
human.FAM230J-LOC105372942-GGTLC5P.NCBI.rev.compl                                      ------------------------------------------------------------ 46364
human.FAM230A.ncRNA.18487127.18500594-GGTLC3.18516335.18518165.rev.compl.REF          ------------------------------------------------------------ 31039

chimp.LOC112206744-LOC107973052-GGT2.revcompl                                          ------------------------------------------------------------ 61762
chimp54546-135457.revcompl.Pan.troglodytes.clone.rp43-41g5.GenBank:AC099533.36         CACCATCTTCTACCTGTGTATTTTCTCCTCTATTTATTTACAAAAAGATCCTTGTTTTCC 74682
human.FAM30B-LOC105372935-GGT2.NCBI.                                                   ------------------------------------------------------------ 112570
human.FAM230E-LOC105377182-GGT3P.NCBI.                                                 ------------------------------------------------------------ 57310
human.FAM230J-LOC105372942-GGTLC5P.NCBI.rev.compl                                      ------------------------------------------------------------ 46364
human.FAM230A.ncRNA.18487127.18500594-GGTLC3.18516335.18518165.rev.compl.REF          ------------------------------------------------------------ 31039

chimp.LOC112206744-LOC107973052-GGT2.revcompl                                          ------------------------------------------------------------ 61762
chimp54546-135457.revcompl.Pan.troglodytes.clone.rp43-41g5.GenBank:AC099533.36         CTGGTCACTCTTGGCTTGGACTGACCACATGGTCCCATGCTGGCCCCAGGGCTGTAAGGG 74742
human.FAM30B-LOC105372935-GGT2.NCBI.                                                   -------------------------cacagggtgccagacaagtctgcgggaggcaggag 112605
human.FAM230E-LOC105377182-GGT3P.NCBI.                                                 ------------------------------------------------------------ 57310
human.FAM230J-LOC105372942-GGTLC5P.NCBI.rev.compl                                      ------------------------------------------------------------ 46364
human.FAM230A.ncRNA.18487127.18500594-GGTLC3.18516335.18518165.rev.compl.REF          ------------------------------------------------------------ 31039

chimp.LOC112206744-LOC107973052-GGT2.revcompl                                          ------------------------------------------------------------ 61762
chimp54546-135457.revcompl.Pan.troglodytes.clone.rp43-41g5.GenBank:AC099533.36         TCAGGTGGTTG------------------------------------------------- 74753
human.FAM30B-LOC105372935-GGT2.NCBI.                                                   gactagggaagccttccaggcaaggtgatgcctgagctgagttgtttttcttttgagttt 112665
human.FAM230E-LOC105377182-GGT3P.NCBI.                                                 ------------------------------------------------------------ 57310
human.FAM230J-LOC105372942-GGTLC5P.NCBI.rev.compl                                      ------------------------------------------------------------ 46364
human.FAM230A.ncRNA.18487127.18500594-GGTLC3.18516335.18518165.rev.compl.REF          ------------------------------------------------------------ 31039

chimp.LOC112206744-LOC107973052-GGT2.revcompl                                          ------------------------------------------------------------ 61762
chimp54546-135457.revcompl.Pan.troglodytes.clone.rp43-41g5.GenBank:AC099533.36         ------------------------------------------------------------ 74753
human.FAM30B-LOC105372935-GGT2.NCBI.                                                   ggaacattaatttaatcggaggaaaacaagggacatgaaaagggcaaggaaataagaaaa 112725
human.FAM230E-LOC105377182-GGT3P.NCBI.                                                 ------------------------------------------------------------ 57310
human.FAM230J-LOC105372942-GGTLC5P.NCBI.rev.compl                                      ------------------------------------------------------------ 46364
human.FAM230A.ncRNA.18487127.18500594-GGTLC3.18516335.18518165.rev.compl.REF          ------------------------------------------------------------ 31039

chimp.LOC112206744-LOC107973052-GGT2.revcompl                                          ------------------------------------------------------------ 61762
chimp54546-135457.revcompl.Pan.troglodytes.clone.rp43-41g5.GenBank:AC099533.36         -------------------------GAGATCTTCTCAGGGAACATTTTCTCTTGTGATGG 74788
human.FAM30B-LOC105372935-GGT2.NCBI.                                                   tgagctgagttttgaaggcaatgatcagatgaagtgagagaagaatattccagcagaggg 112785
human.FAM230E-LOC105377182-GGT3P.NCBI.                                                 ------------------------------------------------------------ 57310
human.FAM230J-LOC105372942-GGTLC5P.NCBI.rev.compl                                      ------------------------------------------------------------ 46364
human.FAM230A.ncRNA.18487127.18500594-GGTLC3.18516335.18518165.rev.compl.REF          ------------------------------------------------------------ 31039

chimp.LOC112206744-LOC107973052-GGT2.revcompl                                          ------------------------------------------------------------ 61762
chimp54546-135457.revcompl.Pan.troglodytes.clone.rp43-41g5.GenBank:AC099533.36         AAATAAACAGACTG-------------------------TTTTCGTGCAGCACCTTTGCT 74823
human.FAM30B-LOC105372935-GGT2.NCBI.                                                   agacaaggagactgcagagatttcaaggggccaagagctgttcggtgcagctcagtgaag 112845
human.FAM230E-LOC105377182-GGT3P.NCBI.                                                 ------------------------------------------------------------ 57310
human.FAM230J-LOC105372942-GGTLC5P.NCBI.rev.compl                                      ------------------------------------------------------------ 46364
human.FAM230A.ncRNA.18487127.18500594-GGTLC3.18516335.18518165.rev.compl.REF          ------------------------------------------------------------ 31039

chimp.LOC112206744-LOC107973052-GGT2.revcompl                                          ------------------------------------------------------------ 61762
chimp54546-135457.revcompl.Pan.troglodytes.clone.rp43-41g5.GenBank:AC099533.36         CTTGAATATGGGTATAATAGCTGGAGCTGCAGCAGCCATCCTGAGGTCTTAAGTCCACAC 74883
human.FAM30B-LOC105372935-GGT2.NCBI.                                                   cacctaaacagaggtgggggaaggagaacaggggtcaaagcccagtattgaagtgttctg 112905
human.FAM230E-LOC105377182-GGT3P.NCBI.                                                 ------------------------------------------------------------ 57310
human.FAM230J-LOC105372942-GGTLC5P.NCBI.rev.compl                                      ------------------------------------------------------------ 46364
human.FAM230A.ncRNA.18487127.18500594-GGTLC3.18516335.18518165.rev.compl.REF          ------------------------------------------------------------ 31039
```

```
chimp.LOC112206744-LOC107973052-GGT2.revcompl                              ------------------------------------------------------------ 61762
chimp54546-135457.revcompl.Pan.troglodytes.clone.rp43-41g5.GenBank:AC099533.36      AAATGAAAGAAAAGCCAACTGTGTCATGA------------TGGTGGAGCAGAAAGTTGG 74931
human.FAM30B-LOC105372935-GGT2.NCBI.                                       gataccaagctaaggagaccttattacgaaggtactggggctgctgaagggtttaaatgg 112965
human.FAM230E-LOC105377182-GGT3P.NCBI.                                     ------------------------------------------------------------ 57310
human.FAM230J-LOC105372942-GGTLC5P.NCBI.rev.compl                          ------------------------------------------------------------ 46364
human.FAM230A.ncRNA.18487127.18500594-GGTLC3.18516335.18518165.rev.compl.REF ------------------------------------------------------------ 31039

chimp.LOC112206744-LOC107973052-GGT2.revcompl                              ------------------------------------------------------------ 61762
chimp54546-135457.revcompl.Pan.troglodytes.clone.rp43-41g5.GenBank:AC099533.36      GTAATTGCTGGGTTTCTCATTATTTGGTGAGTATGTGGGGAACAGCAGTGAA-CTCCACA 74990
human.FAM30B-LOC105372935-GGT2.NCBI.                                       gagagtgattgtgaacagattaatagtttggaaagatacaggcagaagtaaacctctgga 113025
human.FAM230E-LOC105377182-GGT3P.NCBI.                                     ------------------------------------------------------------ 57310
human.FAM230J-LOC105372942-GGTLC5P.NCBI.rev.compl                          ------------------------------------------------------------ 46364
human.FAM230A.ncRNA.18487127.18500594-GGTLC3.18516335.18518165.rev.compl.REF ------------------------------------------------------------ 31039

chimp.LOC112206744-LOC107973052-GGT2.revcompl                              ------------------------------------------------------------ 61762
chimp54546-135457.revcompl.Pan.troglodytes.clone.rp43-41g5.GenBank:AC099533.36      CTTACTTTTTGAGGATAAAATAGCAAATGGTTCATGACTATTTCTCATGAGTGTCCAAGC 75050
human.FAM30B-LOC105372935-GGT2.NCBI.                                       tttaaccaaaaaataaaggacagaaccacaatgaagatgttggaaaagatcaggaaaaac 113085
human.FAM230E-LOC105377182-GGT3P.NCBI.                                     ------------------------------------------------------------ 57310
human.FAM230J-LOC105372942-GGTLC5P.NCBI.rev.compl                          ------------------------------------------------------------ 46364
human.FAM230A.ncRNA.18487127.18500594-GGTLC3.18516335.18518165.rev.compl.REF ------------------------------------------------------------ 31039

chimp.LOC112206744-LOC107973052-GGT2.revcompl                              ------------------------------------------------------------ 61762
chimp54546-135457.revcompl.Pan.troglodytes.clone.rp43-41g5.GenBank:AC099533.36      AGAGGAGAAGCATATTTGATTGATTGTAGGAGTATTTAGCACTAGAAAATCAATTAATAA 75110
human.FAM30B-LOC105372935-GGT2.NCBI.                                       actgttaaaagatattcactctcggggccgggcgctgtggctcacgccttaaatcccggc 113145
human.FAM230E-LOC105377182-GGT3P.NCBI.                                     ------------------------------------------------------------ 57310
human.FAM230J-LOC105372942-GGTLC5P.NCBI.rev.compl                          ------------------------------------------------------------ 46364
human.FAM230A.ncRNA.18487127.18500594-GGTLC3.18516335.18518165.rev.compl.REF ------------------------------------------------------------ 31039

chimp.LOC112206744-LOC107973052-GGT2.revcompl                              ------------------------------------------------------------ 61762
chimp54546-135457.revcompl.Pan.troglodytes.clone.rp43-41g5.GenBank:AC099533.36      ACAGTTAGGAAAAGAGGCATGAAGTTTAGGTGCTCTGCAGATCATTAATAGAATGCATAC 75170
human.FAM30B-LOC105372935-GGT2.NCBI.                                       actttggtaggccgaggcgggctgatcacgaggtcaggagatcgagaccatcctggctaa 113205
human.FAM230E-LOC105377182-GGT3P.NCBI.                                     ------------------------------------------------------------ 57310
human.FAM230J-LOC105372942-GGTLC5P.NCBI.rev.compl                          ------------------------------------------------------------ 46364
human.FAM230A.ncRNA.18487127.18500594-GGTLC3.18516335.18518165.rev.compl.REF ------------------------------------------------------------ 31039

chimp.LOC112206744-LOC107973052-GGT2.revcompl                              ------------------------------------------------------------ 61762
chimp54546-135457.revcompl.Pan.troglodytes.clone.rp43-41g5.GenBank:AC099533.36      ACA--------------------------------------------------------- 75173
human.FAM30B-LOC105372935-GGT2.NCBI.                                       cacggtcaaaccccgtctctactaaaaatacaaaaaattagccgggtgtggtggcgggcg 113265
human.FAM230E-LOC105377182-GGT3P.NCBI.                                     ------------------------------------------------------------ 57310
human.FAM230J-LOC105372942-GGTLC5P.NCBI.rev.compl                          ------------------------------------------------------------ 46364
human.FAM230A.ncRNA.18487127.18500594-GGTLC3.18516335.18518165.rev.compl.REF ------------------------------------------------------------ 31039

chimp.LOC112206744-LOC107973052-GGT2.revcompl                              ------------------------------------------------------------ 61762
chimp54546-135457.revcompl.Pan.troglodytes.clone.rp43-41g5.GenBank:AC099533.36      ---------------------------------------------GAAAATGACAGGCAG 75188
human.FAM30B-LOC105372935-GGT2.NCBI.                                       cctgtagtcccaactactccagaggctgaggcaggagaatggcgtgaacccggtaggcgg 113325
human.FAM230E-LOC105377182-GGT3P.NCBI.                                     ------------------------------------------------------------ 57310
human.FAM230J-LOC105372942-GGTLC5P.NCBI.rev.compl                          ------------------------------------------------------------ 46364
human.FAM230A.ncRNA.18487127.18500594-GGTLC3.18516335.18518165.rev.compl.REF ------------------------------------------------------------ 31039

chimp.LOC112206744-LOC107973052-GGT2.revcompl                              ------------------------------------------------------------ 61762
chimp54546-135457.revcompl.Pan.troglodytes.clone.rp43-41g5.GenBank:AC099533.36      ACTGCTGTGTAATCGGAGGTTGGGGCGCCTCCCTGAAGAGGAGTCTCTGGAGCTGCTCTC 75248
human.FAM30B-LOC105372935-GGT2.NCBI.                                       agcttgcggtgagccgagatcaggccactggaatccagcctgggcgacagagggagactc 113385
human.FAM230E-LOC105377182-GGT3P.NCBI.                                     ------------------------------------------------------------ 57310
human.FAM230J-LOC105372942-GGTLC5P.NCBI.rev.compl                          ------------------------------------------------------------ 46364
human.FAM230A.ncRNA.18487127.18500594-GGTLC3.18516335.18518165.rev.compl.REF ------------------------------------------------------------ 31039

chimp.LOC112206744-LOC107973052-GGT2.revcompl                              ------------------------------------------------------------ 61762
chimp54546-135457.revcompl.Pan.troglodytes.clone.rp43-41g5.GenBank:AC099533.36      AGGAAGGAAGAAACAGTGAAAGGAAGTGTGAGGAAGAAGAGTCTGTCACTGACCTGCAGG 75308
human.FAM30B-LOC105372935-GGT2.NCBI.                                       catctcaaaaaaaaaaaaaaaaaaaaagagag---agagagtctgactctgttgccaaga 113442
human.FAM230E-LOC105377182-GGT3P.NCBI.                                     ------------------------------------------------------------ 57310
human.FAM230J-LOC105372942-GGTLC5P.NCBI.rev.compl                          ------------------------------------------------------------ 46364
human.FAM230A.ncRNA.18487127.18500594-GGTLC3.18516335.18518165.rev.compl.REF ------------------------------------------------------------ 31039

chimp.LOC112206744-LOC107973052-GGT2.revcompl                              ------------------------------------------------------------ 61762
chimp54546-135457.revcompl.Pan.troglodytes.clone.rp43-41g5.GenBank:AC099533.36      GAGGGGT---------------------------------------------------AG 75317
human.FAM30B-LOC105372935-GGT2.NCBI.                                       ctggagtgcagtggcgcgatctaggcacatcacaatcccttccccgcccccgggttcaag 113502
human.FAM230E-LOC105377182-GGT3P.NCBI.                                     ------------------------------------------------------------ 57310
human.FAM230J-LOC105372942-GGTLC5P.NCBI.rev.compl                          ------------------------------------------------------------ 46364
human.FAM230A.ncRNA.18487127.18500594-GGTLC3.18516335.18518165.rev.compl.REF ------------------------------------------------------------ 31039

chimp.LOC112206744-LOC107973052-GGT2.revcompl                              ------------------------------------------------------------ 61762
chimp54546-135457.revcompl.Pan.troglodytes.clone.rp43-41g5.GenBank:AC099533.36      GTGTTTTCGAGGATCTGAAAGAGCAGTGACAAACATCAAGGGGGTCTGGGATCCAAACTC 75377
human.FAM30B-LOC105372935-GGT2.NCBI.                                       tgattctcctgtctcagccgccggagtagctgggactacaggcgcgtgccaccatgtctg 113562
human.FAM230E-LOC105377182-GGT3P.NCBI.                                     ------------------------------------------------------------ 57310
human.FAM230J-LOC105372942-GGTLC5P.NCBI.rev.compl                          ------------------------------------------------------------ 46364
human.FAM230A.ncRNA.18487127.18500594-GGTLC3.18516335.18518165.rev.compl.REF ------------------------------------------------------------ 31039

chimp.LOC112206744-LOC107973052-GGT2.revcompl                              ------------------------------------------------------------ 61762
chimp54546-135457.revcompl.Pan.troglodytes.clone.rp43-41g5.GenBank:AC099533.36      ATTGCAATGCAGTGTATGATA--------------------------------------- 75398
human.FAM30B-LOC105372935-GGT2.NCBI.                                       attaaatctgtattttactagagacggggtttcactctgttggccaggctggtctccaa 113622
human.FAM230E-LOC105377182-GGT3P.NCBI.                                     ------------------------------------------------------------ 57310
human.FAM230J-LOC105372942-GGTLC5P.NCBI.rev.compl                          ------------------------------------------------------------ 46364
human.FAM230A.ncRNA.18487127.18500594-GGTLC3.18516335.18518165.rev.compl.REF ------------------------------------------------------------ 31039

chimp.LOC112206744-LOC107973052-GGT2.revcompl                              ------------------------------------------------------------ 61762
chimp54546-135457.revcompl.Pan.troglodytes.clone.rp43-41g5.GenBank:AC099533.36      ------------------------------------------------------------ 75398
human.FAM30B-LOC105372935-GGT2.NCBI.                                       ctcctgatctcgtgatccgcccgccccgacctcccaaagtgctgggatgcgtgagcctcc 113682
human.FAM230E-LOC105377182-GGT3P.NCBI.                                     ------------------------------------------------------------ 57310
human.FAM230J-LOC105372942-GGTLC5P.NCBI.rev.compl                          ------------------------------------------------------------ 46364
human.FAM230A.ncRNA.18487127.18500594-GGTLC3.18516335.18518165.rev.compl.REF ------------------------------------------------------------ 31039

chimp.LOC112206744-LOC107973052-GGT2.revcompl                              ------------------------------------------------------------ 61762
chimp54546-135457.revcompl.Pan.troglodytes.clone.rp43-41g5.GenBank:AC099533.36      ------------------------------------------------------------ 75398
human.FAM30B-LOC105372935-GGT2.NCBI.                                       acgcctggccactaattttctttctttctttcgtgtgtgtgtgtgtgtgtgtgtgtgtg 113742
human.FAM230E-LOC105377182-GGT3P.NCBI.                                     ------------------------------------------------------------ 57310
human.FAM230J-LOC105372942-GGTLC5P.NCBI.rev.compl                          ------------------------------------------------------------ 46364
human.FAM230A.ncRNA.18487127.18500594-GGTLC3.18516335.18518165.rev.compl.REF ------------------------------------------------------------ 31039

chimp.LOC112206744-LOC107973052-GGT2.revcompl                              ------------------------------------------------------------ 61762
chimp54546-135457.revcompl.Pan.troglodytes.clone.rp43-41g5.GenBank:AC099533.36      -----------------------------------CTTTTAGCTGAGGCAGGAGTGCAGC 75423
human.FAM30B-LOC105372935-GGT2.NCBI.                                       tgtgtgtgtgtgtgtgtgtgtgacgaagtttcgctcttgttgcccaggttggagtgcaat 113802
human.FAM230E-LOC105377182-GGT3P.NCBI.                                     ------------------------------------------------------------ 57310
human.FAM230J-LOC105372942-GGTLC5P.NCBI.rev.compl                          ------------------------------------------------------------ 46364
human.FAM230A.ncRNA.18487127.18500594-GGTLC3.18516335.18518165.rev.compl.REF ------------------------------------------------------------ 31039

chimp.LOC112206744-LOC107973052-GGT2.revcompl                              ------------------------------------------------------------ 61762
chimp54546-135457.revcompl.Pan.troglodytes.clone.rp43-41g5.GenBank:AC099533.36      GTATTAAGGTGTGGTTAGAGGAATTTCCAGGTGATGATGAATGATGTGACCTTGGCATCT 75483
human.FAM30B-LOC105372935-GGT2.NCBI.                                       ggtgtgatctcagctcactgcaatccccgcctcaacaggagagcaggaatctt------- 113855
human.FAM230E-LOC105377182-GGT3P.NCBI.                                     ------------------------------------------------------------ 57310
human.FAM230J-LOC105372942-GGTLC5P.NCBI.rev.compl                          ------------------------------------------------------------ 46364
human.FAM230A.ncRNA.18487127.18500594-GGTLC3.18516335.18518165.rev.compl.REF ------------------------------------------------------------ 31039

chimp.LOC112206744-LOC107973052-GGT2.revcompl                              ------------------------------------------------------------ 61762
chimp54546-135457.revcompl.Pan.troglodytes.clone.rp43-41g5.GenBank:AC099533.36      GCTTAGCACATGGTATGGCCTCCAGGACGCTGCTGCTGGGGCGGGGGCAGGAGAGACACA 75543
human.FAM30B-LOC105372935-GGT2.NCBI.                                       ------------------------------------------------------------ 113855
human.FAM230E-LOC105377182-GGT3P.NCBI.                                     ------------------------------------------------------------ 57310
human.FAM230J-LOC105372942-GGTLC5P.NCBI.rev.compl                          ------------------------------------------------------------ 46364
human.FAM230A.ncRNA.18487127.18500594-GGTLC3.18516335.18518165.rev.compl.REF ------------------------------------------------------------ 31039

chimp.LOC112206744-LOC107973052-GGT2.revcompl                              ------------------------------------------------------------ 61762
chimp54546-135457.revcompl.Pan.troglodytes.clone.rp43-41g5.GenBank:AC099533.36      AGTGGGGTCAGACCTGCCCCAGGCTGTTTCCTATTTTAGCAGGGTCTGAAGCTGCCCAGG 75603
human.FAM30B-LOC105372935-GGT2.NCBI.                                       -----------------------cagtgatccactggcggatctgcagccattgtgcgcg 113892
human.FAM230E-LOC105377182-GGT3P.NCBI.                                     ------------------------------------------------------------ 57310
human.FAM230J-LOC105372942-GGTLC5P.NCBI.rev.compl                          ------------------------------------------------------------ 46364
human.FAM230A.ncRNA.18487127.18500594-GGTLC3.18516335.18518165.rev.compl.REF ------------------------------------------------------------ 31039
```

```
chimp.LOC112206744-LOC107973052-GGT2.revcompl                                      ------------------------------------------------------------ 61762
chimp54546-135457.revcompl.Pan.troglodytes.clone.rp43-41g5.GenBank:AC099533.36     AGGGGGCTTACCCACCCCTTTGTTCCTCCATTACTTGTGCAACAAGCAGCCTTGTGGGGA 75663
human.FAM30B-LOC105372935-GGT2.NCBI.                                               ccaggtcttcccaagtcttttgtgcgcgcgcctctccttccagtacc------------- 113939
human.FAM230E-LOC105377182-GGT3P.NCBI.                                             ------------------------------------------------------------ 57310
human.FAM230J-LOC105372942-GGTLC5P.NCBI.rev.compl                                  ------------------------------------------------------------ 46364
human.FAM230A.ncRNA.18487127.18500594-GGTLC3.18516335.18518165.rev.compl.REF       ------------------------------------------------------------ 31039

chimp.LOC112206744-LOC107973052-GGT2.revcompl                                      ------------------------------------------------------------ 61762
chimp54546-135457.revcompl.Pan.troglodytes.clone.rp43-41g5.GenBank:AC099533.36     CATACAGTGCTCCATCCTGGGACCCCGTCGAGGGCCTCACATGGGTGACTGATCCCTAAT 75723
human.FAM30B-LOC105372935-GGT2.NCBI.                                               tatgccgccagcgtcccctagcctcccgccattgccagcaggtgctgagatcgcgccatt 113999
human.FAM230E-LOC105377182-GGT3P.NCBI.                                             ------------------------------------------------------------ 57310
human.FAM230J-LOC105372942-GGTLC5P.NCBI.rev.compl                                  ------------------------------------------------------------ 46364
human.FAM230A.ncRNA.18487127.18500594-GGTLC3.18516335.18518165.rev.compl.REF       ------------------------------------------------------------ 31039

chimp.LOC112206744-LOC107973052-GGT2.revcompl                                      ------------------------------------------------------------ 61762
chimp54546-135457.revcompl.Pan.troglodytes.clone.rp43-41g5.GenBank:AC099533.36     GTCCCACAGACTGGCTGATCTGAGCTGATCACAGTGGGTGGGGAAAACACTGGACAGAGG 75783
human.FAM30B-LOC105372935-GGT2.NCBI.                                               gcactccagcctgggggacaagagcgaaactccatgtcaaaaaaaaaaa----------- 114048
human.FAM230E-LOC105377182-GGT3P.NCBI.                                             ------------------------------------------------------------ 57310
human.FAM230J-LOC105372942-GGTLC5P.NCBI.rev.compl                                  ------------------------------------------------------------ 46364
human.FAM230A.ncRNA.18487127.18500594-GGTLC3.18516335.18518165.rev.compl.REF       ------------------------------------------------------------ 31039

chimp.LOC112206744-LOC107973052-GGT2.revcompl                                      ------------------------------------------------------------ 61762
chimp54546-135457.revcompl.Pan.troglodytes.clone.rp43-41g5.GenBank:AC099533.36     AGGGAAGGGCCAGAGGGTGAGTAAAGTTCCCAAGGTCGCCTGGGGAGGAAGAGGGGAGAG 75843
human.FAM30B-LOC105372935-GGT2.NCBI.                                               -------------------------------aaggatgaaaattttgggaaaatatggaa 114077
human.FAM230E-LOC105377182-GGT3P.NCBI.                                             ------------------------------------------------------------ 57310
human.FAM230J-LOC105372942-GGTLC5P.NCBI.rev.compl                                  ------------------------------------------------------------ 46364
human.FAM230A.ncRNA.18487127.18500594-GGTLC3.18516335.18518165.rev.compl.REF       ------------------------------------------------------------ 31039

chimp.LOC112206744-LOC107973052-GGT2.revcompl                                      ------------------------------------------------------------ 61762
chimp54546-135457.revcompl.Pan.troglodytes.clone.rp43-41g5.GenBank:AC099533.36     CAAGACCAGGGAGGTCCTGGGCAGAAATAATGATCATTTCTAAGTGTGCAG---GAGCAA 75900
human.FAM30B-LOC105372935-GGT2.NCBI.                                               gaaaccaaatggatttctagctcaactaaatcgtaattattcagtgtctagttttggcaa 114137
human.FAM230E-LOC105377182-GGT3P.NCBI.                                             ------------------------------------------------------------ 57310
human.FAM230J-LOC105372942-GGTLC5P.NCBI.rev.compl                                  ------------------------------------------------------------ 46364
human.FAM230A.ncRNA.18487127.18500594-GGTLC3.18516335.18518165.rev.compl.REF       ------------------------------------------------------------ 31039

chimp.LOC112206744-LOC107973052-GGT2.revcompl                                      ------------------------------------------------------------ 61762
chimp54546-135457.revcompl.Pan.troglodytes.clone.rp43-41g5.GenBank:AC099533.36     GCAAGCTGGATGGGAGTGTC---------GGGAACAATCCTGCACCACAATTGTGGGGC 75951
human.FAM30B-LOC105372935-GGT2.NCBI.                                               gaaaccacatatttcatgtccacaatcagggacacagtcccagctctcaagtgtgggtct 114197
human.FAM230E-LOC105377182-GGT3P.NCBI.                                             ------------------------------------------------------------ 57310
human.FAM230J-LOC105372942-GGTLC5P.NCBI.rev.compl                                  ------------------------------------------------------------ 46364
human.FAM230A.ncRNA.18487127.18500594-GGTLC3.18516335.18518165.rev.compl.REF       ------------------------------------------------------------ 31039

chimp.LOC112206744-LOC107973052-GGT2.revcompl                                      ------------------------------------------------------------ 61762
chimp54546-135457.revcompl.Pan.troglodytes.clone.rp43-41g5.GenBank:AC099533.36     TCAGAAGG---------------------------------------------------- 75959
human.FAM30B-LOC105372935-GGT2.NCBI.                                               ttcctaagcaaattgaagaacacaggcataaaagtacattaaatcaataaacctttttct 114257
human.FAM230E-LOC105377182-GGT3P.NCBI.                                             ------------------------------------------------------------ 57310
human.FAM230J-LOC105372942-GGTLC5P.NCBI.rev.compl                                  ------------------------------------------------------------ 46364
human.FAM230A.ncRNA.18487127.18500594-GGTLC3.18516335.18518165.rev.compl.REF       ------------------------------------------------------------ 31039

chimp.LOC112206744-LOC107973052-GGT2.revcompl                                      ------------------------------------------------------------ 61762
chimp54546-135457.revcompl.Pan.troglodytes.clone.rp43-41g5.GenBank:AC099533.36     --------ATATAGCTCTTCCCTTTCAGTTCTGCCAGCTGCCTGTTGGGGGAGCTGCTGC 76011
human.FAM30B-LOC105372935-GGT2.NCBI.                                               ctctgtctctctctctcttttttttttttttttttgagacggaggttggcactg-----tc 114312
human.FAM230E-LOC105377182-GGT3P.NCBI.                                             ------------------------------------------------------------ 57310
human.FAM230J-LOC105372942-GGTLC5P.NCBI.rev.compl                                  ------------------------------------------------------------ 46364
human.FAM230A.ncRNA.18487127.18500594-GGTLC3.18516335.18518165.rev.compl.REF       ------------------------------------------------------------ 31039

chimp.LOC112206744-LOC107973052-GGT2.revcompl                                      ------------------------------------------------------------ 61762
chimp54546-135457.revcompl.Pan.troglodytes.clone.rp43-41g5.GenBank:AC099533.36     ACCCAGACATGAGTGAGCCAGGCACCTGGGGGGTAGCTTCCAGCTC-------------- 76057
human.FAM30B-LOC105372935-GGT2.NCBI.                                               acccaggctggagtgctgtggtgagatctcgggtcactgcaagctccgcctcccgagttc 114372
human.FAM230E-LOC105377182-GGT3P.NCBI.                                             ------------------------------------------------------------ 57310
human.FAM230J-LOC105372942-GGTLC5P.NCBI.rev.compl                                  ------------------------------------------------------------ 46364
human.FAM230A.ncRNA.18487127.18500594-GGTLC3.18516335.18518165.rev.compl.REF       ------------------------------------------------------------ 31039

chimp.LOC112206744-LOC107973052-GGT2.revcompl                                      ------------------------------------------------------------ 61762
chimp54546-135457.revcompl.Pan.troglodytes.clone.rp43-41g5.GenBank:AC099533.36     ------------------------------------------------------------ 76057
human.FAM30B-LOC105372935-GGT2.NCBI.                                               acgccatcctcctgcctcagcctccagagtagctgggactacaagcgcccgccacgacgc 114432
human.FAM230E-LOC105377182-GGT3P.NCBI.                                             ------------------------------------------------------------ 57310
human.FAM230J-LOC105372942-GGTLC5P.NCBI.rev.compl                                  ------------------------------------------------------------ 46364
human.FAM230A.ncRNA.18487127.18500594-GGTLC3.18516335.18518165.rev.compl.REF       ------------------------------------------------------------ 31039

chimp.LOC112206744-LOC107973052-GGT2.revcompl                                      ------------------------------------------------------------ 61762
chimp54546-135457.revcompl.Pan.troglodytes.clone.rp43-41g5.GenBank:AC099533.36     ------------------------------------------------------------ 76057
human.FAM30B-LOC105372935-GGT2.NCBI.                                               ccggctaatttttttgtagttttagtagagacggggtttcgctatgttggccaggctggtc 114492
human.FAM230E-LOC105377182-GGT3P.NCBI.                                             ------------------------------------------------------------ 57310
human.FAM230J-LOC105372942-GGTLC5P.NCBI.rev.compl                                  ------------------------------------------------------------ 46364
human.FAM230A.ncRNA.18487127.18500594-GGTLC3.18516335.18518165.rev.compl.REF       ------------------------------------------------------------ 31039

chimp.LOC112206744-LOC107973052-GGT2.revcompl                                      ------------------------------------------------------------ 61762
chimp54546-135457.revcompl.Pan.troglodytes.clone.rp43-41g5.GenBank:AC099533.36     ----------------------------------------------CCTGCACCCTTTGG 76071
human.FAM30B-LOC105372935-GGT2.NCBI.                                               tccaactcgtgacatcgtgatccgcccgcctccgactcccaaagtgccacagcccaggcc 114552
human.FAM230E-LOC105377182-GGT3P.NCBI.                                             ------------------------------------------------------------ 57310
human.FAM230J-LOC105372942-GGTLC5P.NCBI.rev.compl                                  ------------------------------------------------------------ 46364
human.FAM230A.ncRNA.18487127.18500594-GGTLC3.18516335.18518165.rev.compl.REF       ------------------------------------------------------------ 31039

chimp.LOC112206744-LOC107973052-GGT2.revcompl                                      ------------------------------------------------------------ 61762
chimp54546-135457.revcompl.Pan.troglodytes.clone.rp43-41g5.GenBank:AC099533.36     TGCTCATAAAAACTCCGAGGGTTGGCTGGGCACGGTGGCTCAAGCCTGTAATCCCAGCAC 76131
human.FAM30B-LOC105372935-GGT2.NCBI.                                               tttttttttaagacagagtctcggcccggcgcggtggctcacgcctttaatcccagcac 114612
human.FAM230E-LOC105377182-GGT3P.NCBI.                                             ------------------------------------------------------------ 57310
human.FAM230J-LOC105372942-GGTLC5P.NCBI.rev.compl                                  ------------------------------------------------------------ 46364
human.FAM230A.ncRNA.18487127.18500594-GGTLC3.18516335.18518165.rev.compl.REF       ------------------------------------------------------------ 31039

chimp.LOC112206744-LOC107973052-GGT2.revcompl                                      ------------------------------------------------------------ 61762
chimp54546-135457.revcompl.Pan.troglodytes.clone.rp43-41g5.GenBank:AC099533.36     TTTGGGAGGCCGAGGCGGGCGGATCACGAGGTCAGGAGATCGAGACCATCCTGGCTAACA 76191
human.FAM30B-LOC105372935-GGT2.NCBI.                                               tttgggaggccgaggcgggctgatcacgaggtcaggaggtcgagaccatcctggctaaca 114672
human.FAM230E-LOC105377182-GGT3P.NCBI.                                             ------------------------------------------------------------ 57310
human.FAM230J-LOC105372942-GGTLC5P.NCBI.rev.compl                                  ------------------------------------------------------------ 46364
human.FAM230A.ncRNA.18487127.18500594-GGTLC3.18516335.18518165.rev.compl.REF       ------------------------------------------------------------ 31039

chimp.LOC112206744-LOC107973052-GGT2.revcompl                                      ------------------------------------------------------------ 61762
chimp54546-135457.revcompl.Pan.troglodytes.clone.rp43-41g5.GenBank:AC099533.36     CGGTGAAACCCCGTCTCTACTAAAAATACAAAAAATTAGCCAGGCGCGGTGGCGGGTGCC 76251
human.FAM30B-LOC105372935-GGT2.NCBI.                                               cggtcaaaccccgtctctactaaaaatacaaaaaattagccgggtgtggtggcgggcgcc 114732
human.FAM230E-LOC105377182-GGT3P.NCBI.                                             ------------------------------------------------------------ 57310
human.FAM230J-LOC105372942-GGTLC5P.NCBI.rev.compl                                  ------------------------------------------------------------ 46364
human.FAM230A.ncRNA.18487127.18500594-GGTLC3.18516335.18518165.rev.compl.REF       ------------------------------------------------------------ 31039

chimp.LOC112206744-LOC107973052-GGT2.revcompl                                      ------------------------------------------------------------ 61762
chimp54546-135457.revcompl.Pan.troglodytes.clone.rp43-41g5.GenBank:AC099533.36     TGTAATCCCAGCTACTCAGGAGGCTGAGGCAGGAGAATGGCGTGAACCCGGGAGGCAGAG 76311
human.FAM30B-LOC105372935-GGT2.NCBI.                                               tgtagtccgagctactcccgaggctgaggcaggagaatggcgtgaacccggtaggcggag 114792
human.FAM230E-LOC105377182-GGT3P.NCBI.                                             ------------------------------------------------------------ 57310
human.FAM230J-LOC105372942-GGTLC5P.NCBI.rev.compl                                  ------------------------------------------------------------ 46364
human.FAM230A.ncRNA.18487127.18500594-GGTLC3.18516335.18518165.rev.compl.REF       ------------------------------------------------------------ 31039

chimp.LOC112206744-LOC107973052-GGT2.revcompl                                      ------------------------------------------------------------ 61762
chimp54546-135457.revcompl.Pan.troglodytes.clone.rp43-41g5.GenBank:AC099533.36     CTTGCAGTGAGCCGAGATCACGCCACTGCACTCCAACCTGGGCGACAGAGCGAGACTCTG 76371
human.FAM30B-LOC105372935-GGT2.NCBI.                                               cttgcggtgagccgagatcaggccactggaatccagcctgggcgacagagggagactccg 114852
human.FAM230E-LOC105377182-GGT3P.NCBI.                                             ------------------------------------------------------------ 57310
human.FAM230J-LOC105372942-GGTLC5P.NCBI.rev.compl                                  ------------------------------------------------------------ 46364
human.FAM230A.ncRNA.18487127.18500594-GGTLC3.18516335.18518165.rev.compl.REF       ------------------------------------------------------------ 31039

chimp.LOC112206744-LOC107973052-GGT2.revcompl                                      ------------------------------------------------------------ 61762
chimp54546-135457.revcompl.Pan.troglodytes.clone.rp43-41g5.GenBank:AC099533.36     ------------------------------------------------------------ 76371
human.FAM30B-LOC105372935-GGT2.NCBI.                                               tctcaaaaaaaaaaaaaagagtctggctctgttgcccaggctggagtgcagtggcgcga 114912
human.FAM230E-LOC105377182-GGT3P.NCBI.                                             ------------------------------------------------------------ 57310
human.FAM230J-LOC105372942-GGTLC5P.NCBI.rev.compl                                  ------------------------------------------------------------ 46364
human.FAM230A.ncRNA.18487127.18500594-GGTLC3.18516335.18518165.rev.compl.REF       ------------------------------------------------------------ 31039
```

```
chimp.LOC112206744-LOC107973052-GGT2.revcompl                                ------------------------------------------------------------ 61762
chimp54546-135457.revcompl.Pan.troglodytes.clone.rp43-41g5.GenBank:AC099533.36 ------------------------------------------------------------ 76371
human.FAM30B-LOC105372935-GGT2.NCBI.                                         tctcggcgcatcgcaatcccttccccgcccccgggttcaagtgattctccagtctcagcc 114972
human.FAM230E-LOC105377182-GGT3P.NCBI.                                       ------------------------------------------------------------ 57310
human.FAM230J-LOC105372942-GGTLC5P.NCBI.rev.compl                            ------------------------------------------------------------ 46364
human.FAM230A.ncRNA.18487127.18500594-GGTLC3.18516335.18518165.rev.compl.REF ------------------------------------------------------------ 31039

chimp.LOC112206744-LOC107973052-GGT2.revcompl                                ------------------------------------------------------------ 61762
chimp54546-135457.revcompl.Pan.troglodytes.clone.rp43-41g5.GenBank:AC099533.36 ------------------------------------------------------------ 76371
human.FAM30B-LOC105372935-GGT2.NCBI.                                         gccggagtagctgggactacaggcgcgtgccaccatgtctgactaaatttgtatttttac 115032
human.FAM230E-LOC105377182-GGT3P.NCBI.                                       ------------------------------------------------------------ 57310
human.FAM230J-LOC105372942-GGTLC5P.NCBI.rev.compl                            ------------------------------------------------------------ 46364
human.FAM230A.ncRNA.18487127.18500594-GGTLC3.18516335.18518165.rev.compl.REF ------------------------------------------------------------ 31039

chimp.LOC112206744-LOC107973052-GGT2.revcompl                                ------------------------------------------------------------ 61762
chimp54546-135457.revcompl.Pan.troglodytes.clone.rp43-41g5.GenBank:AC099533.36 ------------------------------------------------------------ 76371
human.FAM30B-LOC105372935-GGT2.NCBI.                                         tagagacggggtttcactatgttggccaggctggtctccaactcctgatctcgtgatccg 115092
human.FAM230E-LOC105377182-GGT3P.NCBI.                                       ------------------------------------------------------------ 57310
human.FAM230J-LOC105372942-GGTLC5P.NCBI.rev.compl                            ------------------------------------------------------------ 46364
human.FAM230A.ncRNA.18487127.18500594-GGTLC3.18516335.18518165.rev.compl.REF ------------------------------------------------------------ 31039

chimp.LOC112206744-LOC107973052-GGT2.revcompl                                ------------------------------------------------------------ 61762
chimp54546-135457.revcompl.Pan.troglodytes.clone.rp43-41g5.GenBank:AC099533.36 ------------------------------------------------------------ 76371
human.FAM30B-LOC105372935-GGT2.NCBI.                                         tctgccccgacctcccaaagtgctaggattataggcataagccaccacgcccggcctctt 115152
human.FAM230E-LOC105377182-GGT3P.NCBI.                                       ------------------------------------------------------------ 57310
human.FAM230J-LOC105372942-GGTLC5P.NCBI.rev.compl                            ------------------------------------------------------------ 46364
human.FAM230A.ncRNA.18487127.18500594-GGTLC3.18516335.18518165.rev.compl.REF ------------------------------------------------------------ 31039

chimp.LOC112206744-LOC107973052-GGT2.revcompl                                ------------------------------------------------------------ 61762
chimp54546-135457.revcompl.Pan.troglodytes.clone.rp43-41g5.GenBank:AC099533.36 ------------------------------------------------------------ 76371
human.FAM30B-LOC105372935-GGT2.NCBI.                                         tttttctttttcttttctttatctggagactgagttttgctcttgttgcccaggctgga 115212
human.FAM230E-LOC105377182-GGT3P.NCBI.                                       ------------------------------------------------------------ 57310
human.FAM230J-LOC105372942-GGTLC5P.NCBI.rev.compl                            ------------------------------------------------------------ 46364
human.FAM230A.ncRNA.18487127.18500594-GGTLC3.18516335.18518165.rev.compl.REF ------------------------------------------------------------ 31039

chimp.LOC112206744-LOC107973052-GGT2.revcompl                                ------------------------------------------------------------ 61762
chimp54546-135457.revcompl.Pan.troglodytes.clone.rp43-41g5.GenBank:AC099533.36 ------------------------------------------------------------ 76371
human.FAM30B-LOC105372935-GGT2.NCBI.                                         gtgcaatggtgcgatctcagctcactgcaatctccacctcagcaggagagcaggaatctt 115272
human.FAM230E-LOC105377182-GGT3P.NCBI.                                       ------------------------------------------------------------ 57310
human.FAM230J-LOC105372942-GGTLC5P.NCBI.rev.compl                            ------------------------------------------------------------ 46364
human.FAM230A.ncRNA.18487127.18500594-GGTLC3.18516335.18518165.rev.compl.REF ------------------------------------------------------------ 31039

chimp.LOC112206744-LOC107973052-GGT2.revcompl                                ------------------------------------------------------------ 61762
chimp54546-135457.revcompl.Pan.troglodytes.clone.rp43-41g5.GenBank:AC099533.36 ------------------------------------------------------------ 76371
human.FAM30B-LOC105372935-GGT2.NCBI.                                         cagtgatccacgggcagatctgccgccattgtgggcacctgtttctcccgcaacctttgt 115332
human.FAM230E-LOC105377182-GGT3P.NCBI.                                       ------------------------------------------------------------ 57310
human.FAM230J-LOC105372942-GGTLC5P.NCBI.rev.compl                            ------------------------------------------------------------ 46364
human.FAM230A.ncRNA.18487127.18500594-GGTLC3.18516335.18518165.rev.compl.REF ------------------------------------------------------------ 31039

chimp.LOC112206744-LOC107973052-GGT2.revcompl                                ------------------------------------------------------------ 61762
chimp54546-135457.revcompl.Pan.troglodytes.clone.rp43-41g5.GenBank:AC099533.36 ------------------------------------------------------------ 76371
human.FAM30B-LOC105372935-GGT2.NCBI.                                         gcccgcctctctccttcccgtacctattgcatgaccccccacgtccgcctcccgccattg 115392
human.FAM230E-LOC105377182-GGT3P.NCBI.                                       ------------------------------------------------------------ 57310
human.FAM230J-LOC105372942-GGTLC5P.NCBI.rev.compl                            ------------------------------------------------------------ 46364
human.FAM230A.ncRNA.18487127.18500594-GGTLC3.18516335.18518165.rev.compl.REF ------------------------------------------------------------ 31039

chimp.LOC112206744-LOC107973052-GGT2.revcompl                                ------------------------------------------------------------ 61762
chimp54546-135457.revcompl.Pan.troglodytes.clone.rp43-41g5.GenBank:AC099533.36 ------------------------------------------------------------ 76371
human.FAM30B-LOC105372935-GGT2.NCBI.                                         ccagcaagtgcctcgcgcgggtacctggctgcgcttattaatccgttaagctcgctctgt 115452
human.FAM230E-LOC105377182-GGT3P.NCBI.                                       ------------------------------------------------------------ 57310
human.FAM230J-LOC105372942-GGTLC5P.NCBI.rev.compl                            ------------------------------------------------------------ 46364
human.FAM230A.ncRNA.18487127.18500594-GGTLC3.18516335.18518165.rev.compl.REF ------------------------------------------------------------ 31039

chimp.LOC112206744-LOC107973052-GGT2.revcompl                                ------------------------------------------------------------ 61762
chimp54546-135457.revcompl.Pan.troglodytes.clone.rp43-41g5.GenBank:AC099533.36 ------------------------------------------------------------ 76371
human.FAM30B-LOC105372935-GGT2.NCBI.                                         cacgggcgccgtgatgtgctcacgcgcccgctccctcaggtttaaaaggcgcgttgcccg 115512
human.FAM230E-LOC105377182-GGT3P.NCBI.                                       ------------------------------------------------------------ 57310
human.FAM230J-LOC105372942-GGTLC5P.NCBI.rev.compl                            ------------------------------------------------------------ 46364
human.FAM230A.ncRNA.18487127.18500594-GGTLC3.18516335.18518165.rev.compl.REF ------------------------------------------------------------ 31039

chimp.LOC112206744-LOC107973052-GGT2.revcompl                                ------------------------------------------------------------ 61762
chimp54546-135457.revcompl.Pan.troglodytes.clone.rp43-41g5.GenBank:AC099533.36 ------------------------------------------------------------ 76371
human.FAM30B-LOC105372935-GGT2.NCBI.                                         gcaacagaagaaactgctggcttagccgttggccgagttggcggctggacgaggacgctc 115572
human.FAM230E-LOC105377182-GGT3P.NCBI.                                       ------------------------------------------------------------ 57310
human.FAM230J-LOC105372942-GGTLC5P.NCBI.rev.compl                            ------------------------------------------------------------ 46364
human.FAM230A.ncRNA.18487127.18500594-GGTLC3.18516335.18518165.rev.compl.REF ------------------------------------------------------------ 31039

chimp.LOC112206744-LOC107973052-GGT2.revcompl                                ------------------------------------------------------------ 61762
chimp54546-135457.revcompl.Pan.troglodytes.clone.rp43-41g5.GenBank:AC099533.36 ------------------------------------------------------------ 76371
human.FAM30B-LOC105372935-GGT2.NCBI.                                         agagcccagctctcgagagttcaagcaaccgacggttccccactgctcccaggagcggtt 115632
human.FAM230E-LOC105377182-GGT3P.NCBI.                                       ------------------------------------------------------------ 57310
human.FAM230J-LOC105372942-GGTLC5P.NCBI.rev.compl                            ------------------------------------------------------------ 46364
human.FAM230A.ncRNA.18487127.18500594-GGTLC3.18516335.18518165.rev.compl.REF ------------------------------------------------------------ 31039

chimp.LOC112206744-LOC107973052-GGT2.revcompl                                ------------------------------------------------------------ 61762
chimp54546-135457.revcompl.Pan.troglodytes.clone.rp43-41g5.GenBank:AC099533.36 ------------------------------------------------------------ 76371
human.FAM30B-LOC105372935-GGT2.NCBI.                                         acctgggcactctgtgcccctccttcctgttcgggcccaggccgaggacctgccagtagg 115692
human.FAM230E-LOC105377182-GGT3P.NCBI.                                       ------------------------------------------------------------ 57310
human.FAM230J-LOC105372942-GGTLC5P.NCBI.rev.compl                            ------------------------------------------------------------ 46364
human.FAM230A.ncRNA.18487127.18500594-GGTLC3.18516335.18518165.rev.compl.REF ------------------------------------------------------------ 31039

chimp.LOC112206744-LOC107973052-GGT2.revcompl                                ------------------------------------------------------------ 61762
chimp54546-135457.revcompl.Pan.troglodytes.clone.rp43-41g5.GenBank:AC099533.36 ------------------------------------------------------------ 76371
human.FAM30B-LOC105372935-GGT2.NCBI.                                         gctcagttgcctggagcccgttcagcccatcccccagttcactttgcttgtgggatctcc 115752
human.FAM230E-LOC105377182-GGT3P.NCBI.                                       ------------------------------------------------------------ 57310
human.FAM230J-LOC105372942-GGTLC5P.NCBI.rev.compl                            ------------------------------------------------------------ 46364
human.FAM230A.ncRNA.18487127.18500594-GGTLC3.18516335.18518165.rev.compl.REF ------------------------------------------------------------ 31039

chimp.LOC112206744-LOC107973052-GGT2.revcompl                                ------------------------------------------------------------ 61762
chimp54546-135457.revcompl.Pan.troglodytes.clone.rp43-41g5.GenBank:AC099533.36 ------------------------------------------------------------ 76371
human.FAM30B-LOC105372935-GGT2.NCBI.                                         ccgttgctcctgcccgtggactgagtggcaggccatcctacaagcacccggacacttgac 115812
human.FAM230E-LOC105377182-GGT3P.NCBI.                                       ------------------------------------------------------------ 57310
human.FAM230J-LOC105372942-GGTLC5P.NCBI.rev.compl                            ------------------------------------------------------------ 46364
human.FAM230A.ncRNA.18487127.18500594-GGTLC3.18516335.18518165.rev.compl.REF ------------------------------------------------------------ 31039

chimp.LOC112206744-LOC107973052-GGT2.revcompl                                ------------------------------------------------------------ 61762
chimp54546-135457.revcompl.Pan.troglodytes.clone.rp43-41g5.GenBank:AC099533.36 ------------------------------------------------------------ 76371
human.FAM30B-LOC105372935-GGT2.NCBI.                                         atcagtggtgtcaagacaactctaagaaggttttccgtgatcctgcaagccctgccttcc 115872
human.FAM230E-LOC105377182-GGT3P.NCBI.                                       ------------------------------------------------------------ 57310
human.FAM230J-LOC105372942-GGTLC5P.NCBI.rev.compl                            ------------------------------------------------------------ 46364
human.FAM230A.ncRNA.18487127.18500594-GGTLC3.18516335.18518165.rev.compl.REF ------------------------------------------------------------ 31039

chimp.LOC112206744-LOC107973052-GGT2.revcompl                                ------------------------------------------------------------ 61762
chimp54546-135457.revcompl.Pan.troglodytes.clone.rp43-41g5.GenBank:AC099533.36 ------------------------------------------------------------ 76371
human.FAM30B-LOC105372935-GGT2.NCBI.                                         ttcctgggatcctgccttcaatttgattgcacaggtaccacagcaagccagtgctgcgtg 115932
human.FAM230E-LOC105377182-GGT3P.NCBI.                                       ------------------------------------------------------------ 57310
human.FAM230J-LOC105372942-GGTLC5P.NCBI.rev.compl                            ------------------------------------------------------------ 46364
human.FAM230A.ncRNA.18487127.18500594-GGTLC3.18516335.18518165.rev.compl.REF ------------------------------------------------------------ 31039

chimp.LOC112206744-LOC107973052-GGT2.revcompl                                ------------------------------------------------------------ 61762
chimp54546-135457.revcompl.Pan.troglodytes.clone.rp43-41g5.GenBank:AC099533.36 ------------------------------------------------------------ 76371
human.FAM30B-LOC105372935-GGT2.NCBI.                                         ctccgagttccagggcgtcctccagctcagccactgcactgagaacatggactctctgtg 115992
human.FAM230E-LOC105377182-GGT3P.NCBI.                                       ------------------------------------------------------------ 57310
human.FAM230J-LOC105372942-GGTLC5P.NCBI.rev.compl                            ------------------------------------------------------------ 46364
human.FAM230A.ncRNA.18487127.18500594-GGTLC3.18516335.18518165.rev.compl.REF ------------------------------------------------------------ 31039

chimp.LOC112206744-LOC107973052-GGT2.revcompl                                ------------------------------------------------------------ 61762
```

```
chimp54546-135457.revcompl.Pan.troglodytes.clone.rp43-41g5.GenBank:AC099533.36      ------------------------------------------------------------ 76371
human.FAM30B-LOC105372935-GGT2.NCBI.                                                 gggcccaggagccgggagtcacccctttggggtccacaacagccggctgtccccagactt 116052
human.FAM230E-LOC105377182-GGT3P.NCBI.                                               ------------------------------------------------------------ 57310
human.FAM230J-LOC105372942-GGTLC5P.NCBI.rev.compl                                    ------------------------------------------------------------ 46364
human.FAM230A.ncRNA.18487127.18500594-GGTLC3.18516335.18518165.rev.compl.REF         ------------------------------------------------------------ 31039


chimp.LOC112206744-LOC107973052-GGT2.revcompl                                        ------------------------------------------------------------ 61762
chimp54546-135457.revcompl.Pan.troglodytes.clone.rp43-41g5.GenBank:AC099533.36      ------------------------------------------------------------ 76371
human.FAM30B-LOC105372935-GGT2.NCBI.                                                 gtgtccagggaagatagtgttgagggccctcaaggagagcggggcagggatgcctgagca 116112
human.FAM230E-LOC105377182-GGT3P.NCBI.                                               ------------------------------------------------------------ 57310
human.FAM230J-LOC105372942-GGTLC5P.NCBI.rev.compl                                    ------------------------------------------------------------ 46364
human.FAM230A.ncRNA.18487127.18500594-GGTLC3.18516335.18518165.rev.compl.REF         ------------------------------------------------------------ 31039


chimp.LOC112206744-LOC107973052-GGT2.revcompl                                        ---------  61762
chimp54546-135457.revcompl.Pan.troglodytes.clone.rp43-41g5.GenBank:AC099533.36      ---------  76371
human.FAM30B-LOC105372935-GGT2.NCBI.                                                 ggacaagga  116121
human.FAM230E-LOC105377182-GGT3P.NCBI.                                               ---------  57310
human.FAM230J-LOC105372942-GGTLC5P.NCBI.rev.compl                                    ---------  46364
human.FAM230A.ncRNA.18487127.18500594-GGTLC3.18516335.18518165.rev.compl.REF         ---------  31039
```
