# Supplementary figures and images for: Formation of human long intergenic non-coding RNA genes, pseudogenes, and protein genes: Ancestral sequences are key players
Source: PLoS One. 2020 Mar 26;15(3):e0230236. doi: 10.1371/journal.pone.0230236 (PMC7098633; doi:10.1371/journal.pone.0230236)

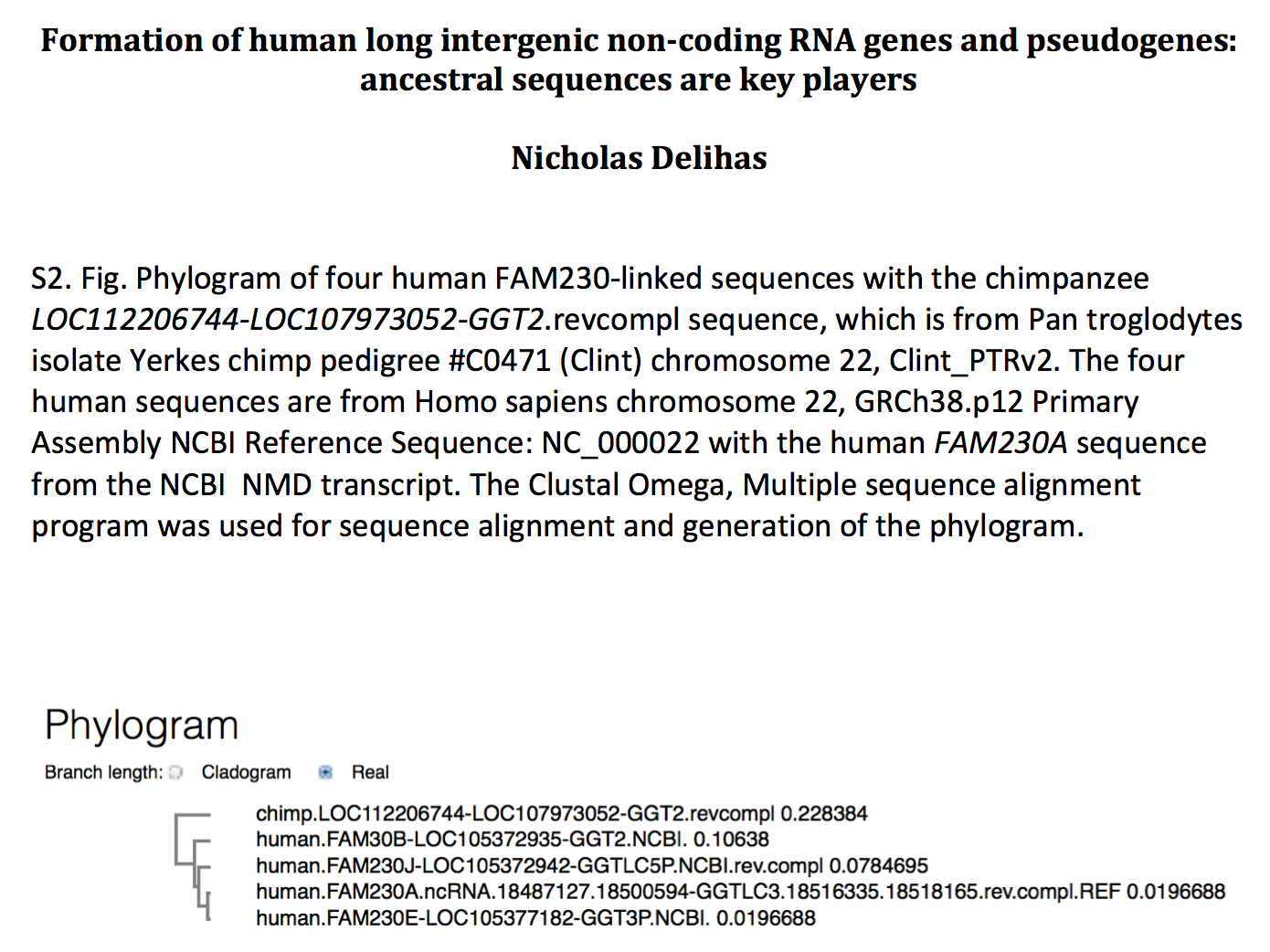

Supplement: S2 Fig — The four human sequences are from homo sapiens chromosome 22, GRDh38.p12 Primary Assembly NCBI reference Sequence: NC_000022 with the human FAM230A sequence from the NCBI NMD transcript. The Clustal Omega, Multiple sequence alignment program was used for sequence alignment and generation of the phylogram. (PNG) [file pone.0230236.s002.png]
