# Supplementary material for: Formation of human long intergenic non-coding RNA genes, pseudogenes, and protein genes: Ancestral sequences are key players
Source: PLoS One. 2020 Mar 26;15(3):e0230236. doi: 10.1371/journal.pone.0230236 (PMC7098633; doi:10.1371/journal.pone.0230236)
Supplement: S3 Fig — The Clustal Omega, Multiple sequence alignment program was used for sequence alignment (PDF) [file pone.0230236.s003.pdf]

Formation of human long intergenic non-coding RNA genes and pseudogenes: ancestral sequences are key players

Nicholas Delihas

Fig. 3S. nt alignment of chimpanzee *LOC112206744-LOC107973052-GGT2-* with human *FAM230B-LOC105372935-GGT2.* The Clustal Omega, Multiple sequence alignment program was used for sequence alignment.

```
CLUSTAL O(1.2.4) multiple sequence alignment


chimp.LOC112206744.LOC107973052.GGT2-.646279-708040.rev.compl      ------------------------------------------------------------   0
FAM230B-LOC105372935-GGT2.NCBI.GGT2.rev.compl                      agcagttggacctcacagtgtggattgtgccttcaccctggaatgtttatgccctatcac   60


chimp.LOC112206744.LOC107973052.GGT2-.646279-708040.rev.compl      ------------------------------------------------------------   0
FAM230B-LOC105372935-GGT2.NCBI.GGT2.rev.compl                      catggtgatgggattagggatctcctgcccttggtcctaagtgccactatctgtgctgag   120


chimp.LOC112206744.LOC107973052.GGT2-.646279-708040.rev.compl      ------------------------------------------------------------   0
FAM230B-LOC105372935-GGT2.NCBI.GGT2.rev.compl                      tttttcaaaggtcagagcagattgaaccattgtggtttcattttccctgattttgatttt   180


chimp.LOC112206744.LOC107973052.GGT2-.646279-708040.rev.compl      ----atggggaacctgtgtggctgcattcaaggtatgttcatactggcctgtcaaatgcg   56
FAM230B-LOC105372935-GGT2.NCBI.GGT2.rev.compl                      tcttatggggaacctgtgtggctgcattcaaggtatgttcatactggcctgtcaaatgcg   240
                                                                       ********************************************************

chimp.LOC112206744.LOC107973052.GGT2-.646279-708040.rev.compl      atcttttcaaattactagttaatgctttcaaaatatgttatttaaaaaattatcctctgt   116
FAM230B-LOC105372935-GGT2.NCBI.GGT2.rev.compl                      atcttttcaaattactagttaatgctttcaaaatatgttatttaaaaaattagcctctgt   300
                                                                   **************************************************** *******

chimp.LOC112206744.LOC107973052.GGT2-.646279-708040.rev.compl      attttccatatggagttataaatatgtttcatggttatgttttattcctcaatttatata   176
FAM230B-LOC105372935-GGT2.NCBI.GGT2.rev.compl                      attttccatatgcagttataaatatgtttcatgattatgttttattcctcaatttatata   360
                                                                   ************ ******************** **************************

chimp.LOC112206744.LOC107973052.GGT2-.646279-708040.rev.compl      tttgattattgtaccaagcagagtacctttgaaatttttcttcatttaaaaaatatgtct   236
FAM230B-LOC105372935-GGT2.NCBI.GGT2.rev.compl                      tttgattattgtaccaagcagagtatctttgaaatttttcttcatttaaaaaatatgtat   420
                                                                   ************************* ******************************** *

chimp.LOC112206744.LOC107973052.GGT2-.646279-708040.rev.compl      cttggctcaggcctgtaatcccagcactttgggaggccaatgcaagaggatcacaaggtg   296
FAM230B-LOC105372935-GGT2.NCBI.GGT2.rev.compl                      cttgactcaggcctgtaatcccagcactttgggaggccaaggcaagaggatcacaaggtg   480
                                                                   **** *********************************** *******************

chimp.LOC112206744.LOC107973052.GGT2-.646279-708040.rev.compl      aggagatcaagaccatcctggtcaatacagtgaaaccctgtctgtacgaaaagtacaaaa   356
FAM230B-LOC105372935-GGT2.NCBI.GGT2.rev.compl                      aggagatcaagaccatcctggccaatacagtgaaaccctgtctctactacaaatacaaaa   540
                                                                   ********************* ********************* *** * ** *******

chimp.LOC112206744.LOC107973052.GGT2-.646279-708040.rev.compl      aattagccaggaatggtggcagctggtgtagtcccagtgtgaattgggattcagtttatt   416
FAM230B-LOC105372935-GGT2.NCBI.GGT2.rev.compl                      atttagccaggcatggtggcagctggtgtagtcccagtgtgaattgggattcagtttatt   600
                                                                   * ********* ************************************************

chimp.LOC112206744.LOC107973052.GGT2-.646279-708040.rev.compl      cccaaattcccaaattatatatat-tatatatataatatattatatatacagtatatata   475
FAM230B-LOC105372935-GGT2.NCBI.GGT2.rev.compl                      cccaaattcccaaattatatatatatatatacatataaaata--------------------   640
                                                                   ************************ **** ****** ***

chimp.LOC112206744.LOC107973052.GGT2-.646279-708040.rev.compl      atatattatatatacagtatataatatactatgtatacagtatatattatatactgtatt   535
FAM230B-LOC105372935-GGT2.NCBI.GGT2.rev.compl                      ------------------------------------------------------------   640


chimp.LOC112206744.LOC107973052.GGT2-.646279-708040.rev.compl      atatacattatatactgtatacattatatactgtattatatattatatactgtatatata   595
FAM230B-LOC105372935-GGT2.NCBI.GGT2.rev.compl                      ------------------------------------------------------------   640


chimp.LOC112206744.LOC107973052.GGT2-.646279-708040.rev.compl      atatatattatatatacagtatatagtacatattatatatactatatgttatataaaata   655
FAM230B-LOC105372935-GGT2.NCBI.GGT2.rev.compl                      ------------------------------------------------------------   640


chimp.LOC112206744.LOC107973052.GGT2-.646279-708040.rev.compl      tataatataaaatatataatatataatatataatatataatatataatatgtataatata   715
FAM230B-LOC105372935-GGT2.NCBI.GGT2.rev.compl                      ------------------------------------------------------------   640


chimp.LOC112206744.LOC107973052.GGT2-.646279-708040.rev.compl      taatatataatatatagtatatatatcgtataatatatataatatatattatatataata   775
FAM230B-LOC105372935-GGT2.NCBI.GGT2.rev.compl                      ------------------------------------------------------------   640


chimp.LOC112206744.LOC107973052.GGT2-.646279-708040.rev.compl      tatattataaattataatttataatttatatatattatatatataataataaattataat   835
FAM230B-LOC105372935-GGT2.NCBI.GGT2.rev.compl                      ------------------------------------------------------------   640


chimp.LOC112206744.LOC107973052.GGT2-.646279-708040.rev.compl      tataaattataatttataatatatattatatataatatatattatatattataatttata   895
FAM230B-LOC105372935-GGT2.NCBI.GGT2.rev.compl                      ------------------------------------------------------------   640


chimp.LOC112206744.LOC107973052.GGT2-.646279-708040.rev.compl      atatacattatttatatataatatataatgtatattatatattatatatttaatatataa   955
FAM230B-LOC105372935-GGT2.NCBI.GGT2.rev.compl                      ------------------------------------------------------------   640


chimp.LOC112206744.LOC107973052.GGT2-.646279-708040.rev.compl      tatgttatatataatatataatatatattgtatataatatatattatattatatattata   1015
FAM230B-LOC105372935-GGT2.NCBI.GGT2.rev.compl                      ---catatataatatattaaatatattatatatataatatatattatattatatataatt   697
                                                                        ******  ****  *******  * *************************  **

chimp.LOC112206744.LOC107973052.GGT2-.646279-708040.rev.compl      atacataatataatatataatatattatatattatatatattatataatatattatatat   1075
FAM230B-LOC105372935-GGT2.NCBI.GGT2.rev.compl                      atatata-----------------------------------------------------   704
                                                                   *** ***

chimp.LOC112206744.LOC107973052.GGT2-.646279-708040.rev.compl      tatatatatataatatataatatattatatatatgttatatattatatatatatgtta   1135
FAM230B-LOC105372935-GGT2.NCBI.GGT2.rev.compl                      ------------------------------------------------------------   704


chimp.LOC112206744.LOC107973052.GGT2-.646279-708040.rev.compl      tatattatagaatatgttatatattatatattatagaatatgttatatattatatattat   1195
FAM230B-LOC105372935-GGT2.NCBI.GGT2.rev.compl                      ------------------------------------------------------------   704


chimp.LOC112206744.LOC107973052.GGT2-.646279-708040.rev.compl      ataatatgttatatattatatattatagaatatgttatatattatataatatgttatata   1255
FAM230B-LOC105372935-GGT2.NCBI.GGT2.rev.compl                      ----------------tatatatttgtgggtgccctatttcccgtctcataacttatttt   748
                                                                                   ********   *  *    *** *    * * ***  **** *

chimp.LOC112206744.LOC107973052.GGT2-.646279-708040.rev.compl      ttatatattatataatatgttat-------------------------------------   1278
FAM230B-LOC105372935-GGT2.NCBI.GGT2.rev.compl                      aagaagccagcataataatgtgtgggcttgggattcagtttttgaaacaaaacactgagc   808
                                                                       *      ******   * *

chimp.LOC112206744.LOC107973052.GGT2-.646279-708040.rev.compl      ------------------------------------------------------------   1278
FAM230B-LOC105372935-GGT2.NCBI.GGT2.rev.compl                      ctttgatgaccttcctgtacttgtaaaagcccacctgtctgcatggcagcagttggacct   868


chimp.LOC112206744.LOC107973052.GGT2-.646279-708040.rev.compl      ---------------------------------------------------------ata   1281
FAM230B-LOC105372935-GGT2.NCBI.GGT2.rev.compl                      cacagtgtggattgtgccttcaccctggaatgtttatgccctatcgccatggtgatggga   928
                                                                                                                              *

chimp.LOC112206744.LOC107973052.GGT2-.646279-708040.rev.compl      ttatatatattatataatatgttatatattatataatatgttatatattatatatattat   1341
FAM230B-LOC105372935-GGT2.NCBI.GGT2.rev.compl                      ttagggatctcctgcccttggtcctaagtgccactatctgtgctgagtttttcaaaggtc   988
                                                                   ***   ** *  *    *  **  **  *   *  ** ***  *   ** *  * *

chimp.LOC112206744.LOC107973052.GGT2-.646279-708040.rev.compl      ataatatgttatatattatatatattatataatatgttatatattatatattatataata   1401
FAM230B-LOC105372935-GGT2.NCBI.GGT2.rev.compl                      agagcagattgaaccattgtggtttcattttccctgattttgatttt-----------tc   1037
                                                                   * *  *  **  *   *     * * ** *    ** * *  *** *           *

chimp.LOC112206744.LOC107973052.GGT2-.646279-708040.rev.compl      tgttatatattatatatattatataatatgttatatattatatattatataatatgttat   1461
FAM230B-LOC105372935-GGT2.NCBI.GGT2.rev.compl                      ttatggggaacctgtgtggctgcattcaaggtatgttcatactggcctgtcaaatgcgat   1097
                                                                   *  *    *   * * *          * * *** *      *    * * * ***  **

chimp.LOC112206744.LOC107973052.GGT2-.646279-708040.rev.compl      atattatatatattatataatatgtt--atatattatatattatataatatgttatatat   1519
FAM230B-LOC105372935-GGT2.NCBI.GGT2.rev.compl                      cttttcaaattactagttaatgctttcaaaatatgttatttaaaaaattagcctctgtat   1157
                                                                    * **  *  ** **  ****   **  * ****  *** * * * * **   * * ***

chimp.LOC112206744.LOC107973052.GGT2-.646279-708040.rev.compl      tatatatattatataatatatatt--------atatcttatataatataatttatatatt   1571
FAM230B-LOC105372935-GGT2.NCBI.GGT2.rev.compl                      tttccatatgcagttataaatatgtttcatgattatgttttattcctcaatttatatatt   1217
                                                                   * *  ****    * *** ****          *** ** ***     ************

chimp.LOC112206744.LOC107973052.GGT2-.646279-708040.rev.compl      ata-tattatacaatatatattatatattatattatatattatacaatatatat------   1624
FAM230B-LOC105372935-GGT2.NCBI.GGT2.rev.compl                      tgattattgtaccaagcagagtatctttgaaatttttcttcatttaaaaaatatgtatct   1277
                                                                     * **** *** *   * * *** * * * *** *   * **  ** * ****

chimp.LOC112206744.LOC107973052.GGT2-.646279-708040.rev.compl      ------------------------------------------------------------   1624
FAM230B-LOC105372935-GGT2.NCBI.GGT2.rev.compl                      tgactcaggcctgtaatcccagcactttgggaggccaaggcaagaggatcacaaggtgag   1337
```

```
chimp.LOC112206744.LOC107973052.GGT2-.646279-708040.rev.compl  ------------------------------------------------------------   1624
FAM230B-LOC105372935-GGT2.NCBI.GGT2.rev.compl                  gagatcaagaccatcctggccaatacagtgaaaccctgtctctactacaaatacaaaaat   1397

chimp.LOC112206744.LOC107973052.GGT2-.646279-708040.rev.compl  ------------------------tatatatatattatatattatacaatatatattata   1660
FAM230B-LOC105372935-GGT2.NCBI.GGT2.rev.compl                  ttagccaggcatggtggcagctggtgtagtcccagtgtgaattgggattcagtttattca   1457
                                                                                       * **     * * *  ***       *  *  *  *

chimp.LOC112206744.LOC107973052.GGT2-.646279-708040.rev.compl  taatatataatatatattatattatataatatatatataatttccttttacatgctgcat   1720
FAM230B-LOC105372935-GGT2.NCBI.GGT2.rev.compl                  gtttattcccaaattcccaaattatat-atatatatataatttccttttacatcctgcat   1516
                                                                  ***     *  *   * ******* ************************* ******

chimp.LOC112206744.LOC107973052.GGT2-.646279-708040.rev.compl  ccttcaacattccatcccccaccccacagattaagttattccccaggggagaatatggca   1780
FAM230B-LOC105372935-GGT2.NCBI.GGT2.rev.compl                  ccttcaacgttacatcccccaccccacagattaagttattccccaggggagaatatggca   1576
                                                               ******** ** ************************************************

chimp.LOC112206744.LOC107973052.GGT2-.646279-708040.rev.compl  aactctattttaatgcagtttttagcctaattaagaa-ttatgaaatcattactttccaa   1839
FAM230B-LOC105372935-GGT2.NCBI.GGT2.rev.compl                  aagtctattttaatgcagtttttaacccaattaagaacctatgaaatcattactttccaa   1636
                                                               ** ********************* ** *********  *********************

chimp.LOC112206744.LOC107973052.GGT2-.646279-708040.rev.compl  atatttggaacaaagccacagtagtatgcatgggttggaggcttttcacagaataaaatg   1899
FAM230B-LOC105372935-GGT2.NCBI.GGT2.rev.compl                  aactttggaacaaagccacagtagtatggatccgttggaggcttttcacacaataaaatg   1696
                                                               *  ************************* **  ***************** *********

chimp.LOC112206744.LOC107973052.GGT2-.646279-708040.rev.compl  tacctatctttgtttttaacatgtttttcccttcctctcttctttctttgtgaaatgtgt   1959
FAM230B-LOC105372935-GGT2.NCBI.GGT2.rev.compl                  tacctctctttgtttttaacatgtttttcccttcctctcttctttttttgtgaaatgtgt   1756
                                                               ***** *************************************** **************

chimp.LOC112206744.LOC107973052.GGT2-.646279-708040.rev.compl  atttactttaataaatttgtagtaagtcacttccattcacatattaattttttaaagtaa   2019
FAM230B-LOC105372935-GGT2.NCBI.GGT2.rev.compl                  atttactttaatatatttgtagtaagtcacttccatgcacatattaattttttaaagtaa   1816
                                                               ************* ********************** ***********************

chimp.LOC112206744.LOC107973052.GGT2-.646279-708040.rev.compl  taagtatgtgtattgtctactagtgaaataaaacacacatttatttttattctttggaag   2079
FAM230B-LOC105372935-GGT2.NCBI.GGT2.rev.compl                  taagtatgtgtattgtctacgtgtgaaagaaaacacacatttatttttatgctttggaag   1876
                                                               ********************  ****** ********************* *********

chimp.LOC112206744.LOC107973052.GGT2-.646279-708040.rev.compl  ttatccagaatcatggcattgtcaatcacagtcaatcacccaacctactcacctttccag   2139
FAM230B-LOC105372935-GGT2.NCBI.GGT2.rev.compl                  ttatccagaatcatggaattgtcaatcacagtcaatcacccaacctactcacctttccag   1936
                                                               **************** *******************************************

chimp.LOC112206744.LOC107973052.GGT2-.646279-708040.rev.compl  tgtaatcttagtctaattttttttttgttatccaatgagatgcagtatttcaactcagag   2199
FAM230B-LOC105372935-GGT2.NCBI.GGT2.rev.compl                  tgtaatcttagtcaaattttttttttgttatccaatgagatgcagtatttcaactcagaa   1996
                                                               ************* *********************************************

chimp.LOC112206744.LOC107973052.GGT2-.646279-708040.rev.compl  agataaatagaatgaattggtagagactattaactaagaatatacagttttatttatgct   2259
FAM230B-LOC105372935-GGT2.NCBI.GGT2.rev.compl                  agataaatagagtgaatttatagagactattaactaagaacatacagttttatttatact   2056
                                                               *********** ******  ******************** **************** **

chimp.LOC112206744.LOC107973052.GGT2-.646279-708040.rev.compl  cagaagcaagtagattatgtatatatgtatgaagataaaaattaaaaggataattgtgta   2319
FAM230B-LOC105372935-GGT2.NCBI.GGT2.rev.compl                  cagaagcaagtagattatgtacatatatatgaagataaaaattaaaaggataattgtgta   2116
                                                               ********************* **** *********************************

chimp.LOC112206744.LOC107973052.GGT2-.646279-708040.rev.compl  aatttgcatgtagagagctttgaaaacctgtttacttgttaatgctgttttgatgtattg   2379
FAM230B-LOC105372935-GGT2.NCBI.GGT2.rev.compl                  aatttgcatgtagagggctttgaaaacctgtttacttgttaatgctgttttgatgtat--   2174
                                                               *************** ******************************************

chimp.LOC112206744.LOC107973052.GGT2-.646279-708040.rev.compl  tgtgtctttgttctcccgacccagcatccagagctctctgctggagctaagtgctcatca   2439
FAM230B-LOC105372935-GGT2.NCBI.GGT2.rev.compl                  tgtgtctttgttctcccgacccatcgtccagagctctctgcaggagctaagtgctcatca   2234
                                                               *********************** * *************** ******************

chimp.LOC112206744.LOC107973052.GGT2-.646279-708040.rev.compl  gttccatgacttggaaactgtctaagtttagaggcacttgtatttgttagtaaataagtc   2499
FAM230B-LOC105372935-GGT2.NCBI.GGT2.rev.compl                  gttccatgacttggaaactgtctaagtttagaggcacttgtatttgttagtaaataaggc   2294
                                                               ********************************************************** *

chimp.LOC112206744.LOC107973052.GGT2-.646279-708040.rev.compl  aagatgatattgtttcacaggctttagtgcagaagactgaatagagaagctgctccaccc   2559
FAM230B-LOC105372935-GGT2.NCBI.GGT2.rev.compl                  aagatgatattgtttcacaggttttagtgccgaagactgaatagataagctgctccaccc   2354
                                                               ********************* ******** ************** **************

chimp.LOC112206744.LOC107973052.GGT2-.646279-708040.rev.compl  agtacactggtgttcatttcatggtcatctcatctgttaaccatggatataaaacactta   2619
FAM230B-LOC105372935-GGT2.NCBI.GGT2.rev.compl                  agtacactggtgttcatttcatggtcatctcatctgttaaccatggatataaaacattta   2414
                                                               ******************************************************** ***

chimp.LOC112206744.LOC107973052.GGT2-.646279-708040.rev.compl  tcttcaatgatcttcctgtacatgtaaaaacacacctgtctgcatggcagcagttggacc   2679
FAM230B-LOC105372935-GGT2.NCBI.GGT2.rev.compl                  tcttcaat----------------------------------------------------   2422
                                                               ********

chimp.LOC112206744.LOC107973052.GGT2-.646279-708040.rev.compl  tcacagtgtggattgtgccttcaccctggaatgtctatgatgccctatcgaccatggtga   2739
FAM230B-LOC105372935-GGT2.NCBI.GGT2.rev.compl                  ------------------------------------------------------------   2422

chimp.LOC112206744.LOC107973052.GGT2-.646279-708040.rev.compl  tgggattagggatctcttcccctttgctcggccactgtctgtgccagccaggccactggg   2799
FAM230B-LOC105372935-GGT2.NCBI.GGT2.rev.compl                  ------------------------------------------------------------   2422

chimp.LOC112206744.LOC107973052.GGT2-.646279-708040.rev.compl  ccattgtggcggatggtgatgccctttgtgtggagcgcactgagtgtgcgctgaggcaga   2859
FAM230B-LOC105372935-GGT2.NCBI.GGT2.rev.compl                  ------------------------------------------------------------   2422

chimp.LOC112206744.LOC107973052.GGT2-.646279-708040.rev.compl  cacagtacttttgacaactttatctgcttctttcaaaactgggtctgcattatgataaag   2919
FAM230B-LOC105372935-GGT2.NCBI.GGT2.rev.compl                  ------------------------------------------------------------   2422

chimp.LOC112206744.LOC107973052.GGT2-.646279-708040.rev.compl  tggtcggttttaggccaagcaggtgtatgtcatagagcaatgacttgggagttcagaatc   2979
FAM230B-LOC105372935-GGT2.NCBI.GGT2.rev.compl                  ------------------------------------------------------------   2422

chimp.LOC112206744.LOC107973052.GGT2-.646279-708040.rev.compl  ctgggcgtgaataagcctctgagccttattaagctgtgaaatctaaagcaagacatttca   3039
FAM230B-LOC105372935-GGT2.NCBI.GGT2.rev.compl                  ------------------------------------------------------------   2422

chimp.LOC112206744.LOC107973052.GGT2-.646279-708040.rev.compl  ctgataattaagcctcatgagcacttatactgtacactttacctacatcaaatcattcgg   3099
FAM230B-LOC105372935-GGT2.NCBI.GGT2.rev.compl                  ------------------------------------------------------------   2422

chimp.LOC112206744.LOC107973052.GGT2-.646279-708040.rev.compl  ccatcaccacaaacaaatggcatgattgatttactatcacctttcgctgggtagaaaaag   3159
FAM230B-LOC105372935-GGT2.NCBI.GGT2.rev.compl                  ------------------------------------------------------------   2422

chimp.LOC112206744.LOC107973052.GGT2-.646279-708040.rev.compl  acagagatgaatgtgcccaagcttatgagcccctcagcaaaagagccaagatgggaaccc   3219
FAM230B-LOC105372935-GGT2.NCBI.GGT2.rev.compl                  ------------------------------------------------------------   2422

chimp.LOC112206744.LOC107973052.GGT2-.646279-708040.rev.compl  aagcatacagccccaatgctgaggctctgaactactgacctgccctcagcactcagcctt   3279
FAM230B-LOC105372935-GGT2.NCBI.GGT2.rev.compl                  ------------------------------------------------------------   2422

chimp.LOC112206744.LOC107973052.GGT2-.646279-708040.rev.compl  gggatcatgagtcactgtgcaagggagttccaacatctgcatgtatgtctggaatgatct   3339
FAM230B-LOC105372935-GGT2.NCBI.GGT2.rev.compl                  ------------------------------------------------------------   2422

chimp.LOC112206744.LOC107973052.GGT2-.646279-708040.rev.compl  gagcctgtagagttcctacacactggccacattatagggtggtgtctgtggtcacacagc   3399
FAM230B-LOC105372935-GGT2.NCBI.GGT2.rev.compl                  ------------------------------------------------------------   2422

chimp.LOC112206744.LOC107973052.GGT2-.646279-708040.rev.compl  tcagggcaggtattagtacatgaatagcttagatgtgtcatagtctttatgtgaaaggca   3459
FAM230B-LOC105372935-GGT2.NCBI.GGT2.rev.compl                  ------------------------------------------------------------   2422

chimp.LOC112206744.LOC107973052.GGT2-.646279-708040.rev.compl  cataaaagcccaaatgcttttgaactggttgcggattttgagttggaggacaacacttgg   3519
FAM230B-LOC105372935-GGT2.NCBI.GGT2.rev.compl                  ------------------------------------------------------------   2422

chimp.LOC112206744.LOC107973052.GGT2-.646279-708040.rev.compl  gacaaccggtcacattccttcagtgcgcggccactcctcagcacatatcccaaagccggt   3579
FAM230B-LOC105372935-GGT2.NCBI.GGT2.rev.compl                  ------------------------------------------------------------   2422

chimp.LOC112206744.LOC107973052.GGT2-.646279-708040.rev.compl  aaataatgggaagaccctgtactgatgtttgatggtgttatgaacttcaaatgacccta   3639
FAM230B-LOC105372935-GGT2.NCBI.GGT2.rev.compl                  ------------------------------------------------------------   2422

chimp.LOC112206744.LOC107973052.GGT2-.646279-708040.rev.compl  tagaaatgttaaaaaaaaaaaaaaaaaaagtctgtgagctacacaacctggggacaag   3699
FAM230B-LOC105372935-GGT2.NCBI.GGT2.rev.compl                  ------------------------------------------------------------   2422
```

```
chimp.LOC112206744.LOC107973052.GGT2-.646279-708040.rev.compl      acaagtaagaacacaaaataccagtaaataatgagcctcatgtgtcaccctgcttgcaac   3759
FAM230B-LOC105372935-GGT2.NCBI.GGT2.rev.compl                      ------------------------------------------------------------   2422

chimp.LOC112206744.LOC107973052.GGT2-.646279-708040.rev.compl      tccctcggcagctggtttgaagagcctcagtgctcaccaggggccatctccccgctctgc   3819
FAM230B-LOC105372935-GGT2.NCBI.GGT2.rev.compl                      ------------------------------------------------------------   2422

chimp.LOC112206744.LOC107973052.GGT2-.646279-708040.rev.compl      cctctctcgctgtgggttgcctgggctgaacataggcagcccctcacatgatgactgcaa   3879
FAM230B-LOC105372935-GGT2.NCBI.GGT2.rev.compl                      ------------------------------------------------------------   2422

chimp.LOC112206744.LOC107973052.GGT2-.646279-708040.rev.compl      acacagggcaaaggaagactgaacggaaaatgtatggttatagtggttattttaaaatat   3939
FAM230B-LOC105372935-GGT2.NCBI.GGT2.rev.compl                      ------------------------------------------------------------   2422

chimp.LOC112206744.LOC107973052.GGT2-.646279-708040.rev.compl      aagtatttcagtattttctaatctttaataattgattattttaggggaggtttttttt   3999
FAM230B-LOC105372935-GGT2.NCBI.GGT2.rev.compl                      ------------------------------------------------------------   2422

chimp.LOC112206744.LOC107973052.GGT2-.646279-708040.rev.compl      tttttttttgagacagagtcttgcgctgttgcccaggctggagtgcagtggcatgatctc   4059
FAM230B-LOC105372935-GGT2.NCBI.GGT2.rev.compl                      ------------------------------------------------------------   2422

chimp.LOC112206744.LOC107973052.GGT2-.646279-708040.rev.compl      agctcactgcaacctctacctcctgtgttcaagcgattctcctgcctcagcctcccgagt   4119
FAM230B-LOC105372935-GGT2.NCBI.GGT2.rev.compl                      ------------------------------------------------------------   2422

chimp.LOC112206744.LOC107973052.GGT2-.646279-708040.rev.compl      agctgggactacaggcacgtgccaccacgcctggcaaagtttttgtgtttttagtagaga   4179
FAM230B-LOC105372935-GGT2.NCBI.GGT2.rev.compl                      ------------------------------------------------------------   2422

chimp.LOC112206744.LOC107973052.GGT2-.646279-708040.rev.compl      cggggtttcactctgttagccaggatggtctcaatcttctgacctcatgatccacccgcc   4239
FAM230B-LOC105372935-GGT2.NCBI.GGT2.rev.compl                      ------------------------------------------------------------   2422

chimp.LOC112206744.LOC107973052.GGT2-.646279-708040.rev.compl      tcggcctcccaaagtgctgggattacaggtgtgagccaccacacctggcctgatttttta   4299
FAM230B-LOC105372935-GGT2.NCBI.GGT2.rev.compl                      ------------------------------------------------------------   2422

chimp.LOC112206744.LOC107973052.GGT2-.646279-708040.rev.compl      tttttttatttttgagatgaagtctctgtcacccaggctggagtgcagtggtgcaatct   4359
FAM230B-LOC105372935-GGT2.NCBI.GGT2.rev.compl                      ------------------------------------------------------------   2422

chimp.LOC112206744.LOC107973052.GGT2-.646279-708040.rev.compl      cagctcactgcaatctctgcctccaggttcaagggattgtcctgcctcagcctcctgagt   4419
FAM230B-LOC105372935-GGT2.NCBI.GGT2.rev.compl                      ------------------------------------------------------------   2422

chimp.LOC112206744.LOC107973052.GGT2-.646279-708040.rev.compl      agctgggttttttttttttttagttgagatggggttttaccatgttggtcaggctggtct   4479
FAM230B-LOC105372935-GGT2.NCBI.GGT2.rev.compl                      ---------------------------gatggggttttaccatgttggtcaggctggtct   2455
                                                                                              *********************************

chimp.LOC112206744.LOC107973052.GGT2-.646279-708040.rev.compl      cgaactcctgacctcaaatgatccgcccacctccaccttccaaactgctgggattacagg   4539
FAM230B-LOC105372935-GGT2.NCBI.GGT2.rev.compl                      cgaactcctgacctcaaatgatccacccacctccaccttccaaactgctgggattacagg   2515
                                                                   ************************ ***********************************

chimp.LOC112206744.LOC107973052.GGT2-.646279-708040.rev.compl      tgtgagccactatgcctggctgattattttcataaccaagaaaagaaataaatataatta   4599
FAM230B-LOC105372935-GGT2.NCBI.GGT2.rev.compl                      tgtgagccactatgcctgactgattattttcataaccaagaaaagaaataaatacaatta   2575
                                                                   ***************** ************************************ *****

chimp.LOC112206744.LOC107973052.GGT2-.646279-708040.rev.compl      atgctggtgcatggtattaaatctagtttttaaaaaattcacacataaaccaggcagaac   4659
FAM230B-LOC105372935-GGT2.NCBI.GGT2.rev.compl                      atgctggtgcatggtattaaatctagtttttaaaaaattcacacataaacaaggcagaac   2635
                                                                   ************************************************** *********

chimp.LOC112206744.LOC107973052.GGT2-.646279-708040.rev.compl      cctataccctccatgataaatgcagtagcagtgtatgtgggtctgtggaggttgaaaggg   4719
FAM230B-LOC105372935-GGT2.NCBI.GGT2.rev.compl                      cctataccctccatgataaatgcagtagcagtgtatgtgggtctgtggaggttgaaaggg   2695
                                                                   ************************************************************

chimp.LOC112206744.LOC107973052.GGT2-.646279-708040.rev.compl      acttggtagatgtcaagaaggtagtggcagtcctgctgggcttttaaagggtctgaagaa   4779
FAM230B-LOC105372935-GGT2.NCBI.GGT2.rev.compl                      acttggtagatgtcaagaagctagtggcagtcctgctgggcttttaaagggtctgaagaa   2755
                                                                   ******************** ***************************************

chimp.LOC112206744.LOC107973052.GGT2-.646279-708040.rev.compl      gtgacaggatgctgtggttgaatcctagcatgtattttagcatttgttcatttggagttt   4839
FAM230B-LOC105372935-GGT2.NCBI.GGT2.rev.compl                      gtgacaggatgctgtggttgaatcctagcatgtattttagcatttgttcatttggag-tt   2814
                                                                   ********************************************************* **

chimp.LOC112206744.LOC107973052.GGT2-.646279-708040.rev.compl      gattatttcacgttgctttcatttgccattacctggaaagccaagggctctactctcatt   4899
FAM230B-LOC105372935-GGT2.NCBI.GGT2.rev.compl                      gattatttcacgttgctttcatttgccattacctggaaagccaagggctctactctcatt   2874
                                                                   ************************************************************

chimp.LOC112206744.LOC107973052.GGT2-.646279-708040.rev.compl      tccttgctgctctttctttgccttccttggtccatgaagaagatggtccaggagaagctc   4959
FAM230B-LOC105372935-GGT2.NCBI.GGT2.rev.compl                      tccttgctgctctttctttgccttccttggtccgtgaagaagatggtccaggagaagctc   2934
                                                                   ********************************* **************************

chimp.LOC112206744.LOC107973052.GGT2-.646279-708040.rev.compl      attccatgcttgttaaccaggcacacccctaagttccagtccctgagtcattcatgagta   5019
FAM230B-LOC105372935-GGT2.NCBI.GGT2.rev.compl                      attccatgcttgttaaccaggcacgcccctaagttccagtccctgagtcattcatgagta   2994
                                                                   ************************ ***********************************

chimp.LOC112206744.LOC107973052.GGT2-.646279-708040.rev.compl      gcactgccaatgaactgacagccatgctgtgtccctccacatcccctaggtgactcgaag   5079
FAM230B-LOC105372935-GGT2.NCBI.GGT2.rev.compl                      gcactgccaatgaactgacagccatgctgtgtccctccacatcccctaggtgactcgaag   3054
                                                                   ************************************************************

chimp.LOC112206744.LOC107973052.GGT2-.646279-708040.rev.compl      aagccttccaaaaagcatgtgaaaagggagccctcctctactaccaaggtaaagtagcct   5139
FAM230B-LOC105372935-GGT2.NCBI.GGT2.rev.compl                      aagccttccaaaaagcatgtgaaaaggaagccctactctactaccaaggtaaagtagcct   3114
                                                                   *************************** ****** *************************

chimp.LOC112206744.LOC107973052.GGT2-.646279-708040.rev.compl      gtctttgcctaagatgtaaatgttgttttcttggatcctttatttttcagttgatatctg   5199
FAM230B-LOC105372935-GGT2.NCBI.GGT2.rev.compl                      gtctttgcctaagatgtaaatgttgttttcttggatcctttatttttcagttgatatcag   3174
                                                                   ********************************************************** *

chimp.LOC112206744.LOC107973052.GGT2-.646279-708040.rev.compl      ctatgggaaaattatccactacattatagatgttagataatatttccttggggatggagg   5259
FAM230B-LOC105372935-GGT2.NCBI.GGT2.rev.compl                      ctatgggaaaattctccactacattataggtgttagataatatttccttggggatggagg   3234
                                                                   ************* *************** ******************************

chimp.LOC112206744.LOC107973052.GGT2-.646279-708040.rev.compl      aggtgtattttaccaactgacacctgattccagaggatgtgcaaaattggcagtgtcaga   5319
FAM230B-LOC105372935-GGT2.NCBI.GGT2.rev.compl                      aggtgtattttaccaactgacacctgattccagaggacgtgcaaaattggcagtgtcaga   3294
                                                                   ************************************ ***********************

chimp.LOC112206744.LOC107973052.GGT2-.646279-708040.rev.compl      tagtacactgcgtgttaagggatgttttcttcaggaacaagctttccactttagataaga   5379
FAM230B-LOC105372935-GGT2.NCBI.GGT2.rev.compl                      tagtacactgggtgttaagggatgttttcttcaggaacaagctttccactttagataaga   3354
                                                                   ********** *************************************************

chimp.LOC112206744.LOC107973052.GGT2-.646279-708040.rev.compl      attctgcaattgctactcaaaaattacctagacagaaacattcttcaagaaaagctcctg   5439
FAM230B-LOC105372935-GGT2.NCBI.GGT2.rev.compl                      attctgcaattgctactcaaaaattacctagacagaaacattcttcaagaaaagctcctg   3414
                                                                   ************************************************************

chimp.LOC112206744.LOC107973052.GGT2-.646279-708040.rev.compl      tgctttcctaagggaactctactctagagttggggcttttgacttgaaccttatttccaa   5499
FAM230B-LOC105372935-GGT2.NCBI.GGT2.rev.compl                      tgctttcctaagggaactctactctagagttggggcttttgacttgaaccttatttccag   3474
                                                                   ***********************************************************

chimp.LOC112206744.LOC107973052.GGT2-.646279-708040.rev.compl      tcttggttacccagagtttccaagtgaacaaaagacctgtgtgagccatccatagcatag   5559
FAM230B-LOC105372935-GGT2.NCBI.GGT2.rev.compl                      tcttggttacccagagtttccaagtgaacaaaagacctgtgtgagccatccatagcatag   3534
                                                                   ************************************************************

chimp.LOC112206744.LOC107973052.GGT2-.646279-708040.rev.compl      cctgattctcagagtgttttccttctctaattacaggtgacttcagggagcacattcaat   5619
FAM230B-LOC105372935-GGT2.NCBI.GGT2.rev.compl                      cctgattctcagagtgttttccttctctaattacaggtgacttcagggagcacattcaat   3594
                                                                   ************************************************************

chimp.LOC112206744.LOC107973052.GGT2-.646279-708040.rev.compl      ggtacgtattctggaatcactcactggttgttagaaaaggattctataggaaatctggag   5679
FAM230B-LOC105372935-GGT2.NCBI.GGT2.rev.compl                      ggtacgtattctggaatcactcactggttgttagaaaaggattctacaggaaatctggag   3654
                                                                   ********************************************** *************

chimp.LOC112206744.LOC107973052.GGT2-.646279-708040.rev.compl      cttaactgctggcttttgtctagagaggctccatgatccaagacatctggtgggaatgag   5739
FAM230B-LOC105372935-GGT2.NCBI.GGT2.rev.compl                      cttaactgctggcttttgtctggagagcctccatgatccaagacatctggtgggaatgag   3714
                                                                   ********************* ***** ********************************

chimp.LOC112206744.LOC107973052.GGT2-.646279-708040.rev.compl      gatgtgggatatagtaaaggaaactggttttccaggtgacatactctttttatctatgta   5799
FAM230B-LOC105372935-GGT2.NCBI.GGT2.rev.compl                      gatgtagggtatagtaaaagaaactggttttcctggtgacatactctttttatctatgta   3774
                                                                   ***** ** ********* ************** **************************

chimp.LOC112206744.LOC107973052.GGT2-.646279-708040.rev.compl      tagtttctgggaatgtgttcacattaggttgtgtgtgggtatgtgtgtattagggcgggg   5859
FAM230B-LOC105372935-GGT2.NCBI.GGT2.rev.compl                      tagtttctgggaacatgttcacattaggttgtgtgtgggtatgtgtgtattagggcgggg   3834
                                                                   *************  *********************************************

chimp.LOC112206744.LOC107973052.GGT2-.646279-708040.rev.compl      gtgggatgaggtggtctgtgtgcacatctgcatgatttgcttgtgaatgtgtgtctatgt   5919
```

```
FAM230B-LOC105372935-GGT2.NCBI.GGT2.rev.compl                        gtggggtgaggtggtctgtgtgcaagtctgcatgatttgcttgtgaatgtgtgtctatgt   3894
                                                                     ***** ******************  *********************************

chimp.LOC112206744.LOC107973052.GGT2-.646279-708040.rev.compl        gtgtttcccccaggaaaaaaatgttgtgtttacccagcacaactctcagtgccatttttc   5979
FAM230B-LOC105372935-GGT2.NCBI.GGT2.rev.compl                        gtgtttcccctaggaaaaaaatgttgtgtttacccagcacaactctcagtgccatttttc   3954
                                                                     ********** *************************************************

chimp.LOC112206744.LOC107973052.GGT2-.646279-708040.rev.compl        ttaatttaacaaatcagaccacatactttacttacattagttcacacctcatcatcatca   6039
FAM230B-LOC105372935-GGT2.NCBI.GGT2.rev.compl                        ttaatttaacaaatcagaccacatactttacttacattagttcacacctcatcatcatca   4014
                                                                     ************************************************************

chimp.LOC112206744.LOC107973052.GGT2-.646279-708040.rev.compl        tgcccatatgttgtgagcttgtttattgagcccacatgccagatggagaaactaagccac   6099
FAM230B-LOC105372935-GGT2.NCBI.GGT2.rev.compl                        tgcccatatgttgtgagcttgtttattgagcccacatgccagatggagaaactcagccac   4074
                                                                     ***************************************************** ******

chimp.LOC112206744.LOC107973052.GGT2-.646279-708040.rev.compl        ataaataaatgtgccctggttcacttgctgcatagtgaagagtcaaaatgtttactcata   6159
FAM230B-LOC105372935-GGT2.NCBI.GGT2.rev.compl                        ataaataaatgtgccctggttcacttgctgcatagtgaagagtcaaaatgtttactcata   4134
                                                                     ************************************************************

chimp.LOC112206744.LOC107973052.GGT2-.646279-708040.rev.compl        cggtgctaatgttgaaggcctgaactacaacctctatttatcagccagtgaagagatcac   6219
FAM230B-LOC105372935-GGT2.NCBI.GGT2.rev.compl                        cggtgctaatgttgaaggcctgaactacaacctctatttatcagccagtgaagagatcac   4194
                                                                     ************************************************************

chimp.LOC112206744.LOC107973052.GGT2-.646279-708040.rev.compl        tattcaccatgcaagggagttccagcaccctctatgcctggaattacccacgcctgcaga   6279
FAM230B-LOC105372935-GGT2.NCBI.GGT2.rev.compl                        tattccccatgcaagggagttccagcaccctctatgcctggaattacccacacctgcaga   4254
                                                                     ***** ********************************************* ********

chimp.LOC112206744.LOC107973052.GGT2-.646279-708040.rev.compl        gatcccaaacgccatccctcacataagagagcctcatgatctcataatccaggtagctat   6339
FAM230B-LOC105372935-GGT2.NCBI.GGT2.rev.compl                        gatcccaaacgccatccctcacataagacagcctcatgatctcataatccaggtagctat   4314
                                                                     **************************** *******************************

chimp.LOC112206744.LOC107973052.GGT2-.646279-708040.rev.compl        gtagacatcttcctgcaggtgtcacatagtccttagtgggaaaccaacatagaaagccca   6399
FAM230B-LOC105372935-GGT2.NCBI.GGT2.rev.compl                        gtagacatcttcctgcaggtgtcacatagtccttagtgtgaaaccaacatagaaagccca   4374
                                                                     ************************************** *********************

chimp.LOC112206744.LOC107973052.GGT2-.646279-708040.rev.compl        tgtttctgatcaaatcacaggttctgaaacactaagggaagcactaagtaggacaatgtg   6459
FAM230B-LOC105372935-GGT2.NCBI.GGT2.rev.compl                        tgtttctgatcaaatcacaggttctgaaacactaagggaggcactaagtaggacaacgtg   4434
                                                                     *************************************** **************** ***

chimp.LOC112206744.LOC107973052.GGT2-.646279-708040.rev.compl        gtgcctgtgtgtcatagctgggtctcctcaagacatggatcaagtccaataagaattggg   6519
FAM230B-LOC105372935-GGT2.NCBI.GGT2.rev.compl                        gtgcctgcgtgtcatagctgggtctcctcaagacatggatcaagtccagtaagaattggg   4494
                                                                     ******* **************************************** ***********

chimp.LOC112206744.LOC107973052.GGT2-.646279-708040.rev.compl        gagatgctttagagtcttgatggagttatcaccacaagccctctgagctacacactttag   6579
FAM230B-LOC105372935-GGT2.NCBI.GGT2.rev.compl                        gagatgctttagagtcttgatggagttatcaccacaagccctctgagctacacactttag   4554
                                                                     ************************************************************

chimp.LOC112206744.LOC107973052.GGT2-.646279-708040.rev.compl        ggatcatgaccattaagtactcaaattaccacttggttgttatccgggtatccgtcgtcc   6639
FAM230B-LOC105372935-GGT2.NCBI.GGT2.rev.compl                        ggatcatgaccattaagtactcaaattaccatttggttgctatccgggtatccgtcgtcc   4614
                                                                     ******************************* ******* ********************

chimp.LOC112206744.LOC107973052.GGT2-.646279-708040.rev.compl        ttgtggcaactctcttgtgaagctggtgtggacagcctcagtgctggagctgtgcctgcc   6699
FAM230B-LOC105372935-GGT2.NCBI.GGT2.rev.compl                        ttgtggcaaccctcttgtgaagctggtgtggacagcctcagtgctggagctgtgcctgcc   4674
                                                                     ********** *************************************************

chimp.LOC112206744.LOC107973052.GGT2-.646279-708040.rev.compl        ttctgaatggaccctttctgtgttagcaggtgggtacaagcatgggggtcagcacactca   6759
FAM230B-LOC105372935-GGT2.NCBI.GGT2.rev.compl                        ttctgagtggaccctttctgtgttagcaggtgggtacaagcgtgggggtcagcacactca   4734
                                                                     ****** ********************************** ******************

chimp.LOC112206744.LOC107973052.GGT2-.646279-708040.rev.compl        gtggatttacacacacagcattgaagagtaaggctgggcttcattatttatacattttca   6819
FAM230B-LOC105372935-GGT2.NCBI.GGT2.rev.compl                        gtggatttacacacacagcgttgaagagtaaggctgggcttcattatttatacattttca   4794
                                                                     ******************* ****************************************

chimp.LOC112206744.LOC107973052.GGT2-.646279-708040.rev.compl        ataaatgatgatcttcataacataaaatcaatgacgtagtacactagaatactgtcccta   6879
FAM230B-LOC105372935-GGT2.NCBI.GGT2.rev.compl                        ataaatgatgatcttcataacataaaatcaatgatgtagtacactagaatactgtcccta   4854
                                                                     ********************************** *************************

chimp.LOC112206744.LOC107973052.GGT2-.646279-708040.rev.compl        gtattgaatcttgtctctcaacaaagggttgcttaaagtcacatgacagattccattcaa   6939
FAM230B-LOC105372935-GGT2.NCBI.GGT2.rev.compl                        gtattgaatcttgtctctcagcaaagggttgcttaaagtcacgtgacagattccattcaa   4914
                                                                     ******************** ********************* *****************

chimp.LOC112206744.LOC107973052.GGT2-.646279-708040.rev.compl        ctgatgacacatgctgtagcagcagttaaagcagtcatttgaaaaggcttttactataaa   6999
FAM230B-LOC105372935-GGT2.NCBI.GGT2.rev.compl                        ctgatgacacatgctgtagcagcagttaaagcagtcatttgaaaaggcttttactataaa   4974
                                                                     ************************************************************

chimp.LOC112206744.LOC107973052.GGT2-.646279-708040.rev.compl        cttatgtgtgagcctgaagtgggggataaaagaggtgattagctcccctgtgccatgttt   7059
FAM230B-LOC105372935-GGT2.NCBI.GGT2.rev.compl                        cttacgtgtgagcctgaagtgggggataaaagaggcgattagctcccctgtgccatgttt   5034
                                                                     **** ****************************** ************************

chimp.LOC112206744.LOC107973052.GGT2-.646279-708040.rev.compl        ctattatgtgcatggtggaggaaaattacacaggaaggtgatggagagaacagagcaaag   7119
FAM230B-LOC105372935-GGT2.NCBI.GGT2.rev.compl                        ctattatgtgcgtggtggaggaaaattacacaggaaggtgatggagagaacagagcaaag   5094
                                                                     *********** ************************************************

chimp.LOC112206744.LOC107973052.GGT2-.646279-708040.rev.compl        gattggacaggtccattgaacccataagactatggtgaggttagtgaatgagattggtca   7179
FAM230B-LOC105372935-GGT2.NCBI.GGT2.rev.compl                        gattggacaggtccattgaacccataagactatggtgaggttagtgaatgagattggtca   5154
                                                                     ************************************************************

chimp.LOC112206744.LOC107973052.GGT2-.646279-708040.rev.compl        ttttaggtcaaattctacccagagctggtgcagccactgcccattcttagccagacctta   7239
FAM230B-LOC105372935-GGT2.NCBI.GGT2.rev.compl                        ttttaggtcaaatttacccagagctggtgcagccactgcccattcttagccagacctta   5214
                                                                     ************* **********************************************

chimp.LOC112206744.LOC107973052.GGT2-.646279-708040.rev.compl        ttgcaggcagctctgatcaatagtcaaggaggcagtggggcttgcagacttaattcatta   7299
FAM230B-LOC105372935-GGT2.NCBI.GGT2.rev.compl                        ttgcaggcagctctgatcaatagtcaaggaggcagtggggcttgcagacttaattcatta   5274
                                                                     ************************************************************

chimp.LOC112206744.LOC107973052.GGT2-.646279-708040.rev.compl        aatcaccaaagcaccagcccacacggccacttttccagttaattcacagtagcttgcata   7359
FAM230B-LOC105372935-GGT2.NCBI.GGT2.rev.compl                        aatcaccaaagcaccagcccaaacggccacttttccagttaattgacagtagcttgcata   5334
                                                                     ********************* ********************** ***************

chimp.LOC112206744.LOC107973052.GGT2-.646279-708040.rev.compl        ttcaggtttgatcagtggaagggaagttactctttgcagacccatcttttgacaatcatt   7419
FAM230B-LOC105372935-GGT2.NCBI.GGT2.rev.compl                        ttcaggtttgatcagtggaagggaagttactctttgcagacccatcttttgacaatcatt   5394
                                                                     ************************************************************

chimp.LOC112206744.LOC107973052.GGT2-.646279-708040.rev.compl        ttgcagtgtcagaaggtctgagcagcctcgggaggcaagcagtccctggtccctcagtgt   7479
FAM230B-LOC105372935-GGT2.NCBI.GGT2.rev.compl                        ttgtggtgtcggaaggtctgagcagcctcgggaggcaagcagtccctggtccctcagtgt   5454
                                                                     ***  ***** *************************************************

chimp.LOC112206744.LOC107973052.GGT2-.646279-708040.rev.compl        agtcactggaggagacagtcactgagaggcagctggcagggtgaagggaaaggggaggca   7539
FAM230B-LOC105372935-GGT2.NCBI.GGT2.rev.compl                        agtcactggaggagacagtcactgagaggcagctggcagggtgaagggaaaggggaggca   5514
                                                                     ************************************************************

chimp.LOC112206744.LOC107973052.GGT2-.646279-708040.rev.compl        ggccacagagatgacagcctttaagctgtcatactgggaggtcaaggatctgaaagagga   7599
FAM230B-LOC105372935-GGT2.NCBI.GGT2.rev.compl                        ggccacagagatgacagcctttaagctgtcatactgggaggtcaaggatctgaaagagga   5574
                                                                     ************************************************************

chimp.LOC112206744.LOC107973052.GGT2-.646279-708040.rev.compl        aggagaattctttatcattaaggacctgtccttatctcaggcatttcctccagagcatca   7659
FAM230B-LOC105372935-GGT2.NCBI.GGT2.rev.compl                        aggagaattctttatcattaaggacctgtccttatctcaggcatttcctccagagcatca   5634
                                                                     ************************************************************

chimp.LOC112206744.LOC107973052.GGT2-.646279-708040.rev.compl        cctttgtccacccacacaccttgggctaggaggactggagaaagacagtgaggggtctct   7719
FAM230B-LOC105372935-GGT2.NCBI.GGT2.rev.compl                        cctttgtccacccacacaccttgggctaggaggactggagaaagacagtgaggggtctct   5694
                                                                     ************************************************************

chimp.LOC112206744.LOC107973052.GGT2-.646279-708040.rev.compl        tgggtctctggcacagggcgtgatgaagaggtggcagtttttcaggaatctctctctcta   7779
FAM230B-LOC105372935-GGT2.NCBI.GGT2.rev.compl                        tgggtctctggcacagggcgtgatgaagaggtggcagtttttcaggaatctctctctcta   5754
                                                                     ************************************************************

chimp.LOC112206744.LOC107973052.GGT2-.646279-708040.rev.compl        gggaaccaaatacatttcccatctcagctccttcactcagcggggttgaggttctgctcg   7839
FAM230B-LOC105372935-GGT2.NCBI.GGT2.rev.compl                        gggaaccaaatacatttcccatctcaggtccttcactcagcggggttgaggttctgctcg   5814
                                                                     *************************** ********************************

chimp.LOC112206744.LOC107973052.GGT2-.646279-708040.rev.compl        tcacttatcatctctgaatgtcagcaccctcaagtgtaaaatctcagccacagccccttc   7899
FAM230B-LOC105372935-GGT2.NCBI.GGT2.rev.compl                        tcacttatcatctctgaatgtcagcaccctcaagtgtaaaatctcagccacagcccctcc   5874
                                                                     ********************************************************** *

chimp.LOC112206744.LOC107973052.GGT2-.646279-708040.rev.compl        tctgcacccccctgcagggctgatgttctccataaaccataaggcatcatgcccacggaaa   7959
FAM230B-LOC105372935-GGT2.NCBI.GGT2.rev.compl                        tctgcacccccctgcagggctgatgttctccataaaccataaggcgtcatgcccacggaaa   5934
                                                                     ******************************************** ***************

chimp.LOC112206744.LOC107973052.GGT2-.646279-708040.rev.compl        agccgaacaggaaagcatgctccactgccccagagccatccaagttccccctccatattc   8019
FAM230B-LOC105372935-GGT2.NCBI.GGT2.rev.compl                        agccgaacaggaaagcatgctccactgccccggagtcatgcaagttccccctccatattc   5994
                                                                     ******************************* *** *** ********************

chimp.LOC112206744.LOC107973052.GGT2-.646279-708040.rev.compl        cgccactgctaagtgtccagcctattcctcctggcatgtagtaaacacttagagaacatt   8079
FAM230B-LOC105372935-GGT2.NCBI.GGT2.rev.compl                        cgccactgctaagtgtccagcttattcctcctggcatgtagtaaacacttagagaacatt   6054
```

```
                                                                     ********************* **************************************

chimp.LOC112206744.LOC107973052.GGT2-.646279-708040.rev.compl      actgaagtaccagtcctctccaaggttttcctgtatttagtgatttttagccctgtact   8139
FAM230B-LOC105372935-GGT2.NCBI.GGT2.rev.compl                      actgaagtaccagtcctctctaaggttttcctgtatttagtgatttttagccctgcact   6114
                                                                   ******************** *********************************** ***

chimp.LOC112206744.LOC107973052.GGT2-.646279-708040.rev.compl      gtgatactaagaagtagggcctaaatagggcctaaaaagtattgctaaaattacattatg   8199
FAM230B-LOC105372935-GGT2.NCBI.GGT2.rev.compl                      gtgatactaagaagtagggcttaaatagggcctaaaaagtattgctaaaattacattatg   6174
                                                                   ******************** ***************************************

chimp.LOC112206744.LOC107973052.GGT2-.646279-708040.rev.compl      acagtgcagagaactgagggcagagggaggacatgagcttgccaggtccacatggcttag   8259
FAM230B-LOC105372935-GGT2.NCBI.GGT2.rev.compl                      acagtgcagagaactgagggcagagggaggacatgagcttgccaggtccacatggcttag   6234
                                                                   ************************************************************

chimp.LOC112206744.LOC107973052.GGT2-.646279-708040.rev.compl      tggaatttgaatccgggcccccactctgcaccagccctgcactcacagtcatcctgctgt   8319
FAM230B-LOC105372935-GGT2.NCBI.GGT2.rev.compl                      tggaatttgaatccgggcccccactctgcaccagccctgcactcacagtcatccggctgt   6294
                                                                   ****************************************************** *****

chimp.LOC112206744.LOC107973052.GGT2-.646279-708040.rev.compl      gttctcctctccaggaaggcactgcccacgcagtctgtctgatagaggtgttgagtgctc   8379
FAM230B-LOC105372935-GGT2.NCBI.GGT2.rev.compl                      gttctcctctccaggaaggcactgcccacgcagtctgtctgatagaggtgttgagtgctc   6354
                                                                   ************************************************************

chimp.LOC112206744.LOC107973052.GGT2-.646279-708040.rev.compl      actgaactccgtgatcttcctgaaacccaactttgattcagtgggttctgcttggaagcc   8439
FAM230B-LOC105372935-GGT2.NCBI.GGT2.rev.compl                      actgaactccgtgatcttcctgaaacccaactttgattcagtgggctctgcttggaagcc   6414
                                                                   ********************************************* **************

chimp.LOC112206744.LOC107973052.GGT2-.646279-708040.rev.compl      tgtaaagaaaaggatcataagtttaaacttagaacagattatcactattttccctctggt   8499
FAM230B-LOC105372935-GGT2.NCBI.GGT2.rev.compl                      tgtaaagaaaaggatcataagtttaaacttagaacagattatcactattttccctctggt   6474
                                                                   ************************************************************

chimp.LOC112206744.LOC107973052.GGT2-.646279-708040.rev.compl      cttctgtcagcaagatgtcaacagccctatctattgtaaatgcattaaccagcatcttct   8559
FAM230B-LOC105372935-GGT2.NCBI.GGT2.rev.compl                      cttctgtcagcaagatgtcaacagccctatctattgtcaatgcattaaccagcatcttct   6534
                                                                   ************************************* **********************

chimp.LOC112206744.LOC107973052.GGT2-.646279-708040.rev.compl      ctgatagagaatataagaagatatgctgtgcacaccaaccagtgttggagacctcatggc   8619
FAM230B-LOC105372935-GGT2.NCBI.GGT2.rev.compl                      ctgatagagaatacaagaagatatgctgtgcacaccaaccagtgtaggagacctcatggc   6594
                                                                   ************* ******************************* **************

chimp.LOC112206744.LOC107973052.GGT2-.646279-708040.rev.compl      tcccgggtaaagaagaagaggtacccacaagaaggtactgtggaagttcattaattaagt   8679
FAM230B-LOC105372935-GGT2.NCBI.GGT2.rev.compl                      tcccgggtaaagaagaagaggtacccacaagaaggtactgtggaagttcattaattaagt   6654
                                                                   ************************************************************

chimp.LOC112206744.LOC107973052.GGT2-.646279-708040.rev.compl      tgattcaagaattgcaattgcggggagtattcagtgtcccatatgtaagaggaaactatg   8739
FAM230B-LOC105372935-GGT2.NCBI.GGT2.rev.compl                      tgattcaagaattgcagttgcggggagtattcagtgtcccatatgtaagaggaaactatg   6714
                                                                   **************** *******************************************

chimp.LOC112206744.LOC107973052.GGT2-.646279-708040.rev.compl      aagagactaagccatatttttaatgtgtcaggattctaatttgcctggtcagtaaatat   8799
FAM230B-LOC105372935-GGT2.NCBI.GGT2.rev.compl                      aagagactaagccatatttttaatgtgtcaggattctaatttgcctggtcagtaaatat   6774
                                                                   ************************************************************

chimp.LOC112206744.LOC107973052.GGT2-.646279-708040.rev.compl      tgctaccaccacaaaagtaaatatctacttaaaagtcaattttggttcatgttgaatgat   8859
FAM230B-LOC105372935-GGT2.NCBI.GGT2.rev.compl                      tgctaccaccacaaaagtaaatatctacttaaaagtcaattttggttcatgtttaatgat   6834
                                                                   ***************************************************** ******

chimp.LOC112206744.LOC107973052.GGT2-.646279-708040.rev.compl      agacaatgtttcaagctaatgtctagaacttacctggttgttaaacataagcatagatct   8919
FAM230B-LOC105372935-GGT2.NCBI.GGT2.rev.compl                      agacaatgtttcaagctaatgtctagaacttacctggttgttaaacataagcatagatct   6894
                                                                   ************************************************************

chimp.LOC112206744.LOC107973052.GGT2-.646279-708040.rev.compl      ccctgaaagagtggtgctatattattatttttcaattaatatatttctttagagagtttt   8979
FAM230B-LOC105372935-GGT2.NCBI.GGT2.rev.compl                      ccctgaaagagtggtgctatattattatttttcaattaatatatttctttagagagtttt   6954
                                                                   ************************************************************

chimp.LOC112206744.LOC107973052.GGT2-.646279-708040.rev.compl      aaattgacataaaaactgagcatatggccgggcgtggtggctcacacttataatcccagc   9039
FAM230B-LOC105372935-GGT2.NCBI.GGT2.rev.compl                      aaattgacataaaaactgagcatatggccgggcgtggtggctcacacttataatcccagc   7014
                                                                   ************************************************************

chimp.LOC112206744.LOC107973052.GGT2-.646279-708040.rev.compl      actttaggaggccaaggcaggcggatcatctgaggtcaagagttggagaccagcctggcc   9099
FAM230B-LOC105372935-GGT2.NCBI.GGT2.rev.compl                      actttaggaggccaaggcaggcggatcatctgaggtcaggagttggagaccagcctggcc   7074
                                                                   ************************************** *********************

chimp.LOC112206744.LOC107973052.GGT2-.646279-708040.rev.compl      aacatggtgaaaccccatctctactaaaatacaaaaaaattagccgggtgtggtggcag   9159
FAM230B-LOC105372935-GGT2.NCBI.GGT2.rev.compl                      aacatggtgaaaccccatctctactaaaatccaaaaaaattagccgggtgtggtggcag   7134
                                                                   ****************************** *****************************

chimp.LOC112206744.LOC107973052.GGT2-.646279-708040.rev.compl      gtgcctgtaatcccagctactcaggaggctgaggcaggagaatcgcttgaacccaggagg   9219
FAM230B-LOC105372935-GGT2.NCBI.GGT2.rev.compl                      gcgcctgtaatcccagctactcaggaggctgaggcaggagaatcgcttgaacccaggagg   7194
                                                                   * **********************************************************

chimp.LOC112206744.LOC107973052.GGT2-.646279-708040.rev.compl      cagaggttgcagtgagccaagatcatgccattgcactccagcctgggtgacaagagtgaa   9279
FAM230B-LOC105372935-GGT2.NCBI.GGT2.rev.compl                      cagaggttgcagtgagccaagatcatgccattgcactccagcctgggtgacaagagtgaa   7254
                                                                   ************************************************************

chimp.LOC112206744.LOC107973052.GGT2-.646279-708040.rev.compl      actccatctcaaaataaataaataaataaataaataaataaataaaaattgagtatataa   9339
FAM230B-LOC105372935-GGT2.NCBI.GGT2.rev.compl                      actccatctca--------aaataaataaataaataaataaataaaaattgagtatataa   7306
                                                                   ***********        *****************************************

chimp.LOC112206744.LOC107973052.GGT2-.646279-708040.rev.compl      tatacgaagttcccatattattctgtctcctcaccctcacttcctaatttcacctattag   9399
FAM230B-LOC105372935-GGT2.NCBI.GGT2.rev.compl                      tacacgaagttcccatattattctgtctcctcaccctcacttcctaatttcacctattag   7366
                                                                   ** *********************************************************

chimp.LOC112206744.LOC107973052.GGT2-.646279-708040.rev.compl      taacatcttacattactgtggtacatttgctagaataatgagaaaatattgacacattat   9459
FAM230B-LOC105372935-GGT2.NCBI.GGT2.rev.compl                      taacatcttacattactgtggtacatttgctagaataatgagaaaatattgacacattat   7426
                                                                   ************************************************************

chimp.LOC112206744.LOC107973052.GGT2-.646279-708040.rev.compl      tatctgaagtctacatttgcataatgttcattctttctgttatacatatatatgaatttt   9519
FAM230B-LOC105372935-GGT2.NCBI.GGT2.rev.compl                      tatctgaagtctacatttgcataatgttcattctttctgttatacatatatatgaatttt   7486
                                                                   ************************************************************

chimp.LOC112206744.LOC107973052.GGT2-.646279-708040.rev.compl      gaaatatttaaaacattatgttcacccttatggtctcataaagaaaatgttcacttccct   9579
FAM230B-LOC105372935-GGT2.NCBI.GGT2.rev.compl                      gaaatatttaaaacattatgttcacccttatggtctcataaagaaaatgttcacttccct   7546
                                                                   ************************************************************

chimp.LOC112206744.LOC107973052.GGT2-.646279-708040.rev.compl      aaaaatcctctcttctcattaatctctgtcctctttctccagaaaccttggcaactatta   9639
FAM230B-LOC105372935-GGT2.NCBI.GGT2.rev.compl                      aaaaatcctctcttctcattaatctctgtcctctttctccagaaaccttggcaactatta   7606
                                                                   ************************************************************

chimp.LOC112206744.LOC107973052.GGT2-.646279-708040.rev.compl      atattttactatcacttcagctttgccttttccagaatgtcatatagttggaatcatat   9699
FAM230B-LOC105372935-GGT2.NCBI.GGT2.rev.compl                      atattttactatcgcttcagctttgccttttccagaatgtcatatagttggaatcatat   7666
                                                                   ************* **********************************************

chimp.LOC112206744.LOC107973052.GGT2-.646279-708040.rev.compl      attatgtagtttttttcagatgaatttattgcactaaattgatgtacgctttagctgcttt   9759
FAM230B-LOC105372935-GGT2.NCBI.GGT2.rev.compl                      attatgtagtttttttcagatgaatttattgcactaaattgatgtacgctttagctgcttt   7726
                                                                   ************************************************************

chimp.LOC112206744.LOC107973052.GGT2-.646279-708040.rev.compl      catgtctttttttatgccttaatggcaaaaaatggcacattaaatcaccaaataatattgc   9819
FAM230B-LOC105372935-GGT2.NCBI.GGT2.rev.compl                      catgtctttttttatgccttaatggcaaaaaatggcacattaaatcaccaaataatattgc   7786
                                                                   ************************************************************

chimp.LOC112206744.LOC107973052.GGT2-.646279-708040.rev.compl      attaaatgaatttttgtctttttattcacctgttgaagaattcggtagatttcatgagag   9879
FAM230B-LOC105372935-GGT2.NCBI.GGT2.rev.compl                      attcaatgaatttttgtctttttattcacctgttgaagaattcggtagatttcatgagag   7846
                                                                   *** ********************************************************

chimp.LOC112206744.LOC107973052.GGT2-.646279-708040.rev.compl      aaaccatctgggcctggtgctttctttttcagaatgctcttaatgtgaattcaacttatt   9939
FAM230B-LOC105372935-GGT2.NCBI.GGT2.rev.compl                      aaaccatctgggcctggtgctttctttttcggaatgctcttaatgtgaattcaacttatt   7906
                                                                   ****************************** *****************************

chimp.LOC112206744.LOC107973052.GGT2-.646279-708040.rev.compl      taatagacataagtttattcaaattaggatcctagcatgaccttgggaagattgcctttc   9999
FAM230B-LOC105372935-GGT2.NCBI.GGT2.rev.compl                      taatagacataagtttattcaaattaggatcctagcatgaccttgggaagattgcctttc   7966
                                                                   ************************************************************

chimp.LOC112206744.LOC107973052.GGT2-.646279-708040.rev.compl      aaggaattgatacatttcactgaggttatcaaactgcggtcatagaactgttcatgatat   10059
FAM230B-LOC105372935-GGT2.NCBI.GGT2.rev.compl                      aaggaattgatacatttcactgaggttatcaaactgcggtcatagaactgttcatgatat   8026
                                                                   ************************************************************

chimp.LOC112206744.LOC107973052.GGT2-.646279-708040.rev.compl      tccttttaatgcctaacagttcagtagagatggctcctcttttatttctgaaattggtca   10119
FAM230B-LOC105372935-GGT2.NCBI.GGT2.rev.compl                      tccttttaatgcctaacagttcagtagagatggctcctcttttatttctgaaattggtca   8086
                                                                   ************************************************************

chimp.LOC112206744.LOC107973052.GGT2-.646279-708040.rev.compl      tttgtgttatcttctttttcttggttagcctgcatatcaattcattcattgtaatgagca   10179
FAM230B-LOC105372935-GGT2.NCBI.GGT2.rev.compl                      tttgtgttatcttctttttcttggttagcctgcatatcaattcattcattgtaatgagca   8146
                                                                   ************************************************************

chimp.LOC112206744.LOC107973052.GGT2-.646279-708040.rev.compl      tatcaaagaaccagcttttggttttattgatttctgatggtttcagtgttttaattta   10239
FAM230B-LOC105372935-GGT2.NCBI.GGT2.rev.compl                      tatcaaagaaccagcttttggttttattgatttctgatgatttcagtgttttaattta   8206
                                                                   **************************************** *******************
```

```
chimp.LOC112206744.LOC107973052.GGT2-.646279-708040.rev.compl      ttgatttctgtgatgttgtttattacttttacttgctttccattgcattcctctattttc   10299
FAM230B-LOC105372935-GGT2.NCBI.GGT2.rev.compl                      ttgatttctgtgatgttgtttattacttttacttgctttccattgcattcctctattttc   8266
                                                                   ************************************************************

chimp.LOC112206744.LOC107973052.GGT2-.646279-708040.rev.compl      tatagttccctaattgaaacatgatattactgattttaggtcttgtgatttttagtatat   10359
FAM230B-LOC105372935-GGT2.NCBI.GGT2.rev.compl                      tatagttccctaattgaaacatgatattactgattttaggtcttgtgatttttagtatat   8326
                                                                   ************************************************************

chimp.LOC112206744.LOC107973052.GGT2-.646279-708040.rev.compl      tgcatccaatgctatagatttccctctaaggactgcttttgctacatccagaaatcttgc   10419
FAM230B-LOC105372935-GGT2.NCBI.GGT2.rev.compl                      tgcatccaatgctatagatttccctctaaggactgcttttgctacatccagaaatcttgc   8386
                                                                   ************************************************************

chimp.LOC112206744.LOC107973052.GGT2-.646279-708040.rev.compl      caagtcacattttcttttaatgtagttaaaagtatttttaattttctattgagacttctt   10479
FAM230B-LOC105372935-GGT2.NCBI.GGT2.rev.compl                      caagtcacattttcttttaatgtagttaaaagtatttttaattttctattgagacttctt   8446
                                                                   ************************************************************

chimp.LOC112206744.LOC107973052.GGT2-.646279-708040.rev.compl      ctttaacccatgagttatttaaaagtgcattgctaatttgcaaatatttggggattttgt   10539
FAM230B-LOC105372935-GGT2.NCBI.GGT2.rev.compl                      ctttaacccatgagttatttaaaagtgcattgctaatttgcaaatatttggggattttgt   8506
                                                                   ************************************************************

chimp.LOC112206744.LOC107973052.GGT2-.646279-708040.rev.compl      ggctcttttacagttgttgatttttttgttgtcaggtatgtgttgcaaaagcagtcgtcta   10599
FAM230B-LOC105372935-GGT2.NCBI.GGT2.rev.compl                      ggctcttttacagttgttgaatttttgttgtcaggtatgtgttgcaaaagcagtcgtcta   8566
                                                                   ******************** ***************************************

chimp.LOC112206744.LOC107973052.GGT2-.646279-708040.rev.compl      cctcatcttgccaccagccaagatggcccaggatgtgggctctccctgagtgaatctttg   10659
FAM230B-LOC105372935-GGT2.NCBI.GGT2.rev.compl                      cctcatcttgccaccacccaagatggcccaggatgtgggctctccctgagtgaatctttg   8626
                                                                   **************** *******************************************

chimp.LOC112206744.LOC107973052.GGT2-.646279-708040.rev.compl      gcaatctgccaacctgatgtgttcggcctccttctttagtctgagtttcccttctgctta   10719
FAM230B-LOC105372935-GGT2.NCBI.GGT2.rev.compl                      gcaatctgccaacctgatgtgttcggcctccttctttagtctgagcttgccttctgctta   8686
                                                                   ********************************************* ** ***********

chimp.LOC112206744.LOC107973052.GGT2-.646279-708040.rev.compl      gaaagggccattctcagttctggcagggagttttcccaacattgagaaggtggcattctt   10779
FAM230B-LOC105372935-GGT2.NCBI.GGT2.rev.compl                      gaaagggccattctcagttctggcagggagttttcccaacattgagaaggtggcattctt   8746
                                                                   ************************************************************

chimp.LOC112206744.LOC107973052.GGT2-.646279-708040.rev.compl      actccccactgcagcctgcacctctgaccggtggtcagcagacaggacagaggtcctcag   10839
FAM230B-LOC105372935-GGT2.NCBI.GGT2.rev.compl                      actccccactgcagcctgcacctctgaccggtggtcagcagacaggacagaggtcctcat   8806
                                                                   ***********************************************************

chimp.LOC112206744.LOC107973052.GGT2-.646279-708040.rev.compl      tagacagagttcagcggggtctctgaccaaagtgcatcttcagagtctgcacctacccac   10899
FAM230B-LOC105372935-GGT2.NCBI.GGT2.rev.compl                      cagacagagttcagcagggtctctgaccaaagtgcatcttcagagtctgcacctacccac   8866
                                                                    ************** ********************************************

chimp.LOC112206744.LOC107973052.GGT2-.646279-708040.rev.compl      tgtgaccacgggcaggctctgagtcctaaagcaggaggaactgtgcgaccatcctgattg   10959
FAM230B-LOC105372935-GGT2.NCBI.GGT2.rev.compl                      tgtgaccacgggcaggctctgagtcctaaagcaggaggaactgtgcgaccatcctgattg   8926
                                                                   ************************************************************

chimp.LOC112206744.LOC107973052.GGT2-.646279-708040.rev.compl      gaaatttgtgaggatcaccgtgttactcaagtaaggtctttggaaagtgtcgtattacta   11019
FAM230B-LOC105372935-GGT2.NCBI.GGT2.rev.compl                      gaaatttgtgaggatcaccgtgttactcaagtaaggtctttggaaagtgtcgtattacta   8986
                                                                   ************************************************************

chimp.LOC112206744.LOC107973052.GGT2-.646279-708040.rev.compl      ctgtttgtgaactgcttgttggtggcctggctgagccacacactttatgaaaaccaggac   11079
FAM230B-LOC105372935-GGT2.NCBI.GGT2.rev.compl                      ctgtttgtgaactgcttgttggtggcctggctgagccacacactttatgaaaaccaggac   9046
                                                                   ************************************************************

chimp.LOC112206744.LOC107973052.GGT2-.646279-708040.rev.compl      ccctcagctggtgtgggtgtctatgcagactgagaccctcatgtgaacagccttgtggca   11139
FAM230B-LOC105372935-GGT2.NCBI.GGT2.rev.compl                      ccctcagctggtgtgggtgtctatgcagcctgagaccctcatgtgaacagcctcgtggca   9106
                                                                   **************************** ************************* ******

chimp.LOC112206744.LOC107973052.GGT2-.646279-708040.rev.compl      gctgtctttgccccttgccaccatcagtgcctccttgttcctgggcactgctttctctga   11199
FAM230B-LOC105372935-GGT2.NCBI.GGT2.rev.compl                      gctgtctttgccccttgccaccatcagtgcctccttgttcctgggcactgctttctctga   9166
                                                                   ************************************************************

chimp.LOC112206744.LOC107973052.GGT2-.646279-708040.rev.compl      tggtgctccattgttttcctgcacctcagtgtctacagctggatgtctcttctgcaatct   11259
FAM230B-LOC105372935-GGT2.NCBI.GGT2.rev.compl                      tggtgctccattgttttcctgcacctcagtgtctacagctggatgtctcttccgcaatct   9226
                                                                   **************************************************** *******

chimp.LOC112206744.LOC107973052.GGT2-.646279-708040.rev.compl      aggcgaggggcatcaatggcagttctgctgtggcactgccctccttcttagcttgtctt   11319
FAM230B-LOC105372935-GGT2.NCBI.GGT2.rev.compl                      aggcgaggggcatcaacggcagttctgctgtggcactgccctccttcttagcttgtctt   9286
                                                                   **************** *******************************************

chimp.LOC112206744.LOC107973052.GGT2-.646279-708040.rev.compl      gctctgtcttaggctccctcaaagatcccacccttcaggttcttccacaagtttcttact   11379
FAM230B-LOC105372935-GGT2.NCBI.GGT2.rev.compl                      gctctgtcttaggctccctcaaagatcccacccttcaggttcttccacaagtttcttatt   9346
                                                                   ********************************************************** *

chimp.LOC112206744.LOC107973052.GGT2-.646279-708040.rev.compl      gaaatccgagcagaaaactatgaccaatatgaccaatcctatacccactgaagacatgaa   11439
FAM230B-LOC105372935-GGT2.NCBI.GGT2.rev.compl                      gaaatccgagcagaaaactatgaccaatatgaccaatcctatacccactgaagacatgaa   9406
                                                                   ************************************************************

chimp.LOC112206744.LOC107973052.GGT2-.646279-708040.rev.compl      tgaagaattaaaacaattctccatggactctaccatatagatccctagaagtaatttcta   11499
FAM230B-LOC105372935-GGT2.NCBI.GGT2.rev.compl                      tgaagaattaaaacaattctccatggactctaccatatagatccctagaagtaatttcta   9466
                                                                   ************************************************************

chimp.LOC112206744.LOC107973052.GGT2-.646279-708040.rev.compl      aaaaaaaaaaaaaaa----caaggaagatgtaatagttttccataaattagaataccctа   11555
FAM230B-LOC105372935-GGT2.NCBI.GGT2.rev.compl                      aaaaaaaaaaaaaaaaaaatcaaggaagatgtaatagttttccataaattagaataccctа   9526
                                                                   ***************    *****************************************

chimp.LOC112206744.LOC107973052.GGT2-.646279-708040.rev.compl      catgtacaattaaatgaaatggctagtatagtcttgaaaccaaaaccagataaggtaaat   11615
FAM230B-LOC105372935-GGT2.NCBI.GGT2.rev.compl                      catgtacaattaaatgaaatggctagtatagtcttgaaaccaaaaccagataaggtaaat   9586
                                                                   ************************************************************

chimp.LOC112206744.LOC107973052.GGT2-.646279-708040.rev.compl      taaattctgtgatattttaaaatactgtaaattctgactaatgtgagttaatctcaatat   11675
FAM230B-LOC105372935-GGT2.NCBI.GGT2.rev.compl                      taaattctgtgatattttaaaatactgtaaattctgactaatgtgagttaatctcaatat   9646
                                                                   ************************************************************

chimp.LOC112206744.LOC107973052.GGT2-.646279-708040.rev.compl      atgaaatagtagattaacattgaaaatgcaataaataaaattagctacctcaagagttta   11735
FAM230B-LOC105372935-GGT2.NCBI.GGT2.rev.compl                      atgaaatagtagattaacattgaaaatgcaataaataaaattagctacctcaagagttta   9706
                                                                   ************************************************************

chimp.LOC112206744.LOC107973052.GGT2-.646279-708040.rev.compl      atggaaaaaaatgtgattattgcaatagattcaggaaattcatgaataacattcaccctа   11795
FAM230B-LOC105372935-GGT2.NCBI.GGT2.rev.compl                      atggaaaaaaatgtgattattgcaatagattcaggaaattcatgaataacattcaccctа   9766
                                                                   ************************************************************

chimp.LOC112206744.LOC107973052.GGT2-.646279-708040.rev.compl      tatttgtaggacaactatctgattaactttaagtgacctgtgacaaccatttgaattaat   11855
FAM230B-LOC105372935-GGT2.NCBI.GGT2.rev.compl                      tatttgtaggacagctatc----taactttaagtgacctgtgacaaccatttgaattaat   9822
                                                                   ************* *****    *************************************

chimp.LOC112206744.LOC107973052.GGT2-.646279-708040.rev.compl      gctgctttcacagcatatctcttggcttgttaaaaacccgacaagaatttccgtaacatt   11915
FAM230B-LOC105372935-GGT2.NCBI.GGT2.rev.compl                      gctgctttcacagcatatctcttggcttgttaaaaacccgacaagaatttccgtaacatt   9882
                                                                   ************************************************************

chimp.LOC112206744.LOC107973052.GGT2-.646279-708040.rev.compl      aatttatttttaacacctatattgggtgtgaacccaccataaagtttgcccactgaaaag   11975
FAM230B-LOC105372935-GGT2.NCBI.GGT2.rev.compl                      aatttatttttaacacctatattgggtgtgaacccaccataaagtttgcccactgaaaag   9942
                                                                   ************************************************************

chimp.LOC112206744.LOC107973052.GGT2-.646279-708040.rev.compl      gtctacaatttgatgctttattaaattgatactgtgtgcacccatcaccaccatctaatt   12035
FAM230B-LOC105372935-GGT2.NCBI.GGT2.rev.compl                      gtctacaatttgatgctttattaaattgatactgtgtgcacccatcaccacgatctaatt   10002
                                                                   *************************************************** ********

chimp.LOC112206744.LOC107973052.GGT2-.646279-708040.rev.compl      taaatatgtttccctcacccaaattctctcttgcgcactggcagttaatccccaccccca   12095
FAM230B-LOC105372935-GGT2.NCBI.GGT2.rev.compl                      taaatatgtttccctcacccaaattctctcttgcgcactggcagttaatccccactccca   10062
                                                                   ******************************************************* ****

chimp.LOC112206744.LOC107973052.GGT2-.646279-708040.rev.compl      tctccagccctaagcaatactgctgtgacattccatctccataaatttcccacttgctta   12155
FAM230B-LOC105372935-GGT2.NCBI.GGT2.rev.compl                      tctccagccctaagcaatactgctgtgacattccatctccataaatttcccatttgctta   10122
                                                                   **************************************************** *******

chimp.LOC112206744.LOC107973052.GGT2-.646279-708040.rev.compl      atagaaatggacatatatatatatttggaatctgacttccttcatttagcatactatgtt   12215
FAM230B-LOC105372935-GGT2.NCBI.GGT2.rev.compl                      atagaaatggacatatatatatatttggaatctgacttccttcatttagcatactatgtt   10182
                                                                   ************************************************************

chimp.LOC112206744.LOC107973052.GGT2-.646279-708040.rev.compl      tgaagttaattgacgtgttagcacgtgctggtcatgtgttttccttcatagtctgctgtg   12275
FAM230B-LOC105372935-GGT2.NCBI.GGT2.rev.compl                      tgaagttaattgacgtgttagcacgtgctggtcatgtgttttccttcatagtctgctgtg   10242
                                                                   ************************************************************

chimp.LOC112206744.LOC107973052.GGT2-.646279-708040.rev.compl      tttactcatacagatagtgtttattcgtttatcagttaatggacatttaattgttttgtt   12335
FAM230B-LOC105372935-GGT2.NCBI.GGT2.rev.compl                      tttactcatacagatagtgtttattcatttatcagttaatggacatttaattgttttgtt   10302
                                                                   ************************** *********************************

chimp.LOC112206744.LOC107973052.GGT2-.646279-708040.rev.compl      attttctttgatgagtaacgtagcttcgagcattcatatacagtcaggtaatgcataatg   12395
FAM230B-LOC105372935-GGT2.NCBI.GGT2.rev.compl                      attttctttgatgagtaatgtagctttgagcattcatatacagtcatgtaatgcataatg   10362
                                                                   ****************** ******* ******************** ************
```

```
chimp.LOC112206744.LOC107973052.GGT2-.646279-708040.rev.compl      acattttggtcaaaaaaaattttttttttctgagacccaggctggagtgcagtggcacaa   12455
FAM230B-LOC105372935-GGT2.NCBI.GGT2.rev.compl                      acattttggtcaaaaaaattttttttttttctgagacccaggctggagtgcagtggcacaa   10422
                                                                   ****************** *****************************************

chimp.LOC112206744.LOC107973052.GGT2-.646279-708040.rev.compl      tctcggctcactggaacctccacctcccaggtttaagcaattcttgtgcctcaacctccc   12515
FAM230B-LOC105372935-GGT2.NCBI.GGT2.rev.compl                      tctcggctcactggaacctccacctcccaggtttaagcaattctcatgcctcaacctccc   10482
                                                                   ********************************************  **************

chimp.LOC112206744.LOC107973052.GGT2-.646279-708040.rev.compl      gagtagctgggacaactggcacacgccaccatgcctggataattttgtattttcagtag   12575
FAM230B-LOC105372935-GGT2.NCBI.GGT2.rev.compl                      gagtagctgggacaactggcacacgccaccatgcctggataattttgtattttcagtag   10542
                                                                   ************************************************************

chimp.LOC112206744.LOC107973052.GGT2-.646279-708040.rev.compl      agacaggattttgctgtgttggtcaggctagtctcaaactcctagcctcaggtgatccac   12635
FAM230B-LOC105372935-GGT2.NCBI.GGT2.rev.compl                      agacaggattttgctgtgttggtcaggctagtctcaaactcccagcctcaggtgatccac   10602
                                                                   ****************************************** *****************

chimp.LOC112206744.LOC107973052.GGT2-.646279-708040.rev.compl      ccatctctgcctcccaaagtgctgggattataggcatgagccaccacacccagcctaatt   12695
FAM230B-LOC105372935-GGT2.NCBI.GGT2.rev.compl                      ctatctctgcctcccaaagtgctgggattataggcatgagccaccacacccagcctaatt   10662
                                                                   * **********************************************************

chimp.LOC112206744.LOC107973052.GGT2-.646279-708040.rev.compl      tttttaagaaacaagggaactattttctaaattacttttgccaatttatatttctaccat   12755
FAM230B-LOC105372935-GGT2.NCBI.GGT2.rev.compl                      tttttaagaaacaagggaactattttctaaattacttttgccaatttatatttctaccat   10722
                                                                   ************************************************************

chimp.LOC112206744.LOC107973052.GGT2-.646279-708040.rev.compl      gatgcatagcactaatttcaccgtacaatgtatggtaggccccaatatgtaagaaatgat   12815
FAM230B-LOC105372935-GGT2.NCBI.GGT2.rev.compl                      gatgcatagcactaatttcaccgtacaatgtatggtaggccccaatatgtaagaaatgat   10782
                                                                   ************************************************************

chimp.LOC112206744.LOC107973052.GGT2-.646279-708040.rev.compl      gaaagtaacacataaagattagtataaaacaaataaaattatcattgttgctatcatctt   12875
FAM230B-LOC105372935-GGT2.NCBI.GGT2.rev.compl                      gaaaataacacataaagattggtataaaacaaataagattatcattgttgctatcatctt   10842
                                                                   **** *************** *************** ***********************

chimp.LOC112206744.LOC107973052.GGT2-.646279-708040.rev.compl      tgtcaaattctgaaaacaatctgagtatatttttatataaatatgcttggcaacatagct   12935
FAM230B-LOC105372935-GGT2.NCBI.GGT2.rev.compl                      tgtcaaattctgaaaacaatctgagtatatttttatataaatatgcttggcaacatagct   10902
                                                                   ************************************************************

chimp.LOC112206744.LOC107973052.GGT2-.646279-708040.rev.compl      gaaaaacgcattatcagttacatttatcagtaacaaagacataaatttgaaggggaaaa   12995
FAM230B-LOC105372935-GGT2.NCBI.GGT2.rev.compl                      gaaaaacgcattatcagttacatttatcagtaacaaagacataaatttgaaggggaaca   10962
                                                                   ********************************************************** *

chimp.LOC112206744.LOC107973052.GGT2-.646279-708040.rev.compl      acacttgtactaacaacgcaatgtcagaattcacataaaaattctgctggtcactttgga   13055
FAM230B-LOC105372935-GGT2.NCBI.GGT2.rev.compl                      acacttgtactaacaacgcaatgtcagaattaacataaaaattctgctggtcactttgga   11022
                                                                   ******************************* ****************************

chimp.LOC112206744.LOC107973052.GGT2-.646279-708040.rev.compl      atatttaattgcctggggcagtgtttagtagacaaatgagcatctatggagcacccaaag   13115
FAM230B-LOC105372935-GGT2.NCBI.GGT2.rev.compl                      atatttaattgcctggggcagtgtttagtagacaaatgagcatctatggagcactcaaag   11082
                                                                   ****************************************************** *****

chimp.LOC112206744.LOC107973052.GGT2-.646279-708040.rev.compl      taggggaatcaacagaacttcggtttgaaaagttatctgggtttagagcgtgaaactttg   13175
FAM230B-LOC105372935-GGT2.NCBI.GGT2.rev.compl                      taggggaatcaacagaacttgggtttgaaaagttatctgggtttagagcgtgaaactttg   11142
                                                                   ******************** ***************************************

chimp.LOC112206744.LOC107973052.GGT2-.646279-708040.rev.compl      ttagaggacacacgccttgcatgagcgaggtgccttggtgtgtgtggacgtaccattatg   13235
FAM230B-LOC105372935-GGT2.NCBI.GGT2.rev.compl                      ttagaggacacgcaccttgcatgagcgaggtgccctggtgtgtgtggacgtaccattatg   11202
                                                                   *********** * ******************** *************************

chimp.LOC112206744.LOC107973052.GGT2-.646279-708040.rev.compl      cttggaggtacagcataatggtggcttcctccagaaagggacatttggggtggattcatt   13295
FAM230B-LOC105372935-GGT2.NCBI.GGT2.rev.compl                      cttggaggtacagcataatggtggcttcctccagaaagggacatttggggtggattcatt   11262
                                                                   ************************************************************

chimp.LOC112206744.LOC107973052.GGT2-.646279-708040.rev.compl      ccatctagacaacacagcctgatgtggcatggacatgaatggaggtgaaatggtcagtag   13355
FAM230B-LOC105372935-GGT2.NCBI.GGT2.rev.compl                      ccatctagacaacacagcctgatgtggcatggacatgaatggaggtgaaatggtcagtag   11322
                                                                   ************************************************************

chimp.LOC112206744.LOC107973052.GGT2-.646279-708040.rev.compl      ttgagaggatcagtcctgacaagggccaaggtgaaaaacctgggaaccccttcaggtaca   13415
FAM230B-LOC105372935-GGT2.NCBI.GGT2.rev.compl                      ttgagaggatcagtcctgacaagggccgaggtgaaaaacctgggaaccccttcaggtaca   11382
                                                                   *************************** ********************************

chimp.LOC112206744.LOC107973052.GGT2-.646279-708040.rev.compl      aagtcttcagttgaaaaaggaggtggtcacaggaaatactgagacaggacagcaatgcat   13475
FAM230B-LOC105372935-GGT2.NCBI.GGT2.rev.compl                      aagtcttcagttgaaaaaggaggtggtcacaggaaatactgagacaggacagcaaagcat   11442
                                                                   ******************************************************* ****

chimp.LOC112206744.LOC107973052.GGT2-.646279-708040.rev.compl      gggagacagagttcttggccctgcagggtgagtagtgtggattctcaagttttctcctct   13535
FAM230B-LOC105372935-GGT2.NCBI.GGT2.rev.compl                      gggagacagagttcttggccctgcagggtgagtagtgtggattctcaagttttctcctct   11502
                                                                   ************************************************************

chimp.LOC112206744.LOC107973052.GGT2-.646279-708040.rev.compl      ctccattaatttctttcccaatgcagatgacttccatcatacagtcttcagcaaccttga   13595
FAM230B-LOC105372935-GGT2.NCBI.GGT2.rev.compl                      ctccattaatttctttcccaatgcagatgacttccatcatacagtcttcagcaaccttga   11562
                                                                   ************************************************************

chimp.LOC112206744.LOC107973052.GGT2-.646279-708040.rev.compl      aagattggacaagcttcagcccactcttgaaggtaaaggaaggcagctaacaagactggc   13655
FAM230B-LOC105372935-GGT2.NCBI.GGT2.rev.compl                      aagattggacaagcttcagcccactcttgaaggtaaaggaaggcagctaacaagactggc   11622
                                                                   ************************************************************

chimp.LOC112206744.LOC107973052.GGT2-.646279-708040.rev.compl      atctgggcttggctgtgcgtgtttctattgtggggaaatatatataacacaatatttat   13715
FAM230B-LOC105372935-GGT2.NCBI.GGT2.rev.compl                      atctgggcttggctgtgcgtgtttctatcgtggggaaatatatataacacaatatttat   11682
                                                                   **************************** *******************************

chimp.LOC112206744.LOC107973052.GGT2-.646279-708040.rev.compl      catttgaaccttttaaccaaagtgtgcactccatggcattcaatatattcacagggttgc   13775
FAM230B-LOC105372935-GGT2.NCBI.GGT2.rev.compl                      catttgaaccttttaaccaaagtgtgcactccatggcattcaatatattcacagggttgc   11742
                                                                   ************************************************************

chimp.LOC112206744.LOC107973052.GGT2-.646279-708040.rev.compl      ataaccaacaccactatctacacccacaattttgatgatttcttacaaaaccttgtccac   13835
FAM230B-LOC105372935-GGT2.NCBI.GGT2.rev.compl                      ataaccaacaccactatctacacccacaattttgatgatttcttacgaaaccttgtccac   11802
                                                                   ********************************************** *************

chimp.LOC112206744.LOC107973052.GGT2-.646279-708040.rev.compl      tataagcaatatagcaccttcccccctatttccagctcatggtgattcctatcccactttc   13895
FAM230B-LOC105372935-GGT2.NCBI.GGT2.rev.compl                      aataagcaatatagcaccttcccccctatttccagcccatggtgattcctatcccactttc   11862
                                                                    ********************************* *************************

chimp.LOC112206744.LOC107973052.GGT2-.646279-708040.rev.compl      tcttgtatgaatttgactattctaggcacttcatgtaattacaattatacaatatattcc   13955
FAM230B-LOC105372935-GGT2.NCBI.GGT2.rev.compl                      tcttgtatgaatttgactattctaggcacttcatgtaattacaattatacaatatattcc   11922
                                                                   ************************************************************

chimp.LOC112206744.LOC107973052.GGT2-.646279-708040.rev.compl      ttttgtgtctggcttatttcactaagcataatgttctcaatgtccacccatgttgtatca   14015
FAM230B-LOC105372935-GGT2.NCBI.GGT2.rev.compl                      ttttgtgtctggcttatttcactaagcataatgttctcaatgtccacccatgttgtatca   11982
                                                                   ************************************************************

chimp.LOC112206744.LOC107973052.GGT2-.646279-708040.rev.compl      tctatcaaaattatgttcgtttttacagatggatgatgtagcattgcatgcagaccacc   14075
FAM230B-LOC105372935-GGT2.NCBI.GGT2.rev.compl                      tctttcaaaatgatgttcgtttttacagatggatgatgtagcattgcatgcagaccacc   12042
                                                                   *** ******* ************************************************

chimp.LOC112206744.LOC107973052.GGT2-.646279-708040.rev.compl      ttgcttttattacattcatttgttcactgatggttggattatttccaccttttggctcct   14135
FAM230B-LOC105372935-GGT2.NCBI.GGT2.rev.compl                      ttgcttttattacattcatttgttcactgatggttggattatttccaccttttggctcct   12102
                                                                   ************************************************************

chimp.LOC112206744.LOC107973052.GGT2-.646279-708040.rev.compl      gtgaaaagtgatgctacaaacattagtatacaaacatctgtttgatttctgttctctatt   14195
FAM230B-LOC105372935-GGT2.NCBI.GGT2.rev.compl                      gtgaaaagtgatgctacaaacattagtatacaaacatctgtttgatttccgttctctatt   12162
                                                                   ************************************************* **********

chimp.LOC112206744.LOC107973052.GGT2-.646279-708040.rev.compl      ctttggggtgcctaagagtagagttcctgggtccaacaggggttctatatttaaacttct   14255
FAM230B-LOC105372935-GGT2.NCBI.GGT2.rev.compl                      ctttggggtgcctaagagtagagttcctgggtccaacaggggttctatatttaaccttct   12222
                                                                   ****************************************************** *****

chimp.LOC112206744.LOC107973052.GGT2-.646279-708040.rev.compl      gagccactgcagactgtttttcagagtggctgcaactttatccatttctaccatcaatgt   14315
FAM230B-LOC105372935-GGT2.NCBI.GGT2.rev.compl                      gagccactgcagactgtttttcacagtggctgcaactttatccatttctaccatcaatgt   12282
                                                                   *********************** ************************************

chimp.LOC112206744.LOC107973052.GGT2-.646279-708040.rev.compl      atcagggttacaatttctttacgcccttcttcacacttattttcctttaaatcatcctag   14375
FAM230B-LOC105372935-GGT2.NCBI.GGT2.rev.compl                      atcagggttacaatttctttacgtccttcttcacacttattttcctttaaatcatcctag   12342
                                                                   *********************** ************************************

chimp.LOC112206744.LOC107973052.GGT2-.646279-708040.rev.compl      taggtgtatattggtggctgcttgtgtttctcatttgcatttccctaatgactaatgatc   14435
FAM230B-LOC105372935-GGT2.NCBI.GGT2.rev.compl                      taggtgtatattggtggctgcttgtgttttcatttgcatttccctaatgactaatgatc   12402
                                                                   ***************************** ******************************

chimp.LOC112206744.LOC107973052.GGT2-.646279-708040.rev.compl      ctgagcagcttttcctgtgctactatctgtggctgtatcttctttagggaaatatatgtt   14495
FAM230B-LOC105372935-GGT2.NCBI.GGT2.rev.compl                      ctgagcagcttttcctgtgctactatctgtggctgtatcttctttagggaaatatatgtt   12462
                                                                   ************************************************************

chimp.LOC112206744.LOC107973052.GGT2-.646279-708040.rev.compl      gaagtcttttgcccatttttaaagagttgtctgattttatttagttagtttgttgttgt   14555
FAM230B-LOC105372935-GGT2.NCBI.GGT2.rev.compl                      gaagtcttttgcccatttttaaagagttgtctgattttatttagttagtttgttgttgt   12522
                                                                   ************************************************************

chimp.LOC112206744.LOC107973052.GGT2-.646279-708040.rev.compl      agatttttgaatatatcttaaatatatttaaaatagattctaaattttagtctcttacaa   14615
```

```
FAM230B-LOC105372935-GGT2.NCBI.GGT2.rev.compl                       agattttgaatatatcttaaatatatttaaaatatattctaaattttagtctcttacaa   12582
                                                                    *********************************** ************************

chimp.LOC112206744.LOC107973052.GGT2-.646279-708040.rev.compl       gataaatgatttgcaaatatttccacctttgtgtagaactttagattcacaaacttcatt   14675
FAM230B-LOC105372935-GGT2.NCBI.GGT2.rev.compl                       gagaaatgatttgcaaatatttccacctttgtgtagaactttagattcacaaacttcgtt   12642
                                                                    ** ****************************************************** **

chimp.LOC112206744.LOC107973052.GGT2-.646279-708040.rev.compl       aatttgtgtgaaatcctcagcagttgaccccaaacagataagactgaagcagtattttag   14735
FAM230B-LOC105372935-GGT2.NCBI.GGT2.rev.compl                       aatttgtatgaaatcctcagcagttgaccccaaacagataagactgaagcagtattttag   12702
                                                                    ******* ****************************************************

chimp.LOC112206744.LOC107973052.GGT2-.646279-708040.rev.compl       gaatagttgaaagtatgatcaccacaaaacataagcgtaatcaaatcctgcaagctacat   14795
FAM230B-LOC105372935-GGT2.NCBI.GGT2.rev.compl                       gaatagttgaaagtatgatcaccacaaaacataagcgtaatcaaatcctgcaagctacat   12762
                                                                    ************************************************************

chimp.LOC112206744.LOC107973052.GGT2-.646279-708040.rev.compl       gtaaagcacaatgacaaataaggcagcaaagggccatctggtgattagttcaccacactt   14855
FAM230B-LOC105372935-GGT2.NCBI.GGT2.rev.compl                       gtaaggcacaatgacaaataaggcagcaaagggccatctggtgattagttcaccacactt   12822
                                                                    **** *******************************************************

chimp.LOC112206744.LOC107973052.GGT2-.646279-708040.rev.compl       gttgcaactgtttgcgctgcagagttaaaacacaccagcattcaacccatgtctcctctc   14915
FAM230B-LOC105372935-GGT2.NCBI.GGT2.rev.compl                       gttgcaactgtttgcgctgcagagttaaaacacaccagcattcaacccgtgtctcctctc   12882
                                                                    ************************************************ ***********

chimp.LOC112206744.LOC107973052.GGT2-.646279-708040.rev.compl       ttgaggtaaactgtcgtatgttggctggcctgaacaagcgtagatattctccatcctcaa   14975
FAM230B-LOC105372935-GGT2.NCBI.GGT2.rev.compl                       ttgaagtaaactgtcgtatgttggctggcctgaacaagcgtagatattctccatcctcaa   12942
                                                                    **** *******************************************************

chimp.LOC112206744.LOC107973052.GGT2-.646279-708040.rev.compl       ttaatatgcatgcatgacaaagaaaaggaggcctggatgaaaaaatattgtgtgattaat   15035
FAM230B-LOC105372935-GGT2.NCBI.GGT2.rev.compl                       ttaatatgcatgcatgacaaagaaaaggaggcctggatgaaaaaatattgtgtgattaat   13002
                                                                    ************************************************************

chimp.LOC112206744.LOC107973052.GGT2-.646279-708040.rev.compl       aattatgctttaattaattttaaaggatataatttcagtacttctaattctcccatcagc   15095
FAM230B-LOC105372935-GGT2.NCBI.GGT2.rev.compl                       aattatgctttaattaattttaaaggatataatttcagtacttctaattctcccatcagc   13062
                                                                    ************************************************************

chimp.LOC112206744.LOC107973052.GGT2-.646279-708040.rev.compl       agttataacaaaggattagtgaataaataccatagactgttttgcctggaattgaatcca   15155
FAM230B-LOC105372935-GGT2.NCBI.GGT2.rev.compl                       agttataacaaaggattagtgaataaataccatagactgttttgcctagaattgaatcca   13122
                                                                    *********************************************** ************

chimp.LOC112206744.LOC107973052.GGT2-.646279-708040.rev.compl       atctatctattaaactttgcttttattcaagtgcaaaatgctaaaacacataactgcagt   15215
FAM230B-LOC105372935-GGT2.NCBI.GGT2.rev.compl                       atctatctattaaactttgcttgtattcaagtgcaaaatgctaaaacacataactgcagt   13182
                                                                    ********************** *************************************

chimp.LOC112206744.LOC107973052.GGT2-.646279-708040.rev.compl       gacagccactgtggatcctcagaggtaaaagtagtcttgggacataaatcctgcaagcta   15275
FAM230B-LOC105372935-GGT2.NCBI.GGT2.rev.compl                       gacagccactgtggatcctcagaggtaaaagtagtcttgggacataaatcctgcaagcta   13242
                                                                    ************************************************************

chimp.LOC112206744.LOC107973052.GGT2-.646279-708040.rev.compl       atattgtttttacaggtttagaaaaccatttagctgggtttcaaacctcacagtgtgagc   15335
FAM230B-LOC105372935-GGT2.NCBI.GGT2.rev.compl                       atattgtttttacaggtttagaaaaccatttagctgggtttcaaacctcacagtgtgagc   13302
                                                                    ************************************************************

chimp.LOC112206744.LOC107973052.GGT2-.646279-708040.rev.compl       agtgggactctcatcaaactatagcatgtgcttcagtaccatttgtagactgattcattc   15395
FAM230B-LOC105372935-GGT2.NCBI.GGT2.rev.compl                       agtgggactctcatcaaactatagcatgtgcttcagtaccatttgtagactgactcattc   13362
                                                                    ***************************************************** ******

chimp.LOC112206744.LOC107973052.GGT2-.646279-708040.rev.compl       ccatttcttaagttgccatcagcaaaatgccagggactctatttcttgctccttagctc   15455
FAM230B-LOC105372935-GGT2.NCBI.GGT2.rev.compl                       ccatttcttcagttgccatcagcaaaatgccagggactctatttcttgctccttagctc   13422
                                                                    ********* **************************************************

chimp.LOC112206744.LOC107973052.GGT2-.646279-708040.rev.compl       ctcgttcttgcctgtctttccacgagggaagattttctagcaggagctcaagctgtgctt   15515
FAM230B-LOC105372935-GGT2.NCBI.GGT2.rev.compl                       ctcgttcttgcctgtctttccacgagggaagattttctagcaggagctcaagctgtgctt   13482
                                                                    ************************************************************

chimp.LOC112206744.LOC107973052.GGT2-.646279-708040.rev.compl       ttaatgaaacacatccacacacactgtcctgttgtccacattaagcagagctccctgaat   15575
FAM230B-LOC105372935-GGT2.NCBI.GGT2.rev.compl                       ttaatgaaacacatccacacacactgtcctgttgtccacattaagcagagctccctgaat   13542
                                                                    ************************************************************

chimp.LOC112206744.LOC107973052.GGT2-.646279-708040.rev.compl       aactcatgaacaaaagcatctatgactaactgttgctctgtgtcctcctagcctctgagg   15635
FAM230B-LOC105372935-GGT2.NCBI.GGT2.rev.compl                       aactcatgaacaaaagcatctatgactaactgttgctctgtgtcctcctagcctctgagg   13602
                                                                    ************************************************************

chimp.LOC112206744.LOC107973052.GGT2-.646279-708040.rev.compl       agtctctagttcacaaggacagaggagatggagagaggccagtcaacgcgagggtaaggt   15695
FAM230B-LOC105372935-GGT2.NCBI.GGT2.rev.compl                       agtctctagttcacaaggacagaggagatggagagaggccagtcaacgcgagggtaaggt   13662
                                                                    ************************************************************

chimp.LOC112206744.LOC107973052.GGT2-.646279-708040.rev.compl       tgccttgctttctctgaaatagaaatgttcctttcttggtgtctttctttttcaactgac   15755
FAM230B-LOC105372935-GGT2.NCBI.GGT2.rev.compl                       tgccttgctttctctgaaatagaaatgttcctttcttggtgtctttctttttcaactgac   13722
                                                                    ************************************************************

chimp.LOC112206744.LOC107973052.GGT2-.646279-708040.rev.compl       tttacatgtgaaaaaatgacaatgtccatgacaggtattaaatgcagttttctgaggggg   15815
FAM230B-LOC105372935-GGT2.NCBI.GGT2.rev.compl                       tttacatgtgaaaaaatgacaatgtccatgacaggtattaaatgcagttttctgaggggg   13782
                                                                    ************************************************************

chimp.LOC112206744.LOC107973052.GGT2-.646279-708040.rev.compl       aggaagaagtgactcttagcaactgatatgtaatccaaaatgacatttagctatgacggc   15875
FAM230B-LOC105372935-GGT2.NCBI.GGT2.rev.compl                       aggaagaagtgactcttagcaactgatatgtaatccaaaatggcatttagctatgacggc   13842
                                                                    ****************************************** *****************

chimp.LOC112206744.LOC107973052.GGT2-.646279-708040.rev.compl       ttcaggttgtagactgtatccttggggtccttgtccttggaagcaatgtcttctccttgg   15935
FAM230B-LOC105372935-GGT2.NCBI.GGT2.rev.compl                       ttcaggttgtggactgtatccttggggtccttgtccttggaagcaatgtcttctccttgg   13902
                                                                    ********** *************************************************

chimp.LOC112206744.LOC107973052.GGT2-.646279-708040.rev.compl       attcagtattttgcacttgccaacctacgtggacctgagagatccaccatccagaagctg   15995
FAM230B-LOC105372935-GGT2.NCBI.GGT2.rev.compl                       attcaggattttgcacttgccaacctacgtggacctgagagatccaccatccagaagctg   13962
                                                                    ****** *****************************************************

chimp.LOC112206744.LOC107973052.GGT2-.646279-708040.rev.compl       atgtcttttccagtgtgtagcctacccttgttttggaggccttgaagttgactacacttt   16055
FAM230B-LOC105372935-GGT2.NCBI.GGT2.rev.compl                       atgtcttttccagtgtgtatcctacccttgttttggaggccttgaagttgactacacttt   14022
                                                                    ******************* ****************************************

chimp.LOC112206744.LOC107973052.GGT2-.646279-708040.rev.compl       ctgatcaagttttcagtattcattgagagaaacagagccttgtgcaaacaatccacaaca   16115
FAM230B-LOC105372935-GGT2.NCBI.GGT2.rev.compl                       ctgatcaagttttcagtattcattgagagaaacagagccttgtgcaaacaatccacaaca   14082
                                                                    ************************************************************

chimp.LOC112206744.LOC107973052.GGT2-.646279-708040.rev.compl       tgacatacccctcaaaaagctttgtttctgtattgcaggtggtgcaggtggcccctctga   16175
FAM230B-LOC105372935-GGT2.NCBI.GGT2.rev.compl                       tgagatacccctgaaaaagctttgtttctgtattgcaggtggtgcaggtggcccctctga   14142
                                                                    *** ******** ***********************************************

chimp.LOC112206744.LOC107973052.GGT2-.646279-708040.rev.compl       ggtgtgaatctagtaagtattctggaatcacttgccaagaaaacaatctggatgccaaga   16235
FAM230B-LOC105372935-GGT2.NCBI.GGT2.rev.compl                       ggcttgaatctagtaagtattctggaatcacttgccaagaaaacaatctggatgccaaga   14202
                                                                    **  ********************************************************

chimp.LOC112206744.LOC107973052.GGT2-.646279-708040.rev.compl       aaggtgtggcatccttgcctggtttcaatgtgaagagcctccctgatcctgggattgtga   16295
FAM230B-LOC105372935-GGT2.NCBI.GGT2.rev.compl                       aaggtgtggcatccttgcctggtttcaatgtgaagagcctccctgatcctgggattgtga   14262
                                                                    ************************************************************

chimp.LOC112206744.LOC107973052.GGT2-.646279-708040.rev.compl       taggaataagtataggggaagtgtttttttaaaacctgaattccccagggaaaaattatg   16355
FAM230B-LOC105372935-GGT2.NCBI.GGT2.rev.compl                       taggaataagtataggggaagtgtttttttaaaacctgaattccccagggaaaaattatg   14322
                                                                    ************************************************************

chimp.LOC112206744.LOC107973052.GGT2-.646279-708040.rev.compl       gccaaattttgaggaagcagttgtgctcccttttgggtggtgccgagttgggtgcttgag   16415
FAM230B-LOC105372935-GGT2.NCBI.GGT2.rev.compl                       gccaaattttgaggaagcagctgtgctcccttttgggtggtgctgagttgggtgcttgag   14382
                                                                    ******************** ********************** ****************

chimp.LOC112206744.LOC107973052.GGT2-.646279-708040.rev.compl       gattggtggtgtcttgtgtgaggctgcatcgtgtggtgaatatgtgtgtttctgtaca   16475
FAM230B-LOC105372935-GGT2.NCBI.GGT2.rev.compl                       gattggtggtgtcttgtgtgaggctgcatcgtgtggtgaatgtgtgtgtttctgtaca   14442
                                                                    ******************************************* ****************

chimp.LOC112206744.LOC107973052.GGT2-.646279-708040.rev.compl       ggtgaggctgtgtgttttctcaggagagatttcccacttatacaacccaatcaccagtgt   16535
FAM230B-LOC105372935-GGT2.NCBI.GGT2.rev.compl                       ggtgaggctgtgtgttttctcaggagagatttcccacttatacaacccaatcaccagtgt   14502
                                                                    ************************************************************

chimp.LOC112206744.LOC107973052.GGT2-.646279-708040.rev.compl       ccacttctaacaataaaatccacccccgctctactctctctgtacagtgactcctccacc   16595
FAM230B-LOC105372935-GGT2.NCBI.GGT2.rev.compl                       ccacttctaacaataaaatccacccccgctctactctctctgtacagtgactcctccacc   14562
                                                                    ************************************************************

chimp.LOC112206744.LOC107973052.GGT2-.646279-708040.rev.compl       ctcactagagccatccccgggtctgccttattatccccactgctcaggtggagaacctga   16655
FAM230B-LOC105372935-GGT2.NCBI.GGT2.rev.compl                       ctcaccagagccatccccgggtctgccttattatccccactgctcaggtggagaacctga   14622
                                                                    ***** ******************************************************

chimp.LOC112206744.LOC107973052.GGT2-.646279-708040.rev.compl       agggccaagggagtggccccagctcccgagttcctgaatgaaaaagtgaaaacacgaacc   16715
FAM230B-LOC105372935-GGT2.NCBI.GGT2.rev.compl                       agggccaagggagtggccccagctcccgagttcctgaatgaaacagtgaaaacacgaacc   14682
                                                                    ******************************************* ****************

chimp.LOC112206744.LOC107973052.GGT2-.646279-708040.rev.compl       cagaagtgtgggccagtgctgatgctgacatgcacttagtcatggggtgttcaccaccac   16775
FAM230B-LOC105372935-GGT2.NCBI.GGT2.rev.compl                       caggagtgtggaccagtgctgacgctgacatgcacttagtcatggggtgttcaccaccac   14742
```

```
                                                            *** ******* ********** *************************************

chimp.LOC112206744.LOC107973052.GGT2-.646279-708040.rev.compl      acagggagtccagcattcatgtataagccctaaagcaacgagcccaaaaggccccagaca   16835
FAM230B-LOC105372935-GGT2.NCBI.GGT2.rev.compl                      acacggagtccagcattcatgtataagccctaaagcaacgagcccaaaaggccccagaca   14802
                                                                   *** ********************************************************

chimp.LOC112206744.LOC107973052.GGT2-.646279-708040.rev.compl      ctgcccatcatcataaagtggcctccgtggtcacacaacccagggcagttataggctcat   16895
FAM230B-LOC105372935-GGT2.NCBI.GGT2.rev.compl                      ctgcccatcatcataaagtggcctccgtggtcacacaacccagggcagttatagcctcat   14862
                                                                   ****************************************************** *****

chimp.LOC112206744.LOC107973052.GGT2-.646279-708040.rev.compl      ctccccacggacaggcatagtcatcagtgtgtcaaaagcacaaagatccccaggtgtttg   16955
FAM230B-LOC105372935-GGT2.NCBI.GGT2.rev.compl                      ctccccacggaccgacatagtcatcagtgtgtcaaaagcacaaagatccccaggtgtttg   14922
                                                                   ************ * *********************************************

chimp.LOC112206744.LOC107973052.GGT2-.646279-708040.rev.compl      gctcagctcacagatcccttttttttttttaacttttaagttcaggggtacatgtgcagg   17015
FAM230B-LOC105372935-GGT2.NCBI.GGT2.rev.compl                      gctcagctcacagatccc-tttttttatttaacttttcagttcaggggtacatgtgcagg   14981
                                                                   ****************** ******* ********** **********************

chimp.LOC112206744.LOC107973052.GGT2-.646279-708040.rev.compl      atgtgcaggtttgctacatatataaatgtgtgtcatgagagtttgttgtacagattattg   17075
FAM230B-LOC105372935-GGT2.NCBI.GGT2.rev.compl                      atgtgcaggtttgctacatatataaatgtgtgttatgagagttttttgtacagattattg   15041
                                                                   ********************************* ********** ***************

chimp.LOC112206744.LOC107973052.GGT2-.646279-708040.rev.compl      catcacccatatattgagcctaatatcagttatttttcctgatcctccccctcctcccac   17135
FAM230B-LOC105372935-GGT2.NCBI.GGT2.rev.compl                      catcacccatatattgagcctaatatcagttatttttcctgatccaccccctcctcccac   15101
                                                                   ********************************************* **************

chimp.LOC112206744.LOC107973052.GGT2-.646279-708040.rev.compl      ctcccaccctccagtaggccccacgctcacaaattctaagaggagtgggggaccacaaag   17195
FAM230B-LOC105372935-GGT2.NCBI.GGT2.rev.compl                      ctcccaccctccagtaggccccacgctcacaaattctaagaggagtgggggaccacaaag   15161
                                                                   ************************************************************

chimp.LOC112206744.LOC107973052.GGT2-.646279-708040.rev.compl      gccagtgtggcccacttcagttgtgaagttaatttgctcagcaactggccaaagtctata   17255
FAM230B-LOC105372935-GGT2.NCBI.GGT2.rev.compl                      gccagtgtggcccacttcagttgtgaagttaatttgctcagcaactggccaaagtctata   15221
                                                                   ************************************************************

chimp.LOC112206744.LOC107973052.GGT2-.646279-708040.rev.compl      aggatgggtgatgtattttagtagatttagtaatactatcttcccaagccctaaaatgct   17315
FAM230B-LOC105372935-GGT2.NCBI.GGT2.rev.compl                      aggatgggtgatgtattttagtagatttagtaatactatcttcccaagccctaaaatgct   15281
                                                                   ************************************************************

chimp.LOC112206744.LOC107973052.GGT2-.646279-708040.rev.compl      caaatcctgccagccaaaaatggtgaggagggacagataggaactctgtgtggcacttgg   17375
FAM230B-LOC105372935-GGT2.NCBI.GGT2.rev.compl                      caaatcctgccagccaaaaatggtgaggagggacagataggaactctgtgtggcacttgg   15341
                                                                   ************************************************************

chimp.LOC112206744.LOC107973052.GGT2-.646279-708040.rev.compl      ttattagcctggcttccatcccttagtggcaagtctcttgtatatgtgggttaaagaccc   17435
FAM230B-LOC105372935-GGT2.NCBI.GGT2.rev.compl                      ttattagcctggcttccatcccttagtggcaactctcttgtatatgtgggttaaagaccc   15401
                                                                   ******************************** ***************************

chimp.LOC112206744.LOC107973052.GGT2-.646279-708040.rev.compl      tcagcctcaagccaagcctcctccatgaggagccatctcactattgactggctagtgccg   17495
FAM230B-LOC105372935-GGT2.NCBI.GGT2.rev.compl                      tcagcctcaagccaagcctcctccatgaggagccatctcactattgaccggctagtgccg   15461
                                                                   ************************************************ ***********

chimp.LOC112206744.LOC107973052.GGT2-.646279-708040.rev.compl      ggtatggccaccagcccaactgaaacaaaatgttgctttaaaacaagtgtaaatctcata   17555
FAM230B-LOC105372935-GGT2.NCBI.GGT2.rev.compl                      ggtatggccaccagcccaactgaaacaaaatgttgctttaaaacaagtgtaaatctcata   15521
                                                                   ************************************************************

chimp.LOC112206744.LOC107973052.GGT2-.646279-708040.rev.compl      catacaacaggcaaatgcagaagcagtgtggtctcgcaagttgtaaagaggacagtcgca   17615
FAM230B-LOC105372935-GGT2.NCBI.GGT2.rev.compl                      catacaacaggcaaatgcagaagcagtgtggtctcgcaagttgtaaagaggacagtcgca   15581
                                                                   ************************************************************

chimp.LOC112206744.LOC107973052.GGT2-.646279-708040.rev.compl      attttgctggacttcaacctgggtagaagacatgagggaactctgtcactaaatcacggc   17675
FAM230B-LOC105372935-GGT2.NCBI.GGT2.rev.compl                      attttgctggacttcaacctgggtagaagacatgagggaac-ctgtcactaaatcacggc   15640
                                                                   ***************************************** ******************

chimp.LOC112206744.LOC107973052.GGT2-.646279-708040.rev.compl      agagttcaaggccacttgcagactatttcatgttacagaaggtggcctttagctactaag   17735
FAM230B-LOC105372935-GGT2.NCBI.GGT2.rev.compl                      agagttcaaggccacttgtagactatttcatgttacagaaggtggcctttagctactaag   15700
                                                                   ****************** *****************************************

chimp.LOC112206744.LOC107973052.GGT2-.646279-708040.rev.compl      caaaggcctctgtttctcatttctttcctgttcatcttcttggtcatccttcttccgcaa   17795
FAM230B-LOC105372935-GGT2.NCBI.GGT2.rev.compl                      caaaggcctctgtttctcatttctttcctgttcatcttcttggtcatccttcttccgcaa   15760
                                                                   ************************************************************

chimp.LOC112206744.LOC107973052.GGT2-.646279-708040.rev.compl      gggaaacgagcccaagcaaaaggcagtttcaatattaatttgaccgaggttttgtgcagt   17855
FAM230B-LOC105372935-GGT2.NCBI.GGT2.rev.compl                      gggaaacgagcccaagcaaaaggcagtttcaatattaatttgaccgaggttttgtgcagt   15820
                                                                   ************************************************************

chimp.LOC112206744.LOC107973052.GGT2-.646279-708040.rev.compl      ttattatcatccaggtaatcaagtgcaacccagtctgcctagcagcccccctatctctgc   17915
FAM230B-LOC105372935-GGT2.NCBI.GGT2.rev.compl                      ttattatcatccaggtaatcaggtgcaacccagtctgcctagccgcccccctatctctgc   15880
                                                                   ********************* ********************* ****************

chimp.LOC112206744.LOC107973052.GGT2-.646279-708040.rev.compl      tctgtgttttcatttaataaacattttggtctacttactatgtgctagattttctcgaga   17975
FAM230B-LOC105372935-GGT2.NCBI.GGT2.rev.compl                      tctgtgttttcatttaataaacattttggtctacttactatgtgctagattttctcgaga   15940
                                                                   ************************************************************

chimp.LOC112206744.LOC107973052.GGT2-.646279-708040.rev.compl      ccaagtaaatgagatagaatctttatgttggcagctaagttagatttaacataactgaca   18035
FAM230B-LOC105372935-GGT2.NCBI.GGT2.rev.compl                      ccaagtaaatgagatagaatctttatgctggcagctaagttagatttcacataactgaca   16000
                                                                   ************************** ******************** ************

chimp.LOC112206744.LOC107973052.GGT2-.646279-708040.rev.compl      aaaattaaaatttctgatttcttgtaaaaatatttgtatgtgtgaatgcatactgaatg   18095
FAM230B-LOC105372935-GGT2.NCBI.GGT2.rev.compl                      aaaattaaaatttctgatttcttgtaaaaatatttgtatgtgtgaatgcatactgaatg   16060
                                                                   ************************************************************

chimp.LOC112206744.LOC107973052.GGT2-.646279-708040.rev.compl      taaagtggataaaaaactcacacttgcactcatggaaggcttttcatgaatttgtcaatt   18155
FAM230B-LOC105372935-GGT2.NCBI.GGT2.rev.compl                      taaagtggataaaaaactcacacttgcactcatggaaggcttttcatgaatttgtcaatt   16120
                                                                   ************************************************************

chimp.LOC112206744.LOC107973052.GGT2-.646279-708040.rev.compl      tttatttttatatttccccacttcaccggataatgcatacctgaacctggaaattgatt   18215
FAM230B-LOC105372935-GGT2.NCBI.GGT2.rev.compl                      tttatttttatatttccccacttcaccagataatgcatacctgaacctggaaactgatt   16180
                                                                   **************************** ************************ *****

chimp.LOC112206744.LOC107973052.GGT2-.646279-708040.rev.compl      cccactgcagaaagtgttctgagccacatcccttagcttcactagtgcaggtccacctgg   18275
FAM230B-LOC105372935-GGT2.NCBI.GGT2.rev.compl                      cccactgcagaaagtgttctgagccacatcccttagcttcactagtgcaggtccacctgg   16240
                                                                   ************************************************************

chimp.LOC112206744.LOC107973052.GGT2-.646279-708040.rev.compl      gaggatgtcccagcatcagcttggcccatgctgtgatcagccacctccatgcaccacacc   18335
FAM230B-LOC105372935-GGT2.NCBI.GGT2.rev.compl                      gaggatgtcccagcatcagcttgacccatgctgtgatcagccacctccatgcatcacacc   16300
                                                                   *********************** ***************************** ******

chimp.LOC112206744.LOC107973052.GGT2-.646279-708040.rev.compl      aagcaagcccctgggtgattcacagtctccaccaccagggcactgaccttaactgtgtgt   18395
FAM230B-LOC105372935-GGT2.NCBI.GGT2.rev.compl                      aagcaagcccctgggtgattcacagtctccaccaccagggcactgaccttaactctgtgt   16360
                                                                   ****************************************************** *****

chimp.LOC112206744.LOC107973052.GGT2-.646279-708040.rev.compl      tcttctagctccccatgaagacaccgtacacgacatcactaacgaggacgccacacacga   18455
FAM230B-LOC105372935-GGT2.NCBI.GGT2.rev.compl                      ttttctagctccccatgaggacaccgtacacgacatcactaacgaggacgccacacacga   16420
                                                                   * **************** *****************************************

chimp.LOC112206744.LOC107973052.GGT2-.646279-708040.rev.compl      catcgccaacgaggacactgtacatgacaccgctaacgaggccgccgacaagggcatcgc   18515
FAM230B-LOC105372935-GGT2.NCBI.GGT2.rev.compl                      catcgctaacgaggatactgtacacgacatcgctaacgaggccgccgacaagggcatcgc   16480
                                                                   ****** ******** ******** **** ******************************

chimp.LOC112206744.LOC107973052.GGT2-.646279-708040.rev.compl      cagcgaggacgccgcccagggcatcgccaacgaggccgccgcccagggcatcgtcagcga   18575
FAM230B-LOC105372935-GGT2.NCBI.GGT2.rev.compl                      caacgaggatgccgcccacggcatcgccaacgagtacgccgcccacggcatcgccagcga   16540
                                                                   ** ****** ******** ***************  ********* ******* ******

chimp.LOC112206744.LOC107973052.GGT2-.646279-708040.rev.compl      ggacgccgcccacggaatcgccaacgaggacgccgtccagggcatcgccaacgtggtcgc   18635
FAM230B-LOC105372935-GGT2.NCBI.GGT2.rev.compl                      ggacgccgcccacggcatcgccagcgaggacgccgcccagggcatcgccagcgaggacgc   16600
                                                                   *************** ******* *********** ************** ** ** ***

chimp.LOC112206744.LOC107973052.GGT2-.646279-708040.rev.compl      cgcccacggaatcgccaacgaggacgccgcccagggcatcgc------------------   18677
FAM230B-LOC105372935-GGT2.NCBI.GGT2.rev.compl                      cgcccagggcatcgccaacgaggacaccatccagggcatcgccaaggagtacgccgtcca   16660
                                                                   ****** ** *************** **  ************

chimp.LOC112206744.LOC107973052.GGT2-.646279-708040.rev.compl      ------------------------------------------------------------   18677
FAM230B-LOC105372935-GGT2.NCBI.GGT2.rev.compl                      cggcatcgccaacgaggacgccgcccagggcatcgccaacgaggacgccgcccagggcat   16720

chimp.LOC112206744.LOC107973052.GGT2-.646279-708040.rev.compl      ------------------------------------------------------------   18677
FAM230B-LOC105372935-GGT2.NCBI.GGT2.rev.compl                      cgccaacgaggacgccgcccagggcatcgccaaggaggacgccgcccagggcatcgccaa   16780

chimp.LOC112206744.LOC107973052.GGT2-.646279-708040.rev.compl      ------------------------------------------------------------   18677
FAM230B-LOC105372935-GGT2.NCBI.GGT2.rev.compl                      cgagggcgccgcccagggcatcgccaaagaggacgccgcccagggcatcgctaacgaggc   16840

chimp.LOC112206744.LOC107973052.GGT2-.646279-708040.rev.compl      ------------------------------------------------------------   18677
FAM230B-LOC105372935-GGT2.NCBI.GGT2.rev.compl                      cgccgacaagggcatcgccaacgaggatgccgcccacggcatcgccaacgaggacgccgc   16900
```

```
chimp.LOC112206744.LOC107973052.GGT2-.646279-708040.rev.compl      ------------------------------------------------------------   18677
FAM230B-LOC105372935-GGT2.NCBI.GGT2.rev.compl                      ccacggcatcgccagcgaggacgccgcccacggcatcgccagcgaggacgccgcccacgg   16960

chimp.LOC112206744.LOC107973052.GGT2-.646279-708040.rev.compl      ---------------------------catcgctaatgaggatgccatatatgacatcgc   18710
FAM230B-LOC105372935-GGT2.NCBI.GGT2.rev.compl                      catcgccagcgaggacgccgcccagggcatcgccaacgaggacgccatccagggcatcgc   17020
                                                                                              ****** ** ***** *****  * * ******

chimp.LOC112206744.LOC107973052.GGT2-.646279-708040.rev.compl      taatg---acaccgtacaaggcat------------------------------------   18731
FAM230B-LOC105372935-GGT2.NCBI.GGT2.rev.compl                      caaggagtacgccgtccacggcatcgccaacgaggacgccgcccagggcatcgccaacga   17080
                                                                    ** *   ** **** ** *****

chimp.LOC112206744.LOC107973052.GGT2-.646279-708040.rev.compl      ------------------------------------------------------------   18731
FAM230B-LOC105372935-GGT2.NCBI.GGT2.rev.compl                      ggacgccgcccagggcatcgccaacgaggacgccgcccagggcatcgccaaggaggacgc   17140

chimp.LOC112206744.LOC107973052.GGT2-.646279-708040.rev.compl      ------------------------------------------------------------   18731
FAM230B-LOC105372935-GGT2.NCBI.GGT2.rev.compl                      cgcccagggcatcgccaaggaggacgccgcccagggcatcgccaacgagggcgccgccca   17200

chimp.LOC112206744.LOC107973052.GGT2-.646279-708040.rev.compl      ------------------------------------------------------------   18731
FAM230B-LOC105372935-GGT2.NCBI.GGT2.rev.compl                      gggcatcgccaaagaggacgccgcccacggcatcgccaacgaggacgccgcccagggcat   17260

chimp.LOC112206744.LOC107973052.GGT2-.646279-708040.rev.compl      ------------------------------------------------------------   18731
FAM230B-LOC105372935-GGT2.NCBI.GGT2.rev.compl                      cgccaacgaggacgccgcccacggaatcgccagtgaggacgccgcccacggcatcgccag   17320

chimp.LOC112206744.LOC107973052.GGT2-.646279-708040.rev.compl      ------------------------------------------------------------   18731
FAM230B-LOC105372935-GGT2.NCBI.GGT2.rev.compl                      cgaggacgccgtccagggcatcgacaaggaggatgccgcccagggcatcgccaacgagga   17380

chimp.LOC112206744.LOC107973052.GGT2-.646279-708040.rev.compl      ------------------------------------------------------------   18731
FAM230B-LOC105372935-GGT2.NCBI.GGT2.rev.compl                      cgccgcccagggcatcgccaacgaggacgccgcccagggcatcgccaaagaggacgccgc   17440

chimp.LOC112206744.LOC107973052.GGT2-.646279-708040.rev.compl      ---------------------------------gctaacgaagacatcgtacaagccgtc   18758
FAM230B-LOC105372935-GGT2.NCBI.GGT2.rev.compl                      ccacggcatcgccaacgagctgtatacgacatcgctaacgaggacaccctacaagccgtc   17500
                                                                                                    ******** **** * ***********

chimp.LOC112206744.LOC107973052.GGT2-.646279-708040.rev.compl      gctaacaagtacactgtacacaacatcgctaatgagggcactgtacaagacatcaccaat   18818
FAM230B-LOC105372935-GGT2.NCBI.GGT2.rev.compl                      gctaacaagtacactgtacacaacatcgctaatgagggcactgtacaagacatcaccaat   17560
                                                                   ************************************************************

chimp.LOC112206744.LOC107973052.GGT2-.646279-708040.rev.compl      gaggacgctttatacgacattgctaatggcaccgacaaggcacgctaacgtggatgctgt   18878
FAM230B-LOC105372935-GGT2.NCBI.GGT2.rev.compl                      gagggcgctttatacgacattgctaatggcaccgacaaggcacgctaacgtggacgctgt   17620
                                                                   **** ************************************************* *****

chimp.LOC112206744.LOC107973052.GGT2-.646279-708040.rev.compl      acacgacattgctaatgaggacaccgtataagacatcgctagtaactatcgcaagaacaa   18938
FAM230B-LOC105372935-GGT2.NCBI.GGT2.rev.compl                      acacgacattgctaatgaggacagcgtataagacatcgctagtaactatcgcaagaacaa   17680
                                                                   *********************** ************************************

chimp.LOC112206744.LOC107973052.GGT2-.646279-708040.rev.compl      aaaaccaaacaccgcatattctcactcataggtgggaattgaacaatgagatcacatgga   18998
FAM230B-LOC105372935-GGT2.NCBI.GGT2.rev.compl                      aaaaccaaacaccgcatattctcactcatagatgggaattgaacaatgagatcacatgga   17740
                                                                   ******************************* ****************************

chimp.LOC112206744.LOC107973052.GGT2-.646279-708040.rev.compl      cacaggaaggggaatatcacactctggggactgttgtggggtgggggagggggaggga   19058
FAM230B-LOC105372935-GGT2.NCBI.GGT2.rev.compl                      cacaggaaggggaatatcacactctggggactgttgtggggttgg----gggggaggga   17796
                                                                   ****************************************** **    ***********

chimp.LOC112206744.LOC107973052.GGT2-.646279-708040.rev.compl      tagcatcgggagatatacctaatgctagatgatgagttagtgggtgcagcgcaccagcat   19118
FAM230B-LOC105372935-GGT2.NCBI.GGT2.rev.compl                      tagcatcgggagatatacctaatgctagatgatgagttagtgggtgcagcgcaccagcac   17856
                                                                   ***********************************************************

chimp.LOC112206744.LOC107973052.GGT2-.646279-708040.rev.compl      ggcacatgtatacatatgtaactaacctgcacaatgtgcacatgtaccctaaaacttaaa   19178
FAM230B-LOC105372935-GGT2.NCBI.GGT2.rev.compl                      ggcacatgtatacatatgtaactaacctgcacaatgtgcacatgtaccctaaaacttaaa   17916
                                                                   ************************************************************

chimp.LOC112206744.LOC107973052.GGT2-.646279-708040.rev.compl      gtatatatataaaaaaaaagacatcgctagtgagcacgctgtatacgacatcactaatga   19238
FAM230B-LOC105372935-GGT2.NCBI.GGT2.rev.compl                      gtatatatataaaaaaaaagacatcgctagtgagcacgc---------------------   17955
                                                                   ***************************************

chimp.LOC112206744.LOC107973052.GGT2-.646279-708040.rev.compl      ggatgctatatatgacatcgctgatgaggacattgtatacgacatcactaatgaggacac   19298
FAM230B-LOC105372935-GGT2.NCBI.GGT2.rev.compl                      ---------------------------------tgtatacgacatcgctaatgaggacac   17982
                                                                                                    ************* *************

chimp.LOC112206744.LOC107973052.GGT2-.646279-708040.rev.compl      catacaaggcatcgctaacaatgatgctgtacacaacatcactaatgatgacaccgtata   19358
FAM230B-LOC105372935-GGT2.NCBI.GGT2.rev.compl                      catacaaggcatcgctaacgatgacgctgtacacaacatcactaatgatgacaccgtata   18042
                                                                   ******************* **** ***********************************

chimp.LOC112206744.LOC107973052.GGT2-.646279-708040.rev.compl      acacatcgctaattatgacgctgtatacgacatcgctaatgacaccgtacaaggcacact   19418
FAM230B-LOC105372935-GGT2.NCBI.GGT2.rev.compl                      agacatcgctaattatgacgctgtatacgacatcgctaatgacaccgtacaaggcacgct   18102
                                                                   * ******************************************************* **

chimp.LOC112206744.LOC107973052.GGT2-.646279-708040.rev.compl      aatgaggatgctgtacacgacatcactaatgaggacagtgtacaagccatcgctaatgag   19478
FAM230B-LOC105372935-GGT2.NCBI.GGT2.rev.compl                      aacgaggatgctgtacacgacatcactaatgaggacagtgtacaagccatcactaatgag   18162
                                                                   ** ************************************************ ********

chimp.LOC112206744.LOC107973052.GGT2-.646279-708040.rev.compl      gacactgtatatgacatcgctaacgaggacactgtacaaggcattgctaacgaggacgct   19538
FAM230B-LOC105372935-GGT2.NCBI.GGT2.rev.compl                      gacactgtatatggcatcgctaacgaggacactgtacaagccattgctaacgaggacgct   18222
                                                                   ************ **************************** ******************

chimp.LOC112206744.LOC107973052.GGT2-.646279-708040.rev.compl      gtac---acatcgctaatgaggacaccatataagacatcaccaatgaggatgctgtatat   19595
FAM230B-LOC105372935-GGT2.NCBI.GGT2.rev.compl                      gtacacaacatcgctaatgaggacaccatataagacatcaccaatgaggatgctgtatat   18282
                                                                   ****   *****************************************************

chimp.LOC112206744.LOC107973052.GGT2-.646279-708040.rev.compl      gacatcactaatgacacccacaaggcatgctaacgaggacgctgtagacgacattgctta   19655
FAM230B-LOC105372935-GGT2.NCBI.GGT2.rev.compl                      gacatcgctaatggcacccacaaggcatgctaacgaggacgctgtagacgacattgctta   18342
                                                                   ****** ****** **********************************************

chimp.LOC112206744.LOC107973052.GGT2-.646279-708040.rev.compl      taaggacaccgtacaagacatcgctaacgaggacgctgtatacgacatcgctaatgagga   19715
FAM230B-LOC105372935-GGT2.NCBI.GGT2.rev.compl                      taaggacaccgtacaagacatcgctaacgaggacgctgtatacgacatcgctaatgagga   18402
                                                                   ************************************************************

chimp.LOC112206744.LOC107973052.GGT2-.646279-708040.rev.compl      cgttgtatatgacatcgctaatgaggatgctttacaagacatagctaatgaggttgctgt   19775
FAM230B-LOC105372935-GGT2.NCBI.GGT2.rev.compl                      cgttgtatatgacatcgctaatgaggatgctttacaagacatagctaatgaggttgctgt   18462
                                                                   ************************************************************

chimp.LOC112206744.LOC107973052.GGT2-.646279-708040.rev.compl      atatgatatcgctaatgaggacattgtatatgacatcgctaatgaggacgctctatacga   19835
FAM230B-LOC105372935-GGT2.NCBI.GGT2.rev.compl                      atatgacatcgctaatgaggacattgtatatgacatcgctaatgaggacgctctatacga   18522
                                                                   ****** *****************************************************

chimp.LOC112206744.LOC107973052.GGT2-.646279-708040.rev.compl      catcactaatgaggatgctgtatacaacatcgctaatgaggacgctgtatatggcatcgc   19895
FAM230B-LOC105372935-GGT2.NCBI.GGT2.rev.compl                      catcactaatgaggacgctgtatacaacatcgctaatgaggacgctgtatatggcatcgc   18582
                                                                   *************** ********************************************

chimp.LOC112206744.LOC107973052.GGT2-.646279-708040.rev.compl      taatgaggatgctgtatacgaattcgctaataaggacgctgtatatgacattgctaatga   19955
FAM230B-LOC105372935-GGT2.NCBI.GGT2.rev.compl                      taatgaggatgctgtatacgaattcgctaataaggacgctgtatatgacattgctaatga   18642
                                                                   ************************************************************

chimp.LOC112206744.LOC107973052.GGT2-.646279-708040.rev.compl      ggacactatacaagacatctgtaaaaaagaagatgctgccaatgtaagacacttttcttt   20015
FAM230B-LOC105372935-GGT2.NCBI.GGT2.rev.compl                      ggacactgtacaagacatctgtaaaaaagaagatgctgccaatgtaagacacttttcttt   18702
                                                                   ******* ****************************************************

chimp.LOC112206744.LOC107973052.GGT2-.646279-708040.rev.compl      gtcttgaacagaaatgttactttcctggcttctttccaatcagatgtagacatgaacatc   20075
FAM230B-LOC105372935-GGT2.NCBI.GGT2.rev.compl                      gtcttgaacagaaatgttactttcctggcttctttccaatcagatgtagacatgaacatc   18762
                                                                   ************************************************************

chimp.LOC112206744.LOC107973052.GGT2-.646279-708040.rev.compl      tgccagtgtgcattattgatgtcatctgcagtttaatcaaatgtagacatgaacatctgc   20135
FAM230B-LOC105372935-GGT2.NCBI.GGT2.rev.compl                      tgccagtgtgcattatcgatgtcatctgcagtttaatcaaatgtagacatgaacatctgc   18822
                                                                   **************** *******************************************

chimp.LOC112206744.LOC107973052.GGT2-.646279-708040.rev.compl      caatgtggactatttatgacatctgcaattcccttggtgtggtgctattgattggcagcc   20195
FAM230B-LOC105372935-GGT2.NCBI.GGT2.rev.compl                      caatgtggactatttatgacatctgcaattcccttggtgtggtgctattgattggcagcc   18882
                                                                   ************************************************************

chimp.LOC112206744.LOC107973052.GGT2-.646279-708040.rev.compl      tctcaccaacccatgccaggcacactggggtgtggtagatggcagcatccacgatccact   20255
FAM230B-LOC105372935-GGT2.NCBI.GGT2.rev.compl                      tctcaccaacccatgccaggcacactggggtgtggtagatggcagcatccacgatccact   18942
                                                                   ************************************************************

chimp.LOC112206744.LOC107973052.GGT2-.646279-708040.rev.compl      gcaatgcagaggtgtttccctccacagcagtttcccccatggattaagagttgtgaaac   20315
FAM230B-LOC105372935-GGT2.NCBI.GGT2.rev.compl                      gcaatgcagaggtgtttccctccacagcagtttcccccatggattaagagttgtgaaac   19002
                                                                   ************************************************************
```

```
chimp.LOC112206744.LOC107973052.GGT2-.646279-708040.rev.compl      tgccaatctagatacactttaaagataaattctgtgggaaaaggtcttgtcttttccaca   20375
FAM230B-LOC105372935-GGT2.NCBI.GGT2.rev.compl                      tgccaatctagatacactttaaagataaattctgtgggaaaaggtcttgtcttttccaca   19062
                                                                   ************************************************************

chimp.LOC112206744.LOC107973052.GGT2-.646279-708040.rev.compl      ggtgtcttccgtgccagttttgggggacttcgacctttgactcaatcactataccccttc   20435
FAM230B-LOC105372935-GGT2.NCBI.GGT2.rev.compl                      ggtgtcttccgtgccagttttgggggacttcgacctttgactcaatcactataccccttc   19122
                                                                   ************************************************************

chimp.LOC112206744.LOC107973052.GGT2-.646279-708040.rev.compl      ttattttctctctcaagttgtcgagagactatcagatctgtgtgacgtgtatggcatcat   20495
FAM230B-LOC105372935-GGT2.NCBI.GGT2.rev.compl                      ctattttctctctcaagttgtcgagagactatcagatctgtgtgacgtgtatggcatcat   19182
                                                                    ***********************************************************

chimp.LOC112206744.LOC107973052.GGT2-.646279-708040.rev.compl      ttcaccctcctaatgttttcttttctataattgcaggagccattgacattggagaatgat   20555
FAM230B-LOC105372935-GGT2.NCBI.GGT2.rev.compl                      ttcaccctcctaatgttttcttttctataattgcaggagccattgacactggagaatgat   19242
                                                                   ************************************************ ***********

chimp.LOC112206744.LOC107973052.GGT2-.646279-708040.rev.compl      acgtaccctgaaataactcacttcctgaggaaaaagcgccatctctagggtacagaaacc   20615
FAM230B-LOC105372935-GGT2.NCBI.GGT2.rev.compl                      acgtaccctgaaataactcacttcctgaggaaaaagcgccatctctagggtacagaaacc   19302
                                                                   ************************************************************

chimp.LOC112206744.LOC107973052.GGT2-.646279-708040.rev.compl      tgattctgggctccttttgggaaggaggatttggggtctggtgagagcaaatgattttgc   20675
FAM230B-LOC105372935-GGT2.NCBI.GGT2.rev.compl                      tgattctgggctccttttgggaaggaggatttggggtctggtgagagcaaatgattttgc   19362
                                                                   ************************************************************

chimp.LOC112206744.LOC107973052.GGT2-.646279-708040.rev.compl      aagtataaaacaatgtccagagaggctgtaggatatctgtgagcccagaggaaacacca   20735
FAM230B-LOC105372935-GGT2.NCBI.GGT2.rev.compl                      aagtataaaaccatgtccagagaggctgtaggatatctgtgagcccagaggaaacacca   19422
                                                                   ********** *************************************************

chimp.LOC112206744.LOC107973052.GGT2-.646279-708040.rev.compl      ggggatcctgtgcgaagcaccatggcttcagctagggtgggaggagtgggtgggcctctc   20795
FAM230B-LOC105372935-GGT2.NCBI.GGT2.rev.compl                      ggggatcctgtgcgaagcaccatggcttcagctagggtgggaggagtgggtgggcctctc   19482
                                                                   ************************************************************

chimp.LOC112206744.LOC107973052.GGT2-.646279-708040.rev.compl      tctaatgacttatcctggtgtttgtgtttctaaagatttgattgtggagagcatatctga   20855
FAM230B-LOC105372935-GGT2.NCBI.GGT2.rev.compl                      tctaatgacttatcctggtgtttgtgtttctaaagatttgattgtggagagcatatctga   19542
                                                                   ************************************************************

chimp.LOC112206744.LOC107973052.GGT2-.646279-708040.rev.compl      tgatggggatttgtaggtaggtaactactttccacgtaagatccaattggagagagttcc   20915
FAM230B-LOC105372935-GGT2.NCBI.GGT2.rev.compl                      tgatggggatttgtaggtaggtaactgctttccacgtaagatccaattggagagagttcc   19602
                                                                   ************************** *********************************

chimp.LOC112206744.LOC107973052.GGT2-.646279-708040.rev.compl      caggggccttcggggtatccatgctgcttgggaggttaagggagggggcatgaaatcaaa   20975
FAM230B-LOC105372935-GGT2.NCBI.GGT2.rev.compl                      caggggccttcggggtatccatgctgcttgggaggttaagggagggggcatgaaatcaaa   19662
                                                                   ************************************************************

chimp.LOC112206744.LOC107973052.GGT2-.646279-708040.rev.compl      acgaaacaggaaatatgtgtcatattggatttggtcttttccgggtttattggcataata   21035
FAM230B-LOC105372935-GGT2.NCBI.GGT2.rev.compl                      acgaaacaggaaatatgtgtcatatcggatttggtcttttccgggtttattggcataata   19722
                                                                   ************************* **********************************

chimp.LOC112206744.LOC107973052.GGT2-.646279-708040.rev.compl      gtaagaactgtctctctgggctatgagggtgctgtgttatttaaaggtggtctttcccag   21095
FAM230B-LOC105372935-GGT2.NCBI.GGT2.rev.compl                      gttagaactgtctctctgggctatgagggtgctgtgttatttaaaggtggtctttcccag   19782
                                                                   ** *********************************************************

chimp.LOC112206744.LOC107973052.GGT2-.646279-708040.rev.compl      aacacctggccttttcttttccgcctctgccaaacatcacagactttgggttggattagt   21155
FAM230B-LOC105372935-GGT2.NCBI.GGT2.rev.compl                      aacacctggccttttcttttctgcctctgccaaacatcacagcctttgggttggattagt   19842
                                                                   ********************* ******************** *****************

chimp.LOC112206744.LOC107973052.GGT2-.646279-708040.rev.compl      cagcaccccttgggattgtgcagaagaggtttggggttgcatcgagtgtcacctgtggtg   21215
FAM230B-LOC105372935-GGT2.NCBI.GGT2.rev.compl                      cagcaccccttgggattgtgcagaagaggtttggggttgcattgagtgtcacctgtggtg   19902
                                                                   ****************************************** *****************

chimp.LOC112206744.LOC107973052.GGT2-.646279-708040.rev.compl      aacagaatctgaggacacaactctctcacaggcacttccttcaacctggagacagagtt   21275
FAM230B-LOC105372935-GGT2.NCBI.GGT2.rev.compl                      aacagaatctgaggacacaactctctcacaggcacttcctccaacctggagacagagtt   19962
                                                                   ***************************************** ******************

chimp.LOC112206744.LOC107973052.GGT2-.646279-708040.rev.compl      ctcctggtgtgtgcccaggggtgcaggagaaattgacagtctgcctctgaactttcagga   21335
FAM230B-LOC105372935-GGT2.NCBI.GGT2.rev.compl                      ctcctggtgtgtgcccaggggtggaggagaaattgacagtctgcctctgaactttcagga   20022
                                                                   *********************** ************************************

chimp.LOC112206744.LOC107973052.GGT2-.646279-708040.rev.compl      ctttaaaaagcactcatgtttccatcctcgctgttgactcctggcttaaagggatctcca   21395
FAM230B-LOC105372935-GGT2.NCBI.GGT2.rev.compl                      ctttaaaaagcactcatgtttccatcctcactgttgactcctggcttaaagggatctccc   20082
                                                                   ***************************** ***************************** 

chimp.LOC112206744.LOC107973052.GGT2-.646279-708040.rev.compl      ggggtgagtgaggaggagggatcggaccctggcagtctgacggcagcacctgtgttcctc   21455
FAM230B-LOC105372935-GGT2.NCBI.GGT2.rev.compl                      ggggtgagtgaggaggcgggatcggaccctggcagtctgacggcagcacctgtgttcctc   20142
                                                                   **************** *******************************************

chimp.LOC112206744.LOC107973052.GGT2-.646279-708040.rev.compl      tgcactgggccgtggatgacattacacaccttggtgagaatcaggaattgaggctaacca   21515
FAM230B-LOC105372935-GGT2.NCBI.GGT2.rev.compl                      tgcactgggccgtggatgacattacataccttggtgagaatcaggaattgaggctaacca   20202
                                                                   ************************** *********************************

chimp.LOC112206744.LOC107973052.GGT2-.646279-708040.rev.compl      catctgaaattgagatgggccttgagtcatataaatagtttggaaaagatgcattttact   21575
FAM230B-LOC105372935-GGT2.NCBI.GGT2.rev.compl                      catctgaaattgagatgggccttgagtcatataaatagtttggaaaagatgcattttact   20262
                                                                   ************************************************************

chimp.LOC112206744.LOC107973052.GGT2-.646279-708040.rev.compl      acgctattgaaagaaaccatttatttctcactccagcaggataaatggttttcagtatcc   21635
FAM230B-LOC105372935-GGT2.NCBI.GGT2.rev.compl                      acgctattgaaagaaaccatttatttctcactccagcaggataaatggttttcagtatcc   20322
                                                                   ************************************************************

chimp.LOC112206744.LOC107973052.GGT2-.646279-708040.rev.compl      atttaactgctcattgactcttactgtagatgaggaggtggccagcagcccctgccctcc   21695
FAM230B-LOC105372935-GGT2.NCBI.GGT2.rev.compl                      atttaactgctcattgactcttactgtagatgaggaggtggccagcagcccctgccctcc   20382
                                                                   ************************************************************

chimp.LOC112206744.LOC107973052.GGT2-.646279-708040.rev.compl      cccagtt-gtaggcccaaggtaaccagcaatggactggatataatggaagagtggtgcat   21754
FAM230B-LOC105372935-GGT2.NCBI.GGT2.rev.compl                      cccagttggtaggcccaaggtaaccagcaattgactggatataatggaagagtggtgcat   20442
                                                                   ******* *********************** ****************************

chimp.LOC112206744.LOC107973052.GGT2-.646279-708040.rev.compl      tcggaggtatctgtattaatgggacccacatgatatggatgagagctattagggtgagaa   21814
FAM230B-LOC105372935-GGT2.NCBI.GGT2.rev.compl                      tcgggggtatctgtattaatgggacccacatgatatggatgagagctattagggtgagaa   20502
                                                                   **** *******************************************************

chimp.LOC112206744.LOC107973052.GGT2-.646279-708040.rev.compl      aaagcctgggagcacaatgaaatatttaaatattaaacaaaacattgttgaaatctccat   21874
FAM230B-LOC105372935-GGT2.NCBI.GGT2.rev.compl                      aaagcctgggagcacaatgaaatatttaaatattaaacaaaacattgttgaaatctccat   20562
                                                                   ************************************************************

chimp.LOC112206744.LOC107973052.GGT2-.646279-708040.rev.compl      tgtactttagtagttgaagtcattcttgtggtcatcactgcctttcccaagcataacaag   21934
FAM230B-LOC105372935-GGT2.NCBI.GGT2.rev.compl                      tgtactttagtagttgaagtcattcttgtggtcatcactgcctttcccaagcataacaag   20622
                                                                   ************************************************************

chimp.LOC112206744.LOC107973052.GGT2-.646279-708040.rev.compl      ctacttaatatcacatagacccgtgccatgaggaatgatgatcagtttgtaaaatgccaa   21994
FAM230B-LOC105372935-GGT2.NCBI.GGT2.rev.compl                      ctacttaatatcacatggacccgtgccatgaggaatgatgatcagtttgtaaaatgccaa   20682
                                                                   **************** *******************************************

chimp.LOC112206744.LOC107973052.GGT2-.646279-708040.rev.compl      taaaacaattgcctatagaagccacaatgtttcatccatatatttcaatttccatgtgta   22054
FAM230B-LOC105372935-GGT2.NCBI.GGT2.rev.compl                      taaaacaattgcctatataagccacaatgtttcatccatatatttcaatttccatgtgta   20742
                                                                   ***************** ******************************************

chimp.LOC112206744.LOC107973052.GGT2-.646279-708040.rev.compl      agtatagttcaaatttcagaaatttattattatctaatagaatatgcatggtatatcaat   22114
FAM230B-LOC105372935-GGT2.NCBI.GGT2.rev.compl                      agtatagttcaaatttcagaaatttattattatctaatagaatatgcatggtatatcaat   20802
                                                                   ************************************************************

chimp.LOC112206744.LOC107973052.GGT2-.646279-708040.rev.compl      gagcaattatcatactgtttctattaacaattatttgtatgatgaaaaaagcagactccc   22174
FAM230B-LOC105372935-GGT2.NCBI.GGT2.rev.compl                      gagcaattatcatactgtttctattaacaattatttgtatgatgaaaaaagcagactccc   20862
                                                                   ************************************************************

chimp.LOC112206744.LOC107973052.GGT2-.646279-708040.rev.compl      attcttggatttttctcagtttgcacacattagcatgacagccccatttccacctgacat   22234
FAM230B-LOC105372935-GGT2.NCBI.GGT2.rev.compl                      attcttggatttttctcagtttgcacacattagcatgacagccccatttccacctgacat   20922
                                                                   ************************************************************

chimp.LOC112206744.LOC107973052.GGT2-.646279-708040.rev.compl      gtgccagcaagaggccaggaacagaggcttttcttat----taagatttctaaatgtatt   22290
FAM230B-LOC105372935-GGT2.NCBI.GGT2.rev.compl                      gtgccagcaagaggccaggaacagaggcttttcttattaactaagatttctaaatgtatt   20982
                                                                   *************************************    *******************

chimp.LOC112206744.LOC107973052.GGT2-.646279-708040.rev.compl      atgtattcacatttagaaactctaaatgtcataaaaggttagcaaggaagtttcccttcc   22350
FAM230B-LOC105372935-GGT2.NCBI.GGT2.rev.compl                      acgtattcacatttagaaactctaaatatcataaaaggttagcaaggaagtttcccttcc   21042
                                                                   * ************************* ********************************

chimp.LOC112206744.LOC107973052.GGT2-.646279-708040.rev.compl      actctgaacttccaaacaccaagtcaacatttttgtttgcatatcatccctgcaatctat   22410
FAM230B-LOC105372935-GGT2.NCBI.GGT2.rev.compl                      actctgaacttccaaacaccaagtcaacatttttgtttgcatatcatccctgcaatctat   21102
                                                                   ************************************************************

chimp.LOC112206744.LOC107973052.GGT2-.646279-708040.rev.compl      gtgcaaatagaagcatgcacctggaatgcaggctgatgtgtgatcgtgtttacacaaagt   22470
FAM230B-LOC105372935-GGT2.NCBI.GGT2.rev.compl                      gtgcaaatagaagcatgcacctggaatgcaggctgatgtgtgatcatgtttacacaaagt   21162
                                                                   ********************************************* **************

chimp.LOC112206744.LOC107973052.GGT2-.646279-708040.rev.compl      cctctgcacctctgcatatatcactgggcaatgcaccttagttatcattccacatttcaa   22530
```

```
FAM230B-LOC105372935-GGT2.NCBI.GGT2.rev.compl                   cctctgcacctctgcatatatcactgggcaatgcaccttagttatcattccacatttcaa   21222
                                                                ************************************************************

chimp.LOC112206744.LOC107973052.GGT2-.646279-708040.rev.compl   atgtaaatccattgtattgtttcagagctatgaagtactgcaccccatgactattcccaa   22590
FAM230B-LOC105372935-GGT2.NCBI.GGT2.rev.compl                   atgtaaatccattgtattgtttcagagctataaagtactgcaccccatgactattcccaa   21282
                                                                ******************************* ****************************

chimp.LOC112206744.LOC107973052.GGT2-.646279-708040.rev.compl   aattacttaagcaccccgctatgggtatccatttgttctgtttccagtcttgctcttata   22650
FAM230B-LOC105372935-GGT2.NCBI.GGT2.rev.compl                   aattacttaagcaccccgctatgggtatccatttgttctgtttccagtcttgctcttata   21342
                                                                ************************************************************

chimp.LOC112206744.LOC107973052.GGT2-.646279-708040.rev.compl   accaatgctgtagtgaacagcactgtgttgagaagtggtgaacgtgggcatctttgtctt   22710
FAM230B-LOC105372935-GGT2.NCBI.GGT2.rev.compl                   accaatgctgtagtgaacagcactgtgttgagaagcggtgaacgtgggcatctttgtctt   21402
                                                                *********************************** ************************

chimp.LOC112206744.LOC107973052.GGT2-.646279-708040.rev.compl   gttcccgtcctcagggggaatgctttcaactttcccccattcaggaaactgttggctgtg   22770
FAM230B-LOC105372935-GGT2.NCBI.GGT2.rev.compl                   gttcccttcctcaggcggaatgctttcaactctcccccattcaggaaaatgttggctgtg   21462
                                                                ****** ******** *************** **************** ***********

chimp.LOC112206744.LOC107973052.GGT2-.646279-708040.rev.compl   ggtttgtcatagatagcttttattaccttaaggtatgtccattctatgctgattttgatg   22830
FAM230B-LOC105372935-GGT2.NCBI.GGT2.rev.compl                   ggtttgtcatagatagcttttattaccttaaggtatgtccgttctacgctgattttgatg   21522
                                                                **************************************** ***** *************

chimp.LOC112206744.LOC107973052.GGT2-.646279-708040.rev.compl   aacggttttaatcataaagaaatgctggattttgtcaaaggctttttctgcatctattca   22890
FAM230B-LOC105372935-GGT2.NCBI.GGT2.rev.compl                   aacggttttaatcataaagaaatgctggattttgtcaaaggctttttctgcatctattca   21582
                                                                ************************************************************

chimp.LOC112206744.LOC107973052.GGT2-.646279-708040.rev.compl   gattatcatgtgatttttgtttttagttttatttatgtgatgtatcacatttattgactt   22950
FAM230B-LOC105372935-GGT2.NCBI.GGT2.rev.compl                   gattatcatgtgatttttgtttttagttttattgatgtgatgtatcacatttattgactt   21642
                                                                ********************************* **************************

chimp.LOC112206744.LOC107973052.GGT2-.646279-708040.rev.compl   gcgtatgttaaaccatccctgcatccctagtatgaaacccacttgaatcatggtggatta   23010
FAM230B-LOC105372935-GGT2.NCBI.GGT2.rev.compl                   gcgtatgttaaaccatccctgcatccctagtatgaaacccacttgaatcatggtggatta   21702
                                                                ************************************************************

chimp.LOC112206744.LOC107973052.GGT2-.646279-708040.rev.compl   tctttttgatatgctgttggattcagttagcttggttgtagcatttctttttattccatc   23070
FAM230B-LOC105372935-GGT2.NCBI.GGT2.rev.compl                   tctttttgatatgctgttggattcagttagcttggttgtagcatttcttattattccatc   21762
                                                                ************************************************* **********

chimp.LOC112206744.LOC107973052.GGT2-.646279-708040.rev.compl   tgtggaatgtattggtttaaataatgaaaacatgttctatcctcactgcttagcactttg   23130
FAM230B-LOC105372935-GGT2.NCBI.GGT2.rev.compl                   tgtggaatgtattggttgaaataatgaaaacatgttctatcctcactgcttagcactttg   21822
                                                                ***************** ******************************************

chimp.LOC112206744.LOC107973052.GGT2-.646279-708040.rev.compl   tgtttctttaatagccttcccaacagggcaacataaaagcaggagccctgctagtcaccc   23190
FAM230B-LOC105372935-GGT2.NCBI.GGT2.rev.compl                   tgtttctttaatagccttcccaacagggcaacataaaagcaggagccctgctagtctccc   21882
                                                                ******************************************************** ***

chimp.LOC112206744.LOC107973052.GGT2-.646279-708040.rev.compl   cttaacccggaatccccccttctccacagctcgcttattggacaggatagactgggagcc   23250
FAM230B-LOC105372935-GGT2.NCBI.GGT2.rev.compl                   cttaacccggaatccccccttctccacagctcgctcattggacaggatagactgggcgcc   21942
                                                                *********************************** ******************** ***

chimp.LOC112206744.LOC107973052.GGT2-.646279-708040.rev.compl   caggcctcaaggtaaggacgtgctctgtcacctagaggtgcagtgcttgggaaggccaac   23310
FAM230B-LOC105372935-GGT2.NCBI.GGT2.rev.compl                   caggcttcaaggtaaggacgtgctctgtcacctagaggtgcagtgcttgggaaggccaac   22002
                                                                ***** ******************************************************

chimp.LOC112206744.LOC107973052.GGT2-.646279-708040.rev.compl   cttggagggttgcctgccagctttacagtgacagaggtgttgggagggactgaccaccag   23370
FAM230B-LOC105372935-GGT2.NCBI.GGT2.rev.compl                   cttggagggttgcctgccagctttacagtgacagaggtgttgagagggactgaccaccag   22062
                                                                ****************************************** *****************

chimp.LOC112206744.LOC107973052.GGT2-.646279-708040.rev.compl   tgcataaggctgtgctttgttggtgacataaaggattgtttcacaggttgttggggaggg   23430
FAM230B-LOC105372935-GGT2.NCBI.GGT2.rev.compl                   tgcataaggctgtgctttgttggtgacataaaggattgtttcacagattgttggggaggg   22122
                                                                ********************************************** *************

chimp.LOC112206744.LOC107973052.GGT2-.646279-708040.rev.compl   acaatcccaaggcctcccctggccctggtgctggctctgcacaaaggcaataagagaggg   23490
FAM230B-LOC105372935-GGT2.NCBI.GGT2.rev.compl                   acaatcccaaggcctcccctggccctggtgctggctctgcacaaaggcaataagagaggg   22182
                                                                ************************************************************

chimp.LOC112206744.LOC107973052.GGT2-.646279-708040.rev.compl   atgctggtaagggctgacctgttgctgtgctggggaggaaggtgctgggctgaaattcag   23550
FAM230B-LOC105372935-GGT2.NCBI.GGT2.rev.compl                   atgctggtaagggctgacctgttgctgtgctggggaggaaggtgctgggctgaaattcag   22242
                                                                ************************************************************

chimp.LOC112206744.LOC107973052.GGT2-.646279-708040.rev.compl   gaggctgaggatgcagcagtcccataggaggtacgtgaccttcaggatacattttcttca   23610
FAM230B-LOC105372935-GGT2.NCBI.GGT2.rev.compl                   gaggctgaggatgcagcagtcccataggaggtacatgaccttcaggatacattttcttca   22302
                                                                ********************************** *************************

chimp.LOC112206744.LOC107973052.GGT2-.646279-708040.rev.compl   ttgatgatcaatggaaatgagaaatcactgactattttttctatcattggaatctactct   23670
FAM230B-LOC105372935-GGT2.NCBI.GGT2.rev.compl                   ttgatgatcaatggaaatgagaaatcactgactattttttctatcattggaatctactct   22362
                                                                ************************************************************

chimp.LOC112206744.LOC107973052.GGT2-.646279-708040.rev.compl   ctactgctcgtgctgttcctgtcttttggggaagatggaggatcaatcagtgtgcgctgc   23730
FAM230B-LOC105372935-GGT2.NCBI.GGT2.rev.compl                   ccactgctcatgctgttcctgtcttttggggaagatggaggatcaatcagtgtgcactgc   22422
                                                                * ******* ********************************************* ****

chimp.LOC112206744.LOC107973052.GGT2-.646279-708040.rev.compl   actgagtggaaggaaggagaactgcgacaaaaattaaggaaggatgagagacgggagggc   23790
FAM230B-LOC105372935-GGT2.NCBI.GGT2.rev.compl                   actgagtggaaggaaggagaactgtgacaaaaattaaggaaggatgagagacgggagggc   22482
                                                                ************************ ***********************************

chimp.LOC112206744.LOC107973052.GGT2-.646279-708040.rev.compl   ccttcatccagctgcttgcagagtcctcctgaggaggaaagccccgtggctccctggcga   23850
FAM230B-LOC105372935-GGT2.NCBI.GGT2.rev.compl                   ccttcatccagctgcttgcagagtcctcctgaggaggaaagccccgtggctccctggaga   22542
                                                                ********************************************************* **

chimp.LOC112206744.LOC107973052.GGT2-.646279-708040.rev.compl   aggagcagtgagggctgcgtgactcccacagtgaagtgtgtggtatgtctgaggacaccc   23910
FAM230B-LOC105372935-GGT2.NCBI.GGT2.rev.compl                   aggagcattgagggctgcgtgactcccacagtgaagtgtgtggtatgtctgaggacaccc   22602
                                                                ******* ****************************************************

chimp.LOC112206744.LOC107973052.GGT2-.646279-708040.rev.compl   aggctggtggtccatgaggagccagtggcagagtgagaagcagaaaggccaggagggtgg   23970
FAM230B-LOC105372935-GGT2.NCBI.GGT2.rev.compl                   aggctggtggtccatgaggagccagtggcagagtgagaagaagaaaggccaggagggtgg   22662
                                                                **************************************** *******************

chimp.LOC112206744.LOC107973052.GGT2-.646279-708040.rev.compl   ctggaggccaggctctgagtcattctccatgtgatggaaacagccggagcccagtgggct   24030
FAM230B-LOC105372935-GGT2.NCBI.GGT2.rev.compl                   ctggaggccaggctctgagtcattctccatgtgatggaaacagccggagcccagtgggct   22722
                                                                ************************************************************

chimp.LOC112206744.LOC107973052.GGT2-.646279-708040.rev.compl   tggaggtacaggatgcggtggctgatgacagaacaatgtggagagaggcgtcatttgtca   24090
FAM230B-LOC105372935-GGT2.NCBI.GGT2.rev.compl                   tggaggtacaggatgcggtggctgatgacagaacaatgtggagagaggcgtcatttgtca   22782
                                                                ************************************************************

chimp.LOC112206744.LOC107973052.GGT2-.646279-708040.rev.compl   aatccttactttgttctgggcattgtgctaaaaattctgatggctcatcccatttagggg   24150
FAM230B-LOC105372935-GGT2.NCBI.GGT2.rev.compl                   aatccttactttgttctgggcattgtgctaaaaattctgatggctcatcccatttagggg   22842
                                                                ************************************************************

chimp.LOC112206744.LOC107973052.GGT2-.646279-708040.rev.compl   ctgaaagttgcagaggtttaggaagctcacccatgatactggagcccccatctcctgccc   24210
FAM230B-LOC105372935-GGT2.NCBI.GGT2.rev.compl                   ctgaaagttgcagaggtttaggaagctcacccacgatactggagcccccatctcctgccc   22902
                                                                ********************************* **************************

chimp.LOC112206744.LOC107973052.GGT2-.646279-708040.rev.compl   tagtgccgtccaccttctcacccagccaccacctgtttcaggggaacacacagaagtggt   24270
FAM230B-LOC105372935-GGT2.NCBI.GGT2.rev.compl                   tagtgctgtccaccttctcacccagccaccacctgtttcggggaacacacagaagtggt   22962
                                                                ****** ******************************** ********************

chimp.LOC112206744.LOC107973052.GGT2-.646279-708040.rev.compl   aacctcttatggagaggcaagtaaattctgctgtttttgttattcacagaaaaacactgg   24330
FAM230B-LOC105372935-GGT2.NCBI.GGT2.rev.compl                   aacctcttatggagaggcaagtaaattctgctgtttttgttattcacagaaaaacactgg   23022
                                                                ************************************************************

chimp.LOC112206744.LOC107973052.GGT2-.646279-708040.rev.compl   ctcatgtgggttgggaaggtgaaataccagaagtatttcatctagttatttctacccatg   24390
FAM230B-LOC105372935-GGT2.NCBI.GGT2.rev.compl                   ctcgtgtgggttgggaaggtgaaataccagaagtatttcatctggttatttctacccatg   23082
                                                                *** *************************************** ****************

chimp.LOC112206744.LOC107973052.GGT2-.646279-708040.rev.compl   caactcctatagtattgaaatgcataggttagcatttttggccaatttactcagcattct   24450
FAM230B-LOC105372935-GGT2.NCBI.GGT2.rev.compl                   cgactcctatagtattgaaatgcataggttagcatttttggccaatttactcagcattct   23142
                                                                * **********************************************************

chimp.LOC112206744.LOC107973052.GGT2-.646279-708040.rev.compl   gggttaaaggct-------------tttatttatttatttatttattttcgagatggagt   24497
FAM230B-LOC105372935-GGT2.NCBI.GGT2.rev.compl                   gggttaaaggcttttatttatttatttatttatttatttatttattttttgagatggagt   23202
                                                                ************             ************************ **********

chimp.LOC112206744.LOC107973052.GGT2-.646279-708040.rev.compl   ttcgctcttgttgcccaagctggagtgcaatggtgcgatcctggttcactgcaacctccg   24557
FAM230B-LOC105372935-GGT2.NCBI.GGT2.rev.compl                   ttcactcttgttgcccaagctggagtgcaatggtgcgatcctggttcattgcaacctccg   23262
                                                                *** ******************************************** ***********

chimp.LOC112206744.LOC107973052.GGT2-.646279-708040.rev.compl   cctcccaggttcaaactattctcctgtctcagcctcccaagtagctgagattataggcac   24617
FAM230B-LOC105372935-GGT2.NCBI.GGT2.rev.compl                   cctcccaggttcaaactattctcctgtctcagcctcccaagtagctgagattataggcac   23322
                                                                ************************************************************

chimp.LOC112206744.LOC107973052.GGT2-.646279-708040.rev.compl   gtgccaccacactgggctaatttttttgtatttttagtagaaatgagatttcaccatgtt   24677
FAM230B-LOC105372935-GGT2.NCBI.GGT2.rev.compl                   atgccaccacactgggctaatttttttgtatttttagtagaaatgagatttcaccatgtt   23382
```

```
                                                                    ***********************************************************

chimp.LOC112206744.LOC107973052.GGT2-.646279-708040.rev.compl      ggtcaggctggtttcaaaatcctgacctcaggtgatctgccctcctcggcctcttaaagt   24737
FAM230B-LOC105372935-GGT2.NCBI.GGT2.rev.compl                      ggtcaggctggtttcgaactcctgacctcaggtgatctgcccccctcggcctcttaaagt   23442
                                                                   *************** ** ************************ ****************

chimp.LOC112206744.LOC107973052.GGT2-.646279-708040.rev.compl      gctgggattacaggcgtgagccaccatgcccaccctaaagtcttttaaaattcacttgta   24797
FAM230B-LOC105372935-GGT2.NCBI.GGT2.rev.compl                      gctgggattacaggcgtgagccaccacgcctgccctaaagtcttttaaaattcacttgta   23502
                                                                   ************************** ***  ****************************

chimp.LOC112206744.LOC107973052.GGT2-.646279-708040.rev.compl      taagttgacttagtttcttttttttttcttttcttttcttttttttttttttattgat   24857
FAM230B-LOC105372935-GGT2.NCBI.GGT2.rev.compl                      taagtt------------------------------------------------------   23508
                                                                   ******

chimp.LOC112206744.LOC107973052.GGT2-.646279-708040.rev.compl      cattcttggacttagtttctttaaccttgtagaaaaat-caaaaatggcaatctgtttt   24916
FAM230B-LOC105372935-GGT2.NCBI.GGT2.rev.compl                      --------gacttagtttctttaaccttgtagaaaaatacaaaaatggcaatctctttt   23560
                                                                           ****************************** *************** ****

chimp.LOC112206744.LOC107973052.GGT2-.646279-708040.rev.compl      atcacacaaataatgtctttttaatggagtgatttttttctaattgaggtattatgtact   24976
FAM230B-LOC105372935-GGT2.NCBI.GGT2.rev.compl                      atcacacaaataatgtctttttaatggagtgatttttttctaattgaggtattatgtact   23620
                                                                   ************************************************************

chimp.LOC112206744.LOC107973052.GGT2-.646279-708040.rev.compl      tttcatttactaattattgtttacatttgaagtgttttatgaattaatatttaattgcat   25036
FAM230B-LOC105372935-GGT2.NCBI.GGT2.rev.compl                      tttcatttactaattattgtttacatttgaagtgttttatgaattaatatttaattgcat   23680
                                                                   ************************************************************

chimp.LOC112206744.LOC107973052.GGT2-.646279-708040.rev.compl      agatgaagattactagttataggcatttagtaaccaatactcattaagcatagcgtgga   25096
FAM230B-LOC105372935-GGT2.NCBI.GGT2.rev.compl                      agatgaagattactagttataggcatttactaaccaatactcattaagcatagcgtgga   23740
                                                                   ***************************** ******************************

chimp.LOC112206744.LOC107973052.GGT2-.646279-708040.rev.compl      ttcatatgacatcaaggagctattttatttggtaaaacgaaaaagcacaagaatgaacga   25156
FAM230B-LOC105372935-GGT2.NCBI.GGT2.rev.compl                      ttcctatgacatcaaggagctattttatttggtaaaacgaaaaagcacaagaatgaacga   23800
                                                                   *** ********************************************************

chimp.LOC112206744.LOC107973052.GGT2-.646279-708040.rev.compl      acgcaagaactgaaacggtggagacacctagaatgacttgtctaagatctaaatcatttt   25216
FAM230B-LOC105372935-GGT2.NCBI.GGT2.rev.compl                      acgcaagaactgaaacagtggagacacctagaatgacttgtctaagatctaaatcatttt   23860
                                                                   *************** ********************************************

chimp.LOC112206744.LOC107973052.GGT2-.646279-708040.rev.compl      gttgtcttcccagcgtacttattatcctgatcattgtcatcagcattgtttgggtccttt   25276
FAM230B-LOC105372935-GGT2.NCBI.GGT2.rev.compl                      gttgtcttcccagcgtacttattatcctgatcattgtcatcagcattgtttgggtccttt   23920
                                                                   ************************************************************

chimp.LOC112206744.LOC107973052.GGT2-.646279-708040.rev.compl      tagcacagatttctcaaaatgggtaactccataacagttggaagcttacgaattcatata   25336
FAM230B-LOC105372935-GGT2.NCBI.GGT2.rev.compl                      tagcacagatttctcaaaatgggtaactccataacagttggaagcttacgaattcatata   23980
                                                                   ************************************************************

chimp.LOC112206744.LOC107973052.GGT2-.646279-708040.rev.compl      atttgtaagaggtcaatttggaagtacctatctattttaaaattccaataacctgggaat   25396
FAM230B-LOC105372935-GGT2.NCBI.GGT2.rev.compl                      atttgtaagaggtcaatttgggagtacctatctattttaaaattccaataacctgggaat   24040
                                                                   ********************* **************************************

chimp.LOC112206744.LOC107973052.GGT2-.646279-708040.rev.compl      ttcatcccatgtctagagtcttccatgtaaaatatttccacaattaggagaaatatgtgc   25456
FAM230B-LOC105372935-GGT2.NCBI.GGT2.rev.compl                      ttcatcccatgtctagagtcttttatgtaaaatatttccacaattaggagaaatatgtgc   24100
                                                                   **********************  ************************************

chimp.LOC112206744.LOC107973052.GGT2-.646279-708040.rev.compl      atggggattttctatgtagcggtgttttgatagaatagaaaaatgggataaaccaaattt   25516
FAM230B-LOC105372935-GGT2.NCBI.GGT2.rev.compl                      atggggattttctatgtagcggtgttttgatagaatagaaaattgggataaaccaaattt   24160
                                                                   ***************************************** ******************

chimp.LOC112206744.LOC107973052.GGT2-.646279-708040.rev.compl      ccatcacgaaggaaatagtaatatgctgaataataatacagcgaatattatgcaggcttt   25576
FAM230B-LOC105372935-GGT2.NCBI.GGT2.rev.compl                      ccatcacgaaggaaatagtaatatgctgaataataatacagcgaatattatgcaggcttt   24220
                                                                   ************************************************************

chimp.LOC112206744.LOC107973052.GGT2-.646279-708040.rev.compl      aaacatcaaaaaagagttcaacttctgacttccgatgatggtgttgaagcaggtcactgc   25636
FAM230B-LOC105372935-GGT2.NCBI.GGT2.rev.compl                      aaacatcaaaaaagagttcaacttctgacttccgatgatggtgttgaagcaggtcactgc   24280
                                                                   ************************************************************

chimp.LOC112206744.LOC107973052.GGT2-.646279-708040.rev.compl      tggtttacatttgattttcatgtgggaactctggaagtctgccttagtgattttacatgt   25696
FAM230B-LOC105372935-GGT2.NCBI.GGT2.rev.compl                      tggtttacatttgattttcatgtgggaactctggaagtccgccttagtgattttacatgt   24340
                                                                   *************************************** ********************

chimp.LOC112206744.LOC107973052.GGT2-.646279-708040.rev.compl      ggctaaattgagctaatgacaagctgttcgaagtatggcaaaatggaactttaaaacagt   25756
FAM230B-LOC105372935-GGT2.NCBI.GGT2.rev.compl                      ggctaaattgagctaatgacaagctgtttgcagtatggcaaaatggaactttaaaacagt   24400
                                                                   **************************** * *****************************

chimp.LOC112206744.LOC107973052.GGT2-.646279-708040.rev.compl      atcttgtcagcaaccaagtggacctgtttcacgtaaagcccatgcattcatctgcctgcc   25816
FAM230B-LOC105372935-GGT2.NCBI.GGT2.rev.compl                      atcttgtcaacaaccaagaggacctgtttcacataaagcccacgcattcatctgc-----   24455
                                                                   ********* ******** ************* ********* ************

chimp.LOC112206744.LOC107973052.GGT2-.646279-708040.rev.compl      catcattctgtctgtccatcattctgtctgtccacacgggcatcatttgttagtggaact   25876
FAM230B-LOC105372935-GGT2.NCBI.GGT2.rev.compl                      -----------ctgtccatcattctgtctgtccacacgggcatcattcgttagtggaact   24504
                                                                              ************************************ ************

chimp.LOC112206744.LOC107973052.GGT2-.646279-708040.rev.compl      gagtgcccactgtcgagctgacaagcccataacctccctgttcctagtcacacattaatt   25936
FAM230B-LOC105372935-GGT2.NCBI.GGT2.rev.compl                      gagtgcccgctgtcgagctgacaagcccataacctccctgttcctagtcacacattaatt   24564
                                                                   ******** ***************************************************

chimp.LOC112206744.LOC107973052.GGT2-.646279-708040.rev.compl      cttcaacaagttccttttgatagattgtgattaagcttagctgctatttccaattgcttc   25996
FAM230B-LOC105372935-GGT2.NCBI.GGT2.rev.compl                      cttcaacaagtcccttttgatagattgtgattaagcttagctactatttccaattgcttc   24624
                                                                   *********** ****************************** *****************

chimp.LOC112206744.LOC107973052.GGT2-.646279-708040.rev.compl      cccaaacgtacttctcactgttctcccatcacacccttcagcccatccatgcggggttcc   26056
FAM230B-LOC105372935-GGT2.NCBI.GGT2.rev.compl                      cccaaacgtacttctcactgttctcccatcacacccttcagcccatccatgcggggttcc   24684
                                                                   ************************************************************

chimp.LOC112206744.LOC107973052.GGT2-.646279-708040.rev.compl      tttgcttttcccaccttacaccaaactccctatttttactcccactttacctcctctcc   26116
FAM230B-LOC105372935-GGT2.NCBI.GGT2.rev.compl                      tttgcttttcccaccttacaccaaactccctatttttactcccactttacctcctctcc   24744
                                                                   ************************************************************

chimp.LOC112206744.LOC107973052.GGT2-.646279-708040.rev.compl      aagacaaaacaaacaaaactagcattttaaaacttagttgtaatctttcttccttcatga   26176
FAM230B-LOC105372935-GGT2.NCBI.GGT2.rev.compl                      aagacacaacaaacaaaactagcattttaaaacttagttgtaatctttcttccttcatga   24804
                                                                   ****** *****************************************************

chimp.LOC112206744.LOC107973052.GGT2-.646279-708040.rev.compl      aaatttctccaacagccactcccacggtcctgtgtgttccggatattttaaaataatggc   26236
FAM230B-LOC105372935-GGT2.NCBI.GGT2.rev.compl                      aaatttctccaacagccactcccacggtcctgtgtgttccggatattttaaaataatggc   24864
                                                                   ************************************************************

chimp.LOC112206744.LOC107973052.GGT2-.646279-708040.rev.compl      tataaggttgagcacttcaggatatgctgttttgctgtgtgcagatggaggcagtggctg   26296
FAM230B-LOC105372935-GGT2.NCBI.GGT2.rev.compl                      tataaggttgagcacttcaggatacgctgttttgctgtgtgcagatggaggcagtggctg   24924
                                                                   ************************ ***********************************

chimp.LOC112206744.LOC107973052.GGT2-.646279-708040.rev.compl      gagtgaatgaacggcaacacttgctggcaaccggcagaagctgagagacagggaacaggc   26356
FAM230B-LOC105372935-GGT2.NCBI.GGT2.rev.compl                      gagtgaatgaacggcaacacttgctggcaaccggcagaagctgagagacagggaacaggc   24984
                                                                   ************************************************************

chimp.LOC112206744.LOC107973052.GGT2-.646279-708040.rev.compl      tctcctccagagcctccaggagccaggcctttggacaccttgaatgtgggcttctgggag   26416
FAM230B-LOC105372935-GGT2.NCBI.GGT2.rev.compl                      tctcctccagagcctccaggagccaggcctttggacaacttgaatgtgggcttctgggag   25044
                                                                   ************************************* **********************

chimp.LOC112206744.LOC107973052.GGT2-.646279-708040.rev.compl      accatgcgtttctgttataagcagcccagtcgctggcagtttttacggctgcccccgaac   26476
FAM230B-LOC105372935-GGT2.NCBI.GGT2.rev.compl                      accatgcgtttctgttataagcagcccagtctctggcagtttttacggctgccccggaac   25104
                                                                   ******************************* *********************** ****

chimp.LOC112206744.LOC107973052.GGT2-.646279-708040.rev.compl      actcatctatacctgtctgacaaggtcaagctccaaggaagggattctctacatatctac   26536
FAM230B-LOC105372935-GGT2.NCBI.GGT2.rev.compl                      actcatctatacctgtctgacaaggtcaagctccaaggaagggactctctacatatctac   25164
                                                                   ******************************************** ***************

chimp.LOC112206744.LOC107973052.GGT2-.646279-708040.rev.compl      attgtttgcagattttacaataatcatttattcttgcatggctgatcattgttaaccaat   26596
FAM230B-LOC105372935-GGT2.NCBI.GGT2.rev.compl                      attgtttgcagattttacaataatcatttattcttgcatggctgatcattgttaaccaat   25224
                                                                   ************************************************************

chimp.LOC112206744.LOC107973052.GGT2-.646279-708040.rev.compl      acaaataaaataataaagaaatgacccacattttatgttgggagtttgatctgccattta   26656
FAM230B-LOC105372935-GGT2.NCBI.GGT2.rev.compl                      acaaataaaataataaagaaatgacccacattttatgttgggagtttgatctgccattta   25284
                                                                   ************************************************************

chimp.LOC112206744.LOC107973052.GGT2-.646279-708040.rev.compl      tcaagtatggaatcttgaacaaggggttaaacatctgaatgtctccatcacttcatctct   26716
FAM230B-LOC105372935-GGT2.NCBI.GGT2.rev.compl                      tcaagtatggaatcttgaacaaggggttaaacatctgaatgtctccatcacttcatctct   25344
                                                                   ************************************************************

chimp.LOC112206744.LOC107973052.GGT2-.646279-708040.rev.compl      aaagtgggggtgctcacacccactgggctcccccct-ccaggttggtgccggactctccct   26775
FAM230B-LOC105372935-GGT2.NCBI.GGT2.rev.compl                      aaagtgggggtgctcacacccactgggctcccccgcccaggttggtgccggactctccct   25404
                                                                   ***********************************  ***********************

chimp.LOC112206744.LOC107973052.GGT2-.646279-708040.rev.compl      gggccccccctgttctctcaccagccacatccattctccccccagaggcgctagtgactgt   26835
FAM230B-LOC105372935-GGT2.NCBI.GGT2.rev.compl                      gggccccccctgttctctcaccagccacatccattctccccccagaggcgctagtgactgt   25464
                                                                   ************************************************************
```

```
chimp.LOC112206744.LOC107973052.GGT2-.646279-708040.rev.compl      gcgtggctttccattcccaccacgtttgtctctaaccccagtggcagatcagtgtaagaa   26895
FAM230B-LOC105372935-GGT2.NCBI.GGT2.rev.compl                      gcgtggctttccattcccaccacgtttgtctctaaccccagtggcagatcagtgtaagaa   25524
                                                                   ************************************************************

chimp.LOC112206744.LOC107973052.GGT2-.646279-708040.rev.compl      cacagctgagtgctcctcgcctccttgccccttcaagggctcctcaccacccaccagatc   26955
FAM230B-LOC105372935-GGT2.NCBI.GGT2.rev.compl                      cacagctgagtgctcctcgcctccttgccccttcaagggcccctcaccacccaccagatc   25584
                                                                   **************************************** *******************

chimp.LOC112206744.LOC107973052.GGT2-.646279-708040.rev.compl      aggtgcaaacttccaagccttactggatccccttccacattctgagctccgcctgccttc   27015
FAM230B-LOC105372935-GGT2.NCBI.GGT2.rev.compl                      aggtgcaaacttccaagccttactggatccccttccacattctgagctccgcctgccttc   25644
                                                                   ************************************************************

chimp.LOC112206744.LOC107973052.GGT2-.646279-708040.rev.compl      ccatcgctatccttccccacctgcctccctggtagagaaaagcagagtgtgtgatgctgt   27075
FAM230B-LOC105372935-GGT2.NCBI.GGT2.rev.compl                      ccatcgctatccttccccacctgcctccctggtagagaaaagcggagtgtgtgatgctgt   25704
                                                                   ****************************************** *****************

chimp.LOC112206744.LOC107973052.GGT2-.646279-708040.rev.compl      ctgaatgctgagcacggccttttgcagccagtccactgtgtacgctgcccctatcggaga   27135
FAM230B-LOC105372935-GGT2.NCBI.GGT2.rev.compl                      ctgaatgctgagcacggccttttgcagccagtccactgtgtacgctgcccctatcggaga   25764
                                                                   ************************************************************

chimp.LOC112206744.LOC107973052.GGT2-.646279-708040.rev.compl      cctccaccttaaccctttccagcctggaggctcctcccagggccccacaacagaagtgac   27195
FAM230B-LOC105372935-GGT2.NCBI.GGT2.rev.compl                      cctccaccttaaccctttccagcctggaggctcctcccagggccccacaacagaagtgac   25824
                                                                   ************************************************************

chimp.LOC112206744.LOC107973052.GGT2-.646279-708040.rev.compl      ttcccttgcctttgaatttctatagcacaatccctactgcccccgttaaaactgcaaag   27255
FAM230B-LOC105372935-GGT2.NCBI.GGT2.rev.compl                      ttcccttgcctttgaatttctatagcacaatccctactgcccccgttaaaactgcaaag   25884
                                                                   ************************************************************

chimp.LOC112206744.LOC107973052.GGT2-.646279-708040.rev.compl      tccttttgtggaaaataactttattcatgactgtgtttatcacactatcttatggagaag   27315
FAM230B-LOC105372935-GGT2.NCBI.GGT2.rev.compl                      tccttttgtggaaaataactttattcatgactgtgtttatcacactatcttatggagaag   25944
                                                                   ************************************************************

chimp.LOC112206744.LOC107973052.GGT2-.646279-708040.rev.compl      agatgatcaataaatatttgcggaataaatgaatagcagttacaaaacacttgattcata   27375
FAM230B-LOC105372935-GGT2.NCBI.GGT2.rev.compl                      agatgatcaataaatatttgctgaataaatgaatagcagttacaaaacacttgattcata   26004
                                                                   ********************* **************************************

chimp.LOC112206744.LOC107973052.GGT2-.646279-708040.rev.compl      tggaattaatgttggttctcaaagtgaaaaattacaaacagcactgatattcagccagta   27435
FAM230B-LOC105372935-GGT2.NCBI.GGT2.rev.compl                      tggaattaatgttggttctcaaagtgaaaaattacaaacagcactgatattcagccagta   26064
                                                                   ************************************************************

chimp.LOC112206744.LOC107973052.GGT2-.646279-708040.rev.compl      tacaagtctggtcacagcagttgtataatactgaaatacccccctgccactgacctttggc   27495
FAM230B-LOC105372935-GGT2.NCBI.GGT2.rev.compl                      tacaagtctggtcacagcagttgtataatactgaaatacccccctgccactgacctttggc   26124
                                                                   ************************************************************

chimp.LOC112206744.LOC107973052.GGT2-.646279-708040.rev.compl      ccccagatgcctcccactgccactgctctccccactgggaacccctgaagttcccacagg   27555
FAM230B-LOC105372935-GGT2.NCBI.GGT2.rev.compl                      ccccagatgcctcccactgccactgctctccccactgggaacccctgaagttcccacagg   26184
                                                                   ************************************************************

chimp.LOC112206744.LOC107973052.GGT2-.646279-708040.rev.compl      ctcataactaaagggctaatgtcctgcacagcagcgagcacccaggaccgagcagccaca   27615
FAM230B-LOC105372935-GGT2.NCBI.GGT2.rev.compl                      ctcataactaaagggctaatgtcttgcacagcagcgagcacccaggaccgagcagccaca   26244
                                                                   ************************ ***********************************

chimp.LOC112206744.LOC107973052.GGT2-.646279-708040.rev.compl      tggccgggtctgctggtgaaagcatccattctgactgatcaggacctgaggggcctcatg   27675
FAM230B-LOC105372935-GGT2.NCBI.GGT2.rev.compl                      tggccgggtctgctggtgaaagcatccattctgactgatcaggacctgaggggcctcatg   26304
                                                                   ************************************************************

chimp.LOC112206744.LOC107973052.GGT2-.646279-708040.rev.compl      gttacatattttgataatatccctaattataaataaggctcagttatatagtttgaaaac   27735
FAM230B-LOC105372935-GGT2.NCBI.GGT2.rev.compl                      gttacatattttgataatatccctaattataaataaggctcagttatatagtttgaaaac   26364
                                                                   ************************************************************

chimp.LOC112206744.LOC107973052.GGT2-.646279-708040.rev.compl      aatgcttctcctcattgcaaaatctcttagaagactccgtagatccaggaacggaaatgg   27795
FAM230B-LOC105372935-GGT2.NCBI.GGT2.rev.compl                      agtgcttctcctcattgcaaaatctcttagaagactccgtagatccaggaacggaaatgg   26424
                                                                   * **********************************************************

chimp.LOC112206744.LOC107973052.GGT2-.646279-708040.rev.compl      aaaatgacagcatgtcaatctctgaaggttttgggcatttccattagcactccatcttca   27855
FAM230B-LOC105372935-GGT2.NCBI.GGT2.rev.compl                      aaaatgacagcgtgtcaatctctgaaggttttgggcatttccattagcactccatcttca   26484
                                                                   *********** ************************************************

chimp.LOC112206744.LOC107973052.GGT2-.646279-708040.rev.compl      tgtaaaccagaagatatgcagtttcctgcctagagagaagagaagacacatcagcacagc   27915
FAM230B-LOC105372935-GGT2.NCBI.GGT2.rev.compl                      tgtaaaccagaagatatgcagtttcctgcctagagagaagagaagacacatcagcacagc   26544
                                                                   ************************************************************

chimp.LOC112206744.LOC107973052.GGT2-.646279-708040.rev.compl      ggcatgaaaccttcatcagaaaacaatgcttcattaatccttgacaggacaagcgtcagc   27975
FAM230B-LOC105372935-GGT2.NCBI.GGT2.rev.compl                      ggcatgaaaccttcatcagaaaacaatgcttcattaatccgtgacaggacaagcgtcagc   26604
                                                                   **************************************** *******************

chimp.LOC112206744.LOC107973052.GGT2-.646279-708040.rev.compl      aaacttccaggccgctggattaagccttcatctatccatcaccttggagaggaacaaaat   28035
FAM230B-LOC105372935-GGT2.NCBI.GGT2.rev.compl                      aaacttccaggcggctggattaggccttcatctatccatcaccttggagaggaacaaaat   26664
                                                                   ************ ********** ************************************

chimp.LOC112206744.LOC107973052.GGT2-.646279-708040.rev.compl      aggtggcctgggaagataagcactatgtttctattagttaatatctaaagcggaggttaa   28095
FAM230B-LOC105372935-GGT2.NCBI.GGT2.rev.compl                      aggtggcctgggaagataagcactatgtttctattagttaatatctaaagcggaggttaa   26724
                                                                   ************************************************************

chimp.LOC112206744.LOC107973052.GGT2-.646279-708040.rev.compl      caagctatggacacacaagccaaacccagccctcttggggtttttaaatctactttcaa   28155
FAM230B-LOC105372935-GGT2.NCBI.GGT2.rev.compl                      caagctatggacacacaagccaaacccagccctcttggggtttttaaatctactttcaa   26784
                                                                   ************************************************************

chimp.LOC112206744.LOC107973052.GGT2-.646279-708040.rev.compl      cttttatttagattcagcgggcacatgtgcaggtttgtcacgtggatatgagcatactc   28215
FAM230B-LOC105372935-GGT2.NCBI.GGT2.rev.compl                      cttttatttagattcagcgggcacatgtgcaggtttgtcacatggatatgagcatactc   26844
                                                                   ***************************************** ******************

chimp.LOC112206744.LOC107973052.GGT2-.646279-708040.rev.compl      cccaacagttggcctttcacccctcccctccctccccatccagcagttcccagttgttgc   28275
FAM230B-LOC105372935-GGT2.NCBI.GGT2.rev.compl                      cccaacagttggcctttcacccctcccctccctccccatccagcagttcccagttgttgc   26904
                                                                   ************************************************************

chimp.LOC112206744.LOC107973052.GGT2-.646279-708040.rev.compl      catctttaagtcaatgactgcccatgtttagctcccatttataagagagaatatgcatta   28335
FAM230B-LOC105372935-GGT2.NCBI.GGT2.rev.compl                      catctttaagtcaatgagtccccatgtttagctcccatttataagagagaacatgcatta   26964
                                                                   ***************** * ******************************* ********

chimp.LOC112206744.LOC107973052.GGT2-.646279-708040.rev.compl      tgttttgtttggttttgctgg-----ttttttttttttaatggagtcttgccctgtagc   28390
FAM230B-LOC105372935-GGT2.NCBI.GGT2.rev.compl                      tgttttgtttggttttgctggttttttttttttttttttaatggagtcttgccctgtagc   27024
                                                                   *********************     **********************************

chimp.LOC112206744.LOC107973052.GGT2-.646279-708040.rev.compl      ccaggctagagtgcagtggcacaatcttggctcactgcaacctccgcctcccaggttcaa   28450
FAM230B-LOC105372935-GGT2.NCBI.GGT2.rev.compl                      ccaggctagagtgcagtggcacaaccttggctcactgcaacctccgcctcccaggttcaa   27084
                                                                   ************************ ***********************************

chimp.LOC112206744.LOC107973052.GGT2-.646279-708040.rev.compl      acaattctccctcctcagcctcccgagtagctgggactacaggtgcctgccaccacgccc   28510
FAM230B-LOC105372935-GGT2.NCBI.GGT2.rev.compl                      acgattctccctcctcagcctcccgagtggctgggactacaggcgcccaccaccacgccc   27144
                                                                   ** ************************* ************** ***  ***********

chimp.LOC112206744.LOC107973052.GGT2-.646279-708040.rev.compl      agctaacttttgtatttttagtagagacagggtttcactgtgttagccaggatggtctc   28570
FAM230B-LOC105372935-GGT2.NCBI.GGT2.rev.compl                      ggctaacttttgtatttttagtagagacagggtttcaccgtgttagccaggatggtctc   27204
                                                                    ************************************** ********************

chimp.LOC112206744.LOC107973052.GGT2-.646279-708040.rev.compl      aatctcctgacctcatgatctgcccacctcagtctcccaaagtgcagggattacaggcat   28630
FAM230B-LOC105372935-GGT2.NCBI.GGT2.rev.compl                      aatctcctgacctcatgatctgcccaccttagtctcccaaagtgcagggattacaggcgt   27264
                                                                   ***************************** **************************** *

chimp.LOC112206744.LOC107973052.GGT2-.646279-708040.rev.compl      gagccaccgtgcccagtcttttgtttatttttgacgagaccgttcttgctctgtcacca   28690
FAM230B-LOC105372935-GGT2.NCBI.GGT2.rev.compl                      gagccaccgtgcccagccttttgtttatttttgatgagacggttcttgctctgtcacca   27324
                                                                   **************** ***************** ***** *******************

chimp.LOC112206744.LOC107973052.GGT2-.646279-708040.rev.compl      ggctggagtgcactggcacagtaatagctcaccacagccttgtgctcctgggctcaactg   28750
FAM230B-LOC105372935-GGT2.NCBI.GGT2.rev.compl                      ggctggagtgcactggcacaataatagctcaccacagcctcgtgctcctgggctcaactg   27384
                                                                   ******************** ******************* *******************

chimp.LOC112206744.LOC107973052.GGT2-.646279-708040.rev.compl      accctcctgcctcagttttagcttcctgagtagctaggactacgggtgtgtaccaccatg   28810
FAM230B-LOC105372935-GGT2.NCBI.GGT2.rev.compl                      accctcctgcctcagttttagcttcctgagtagctaggactacgggtgtgtaccaccatg   27444
                                                                   ************************************************************

chimp.LOC112206744.LOC107973052.GGT2-.646279-708040.rev.compl      cctagctataataattttatttttttgtagagatggagtcttgctttgttgcccaggct   28870
FAM230B-LOC105372935-GGT2.NCBI.GGT2.rev.compl                      cctagctataataattttatttttttgtagagatggagtcttgctttgttgcccaggct   27504
                                                                   ************************************************************

chimp.LOC112206744.LOC107973052.GGT2-.646279-708040.rev.compl      ggtcttgaactcctggcttaaagtgatcctcctgcctcggcctcccaaagtgctgggatt   28930
FAM230B-LOC105372935-GGT2.NCBI.GGT2.rev.compl                      ggtcttgaactcctggcttgaagtgatcctcctgcctcggcctcccaaagtgccgggatt   27564
                                                                   ******************* ********************************* ******

chimp.LOC112206744.LOC107973052.GGT2-.646279-708040.rev.compl      aaaggtgtgagatcacacccagtctccaaccctcttttgcaagtaaatgtaactggacc   28990
FAM230B-LOC105372935-GGT2.NCBI.GGT2.rev.compl                      aaaggtgggagatcgcacccagtctccaaccctcttttgcaagtaaatgtaactggacc   27624
                                                                   ******* ****** *********************************************
```

```
chimp.LOC112206744.LOC107973052.GGT2-.646279-708040.rev.compl      ccagccatgctcatctgcccatgtactgtctacggctgcttttgctctatagggcagagt   29050
FAM230B-LOC105372935-GGT2.NCBI.GGT2.rev.compl                      ccagccatgctcatctgcccatgtactgtctacggctgcttttgctctacagggcagagt   27684
                                                                   ************************************************* **********

chimp.LOC112206744.LOC107973052.GGT2-.646279-708040.rev.compl      taagtggttgcaacagacactgcacagaccacaaagtctggaagtactttctctccagcc   29110
FAM230B-LOC105372935-GGT2.NCBI.GGT2.rev.compl                      taagtggttgcaacagacactgcacagaccacaaagtct-gaagtactttctctccagcc   27743
                                                                   *************************************** ********************

chimp.LOC112206744.LOC107973052.GGT2-.646279-708040.rev.compl      ctttacagagaaagtctgccaacctctaatctcaataacagggaaatcaatgacaaccac   29170
FAM230B-LOC105372935-GGT2.NCBI.GGT2.rev.compl                      ctttacagagaaagtctgccaacctctaatctcaataacagggaaatcaatgacaaccac   27803
                                                                   ************************************************************

chimp.LOC112206744.LOC107973052.GGT2-.646279-708040.rev.compl      aaagtgacaaagattgggtgtctaagatggatggtcagaataaacaagagagaaagatga   29230
FAM230B-LOC105372935-GGT2.NCBI.GGT2.rev.compl                      aaagtgacaaagattgggtgtctaagatggatgttcagaataaacaagagagaaagatga   27863
                                                                   ********************************* **************************

chimp.LOC112206744.LOC107973052.GGT2-.646279-708040.rev.compl      aaagtagaaggaggatttcaagcgcaagcttcacctaatccgttatttttcaaatgacca   29290
FAM230B-LOC105372935-GGT2.NCBI.GGT2.rev.compl                      gaagtagaaggaggatttcaaacgcaagcttcacctaatccgttatttttcaaatgacca   27923
                                                                    ******************** **************************************

chimp.LOC112206744.LOC107973052.GGT2-.646279-708040.rev.compl      ggcctatctctgtagctgaaaatcacctcaaataggatctctgatatacagtctccaaaa   29350
FAM230B-LOC105372935-GGT2.NCBI.GGT2.rev.compl                      ggcctatctctgtagccgaaaatcacctcaaataggatctctgatatacagtctccaaaa   27983
                                                                   **************** *******************************************

chimp.LOC112206744.LOC107973052.GGT2-.646279-708040.rev.compl      gctcagctaagaaacttac--agtctctctgccttaacttcatccacctttttctctcc   29408
FAM230B-LOC105372935-GGT2.NCBI.GGT2.rev.compl                      gctcagctaagaaacttacaaagtctctctgccttaacttcatccacctttttctctcc   28043
                                                                   *******************  ***************************************

chimp.LOC112206744.LOC107973052.GGT2-.646279-708040.rev.compl      agcttctcctcggtagttaatgattataaaaatatttattggctcatgcctgtaatccca   29468
FAM230B-LOC105372935-GGT2.NCBI.GGT2.rev.compl                      agcttctcctcggtagttaatgattataaaaatatttattggctcatgcctgtaatccca   28103
                                                                   ************************************************************

chimp.LOC112206744.LOC107973052.GGT2-.646279-708040.rev.compl      gcactttgagaggccaaggcgggcagatcacgaggtcaggagagcgagaccatcctggct   29528
FAM230B-LOC105372935-GGT2.NCBI.GGT2.rev.compl                      gcactttgagaggccgaggcgggcagatcac--ggtcaggagatcgagaccatcctggct   28161
                                                                   *************** ***************  ********** ****************

chimp.LOC112206744.LOC107973052.GGT2-.646279-708040.rev.compl      aacacggtgaaatcccgtctctactaaaaatacaaaaaattagccaggcgtggtggcggg   29588
FAM230B-LOC105372935-GGT2.NCBI.GGT2.rev.compl                      aacacggtgaaatcccgtctctactaaaaatacaaaaaattagccaggcgtggtggcggg   28221
                                                                   ************************************************************

chimp.LOC112206744.LOC107973052.GGT2-.646279-708040.rev.compl      cacctgtaatcccagctactcgggaggctgaggcaggagaatggcgtgaacccacaaggc   29648
FAM230B-LOC105372935-GGT2.NCBI.GGT2.rev.compl                      cgcctgtaatcccagctactcaggaggctgaggcaggagaatggcgtgaacccacaaggc   28281
                                                                   * ******************* **************************************

chimp.LOC112206744.LOC107973052.GGT2-.646279-708040.rev.compl      ggagcttgcaatgaggtgagatcccactactgcactccagcctgggcgacagagcaagac   29708
FAM230B-LOC105372935-GGT2.NCBI.GGT2.rev.compl                      ggagcttgcaatgaggtgagatcccactactgcactccagcctgggcgacagagcaagac   28341
                                                                   ************************************************************

chimp.LOC112206744.LOC107973052.GGT2-.646279-708040.rev.compl      tccatctca----aaacaaacaaacaaacaaaaaaacagtgtgatggccaggcgcggtgc   29764
FAM230B-LOC105372935-GGT2.NCBI.GGT2.rev.compl                      tccatctcaaaacaaacaaacaaacaaacaaaaaaacagtgtgatggccaggcgcagtgc   28401
                                                                   *********    ****************************************** ****

chimp.LOC112206744.LOC107973052.GGT2-.646279-708040.rev.compl      tcaagcctataatccaagcactttgggaggctgaaatggatcgatggcttgagcccagta   29824
FAM230B-LOC105372935-GGT2.NCBI.GGT2.rev.compl                      tcatgcctataatccaagcactttgggaggctgaaatggatggatggcttgagcccagta   28461
                                                                   *** ************************************* ******************

chimp.LOC112206744.LOC107973052.GGT2-.646279-708040.rev.compl      gtttgagacaagcctggcaacatagcgagacctcatctctacaaacattttaaaatatg   29884
FAM230B-LOC105372935-GGT2.NCBI.GGT2.rev.compl                      gtttgagacaagcctggcaacatagcgagacctcatctctacaaacatctttaaaatatg   28521
                                                                   ************************************************ ***********

chimp.LOC112206744.LOC107973052.GGT2-.646279-708040.rev.compl      ccaggcatggtgtgcagactgcatgcctgtagtcccagctattcgggaggctgaggtggg   29944
FAM230B-LOC105372935-GGT2.NCBI.GGT2.rev.compl                      ccaggcatggtg------gtgcatgcctgtagtcccagctattcaggaggctgaggtggg   28575
                                                                   ************       ************************* ***************

chimp.LOC112206744.LOC107973052.GGT2-.646279-708040.rev.compl      aggatcacctgtgcccgggagttcaaggctgcagtgagctatgatcacaccacagtgctc   30004
FAM230B-LOC105372935-GGT2.NCBI.GGT2.rev.compl                      aggatcacctgtgcccgggagttcaaggctgcagtgagctatgatcacaccacagtgctc   28635
                                                                   ************************************************************

chimp.LOC112206744.LOC107973052.GGT2-.646279-708040.rev.compl      cagcctgggcaacaaagcaagactccatctctaaaaataaaataaaa-ttaaagaaaaga   30063
FAM230B-LOC105372935-GGT2.NCBI.GGT2.rev.compl                      cagcctgggcaacaaagcaagactccatctctaaaaataaaataaaatttaaaaaaaaga   28695
                                                                   *********************************************** ***** ******

chimp.LOC112206744.LOC107973052.GGT2-.646279-708040.rev.compl      tctttgctgtaaaagaggtacgctcaaatgcaataaaagcatataagaaggccgggtgtg   30123
FAM230B-LOC105372935-GGT2.NCBI.GGT2.rev.compl                      tcttcgctgtaaaagaggtacgctcaaatgcaataaaagcatataagaaggccgggtgtg   28755
                                                                   **** *******************************************************

chimp.LOC112206744.LOC107973052.GGT2-.646279-708040.rev.compl      gtggctcatgcctgtaatctcagcactttgggaggccaagacgggcggatcacgaggtca   30183
FAM230B-LOC105372935-GGT2.NCBI.GGT2.rev.compl                      gtggctcatgcctgtaatcccagcactttgggaggccgagacgggcggatcacgaggtca   28815
                                                                   ******************* ***************** **********************

chimp.LOC112206744.LOC107973052.GGT2-.646279-708040.rev.compl      ggagatcgagactatcctggctaacgcggagaaaccccatctcctctaaaagtacaaaaa   30243
FAM230B-LOC105372935-GGT2.NCBI.GGT2.rev.compl                      ggagattgagactatcctggctaacgcggtgaaaccccatctcctctaaaagtacaaaaa   28875
                                                                   ****** ********************** ******************************

chimp.LOC112206744.LOC107973052.GGT2-.646279-708040.rev.compl      aattagttgggctaggtggcaggcgcctgtagtcccagctactcaggaggctgaggcagg   30303
FAM230B-LOC105372935-GGT2.NCBI.GGT2.rev.compl                      aattagctgggctaggtggcaggcgcctgtagtcccagctactgaggaggctgaggcagg   28935
                                                                   ****** *********************************** ****************

chimp.LOC112206744.LOC107973052.GGT2-.646279-708040.rev.compl      agaatggcataaacccgggaggcagagcttgcagtgagccaagatcgcaccactgcactc   30363
FAM230B-LOC105372935-GGT2.NCBI.GGT2.rev.compl                      agaatggcataaaccagggaggcagagcttgcagtgagcctagatcacaccactgcactc   28995
                                                                   *************** ************************ ***** *************

chimp.LOC112206744.LOC107973052.GGT2-.646279-708040.rev.compl      cagcctgggtgacagagcgagactccgtctcaaaaaaaaaaaaa--aaaagaaaaagaaa   30421
FAM230B-LOC105372935-GGT2.NCBI.GGT2.rev.compl                      cagcctgggtgacagagcgagactccgtctcaaaaaaaaaaaaaagaaaagaaaaagaaa   29055
                                                                   ********************************************  **************

chimp.LOC112206744.LOC107973052.GGT2-.646279-708040.rev.compl      agaaaagttcttgtgacatttgtgtatgaaatcagccttcactacatggataggaccagc   30481
FAM230B-LOC105372935-GGT2.NCBI.GGT2.rev.compl                      agaaaagttcttctgacatttgtgtatgaaatcagccttcactacatggataggaccagc   29115
                                                                   ************ ***********************************************

chimp.LOC112206744.LOC107973052.GGT2-.646279-708040.rev.compl      acgcttccgcggcacgactctgcaatcttactacatttttttttactttgtattttattt   30541
FAM230B-LOC105372935-GGT2.NCBI.GGT2.rev.compl                      acgcttctgcggcacgactctgcaatcttactacatttttttttactttgtattttattt   29175
                                                                   ******* ****************************************************

chimp.LOC112206744.LOC107973052.GGT2-.646279-708040.rev.compl      attccttttgagacagagtctcactctgtcacccaggctgaagtgcagccgagatctcag   30601
FAM230B-LOC105372935-GGT2.NCBI.GGT2.rev.compl                      attccttttgagacagagtctcactctgtcacccaggctgaagtgcagccgagatctcgg   29235
                                                                   ********************************************************** *

chimp.LOC112206744.LOC107973052.GGT2-.646279-708040.rev.compl      ctcactgcaacctccacctcctgggttcaagcaattctcttgtctcagcctcccaagtag   30661
FAM230B-LOC105372935-GGT2.NCBI.GGT2.rev.compl                      ctcactgcaacctccacctcttgggttcaagcaattctcttgtctcagcctcccaagtag   29295
                                                                   ******************** ***************************************

chimp.LOC112206744.LOC107973052.GGT2-.646279-708040.rev.compl      ctgggactacaggcacacgtcaaaacgcccggctaatttttgtatttttagtagagatgg   30721
FAM230B-LOC105372935-GGT2.NCBI.GGT2.rev.compl                      ctgggactacaggcacacgtcaaaacgcccggctaatttttgtatttttagtagagatgg   29355
                                                                   ************************************************************

chimp.LOC112206744.LOC107973052.GGT2-.646279-708040.rev.compl      agtttagccatattggtcaggctggtctcgaactcctgacctcaggtgatcgacctgtct   30781
FAM230B-LOC105372935-GGT2.NCBI.GGT2.rev.compl                      agttttgccatattggtcaggctggtctcgaactcctgacctcaggtgatcgacctgtct   29415
                                                                   ***** ******************************************************

chimp.LOC112206744.LOC107973052.GGT2-.646279-708040.rev.compl      tagcctcccaaagtgctaggattacaggtgtacatttatttatttatttgagatggaatc   30841
FAM230B-LOC105372935-GGT2.NCBI.GGT2.rev.compl                      tagcctcccaaagtgctaggattacaggtgtacatttatttatttatttgagatggaatc   29475
                                                                   ************************************************************

chimp.LOC112206744.LOC107973052.GGT2-.646279-708040.rev.compl      ctgctctgtatttattaatttatttatttgagatggagtcttgctccattgcccaggcta   30901
FAM230B-LOC105372935-GGT2.NCBI.GGT2.rev.compl                      ttgctctgtatttattaatttatttatttgagatggagtcttgctccatcgcccaggcta   29535
                                                                    ************************************************ **********

chimp.LOC112206744.LOC107973052.GGT2-.646279-708040.rev.compl      gagtgcagtggtgcaatctcggctcattgcaacctctgacttccaggttcaagcgattct   30961
FAM230B-LOC105372935-GGT2.NCBI.GGT2.rev.compl                      gagtgcagcggtgcaatctcggctcattgcaacctctgccttccaggttcaagcgattct   29595
                                                                   ******** ***************************** *********************

chimp.LOC112206744.LOC107973052.GGT2-.646279-708040.rev.compl      cctgcctcagtgtcccaagtagctgggattacaggtgcctgccaccacagctggttaatt   31021
FAM230B-LOC105372935-GGT2.NCBI.GGT2.rev.compl                      cctgcctcagtgtcccaagtagctgggattacaggtgcctgccaccacagctggctaatt   29655
                                                                   ****************************************************** *****

chimp.LOC112206744.LOC107973052.GGT2-.646279-708040.rev.compl      tttgtatttttagtagagacagtgtttcaccatcttggccaggctggtctcgggctcctg   31081
FAM230B-LOC105372935-GGT2.NCBI.GGT2.rev.compl                      tttgtatttttagtagagacagtgtttcaccatcttggccaggctggtctcgggctcctg   29715
                                                                   ************************************************************

chimp.LOC112206744.LOC107973052.GGT2-.646279-708040.rev.compl      acctcatgaaccacctgcctcagcctcccaaagtgttgggattacaggcctaaggcacca   31141
FAM230B-LOC105372935-GGT2.NCBI.GGT2.rev.compl                      acctcatgaaccacctgcctcagcctcccaaagtgttgggattacaggcctaaggcacca   29775
                                                                   ************************************************************

chimp.LOC112206744.LOC107973052.GGT2-.646279-708040.rev.compl      tgttcggccatatttatttatttaattatttagagacaaagtcttgctctgtcacccagg   31201
```

```
FAM230B-LOC105372935-GGT2.NCBI.GGT2.rev.compl                      tgctcggccatatttatttatttaattatttagagacaaagtcttgctctgtcacccagg   29835
                                                                   ** *********************************************************

chimp.LOC112206744.LOC107973052.GGT2-.646279-708040.rev.compl      ctggagtgcagtggcgccatctcagctcactgcagcctccgcctctgaggtttaagcgat   31261
FAM230B-LOC105372935-GGT2.NCBI.GGT2.rev.compl                      ctggagtgcagtggcgccatctcagctcactgcagcctccgcctccgaggtttaagcaat   29895
                                                                   ********************************************* *********** **

chimp.LOC112206744.LOC107973052.GGT2-.646279-708040.rev.compl      tctcatgcctcagcctcctgaataactaggactacagatactcgccaccacgcagggatt   31321
FAM230B-LOC105372935-GGT2.NCBI.GGT2.rev.compl                      tctcatgcctcagcctcctgagtaactgggactacagatacttgccaccacgcaggga--   29953
                                                                   ********************* ***** ************** ***************

chimp.LOC112206744.LOC107973052.GGT2-.646279-708040.rev.compl      tttttttttctatttttttgtagagacacagtttcaccatgttggccaggctggtctcga   31381
FAM230B-LOC105372935-GGT2.NCBI.GGT2.rev.compl                      tttttttttctatttttttgtagagacacagtttcaccatgttggccaggctggtctcga   30013
                                                                   ************************************************************

chimp.LOC112206744.LOC107973052.GGT2-.646279-708040.rev.compl      actcctgaccttaggtgatctgacagcctcgtcctctctaagtactgggattacaggcat   31441
FAM230B-LOC105372935-GGT2.NCBI.GGT2.rev.compl                      actcctgaccttaggtgatctgacagcctcgtcctctcaaagcactgggattacaggcat   30073
                                                                   ************************************** *** *****************

chimp.LOC112206744.LOC107973052.GGT2-.646279-708040.rev.compl      gagccccttgcccggcctctcactacatttaagtgacgccatggctcatgcctgtaatcc   31501
FAM230B-LOC105372935-GGT2.NCBI.GGT2.rev.compl                      gagccccttgcccggcctctcactacatttaagtgacgccatggctcatgcctgtaatcc   30133
                                                                   ************************************************************

chimp.LOC112206744.LOC107973052.GGT2-.646279-708040.rev.compl      tagcactttgggaggccaaggcaggtggatcacctgaggtcaggagttcgacacgagcct   31561
FAM230B-LOC105372935-GGT2.NCBI.GGT2.rev.compl                      tagcactttgggaggccaaggcaggtggatcacctgaggtcaggagttcgacacgagcct   30193
                                                                   ************************************************************

chimp.LOC112206744.LOC107973052.GGT2-.646279-708040.rev.compl      ggccaacatggggaaaccccgtctctagtaaaaatacaaaaattagtcacgcgtggtggt   31621
FAM230B-LOC105372935-GGT2.NCBI.GGT2.rev.compl                      ggccaacatggggaaaccccgtctctagtaaaaatacaaaaattagtcaggtgtggtggt   30253
                                                                   ************************************************* * ********

chimp.LOC112206744.LOC107973052.GGT2-.646279-708040.rev.compl      acaagcctgtaggcccagctacttggaagactgaggcaggagaatcactttaagcgggag   31681
FAM230B-LOC105372935-GGT2.NCBI.GGT2.rev.compl                      acaagcctgtaggcccagctacttggaagactgaggcaggagaatcactttaagcgggag   30313
                                                                   ************************************************************

chimp.LOC112206744.LOC107973052.GGT2-.646279-708040.rev.compl      gcagaggttgcagtgagccaatatcatgccactgcactccagcttgggtgacggagtgag   31741
FAM230B-LOC105372935-GGT2.NCBI.GGT2.rev.compl                      gcagaggttgcagtgagccaatatcatgccactgcactccagcttgggtgacagagtgag   30373
                                                                   **************************************************** *******

chimp.LOC112206744.LOC107973052.GGT2-.646279-708040.rev.compl      acactgtctcaaa-------aaaaaaaaagagaaaaaaaatatgatgccggggcatctcgg   31794
FAM230B-LOC105372935-GGT2.NCBI.GGT2.rev.compl                      atactgtctcaaaaaaaaagaaaaaaagagagaaaaacatatgatgccggggcatctcgg   30433
                                                                   * ***********       ******* ********* **********************

chimp.LOC112206744.LOC107973052.GGT2-.646279-708040.rev.compl      cctcaatacctgcgtgagcacagtcatgtccaggccagggctgctggtcgaggtccggtc   31854
FAM230B-LOC105372935-GGT2.NCBI.GGT2.rev.compl                      cctcaatacctgggtgagcacagtcatgtccaggccagggctgctggtcgaggtccggcc   30493
                                                                   ************ ********************************************* *

chimp.LOC112206744.LOC107973052.GGT2-.646279-708040.rev.compl      ccatctcttccagcagaaagggagtaagcttgcagggaggctgggggacaagatcccagg   31914
FAM230B-LOC105372935-GGT2.NCBI.GGT2.rev.compl                      ccatctcttccagcagaaagggagtaagcttgcagggcggctgggggacaagatcccagg   30553
                                                                   ************************************ ***********************

chimp.LOC112206744.LOC107973052.GGT2-.646279-708040.rev.compl      atctcggcctccgctcatggatcagctctgataccccgagtgagctgggggtgctctgtg   31974
FAM230B-LOC105372935-GGT2.NCBI.GGT2.rev.compl                      atctcagcctctgctcatggatcagctctgagaccccgagtgagctgggggtgctctgtg   30613
                                                                   ***** ***** ******************* ****************************

chimp.LOC112206744.LOC107973052.GGT2-.646279-708040.rev.compl      cgcattggtttccccagctgtcaagtaaagggattgtatgaggaagtcttgtcaaggtgg   32034
FAM230B-LOC105372935-GGT2.NCBI.GGT2.rev.compl                      cgcattggtttccccagctgtcaagtaaagggattggatgaggaagtcttgtcaaggtgg   30673
                                                                   *********************************** ************************

chimp.LOC112206744.LOC107973052.GGT2-.646279-708040.rev.compl      aatgatctcagatttggggcagcagtgaatgatcccgctccctgggccatgccagtggcc   32094
FAM230B-LOC105372935-GGT2.NCBI.GGT2.rev.compl                      aatgatctcagatttggggcagcagtgaatgatcccgctccctgggccatgccagtggcc   30733
                                                                   ************************************************************

chimp.LOC112206744.LOC107973052.GGT2-.646279-708040.rev.compl      cggcctcggctcaacacagccccaacactctggaatggggatgagggggcagtcagctct   32154
FAM230B-LOC105372935-GGT2.NCBI.GGT2.rev.compl                      cggcctcggctgaacacagccccaacactctggaatggggatgagggggcagtcagctct   30793
                                                                   *********** ************************************************

chimp.LOC112206744.LOC107973052.GGT2-.646279-708040.rev.compl      tgctcctagtaagagagatgcaacggggctctgtggctgagctgggtgccttgcctcaca   32214
FAM230B-LOC105372935-GGT2.NCBI.GGT2.rev.compl                      tgctcctagtaagagagatgcaacagggctctgtggctgagctgggtgccttgcctcaca   30853
                                                                   ************************ ***********************************

chimp.LOC112206744.LOC107973052.GGT2-.646279-708040.rev.compl      cctgtaatcccaacctttgagaggctgaggcaggaggattgctcgaggccgggaattttg   32274
FAM230B-LOC105372935-GGT2.NCBI.GGT2.rev.compl                      cctgtaatcccaacctttgagaggccgaggcaggaggattgctcgaggccgggaattttg   30913
                                                                   ************************* **********************************

chimp.LOC112206744.LOC107973052.GGT2-.646279-708040.rev.compl      agaatagcctggacaacatagccagaccccatgtctacaaaataataataaaacatacag   32334
FAM230B-LOC105372935-GGT2.NCBI.GGT2.rev.compl                      agaatagcctggacaacatagccagaccccatgtctacaaaataataagaaaacacacag   30973
                                                                   *********************************************** ****** ****

chimp.LOC112206744.LOC107973052.GGT2-.646279-708040.rev.compl      ctatagtccaggctacttggcaggctgaggcaggagggtcccttgagtccaggaattgga   32394
FAM230B-LOC105372935-GGT2.NCBI.GGT2.rev.compl                      ctatagtccaagctacttggcaggctgaggcaggagggtcccttgagtccaggaattgga   31033
                                                                   ********** *************************************************

chimp.LOC112206744.LOC107973052.GGT2-.646279-708040.rev.compl      ggctgcattgagctataatcgcaccactgcactccagcttgggtgacaaagtgagaccct   32454
FAM230B-LOC105372935-GGT2.NCBI.GGT2.rev.compl                      ggctgcattgagctataatcgcaccactgcactccagcttgggtgacaaagtgagaccct   31093
                                                                   ************************************************************

chimp.LOC112206744.LOC107973052.GGT2-.646279-708040.rev.compl      gtctctaaaagaaaaaacattggcctgtgagcatgggtttgagtttcaaacaggagccgg   32514
FAM230B-LOC105372935-GGT2.NCBI.GGT2.rev.compl                      gtctctaaaagaaaaaaaattggcctgtgagcatgggcttgattttcaaacaggacccgg   31153
                                                                   ***************** ******************* **** ************ ****

chimp.LOC112206744.LOC107973052.GGT2-.646279-708040.rev.compl      agggtagggtaaacttgtcggtaaatctaaatgaatgttattggtataaaattacaatag   32574
FAM230B-LOC105372935-GGT2.NCBI.GGT2.rev.compl                      agggtagggtaaacgtgtgggtaaatctaaatgaatgttattggtataaaattacagtag   31213
                                                                   ************** *** ************************************* ***

chimp.LOC112206744.LOC107973052.GGT2-.646279-708040.rev.compl      tatagaaaatgatatcttgtggggtttaaaataaataaaacattctgaaatatgtatggg   32634
FAM230B-LOC105372935-GGT2.NCBI.GGT2.rev.compl                      tatagaaaatgatatcttgtggggtttaaaataaataaaacatactgaaatatgtattgg   31273
                                                                   ****************************************** ************** **

chimp.LOC112206744.LOC107973052.GGT2-.646279-708040.rev.compl      tacagttatatatctgggatttgcactgaaataatgtggggtagagggaagcaggaaaga   32694
FAM230B-LOC105372935-GGT2.NCBI.GGT2.rev.compl                      tacagttatatatctgggatttgcactgaaataatgtggggtagagggaagcaggaaaga   31333
                                                                   ************************************************************

chimp.LOC112206744.LOC107973052.GGT2-.646279-708040.rev.compl      ggatacatgaaattagcttggccataagattgttgttgaaattgaatggatactcggggc   32754
FAM230B-LOC105372935-GGT2.NCBI.GGT2.rev.compl                      gtatacatgaaatgagcttggccataagattgttgttgaaattgaatggatactcggggc   31393
                                                                   * *********** **********************************************

chimp.LOC112206744.LOC107973052.GGT2-.646279-708040.rev.compl      ttcattacacaattctttttactcttacatagctctacactctcaacataaataagaata   32814
FAM230B-LOC105372935-GGT2.NCBI.GGT2.rev.compl                      ttcattacacaattctctttactcttacatagctctacactctcaacataaataagaata   31453
                                                                   **************** *******************************************

chimp.LOC112206744.LOC107973052.GGT2-.646279-708040.rev.compl      aaaacacaaaaaacacacagatacatctatgcacacacatgtattttaaatacacaaaaa   32874
FAM230B-LOC105372935-GGT2.NCBI.GGT2.rev.compl                      aaaacacaaaaaacacacagatacatctatgcacacacacatatttaaaatacacaaaaa   31513
                                                                   ***************************************  ***** *************

chimp.LOC112206744.LOC107973052.GGT2-.646279-708040.rev.compl      tattagcatataagtcactggggtaaatttagttcctgttccaaggttcttgtactgac   32934
FAM230B-LOC105372935-GGT2.NCBI.GGT2.rev.compl                      tattagcatataagtcactggggtaaatttagttcctgttccaaggttcttgtactgac   31573
                                                                   ************************************************************

chimp.LOC112206744.LOC107973052.GGT2-.646279-708040.rev.compl      taggaagaggatagaagtactaactcataggctgggcgtggtggctcacgcctgtaatcc   32994
FAM230B-LOC105372935-GGT2.NCBI.GGT2.rev.compl                      taggaagaggatagaagtactaactcataggctgggcgcggtggctcacgcctgtaatcc   31633
                                                                   ************************************** *********************

chimp.LOC112206744.LOC107973052.GGT2-.646279-708040.rev.compl      caacactttaggacgccgaggtaagcagatctcttaaggtcaggagttcaagaccagcct   33054
FAM230B-LOC105372935-GGT2.NCBI.GGT2.rev.compl                      caacactttaggatgccgaggtaggcagatctcttaaggtccggagttcaagaccagcct   31693
                                                                   ************* ********* ***************** ******************

chimp.LOC112206744.LOC107973052.GGT2-.646279-708040.rev.compl      ggccaacatggtgaaaccctgtctctactaaaaagaatacaaaaattggccgggcatag   33114
FAM230B-LOC105372935-GGT2.NCBI.GGT2.rev.compl                      ggccaacatggtgaaaccctgtctctactgaaaagaatacaaaaattggccgggcatag   31753
                                                                   **************************** *******************************

chimp.LOC112206744.LOC107973052.GGT2-.646279-708040.rev.compl      tggtgcacacctgtggtcccagctactcaggtgactgaggcaggagaattgcttgaaccc   33174
FAM230B-LOC105372935-GGT2.NCBI.GGT2.rev.compl                      tggtgcacacctgtggtcccagctactcaggtgactgaggcaggagaattgcttgaaccc   31813
                                                                   ************************************************************

chimp.LOC112206744.LOC107973052.GGT2-.646279-708040.rev.compl      aagaagtggaggttgcagtgaaccaagattgctccactgcactccagcctgggcagcaga   33234
FAM230B-LOC105372935-GGT2.NCBI.GGT2.rev.compl                      aagaagtggaggttgcagtgaaccaagattgctccactgcactccagcctgggcagcaga   31873
                                                                   ************************************************************

chimp.LOC112206744.LOC107973052.GGT2-.646279-708040.rev.compl      ggaagactctctctgtctcaacaacaacaaaaagtactagctcatgttagactttgataa   33294
FAM230B-LOC105372935-GGT2.NCBI.GGT2.rev.compl                      ggaagactctctctatctcaaccacaacaaaaagtactagctcatgttagactttgataa   31933
                                                                   ************** ******* *************************************

chimp.LOC112206744.LOC107973052.GGT2-.646279-708040.rev.compl      gtgaaggatgcatgttgtaagctctaaaataatccactgtcatcttttaaaataactcta   33354
FAM230B-LOC105372935-GGT2.NCBI.GGT2.rev.compl                      gggaaggatgcatgttgtaagctctaaaataatcca--gtcatcttttaaaataactcta   31991
```

```
                                                                   * **********************************  **********************

chimp.LOC112206744.LOC107973052.GGT2-.646279-708040.rev.compl      agactgcacagttatgaaactaatag---aggaggaaattaaataataaaaataataaat   33411
FAM230B-LOC105372935-GGT2.NCBI.GGT2.rev.compl                      agactgcacagttatgaaactaatagagaaggaggaaattaaataataaaaataataaat   32051
                                                                   **************************   *******************************

chimp.LOC112206744.LOC107973052.GGT2-.646279-708040.rev.compl      ccaaaacaagatgtgagaggagataagaagaaatagaataggcatggaaaacaaattggt   33471
FAM230B-LOC105372935-GGT2.NCBI.GGT2.rev.compl                      ccaaaacaagatgtgagaggagataagaagaaatagaataggcatggaaaacaaattggt   32111
                                                                   ************************************************************

chimp.LOC112206744.LOC107973052.GGT2-.646279-708040.rev.compl      ggtgggtttcaacccaaataaatcattagttacatttaaaaggacaataaaaattaaaat   33531
FAM230B-LOC105372935-GGT2.NCBI.GGT2.rev.compl                      ggtgggtttcaacccaaataaatcattagttacatttaaaaggacaataaaaattaaaat   32171
                                                                   ************************************************************

chimp.LOC112206744.LOC107973052.GGT2-.646279-708040.rev.compl      aatcgaaaataaagtaaaacccaactaatgccttttatataagaatacagagaggtggaa   33591
FAM230B-LOC105372935-GGT2.NCBI.GGT2.rev.compl                      aattgaaaataaagtaaaacccaactaatgccttttatataagggtacagagaggtggaa   32231
                                                                   *** ***************************************  ***************

chimp.LOC112206744.LOC107973052.GGT2-.646279-708040.rev.compl      gataatgaaaaatatgtcatgcatgcactaaccaagaaagctgtataac-----------   33640
FAM230B-LOC105372935-GGT2.NCBI.GGT2.rev.compl                      gatcatgaaaaatatgtcatgcatgtactaaccaagaaagctgtataacttttttttttt   32291
                                                                   *** ********************* ***********************

chimp.LOC112206744.LOC107973052.GGT2-.646279-708040.rev.compl      tttttttttttttttggagatagagcctcactctgtctcccaggctggagtgcagtgat   33700
FAM230B-LOC105372935-GGT2.NCBI.GGT2.rev.compl                      tttttttttttttttggagatagagcctcactctgtctcccaggctggagtgcagtgat   32351
                                                                   ************************************************************

chimp.LOC112206744.LOC107973052.GGT2-.646279-708040.rev.compl      gtgatcttggcttacagcaatctctcccttctaggctcaagcgattctcccacctcagca   33760
FAM230B-LOC105372935-GGT2.NCBI.GGT2.rev.compl                      gtgatcttggcttacagcaatctctcccttctaggctcaagcgattctcccacctcagca   32411
                                                                   ************************************************************

chimp.LOC112206744.LOC107973052.GGT2-.646279-708040.rev.compl      tcccaagtagctgggactacaagtgtgccaacttagaattatattagccacacccagcta   33820
FAM230B-LOC105372935-GGT2.NCBI.GGT2.rev.compl                      tcccaagtagctgggactacaagtgtgccaacttagaattatattagccacacccagcta   32471
                                                                   ************************************************************

chimp.LOC112206744.LOC107973052.GGT2-.646279-708040.rev.compl      attttgtatttttttgtagaggcagggtctcgccatgttgcccaggctggtcttgaactc   33880
FAM230B-LOC105372935-GGT2.NCBI.GGT2.rev.compl                      attttgtatttttttgtagaggcagggtctcgccatgttgcccaggttggtcttgaactc   32531
                                                                   ********************************************** *************

chimp.LOC112206744.LOC107973052.GGT2-.646279-708040.rev.compl      ctgggcttcagtgatccacccacctcgacatccagcaaagtgccaagattacagccatga   33940
FAM230B-LOC105372935-GGT2.NCBI.GGT2.rev.compl                      ctgggcttcagtgatccacccacctcgacctccagcaaagtgccaagattacagccatga   32591
                                                                   ***************************** ******************************

chimp.LOC112206744.LOC107973052.GGT2-.646279-708040.rev.compl      gccaccatgcccagcataaccattttaatgaagtagactttaagaagaaaagtattatt   34000
FAM230B-LOC105372935-GGT2.NCBI.GGT2.rev.compl                      gccaccatgcccagcataactattttaatgaagtagactttaagaagaaaagtattatt   32651
                                                                   ******************** ***************************************

chimp.LOC112206744.LOC107973052.GGT2-.646279-708040.rev.compl      agaggtaagagacacatcacggaaaagaagaatttactaggagccaggcgcagtggctca   34060
FAM230B-LOC105372935-GGT2.NCBI.GGT2.rev.compl                      agaggtaagagacacatcacagaaaagaagaatttactaggagccaggcgcaatggctcg   32711
                                                                   ******************** ******************************* ******

chimp.LOC112206744.LOC107973052.GGT2-.646279-708040.rev.compl      tgcctgtaattccagcactttgtgaggccaaggcggcggatcacctgaggttgggagttc   34120
FAM230B-LOC105372935-GGT2.NCBI.GGT2.rev.compl                      tgcctgtaattccagcactttgtgaggccaaggcggcggatcacctgaggttgggagttc   32771
                                                                   ************************************************************

chimp.LOC112206744.LOC107973052.GGT2-.646279-708040.rev.compl      aagaccagcctgaccaacatggagaagccctgtctctactaaaaatacaaaaattagcca   34180
FAM230B-LOC105372935-GGT2.NCBI.GGT2.rev.compl                      aagaccagcctgaccaacatggagaagccctgtctctactaaaaatacaaaaattagcca   32831
                                                                   ************************************************************

chimp.LOC112206744.LOC107973052.GGT2-.646279-708040.rev.compl      agcatggtggcacatgcctgtaatcccagctactcaggaggctgagggaggagaattgct   34240
FAM230B-LOC105372935-GGT2.NCBI.GGT2.rev.compl                      agcatggtggcacatgcctgtaatcccagctactcaggaggctgagggaggagaattgct   32891
                                                                   ************************************************************

chimp.LOC112206744.LOC107973052.GGT2-.646279-708040.rev.compl      tggacccaggaagtggaggttgcggtgagctgagattgtgccattgcattccagcctggg   34300
FAM230B-LOC105372935-GGT2.NCBI.GGT2.rev.compl                      tggacccaggaagtggaggttgcggtgagctgagattgtgccattgcattccagcctggg   32951
                                                                   ************************************************************

chimp.LOC112206744.LOC107973052.GGT2-.646279-708040.rev.compl      caacaagagcaaaactctgtctc----------aaaaaaaaagaagttactagctagtta   34350
FAM230B-LOC105372935-GGT2.NCBI.GGT2.rev.compl                      caacaagagcaaaactctgtctcaaaaaaaaaaaaaaaaaaagaagttactagctagttt   33011
                                                                   ***********************          **************************

chimp.LOC112206744.LOC107973052.GGT2-.646279-708040.rev.compl      cggtaattcttaacatccaggaaactggttgtgaaagtttttcagagaaactaaaccaat   34410
FAM230B-LOC105372935-GGT2.NCBI.GGT2.rev.compl                      cggtaattcttaacaaccaggaaactggatgtgaaagtttttcagagaaactaaaccaat   33071
                                                                   *************** ************ *******************************

chimp.LOC112206744.LOC107973052.GGT2-.646279-708040.rev.compl      agattatacatagagagagatttattgaggaattggctcacatgattgtggggactagca   34470
FAM230B-LOC105372935-GGT2.NCBI.GGT2.rev.compl                      agattatacatagagagagatttatttaggaattggctcacatgattgtggggactagca   33131
                                                                   ************************** *********************************

chimp.LOC112206744.LOC107973052.GGT2-.646279-708040.rev.compl      ag-tttaaaatctgtagggcaagccagcaggctataaattcaggtaagagttgatctcga   34529
FAM230B-LOC105372935-GGT2.NCBI.GGT2.rev.compl                      agttttaaaatctgtagggcaagccagcaggctataaattcaggtaagagttgatctcga   33191
                                                                   ** *********************************************************

chimp.LOC112206744.LOC107973052.GGT2-.646279-708040.rev.compl      agtctggaacctaaaatctgtagagcagtcagcaggccagaaactcaggcagggtttgtg   34589
FAM230B-LOC105372935-GGT2.NCBI.GGT2.rev.compl                      agtctggaacctaaaatctgtagagcagtcagcaggccagaaactcaggcagggtttgtg   33251
                                                                   ************************************************************

chimp.LOC112206744.LOC107973052.GGT2-.646279-708040.rev.compl      tgttacagtcttgaagcagaattcctgcttctttgggaaacct--gtttttgttcttaag   34647
FAM230B-LOC105372935-GGT2.NCBI.GGT2.rev.compl                      tgttacagtcttgaagcagaattcctgcttctctgggaaacctcagtttttgttcttaag   33311
                                                                   ******************************** **********  ***************

chimp.LOC112206744.LOC107973052.GGT2-.646279-708040.rev.compl      gccttcaagtgattggaggtggcccacccatattatggtgggtaatctgttttacttaaa   34707
FAM230B-LOC105372935-GGT2.NCBI.GGT2.rev.compl                      gccttcaactgattggaggtggcccacccatattatggtgggtaatctgttttacttaaa   33371
                                                                   ******** ***************************************************

chimp.LOC112206744.LOC107973052.GGT2-.646279-708040.rev.compl      gtcaattgactgtcagtgttaaccacatctatgaaataacctcccagcaagatattgaca   34767
FAM230B-LOC105372935-GGT2.NCBI.GGT2.rev.compl                      gtcaattgactgtcagtgttaatcacatctatgaaataacctcccagcaagatattgaca   33431
                                                                   ********************** *************************************

chimp.LOC112206744.LOC107973052.GGT2-.646279-708040.rev.compl      agtatttgaccaaacaacggggcaccatagcttagccaagttgacacataaattaaccat   34827
FAM230B-LOC105372935-GGT2.NCBI.GGT2.rev.compl                      agtatttgaccaaacaacaggacaccatagcttagccaagttgacacataaattaaccat   33491
                                                                   ****************** ** **************************************

chimp.LOC112206744.LOC107973052.GGT2-.646279-708040.rev.compl      caggagcgagtagaatatccaaaaaacaacatactaggggtattatatcttatatagc--   34885
FAM230B-LOC105372935-GGT2.NCBI.GGT2.rev.compl                      caggagcaagtagaatatccaaaaaacaacatactaggggtattatatcttatatagcta   33551
                                                                   ******* **************************************************

chimp.LOC112206744.LOC107973052.GGT2-.646279-708040.rev.compl      ---taattatataaaacatataattatagaatgaagatattaagataaccattagaacaa   34942
FAM230B-LOC105372935-GGT2.NCBI.GGT2.rev.compl                      ttataattatataaaacatataattatagaatgacgatattaagataaccattagaacaa   33611
                                                                      ******************************* *************************

chimp.LOC112206744.LOC107973052.GGT2-.646279-708040.rev.compl      aaatataaacttttctt------ttcttttttttttgagaccaagtcttgctctgtcac   34996
FAM230B-LOC105372935-GGT2.NCBI.GGT2.rev.compl                      aaatataaacttttctttctttctttttttttttttgagaccaagtcttgctctgtcac   33671
                                                                   *****************      ** **********************************

chimp.LOC112206744.LOC107973052.GGT2-.646279-708040.rev.compl      ccaggctggagtgcagtggtgcaatcttggcttactgcaacctttgcctcctgagttcaa   35056
FAM230B-LOC105372935-GGT2.NCBI.GGT2.rev.compl                      ccaggctggagtgcagtggtgcaatcttggcttactgaaacctttgcctcctgggttcaa   33731
                                                                   ************************************* *************** ******

chimp.LOC112206744.LOC107973052.GGT2-.646279-708040.rev.compl      gtgatcctcctgtctcagcctcccaagtagctgggattacaggcacctgctaccatgccc   35116
FAM230B-LOC105372935-GGT2.NCBI.GGT2.rev.compl                      gtgattctcctgtctcagcctcccaagtatctgggattacaggcacctgctaccatgccc   33791
                                                                   ***** *********************** ******************************

chimp.LOC112206744.LOC107973052.GGT2-.646279-708040.rev.compl      agctaattttgtatttttagtagagacatggtttcaccatgttgcccaggctggtctcc   35176
FAM230B-LOC105372935-GGT2.NCBI.GGT2.rev.compl                      agctaattttgtatttttagtagagacatggtttcaccatgttgcccaggctggtctcc   33851
                                                                   ************************************************************

chimp.LOC112206744.LOC107973052.GGT2-.646279-708040.rev.compl      aactcctgacctcaagtgagccagcccccctcggcctcccaaagtgctgggattacaggtg   35236
FAM230B-LOC105372935-GGT2.NCBI.GGT2.rev.compl                      aactcctgacctcaagtgagccaccccccctcggcctcccaaagtgctgggattacaggtg   33911
                                                                   *********************** ************************************

chimp.LOC112206744.LOC107973052.GGT2-.646279-708040.rev.compl      tgagccaccacacccagccaaaaatcacctttttcacaaggatcaaaacagtcattacgc   35296
FAM230B-LOC105372935-GGT2.NCBI.GGT2.rev.compl                      tgagccaccacacccagccaaaaatcaccttttttacaaggatcaaaacagtcattatgc   33971
                                                                   ********************************** ********************** **

chimp.LOC112206744.LOC107973052.GGT2-.646279-708040.rev.compl      tggagatgacagacctcactgtcaccatgctccttttgtatgtctactaggcatggtgct   35356
FAM230B-LOC105372935-GGT2.NCBI.GGT2.rev.compl                      tggagatgacagacctcactgtcaccatgctccttttgtatgtctactaggcacggtgct   34031
                                                                   ***************************************************** ******

chimp.LOC112206744.LOC107973052.GGT2-.646279-708040.rev.compl      gggtccacactcacagaaagcttaggagctcgcacccaggggctccggctgtagcagaat   35416
FAM230B-LOC105372935-GGT2.NCBI.GGT2.rev.compl                      gggtccacactcacagaaaccttaggaactcgcacccaggggctccggctgtagcagaat   34091
                                                                   ******************* ******* ********************************

chimp.LOC112206744.LOC107973052.GGT2-.646279-708040.rev.compl      cccaagaataaaacctggtgctgaaagagtaggagatgaggccggatgccatgactcact   35476
FAM230B-LOC105372935-GGT2.NCBI.GGT2.rev.compl                      cccaagaataaaacctggtgctgaaagagtaggagatgaggccaggtgccatgactcact   34151
                                                                   ******************************************* * **************
```

```
chimp.LOC112206744.LOC107973052.GGT2-.646279-708040.rev.compl      cctgtaatgccagcactttgggtggccaaggcgggtgaatcaagagatagagaccatcct   35536
FAM230B-LOC105372935-GGT2.NCBI.GGT2.rev.compl                      cctgtaatgccagcactttgggtggccaaggcgggtgaatcaagagatagagaccatcct   34211
                                                                   ************************************************************

chimp.LOC112206744.LOC107973052.GGT2-.646279-708040.rev.compl      ggccaacatggtgaaaccccgtctctactaaaatacaaaaattagctgggcgtggtggc   35596
FAM230B-LOC105372935-GGT2.NCBI.GGT2.rev.compl                      ggccaacacggtgaaaccccgtctctactaaaatacagaaattagcggggcgtggtggc   34271
                                                                   ******** ***************************** ******** ************

chimp.LOC112206744.LOC107973052.GGT2-.646279-708040.rev.compl      tggcacctgtagtcccagctactcaggaggctgaggcaggagaatcatttgaaccgagga   35656
FAM230B-LOC105372935-GGT2.NCBI.GGT2.rev.compl                      tggcacctgtagtcccagctactcaggaggctgaggcaggagaatcatttgaaccgagga   34331
                                                                   ************************************************************

chimp.LOC112206744.LOC107973052.GGT2-.646279-708040.rev.compl      agcagaggttgcagtaagctgagatcgtgccactgcactccagcctggtgacagagtgag   35716
FAM230B-LOC105372935-GGT2.NCBI.GGT2.rev.compl                      agcagaggttgcagtaagctgagatcgcgccactgcactccagcctggtgacagagtgag   34391
                                                                   *************************** ********************************

chimp.LOC112206744.LOC107973052.GGT2-.646279-708040.rev.compl      acaccgtctcaaaaaaaaaaaaaaaaaaaaaaaagcaggacactgaactctgggagggcc   35776
FAM230B-LOC105372935-GGT2.NCBI.GGT2.rev.compl                      acaccgtc-------acaaaaaaaaaaaaaaaaagcaggacactgaactctgggagggcc   34444
                                                                   ********       * *******************************************

chimp.LOC112206744.LOC107973052.GGT2-.646279-708040.rev.compl      tcctggtgagaggtgagcacagaggggagagatggaggcaggagcatgggcttctggtgg   35836
FAM230B-LOC105372935-GGT2.NCBI.GGT2.rev.compl                      tcctggtgagaggtgagcacagaggggagagatggaggcaggagcatgggcttctggtgg   34504
                                                                   ************************************************************

chimp.LOC112206744.LOC107973052.GGT2-.646279-708040.rev.compl      ccccagcagaccctgtggcagcgtggccagggtcctctgcagggaggaatcttggccagg   35896
FAM230B-LOC105372935-GGT2.NCBI.GGT2.rev.compl                      ccccagcagaccctgtggcagcgtggccagggtcctctgcagggaggaatcttggccagg   34564
                                                                   ************************************************************

chimp.LOC112206744.LOC107973052.GGT2-.646279-708040.rev.compl      atgacgctgtagcaggcctcttcctgaggcctccagccagcccggccagggacccagcgt   35956
FAM230B-LOC105372935-GGT2.NCBI.GGT2.rev.compl                      atgacgctgtagcaggcctcttcctgaggcctccagccagcccggccagggtcccagcgt   34624
                                                                   *************************************************** ********

chimp.LOC112206744.LOC107973052.GGT2-.646279-708040.rev.compl      ccagtgacccctgtttcacagcagcagctggggccagccccaggctctcttccactccca   36016
FAM230B-LOC105372935-GGT2.NCBI.GGT2.rev.compl                      ccagtgacccctgtttcacagcagcagctggggccagccccaggctctcttccactccca   34684
                                                                   ************************************************************

chimp.LOC112206744.LOC107973052.GGT2-.646279-708040.rev.compl      gcttcttaaaactggaagtggagagagttgtttgataaaacactggggcaaaccacatcc   36076
FAM230B-LOC105372935-GGT2.NCBI.GGT2.rev.compl                      gcttcttaaaactggaagtggagagagttgtttgataaaacactggggcaaaccacatcc   34744
                                                                   ************************************************************

chimp.LOC112206744.LOC107973052.GGT2-.646279-708040.rev.compl      tctcttcaccaagggagagttcgaggggatgccagcagagggcgctttagagtagagacc   36136
FAM230B-LOC105372935-GGT2.NCBI.GGT2.rev.compl                      tctcttcaccaagggagagttggaggggatgccggcagagggagctttagagtagagacc   34804
                                                                   ********************* *********** ******** *****************

chimp.LOC112206744.LOC107973052.GGT2-.646279-708040.rev.compl      cctacccaaccagtgaccgtcacgcacacagcagggcatgctatggagacccccagacag   36196
FAM230B-LOC105372935-GGT2.NCBI.GGT2.rev.compl                      cctacccaaccagtgaccgtcacgcacacagcagggcatgctatggagacccccagacag   34864
                                                                   ************************************************************

chimp.LOC112206744.LOC107973052.GGT2-.646279-708040.rev.compl      tcactcggggagacccagcaggtccagactcttcagagatctgtggcagcaggtccccac   36256
FAM230B-LOC105372935-GGT2.NCBI.GGT2.rev.compl                      tcactcggggagacccagcaggtccagactcttcagagatctgtggcagcaggtccccac   34924
                                                                   ************************************************************

chimp.LOC112206744.LOC107973052.GGT2-.646279-708040.rev.compl      tcccaaaagccacgtgcccacgggtggtctctggtgcctgagaccccagtctcatttgca   36316
FAM230B-LOC105372935-GGT2.NCBI.GGT2.rev.compl                      tcccaaaagccacgtgcccacgggtggtctctggtgcctgagaccccagtctcatttgca   34984
                                                                   ************************************************************

chimp.LOC112206744.LOC107973052.GGT2-.646279-708040.rev.compl      tctttgcaacttcgagtttaagtgggtgtcgcatctctgtatgtcctcctgagcagagga   36376
FAM230B-LOC105372935-GGT2.NCBI.GGT2.rev.compl                      tctttgcaacttcgagtttaagtgggtgtcgcatctctgtatgtcctcccgagcagagga   35044
                                                                   ************************************************* **********

chimp.LOC112206744.LOC107973052.GGT2-.646279-708040.rev.compl      ggggcacagcctggggtggcagctggcgtcaaaccctcaaatcccctgagagccactggg   36436
FAM230B-LOC105372935-GGT2.NCBI.GGT2.rev.compl                      ggggcacagcctggggtggcagctggcgtcaaaccctcaaatcccctgagagccactggg   35104
                                                                   ************************************************************

chimp.LOC112206744.LOC107973052.GGT2-.646279-708040.rev.compl      gagactaagcagtccccagcccccacttgtccctgagctgccattctcagccctgtggga   36496
FAM230B-LOC105372935-GGT2.NCBI.GGT2.rev.compl                      gagactaagcagtccccagcccctacttgtccctgagctgccattctcagccctgtggga   35164
                                                                   *********************** ************************************

chimp.LOC112206744.LOC107973052.GGT2-.646279-708040.rev.compl      ggagacagaaagccctgaagagaaaccaaaggaccaggtcaggaggggct--gggggggt   36554
FAM230B-LOC105372935-GGT2.NCBI.GGT2.rev.compl                      ggagacagaaagccctgaagagaaaccaaaggaccaggtcaggaggggctggggggggt   35224
                                                                   **************************************************  ********

chimp.LOC112206744.LOC107973052.GGT2-.646279-708040.rev.compl      ggcatgagcaatcagggcagggaaggatggacagatggggaatggagggaagaaggaat   36614
FAM230B-LOC105372935-GGT2.NCBI.GGT2.rev.compl                      ggcatgagcaatcagggcagggaaggatgcacagatggggaatggagggaagaaggaat   35284
                                                                   ***************************** ******************************

chimp.LOC112206744.LOC107973052.GGT2-.646279-708040.rev.compl      gaatgaaacggtgaatgaatgaacaaagagagagaacggccattcctcccttgctttagt   36674
FAM230B-LOC105372935-GGT2.NCBI.GGT2.rev.compl                      gaatgaaaaggtgaatgaatgaacaaagagagagaacggccactcctcccttgctttagt   35344
                                                                   ******** ********************************* *****************

chimp.LOC112206744.LOC107973052.GGT2-.646279-708040.rev.compl      ttacaaagtactgggatcctcccaacagcctgcaagacagaatttctgggaagcagacca   36734
FAM230B-LOC105372935-GGT2.NCBI.GGT2.rev.compl                      ttacaaagtactgggatcctcccaacagcctgcaagacagaatttctgggaagcagacca   35404
                                                                   ************************************************************

chimp.LOC112206744.LOC107973052.GGT2-.646279-708040.rev.compl      ggtggctggcagggaggggaggcttgccctggcttttgtgggcccaatgggaggcagggg   36794
FAM230B-LOC105372935-GGT2.NCBI.GGT2.rev.compl                      ggtggctggcagggaggggaggcttgccctggcttttgtgggcccaatgggaggcagggg   35464
                                                                   ************************************************************

chimp.LOC112206744.LOC107973052.GGT2-.646279-708040.rev.compl      gcaagaaggggcatcctgtgtgtgtcctccctgcagcggcagcagcaccttcctggaaga   36854
FAM230B-LOC105372935-GGT2.NCBI.GGT2.rev.compl                      gcaagaaggggcatcctgtgtgtgtcctccctgcagcggcagcagcaccttcctggaaga   35524
                                                                   ************************************************************

chimp.LOC112206744.LOC107973052.GGT2-.646279-708040.rev.compl      gggtcaggaaacactcactgtggcccctctccatcacgacctcatccaggacaccaaata   36914
FAM230B-LOC105372935-GGT2.NCBI.GGT2.rev.compl                      gggtcaggaaacacccgctgtggcccctctccaccacgccctcatccaggacaccaagta   35584
                                                                   ************** * **************** **** ****************** **

chimp.LOC112206744.LOC107973052.GGT2-.646279-708040.rev.compl      tcaagtcactcagctcacgagacccaggccctgactcagggagagaggatgtgaggggtg   36974
FAM230B-LOC105372935-GGT2.NCBI.GGT2.rev.compl                      tc-agtcactcagctcacgagacccaggccctgactcagggagagaggatgtgaggggtg   35643
                                                                   ** *********************************************************

chimp.LOC112206744.LOC107973052.GGT2-.646279-708040.rev.compl      gggcaccgggctcctcaggactgagagacctgagatgtggccccgggctgggtgttgggg   37034
FAM230B-LOC105372935-GGT2.NCBI.GGT2.rev.compl                      gggcaccgggctcctcaggactgagagacctgagatgtggccccgggctgggtgtagggg   35703
                                                                   ******************************************************* ****

chimp.LOC112206744.LOC107973052.GGT2-.646279-708040.rev.compl      cagactggctatggcagcattgtgtgtaccccagcaggccagtacccatgcagggagcct   37094
FAM230B-LOC105372935-GGT2.NCBI.GGT2.rev.compl                      cagactggctatggcagcattgtgtgtaccccagcaggccagtacccacgcagggagcct   35763
                                                                   ************************************************ ***********

chimp.LOC112206744.LOC107973052.GGT2-.646279-708040.rev.compl      ccaaaccccttcacccatgaccctgggagaagaccgcaggcttggagaattggcctcact   37154
FAM230B-LOC105372935-GGT2.NCBI.GGT2.rev.compl                      ccaaaccccttcacccatgaccctgggagaagaccgcagccttggagaattggcctcact   35823
                                                                   **************************************** *******************

chimp.LOC112206744.LOC107973052.GGT2-.646279-708040.rev.compl      gaagggccctgcaccggccagcagggtcaggcggggccagacaggttcccacctgggata   37214
FAM230B-LOC105372935-GGT2.NCBI.GGT2.rev.compl                      gaaggggcctgcgccggccagcagggtcaggcggggccagacaggttcccacctgggata   35883
                                                                   ****** ***** ***********************************************

chimp.LOC112206744.LOC107973052.GGT2-.646279-708040.rev.compl      tgcaaatgggcctcctgaatcctggagccaggtatggactcacacaccaccattgtcccc   37274
FAM230B-LOC105372935-GGT2.NCBI.GGT2.rev.compl                      tgcaaatgggcctcctgaatcctggagccaggtatggactcacacaccaccattgtcccc   35943
                                                                   ************************************************************

chimp.LOC112206744.LOC107973052.GGT2-.646279-708040.rev.compl      aagtccccatctgccccacgggcacaccctgccacctgttctgtgcaagagccctgaggc   37334
FAM230B-LOC105372935-GGT2.NCBI.GGT2.rev.compl                      aagtccccatctgccccacgggcacaccctgccgcctgttctgtgcaagggccctgaggc   36003
                                                                   ********************************* *************** **********

chimp.LOC112206744.LOC107973052.GGT2-.646279-708040.rev.compl      tgtctccttgcgctcaagccctgcaggtgctgaagcccacacacacagctcctgcttcct   37394
FAM230B-LOC105372935-GGT2.NCBI.GGT2.rev.compl                      tgtctccttgcgctcaagccctgcaggtgctgaagcccacacacacggctcctgcttcct   36063
                                                                   ********************************************** *************

chimp.LOC112206744.LOC107973052.GGT2-.646279-708040.rev.compl      gggccagtgcacgtgcacacacacacacgtgcacacacaca------cccacaaatatac   37448
FAM230B-LOC105372935-GGT2.NCBI.GGT2.rev.compl                      gggccagtgcacgtgcacacacacacacgtgcacacacacacacaccccccacaaatatac   36123
                                                                   *****************************************      *************

chimp.LOC112206744.LOC107973052.GGT2-.646279-708040.rev.compl      ccacacacaatcacacacattcacacatacccacccccatacactcacacattcacaccc   37508
FAM230B-LOC105372935-GGT2.NCBI.GGT2.rev.compl                      ccacacacaatcacacacattcacacatacccacccccccatactcaca-----------   36172
                                                                   **************************************  * *******

chimp.LOC112206744.LOC107973052.GGT2-.646279-708040.rev.compl      acacccacacatacacacacacaatcacacatttacacacacccacacacccacactc   37568
FAM230B-LOC105372935-GGT2.NCBI.GGT2.rev.compl                      -------------------------ctcacatttacacacacccacacacccacactc   36207
                                                                                            * *********************************

chimp.LOC112206744.LOC107973052.GGT2-.646279-708040.rev.compl      acacact--cagtcacacacaccctcacacagcgaaacacaat--cacacattcacaccc   37624
FAM230B-LOC105372935-GGT2.NCBI.GGT2.rev.compl                      acacactcacagtcacacacaccctcacacagcgaaacacaatcacacattcacaccc   36267
                                                                   *******  **********************************  ***************
```

```
chimp.LOC112206744.LOC107973052.GGT2-.646279-708040.rev.compl      acccacacccccacacactcacactcacatatactcacacacacccacacacacatacac   37684
FAM230B-LOC105372935-GGT2.NCBI.GGT2.rev.compl                      acccacacccccacacactcacactcacatatactcacacacacccacacacacatacac   36327
                                                                   ************************************************************

chimp.LOC112206744.LOC107973052.GGT2-.646279-708040.rev.compl      aaacacaatcacacacattcacacaca---------------------------------   37711
FAM230B-LOC105372935-GGT2.NCBI.GGT2.rev.compl                      aaacacaatcacacacattcacacacacccacactcacacatacacacacccaaacacga   36387
                                                                   ***************************

chimp.LOC112206744.LOC107973052.GGT2-.646279-708040.rev.compl      ---------------------caccccccacaaacacactcacacatatacccacacaca   37750
FAM230B-LOC105372935-GGT2.NCBI.GGT2.rev.compl                      tcacacacattcacacacacccacaccccacaaacacactcacacatatacccacacaca   36447
                                                                                        *** ***********************************

chimp.LOC112206744.LOC107973052.GGT2-.646279-708040.rev.compl      ctcacactcacacataatctcacacacacacacacatgctcacacacacacgccttctcc   37810
FAM230B-LOC105372935-GGT2.NCBI.GGT2.rev.compl                      ctcacacata--------atctctcacacacacacatgctcacacacacacgccttctcc   36499
                                                                   *******  *         ** * ************************************

chimp.LOC112206744.LOC107973052.GGT2-.646279-708040.rev.compl      aggaggggctggctgccaaaggccacccagcttcctcccacgtctcactcaccgtacaat   37870
FAM230B-LOC105372935-GGT2.NCBI.GGT2.rev.compl                      aggaggggctggctgccaaaggccacccagcttcctcccacgtctcactcaccgtacaat   36559
                                                                   ************************************************************

chimp.LOC112206744.LOC107973052.GGT2-.646279-708040.rev.compl      atttgagcagaccttggagtcagcagcaacagcagcgtgggcaaaggcctgggagccaca   37930
FAM230B-LOC105372935-GGT2.NCBI.GGT2.rev.compl                      atttgagaagaccttggagtcagcagcaacagcggcgtgggcaaaggcc-gggggtcaaa   36618
                                                                   ******* ************************* *************** *** * ** *

chimp.LOC112206744.LOC107973052.GGT2-.646279-708040.rev.compl      tggggcctggtgtcgagagaggaccacagccagcacaatgacagccagcgccagccccag   37990
FAM230B-LOC105372935-GGT2.NCBI.GGT2.rev.compl                      tggggcctggtgtcgagagaggaccacagccagcacaatgacagccagcgccagccccag   36678
                                                                   ************************************************************

chimp.LOC112206744.LOC107973052.GGT2-.646279-708040.rev.compl      ccccagccccagcaggaccaggtcacccatggctctgcagtcctgggccatggctctgcg   38050
FAM230B-LOC105372935-GGT2.NCBI.GGT2.rev.compl                      ccccagccccagcaggaccaggtcacccatggctccgtagtcctgggccatggctctgcg   36738
                                                                   *********************************** * **********************

chimp.LOC112206744.LOC107973052.GGT2-.646279-708040.rev.compl      gcccagaaggagaggggaggccggtgggcagacggagggacagatgggtgggcagatgaa   38110
FAM230B-LOC105372935-GGT2.NCBI.GGT2.rev.compl                      gcccagaaggagaggggaggccggtgggcagatggagggacagatgggtgggcagatgaa   36798
                                                                   ******************************** ***************************

chimp.LOC112206744.LOC107973052.GGT2-.646279-708040.rev.compl      tggacaagaagatgcatagatagactcacaggtaattggacagatggacaaacaggtggg   38170
FAM230B-LOC105372935-GGT2.NCBI.GGT2.rev.compl                      tggacaagaagatgcatagatagactcacaggtaattggacagatggacaaacaggtggg   36858
                                                                   ************************************************************

chimp.LOC112206744.LOC107973052.GGT2-.646279-708040.rev.compl      ggctgaagacagacacgaagatggatcgacagacaggccagatagctagacaaagaggac   38230
FAM230B-LOC105372935-GGT2.NCBI.GGT2.rev.compl                      ggctgaagacagacacgaagatggatcgacagacaggccagatagctagacaaagaggac   36918
                                                                   ************************************************************

chimp.LOC112206744.LOC107973052.GGT2-.646279-708040.rev.compl      agtaagagaaagatggtcagatagacaatggaacagagatgggcttacagatgggcggac   38290
FAM230B-LOC105372935-GGT2.NCBI.GGT2.rev.compl                      agtaagagaaagatggtcagatagacaatgggacagagatgggcttacagatgggcggac   36978
                                                                   ******************************* ****************************

chimp.LOC112206744.LOC107973052.GGT2-.646279-708040.rev.compl      agacagacaggtctgaacagcgggctgccagatggacagatgggtgaatggacagatggc   38350
FAM230B-LOC105372935-GGT2.NCBI.GGT2.rev.compl                      agacagacaggtctgaacagcgggctgccagatggacagatgggtgaatggacagatggc   37038
                                                                   ************************************************************

chimp.LOC112206744.LOC107973052.GGT2-.646279-708040.rev.compl      tggcagctgtggcgagctgctgccctcaccaagtgcacactacggagtggccaaactcat   38410
FAM230B-LOC105372935-GGT2.NCBI.GGT2.rev.compl                      tggcagctgtggcgagctgctgccctcaccaagtgcacactacggagtggccaaactcat   37098
                                                                   ************************************************************

chimp.LOC112206744.LOC107973052.GGT2-.646279-708040.rev.compl      gcctcaacttctagttttcagctcctgcttgttcctggcaggaggccaggcagcaaagcg   38470
FAM230B-LOC105372935-GGT2.NCBI.GGT2.rev.compl                      gcctcaacttctagttttcagctcctgcttgttcctggcaggaggccaggcagcaaagcg   37158
                                                                   ************************************************************

chimp.LOC112206744.LOC107973052.GGT2-.646279-708040.rev.compl      tctgaggggagttttctttgcctagagaagtcagctgctgtgttaactccctcactgctg   38530
FAM230B-LOC105372935-GGT2.NCBI.GGT2.rev.compl                      tctgaggggagttttctttgcctagagaagtcagctgctgtgttaactccctcactgctg   37218
                                                                   ************************************************************

chimp.LOC112206744.LOC107973052.GGT2-.646279-708040.rev.compl      gtaggtccaaaggccccacctaccgcccgccagagcccatggtcacactgtcgcaatgtg   38590
FAM230B-LOC105372935-GGT2.NCBI.GGT2.rev.compl                      gtaggtccaaaggccccacctactgcccgccagagcccatggtcacactgtcgcaatgtg   37278
                                                                   *********************** ************************************

chimp.LOC112206744.LOC107973052.GGT2-.646279-708040.rev.compl      caggagaacttggtgcctgctgcactgcgattgccaggtaggggcagggctccctggaac   38650
FAM230B-LOC105372935-GGT2.NCBI.GGT2.rev.compl                      caggagaacttggtgcgtgctgcactgcggttgccaggtaggggcagggctccctggaac   37338
                                                                   **************** ************ ******************************

chimp.LOC112206744.LOC107973052.GGT2-.646279-708040.rev.compl      ctccacaccattccccacgttctcagcagctctgggaaagcagagctggggccgcttaac   38710
FAM230B-LOC105372935-GGT2.NCBI.GGT2.rev.compl                      ctccacaccattccccaggttctcagcagctctgggaaagcagagctggggccgcttaac   37398
                                                                   ***************** ******************************************

chimp.LOC112206744.LOC107973052.GGT2-.646279-708040.rev.compl      tctgtcctggatccggcaaggctgccccactcccagagtggagccctgctccccagctcc   38770
FAM230B-LOC105372935-GGT2.NCBI.GGT2.rev.compl                      tctgccctggatccggcaaggctgcccacctcccagagtggagccctgctccccagctcc   37458
                                                                   **** **********************  *******************************

chimp.LOC112206744.LOC107973052.GGT2-.646279-708040.rev.compl      catctctatcccctaaccctctccgcatggcccagcctagtcagcatcaaggtggagctg   38830
FAM230B-LOC105372935-GGT2.NCBI.GGT2.rev.compl                      catctctatcccctaaccctctccgcatggcccagcctagtcagcatcaaggtggagctg   37518
                                                                   ************************************************************

chimp.LOC112206744.LOC107973052.GGT2-.646279-708040.rev.compl      aacagaggcagaaggaggaggacccaaggtggtgtcactcaggacccgggttcaagtcct   38890
FAM230B-LOC105372935-GGT2.NCBI.GGT2.rev.compl                      aacagaggcagaaggaggaggacccaaggtggtgtcactcaggacccgggttcaagtcct   37578
                                                                   ************************************************************

chimp.LOC112206744.LOC107973052.GGT2-.646279-708040.rev.compl      tatgcttctgcagcctggcctgggtcccccaatcccccccagggtgaccaagggcttccca   38950
FAM230B-LOC105372935-GGT2.NCBI.GGT2.rev.compl                      tatgtttctgcagcctggcctgggtcccccaacccccccagggtgaccaagggcttccca   37638
                                                                   **** ************************** ****************************

chimp.LOC112206744.LOC107973052.GGT2-.646279-708040.rev.compl      gtctgcacagaggacagggggtcttgacagcatcaaatgctggtgactatgagacactta   39010
FAM230B-LOC105372935-GGT2.NCBI.GGT2.rev.compl                      gtctgcacagaggacagggggtcttgacagcatcaaatgctggtgactatgagacactta   37698
                                                                   ************************************************************

chimp.LOC112206744.LOC107973052.GGT2-.646279-708040.rev.compl      catgggaaatgcagacagaccatgcctctagcccttggtacccggcaccatccatccctg   39070
FAM230B-LOC105372935-GGT2.NCBI.GGT2.rev.compl                      catgggaaatgcagacagaccatgcctctagcccttggcaccaggcaccatccatccctg   37758
                                                                   ************************************** *** *****************

chimp.LOC112206744.LOC107973052.GGT2-.646279-708040.rev.compl      ggacttgctgtcctggaaatgcagcatggacctccagggagggggggctgtgccatgtggg   39130
FAM230B-LOC105372935-GGT2.NCBI.GGT2.rev.compl                      ggacttgctgtcctggaaatgcagcatggacctccagggagggggggctgtgccatgtggg   37818
                                                                   ************************************************************

chimp.LOC112206744.LOC107973052.GGT2-.646279-708040.rev.compl      ggcctcaccccacctgcagctctttcccaccctggctgcaggtctgcttccctgaatcca   39190
FAM230B-LOC105372935-GGT2.NCBI.GGT2.rev.compl                      ggccccaccccacctgcagctctttcccaccctggctgcaggtctgcttctctgaatcca   37878
                                                                   **** ********************************************* *********

chimp.LOC112206744.LOC107973052.GGT2-.646279-708040.rev.compl      aatccgctactactgtgctggcagcgcagcctctctggggacactggcctggctctgttc   39250
FAM230B-LOC105372935-GGT2.NCBI.GGT2.rev.compl                      aatccgctactactgtgctggcagcgcagcctctctggggacactggcctggctctgttc   37938
                                                                   ************************************************************

chimp.LOC112206744.LOC107973052.GGT2-.646279-708040.rev.compl      tccccaggcctcagggtgcctaaatgggaggcaaccaggggagtgaggacccactgaggg   39310
FAM230B-LOC105372935-GGT2.NCBI.GGT2.rev.compl                      tccccaggcctcagggtgcctaaatgggaggcaaccaggggagtgaggacccactgaggg   37998
                                                                   ************************************************************

chimp.LOC112206744.LOC107973052.GGT2-.646279-708040.rev.compl      gctccgttgaccaggctcagcaggggtgcaggtgatgtgggctggacttcttcccacgtg   39370
FAM230B-LOC105372935-GGT2.NCBI.GGT2.rev.compl                      gctccgttgaccaggctcagcaggggtgcaggtgatgtggggtggaatccttcccacatg   38058
                                                                   ***************************************** **** * ******** **

chimp.LOC112206744.LOC107973052.GGT2-.646279-708040.rev.compl      gcccccacagtcctccccgcttcctccccagctgaacactgcctgctccagatgtctaca   39430
FAM230B-LOC105372935-GGT2.NCBI.GGT2.rev.compl                      gcccccacagtcctccccgcttcctccccaactgaacactgcctgctccagatgtgtaca   38118
                                                                   ****************************** ************************ ****

chimp.LOC112206744.LOC107973052.GGT2-.646279-708040.rev.compl      cctggagtccgggcccctccatctgggcagcagagaaactgaggcacagagacagactgt   39490
FAM230B-LOC105372935-GGT2.NCBI.GGT2.rev.compl                      cctggagtctgggcccctccatctgggcagcagagaaactgaggcacagagacagactgt   38178
                                                                   ********* **************************************************

chimp.LOC112206744.LOC107973052.GGT2-.646279-708040.rev.compl      gtccttacaggccacacagcctgccaggcccctatgtccggccagagcccctggtcagcc   39550
FAM230B-LOC105372935-GGT2.NCBI.GGT2.rev.compl                      gtccttacagggcacacagcctgccaggcccctatgtccggccagagcccctggtcagcc   38238
                                                                   *********** ************************************************

chimp.LOC112206744.LOC107973052.GGT2-.646279-708040.rev.compl      tgggctgcagtgattgtttagaggtaggctgttcccacggctgcctctcacggtaggggg   39610
FAM230B-LOC105372935-GGT2.NCBI.GGT2.rev.compl                      tgggctgcagtgattgtttagaggtaggctgttcccacggctgcctctcacggtaggggg   38298
                                                                   ************************************************************

chimp.LOC112206744.LOC107973052.GGT2-.646279-708040.rev.compl      gcctgcggacgcctcctcccgtccccacccgactcccaagcctcagtgacattgctcaac   39670
FAM230B-LOC105372935-GGT2.NCBI.GGT2.rev.compl                      gcctgcggacgcctcctcccgcccccacccgactcccaagcctcagtgacattgctcaac   38358
                                                                   ******************** ***************************************

chimp.LOC112206744.LOC107973052.GGT2-.646279-708040.rev.compl      caggagctgaagtgcattcctgggctcaggccagcccacccacccacccgctgcagtcct   39730
FAM230B-LOC105372935-GGT2.NCBI.GGT2.rev.compl                      caggaactgaagtgcattcctgggctcaggccagcccacccacccacccgctgcagtcct   38418
                                                                   ***** ******************************************************

chimp.LOC112206744.LOC107973052.GGT2-.646279-708040.rev.compl      ggaagcccagaggcctgggcagcaggaacagtggagacagcagtgtgggggacgtccccc   39790
```

```
FAM230B-LOC105372935-GGT2.NCBI.GGT2.rev.compl                      ggaagcccagaggcctgggcagcaggaacagtggagacagcagtgtgggggacgtccccc   38478
                                                                   ************************************************************

chimp.LOC112206744.LOC107973052.GGT2-.646279-708040.rev.compl      ctcctctccccaccatcctcgtcaggcagaggccagggtgcagggaccacccgagcaaag   39850
FAM230B-LOC105372935-GGT2.NCBI.GGT2.rev.compl                      ctcctctccccaccatcctcgtcaggcagaggccagggtgcagggaccaccggagcaaag   38538
                                                                   *************************************************** ********

chimp.LOC112206744.LOC107973052.GGT2-.646279-708040.rev.compl      gcccagggaaatgaatgggtgtcatttggtcctgacccgaggcacagccaggaaggtcc   39910
FAM230B-LOC105372935-GGT2.NCBI.GGT2.rev.compl                      gcccagggaaatgaatgggtgtcattctggtcctgacccgaggcacagccaggaaggtcc   38598
                                                                   ************************** *********************************

chimp.LOC112206744.LOC107973052.GGT2-.646279-708040.rev.compl      ctgtggggaaaagaaacagatatcagactgttactgtgtctatgtagaaagaagtagagg   39970
FAM230B-LOC105372935-GGT2.NCBI.GGT2.rev.compl                      ctgtggggaaaagaaagagatatcagactgttactgtgtctatgtagaaagaagtagacg   38658
                                                                   **************** ***************************************** *

chimp.LOC112206744.LOC107973052.GGT2-.646279-708040.rev.compl      taagaggctccattttgttgtgtagtaagaaaaattcttttgccttgagatactgttaat   40030
FAM230B-LOC105372935-GGT2.NCBI.GGT2.rev.compl                      taagaggctccattttgttgtgtagtaagaaaaattcttttgccttgagatgccgttaat   38718
                                                                   *************************************************** * ******

chimp.LOC112206744.LOC107973052.GGT2-.646279-708040.rev.compl      ctgtaaccctagccccaaccctgtgctcacagaaacatgtactgtgtcgactcaaggttt   40090
FAM230B-LOC105372935-GGT2.NCBI.GGT2.rev.compl                      ctgtaaccctagccccaaccctgtgctcacagaaacatgtactgtgtcgactcaaggttt   38778
                                                                   ************************************************************

chimp.LOC112206744.LOC107973052.GGT2-.646279-708040.rev.compl      aatggatttagggctgtgcaggatgtgctttgttaaacaaatgcttgaaggcagcatgct   40150
FAM230B-LOC105372935-GGT2.NCBI.GGT2.rev.compl                      aatggattcagggctgtgcaggatgtgctttgttaaacaaatgcttgaaggcagcatgct   38838
                                                                   ******** ***************************************************

chimp.LOC112206744.LOC107973052.GGT2-.646279-708040.rev.compl      tgttaaaagtcatcaccactccctaatctcaagtaagcagggacacaaaacactgcagaa   40210
FAM230B-LOC105372935-GGT2.NCBI.GGT2.rev.compl                      tgttaagagtcatcaccactccctaatctcaagtaagcagggacacaaaacactgcagaa   38898
                                                                   ****** *****************************************************

chimp.LOC112206744.LOC107973052.GGT2-.646279-708040.rev.compl      ggccgcagggacctctgcctaggaaagccaggtattgtccaaggtttctccccatgtgac   40270
FAM230B-LOC105372935-GGT2.NCBI.GGT2.rev.compl                      ggccgcagggacctctgcctaggaaagccaggtatcgtccaaggtttctccccaggtgac   38958
                                                                   ********************************** ******************* *****

chimp.LOC112206744.LOC107973052.GGT2-.646279-708040.rev.compl      agtctgaaatatggcctcgtgggaagggaaagacctgaccgtcccccagcccgacacctg   40330
FAM230B-LOC105372935-GGT2.NCBI.GGT2.rev.compl                      agtctgaaatatggcctcgtgggaagggaaagacctgaccgtcccccagcccgacacctg   39018
                                                                   ************************************************************

chimp.LOC112206744.LOC107973052.GGT2-.646279-708040.rev.compl      taaagggtctgtgctgaggaggattagtaaaaggggaaggcctctttgcagttgagataa   40390
FAM230B-LOC105372935-GGT2.NCBI.GGT2.rev.compl                      taaagggtctgtgctgaggaggattagtaaaagaggaaggcctctttgcagttgagataa   39078
                                                                   ********************************* **************************

chimp.LOC112206744.LOC107973052.GGT2-.646279-708040.rev.compl      gaggaaggcatctctctcctgatcgtccctgggcaaaggaatgtctcagtgttgatggta   40450
FAM230B-LOC105372935-GGT2.NCBI.GGT2.rev.compl                      gaggaaggcatctctctcctgatcgtccctgggcaaaggaatgtctcagtgttgattgta   39138
                                                                   ******************************************************** ***

chimp.LOC112206744.LOC107973052.GGT2-.646279-708040.rev.compl      tattccatctactgagataggagaaaactgccttagggctggaggtgggacatgctggtg   40510
FAM230B-LOC105372935-GGT2.NCBI.GGT2.rev.compl                      tattccatctgctgagataggagaaaactgccttagggctggagatgggacatgctggtg   39198
                                                                   ********** ********************************* ***************

chimp.LOC112206744.LOC107973052.GGT2-.646279-708040.rev.compl      gcaatactgctatttaatgcattgagatgtttatgtatatgcacatcaaagcacagcacc   40570
FAM230B-LOC105372935-GGT2.NCBI.GGT2.rev.compl                      gcaatactgctctttaatgcattgagatgtttatgtatatgcacatcaaagcatagcacc   39258
                                                                   *********** ***************************************** ******

chimp.LOC112206744.LOC107973052.GGT2-.646279-708040.rev.compl      tttttcttaaccttgtttatgacacagagacatttgttcacgtgttttcctgctgaccct   40630
FAM230B-LOC105372935-GGT2.NCBI.GGT2.rev.compl                      tttttcttaaccttgtttatgacacagagacatttgttcacgtgttttcctgctgaccct   39318
                                                                   ************************************************************

chimp.LOC112206744.LOC107973052.GGT2-.646279-708040.rev.compl      ctccccactattaccctattgtcctgccacatcccccctctctgagatggtagagataatg   40690
FAM230B-LOC105372935-GGT2.NCBI.GGT2.rev.compl                      ctccccactattaccctattgtcctgccacatcccccctctctgagatggtagagataatg   39378
                                                                   ************************************************************

chimp.LOC112206744.LOC107973052.GGT2-.646279-708040.rev.compl      atcaataaatactagggaactcagagactggtgccagcgtgggtcctccatatgctgagt   40750
FAM230B-LOC105372935-GGT2.NCBI.GGT2.rev.compl                      atcaataaatactagggaactcagagactggtgccagcgtggggcctccgtatgctgagc   39438
                                                                   ******************************************* ***** *********

chimp.LOC112206744.LOC107973052.GGT2-.646279-708040.rev.compl      gcaggtcccctgggcccacttttctttctctatactttgtctctgtgtctctttcttttc   40810
FAM230B-LOC105372935-GGT2.NCBI.GGT2.rev.compl                      gcaggtcccctgggcccacttttctttctctatactttgtctctgtgtctctttcttttc   39498
                                                                   ************************************************************

chimp.LOC112206744.LOC107973052.GGT2-.646279-708040.rev.compl      tcagtctcttgtcccacctgatgagaaacacccacagatgtggaggggcaggccacccct   40870
FAM230B-LOC105372935-GGT2.NCBI.GGT2.rev.compl                      tcagtctcttgtcccagctgatgggaaacacccacaggtgtggaggggcaggccacccct   39558
                                                                   **************** ****** ************* **********************

chimp.LOC112206744.LOC107973052.GGT2-.646279-708040.rev.compl      tcaggtccctgaatgtccttcctcaggaaatgatgggggaaggggtgatgagaatgaagg   40930
FAM230B-LOC105372935-GGT2.NCBI.GGT2.rev.compl                      tcaggtccctgaatgtccttcctcaggaaatgatgggggaaggggtgatgagaatgaagg   39618
                                                                   ************************************************************

chimp.LOC112206744.LOC107973052.GGT2-.646279-708040.rev.compl      agaggatttaagtccctcacccccccgaggtagtcctgggctgagccccatgggacctgga   40990
FAM230B-LOC105372935-GGT2.NCBI.GGT2.rev.compl                      agacgatttaagtccctcacccccccgaggtagtcctgggctgagccccatgggacctaga   39678
                                                                   *** ***************************************************** **

chimp.LOC112206744.LOC107973052.GGT2-.646279-708040.rev.compl      gaaccagggtgtaccccaccagcgtgtcgggtccaggaagcctcgtggccagctcccact   41050
FAM230B-LOC105372935-GGT2.NCBI.GGT2.rev.compl                      gaaccagggtgtaccccaccagcgtgtcgggtccaggaagcctcgtggccagctcccact   39738
                                                                   ************************************************************

chimp.LOC112206744.LOC107973052.GGT2-.646279-708040.rev.compl      tctcttgctgctgtgcaacccagagcaaggcctgcccctccagcttcagtcttctcccct   41110
FAM230B-LOC105372935-GGT2.NCBI.GGT2.rev.compl                      tctcttcctgctgtgcaacccagagcaaggcctgcccctccagcttcagtcttctcccct   39798
                                                                   ****** *****************************************************

chimp.LOC112206744.LOC107973052.GGT2-.646279-708040.rev.compl      gcaaatggggccacggcctttcctctcaggccaaaataaggattgaggccgggtgcagtg   41170
FAM230B-LOC105372935-GGT2.NCBI.GGT2.rev.compl                      gcaaatggggccacggcctttcctctcaggccaatataaggattgaggccgggtgcagtg   39858
                                                                   ********************************** *************************

chimp.LOC112206744.LOC107973052.GGT2-.646279-708040.rev.compl      gctcacccctgtaatcctagcactttgggagactgagatgggggggactgcttgaagtcag   41230
FAM230B-LOC105372935-GGT2.NCBI.GGT2.rev.compl                      gct-acccctgtaatcctagcactttgggagactgagatgggggggactgcttgaagtcag   39917
                                                                   *** ********************************************************

chimp.LOC112206744.LOC107973052.GGT2-.646279-708040.rev.compl      gagttaagaccagcctggtcaacatagtgagaccccatctctattggtttaaattttgtt   41290
FAM230B-LOC105372935-GGT2.NCBI.GGT2.rev.compl                      gagttaagaccagcctggtcaacatagtgagaccccatctctattggtttaaattttttt   39977
                                                                   ********************************************************* **

chimp.LOC112206744.LOC107973052.GGT2-.646279-708040.rev.compl      t--tttaattaaataaataaaataaggattgaagagtgacttgtacaccagttgagccca   41348
FAM230B-LOC105372935-GGT2.NCBI.GGT2.rev.compl                      taaaaaaattaaataaataaaataaggattgaagagtgacttgtacaccagttgagccca   40037
                                                                   *     ******************************************************

chimp.LOC112206744.LOC107973052.GGT2-.646279-708040.rev.compl      cctccatctcacccttgcagagccccagagacacagccctccagggctcagacccagtgg   41408
FAM230B-LOC105372935-GGT2.NCBI.GGT2.rev.compl                      cctccatctcacccttgcagagccccagagacacagccctccagagctcagacccagtgg   40097
                                                                   ******************************************** ***************

chimp.LOC112206744.LOC107973052.GGT2-.646279-708040.rev.compl      gacttgactccacaggcataaaaccctgtttgtctatgggccctttggaatcaccaggtt   41468
FAM230B-LOC105372935-GGT2.NCBI.GGT2.rev.compl                      gacttgactccacaggcataaaaccctgtttgtctatgggccctttggaatcaccaggtt   40157
                                                                   ************************************************************

chimp.LOC112206744.LOC107973052.GGT2-.646279-708040.rev.compl      ttcggggatcctgaaggatagccccgacctggcctcacctggcccctggccccagtgccc   41528
FAM230B-LOC105372935-GGT2.NCBI.GGT2.rev.compl                      ttcggggctcctgaaggatagccccgacctggcctcacctggcccctggccccagtgccc   40217
                                                                   ******* ****************************************************

chimp.LOC112206744.LOC107973052.GGT2-.646279-708040.rev.compl      ctggtgatatccaggtgc------------------------------------------   41546
FAM230B-LOC105372935-GGT2.NCBI.GGT2.rev.compl                      ctggtgatatccaggtgctgggctgtgatcaccgcctcccaccagcccacctccaccagc   40277
                                                                   ******************

chimp.LOC112206744.LOC107973052.GGT2-.646279-708040.rev.compl      --------------------------tgggctgtgatctccgcctcccaccagcccacct   41580
FAM230B-LOC105372935-GGT2.NCBI.GGT2.rev.compl                      ccttcccagaaccctgctccaggtgttgggctgtgatcaccgcctcccaccagcccacct   40337
                                                                                             ************ *********************

chimp.LOC112206744.LOC107973052.GGT2-.646279-708040.rev.compl      ccaccagcccttcccagaaccctgccccaggtgttggaactgtgcacagaggagggagca   41640
FAM230B-LOC105372935-GGT2.NCBI.GGT2.rev.compl                      ccaccagcccttcccagaaccctgctccaggtgttggaactgtgcacagaggagggagca   40397
                                                                   ************************* **********************************

chimp.LOC112206744.LOC107973052.GGT2-.646279-708040.rev.compl      ggccctgagggaggcctggaggggctgcggatggtgaaggctgctgtgtctagctgtttc   41700
FAM230B-LOC105372935-GGT2.NCBI.GGT2.rev.compl                      ggccccgagggaggcctggaggggctgccaatggtgaaggctgctgtgtcgagctgtttc   40457
                                                                   ***** **********************  ******************** *********

chimp.LOC112206744.LOC107973052.GGT2-.646279-708040.rev.compl      cttctggacccactccctctgggctgcgtccccggctggtccaagccctgatccctggga   41760
FAM230B-LOC105372935-GGT2.NCBI.GGT2.rev.compl                      cttccggacccactccctctgggctgcgtccccggctggtccaagccctgatccctggga   40517
                                                                   **** *******************************************************

chimp.LOC112206744.LOC107973052.GGT2-.646279-708040.rev.compl      tctggggacatcttcccgtttgttgttccctgagaaccaggcctccctctggagaggatc   41820
FAM230B-LOC105372935-GGT2.NCBI.GGT2.rev.compl                      tctggggacatcttcccgtttgctgttccctgagaaccaggcctccctctggagaggatc   40577
                                                                   ********************** *************************************

chimp.LOC112206744.LOC107973052.GGT2-.646279-708040.rev.compl      acaagcttgggtttcactctgggcttgctcttgggaacccccagggggcgtggctctgac   41880
FAM230B-LOC105372935-GGT2.NCBI.GGT2.rev.compl                      acaagcttgggtttcactctgggcttgctcttgggaacccccagggggcatggctctgac   40637
```

```
                                                                  ************************************************* **********

chimp.LOC112206744.LOC107973052.GGT2-.646279-708040.rev.compl     cgagatgttttcctccagcctgttgcctagtccccattcctcgga-cctcagcttcacct   41939
FAM230B-LOC105372935-GGT2.NCBI.GGT2.rev.compl                     cgagatgttttcctccagcctgttgcccagtccccattcctcggaccctcagcttcacct   40697
                                                                  *************************** ***************** **************

chimp.LOC112206744.LOC107973052.GGT2-.646279-708040.rev.compl     ccagtgtcatcggcagggtgagctggacgcctacgggtctgagaaggcacccgggttccc   41999
FAM230B-LOC105372935-GGT2.NCBI.GGT2.rev.compl                     ccagtgtcatcggcagggtgagctggacgcctacgggtctgagaaggcgcccgggttccc   40757
                                                                  ************************************************ ***********

chimp.LOC112206744.LOC107973052.GGT2-.646279-708040.rev.compl     agcatcagctggccacc-------------------------------------------   42016
FAM230B-LOC105372935-GGT2.NCBI.GGT2.rev.compl                     agcatcggctggccaccctctgcctaagaaagcgccagggtcgtgacacccccctggtggc   40817
                                                                  ****** **********

chimp.LOC112206744.LOC107973052.GGT2-.646279-708040.rev.compl     ------------------------------------------------------------   42016
FAM230B-LOC105372935-GGT2.NCBI.GGT2.rev.compl                     tgatcctaggtagtgtcactgcccagccccagtaagggagggcctggccccaaagtctga   40877

chimp.LOC112206744.LOC107973052.GGT2-.646279-708040.rev.compl     ------------------------------------------------------------   42016
FAM230B-LOC105372935-GGT2.NCBI.GGT2.rev.compl                     gggatcagggtgggaaggggcagggtttggtgtgaaccttcccctggcccccagccatgt   40937

chimp.LOC112206744.LOC107973052.GGT2-.646279-708040.rev.compl     ------------------------------------------------------------   42016
FAM230B-LOC105372935-GGT2.NCBI.GGT2.rev.compl                     gccttgctctccccatgctgaagatgctgaggctagttccagtgtccgcattgtgaagat   40997

chimp.LOC112206744.LOC107973052.GGT2-.646279-708040.rev.compl     ---------------ctctgttcctccccagccagatggctccatttcacacacaataca   42061
FAM230B-LOC105372935-GGT2.NCBI.GGT2.rev.compl                     ctccgaatcccacctctctgttcctccccagccagatggctccatttcacacacaataca   41057
                                                                                 *********************************************

chimp.LOC112206744.LOC107973052.GGT2-.646279-708040.rev.compl     ctgaggcccagagagtggggagacaggccagggaggccacctggagcctggcacagtggc   42121
FAM230B-LOC105372935-GGT2.NCBI.GGT2.rev.compl                     ctgaggcccagagagtggggagacaggccagggaggccacctggagcctggcacagtggc   41117
                                                                  ************************************************************

chimp.LOC112206744.LOC107973052.GGT2-.646279-708040.rev.compl     ctcatttattatgctgctctgctgctcacaggggaagcccgtcccccaaagtcctcttcc   42181
FAM230B-LOC105372935-GGT2.NCBI.GGT2.rev.compl                     ctcatttattatgctgctctgctgctcacaggggaagcccgtcccccaaagtcctcttcc   41177
                                                                  ************************************************************

chimp.LOC112206744.LOC107973052.GGT2-.646279-708040.rev.compl     tcatcctggtgagtatcttgtccctggattgcttgtcagccttgtctgcctggagcaatc   42241
FAM230B-LOC105372935-GGT2.NCBI.GGT2.rev.compl                     tcatcctggtgagtatcttgtccctggattgcttgtcagccttgtctgcctggagcaatc   41237
                                                                  ************************************************************

chimp.LOC112206744.LOC107973052.GGT2-.646279-708040.rev.compl     agtagccagcaggttccccgcctttcctggagtccgaggcagctgcccagccaccagcca   42301
FAM230B-LOC105372935-GGT2.NCBI.GGT2.rev.compl                     agtagccagcaggttccccgcctttcctggagtccgaggcagctgcccagccaccagccg   41297
                                                                  ***********************************************************

chimp.LOC112206744.LOC107973052.GGT2-.646279-708040.rev.compl     tgcggacgatggcttgcaccacagcgatgaaggtggacgcgatctgggtgtgatggtgcc   42361
FAM230B-LOC105372935-GGT2.NCBI.GGT2.rev.compl                     tgcggacgatggcttgcaccacagcgatgaaggtggacgcgatctgggtgtgatggtgcc   41357
                                                                  ************************************************************

chimp.LOC112206744.LOC107973052.GGT2-.646279-708040.rev.compl     gggtctccagggctgcagtcactgcctggggggtgggaggagaggggaagcctgagcaggg   42421
FAM230B-LOC105372935-GGT2.NCBI.GGT2.rev.compl                     gggtctccagggctgcagtcactgcctggggggtgggaggagaggggaagcctgagcaggg   41417
                                                                  ************************************************************

chimp.LOC112206744.LOC107973052.GGT2-.646279-708040.rev.compl     ctccagatgccacctgaaccacgcctgtgtggtcacaggcctcagcccaggtggtgccat   42481
FAM230B-LOC105372935-GGT2.NCBI.GGT2.rev.compl                     ctccagatgccacctgaaccacacctgtgtggtcacaggcctcagcccaggtggtgccat   41477
                                                                  ********************** *************************************

chimp.LOC112206744.LOC107973052.GGT2-.646279-708040.rev.compl     ttcaggccaggtcatcaggaagagcaggttggggcctgctgggtctcactggagcagggg   42541
FAM230B-LOC105372935-GGT2.NCBI.GGT2.rev.compl                     ttcaggccaggtcatcaggaagagcaggttggggcctgctgggtctcactggagcagggg   41537
                                                                  ************************************************************

chimp.LOC112206744.LOC107973052.GGT2-.646279-708040.rev.compl     gcttggccctcatggcacaggggctccagatggcccaggcactagagagaggacaccaac   42601
FAM230B-LOC105372935-GGT2.NCBI.GGT2.rev.compl                     gcttggctctcatgtcacaggggctccagatggcccaggcactagagagaggacaccaac   41597
                                                                  ******* ****** *********************************************

chimp.LOC112206744.LOC107973052.GGT2-.646279-708040.rev.compl     cattgtccactctgtgatgatccaggcctccagcccaggatgccctggggccccacaccg   42661
FAM230B-LOC105372935-GGT2.NCBI.GGT2.rev.compl                     cattgtccactctgtgatgatccaggcctccagcccaggatgccctgggaccccacaccg   41657
                                                                  ************************************************* **********

chimp.LOC112206744.LOC107973052.GGT2-.646279-708040.rev.compl     tgactcagtttctccaacccccggcccacctggtcaatgtttctctccactgtcgtgacg   42721
FAM230B-LOC105372935-GGT2.NCBI.GGT2.rev.compl                     tgactcagtttctccaacccctggcccacctggtcaatgtttctctccactgtcgtgacg   41717
                                                                  ********************* **************************************

chimp.LOC112206744.LOC107973052.GGT2-.646279-708040.rev.compl     ttgggcagaagcttgttgtgcagccggggctcctccacggccttcttcacgtcatagccg   42781
FAM230B-LOC105372935-GGT2.NCBI.GGT2.rev.compl                     ttgggcagaagcttgttgtgcagccggggctcctccacggccctcttcacgtcatagccg   41777
                                                                  ****************************************** *****************

chimp.LOC112206744.LOC107973052.GGT2-.646279-708040.rev.compl     aaccagaggttgtagatgatggcctggggcatgggagtgtgatcagcgtggcttgggggc   42841
FAM230B-LOC105372935-GGT2.NCBI.GGT2.rev.compl                     aaccagaggttgtagatgatggcctggggcatgggagtgtgatcagggtggcttgggggc   41837
                                                                  ********************************************** *************

chimp.LOC112206744.LOC107973052.GGT2-.646279-708040.rev.compl     tgtgcagagtgggcagggccagggagaaaaggggtgacacataccagtgcagtgtctgtg   42901
FAM230B-LOC105372935-GGT2.NCBI.GGT2.rev.compl                     tgtgcagagtgggcagggccagggagaaaaggggtgacacataccagtgcagtgtctgtg   41897
                                                                  ************************************************************

chimp.LOC112206744.LOC107973052.GGT2-.646279-708040.rev.compl     gtgatctgcgtgcccccagcagctcccaccaccaaccggacctggccgtcctggcccacc   42961
FAM230B-LOC105372935-GGT2.NCBI.GGT2.rev.compl                     gtgatctgcgtgcccccagcagctcccaccaccatccggacctggccgtcctggcccacc   41957
                                                                  ********************************** *************************

chimp.LOC112206744.LOC107973052.GGT2-.646279-708040.rev.compl     atgatcgtcgggcacatggacgagagcggctgcttccctgcggctgatgggagaagacag   43021
FAM230B-LOC105372935-GGT2.NCBI.GGT2.rev.compl                     atgatcgtcaggcacatggacaagagcggctgcttccctgcggccgatgggagaagacag   42017
                                                                  ********* *********** *********************** ***************

chimp.LOC112206744.LOC107973052.GGT2-.646279-708040.rev.compl     ggatgcccgtcagctgcctgcccaggacacccgcccctctccaccccagtccccca-ccc   43080
FAM230B-LOC105372935-GGT2.NCBI.GGT2.rev.compl                     ggatgcccgtcagctgcctgcccaggacacccgcccctctccaccccagtcccccacccc   42077
                                                                  ******************************************************** ***

chimp.LOC112206744.LOC107973052.GGT2-.646279-708040.rev.compl     ccagacctccaccccatacctggctggatgaaattggcaggtgaggggggcaccccaaac   43140
FAM230B-LOC105372935-GGT2.NCBI.GGT2.rev.compl                     ccggacctccaccccatacctggctggatgaaattggcaggtgaggggggtgccccaaac   42137
                                                                  ** ***********************************************  ********

chimp.LOC112206744.LOC107973052.GGT2-.646279-708040.rev.compl     tcattggtg-atgctgggagagctgaagtcgtccattcattattgaacaggatcccactg   43199
FAM230B-LOC105372935-GGT2.NCBI.GGT2.rev.compl                     tcattggtgaatgctgggagagctgaagtcgtccattcattattgaacaggatcccactg   42197
                                                                  ********* **************************************************

chimp.LOC112206744.LOC107973052.GGT2-.646279-708040.rev.compl     accggggagcagaccttggagccaaagctaccgcccagccaggtcagacagcacctgacc   43259
FAM230B-LOC105372935-GGT2.NCBI.GGT2.rev.compl                     accggggagcagaccttggagccaaagctaccgcccagccaggtcagacagcacccgacc   42257
                                                                  ******************************************************* ****

chimp.LOC112206744.LOC107973052.GGT2-.646279-708040.rev.compl     ttgcctggcccagcctggtccctatccacccactgaggctcaaacatactcactgagagg   43319
FAM230B-LOC105372935-GGT2.NCBI.GGT2.rev.compl                     ttgcctggcccagcctggtccctatccacccactgaggctcaaacatactcactgagagg   42317
                                                                  ************************************************************

chimp.LOC112206744.LOC107973052.GGT2-.646279-708040.rev.compl     cccaggataagctaccaaggttgggcctcagtttcccaccaggaaaagaggtgatggagc   43379
FAM230B-LOC105372935-GGT2.NCBI.GGT2.rev.compl                     cccaggataagctaccaaggttgggcctcagtttcccaccaggaaaagaggtgatggagc   42377
                                                                  ************************************************************

chimp.LOC112206744.LOC107973052.GGT2-.646279-708040.rev.compl     caccttactggataagtgggcagtccctgggccacccgcccctggccctttcccacccag   43439
FAM230B-LOC105372935-GGT2.NCBI.GGT2.rev.compl                     caccttactggataagtgggcagtccctgggccacccgcccctggccctttcccacccag   42437
                                                                  ************************************************************

chimp.LOC112206744.LOC107973052.GGT2-.646279-708040.rev.compl     gcggcccagcagcccctactagaggttgatggtgctggtggccgacacagcactgccgtc   43499
FAM230B-LOC105372935-GGT2.NCBI.GGT2.rev.compl                     gcggcccagcagcccctactagaggttgatggtgctggtggcggacacagcactgccgtc   42497
                                                                  ****************************************** *****************

chimp.LOC112206744.LOC107973052.GGT2-.646279-708040.rev.compl     ctctgcgacgacagacaggtgagcagtgcccccgtcatccggcgtgtagaactcgggctt   43559
FAM230B-LOC105372935-GGT2.NCBI.GGT2.rev.compl                     ctctgcgacgacagacaggtgagcagtgcccccgtcatccggcgtgtagaactcgggctt   42557
                                                                  ************************************************************

chimp.LOC112206744.LOC107973052.GGT2-.646279-708040.rev.compl     gtagtaggagatcgggtgagtggtgtggtcagagatctgggcctggagctgggcagcgaa   43619
FAM230B-LOC105372935-GGT2.NCBI.GGT2.rev.compl                     gtagtaggagatcgggtgagtggtgtggtcagagatctgggaccggagctgggcagcgaa   42617
                                                                  ***************************************** * ****************

chimp.LOC112206744.LOC107973052.GGT2-.646279-708040.rev.compl     gaactcggaggtcatgttgcggaccacctgccgagaccccagagctggcctgaggaggtg   43679
FAM230B-LOC105372935-GGT2.NCBI.GGT2.rev.compl                     gaactcagaggtcatgttgcggaccacctgctgagaccccagagctggcctgaggaggtg   42677
                                                                  ****** ************************ ****************************

chimp.LOC112206744.LOC107973052.GGT2-.646279-708040.rev.compl     gggagggagggtggggagggggcacaggtctcagaaggcccctgactgtgactctgaccg   43739
FAM230B-LOC105372935-GGT2.NCBI.GGT2.rev.compl                     gggagggagggtggggagggggcacaggtctcagaaggcccttgactgtgactctgaccg   42737
                                                                  ***************************************** ******************

chimp.LOC112206744.LOC107973052.GGT2-.646279-708040.rev.compl     caaccctctggcacccacaaccttccgtggctccccaggacccaagggcaggcccaggac   43799
FAM230B-LOC105372935-GGT2.NCBI.GGT2.rev.compl                     caaccctctggcacccacaaccttccgtggctccccaggacccaagggcaggcccaagac   42797
                                                                  ******************************************************** ***
```

```
chimp.LOC112206744.LOC107973052.GGT2-.646279-708040.rev.compl      cttgcacaaccagtctgactccctgtctctgtcgcgttccagcaactctgaatgtctgtc   43859
FAM230B-LOC105372935-GGT2.NCBI.GGT2.rev.compl                      cttgcatgaccagtctgactccctgtctctgtcgcgttccagcaactctgaatgtctgtc   42857
                                                                   ******  ****************************************************

chimp.LOC112206744.LOC107973052.GGT2-.646279-708040.rev.compl      tgcgtggtcctcagcctccagacccttgccacattcaatcactcattctttcatgcaaaa   43919
FAM230B-LOC105372935-GGT2.NCBI.GGT2.rev.compl                      tgcctggtcctcagcctccagacccttgccgcattcaatcactcattccttcatgcaaaa   42917
                                                                   *** ************************** ***************** ***********

chimp.LOC112206744.LOC107973052.GGT2-.646279-708040.rev.compl      aatatttctagaatttgcactgcatgcctggcactggggaatcaacagggaacagacatt   43979
FAM230B-LOC105372935-GGT2.NCBI.GGT2.rev.compl                      aatatttctagaatttgcactgcatgcctggcactggggaatcaacagggaacagacact   42977
                                                                   ********************************************************** *

chimp.LOC112206744.LOC107973052.GGT2-.646279-708040.rev.compl      taggtcctgccctcatgccaagaaaaacaaacacacacagggaaagtgctgaaaccacaa   44039
FAM230B-LOC105372935-GGT2.NCBI.GGT2.rev.compl                      taagtcctgccctcatgccaagaaaaacaaacacacccagggaaagtgctgaaaccacag   43037
                                                                   ** ********************************* ********************** 

chimp.LOC112206744.LOC107973052.GGT2-.646279-708040.rev.compl      gccaggtaaggggaatcaagaggcatgaggtatgggcagagtggtcagggagggcttctc   44099
FAM230B-LOC105372935-GGT2.NCBI.GGT2.rev.compl                      gccaggtaaggggaatcaagaggcatgaggtatgggcagagtggtcagggagggcttctc   43097
                                                                   ************************************************************

chimp.LOC112206744.LOC107973052.GGT2-.646279-708040.rev.compl      agaggaggcagcgtgtgaaaagagcctggaatgtggcctaaatggtcagtgcaaaggccc   44159
FAM230B-LOC105372935-GGT2.NCBI.GGT2.rev.compl                      agaggaggcaacgtgtgaaaagagcctggaatgtggcctaaatggtcagtgcaaaggccc   43157
                                                                   ********** *************************************************

chimp.LOC112206744.LOC107973052.GGT2-.646279-708040.rev.compl      tgaggcaggtggtataggctggtgagcgataggcagagagtgaatggagtaggatgggga   44219
FAM230B-LOC105372935-GGT2.NCBI.GGT2.rev.compl                      tgaggcaggtggtataggctggtgagcgataggcagagagtgaatggagtagggtgggga   43217
                                                                   ***************************************************** ******

chimp.LOC112206744.LOC107973052.GGT2-.646279-708040.rev.compl      gaagaggatgaagatgcaggctggggcccatcccacaggacctcctaggtcccataacaa   44279
FAM230B-LOC105372935-GGT2.NCBI.GGT2.rev.compl                      gaagaggatgaagatgcaggctggggcccatcccacaggacctcctaggtcccataacaa   43277
                                                                   ************************************************************

chimp.LOC112206744.LOC107973052.GGT2-.646279-708040.rev.compl      ctggcttttgctctgtgccatgcaggcttagggcagaggaatgaggagggtggggagtgt   44339
FAM230B-LOC105372935-GGT2.NCBI.GGT2.rev.compl                      ctggcttttttctctgtgccatgcaggcttagggcagaggaatgaggaggctggggagtgt   43337
                                                                   ********* *************************************** **********

chimp.LOC112206744.LOC107973052.GGT2-.646279-708040.rev.compl      tttcacagggtccctctggcagctatgacggggataaggataaagcccaaaggggaggct   44399
FAM230B-LOC105372935-GGT2.NCBI.GGT2.rev.compl                      tttcacagggtccctctggcagctatgacggggataaggataaagcccaaaggggaggct   43397
                                                                   ************************************************************

chimp.LOC112206744.LOC107973052.GGT2-.646279-708040.rev.compl      gtgggtatcaaccaggcaagagatgatggcctgggtgggagaaagagaagaatcaaggat   44459
FAM230B-LOC105372935-GGT2.NCBI.GGT2.rev.compl                      gtgggtatcaaccaggcaagagatgatggcctgggtgggagaaagagaagaatcaaggat   43457
                                                                   ************************************************************

chimp.LOC112206744.LOC107973052.GGT2-.646279-708040.rev.compl      ggtgccgactagcgaggtaaaccctgcagaaggggcaggtttggggatggtcaggagctt   44519
FAM230B-LOC105372935-GGT2.NCBI.GGT2.rev.compl                      ggtgccgactagcgagg-----ccggcagaaggggccggtttggggatggtcaggagctt   43512
                                                                   *****************     ** *********** ***********************

chimp.LOC112206744.LOC107973052.GGT2-.646279-708040.rev.compl      gattttggatacttcatcagacctgaagagcatgggtgcacgtataaaaaaaataaataa   44579
FAM230B-LOC105372935-GGT2.NCBI.GGT2.rev.compl                      gattttggatacttcatcagacccaaagagcatgggtgcacgtataaaaaaaataaataa   43572
                                                                   ***********************  ***********************************

chimp.LOC112206744.LOC107973052.GGT2-.646279-708040.rev.compl      ataagcatgggttcacgggcaagggcgggctgagagatgaacatggaggtattgacattg   44639
FAM230B-LOC105372935-GGT2.NCBI.GGT2.rev.compl                      ataagcatgggttcacgggcaagggcgggctgagagatgaacatggaggtattgacattg   43632
                                                                   ************************************************************

chimp.LOC112206744.LOC107973052.GGT2-.646279-708040.rev.compl      agtggctgctggatgccatgagcctggccaaggtccccaaggcagtggcgaggaggagat   44699
FAM230B-LOC105372935-GGT2.NCBI.GGT2.rev.compl                      agtggctgctggatgccatgagcctggccaaggtccccaaggcagtggcgaggaggagat   43692
                                                                   ************************************************************

chimp.LOC112206744.LOC107973052.GGT2-.646279-708040.rev.compl      gaggaggtcaaggaggagac--agaggatggacccgaaggccaaagaaaatgcctcaaga   44757
FAM230B-LOC105372935-GGT2.NCBI.GGT2.rev.compl                      gaggaggtcaaagaggagacagagaggatggacccgaaggccgaagaaaatgcctcaaga   43752
                                                                   *********** ********  ******************** *****************

chimp.LOC112206744.LOC107973052.GGT2-.646279-708040.rev.compl      gagtttcaacaccgggcgcggtggctcacgcctgtaatcccagcactttgggaggccgag   44817
FAM230B-LOC105372935-GGT2.NCBI.GGT2.rev.compl                      gagtttcaacaccgggcgcggtggctcacgcctgtaatcccagcactttgggaggccgag   43812
                                                                   ************************************************************

chimp.LOC112206744.LOC107973052.GGT2-.646279-708040.rev.compl      gcctgtaatcccagcactttgggagggcggatcacgacgtcaggagatcaagaccatcct   44877
FAM230B-LOC105372935-GGT2.NCBI.GGT2.rev.compl                      gcctgtaatcccagcactttgggagggcggatcatgacgtcaggagatcgagaccatcct   43872
                                                                   ********************************** ************** **********

chimp.LOC112206744.LOC107973052.GGT2-.646279-708040.rev.compl      ggctaacacagtgaaaccccgtctctactaaaaatacaaaaaattagctgggcgcggtgg   44937
FAM230B-LOC105372935-GGT2.NCBI.GGT2.rev.compl                      ggctaacacagtgaaaccccgtctctactaaaaatacaaaaaattagctgggcgcggtgg   43932
                                                                   ************************************************************

chimp.LOC112206744.LOC107973052.GGT2-.646279-708040.rev.compl      tgggcacctgtagtcccagctacttgggggggctgaggcaggagaatggcatgaacctgg   44997
FAM230B-LOC105372935-GGT2.NCBI.GGT2.rev.compl                      tgggcacctgtagtcccagctactt-ggggggctgaggcaggagaatggcgtgaacctgg   43991
                                                                   ************************* ************************ *********

chimp.LOC112206744.LOC107973052.GGT2-.646279-708040.rev.compl      gaggcggagcttgcagtgagccgagatcccgccactgcactccagcctgggggacagcct   45057
FAM230B-LOC105372935-GGT2.NCBI.GGT2.rev.compl                      gaggcggagcttgcagtgagcggagatcctgccactgcactccagcctgggggacagcct   44051
                                                                   ********************* ******* ******************************

chimp.LOC112206744.LOC107973052.GGT2-.646279-708040.rev.compl      gggggacagagcgagactccgtctcaaaaataaaataaaataaaataaaataaagtttca   45117
FAM230B-LOC105372935-GGT2.NCBI.GGT2.rev.compl                      gggggacagagcgagactccgtctcaaaaataaaataaaataaaataaaataaagtttca   44111
                                                                   ************************************************************

chimp.LOC112206744.LOC107973052.GGT2-.646279-708040.rev.compl      gcaacaccccacggattagttgaccaatcccagggaaaggtgttctgtctgaatctgccc   45177
FAM230B-LOC105372935-GGT2.NCBI.GGT2.rev.compl                      gcaacaccccacggattagttgaccaatcccagggaaaggtgttctgtctgaatctgccc   44171
                                                                   ************************************************************

chimp.LOC112206744.LOC107973052.GGT2-.646279-708040.rev.compl      tcaaggaaacagaaaggcaaatccacgatgtgggacattttccaagactactgccctggg   45237
FAM230B-LOC105372935-GGT2.NCBI.GGT2.rev.compl                      tcaaggaaacagaaaggcaaatccacgatgtgggacattttccaagactactgccctggg   44231
                                                                   ************************************************************

chimp.LOC112206744.LOC107973052.GGT2-.646279-708040.rev.compl      ctttaaaaatcaacaaaacaggccaggcacggtggctcatgcctgtaatcccagcacttt   45297
FAM230B-LOC105372935-GGT2.NCBI.GGT2.rev.compl                      ctttaaaaatcaacaaaacaggccaggcacggtggttcatgcctgtaatcccagcacttt   44291
                                                                   ********************************** *************************

chimp.LOC112206744.LOC107973052.GGT2-.646279-708040.rev.compl      gggaggccgaggcaggcggatcacgaggtcaggagatcgcgatcacggtgaaaccccgtc   45357
FAM230B-LOC105372935-GGT2.NCBI.GGT2.rev.compl                      gggaggccgaggcaggcggatcacgaggtcaggagatcgcgatcacggtgaaaccccgtc   44351
                                                                   ************************************************************

chimp.LOC112206744.LOC107973052.GGT2-.646279-708040.rev.compl      tctactaaaaatacaaaaaattagctgggcgcagtggcgggtgcctgtagtcccagctac   45417
FAM230B-LOC105372935-GGT2.NCBI.GGT2.rev.compl                      tctactaaaaatacaaaaaattagctgggcgcagtgtcgggcgcctgtagtcccagctac   44411
                                                                   ************************************ **** ******************

chimp.LOC112206744.LOC107973052.GGT2-.646279-708040.rev.compl      tcgggaggctgaggcaggagaatggcgtgaacccaggaggcggagcttgcagtgagccga   45477
FAM230B-LOC105372935-GGT2.NCBI.GGT2.rev.compl                      tcgggaggctgaggcaggagaatggcgtgaacccaggaggcggagcttgcagtgagccga   44471
                                                                   ************************************************************

chimp.LOC112206744.LOC107973052.GGT2-.646279-708040.rev.compl      gatagcaccactgcactccagcctgggcgacagagcgacaccctgtctcacaaaaaagcc   45537
FAM230B-LOC105372935-GGT2.NCBI.GGT2.rev.compl                      gatagcgccactgcactccagcctgggcaacagagcgagactccgtctcaaagaaaaaac   44531
                                                                   ****** ********************* ********* ** * ****** * ****  *

chimp.LOC112206744.LOC107973052.GGT2-.646279-708040.rev.compl      acacctgtaatcccagcgctttgggaggctgaggtgggaagatagtttgatcccaggagt   45597
FAM230B-LOC105372935-GGT2.NCBI.GGT2.rev.compl                      acacctgtaatcccagcgctttgggaggctgaggtgggaagacagtttgatcccaggagt   44591
                                                                   ****************************************** *****************

chimp.LOC112206744.LOC107973052.GGT2-.646279-708040.rev.compl      ttgagaccagtctgggcaagaccctgtctctaaaaaaatatataaaaattatccaggttt   45657
FAM230B-LOC105372935-GGT2.NCBI.GGT2.rev.compl                      ttgagaccagtctgggcaagaccctgtctctaaaaaaa-atacaaaaattatccaggttt   44650
                                                                   ************************************** *** *****************

chimp.LOC112206744.LOC107973052.GGT2-.646279-708040.rev.compl      ggtggcacgtgcctctggtcccagctgctcagggggctgaagtgggaggattgcttgagc   45717
FAM230B-LOC105372935-GGT2.NCBI.GGT2.rev.compl                      ggtggcacgtgcctctggtcccagctgctcagggggctgaagtgggaggattgcttgagc   44710
                                                                   ************************************************************

chimp.LOC112206744.LOC107973052.GGT2-.646279-708040.rev.compl      cctggaggttgaggctgcagtgagccaagatcacaccactgcactccaacctggatgaca   45777
FAM230B-LOC105372935-GGT2.NCBI.GGT2.rev.compl                      cctggaggttgaggctgcagtgagccaagatcacaccactgcactccaacctggatgaca   44770
                                                                   ************************************************************

chimp.LOC112206744.LOC107973052.GGT2-.646279-708040.rev.compl      gagactctgtgtccaaacaaaaacacaacaaaaacaaaacagccaagagagcctactgta   45837
FAM230B-LOC105372935-GGT2.NCBI.GGT2.rev.compl                      gagactctgtgtacaaacaaaaacacaacaaaaacaaaacagccaagggagcctactgta   44830
                                                                   ************ *********************************** ************

chimp.LOC112206744.LOC107973052.GGT2-.646279-708040.rev.compl      ggtaaagagaagggacaacagcaggttgtgtcaccccgacatcctgctgtgctatgttca   45897
FAM230B-LOC105372935-GGT2.NCBI.GGT2.rev.compl                      ggtaaagagaaggg---acagcaggttgtgtcaccccgacatcctgctgtgctatgttca   44887
                                                                   **************   *******************************************

chimp.LOC112206744.LOC107973052.GGT2-.646279-708040.rev.compl      agtctcacttttgagacacaccctgaggtgcgacatcagtaacctactgtggaatccctc   45957
FAM230B-LOC105372935-GGT2.NCBI.GGT2.rev.compl                      agtctcacttttgagacacaccctgaggtgcgacatcagtaacctactgtggaatccctc   44947
                                                                   ************************************************************
```

```
chimp.LOC112206744.LOC107973052.GGT2-.646279-708040.rev.compl      agaaaaacacgaatcccaatagacgtgggtggagacggagagagttaggaaatccggcag   46017
FAM230B-LOC105372935-GGT2.NCBI.GGT2.rev.compl                      agaaaaacacgaatcccaatagatgtgggtggagacggagagagttaggaaatccggcag   45007
                                                                   *********************** ************************************

chimp.LOC112206744.LOC107973052.GGT2-.646279-708040.rev.compl      aaacgtccacactgcagactccaggaaaagggaacattgatgcttgggcagttttggggt   46077
FAM230B-LOC105372935-GGT2.NCBI.GGT2.rev.compl                      aaatgtccacactgcagaatccaggaaaagggaacattgatgcttgggcagttttgggtt   45067
                                                                   *** ************** *************************************** *

chimp.LOC112206744.LOC107973052.GGT2-.646279-708040.rev.compl      atttttacatttttgtaggtgtgaaaatttgcaaaatgaaaactcgag--gagtgtggtg   46135
FAM230B-LOC105372935-GGT2.NCBI.GGT2.rev.compl                      ttttttacatttttgtaggtgcgaaaatttgcaaaatgaaaactcgaggagagtgtggtg   45127
                                                                    ******************** **************************  **********

chimp.LOC112206744.LOC107973052.GGT2-.646279-708040.rev.compl      agctgtgtgagatgctgctgagtggggcctgatggggaaactgaggctggacatggcgat   46195
FAM230B-LOC105372935-GGT2.NCBI.GGT2.rev.compl                      agctgtgtgagatgctgctgagtggggcctgatggggaaactgaggctggacatggcgat   45187
                                                                   ************************************************************

chimp.LOC112206744.LOC107973052.GGT2-.646279-708040.rev.compl      ctggtggcatggggatagagcaggggaggggataccctgaagggagagaggacatagccc   46255
FAM230B-LOC105372935-GGT2.NCBI.GGT2.rev.compl                      ctggtggcatggggatagagcaggggaggggataccctgaagggagagaggacatagccc   45247
                                                                   ************************************************************

chimp.LOC112206744.LOC107973052.GGT2-.646279-708040.rev.compl      agccatgtttgctctaagaggagcaaaggacagaaggaggcagcagatagaaatttctag   46315
FAM230B-LOC105372935-GGT2.NCBI.GGT2.rev.compl                      agccatgtttgctctaagaggagcaaaggacagaaggaggcagcagatagaagtttctag   45307
                                                                   **************************************************** *******

chimp.LOC112206744.LOC107973052.GGT2-.646279-708040.rev.compl      agcaacaacagctgcctttcttttgggaataatccgtgataaagaagtaaatcgtcagag   46375
FAM230B-LOC105372935-GGT2.NCBI.GGT2.rev.compl                      agcaacaacagctgcctttctttcgggaataatccgtgataaagaaataaatcatcagag   45367
                                                                   *********************** ********************** ****** ******

chimp.LOC112206744.LOC107973052.GGT2-.646279-708040.rev.compl      gcggaaaggagcattgtaggaacagcaccccgagccagcgagtagatgaaggagctggcc   46435
FAM230B-LOC105372935-GGT2.NCBI.GGT2.rev.compl                      gcagacaggagcattgtaggaacagcaccccgagccagtgagtagatgaaggagctggcc   45427
                                                                   ** ** ******************************** *********************

chimp.LOC112206744.LOC107973052.GGT2-.646279-708040.rev.compl      ttagccaaaagggagggcagagggacacgctgcagtggctctgtcccctcagagagacaa   46495
FAM230B-LOC105372935-GGT2.NCBI.GGT2.rev.compl                      ttagccaaaaaggagggcagagggacacgctgcagtggctctgtcccctcagagagacaa   45487
                                                                   ********** *************************************************

chimp.LOC112206744.LOC107973052.GGT2-.646279-708040.rev.compl      gacaccaggtcactggctgcagcgggagtcagaggtgcagaatgctcacggggaaagaga   46555
FAM230B-LOC105372935-GGT2.NCBI.GGT2.rev.compl                      gacaccaggtcactggctgcagcgggagtcagaggtgcagaatgctcacagggaaagaga   45547
                                                                   ************************************************* **********

chimp.LOC112206744.LOC107973052.GGT2-.646279-708040.rev.compl      agacaccacccgggcagctgacgcccctcctgggaggtcactggtcagtgtggggggtc   46615
FAM230B-LOC105372935-GGT2.NCBI.GGT2.rev.compl                      agacaccacccgggcagctgacgcccctcctgggaggtcactggtcagtgtggggggtc   45607
                                                                   ************************************************************

chimp.LOC112206744.LOC107973052.GGT2-.646279-708040.rev.compl      tgcagatccgcccaggaatgccaaggacccaagtaggtaaggagggatgtgaggatcctc   46675
FAM230B-LOC105372935-GGT2.NCBI.GGT2.rev.compl                      tgcagatccgcccaggaatgccaaggacccaagtaggtaaggagggatgtgaggatcctc   45667
                                                                   ************************************************************

chimp.LOC112206744.LOC107973052.GGT2-.646279-708040.rev.compl      agcaggaagggatatgacagggtcttatagggacccagcgtggagctggggcaactactg   46735
FAM230B-LOC105372935-GGT2.NCBI.GGT2.rev.compl                      agtgggaagggatatgacagggtcttatagggacccagcatggagctggggcaactactg   45727
                                                                   **  *********************************** ********************

chimp.LOC112206744.LOC107973052.GGT2-.646279-708040.rev.compl      agtgagtgggtcaggtggtgcatggctgagggtggcatctgggaagcgttagtttggagt   46795
FAM230B-LOC105372935-GGT2.NCBI.GGT2.rev.compl                      ggtgagtgggtcaggtggtgcagggctgagggtggcatctgggaagcattagtttggagt   45787
                                                                    ********************* ************************ ************

chimp.LOC112206744.LOC107973052.GGT2-.646279-708040.rev.compl      gacagggagtgggtggccaaggtctctgttcacctggccctctccctttcacccatccat   46855
FAM230B-LOC105372935-GGT2.NCBI.GGT2.rev.compl                      gacagggagtgggtggccgaggtctctgttcacctggccctctccctttcacccatccat   45847
                                                                   ****************** *****************************************

chimp.LOC112206744.LOC107973052.GGT2-.646279-708040.rev.compl      ctcaccatgcccgagggctgcaatgcccccatgcattcctcatctcagcactgagcactc   46915
FAM230B-LOC105372935-GGT2.NCBI.GGT2.rev.compl                      ctcaccatgcccgagggctgcaatgcccccatgcattcctcatctcagcactgagcactc   45907
                                                                   ************************************************************

chimp.LOC112206744.LOC107973052.GGT2-.646279-708040.rev.compl      gagaaccacagcatgtgcaaaggacctgaggtaggagggtggccactaattccccacact   46975
FAM230B-LOC105372935-GGT2.NCBI.GGT2.rev.compl                      aagaaccacagcatgtgcaaaggacctgaggtaggagggtggccactaattccccgcact   45967
                                                                    ****************************************************** ****

chimp.LOC112206744.LOC107973052.GGT2-.646279-708040.rev.compl      gtcagttctgtgcggcagaagccaagactggggtcacccacgagtccatccgagcacac   47035
FAM230B-LOC105372935-GGT2.NCBI.GGT2.rev.compl                      gtcagttctgtggggcagaagccaagactggggtcacccacgagtccatccgagcacac   46027
                                                                   ************ ***********************************************

chimp.LOC112206744.LOC107973052.GGT2-.646279-708040.rev.compl      agtaggcccgcaatcaaagtctgtggcaggaatggattcataaagcatatgtgaggctgt   47095
FAM230B-LOC105372935-GGT2.NCBI.GGT2.rev.compl                      agtaggcccgcaatcgaattctgtggcaggaatggattcataaagcatatgtgaggctgt   46087
                                                                   *************** ** *****************************************

chimp.LOC112206744.LOC107973052.GGT2-.646279-708040.rev.compl      agccaccctggggagcccacctgaggcctctacagcaggccccacacccacagtgggcca   47155
FAM230B-LOC105372935-GGT2.NCBI.GGT2.rev.compl                      agccaccctggggagcccacctgatgcctctacggcaggccccacacccacagtgagcca   46147
                                                                   ************************ ******** ********************* ****

chimp.LOC112206744.LOC107973052.GGT2-.646279-708040.rev.compl      gcccctgccccttaccccagtcacatccacaaacttggggtccccaagcagggtcctctt   47215
FAM230B-LOC105372935-GGT2.NCBI.GGT2.rev.compl                      gcccctgccccttacctcagtcacatccacaaacttggggtccccaagcagggtcctctt   46207
                                                                   **************** *******************************************

chimp.LOC112206744.LOC107973052.GGT2-.646279-708040.rev.compl      ggcgtaggcaaaccggaaaggcctctacgatgtggtggtacgtcaggcccttctgcttgg   47275
FAM230B-LOC105372935-GGT2.NCBI.GGT2.rev.compl                      ggcgtaggcaaaccggaaa-gcctctacgatgcggtggtacgtcaggcccttctgctcgg   46266
                                                                   ******************* ************ ************************ **

chimp.LOC112206744.LOC107973052.GGT2-.646279-708040.rev.compl      gggtctccacgctctcctgggagaagttgtaccctggttgatcagaaccaggtgcatgtt   47335
FAM230B-LOC105372935-GGT2.NCBI.GGT2.rev.compl                      gggtctccacgctctcccgggagaagttgtaccctggttgatcagagccaggtgcatgtt   46326
                                                                   ***************** ***************************** ************

chimp.LOC112206744.LOC107973052.GGT2-.646279-708040.rev.compl      gctgagccccagaggctctgaggggctcagagggttaacacctgcctgagccactttgcc   47395
FAM230B-LOC105372935-GGT2.NCBI.GGT2.rev.compl                      gctgagccccagaggctctgaggggctcagagggttaacacctgcctgagccactttgcc   46386
                                                                   ************************************************************

chimp.LOC112206744.LOC107973052.GGT2-.646279-708040.rev.compl      cacctcaaggagcgtttaataaccaatagcagcagctgcttcagaaggctgtggtgagag   47455
FAM230B-LOC105372935-GGT2.NCBI.GGT2.rev.compl                      cacctcaaggagcgtttaataaccaatagcagcagctgcttcagaaggctgtggtgagag   46446
                                                                   ************************************************************

chimp.LOC112206744.LOC107973052.GGT2-.646279-708040.rev.compl      tgaagtaaggtgagggctcccgagcctgggcctcacgtcatgcatcagctctggccattc   47515
FAM230B-LOC105372935-GGT2.NCBI.GGT2.rev.compl                      tgaagtaaggtgagggctcccgagcctggacctcacgtcacgcatcagctctggccattc   46506
                                                                   ***************************** ********** *******************

chimp.LOC112206744.LOC107973052.GGT2-.646279-708040.rev.compl      agtgaccaagtggcagggtcacccactggaccagggtaccctctggaccagccctgcagc   47575
FAM230B-LOC105372935-GGT2.NCBI.GGT2.rev.compl                      aatgaccaagtggcagggtcacccactggaccggggtaccctctggaccggccctgcagc   46566
                                                                   * ****************************** **************** **********

chimp.LOC112206744.LOC107973052.GGT2-.646279-708040.rev.compl      cctgagctcctgcacctctctccctcctgatgactcctgttcctcctccaacccttgagc   47635
FAM230B-LOC105372935-GGT2.NCBI.GGT2.rev.compl                      cctgagctcctgcacctctctccctcctgatgactcctgttcctcctccaacccttgagc   46626
                                                                   ************************************************************

chimp.LOC112206744.LOC107973052.GGT2-.646279-708040.rev.compl      attgcccgcttcagccctgtcgctctgcactgcctccttcaggacatggtgagctgtgat   47695
FAM230B-LOC105372935-GGT2.NCBI.GGT2.rev.compl                      attgcccgcttcagccctgttgctctgcactgcctccttcaggacatggtgagctgtgac   46686
                                                                   ******************** ***************************************

chimp.LOC112206744.LOC107973052.GGT2-.646279-708040.rev.compl      gcagggacacgcctcgggagctcagtgatggaaagacgtggcatgggggcgagcagaga   47755
FAM230B-LOC105372935-GGT2.NCBI.GGT2.rev.compl                      gcagggacacacctcaggagctcagtgatggaaagacgtggcatgggggcgagcagaga   46746
                                                                   ********** **** ********************************************

chimp.LOC112206744.LOC107973052.GGT2-.646279-708040.rev.compl      tgcagcaggggtggggcgtggggagagagaggcagtgtcatgggtcctaccacacggctg   47815
FAM230B-LOC105372935-GGT2.NCBI.GGT2.rev.compl                      tgcaggaggggtggggcgtggggagagagaggcagtgtcatgggtcctaccacacggctg   46806
                                                                   ***** ******************************************************

chimp.LOC112206744.LOC107973052.GGT2-.646279-708040.rev.compl      tggtgcgaccacccacctttgaggatgttgaggatgagggccagcacgggcccgctgagc   47875
FAM230B-LOC105372935-GGT2.NCBI.GGT2.rev.compl                      tggtgcgaccactcacctttgaggatgttgaggatgagggccagcacgggcccgctgagc   46866
                                                                   ************ ***********************************************

chimp.LOC112206744.LOC107973052.GGT2-.646279-708040.rev.compl      ggcgcactgggcatgtacagcaccgcgtctcccaggctgatgttcagcgggtgctcgatc   47935
FAM230B-LOC105372935-GGT2.NCBI.GGT2.rev.compl                      cgcgcactgggcatgtacagcaccgcgtctcccaggctgatgttcagcgggtgctcgatc   46926
                                                                    ***********************************************************

chimp.LOC112206744.LOC107973052.GGT2-.646279-708040.rev.compl      agctcagcacggtagttgttcaggtcctcagctgtcacaatgccccctgcaatgggacag   47995
FAM230B-LOC105372935-GGT2.NCBI.GGT2.rev.compl                      agctcagcacggtagttgttcaggtcctcagctgtcacaatgccccctgcaatgggacag   46986
                                                                   ************************************************************

chimp.LOC112206744.LOC107973052.GGT2-.646279-708040.rev.compl      cagctcgaatgggcgctgggatggggctgcaccactgcgtggaggatggagctgcaccag   48055
FAM230B-LOC105372935-GGT2.NCBI.GGT2.rev.compl                      cagctcgaatgggtgctgggatggggctgcaccactgcgtggaggatggaactgcaccag   47046
                                                                   ************* ************************************* ********

chimp.LOC112206744.LOC107973052.GGT2-.646279-708040.rev.compl      tggtgttgggggcaggcatggctgcaccatggtggtggggaaaagcctgtacctaccagg   48115
FAM230B-LOC105372935-GGT2.NCBI.GGT2.rev.compl                      tggtgttgggggcaggcatggctgcaccatggtggtggggaaaagcctgtacctaccagg   47106
                                                                   ************************************************************

chimp.LOC112206744.LOC107973052.GGT2-.646279-708040.rev.compl      gaggacagagtgcactactggaggggtgggactgtgccctgggaggggggccacaggcaac   48175
```

```
FAM230B-LOC105372935-GGT2.NCBI.GGT2.rev.compl                    gaggacagagtgcactactggaggggtgggactgtgccctgggagggggcacaggcaac    47166
                                                                 ***********************************************************

chimp.LOC112206744.LOC107973052.GGT2-.646279-708040.rev.compl    ctcacctccttgggaacctcaccagctccggcactcctgtctccctgacactgctcacca   48235
FAM230B-LOC105372935-GGT2.NCBI.GGT2.rev.compl                    ctcacctccttgggaacctcaccagctccggcactcctgtctccctgacactgctcacca   47226
                                                                 ************************************************************

chimp.LOC112206744.LOC107973052.GGT2-.646279-708040.rev.compl    cccgacagctgggctggggccactgccctctgcctgcttggcttactggcttcctgtct   48295
FAM230B-LOC105372935-GGT2.NCBI.GGT2.rev.compl                    cccgacagctgggctggggccactgccctctgcctgcttggcttactggcttcctgtct   47286
                                                                 ************************************************************

chimp.LOC112206744.LOC107973052.GGT2-.646279-708040.rev.compl    gccttctctcatctgtggccggagagtgtttccttttttcttttttttttttagagatac   48355
FAM230B-LOC105372935-GGT2.NCBI.GGT2.rev.compl                    gccttctctcatctgtggccagagagtgttttctttttttcttttttttttttagagatac   47346
                                                                 ******************** ********** ***************************

chimp.LOC112206744.LOC107973052.GGT2-.646279-708040.rev.compl    gatcttgctctgttacccaggctggagtgcagtggcttaatcacagccttgaactcccgg   48415
FAM230B-LOC105372935-GGT2.NCBI.GGT2.rev.compl                    gatcttgctctgttacccaggctggagtgcagtggcttaatcacagccttgaacccctgg   47406
                                                                 ****************************************************** ** **

chimp.LOC112206744.LOC107973052.GGT2-.646279-708040.rev.compl    gctcaagtgatcctccagcagaccctccccagtagctgagactaaaggcacaactacacc   48475
FAM230B-LOC105372935-GGT2.NCBI.GGT2.rev.compl                    gctcaagtgatcctccagcagaccctccccagtagctgagactaaaggcacaactacacc   47466
                                                                 ************************************************************

chimp.LOC112206744.LOC107973052.GGT2-.646279-708040.rev.compl    cagcaaattttaatttttttgttgtgttttgctatatttcttcttcttcttcttctttt   48535
FAM230B-LOC105372935-GGT2.NCBI.GGT2.rev.compl                    cagcaaattttaatttttttgttgtgttttgctatatttcttcttcttctttt-tttttttt   47525
                                                                 ***************************************************** *** ****

chimp.LOC112206744.LOC107973052.GGT2-.646279-708040.rev.compl    ttttttgtgagacggagtctcactctatcgcccaggctggagtgcagtggcgcgatatcg   48595
FAM230B-LOC105372935-GGT2.NCBI.GGT2.rev.compl                    ttttttgtgagacggagtctcgctctatcgcccaggctggagtgcagtggcgcgatatcg   47585
                                                                 ********************* **************************************

chimp.LOC112206744.LOC107973052.GGT2-.646279-708040.rev.compl    gctcactgcaagctccgcctcccaggttcacgccattctcctgcctcagcctcccaagta   48655
FAM230B-LOC105372935-GGT2.NCBI.GGT2.rev.compl                    gctcactgcaagctccgcctcccaggttcacgccattctcctgcctcagcctcccaagta   47645
                                                                 ************************************************************

chimp.LOC112206744.LOC107973052.GGT2-.646279-708040.rev.compl    gctgggactgcaggcacccgccaccacacctggctaattttttttaaattttttagtagaga   48715
FAM230B-LOC105372935-GGT2.NCBI.GGT2.rev.compl                    gctgggactgcaggcacccgccaccacatgtggctaattttctgtattttttagtagaga   47705
                                                                 ****************************  *********** *  **************

chimp.LOC112206744.LOC107973052.GGT2-.646279-708040.rev.compl    caggtttcactgtgttagccaggatggtctcgatctcctgaccctgtgatccacacgcct   48775
FAM230B-LOC105372935-GGT2.NCBI.GGT2.rev.compl                    caggtttcactgtgttagccaggatggtctcgatctcctgacccgtgatccacacgcct   47765
                                                                 ********************************************* **************

chimp.LOC112206744.LOC107973052.GGT2-.646279-708040.rev.compl    cggcctctcaaagtgctgggattacaggcgtgaggcaccatgcccggccgggttttgcta   48835
FAM230B-LOC105372935-GGT2.NCBI.GGT2.rev.compl                    cggcctctcaaagtgctgagattacaggcgtgaggcaccatgcccggccgggttttgcta   47825
                                                                 ****************** *****************************************

chimp.LOC112206744.LOC107973052.GGT2-.646279-708040.rev.compl    tatttcttttcactatgctttgaatttttttgttttcttgttccccacccccacccccact   48895
FAM230B-LOC105372935-GGT2.NCBI.GGT2.rev.compl                    tatttcttttcactatgctttgaatttttttgttttcttgttccccacccccacccccact   47885
                                                                 ************************************************************

chimp.LOC112206744.LOC107973052.GGT2-.646279-708040.rev.compl    atatttatgtagattctcaattttttttttgtagactcactatgttgcccaggcttgtct   48955
FAM230B-LOC105372935-GGT2.NCBI.GGT2.rev.compl                    atatttatgtagattctcaatattttttttgtagactcactatgttgcccaggcttgtct   47945
                                                                 ********************* **************************************

chimp.LOC112206744.LOC107973052.GGT2-.646279-708040.rev.compl    tggacctcctgacctcaacttctacctcagcctcccaaagtgttgggattacaggcatga   49015
FAM230B-LOC105372935-GGT2.NCBI.GGT2.rev.compl                    tggacccctgacctcaacttctacctcagcctcccaaagtgttgggattacaggcatga   48005
                                                                 ****** *****************************************************

chimp.LOC112206744.LOC107973052.GGT2-.646279-708040.rev.compl    gacaccaggctttgcctcagacggccttttcttcttcttttttttttttgagatggagtct   49075
FAM230B-LOC105372935-GGT2.NCBI.GGT2.rev.compl                    gacaccaagcttggcctcagagggccttttc--tctcttttttttttttgagatggagtct   48063
                                                                 ******* **** ******** *********  *** **********************

chimp.LOC112206744.LOC107973052.GGT2-.646279-708040.rev.compl    cactctgtggagtgcagtggtgcaatctcggctcactgcaacctcagcccccaggttct   49135
FAM230B-LOC105372935-GGT2.NCBI.GGT2.rev.compl                    cactctgtggagtgcagtggtgcaatctcggctcactgcaacctcagccccacaggttct   48123
                                                                 **************************************************** ********

chimp.LOC112206744.LOC107973052.GGT2-.646279-708040.rev.compl    agcgattctcctgccacagcctcccacgtagctgggattacaggcacaacccaccatgcc   49195
FAM230B-LOC105372935-GGT2.NCBI.GGT2.rev.compl                    agcgattctcctgccacagcctcccaagtagctgggattacaggcacaacccaccatgcc   48183
                                                                 ************************** *********************************

chimp.LOC112206744.LOC107973052.GGT2-.646279-708040.rev.compl    tgactaattttgcatttttagtagagacagggtttcaccatgttggccaggctggtcttg   49255
FAM230B-LOC105372935-GGT2.NCBI.GGT2.rev.compl                    tgactaattttgcatttttagtagagacagggtttcaccatgttggccaggctggtcttg   48243
                                                                 ************************************************************

chimp.LOC112206744.LOC107973052.GGT2-.646279-708040.rev.compl    aactcctgagctcaggtgttccacccgcctcggcctcccaaagtgctgggattacagacg   49315
FAM230B-LOC105372935-GGT2.NCBI.GGT2.rev.compl                    aactcctgacctcaggtgatccacccgcctcggcctcccaaagtgctgggattacagacg   48303
                                                                 ********* ******** *****************************************

chimp.LOC112206744.LOC107973052.GGT2-.646279-708040.rev.compl    tgagccactgcgcctggctcagagggccttttctaactggagaattcctgcggtgtccc   49375
FAM230B-LOC105372935-GGT2.NCBI.GGT2.rev.compl                    tgagccaccgcacctggctcagagggccttttctaactggagaattcctgcggtgtccc   48363
                                                                 ******** ** ************************************************

chimp.LOC112206744.LOC107973052.GGT2-.646279-708040.rev.compl    tgctgcttggcctcttctcctcatgaggaatggatagagggaggagggaggctcttaat   49435
FAM230B-LOC105372935-GGT2.NCBI.GGT2.rev.compl                    tgctgcttggcctcttctcctcacgatgaatggatagagggaggagggaggctcttaat   48423
                                                                 *********************** ** *********************************

chimp.LOC112206744.LOC107973052.GGT2-.646279-708040.rev.compl    tctcctggagtcagctccagacagacagggtattggcatgccaatttccagcctcagtgg   49495
FAM230B-LOC105372935-GGT2.NCBI.GGT2.rev.compl                    tctcctggagtcagctc----cagacagggtattggcatgccaatttccagcctcagtgg   48479
                                                                 *****************    ***************************************

chimp.LOC112206744.LOC107973052.GGT2-.646279-708040.rev.compl    taaaggtcgacacgctaatcaccctcctccatgaagcagtgacaaaaattacctgaagaa   49555
FAM230B-LOC105372935-GGT2.NCBI.GGT2.rev.compl                    taaaggtcgacacgctaatcaccctcctccatgaaacagtgacaaaaattacctgaagaa   48539
                                                                 *********************************** ************************

chimp.LOC112206744.LOC107973052.GGT2-.646279-708040.rev.compl    agccacagccaagctccaggcccctgccccacaaatccccttccccatgcctctctcaag   49615
FAM230B-LOC105372935-GGT2.NCBI.GGT2.rev.compl                    agccacagccaagctccaggcccctgccccacaaatccccttccccatgcctctctcaag   48599
                                                                 ************************************************************

chimp.LOC112206744.LOC107973052.GGT2-.646279-708040.rev.compl    gcgaccctcatcccttgtaaccctcttggtgaatcaaagcgccctctactgggccttagc   49675
FAM230B-LOC105372935-GGT2.NCBI.GGT2.rev.compl                    gcgaccctcatcccttgtaaccctcttggtgaatcaaagcgccctctactgggccttagc   48659
                                                                 ************************************************************

chimp.LOC112206744.LOC107973052.GGT2-.646279-708040.rev.compl    ccagctgttcctctgctaggaatcccttcctctctctgcctaacgaagttatctgcagcc   49735
FAM230B-LOC105372935-GGT2.NCBI.GGT2.rev.compl                    ccagctgttcctctgctaggaatcccttcctctctctgcctaacgaagttatctgcagcc   48719
                                                                 ************************************************************

chimp.LOC112206744.LOC107973052.GGT2-.646279-708040.rev.compl    cagcgccacctcctccaagaagtcctcctggatcttcaggctgtattctagtgcttccc   49795
FAM230B-LOC105372935-GGT2.NCBI.GGT2.rev.compl                    cagcgccacctcctccaagaagtcctcctggatcttcaggctgtattctagtgcttccc   48779
                                                                 ************************************************************

chimp.LOC112206744.LOC107973052.GGT2-.646279-708040.rev.compl    tagccctggctcttgcctacacctgcatttaccccaacaggacttgctctcctggactg   49855
FAM230B-LOC105372935-GGT2.NCBI.GGT2.rev.compl                    tagccctggctcttgcctacacctgcatttaccccaacaggacttgctctcctggactg   48839
                                                                 ************************************************************

chimp.LOC112206744.LOC107973052.GGT2-.646279-708040.rev.compl    tgtggcctctcttggttttgatataagcaggagctgtggacccacatggccagtcactga   49915
FAM230B-LOC105372935-GGT2.NCBI.GGT2.rev.compl                    tgtggcctctcttggttttgatataagcaggagctgtggacccacatggccagtcactga   48899
                                                                 ************************************************************

chimp.LOC112206744.LOC107973052.GGT2-.646279-708040.rev.compl    ccctcctccaccaggaacttcctgcaggctcaggcaagacaggaggaccccatggctctg   49975
FAM230B-LOC105372935-GGT2.NCBI.GGT2.rev.compl                    ccctcctccaccaggaacttcctgcaggctcaggcaagacaggaggaccccatggctctg   48959
                                                                 ************************************************************

chimp.LOC112206744.LOC107973052.GGT2-.646279-708040.rev.compl    ggctacagctcagggtttccactgcagagttcctcacccaggtccttgaggttacccact   50035
FAM230B-LOC105372935-GGT2.NCBI.GGT2.rev.compl                    ggctacagctcagggtttccactgcagagttcctcacccaggtccttgaggttacccact   49019
                                                                 ************************************************************

chimp.LOC112206744.LOC107973052.GGT2-.646279-708040.rev.compl    caccagccgcctggatgtccttcacaatctgggccatgaggctgccgttgtagaaggcct   50095
FAM230B-LOC105372935-GGT2.NCBI.GGT2.rev.compl                    caccagccgcctggatgtccttcacaatctgggccatgaggctgccgttgtagaaggcct   49079
                                                                 ************************************************************

chimp.LOC112206744.LOC107973052.GGT2-.646279-708040.rev.compl    gggcgccctcgatggccagcatctcgtaggtgtcagccagccgcggcagggtcagtctct   50155
FAM230B-LOC105372935-GGT2.NCBI.GGT2.rev.compl                    gggcaccctcgatggccagcatctcataggtgtcagccagccgcggcagggtcagtctct   49139
                                                                 **** ******************** **********************************

chimp.LOC112206744.LOC107973052.GGT2-.646279-708040.rev.compl    ccccctcccgaagcacctttctatcccggcagaacacctcactggggcagagggggctca   50215
FAM230B-LOC105372935-GGT2.NCBI.GGT2.rev.compl                    ccccctcccgaagcacctttctatcccggcagaacacctcaccggggcagagggggctca   49199
                                                                 ******************************************* ****************

chimp.LOC112206744.LOC107973052.GGT2-.646279-708040.rev.compl    tgtgaggcagcaggtggggtggactcagctagaccacccccacacccgtccatacagga   50275
FAM230B-LOC105372935-GGT2.NCBI.GGT2.rev.compl                    tgtgaggcagcaggtggggtggactcagctagaccacccccacacctgcccacacagga   49259
                                                                 ********************************************** * *** ******

chimp.LOC112206744.LOC107973052.GGT2-.646279-708040.rev.compl    gaccagcacaaagcaggggcagcgcctgtcacaggtgggtggccctgtcactcagcgctc   50335
FAM230B-LOC105372935-GGT2.NCBI.GGT2.rev.compl                    gaccagcacaaagcaggggcagcacctgtcacaggtgggtggccctgtcactcagcgctc   49319
```

```
                                                         *********************** ************************************

chimp.LOC112206744.LOC107973052.GGT2-.646279-708040.rev.compl      atcctcctagtgtcccttccgggaacctcctagtgtcccttgccactcaggacacatggc   50395
FAM230B-LOC105372935-GGT2.NCBI.GGT2.rev.compl                      atcctcctagtgtcccttccgggagcctcctagtgtcccttgccactcaggacacgtggc   49379
                                                                   ************************ ****************************** ****

chimp.LOC112206744.LOC107973052.GGT2-.646279-708040.rev.compl      cagccacagtggccactgggaccccacgctcagaatgtgtccccacacgtggtgggaagg   50455
FAM230B-LOC105372935-GGT2.NCBI.GGT2.rev.compl                      cagccacagtggccactgggaccccatgctcagaatgtgtccccacacgtggtgggaagg   49439
                                                                   ************************** *********************************

chimp.LOC112206744.LOC107973052.GGT2-.646279-708040.rev.compl      gtctgtatctcctcatcccattatcagcacagggtcctgaaggcagagggccactccact   50515
FAM230B-LOC105372935-GGT2.NCBI.GGT2.rev.compl                      gtctgtatctcctcatcccattatcagcacagggtcctgaaggcagagggccgctccact   49499
                                                                   **************************************************** *******

chimp.LOC112206744.LOC107973052.GGT2-.646279-708040.rev.compl      gctgctacggcctgcaaggtccttgggctgtgcctgccctgcctgtgtcaggggggccgca   50575
FAM230B-LOC105372935-GGT2.NCBI.GGT2.rev.compl                      gctgctacggcctgcaaggtccttgggctgtgcctgccctgcctgtgtcaggggggccgca   49559
                                                                   ************************************************************

chimp.LOC112206744.LOC107973052.GGT2-.646279-708040.rev.compl      cccacagacatgccacaagacaggctgctgctcgatgacggtccgcttgttttccagggc   50635
FAM230B-LOC105372935-GGT2.NCBI.GGT2.rev.compl                      cccacagacataccacaagacaggctgctgctcgatgacagtccgcttgttttccaggac   49619
                                                                   *********** *************************** ****************** *

chimp.LOC112206744.LOC107973052.GGT2-.646279-708040.rev.compl      tgccgccaagcccttgcccacagggaagccctggcgggccagctggatgctgggctggaa   50695
FAM230B-LOC105372935-GGT2.NCBI.GGT2.rev.compl                      tgccgccaagcccttgcccacagggaagccctggcgggccagctggatgctgggctggaa   49679
                                                                   ************************************************************

chimp.LOC112206744.LOC107973052.GGT2-.646279-708040.rev.compl      gaggcgagcccagggcagctgcccatgccactgatgtgccagctcatagcctcggatctc   50755
FAM230B-LOC105372935-GGT2.NCBI.GGT2.rev.compl                      gaggcgagcccagggcagccgcccatgccgctggtgtgccagctcatagcctcggatctc   49739
                                                                   ******************* ********* *** **************************

chimp.LOC112206744.LOC107973052.GGT2-.646279-708040.rev.compl      cccaggcaccgccaccgacagccctcctggggagagagagccacagttagtgaccctgag   50815
FAM230B-LOC105372935-GGT2.NCBI.GGT2.rev.compl                      cccaggcaccgccaccgacagccctcctggggagagagagccacagttagtgaccctgag   49799
                                                                   ************************************************************

chimp.LOC112206744.LOC107973052.GGT2-.646279-708040.rev.compl      tgggggacatccggatctctcgcaggcagcatcccaggcacagtccctgactcgctttac   50875
FAM230B-LOC105372935-GGT2.NCBI.GGT2.rev.compl                      tgggggacatcgggatctctagcaggcagcatcccaggcacagtccctgactcattttac   49859
                                                                   *********** ******** ********************************  *****

chimp.LOC112206744.LOC107973052.GGT2-.646279-708040.rev.compl      agatggggcaatgaggcttaggaggaaagatttttttttctttttttgagttggggtcttg   50935
FAM230B-LOC105372935-GGT2.NCBI.GGT2.rev.compl                      agatggggcaatgaggcttaggaggaaagatttttttttctttttttgagttggggtcttg   49919
                                                                   ************************************************************

chimp.LOC112206744.LOC107973052.GGT2-.646279-708040.rev.compl      ccatcttgcccagcctgatctcgaactcctggattcaagcaatcctcccacctcagcctc   50995
FAM230B-LOC105372935-GGT2.NCBI.GGT2.rev.compl                      ccatcttgcccagcctgatctcgaactcctggattcaagcaatcctcccacctcagcctc   49979
                                                                   ************************************************************

chimp.LOC112206744.LOC107973052.GGT2-.646279-708040.rev.compl      ctgagtagctgagattacaggcgtgaacaccacacccagcagaaggggattttaatttt   51055
FAM230B-LOC105372935-GGT2.NCBI.GGT2.rev.compl                      ctgagtcgctgagattacaggcgtgaacagcacacctagcagaaggggattttaatttt   50039
                                                                   ****** ********************* ****** ************************

chimp.LOC112206744.LOC107973052.GGT2-.646279-708040.rev.compl      ttaatttattttttaattttaattttttttttttaggaggggatgtttaa--tttttttt   51113
FAM230B-LOC105372935-GGT2.NCBI.GGT2.rev.compl                      taaatttatttttaattttaatttttttttttttaggaggggatgtttaattttttttttt   50099
                                                                   * *********** * *** * ****************************  ********

chimp.LOC112206744.LOC107973052.GGT2-.646279-708040.rev.compl      taggaggggctcagca-gtaggagtgtacatggacaagggatgtctgaggagggcacagg   51172
FAM230B-LOC105372935-GGT2.NCBI.GGT2.rev.compl                      taggaggggctcagcaggtaggagtgtacatggaccagggatgtctgaggagggcacagc   50159
                                                                   **************** ****************** ***********************

chimp.LOC112206744.LOC107973052.GGT2-.646279-708040.rev.compl      aggggaagcagtagcatgcggctgggttttgctgtcccaggatgaggtgtctgtctgtgc   51232
FAM230B-LOC105372935-GGT2.NCBI.GGT2.rev.compl                      aggggaagcagtagcatgcggctgggttttgctgtcccaggatgaggtgtctgtctgtac   50219
                                                                   ********************************************************** *

chimp.LOC112206744.LOC107973052.GGT2-.646279-708040.rev.compl      aggtgcctgcatgtctaaaatcctgtgccaggccagacccctcccatctcgctgaccac   51292
FAM230B-LOC105372935-GGT2.NCBI.GGT2.rev.compl                      aggtgcctgcatgtctaaaatcctgtgccaggccagacccctcccatctcgctgaccac   50279
                                                                   ************************************************************

chimp.LOC112206744.LOC107973052.GGT2-.646279-708040.rev.compl      aaggccttatcctgtaagactcatgggctccaccagaatgtgccaaaacaagagcagatc   51352
FAM230B-LOC105372935-GGT2.NCBI.GGT2.rev.compl                      aaggccttatcctgtaagactcatgggctccaccagaatgtgccaaaacaagagcagatc   50339
                                                                   ************************************************************

chimp.LOC112206744.LOC107973052.GGT2-.646279-708040.rev.compl      ccaccctgacccaggtcaagcacaggccaccttcaagacacagccagccccaagaaaggg   51412
FAM230B-LOC105372935-GGT2.NCBI.GGT2.rev.compl                      ccaccctgacccaggtcaagcacaggccaccttcaagacacagccagccccaagaaaggg   50399
                                                                   ************************************************************

chimp.LOC112206744.LOC107973052.GGT2-.646279-708040.rev.compl      ctcccttcctcttttctactgccccagagaggcaagactgagccttaacctccgtcctgt   51472
FAM230B-LOC105372935-GGT2.NCBI.GGT2.rev.compl                      ctcccttcctcttttctactgccccagagaggcaagactgagccttaacctccatcctgt   50459
                                                                   ***************************************************** ******

chimp.LOC112206744.LOC107973052.GGT2-.646279-708040.rev.compl      cccctctcccagcctcagtttctccatccaactataagggtt----tttgtttgtctgtt   51528
FAM230B-LOC105372935-GGT2.NCBI.GGT2.rev.compl                      cccctctcccagcctcagtttctccaaccaactataagggttttgtttgtttgtctgtt   50519
                                                                   ************************** ***************    **************

chimp.LOC112206744.LOC107973052.GGT2-.646279-708040.rev.compl      ttgagacagggtctcattctgttgtcccagctggaatgcagtggtgcaatcatggctcac   51588
FAM230B-LOC105372935-GGT2.NCBI.GGT2.rev.compl                      ttgagacagggtctcactctgttgtcccagctggagtgcagtggtgcaatcatggctcac   50579
                                                                   **************** ****************** ************************

chimp.LOC112206744.LOC107973052.GGT2-.646279-708040.rev.compl      tgcagccttggcttcccaggctcaagcgatcctcccacctcagcctctgaagtacctaag   51648
FAM230B-LOC105372935-GGT2.NCBI.GGT2.rev.compl                      tgcagccttggcttcccaggctcaagcgatcctcccacttcagcctctgaagtacctaag   50639
                                                                   ************************************** *********************

chimp.LOC112206744.LOC107973052.GGT2-.646279-708040.rev.compl      actacagacacaccccactacacgtggc--ttttttttttttttttttttgagatggagtt   51706
FAM230B-LOC105372935-GGT2.NCBI.GGT2.rev.compl                      actacagacataccccactgcacgtggcttttttttttttttttttttttgagatggagtt   50699
                                                                   ********** ******** ********  ******************************

chimp.LOC112206744.LOC107973052.GGT2-.646279-708040.rev.compl      tcactcttgttgcccaggctggagtgcaatggcacaatcttggctcactccaacctccac   51766
FAM230B-LOC105372935-GGT2.NCBI.GGT2.rev.compl                      tcactcttgttgcccaggctggagtgcaatggcacaatcttggctcactccaacctccac   50759
                                                                   ************************************************************

chimp.LOC112206744.LOC107973052.GGT2-.646279-708040.rev.compl      ctcccgagttcaagcaattctcctgcctcagcctcccaagtagctgggattacaggcatg   51826
FAM230B-LOC105372935-GGT2.NCBI.GGT2.rev.compl                      ctcccgagttcaagtaattctcctgcctcagcctcccaagtacctgggattacaggcatg   50819
                                                                   ************** *************************** *****************

chimp.LOC112206744.LOC107973052.GGT2-.646279-708040.rev.compl      caccaccacgccttgctaatttttgtatttttagtggagacggggtttcaccaggttggt   51886
FAM230B-LOC105372935-GGT2.NCBI.GGT2.rev.compl                      taccaccacgccttgctaatttttgtatttttagtagagacggggtttcaccaggttggt   50879
                                                                    ********************************** ************************

chimp.LOC112206744.LOC107973052.GGT2-.646279-708040.rev.compl      caggctggtcttgaactcctaacatcaggtgatccatcctcctcagcctctcagagttct   51946
FAM230B-LOC105372935-GGT2.NCBI.GGT2.rev.compl                      caggctggtcttgaactcctaacatcaggtgatccatcctcctcagcttctcagagttct   50939
                                                                   *********************************************** ************

chimp.LOC112206744.LOC107973052.GGT2-.646279-708040.rev.compl      gggattacaggcgtgagccaccactcccagcctaatttttattttttattttttgtagag   52006
FAM230B-LOC105372935-GGT2.NCBI.GGT2.rev.compl                      gggattacaggcgtgagccaccactcccagcctaatttttattttttattttttgtagag   50999
                                                                   ************************************************************

chimp.LOC112206744.LOC107973052.GGT2-.646279-708040.rev.compl      acaggggtcttgctacgttgccaagactggtctcaaactctggcctcaagcaatcctccc   52066
FAM230B-LOC105372935-GGT2.NCBI.GGT2.rev.compl                      acaggggtcttgctacattgccaagactggtctcaaactctggcctcaagcaatcctccc   51059
                                                                   **************** *******************************************

chimp.LOC112206744.LOC107973052.GGT2-.646279-708040.rev.compl      acctcagcctcccaacatgctgggattacaggtgcactcagcctataaggggttttgcct   52126
FAM230B-LOC105372935-GGT2.NCBI.GGT2.rev.compl                      acctcagcctcccaacatgctgggattacaggtgcactcagcctataaggggttttgcct   51119
                                                                   ************************************************************

chimp.LOC112206744.LOC107973052.GGT2-.646279-708040.rev.compl      tccagttctgacttttgaggaggtcattggaaacagacccctgggcctgctttctccctg   52186
FAM230B-LOC105372935-GGT2.NCBI.GGT2.rev.compl                      tccagttctgacttttgaggaggtcattggaaacagacccctgggcctgcttcccccctg   51179
                                                                   **************************************************** * *****

chimp.LOC112206744.LOC107973052.GGT2-.646279-708040.rev.compl      agcctcactgcccatatggacactacagacactgaccctttgcccagaaaggtacaacta   52246
FAM230B-LOC105372935-GGT2.NCBI.GGT2.rev.compl                      agccccactgcccatatggacactacagacactgaccctttgcccagaaaggtacaacta   51239
                                                                   **** *******************************************************

chimp.LOC112206744.LOC107973052.GGT2-.646279-708040.rev.compl      tggcctctgcccccagggactctcctgctcttgcgagagatgatggggccatttgccttg   52306
FAM230B-LOC105372935-GGT2.NCBI.GGT2.rev.compl                      tggcctctgcccccagggactctcctgctcttgcgagagatgatggggccatttgccttg   51299
                                                                   ************************************************************

chimp.LOC112206744.LOC107973052.GGT2-.646279-708040.rev.compl      gcttggcggctgtggctctagaactgcctctcccaccctgaagcctggcacaagtttcca   52366
FAM230B-LOC105372935-GGT2.NCBI.GGT2.rev.compl                      gcttggcggctgtggctctagaactgcctctcccaccctgaagcctggcacaagtttcca   51359
                                                                   ************************************************************

chimp.LOC112206744.LOC107973052.GGT2-.646279-708040.rev.compl      agagctggtggtttcaattcctagaagctgcacgtacatcccggaaggtctgacacccag   52426
FAM230B-LOC105372935-GGT2.NCBI.GGT2.rev.compl                      agagctggtggtttcaattcctagaagctgcacatatatcccggaaggtctgacacccag   51419
                                                                   ********************************* ** ***********************

chimp.LOC112206744.LOC107973052.GGT2-.646279-708040.rev.compl      cacatgattccttccaccttgtagttagacagaagttcttttttgttttgttttg-tttt   52485
FAM230B-LOC105372935-GGT2.NCBI.GGT2.rev.compl                      catatgattccttccaccttgtagttagacagaagttcttttttgttttgttttgttttt   51479
                                                                   ** **************************************************** ****
```

```
chimp.LOC112206744.LOC107973052.GGT2-.646279-708040.rev.compl      ctttgtttgtttttgagatggagtcttgctctgtctcccagactgcagtgcagtggcatg   52545
FAM230B-LOC105372935-GGT2.NCBI.GGT2.rev.compl                      ctttgtttgtttttgagatggagtcttgctctgtctcccagactgcagtgcagtggcatg   51539
                                                                   ************************************************************

chimp.LOC112206744.LOC107973052.GGT2-.646279-708040.rev.compl      atctcagctcactataacctccgcctcccaggttcaagcgattctcctgcctcagcctcc   52605
FAM230B-LOC105372935-GGT2.NCBI.GGT2.rev.compl                      atctcagctcactataacctccgcctcccaggttcaagcgattctcctgcctcagcctcc   51599
                                                                   ************************************************************

chimp.LOC112206744.LOC107973052.GGT2-.646279-708040.rev.compl      cgagtagctgggattacaggcacaggccagcacgccaggctaatttttgtatttttagta   52665
FAM230B-LOC105372935-GGT2.NCBI.GGT2.rev.compl                      cgagtagctgggattacaggcacaggccagcacgccaggctaatttttgtatttttagta   51659
                                                                   ************************************************************

chimp.LOC112206744.LOC107973052.GGT2-.646279-708040.rev.compl      cagatggtgttttgccatgttggccaggctgttttcaaactcttgacctcaggtcatcca   52725
FAM230B-LOC105372935-GGT2.NCBI.GGT2.rev.compl                      cagatggggttttgccatgttggccaggctgttttcaaactcctgacctcaggtcatcca   51719
                                                                   ******* ********************************** *****************

chimp.LOC112206744.LOC107973052.GGT2-.646279-708040.rev.compl      cccacctcagccttccaaggtgctgggatgacaggcgtgagccaccgtgcccagccaaga   52785
FAM230B-LOC105372935-GGT2.NCBI.GGT2.rev.compl                      cccacctcagccttccaaggtgctgggatgacaggcgtgagccaccgtgcccagccaaga   51779
                                                                   ************************************************************

chimp.LOC112206744.LOC107973052.GGT2-.646279-708040.rev.compl      caggagaagttctaatctttgatagcagaccagggtgacgatgcttagcaacagtatttt   52845
FAM230B-LOC105372935-GGT2.NCBI.GGT2.rev.compl                      caggagaagttctaatctttgatagcagaccagggtgacgatgcttagcaacagtatttt   51839
                                                                   ************************************************************

chimp.LOC112206744.LOC107973052.GGT2-.646279-708040.rev.compl      gtatatttcaaagtaacgaagagaggactatggtgctaacacccagaaatgaaaaatatt   52905
FAM230B-LOC105372935-GGT2.NCBI.GGT2.rev.compl                      gtatatttcaaagtaacgaagagaggactatggtgctaacacccggaaatgaaaaatatt   51899
                                                                   ******************************************** ***************

chimp.LOC112206744.LOC107973052.GGT2-.646279-708040.rev.compl      caaggtgacggagactccaaataccctgccttgatcattatacactatatgcatggaaca   52965
FAM230B-LOC105372935-GGT2.NCBI.GGT2.rev.compl                      caaggtgacggagactccaaataccctgccttgatcattatacactctatgcatggaaca   51959
                                                                   ********************************************** *************

chimp.LOC112206744.LOC107973052.GGT2-.646279-708040.rev.compl      agcactcacatgtacccataaatatagaaaatatcatgtatcaatatcagaaaaaaatct   53025
FAM230B-LOC105372935-GGT2.NCBI.GGT2.rev.compl                      agcactcacatgtacccataaatatagaaaatatcatgtatcaatatcagaaaaaaatct   52019
                                                                   ************************************************************

chimp.LOC112206744.LOC107973052.GGT2-.646279-708040.rev.compl      cctcctgacctcagcccaatcaggctctcatgccaccacacttgccaagttctctggtga   53085
FAM230B-LOC105372935-GGT2.NCBI.GGT2.rev.compl                      cctcctgacctcagcccaatcaggctctcatgccaccacacttgccaagttctctggtga   52079
                                                                   ************************************************************

chimp.LOC112206744.LOC107973052.GGT2-.646279-708040.rev.compl      cccccacactgccagacccagtgccccctctcagctttactgggctcatcactctccctg   53145
FAM230B-LOC105372935-GGT2.NCBI.GGT2.rev.compl                      cccccacactgccagacccagtgccccctctcagctttactgggctcatcactctccctg   52139
                                                                   ************************************************************

chimp.LOC112206744.LOC107973052.GGT2-.646279-708040.rev.compl      agagccgcccctgcatcccagcacctggctccacccgagtctcccccgcctgccatccta   53205
FAM230B-LOC105372935-GGT2.NCBI.GGT2.rev.compl                      agagccgcccctgcatcccagcacctggctccacccgagtctcccccgcctgccatccta   52199
                                                                   ************************************************************

chimp.LOC112206744.LOC107973052.GGT2-.646279-708040.rev.compl      gctcctactctcccctctgtctttgctctctctcctggtggtctgcttgacatctgagct   53265
FAM230B-LOC105372935-GGT2.NCBI.GGT2.rev.compl                      gctcctactctcccctctgtctttgctctctctcctggtggtctgcttgacatctgagct   52259
                                                                   ************************************************************

chimp.LOC112206744.LOC107973052.GGT2-.646279-708040.rev.compl      tcagcctccatttatgcactgacaactcccaaattgacctgctggcctggactgctcctc   53325
FAM230B-LOC105372935-GGT2.NCBI.GGT2.rev.compl                      tcagcctccatttatgcactgacaactctcaaattgacctgctggcctggactgctcctc   52319
                                                                   **************************** *******************************

chimp.LOC112206744.LOC107973052.GGT2-.646279-708040.rev.compl      tgatcaccagacctgagtatctacctgcctgcttgaagaaagcatctcaaacttcaacgt   53385
FAM230B-LOC105372935-GGT2.NCBI.GGT2.rev.compl                      cgatcaccagacctgaggatctacctgcctgctcgaagaaagcatctcaaacttcaacgt   52379
                                                                    **************** *************** **************************

chimp.LOC112206744.LOC107973052.GGT2-.646279-708040.rev.compl      gcccaaaaccgagctcctgagtgtctgctcaacctgcttcctgagaaccctgcctgtctc   53445
FAM230B-LOC105372935-GGT2.NCBI.GGT2.rev.compl                      gcccaaaaccgagctcctgagtgtctgctcaacctgcttcctgagaaccctgcctgtctc   52439
                                                                   ************************************************************

chimp.LOC112206744.LOC107973052.GGT2-.646279-708040.rev.compl      cattagggtcaccccatccttccaggtacagacaaaagatcaggggtccccggggactcc   53505
FAM230B-LOC105372935-GGT2.NCBI.GGT2.rev.compl                      cattagggtcaccccatccttccaggtacagacaaaagatcaggggtccccggggactcc   52499
                                                                   ************************************************************

chimp.LOC112206744.LOC107973052.GGT2-.646279-708040.rev.compl      ctacacaagcgtcacacccaacccatcctcaaatccgcaggctccacttccaagtgtgtc   53565
FAM230B-LOC105372935-GGT2.NCBI.GGT2.rev.compl                      ctacacaagcgtcacacccaacccatcctcaaatccacaggctccacttccaagtgtgtc   52559
                                                                   ************************************ ***********************

chimp.LOC112206744.LOC107973052.GGT2-.646279-708040.rev.compl      tgtccagcgtcagccacttcccagcactctctccacgaattactgcagtgacctccggac   53625
FAM230B-LOC105372935-GGT2.NCBI.GGT2.rev.compl                      tgtgcagcgtcagccacttcccagcaccctctccacgaattactgcagtgacctccggac   52619
                                                                   *** *********************** ********************************

chimp.LOC112206744.LOC107973052.GGT2-.646279-708040.rev.compl      aggcccccacatgctccctgccccttacacagcaacccaaggggtccatagaccagatcc   53685
FAM230B-LOC105372935-GGT2.NCBI.GGT2.rev.compl                      aggtccccacatgctccctgccccttacacagcaatccaaggggtccatagaccagatcc   52679
                                                                   *** ****************************** *************************

chimp.LOC112206744.LOC107973052.GGT2-.646279-708040.rev.compl      atcgccttccactcacatactccacgagcccccacttccctcagacaggaagcagaggct   53745
FAM230B-LOC105372935-GGT2.NCBI.GGT2.rev.compl                      atccccttccagtcacacactccacgagcccccacttccctcagacaggaagcagaggct   52739
                                                                   *** ******* ***** ******************************************

chimp.LOC112206744.LOC107973052.GGT2-.646279-708040.rev.compl      tcaccataacctaagagatcccacacaacctgggccattccccttgggtcacttgctgca   53805
FAM230B-LOC105372935-GGT2.NCBI.GGT2.rev.compl                      tcaccataacctaagagatcccgcacaacctgggccattccccttggatcacttgctgca   52799
                                                                   ********************** ************************ ************

chimp.LOC112206744.LOC107973052.GGT2-.646279-708040.rev.compl      gcctccccagctccccacagggctctgtccctgccatcacacctggatagcagaccagga   53865
FAM230B-LOC105372935-GGT2.NCBI.GGT2.rev.compl                      gcctccccagctccccacagggctctgtccctgccatcacacctggatagcagaccagga   52859
                                                                   ************************************************************

chimp.LOC112206744.LOC107973052.GGT2-.646279-708040.rev.compl      gatagctcccctgaccccatctctgcctctgggtctttgctcagatgtccccttccctga   53925
FAM230B-LOC105372935-GGT2.NCBI.GGT2.rev.compl                      gataactcccctgaccccatctctgcctctgggtctttgctcagatgtccccttccctga   52919
                                                                   **** *******************************************************

chimp.LOC112206744.LOC107973052.GGT2-.646279-708040.rev.compl      ctaggtcaccctccatagagtcccagattttgaggccctccaggtctgtttttctacagc   53985
FAM230B-LOC105372935-GGT2.NCBI.GGT2.rev.compl                      ctaggtcaccctccatagagtcccagattttgaggccctccaggtctgtttttctacagc   52979
                                                                   ************************************************************

chimp.LOC112206744.LOC107973052.GGT2-.646279-708040.rev.compl      ctgtaacacacccgcacctgcctagtttcttttcccacctggagtgtcacagatttcatc   54045
FAM230B-LOC105372935-GGT2.NCBI.GGT2.rev.compl                      ccgtaacacacccgcacttgcctagtttctcctcccacctggagtgtcacagatttcatc   53039
                                                                   * *************** ************  ****************************

chimp.LOC112206744.LOC107973052.GGT2-.646279-708040.rev.compl      tgccgtctttgtttttcaccccagcttcaggaacaacagctgattctttaagacaatgct   54105
FAM230B-LOC105372935-GGT2.NCBI.GGT2.rev.compl                      tgccgtctttgtttttcaccccagcttcaggaacaacagctgattctttaagacaatgct   53099
                                                                   ************************************************************

chimp.LOC112206744.LOC107973052.GGT2-.646279-708040.rev.compl      caatacattctagttaaataaatgattctaagcgtccacaaggtgccaagcctgtgattc   54165
FAM230B-LOC105372935-GGT2.NCBI.GGT2.rev.compl                      caatacattctagtcaaataaatggttttaagcgtccacaaggtgccaagcctatggttc   53159
                                                                   ************** ********* ** ************************* ** ***

chimp.LOC112206744.LOC107973052.GGT2-.646279-708040.rev.compl      ccgcattgtcttaccctgagcaacttcacatctacagatgctgagtttctcaatgagtat   54225
FAM230B-LOC105372935-GGT2.NCBI.GGT2.rev.compl                      ccgcattctcttaccctcagcaacttcatgtctacagatgctgagtttctcaatgagtat   53219
                                                                   ******* ********* **********  ******************************

chimp.LOC112206744.LOC107973052.GGT2-.646279-708040.rev.compl      taaaaacaaatgaaagattggtcgggcacagtggctcacgcctgtaattccagcactttg   54285
FAM230B-LOC105372935-GGT2.NCBI.GGT2.rev.compl                      taaaaacaaatgaaagattggtcgggcacagtggctcacgcctgtaattccagcactttg   53279
                                                                   ************************************************************

chimp.LOC112206744.LOC107973052.GGT2-.646279-708040.rev.compl      ggaggctgaggcagggggatcacgaggtcaggagatcgagggaccagcctgggcaacata   54345
FAM230B-LOC105372935-GGT2.NCBI.GGT2.rev.compl                      ggaggctgaggcagggggatcacgaggtcaggagatcgagggaccagcctgggcaacata   53339
                                                                   ************************************************************

chimp.LOC112206744.LOC107973052.GGT2-.646279-708040.rev.compl      gtgaaaacccgtctctactaaaaaatacaaaaaattagccgggtgtggtggcaggcgcct   54405
FAM230B-LOC105372935-GGT2.NCBI.GGT2.rev.compl                      gtgaaaacccgtctctactaaaaaatacaaaaaattagccgggcgcggtggcgggcgcct   53399
                                                                   ******************************************* * ****** *******

chimp.LOC112206744.LOC107973052.GGT2-.646279-708040.rev.compl      gtagtcccagctactcaggaggctgaggcaggagaatggcgtgaactcaggaggcagagc   54465
FAM230B-LOC105372935-GGT2.NCBI.GGT2.rev.compl                      gtagtcccagctactcgggaggctgaggcaggagaatggcgcgaactcaggaggcagagc   53459
                                                                   **************** ************************ ******************

chimp.LOC112206744.LOC107973052.GGT2-.646279-708040.rev.compl      ttgcagtgagccgagatcgcaccactgcactccaacctgggcgacagagtaagactccgt   54525
FAM230B-LOC105372935-GGT2.NCBI.GGT2.rev.compl                      ttgcagtgagccgagatcgcaccactgcactccaacctgggcgacagagtgagactctgt   53519
                                                                   ************************************************** ****** **

chimp.LOC112206744.LOC107973052.GGT2-.646279-708040.rev.compl      ctcaaaaaaaaaaaaagaaaaaaaaaatcaaagattgagtatgttgcagaagactccaaa   54585
FAM230B-LOC105372935-GGT2.NCBI.GGT2.rev.compl                      ctcaaaaaaaaaaaaagaaaaaaaaaatcaaagattgagtatgttgcagaagactccaaa   53579
                                                                   ****************  ******************************************

chimp.LOC112206744.LOC107973052.GGT2-.646279-708040.rev.compl      gggcaccacccaggacccccacctgaagtctaagacctgctgtggtaagtgtgtcctgcc   54645
FAM230B-LOC105372935-GGT2.NCBI.GGT2.rev.compl                      gggcaccacccaggacccccacctgaagtctaagacctgctatggtgagtgtgtcctgcc   53639
                                                                   ***************************************** **** ************
```

```
chimp.LOC112206744.LOC107973052.GGT2-.646279-708040.rev.compl      cctccatcctccaac---tttttttttttttttttttgagacggagcttcgttcttgtt   54702
FAM230B-LOC105372935-GGT2.NCBI.GGT2.rev.compl                      cctccatcctccaacttttttttttttttttttttttgagacggagcttcgttcttgtt   53699
                                                                   ***************   ******************************************

chimp.LOC112206744.LOC107973052.GGT2-.646279-708040.rev.compl      gcccaggctggagtgcagtggcatgatctcggctcactgcaatctccacctcgtgggttc   54762
FAM230B-LOC105372935-GGT2.NCBI.GGT2.rev.compl                      gcccaggctggagtgcagtggcatgatctcggctcactgcaatctctacctcgtgggttc   53759
                                                                   ********************************************** *************

chimp.LOC112206744.LOC107973052.GGT2-.646279-708040.rev.compl      aagcgattctcctgcctcagcctcctgagtagctgggatattacaggcctgtgccaccac   54822
FAM230B-LOC105372935-GGT2.NCBI.GGT2.rev.compl                      aagcgattctcctgccttagcctcctgagtagctgggatattacaggcctgtgccaccac   53819
                                                                   ***************** ******************************************

chimp.LOC112206744.LOC107973052.GGT2-.646279-708040.rev.compl      gcccaactaattattatacttttagtagagaaagggtttcactatgttggccaggctggt   54882
FAM230B-LOC105372935-GGT2.NCBI.GGT2.rev.compl                      gcccgactaattattgtacttttagtagagaaagggtttcactatgttggccaggctggt   53879
                                                                   **** ********** ********************************************

chimp.LOC112206744.LOC107973052.GGT2-.646279-708040.rev.compl      cttgaactcctgacctaggtgatccatctgcctcagcctcccaaagtgctgggattacag   54942
FAM230B-LOC105372935-GGT2.NCBI.GGT2.rev.compl                      cttggactcctgacctaggtgatccatctgcctcagcctcccaaagtgctgggattacag   53939
                                                                   **** *******************************************************

chimp.LOC112206744.LOC107973052.GGT2-.646279-708040.rev.compl      gcgtgagcctgtgaaaaaaaggcccagccttttttttttttttttttttttgacagcgtc   55002
FAM230B-LOC105372935-GGT2.NCBI.GGT2.rev.compl                      gcgtgagcctgtgaaaaaaaggcccagcc----ttttttttttttttttttgacagggtc   53995
                                                                   *****************************    *********************** ***

chimp.LOC112206744.LOC107973052.GGT2-.646279-708040.rev.compl      tcactttgttgcccaagctagagtgtagtggtataatcatggctgactgcagcctcaacc   55062
FAM230B-LOC105372935-GGT2.NCBI.GGT2.rev.compl                      tcactttgttgcccaagctagagtgtagtggtataatcatggctgactgcagcctcaacc   54055
                                                                   ************************************************************

chimp.LOC112206744.LOC107973052.GGT2-.646279-708040.rev.compl      tcctgggctcaagtgatcctcccaccttagcctcccgagtagctgggaccataagcacac   55122
FAM230B-LOC105372935-GGT2.NCBI.GGT2.rev.compl                      tcctgggctcaagtgatcctcccaccttagcctcccgagtaactgggaccataagcacac   54115
                                                                   ***************************************** ******************

chimp.LOC112206744.LOC107973052.GGT2-.646279-708040.rev.compl      accgccatacctagctaattttttttccattttttgtagagatggagtcttgctatgttg   55182
FAM230B-LOC105372935-GGT2.NCBI.GGT2.rev.compl                      accgccatacctgctaattttttttttccattttttgtagagatggagtcttgctatgttg   54175
                                                                   ************    * ******************************************

chimp.LOC112206744.LOC107973052.GGT2-.646279-708040.rev.compl      tccaggcaggtctcctgggctcatgcgctcctcctgacttggcctcctaaagtgctagga   55242
FAM230B-LOC105372935-GGT2.NCBI.GGT2.rev.compl                      tccaggcaggtctcctgggctcatgcgctcctcctgacttggcctcctaaagtgctagga   54235
                                                                   ************************************************************

chimp.LOC112206744.LOC107973052.GGT2-.646279-708040.rev.compl      ttagaggcgtgagttgttgagaccctcccatcctccaacttttatctcacaatctattgt   55302
FAM230B-LOC105372935-GGT2.NCBI.GGT2.rev.compl                      ttagaggcgtgagttgttgagaccctcccatcctccaacttttatctcacaatctattgt   54295
                                                                   ************************************************************

chimp.LOC112206744.LOC107973052.GGT2-.646279-708040.rev.compl      gcctcctttggggacagacagtggcttcctggatggacagtggcttcccttcaggtacct   55362
FAM230B-LOC105372935-GGT2.NCBI.GGT2.rev.compl                      gcctcctttggggacagacagtggcttcctggatggacagtggcttcccctcaggtacct   54355
                                                                   ************************************************* **********

chimp.LOC112206744.LOC107973052.GGT2-.646279-708040.rev.compl      ggggaatttggggggcctcctctccacttaagaccagattagaaaagagggactccacctc   55422
FAM230B-LOC105372935-GGT2.NCBI.GGT2.rev.compl                      ggggaatttggggggcctcctccccacttaagaccagattagaaaagagagactccacctc   54415
                                                                   ******************** *************************** ***********

chimp.LOC112206744.LOC107973052.GGT2-.646279-708040.rev.compl      acattctagagcaccatccccacaaatgaacaaatgagtgaatgggatgcctgttgaaaa   55482
FAM230B-LOC105372935-GGT2.NCBI.GGT2.rev.compl                      acattctagagcgccatccccacaaatgaacaaatgagtgaatgggatgcctgttgaaaa   54475
                                                                   ************ ***********************************************

chimp.LOC112206744.LOC107973052.GGT2-.646279-708040.rev.compl      agcaggatatagacagcctgggttcaattttagctttaccacctcccagctgtgtgacct   55542
FAM230B-LOC105372935-GGT2.NCBI.GGT2.rev.compl                      ggcaggatatagacagcctgggttcaattttagcttcaccacctcccagctgtgtgacct   54535
                                                                    *********************************** ***********************

chimp.LOC112206744.LOC107973052.GGT2-.646279-708040.rev.compl      caactgatttgcatgacctgtctgagcctcagcatccccaccctgtaaaatgggaatcca   55602
FAM230B-LOC105372935-GGT2.NCBI.GGT2.rev.compl                      cagctgatttgcatgacctgtctgagcctcagcatccccaccctgtaaaatgggaatcca   54595
                                                                   ** *********************************************************

chimp.LOC112206744.LOC107973052.GGT2-.646279-708040.rev.compl      cacagcatcccctagcccaaaggagcagggagggttgtgagaggctcgtgggtgaaaagc   55662
FAM230B-LOC105372935-GGT2.NCBI.GGT2.rev.compl                      cacagcatcccctagcccaaaggagcagggagggttgtgagaggctcgtgggtgaaaagc   54655
                                                                   ************************************************************

chimp.LOC112206744.LOC107973052.GGT2-.646279-708040.rev.compl      acagagcagagcgtgggccccagtgagccctggtccatgaggtctgctagcataataatt   55722
FAM230B-LOC105372935-GGT2.NCBI.GGT2.rev.compl                      acagagcagagcatgggccccagtgagccctgatccatgaggtctgctagcataataatt   54715
                                                                   ************ ******************* ***************************

chimp.LOC112206744.LOC107973052.GGT2-.646279-708040.rev.compl      attctttccatgtgctgcacagagtggccccggaggccttagcagaaataacagaagctc   55782
FAM230B-LOC105372935-GGT2.NCBI.GGT2.rev.compl                      attctttccatgtgctgcacagagtggccccggaggccttagcagaaataacagaagctc   54775
                                                                   ************************************************************

chimp.LOC112206744.LOC107973052.GGT2-.646279-708040.rev.compl      ccggccctttaccgtggtgatgatggtcctgaccactcattgtgggagggtgctatgggg   55842
FAM230B-LOC105372935-GGT2.NCBI.GGT2.rev.compl                      ccggccctttaccgtggtgatgatggtcctgaccactcattgtgggagggtgctatgggg   54835
                                                                   ************************************************************

chimp.LOC112206744.LOC107973052.GGT2-.646279-708040.rev.compl      caaggaagggatgggggtgctagaactgcccctgaaccctgacgggagcaggctcctgt   55902
FAM230B-LOC105372935-GGT2.NCBI.GGT2.rev.compl                      ccaggaagggatgggggtgacagaactgcccctgaaccctgacgggagcaggcttctgt   54895
                                                                   * ******************  *********************************** ****

chimp.LOC112206744.LOC107973052.GGT2-.646279-708040.rev.compl      gggcaaggccccttcccggtggctcagccagctctgcacccatgccccaagtctgcagca   55962
FAM230B-LOC105372935-GGT2.NCBI.GGT2.rev.compl                      gggcaaggccccttcccggtggctcagccagctctgcacccatgccccaagtctgcagca   54955
                                                                   ************************************************************

chimp.LOC112206744.LOC107973052.GGT2-.646279-708040.rev.compl      tggcttacccttctgggactgctccgagctgttgaacatgctggcaaaggccagcctggg   56022
FAM230B-LOC105372935-GGT2.NCBI.GGT2.rev.compl                      tggcttacccttctgggactgctccgagctgttgaacatgctggcaaaggccagcctggg   55015
                                                                   ************************************************************

chimp.LOC112206744.LOC107973052.GGT2-.646279-708040.rev.compl      ggccacctcgcgggcgttgatgacctcagcttttcctagaaggagaagcaggtaggcagg   56082
FAM230B-LOC105372935-GGT2.NCBI.GGT2.rev.compl                      ggccacctcgcgggcattgatgacctcagcttttcctaga---agaagcaggtaggcagg   55072
                                                                   *************** ************************   *****************

chimp.LOC112206744.LOC107973052.GGT2-.646279-708040.rev.compl      cccacccacccaaaccctttatgccacgtgagcctgggggccacccagctgtgcctcggc   56142
FAM230B-LOC105372935-GGT2.NCBI.GGT2.rev.compl                      cccacccaccaaaaccctttatgccacgtgagcctgggggccacccagctgtgcctcggc   55132
                                                                   ********** *************************************************

chimp.LOC112206744.LOC107973052.GGT2-.646279-708040.rev.compl      ccaacccacacccccctgcccctctccctctcctcttccgaggcactcatgagtggtgctg   56202
FAM230B-LOC105372935-GGT2.NCBI.GGT2.rev.compl                      ccaacccacacccccctgcccctctccctctcctcttctgaggcactcatgagtggtgctg   55192
                                                                   ************************************* **********************

chimp.LOC112206744.LOC107973052.GGT2-.646279-708040.rev.compl      ttgtagatggtgaggaagaggccacccccgatgcccatgctgtgggcattcatgagcccc   56262
FAM230B-LOC105372935-GGT2.NCBI.GGT2.rev.compl                      ttgtagatggtgaggaagaggccaaccccgatgcccatgctgtgggcattcatgagcccc   55252
                                                                   ************************ ***********************************

chimp.LOC112206744.LOC107973052.GGT2-.646279-708040.rev.compl      acacacaacagggctgcaatggctgcatccaccgcagagccaccgtcccgcagtgcgtcc   56322
FAM230B-LOC105372935-GGT2.NCBI.GGT2.rev.compl                      acacacaacagggctgcaatggctgcatccactgcagagccaccgtcccgcagtgtgtcc   55312
                                                                   ******************************** ********************** ****

chimp.LOC112206744.LOC107973052.GGT2-.646279-708040.rev.compl      ctgccatgtggcacataaaggcatgagaacctgcaggcttccaccctggccccgcataca   56382
FAM230B-LOC105372935-GGT2.NCBI.GGT2.rev.compl                      ctgccatgtggcacataaaggcatgagaacctgcaggcttccaccctggccccgcataca   55372
                                                                   ************************************************************

chimp.LOC112206744.LOC107973052.GGT2-.646279-708040.rev.compl      caccctgctgcccacctgcccaaaggaggatggaagagaagtccattcgagttttggggt   56442
FAM230B-LOC105372935-GGT2.NCBI.GGT2.rev.compl                      caccctgctgcccacctgcccaaaggaggatggaagagaagtccattcgagttttggggt   55432
                                                                   ************************************************************

chimp.LOC112206744.LOC107973052.GGT2-.646279-708040.rev.compl      ttttgtttttagtttctttctcttttttttgagatggagtcttgctctattgccaggctg   56502
FAM230B-LOC105372935-GGT2.NCBI.GGT2.rev.compl                      ttttgtttttagtttctttctcttttttttgagatggagtcttgctctattgccaggctg   55492
                                                                   ************************************************************

chimp.LOC112206744.LOC107973052.GGT2-.646279-708040.rev.compl      gagtgcagtggcacgatctcagctcactgcaacctctgcctcccgggttcaagccattct   56562
FAM230B-LOC105372935-GGT2.NCBI.GGT2.rev.compl                      gagtgcagtggcacgatctcagctcactgcaacctctgcctcccgggttcaagccattct   55552
                                                                   ************************************************************

chimp.LOC112206744.LOC107973052.GGT2-.646279-708040.rev.compl      cctgcctcagcctcctgagtagctgggactacaggcgcacaccaccacgcccggctaatt   56622
FAM230B-LOC105372935-GGT2.NCBI.GGT2.rev.compl                      cctgcctcagcctcctgagtagctgggactacaggtgcatgccaccacgcccagctaatt   55612
                                                                   *********************************** ***  *********** *******

chimp.LOC112206744.LOC107973052.GGT2-.646279-708040.rev.compl      tttgtattttagtagagacggggtttcaccatgttggccaggatggtctctatctcttg   56682
FAM230B-LOC105372935-GGT2.NCBI.GGT2.rev.compl                      tttgtattttagtagagatggggtttcaccatgttggccaggatggtctctatctcttg   55672
                                                                   ****************** *****************************************

chimp.LOC112206744.LOC107973052.GGT2-.646279-708040.rev.compl      acttcatgatccacctgccttggcctc--------tttttttttttttttggagacaaag   56734
FAM230B-LOC105372935-GGT2.NCBI.GGT2.rev.compl                      acttcatgatccgcctgccttggcctcttttttttttttttttttttttggagacaaag   55732
                                                                   ************ **************        *************************

chimp.LOC112206744.LOC107973052.GGT2-.646279-708040.rev.compl      tctctctttgttgctcaggctggagtgcagtggtgtgatctcggatcactgcagcctcag   56794
FAM230B-LOC105372935-GGT2.NCBI.GGT2.rev.compl                      tctctctttgttgctcaggctggagtgcagtggtgtgatcttggatcactgcagcctcag   55792
                                                                   ***************************************** ******************

chimp.LOC112206744.LOC107973052.GGT2-.646279-708040.rev.compl      tctcctgggctcaattgaccctcccatctcagcctcccaagtagctgggactatgggcac   56854
```

```
FAM230B-LOC105372935-GGT2.NCBI.GGT2.rev.compl                      tctcctgggctcaattgaccctcccatctcagcctcccaagtagctgggactatgggcac   55852
                                                                   ************************************************************

chimp.LOC112206744.LOC107973052.GGT2-.646279-708040.rev.compl      atgccaccatgcccagccaat----tttgtttgtgtgttttgtagagatggggtttcatc   56910
FAM230B-LOC105372935-GGT2.NCBI.GGT2.rev.compl                      atgccaccatgcccagccaattttgtttgtttgtgtattttgtagagatggggtttcatc   55912
                                                                   *********************    *********** ***********************

chimp.LOC112206744.LOC107973052.GGT2-.646279-708040.rev.compl      atgttgcccaggctggtcaagaactcctgtgctcaagtgatccacccaccttggcctccc   56970
FAM230B-LOC105372935-GGT2.NCBI.GGT2.rev.compl                      atgttgcccaggctggtcaagaactcctgtgctcaagtaatccacccaccttggcctccc   55972
                                                                   ************************************** *********************

chimp.LOC112206744.LOC107973052.GGT2-.646279-708040.rev.compl      aaagtgctggtattacaggcatgagccactgtgctcagcctttgttttatgagacagggt   57030
FAM230B-LOC105372935-GGT2.NCBI.GGT2.rev.compl                      aaagtgctggtattacaggcatgagccactgtgcccagcctttgttttatgagacagggt   56032
                                                                   ********************************** *************************

chimp.LOC112206744.LOC107973052.GGT2-.646279-708040.rev.compl      ctcactttgtcacccaggatgaagtgcagtggcacagtcttggctcaatgcagctttgac   57090
FAM230B-LOC105372935-GGT2.NCBI.GGT2.rev.compl                      ctcactttgtcacccaggatgaagtgcagtggcacagtcttggctcaatgcagctttgac   56092
                                                                   ************************************************************

chimp.LOC112206744.LOC107973052.GGT2-.646279-708040.rev.compl      ctcctgggctcaagcaatcctcccacttcagtctcctgagtagctgggactacaggtaag   57150
FAM230B-LOC105372935-GGT2.NCBI.GGT2.rev.compl                      ctcctgggctcaagcaatcctcccacttcagtctcctgagtagctgggactacaggtaag   56152
                                                                   ************************************************************

chimp.LOC112206744.LOC107973052.GGT2-.646279-708040.rev.compl      aaccaccacaccgggcaatttttgtctttttgtagagatagggtctttctatgttgcc   57210
FAM230B-LOC105372935-GGT2.NCBI.GGT2.rev.compl                      aaccaccacaccgggcaatttttgtctttttgtagagatagggtctttctatgttgcc   56212
                                                                   ************************************************************

chimp.LOC112206744.LOC107973052.GGT2-.646279-708040.rev.compl      caggctggtctcaaactcatggtctaaagcaatcttatctcctcaacctcccaaagtgct   57270
FAM230B-LOC105372935-GGT2.NCBI.GGT2.rev.compl                      caggctggtctcaaactcatggtctaaagcaatcctatcgcctcaacctcccaaagtgct   56272
                                                                   ********************************** **** ********************

chimp.LOC112206744.LOC107973052.GGT2-.646279-708040.rev.compl      gggattacagtttcttctttttcttttc----------ttttttttttctgagacagagt   57320
FAM230B-LOC105372935-GGT2.NCBI.GGT2.rev.compl                      gggattacagtttcttctttttcttttctttttcttttttttttttttctgagacagagt   56332
                                                                   ****************************          **********************

chimp.LOC112206744.LOC107973052.GGT2-.646279-708040.rev.compl      ttcactcagttgcccaggctggagtgcagtggcatgatcttagctcactgcaacctctgc   57380
FAM230B-LOC105372935-GGT2.NCBI.GGT2.rev.compl                      ttcactcagttgcccaggctggagtgcagtggcatgatcttagctcactgcaacctctgc   56392
                                                                   ************************************************************

chimp.LOC112206744.LOC107973052.GGT2-.646279-708040.rev.compl      ctcctgggttcaagcgattctcctgcctcagcctcctgagtagctgggattacaggcgca   57440
FAM230B-LOC105372935-GGT2.NCBI.GGT2.rev.compl                      ctcctgggttcaagcgattctcctgcctcagcctcctgagtagctgggattacaggcgca   56452
                                                                   ************************************************************

chimp.LOC112206744.LOC107973052.GGT2-.646279-708040.rev.compl      caccaccatgcccggctaacttttatatttttagtagggacagagtgcaccatgttggc   57500
FAM230B-LOC105372935-GGT2.NCBI.GGT2.rev.compl                      caccaccatgcccggctaacttttatatttttagtagggacagagtgcaccatgttggc   56512
                                                                   ************************************************************

chimp.LOC112206744.LOC107973052.GGT2-.646279-708040.rev.compl      caggctggtctcgaactcctgacctcgggtggtctgcccgcctcaaccccccaaagtgct   57560
FAM230B-LOC105372935-GGT2.NCBI.GGT2.rev.compl                      caggctgatctcgaactcctgacctc-agtggtctgcccgcctcaaccccccaaagtgct   56571
                                                                   ******* ******************  ********************************

chimp.LOC112206744.LOC107973052.GGT2-.646279-708040.rev.compl      gggattactggtgtggaccaccttgcccagacagtttcctctttatcaagcaaacaaatg   57620
FAM230B-LOC105372935-GGT2.NCBI.GGT2.rev.compl                      gggattacaggtgtggaccaccttgcccagacagtttcctctttattaagcaaacaaatg   56631
                                                                   ******** ************************************* *************

chimp.LOC112206744.LOC107973052.GGT2-.646279-708040.rev.compl      tacatgacttttataattgggacaaaaagggaaattgctatactttattaataacatttt   57680
FAM230B-LOC105372935-GGT2.NCBI.GGT2.rev.compl                      tacatgacttttataattgggacaaaaagggaaattgctatactttattaataacatttt   56691
                                                                   ************************************************************

chimp.LOC112206744.LOC107973052.GGT2-.646279-708040.rev.compl      ttttttccttgctagagatggtggcttacacctgtaatctcagcactctgggaggccaag   57740
FAM230B-LOC105372935-GGT2.NCBI.GGT2.rev.compl                      ttttttccctgctagagatggtggcttacacctgtaatctcagcactttgggaggccaag   56751
                                                                   ******** ************************************** ************

chimp.LOC112206744.LOC107973052.GGT2-.646279-708040.rev.compl      gtggaggatcacttgaggccaggagttcaagaccagcctgggcaatagagtgagaccatc   57800
FAM230B-LOC105372935-GGT2.NCBI.GGT2.rev.compl                      gtggaggatcacttgaggccaggagttcaagaccagcctgggcaatagagtgagaccatc   56811
                                                                   ************************************************************

chimp.LOC112206744.LOC107973052.GGT2-.646279-708040.rev.compl      tctagaaaacagtcttttttaattagtcaggtgtgatgcacgcctgtagtcctagctact   57860
FAM230B-LOC105372935-GGT2.NCBI.GGT2.rev.compl                      tctacaaaacagtcttttttaattagtcaggtatgatgcacgcctgtagtcctagctact   56871
                                                                   **** *************************** ***************************

chimp.LOC112206744.LOC107973052.GGT2-.646279-708040.rev.compl      caggggctgaggtgagaggatcgcttaagcccaagagttcaaggctgcagtgagctatg   57920
FAM230B-LOC105372935-GGT2.NCBI.GGT2.rev.compl                      caggggctgaggtgagaggatcgcttaagcccaagagttcaaggctgcagtgagctatg   56931
                                                                   ************************************************************

chimp.LOC112206744.LOC107973052.GGT2-.646279-708040.rev.compl      atcatgccactgcactccagcctgggtgacagaacaagaccctgtctcaaaatatgaaaa   57980
FAM230B-LOC105372935-GGT2.NCBI.GGT2.rev.compl                      atcatgccactgcactccagcctgggtgacagaacaagaccctgtctcaaaatatgaaaa   56991
                                                                   ************************************************************

chimp.LOC112206744.LOC107973052.GGT2-.646279-708040.rev.compl      acataatattttttctgtttaagtctttaagagggaactgatctttatatataacatgag   58040
FAM230B-LOC105372935-GGT2.NCBI.GGT2.rev.compl                      acataatattttttctgtttaagtctttaggagggaacttatctttatatataacatgag   57051
                                                                   ***************************** ********* ********************

chimp.LOC112206744.LOC107973052.GGT2-.646279-708040.rev.compl      ataagagtctaaaatagaataaaacagtagaagccaggcaccatggctcacacctgtaat   58100
FAM230B-LOC105372935-GGT2.NCBI.GGT2.rev.compl                      ataagagtctaaaatagaataacacagtagaagccaggcaccatggctcacacctgtaat   57111
                                                                   ********************** *************************************

chimp.LOC112206744.LOC107973052.GGT2-.646279-708040.rev.compl      cccagcactttaggaagctgaggcgggaggatcacttaaacccaagagtttgaggctgca   58160
FAM230B-LOC105372935-GGT2.NCBI.GGT2.rev.compl                      cccagcactttaggaagctgaggcgggaggatcacttaaacccaagagtttgaggctgca   57171
                                                                   ************************************************************

chimp.LOC112206744.LOC107973052.GGT2-.646279-708040.rev.compl      gtgggctatgatcgctcccctatactccagcctgggtgacagagtaagactccaccttaa   58220
FAM230B-LOC105372935-GGT2.NCBI.GGT2.rev.compl                      gtgggctatgatcgctcccctatactccagcctgggtgacagagtaagactccaccttaa   57231
                                                                   ************************************************************

chimp.LOC112206744.LOC107973052.GGT2-.646279-708040.rev.compl      aaaaagaataagcccttcatgtccctgtttgggcaacagcgttcctt-------gggggg   58273
FAM230B-LOC105372935-GGT2.NCBI.GGT2.rev.compl                      aaaaagaataagcccttcatgtccctgtttgggcaacagcgttccttgggggaggggggg   57291
                                                                   ***********************************************       ******

chimp.LOC112206744.LOC107973052.GGT2-.646279-708040.rev.compl      atgggggagggatggcattgggagatatacataatgctaaatgatgagttggtgggtgca   58333
FAM230B-LOC105372935-GGT2.NCBI.GGT2.rev.compl                      aggggggagggatggcattgggagatatacctaatgctaaatgacgagttggtgggtgca   57351
                                                                   * **************************** ************* ***************

chimp.LOC112206744.LOC107973052.GGT2-.646279-708040.rev.compl      gcacaccaacatggcacatgtgtacatatgtaacaaacctgcacattgtgcacatgtacc   58393
FAM230B-LOC105372935-GGT2.NCBI.GGT2.rev.compl                      gcacaccaacatggcacatgtgtacatatgtaacaaacctgcacattgtgcacatgtacc   57411
                                                                   ************************************************************

chimp.LOC112206744.LOC107973052.GGT2-.646279-708040.rev.compl      ctaaaacttaaagtataataataaaataaaataaaaaaaaaaagaaagaaagaaattgtc   58453
FAM230B-LOC105372935-GGT2.NCBI.GGT2.rev.compl                      ctaaaacttaaagt---ataataaaataaaataaaaaaaaaaagaaagaaagaaattgtc   57468
                                                                   **************   *******************************************

chimp.LOC112206744.LOC107973052.GGT2-.646279-708040.rev.compl      tcctaaccaaaaaaaaaaaaaaaa-----aaaagaaaagaaaagaataagccgggcgtgg   58508
FAM230B-LOC105372935-GGT2.NCBI.GGT2.rev.compl                      tcctaaccaaaaaaaaaaaaaaaaaaaagaaaagaaaagaaaagaataagccgggcgtgg   57528
                                                                   ************************     *******************************

chimp.LOC112206744.LOC107973052.GGT2-.646279-708040.rev.compl      tggctcacacctgtaatcccaacacttcgggaggcccaggtgggtggattacctgaggtc   58568
FAM230B-LOC105372935-GGT2.NCBI.GGT2.rev.compl                      tggctcatacctgtaatcccaacactttgggaggcccaggtgggtggattacctgaggtc   57588
                                                                   ******* ******************* ********************************

chimp.LOC112206744.LOC107973052.GGT2-.646279-708040.rev.compl      aggagttcgagaccagcctgaccaacatggtgaaaccccatctctactaaaaacaccata   58628
FAM230B-LOC105372935-GGT2.NCBI.GGT2.rev.compl                      aggagttcgagaccagcctgaccaacatggtgaaaccccatctctactaaaaacaccata   57648
                                                                   ************************************************************

chimp.LOC112206744.LOC107973052.GGT2-.646279-708040.rev.compl      attaggccaggcgcagtggctcacacctgtaatcccagcactttgagaggcagaggtggg   58688
FAM230B-LOC105372935-GGT2.NCBI.GGT2.rev.compl                      attaggccaggcgcagtggctcacacctgtaatcccagcactttgagaggcagaggtggg   57708
                                                                   ************************************************************

chimp.LOC112206744.LOC107973052.GGT2-.646279-708040.rev.compl      aggatcacaaggtcaggagttcaagaccagcctggccaacatagcaaaaccctgtctcta   58748
FAM230B-LOC105372935-GGT2.NCBI.GGT2.rev.compl                      aggatcacaaggtcaggagttcaagaccagcctggccaacatagcaaaaccctgtctcta   57768
                                                                   ************************************************************

chimp.LOC112206744.LOC107973052.GGT2-.646279-708040.rev.compl      ctaaaaatacaaaaattagctgggcatggtggcacatgcctgtagtctcagctactgtgg   58808
FAM230B-LOC105372935-GGT2.NCBI.GGT2.rev.compl                      ctaaaaatacaaaaattagctgggcatggtggcacatgcctgtagtctcagctactgtgg   57828
                                                                   ************************************************************

chimp.LOC112206744.LOC107973052.GGT2-.646279-708040.rev.compl      aggctgaggcaggagaatcacttgaacctgggaggtggaggctgcagtaagccaagattg   58868
FAM230B-LOC105372935-GGT2.NCBI.GGT2.rev.compl                      aggctgaggcaggagaatcacttgaacctgggaggtggaggctgcagtaagccaagattg   57888
                                                                   ************************************************************

chimp.LOC112206744.LOC107973052.GGT2-.646279-708040.rev.compl      caccactgccctccagcctgggtaacagagcgatactctgtctcagaaa-----------   58917
FAM230B-LOC105372935-GGT2.NCBI.GGT2.rev.compl                      caccactgccctccagcctgggtaacagagcgatactctgtctcagaaaacacacacaca   57948
                                                                   *************************************************

chimp.LOC112206744.LOC107973052.GGT2-.646279-708040.rev.compl      ---acacacacacacacacacacacatatacacacacacaaatttgttgggcgtggtg   58974
FAM230B-LOC105372935-GGT2.NCBI.GGT2.rev.compl                      cacacacacacacacacacacacacacatatacacacacacaaatttgttgggcgtggtg   58008
```

```
                                                                      *********************************************************

chimp.LOC112206744.LOC107973052.GGT2-.646279-708040.rev.compl      gcacactcctgtaatcccagctacttgggaggcttaggcatgagaatcacttgaacttgg   59034
FAM230B-LOC105372935-GGT2.NCBI.GGT2.rev.compl                      gcgcactcctataatcccagctacttgggaggcttaggcatgagaatcgcttgaacttgg   58068
                                                                   ** ******* ************************************* ***********

chimp.LOC112206744.LOC107973052.GGT2-.646279-708040.rev.compl      gaggcggaggttgcagttaactgagattgcaccactgcactccagcctggcaacagagca   59094
FAM230B-LOC105372935-GGT2.NCBI.GGT2.rev.compl                      gaggcggaggttgcagttaactgagattgcaccactgcactccagcctggcaacagagca   58128
                                                                   ************************************************************

chimp.LOC112206744.LOC107973052.GGT2-.646279-708040.rev.compl      aaactccatctctaaatagat-------------tagatagatagatagatagatagata   59141
FAM230B-LOC105372935-GGT2.NCBI.GGT2.rev.compl                      aaactccatctctaaatagatatagatagatagatagatagatagatagatagatagata   58188
                                                                   *********************             **************************

chimp.LOC112206744.LOC107973052.GGT2-.646279-708040.rev.compl      atagatagatagatagatagatagttagataggaagaaataagtaaaaataacaaccaaa   59201
FAM230B-LOC105372935-GGT2.NCBI.GGT2.rev.compl                      gatagatgatagatagatagatagatagataggaagaaataagtaaaaataacaaccaaa   58248
                                                                          ***************** ***********************************

chimp.LOC112206744.LOC107973052.GGT2-.646279-708040.rev.compl      caacaaaacagtggagtttatccaaagagacagagactcttagaactgagaaaaggggcc   59261
FAM230B-LOC105372935-GGT2.NCBI.GGT2.rev.compl                      caacaaaacagtggagtttatccaaagaaacagagactcttagaactgagaaaaggggcc   58308
                                                                   **************************** *******************************

chimp.LOC112206744.LOC107973052.GGT2-.646279-708040.rev.compl      ctgtttggctctaggagacccacaccctgctctcggagtcaccgtccccttcccaaaggc   59321
FAM230B-LOC105372935-GGT2.NCBI.GGT2.rev.compl                      ctgtttggctcta-gagacccacaccctgctctcggagtcaccgtccccttcccaaaggc   58367
                                                                   ************* **********************************************

chimp.LOC112206744.LOC107973052.GGT2-.646279-708040.rev.compl      tactgagagagtccaagcaaagcttacatgtggggaaactgagtctcagaggggtgaagg   59381
FAM230B-LOC105372935-GGT2.NCBI.GGT2.rev.compl                      tactgagagagtccaagcgaagcttgcatgtggggaaactgagtctcagaggggtgaagg   58427
                                                                   ****************** ****** **********************************

chimp.LOC112206744.LOC107973052.GGT2-.646279-708040.rev.compl      tattgctcaggtccacttgcccagttttcagggcccatgtcccatgccctgccccgctca   59441
FAM230B-LOC105372935-GGT2.NCBI.GGT2.rev.compl                      tattgctcaggtccacttgcccagttttcagggcccatgtcccatgccctgccccgctca   58487
                                                                   ************************************************************

chimp.LOC112206744.LOC107973052.GGT2-.646279-708040.rev.compl      cctcccaatctccgagcactgcttggcatccgcgcccacggcagccctggtgtacacatg   59501
FAM230B-LOC105372935-GGT2.NCBI.GGT2.rev.compl                      cctcccaatctccaagcactgcttggcatccgcggccatggcagccctggtgtacacatg   58547
                                                                   ************* ******************** *** *********************

chimp.LOC112206744.LOC107973052.GGT2-.646279-708040.rev.compl      gttgtcaggttccttggaggctgagggcagccagagacagaggccgacaatgaccagcac   59561
FAM230B-LOC105372935-GGT2.NCBI.GGT2.rev.compl                      gttgtcaggttccttggaggccgagggcagccagagacagaggccgacaatgaccagcac   58607
                                                                   ********************* **************************************

chimp.LOC112206744.LOC107973052.GGT2-.646279-708040.rev.compl      gaggaccacggccagcaggcccagcaccactaacttcttcttcatggctctgctgcaccc   59621
FAM230B-LOC105372935-GGT2.NCBI.GGT2.rev.compl                      caggaccacggccagcaggcccagcaccactaacttcttcttcatggctctgctgcaccc   58667
                                                                    ***********************************************************

chimp.LOC112206744.LOC107973052.GGT2-.646279-708040.rev.compl      acggggtaaggagcagggtcaggcccagcctcagacattccctggcccctccccaacagg   59681
FAM230B-LOC105372935-GGT2.NCBI.GGT2.rev.compl                      acggggtaaggagcagggtcaggcccagcctcagacatgccctggcccctccccaacagg   58727
                                                                   ************************************** *********************

chimp.LOC112206744.LOC107973052.GGT2-.646279-708040.rev.compl      gcacagtctaaagtcaggcctcagaaacacaaggcctgtgtctccttcccgcttcccaga   59741
FAM230B-LOC105372935-GGT2.NCBI.GGT2.rev.compl                      gcacagtctaaagtcgggcctcagaaacacaaggccggagtctccttcccgcttcccaga   58787
                                                                   *************** ******************** * *********************

chimp.LOC112206744.LOC107973052.GGT2-.646279-708040.rev.compl      atacgtgcaggctgtccggcccccagacctttgcgcaggccatgccctctgccagaagct   59801
FAM230B-LOC105372935-GGT2.NCBI.GGT2.rev.compl                      atacgtgcaggctgtccggcccccagacctttgcgcaggccatgccctctgccagaagct   58847
                                                                   ************************************************************

chimp.LOC112206744.LOC107973052.GGT2-.646279-708040.rev.compl      ctgggcctcatctctgccctcccaaatcctccctgcttatcttcagagcccatcctggta   59861
FAM230B-LOC105372935-GGT2.NCBI.GGT2.rev.compl                      ctgggcctcatctctgccctcccaaatcctccctgcttatcttcagagcccatcctggta   58907
                                                                   ************************************************************

chimp.LOC112206744.LOC107973052.GGT2-.646279-708040.rev.compl      agaaccccatctccaccagcggcccttcctgggagcccccagatttccacacccctcttt   59921
FAM230B-LOC105372935-GGT2.NCBI.GGT2.rev.compl                      agaaccccatctccagcagcggcccttcctgggagcccccagatttccacacccctcttt   58967
                                                                   *************** ********************************************

chimp.LOC112206744.LOC107973052.GGT2-.646279-708040.rev.compl      ctgcagggcctggcctacctcctcacagtggctgagcctccactgcttagggagaagctc   59981
FAM230B-LOC105372935-GGT2.NCBI.GGT2.rev.compl                      ctgcagggcctggcctacctcctcacagtggctgagcctccactgcttagggagaagctc   59027
                                                                   ************************************************************

chimp.LOC112206744.LOC107973052.GGT2-.646279-708040.rev.compl      cagtagggatgggcctggcctggtttctcctgtgtcccccacctcagcctagagcctggc   60041
FAM230B-LOC105372935-GGT2.NCBI.GGT2.rev.compl                      cagcagggatgggcctggcctggtttctcctgcgtcccccaccccagcctagagcctggc   59087
                                                                   *** **************************** ********** ****************

chimp.LOC112206744.LOC107973052.GGT2-.646279-708040.rev.compl      actgtccaggagtcctctgaagaccctccaccccacctggagcatggggtttagcttcca   60101
FAM230B-LOC105372935-GGT2.NCBI.GGT2.rev.compl                      actgtccaggagtcctctgaagaccctccaccccacctggagcatggggtttagcttcca   59147
                                                                   ************************************************************

chimp.LOC112206744.LOC107973052.GGT2-.646279-708040.rev.compl      tagtgcccacaatcagagcgccccacagattcactgccacggggccaggacttaccgtcc   60161
FAM230B-LOC105372935-GGT2.NCBI.GGT2.rev.compl                      tagtgcccacaatcagagcgccccacagattcactgccacggggccaggacttaccgtcc   59207
                                                                   ************************************************************

chimp.LOC112206744.LOC107973052.GGT2-.646279-708040.rev.compl      agcagcagacaggggccccaagccttgcctggggtgttggccacgaaagacaggaggatt   60221
FAM230B-LOC105372935-GGT2.NCBI.GGT2.rev.compl                      agcagcagacgggggccccaagccttgcctggggtgttggccacgaaagacaggaggatt   59267
                                                                   ********** *************************************************

chimp.LOC112206744.LOC107973052.GGT2-.646279-708040.rev.compl      tggtggaaacagctgaggaaataaccggggtctccctcacactctgctgaagcctgtagc   60281
FAM230B-LOC105372935-GGT2.NCBI.GGT2.rev.compl                      tggtggaaacagctgaggaaataaccggggtctccctcacactctgctgaagcctgtagc   59327
                                                                   ************************************************************

chimp.LOC112206744.LOC107973052.GGT2-.646279-708040.rev.compl      cacagaatcttcttcagagactccctgatcaggcagccttctcgttctcctgaaggtcaa   60341
FAM230B-LOC105372935-GGT2.NCBI.GGT2.rev.compl                      cacagaatcttcttcagagactctctgatcaggcagccttctcgttctcctgaaggtcaa   59387
                                                                   *********************** ************************************

chimp.LOC112206744.LOC107973052.GGT2-.646279-708040.rev.compl      aggaggttacctgaagcacgcacagctcagacctttctggggtactccgtgttacctccc   60401
FAM230B-LOC105372935-GGT2.NCBI.GGT2.rev.compl                      gggaggttacctgaagcacgcacagcccagacctttctgggggactccgtgttacctccc   59447
                                                                    ************************* *************** *****************

chimp.LOC112206744.LOC107973052.GGT2-.646279-708040.rev.compl      tctgcctctagctggtttctctgtctccagttgaactctggaggcaaagaggctgtcagt   60461
FAM230B-LOC105372935-GGT2.NCBI.GGT2.rev.compl                      tctgcctctagctggtttctctgtctccagttgaactctggaggcaaagaggctgtcagt   59507
                                                                   ************************************************************

chimp.LOC112206744.LOC107973052.GGT2-.646279-708040.rev.compl      aacacatttgtttccatgaattctctcagcatgtctcccaggcacagctcaaagagggtt   60521
FAM230B-LOC105372935-GGT2.NCBI.GGT2.rev.compl                      aacacatttgtttccatgaattctctcagcatgtctcccaggcac---------agggtt   59558
                                                                   *********************************************         ******

chimp.LOC112206744.LOC107973052.GGT2-.646279-708040.rev.compl      ttgcacggagcagggcaggtaggggacagggcattcctgcacaagcccaggatgtccatg   60581
FAM230B-LOC105372935-GGT2.NCBI.GGT2.rev.compl                      ttgcacggagcagggcaggtaggggacagggcattcctgcacaagcccaggatgtgcatg   59618
                                                                   ******************************************************* ****

chimp.LOC112206744.LOC107973052.GGT2-.646279-708040.rev.compl      cggtaagcatggcaaaggggctcagggggcaccgccagcctgccctgctctgacgctgg   60641
FAM230B-LOC105372935-GGT2.NCBI.GGT2.rev.compl                      cggtaagcatggcaaaggggctcaggaggcaccgccagcctgccctgctctgacgctgg   59678
                                                                   ************************** *********************************

chimp.LOC112206744.LOC107973052.GGT2-.646279-708040.rev.compl      acttgccactcacctgctgtggggcctcaggcaaatcactgaactgtccagcctggatga   60701
FAM230B-LOC105372935-GGT2.NCBI.GGT2.rev.compl                      acttgccactcacctgctgtggggcctcaggcaaatcactgaactgtccagcctggatga   59738
                                                                   ************************************************************

chimp.LOC112206744.LOC107973052.GGT2-.646279-708040.rev.compl      cggcagcacctcacttgccttgctgctgggagtgttgtgaatagagtaggttagactgtg   60761
FAM230B-LOC105372935-GGT2.NCBI.GGT2.rev.compl                      cggcagcacctcacttgccttgctgctgggagtgttgtgaatagagtaggttagactgtg   59798
                                                                   ************************************************************

chimp.LOC112206744.LOC107973052.GGT2-.646279-708040.rev.compl      ggcagggcttggtgaatggtagctgtgattatcatcatggctgcaccggggacacccca   60821
FAM230B-LOC105372935-GGT2.NCBI.GGT2.rev.compl                      ggcagggcttggtgaatggtagctgtgattatcatcatggctgcactggggacacccca   59858
                                                                   ********************************************** *************

chimp.LOC112206744.LOC107973052.GGT2-.646279-708040.rev.compl      ggaggcctgagtggcacaggtctcttgctcactgtatgtcccctgtggactcccttccag   60881
FAM230B-LOC105372935-GGT2.NCBI.GGT2.rev.compl                      ggaggcctgagtggcacaggtctcttgctcactgtatgtcccctgtggactcccttccag   59918
                                                                   ************************************************************

chimp.LOC112206744.LOC107973052.GGT2-.646279-708040.rev.compl      gctgtgcagtgagtggcagcagtgacccttgggaagcctcatggctatggcagcaggtga   60941
FAM230B-LOC105372935-GGT2.NCBI.GGT2.rev.compl                      gctgtgcagtgagtggcagcagtgacccttgggaagtctcatggctacggcagcaggtga   59978
                                                                   ************************************ ********** ************

chimp.LOC112206744.LOC107973052.GGT2-.646279-708040.rev.compl      caggtgtgacaacagggaagaaggatgtggtgacagaggttggggttcccctctcccaca   61001
FAM230B-LOC105372935-GGT2.NCBI.GGT2.rev.compl                      caggtgtgacaacagggaagagggatgtggtgacagaggttggggttcccctctcccaca   60038
                                                                   ********************* **************************************

chimp.LOC112206744.LOC107973052.GGT2-.646279-708040.rev.compl      gtcagtttcccacaaagggcggtgtctgccagcaagcccctccaatgagccccaagctgg   61061
FAM230B-LOC105372935-GGT2.NCBI.GGT2.rev.compl                      gtcagtttcccacaaagggcggtgtctgccagcaagcccctccaatgagccccaagcttg   60098
                                                                   ********************************************************** *

chimp.LOC112206744.LOC107973052.GGT2-.646279-708040.rev.compl      gtttccctccactccaccctgtcccagtgcagagcgtctgacctcagaggcagacacact   61121
FAM230B-LOC105372935-GGT2.NCBI.GGT2.rev.compl                      gtttccctccactccacgctgtcccagtgcagagcatctgacctcagaggcagacacact   60158
                                                                   ***************** ***************** ************************
```

```
chimp.LOC112206744.LOC107973052.GGT2-.646279-708040.rev.compl      gtcccagaggtggtctacgaatggagtccctgtgccctccccacacacagggaacatcca   61181
FAM230B-LOC105372935-GGT2.NCBI.GGT2.rev.compl                      gtcccagaggtggtctacgaatggagtccccgtgccctccccacacacagggaacatcca   60218
                                                                   ****************************** *****************************

chimp.LOC112206744.LOC107973052.GGT2-.646279-708040.rev.compl      aatgccatcgtggaagggtgg-cacctccccaggcttggtgggcctggggccgatagtgt   61240
FAM230B-LOC105372935-GGT2.NCBI.GGT2.rev.compl                      aatgccatcgtggaagggtggccacctccccaggcttggtgggcctggggccgatagtgt   60278
                                                                   ********************* **************************************

chimp.LOC112206744.LOC107973052.GGT2-.646279-708040.rev.compl      gatacatttgacccccctcccagccctggatgcagacaccaagagcagagagacctggcag   61300
FAM230B-LOC105372935-GGT2.NCBI.GGT2.rev.compl                      gatacatttgacccccctcccagccctggatgcagacaccaagagcagagagacctggcag   60338
                                                                   ************************************************************

chimp.LOC112206744.LOC107973052.GGT2-.646279-708040.rev.compl      tagtcatgcagcagcgcaccaccccacatccccagcacaatccaaagcagcccgtcatcc   61360
FAM230B-LOC105372935-GGT2.NCBI.GGT2.rev.compl                      tagccatgcagcagcgcaccaccccacatccccagcacaatccaaagcagcccctcatcc   60398
                                                                   *** ************************************************* ******

chimp.LOC112206744.LOC107973052.GGT2-.646279-708040.rev.compl      ccaccgtgaccaccacagcctgaatccaggtgccacctgtttctgacctgaactccctca   61420
FAM230B-LOC105372935-GGT2.NCBI.GGT2.rev.compl                      ccaccgtgaccaccacagcctgaatccaggcgccacctgtttctgacctgaactccctca   60458
                                                                   ****************************** *****************************

chimp.LOC112206744.LOC107973052.GGT2-.646279-708040.rev.compl      cagcccctgcctgcactccctccctccaacatcgcctgcccttcagtcttccagaaagca   61480
FAM230B-LOC105372935-GGT2.NCBI.GGT2.rev.compl                      cagcccctgcctgcactccctccctccaacatcacctgcccttcagtcttccagaaagca   60518
                                                                   ********************************* **************************

chimp.LOC112206744.LOC107973052.GGT2-.646279-708040.rev.compl      gctagagggctctgtctgtccaactgcagaacaggccctgcctcctccctgtcccgttgg   61540
FAM230B-LOC105372935-GGT2.NCBI.GGT2.rev.compl                      gctagagggctctgtctgtccaactgcagaacaggccctgcctcctccctgccctgttgg   60578
                                                                   *************************************************** ** *****

chimp.LOC112206744.LOC107973052.GGT2-.646279-708040.rev.compl      acagctcacacccttcaccaggcctgacagcactcttgccactccaacaccctgggtccc   61600
FAM230B-LOC105372935-GGT2.NCBI.GGT2.rev.compl                      acagctcacacccttcaccaggcctgacagcgctcttgccactccaacaccccgggtccc   60638
                                                                   ******************************* ******************** *******

chimp.LOC112206744.LOC107973052.GGT2-.646279-708040.rev.compl      agccgggagtccaggtcagggttaagggttcctgatagagacaccgattcctggaggtcc   61660
FAM230B-LOC105372935-GGT2.NCBI.GGT2.rev.compl                      agccgggagtccgggtcagggttaagggttcctggtagagacaccgattcctggaggtcc   60698
                                                                   ************ ********************* *************************

chimp.LOC112206744.LOC107973052.GGT2-.646279-708040.rev.compl      aaagagcctcaggagctgggccagcaatatgcagcatctattatggacacagaacattcc   61720
FAM230B-LOC105372935-GGT2.NCBI.GGT2.rev.compl                      aaagagcctcaggagctgggccagcaatatgcagcatctattatggacacagaacattcc   60758
                                                                   ************************************************************

chimp.LOC112206744.LOC107973052.GGT2-.646279-708040.rev.compl      catcacatggccgggtgcagtggctcacgcctataatcccag------------------   61762
FAM230B-LOC105372935-GGT2.NCBI.GGT2.rev.compl                      catcacatggccgggtgcagtggctcacgcctttaatcccagcactttgggaggcagagg   60818
                                                                   ******************************** *********

chimp.LOC112206744.LOC107973052.GGT2-.646279-708040.rev.compl      ------------------------------------------------------------   61762
FAM230B-LOC105372935-GGT2.NCBI.GGT2.rev.compl                      caggtggatcacctgaggtgaagagttcgagaccagtctggccaacatggtgaaaccccc   60878

chimp.LOC112206744.LOC107973052.GGT2-.646279-708040.rev.compl      ------------------------------------------------------------   61762
FAM230B-LOC105372935-GGT2.NCBI.GGT2.rev.compl                      atctccactaaaaatacaaaaaattagccaggcatggtggcaggtgcctgtaatcccagc   60938

chimp.LOC112206744.LOC107973052.GGT2-.646279-708040.rev.compl      ------------------------------------------------------------   61762
FAM230B-LOC105372935-GGT2.NCBI.GGT2.rev.compl                      tactcaggagctgaggcaggagaattgcttgaacccgggaggtggaggttgcagtgaggc   60998

chimp.LOC112206744.LOC107973052.GGT2-.646279-708040.rev.compl      ------------------------------------------------------------   61762
FAM230B-LOC105372935-GGT2.NCBI.GGT2.rev.compl                      aagattgcaccactgcactccagcctgggccacaagagtgaaactccgtcacacacacac   61058

chimp.LOC112206744.LOC107973052.GGT2-.646279-708040.rev.compl      ------------------------------------------------------------   61762
FAM230B-LOC105372935-GGT2.NCBI.GGT2.rev.compl                      acacacacacacacaaaaaaaaaaaaaaagaaaaaaaaggtctcctgctggaacacagac   61118

chimp.LOC112206744.LOC107973052.GGT2-.646279-708040.rev.compl      ------------------------------------------------------------   61762
FAM230B-LOC105372935-GGT2.NCBI.GGT2.rev.compl                      tagttagagaaaggaaaaataaacaaatgatagtatgtgtattaataaaagaactagcaa   61178

chimp.LOC112206744.LOC107973052.GGT2-.646279-708040.rev.compl      ------------------------------------------------------------   61762
FAM230B-LOC105372935-GGT2.NCBI.GGT2.rev.compl                      cacccactgcttagttgtgataataaaaactggacattaatataggcagaaaaacagaac   61238

chimp.LOC112206744.LOC107973052.GGT2-.646279-708040.rev.compl      ------------------------------------------------------------   61762
FAM230B-LOC105372935-GGT2.NCBI.GGT2.rev.compl                      agccttgatatggttaaccctttgacactggcaaacactgtgaccagtgctgcccacact   61298

chimp.LOC112206744.LOC107973052.GGT2-.646279-708040.rev.compl      ------------------------------------------------------------   61762
FAM230B-LOC105372935-GGT2.NCBI.GGT2.rev.compl                      ggaagctcctgcatccctcttcctcatgtttccttcagcattaaggagcaacaagggaga   61358

chimp.LOC112206744.LOC107973052.GGT2-.646279-708040.rev.compl      ------------------------------------------------------------   61762
FAM230B-LOC105372935-GGT2.NCBI.GGT2.rev.compl                      cagccagttcatagttccttacgcatggagccaaaggaccttcaatgtacaaggtctgag   61418

chimp.LOC112206744.LOC107973052.GGT2-.646279-708040.rev.compl      ------------------------------------------------------------   61762
FAM230B-LOC105372935-GGT2.NCBI.GGT2.rev.compl                      caaggacccgcagccacatgtgcttcctgcttcagcagtgccccgtgggtctcagagcta   61478

chimp.LOC112206744.LOC107973052.GGT2-.646279-708040.rev.compl      ------------------------------------------------------------   61762
FAM230B-LOC105372935-GGT2.NCBI.GGT2.rev.compl                      cccaaaggcctatccttccgagaggtttctcctctctcccaactgatgtcatacgtctca   61538

chimp.LOC112206744.LOC107973052.GGT2-.646279-708040.rev.compl      ------------------------------------------------------------   61762
FAM230B-LOC105372935-GGT2.NCBI.GGT2.rev.compl                      tcttccttgtcattcagatcatcaaccacaaacgtccttgccttatattttcatatccct   61598

chimp.LOC112206744.LOC107973052.GGT2-.646279-708040.rev.compl      ------------------------------------------------------------   61762
FAM230B-LOC105372935-GGT2.NCBI.GGT2.rev.compl                      tttcaccagttacagggttagttaacttacgaaattcttaacatctgcattagatctttt   61658

chimp.LOC112206744.LOC107973052.GGT2-.646279-708040.rev.compl      ------------------------------------------------------------   61762
FAM230B-LOC105372935-GGT2.NCBI.GGT2.rev.compl                      taaagtttcaccctcaaccacctattatttagaagtgaacacagaaatttagtttccttg   61718

chimp.LOC112206744.LOC107973052.GGT2-.646279-708040.rev.compl      ------------------------------------------------------------   61762
FAM230B-LOC105372935-GGT2.NCBI.GGT2.rev.compl                      tgctatctgttgaccccctaaaatatgctgggagttttgggatttttttaaagtcaaatg   61778

chimp.LOC112206744.LOC107973052.GGT2-.646279-708040.rev.compl      ------------------------------------------------------------   61762
FAM230B-LOC105372935-GGT2.NCBI.GGT2.rev.compl                      catggcataaagcaaaattacactactaaagaactgagtcaggccagacgctggcaacgt   61838

chimp.LOC112206744.LOC107973052.GGT2-.646279-708040.rev.compl      ------------------------------------------------------------   61762
FAM230B-LOC105372935-GGT2.NCBI.GGT2.rev.compl                      gaagacagcttctccttacccactaggttcatctttgcactgttaactcccaaagtattg   61898

chimp.LOC112206744.LOC107973052.GGT2-.646279-708040.rev.compl      ------------------------------------------------------------   61762
FAM230B-LOC105372935-GGT2.NCBI.GGT2.rev.compl                      cttctcggtgctgggtgtgtggcattcatattccccggcatcccgggcctgaagatctgt   61958

chimp.LOC112206744.LOC107973052.GGT2-.646279-708040.rev.compl      ------------------------------------------------------------   61762
FAM230B-LOC105372935-GGT2.NCBI.GGT2.rev.compl                      gatgtgcaatagggttgggttcccctggactctttctatgaagatcttccctccgcggac   62018

chimp.LOC112206744.LOC107973052.GGT2-.646279-708040.rev.compl      ------------------------------------------------------------   61762
FAM230B-LOC105372935-GGT2.NCBI.GGT2.rev.compl                      gcgctgggtgtagatggcataggggaaggaagagtccatggtgctgacgatctgcacctc   62078

chimp.LOC112206744.LOC107973052.GGT2-.646279-708040.rev.compl      ------------------------------------------------------------   61762
FAM230B-LOC105372935-GGT2.NCBI.GGT2.rev.compl                      tcgctctggcgacgaaggcaggtaaatggaccactggaaattctgttcagaaggtccctg   62138

chimp.LOC112206744.LOC107973052.GGT2-.646279-708040.rev.compl      ------------------------------------------------------------   61762
FAM230B-LOC105372935-GGT2.NCBI.GGT2.rev.compl                      gtagccactcacattgcaccagatagtgatgtgggagccctccgtgcggtacaagggtcc   62198

chimp.LOC112206744.LOC107973052.GGT2-.646279-708040.rev.compl      ------------------------------------------------------------   61762
FAM230B-LOC105372935-GGT2.NCBI.GGT2.rev.compl                      ttcctgaacggtgacctgccgctgtgctgacaccacacctacgagggagagaaacacacg   62258

chimp.LOC112206744.LOC107973052.GGT2-.646279-708040.rev.compl      ------------------------------------------------------------   61762
FAM230B-LOC105372935-GGT2.NCBI.GGT2.rev.compl                      caacatgctcacttacttctcagaccaaaatgcaaagtaggcagcaatctccaaagggtt   62318
```

```
chimp.LOC112206744.LOC107973052.GGT2-.646279-708040.rev.compl      ------------------------------------------------------------   61762
FAM230B-LOC105372935-GGT2.NCBI.GGT2.rev.compl                      tgttatttgtgtgacatgataataatataaacagcttactggccctttcaaaggcactct   62378

chimp.LOC112206744.LOC107973052.GGT2-.646279-708040.rev.compl      ------------------------------------------------------------   61762
FAM230B-LOC105372935-GGT2.NCBI.GGT2.rev.compl                      gtgatttataaatattaattaaaacactctgtggtaaagcactgtccctaatttgcatat   62438

chimp.LOC112206744.LOC107973052.GGT2-.646279-708040.rev.compl      ------------------------------------------------------------   61762
FAM230B-LOC105372935-GGT2.NCBI.GGT2.rev.compl                      atgggaatttgatcatgccaagatgtgcttttgttacttattcaacagctgacccttggg   62498

chimp.LOC112206744.LOC107973052.GGT2-.646279-708040.rev.compl      ------------------------------------------------------------   61762
FAM230B-LOC105372935-GGT2.NCBI.GGT2.rev.compl                      aatttcatttagcccctctgtttgtcatgtatacggcagttttatatctatttattaatg   62558

chimp.LOC112206744.LOC107973052.GGT2-.646279-708040.rev.compl      ------------------------------------------------------------   61762
FAM230B-LOC105372935-GGT2.NCBI.GGT2.rev.compl                      ctgtgtataaagtttttaataaaataagcaggaaaagttgaatccttggcacaaattcag   62618

chimp.LOC112206744.LOC107973052.GGT2-.646279-708040.rev.compl      ------------------------------------------------------------   61762
FAM230B-LOC105372935-GGT2.NCBI.GGT2.rev.compl                      atgaaaacaaataaaagcaaccatctaggaatctggctgaaattaaatgcttcttccttg   62678

chimp.LOC112206744.LOC107973052.GGT2-.646279-708040.rev.compl      ------------------------------------------------------------   61762
FAM230B-LOC105372935-GGT2.NCBI.GGT2.rev.compl                      gtccgagtgctggtgggagggagtcctgggagcacctttttactggtgctgcacccttca   62738

chimp.LOC112206744.LOC107973052.GGT2-.646279-708040.rev.compl      ------------------------------------------------------------   61762
FAM230B-LOC105372935-GGT2.NCBI.GGT2.rev.compl                      ttttcccatgtagctgctccccaacaccaccccaaggagcattccttagtttctcttccc   62798

chimp.LOC112206744.LOC107973052.GGT2-.646279-708040.rev.compl      ------------------------------------------------------------   61762
FAM230B-LOC105372935-GGT2.NCBI.GGT2.rev.compl                      tccataaatggaggcagagctgagctcctgcaggctgctatcgttttaagctgagcacac   62858

chimp.LOC112206744.LOC107973052.GGT2-.646279-708040.rev.compl      ------------------------------------------------------------   61762
FAM230B-LOC105372935-GGT2.NCBI.GGT2.rev.compl                      ccaaatacaaatgtgaagggctgaaacagatccctgatgcccagattcccacaaactgac   62918

chimp.LOC112206744.LOC107973052.GGT2-.646279-708040.rev.compl      ------------------------------------------------------------   61762
FAM230B-LOC105372935-GGT2.NCBI.GGT2.rev.compl                      aaagggggaactaacagtgcccagctaaacctgtgacaaggacagtcaggctacccttg   62978

chimp.LOC112206744.LOC107973052.GGT2-.646279-708040.rev.compl      ------------------------------------------------------------   61762
FAM230B-LOC105372935-GGT2.NCBI.GGT2.rev.compl                      agtttcccactcaagactcccccgctgacctcctgccttctggctcagatactgttctcc   63038

chimp.LOC112206744.LOC107973052.GGT2-.646279-708040.rev.compl      ------------------------------------------------------------   61762
FAM230B-LOC105372935-GGT2.NCBI.GGT2.rev.compl                      aaggtcacttctgcctgccttctccatgttcttcttaaagtggcatggtgagtttaattt   63098

chimp.LOC112206744.LOC107973052.GGT2-.646279-708040.rev.compl      ------------------------------------------------------------   61762
FAM230B-LOC105372935-GGT2.NCBI.GGT2.rev.compl                      ctcatttcgtgccaactcaacattgattgtgcctgagccactcagaaggtgggaggggc   63158

chimp.LOC112206744.LOC107973052.GGT2-.646279-708040.rev.compl      ------------------------------------------------------------   61762
FAM230B-LOC105372935-GGT2.NCBI.GGT2.rev.compl                      acacccctgcctcttcatttcggcagtggtcacgtactgcaggttgtgaaaagcgcaggt   63218

chimp.LOC112206744.LOC107973052.GGT2-.646279-708040.rev.compl      ------------------------------------------------------------   61762
FAM230B-LOC105372935-GGT2.NCBI.GGT2.rev.compl                      gagttataggcgcagctttgccaccaattagagctggttgtgtcatctcagacaacttgc   63278

chimp.LOC112206744.LOC107973052.GGT2-.646279-708040.rev.compl      ------------------------------------------------------------   61762
FAM230B-LOC105372935-GGT2.NCBI.GGT2.rev.compl                      ttttcttctccagaccttgatcttctcatctatcacatgatttctaagaacgctttagag   63338

chimp.LOC112206744.LOC107973052.GGT2-.646279-708040.rev.compl      ------------------------------------------------------------   61762
FAM230B-LOC105372935-GGT2.NCBI.GGT2.rev.compl                      agcactagcatgggttccgatgctggctttgtcacttattaactgtgatcttggataagc   63398

chimp.LOC112206744.LOC107973052.GGT2-.646279-708040.rev.compl      ------------------------------------------------------------   61762
FAM230B-LOC105372935-GGT2.NCBI.GGT2.rev.compl                      tgcttaacctctctaagcctcagttttctcatcggtaagatggggataataatgcctgtc   63458

chimp.LOC112206744.LOC107973052.GGT2-.646279-708040.rev.compl      ------------------------------------------------------------   61762
FAM230B-LOC105372935-GGT2.NCBI.GGT2.rev.compl                      tcatagggttattgagaagatttagtaacatctgtaaagaccctagcacagagcctagcc   63518

chimp.LOC112206744.LOC107973052.GGT2-.646279-708040.rev.compl      ------------------------------------------------------------   61762
FAM230B-LOC105372935-GGT2.NCBI.GGT2.rev.compl                      cacaatcagtcctcaataaagagctgctggctaagacatatttctgctggaacattctgt   63578

chimp.LOC112206744.LOC107973052.GGT2-.646279-708040.rev.compl      ------------------------------------------------------------   61762
FAM230B-LOC105372935-GGT2.NCBI.GGT2.rev.compl                      ggctctggatccatagaggacaggatttggccaatgactgcttaaggacatttcaacggc   63638

chimp.LOC112206744.LOC107973052.GGT2-.646279-708040.rev.compl      ------------------------------------------------------------   61762
FAM230B-LOC105372935-GGT2.NCBI.GGT2.rev.compl                      cccttgacaatgacaccatcactgggctgcccacctcatcccacagctgcagcccattct   63698

chimp.LOC112206744.LOC107973052.GGT2-.646279-708040.rev.compl      ------------------------------------------------------------   61762
FAM230B-LOC105372935-GGT2.NCBI.GGT2.rev.compl                      tgtgatagcttccctttttcccccacaaatgggaatggcggctgccctggctatgcctatc   63758

chimp.LOC112206744.LOC107973052.GGT2-.646279-708040.rev.compl      ------------------------------------------------------------   61762
FAM230B-LOC105372935-GGT2.NCBI.GGT2.rev.compl                      tgaagtggatccagctggtgaacagccgggacagctcaaatactagtggagtgcagctct   63818

chimp.LOC112206744.LOC107973052.GGT2-.646279-708040.rev.compl      ------------------------------------------------------------   61762
FAM230B-LOC105372935-GGT2.NCBI.GGT2.rev.compl                      ctgcccagcccagcaggtgactgtgcctcagtgtaagctaatctaatctgcaagatgaca   63878

chimp.LOC112206744.LOC107973052.GGT2-.646279-708040.rev.compl      ------------------------------------------------------------   61762
FAM230B-LOC105372935-GGT2.NCBI.GGT2.rev.compl                      gacctcaaaacggcccatacaatgaaatgcccatggcttgcaaaactttaattcatgcag   63938

chimp.LOC112206744.LOC107973052.GGT2-.646279-708040.rev.compl      ------------------------------------------------------------   61762
FAM230B-LOC105372935-GGT2.NCBI.GGT2.rev.compl                      aacctttcctcacaaacaaaatcttacacagcaccccatcttggcttcagcagaaccagg   63998

chimp.LOC112206744.LOC107973052.GGT2-.646279-708040.rev.compl      ------------------------------------------------------------   61762
FAM230B-LOC105372935-GGT2.NCBI.GGT2.rev.compl                      aaaaataaaaaattggcaggggaaggaaataaacagaacaagtggtatctgaatttattt   64058

chimp.LOC112206744.LOC107973052.GGT2-.646279-708040.rev.compl      ------------------------------------------------------------   61762
FAM230B-LOC105372935-GGT2.NCBI.GGT2.rev.compl                      gatatattctggttgatggcattaattatgacatttaaagtcaccatgaggacaactcat   64118

chimp.LOC112206744.LOC107973052.GGT2-.646279-708040.rev.compl      ------------------------------------------------------------   61762
FAM230B-LOC105372935-GGT2.NCBI.GGT2.rev.compl                      tagtaatatcagtatgttaaaatatggtgcataacatttttaatatatgtacagtcatgc   64178

chimp.LOC112206744.LOC107973052.GGT2-.646279-708040.rev.compl      ------------------------------------------------------------   61762
FAM230B-LOC105372935-GGT2.NCBI.GGT2.rev.compl                      actgaataacatttcagtcaatgaggaaacacatgtgcaacagtgatcctgtaagattat   64238

chimp.LOC112206744.LOC107973052.GGT2-.646279-708040.rev.compl      ------------------------------------------------------------   61762
FAM230B-LOC105372935-GGT2.NCBI.GGT2.rev.compl                      aatggagcatatatagagatctaatatatggcacttaatgttggcatggcagatcaagta   64298

chimp.LOC112206744.LOC107973052.GGT2-.646279-708040.rev.compl      ------------------------------------------------------------   61762
FAM230B-LOC105372935-GGT2.NCBI.GGT2.rev.compl                      ggggaaatgactgatatttagtaacagtgctgggacatttgattttccataataaaatat   64358

chimp.LOC112206744.LOC107973052.GGT2-.646279-708040.rev.compl      ------------------------------------------------------------   61762
FAM230B-LOC105372935-GGT2.NCBI.GGT2.rev.compl                      ataaatgaaaatatatatcccatctaggtttgttgaaatacaccctatgatgttcacaca   64418

chimp.LOC112206744.LOC107973052.GGT2-.646279-708040.rev.compl      ------------------------------------------------------------   61762
FAM230B-LOC105372935-GGT2.NCBI.GGT2.rev.compl                      agaatgaaattgcctaatgatgcatttcttaaaacatgtccccatcattaagtgacccat   64478

chimp.LOC112206744.LOC107973052.GGT2-.646279-708040.rev.compl      ------------------------------------------------------------   61762
```

```
FAM230B-LOC105372935-GGT2.NCBI.GGT2.rev.compl              gactgtatacacacacacatatggtgacacttaaatcaaatgattgcagtattcctgaaa   64538

chimp.LOC112206744.LOC107973052.GGT2-.646279-708040.rev.compl   ------------------------------------------------------------   61762
FAM230B-LOC105372935-GGT2.NCBI.GGT2.rev.compl              cacagaacattttgcaaacaatttacctacattcataacattaggcatccttagaattgc   64598

chimp.LOC112206744.LOC107973052.GGT2-.646279-708040.rev.compl   ------------------------------------------------------------   61762
FAM230B-LOC105372935-GGT2.NCBI.GGT2.rev.compl              agtgttctatgacagagcaactacaaccaacccccatccatctcctgcaaagaaggctgg   64658

chimp.LOC112206744.LOC107973052.GGT2-.646279-708040.rev.compl   ------------------------------------------------------------   61762
FAM230B-LOC105372935-GGT2.NCBI.GGT2.rev.compl              aggcaggtcagggtacaccagcatcttcaagaactgcttctcaatcctggacctacactg   64718

chimp.LOC112206744.LOC107973052.GGT2-.646279-708040.rev.compl   ------------------------------------------------------------   61762
FAM230B-LOC105372935-GGT2.NCBI.GGT2.rev.compl              gaatcacctggggagctttaaaaaaataacagtgcctggaccccacctgacataactgg   64778

chimp.LOC112206744.LOC107973052.GGT2-.646279-708040.rev.compl   ------------------------------------------------------------   61762
FAM230B-LOC105372935-GGT2.NCBI.GGT2.rev.compl              tcttaggattaattagggttttttttgaagctccacagatgattcaaatcaggtgtagca   64838

chimp.LOC112206744.LOC107973052.GGT2-.646279-708040.rev.compl   ------------------------------------------------------------   61762
FAM230B-LOC105372935-GGT2.NCBI.GGT2.rev.compl              aagcactgttactttaaagtgtctctacctacatggtgccagccaagtgctcaaatgaaa   64898

chimp.LOC112206744.LOC107973052.GGT2-.646279-708040.rev.compl   ------------------------------------------------------------   61762
FAM230B-LOC105372935-GGT2.NCBI.GGT2.rev.compl              tatcttaaggctctcactggctttaagtttctctctttggtagagacccaatcactggtt   64958

chimp.LOC112206744.LOC107973052.GGT2-.646279-708040.rev.compl   ------------------------------------------------------------   61762
FAM230B-LOC105372935-GGT2.NCBI.GGT2.rev.compl              caaggaagtttcattctcctccagtcttccccagtgcaaaagaaaacagctgagacccat   65018

chimp.LOC112206744.LOC107973052.GGT2-.646279-708040.rev.compl   ------------------------------------------------------------   61762
FAM230B-LOC105372935-GGT2.NCBI.GGT2.rev.compl              cagatactggcttttgtgatgcaataatgagtttaaccagaatgcatccgatttacagac   65078

chimp.LOC112206744.LOC107973052.GGT2-.646279-708040.rev.compl   ------------------------------------------------------------   61762
FAM230B-LOC105372935-GGT2.NCBI.GGT2.rev.compl              cttacaaaggcactaggccagcggatcatgcacgtcctccccacccaaaggtaaacagtg   65138

chimp.LOC112206744.LOC107973052.GGT2-.646279-708040.rev.compl   ------------------------------------------------------------   61762
FAM230B-LOC105372935-GGT2.NCBI.GGT2.rev.compl              ttaagtggcctgaatgagccaggacagcagggtcaaatcaacttgtctaggctggaagca   65198

chimp.LOC112206744.LOC107973052.GGT2-.646279-708040.rev.compl   ------------------------------------------------------------   61762
FAM230B-LOC105372935-GGT2.NCBI.GGT2.rev.compl              ggttataaacaatccggacaaataaataactactggattgcactttaaccacacacattc   65258

chimp.LOC112206744.LOC107973052.GGT2-.646279-708040.rev.compl   ------------------------------------------------------------   61762
FAM230B-LOC105372935-GGT2.NCBI.GGT2.rev.compl              agctgcacctgtttattaaatacctccttaatttccctctgccccacaaagggcttctga   65318

chimp.LOC112206744.LOC107973052.GGT2-.646279-708040.rev.compl   ------------------------------------------------------------   61762
FAM230B-LOC105372935-GGT2.NCBI.GGT2.rev.compl              cccctgaaaataatgcttctcaacataaaaagaattgttttcttcttggcagtttaattc   65378

chimp.LOC112206744.LOC107973052.GGT2-.646279-708040.rev.compl   ------------------------------------------------------------   61762
FAM230B-LOC105372935-GGT2.NCBI.GGT2.rev.compl              cccttccacacatcccaccccaccatttccctataatggtaataccccgctgtgcagacc   65438

chimp.LOC112206744.LOC107973052.GGT2-.646279-708040.rev.compl   ------------------------------------------------------------   61762
FAM230B-LOC105372935-GGT2.NCBI.GGT2.rev.compl              tctgctgcctccagagagacacaggcgcccaaccctgaccagggcatcctctgacccaca   65498

chimp.LOC112206744.LOC107973052.GGT2-.646279-708040.rev.compl   ------------------------------------------------------------   61762
FAM230B-LOC105372935-GGT2.NCBI.GGT2.rev.compl              gcccctctatctcccctctcagcctacaatagaaagttcctcccaggcaaggatcatttt   65558

chimp.LOC112206744.LOC107973052.GGT2-.646279-708040.rev.compl   ------------------------------------------------------------   61762
FAM230B-LOC105372935-GGT2.NCBI.GGT2.rev.compl              ttttaaataactttttaactttagacattctgtactctttggattactctgcaataagc   65618

chimp.LOC112206744.LOC107973052.GGT2-.646279-708040.rev.compl   ------------------------------------------------------------   61762
FAM230B-LOC105372935-GGT2.NCBI.GGT2.rev.compl              aaatatgacttctgtaacataaaaagaaagatcaaaatgttatatataactgcatgaaaa   65678

chimp.LOC112206744.LOC107973052.GGT2-.646279-708040.rev.compl   ------------------------------------------------------------   61762
FAM230B-LOC105372935-GGT2.NCBI.GGT2.rev.compl              gaactagaaagaaaatactaggtgacaggatggcaagtgatttttatttttttcttaata   65738

chimp.LOC112206744.LOC107973052.GGT2-.646279-708040.rev.compl   ------------------------------------------------------------   61762
FAM230B-LOC105372935-GGT2.NCBI.GGT2.rev.compl              ttttactccttcccccagtcctctgcaatgagtatgtaccagttttataactagaaaaaa   65798

chimp.LOC112206744.LOC107973052.GGT2-.646279-708040.rev.compl   ------------------------------------------------------------   61762
FAM230B-LOC105372935-GGT2.NCBI.GGT2.rev.compl              attttagccaaaaagaaaaataatgcatgtttgctatataaaattcactactgtagtata   65858

chimp.LOC112206744.LOC107973052.GGT2-.646279-708040.rev.compl   ------------------------------------------------------------   61762
FAM230B-LOC105372935-GGT2.NCBI.GGT2.rev.compl              tatatatacatacatacatatttggtctttgtccccagttcctggcactgagctcctaaa   65918

chimp.LOC112206744.LOC107973052.GGT2-.646279-708040.rev.compl   ------------------------------------------------------------   61762
FAM230B-LOC105372935-GGT2.NCBI.GGT2.rev.compl              ccccttggaacttcctaagcaatgggagtaccttttgttatttataagaagccccttttg   65978

chimp.LOC112206744.LOC107973052.GGT2-.646279-708040.rev.compl   ------------------------------------------------------------   61762
FAM230B-LOC105372935-GGT2.NCBI.GGT2.rev.compl              gccatcccagagtttatgctaacgaggtgactgaaggtgagcacggaggcagtttcaagg   66038

chimp.LOC112206744.LOC107973052.GGT2-.646279-708040.rev.compl   ------------------------------------------------------------   61762
FAM230B-LOC105372935-GGT2.NCBI.GGT2.rev.compl              aaggagctggccacgcttagaattgtggagttttcagccccgcccttagacctccaagga   66098

chimp.LOC112206744.LOC107973052.GGT2-.646279-708040.rev.compl   ------------------------------------------------------------   61762
FAM230B-LOC105372935-GGT2.NCBI.GGT2.rev.compl              caagaggggaccgcagattgatccaatcagcattggtcagtgatttgatcagtcatacc   66158

chimp.LOC112206744.LOC107973052.GGT2-.646279-708040.rev.compl   ------------------------------------------------------------   61762
FAM230B-LOC105372935-GGT2.NCBI.GGT2.rev.compl              cacgtaatgaaatcccatatgaaaaccctaaataatggagttaggagagcttctgggtgc   66218

chimp.LOC112206744.LOC107973052.GGT2-.646279-708040.rev.compl   ------------------------------------------------------------   61762
FAM230B-LOC105372935-GGT2.NCBI.GGT2.rev.compl              tggaagcggcgtgcaccaggagagggcatgggcagtgagcactactcccctctcagacct   66278

chimp.LOC112206744.LOC107973052.GGT2-.646279-708040.rev.compl   ------------------------------------------------------------   61762
FAM230B-LOC105372935-GGT2.NCBI.GGT2.rev.compl              tgccctatgcactaaataggactgacctatgtgaccaataggaaatgcacaaatggtgga   66338

chimp.LOC112206744.LOC107973052.GGT2-.646279-708040.rev.compl   ------------------------------------------------------------   61762
FAM230B-LOC105372935-GGT2.NCBI.GGT2.rev.compl              atgtgacttccagggctaggtcataaaaagacaagcacgatgttatgaggacatcaaagc   66398

chimp.LOC112206744.LOC107973052.GGT2-.646279-708040.rev.compl   ------------------------------------------------------------   61762
FAM230B-LOC105372935-GGT2.NCBI.GGT2.rev.compl              agccatatggggaggaccccatgaggcctcctgccagcagctagcactaacttggctagc   66458

chimp.LOC112206744.LOC107973052.GGT2-.646279-708040.rev.compl   ------------------------------------------------------------   61762
FAM230B-LOC105372935-GGT2.NCBI.GGT2.rev.compl              atgtgactggaagtagattctccagcctcagtcaactcagtcaagccttcagataatgtc   66518

chimp.LOC112206744.LOC107973052.GGT2-.646279-708040.rev.compl   ------------------------------------------------------------   61762
FAM230B-LOC105372935-GGT2.NCBI.GGT2.rev.compl              aaccccaggcatctttttgttgttgttgagacagggtctcactctgtcacccaggctgga   66578

chimp.LOC112206744.LOC107973052.GGT2-.646279-708040.rev.compl   ------------------------------------------------------------   61762
FAM230B-LOC105372935-GGT2.NCBI.GGT2.rev.compl              gtgcagtggtgcaatcatagctcactgcagccttgacctctgaggctcaagtgaccctcc   66638

chimp.LOC112206744.LOC107973052.GGT2-.646279-708040.rev.compl   ------------------------------------------------------------   61762
FAM230B-LOC105372935-GGT2.NCBI.GGT2.rev.compl              cacatcagcctcctgagtatctgggaccaccagcacacaccaccatgcctggctaacttt   66698
```

```
chimp.LOC112206744.LOC107973052.GGT2-.646279-708040.rev.compl      ------------------------------------------------------------   61762
FAM230B-LOC105372935-GGT2.NCBI.GGT2.rev.compl                      tttatttcttatagagatgggatgatatggttggggtctgtgtacccacctaaatctcac   66758

chimp.LOC112206744.LOC107973052.GGT2-.646279-708040.rev.compl      ------------------------------------------------------------   61762
FAM230B-LOC105372935-GGT2.NCBI.GGT2.rev.compl                      gttcaattataatccccaatgttagaggtggggcctggtgggaggtgactggatcacagg   66818

chimp.LOC112206744.LOC107973052.GGT2-.646279-708040.rev.compl      ------------------------------------------------------------   61762
FAM230B-LOC105372935-GGT2.NCBI.GGT2.rev.compl                      aatggatctttcatgaatgatttagcatcacccttttggtgctgtgcttgttagagttct   66878

chimp.LOC112206744.LOC107973052.GGT2-.646279-708040.rev.compl      ------------------------------------------------------------   61762
FAM230B-LOC105372935-GGT2.NCBI.GGT2.rev.compl                      cccagtatctaattgtttaaaagtgtgtggcacctccccctctctctcttgctcctgct   66938

chimp.LOC112206744.LOC107973052.GGT2-.646279-708040.rev.compl      ------------------------------------------------------------   61762
FAM230B-LOC105372935-GGT2.NCBI.GGT2.rev.compl                      ttggccatgtaaggcgtgcctgcttccccttcaccttccaccatgattgaaagtttcctg   66998

chimp.LOC112206744.LOC107973052.GGT2-.646279-708040.rev.compl      ------------------------------------------------------------   61762
FAM230B-LOC105372935-GGT2.NCBI.GGT2.rev.compl                      tgcttgccccagaagctgagcagatgccagcattatgctccctgaacagcctgtggaact   67058

chimp.LOC112206744.LOC107973052.GGT2-.646279-708040.rev.compl      ------------------------------------------------------------   61762
FAM230B-LOC105372935-GGT2.NCBI.GGT2.rev.compl                      gcgagacaattaaacctcttttctttataaattctccagtctcatgtatttatagcaatg   67118

chimp.LOC112206744.LOC107973052.GGT2-.646279-708040.rev.compl      ------------------------------------------------------------   61762
FAM230B-LOC105372935-GGT2.NCBI.GGT2.rev.compl                      tgagaactgactaatacatagggtctcactgtgtttcccaggctggtctcaaactcctgg   67178

chimp.LOC112206744.LOC107973052.GGT2-.646279-708040.rev.compl      ------------------------------------------------------------   61762
FAM230B-LOC105372935-GGT2.NCBI.GGT2.rev.compl                      gctcaagtaatcctcctggctttgcttcccagagtgtagagattacaggcatgagccact   67238

chimp.LOC112206744.LOC107973052.GGT2-.646279-708040.rev.compl      ------------------------------------------------------------   61762
FAM230B-LOC105372935-GGT2.NCBI.GGT2.rev.compl                      gaacctgcccctttcccccaacaacatcttgattgcaacctcctaagagactccgagtc   67298

chimp.LOC112206744.LOC107973052.GGT2-.646279-708040.rev.compl      ------------------------------------------------------------   61762
FAM230B-LOC105372935-GGT2.NCBI.GGT2.rev.compl                      agaagtatccagttaagatgttactgactttctgaactacagaaactgtgagataataaa   67358

chimp.LOC112206744.LOC107973052.GGT2-.646279-708040.rev.compl      ------------------------------------------------------------   61762
FAM230B-LOC105372935-GGT2.NCBI.GGT2.rev.compl                      catctattgttcctttaagccttaagtttggggcttttttttttacaccaatatataac   67418

chimp.LOC112206744.LOC107973052.GGT2-.646279-708040.rev.compl      ------------------------------------------------------------   61762
FAM230B-LOC105372935-GGT2.NCBI.GGT2.rev.compl                      taatgtagacacctgcagtccctaagattgaatgcaaaattgtgttacatacacatgagt   67478

chimp.LOC112206744.LOC107973052.GGT2-.646279-708040.rev.compl      ------------------------------------------------------------   61762
FAM230B-LOC105372935-GGT2.NCBI.GGT2.rev.compl                      gtgttcttctgggaaaagagtccataaacttcaaaagattctcaaaggaatccaagaccc   67538

chimp.LOC112206744.LOC107973052.GGT2-.646279-708040.rev.compl      ------------------------------------------------------------   61762
FAM230B-LOC105372935-GGT2.NCBI.GGT2.rev.compl                      aaaaagattaagaaacattgcatcagacaataaattgtcaatgagcaaggtcctgtcctt   67598

chimp.LOC112206744.LOC107973052.GGT2-.646279-708040.rev.compl      ------------------------------------------------------------   61762
FAM230B-LOC105372935-GGT2.NCBI.GGT2.rev.compl                      ttagattgggttttgaaatcacccatcccacaaatatatgataccacttaactagttttt   67658

chimp.LOC112206744.LOC107973052.GGT2-.646279-708040.rev.compl      ------------------------------------------------------------   61762
FAM230B-LOC105372935-GGT2.NCBI.GGT2.rev.compl                      caagatattccttgttcaaagatgctcccactttttctttgtgctcaagtcctagataaa   67718

chimp.LOC112206744.LOC107973052.GGT2-.646279-708040.rev.compl      ------------------------------------------------------------   61762
FAM230B-LOC105372935-GGT2.NCBI.GGT2.rev.compl                      cttggctaggggcaggaggtatgattcaggagttagtgagccgggagggctcactgtgcc   67778

chimp.LOC112206744.LOC107973052.GGT2-.646279-708040.rev.compl      ------------------------------------------------------------   61762
FAM230B-LOC105372935-GGT2.NCBI.GGT2.rev.compl                      tgggaaagccagcttctcatgaattttggaatatgccttggctcagaaacctacctctc   67838

chimp.LOC112206744.LOC107973052.GGT2-.646279-708040.rev.compl      ------------------------------------------------------------   61762
FAM230B-LOC105372935-GGT2.NCBI.GGT2.rev.compl                      taggcacaggatccaacttatacgttcaagatgcgtgactgagcatgtgaaagtgtcaga   67898

chimp.LOC112206744.LOC107973052.GGT2-.646279-708040.rev.compl      ------------------------------------------------------------   61762
FAM230B-LOC105372935-GGT2.NCBI.GGT2.rev.compl                      gggatcgacctcaccattttacatctacttttccagttgtggcccctacaatcatcaagg   67958

chimp.LOC112206744.LOC107973052.GGT2-.646279-708040.rev.compl      ------------------------------------------------------------   61762
FAM230B-LOC105372935-GGT2.NCBI.GGT2.rev.compl                      ctcattcggcaccatgctcctccaaattctgcatgcgactcagccacactaatatctttc   68018

chimp.LOC112206744.LOC107973052.GGT2-.646279-708040.rev.compl      ------------------------------------------------------------   61762
FAM230B-LOC105372935-GGT2.NCBI.GGT2.rev.compl                      ttcatctataagatgaaagtaatacaacgcaccatgtagcaaactcacaggagaccctag   68078

chimp.LOC112206744.LOC107973052.GGT2-.646279-708040.rev.compl      ------------------------------------------------------------   61762
FAM230B-LOC105372935-GGT2.NCBI.GGT2.rev.compl                      agatcaaataacactgtgaaagccaccaaatgaatattttaaaattttatctcatttctg   68138

chimp.LOC112206744.LOC107973052.GGT2-.646279-708040.rev.compl      ------------------------------------------------------------   61762
FAM230B-LOC105372935-GGT2.NCBI.GGT2.rev.compl                      ccaaggtgagatatcaatctaaaaacaggataaatctttgtaagtacaagaacattaaaa   68198

chimp.LOC112206744.LOC107973052.GGT2-.646279-708040.rev.compl      ------------------------------------------------------------   61762
FAM230B-LOC105372935-GGT2.NCBI.GGT2.rev.compl                      agaacattaagtttgggttaaaaatgcaaaactgcatatgcaggaaaattacgttaaaaa   68258

chimp.LOC112206744.LOC107973052.GGT2-.646279-708040.rev.compl      ------------------------------------------------------------   61762
FAM230B-LOC105372935-GGT2.NCBI.GGT2.rev.compl                      aaaaaaaatcctacacctacaaaaacaaccaaacaaaacattgatgatactgggctgatt   68318

chimp.LOC112206744.LOC107973052.GGT2-.646279-708040.rev.compl      ------------------------------------------------------------   61762
FAM230B-LOC105372935-GGT2.NCBI.GGT2.rev.compl                      cactgaatggtgagaatatgagtgattttctcctctcctatttttctcaatacctttttct   68378

chimp.LOC112206744.LOC107973052.GGT2-.646279-708040.rev.compl      ------------------------------------------------------------   61762
FAM230B-LOC105372935-GGT2.NCBI.GGT2.rev.compl                      ctattatctcctttttaaataaacaagtatatattgttctaaaccgaagataaatgatc   68438

chimp.LOC112206744.LOC107973052.GGT2-.646279-708040.rev.compl      ------------------------------------------------------------   61762
FAM230B-LOC105372935-GGT2.NCBI.GGT2.rev.compl                      atgagcagcactttcccaggcactgtcctagttgctctgcatacatcatcgcatttaatc   68498

chimp.LOC112206744.LOC107973052.GGT2-.646279-708040.rev.compl      ------------------------------------------------------------   61762
FAM230B-LOC105372935-GGT2.NCBI.GGT2.rev.compl                      ctcacaacagtgaatggggtcgatttcattacagccacatttaccagacaaggaaatggt   68558

chimp.LOC112206744.LOC107973052.GGT2-.646279-708040.rev.compl      ------------------------------------------------------------   61762
FAM230B-LOC105372935-GGT2.NCBI.GGT2.rev.compl                      tttcaaggttaagtaaccttctcatgacacagaatgtgactaagagttttcttattccat   68618

chimp.LOC112206744.LOC107973052.GGT2-.646279-708040.rev.compl      ------------------------------------------------------------   61762
FAM230B-LOC105372935-GGT2.NCBI.GGT2.rev.compl                      ggctcacactaaatgtgctctgtgaatatcagtgagaagtaataaagcactgactggatt   68678

chimp.LOC112206744.LOC107973052.GGT2-.646279-708040.rev.compl      ------------------------------------------------------------   61762
FAM230B-LOC105372935-GGT2.NCBI.GGT2.rev.compl                      ttcttggtcaccgggtcctaaaagaaatgttggggaatctatgcagtcccaagttaaaaa   68738

chimp.LOC112206744.LOC107973052.GGT2-.646279-708040.rev.compl      ------------------------------------------------------------   61762
FAM230B-LOC105372935-GGT2.NCBI.GGT2.rev.compl                      tgggaactgaactgagctgccacagcatgctgaataggtggagtccaggccaactaagtg   68798

chimp.LOC112206744.LOC107973052.GGT2-.646279-708040.rev.compl      ------------------------------------------------------------   61762
FAM230B-LOC105372935-GGT2.NCBI.GGT2.rev.compl                      cttcatggaacttaaaggctccagagggaaagcaatcgggaaggcacttccacaggcaca   68858
```

```
chimp.LOC112206744.LOC107973052.GGT2-.646279-708040.rev.compl      ------------------------------------------------------------   61762
FAM230B-LOC105372935-GGT2.NCBI.GGT2.rev.compl                      tcggtgacaacttctggggattcttacctgtcagccttcagccaaggaaggacaggaagt   68918

chimp.LOC112206744.LOC107973052.GGT2-.646279-708040.rev.compl      ------------------------------------------------------------   61762
FAM230B-LOC105372935-GGT2.NCBI.GGT2.rev.compl                      ggcatgggctaaaagatcctttgtttgacctctttccagatcaggtgacaggaaaacaca   68978

chimp.LOC112206744.LOC107973052.GGT2-.646279-708040.rev.compl      ------------------------------------------------------------   61762
FAM230B-LOC105372935-GGT2.NCBI.GGT2.rev.compl                      acacttgaacctttccaataaggacagccagcacttactgagccccagccaggtaactga   69038

chimp.LOC112206744.LOC107973052.GGT2-.646279-708040.rev.compl      ------------------------------------------------------------   61762
FAM230B-LOC105372935-GGT2.NCBI.GGT2.rev.compl                      cactgttccaagtactctgcttgtataaccagctttaaccttgcaacagccctctgaggt   69098

chimp.LOC112206744.LOC107973052.GGT2-.646279-708040.rev.compl      ------------------------------------------------------------   61762
FAM230B-LOC105372935-GGT2.NCBI.GGT2.rev.compl                      aaatactcctagtgacctcactttacggatgcagaacctgaggcaaggagttagggagta   69158

chimp.LOC112206744.LOC107973052.GGT2-.646279-708040.rev.compl      ------------------------------------------------------------   61762
FAM230B-LOC105372935-GGT2.NCBI.GGT2.rev.compl                      aatgaagctggctaaaggcacttgcactggccaggcacagtggctcatgcctgtaatccc   69218

chimp.LOC112206744.LOC107973052.GGT2-.646279-708040.rev.compl      ------------------------------------------------------------   61762
FAM230B-LOC105372935-GGT2.NCBI.GGT2.rev.compl                      agcacttccagatggatcacttgaggttaggggttcgagaccagcctggccaacatggtg   69278

chimp.LOC112206744.LOC107973052.GGT2-.646279-708040.rev.compl      ------------------------------------------------------------   61762
FAM230B-LOC105372935-GGT2.NCBI.GGT2.rev.compl                      aatccccagctctactaaaaatacaaaaattagccgggtgtgatggcacaagcctgtaat   69338

chimp.LOC112206744.LOC107973052.GGT2-.646279-708040.rev.compl      ------------------------------------------------------------   61762
FAM230B-LOC105372935-GGT2.NCBI.GGT2.rev.compl                      cccagcactttgggaggccaaggcagatgggtcacttgaggttaggggttcgagaccagc   69398

chimp.LOC112206744.LOC107973052.GGT2-.646279-708040.rev.compl      ------------------------------------------------------------   61762
FAM230B-LOC105372935-GGT2.NCBI.GGT2.rev.compl                      ctggccaacatggtgaatccccagctctactaaaaatacaaaaattagccgggtgtgatg   69458

chimp.LOC112206744.LOC107973052.GGT2-.646279-708040.rev.compl      ------------------------------------------------------------   61762
FAM230B-LOC105372935-GGT2.NCBI.GGT2.rev.compl                      gcacacgcctgtaatcccagctactcaggaggctgaggcaggagaattgcttgaacctgg   69518

chimp.LOC112206744.LOC107973052.GGT2-.646279-708040.rev.compl      ------------------------------------------------------------   61762
FAM230B-LOC105372935-GGT2.NCBI.GGT2.rev.compl                      gagatggaggttgcagcaagccgagatcgcaccactgcactccagcccactcggtcaatg   69578

chimp.LOC112206744.LOC107973052.GGT2-.646279-708040.rev.compl      ------------------------------------------------------------   61762
FAM230B-LOC105372935-GGT2.NCBI.GGT2.rev.compl                      tcactgccatcaagggacagtctaaccaaggtccagatggcacaagtggagccataaaac   69638

chimp.LOC112206744.LOC107973052.GGT2-.646279-708040.rev.compl      ------------------------------------------------------------   61762
FAM230B-LOC105372935-GGT2.NCBI.GGT2.rev.compl                      caggacagcttgaggttcagcttcctaagcccacaggtgcaaggaccttatctggatcca   69698

chimp.LOC112206744.LOC107973052.GGT2-.646279-708040.rev.compl      ------------------------------------------------------------   61762
FAM230B-LOC105372935-GGT2.NCBI.GGT2.rev.compl                      tactttcatcaaaaatgtcccttgaggccaggcacagtggctcatgcctataatcccagc   69758

chimp.LOC112206744.LOC107973052.GGT2-.646279-708040.rev.compl      ------------------------------------------------------------   61762
FAM230B-LOC105372935-GGT2.NCBI.GGT2.rev.compl                      actttgggagggcgaggcaggtggatcacctgaggtcaggagttcgagaccagcctggcc   69818

chimp.LOC112206744.LOC107973052.GGT2-.646279-708040.rev.compl      ------------------------------------------------------------   61762
FAM230B-LOC105372935-GGT2.NCBI.GGT2.rev.compl                      aatatggtgaaaccctgtccctactgaaaatacaaaaatttagcccagcatggtgatgtg   69878

chimp.LOC112206744.LOC107973052.GGT2-.646279-708040.rev.compl      ------------------------------------------------------------   61762
FAM230B-LOC105372935-GGT2.NCBI.GGT2.rev.compl                      cgcctgtaatcccagctactccggaggctgaggcaggagaatcgcttgaacccagcaggt   69938

chimp.LOC112206744.LOC107973052.GGT2-.646279-708040.rev.compl      ------------------------------------------------------------   61762
FAM230B-LOC105372935-GGT2.NCBI.GGT2.rev.compl                      ggaggttgcagtgagccgagatcatgtcattgcactgtgggctgggcaacagagcaacac   69998

chimp.LOC112206744.LOC107973052.GGT2-.646279-708040.rev.compl      ------------------------------------------------------------   61762
FAM230B-LOC105372935-GGT2.NCBI.GGT2.rev.compl                      tctatgtaaaaaaaaaaaaaaaggtcccttcaggattcctcgagcctgggaggccgaggc   70058

chimp.LOC112206744.LOC107973052.GGT2-.646279-708040.rev.compl      ------------------------------------------------------------   61762
FAM230B-LOC105372935-GGT2.NCBI.GGT2.rev.compl                      tgccatgaacaatgattgtgccactgcactccagcctgggtgacagagcgagatcttgtc   70118

chimp.LOC112206744.LOC107973052.GGT2-.646279-708040.rev.compl      ------------------------------------------------------------   61762
FAM230B-LOC105372935-GGT2.NCBI.GGT2.rev.compl                      tcaaaaaaaaaaaaaaaaaaaaaaatggctcttgaaacagccatcaactcccatgtgata   70178

chimp.LOC112206744.LOC107973052.GGT2-.646279-708040.rev.compl      ------------------------------------------------------------   61762
FAM230B-LOC105372935-GGT2.NCBI.GGT2.rev.compl                      aatgcactgccacaatccagcagcaaacctaaagttcggagtgaattcctaagtcttcgc   70238

chimp.LOC112206744.LOC107973052.GGT2-.646279-708040.rev.compl      ------------------------------------------------------------   61762
FAM230B-LOC105372935-GGT2.NCBI.GGT2.rev.compl                      cttcactgacttcccacagcatgggcagtagctgtgagaccttgggctggttacttaacc   70298

chimp.LOC112206744.LOC107973052.GGT2-.646279-708040.rev.compl      ------------------------------------------------------------   61762
FAM230B-LOC105372935-GGT2.NCBI.GGT2.rev.compl                      tctctgcacctcagtttcctcatctgtaaagtggtataatcgtcacagtgttatggaagg   70358

chimp.LOC112206744.LOC107973052.GGT2-.646279-708040.rev.compl      ------------------------------------------------------------   61762
FAM230B-LOC105372935-GGT2.NCBI.GGT2.rev.compl                      atttaatgagttgctacatttaaaacttaattcctggcacacaataagtgctcaataaat   70418

chimp.LOC112206744.LOC107973052.GGT2-.646279-708040.rev.compl      ------------------------------------------------------------   61762
FAM230B-LOC105372935-GGT2.NCBI.GGT2.rev.compl                      gtttagccgctagttgcagtagtagcaatagtagtgatattaactctagctgtggaacta   70478

chimp.LOC112206744.LOC107973052.GGT2-.646279-708040.rev.compl      ------------------------------------------------------------   61762
FAM230B-LOC105372935-GGT2.NCBI.GGT2.rev.compl                      atttctcccacacaccgtggcaggttgtattctccagacacagcagtaacaatatctcct   70538

chimp.LOC112206744.LOC107973052.GGT2-.646279-708040.rev.compl      ------------------------------------------------------------   61762
FAM230B-LOC105372935-GGT2.NCBI.GGT2.rev.compl                      tcccacacgctcttttgcaatgtgaccatgccacacactcccatcaagaggtggagttta   70598

chimp.LOC112206744.LOC107973052.GGT2-.646279-708040.rev.compl      ------------------------------------------------------------   61762
FAM230B-LOC105372935-GGT2.NCBI.GGT2.rev.compl                      gaataattccctcctctgtggatctgggcaggaagtcactgtgtgacttccagtcctagg   70658

chimp.LOC112206744.LOC107973052.GGT2-.646279-708040.rev.compl      ------------------------------------------------------------   61762
FAM230B-LOC105372935-GGT2.NCBI.GGT2.rev.compl                      tcatgacaagtgatgtagcttccatttcactcagggtgccctgaaccaccacataagaaa   70718

chimp.LOC112206744.LOC107973052.GGT2-.646279-708040.rev.compl      ------------------------------------------------------------   61762
FAM230B-LOC105372935-GGT2.NCBI.GGT2.rev.compl                      gccaacggtcctgaggccatgctgtgagggggcccaaggcacatggagaggcccagggca   70778

chimp.LOC112206744.LOC107973052.GGT2-.646279-708040.rev.compl      ------------------------------------------------------------   61762
FAM230B-LOC105372935-GGT2.NCBI.GGT2.rev.compl                      ggtgctccagtccagagctggctccagccaggtcccccctgccaccatgtgagtgaatgag   70838

chimp.LOC112206744.LOC107973052.GGT2-.646279-708040.rev.compl      ------------------------------------------------------------   61762
FAM230B-LOC105372935-GGT2.NCBI.GGT2.rev.compl                      ccaatcaccacccagctccacctgccaccatgtgagtgaacaagcctcccctgtgagctg   70898

chimp.LOC112206744.LOC107973052.GGT2-.646279-708040.rev.compl      ------------------------------------------------------------   61762
FAM230B-LOC105372935-GGT2.NCBI.GGT2.rev.compl                      cccccagccttggaacttccagccatgcctgccgtgcctgtaaattctcaacccccagat   70958

chimp.LOC112206744.LOC107973052.GGT2-.646279-708040.rev.compl      ------------------------------------------------------------   61762
FAM230B-LOC105372935-GGT2.NCBI.GGT2.rev.compl                      tctgtgagcataacaaaattgttgttttaagccactgaggttgggaaacatttgttacat   71018
```

```
chimp.LOC112206744.LOC107973052.GGT2-.646279-708040.rev.compl      ------------------------------------------------------------   61762
FAM230B-LOC105372935-GGT2.NCBI.GGT2.rev.compl                      agaaaggtaacaagaacatatgctatggcttcaatcaacatttttttcagtacctattat   71078

chimp.LOC112206744.LOC107973052.GGT2-.646279-708040.rev.compl      ------------------------------------------------------------   61762
FAM230B-LOC105372935-GGT2.NCBI.GGT2.rev.compl                      gatggacactaggacagaaactgtgagggagagattaataagacatagttgacaagttgt   71138

chimp.LOC112206744.LOC107973052.GGT2-.646279-708040.rev.compl      ------------------------------------------------------------   61762
FAM230B-LOC105372935-GGT2.NCBI.GGT2.rev.compl                      cctaagctgacaatcctaaaataaatacacataactaatcattttaatggtatacgatag   71198

chimp.LOC112206744.LOC107973052.GGT2-.646279-708040.rev.compl      ------------------------------------------------------------   61762
FAM230B-LOC105372935-GGT2.NCBI.GGT2.rev.compl                      cagtgaggtgcaaagtgcaaggttaagccctttcagaaagtgaaagcattcaaaaagcct   71258

chimp.LOC112206744.LOC107973052.GGT2-.646279-708040.rev.compl      ------------------------------------------------------------   61762
FAM230B-LOC105372935-GGT2.NCBI.GGT2.rev.compl                      tcaaaaaagtagtgtcatctgaattggatcccaaagcgcaagggaaatatataccacatg   71318

chimp.LOC112206744.LOC107973052.GGT2-.646279-708040.rev.compl      ------------------------------------------------------------   61762
FAM230B-LOC105372935-GGT2.NCBI.GGT2.rev.compl                      aaaacaccgcagtgcattccaggattagcaagaagtttgcggaggtcgttttgtcacatg   71378

chimp.LOC112206744.LOC107973052.GGT2-.646279-708040.rev.compl      ------------------------------------------------------------   61762
FAM230B-LOC105372935-GGT2.NCBI.GGT2.rev.compl                      actggagggcacatcagtgccaggcggaaaagggcaccgaaaggcacattaataaagtca   71438

chimp.LOC112206744.LOC107973052.GGT2-.646279-708040.rev.compl      ------------------------------------------------------------   61762
FAM230B-LOC105372935-GGT2.NCBI.GGT2.rev.compl                      aacacctcctctgggacaggggaagttgagaaagagcctaacagaggtaaggaagaagac   71498

chimp.LOC112206744.LOC107973052.GGT2-.646279-708040.rev.compl      ------------------------------------------------------------   61762
FAM230B-LOC105372935-GGT2.NCBI.GGT2.rev.compl                      ggatcagatttacactttcgaattatcactctggtggcaggtgaaggacagagcagaggg   71558

chimp.LOC112206744.LOC107973052.GGT2-.646279-708040.rev.compl      ------------------------------------------------------------   61762
FAM230B-LOC105372935-GGT2.NCBI.GGT2.rev.compl                      caggactggaagcagacagaccaaattcacagtcaaatgcgacaagatgagggcctggcc   71618

chimp.LOC112206744.LOC107973052.GGT2-.646279-708040.rev.compl      ------------------------------------------------------------   61762
FAM230B-LOC105372935-GGT2.NCBI.GGT2.rev.compl                      tggttttaaatccaaggacaaaaatatcattcattcactctttcattctttctcttatct   71678

chimp.LOC112206744.LOC107973052.GGT2-.646279-708040.rev.compl      ------------------------------------------------------------   61762
FAM230B-LOC105372935-GGT2.NCBI.GGT2.rev.compl                      gaatccccagtggtacctaaaatagagctggtctaaagatgatgattaatgaatgttaaa   71738

chimp.LOC112206744.LOC107973052.GGT2-.646279-708040.rev.compl      ------------------------------------------------------------   61762
FAM230B-LOC105372935-GGT2.NCBI.GGT2.rev.compl                      ttattgatactacttctaagaagatgtaactgccagctacagaaccagccaaaaggactg   71798

chimp.LOC112206744.LOC107973052.GGT2-.646279-708040.rev.compl      ------------------------------------------------------------   61762
FAM230B-LOC105372935-GGT2.NCBI.GGT2.rev.compl                      caaacccagtaattcatttcatcccaacctgactgatctctctgatgtctgcactgctgt   71858

chimp.LOC112206744.LOC107973052.GGT2-.646279-708040.rev.compl      ------------------------------------------------------------   61762
FAM230B-LOC105372935-GGT2.NCBI.GGT2.rev.compl                      gtcactagactgcccgattgtcctaggttgagacatttacctactgagccccagggatgg   71918

chimp.LOC112206744.LOC107973052.GGT2-.646279-708040.rev.compl      ------------------------------------------------------------   61762
FAM230B-LOC105372935-GGT2.NCBI.GGT2.rev.compl                      gtggctgcttctaagctgctgcctaattccctcattactaaatgttgacgctgcttcctt   71978

chimp.LOC112206744.LOC107973052.GGT2-.646279-708040.rev.compl      ------------------------------------------------------------   61762
FAM230B-LOC105372935-GGT2.NCBI.GGT2.rev.compl                      agaggactgctaaaccataactcaaaaatgttttaaacatgctactatttacttagatta   72038

chimp.LOC112206744.LOC107973052.GGT2-.646279-708040.rev.compl      ------------------------------------------------------------   61762
FAM230B-LOC105372935-GGT2.NCBI.GGT2.rev.compl                      gaatatttacttaaatgttttaaaaaatacagtattttaagtcttttatctcttctgact   72098

chimp.LOC112206744.LOC107973052.GGT2-.646279-708040.rev.compl      ------------------------------------------------------------   61762
FAM230B-LOC105372935-GGT2.NCBI.GGT2.rev.compl                      actaccactaaccctgcttcccctcatttttccagagattagcactactgtgatttaagg   72158

chimp.LOC112206744.LOC107973052.GGT2-.646279-708040.rev.compl      ------------------------------------------------------------   61762
FAM230B-LOC105372935-GGT2.NCBI.GGT2.rev.compl                      atagagctctccagacttaaaaaaaaaccaaaaaactgcttttatatccagatatatgta   72218

chimp.LOC112206744.LOC107973052.GGT2-.646279-708040.rev.compl      ------------------------------------------------------------   61762
FAM230B-LOC105372935-GGT2.NCBI.GGT2.rev.compl                      accactgaaaaatatacattatttttcagcatttttacataaacattatcatccctttc   72278

chimp.LOC112206744.LOC107973052.GGT2-.646279-708040.rev.compl      ------------------------------------------------------------   61762
FAM230B-LOC105372935-GGT2.NCBI.GGT2.rev.compl                      attgagttatgttcctcagttttcctcctcaaagtttttgagatcaaatatagttcccat   72338

chimp.LOC112206744.LOC107973052.GGT2-.646279-708040.rev.compl      ------------------------------------------------------------   61762
FAM230B-LOC105372935-GGT2.NCBI.GGT2.rev.compl                      ttttcttctttatatagctgcacagtattccatcataggattacaccacattttatttat   72398

chimp.LOC112206744.LOC107973052.GGT2-.646279-708040.rev.compl      ------------------------------------------------------------   61762
FAM230B-LOC105372935-GGT2.NCBI.GGT2.rev.compl                      tccaatattaatgaatatttaggttatttatactttttttttctataaccaacaagcccat   72458

chimp.LOC112206744.LOC107973052.GGT2-.646279-708040.rev.compl      ------------------------------------------------------------   61762
FAM230B-LOC105372935-GGT2.NCBI.GGT2.rev.compl                      gccttctactgcttatcctttccccacctgaatttgtacaatccccagcttctcttatcc   72518

chimp.LOC112206744.LOC107973052.GGT2-.646279-708040.rev.compl      ------------------------------------------------------------   61762
FAM230B-LOC105372935-GGT2.NCBI.GGT2.rev.compl                      atgaccactgaaaccccagggaagaccttatcactttcaggatgtccatggctctttcac   72578

chimp.LOC112206744.LOC107973052.GGT2-.646279-708040.rev.compl      ------------------------------------------------------------   61762
FAM230B-LOC105372935-GGT2.NCBI.GGT2.rev.compl                      cctatgccatgcttggaaccaggaaagcccaaaagaccccacttcctgcccttctattct   72638

chimp.LOC112206744.LOC107973052.GGT2-.646279-708040.rev.compl      ------------------------------------------------------------   61762
FAM230B-LOC105372935-GGT2.NCBI.GGT2.rev.compl                      gctcctcctctcctatcgctcatttctttgcctctatcctccactcctcccagcaaaggt   72698

chimp.LOC112206744.LOC107973052.GGT2-.646279-708040.rev.compl      ------------------------------------------------------------   61762
FAM230B-LOC105372935-GGT2.NCBI.GGT2.rev.compl                      agggatagcacctgagtcctgccaccctctcacctgcattccccctcactctcatgcgaa   72758

chimp.LOC112206744.LOC107973052.GGT2-.646279-708040.rev.compl      ------------------------------------------------------------   61762
FAM230B-LOC105372935-GGT2.NCBI.GGT2.rev.compl                      ggacagcacagaagcagcatcaaaaaagaaaggcagcgagggagccctgcagcctgccct   72818

chimp.LOC112206744.LOC107973052.GGT2-.646279-708040.rev.compl      ------------------------------------------------------------   61762
FAM230B-LOC105372935-GGT2.NCBI.GGT2.rev.compl                      aggccagccatgcccacagccacaggaatagacactgttttcagccccacctagattcaa   72878

chimp.LOC112206744.LOC107973052.GGT2-.646279-708040.rev.compl      ------------------------------------------------------------   61762
FAM230B-LOC105372935-GGT2.NCBI.GGT2.rev.compl                      ggcagcatctaatagaaatgcttataagtaaatataagtgccagtatttgccttctattt   72938

chimp.LOC112206744.LOC107973052.GGT2-.646279-708040.rev.compl      ------------------------------------------------------------   61762
FAM230B-LOC105372935-GGT2.NCBI.GGT2.rev.compl                      tctagaggggaaaaaaattaaaaggcaagatcagattacttgcctggaaatgttctccct   72998

chimp.LOC112206744.LOC107973052.GGT2-.646279-708040.rev.compl      ------------------------------------------------------------   61762
FAM230B-LOC105372935-GGT2.NCBI.GGT2.rev.compl                      gtaattactctgaccgctctttctttccctccggttttggagataatggaaataggctga   73058

chimp.LOC112206744.LOC107973052.GGT2-.646279-708040.rev.compl      ------------------------------------------------------------   61762
FAM230B-LOC105372935-GGT2.NCBI.GGT2.rev.compl                      gtgttgggcatttttttttttaaagagggcagcttcatggcaacactccctctgagaagt   73118

chimp.LOC112206744.LOC107973052.GGT2-.646279-708040.rev.compl      ------------------------------------------------------------   61762
FAM230B-LOC105372935-GGT2.NCBI.GGT2.rev.compl                      ccagaggctcagtgaaccgcagcttagagtgagttccagtaaaaggaccatcacttgaac   73178

chimp.LOC112206744.LOC107973052.GGT2-.646279-708040.rev.compl      ------------------------------------------------------------   61762
```

```
FAM230B-LOC105372935-GGT2.NCBI.GGT2.rev.compl                cagtaagtgcctcattccttctcacagcatacatttcacagcaattactgttgctattat   73238

chimp.LOC112206744.LOC107973052.GGT2-.646279-708040.rev.compl  ------------------------------------------------------------   61762
FAM230B-LOC105372935-GGT2.NCBI.GGT2.rev.compl                tttacgcttcatttctggacctaggatacctcagtttctccagactctcagtctggtccc   73298

chimp.LOC112206744.LOC107973052.GGT2-.646279-708040.rev.compl  ------------------------------------------------------------   61762
FAM230B-LOC105372935-GGT2.NCBI.GGT2.rev.compl                caggttttcagtctgggagaaaggtgtctgctggatttcaaacagtccctctactggccg   73358

chimp.LOC112206744.LOC107973052.GGT2-.646279-708040.rev.compl  ------------------------------------------------------------   61762
FAM230B-LOC105372935-GGT2.NCBI.GGT2.rev.compl                ccaccaccaccctctcctaaagaggactggaataactcttccacagctcccgccacactc   73418

chimp.LOC112206744.LOC107973052.GGT2-.646279-708040.rev.compl  ------------------------------------------------------------   61762
FAM230B-LOC105372935-GGT2.NCBI.GGT2.rev.compl                agcactgcctgcacctcctcctggagctggcagggccccagctgtgcacccctcaatgct   73478

chimp.LOC112206744.LOC107973052.GGT2-.646279-708040.rev.compl  ------------------------------------------------------------   61762
FAM230B-LOC105372935-GGT2.NCBI.GGT2.rev.compl                ctcacagcttcaggaagaggactgctaagcccagggccctgctgacaacatgccgggagc   73538

chimp.LOC112206744.LOC107973052.GGT2-.646279-708040.rev.compl  ------------------------------------------------------------   61762
FAM230B-LOC105372935-GGT2.NCBI.GGT2.rev.compl                gcatctgcaggcagggtcagaatgtcagctaccaggcagcagagagatgcggggcgccaa   73598

chimp.LOC112206744.LOC107973052.GGT2-.646279-708040.rev.compl  ------------------------------------------------------------   61762
FAM230B-LOC105372935-GGT2.NCBI.GGT2.rev.compl                cagcaaaagcacttgggggctttttcaagtggcatcagaactagacctggatacaaagg   73658

chimp.LOC112206744.LOC107973052.GGT2-.646279-708040.rev.compl  ------------------------------------------------------------   61762
FAM230B-LOC105372935-GGT2.NCBI.GGT2.rev.compl                ggtggccctggcttcaggaagcccatgataagtcagtctgctgtgtccttcaacacagtg   73718

chimp.LOC112206744.LOC107973052.GGT2-.646279-708040.rev.compl  ------------------------------------------------------------   61762
FAM230B-LOC105372935-GGT2.NCBI.GGT2.rev.compl                tccccaccttctgtctcccaacactaggccatggagacacaaaactagaaaatggggctg   73778

chimp.LOC112206744.LOC107973052.GGT2-.646279-708040.rev.compl  ------------------------------------------------------------   61762
FAM230B-LOC105372935-GGT2.NCBI.GGT2.rev.compl                cctagctacgaatgcccaggctgtgaattggggtctccaaaggaacctcagtgccgtgtc   73838

chimp.LOC112206744.LOC107973052.GGT2-.646279-708040.rev.compl  ------------------------------------------------------------   61762
FAM230B-LOC105372935-GGT2.NCBI.GGT2.rev.compl                ctactcagagcagctccttcagctcttcaccctggaaagcactccggtacaaaacaggaa   73898

chimp.LOC112206744.LOC107973052.GGT2-.646279-708040.rev.compl  ------------------------------------------------------------   61762
FAM230B-LOC105372935-GGT2.NCBI.GGT2.rev.compl                tattcctcttctcctgactctgcccaaggcactgctgtgaccctctcagcagtggaagca   73958

chimp.LOC112206744.LOC107973052.GGT2-.646279-708040.rev.compl  ------------------------------------------------------------   61762
FAM230B-LOC105372935-GGT2.NCBI.GGT2.rev.compl                ctagccaatgtgccatcctcctggacgatagctggataatcgcaataagcagagccccaa   74018

chimp.LOC112206744.LOC107973052.GGT2-.646279-708040.rev.compl  ------------------------------------------------------------   61762
FAM230B-LOC105372935-GGT2.NCBI.GGT2.rev.compl                caactagctctagtgtgtggggagaagtggggaggatggagaaaggatacaaaaagacac   74078

chimp.LOC112206744.LOC107973052.GGT2-.646279-708040.rev.compl  ------------------------------------------------------------   61762
FAM230B-LOC105372935-GGT2.NCBI.GGT2.rev.compl                tgtgggatttcagggcaacccatgagtgaggatcacagcagagctatgccttgcaaggac   74138

chimp.LOC112206744.LOC107973052.GGT2-.646279-708040.rev.compl  ------------------------------------------------------------   61762
FAM230B-LOC105372935-GGT2.NCBI.GGT2.rev.compl                cagaactggaaaacctttgtgacttagaccgccctcctcaggacagctcccgccactctt   74198

chimp.LOC112206744.LOC107973052.GGT2-.646279-708040.rev.compl  ------------------------------------------------------------   61762
FAM230B-LOC105372935-GGT2.NCBI.GGT2.rev.compl                cagggacaaagttcctttactcagccatagtttctgtgctccaaatactcagcttacaca   74258

chimp.LOC112206744.LOC107973052.GGT2-.646279-708040.rev.compl  ------------------------------------------------------------   61762
FAM230B-LOC105372935-GGT2.NCBI.GGT2.rev.compl                tgcctgttgctgctgcactcaccactgtggttctcagtgcaaattccccagaaagcagtc   74318

chimp.LOC112206744.LOC107973052.GGT2-.646279-708040.rev.compl  ------------------------------------------------------------   61762
FAM230B-LOC105372935-GGT2.NCBI.GGT2.rev.compl                tgactggctgggagacctttttctttaaccaggcccccaaagttgtagattttggggt   74378

chimp.LOC112206744.LOC107973052.GGT2-.646279-708040.rev.compl  ------------------------------------------------------------   61762
FAM230B-LOC105372935-GGT2.NCBI.GGT2.rev.compl                catttacaatatgcatttccccaacacactcagatctaggtggacttgaccagatgggca   74438

chimp.LOC112206744.LOC107973052.GGT2-.646279-708040.rev.compl  ------------------------------------------------------------   61762
FAM230B-LOC105372935-GGT2.NCBI.GGT2.rev.compl                tctgtattccattacccagcatataaccactttgggcaacagggaatgtgaagggcccca   74498

chimp.LOC112206744.LOC107973052.GGT2-.646279-708040.rev.compl  ------------------------------------------------------------   61762
FAM230B-LOC105372935-GGT2.NCBI.GGT2.rev.compl                aaccgttccccaccaagagtgtgggtggcaggcaccctgaaatatgtctagcacactcat   74558

chimp.LOC112206744.LOC107973052.GGT2-.646279-708040.rev.compl  ------------------------------------------------------------   61762
FAM230B-LOC105372935-GGT2.NCBI.GGT2.rev.compl                aaagcatgaacatctacacacaactaaaaataaatagtgagagccctgacactgcttctg   74618

chimp.LOC112206744.LOC107973052.GGT2-.646279-708040.rev.compl  ------------------------------------------------------------   61762
FAM230B-LOC105372935-GGT2.NCBI.GGT2.rev.compl                ggggttttcccaagcacctgttacgaatggtaacaatggatggccgctccttctccctca   74678

chimp.LOC112206744.LOC107973052.GGT2-.646279-708040.rev.compl  ------------------------------------------------------------   61762
FAM230B-LOC105372935-GGT2.NCBI.GGT2.rev.compl                gctttgactttcattgctggtggctcaggctcctgtgcttggctgtcatcggtgggtagc   74738

chimp.LOC112206744.LOC107973052.GGT2-.646279-708040.rev.compl  ------------------------------------------------------------   61762
FAM230B-LOC105372935-GGT2.NCBI.GGT2.rev.compl                cctggattggacattttccctattcaattcaaggaccatgttcttgagcataactatgac   74798

chimp.LOC112206744.LOC107973052.GGT2-.646279-708040.rev.compl  ------------------------------------------------------------   61762
FAM230B-LOC105372935-GGT2.NCBI.GGT2.rev.compl                caggcagtaggcagtgggccctgctctttctttccagggaggcacagataagccagcatc   74858

chimp.LOC112206744.LOC107973052.GGT2-.646279-708040.rev.compl  ------------------------------------------------------------   61762
FAM230B-LOC105372935-GGT2.NCBI.GGT2.rev.compl                acagatgcaaaaaacagaaccgtggaggtagagactaattcttcagcagcccagagaaga   74918

chimp.LOC112206744.LOC107973052.GGT2-.646279-708040.rev.compl  ------------------------------------------------------------   61762
FAM230B-LOC105372935-GGT2.NCBI.GGT2.rev.compl                gaactgggaaacagggccctcgcagatacacaagaccaagctggagagttcagggacttc   74978

chimp.LOC112206744.LOC107973052.GGT2-.646279-708040.rev.compl  ------------------------------------------------------------   61762
FAM230B-LOC105372935-GGT2.NCBI.GGT2.rev.compl                caaaagaaggtacaagaatgagcactataccatagtggggtgggtcacaaaagcaggaaa   75038

chimp.LOC112206744.LOC107973052.GGT2-.646279-708040.rev.compl  ------------------------------------------------------------   61762
FAM230B-LOC105372935-GGT2.NCBI.GGT2.rev.compl                aggtccccaaacaaagaatccatcagtcagttacagcactctccacatgtggtgaaacag   75098

chimp.LOC112206744.LOC107973052.GGT2-.646279-708040.rev.compl  ------------------------------------------------------------   61762
FAM230B-LOC105372935-GGT2.NCBI.GGT2.rev.compl                tatgcaaccatttaaaaactgcagagaacaatatttatgaacacctaaaaatgctgaaag   75158

chimp.LOC112206744.LOC107973052.GGT2-.646279-708040.rev.compl  ------------------------------------------------------------   61762
FAM230B-LOC105372935-GGT2.NCBI.GGT2.rev.compl                cacactactaagtaaaaggggagaaaggctataaaaggatttgaaagtattttcgtaaag   75218

chimp.LOC112206744.LOC107973052.GGT2-.646279-708040.rev.compl  ------------------------------------------------------------   61762
FAM230B-LOC105372935-GGT2.NCBI.GGT2.rev.compl                ataaacacccacacacacccacacacctctatatatgtgtggaaaaaggactgggctagt   75278

chimp.LOC112206744.LOC107973052.GGT2-.646279-708040.rev.compl  ------------------------------------------------------------   61762
FAM230B-LOC105372935-GGT2.NCBI.GGT2.rev.compl                atatttaaaaaacgctaaagggaggtcgggtaataagggattgtttttccttgcacttt   75338

chimp.LOC112206744.LOC107973052.GGT2-.646279-708040.rev.compl  ------------------------------------------------------------   61762
FAM230B-LOC105372935-GGT2.NCBI.GGT2.rev.compl                gtgttttctaagtattctatagtctgaatgaattagattcataattaggaccaacagtca   75398
```

```
chimp.LOC112206744.LOC107973052.GGT2-.646279-708040.rev.compl      ------------------------------------------------------------   61762
FAM230B-LOC105372935-GGT2.NCBI.GGT2.rev.compl                      taagcaatttctcagaccacctgcctcataaaacccaaaaaaagaagtcagagaggggtg   75458

chimp.LOC112206744.LOC107973052.GGT2-.646279-708040.rev.compl      ------------------------------------------------------------   61762
FAM230B-LOC105372935-GGT2.NCBI.GGT2.rev.compl                      aatattttggtacagatgaattcaggttgtgtaagtggagaggagggaggtggccaagga   75518

chimp.LOC112206744.LOC107973052.GGT2-.646279-708040.rev.compl      ------------------------------------------------------------   61762
FAM230B-LOC105372935-GGT2.NCBI.GGT2.rev.compl                      gcctgaagtgggggagggggctgatggtgaaatagggaatgggtggagagagaaaatga   75578

chimp.LOC112206744.LOC107973052.GGT2-.646279-708040.rev.compl      ------------------------------------------------------------   61762
FAM230B-LOC105372935-GGT2.NCBI.GGT2.rev.compl                      ttgcaaaattggctggagccagcctggtcaggtcaactttgcaggtgtgtgaagtgtcaa   75638

chimp.LOC112206744.LOC107973052.GGT2-.646279-708040.rev.compl      ------------------------------------------------------------   61762
FAM230B-LOC105372935-GGT2.NCBI.GGT2.rev.compl                      atggttaatcagttgccctctgtttgagctgcttaatgcacaagggcctggttgaacagg   75698

chimp.LOC112206744.LOC107973052.GGT2-.646279-708040.rev.compl      ------------------------------------------------------------   61762
FAM230B-LOC105372935-GGT2.NCBI.GGT2.rev.compl                      tttgacctcatttactttttcttaaagctgtttagccactttcctaaaaactcctatttt   75758

chimp.LOC112206744.LOC107973052.GGT2-.646279-708040.rev.compl      ------------------------------------------------------------   61762
FAM230B-LOC105372935-GGT2.NCBI.GGT2.rev.compl                      ctgagttattaaactttccattccatgttgatcccttccgagccctctaacgcatctctt   75818

chimp.LOC112206744.LOC107973052.GGT2-.646279-708040.rev.compl      ------------------------------------------------------------   61762
FAM230B-LOC105372935-GGT2.NCBI.GGT2.rev.compl                      ctcttgcttcttccaactggatgccttcatgaggcattaacaggtactgtgagccctgat   75878

chimp.LOC112206744.LOC107973052.GGT2-.646279-708040.rev.compl      ------------------------------------------------------------   61762
FAM230B-LOC105372935-GGT2.NCBI.GGT2.rev.compl                      gaaaatcatcctttcagggtacaaggaaggcacctgaatgacagatggttggaggaatct   75938

chimp.LOC112206744.LOC107973052.GGT2-.646279-708040.rev.compl      ------------------------------------------------------------   61762
FAM230B-LOC105372935-GGT2.NCBI.GGT2.rev.compl                      tgagaaagcagtttaggcattaacatgcaggctgtcgctgacttacacagagccaaggac   75998

chimp.LOC112206744.LOC107973052.GGT2-.646279-708040.rev.compl      ------------------------------------------------------------   61762
FAM230B-LOC105372935-GGT2.NCBI.GGT2.rev.compl                      gtgacatacagcatggggccatttctaaatccccactgaggacaagggtcctggagggaa   76058

chimp.LOC112206744.LOC107973052.GGT2-.646279-708040.rev.compl      ------------------------------------------------------------   61762
FAM230B-LOC105372935-GGT2.NCBI.GGT2.rev.compl                      tgggatttggaacaagatttgggctttgaacattcgctacaggctctgacaaagcagtga   76118

chimp.LOC112206744.LOC107973052.GGT2-.646279-708040.rev.compl      ------------------------------------------------------------   61762
FAM230B-LOC105372935-GGT2.NCBI.GGT2.rev.compl                      tggttcaaaagcctctgccccctggtggcaagccctgcccagatgctgttaaggggctct   76178

chimp.LOC112206744.LOC107973052.GGT2-.646279-708040.rev.compl      ------------------------------------------------------------   61762
FAM230B-LOC105372935-GGT2.NCBI.GGT2.rev.compl                      ccaggcacgccctcctaattaggggcaaaatcagagaagcctctttggaagaagacaaaa   76238

chimp.LOC112206744.LOC107973052.GGT2-.646279-708040.rev.compl      ------------------------------------------------------------   61762
FAM230B-LOC105372935-GGT2.NCBI.GGT2.rev.compl                      ggcacaatgagatgcggtctcctgaagtagaggctttctctctggttatcacctgaaaca   76298

chimp.LOC112206744.LOC107973052.GGT2-.646279-708040.rev.compl      ------------------------------------------------------------   61762
FAM230B-LOC105372935-GGT2.NCBI.GGT2.rev.compl                      ggaaataaggactgagtacctgctcaattccaggcactagccaaatctgcagcaaactag   76358

chimp.LOC112206744.LOC107973052.GGT2-.646279-708040.rev.compl      ------------------------------------------------------------   61762
FAM230B-LOC105372935-GGT2.NCBI.GGT2.rev.compl                      catttccaaaggtgtcttcttactttaatctcacagccaccctgtgagggaagtgtacat   76418

chimp.LOC112206744.LOC107973052.GGT2-.646279-708040.rev.compl      ------------------------------------------------------------   61762
FAM230B-LOC105372935-GGT2.NCBI.GGT2.rev.compl                      tccccatttcgctgatgagatgagcaaactgaggctcggagaaattgtcagtgggataca   76478

chimp.LOC112206744.LOC107973052.GGT2-.646279-708040.rev.compl      ------------------------------------------------------------   61762
FAM230B-LOC105372935-GGT2.NCBI.GGT2.rev.compl                      gagttggcatccaaacccacaatctctgactctaagcttctgtttcattcattccacaaa   76538

chimp.LOC112206744.LOC107973052.GGT2-.646279-708040.rev.compl      ------------------------------------------------------------   61762
FAM230B-LOC105372935-GGT2.NCBI.GGT2.rev.compl                      tatatatggtgtctcctctttgctggacactgtactgggctgatgagctacaagacccaa   76598

chimp.LOC112206744.LOC107973052.GGT2-.646279-708040.rev.compl      ------------------------------------------------------------   61762
FAM230B-LOC105372935-GGT2.NCBI.GGT2.rev.compl                      tctttacagtctaggaggcagagatagataagtaaacagaggcggcaaagcgtggcactg   76658

chimp.LOC112206744.LOC107973052.GGT2-.646279-708040.rev.compl      ------------------------------------------------------------   61762
FAM230B-LOC105372935-GGT2.NCBI.GGT2.rev.compl                      tgaaagatgcactgaggacaccgagctaggtgttccacaggcgtcaggaaaggcttcaca   76718

chimp.LOC112206744.LOC107973052.GGT2-.646279-708040.rev.compl      ------------------------------------------------------------   61762
FAM230B-LOC105372935-GGT2.NCBI.GGT2.rev.compl                      caagagaggttgcttgtgcccagttctaattaggatgagtaggtatttctgctagatcgt   76778

chimp.LOC112206744.LOC107973052.GGT2-.646279-708040.rev.compl      ------------------------------------------------------------   61762
FAM230B-LOC105372935-GGT2.NCBI.GGT2.rev.compl                      ggtggggtggggaggacatcacataggacctcagcagagagcagctcgaatctatgcaga   76838

chimp.LOC112206744.LOC107973052.GGT2-.646279-708040.rev.compl      ------------------------------------------------------------   61762
FAM230B-LOC105372935-GGT2.NCBI.GGT2.rev.compl                      gctgcacgaggagccggaggtccagagtactgcagcagtttgacattgatgggtggtagg   76898

chimp.LOC112206744.LOC107973052.GGT2-.646279-708040.rev.compl      ------------------------------------------------------------   61762
FAM230B-LOC105372935-GGT2.NCBI.GGT2.rev.compl                      gtccatgaaagtagaggaagaccaagaggaagcagcagacaggggccaggccagggcagg   76958

chimp.LOC112206744.LOC107973052.GGT2-.646279-708040.rev.compl      ------------------------------------------------------------   61762
FAM230B-LOC105372935-GGT2.NCBI.GGT2.rev.compl                      gatagggtgctctactaaagagcatgggctttgaactatgagcagtggagagccactggg   77018

chimp.LOC112206744.LOC107973052.GGT2-.646279-708040.rev.compl      ------------------------------------------------------------   61762
FAM230B-LOC105372935-GGT2.NCBI.GGT2.rev.compl                      ggtgtaaataaggaaacagatattccaaatcctgccttgggaagattgttctggcaggag   77078

chimp.LOC112206744.LOC107973052.GGT2-.646279-708040.rev.compl      ------------------------------------------------------------   61762
FAM230B-LOC105372935-GGT2.NCBI.GGT2.rev.compl                      tgatgggaaaggggccctggcagaagattccttcatgctcctgcggtgatccaggtgaga   77138

chimp.LOC112206744.LOC107973052.GGT2-.646279-708040.rev.compl      ------------------------------------------------------------   61762
FAM230B-LOC105372935-GGT2.NCBI.GGT2.rev.compl                      gctgctgaggaccccaaccaaatcagatcactggacctccactgggagtgaaaggggtg   77198

chimp.LOC112206744.LOC107973052.GGT2-.646279-708040.rev.compl      ------------------------------------------------------------   61762
FAM230B-LOC105372935-GGT2.NCBI.GGT2.rev.compl                      gtctggccagagagatagtaaagagatgtagttaacaggcaagagaatgtggggatatcc   77258

chimp.LOC112206744.LOC107973052.GGT2-.646279-708040.rev.compl      ------------------------------------------------------------   61762
FAM230B-LOC105372935-GGT2.NCBI.GGT2.rev.compl                      agggagctgtccacaattcgcaggtcttaggcaactgcttgggagaagttgatgaggtca   77318

chimp.LOC112206744.LOC107973052.GGT2-.646279-708040.rev.compl      ------------------------------------------------------------   61762
FAM230B-LOC105372935-GGT2.NCBI.GGT2.rev.compl                      tcactggacagagaatctattccctgggcccaaccaactctctagaccatcctgccatgt   77378

chimp.LOC112206744.LOC107973052.GGT2-.646279-708040.rev.compl      ------------------------------------------------------------   61762
FAM230B-LOC105372935-GGT2.NCBI.GGT2.rev.compl                      gcctctgagaagtgagcttccccagccaagcccacaggaggacatatggagcaaaggtca   77438

chimp.LOC112206744.LOC107973052.GGT2-.646279-708040.rev.compl      ------------------------------------------------------------   61762
FAM230B-LOC105372935-GGT2.NCBI.GGT2.rev.compl                      acagtcactcagctgatggatggcaagcattcagatgggaggtgcctccacaggccatga   77498

chimp.LOC112206744.LOC107973052.GGT2-.646279-708040.rev.compl      ------------------------------------------------------------   61762
FAM230B-LOC105372935-GGT2.NCBI.GGT2.rev.compl                      taacccatggatccatttgagaaagatgccaaaaaacagttaattcaaggccactttaaa   77558
```

```
chimp.LOC112206744.LOC107973052.GGT2-.646279-708040.rev.compl       ------------------------------------------------------------   61762
FAM230B-LOC105372935-GGT2.NCBI.GGT2.rev.compl                       atacctggcattcaatcctcctcaatgtctccatcaactgtagaccctataactaccaaa   77618

chimp.LOC112206744.LOC107973052.GGT2-.646279-708040.rev.compl       ------------------------------------------------------------   61762
FAM230B-LOC105372935-GGT2.NCBI.GGT2.rev.compl                       tgagctccactcgccacaaaggtcaagacacagaaacggtcagagagcagagcctattcc   77678

chimp.LOC112206744.LOC107973052.GGT2-.646279-708040.rev.compl       ------------------------------------------------------------   61762
FAM230B-LOC105372935-GGT2.NCBI.GGT2.rev.compl                       tgaaaagcaaaagactccaaactgccacaactcaaccagtgaagggctggggaagactga   77738

chimp.LOC112206744.LOC107973052.GGT2-.646279-708040.rev.compl       ------------------------------------------------------------   61762
FAM230B-LOC105372935-GGT2.NCBI.GGT2.rev.compl                       caggttcactaagaaatgagaggaaggacaaacaaggtgttcaaattagaaaatgattcc   77798

chimp.LOC112206744.LOC107973052.GGT2-.646279-708040.rev.compl       ------------------------------------------------------------   61762
FAM230B-LOC105372935-GGT2.NCBI.GGT2.rev.compl                       attaatgagggctctgatgatatagttgtggaggttatcagtttgccagagctgcagatt   77858

chimp.LOC112206744.LOC107973052.GGT2-.646279-708040.rev.compl       ------------------------------------------------------------   61762
FAM230B-LOC105372935-GGT2.NCBI.GGT2.rev.compl                       taaaatacttacatggtcatcgaaaccaaaaaatgctgaccatcccccaaaacctcccac   77918

chimp.LOC112206744.LOC107973052.GGT2-.646279-708040.rev.compl       ------------------------------------------------------------   61762
FAM230B-LOC105372935-GGT2.NCBI.GGT2.rev.compl                       cccacctagtgctgataacaggagttatcagttgccttttgtgtacagctagatgagata   77978

chimp.LOC112206744.LOC107973052.GGT2-.646279-708040.rev.compl       ------------------------------------------------------------   61762
FAM230B-LOC105372935-GGT2.NCBI.GGT2.rev.compl                       tggatctgtggggggaagaaggggtgggcgtgctagcatctctcagctagactgtcatcg   78038

chimp.LOC112206744.LOC107973052.GGT2-.646279-708040.rev.compl       ------------------------------------------------------------   61762
FAM230B-LOC105372935-GGT2.NCBI.GGT2.rev.compl                       gatggtgacattccccaggactcaagcagcatctgagaagccactttcagtatgactatg   78098

chimp.LOC112206744.LOC107973052.GGT2-.646279-708040.rev.compl       ------------------------------------------------------------   61762
FAM230B-LOC105372935-GGT2.NCBI.GGT2.rev.compl                       gaacagctaaccacacaacacagcatactgaatgaggagcctcgcaggctgagaagaaag   78158

chimp.LOC112206744.LOC107973052.GGT2-.646279-708040.rev.compl       ------------------------------------------------------------   61762
FAM230B-LOC105372935-GGT2.NCBI.GGT2.rev.compl                       caaacccggaaggcaacaaatggatacgtgcatgctgaaggactcgtgtttaattcttcc   78218

chimp.LOC112206744.LOC107973052.GGT2-.646279-708040.rev.compl       ------------------------------------------------------------   61762
FAM230B-LOC105372935-GGT2.NCBI.GGT2.rev.compl                       cggtaatcctttgtcaggcatatatttttgtctcctcaaacttaagaaagaaatccatgc   78278

chimp.LOC112206744.LOC107973052.GGT2-.646279-708040.rev.compl       ------------------------------------------------------------   61762
FAM230B-LOC105372935-GGT2.NCBI.GGT2.rev.compl                       tctggaggggagaaagagaggctgtactcattaactcagagctgacctcgtctggcagga   78338

chimp.LOC112206744.LOC107973052.GGT2-.646279-708040.rev.compl       ------------------------------------------------------------   61762
FAM230B-LOC105372935-GGT2.NCBI.GGT2.rev.compl                       ttaactcgccatacctgtccagcccacagaacctggcctggagcatggagggagaaccac   78398

chimp.LOC112206744.LOC107973052.GGT2-.646279-708040.rev.compl       ------------------------------------------------------------   61762
FAM230B-LOC105372935-GGT2.NCBI.GGT2.rev.compl                       agccaactccagaaaaataaaaggctgcagcgagaaacaagtggggccacagagagaaag   78458

chimp.LOC112206744.LOC107973052.GGT2-.646279-708040.rev.compl       ------------------------------------------------------------   61762
FAM230B-LOC105372935-GGT2.NCBI.GGT2.rev.compl                       gggttttgaaaaagttctaaatccccatggctagggacagtgacatcagtgtgtttacca   78518

chimp.LOC112206744.LOC107973052.GGT2-.646279-708040.rev.compl       ------------------------------------------------------------   61762
FAM230B-LOC105372935-GGT2.NCBI.GGT2.rev.compl                       agtatttgatttacttgctcaggcaaaattccagaagagacactgggacctgtgtggaga   78578

chimp.LOC112206744.LOC107973052.GGT2-.646279-708040.rev.compl       ------------------------------------------------------------   61762
FAM230B-LOC105372935-GGT2.NCBI.GGT2.rev.compl                       gtttcttcactgcagctatcctgccaagctcattgtaaagtgacgaattttgtggacact   78638

chimp.LOC112206744.LOC107973052.GGT2-.646279-708040.rev.compl       ------------------------------------------------------------   61762
FAM230B-LOC105372935-GGT2.NCBI.GGT2.rev.compl                       tacagagaaaaaatagcttcaaatgctatagaccaatgctgcacgctgcagagacacagt   78698

chimp.LOC112206744.LOC107973052.GGT2-.646279-708040.rev.compl       ------------------------------------------------------------   61762
FAM230B-LOC105372935-GGT2.NCBI.GGT2.rev.compl                       ctttgggttctgtgagctgtatttgggcattacgcagagttcagctgccatctgtccctt   78758

chimp.LOC112206744.LOC107973052.GGT2-.646279-708040.rev.compl       ------------------------------------------------------------   61762
FAM230B-LOC105372935-GGT2.NCBI.GGT2.rev.compl                       tgaaggccagctaagaagttggtcccagcctgggcatggtaccactgtcctacattccag   78818

chimp.LOC112206744.LOC107973052.GGT2-.646279-708040.rev.compl       ------------------------------------------------------------   61762
FAM230B-LOC105372935-GGT2.NCBI.GGT2.rev.compl                       ccatttgggatatcctgggttcaagcattccctcccattgctggatcaagcacttcccac   78878

chimp.LOC112206744.LOC107973052.GGT2-.646279-708040.rev.compl       ------------------------------------------------------------   61762
FAM230B-LOC105372935-GGT2.NCBI.GGT2.rev.compl                       ctcctgctctgggaaatcagatctcagtaggtcatcaaccatcccagaaagtccagcaga   78938

chimp.LOC112206744.LOC107973052.GGT2-.646279-708040.rev.compl       ------------------------------------------------------------   61762
FAM230B-LOC105372935-GGT2.NCBI.GGT2.rev.compl                       gttggttttggtttgggggtgttttttgagttgggtggaagaagagaccttacataatct   78998

chimp.LOC112206744.LOC107973052.GGT2-.646279-708040.rev.compl       ------------------------------------------------------------   61762
FAM230B-LOC105372935-GGT2.NCBI.GGT2.rev.compl                       tttaacttttctctaaggctgaattccctataagcaaaattttaaaatttttttttaaag   79058

chimp.LOC112206744.LOC107973052.GGT2-.646279-708040.rev.compl       ------------------------------------------------------------   61762
FAM230B-LOC105372935-GGT2.NCBI.GGT2.rev.compl                       aagaaaaacacccagacaatccttaaaatcccctttcagctccaagactgtgtaattctg   79118

chimp.LOC112206744.LOC107973052.GGT2-.646279-708040.rev.compl       ------------------------------------------------------------   61762
FAM230B-LOC105372935-GGT2.NCBI.GGT2.rev.compl                       tgataccagtattgaaagcccctgcatttccctaactatatattcccctatgccagggaa   79178

chimp.LOC112206744.LOC107973052.GGT2-.646279-708040.rev.compl       ------------------------------------------------------------   61762
FAM230B-LOC105372935-GGT2.NCBI.GGT2.rev.compl                       aggtgcatgtaagtgagagaacaaaaaatttcacaatcgtggggacctcttctttcggct   79238

chimp.LOC112206744.LOC107973052.GGT2-.646279-708040.rev.compl       ------------------------------------------------------------   61762
FAM230B-LOC105372935-GGT2.NCBI.GGT2.rev.compl                       ttggagccccccttccctctgtctctgtacggggcagctttttccttctgtcttctccctt   79298

chimp.LOC112206744.LOC107973052.GGT2-.646279-708040.rev.compl       ------------------------------------------------------------   61762
FAM230B-LOC105372935-GGT2.NCBI.GGT2.rev.compl                       ccttcttgtctactaaactctctgcgccttagaaccaaaaataaaaattcaaaatcatga   79358

chimp.LOC112206744.LOC107973052.GGT2-.646279-708040.rev.compl       ------------------------------------------------------------   61762
FAM230B-LOC105372935-GGT2.NCBI.GGT2.rev.compl                       tcttaggggtgaaagaacactgagaaaccatagaaagcaaaaacgaaaagccaacataga   79418

chimp.LOC112206744.LOC107973052.GGT2-.646279-708040.rev.compl       ------------------------------------------------------------   61762
FAM230B-LOC105372935-GGT2.NCBI.GGT2.rev.compl                       aaaagtctgacttgaattagaagcagcacttaaaattacaggttcagaaaaagcttgaac   79478

chimp.LOC112206744.LOC107973052.GGT2-.646279-708040.rev.compl       ------------------------------------------------------------   61762
FAM230B-LOC105372935-GGT2.NCBI.GGT2.rev.compl                       agaatccagggctgaggtcagccctgagtctgcaggctctgccctctcacaaaggtgagt   79538

chimp.LOC112206744.LOC107973052.GGT2-.646279-708040.rev.compl       ------------------------------------------------------------   61762
FAM230B-LOC105372935-GGT2.NCBI.GGT2.rev.compl                       agatgggtctggagagggctgttcatgctatggaagggcctctatgccatcgaggctaaa   79598

chimp.LOC112206744.LOC107973052.GGT2-.646279-708040.rev.compl       ------------------------------------------------------------   61762
FAM230B-LOC105372935-GGT2.NCBI.GGT2.rev.compl                       accaaagcaggagcaaatcagccctgctagtgtgtacagtcggcatcaaagaggcaacaa   79658

chimp.LOC112206744.LOC107973052.GGT2-.646279-708040.rev.compl       ------------------------------------------------------------   61762
FAM230B-LOC105372935-GGT2.NCBI.GGT2.rev.compl                       cggtagagaatattttctgaaagacacacatgaaaccagtaacggcgatggtctctggaa   79718
```

```
chimp.LOC112206744.LOC107973052.GGT2-.646279-708040.rev.compl      ------------------------------------------------------------   61762
FAM230B-LOC105372935-GGT2.NCBI.GGT2.rev.compl                      gaattaggtcagaagcagacttaatttgcactgtgtacattctttggtatccttttaat   79778

chimp.LOC112206744.LOC107973052.GGT2-.646279-708040.rev.compl      ------------------------------------------------------------   61762
FAM230B-LOC105372935-GGT2.NCBI.GGT2.rev.compl                      tgaacgcatgtgaatgcattacctattcaaaaataaatgtgtaaaaacacatgccacgac   79838

chimp.LOC112206744.LOC107973052.GGT2-.646279-708040.rev.compl      ------------------------------------------------------------   61762
FAM230B-LOC105372935-GGT2.NCBI.GGT2.rev.compl                      aaagcaaaatgaggagacagtataaaaaagacaaggataattttaaatttctcctaataa   79898

chimp.LOC112206744.LOC107973052.GGT2-.646279-708040.rev.compl      ------------------------------------------------------------   61762
FAM230B-LOC105372935-GGT2.NCBI.GGT2.rev.compl                      aacgctgtttaaaaatctaagaggaaaaaagggtaggctccagctagtggagagaacaac   79958

chimp.LOC112206744.LOC107973052.GGT2-.646279-708040.rev.compl      ------------------------------------------------------------   61762
FAM230B-LOC105372935-GGT2.NCBI.GGT2.rev.compl                      tgaaaaacaagagccacgtgttcccatggtcctggccaaccccacttctttcccatcctc   80018

chimp.LOC112206744.LOC107973052.GGT2-.646279-708040.rev.compl      ------------------------------------------------------------   61762
FAM230B-LOC105372935-GGT2.NCBI.GGT2.rev.compl                      actcgttcacatctgccttggagcacccacagcaagcctggcttcatgcccagaacatca   80078

chimp.LOC112206744.LOC107973052.GGT2-.646279-708040.rev.compl      ------------------------------------------------------------   61762
FAM230B-LOC105372935-GGT2.NCBI.GGT2.rev.compl                      cctggctttcctcctctctccctgcccacatctcctcagtcactcgctgcctcctctctt   80138

chimp.LOC112206744.LOC107973052.GGT2-.646279-708040.rev.compl      ------------------------------------------------------------   61762
FAM230B-LOC105372935-GGT2.NCBI.GGT2.rev.compl                      gcttctctcccagttctcagtttgcccatcaaatctcaggtggtactctccattctctgc   80198

chimp.LOC112206744.LOC107973052.GGT2-.646279-708040.rev.compl      ------------------------------------------------------------   61762
FAM230B-LOC105372935-GGT2.NCBI.GGT2.rev.compl                      tatgagggagttcatttgttcatgcaatttccccaccggagacaaaatgtatttcccaaa   80258

chimp.LOC112206744.LOC107973052.GGT2-.646279-708040.rev.compl      ------------------------------------------------------------   61762
FAM230B-LOC105372935-GGT2.NCBI.GGT2.rev.compl                      gttggcttgcaatgacatctcccatgcctgctctgcttacccatgactttgacactcctt   80318

chimp.LOC112206744.LOC107973052.GGT2-.646279-708040.rev.compl      ------------------------------------------------------------   61762
FAM230B-LOC105372935-GGT2.NCBI.GGT2.rev.compl                      ccatcgagagggcagaacctgtgtttcctctctctgaatctaggtgggctacagctctgg   80378

chimp.LOC112206744.LOC107973052.GGT2-.646279-708040.rev.compl      ------------------------------------------------------------   61762
FAM230B-LOC105372935-GGT2.NCBI.GGT2.rev.compl                      tagaagtgacactctgtgaccttaaggctaggtcacaaaaagggaatgcagcttctgcca   80438

chimp.LOC112206744.LOC107973052.GGT2-.646279-708040.rev.compl      ------------------------------------------------------------   61762
FAM230B-LOC105372935-GGT2.NCBI.GGT2.rev.compl                      gcaatcctgggacacacacttggagcccgagctgctgtgtaagccatctgcctgctctga   80498

chimp.LOC112206744.LOC107973052.GGT2-.646279-708040.rev.compl      ------------------------------------------------------------   61762
FAM230B-LOC105372935-GGT2.NCBI.GGT2.rev.compl                      gcccaccacactataaggaagcccaagctagcccacaggagagacgaggtgaagagaatg   80558

chimp.LOC112206744.LOC107973052.GGT2-.646279-708040.rev.compl      ------------------------------------------------------------   61762
FAM230B-LOC105372935-GGT2.NCBI.GGT2.rev.compl                      agatgccagtctccactgctccagctctctagtgtcccagccccagccactatctgactg   80618

chimp.LOC112206744.LOC107973052.GGT2-.646279-708040.rev.compl      ------------------------------------------------------------   61762
FAM230B-LOC105372935-GGT2.NCBI.GGT2.rev.compl                      caaccccatgaaagactgagccagaaccacgagcccagcccttcctgatgttgacataca   80678

chimp.LOC112206744.LOC107973052.GGT2-.646279-708040.rev.compl      ------------------------------------------------------------   61762
FAM230B-LOC105372935-GGT2.NCBI.GGT2.rev.compl                      gacttcatgaaagagaataaactaattttgctttaagccactatgatttgggatgattt   80738

chimp.LOC112206744.LOC107973052.GGT2-.646279-708040.rev.compl      ------------------------------------------------------------   61762
FAM230B-LOC105372935-GGT2.NCBI.GGT2.rev.compl                      gttatgcagcactggataactgcaacacctgcttctaaacagtagaatcccaaacgtcta   80798

chimp.LOC112206744.LOC107973052.GGT2-.646279-708040.rev.compl      ------------------------------------------------------------   61762
FAM230B-LOC105372935-GGT2.NCBI.GGT2.rev.compl                      ggccccatctctaacttacttcatgttcctgttctcactgatttttgccactgagcttg   80858

chimp.LOC112206744.LOC107973052.GGT2-.646279-708040.rev.compl      ------------------------------------------------------------   61762
FAM230B-LOC105372935-GGT2.NCBI.GGT2.rev.compl                      accaagcccttgagtcaccaattccagatatttgtttaaacaaattcactgtgactgtct   80918

chimp.LOC112206744.LOC107973052.GGT2-.646279-708040.rev.compl      ------------------------------------------------------------   61762
FAM230B-LOC105372935-GGT2.NCBI.GGT2.rev.compl                      acactatccattcctccaaaacaatccctgttatagttggccttccccagttctcttcct   80978

chimp.LOC112206744.LOC107973052.GGT2-.646279-708040.rev.compl      ------------------------------------------------------------   61762
FAM230B-LOC105372935-GGT2.NCBI.GGT2.rev.compl                      ggcatagagtagtctgtgcccttctctcatttcttaatatgaccctcacccatggagtgt   81038

chimp.LOC112206744.LOC107973052.GGT2-.646279-708040.rev.compl      ------------------------------------------------------------   61762
FAM230B-LOC105372935-GGT2.NCBI.GGT2.rev.compl                      tcctagattctgctcattcataatgcttcttcacccaccctaacctctccctcctgtagc   81098

chimp.LOC112206744.LOC107973052.GGT2-.646279-708040.rev.compl      ------------------------------------------------------------   61762
FAM230B-LOC105372935-GGT2.NCBI.GGT2.rev.compl                      cacagatcatccacttttccaggcctctgcactggaggctgcatcagccacctcccagta   81158

chimp.LOC112206744.LOC107973052.GGT2-.646279-708040.rev.compl      ------------------------------------------------------------   61762
FAM230B-LOC105372935-GGT2.NCBI.GGT2.rev.compl                      cgtcatcctttccaatgccctacaacacggggcagaaaggactctttttgctgcacatc   81218

chimp.LOC112206744.LOC107973052.GGT2-.646279-708040.rev.compl      ------------------------------------------------------------   61762
FAM230B-LOC105372935-GGT2.NCBI.GGT2.rev.compl                      caggctgtttaagaaaaggcttaaggcagcttaagcacaaagaatgtcatcttcaaacat   81278

chimp.LOC112206744.LOC107973052.GGT2-.646279-708040.rev.compl      ------------------------------------------------------------   61762
FAM230B-LOC105372935-GGT2.NCBI.GGT2.rev.compl                      tgtaacggcatcttctttttggggcactgaagtattcccatgattccaagaatcaaatga   81338

chimp.LOC112206744.LOC107973052.GGT2-.646279-708040.rev.compl      ------------------------------------------------------------   61762
FAM230B-LOC105372935-GGT2.NCBI.GGT2.rev.compl                      acagcatctggttatggggtctctggtctctgccaaggcaaactcataaagcatttcaga   81398

chimp.LOC112206744.LOC107973052.GGT2-.646279-708040.rev.compl      ------------------------------------------------------------   61762
FAM230B-LOC105372935-GGT2.NCBI.GGT2.rev.compl                      gcccttcatctggaaaggccttttctccaccccacgcccagccaatgcccactcatactt   81458

chimp.LOC112206744.LOC107973052.GGT2-.646279-708040.rev.compl      ------------------------------------------------------------   61762
FAM230B-LOC105372935-GGT2.NCBI.GGT2.rev.compl                      taaggcctagctcatccgtcatctctgctagggaaccttcctcagtgccttatgcaggca   81518

chimp.LOC112206744.LOC107973052.GGT2-.646279-708040.rev.compl      ------------------------------------------------------------   61762
FAM230B-LOC105372935-GGT2.NCBI.GGT2.rev.compl                      accattcctgccttgtgttgtgttgtgttgtgttgtgccttaagtcaaatccttaactca   81578

chimp.LOC112206744.LOC107973052.GGT2-.646279-708040.rev.compl      ------------------------------------------------------------   61762
FAM230B-LOC105372935-GGT2.NCBI.GGT2.rev.compl                      tctttgtgtgtccccttgcatttcagacagtaggtgcccaataaatgtttgtgggatgaa   81638

chimp.LOC112206744.LOC107973052.GGT2-.646279-708040.rev.compl      ------------------------------------------------------------   61762
FAM230B-LOC105372935-GGT2.NCBI.GGT2.rev.compl                      tcaactggttattctcctggaacaccctttcccagtacacctccactcgccagaacaact   81698

chimp.LOC112206744.LOC107973052.GGT2-.646279-708040.rev.compl      ------------------------------------------------------------   61762
FAM230B-LOC105372935-GGT2.NCBI.GGT2.rev.compl                      gtcttcctcacaggtgccagccatcaccccgaccacaccctctcaaaaggcctttgccag   81758

chimp.LOC112206744.LOC107973052.GGT2-.646279-708040.rev.compl      ------------------------------------------------------------   61762
FAM230B-LOC105372935-GGT2.NCBI.GGT2.rev.compl                      tcatcaaacaaagcctcttgtctcctgagagccgctccacaatcaccttcccaagaagaa   81818

chimp.LOC112206744.LOC107973052.GGT2-.646279-708040.rev.compl      ------------------------------------------------------------   61762
FAM230B-LOC105372935-GGT2.NCBI.GGT2.rev.compl                      ttccccataccgagtccaccattccttccctgatcaaagttgctttcacaggcatctttt   81878

chimp.LOC112206744.LOC107973052.GGT2-.646279-708040.rev.compl      ------------------------------------------------------------   61762
```

```
FAM230B-LOC105372935-GGT2.NCBI.GGT2.rev.compl            cagctatccactcttcacggcttggttttcatgcactctgagttggccctatctcccctt   81938

chimp.LOC112206744.LOC107973052.GGT2-.646279-708040.rev.compl  ------------------------------------------------------------   61762
FAM230B-LOC105372935-GGT2.NCBI.GGT2.rev.compl            tttggcctcaagcacgtcaagaggaaggctggtgacagcctttcccgagctcaggacagt   81998

chimp.LOC112206744.LOC107973052.GGT2-.646279-708040.rev.compl  ------------------------------------------------------------   61762
FAM230B-LOC105372935-GGT2.NCBI.GGT2.rev.compl            tttcatgcaaactggttcttccaggttcttatgcctggaagaactgctgctctccaatag   82058

chimp.LOC112206744.LOC107973052.GGT2-.646279-708040.rev.compl  ------------------------------------------------------------   61762
FAM230B-LOC105372935-GGT2.NCBI.GGT2.rev.compl            catgaagtagacaatgatgttcttagcatccagtcttaaaactagaaaaagtattggcac   82118

chimp.LOC112206744.LOC107973052.GGT2-.646279-708040.rev.compl  ------------------------------------------------------------   61762
FAM230B-LOC105372935-GGT2.NCBI.GGT2.rev.compl            tgctggatccccagctcctagaatggtgtgtggcacctaaacagagctcaaaaaatatta   82178

chimp.LOC112206744.LOC107973052.GGT2-.646279-708040.rev.compl  ------------------------------------------------------------   61762
FAM230B-LOC105372935-GGT2.NCBI.GGT2.rev.compl            gttgaatggaggaaagaatgaacagtcatctggcccagacttggagaagctgtgatctgt   82238

chimp.LOC112206744.LOC107973052.GGT2-.646279-708040.rev.compl  ------------------------------------------------------------   61762
FAM230B-LOC105372935-GGT2.NCBI.GGT2.rev.compl            ctagcccttctacaccaggagcgaggcctgaaaagctgcatgactcacccaaggtcacaa   82298

chimp.LOC112206744.LOC107973052.GGT2-.646279-708040.rev.compl  ------------------------------------------------------------   61762
FAM230B-LOC105372935-GGT2.NCBI.GGT2.rev.compl            gcaagccagtgaagaggggctggaaccctgtcttttgcctcaaagcacagggccctcctt   82358

chimp.LOC112206744.LOC107973052.GGT2-.646279-708040.rev.compl  ------------------------------------------------------------   61762
FAM230B-LOC105372935-GGT2.NCBI.GGT2.rev.compl            ctgtcccacccagctttctcaccacccaccccaccccttccacactccaaagacagcatc   82418

chimp.LOC112206744.LOC107973052.GGT2-.646279-708040.rev.compl  ------------------------------------------------------------   61762
FAM230B-LOC105372935-GGT2.NCBI.GGT2.rev.compl            tccgtgagaagtgttgggcagtgtccaggagtgtttttcttcctggctttaaaacaaatt   82478

chimp.LOC112206744.LOC107973052.GGT2-.646279-708040.rev.compl  ------------------------------------------------------------   61762
FAM230B-LOC105372935-GGT2.NCBI.GGT2.rev.compl            gtgacaaatggctccacagatctaccgggccatggagctgagcagatgggactgcaattc   82538

chimp.LOC112206744.LOC107973052.GGT2-.646279-708040.rev.compl  ------------------------------------------------------------   61762
FAM230B-LOC105372935-GGT2.NCBI.GGT2.rev.compl            acgctattaactcaaattctacttgactttctttcaaatcagggcattttatctagaaat   82598

chimp.LOC112206744.LOC107973052.GGT2-.646279-708040.rev.compl  ------------------------------------------------------------   61762
FAM230B-LOC105372935-GGT2.NCBI.GGT2.rev.compl            tataaaaaattgttgggggagtgcctttgtaaggaagtttgccagcacattcatttacaa   82658

chimp.LOC112206744.LOC107973052.GGT2-.646279-708040.rev.compl  ------------------------------------------------------------   61762
FAM230B-LOC105372935-GGT2.NCBI.GGT2.rev.compl            atcgacttgctcctggccaccctcactgatcacggacttttactcacgctcccttactct   82718

chimp.LOC112206744.LOC107973052.GGT2-.646279-708040.rev.compl  ------------------------------------------------------------   61762
FAM230B-LOC105372935-GGT2.NCBI.GGT2.rev.compl            ctcccatcaaagagtccagcgcatggagagagtgatggaatcttgaggcccaccggtcta   82778

chimp.LOC112206744.LOC107973052.GGT2-.646279-708040.rev.compl  ------------------------------------------------------------   61762
FAM230B-LOC105372935-GGT2.NCBI.GGT2.rev.compl            accacccacctaagcttgactcccctgtgcctgtggacatcctgcgtgcagatgcacatt   82838

chimp.LOC112206744.LOC107973052.GGT2-.646279-708040.rev.compl  ------------------------------------------------------------   61762
FAM230B-LOC105372935-GGT2.NCBI.GGT2.rev.compl            cccactaatggcagccaggggaaagcactattcgaaagttcttctttactttgaactcaa   82898

chimp.LOC112206744.LOC107973052.GGT2-.646279-708040.rev.compl  ------------------------------------------------------------   61762
FAM230B-LOC105372935-GGT2.NCBI.GGT2.rev.compl            attgggctcaggggtcctggatacaggacaaccccaatctcttatttattcaatggacaa   82958

chimp.LOC112206744.LOC107973052.GGT2-.646279-708040.rev.compl  ------------------------------------------------------------   61762
FAM230B-LOC105372935-GGT2.NCBI.GGT2.rev.compl            caggtgagcacatactaggcctggacactgatgtaggtaggcactaaagcttcagagaga   83018

chimp.LOC112206744.LOC107973052.GGT2-.646279-708040.rev.compl  ------------------------------------------------------------   61762
FAM230B-LOC105372935-GGT2.NCBI.GGT2.rev.compl            tgcaaatgaacaatctttaccctgaaggagctcatgttctatgggttcatttcctttccc   83078

chimp.LOC112206744.LOC107973052.GGT2-.646279-708040.rev.compl  ------------------------------------------------------------   61762
FAM230B-LOC105372935-GGT2.NCBI.GGT2.rev.compl            tgtgtcttacaaacaggttccagtttgtttccgtccttcagagtgtactgcctagaactg   83138

chimp.LOC112206744.LOC107973052.GGT2-.646279-708040.rev.compl  ------------------------------------------------------------   61762
FAM230B-LOC105372935-GGT2.NCBI.GGT2.rev.compl            catgctatactccacaagtggcctgaccagcttgtggacaaggatgaagcaagcagacca   83198

chimp.LOC112206744.LOC107973052.GGT2-.646279-708040.rev.compl  ------------------------------------------------------------   61762
FAM230B-LOC105372935-GGT2.NCBI.GGT2.rev.compl            ccttctttcagtgcagacaagatggctgtttagaggccacatcacgtggcagacacctgc   83258

chimp.LOC112206744.LOC107973052.GGT2-.646279-708040.rev.compl  ------------------------------------------------------------   61762
FAM230B-LOC105372935-GGT2.NCBI.GGT2.rev.compl            caactaaaccctccatccttcccacagcctcccctcctacactttgcagctggattca   83318

chimp.LOC112206744.LOC107973052.GGT2-.646279-708040.rev.compl  ------------------------------------------------------------   61762
FAM230B-LOC105372935-GGT2.NCBI.GGT2.rev.compl            aaaccttgcattcacccttgttccatcaacagggccaggccatcagtctatctcatccgg   83378

chimp.LOC112206744.LOC107973052.GGT2-.646279-708040.rev.compl  ------------------------------------------------------------   61762
FAM230B-LOC105372935-GGT2.NCBI.GGT2.rev.compl            actgtttttatcttacttgccaacctacacacttgctgttccttcttgccttaaagtca   83438

chimp.LOC112206744.LOC107973052.GGT2-.646279-708040.rev.compl  ------------------------------------------------------------   61762
FAM230B-LOC105372935-GGT2.NCBI.GGT2.rev.compl            tacagatgactttaggtgattcagatgagtgtgacctctactttcaccaaacacaatagt   83498

chimp.LOC112206744.LOC107973052.GGT2-.646279-708040.rev.compl  ------------------------------------------------------------   61762
FAM230B-LOC105372935-GGT2.NCBI.GGT2.rev.compl            caattcagaaaggtcaaggacagaaccctccagcatgctaacgcagccaaagaagtccga   83558

chimp.LOC112206744.LOC107973052.GGT2-.646279-708040.rev.compl  ------------------------------------------------------------   61762
FAM230B-LOC105372935-GGT2.NCBI.GGT2.rev.compl            aaacaatacttggccacactgttttcctgcttacggatgtggactgctcttgtcctgcct   83618

chimp.LOC112206744.LOC107973052.GGT2-.646279-708040.rev.compl  ------------------------------------------------------------   61762
FAM230B-LOC105372935-GGT2.NCBI.GGT2.rev.compl            gtaaatatatcggatcgctctccttcttgctggcatccacagtaactatactcaataaac   83678

chimp.LOC112206744.LOC107973052.GGT2-.646279-708040.rev.compl  ------------------------------------------------------------   61762
FAM230B-LOC105372935-GGT2.NCBI.GGT2.rev.compl            acaccaactttgatgccaaatcctaggccatcccacacatcttaaaaataaaacaaaaat   83738

chimp.LOC112206744.LOC107973052.GGT2-.646279-708040.rev.compl  ------------------------------------------------------------   61762
FAM230B-LOC105372935-GGT2.NCBI.GGT2.rev.compl            atgctattccagctaccctcactctttttgtggtttcctgcagtccaactagctacaaat   83798

chimp.LOC112206744.LOC107973052.GGT2-.646279-708040.rev.compl  ------------------------------------------------------------   61762
FAM230B-LOC105372935-GGT2.NCBI.GGT2.rev.compl            ctcaaagccaaagattgtagctccctctcttttgttctttcatcaagccagggagtcctg   83858

chimp.LOC112206744.LOC107973052.GGT2-.646279-708040.rev.compl  ------------------------------------------------------------   61762
FAM230B-LOC105372935-GGT2.NCBI.GGT2.rev.compl            ctcctctctctctagtctctcctgtgtcagtgccacttgtcaactgcctaatccagccac   83918

chimp.LOC112206744.LOC107973052.GGT2-.646279-708040.rev.compl  ------------------------------------------------------------   61762
FAM230B-LOC105372935-GGT2.NCBI.GGT2.rev.compl            gctgctcttcaatattaaaggtcttcagcatattttaaagttagtctcctactgccactc   83978

chimp.LOC112206744.LOC107973052.GGT2-.646279-708040.rev.compl  ------------------------------------------------------------   61762
FAM230B-LOC105372935-GGT2.NCBI.GGT2.rev.compl            ttttccctactcttgccagtccactcagtaaactactgccatacaggtctcccacagcag   84038

chimp.LOC112206744.LOC107973052.GGT2-.646279-708040.rev.compl  ------------------------------------------------------------   61762
FAM230B-LOC105372935-GGT2.NCBI.GGT2.rev.compl            tttctcaccagataaacaatggccggagattccactttgccaatcacatcaagtccaaac   84098
```

```
chimp.LOC112206744.LOC107973052.GGT2-.646279-708040.rev.compl      ------------------------------------------------------------   61762
FAM230B-LOC105372935-GGT2.NCBI.GGT2.rev.compl                      tccgtgtcttggtatccaaggcttctaaagtctggccccactccacctactcactctgac   84158

chimp.LOC112206744.LOC107973052.GGT2-.646279-708040.rev.compl      ------------------------------------------------------------   61762
FAM230B-LOC105372935-GGT2.NCBI.GGT2.rev.compl                      acccaccactccctccaaacatccccggtacgtcttactggattttgccatttcctccaa   84218

chimp.LOC112206744.LOC107973052.GGT2-.646279-708040.rev.compl      ------------------------------------------------------------   61762
FAM230B-LOC105372935-GGT2.NCBI.GGT2.rev.compl                      gttgaccaggagtcattcacacctaaaggctatgcttacatcgttttccatctcttgtgt   84278

chimp.LOC112206744.LOC107973052.GGT2-.646279-708040.rev.compl      ------------------------------------------------------------   61762
FAM230B-LOC105372935-GGT2.NCBI.GGT2.rev.compl                      tggtgagctaaaaacaagatggaaaagaaaaaagaagaggaagagagaaaaagagaaaag   84338

chimp.LOC112206744.LOC107973052.GGT2-.646279-708040.rev.compl      ------------------------------------------------------------   61762
FAM230B-LOC105372935-GGT2.NCBI.GGT2.rev.compl                      gaagaaatggggaaaacagcaataggaccagctaactcctacagcagagtcttgccaggc   84398

chimp.LOC112206744.LOC107973052.GGT2-.646279-708040.rev.compl      ------------------------------------------------------------   61762
FAM230B-LOC105372935-GGT2.NCBI.GGT2.rev.compl                      cctcccctaattgctttatatgtgttacaatgtttaaccctcacaactacaacaggagtt   84458

chimp.LOC112206744.LOC107973052.GGT2-.646279-708040.rev.compl      ------------------------------------------------------------   61762
FAM230B-LOC105372935-GGT2.NCBI.GGT2.rev.compl                      gggtaccattccatccccacctgcagatgagtgtggcaccccaagattaagtatctgccc   84518

chimp.LOC112206744.LOC107973052.GGT2-.646279-708040.rev.compl      ------------------------------------------------------------   61762
FAM230B-LOC105372935-GGT2.NCBI.GGT2.rev.compl                      agggtcacgtgaaaagtctgcatgcatgctgaaccattccagctgcaccactgcccagga   84578

chimp.LOC112206744.LOC107973052.GGT2-.646279-708040.rev.compl      ------------------------------------------------------------   61762
FAM230B-LOC105372935-GGT2.NCBI.GGT2.rev.compl                      aaaaagtcacaggtaggaagacaagcatagaattaagaacaaagcaactaactgtaccag   84638

chimp.LOC112206744.LOC107973052.GGT2-.646279-708040.rev.compl      ------------------------------------------------------------   61762
FAM230B-LOC105372935-GGT2.NCBI.GGT2.rev.compl                      gtgctacagaagggtggtcagcctggaaaatcaggaggtgcattccttgggagattgccg   84698

chimp.LOC112206744.LOC107973052.GGT2-.646279-708040.rev.compl      ------------------------------------------------------------   61762
FAM230B-LOC105372935-GGT2.NCBI.GGT2.rev.compl                      caatcagagtctgggagtatgcaggagactggcaggcagcccagggacaggtgaggcaca   84758

chimp.LOC112206744.LOC107973052.GGT2-.646279-708040.rev.compl      ------------------------------------------------------------   61762
FAM230B-LOC105372935-GGT2.NCBI.GGT2.rev.compl                      ggcactcattcccttccatgtgagaagtcagggcacgtgcaaagccaaggccagaggaca   84818

chimp.LOC112206744.LOC107973052.GGT2-.646279-708040.rev.compl      ------------------------------------------------------------   61762
FAM230B-LOC105372935-GGT2.NCBI.GGT2.rev.compl                      aaggtgagctcgccaatagggccaatactgggtctggctggaaggggagggtggacagag   84878

chimp.LOC112206744.LOC107973052.GGT2-.646279-708040.rev.compl      ------------------------------------------------------------   61762
FAM230B-LOC105372935-GGT2.NCBI.GGT2.rev.compl                      atgggtactaagaagtgaagatggacaggcaggaccaggtcttaaagggtttgtgtcttg   84938

chimp.LOC112206744.LOC107973052.GGT2-.646279-708040.rev.compl      ------------------------------------------------------------   61762
FAM230B-LOC105372935-GGT2.NCBI.GGT2.rev.compl                      ctaaagatggacccttaattccgtgacacatctattcaaccactcctcattatctaccta   84998

chimp.LOC112206744.LOC107973052.GGT2-.646279-708040.rev.compl      ------------------------------------------------------------   61762
FAM230B-LOC105372935-GGT2.NCBI.GGT2.rev.compl                      aattctactgtgcctttgcaaagcccagctcaagtccttttcctctcaggcctgtcctc   85058

chimp.LOC112206744.LOC107973052.GGT2-.646279-708040.rev.compl      ------------------------------------------------------------   61762
FAM230B-LOC105372935-GGT2.NCBI.GGT2.rev.compl                      cctactcctgagcaaaccacgggtgtggagacaggtcccaaccacctgcagatgacagaa   85118

chimp.LOC112206744.LOC107973052.GGT2-.646279-708040.rev.compl      ------------------------------------------------------------   61762
FAM230B-LOC105372935-GGT2.NCBI.GGT2.rev.compl                      ggaagctgcatctaggatcggtctacacagaggcaagaagggatcctcctcctctgcctt   85178

chimp.LOC112206744.LOC107973052.GGT2-.646279-708040.rev.compl      ------------------------------------------------------------   61762
FAM230B-LOC105372935-GGT2.NCBI.GGT2.rev.compl                      ctcctttcatgctgttcttactttctctcaaaaagaaagaaaatacagtgtcattgcttc   85238

chimp.LOC112206744.LOC107973052.GGT2-.646279-708040.rev.compl      ------------------------------------------------------------   61762
FAM230B-LOC105372935-GGT2.NCBI.GGT2.rev.compl                      ttccctaagcttgttaagtcaaagcaagtcaaagcatcagagcttcattacttggctttt   85298

chimp.LOC112206744.LOC107973052.GGT2-.646279-708040.rev.compl      ------------------------------------------------------------   61762
FAM230B-LOC105372935-GGT2.NCBI.GGT2.rev.compl                      tctagctataagcgtctggaaaatatcaggagtcactggcctctaggaatccattaagtt   85358

chimp.LOC112206744.LOC107973052.GGT2-.646279-708040.rev.compl      ------------------------------------------------------------   61762
FAM230B-LOC105372935-GGT2.NCBI.GGT2.rev.compl                      aaaggagctccatgatctactatcataagatgacaaagaaaatatgcactttctttggga   85418

chimp.LOC112206744.LOC107973052.GGT2-.646279-708040.rev.compl      ------------------------------------------------------------   61762
FAM230B-LOC105372935-GGT2.NCBI.GGT2.rev.compl                      ggtcaagttgggcgaatcacttgaggtcaggagttcaagaccaatctggccaacatggtg   85478

chimp.LOC112206744.LOC107973052.GGT2-.646279-708040.rev.compl      ------------------------------------------------------------   61762
FAM230B-LOC105372935-GGT2.NCBI.GGT2.rev.compl                      aaaccccgtctctactgaaaacacaaaaattagctgggcttggtggcaggcacctgtaat   85538

chimp.LOC112206744.LOC107973052.GGT2-.646279-708040.rev.compl      ------------------------------------------------------------   61762
FAM230B-LOC105372935-GGT2.NCBI.GGT2.rev.compl                      cccagctactcaggagactgaggcaggagaatcgattgaacccttacccaggagacagag   85598

chimp.LOC112206744.LOC107973052.GGT2-.646279-708040.rev.compl      ------------------------------------------------------------   61762
FAM230B-LOC105372935-GGT2.NCBI.GGT2.rev.compl                      gttgcaatgagccgagatggcaccacggcactccagcctgagcaacagcgtgactccatc   85658

chimp.LOC112206744.LOC107973052.GGT2-.646279-708040.rev.compl      ------------------------------------------------------------   61762
FAM230B-LOC105372935-GGT2.NCBI.GGT2.rev.compl                      tcaaaaaaaaaaaaaagaaagaaagaaaggaaggaaggaagaaagaagaaaatatgcatt   85718

chimp.LOC112206744.LOC107973052.GGT2-.646279-708040.rev.compl      ------------------------------------------------------------   61762
FAM230B-LOC105372935-GGT2.NCBI.GGT2.rev.compl                      ttctcaggattaaataacaaaaaatgccaagtttcttagctccagtcaagaaaatgacat   85778

chimp.LOC112206744.LOC107973052.GGT2-.646279-708040.rev.compl      ------------------------------------------------------------   61762
FAM230B-LOC105372935-GGT2.NCBI.GGT2.rev.compl                      catgtgacaacagcagccattgcaagtttatcagaagatgaacaattacagatttttcat   85838

chimp.LOC112206744.LOC107973052.GGT2-.646279-708040.rev.compl      ------------------------------------------------------------   61762
FAM230B-LOC105372935-GGT2.NCBI.GGT2.rev.compl                      ttctgaataaggaaccatttacacagacacatgtataacaaatgtctgtgcacatccagg   85898

chimp.LOC112206744.LOC107973052.GGT2-.646279-708040.rev.compl      ------------------------------------------------------------   61762
FAM230B-LOC105372935-GGT2.NCBI.GGT2.rev.compl                      acttgtcaaatcttaacattttgccacacctggtttagtttttttatttttcctttcaag   85958

chimp.LOC112206744.LOC107973052.GGT2-.646279-708040.rev.compl      ------------------------------------------------------------   61762
FAM230B-LOC105372935-GGT2.NCBI.GGT2.rev.compl                      aaagaaagcattacaggctgtgcttcaatccctatttcacccccctttcaagccccactat   86018

chimp.LOC112206744.LOC107973052.GGT2-.646279-708040.rev.compl      ------------------------------------------------------------   61762
FAM230B-LOC105372935-GGT2.NCBI.GGT2.rev.compl                      tccacccatcccagaaatgacaacatcccgaatttggtatttaccactcttgcacattta   86078

chimp.LOC112206744.LOC107973052.GGT2-.646279-708040.rev.compl      ------------------------------------------------------------   61762
FAM230B-LOC105372935-GGT2.NCBI.GGT2.rev.compl                      tttttatgcttttactacctatgtatgtagccataaacaatatacagagtttttggatgc   86138

chimp.LOC112206744.LOC107973052.GGT2-.646279-708040.rev.compl      ------------------------------------------------------------   61762
FAM230B-LOC105372935-GGT2.NCBI.GGT2.rev.compl                      tataaaactgtttaaaatggcatcacactgtatgcaccctttttgcatctgttttaattga   86198

chimp.LOC112206744.LOC107973052.GGT2-.646279-708040.rev.compl      ------------------------------------------------------------   61762
FAM230B-LOC105372935-GGT2.NCBI.GGT2.rev.compl                      caaaacatttttgagggtaatccttgttgacatatgtcactccagggcattcctttcaac   86258
```

```
chimp.LOC112206744.LOC107973052.GGT2-.646279-708040.rev.compl      ------------------------------------------------------------   61762
FAM230B-LOC105372935-GGT2.NCBI.GGT2.rev.compl                      tgctttataccatggtccactgtctggataatcattatctatagcacaatgtattcattt   86318

chimp.LOC112206744.LOC107973052.GGT2-.646279-708040.rev.compl      ------------------------------------------------------------   61762
FAM230B-LOC105372935-GGT2.NCBI.GGT2.rev.compl                      tcctactgataaatttttaagtggtttccaacttttcattgcaataaatattcttgtaaa   86378

chimp.LOC112206744.LOC107973052.GGT2-.646279-708040.rev.compl      ------------------------------------------------------------   61762
FAM230B-LOC105372935-GGT2.NCBI.GGT2.rev.compl                      tcttcgtccaaggttatcttcaaccacactaggcattgcccaaattgctctgcaacatca   86438

chimp.LOC112206744.LOC107973052.GGT2-.646279-708040.rev.compl      ------------------------------------------------------------   61762
FAM230B-LOC105372935-GGT2.NCBI.GGT2.rev.compl                      gtgcaccaatttacattgccaagatcagtgtttaagtgtttctattctttgatgtctttg   86498

chimp.LOC112206744.LOC107973052.GGT2-.646279-708040.rev.compl      ------------------------------------------------------------   61762
FAM230B-LOC105372935-GGT2.NCBI.GGT2.rev.compl                      tcaaccttggtactatttgactactacacttttcaccaatcagatataaggatgaaatgg   86558

chimp.LOC112206744.LOC107973052.GGT2-.646279-708040.rev.compl      ------------------------------------------------------------   61762
FAM230B-LOC105372935-GGT2.NCBI.GGT2.rev.compl                      tgccttcttggtattctgaaaattaattttaaatgggaaaataaattactaataagtgca   86618

chimp.LOC112206744.LOC107973052.GGT2-.646279-708040.rev.compl      ------------------------------------------------------------   61762
FAM230B-LOC105372935-GGT2.NCBI.GGT2.rev.compl                      gcttaatcaacaagagttttcattaacatgtgggaatacaccaggtgttccttggatttt   86678

chimp.LOC112206744.LOC107973052.GGT2-.646279-708040.rev.compl      ------------------------------------------------------------   61762
FAM230B-LOC105372935-GGT2.NCBI.GGT2.rev.compl                      cttgaaaattctacagggagggtacgggagtgacggtacagataagattggacatgagct   86738

chimp.LOC112206744.LOC107973052.GGT2-.646279-708040.rev.compl      ------------------------------------------------------------   61762
FAM230B-LOC105372935-GGT2.NCBI.GGT2.rev.compl                      gataattgttggaactaggtgatgagtgtatgagggttaattattctgctctctccactt   86798

chimp.LOC112206744.LOC107973052.GGT2-.646279-708040.rev.compl      ------------------------------------------------------------   61762
FAM230B-LOC105372935-GGT2.NCBI.GGT2.rev.compl                      tggtacatatttgaaatttttcacaatagaaattaaaagaagaacggggcgtggtggctc   86858

chimp.LOC112206744.LOC107973052.GGT2-.646279-708040.rev.compl      ------------------------------------------------------------   61762
FAM230B-LOC105372935-GGT2.NCBI.GGT2.rev.compl                      acgcctgtaattccagcactttggaaggccatggcagaggatcacttgaggtcaggaatt   86918

chimp.LOC112206744.LOC107973052.GGT2-.646279-708040.rev.compl      ------------------------------------------------------------   61762
FAM230B-LOC105372935-GGT2.NCBI.GGT2.rev.compl                      aaagaccagctggtcaacatggggaaaccccacctctactaaaaatacaaaaattagctg   86978

chimp.LOC112206744.LOC107973052.GGT2-.646279-708040.rev.compl      ------------------------------------------------------------   61762
FAM230B-LOC105372935-GGT2.NCBI.GGT2.rev.compl                      ggtatggtggtgtgtaatcccagctattgaggaggctgaggcacgagaatcgcttggacc   87038

chimp.LOC112206744.LOC107973052.GGT2-.646279-708040.rev.compl      ------------------------------------------------------------   61762
FAM230B-LOC105372935-GGT2.NCBI.GGT2.rev.compl                      caggaggcagaggttgcagtgagccaagatcgtgccactgcactctagcctgggcaacag   87098

chimp.LOC112206744.LOC107973052.GGT2-.646279-708040.rev.compl      ------------------------------------------------------------   61762
FAM230B-LOC105372935-GGT2.NCBI.GGT2.rev.compl                      ggcaagcctctgtctcaaaaaaaaaaaaaaaaaaaaaaagaagaagaagaagaaagaaaa   87158

chimp.LOC112206744.LOC107973052.GGT2-.646279-708040.rev.compl      ------------------------------------------------------------   61762
FAM230B-LOC105372935-GGT2.NCBI.GGT2.rev.compl                      caaagaagaaagaaagaaagaaaaagaaagaaagaaagaaaattaaaagaaggaaatgcc   87218

chimp.LOC112206744.LOC107973052.GGT2-.646279-708040.rev.compl      ------------------------------------------------------------   61762
FAM230B-LOC105372935-GGT2.NCBI.GGT2.rev.compl                      ataaagcatctggagcaaagaacatccaaagaagagcactgatcactgcagcagacagcc   87278

chimp.LOC112206744.LOC107973052.GGT2-.646279-708040.rev.compl      ------------------------------------------------------------   61762
FAM230B-LOC105372935-GGT2.NCBI.GGT2.rev.compl                      attcagaatgggcagagagcagagctggcaggaagagaggacaaaaggaagaccaggaag   87338

chimp.LOC112206744.LOC107973052.GGT2-.646279-708040.rev.compl      ------------------------------------------------------------   61762
FAM230B-LOC105372935-GGT2.NCBI.GGT2.rev.compl                      ggaagccaggaaaagagattttgcattgtgttattatttcaatcagaagacttaggaaca   87398

chimp.LOC112206744.LOC107973052.GGT2-.646279-708040.rev.compl      ------------------------------------------------------------   61762
FAM230B-LOC105372935-GGT2.NCBI.GGT2.rev.compl                      ttatttaattatcaaatttgtgtgtctgatgatcctttacaacactgcagcatcaaataa   87458

chimp.LOC112206744.LOC107973052.GGT2-.646279-708040.rev.compl      ------------------------------------------------------------   61762
FAM230B-LOC105372935-GGT2.NCBI.GGT2.rev.compl                      agtgttatcagatttctttcttttttttttttttgagactgggtctgattctgtagccca   87518

chimp.LOC112206744.LOC107973052.GGT2-.646279-708040.rev.compl      ------------------------------------------------------------   61762
FAM230B-LOC105372935-GGT2.NCBI.GGT2.rev.compl                      ggctggagtgcaggggtataataatagctcactgcagcttcaaactcctgagtttgtttg   87578

chimp.LOC112206744.LOC107973052.GGT2-.646279-708040.rev.compl      ------------------------------------------------------------   61762
FAM230B-LOC105372935-GGT2.NCBI.GGT2.rev.compl                      tttgtttgtctgtttgtttgtttgtttggagagacagcgtctcgaactcctgggctccag   87638

chimp.LOC112206744.LOC107973052.GGT2-.646279-708040.rev.compl      ------------------------------------------------------------   61762
FAM230B-LOC105372935-GGT2.NCBI.GGT2.rev.compl                      tgatcctcccacctcagcctcccaaagtgctgggattacaagcatgagccactgtgcccc   87698

chimp.LOC112206744.LOC107973052.GGT2-.646279-708040.rev.compl      ------------------------------------------------------------   61762
FAM230B-LOC105372935-GGT2.NCBI.GGT2.rev.compl                      agcccaacatctgatttcaaaagtactattgatattataatcaccattaaaaatcactga   87758

chimp.LOC112206744.LOC107973052.GGT2-.646279-708040.rev.compl      ------------------------------------------------------------   61762
FAM230B-LOC105372935-GGT2.NCBI.GGT2.rev.compl                      agtttttgtcacccaaagttttacgttgaaatatagaacatttcatttttttcccacaca   87818

chimp.LOC112206744.LOC107973052.GGT2-.646279-708040.rev.compl      ------------------------------------------------------------   61762
FAM230B-LOC105372935-GGT2.NCBI.GGT2.rev.compl                      cctcacatctgcttttgatttttaacatgtgcatgaattacctcttattagaaaaaaaaa   87878

chimp.LOC112206744.LOC107973052.GGT2-.646279-708040.rev.compl      ------------------------------------------------------------   61762
FAM230B-LOC105372935-GGT2.NCBI.GGT2.rev.compl                      cacaaaatttttggtaaaagaattttccttaagtgggtatttttttccctaagtctgaaa   87938

chimp.LOC112206744.LOC107973052.GGT2-.646279-708040.rev.compl      ------------------------------------------------------------   61762
FAM230B-LOC105372935-GGT2.NCBI.GGT2.rev.compl                      tgacttagtaaggataagaggtcaaaccatatgctttgtagtgaaactgcaactatgatg   87998

chimp.LOC112206744.LOC107973052.GGT2-.646279-708040.rev.compl      ------------------------------------------------------------   61762
FAM230B-LOC105372935-GGT2.NCBI.GGT2.rev.compl                      aataacattagaattctggatgccttccagcagctggaacagggcagacctgaggcggga   88058

chimp.LOC112206744.LOC107973052.GGT2-.646279-708040.rev.compl      ------------------------------------------------------------   61762
FAM230B-LOC105372935-GGT2.NCBI.GGT2.rev.compl                      gtcctcatgcttaggggaaggggctcaggcactgggagtcggtcagggctcccaggatgt   88118

chimp.LOC112206744.LOC107973052.GGT2-.646279-708040.rev.compl      ------------------------------------------------------------   61762
FAM230B-LOC105372935-GGT2.NCBI.GGT2.rev.compl                      aaaagatacacatgaaagaaacaaacagtgggactcgggccagtgcaaaatttggcctgg   88178

chimp.LOC112206744.LOC107973052.GGT2-.646279-708040.rev.compl      ------------------------------------------------------------   61762
FAM230B-LOC105372935-GGT2.NCBI.GGT2.rev.compl                      aactgagctaaaaactgtcatactggtgggaagaagcagattggttcaacagggtaacca   88238

chimp.LOC112206744.LOC107973052.GGT2-.646279-708040.rev.compl      ------------------------------------------------------------   61762
FAM230B-LOC105372935-GGT2.NCBI.GGT2.rev.compl                      aatgtgaaaaagaaaagcctcacaagaattaaaattgatggttggaactgggcgctccga   88298

chimp.LOC112206744.LOC107973052.GGT2-.646279-708040.rev.compl      ------------------------------------------------------------   61762
FAM230B-LOC105372935-GGT2.NCBI.GGT2.rev.compl                      ggctgtgggccggtgtcctgccccgaagggtttggctgccagcaggtgcagcgctgccct   88358

chimp.LOC112206744.LOC107973052.GGT2-.646279-708040.rev.compl      ------------------------------------------------------------   61762
FAM230B-LOC105372935-GGT2.NCBI.GGT2.rev.compl                      gggataccagctcctgggatgcgctgtagtatcatcaatcaccggtgctgctcgctgggc   88418
```

```
chimp.LOC112206744.LOC107973052.GGT2-.646279-708040.rev.compl      ------------------------------------------------------------   61762
FAM230B-LOC105372935-GGT2.NCBI.GGT2.rev.compl                      cttggaagcacacagtagtagcagatgtcagcaggggccaccagggccacctgccagtg   88478

chimp.LOC112206744.LOC107973052.GGT2-.646279-708040.rev.compl      ------------------------------------------------------------   61762
FAM230B-LOC105372935-GGT2.NCBI.GGT2.rev.compl                      agcccactgcttagctgagtcatggtcaagtttctctgcagattgttgcctgaaaatatt   88538

chimp.LOC112206744.LOC107973052.GGT2-.646279-708040.rev.compl      ------------------------------------------------------------   61762
FAM230B-LOC105372935-GGT2.NCBI.GGT2.rev.compl                      tcaccacctggaacctccctgtcccacccatcttcacttgcaatactcactcccctcacc   88598

chimp.LOC112206744.LOC107973052.GGT2-.646279-708040.rev.compl      ------------------------------------------------------------   61762
FAM230B-LOC105372935-GGT2.NCBI.GGT2.rev.compl                      tctgcccctaaagcaattattcattaaccacagggatccaccatccaggaacaaaagagg   88658

chimp.LOC112206744.LOC107973052.GGT2-.646279-708040.rev.compl      ------------------------------------------------------------   61762
FAM230B-LOC105372935-GGT2.NCBI.GGT2.rev.compl                      cttgaaagctctatctgtgcctgcacttcttggtccaaaaggcgcaggggggatgggggag   88718

chimp.LOC112206744.LOC107973052.GGT2-.646279-708040.rev.compl      ------------------------------------------------------------   61762
FAM230B-LOC105372935-GGT2.NCBI.GGT2.rev.compl                      aggcaccggggtcatggtgcaggctcggctgttgagcctcattccccaaacacaatgtga   88778

chimp.LOC112206744.LOC107973052.GGT2-.646279-708040.rev.compl      ------------------------------------------------------------   61762
FAM230B-LOC105372935-GGT2.NCBI.GGT2.rev.compl                      caggtatcaatagttctggagttgatcatttcacagaaaggaaactgctgtcaacctgcc   88838

chimp.LOC112206744.LOC107973052.GGT2-.646279-708040.rev.compl      ------------------------------------------------------------   61762
FAM230B-LOC105372935-GGT2.NCBI.GGT2.rev.compl                      caaggccacacagctagtcctggataaattgacttaaagaacccaggtctctgattccca   88898

chimp.LOC112206744.LOC107973052.GGT2-.646279-708040.rev.compl      ------------------------------------------------------------   61762
FAM230B-LOC105372935-GGT2.NCBI.GGT2.rev.compl                      aatcagtgctcttacccctcccacgccacctctctcttctaaagatccacccaatggaac   88958

chimp.LOC112206744.LOC107973052.GGT2-.646279-708040.rev.compl      ------------------------------------------------------------   61762
FAM230B-LOC105372935-GGT2.NCBI.GGT2.rev.compl                      tgccccagggctggtatcccagctacttctccattcatgccgcaggtcaatgattcaggg   89018

chimp.LOC112206744.LOC107973052.GGT2-.646279-708040.rev.compl      ------------------------------------------------------------   61762
FAM230B-LOC105372935-GGT2.NCBI.GGT2.rev.compl                      cggacactcctcaagtgaccttcagaggccctgactcactcctccgacagtcctttgtgt   89078

chimp.LOC112206744.LOC107973052.GGT2-.646279-708040.rev.compl      ------------------------------------------------------------   61762
FAM230B-LOC105372935-GGT2.NCBI.GGT2.rev.compl                      ctggactcccagccgctcctcagcagctctgtcctcttttgtacatttattactgtcagc   89138

chimp.LOC112206744.LOC107973052.GGT2-.646279-708040.rev.compl      ------------------------------------------------------------   61762
FAM230B-LOC105372935-GGT2.NCBI.GGT2.rev.compl                      ctgaggctagcaggaggtctcaatgcctggtgatctgaggccagcctgtcccaggccctc   89198

chimp.LOC112206744.LOC107973052.GGT2-.646279-708040.rev.compl      ------------------------------------------------------------   61762
FAM230B-LOC105372935-GGT2.NCBI.GGT2.rev.compl                      ccagtcccgtgaggtcctcctggggatgcaggcacagcgactgacaaggataaggagcag   89258

chimp.LOC112206744.LOC107973052.GGT2-.646279-708040.rev.compl      ------------------------------------------------------------   61762
FAM230B-LOC105372935-GGT2.NCBI.GGT2.rev.compl                      aagggcaggtggctttctgggtgtgagcttggccttctgccacacccatgttcagaaagg   89318

chimp.LOC112206744.LOC107973052.GGT2-.646279-708040.rev.compl      ------------------------------------------------------------   61762
FAM230B-LOC105372935-GGT2.NCBI.GGT2.rev.compl                      tgtctacgggcacatcttgtgttgtcacaggtgtagaaagggctttgacacgcggcagat   89378

chimp.LOC112206744.LOC107973052.GGT2-.646279-708040.rev.compl      ------------------------------------------------------------   61762
FAM230B-LOC105372935-GGT2.NCBI.GGT2.rev.compl                      cctccaatccctgtgaaagagggatcattctttctattatacagtgaggacgctgaagtc   89438

chimp.LOC112206744.LOC107973052.GGT2-.646279-708040.rev.compl      ------------------------------------------------------------   61762
FAM230B-LOC105372935-GGT2.NCBI.GGT2.rev.compl                      cagaaaggtcaggtgactgatgaaaggtcataaaattagcaggaatcagggaaagggcag   89498

chimp.LOC112206744.LOC107973052.GGT2-.646279-708040.rev.compl      ------------------------------------------------------------   61762
FAM230B-LOC105372935-GGT2.NCBI.GGT2.rev.compl                      gaaagcagagcttccagttgcaaggtcaggactcttcagcccagcaggcgagtttgccag   89558

chimp.LOC112206744.LOC107973052.GGT2-.646279-708040.rev.compl      ------------------------------------------------------------   61762
FAM230B-LOC105372935-GGT2.NCBI.GGT2.rev.compl                      ccagtctgtgccctggtctctgccctccccaaacacgtgcaccaatttggaggggctgct   89618

chimp.LOC112206744.LOC107973052.GGT2-.646279-708040.rev.compl      ------------------------------------------------------------   61762
FAM230B-LOC105372935-GGT2.NCBI.GGT2.rev.compl                      cccagcacgcatgctgcaagataaacttcagtgctcagccctctcactgtgacaacaatc   89678

chimp.LOC112206744.LOC107973052.GGT2-.646279-708040.rev.compl      ------------------------------------------------------------   61762
FAM230B-LOC105372935-GGT2.NCBI.GGT2.rev.compl                      ccttagaaaggggtccacgctgtccatctgggcccctacaccaaagaacacctaattatc   89738

chimp.LOC112206744.LOC107973052.GGT2-.646279-708040.rev.compl      ------------------------------------------------------------   61762
FAM230B-LOC105372935-GGT2.NCBI.GGT2.rev.compl                      agtggtcctcactcaggaaacatgcccacaaataaagacaaaagtaaacccaactacaca   89798

chimp.LOC112206744.LOC107973052.GGT2-.646279-708040.rev.compl      ------------------------------------------------------------   61762
FAM230B-LOC105372935-GGT2.NCBI.GGT2.rev.compl                      ttcctcaaaatcctcctctccatttcacagaaacaccacaacagagcagaactgcaacct   89858

chimp.LOC112206744.LOC107973052.GGT2-.646279-708040.rev.compl      ------------------------------------------------------------   61762
FAM230B-LOC105372935-GGT2.NCBI.GGT2.rev.compl                      gatagcatccctgtctggttttggctctttcaaaacatcagggacctcacaggaaagcaa   89918

chimp.LOC112206744.LOC107973052.GGT2-.646279-708040.rev.compl      ------------------------------------------------------------   61762
FAM230B-LOC105372935-GGT2.NCBI.GGT2.rev.compl                      tgcagaaaactgcagtcatttggccgcttgttaaggagagccctgagcagcaagctccag   89978

chimp.LOC112206744.LOC107973052.GGT2-.646279-708040.rev.compl      ------------------------------------------------------------   61762
FAM230B-LOC105372935-GGT2.NCBI.GGT2.rev.compl                      accggagatgcgcatataacagcagcagttccacacagccctgcacagcagcaattcagc   90038

chimp.LOC112206744.LOC107973052.GGT2-.646279-708040.rev.compl      ------------------------------------------------------------   61762
FAM230B-LOC105372935-GGT2.NCBI.GGT2.rev.compl                      ctctggcagctgtgatgatgctgaggcagtgtccccactgccccagtggcttctgcatgc   90098

chimp.LOC112206744.LOC107973052.GGT2-.646279-708040.rev.compl      ------------------------------------------------------------   61762
FAM230B-LOC105372935-GGT2.NCBI.GGT2.rev.compl                      aggttcatttgggacaggacacctctaactccatctctggccaggagccagcatagtagg   90158

chimp.LOC112206744.LOC107973052.GGT2-.646279-708040.rev.compl      ------------------------------------------------------------   61762
FAM230B-LOC105372935-GGT2.NCBI.GGT2.rev.compl                      tgccgtgaaagccaggaagtgaactgcacagaatggactgttccagtgtccatgggctgc   90218

chimp.LOC112206744.LOC107973052.GGT2-.646279-708040.rev.compl      ------------------------------------------------------------   61762
FAM230B-LOC105372935-GGT2.NCBI.GGT2.rev.compl                      aagtgggtcccacgtcacccacaggtgaatcttaattatgaaccaaggtgacggcagaaa   90278

chimp.LOC112206744.LOC107973052.GGT2-.646279-708040.rev.compl      ------------------------------------------------------------   61762
FAM230B-LOC105372935-GGT2.NCBI.GGT2.rev.compl                      ggatgggcagcaaaggagggtgtgtgtggtgatcagccatcagaggagacggccctgtgt   90338

chimp.LOC112206744.LOC107973052.GGT2-.646279-708040.rev.compl      ------------------------------------------------------------   61762
FAM230B-LOC105372935-GGT2.NCBI.GGT2.rev.compl                      gatgaaaggacctcctcaggaaacctccccaccagcaactggctccaaatggtcagactt   90398

chimp.LOC112206744.LOC107973052.GGT2-.646279-708040.rev.compl      ------------------------------------------------------------   61762
FAM230B-LOC105372935-GGT2.NCBI.GGT2.rev.compl                      tccaagaaattcctgtgaaaaggacaggctcagggcatgtgaatgtgatagtcagcaatt   90458

chimp.LOC112206744.LOC107973052.GGT2-.646279-708040.rev.compl      ------------------------------------------------------------   61762
FAM230B-LOC105372935-GGT2.NCBI.GGT2.rev.compl                      cgcacctcagcagccagtgacactatggtatagggaaaagaacttagattaggcatcaaa   90518

chimp.LOC112206744.LOC107973052.GGT2-.646279-708040.rev.compl      ------------------------------------------------------------   61762
FAM230B-LOC105372935-GGT2.NCBI.GGT2.rev.compl                      agtacccggtccccttcatacatggctggtggggaggtaggatggtgcagccactttgga   90578

chimp.LOC112206744.LOC107973052.GGT2-.646279-708040.rev.compl      ------------------------------------------------------------   61762
```

```
FAM230B-LOC105372935-GGT2.NCBI.GGT2.rev.compl                   aaacagtctggtagtttctttcaaagttaccaaatgacccagcaatcccactcctaagta   90638

chimp.LOC112206744.LOC107973052.GGT2-.646279-708040.rev.compl   ------------------------------------------------------------   61762
FAM230B-LOC105372935-GGT2.NCBI.GGT2.rev.compl                   tttacccaagagaaataaaacctgtgtccacacaaagacttgtacacatcctaatagctg   90698

chimp.LOC112206744.LOC107973052.GGT2-.646279-708040.rev.compl   ------------------------------------------------------------   61762
FAM230B-LOC105372935-GGT2.NCBI.GGT2.rev.compl                   tattcatagtagccccaaactggaaacaacccaaatgcccatcagtttgtgaagagatgg   90758

chimp.LOC112206744.LOC107973052.GGT2-.646279-708040.rev.compl   ------------------------------------------------------------   61762
FAM230B-LOC105372935-GGT2.NCBI.GGT2.rev.compl                   acaaaatgtgctatatccataaaacggaaccctactcagcaataaaaaggtgtgaactag   90818

chimp.LOC112206744.LOC107973052.GGT2-.646279-708040.rev.compl   ------------------------------------------------------------   61762
FAM230B-LOC105372935-GGT2.NCBI.GGT2.rev.compl                   atacacacaagaacatggagggatctcaaaagcactgtgctgagcgagagaagccagatt   90878

chimp.LOC112206744.LOC107973052.GGT2-.646279-708040.rev.compl   ------------------------------------------------------------   61762
FAM230B-LOC105372935-GGT2.NCBI.GGT2.rev.compl                   caaagggctacacactgtaggatgccatgtgcatcacattctgtacagtgcaaaactgta   90938

chimp.LOC112206744.LOC107973052.GGT2-.646279-708040.rev.compl   ------------------------------------------------------------   61762
FAM230B-LOC105372935-GGT2.NCBI.GGT2.rev.compl                   gtggcaggattcaggtcccctgaggctggggtcctggaggcaggagaggggctggtagca   90998

chimp.LOC112206744.LOC107973052.GGT2-.646279-708040.rev.compl   ------------------------------------------------------------   61762
FAM230B-LOC105372935-GGT2.NCBI.GGT2.rev.compl                   aggaggcacaagggaacttttggggacagaggagtggtccatatttgataggtggtggt   91058

chimp.LOC112206744.LOC107973052.GGT2-.646279-708040.rev.compl   ------------------------------------------------------------   61762
FAM230B-LOC105372935-GGT2.NCBI.GGT2.rev.compl                   tgtgtggctgctcatgtttgtcaacaaagaattacatattttaaactggtgagttccgtt   91118

chimp.LOC112206744.LOC107973052.GGT2-.646279-708040.rev.compl   ------------------------------------------------------------   61762
FAM230B-LOC105372935-GGT2.NCBI.GGT2.rev.compl                   gtatgcaagcaaattataccacaatgaagcaaatctttaaaacactaggttccttttctt   91178

chimp.LOC112206744.LOC107973052.GGT2-.646279-708040.rev.compl   ------------------------------------------------------------   61762
FAM230B-LOC105372935-GGT2.NCBI.GGT2.rev.compl                   tatctagagctcaaagtagtggcccatgctggctatgacttggagaaatcacacaagctt   91238

chimp.LOC112206744.LOC107973052.GGT2-.646279-708040.rev.compl   ------------------------------------------------------------   61762
FAM230B-LOC105372935-GGT2.NCBI.GGT2.rev.compl                   actgggtctcaactgcttcatttgtacaggtggctgcataacactccccgggcaggggaa   91298

chimp.LOC112206744.LOC107973052.GGT2-.646279-708040.rev.compl   ------------------------------------------------------------   61762
FAM230B-LOC105372935-GGT2.NCBI.GGT2.rev.compl                   ctgtagggatgaaatcagcaaatgtgagtgaacacacagaggaagcaacacataggcctt   91358

chimp.LOC112206744.LOC107973052.GGT2-.646279-708040.rev.compl   ------------------------------------------------------------   61762
FAM230B-LOC105372935-GGT2.NCBI.GGT2.rev.compl                   ccccaaatacccacttctgttttctttcctcagtgtgagtaagtggctctaatgatccac   91418

chimp.LOC112206744.LOC107973052.GGT2-.646279-708040.rev.compl   ------------------------------------------------------------   61762
FAM230B-LOC105372935-GGT2.NCBI.GGT2.rev.compl                   aaaccttccaaccagccaagtcaaggaccagcaggaggactcaggaaagtactgaaatgc   91478

chimp.LOC112206744.LOC107973052.GGT2-.646279-708040.rev.compl   ------------------------------------------------------------   61762
FAM230B-LOC105372935-GGT2.NCBI.GGT2.rev.compl                   tggggagaaccaggacagcggctgagcacagccagacagggagggcagcctggtggggcc   91538

chimp.LOC112206744.LOC107973052.GGT2-.646279-708040.rev.compl   ------------------------------------------------------------   61762
FAM230B-LOC105372935-GGT2.NCBI.GGT2.rev.compl                   ctgggagccatggacgcgactgcgctgggagtggggatgggagatagcaggagttggtgg   91598

chimp.LOC112206744.LOC107973052.GGT2-.646279-708040.rev.compl   ------------------------------------------------------------   61762
FAM230B-LOC105372935-GGT2.NCBI.GGT2.rev.compl                   gtggggcacaaagtgggagccagggccaaattgctgctgatttgaccattttgcaaaagg   91658

chimp.LOC112206744.LOC107973052.GGT2-.646279-708040.rev.compl   ------------------------------------------------------------   61762
FAM230B-LOC105372935-GGT2.NCBI.GGT2.rev.compl                   ccagcctggctgtggcctcaggcctctcatttcctctaccataatgaggggtcacggaag   91718

chimp.LOC112206744.LOC107973052.GGT2-.646279-708040.rev.compl   ------------------------------------------------------------   61762
FAM230B-LOC105372935-GGT2.NCBI.GGT2.rev.compl                   tgccttcccacaactgcagcatctccctccacatttgggctcaaatacagagtggggccg   91778

chimp.LOC112206744.LOC107973052.GGT2-.646279-708040.rev.compl   ------------------------------------------------------------   61762
FAM230B-LOC105372935-GGT2.NCBI.GGT2.rev.compl                   gagacagaaaaagtctccgtgacacactgtgatttgtttctgcactgtgtgaacccttgg   91838

chimp.LOC112206744.LOC107973052.GGT2-.646279-708040.rev.compl   ------------------------------------------------------------   61762
FAM230B-LOC105372935-GGT2.NCBI.GGT2.rev.compl                   ctctgtgacacagaacacaggtttcagcagttcctgtcacttcccacaggcgaaacagac   91898

chimp.LOC112206744.LOC107973052.GGT2-.646279-708040.rev.compl   ------------------------------------------------------------   61762
FAM230B-LOC105372935-GGT2.NCBI.GGT2.rev.compl                   agagcattcttttgtccggcttctggtgaaacgccattatcgttattttaggaggaaga   91958

chimp.LOC112206744.LOC107973052.GGT2-.646279-708040.rev.compl   ------------------------------------------------------------   61762
FAM230B-LOC105372935-GGT2.NCBI.GGT2.rev.compl                   tggagaattggggtgggtccccacaaagaggatggattcagaagacttccaggaacctta   92018

chimp.LOC112206744.LOC107973052.GGT2-.646279-708040.rev.compl   ------------------------------------------------------------   61762
FAM230B-LOC105372935-GGT2.NCBI.GGT2.rev.compl                   aacggcttggaaactaattgtgccagttctttttgatgtctaattttattctaaaatttc   92078

chimp.LOC112206744.LOC107973052.GGT2-.646279-708040.rev.compl   ------------------------------------------------------------   61762
FAM230B-LOC105372935-GGT2.NCBI.GGT2.rev.compl                   ctctcagcacaatatattaatactgatcagccaagagaaggttaggttttggctcacaaa   92138

chimp.LOC112206744.LOC107973052.GGT2-.646279-708040.rev.compl   ------------------------------------------------------------   61762
FAM230B-LOC105372935-GGT2.NCBI.GGT2.rev.compl                   tagtcaaggagagaatctccttcattcattgagaaggcagaagatggcgttaaagagtat   92198

chimp.LOC112206744.LOC107973052.GGT2-.646279-708040.rev.compl   ------------------------------------------------------------   61762
FAM230B-LOC105372935-GGT2.NCBI.GGT2.rev.compl                   tcacaggtaactaagattgccagagggagaatcgagtgagttgtcagtggtgaaatacag   92258

chimp.LOC112206744.LOC107973052.GGT2-.646279-708040.rev.compl   ------------------------------------------------------------   61762
FAM230B-LOC105372935-GGT2.NCBI.GGT2.rev.compl                   aggaggtagagagagctacactgagacaacaaaggcagcttaagggaacaaagccagcta   92318

chimp.LOC112206744.LOC107973052.GGT2-.646279-708040.rev.compl   ------------------------------------------------------------   61762
FAM230B-LOC105372935-GGT2.NCBI.GGT2.rev.compl                   agggaacaaagccggggtccctcaggtctgtggccaccggctccagccaggacagcctgg   92378

chimp.LOC112206744.LOC107973052.GGT2-.646279-708040.rev.compl   ------------------------------------------------------------   61762
FAM230B-LOC105372935-GGT2.NCBI.GGT2.rev.compl                   tagctgaagagcacatccttgcccgttacatgcctgtgtttcaagtgatgctaacttgct   92438

chimp.LOC112206744.LOC107973052.GGT2-.646279-708040.rev.compl   ------------------------------------------------------------   61762
FAM230B-LOC105372935-GGT2.NCBI.GGT2.rev.compl                   aatgcagatatctccccaaaccctgcccctggctcaccacccagtgagcaatcactcact   92498

chimp.LOC112206744.LOC107973052.GGT2-.646279-708040.rev.compl   ------------------------------------------------------------   61762
FAM230B-LOC105372935-GGT2.NCBI.GGT2.rev.compl                   ttccccaggacaccttcagggaggtactctgccagcaccgcacacttaaacagctactgc   92558

chimp.LOC112206744.LOC107973052.GGT2-.646279-708040.rev.compl   ------------------------------------------------------------   61762
FAM230B-LOC105372935-GGT2.NCBI.GGT2.rev.compl                   aggttcttctctcagacgcagcacggttcctgagatgacgtcagcgctgctcgctgaaag   92618

chimp.LOC112206744.LOC107973052.GGT2-.646279-708040.rev.compl   ------------------------------------------------------------   61762
FAM230B-LOC105372935-GGT2.NCBI.GGT2.rev.compl                   caccaggcaaggaggcaaaacaaaggaaatcctaaagagcaggcgagagaacagtggaac   92678

chimp.LOC112206744.LOC107973052.GGT2-.646279-708040.rev.compl   ------------------------------------------------------------   61762
FAM230B-LOC105372935-GGT2.NCBI.GGT2.rev.compl                   tggcagagagaggcactggtgcggatggagagggcagcaataaaaactgggggacactta   92738

chimp.LOC112206744.LOC107973052.GGT2-.646279-708040.rev.compl   ------------------------------------------------------------   61762
FAM230B-LOC105372935-GGT2.NCBI.GGT2.rev.compl                   gaggcaaaggtatggcaggtgacaaaaaagatgagctgaagctcaggagagacccaggca   92798
```

```
chimp.LOC112206744.LOC107973052.GGT2-.646279-708040.rev.compl    ------------------------------------------------------------   61762
FAM230B-LOC105372935-GGT2.NCBI.GGT2.rev.compl                    gcgatgcccaaatgaccggctgaggaatcacctcactgctgctgatgcccttgacagcga   92858

chimp.LOC112206744.LOC107973052.GGT2-.646279-708040.rev.compl    ------------------------------------------------------------   61762
FAM230B-LOC105372935-GGT2.NCBI.GGT2.rev.compl                    tgtctgaggctggcgtgagtgctccatgctcagagatacctagaacccagctacaatggc   92918

chimp.LOC112206744.LOC107973052.GGT2-.646279-708040.rev.compl    ------------------------------------------------------------   61762
FAM230B-LOC105372935-GGT2.NCBI.GGT2.rev.compl                    cttatcagactgaaacaagaacaccaagatattccaagggaggtgacaatggggtccaaa   92978

chimp.LOC112206744.LOC107973052.GGT2-.646279-708040.rev.compl    ------------------------------------------------------------   61762
FAM230B-LOC105372935-GGT2.NCBI.GGT2.rev.compl                    ggttgtgcatttatggatcacaactatgtgctgagtgtggcccaaatcccagctccatat   93038

chimp.LOC112206744.LOC107973052.GGT2-.646279-708040.rev.compl    ------------------------------------------------------------   61762
FAM230B-LOC105372935-GGT2.NCBI.GGT2.rev.compl                    ggtcacaccatgtctacagccaatcagctgaccggggtggcaacccctggctctgctcaa   93098

chimp.LOC112206744.LOC107973052.GGT2-.646279-708040.rev.compl    ------------------------------------------------------------   61762
FAM230B-LOC105372935-GGT2.NCBI.GGT2.rev.compl                    caataatgacaaccatcatttactgagcacttactatgtgccagaccgatgctaagtgct   93158

chimp.LOC112206744.LOC107973052.GGT2-.646279-708040.rev.compl    ------------------------------------------------------------   61762
FAM230B-LOC105372935-GGT2.NCBI.GGT2.rev.compl                    ttcctcagatgtttccatttaactcttagaatcaccctaatgagtatctcctattatttt   93218

chimp.LOC112206744.LOC107973052.GGT2-.646279-708040.rev.compl    ------------------------------------------------------------   61762
FAM230B-LOC105372935-GGT2.NCBI.GGT2.rev.compl                    acagatgaggaaatgagagtttagaaaagtaacttgcaaaatcatactgctggctccata   93278

chimp.LOC112206744.LOC107973052.GGT2-.646279-708040.rev.compl    ------------------------------------------------------------   61762
FAM230B-LOC105372935-GGT2.NCBI.GGT2.rev.compl                    acaaatgttgattgaatgttgtctgggtggacagaggcaaggatgaccggaacgagacaa   93338

chimp.LOC112206744.LOC107973052.GGT2-.646279-708040.rev.compl    ------------------------------------------------------------   61762
FAM230B-LOC105372935-GGT2.NCBI.GGT2.rev.compl                    acaaacaaaaggctgactgacagaccattcgggtagcacttttcctgggattaagcttag   93398

chimp.LOC112206744.LOC107973052.GGT2-.646279-708040.rev.compl    ------------------------------------------------------------   61762
FAM230B-LOC105372935-GGT2.NCBI.GGT2.rev.compl                    accttgtaggcctatcagccagaagcagtgtgtaaaataagagtggctgactgggcacgg   93458

chimp.LOC112206744.LOC107973052.GGT2-.646279-708040.rev.compl    ------------------------------------------------------------   61762
FAM230B-LOC105372935-GGT2.NCBI.GGT2.rev.compl                    tggctcaagcctgtaatccaagcactttgggaggccaaggcgggcggatcacctgaggtc   93518

chimp.LOC112206744.LOC107973052.GGT2-.646279-708040.rev.compl    ------------------------------------------------------------   61762
FAM230B-LOC105372935-GGT2.NCBI.GGT2.rev.compl                    aggagttcaagaccagcctggccaacatgatgaaaccccgtctctactacaaatacaaaa   93578

chimp.LOC112206744.LOC107973052.GGT2-.646279-708040.rev.compl    ------------------------------------------------------------   61762
FAM230B-LOC105372935-GGT2.NCBI.GGT2.rev.compl                    attagccgggtgtgatggcacacgcctgtaatcctagttactcgggaggctgaggcacga   93638

chimp.LOC112206744.LOC107973052.GGT2-.646279-708040.rev.compl    ------------------------------------------------------------   61762
FAM230B-LOC105372935-GGT2.NCBI.GGT2.rev.compl                    gaatcgcttgaacctgggaggcagaggttgcgataagctgagattgggccactgcactcc   93698

chimp.LOC112206744.LOC107973052.GGT2-.646279-708040.rev.compl    ------------------------------------------------------------   61762
FAM230B-LOC105372935-GGT2.NCBI.GGT2.rev.compl                    agcctgggcgacagagcaagactccctctgaaaaaaaaaaaaaaaaagagtggctaccca   93758

chimp.LOC112206744.LOC107973052.GGT2-.646279-708040.rev.compl    ------------------------------------------------------------   61762
FAM230B-LOC105372935-GGT2.NCBI.GGT2.rev.compl                    agaacgcaacagcagcagcaaacactgatctgatgacaccccagttttgcagaagaggaa   93818

chimp.LOC112206744.LOC107973052.GGT2-.646279-708040.rev.compl    ------------------------------------------------------------   61762
FAM230B-LOC105372935-GGT2.NCBI.GGT2.rev.compl                    ggaaaaataagaatgtcaactctccattcaacagagaaacagtgccaccactgtctttaa   93878

chimp.LOC112206744.LOC107973052.GGT2-.646279-708040.rev.compl    ------------------------------------------------------------   61762
FAM230B-LOC105372935-GGT2.NCBI.GGT2.rev.compl                    attttcaaacttatcgagaaaagctccagagaggccagtgccagcaatgccggctgtctc   93938

chimp.LOC112206744.LOC107973052.GGT2-.646279-708040.rev.compl    ------------------------------------------------------------   61762
FAM230B-LOC105372935-GGT2.NCBI.GGT2.rev.compl                    ccaagtctgaagggttttcctctgggtattaggagttgaggtgtttggttacagaaggaa   93998

chimp.LOC112206744.LOC107973052.GGT2-.646279-708040.rev.compl    ------------------------------------------------------------   61762
FAM230B-LOC105372935-GGT2.NCBI.GGT2.rev.compl                    acaggatgcacgggagatcacacacagagtaagaggtgcctctcaaatgcctttcccacg   94058

chimp.LOC112206744.LOC107973052.GGT2-.646279-708040.rev.compl    ------------------------------------------------------------   61762
FAM230B-LOC105372935-GGT2.NCBI.GGT2.rev.compl                    tttctaactctgctttttttaaagcacgatcagacccgccatctgcacttttacctgctg   94118

chimp.LOC112206744.LOC107973052.GGT2-.646279-708040.rev.compl    ------------------------------------------------------------   61762
FAM230B-LOC105372935-GGT2.NCBI.GGT2.rev.compl                    cctgacgaaaaccagatgccaaaggcctcccagcactcacggggccctttacccttagga   94178

chimp.LOC112206744.LOC107973052.GGT2-.646279-708040.rev.compl    ------------------------------------------------------------   61762
FAM230B-LOC105372935-GGT2.NCBI.GGT2.rev.compl                    aaacaccgtgattcagccaggcgagattgatgcaacattgtaacaaagcgtatgcaaagt   94238

chimp.LOC112206744.LOC107973052.GGT2-.646279-708040.rev.compl    ------------------------------------------------------------   61762
FAM230B-LOC105372935-GGT2.NCBI.GGT2.rev.compl                    gcctagctccggactgcttgcagaaaaggcacattttgactcttctgagagtgggaaaga   94298

chimp.LOC112206744.LOC107973052.GGT2-.646279-708040.rev.compl    ------------------------------------------------------------   61762
FAM230B-LOC105372935-GGT2.NCBI.GGT2.rev.compl                    tttgaaagatgggatttgcaccttattcttattcaagtctggagatctggaacttcttcc   94358

chimp.LOC112206744.LOC107973052.GGT2-.646279-708040.rev.compl    ------------------------------------------------------------   61762
FAM230B-LOC105372935-GGT2.NCBI.GGT2.rev.compl                    tgaagctccatgtttagccagataattgagacgctccacctggtcaccactggacaccag   94418

chimp.LOC112206744.LOC107973052.GGT2-.646279-708040.rev.compl    ------------------------------------------------------------   61762
FAM230B-LOC105372935-GGT2.NCBI.GGT2.rev.compl                    ccacccaaacattctcctctgcaaggcacttggcattctggtgcctgtggagagagcagc   94478

chimp.LOC112206744.LOC107973052.GGT2-.646279-708040.rev.compl    ------------------------------------------------------------   61762
FAM230B-LOC105372935-GGT2.NCBI.GGT2.rev.compl                    cttctcactagcacagctcatgaacaaggatccccttccctggacagccaagatgtaaac   94538

chimp.LOC112206744.LOC107973052.GGT2-.646279-708040.rev.compl    ------------------------------------------------------------   61762
FAM230B-LOC105372935-GGT2.NCBI.GGT2.rev.compl                    ctgcccccagaaacactgagagtcctagaggtagaatgagtcggccagcaagctccagcc   94598

chimp.LOC112206744.LOC107973052.GGT2-.646279-708040.rev.compl    ------------------------------------------------------------   61762
FAM230B-LOC105372935-GGT2.NCBI.GGT2.rev.compl                    cattgaatgcagacccctgagcaaatcaagtcaggagaaggtggacaactgtaccctgaa   94658

chimp.LOC112206744.LOC107973052.GGT2-.646279-708040.rev.compl    ------------------------------------------------------------   61762
FAM230B-LOC105372935-GGT2.NCBI.GGT2.rev.compl                    gaccacataccacccagacccaacacaaatagctgtcagcactgccacccattacagctt   94718

chimp.LOC112206744.LOC107973052.GGT2-.646279-708040.rev.compl    ------------------------------------------------------------   61762
FAM230B-LOC105372935-GGT2.NCBI.GGT2.rev.compl                    cagatctcgcttctaccagcatttcccagctgagcaacaaccgtcaaagtctggtcctgg   94778

chimp.LOC112206744.LOC107973052.GGT2-.646279-708040.rev.compl    ------------------------------------------------------------   61762
FAM230B-LOC105372935-GGT2.NCBI.GGT2.rev.compl                    aaggacagccctccccgtcttggcagccacactttctggccctagcccaaggctgctgtg   94838

chimp.LOC112206744.LOC107973052.GGT2-.646279-708040.rev.compl    ------------------------------------------------------------   61762
FAM230B-LOC105372935-GGT2.NCBI.GGT2.rev.compl                    gcatgggaggaatcaatggaggaatacatggctcttccctggttccttcctctcccactt   94898

chimp.LOC112206744.LOC107973052.GGT2-.646279-708040.rev.compl    ------------------------------------------------------------   61762
FAM230B-LOC105372935-GGT2.NCBI.GGT2.rev.compl                    cattgactaacactagtcacatggccccaactgaactgtgagggaggctggaaaatctgg   94958
```

```
chimp.LOC112206744.LOC107973052.GGT2-.646279-708040.rev.compl     ------------------------------------------------------------   61762
FAM230B-LOC105372935-GGT2.NCBI.GGT2.rev.compl                     agtatgtgcatgcctagtgagttgcctctaccacatcctcttaacccttggcctctcatt   95018

chimp.LOC112206744.LOC107973052.GGT2-.646279-708040.rev.compl     ------------------------------------------------------------   61762
FAM230B-LOC105372935-GGT2.NCBI.GGT2.rev.compl                     cccaaagtctagccctattttcaaatgaattcaacgcctcattacttagatctttcctca   95078

chimp.LOC112206744.LOC107973052.GGT2-.646279-708040.rev.compl     ------------------------------------------------------------   61762
FAM230B-LOC105372935-GGT2.NCBI.GGT2.rev.compl                     ttacctcaaagccatagtggcaaaaagcaaacagcatcttcctaaaatcttcctcttcca   95138

chimp.LOC112206744.LOC107973052.GGT2-.646279-708040.rev.compl     ------------------------------------------------------------   61762
FAM230B-LOC105372935-GGT2.NCBI.GGT2.rev.compl                     tcttcctctcctagatatccatcttacccttctttgccagttaatttattacaatcttcc   95198

chimp.LOC112206744.LOC107973052.GGT2-.646279-708040.rev.compl     ------------------------------------------------------------   61762
FAM230B-LOC105372935-GGT2.NCBI.GGT2.rev.compl                     tttgacagcttcactccctccccgcactgctatcaactcaaatgtgagttatttcagtag   95258

chimp.LOC112206744.LOC107973052.GGT2-.646279-708040.rev.compl     ------------------------------------------------------------   61762
FAM230B-LOC105372935-GGT2.NCBI.GGT2.rev.compl                     cttgagcttgtcactggccttttactttttccccttccaaagaacctggtacccagcta   95318

chimp.LOC112206744.LOC107973052.GGT2-.646279-708040.rev.compl     ------------------------------------------------------------   61762
FAM230B-LOC105372935-GGT2.NCBI.GGT2.rev.compl                     cttaatccttatccttcttcccaaggcccactttcattcctttctcccccattcatgagc   95378

chimp.LOC112206744.LOC107973052.GGT2-.646279-708040.rev.compl     ------------------------------------------------------------   61762
FAM230B-LOC105372935-GGT2.NCBI.GGT2.rev.compl                     ctgcaatacttttcccgctgcataagtcacgtgtcaccatataaactcacaatttgaggt   95438

chimp.LOC112206744.LOC107973052.GGT2-.646279-708040.rev.compl     ------------------------------------------------------------   61762
FAM230B-LOC105372935-GGT2.NCBI.GGT2.rev.compl                     gctctagaatctgtccacggaattagtcaggacataatctccctgtttttcggcagtgcc   95498

chimp.LOC112206744.LOC107973052.GGT2-.646279-708040.rev.compl     ------------------------------------------------------------   61762
FAM230B-LOC105372935-GGT2.NCBI.GGT2.rev.compl                     acatccatacctgtcttcacaggtttgctcatgccaccattcctttccccagaacgccct   95558

chimp.LOC112206744.LOC107973052.GGT2-.646279-708040.rev.compl     ------------------------------------------------------------   61762
FAM230B-LOC105372935-GGT2.NCBI.GGT2.rev.compl                     tctgctcctcccacacaaatcctaatcctccctcaggacccatttaaaatacaatctcct   95618

chimp.LOC112206744.LOC107973052.GGT2-.646279-708040.rev.compl     ------------------------------------------------------------   61762
FAM230B-LOC105372935-GGT2.NCBI.GGT2.rev.compl                     cactcaactctttcctaccaacttgctttcccctctcctttctgccttaccacaaagcta   95678

chimp.LOC112206744.LOC107973052.GGT2-.646279-708040.rev.compl     ------------------------------------------------------------   61762
FAM230B-LOC105372935-GGT2.NCBI.GGT2.rev.compl                     agctctgtctctgaataatgtgctttttgatgataatctgaatggtacaagaagaaatcc   95738

chimp.LOC112206744.LOC107973052.GGT2-.646279-708040.rev.compl     ------------------------------------------------------------   61762
FAM230B-LOC105372935-GGT2.NCBI.GGT2.rev.compl                     tatttcatgctcctttgagggagtgtctaatataggttgccttcgactataagtagtaga   95798

chimp.LOC112206744.LOC107973052.GGT2-.646279-708040.rev.compl     ------------------------------------------------------------   61762
FAM230B-LOC105372935-GGT2.NCBI.GGT2.rev.compl                     aaattcccactcaactggcttcaacagtgaggacaaaatgtatggagattggacgcttca   95858

chimp.LOC112206744.LOC107973052.GGT2-.646279-708040.rev.compl     ------------------------------------------------------------   61762
FAM230B-LOC105372935-GGT2.NCBI.GGT2.rev.compl                     gtcttcaggggcttggccctcagggtttttcccatctcttcccgtctgctggcctcaaca   95918

chimp.LOC112206744.LOC107973052.GGT2-.646279-708040.rev.compl     ------------------------------------------------------------   61762
FAM230B-LOC105372935-GGT2.NCBI.GGT2.rev.compl                     aacctgctttcctcattgcaacaagatggctgcttcacgtccgagaatctagtgaaagaa   95978

chimp.LOC112206744.LOC107973052.GGT2-.646279-708040.rev.compl     ------------------------------------------------------------   61762
FAM230B-LOC105372935-GGT2.NCBI.GGT2.rev.compl                     gctgtcccttctgcatttccttttacaaggaaaaacatctttcccagcatctcccagcag   96038

chimp.LOC112206744.LOC107973052.GGT2-.646279-708040.rev.compl     ------------------------------------------------------------   61762
FAM230B-LOC105372935-GGT2.NCBI.GGT2.rev.compl                     acttctcatacctcagtggccaaaaccatgtctaaactacgggcctccaggatgggctca   96098

chimp.LOC112206744.LOC107973052.GGT2-.646279-708040.rev.compl     ------------------------------------------------------------   61762
FAM230B-LOC105372935-GGT2.NCBI.GGT2.rev.compl                     gctcaatcagcacataccctagagctggaggcagggtcacctttccttgagtggcgtgtg   96158

chimp.LOC112206744.LOC107973052.GGT2-.646279-708040.rev.compl     ------------------------------------------------------------   61762
FAM230B-LOC105372935-GGT2.NCBI.GGT2.rev.compl                     ggagaggtgtggcatccaacccaactgggtcctgccagcaggaagagaaaagaagagcca   96218

chimp.LOC112206744.LOC107973052.GGT2-.646279-708040.rev.compl     ------------------------------------------------------------   61762
FAM230B-LOC105372935-GGT2.NCBI.GGT2.rev.compl                     ctgtaggtaaccaatcacgtctaccacaggcagggaccaataccacatatctgtgtttcc   96278

chimp.LOC112206744.LOC107973052.GGT2-.646279-708040.rev.compl     ------------------------------------------------------------   61762
FAM230B-LOC105372935-GGT2.NCBI.GGT2.rev.compl                     cctcacttgccacccccatcctgtgctggcagatgaggatgctcaacaaatagctgtgga   96338

chimp.LOC112206744.LOC107973052.GGT2-.646279-708040.rev.compl     ------------------------------------------------------------   61762
FAM230B-LOC105372935-GGT2.NCBI.GGT2.rev.compl                     actacaaattatcctgtgggtgtgagaaaagagtgtgcattataatagggtggtacaatt   96398

chimp.LOC112206744.LOC107973052.GGT2-.646279-708040.rev.compl     ------------------------------------------------------------   61762
FAM230B-LOC105372935-GGT2.NCBI.GGT2.rev.compl                     ggacatgtgtgcacggaaaatggaaaaacagccatgaaatccaaacagatgcaaaaacgt   96458

chimp.LOC112206744.LOC107973052.GGT2-.646279-708040.rev.compl     ------------------------------------------------------------   61762
FAM230B-LOC105372935-GGT2.NCBI.GGT2.rev.compl                     tcttaacacagaaggtttgggaagctctgatctaagccctagcctgggccctactcccag   96518

chimp.LOC112206744.LOC107973052.GGT2-.646279-708040.rev.compl     ------------------------------------------------------------   61762
FAM230B-LOC105372935-GGT2.NCBI.GGT2.rev.compl                     ttccacagatagggagcaggccccaggccagagtgctgggcttctcccagcaggatccag   96578

chimp.LOC112206744.LOC107973052.GGT2-.646279-708040.rev.compl     ------------------------------------------------------------   61762
FAM230B-LOC105372935-GGT2.NCBI.GGT2.rev.compl                     ctggggctttctcctcagttcaacccttgcctctgaggaccagtggtctacagtccagcc   96638

chimp.LOC112206744.LOC107973052.GGT2-.646279-708040.rev.compl     ------------------------------------------------------------   61762
FAM230B-LOC105372935-GGT2.NCBI.GGT2.rev.compl                     caccacccttcagacagaagttccaggcacaaaaaggaagctcacaataaggaacgccca   96698

chimp.LOC112206744.LOC107973052.GGT2-.646279-708040.rev.compl     ------------------------------------------------------------   61762
FAM230B-LOC105372935-GGT2.NCBI.GGT2.rev.compl                     tgcctagccataactgagcgcatcacagactcacacaaatctgtggaatgagtgcatgat   96758

chimp.LOC112206744.LOC107973052.GGT2-.646279-708040.rev.compl     ------------------------------------------------------------   61762
FAM230B-LOC105372935-GGT2.NCBI.GGT2.rev.compl                     ctcatgagctctggtctggaaaagtaagtctccctacactaggacttaaagggaaggctt   96818

chimp.LOC112206744.LOC107973052.GGT2-.646279-708040.rev.compl     ------------------------------------------------------------   61762
FAM230B-LOC105372935-GGT2.NCBI.GGT2.rev.compl                     ctttggctatgccaatatctttcctgccactttcttcttcaggccaaacagctcctatcc   96878

chimp.LOC112206744.LOC107973052.GGT2-.646279-708040.rev.compl     ------------------------------------------------------------   61762
FAM230B-LOC105372935-GGT2.NCBI.GGT2.rev.compl                     ctgccttccatcttagctttccagcatactcttcccaggttctttgggctactgacaagc   96938

chimp.LOC112206744.LOC107973052.GGT2-.646279-708040.rev.compl     ------------------------------------------------------------   61762
FAM230B-LOC105372935-GGT2.NCBI.GGT2.rev.compl                     actccagacccaaatacacatgctcccacctgaggctgggaaatgtaccaagaactgcat   96998

chimp.LOC112206744.LOC107973052.GGT2-.646279-708040.rev.compl     ------------------------------------------------------------   61762
FAM230B-LOC105372935-GGT2.NCBI.GGT2.rev.compl                     agaaaatatactggacaaaaggcactccagtctcacagatctccagtcccacagtctata   97058

chimp.LOC112206744.LOC107973052.GGT2-.646279-708040.rev.compl     ------------------------------------------------------------   61762
FAM230B-LOC105372935-GGT2.NCBI.GGT2.rev.compl                     tcaccagttttagctgtttgaagttttagaattccttttcaaaaccatagctagcatttc   97118
```

```
chimp.LOC112206744.LOC107973052.GGT2-.646279-708040.rev.compl      ------------------------------------------------------------   61762
FAM230B-LOC105372935-GGT2.NCBI.GGT2.rev.compl                      tttggctagagaaaactgtgtgaaatgcagcctaccataggatcactgattacaacatgc   97178

chimp.LOC112206744.LOC107973052.GGT2-.646279-708040.rev.compl      ------------------------------------------------------------   61762
FAM230B-LOC105372935-GGT2.NCBI.GGT2.rev.compl                      attcaacattcaatacatattgactgagcaactattaggttgaactatgtgacattattg   97238

chimp.LOC112206744.LOC107973052.GGT2-.646279-708040.rev.compl      ------------------------------------------------------------   61762
FAM230B-LOC105372935-GGT2.NCBI.GGT2.rev.compl                      acattcgactgcttctgacctatcaaaatggcaataaagctcaacttaatatttactcta   97298

chimp.LOC112206744.LOC107973052.GGT2-.646279-708040.rev.compl      ------------------------------------------------------------   61762
FAM230B-LOC105372935-GGT2.NCBI.GGT2.rev.compl                      tgccaggcagtaggctaaggaaggatttgggacaccatgacaaacaacacagaagtaacc   97358

chimp.LOC112206744.LOC107973052.GGT2-.646279-708040.rev.compl      ------------------------------------------------------------   61762
FAM230B-LOC105372935-GGT2.NCBI.GGT2.rev.compl                      cctatcttaatggaacttaaaatctagttgattaaaccattacttacaaaagtacaatct   97418

chimp.LOC112206744.LOC107973052.GGT2-.646279-708040.rev.compl      ------------------------------------------------------------   61762
FAM230B-LOC105372935-GGT2.NCBI.GGT2.rev.compl                      aacttcaggattgtaattaattataaatgcatttatttgtttactgcctgacttctttgc   97478

chimp.LOC112206744.LOC107973052.GGT2-.646279-708040.rev.compl      ------------------------------------------------------------   61762
FAM230B-LOC105372935-GGT2.NCBI.GGT2.rev.compl                      tagcctataaactacacaaagatgagggttatgtctcctccatttcacacactggcaaag   97538

chimp.LOC112206744.LOC107973052.GGT2-.646279-708040.rev.compl      ------------------------------------------------------------   61762
FAM230B-LOC105372935-GGT2.NCBI.GGT2.rev.compl                      catatagcagaatacatcatatatttgttaaatgtattgattagtgttacaacagacaat   97598

chimp.LOC112206744.LOC107973052.GGT2-.646279-708040.rev.compl      ------------------------------------------------------------   61762
FAM230B-LOC105372935-GGT2.NCBI.GGT2.rev.compl                      tgcatgtgctatggaagcgtttggcagagagatctaatctaatcttggaaggtgaggatc   97658

chimp.LOC112206744.LOC107973052.GGT2-.646279-708040.rev.compl      ------------------------------------------------------------   61762
FAM230B-LOC105372935-GGT2.NCBI.GGT2.rev.compl                      ctggccatcactaacttggaatttctgatactgatgatataaggttgagttatgtcttcg   97718

chimp.LOC112206744.LOC107973052.GGT2-.646279-708040.rev.compl      ------------------------------------------------------------   61762
FAM230B-LOC105372935-GGT2.NCBI.GGT2.rev.compl                      tgaaacaggtctctatacagcatataaacaactaccagcatgcacaggggttccgtaggg   97778

chimp.LOC112206744.LOC107973052.GGT2-.646279-708040.rev.compl      ------------------------------------------------------------   61762
FAM230B-LOC105372935-GGT2.NCBI.GGT2.rev.compl                      actatttaaaatacttaagggaacaaacggttttcaagctcagtctagtatttcagaaat   97838

chimp.LOC112206744.LOC107973052.GGT2-.646279-708040.rev.compl      ------------------------------------------------------------   61762
FAM230B-LOC105372935-GGT2.NCBI.GGT2.rev.compl                      tccatttacataaacaattggtgcactaattgcaaatcattcttatttctctcctaaagc   97898

chimp.LOC112206744.LOC107973052.GGT2-.646279-708040.rev.compl      ------------------------------------------------------------   61762
FAM230B-LOC105372935-GGT2.NCBI.GGT2.rev.compl                      aggatcccctaaggatctttattaggcattctattagcctagtttgagttacagtattta   97958

chimp.LOC112206744.LOC107973052.GGT2-.646279-708040.rev.compl      ------------------------------------------------------------   61762
FAM230B-LOC105372935-GGT2.NCBI.GGT2.rev.compl                      cttaaaagtaataacagtgaagttcttgaaaaatatttggtaattcagacattttactaa   98018

chimp.LOC112206744.LOC107973052.GGT2-.646279-708040.rev.compl      ------------------------------------------------------------   61762
FAM230B-LOC105372935-GGT2.NCBI.GGT2.rev.compl                      ttcaaactaatcttcctcattagtctacgttgttagaggtttaaccattgtattaagaga   98078

chimp.LOC112206744.LOC107973052.GGT2-.646279-708040.rev.compl      ------------------------------------------------------------   61762
FAM230B-LOC105372935-GGT2.NCBI.GGT2.rev.compl                      aagtatcaaaggcttcacttagagtaatagtcaccaaatttttttgcaagacaccttggt   98138

chimp.LOC112206744.LOC107973052.GGT2-.646279-708040.rev.compl      ------------------------------------------------------------   61762
FAM230B-LOC105372935-GGT2.NCBI.GGT2.rev.compl                      agcaggaacctgagctaacatcaggaggtaggtagaaatagaaaataaaggaatgacaat   98198

chimp.LOC112206744.LOC107973052.GGT2-.646279-708040.rev.compl      ------------------------------------------------------------   61762
FAM230B-LOC105372935-GGT2.NCBI.GGT2.rev.compl                      ttgaagagcaactgcaatccaatttagtccaacagatgacacacatatgaagtcacattc   98258

chimp.LOC112206744.LOC107973052.GGT2-.646279-708040.rev.compl      ------------------------------------------------------------   61762
FAM230B-LOC105372935-GGT2.NCBI.GGT2.rev.compl                      aaattataagcagagcacaaaaatatttagatccttatcttttgattaacctggtatca   98318

chimp.LOC112206744.LOC107973052.GGT2-.646279-708040.rev.compl      ------------------------------------------------------------   61762
FAM230B-LOC105372935-GGT2.NCBI.GGT2.rev.compl                      ttcagggaaaaatgtcagggaacaccactgaccagcagtcctctcttatctattttgcag   98378

chimp.LOC112206744.LOC107973052.GGT2-.646279-708040.rev.compl      ------------------------------------------------------------   61762
FAM230B-LOC105372935-GGT2.NCBI.GGT2.rev.compl                      atcttatctatttttctccctgagaaccagccctggacacaaactgcataaacatcaatg   98438

chimp.LOC112206744.LOC107973052.GGT2-.646279-708040.rev.compl      ------------------------------------------------------------   61762
FAM230B-LOC105372935-GGT2.NCBI.GGT2.rev.compl                      agcctagacagggtcttcatgtgtgtttggcctgccattgttctctgtaccagtacggag   98498

chimp.LOC112206744.LOC107973052.GGT2-.646279-708040.rev.compl      ------------------------------------------------------------   61762
FAM230B-LOC105372935-GGT2.NCBI.GGT2.rev.compl                      atttagtaacacactggtcacattcatggggcatcttgaactctcttgacatggtaaatg   98558

chimp.LOC112206744.LOC107973052.GGT2-.646279-708040.rev.compl      ------------------------------------------------------------   61762
FAM230B-LOC105372935-GGT2.NCBI.GGT2.rev.compl                      aacagcaggtcctgttcccatgccaaaagcaaacaaaaacctgtcaccagcaaggaagac   98618

chimp.LOC112206744.LOC107973052.GGT2-.646279-708040.rev.compl      ------------------------------------------------------------   61762
FAM230B-LOC105372935-GGT2.NCBI.GGT2.rev.compl                      acccagtctctccaaaggatgccaaagtccacatccagcctcctttgcagagcctcacaa   98678

chimp.LOC112206744.LOC107973052.GGT2-.646279-708040.rev.compl      ------------------------------------------------------------   61762
FAM230B-LOC105372935-GGT2.NCBI.GGT2.rev.compl                      atctctcggatgcaggcaggatgatcacgggctagggaagattatgctctgaatgtctta   98738

chimp.LOC112206744.LOC107973052.GGT2-.646279-708040.rev.compl      ------------------------------------------------------------   61762
FAM230B-LOC105372935-GGT2.NCBI.GGT2.rev.compl                      agagattagttacttaaaatcctccacatgacttcagtcatccccgttgcagatcacaga   98798

chimp.LOC112206744.LOC107973052.GGT2-.646279-708040.rev.compl      ------------------------------------------------------------   61762
FAM230B-LOC105372935-GGT2.NCBI.GGT2.rev.compl                      gtaaaaagcctacctgattaaagctgaatgcctatacatttgcaaaagggaaaatatgca   98858

chimp.LOC112206744.LOC107973052.GGT2-.646279-708040.rev.compl      ------------------------------------------------------------   61762
FAM230B-LOC105372935-GGT2.NCBI.GGT2.rev.compl                      gaagaaaacgagagtcagatactgatgggcatagcaccagctctaggacatcagaggtgt   98918

chimp.LOC112206744.LOC107973052.GGT2-.646279-708040.rev.compl      ------------------------------------------------------------   61762
FAM230B-LOC105372935-GGT2.NCBI.GGT2.rev.compl                      tgaataaatgatgacaaggaggcacacatagttctgatatattcaccataaataccacag   98978

chimp.LOC112206744.LOC107973052.GGT2-.646279-708040.rev.compl      ------------------------------------------------------------   61762
FAM230B-LOC105372935-GGT2.NCBI.GGT2.rev.compl                      tcctcaaggctctctcaattacttacttccccccaaatcagagactgaatctggccttat   99038

chimp.LOC112206744.LOC107973052.GGT2-.646279-708040.rev.compl      ------------------------------------------------------------   61762
FAM230B-LOC105372935-GGT2.NCBI.GGT2.rev.compl                      cttcaaatgaggcccagctctccccaaattccaaatatgggaaattacagctcctctgtg   99098

chimp.LOC112206744.LOC107973052.GGT2-.646279-708040.rev.compl      ------------------------------------------------------------   61762
FAM230B-LOC105372935-GGT2.NCBI.GGT2.rev.compl                      gtagggggaagagtgggaaggcgaatgtttgtattgcataacgttttaattaaaacaaag   99158

chimp.LOC112206744.LOC107973052.GGT2-.646279-708040.rev.compl      ------------------------------------------------------------   61762
FAM230B-LOC105372935-GGT2.NCBI.GGT2.rev.compl                      atgacgtgcaccagagtctctttctcatctcctgttaggggcgttttccagtactacttc   99218

chimp.LOC112206744.LOC107973052.GGT2-.646279-708040.rev.compl      ------------------------------------------------------------   61762
FAM230B-LOC105372935-GGT2.NCBI.GGT2.rev.compl                      caccctttacccacccaggtcaccatggtctttttcttccctctaggacaattcaccttt   99278

chimp.LOC112206744.LOC107973052.GGT2-.646279-708040.rev.compl      ------------------------------------------------------------   61762
```

```
FAM230B-LOC105372935-GGT2.NCBI.GGT2.rev.compl                  aaaataagaggattgggcaagaaatctacaaagatttttccaaaatgaactcaggcctag   99338

chimp.LOC112206744.LOC107973052.GGT2-.646279-708040.rev.compl  ------------------------------------------------------------   61762
FAM230B-LOC105372935-GGT2.NCBI.GGT2.rev.compl                  aggcatattagttctctatcttgttgctcccttctagaagtgcacagttcagagggatgc   99398

chimp.LOC112206744.LOC107973052.GGT2-.646279-708040.rev.compl  ------------------------------------------------------------   61762
FAM230B-LOC105372935-GGT2.NCBI.GGT2.rev.compl                  ccagccacagaggtgccaaaacgcagagctggaaccaatcacctgctccaacagagacaa   99458

chimp.LOC112206744.LOC107973052.GGT2-.646279-708040.rev.compl  ------------------------------------------------------------   61762
FAM230B-LOC105372935-GGT2.NCBI.GGT2.rev.compl                  ggtgaaagcactgggcagggtgctcagaatgtgctcttgcttctttgtaatgaaatatca   99518

chimp.LOC112206744.LOC107973052.GGT2-.646279-708040.rev.compl  ------------------------------------------------------------   61762
FAM230B-LOC105372935-GGT2.NCBI.GGT2.rev.compl                  gaagacagattgtaacattgttcagcaagctatttttttgtactgaaacatcagaaggcat   99578

chimp.LOC112206744.LOC107973052.GGT2-.646279-708040.rev.compl  ------------------------------------------------------------   61762
FAM230B-LOC105372935-GGT2.NCBI.GGT2.rev.compl                  tatgaaactgttcagcaaatcatctctaaacaatgtcataaagggttacctagtttgggg   99638

chimp.LOC112206744.LOC107973052.GGT2-.646279-708040.rev.compl  ------------------------------------------------------------   61762
FAM230B-LOC105372935-GGT2.NCBI.GGT2.rev.compl                  taagcccggaatgaatatctgcaccatcattcctcccgctaacaaacatgtccatctatg   99698

chimp.LOC112206744.LOC107973052.GGT2-.646279-708040.rev.compl  ------------------------------------------------------------   61762
FAM230B-LOC105372935-GGT2.NCBI.GGT2.rev.compl                  tgaagcataattcccaacacaccaccatctccaaaccaacaccaaaccactgccaaacaa   99758

chimp.LOC112206744.LOC107973052.GGT2-.646279-708040.rev.compl  ------------------------------------------------------------   61762
FAM230B-LOC105372935-GGT2.NCBI.GGT2.rev.compl                  agctgaagaggaaaaccagaaaggtctccgagaagtgctgcttgcagggagaagaatggc   99818

chimp.LOC112206744.LOC107973052.GGT2-.646279-708040.rev.compl  ------------------------------------------------------------   61762
FAM230B-LOC105372935-GGT2.NCBI.GGT2.rev.compl                  cctgcacagaggctcctgagggcagcaggtgcctgctttggagccttggcaatttccacc   99878

chimp.LOC112206744.LOC107973052.GGT2-.646279-708040.rev.compl  ------------------------------------------------------------   61762
FAM230B-LOC105372935-GGT2.NCBI.GGT2.rev.compl                  aggcagaatttacaaccgtctgggacctgcagtctcagagggctcacggagaagggggaa   99938

chimp.LOC112206744.LOC107973052.GGT2-.646279-708040.rev.compl  ------------------------------------------------------------   61762
FAM230B-LOC105372935-GGT2.NCBI.GGT2.rev.compl                  gctcttggctacccgggaggtcaccagtgactctgagcaaagtgcagatcaatgcctgct   99998

chimp.LOC112206744.LOC107973052.GGT2-.646279-708040.rev.compl  ------------------------------------------------------------   61762
FAM230B-LOC105372935-GGT2.NCBI.GGT2.rev.compl                  tagggttaccccaggctgggagagggcatctggcaaggaacagacctggaagagcctgc   100058

chimp.LOC112206744.LOC107973052.GGT2-.646279-708040.rev.compl  ------------------------------------------------------------   61762
FAM230B-LOC105372935-GGT2.NCBI.GGT2.rev.compl                  ccctctttgagctgctacagcagcttctgggaatttgtaagagccatactgatgagggtc   100118

chimp.LOC112206744.LOC107973052.GGT2-.646279-708040.rev.compl  ------------------------------------------------------------   61762
FAM230B-LOC105372935-GGT2.NCBI.GGT2.rev.compl                  ccacaccctcccttgccagggcgccctcagggaggccacactgcaggaggcagagtatga   100178

chimp.LOC112206744.LOC107973052.GGT2-.646279-708040.rev.compl  ------------------------------------------------------------   61762
FAM230B-LOC105372935-GGT2.NCBI.GGT2.rev.compl                  ggtggggaagatggatcggccttatcctcaccacccccatacgagtctcctctggtatg   100238

chimp.LOC112206744.LOC107973052.GGT2-.646279-708040.rev.compl  ------------------------------------------------------------   61762
FAM230B-LOC105372935-GGT2.NCBI.GGT2.rev.compl                  gatggtgtctcctctctggtgggaagtatgtgatttctctatttctatttctcttttgtt   100298

chimp.LOC112206744.LOC107973052.GGT2-.646279-708040.rev.compl  ------------------------------------------------------------   61762
FAM230B-LOC105372935-GGT2.NCBI.GGT2.rev.compl                  cgggcttcctagaaattcagagctaggtgtgaaattcccaattcccacagacaagcaaca   100358

chimp.LOC112206744.LOC107973052.GGT2-.646279-708040.rev.compl  ------------------------------------------------------------   61762
FAM230B-LOC105372935-GGT2.NCBI.GGT2.rev.compl                  tccccagcagtaataaggacgttccacacacccaggacttgggatatttgtgtattttca   100418

chimp.LOC112206744.LOC107973052.GGT2-.646279-708040.rev.compl  ------------------------------------------------------------   61762
FAM230B-LOC105372935-GGT2.NCBI.GGT2.rev.compl                  agaataaaagtttgccctttagcctcctcagagcccctcccttctcctcgctctcccct   100478

chimp.LOC112206744.LOC107973052.GGT2-.646279-708040.rev.compl  ------------------------------------------------------------   61762
FAM230B-LOC105372935-GGT2.NCBI.GGT2.rev.compl                  taccactgctattcatcatcttactcctacagatacccccctcttcgtggtggcaccgggc   100538

chimp.LOC112206744.LOC107973052.GGT2-.646279-708040.rev.compl  ------------------------------------------------------------   61762
FAM230B-LOC105372935-GGT2.NCBI.GGT2.rev.compl                  actagtaactcatggctgtaaagaagagctgagaacaaaacaggaagaaaagaggttaag   100598

chimp.LOC112206744.LOC107973052.GGT2-.646279-708040.rev.compl  ------------------------------------------------------------   61762
FAM230B-LOC105372935-GGT2.NCBI.GGT2.rev.compl                  taatggcaagaaaaaagatgtaccttaggaataaaaaagagttcccgaatgtcacagtgt   100658

chimp.LOC112206744.LOC107973052.GGT2-.646279-708040.rev.compl  ------------------------------------------------------------   61762
FAM230B-LOC105372935-GGT2.NCBI.GGT2.rev.compl                  atttaattaatgcttctatttattaaagattaaaaatacatatcaggtgacgacttaaaa   100718

chimp.LOC112206744.LOC107973052.GGT2-.646279-708040.rev.compl  ------------------------------------------------------------   61762
FAM230B-LOC105372935-GGT2.NCBI.GGT2.rev.compl                  tgttagtcatgattttaactgtgaattctgtggcaccggagataacagaacaatgagtgc   100778

chimp.LOC112206744.LOC107973052.GGT2-.646279-708040.rev.compl  ------------------------------------------------------------   61762
FAM230B-LOC105372935-GGT2.NCBI.GGT2.rev.compl                  ttgtagaaggcactcggttatttgttgaatgaacttcttgggaaagccttcttaggaaag   100838

chimp.LOC112206744.LOC107973052.GGT2-.646279-708040.rev.compl  ------------------------------------------------------------   61762
FAM230B-LOC105372935-GGT2.NCBI.GGT2.rev.compl                  tattagaagtagattctgctaaaacaggggctaccctcagaggtctcaggacaagggata   100898

chimp.LOC112206744.LOC107973052.GGT2-.646279-708040.rev.compl  ------------------------------------------------------------   61762
FAM230B-LOC105372935-GGT2.NCBI.GGT2.rev.compl                  gaaagtcaaacactgatgtttaagaagggatgcctatgacacatgaatcccacagccatc   100958

chimp.LOC112206744.LOC107973052.GGT2-.646279-708040.rev.compl  ------------------------------------------------------------   61762
FAM230B-LOC105372935-GGT2.NCBI.GGT2.rev.compl                  tctccagaagagcaactattttgactgcgcatgcgggaagatggcgggccgggcgacttg   101018

chimp.LOC112206744.LOC107973052.GGT2-.646279-708040.rev.compl  ------------------------------------------------------------   61762
FAM230B-LOC105372935-GGT2.NCBI.GGT2.rev.compl                  agatccgcgggtctccctgctccttttccgtctgcgtcgggagctcccgggcacgtgagg   101078

chimp.LOC112206744.LOC107973052.GGT2-.646279-708040.rev.compl  ------------------------------------------------------------   61762
FAM230B-LOC105372935-GGT2.NCBI.GGT2.rev.compl                  ccgtgccgcgtttactggcgggagggacggcctagccgggcgacgcctcggaggaggccg   101138

chimp.LOC112206744.LOC107973052.GGT2-.646279-708040.rev.compl  ------------------------------------------------------------   61762
FAM230B-LOC105372935-GGT2.NCBI.GGT2.rev.compl                  cggaccccttaggtgctgggcccttggaaatcggcgcgtgggggcggtgctcgagctga   101198

chimp.LOC112206744.LOC107973052.GGT2-.646279-708040.rev.compl  ------------------------------------------------------------   61762
FAM230B-LOC105372935-GGT2.NCBI.GGT2.rev.compl                  gcgcgagagggcgggagagctcgtggggtgcgaggggagcaggacgcccggccgggcagc   101258

chimp.LOC112206744.LOC107973052.GGT2-.646279-708040.rev.compl  ------------------------------------------------------------   61762
FAM230B-LOC105372935-GGT2.NCBI.GGT2.rev.compl                  atgagtcagcagcggccggcgaggagattacccagtctcctcctgcacccgacggaggag   101318

chimp.LOC112206744.LOC107973052.GGT2-.646279-708040.rev.compl  ------------------------------------------------------------   61762
FAM230B-LOC105372935-GGT2.NCBI.GGT2.rev.compl                  accccatcaacgtggagggcctgctgccatcaaaaataaggattaatttagaagataatg   101378

chimp.LOC112206744.LOC107973052.GGT2-.646279-708040.rev.compl  ------------------------------------------------------------   61762
FAM230B-LOC105372935-GGT2.NCBI.GGT2.rev.compl                  tacaatatgtgtccatgagaaatctgctcccgggggtattcttgacttaaacaaggttgc   101438

chimp.LOC112206744.LOC107973052.GGT2-.646279-708040.rev.compl  ------------------------------------------------------------   61762
FAM230B-LOC105372935-GGT2.NCBI.GGT2.rev.compl                  aacgaaactgggagtccgaaagcggagagtgtatgacatcaccgatgtcttagatggaat   101498
```

```
chimp.LOC112206744.LOC107973052.GGT2-.646279-708040.rev.compl    ------------------------------------------------------------    61762
FAM230B-LOC105372935-GGT2.NCBI.GGT2.rev.compl                    cgacctcgttgaaaagaaatccaagaaccatattagatggataggatctgatcttagcaa    101558

chimp.LOC112206744.LOC107973052.GGT2-.646279-708040.rev.compl    ------------------------------------------------------------    61762
FAM230B-LOC105372935-GGT2.NCBI.GGT2.rev.compl                    ttttggagcagttccccaacaaaagaagctacaggaggaactttctgacttaccagcaat    101618

chimp.LOC112206744.LOC107973052.GGT2-.646279-708040.rev.compl    ------------------------------------------------------------    61762
FAM230B-LOC105372935-GGT2.NCBI.GGT2.rev.compl                    ggaagatgctttggatgagttaattaaggattgtgctcagcagctgtttgagttaacaga    101678

chimp.LOC112206744.LOC107973052.GGT2-.646279-708040.rev.compl    ------------------------------------------------------------    61762
FAM230B-LOC105372935-GGT2.NCBI.GGT2.rev.compl                    tgacaaagaaaatgaaagactagcatatgtgacctatcaagacattcatagcattcaggc    101738

chimp.LOC112206744.LOC107973052.GGT2-.646279-708040.rev.compl    ------------------------------------------------------------    61762
FAM230B-LOC105372935-GGT2.NCBI.GGT2.rev.compl                    cttccatgaacagatcgtcattgcagttaaagctccagcagaaaccagattggatgttcc    101798

chimp.LOC112206744.LOC107973052.GGT2-.646279-708040.rev.compl    ------------------------------------------------------------    61762
FAM230B-LOC105372935-GGT2.NCBI.GGT2.rev.compl                    agctcccagagaagactctatcacagtgcacataaggagcaccaacggacctatcgatgt    101858

chimp.LOC112206744.LOC107973052.GGT2-.646279-708040.rev.compl    ------------------------------------------------------------    61762
FAM230B-LOC105372935-GGT2.NCBI.GGT2.rev.compl                    ctatttgtgagaagtggagcagggtcagaccagtaacaaaaggtctgaaggtgtcaggac    101918

chimp.LOC112206744.LOC107973052.GGT2-.646279-708040.rev.compl    ------------------------------------------------------------    61762
FAM230B-LOC105372935-GGT2.NCBI.GGT2.rev.compl                    ctcttcatctgagagcactcatccagaaggccctgaggaagaagaaaatcctcagcaaag    101978

chimp.LOC112206744.LOC107973052.GGT2-.646279-708040.rev.compl    ------------------------------------------------------------    61762
FAM230B-LOC105372935-GGT2.NCBI.GGT2.rev.compl                    tgaagaattgcttgaagtaagcaactgatggcatttgagaatttatgtatcactgagttt    102038

chimp.LOC112206744.LOC107973052.GGT2-.646279-708040.rev.compl    ------------------------------------------------------------    61762
FAM230B-LOC105372935-GGT2.NCBI.GGT2.rev.compl                    tttgggaatatcttcctggagaattacgcatcaaatttgattctcagagcaataaattat    102098

chimp.LOC112206744.LOC107973052.GGT2-.646279-708040.rev.compl    ------------------------------------------------------------    61762
FAM230B-LOC105372935-GGT2.NCBI.GGT2.rev.compl                    ccatgaagtgctctcgttctcagtagcggcatcatggccagtagtgtctttgaggagttc    102158

chimp.LOC112206744.LOC107973052.GGT2-.646279-708040.rev.compl    ------------------------------------------------------------    61762
FAM230B-LOC105372935-GGT2.NCBI.GGT2.rev.compl                    accacttagattactgagtaattgtggtttccacatttgaaaacaactccttttataatt    102218

chimp.LOC112206744.LOC107973052.GGT2-.646279-708040.rev.compl    ------------------------------------------------------------    61762
FAM230B-LOC105372935-GGT2.NCBI.GGT2.rev.compl                    attcactgctttttgtcagtgaaatagacatcttgcctcctgaagtagcttcatcacaga    102278

chimp.LOC112206744.LOC107973052.GGT2-.646279-708040.rev.compl    ------------------------------------------------------------    61762
FAM230B-LOC105372935-GGT2.NCBI.GGT2.rev.compl                    gtgtcatgaagacagacagtcaggctgaaaaggacagttctttgtggactctacccttcc    102338

chimp.LOC112206744.LOC107973052.GGT2-.646279-708040.rev.compl    ------------------------------------------------------------    61762
FAM230B-LOC105372935-GGT2.NCBI.GGT2.rev.compl                    cttcaaggagtatgtcatatgtcacaaaagaaattgccttacactggttcatgtttgcag    102398

chimp.LOC112206744.LOC107973052.GGT2-.646279-708040.rev.compl    ------------------------------------------------------------    61762
FAM230B-LOC105372935-GGT2.NCBI.GGT2.rev.compl                    ttactgttgtacattgcatagatgtacacacgaatttaaatgtgatgtctttgtatatat    102458

chimp.LOC112206744.LOC107973052.GGT2-.646279-708040.rev.compl    ------------------------------------------------------------    61762
FAM230B-LOC105372935-GGT2.NCBI.GGT2.rev.compl                    ctgtataatgttgagattacttacgaaatatgtctgagtgacacttttcactctcgtaca    102518

chimp.LOC112206744.LOC107973052.GGT2-.646279-708040.rev.compl    ------------------------------------------------------------    61762
FAM230B-LOC105372935-GGT2.NCBI.GGT2.rev.compl                    gccaaaataatgtatatatggaaagtgacagacaaattctctaatctctttggtatctat    102578

chimp.LOC112206744.LOC107973052.GGT2-.646279-708040.rev.compl    ------------------------------------------------------------    61762
FAM230B-LOC105372935-GGT2.NCBI.GGT2.rev.compl                    aacttattagaatcctctggatgagggttagaagagactttttccaaacttctacatgta    102638

chimp.LOC112206744.LOC107973052.GGT2-.646279-708040.rev.compl    ------------------------------------------------------------    61762
FAM230B-LOC105372935-GGT2.NCBI.GGT2.rev.compl                    gaagtatcataaatgtgctacacatttatgtttgtggatttaattaaagtattttaatat    102698

chimp.LOC112206744.LOC107973052.GGT2-.646279-708040.rev.compl    ------------------------------------------------------------    61762
FAM230B-LOC105372935-GGT2.NCBI.GGT2.rev.compl                    ggttttcagtgctaaaattggagtcagatacttcttggttttaagctgtctacctaattg    102758

chimp.LOC112206744.LOC107973052.GGT2-.646279-708040.rev.compl    ------------------------------------------------------------    61762
FAM230B-LOC105372935-GGT2.NCBI.GGT2.rev.compl                    ctgtctcccagcagatcggtggcatgcccagtggcttggggggcaaggatagaaatgtca    102818

chimp.LOC112206744.LOC107973052.GGT2-.646279-708040.rev.compl    ------------------------------------------------------------    61762
FAM230B-LOC105372935-GGT2.NCBI.GGT2.rev.compl                    tcaggaaatagctgaattcattgtgaaacatgaattcagtcatggtgataattggaaact    102878

chimp.LOC112206744.LOC107973052.GGT2-.646279-708040.rev.compl    ------------------------------------------------------------    61762
FAM230B-LOC105372935-GGT2.NCBI.GGT2.rev.compl                    cctttcaggtttttgcaagtagattttgtaatgtttgtgtatgcagccttgctgttgagt    102938

chimp.LOC112206744.LOC107973052.GGT2-.646279-708040.rev.compl    ------------------------------------------------------------    61762
FAM230B-LOC105372935-GGT2.NCBI.GGT2.rev.compl                    cagtccaaggggttttacttaggacaagttgtaccttgccctctctccagctctgctccc    102998

chimp.LOC112206744.LOC107973052.GGT2-.646279-708040.rev.compl    ------------------------------------------------------------    61762
FAM230B-LOC105372935-GGT2.NCBI.GGT2.rev.compl                    acattttcacatacctagctatttctacctcattgggtaagtcatttaccactctgtgcc    103058

chimp.LOC112206744.LOC107973052.GGT2-.646279-708040.rev.compl    ------------------------------------------------------------    61762
FAM230B-LOC105372935-GGT2.NCBI.GGT2.rev.compl                    tcagtttactctgtagtttaccattagactgtgagctccctgagggactttgtcataatc    103118

chimp.LOC112206744.LOC107973052.GGT2-.646279-708040.rev.compl    ------------------------------------------------------------    61762
FAM230B-LOC105372935-GGT2.NCBI.GGT2.rev.compl                    actgttacatcccagtgcctcacaccatgcctggcccttaagaagtgctcaataaatgtc    103178

chimp.LOC112206744.LOC107973052.GGT2-.646279-708040.rev.compl    ------------------------------------------------------------    61762
FAM230B-LOC105372935-GGT2.NCBI.GGT2.rev.compl                    tgaacaaaaaaaaaaaaaaaaaaaaaaaaaatcaggtctaaagatgagccatccaagaaa    103238

chimp.LOC112206744.LOC107973052.GGT2-.646279-708040.rev.compl    ------------------------------------------------------------    61762
FAM230B-LOC105372935-GGT2.NCBI.GGT2.rev.compl                    ctgctcttgctcccagaggttttaaagaaagtcattcccatgaacgtgagaagtaaccca    103298

chimp.LOC112206744.LOC107973052.GGT2-.646279-708040.rev.compl    ------------------------------------------------------------    61762
FAM230B-LOC105372935-GGT2.NCBI.GGT2.rev.compl                    acactttgaaatgataatctttttacagcaataactgttgctgcagggcttgagaaaaaa    103358

chimp.LOC112206744.LOC107973052.GGT2-.646279-708040.rev.compl    ------------------------------------------------------------    61762
FAM230B-LOC105372935-GGT2.NCBI.GGT2.rev.compl                    agttctcttcctttgggttaaagtcttaaatgctgagcccgtagccagcaaccttaaaga    103418

chimp.LOC112206744.LOC107973052.GGT2-.646279-708040.rev.compl    ------------------------------------------------------------    61762
FAM230B-LOC105372935-GGT2.NCBI.GGT2.rev.compl                    ctactcgggtccagttcattctatctggaggagtcacttgggaattgaaaacacaggaaa    103478

chimp.LOC112206744.LOC107973052.GGT2-.646279-708040.rev.compl    ------------------------------------------------------------    61762
FAM230B-LOC105372935-GGT2.NCBI.GGT2.rev.compl                    acttttttctggtccatgaacaaacaagaacgcttaactgaattagacacaaaactaag    103538

chimp.LOC112206744.LOC107973052.GGT2-.646279-708040.rev.compl    ------------------------------------------------------------    61762
FAM230B-LOC105372935-GGT2.NCBI.GGT2.rev.compl                    tatttatggttgtcatcttaacaggtttcaaacaggcttcattttaatttaacataattt    103598

chimp.LOC112206744.LOC107973052.GGT2-.646279-708040.rev.compl    ------------------------------------------------------------    61762
FAM230B-LOC105372935-GGT2.NCBI.GGT2.rev.compl                    caaaattatagcaaaatagtcaataaaatacgaacgtgcttaatttgatgaatttatgtg    103658
```

```
chimp.LOC112206744.LOC107973052.GGT2-.646279-708040.rev.compl      ------------------------------------------------------------   61762
FAM230B-LOC105372935-GGT2.NCBI.GGT2.rev.compl                      tttttgaagaaaatatgcagaataaacaagtcaattattctcactaaatacattaaaat   103718

chimp.LOC112206744.LOC107973052.GGT2-.646279-708040.rev.compl      ------------------------------------------------------------   61762
FAM230B-LOC105372935-GGT2.NCBI.GGT2.rev.compl                      ttaagggtaatatttttattatgacttgctctcctgatatagcaaaatttattgttatat   103778

chimp.LOC112206744.LOC107973052.GGT2-.646279-708040.rev.compl      ------------------------------------------------------------   61762
FAM230B-LOC105372935-GGT2.NCBI.GGT2.rev.compl                      agttatggaaggaggctcactactccccaatgatttcacaaattacagcttcagccagct   103838

chimp.LOC112206744.LOC107973052.GGT2-.646279-708040.rev.compl      ------------------------------------------------------------   61762
FAM230B-LOC105372935-GGT2.NCBI.GGT2.rev.compl                      ataaaataaccaaatcagttctgatatagccataaaattaagcctgaaaaaatagtgaag   103898

chimp.LOC112206744.LOC107973052.GGT2-.646279-708040.rev.compl      ------------------------------------------------------------   61762
FAM230B-LOC105372935-GGT2.NCBI.GGT2.rev.compl                      taaatcatccctttaaaatcaagttgttgaacaatatgttctgcatgattccatttatgt   103958

chimp.LOC112206744.LOC107973052.GGT2-.646279-708040.rev.compl      ------------------------------------------------------------   61762
FAM230B-LOC105372935-GGT2.NCBI.GGT2.rev.compl                      cagaaaaaatatatttgtgtaagtatatacacagaaaagggtctgggagaatatatacca   104018

chimp.LOC112206744.LOC107973052.GGT2-.646279-708040.rev.compl      ------------------------------------------------------------   61762
FAM230B-LOC105372935-GGT2.NCBI.GGT2.rev.compl                      aactataaagaatctatatcaaagtcaataaggggcatagacaaggaggggaactttcac   104078

chimp.LOC112206744.LOC107973052.GGT2-.646279-708040.rev.compl      ------------------------------------------------------------   61762
FAM230B-LOC105372935-GGT2.NCBI.GGT2.rev.compl                      attttactttccacattcttttttttttttttttgacggagtctcactctgttgcccagg   104138

chimp.LOC112206744.LOC107973052.GGT2-.646279-708040.rev.compl      ------------------------------------------------------------   61762
FAM230B-LOC105372935-GGT2.NCBI.GGT2.rev.compl                      ctggagtgcagtggcgcaatcttggctcactgcaagctccgcctcccgggttcacaccat   104198

chimp.LOC112206744.LOC107973052.GGT2-.646279-708040.rev.compl      ------------------------------------------------------------   61762
FAM230B-LOC105372935-GGT2.NCBI.GGT2.rev.compl                      tctcctgcctcagcctcccgagtagctgggactacaggcacccgccaccacgcccagcta   104258

chimp.LOC112206744.LOC107973052.GGT2-.646279-708040.rev.compl      ------------------------------------------------------------   61762
FAM230B-LOC105372935-GGT2.NCBI.GGT2.rev.compl                      atttttttatatttggggtttcactgcgttagccaggatggtctcaatctcctgaccttg   104318

chimp.LOC112206744.LOC107973052.GGT2-.646279-708040.rev.compl      ------------------------------------------------------------   61762
FAM230B-LOC105372935-GGT2.NCBI.GGT2.rev.compl                      tgatctgcccacctcacccacctcagcctcccaaagtgctgggattacaggcgtgacttt   104378

chimp.LOC112206744.LOC107973052.GGT2-.646279-708040.rev.compl      ------------------------------------------------------------   61762
FAM230B-LOC105372935-GGT2.NCBI.GGT2.rev.compl                      ctacattcttgtaacagaattatagtcaccgttacctccatcaaaaaattaaaattcaaa   104438

chimp.LOC112206744.LOC107973052.GGT2-.646279-708040.rev.compl      ------------------------------------------------------------   61762
FAM230B-LOC105372935-GGT2.NCBI.GGT2.rev.compl                      aattaggccaggcacggtggctcacgcctgtaatcccagcactttgggagtctggagcag   104498

chimp.LOC112206744.LOC107973052.GGT2-.646279-708040.rev.compl      ------------------------------------------------------------   61762
FAM230B-LOC105372935-GGT2.NCBI.GGT2.rev.compl                      cagatcacctgaggtcaggagttggagagcagcctgactggccaacatggtgaaacccca   104558

chimp.LOC112206744.LOC107973052.GGT2-.646279-708040.rev.compl      ------------------------------------------------------------   61762
FAM230B-LOC105372935-GGT2.NCBI.GGT2.rev.compl                      tctctactaaaaatacaaaaattagccgggcgtggtggagggcacctgtaatcccagcta   104618

chimp.LOC112206744.LOC107973052.GGT2-.646279-708040.rev.compl      ------------------------------------------------------------   61762
FAM230B-LOC105372935-GGT2.NCBI.GGT2.rev.compl                      cttgggaggctaaggcaggagaatcacttaaacccgggaggcggaggttgcagtgagcca   104678

chimp.LOC112206744.LOC107973052.GGT2-.646279-708040.rev.compl      ------------------------------------------------------------   61762
FAM230B-LOC105372935-GGT2.NCBI.GGT2.rev.compl                      agattgtgccactgcactccggcccgggtaacagagcgagactccatctcaaaaatgaat   104738

chimp.LOC112206744.LOC107973052.GGT2-.646279-708040.rev.compl      ------------------------------------------------------------   61762
FAM230B-LOC105372935-GGT2.NCBI.GGT2.rev.compl                      gaattaattaattaattaaagcaaacaaatataagctacctcctgaagtagaaaccagta   104798

chimp.LOC112206744.LOC107973052.GGT2-.646279-708040.rev.compl      ------------------------------------------------------------   61762
FAM230B-LOC105372935-GGT2.NCBI.GGT2.rev.compl                      tctcttcaccttacctttacataatactttaccctaatgcattttatctgctgttctaaa   104858

chimp.LOC112206744.LOC107973052.GGT2-.646279-708040.rev.compl      ------------------------------------------------------------   61762
FAM230B-LOC105372935-GGT2.NCBI.GGT2.rev.compl                      tcatgacttacttcaaatcaaatcttaagtgaaaatcaagatttccttttggggcatta   104918

chimp.LOC112206744.LOC107973052.GGT2-.646279-708040.rev.compl      ------------------------------------------------------------   61762
FAM230B-LOC105372935-GGT2.NCBI.GGT2.rev.compl                      tacaaaaaagactgtaaaatttcattctctgaaagtctttttaaaaagggatagattgta   104978

chimp.LOC112206744.LOC107973052.GGT2-.646279-708040.rev.compl      ------------------------------------------------------------   61762
FAM230B-LOC105372935-GGT2.NCBI.GGT2.rev.compl                      tctggctgggatagggtaggtgtgatcggacccggaggaaatgacggctgctcaactgtg   105038

chimp.LOC112206744.LOC107973052.GGT2-.646279-708040.rev.compl      ------------------------------------------------------------   61762
FAM230B-LOC105372935-GGT2.NCBI.GGT2.rev.compl                      catctcctttcctcaccctgctgaaaagtgccacctagcatggtcaatcaaggtcaccaa   105098

chimp.LOC112206744.LOC107973052.GGT2-.646279-708040.rev.compl      ------------------------------------------------------------   61762
FAM230B-LOC105372935-GGT2.NCBI.GGT2.rev.compl                      tgcaaagcagtagtgggcaaacaccacccaggttatccaaagcagcacatggggttggga   105158

chimp.LOC112206744.LOC107973052.GGT2-.646279-708040.rev.compl      ------------------------------------------------------------   61762
FAM230B-LOC105372935-GGT2.NCBI.GGT2.rev.compl                      aactgatgaccttggagatttcaaaatcacctaacaataaccccaccttccctaaccaca   105218

chimp.LOC112206744.LOC107973052.GGT2-.646279-708040.rev.compl      ------------------------------------------------------------   61762
FAM230B-LOC105372935-GGT2.NCBI.GGT2.rev.compl                      gggctaagcaacctaatggatggaacatatgtgtgctttggtcatccgcaacccagacca   105278

chimp.LOC112206744.LOC107973052.GGT2-.646279-708040.rev.compl      ------------------------------------------------------------   61762
FAM230B-LOC105372935-GGT2.NCBI.GGT2.rev.compl                      ctagactaattttgctttagaactggactatatcaagtgtgaacgatcatacttgttatc   105338

chimp.LOC112206744.LOC107973052.GGT2-.646279-708040.rev.compl      ------------------------------------------------------------   61762
FAM230B-LOC105372935-GGT2.NCBI.GGT2.rev.compl                      atgtgggaaaaaaggtttaattactccctaattgtctgggaaacaaaagatttcaaaata   105398

chimp.LOC112206744.LOC107973052.GGT2-.646279-708040.rev.compl      ------------------------------------------------------------   61762
FAM230B-LOC105372935-GGT2.NCBI.GGT2.rev.compl                      tggagctcctgggggaaggagagttaataaaatgggtccttgagaagtgaagtgggaagc   105458

chimp.LOC112206744.LOC107973052.GGT2-.646279-708040.rev.compl      ------------------------------------------------------------   61762
FAM230B-LOC105372935-GGT2.NCBI.GGT2.rev.compl                      ctgcctcatgccatggcaccagcgatgccacaggtgtacccagtgccctgccgagagcag   105518

chimp.LOC112206744.LOC107973052.GGT2-.646279-708040.rev.compl      ------------------------------------------------------------   61762
FAM230B-LOC105372935-GGT2.NCBI.GGT2.rev.compl                      agacggtgttctcagagttgctatcaaaaaggaggcagcagactgcattctgaagcatca   105578

chimp.LOC112206744.LOC107973052.GGT2-.646279-708040.rev.compl      ------------------------------------------------------------   61762
FAM230B-LOC105372935-GGT2.NCBI.GGT2.rev.compl                      gacaatgggtcacatgcggaaacagaattaaatacacaaaaatagcaccaacttaaggca   105638

chimp.LOC112206744.LOC107973052.GGT2-.646279-708040.rev.compl      ------------------------------------------------------------   61762
FAM230B-LOC105372935-GGT2.NCBI.GGT2.rev.compl                      agcctgggcaaactgcactacatcaaagaaagaacacacaactggtatcagatttttttt   105698

chimp.LOC112206744.LOC107973052.GGT2-.646279-708040.rev.compl      ------------------------------------------------------------   61762
FAM230B-LOC105372935-GGT2.NCBI.GGT2.rev.compl                      tctgttttaataaagagaaggcctggacacctgaaagttgacgggaagcaggcatcacta   105758

chimp.LOC112206744.LOC107973052.GGT2-.646279-708040.rev.compl      ------------------------------------------------------------   61762
FAM230B-LOC105372935-GGT2.NCBI.GGT2.rev.compl                      atttctccgagttcacagttttgttagaatgtctttgctctgattatggttttatttatt   105818
```

```
chimp.LOC112206744.LOC107973052.GGT2-.646279-708040.rev.compl    ------------------------------------------------------------   61762
FAM230B-LOC105372935-GGT2.NCBI.GGT2.rev.compl                    tattttttgagatggagtttttttgttttgttttatttgcttttgttgccctggctgg   105878

chimp.LOC112206744.LOC107973052.GGT2-.646279-708040.rev.compl    ------------------------------------------------------------   61762
FAM230B-LOC105372935-GGT2.NCBI.GGT2.rev.compl                    agtgcagtggtgcaatctcggctcaccgcaaactccgcctcccaggttcaagagattctc   105938

chimp.LOC112206744.LOC107973052.GGT2-.646279-708040.rev.compl    ------------------------------------------------------------   61762
FAM230B-LOC105372935-GGT2.NCBI.GGT2.rev.compl                    ctgcctcagcctcccaagtagctaggattacaggcacccactaccacgtctggcttcttt   105998

chimp.LOC112206744.LOC107973052.GGT2-.646279-708040.rev.compl    ------------------------------------------------------------   61762
FAM230B-LOC105372935-GGT2.NCBI.GGT2.rev.compl                    gtgtttttagtagagatggggtttcaccatgttggtcaggctggtcttgaactcttgacc   106058

chimp.LOC112206744.LOC107973052.GGT2-.646279-708040.rev.compl    ------------------------------------------------------------   61762
FAM230B-LOC105372935-GGT2.NCBI.GGT2.rev.compl                    tcaagcgatccacccgcctcagcccccaaagtgctggaattacaggcatgagccaccac   106118

chimp.LOC112206744.LOC107973052.GGT2-.646279-708040.rev.compl    ------------------------------------------------------------   61762
FAM230B-LOC105372935-GGT2.NCBI.GGT2.rev.compl                    gcctggcctgattatgttttgatggatatacagaagctcttgctttggtacagaaacagg   106178

chimp.LOC112206744.LOC107973052.GGT2-.646279-708040.rev.compl    ------------------------------------------------------------   61762
FAM230B-LOC105372935-GGT2.NCBI.GGT2.rev.compl                    ccccgctttgcttcgacctctgggcccccaccttcagaaatgtctggtgccattcctgg   106238

chimp.LOC112206744.LOC107973052.GGT2-.646279-708040.rev.compl    ------------------------------------------------------------   61762
FAM230B-LOC105372935-GGT2.NCBI.GGT2.rev.compl                    ctcctcctctcagtggtcttccaggatatccactcacagctttctgatgtcacccacctg   106298

chimp.LOC112206744.LOC107973052.GGT2-.646279-708040.rev.compl    ------------------------------------------------------------   61762
FAM230B-LOC105372935-GGT2.NCBI.GGT2.rev.compl                    tctctagcccagatacccagtcctcatggcaagtgactgcagcaatttttacaaatctac   106358

chimp.LOC112206744.LOC107973052.GGT2-.646279-708040.rev.compl    ------------------------------------------------------------   61762
FAM230B-LOC105372935-GGT2.NCBI.GGT2.rev.compl                    gcccttagggttcccatcccaggacgttcaggtttctccacccctttagaagtgcccaga   106418

chimp.LOC112206744.LOC107973052.GGT2-.646279-708040.rev.compl    ------------------------------------------------------------   61762
FAM230B-LOC105372935-GGT2.NCBI.GGT2.rev.compl                    atatcccctaccccacgattccaacaggacgtcactctcctcttacttaagaaaaatgcc   106478

chimp.LOC112206744.LOC107973052.GGT2-.646279-708040.rev.compl    ------------------------------------------------------------   61762
FAM230B-LOC105372935-GGT2.NCBI.GGT2.rev.compl                    taaaattccagcccttccaaaaggcctttctagattgaccactcaggagttactctggcc   106538

chimp.LOC112206744.LOC107973052.GGT2-.646279-708040.rev.compl    ------------------------------------------------------------   61762
FAM230B-LOC105372935-GGT2.NCBI.GGT2.rev.compl                    tccccttcactcctgctagactgaaaacaaggacagacttgcctggcctctctcccccag   106598

chimp.LOC112206744.LOC107973052.GGT2-.646279-708040.rev.compl    ------------------------------------------------------------   61762
FAM230B-LOC105372935-GGT2.NCBI.GGT2.rev.compl                    tagacacctaccccccagtgaacacatcctgtcagtgtcctcccgtccatttgcaaattga   106658

chimp.LOC112206744.LOC107973052.GGT2-.646279-708040.rev.compl    ------------------------------------------------------------   61762
FAM230B-LOC105372935-GGT2.NCBI.GGT2.rev.compl                    ttgactgtttcattgctggccttggatgacctctcctagagggcagggacggtggtgttc   106718

chimp.LOC112206744.LOC107973052.GGT2-.646279-708040.rev.compl    ------------------------------------------------------------   61762
FAM230B-LOC105372935-GGT2.NCBI.GGT2.rev.compl                    ggcaaagccttggtcgtctggataatgggaaatggggtcttctggtgaatttaattacag   106778

chimp.LOC112206744.LOC107973052.GGT2-.646279-708040.rev.compl    ------------------------------------------------------------   61762
FAM230B-LOC105372935-GGT2.NCBI.GGT2.rev.compl                    ggttaggtggaaactttgcttcaactctcattattagaaagtctattgtaaagcttttta   106838

chimp.LOC112206744.LOC107973052.GGT2-.646279-708040.rev.compl    ------------------------------------------------------------   61762
FAM230B-LOC105372935-GGT2.NCBI.GGT2.rev.compl                    aaatacaactgaccactttccagcgagggtagagttaatatgccttaatctttgatttag   106898

chimp.LOC112206744.LOC107973052.GGT2-.646279-708040.rev.compl    ------------------------------------------------------------   61762
FAM230B-LOC105372935-GGT2.NCBI.GGT2.rev.compl                    gatcttgcagtgccccaggctcaccattctcttctgctactataattatagatgcggaag   106958

chimp.LOC112206744.LOC107973052.GGT2-.646279-708040.rev.compl    ------------------------------------------------------------   61762
FAM230B-LOC105372935-GGT2.NCBI.GGT2.rev.compl                    acatacagactgggctgacaactgcttggaagctaatttctagaataaacccttgctacc   107018

chimp.LOC112206744.LOC107973052.GGT2-.646279-708040.rev.compl    ------------------------------------------------------------   61762
FAM230B-LOC105372935-GGT2.NCBI.GGT2.rev.compl                    acatcttgtgattttttgcccttctgaaggtggagttccaaaataaaaacaaaatcccgct   107078

chimp.LOC112206744.LOC107973052.GGT2-.646279-708040.rev.compl    ------------------------------------------------------------   61762
FAM230B-LOC105372935-GGT2.NCBI.GGT2.rev.compl                    taactgagggttccagttgagggaggtctgctgccatggtctttgttaagtgcctgccac   107138

chimp.LOC112206744.LOC107973052.GGT2-.646279-708040.rev.compl    ------------------------------------------------------------   61762
FAM230B-LOC105372935-GGT2.NCBI.GGT2.rev.compl                    catggcccacaattagtcagttaataaagggttcttgatgagagaatttggcaacatgtg   107198

chimp.LOC112206744.LOC107973052.GGT2-.646279-708040.rev.compl    ------------------------------------------------------------   61762
FAM230B-LOC105372935-GGT2.NCBI.GGT2.rev.compl                    attttccacaaatataagcttgattttgatgcaaccactcatttctttgaaaatcagggg   107258

chimp.LOC112206744.LOC107973052.GGT2-.646279-708040.rev.compl    ------------------------------------------------------------   61762
FAM230B-LOC105372935-GGT2.NCBI.GGT2.rev.compl                    gcattcacacacaggtgttctagatttccttacagtaaaaccaacccttttacaaggaata   107318

chimp.LOC112206744.LOC107973052.GGT2-.646279-708040.rev.compl    ------------------------------------------------------------   61762
FAM230B-LOC105372935-GGT2.NCBI.GGT2.rev.compl                    gctgtgttcactttgaagcagtaaagaagcaggtgtccactcatggcaaatgaggaactc   107378

chimp.LOC112206744.LOC107973052.GGT2-.646279-708040.rev.compl    ------------------------------------------------------------   61762
FAM230B-LOC105372935-GGT2.NCBI.GGT2.rev.compl                    cctttttttttttttttttttttttttttttttgagacggggtctcgctctttttttgt   107438

chimp.LOC112206744.LOC107973052.GGT2-.646279-708040.rev.compl    ------------------------------------------------------------   61762
FAM230B-LOC105372935-GGT2.NCBI.GGT2.rev.compl                    gtgtgtgagatgggggccacccaggctagagtgcagtagtgcaattacagctcactgtag   107498

chimp.LOC112206744.LOC107973052.GGT2-.646279-708040.rev.compl    ------------------------------------------------------------   61762
FAM230B-LOC105372935-GGT2.NCBI.GGT2.rev.compl                    cctcaaccttctgggctcaaccagttcttcccacctcaacttctgaagcagctgagacta   107558

chimp.LOC112206744.LOC107973052.GGT2-.646279-708040.rev.compl    ------------------------------------------------------------   61762
FAM230B-LOC105372935-GGT2.NCBI.GGT2.rev.compl                    caggtgtgtaccaccatgcccaggtaatgcttttgatttttagtaagatgaggtctcgtt   107618

chimp.LOC112206744.LOC107973052.GGT2-.646279-708040.rev.compl    ------------------------------------------------------------   61762
FAM230B-LOC105372935-GGT2.NCBI.GGT2.rev.compl                    atattgcccaggctggtctcaaactcctgagctcaagtgatcctcctgcctcagcctccc   107678

chimp.LOC112206744.LOC107973052.GGT2-.646279-708040.rev.compl    ------------------------------------------------------------   61762
FAM230B-LOC105372935-GGT2.NCBI.GGT2.rev.compl                    aaagtgctaggattatcggcatgagccactatgcccagccagaagctaaaaatattaatc   107738

chimp.LOC112206744.LOC107973052.GGT2-.646279-708040.rev.compl    ------------------------------------------------------------   61762
FAM230B-LOC105372935-GGT2.NCBI.GGT2.rev.compl                    aaggagtcacaactaaggatgcactgcacacccgaagccagggaccacaagaggtcagca   107798

chimp.LOC112206744.LOC107973052.GGT2-.646279-708040.rev.compl    ------------------------------------------------------------   61762
FAM230B-LOC105372935-GGT2.NCBI.GGT2.rev.compl                    actgggactgcttcctccagttcccaggctggggctcagcagcagtggcctgggctctgg   107858

chimp.LOC112206744.LOC107973052.GGT2-.646279-708040.rev.compl    ------------------------------------------------------------   61762
FAM230B-LOC105372935-GGT2.NCBI.GGT2.rev.compl                    ggacttactttaagccgaaatcccaaactcattatcattcaaaccaattttctgtgtcca   107918

chimp.LOC112206744.LOC107973052.GGT2-.646279-708040.rev.compl    ------------------------------------------------------------   61762
FAM230B-LOC105372935-GGT2.NCBI.GGT2.rev.compl                    gcatcatcccacctaaactggtccttaaaaccactgacagtcatttctccaatctgtaaa   107978

chimp.LOC112206744.LOC107973052.GGT2-.646279-708040.rev.compl    ------------------------------------------------------------   61762
```

```
FAM230B-LOC105372935-GGT2.NCBI.GGT2.rev.compl                   aggcctgctgaggcctttgcattatcaggacttaactgggagtagaatgccctccccatc   108038

chimp.LOC112206744.LOC107973052.GGT2-.646279-708040.rev.compl   ------------------------------------------------------------   61762
FAM230B-LOC105372935-GGT2.NCBI.GGT2.rev.compl                   caaatcctgaccatttctgaacactcagcccatgtcctcttcctgagtgtcttccccaac   108098

chimp.LOC112206744.LOC107973052.GGT2-.646279-708040.rev.compl   ------------------------------------------------------------   61762
FAM230B-LOC105372935-GGT2.NCBI.GGT2.rev.compl                   aacaaccacctacactgaactttactttgatctctgcaggaaactgtagatagagatggg   108158

chimp.LOC112206744.LOC107973052.GGT2-.646279-708040.rev.compl   ------------------------------------------------------------   61762
FAM230B-LOC105372935-GGT2.NCBI.GGT2.rev.compl                   gtctcactatgttgcctagctggtcttgaactgctgagctcaagtgatctccctgcttct   108218

chimp.LOC112206744.LOC107973052.GGT2-.646279-708040.rev.compl   ------------------------------------------------------------   61762
FAM230B-LOC105372935-GGT2.NCBI.GGT2.rev.compl                   gcctcccaaagtgctaggattacagcagtgagccaccacacctggcctatgtatgctttt   108278

chimp.LOC112206744.LOC107973052.GGT2-.646279-708040.rev.compl   ------------------------------------------------------------   61762
FAM230B-LOC105372935-GGT2.NCBI.GGT2.rev.compl                   tttttcttttcttttttttttttttttgagacagagtcttgctctgtggcccagtccgc   108338

chimp.LOC112206744.LOC107973052.GGT2-.646279-708040.rev.compl   ------------------------------------------------------------   61762
FAM230B-LOC105372935-GGT2.NCBI.GGT2.rev.compl                   agtgcagtggtgcaatctcggctcatggcaacctccaggttcaaggcattttcctgcctc   108398

chimp.LOC112206744.LOC107973052.GGT2-.646279-708040.rev.compl   ------------------------------------------------------------   61762
FAM230B-LOC105372935-GGT2.NCBI.GGT2.rev.compl                   agccttccaaggagctgggattacaggcacgcaccatcacgcccagctaattggccaggc   108458

chimp.LOC112206744.LOC107973052.GGT2-.646279-708040.rev.compl   ------------------------------------------------------------   61762
FAM230B-LOC105372935-GGT2.NCBI.GGT2.rev.compl                   tggtctcaaactcctgacctcaggtgatccacccaccttggcctcccaaagtgctgggat   108518

chimp.LOC112206744.LOC107973052.GGT2-.646279-708040.rev.compl   ------------------------------------------------------------   61762
FAM230B-LOC105372935-GGT2.NCBI.GGT2.rev.compl                   tacaggtgtaagccaccacaccgggccacgtatgcattttaaaacgttatgctcagacgg   108578

chimp.LOC112206744.LOC107973052.GGT2-.646279-708040.rev.compl   ------------------------------------------------------------   61762
FAM230B-LOC105372935-GGT2.NCBI.GGT2.rev.compl                   ggtctataggtttcaccagactgccaaaaggatccatgacacaaaaaaagtactctcagt   108638

chimp.LOC112206744.LOC107973052.GGT2-.646279-708040.rev.compl   ------------------------------------------------------------   61762
FAM230B-LOC105372935-GGT2.NCBI.GGT2.rev.compl                   tgcatccttcggaacctttcaacacatacacatatacctcactcatccttttgtgaagag   108698

chimp.LOC112206744.LOC107973052.GGT2-.646279-708040.rev.compl   ------------------------------------------------------------   61762
FAM230B-LOC105372935-GGT2.NCBI.GGT2.rev.compl                   tgtctattaatcactagattaagaggatttggataaaatttataaatgtacaaactttgc   108758

chimp.LOC112206744.LOC107973052.GGT2-.646279-708040.rev.compl   ------------------------------------------------------------   61762
FAM230B-LOC105372935-GGT2.NCBI.GGT2.rev.compl                   taccaaggactgacacatttacttttctagctcatcagtttatgtcctagaaaaattcca   108818

chimp.LOC112206744.LOC107973052.GGT2-.646279-708040.rev.compl   ------------------------------------------------------------   61762
FAM230B-LOC105372935-GGT2.NCBI.GGT2.rev.compl                   gagccacattaaccagctgtttcctatttttcattttacgatggtgtttccactttcacc   108878

chimp.LOC112206744.LOC107973052.GGT2-.646279-708040.rev.compl   ------------------------------------------------------------   61762
FAM230B-LOC105372935-GGT2.NCBI.GGT2.rev.compl                   tgcaaagtgactaatagtaatacctattacagtaatttaagtaacaccatttagagtaat   108938

chimp.LOC112206744.LOC107973052.GGT2-.646279-708040.rev.compl   ------------------------------------------------------------   61762
FAM230B-LOC105372935-GGT2.NCBI.GGT2.rev.compl                   ttcatctttagtactgtatttatactactaagaaagccgccaacgattcaagctgcactc   108998

chimp.LOC112206744.LOC107973052.GGT2-.646279-708040.rev.compl   ------------------------------------------------------------   61762
FAM230B-LOC105372935-GGT2.NCBI.GGT2.rev.compl                   actggtgaaaccctatgctatatatgtaattaatttatttcctaaattcttacctccctc   109058

chimp.LOC112206744.LOC107973052.GGT2-.646279-708040.rev.compl   ------------------------------------------------------------   61762
FAM230B-LOC105372935-GGT2.NCBI.GGT2.rev.compl                   cacccccctccattggaaaataaactctaagaatgcaaaagtttttgtgtgtttttcttca   109118

chimp.LOC112206744.LOC107973052.GGT2-.646279-708040.rev.compl   ------------------------------------------------------------   61762
FAM230B-LOC105372935-GGT2.NCBI.GGT2.rev.compl                   acgcttagagcagtgcctggcacctaacagtcaatatctgctaactttgatgaatgaatg   109178

chimp.LOC112206744.LOC107973052.GGT2-.646279-708040.rev.compl   ------------------------------------------------------------   61762
FAM230B-LOC105372935-GGT2.NCBI.GGT2.rev.compl                   gtatatttatattatgcaatattactatgcagccatgaaacacaatgagttagagtggta   109238

chimp.LOC112206744.LOC107973052.GGT2-.646279-708040.rev.compl   ------------------------------------------------------------   61762
FAM230B-LOC105372935-GGT2.NCBI.GGT2.rev.compl                   ccagctaatttaatggaatttccagaagtatgaaattaagaaaacaagaagaaaagtgtc   109298

chimp.LOC112206744.LOC107973052.GGT2-.646279-708040.rev.compl   ------------------------------------------------------------   61762
FAM230B-LOC105372935-GGT2.NCBI.GGT2.rev.compl                   aaaaatatcatcccactttttttttttcttccctgaaacggagtcgtgctctgacaccca   109358

chimp.LOC112206744.LOC107973052.GGT2-.646279-708040.rev.compl   ------------------------------------------------------------   61762
FAM230B-LOC105372935-GGT2.NCBI.GGT2.rev.compl                   ggctacagtatagtgctgccatcatggctcactgcaacctcaaccttctgggctcaggga   109418

chimp.LOC112206744.LOC107973052.GGT2-.646279-708040.rev.compl   ------------------------------------------------------------   61762
FAM230B-LOC105372935-GGT2.NCBI.GGT2.rev.compl                   atccttccacctcagcctcccatgtagctgggactacaggtgtgtgccacaacgcccagc   109478

chimp.LOC112206744.LOC107973052.GGT2-.646279-708040.rev.compl   ------------------------------------------------------------   61762
FAM230B-LOC105372935-GGT2.NCBI.GGT2.rev.compl                   taatttattttaaattatttttttgtagagacagggtctccctatgttgcccaggctggtc   109538

chimp.LOC112206744.LOC107973052.GGT2-.646279-708040.rev.compl   ------------------------------------------------------------   61762
FAM230B-LOC105372935-GGT2.NCBI.GGT2.rev.compl                   tcaaactactaggctcaagtgatcctcccacctcagcttcccaaagtgttgggattacag   109598

chimp.LOC112206744.LOC107973052.GGT2-.646279-708040.rev.compl   ------------------------------------------------------------   61762
FAM230B-LOC105372935-GGT2.NCBI.GGT2.rev.compl                   gtgtgagccactgtgcccagcccaatcccactttttaaaaagtatacccccaaatccata   109658

chimp.LOC112206744.LOC107973052.GGT2-.646279-708040.rev.compl   ------------------------------------------------------------   61762
FAM230B-LOC105372935-GGT2.NCBI.GGT2.rev.compl                   cactggtgaggaagtggagaacgtggaatcctcacacacagaatgtaaaatggtacagct   109718

chimp.LOC112206744.LOC107973052.GGT2-.646279-708040.rev.compl   ------------------------------------------------------------   61762
FAM230B-LOC105372935-GGT2.NCBI.GGT2.rev.compl                   ctggagaacattttagcagtttctcaaaatgtttaacgtagctactacacgacccagcaa   109778

chimp.LOC112206744.LOC107973052.GGT2-.646279-708040.rev.compl   ------------------------------------------------------------   61762
FAM230B-LOC105372935-GGT2.NCBI.GGT2.rev.compl                   ttcaactcccagatatacacctgagagaactgaaaggatatgtccacacaaaaatttgca   109838

chimp.LOC112206744.LOC107973052.GGT2-.646279-708040.rev.compl   ------------------------------------------------------------   61762
FAM230B-LOC105372935-GGT2.NCBI.GGT2.rev.compl                   gaggaacttgtacacacttgttcacagtagcattattcataatagcccaaagtggcaaaa   109898

chimp.LOC112206744.LOC107973052.GGT2-.646279-708040.rev.compl   ------------------------------------------------------------   61762
FAM230B-LOC105372935-GGT2.NCBI.GGT2.rev.compl                   acgcaaatgttcatcaactgatggacaaataaagcatgaacaactgatggacaaataaag   109958

chimp.LOC112206744.LOC107973052.GGT2-.646279-708040.rev.compl   ------------------------------------------------------------   61762
FAM230B-LOC105372935-GGT2.NCBI.GGT2.rev.compl                   tatatcattcagcatataagaaatgaagtgctaatacatgctacaacaggaatagacctt   110018

chimp.LOC112206744.LOC107973052.GGT2-.646279-708040.rev.compl   ------------------------------------------------------------   61762
FAM230B-LOC105372935-GGT2.NCBI.GGT2.rev.compl                   aataacattttgctaaatgaaaaaagccaaacaaaaggtcacatgttgtatgattctatt   110078

chimp.LOC112206744.LOC107973052.GGT2-.646279-708040.rev.compl   ------------------------------------------------------------   61762
FAM230B-LOC105372935-GGT2.NCBI.GGT2.rev.compl                   tacataaaaatacctagactaggcaaatccatagagacacaaagatcagtgactgcccag   110138

chimp.LOC112206744.LOC107973052.GGT2-.646279-708040.rev.compl   ------------------------------------------------------------   61762
FAM230B-LOC105372935-GGT2.NCBI.GGT2.rev.compl                   agccagagggagtggtgaatgtgaaatggctgctaatgagtacagacttctttttgaggt   110198
```

```
chimp.LOC112206744.LOC107973052.GGT2-.646279-708040.rev.compl      ------------------------------------------------------------   61762
FAM230B-LOC105372935-GGT2.NCBI.GGT2.rev.compl                      attgaacatgttatggaatcagatggtggtgatcaatgcataactttgtgaatataccaa   110258

chimp.LOC112206744.LOC107973052.GGT2-.646279-708040.rev.compl      ------------------------------------------------------------   61762
FAM230B-LOC105372935-GGT2.NCBI.GGT2.rev.compl                      aaaccacatgaaaggtacactttaaaagcctgaattttaaggtatgtgaattgtatctcc   110318

chimp.LOC112206744.LOC107973052.GGT2-.646279-708040.rev.compl      ------------------------------------------------------------   61762
FAM230B-LOC105372935-GGT2.NCBI.GGT2.rev.compl                      ataaacctgtgattttgttaatatacgcttatttgcaaatgaatacatggataatgagga   110378

chimp.LOC112206744.LOC107973052.GGT2-.646279-708040.rev.compl      ------------------------------------------------------------   61762
FAM230B-LOC105372935-GGT2.NCBI.GGT2.rev.compl                      ataatatgaaaggttaatgacaaccttggtgatagggtacaggagactgggagaggaaga   110438

chimp.LOC112206744.LOC107973052.GGT2-.646279-708040.rev.compl      ------------------------------------------------------------   61762
FAM230B-LOC105372935-GGT2.NCBI.GGT2.rev.compl                      taagtgttgaggtagaagaagacaaaaaataaaaggctaaaacaataatccagggccaga   110498

chimp.LOC112206744.LOC107973052.GGT2-.646279-708040.rev.compl      ------------------------------------------------------------   61762
FAM230B-LOC105372935-GGT2.NCBI.GGT2.rev.compl                      ggccatgagttgggagatggagcaagacgaagtgtacaaagaaatgagcataaatcataa   110558

chimp.LOC112206744.LOC107973052.GGT2-.646279-708040.rev.compl      ------------------------------------------------------------   61762
FAM230B-LOC105372935-GGT2.NCBI.GGT2.rev.compl                      aaatgagggaactagggttgaaaactccagcaagtagtttgtaatgggggagatgaagca   110618

chimp.LOC112206744.LOC107973052.GGT2-.646279-708040.rev.compl      ------------------------------------------------------------   61762
FAM230B-LOC105372935-GGT2.NCBI.GGT2.rev.compl                      aacctaagtaactgcaatgattcaacggaatttgcagaaaatgcagaagggcgagaaaag   110678

chimp.LOC112206744.LOC107973052.GGT2-.646279-708040.rev.compl      ------------------------------------------------------------   61762
FAM230B-LOC105372935-GGT2.NCBI.GGT2.rev.compl                      caatgaaaaaatggatcagaagatttaaaattggctctaccccaccaaagttttaaaaag   110738

chimp.LOC112206744.LOC107973052.GGT2-.646279-708040.rev.compl      ------------------------------------------------------------   61762
FAM230B-LOC105372935-GGT2.NCBI.GGT2.rev.compl                      caagcaagaagaaaattcagttgctcctgggcaagaattcttgggcccagtggggtagaa   110798

chimp.LOC112206744.LOC107973052.GGT2-.646279-708040.rev.compl      ------------------------------------------------------------   61762
FAM230B-LOC105372935-GGT2.NCBI.GGT2.rev.compl                      ccaaaccatgataaggagacaccagtgttttgtaagcaagagaatatattaaggagagaa   110858

chimp.LOC112206744.LOC107973052.GGT2-.646279-708040.rev.compl      ------------------------------------------------------------   61762
FAM230B-LOC105372935-GGT2.NCBI.GGT2.rev.compl                      aaggggcatttattgggagaaaaggtctcactgtgattgttctggaataaagtagcccca   110918

chimp.LOC112206744.LOC107973052.GGT2-.646279-708040.rev.compl      ------------------------------------------------------------   61762
FAM230B-LOC105372935-GGT2.NCBI.GGT2.rev.compl                      tcggactgcctcaggaactcatggtggaagaggtgccactggaaaatggctaagtcggga   110978

chimp.LOC112206744.LOC107973052.GGT2-.646279-708040.rev.compl      ------------------------------------------------------------   61762
FAM230B-LOC105372935-GGT2.NCBI.GGT2.rev.compl                      tcttccccaagaacactagaagagacagaggaagaatagaagagctttaaattttagcag   111038

chimp.LOC112206744.LOC107973052.GGT2-.646279-708040.rev.compl      ------------------------------------------------------------   61762
FAM230B-LOC105372935-GGT2.NCBI.GGT2.rev.compl                      atatttctttttcttgtttaattaaaataaatcttattgacctaatatttttgagactgg   111098

chimp.LOC112206744.LOC107973052.GGT2-.646279-708040.rev.compl      ------------------------------------------------------------   61762
FAM230B-LOC105372935-GGT2.NCBI.GGT2.rev.compl                      gctcttgcaatgctccccagactgaactcaaactcctaggctcaagagattcttccactt   111158

chimp.LOC112206744.LOC107973052.GGT2-.646279-708040.rev.compl      ------------------------------------------------------------   61762
FAM230B-LOC105372935-GGT2.NCBI.GGT2.rev.compl                      cagcctcctgaaaagctggcactacaggtgcacaccactgggcccagcataatggctatt   111218

chimp.LOC112206744.LOC107973052.GGT2-.646279-708040.rev.compl      ------------------------------------------------------------   61762
FAM230B-LOC105372935-GGT2.NCBI.GGT2.rev.compl                      tcttgacaaatcttgacaatgccaccagtatgtttttctttagagacagagtctcgcttt   111278

chimp.LOC112206744.LOC107973052.GGT2-.646279-708040.rev.compl      ------------------------------------------------------------   61762
FAM230B-LOC105372935-GGT2.NCBI.GGT2.rev.compl                      gtagcccaggctggagaacagtggtgtgatcctggctcactgcagcctcaaacacctgga   111338

chimp.LOC112206744.LOC107973052.GGT2-.646279-708040.rev.compl      ------------------------------------------------------------   61762
FAM230B-LOC105372935-GGT2.NCBI.GGT2.rev.compl                      ctcaggagatccccctgcctcagcctctcaggtagctgagactacaggtgcgcaccacca   111398

chimp.LOC112206744.LOC107973052.GGT2-.646279-708040.rev.compl      ------------------------------------------------------------   61762
FAM230B-LOC105372935-GGT2.NCBI.GGT2.rev.compl                      cacttggcgaattttattttatttttttttattatactttaagttctagggtacatacac   111458

chimp.LOC112206744.LOC107973052.GGT2-.646279-708040.rev.compl      ------------------------------------------------------------   61762
FAM230B-LOC105372935-GGT2.NCBI.GGT2.rev.compl                      ctggctaattataaaatgtttatctgtacagacaggttctcgttatgttgcccaggctgg   111518

chimp.LOC112206744.LOC107973052.GGT2-.646279-708040.rev.compl      ------------------------------------------------------------   61762
FAM230B-LOC105372935-GGT2.NCBI.GGT2.rev.compl                      tctcgaactctcggcctcaagcaatcctctcctcctcaccttccaaagccccgaaatatg   111578

chimp.LOC112206744.LOC107973052.GGT2-.646279-708040.rev.compl      ------------------------------------------------------------   61762
FAM230B-LOC105372935-GGT2.NCBI.GGT2.rev.compl                      cccacatccagcctgccaccagtttctttgcatacttctttcctaggcttgaccaagacc   111638

chimp.LOC112206744.LOC107973052.GGT2-.646279-708040.rev.compl      ------------------------------------------------------------   61762
FAM230B-LOC105372935-GGT2.NCBI.GGT2.rev.compl                      tttactttcttctgtgtacagctcagcattgaaaaaaaacaaaaaagtttaggctgggca   111698

chimp.LOC112206744.LOC107973052.GGT2-.646279-708040.rev.compl      ------------------------------------------------------------   61762
FAM230B-LOC105372935-GGT2.NCBI.GGT2.rev.compl                      caatgggtcacgcctgtaatcccaacacttagggtggccaaggtgggaggtttgcttgaa   111758

chimp.LOC112206744.LOC107973052.GGT2-.646279-708040.rev.compl      ------------------------------------------------------------   61762
FAM230B-LOC105372935-GGT2.NCBI.GGT2.rev.compl                      cccaggagttccacaccagcctaggcaagatggcaagaccacatctctccaaaacaattt   111818

chimp.LOC112206744.LOC107973052.GGT2-.646279-708040.rev.compl      ------------------------------------------------------------   61762
FAM230B-LOC105372935-GGT2.NCBI.GGT2.rev.compl                      ttgtaaaattagccaggtgtgctggtgcgggcctgcagtctcagctagtgagaagactaa   111878

chimp.LOC112206744.LOC107973052.GGT2-.646279-708040.rev.compl      ------------------------------------------------------------   61762
FAM230B-LOC105372935-GGT2.NCBI.GGT2.rev.compl                      ggcaagaggaacccttgaacccaggagttcaaggctacggtgagctgtgatcacctctcc   111938

chimp.LOC112206744.LOC107973052.GGT2-.646279-708040.rev.compl      ------------------------------------------------------------   61762
FAM230B-LOC105372935-GGT2.NCBI.GGT2.rev.compl                      agcctgggtgatagggtgagacccagtctctgaaaacaattaaaaataaaaaaagttgaa   111998

chimp.LOC112206744.LOC107973052.GGT2-.646279-708040.rev.compl      ------------------------------------------------------------   61762
FAM230B-LOC105372935-GGT2.NCBI.GGT2.rev.compl                      taatgagtcttctttttagaggcaatgtatttttcctctttttgttttccttaggctata   112058

chimp.LOC112206744.LOC107973052.GGT2-.646279-708040.rev.compl      ------------------------------------------------------------   61762
FAM230B-LOC105372935-GGT2.NCBI.GGT2.rev.compl                      tgcacaatagacaaagttgaaatttaatcttaatctcgtaaacccttactgtccaaacac   112118

chimp.LOC112206744.LOC107973052.GGT2-.646279-708040.rev.compl      ------------------------------------------------------------   61762
FAM230B-LOC105372935-GGT2.NCBI.GGT2.rev.compl                      atcaaactcaaaaaaaaaaaaagacaaaaacaaaaaaggtgaaaggcgtacaagagaaaa   112178

chimp.LOC112206744.LOC107973052.GGT2-.646279-708040.rev.compl      ------------------------------------------------------------   61762
FAM230B-LOC105372935-GGT2.NCBI.GGT2.rev.compl                      aagggaagtaatctgtgccaaatgtacattccttccacaatttgggacaaaaatgatgcc   112238

chimp.LOC112206744.LOC107973052.GGT2-.646279-708040.rev.compl      ------------------------------------------------------------   61762
FAM230B-LOC105372935-GGT2.NCBI.GGT2.rev.compl                      tacctggtctctgggtgccaaaataagacattagagacactcccctccttcaagaaataa   112298

chimp.LOC112206744.LOC107973052.GGT2-.646279-708040.rev.compl      ------------------------------------------------------------   61762
FAM230B-LOC105372935-GGT2.NCBI.GGT2.rev.compl                      aattggaatcagacactaagatacaattaaacagactttgaaagttggtatgcactactg   112358
```

```
chimp.LOC112206744.LOC107973052.GGT2-.646279-708040.rev.compl      ------------------------------------------------------------   61762
FAM230B-LOC105372935-GGT2.NCBI.GGT2.rev.compl                      ggaggtgtctcacagctggcagggtcaactgcaactagaagaaaagctcctaagggcaga   112418

chimp.LOC112206744.LOC107973052.GGT2-.646279-708040.rev.compl      ------------------------------------------------------------   61762
FAM230B-LOC105372935-GGT2.NCBI.GGT2.rev.compl                      aactaagtggctcacttcctctgtaacactgccgtgcccagcactgtaccagcaccccag   112478

chimp.LOC112206744.LOC107973052.GGT2-.646279-708040.rev.compl      ------------------------------------------------------------   61762
FAM230B-LOC105372935-GGT2.NCBI.GGT2.rev.compl                      cagagtcaacaactttccgtcaagccacacacttacggtctcatcgtccagtggaggccg   112538

chimp.LOC112206744.LOC107973052.GGT2-.646279-708040.rev.compl      ------------------------------------------------------------   61762
FAM230B-LOC105372935-GGT2.NCBI.GGT2.rev.compl                      cagatgagacatagtgtgccaaatcccaaatgcacagggtgccagacaagtctgcgggag   112598

chimp.LOC112206744.LOC107973052.GGT2-.646279-708040.rev.compl      ------------------------------------------------------------   61762
FAM230B-LOC105372935-GGT2.NCBI.GGT2.rev.compl                      gcaggaggactagggaagccttccaggcaaggtgatgcctgagctgagttgtttttcttt   112658

chimp.LOC112206744.LOC107973052.GGT2-.646279-708040.rev.compl      ------------------------------------------------------------   61762
FAM230B-LOC105372935-GGT2.NCBI.GGT2.rev.compl                      tgagtttggaacattaatttaatcggaggaaaacaagggacatgaaaagggcaaggaaat   112718

chimp.LOC112206744.LOC107973052.GGT2-.646279-708040.rev.compl      ------------------------------------------------------------   61762
FAM230B-LOC105372935-GGT2.NCBI.GGT2.rev.compl                      aagaaaatgagctgagttttgaaggcaatgatcagatgaagtgagagaagaatattccag   112778

chimp.LOC112206744.LOC107973052.GGT2-.646279-708040.rev.compl      ------------------------------------------------------------   61762
FAM230B-LOC105372935-GGT2.NCBI.GGT2.rev.compl                      cagagggagacaaggagactgcagagatttcaaggggccaagagctgttcggtgcagctc   112838

chimp.LOC112206744.LOC107973052.GGT2-.646279-708040.rev.compl      ------------------------------------------------------------   61762
FAM230B-LOC105372935-GGT2.NCBI.GGT2.rev.compl                      agtgaagcacctaaacagaggtggggaaggagaacaggggtcaaagcccagtattgaag   112898

chimp.LOC112206744.LOC107973052.GGT2-.646279-708040.rev.compl      ------------------------------------------------------------   61762
FAM230B-LOC105372935-GGT2.NCBI.GGT2.rev.compl                      tgttctggataccaagctaaggagaccttattacgaaggtactggggctgctgaagggtt   112958

chimp.LOC112206744.LOC107973052.GGT2-.646279-708040.rev.compl      ------------------------------------------------------------   61762
FAM230B-LOC105372935-GGT2.NCBI.GGT2.rev.compl                      taaatgggagagtgattgtgaacagattaatagtttggaaagatacaggcagaagtaaac   113018

chimp.LOC112206744.LOC107973052.GGT2-.646279-708040.rev.compl      ------------------------------------------------------------   61762
FAM230B-LOC105372935-GGT2.NCBI.GGT2.rev.compl                      ctctggatttaaccaaaaaataaaggacagaaccacaatgaagatgttggaaaagatcag   113078

chimp.LOC112206744.LOC107973052.GGT2-.646279-708040.rev.compl      ------------------------------------------------------------   61762
FAM230B-LOC105372935-GGT2.NCBI.GGT2.rev.compl                      gaaaaacactgttaaaagatattcactctcggggccgggcgctgtggctcacgccttaaa   113138

chimp.LOC112206744.LOC107973052.GGT2-.646279-708040.rev.compl      ------------------------------------------------------------   61762
FAM230B-LOC105372935-GGT2.NCBI.GGT2.rev.compl                      tcccggcactttggtaggccgaggcgggctgatcacgaggtcaggagatcgagaccatcc   113198

chimp.LOC112206744.LOC107973052.GGT2-.646279-708040.rev.compl      ------------------------------------------------------------   61762
FAM230B-LOC105372935-GGT2.NCBI.GGT2.rev.compl                      tggctaacacggtcaaaccccgtctctactaaaaatacaaaaaattagccgggtgtggtg   113258

chimp.LOC112206744.LOC107973052.GGT2-.646279-708040.rev.compl      ------------------------------------------------------------   61762
FAM230B-LOC105372935-GGT2.NCBI.GGT2.rev.compl                      gcgggcgcctgtagtcccaactactccagaggctgaggcaggagaatggcgtgaacccgg   113318

chimp.LOC112206744.LOC107973052.GGT2-.646279-708040.rev.compl      ------------------------------------------------------------   61762
FAM230B-LOC105372935-GGT2.NCBI.GGT2.rev.compl                      taggcggagcttgcggtgagccgagatcaggccactggaatccagcctgggcgacagagg   113378

chimp.LOC112206744.LOC107973052.GGT2-.646279-708040.rev.compl      ------------------------------------------------------------   61762
FAM230B-LOC105372935-GGT2.NCBI.GGT2.rev.compl                      gagactccatctcaaaaaaaaaaaaaaaaaaaaagagagagagagtctgactctgttgcc   113438

chimp.LOC112206744.LOC107973052.GGT2-.646279-708040.rev.compl      ------------------------------------------------------------   61762
FAM230B-LOC105372935-GGT2.NCBI.GGT2.rev.compl                      aagactggagtgcagtggcgcgatctaggcacatcacaatcccttccccgcccccgggtt   113498

chimp.LOC112206744.LOC107973052.GGT2-.646279-708040.rev.compl      ------------------------------------------------------------   61762
FAM230B-LOC105372935-GGT2.NCBI.GGT2.rev.compl                      caagtgattctcctgtctcagccgccggagtagctgggactacaggcgcgtgccaccatg   113558

chimp.LOC112206744.LOC107973052.GGT2-.646279-708040.rev.compl      ------------------------------------------------------------   61762
FAM230B-LOC105372935-GGT2.NCBI.GGT2.rev.compl                      tctgattaaatctgtatttttactagagacggggtttcactctgttggccaggctggtct   113618

chimp.LOC112206744.LOC107973052.GGT2-.646279-708040.rev.compl      ------------------------------------------------------------   61762
FAM230B-LOC105372935-GGT2.NCBI.GGT2.rev.compl                      ccaactcctgatctcgtgatccgcccgccccgacctcccaaagtgctgggatgcgtgagc   113678

chimp.LOC112206744.LOC107973052.GGT2-.646279-708040.rev.compl      ------------------------------------------------------------   61762
FAM230B-LOC105372935-GGT2.NCBI.GGT2.rev.compl                      ctccacgcctggccactaatttttctttctttctttcgtgtgtgtgtgtgtgtgtgtgtg   113738

chimp.LOC112206744.LOC107973052.GGT2-.646279-708040.rev.compl      ------------------------------------------------------------   61762
FAM230B-LOC105372935-GGT2.NCBI.GGT2.rev.compl                      tgtgtgtgtgtgtgtgtgtgtgtgtgacgaagtttcgctcttgttgcccaggttggagtg   113798

chimp.LOC112206744.LOC107973052.GGT2-.646279-708040.rev.compl      ------------------------------------------------------------   61762
FAM230B-LOC105372935-GGT2.NCBI.GGT2.rev.compl                      caatggtgtgatctcagctcactgcaatccccgcctcaacaggagagcaggaatcttcag   113858

chimp.LOC112206744.LOC107973052.GGT2-.646279-708040.rev.compl      ------------------------------------------------------------   61762
FAM230B-LOC105372935-GGT2.NCBI.GGT2.rev.compl                      tgatccactggcggatctgcagccattgtgcgcgccaggtcttcccaagtcttttgtgcg   113918

chimp.LOC112206744.LOC107973052.GGT2-.646279-708040.rev.compl      ------------------------------------------------------------   61762
FAM230B-LOC105372935-GGT2.NCBI.GGT2.rev.compl                      cgcgcctctccttccagtacctatgccgccagcgtcccctagcctcccgccattgccagc   113978

chimp.LOC112206744.LOC107973052.GGT2-.646279-708040.rev.compl      ------------------------------------------------------------   61762
FAM230B-LOC105372935-GGT2.NCBI.GGT2.rev.compl                      aggtgctgagatcgcgccattgcactccagcctggggacaagagcgaaactccatgtca   114038

chimp.LOC112206744.LOC107973052.GGT2-.646279-708040.rev.compl      ------------------------------------------------------------   61762
FAM230B-LOC105372935-GGT2.NCBI.GGT2.rev.compl                      aaaaaaaaaaaaaggatgaaaattttgggaaaatatggaagaaaccaaatggatttctagc   114098

chimp.LOC112206744.LOC107973052.GGT2-.646279-708040.rev.compl      ------------------------------------------------------------   61762
FAM230B-LOC105372935-GGT2.NCBI.GGT2.rev.compl                      tcaactaaatcgtaattattcagtgtctagttttggcaagaaaccacatatttcatgtcc   114158

chimp.LOC112206744.LOC107973052.GGT2-.646279-708040.rev.compl      ------------------------------------------------------------   61762
FAM230B-LOC105372935-GGT2.NCBI.GGT2.rev.compl                      acaatcagggacacagtcccagctctcaagtgtgggtctttcctaagcaaattgaagaac   114218

chimp.LOC112206744.LOC107973052.GGT2-.646279-708040.rev.compl      ------------------------------------------------------------   61762
FAM230B-LOC105372935-GGT2.NCBI.GGT2.rev.compl                      acaggcataaaagtacattaaatcaataaaccttttttctctctgtctctctctctcttt   114278

chimp.LOC112206744.LOC107973052.GGT2-.646279-708040.rev.compl      ------------------------------------------------------------   61762
FAM230B-LOC105372935-GGT2.NCBI.GGT2.rev.compl                      tttttttttttttgagacggaggttggcactgtcacccaggctggagtgctgtggtgaga   114338

chimp.LOC112206744.LOC107973052.GGT2-.646279-708040.rev.compl      ------------------------------------------------------------   61762
FAM230B-LOC105372935-GGT2.NCBI.GGT2.rev.compl                      tctcgggtcactgcaagctccgcctcccgagttcacgccatcctcctgcctcagcctcca   114398

chimp.LOC112206744.LOC107973052.GGT2-.646279-708040.rev.compl      ------------------------------------------------------------   61762
FAM230B-LOC105372935-GGT2.NCBI.GGT2.rev.compl                      gagtagctgggactacaagcgcccgccacgacgcccggctaattttttgtagttttagta   114458

chimp.LOC112206744.LOC107973052.GGT2-.646279-708040.rev.compl      ------------------------------------------------------------   61762
FAM230B-LOC105372935-GGT2.NCBI.GGT2.rev.compl                      gagacggggtttcgctatgttggccaggctggtctccaactcgtgacatcgtgatccgcc   114518
```

```
chimp.LOC112206744.LOC107973052.GGT2-.646279-708040.rev.compl     ------------------------------------------------------------   61762
FAM230B-LOC105372935-GGT2.NCBI.GGT2.rev.compl                     cgcctccgactcccaaagtgccacagcccaggcctttttttttttaagacagagtctcggc   114578

chimp.LOC112206744.LOC107973052.GGT2-.646279-708040.rev.compl     ------------------------------------------------------------   61762
FAM230B-LOC105372935-GGT2.NCBI.GGT2.rev.compl                     ccggcgcggtggctcacgcctttaatcccagcactttgggaggccgaggcgggctgatca   114638

chimp.LOC112206744.LOC107973052.GGT2-.646279-708040.rev.compl     ------------------------------------------------------------   61762
FAM230B-LOC105372935-GGT2.NCBI.GGT2.rev.compl                     cgaggtcaggaggtcgagaccatcctggctaacacggtcaaaccccgtctctactaaaaa   114698

chimp.LOC112206744.LOC107973052.GGT2-.646279-708040.rev.compl     ------------------------------------------------------------   61762
FAM230B-LOC105372935-GGT2.NCBI.GGT2.rev.compl                     tacaaaaaattagccgggtgtggtggcgggcgcctgtagtccgagctactcccgaggctg   114758

chimp.LOC112206744.LOC107973052.GGT2-.646279-708040.rev.compl     ------------------------------------------------------------   61762
FAM230B-LOC105372935-GGT2.NCBI.GGT2.rev.compl                     aggcaggagaatggcgtgaacccggtaggcggagcttgcggtgagccgagatcaggccac   114818

chimp.LOC112206744.LOC107973052.GGT2-.646279-708040.rev.compl     ------------------------------------------------------------   61762
FAM230B-LOC105372935-GGT2.NCBI.GGT2.rev.compl                     tggaatccagcctgggcgacagagggagactccgtctcaaaaaaaaaaaaaaagagtctg   114878

chimp.LOC112206744.LOC107973052.GGT2-.646279-708040.rev.compl     ------------------------------------------------------------   61762
FAM230B-LOC105372935-GGT2.NCBI.GGT2.rev.compl                     gctctgttgcccaggctggagtgcagtggcgcgatctcggcgcatcgcaatcccttcccc   114938

chimp.LOC112206744.LOC107973052.GGT2-.646279-708040.rev.compl     ------------------------------------------------------------   61762
FAM230B-LOC105372935-GGT2.NCBI.GGT2.rev.compl                     gcccccgggttcaagtgattctccagtctcagccgccggagtagctgggactacaggcgc   114998

chimp.LOC112206744.LOC107973052.GGT2-.646279-708040.rev.compl     ------------------------------------------------------------   61762
FAM230B-LOC105372935-GGT2.NCBI.GGT2.rev.compl                     gtgccaccatgtctgactaaatttgtatttttactagagacggggtttcactatgttggc   115058

chimp.LOC112206744.LOC107973052.GGT2-.646279-708040.rev.compl     ------------------------------------------------------------   61762
FAM230B-LOC105372935-GGT2.NCBI.GGT2.rev.compl                     caggctggtctccaactcctgatctcgtgatccgtctgccccgacctcccaaagtgctag   115118

chimp.LOC112206744.LOC107973052.GGT2-.646279-708040.rev.compl     ------------------------------------------------------------   61762
FAM230B-LOC105372935-GGT2.NCBI.GGT2.rev.compl                     gattataggcataagccaccacgcccggcctctttttttttctttttcttttctttatctg   115178

chimp.LOC112206744.LOC107973052.GGT2-.646279-708040.rev.compl     ------------------------------------------------------------   61762
FAM230B-LOC105372935-GGT2.NCBI.GGT2.rev.compl                     gagactgagttttgctcttgttgcccaggctggagtgcaatggtgcgatctcagctcact   115238

chimp.LOC112206744.LOC107973052.GGT2-.646279-708040.rev.compl     ------------------------------------------------------------   61762
FAM230B-LOC105372935-GGT2.NCBI.GGT2.rev.compl                     gcaatctccacctcagcaggagagcaggaatcttcagtgatccacgggcagatctgccgc   115298

chimp.LOC112206744.LOC107973052.GGT2-.646279-708040.rev.compl     ------------------------------------------------------------   61762
FAM230B-LOC105372935-GGT2.NCBI.GGT2.rev.compl                     cattgtgggcacctgtttctcccgcaacctttgtgcccgcctctctccttcccgtaccta   115358

chimp.LOC112206744.LOC107973052.GGT2-.646279-708040.rev.compl     ------------------------------------------------------------   61762
FAM230B-LOC105372935-GGT2.NCBI.GGT2.rev.compl                     ttgcatgacccccccacgtccgcctcccgccattgccagcaagtgcctcgcgcgggtacct   115418

chimp.LOC112206744.LOC107973052.GGT2-.646279-708040.rev.compl     ------------------------------------------------------------   61762
FAM230B-LOC105372935-GGT2.NCBI.GGT2.rev.compl                     ggctgcgcttattaatccgttaagctcgctctgtcacgggcgccgtgatgtgctcacgcg   115478

chimp.LOC112206744.LOC107973052.GGT2-.646279-708040.rev.compl     ------------------------------------------------------------   61762
FAM230B-LOC105372935-GGT2.NCBI.GGT2.rev.compl                     cccgctccctcaggtttaaaaggcgcgttgcccggcaacagaagaaactgctggcttagc   115538

chimp.LOC112206744.LOC107973052.GGT2-.646279-708040.rev.compl     ------------------------------------------------------------   61762
FAM230B-LOC105372935-GGT2.NCBI.GGT2.rev.compl                     cgttggccgagttggcggctggacgaggacgctcagagcccagctctcgagagttcaagc   115598

chimp.LOC112206744.LOC107973052.GGT2-.646279-708040.rev.compl     ------------------------------------------------------------   61762
FAM230B-LOC105372935-GGT2.NCBI.GGT2.rev.compl                     aaccgacggttccccactgctcccaggagcggttacctgggcactctgtgcccctccttc   115658

chimp.LOC112206744.LOC107973052.GGT2-.646279-708040.rev.compl     ------------------------------------------------------------   61762
FAM230B-LOC105372935-GGT2.NCBI.GGT2.rev.compl                     ctgttcgggcccaggccgaggacctgccagtagggctcagttgcctggagcccgttcagc   115718

chimp.LOC112206744.LOC107973052.GGT2-.646279-708040.rev.compl     ------------------------------------------------------------   61762
FAM230B-LOC105372935-GGT2.NCBI.GGT2.rev.compl                     ccatcccccagttcactttgcttgtgggatctccccgttgctcctgcccgtggactgagt   115778

chimp.LOC112206744.LOC107973052.GGT2-.646279-708040.rev.compl     ------------------------------------------------------------   61762
FAM230B-LOC105372935-GGT2.NCBI.GGT2.rev.compl                     ggcaggccatcctacaagcacccggacacttgacatcagtggtgtcaagacaactctaag   115838

chimp.LOC112206744.LOC107973052.GGT2-.646279-708040.rev.compl     ------------------------------------------------------------   61762
FAM230B-LOC105372935-GGT2.NCBI.GGT2.rev.compl                     aaggttttccgtgatcctgcaagccctgccttccttcctgggatcctgccttcaatttga   115898

chimp.LOC112206744.LOC107973052.GGT2-.646279-708040.rev.compl     ------------------------------------------------------------   61762
FAM230B-LOC105372935-GGT2.NCBI.GGT2.rev.compl                     ttgcacaggtaccacagcaagccagtgctgcgtgctccgagttccagggcgtcctccagc   115958

chimp.LOC112206744.LOC107973052.GGT2-.646279-708040.rev.compl     ------------------------------------------------------------   61762
FAM230B-LOC105372935-GGT2.NCBI.GGT2.rev.compl                     tcagccactgcactgagaacatggactctctgtggggcccaggagccgggagtcacccct   116018

chimp.LOC112206744.LOC107973052.GGT2-.646279-708040.rev.compl     ------------------------------------------------------------   61762
FAM230B-LOC105372935-GGT2.NCBI.GGT2.rev.compl                     ttggggtccacaacagccggctgtccccagacttgtgtccagggaagatagtgttgaggg   116078

chimp.LOC112206744.LOC107973052.GGT2-.646279-708040.rev.compl     -------------------------------------------     61762
FAM230B-LOC105372935-GGT2.NCBI.GGT2.rev.compl                     ccctcaaggagagcggggcagggatgcctgagcaggacaagga     116121
```
