# Supplementary material for: Formation of human long intergenic non-coding RNA genes, pseudogenes, and protein genes: Ancestral sequences are key players
Source: PLoS One. 2020 Mar 26;15(3):e0230236. doi: 10.1371/journal.pone.0230236 (PMC7098633; doi:10.1371/journal.pone.0230236)

Formation of human long intergenic non-coding RNA genes and pseudogenes: ancestral sequences are key players

Nicholas Delihas

S4 Fig. Circular RNA expression from lincRNA genes *FAM230E* (top) and *FAM230B* (bottom) in various tissues during human fetal development.

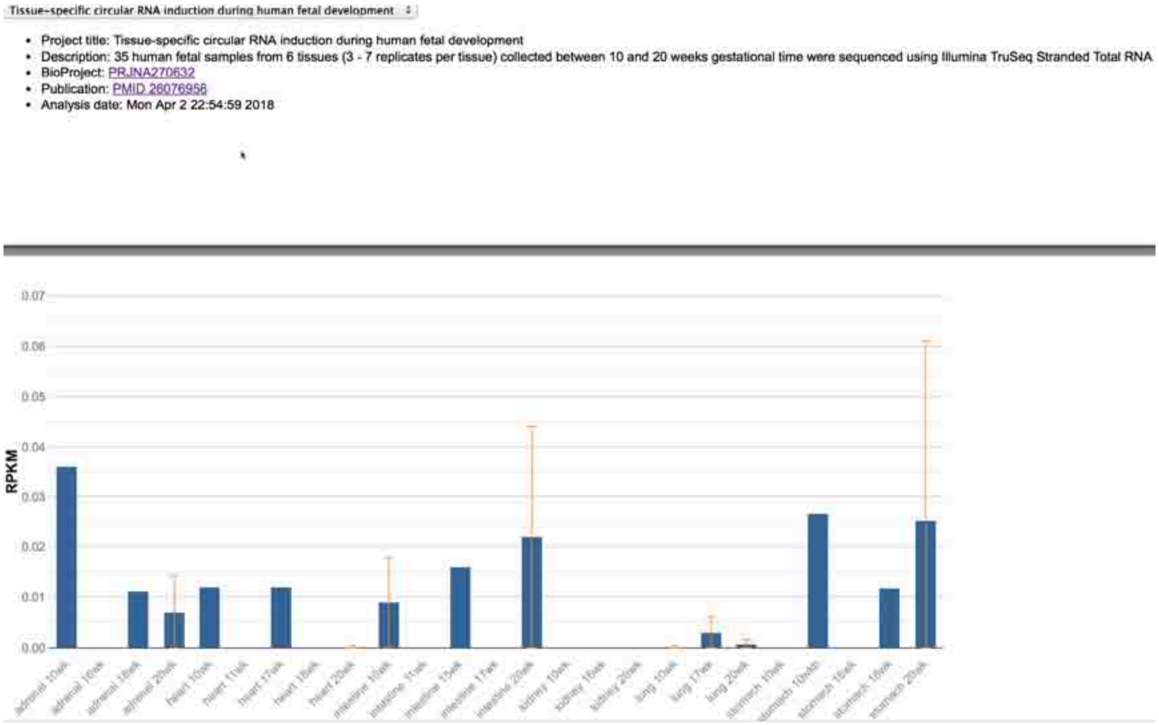

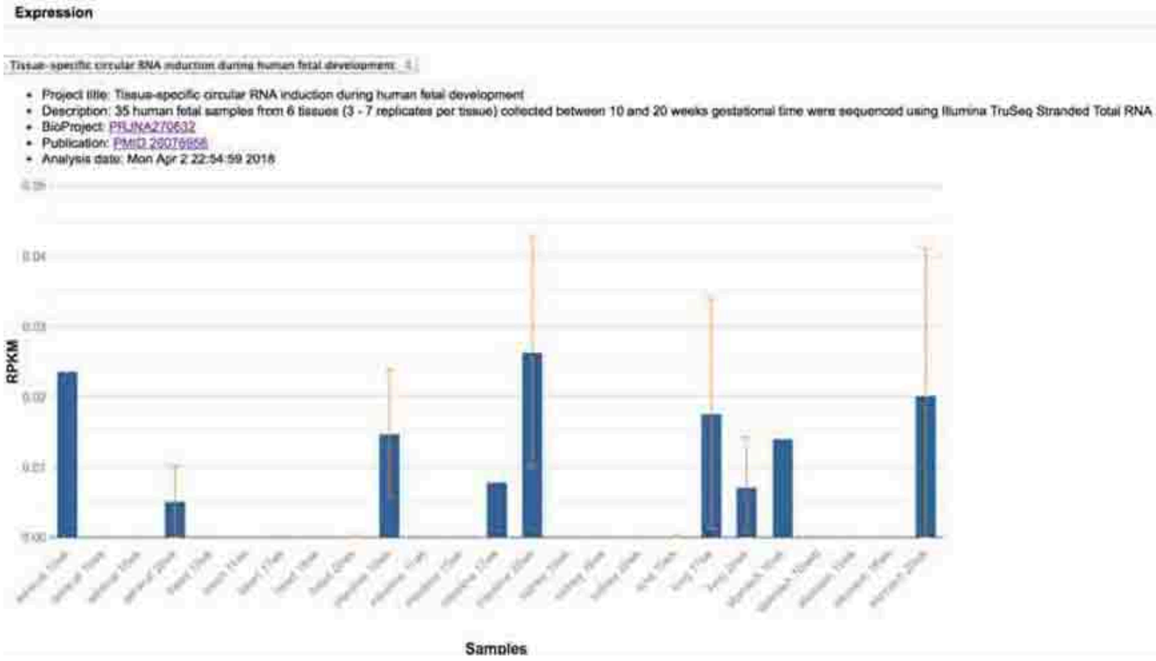

Supplement: S4 Fig — The data are from Szabo et al [23] as shown on the NCBI websites for these genes. (PDF) [file pone.0230236.s004.pdf]
