# Supplementary material for: Formation of human long intergenic non-coding RNA genes, pseudogenes, and protein genes: Ancestral sequences are key players
Source: PLoS One. 2020 Mar 26;15(3):e0230236. doi: 10.1371/journal.pone.0230236 (PMC7098633; doi:10.1371/journal.pone.0230236)
Supplement: S5 Fig — The Clustal Omega, Multiple sequence alignment program was used for sequence alignment. (PDF) [file pone.0230236.s005.pdf]

Formation of human long intergenic non-coding RNA genes and pseudogenes: ancestral sequences are key players

Nicholas Delihas

S5 Fig. Alignment of human *GGT5* with *LOC105372935* clincRNA gene sequences.

```
CLUSTAL O(1.2.4) multiple sequence alignment


GGT5.NCBI.REF                       tgcctggcctcctgccagcaacaagcaggagctgaaaaccagaagttgaggcgtgagttt    60
LOC105372935.clincRNA.NCBI.ref      --------------------------------tgaaaactagaagttgaggcatgagttt    28
                                                                    ******* ************ *******

GGT5.NCBI.REF                       ggccactccgtagtgtgcacttggtgagggcagcagctcgccacagctgccagccatctg    120
LOC105372935.clincRNA.NCBI.ref      ggccactccgtagtgtgcacttggtgagggcagcagctcgccacagctgccagccatctg    88
                                    ************************************************************

GGT5.NCBI.REF                       tccattcacccatctgtccatctggcagcccgctgttcagacctgtctgtctgtccgccc    180
LOC105372935.clincRNA.NCBI.ref      tccattcacccatctgtccatctggcagcccgctgttcagacctgtctgtctgtccgccc    148
                                    ************************************************************

GGT5.NCBI.REF                       atctgtaagcccatctctgtcccattgtctatctgaccatctttctcttactgtcctctt    240
LOC105372935.clincRNA.NCBI.ref      atctgtaagcccatctctgtcccattgtctatctgaccatctttctcttactgtcctctt    208
                                    ************************************************************

GGT5.NCBI.REF                       tgtctagctatctggcctatctgtcgatccatcttcgtgtctgtcttcagcccccacctg    300
LOC105372935.clincRNA.NCBI.ref      tgtctagctatctggcctgtctgtcgatccatcttcgtgtctgtcttcagcccccacctg    268
                                    ****************** *****************************************

GGT5.NCBI.REF                       tttgtccatctgtccaattacctgtga----ctctgtgcatcttcttgtccattcatctg    356
LOC105372935.clincRNA.NCBI.ref      tttgtccatctgtccaattacctgtgagtctatctatgcatcttcttgtccattcatctg    328
                                    ***************************     *** ************************

GGT5.NCBI.REF                       cccacccatccgtccctccgtctgcccaccagccgcccctctcctcctgggctgcagagc    416
LOC105372935.clincRNA.NCBI.ref      cccacccatctgtccctccatctgcccaccggcctcccctctccttctgggccgcagagc    388
                                    ********** ******** ********** *** ********** ****** *******

GGT5.NCBI.REF                       catggcccggggctacggggccacggtcagcctagtcct------gctgggtctggggct    470
LOC105372935.clincRNA.NCBI.ref      catggcccaggactacggagccatgggtgacctggtcctgctggggctggggctggggct    448
                                    ******** ** ****** **** **    *** *****      ****** ********

GGT5.NCBI.REF                       ggcgctggctgtcattgtgctggctgtggtcctctctcgacaccaggccccatgtggccc    530
LOC105372935.clincRNA.NCBI.ref      ggcgctggctgtcattgtgctggctgtggtcctctctcgacaccaggccccatttgaccc    508
                                    ***************************************************** ** ***

GGT5.NCBI.REF                       ccaggcctttgcccacgctgctgttgccgccgactccaaggtctgctcggatattggacg    590
LOC105372935.clincRNA.NCBI.ref      cc-ggcctttgcccacgccgctgttgctgctgactccaaggtcttctcaaatattgtacg    567
                                    ** *************** ******** ** ************* ***  ****** ***

GGT5.NCBI.REF                       gtgagtgagacgtgggaggaagctgggtggcccttggcagccagcccctcctggagaagg    650
LOC105372935.clincRNA.NCBI.ref      gtgagtgagacgtgggaggaagctgggtggcctttggcagccagcccctcctggagaagg    627
                                    ******************************** ***************************

GGT5.NCBI.REF                       cgtgtgtgtgt-------------------------------------------------    661
LOC105372935.clincRNA.NCBI.ref      cgtgtgtgtgtgagcatgtgtgtgtgtgagagattatgtgtgagtgtgtgtgggtatatg    687
                                    ***********

GGT5.NCBI.REF                       ----------------------------------------------gtgtgtgtgtgagt    675
LOC105372935.clincRNA.NCBI.ref      tgtgagtgtgtttgtggggtgtgggtgtgtgtgaatgtgtgtgatcgtgtttgggtgtgt    747
                                                                                  **** ** *** **

GGT5.NCBI.REF                       gaatgtatgtgtgggtgtgtgtgtgagtgtctgggtatgtgtgattgcatgcatgtgtgg    735
LOC105372935.clincRNA.NCBI.ref      gtatgtgtgagtgtgggtgtgtgtgaatgtgtgtgattgtgtttgtgtatgtgtgtgtgg    807
                                    * **** ** *** * ********** *** ** *  *****   ** ***  *******

GGT5.NCBI.REF                       gtgtgtgtgagtgtatgtgagtgtgagtgtg-----------------------------    766
LOC105372935.clincRNA.NCBI.ref      gtgtgtgtgagtatatgtgagtgtgagtgtgtgggggtgtgggtgggtgtgaatgtgtgt    867
                                    ************ ******************

GGT5.NCBI.REF                       ------------------------------------------------------------    766
LOC105372935.clincRNA.NCBI.ref      gattgtgtttcgctgtgtgagggtgtgtgtgactgtgagtgtgtgagtgtgggtgtgtgg    927


GGT5.NCBI.REF                       ----------------------------gggagtgtgggtgtgtgtgaatgtgtgtgatt    798
LOC105372935.clincRNA.NCBI.ref      gtgtgtgtaaatgtgtgagtgtgagtatgggggggtgggtatgtgtgaatgtgtgtgatt    987
                                                                *** * ****** *******************

GGT5.NCBI.REF                       gtgtgtgggtatgtgtatgtgtgggtgggtgtgtgt----gtgtgtgtgtgtgcacgtgc    854
LOC105372935.clincRNA.NCBI.ref      gtgtgtgggtatatttgtgggggtgtgtgtgtgtgtgcacgtgtgtgtgtgtgcacgtgc    1047
                                    ************ * * ** * * *** ********    ********************

GGT5.NCBI.REF                       actggcccaggaagcaggagccgtgtgtgtgtgggcttcagcacctgcaggtcttgggcg    914
LOC105372935.clincRNA.NCBI.ref      actggcccaggaagcaggagcc--gtgtgtgtgggcttcagcacctgcagggcttgagcg    1105
```

```
                                       **********************  *************************** **** ***

GGT5.NCBI.REF                          caaggaggcagcctcagggcccttgcacagaacaggtggcagggtgtgctcgtggggcag    974
LOC105372935.clincRNA.NCBI.ref         caaggagacagcctcagggcccttgcacagaacaggcggcagggtgtgcccgtggggcag    1165
                                       ******* **************************** ************ **********

GGT5.NCBI.REF                          atggggacttggggacaatggtggtgtgtgagtccacgcctggctccaggattcaggagg    1034
LOC105372935.clincRNA.NCBI.ref         atggggacttggggacaatggtggtgtgtgagtccatacctggctccaggattcaggagg    1225
                                       ************************************  **********************

GGT5.NCBI.REF                          cccatttgcatatcccaggtgggaacctgtctggccccgcctgaccctgctggccggtgc    1094
LOC105372935.clincRNA.NCBI.ref         cccatttgcatatcccaggtgggaacctgtctggccccgcctgaccctgctggccggcgc    1285
                                       ********************************************************* **

GGT5.NCBI.REF                          aggccccttcagtgaggccaattctccaaggctgcggtcttctcccagggtcatgggtga    1154
LOC105372935.clincRNA.NCBI.ref         aggccccttcagtgaggccaattctccaaggctgcggtcttctcccagggtcatgggtga    1345
                                       ************************************************************

GGT5.NCBI.REF                          aggggtttggaggctccctgcgtgggtactggcctgctggggtacacacaatgctgccat    1214
LOC105372935.clincRNA.NCBI.ref         aggggtttggaggctccctgcgtgggtactggcctgctggggtacacacaatgctgccat    1405
                                       ************************************************************

GGT5.NCBI.REF                          agccagtctgccccaacacccagcctggggccacatctcgggtctctcagtcctgaggag    1274
LOC105372935.clincRNA.NCBI.ref         agccagtctgcccctacacccagcccggggccacatctcaggtctctcagtcctgaggag    1465
                                       ************** ********** ************* ********************

GGT5.NCBI.REF                          cccggtgccccacccctcacatcctctctccctgagtcagggcctgggtctcgtgagctg    1334
LOC105372935.clincRNA.NCBI.ref         cccggtgccccacccctcacatcctctctccctgagtcagggcctgggtctcgtgagctg    1525
                                       ************************************************************

GGT5.NCBI.REF                          agtgactgatacttggtgtcctggatgagggcgtggtggagaggggccacagcgggtgtt    1394
LOC105372935.clincRNA.NCBI.ref         agtgactgatacttggtgtcctggatgagggcgtggtggagaggggccacagcgggtgtt    1585
                                       ************************************************************

GGT5.NCBI.REF                          tcctgaccctcttccaggaagcccagcccaagggaggcctccgctgctgctgctgca--g    1452
LOC105372935.clincRNA.NCBI.ref         tcctgaccctcttccaggaag-------------------gtgctgctgccgctgcaggg    1626
                                       *********************                     ******** ******  *

GGT5.NCBI.REF                          aggacacatacaggatgccccttcctgctccctgcctcccactgggcccacaaaagccag    1512
LOC105372935.clincRNA.NCBI.ref         aggacacacacaggatgccccttcttgccccctgcctcccattgggcccacaaaagccag    1686
                                       ******** *************** *** ************ ******************

GGT5.NCBI.REF                          ggcaagcctcccctccctgccagccacctggtctgcttcccagaagttctgtcttgcagg    1572
LOC105372935.clincRNA.NCBI.ref         ggcaagcctcccctccctgccagccacctggtctgcttcccagaaattctgtcttgcagg    1746
                                       ********************************************* **************

GGT5.NCBI.REF                          ctgttgggaggatcccagtgctttgtaaattaaagcaagggaggagtggctgctctctct    1632
LOC105372935.clincRNA.NCBI.ref         ctgttgggaggatcccagtactttgtaaactaaagcaagggaggagtggccgttctctct    1806
                                       ******************* ********* ******************** * *******

GGT5.NCBI.REF                          ctctgttcattcattcaccttttcattcattccttcttccctccattcccccatctgtcc    1692
LOC105372935.clincRNA.NCBI.ref         ctttgttcattcattcaccttttcattcattccttcttccctccattcccccatctgtgc    1866
                                       ** ******************************************************* *

GGT5.NCBI.REF                          atccttccctgccctgatttctcatgccacccccc----gcccctcctgacctggtcctt    1748
LOC105372935.clincRNA.NCBI.ref         atccttccctgccctgattgctcatgccacccccccccagcccctcctgacctggtcctt    1926
                                       ******************* ***************    *********************

GGT5.NCBI.REF                          tggtttctcttcaggtctttctgtctcctcccacagggctgagaatggcagctcagggac    1808
LOC105372935.clincRNA.NCBI.ref         tggtttctcttcagggctttctgtctcctcccacagggctgagaatggcagctcagggac    1986
                                       *************** ********************************************

GGT5.NCBI.REF                          aagtcggggctggggactgcttagtctccccagtggctctcaggggatttgagggattga    1868
LOC105372935.clincRNA.NCBI.ref         aagtaggggctggggactgcttagtctccccagtggctctcaggggatttgagggtttga    2046
                                       **** ************************************************** ****

GGT5.NCBI.REF                          cgccagccgccaccccaggctgtgcccctcctctgctcaggaggacattcag-gatgcga    1927
LOC105372935.clincRNA.NCBI.ref         cgccagctgccaccccaggctgtgcccctcctctgctcgggaggacatacagagatgcga    2106
                                       ******* ****************************** ********* *** *******

GGT5.NCBI.REF                          cacccacttaaactcgaagttgcaaagatgcaaatgagactggagtctcaggcaccagag    1987
LOC105372935.clincRNA.NCBI.ref         cacccacttaaactcgaagttgcaaagatgcaaatgagactggggtctcaggcaccagag    2166
                                       ******************************************* ****************

GGT5.NCBI.REF                          accacccgtgggcatgtggcctttgggagtggggacctgctgccacaaatttctaggtgg    2047
LOC105372935.clincRNA.NCBI.ref         accacccgtgggcacgtggcttttgggagtggggacctgctgccacagatctct--gaag    2224
                                       ************** ***** ************************** ** ***  *  *

GGT5.NCBI.REF                          agtctggacctgctgggtctccctgagtgactgtctgggggtctccatagcgtgccctgc    2107
LOC105372935.clincRNA.NCBI.ref         agtctggacctgctgggtctccccgagtgactgtctgggggtctccatagcatgccctgc    2284
                                       *********************** *************************** ********

GGT5.NCBI.REF                          tgtgtgcgtgacggtcactggttgggtaggggtctctactctaaagctccctctgctggc    2167
LOC105372935.clincRNA.NCBI.ref         tgtgtgcgtgacggtcactggttgggtaggggtctctactctaaagctccctctgccggc    2344
                                       ******************************************************** ***

GGT5.NCBI.REF                          atcccctcaaactgtcccttgaag-agagaggatgtggtttgccccagtgttttgtcaaa    2226
LOC105372935.clincRNA.NCBI.ref         atcccctccaactctcccttggtgaagagaggatgtggtttgccccagtgttttatcaaa    2404
                                       ******** **** *******  * ***************************** *****

GGT5.NCBI.REF                          caactctctccacttcctgtcttaagaagctgggagtggaagagagcctggggctggccc    2286
LOC105372935.clincRNA.NCBI.ref         caactctctccacttccagttttaagaagctgggagtggaagagagcctggggctggccc    2464
                                       ***************** ** ***************************************
```

```
GGT5.NCBI.REF                        cagctgctgctgcggaacaggggtcattggacactgggaccctggccggactggctgggg    2346
LOC105372935.clincRNA.NCBI.ref       cagctgctgctgtgaaacaggggtcactggacgctgggaccctggccgggctggct-gga    2523
                                     ************ * *********** ***** **************** ****** ** 

GGT5.NCBI.REF                        ggcctcaggaagaggcctgctgcagcgtcatcctggccaagatccctccttgcagaggcc    2406
LOC105372935.clincRNA.NCBI.ref       ggcctcaggaagaggcctgctacagcgtcatcctggccaagattcctccctgcagaggac    2583
                                     ********************* ********************* ***** ******** *

GGT5.NCBI.REF                        cctggccacactgccacagggtctgctggggccaccagaagcccatgctcctgactccat    2466
LOC105372935.clincRNA.NCBI.ref       cctggccacgctgccacagggtctgctggggccaccagaagcccatgctcctgcctc---    2640
                                     ********* ******************************************* ***   

GGT5.NCBI.REF                        catctctcccctctgtgctcacctctcaccaggaggccctcccagagtccagtctcctgc    2526
LOC105372935.clincRNA.NCBI.ref       catctctcccctctgtgctcacctctcaccaggaggccctcccagagttcagtgtcctgc    2700
                                     ************************************************ **** ******

GGT5.NCBI.REF                        tcttttttctgttttgtttttgagatgctgtttcgctctgtcaccaggctggagtgcagt    2586
LOC105372935.clincRNA.NCBI.ref       ttttttt----tttttttttttgtgacggtgtctcactctgtcaccaggctggagtgcagt    2756
                                     * *****    **** ****** ** * *** ** *************************

GGT5.NCBI.REF                        ggcatgatctcggctcgctgcaacctctgcctccttggttcaaatgattctcctgcctca    2646
LOC105372935.clincRNA.NCBI.ref       ggcgcgatctcagcttactgcaacctctgcttcctcggttcaaatgattctcctgcctca    2816
                                     ***  ****** ***  ************* **** ************************

GGT5.NCBI.REF                        gcctcctgagtagctgggactacaggtgctagccaccacgcccagctaattttgtattt    2706
LOC105372935.clincRNA.NCBI.ref       gcctcctgagtagctgggactacaggtgccagccaccacgccccgctaatttctgtattt    2876
                                     ***************************** ************* ******** *******

GGT5.NCBI.REF                        ttagtagagatggggtttcaccatgttagccaggatggtctccaactctagacctcgtgc    2766
LOC105372935.clincRNA.NCBI.ref       ttagtagagacggggtttcaccgtgttggccaggatggtctctatctcttga--------    2928
                                     ********** *********** **** ************** * **** **        

GGT5.NCBI.REF                        tttgcccacctccgcctcccaaagtgctgggattacaggagtgagtcacggcacccagcc    2826
LOC105372935.clincRNA.NCBI.ref       ttcacccgccttggccacccaaagtgctggcattacaggagtgagtcatggcacctggcc    2988
                                     **  *** ***  *** ************* ***************** ******  ***

GGT5.NCBI.REF                        ccatctcctactctttcagcactaggttttattcttgggattctgctacagccggagccc    2886
LOC105372935.clincRNA.NCBI.ref       tcatctcctactctttcagcaccaggttttattcttgggattctgctacagccggagccc    3048
                                      ********************* *************************************

GGT5.NCBI.REF                        ctgggtgcaagctcctaagctttctgtgagtgtggacccagcaccgtgcctagtagacat    2946
LOC105372935.clincRNA.NCBI.ref       ctgggtgcgagttcctaaggtttctgtgagtgtggacccagcaccgtgcctagtagacat    3108
                                     ******** ** ******* ****************************************

GGT5.NCBI.REF                        acaaaaggagcatggtgacagtgaggtctgttatctccagcataatgactgttttgatcc    3006
LOC105372935.clincRNA.NCBI.ref       acaaaaggagcatggtgacagtgaggtctgtcatctccagcataatgactgttttgatcc    3168
                                     ******************************* ****************************

GGT5.NCBI.REF                        ttgtaaaaaaggtgatttttggctgggtgtggtggctcacacctgtgatcccagcacttt    3066
LOC105372935.clincRNA.NCBI.ref       ttgtaaaaaaggtgatttttggctgggtgtggtggctcacacctgtaatcccagcacttt    3228
                                     ********************************************** *************

GGT5.NCBI.REF                        gggaggctgaggcgggtggatcatttaaggtcaggagttggagaccagcctgggcaacat    3126
LOC105372935.clincRNA.NCBI.ref       gggaggccgagggggtggctcacttgaggtcaggagttggagaccagcctgggcaacat    3288
                                     ******* **** ****** *** ** *********************************

GGT5.NCBI.REF                        ggtgaaaccacgtctctactaaaaatacaaaaattagctgggcatggtagcgggtgcctg    3186
LOC105372935.clincRNA.NCBI.ref       ggtgaaaccatgtctctactaaaaatacaaaaattagctgggcatggtagcaggtgcctg    3348
                                     ********** **************************************** ********

GGT5.NCBI.REF                        taatcccagctacttgggaggctgagacaggagaatcacttgaacccaggaggcaaaggt    3246
LOC105372935.clincRNA.NCBI.ref       taatcccagatacttgggaggctgagacaggagaatcacttgaacccaggaggcaaaggt    3408
                                     ********* **************************************************

GGT5.NCBI.REF                        tgcagtaagccaagattgcaccactgcactccagcctgggtgacagagcaagacttggtc    3306
LOC105372935.clincRNA.NCBI.ref       ttcagtaagccaagattgcaccactgcactccagcctgggtgacagagcaagacttggtc    3468
                                     * **********************************************************

GGT5.NCBI.REF                        tcaggaaaaaaaaaaaaagaaagaaagaaaagtttatatttttgttctaatggttatctt    3366
LOC105372935.clincRNA.NCBI.ref       tca--aaaaaaaaaaaaagaaagaaagaaaagtttatatttttgttctaatggttatctt    3526
                                     ***  *******************************************************

GGT5.NCBI.REF                        aatatcttcattctataattatatgttttatataattataatagctatataagatataat    3426
LOC105372935.clincRNA.NCBI.ref       aatatcgtcattctataattatatgttttatataattataatagctatataagatataat    3586
                                     ****** *****************************************************

GGT5.NCBI.REF                        acccctagtatgttgtttttggatattctactcgttcctgatggttaatttatgtgtca    3486
LOC105372935.clincRNA.NCBI.ref       acccctagtatgttgtttttggatattctacttgctcctgatggttaatttatgtgtca    3646
                                     ********************************* * ************************

GGT5.NCBI.REF                        acttggctaagctctggtgccccgttgtttggtcaaatacttgtcaatatcttgctggga    3546
LOC105372935.clincRNA.NCBI.ref       acttggctaagctatggtgtcctgttgtttggtcaaatacttgtcaatatcttgctggga    3706
                                     ************ ***** ** **************************************

GGT5.NCBI.REF                        ggttatttcatagatgtgattaacactgacagtcagttgactttaggtaaaacagattac    3606
LOC105372935.clincRNA.NCBI.ref       ggttatttcatagatgtgattaacactgacagtcaattgactttaagtaaaacagattac    3766
                                     *********************************** ********* **************

GGT5.NCBI.REF                        ccaccataatatgggtgggccacctccaatcagttgaaggccgtaagaacaaaaactgag    3666
LOC105372935.clincRNA.NCBI.ref       ccaccataatatgggtgggccacctccaatcagttgaaggccttaagaacaaaaactgag    3826
                                     ******************************************* ****************
```

```
GGT5.NCBI.REF                 gtttcccagagaagcaggaattctgcttcaacactataacacacaaaccctgcctgagtt    3726
LOC105372935.clincRNA.NCBI.ref gtttcccagagaagcaggaattctgcttcaagactgtaacacacaaaccctgcctgagtt    3886
                              ******************************* *** ************************

GGT5.NCBI.REF                 tctggcctgctgactgctctacagatgttaggttccagacttcgagatcaactcttacct    3786
LOC105372935.clincRNA.NCBI.ref tctggcctgctgactgctctacagattttaggttccagacttcgagatcaactcttacct    3946
                              ************************** *********************************

GGT5.NCBI.REF                 gaatttatagcctgctggcttgccctacagatttt-aaacttgccagtccccaaaatcat    3845
LOC105372935.clincRNA.NCBI.ref gaatttatagcctgctggcttgccctacagattttaaaacttgctagtccccacaatcat    4006
                              *********************************** ******** ******** ******

GGT5.NCBI.REF                 gtgagccaattcctaaataaa----tctctatgtataacctattggtttagtttctctaa    3901
LOC105372935.clincRNA.NCBI.ref gtgagccaattcctaaataaatctctctatgtataatctattggtttagtttctctga    4066
                              *********************    ************* ******************* *

GGT5.NCBI.REF                 aaaaacttctatatccagtttcctggatgttaagtaatactgaaactagctagtaacctc    3961
LOC105372935.clincRNA.NCBI.ref aaaactttc-acatccagtttcctggttgttaagaattaccgaaactagctagtaacttc    4125
                              ****  *** * ************** ******* * *** **************** **

GGT5.NCBI.REF                 gtgtttttttttttttttttttttgagatggagttttgttcttgttgcacaggctggagt    4021
LOC105372935.clincRNA.NCBI.ref -----ttttttttttttttttttttgagacagagttttgctcttgttgcccaggctggaat    4180
                                   ***********************  ******** ********* ********* *

GGT5.NCBI.REF                 acaatagcacgatcttggctcaccgcaacctccacctcctgggttcaagcgattctcctg    4081
LOC105372935.clincRNA.NCBI.ref gcaatggcacaatctcagctcaccgcaacctccacttcctgggtccaagcaattctcctc    4240
                               **** **** ****  ****************** ******** ***** ********

GGT5.NCBI.REF                 cctcagcctcctgagtagctgggattacaggcatgcgccaccacacctggctaa-ttttg    4140
LOC105372935.clincRNA.NCBI.ref cctcagcctcctgagtagctgggattacaggcatgtgccaccatgcttggctaatttttg    4300
                              *********************************** *******  * ******* *****

GGT5.NCBI.REF                 tatttttggtagagacagggtttctccatgtgggtcaggctggtctcaaactcccgacct    4200
LOC105372935.clincRNA.NCBI.ref tatttttagtagagacagggcttctccatgttggtcaggctggtcttgaactcccaacct    4360
                              ******* ************ ********** **************  ******* ****

GGT5.NCBI.REF                 caggtgatctgcccgctttggcctcccaaagtgctgggattacaggcatgaactactgca    4260
LOC105372935.clincRNA.NCBI.ref caggtgatccg-ccgccttggcctcacaaagtgctggaattacaggcacgagccattgcg    4419
                              ********* * **** ******** *********** ********** ** * * ***

GGT5.NCBI.REF                 cccgtctcctagtaatttcttcttttccatgatgtgtctcttatctctaataatactttt    4320
LOC105372935.clincRNA.NCBI.ref cctggctcctagtaaattcttcttttctgtgatgtgtctcttacctctaataatactttt    4479
                              ** * ********** ***********  ************** ****************

GGT5.NCBI.REF                 cttcttaaagtctacttcattaaaaatagttatgctgggcatggtggctcatgcctgtaa    4380
LOC105372935.clincRNA.NCBI.ref cttcttaaagtctacttcattaaaaatagttatgctgggcatggtggctcatggctgtaa    4539
                              ***************************************************** ******

GGT5.NCBI.REF                 --ttggcactttg--ggaggtcaaggtgagtgggtcgctgaagcccaggagttcaagacc    4436
LOC105372935.clincRNA.NCBI.ref tcttggcactttgctggaggtcgaggtgggtggatcactgaagcccaggagttcaagacc    4599
                                ***********  ******* ***** **** ** ***********************

GGT5.NCBI.REF                 agcctgggcaacatggtgag-----------acaaaaagtacaaaaattagctgggtgtg    4485
LOC105372935.clincRNA.NCBI.ref aacctgggcaacatggcgagaccctgcctctacaaaaaatacaaaaattagctgggtgtg    4659
                              * ************** ***           ******* *********************

GGT5.NCBI.REF                 gctaatataattctaagttggcacacttgtagtcccagctacttgggaggctgagcgggg    4545
LOC105372935.clincRNA.NCBI.ref gctaatataattctaagttggcacacttgtagtcccagctacttgggatgctgaggtggg    4719
                              ************************************************ ******  ***

GGT5.NCBI.REF                 agaatcgcatgatgctagaagggagagattgctgtgagccaagatcacgtcgctgcactc    4605
LOC105372935.clincRNA.NCBI.ref agaatcgcttgagcctagaagggagagattgctgtaagccaagatcacatcactgcactc    4779
                              ******** ***  ********************* ************ ** ********

GGT5.NCBI.REF                 cggcctgggagacagagtgaggctctatctc-------------aaaaaaaaaaaaaaag    4652
LOC105372935.clincRNA.NCBI.ref cagcctgggagacagagtgaggctctatctccaaaaaaaaaaaaaaaaaaaaaaaaaaag    4839
                              * *****************************             ****************

GGT5.NCBI.REF                 ttatacagctttcttggttagtgcatgcatgatatatttttcattattttccacctttct    4712
LOC105372935.clincRNA.NCBI.ref ttatacagctttcttggttagtacatgcatgacatatttttcatgatcttccacctctct    4899
                              ********************** ********* *********** ** ******** ***

GGT5.NCBI.REF                 gtatccttacataaaaggca----ttgggttttactttatttccaattactttaattttt    4768
LOC105372935.clincRNA.NCBI.ref gtacccttatataaaaggcattagttgggttttactttattttcaattattttaattttt    4959
                              *** ***** **********    ****************** ****** **********

GGT5.NCBI.REF                 attgattgtccttttaaaagtaggtgatgatttatttgggttgaaacccaccaccaactt    4828
LOC105372935.clincRNA.NCBI.ref ----attgtccttttaaatgtaactaatgatttatttgggttgaaacccaccaccaattt    5015
                                  ************** ***  * ******************************* **

GGT5.NCBI.REF                 gttttccatgcctattctgtttcttcttatctcctttcacatcttgttttgcatttatta    4888
LOC105372935.clincRNA.NCBI.ref gttttccatgcctattctatttcttcttatctcctctcacatcttgttttggatttatta    5075
                              ****************** **************** *************** ********

GGT5.NCBI.REF                 tttttattattcaatttcctttttctctattagtttcctaactgtgcagtcttggggtta    4948
LOC105372935.clincRNA.NCBI.ref tttttattatttaatttcctccttctctattagtttcataactgtgcagtcttagagtta    5135
                              *********** ********  *************** *************** * ****

GGT5.NCBI.REF                 ttttaaaagatgacagtagattattttagagcttacaacatgcatccttcacttatcaaa    5008
LOC105372935.clincRNA.NCBI.ref ttttaaaagatgac--tggattattttagagcttacaacatgcatccttcccttatcaaa    5193
                              **************  * ******************************** *********

GGT5.NCBI.REF                 gtctaacatgagctagtactttttttttgtttatttgtttttcttgagatagagggagtc    5068
```

```
LOC105372935.clincRNA.NCBI.ref      gtctaacatgagctagtactttttgttg------------tggttgagatagagagagtc    5241
                                    ************************ ***            *  *********** *****

GGT5.NCBI.REF                       ttgctctgctgcccaggctggagtgcagtggagcaatcttggttcactgcaacctccacc    5128
LOC105372935.clincRNA.NCBI.ref      ttcctctgctgcccaggctggagtgcagtggagcaatcttggttcactgcaacctccact    5301
                                    ** ********************************************************

GGT5.NCBI.REF                       tcttgggttcaagcaattctcctgtctcagtctcctgagtagctgggaccactggtgtgc    5188
LOC105372935.clincRNA.NCBI.ref      tcttgggttcaagcaattctcctgcctcagtcacctgagtagctgggaccacaggtgtgc    5361
                                    ************************ ******* ******************* *******

GGT5.NCBI.REF                       accactatgcccggctaatttttgtattcttttttagtagagacagggtttcaccatgtt    5248
LOC105372935.clincRNA.NCBI.ref      accactatgcccggccaatttttgtattctttttcagtagagacagggtttcaccatgtt    5421
                                    *************** ****************** *************************

GGT5.NCBI.REF                       ggccaggctggtcttgaactcctcaccttaagagatctgcttacctcggcgtcctaaagt    5308
LOC105372935.clincRNA.NCBI.ref      ggccaggctggtcttgaactccggaccttaagagatctgcctacctcggcatcctaaagt    5481
                                    **********************  **************** ********* *********

GGT5.NCBI.REF                       gttgggattacaggcgtgagccaccacgcccagcctatgagttagtacttctatcctctt    5368
LOC105372935.clincRNA.NCBI.ref      gttgggattacaggcgtgagccaccgcgcccagcctatgagttagtacttctatcctctt    5541
                                    ************************* **********************************

GGT5.NCBI.REF                       cctagtcagtagaagaaccttggaacaggaactaaatttacccccagtgacttatatgct    5428
LOC105372935.clincRNA.NCBI.ref      cctagtcagtacaagaaccttggaacaggaactaaatttacccccagtgacttatatgct    5601
                                    *********** ************************************************

GGT5.NCBI.REF                       aatatttttgtgtattttaaatatatgtgtgtgcatagatgtatctgtgtgtttttttgtg    5488
LOC105372935.clincRNA.NCBI.ref      aatatttttgtgtattttaaatatgtgtgtgtgcatagatgtatctgtgtgtttttttgtg    5661
                                    ************************ ***********************************

GGT5.NCBI.REF                       tttttattcttatttatgttgagagtgtagagctatgtaagagtaaagagaattgtgtaa    5548
LOC105372935.clincRNA.NCBI.ref      tttttattcttatttatgttgagagtgtagagctatgtaagagtaaagagaattgtgtaa    5721
                                    ************************************************************

GGT5.NCBI.REF                       tgaagccccgagtatccattcaatttcaacaacaatcttatggccaagctcatttcatgt    5608
LOC105372935.clincRNA.NCBI.ref      tgaagccccgagtatccattcaatttcaacaacaatcttatggccaagctcatttcatgt    5781
                                    ************************************************************

GGT5.NCBI.REF                       atactctttcctgcttccctctaccctacattatttcagtgcaaatcccagatatataac    5668
LOC105372935.clincRNA.NCBI.ref      atactctttcctgcttccctctaccccacattatttcagtgcaaatcccagatatataac    5841
                                    ************************** *********************************

GGT5.NCBI.REF                       tttacccatacatatttcagtatgt----tttattttaagccccacaagatatcattttc    5724
LOC105372935.clincRNA.NCBI.ref      tgtaccaatacatatttcagtatgttttatttattttaaaccccacaagatatcattttc    5901
                                    * **** ******************    ********** ********************

GGT5.NCBI.REF                       tatactactataattttataccaataacgttcatttagatttacccacacgtttacctct    5784
LOC105372935.clincRNA.NCBI.ref      tatactactgtaattttataccaataacattcatttagatttacccacacgtttacc---    5958
                                    ********* ****************** ****************************

GGT5.NCBI.REF                       tctgttaccctttttttttttgagacagagtctcgctctgtcgcccaggctggagtgcagt    5844
LOC105372935.clincRNA.NCBI.ref      ----ctaccctccgggtcctgtttgaaaatcaagcccatgctcacaggcca---------    6005
                                         ******     *  **    * * **  ** *   * * *****

GGT5.NCBI.REF                       ggcacaatctcggctcactgcaagctctgcctcctgggttcacgccattctcctgcttca    5904
LOC105372935.clincRNA.NCBI.ref      ------------------------------------------------------------    6005

GGT5.NCBI.REF                       gcctcccgagtagctgggactacagtcacccgccaccacgcctggctaatttttttgtatt    5964
LOC105372935.clincRNA.NCBI.ref      ------------------------------------------------------------    6005

GGT5.NCBI.REF                       ttttagtagagacgggtttcactgtgttagccaggatggtctcgatctcctaacctcatg    6024
LOC105372935.clincRNA.NCBI.ref      ------------------------------------------------------------    6005

GGT5.NCBI.REF                       atacacccgcctcggcctcccaaagtgctgggattacaggcgtgagccaccacgcctggc    6084
LOC105372935.clincRNA.NCBI.ref      ------------------------------------------------------------    6005

GGT5.NCBI.REF                       caccctttattttatttataaaaatatctttgggaaaaaatatctttgggcacatggtc    6144
LOC105372935.clincRNA.NCBI.ref      ------------------------------------------------------------    6005

GGT5.NCBI.REF                       aaggatctcctgaggactgtgtcatgggctatatatatattatatatatatatatatatt    6204
LOC105372935.clincRNA.NCBI.ref      ------------------------------------------------------------    6005

GGT5.NCBI.REF                       atataatatatattatatatataatatatataatatatatatatataaaatatataatat    6264
LOC105372935.clincRNA.NCBI.ref      ------------------------------------------------------------    6005

GGT5.NCBI.REF                       atattatatatattatatatataatatatataatatatatatataaatatatatatatat    6324
LOC105372935.clincRNA.NCBI.ref      ------------------------------------------------------------    6005

GGT5.NCBI.REF                       atataatatattatatatatataaatatataatatatattatatatatatatatatattc    6384
LOC105372935.clincRNA.NCBI.ref      ------------------------------------------------------------    6005

GGT5.NCBI.REF                       catatatatacacacacatatatatatattccactttcacttttttttttttttttgagac    6444
LOC105372935.clincRNA.NCBI.ref      ------------------------------------------------------------    6005
```

```
GGT5.NCBI.REF                       ggtgtcttgctctgtcgcccaggctggagtatagtggcgtgatctcagctcactgcaact    6504
LOC105372935.clincRNA.NCBI.ref      ------------------------------------------------------------    6005

GGT5.NCBI.REF                       tctgcctcccgggttcaagtgattctcctgtctcagccacctgagtagctgggattccag    6564
LOC105372935.clincRNA.NCBI.ref      ------------------------------------------------------------    6005

GGT5.NCBI.REF                       gcatgagccaccacgcctggctaatttttgtattttttttttttgagatggggtctcact    6624
LOC105372935.clincRNA.NCBI.ref      -----------------------------attttttttctttttagagacagggtctcact    6036
                                                                  * ****** **** ****  **********

GGT5.NCBI.REF                       gtgtcgcccaggctgaagtgcaatggcacgatatctgctcactgcaacctccacctccct    6684
LOC105372935.clincRNA.NCBI.ref      ttgtcacccaagctggagtgcagtggtgcgattatagctcaatgcagcctccaattcctg    6096
                                     **** **** **** ****** ***  ****    ***** **** ******  ***

GGT5.NCBI.REF                       ggttcaagcagttcccctgcctcagtctcccaagtagctgggattacaacaggcgcatga    6744
LOC105372935.clincRNA.NCBI.ref      gactcaagggaccctcctgcctcagcctgccaagtagcttggactatagctg--------    6148
                                    *  *****     * ********** ** ********** *** ** * * *

GGT5.NCBI.REF                       caccatgccctgctaatttttttgtatttgtagtagagacagagtttcaccatattcgcc    6804
LOC105372935.clincRNA.NCBI.ref      -------------tgtgttttcttattattttgtagacatggggtctggctatgttgtcc    6195
                                                 *   **** *   * * * ***** *  * ** *  * ** **  **

GGT5.NCBI.REF                       agactggtctcaaac-tcctgacctcaagcaatttgcctgccttggcctcccaaagtgct    6863
LOC105372935.clincRNA.NCBI.ref      aggctattctcaaaattcccggcctcgagcaatcctcctgcctcggcctctcaaag-gtt    6254
                                    ** **  *******  *** * **** ******   ******* ****** ***** * *

GGT5.NCBI.REF                       gagattacaggcgtgagtcaccgcgcctggc-----------------------------    6894
LOC105372935.clincRNA.NCBI.ref      gggattacaggtgtgaggcaaggcacccagctcagccacagagccctgttgcatctctct    6314
                                    * ********* ***** **  ** **  **

GGT5.NCBI.REF                       ------------------------------------------------------------    6894
LOC105372935.clincRNA.NCBI.ref      tactaggagcaagagctgactgccccctcatccccattccagagtgttggggctgtgttc    6374

GGT5.NCBI.REF                       ------------------------------------------------------------    6894
LOC105372935.clincRNA.NCBI.ref      agccgaggccgggccactggcatggcccagggagcgggatcattcactgctgccccaaat    6434

GGT5.NCBI.REF                       ------------------------------------------------------------    6894
LOC105372935.clincRNA.NCBI.ref      ctgagatcattccaccttgacaagacttcctcatccaatccctttacttgacagctgggg    6494

GGT5.NCBI.REF                       ------------------------------------------------------------    6894
LOC105372935.clincRNA.NCBI.ref      aaaccaatgcgcacagagcacccccagctcactcggggtctcagagctgatccatgagca    6554

GGT5.NCBI.REF                       ------------------------------------------------------------    6894
LOC105372935.clincRNA.NCBI.ref      gaggctgagatcctgggatcttgtcccccagccgccctgcaagcttactccctttctgct    6614

GGT5.NCBI.REF                       ------------------------------------------------------------    6894
LOC105372935.clincRNA.NCBI.ref      ggaagagatggggccggacctcgaccagcagccctggcctggacatgactgtgctcaccc    6674

GGT5.NCBI.REF                       -----------------------------------------ctttttttttttttttttt    6913
LOC105372935.clincRNA.NCBI.ref      aggtattgaggccgagatgccccggcatcatatgtttttctctctttttttctttttttt    6734
                                                                             ** ******* ********

GGT5.NCBI.REF                       tgagacggagcctcactctgtcccccaggctggggtgtggtgggaagatctcggcttcct    6973
LOC105372935.clincRNA.NCBI.ref      tgagacagtatctcactctgtcacccaagctggagtgcagtggcatgatattggctcact    6794
                                    ****** *   *********** **** ***** ***  **** * *** * ****  **

GGT5.NCBI.REF                       gcaacctccacctgctgggttcaaatgtttcttgtgcctcagcctcccaagtagatggga    7033
LOC105372935.clincRNA.NCBI.ref      gcaacctctgcctcc-cgcttaaagtgattctcctgcctcagtcttccaagtagctgggc    6853
                                    ********  *** *  * ** ** ** ****  ******** ** ******** ****

GGT5.NCBI.REF                       ttacaggtgcccacaaccacacctggctaatttttgtatttttggtagagatggggtttt    7093
LOC105372935.clincRNA.NCBI.ref      ctacaggcttgtaccaccacacctgactaatttttgtatttttactagagacgggg-ttt    6912
                                     ******     ** ********** *****************  ****** **** ***

GGT5.NCBI.REF                       aaccacgttggccaggctggtctcgaactcctgacctcaggtgacctacccaccttggcc    7153
LOC105372935.clincRNA.NCBI.ref      ccccatgttggccaggctcgtgtcgaactcctgacctcaggtgatccacctgccttggcc    6972
                                      *** ************ ** ********************** * ***  ********

GGT5.NCBI.REF                       ttccaaagtgatgggattacaggcgtgagccaccactcccagcctccatttttaatagat    7213
LOC105372935.clincRNA.NCBI.ref      tcccaaagtgctaggattacaggcatgagccatggcgtcac-------------------    7013
                                    * ******** * *********** *******   *  *

GGT5.NCBI.REF                       gacagttctttgataaaattcttatttctttatatccttgaatatagatacaaagtactt    7273
LOC105372935.clincRNA.NCBI.ref      ------------------------------------------------------------    7013

GGT5.NCBI.REF                       attttaaagtacatggtctgccaatttcataatctggagatcctatggacctttttaaaa    7333
LOC105372935.clincRNA.NCBI.ref      ------------------------------------------------------------    7013

GGT5.NCBI.REF                       gttgtctgtgctttctcatgagctttgttcctgctgtcttatttccttgtttgcttggtt    7393
LOC105372935.clincRNA.NCBI.ref      ------------------------------------------------------------    7013
```

```
GGT5.NCBI.REF                       gtttttaatttggcactggagagtatgtataaaaattgttagaggccgggtgcggtggct    7453
LOC105372935.clincRNA.NCBI.ref      ----------------------------ttaaatgtagtgagaggccgggcaagg-ggct    7044
                                                                 ****  * ** **********   ** ****

GGT5.NCBI.REF                       cacgcttgtaatcccagcactttgggaggccaaggcaggcagatcac--gaggtcaggag    7511
LOC105372935.clincRNA.NCBI.ref      catgcctgtaatcccagtgctttgagaggacgaggctgtcagatcacctaaggtcaggag    7104
                                    ** ** ***********  ***** **** * **** * ********   **********

GGT5.NCBI.REF                       tacaagaccagcctggccaacacagtgaaaccctgtctctactgaaaa--tacaaaaatt    7569
LOC105372935.clincRNA.NCBI.ref      ttcgagaccagcctggccaacatggtgaaactgtgtctctacaaaaaaatagaaaaaaaa    7164
                                    * * ******************  *******  *********  ****     *****

GGT5.NCBI.REF                       agctctgcgtgatggctggtggctgtaatcccagctacttgggaggctgagacaggagaa    7629
LOC105372935.clincRNA.NCBI.ref      atccctgcgtggtggcaagtatctgtagtcccagttactcaggaggctgaggcatgagaa    7224
                                    * * ******* ****  **  ***** ****** ****  ********** ** *****

GGT5.NCBI.REF                       ttgcttgaacccaggaggtgaaggttgcagtgagccgagatcgaggcactgcactccagc    7689
LOC105372935.clincRNA.NCBI.ref      ttgcttaaacctcggaggcggaggctgcagtgagctgagatggcgccactgcactccagc    7284
                                    ****** ****  ***** * *** ********** ***** * * **************

GGT5.NCBI.REF                       ctggacaacagagtgagatgctgtctc-aaataaataaataaataaatataaaataattt    7748
LOC105372935.clincRNA.NCBI.ref      ctgggtgacagagcaagactttgtctctaaataattaaataaataaatatggccgagcat    7344
                                    ****   ******  ***   ****** ****** ***************     *   *

GGT5.NCBI.REF                       gaggcctagggatctgaaattctgggatctcctttatgcatttgagtgactgagatgatt    7808
LOC105372935.clincRNA.NCBI.ref      ggtgccttaggcctgtaat-----------------------------------------    7363
                                    *  ****  **      **

GGT5.NCBI.REF                       tgaagctggatccagtgctcctgagggctgctctatttctagttgactcctagagtaaga    7868
LOC105372935.clincRNA.NCBI.ref      ------------------------------------------------------------    7363


GGT5.NCBI.REF                       aacctgcaccccacgtgtggggcattatggcatcccctccctcagcacatgagtaggtca    7928
LOC105372935.clincRNA.NCBI.ref      -------cccaacactttgggaggctgaggcaggtggttcatgaggtcaggagcccgaga    7416
                                            **     * ****    *  ****     * * * **  ** ***   *  *

GGT5.NCBI.REF                       acagc-------------------------------------actgctctagaccaggtg    7951
LOC105372935.clincRNA.NCBI.ref      ccagcctggccaagatggtgaaacactgtctctactaaaaatacaaaaattagccagctg    7476
                                     ****                                     **     *   **** **

GGT5.NCBI.REF                       tggtggctcacgcctatagtcccagctactcaggagactgaggcaggaggattgcttcag    8011
LOC105372935.clincRNA.NCBI.ref      tggtggcaggcacctgtaatcccagctacttgggacactgaggcaggagaatcgcttga-    7535
                                    *******   * *** ** ***********  *** ************* ** **** *

GGT5.NCBI.REF                       gccaagaatttgagaccagccagagcaatatattaggttggtacaaaagtaattgcggtt    8071
LOC105372935.clincRNA.NCBI.ref      ------acctggaaggcagaggttgcaatgagccgagattgcaccgctgcac-------t    7582
                                          *  * **   ***     *****       * * * **    * *        *

GGT5.NCBI.REF                       tttgccattaaaagtaatggtgaccctgtctcagaaaaataaa--aagcaaaaaaaaaaa    8129
LOC105372935.clincRNA.NCBI.ref      ctagcctgggcgatggagcaagactccatctcaaataaataaattaataaatacagagca    7642
                                     * ***      *   *    *** *  ***** * *******  **  ** * * *  *

GGT5.NCBI.REF                       aaaaaagagagaaaagaaaaaaagaatccctgatctatttctcagcctcctcttcaggat    8189
LOC105372935.clincRNA.NCBI.ref      agattccatctcaaataaataaataaatg-------------------------------    7671
                                    * *    *    *** *** *** **

GGT5.NCBI.REF                       tggccgtcaccttgaggggaatgctggccttgcctgtctccagccctgtacttctctgcc    8249
LOC105372935.clincRNA.NCBI.ref      ------------------------------------------------------------    7671


GGT5.NCBI.REF                       tcctatgcctttaagcacatgttttctatttgctgggcttttctagttctctgagggaag    8309
LOC105372935.clincRNA.NCBI.ref      ------------------------------------------------------------    7671


GGT5.NCBI.REF                       tgtggtctgaattacagtctcccattaccaggaagtctgatttacgtttaacagattttt    8369
LOC105372935.clincRNA.NCBI.ref      ------------------------------------------------------------    7671


GGT5.NCBI.REF                       tttttttttttgagatggagtctctctccgttgcccaggctggagtgcagtggtacaacc    8429
LOC105372935.clincRNA.NCBI.ref      ------------------------------------------------------------    7671


GGT5.NCBI.REF                       ttggctcactgcaagctccgacttccaggttcacaccattctcctgcctcagcctcctga    8489
LOC105372935.clincRNA.NCBI.ref      ------------------------------------------------------------    7671


GGT5.NCBI.REF                       gtagctgggactacaggcacccgccaccatgcccagctaatttttttgtatttttaataga    8549
LOC105372935.clincRNA.NCBI.ref      ------------------------------------------------------------    7671


GGT5.NCBI.REF                       gatggggtttcaccgtgttagccaggatggtctcaatctcctgacctcatgatctgcctg    8609
LOC105372935.clincRNA.NCBI.ref      ------------------------------------------------------------    7671


GGT5.NCBI.REF                       cctgggcctcccaaagtgctgggattacaggcatgagccaccgcgcccggccttaacaga    8669
LOC105372935.clincRNA.NCBI.ref      ------------------------------------------------------------    7671


GGT5.NCBI.REF                       ttatttttttaaaatgtaagggagcaaggccaacgctggggcctgggtaggaggccattg    8729
LOC105372935.clincRNA.NCBI.ref      ------------------------------------------------------------    7671
```

```
GGT5.NCBI.REF                       caataacccagctgagagaggttggtggcttggaccagtgtggtggagatggaggtagaa    8789
LOC105372935.clincRNA.NCBI.ref      ------------------------------------------------------------    7671

GGT5.NCBI.REF                       cctgcatccagagtcactggtgggttggatgtggggaaggaagaaagtgaccaatccagg    8849
LOC105372935.clincRNA.NCBI.ref      ------------------------------------------------------------    7671

GGT5.NCBI.REF                       atggctccagggtgtaggcgctgagcaaaagggggagtggttgtgtcaatttctaagaca    8909
LOC105372935.clincRNA.NCBI.ref      ------------------------------------------------------------    7671

GGT5.NCBI.REF                       acgaggactaggagaggagccatttcgcagggacggttggaaggaaaatcaaaagtacag    8969
LOC105372935.clincRNA.NCBI.ref      ------------------------------------------------------------    7671

GGT5.NCBI.REF                       tttaggtcatggtatgtttgaaatgtttcttagacacccagagtgggtaccttgtgcata    9029
LOC105372935.clincRNA.NCBI.ref      ------------------------------------------------------------    7671

GGT5.NCBI.REF                       gctgggtataagtgtctcgagtgtagggagaggactggcggagttaaagtcggggagtta    9089
LOC105372935.clincRNA.NCBI.ref      ------------------------------------------------------------    7671

GGT5.NCBI.REF                       ccatcacacaggttgcatctcatgccatgagcctggatggactcacccagggagaagata    9149
LOC105372935.clincRNA.NCBI.ref      ------------------------------------------------------------    7671

GGT5.NCBI.REF                       tagatgcacaaggaccatggaccaagcctggggcccccaacatcaggagaccagggaggt    9209
LOC105372935.clincRNA.NCBI.ref      ------------------------------------------------------------    7671

GGT5.NCBI.REF                       gggacgaaaaccaaagcctggcagggtgggcgaggtgggtgcagggacctggcccattcc    9269
LOC105372935.clincRNA.NCBI.ref      ------------------------------------------------------------    7671

GGT5.NCBI.REF                       ctcttggggctttggtttccccatctgtaaccatttaccccattttgccctggaagccaa    9329
LOC105372935.clincRNA.NCBI.ref      ------------------------------------------------------------    7671

GGT5.NCBI.REF                       gacaactgactaggctggaagcagacaggttggggaagcaagggtgcactccatttagtt    9389
LOC105372935.clincRNA.NCBI.ref      ------------------------------------------------------------    7671

GGT5.NCBI.REF                       aggaagtgaggcctttgtctgggatgggagtttaaagtgttttttcccccagtttgtcat    9449
LOC105372935.clincRNA.NCBI.ref      ------------------------------------------------------------    7671

GGT5.NCBI.REF                       ttggctttgtttatggtgttttattcttataaaaattagtgtttatgcctgacttgttta    9509
LOC105372935.clincRNA.NCBI.ref      ------------------------------------------------------------    7671

GGT5.NCBI.REF                       tttcattttctggctttgtgctctctttcctgttccaagattacatgtgtccctgtggtg    9569
LOC105372935.clincRNA.NCBI.ref      ------------------------------------------------------------    7671

GGT5.NCBI.REF                       cccatgtgtgccacgtgtgggcccagagagggacatgtggctcaggagctggggtgggtt    9629
LOC105372935.clincRNA.NCBI.ref      ------------------------------------------------------------    7671

GGT5.NCBI.REF                       tgtgtccagatggacatgctaggtcaggacagacacctgagccaaaggagggtgctaggg    9689
LOC105372935.clincRNA.NCBI.ref      ------------------------------------------------------------    7671

GGT5.NCBI.REF                       ccagaggtcccacggggtgggtgcagggacccaggtccattccctctttgggccttggtt    9749
LOC105372935.clincRNA.NCBI.ref      ------------------------------------------------------------    7671

GGT5.NCBI.REF                       tccccatctgcaactggggcttaaatgctggcttctggccgggcacagtggctcacgcct    9809
LOC105372935.clincRNA.NCBI.ref      -----------------------------------------------------tacacct    7678
                                                                                          ** ***

GGT5.NCBI.REF                       gtaatcccagcactttgggaggccgaggtgagcagatcatctgatgtcgggagtttgaga    9869
LOC105372935.clincRNA.NCBI.ref      gtaatcctagcactttgggaggctaagacaggtcgatcacctgaggtcaggagttcgaga    7738
                                    ******* ***************  **    *  ***** **** *** ****** ****

GGT5.NCBI.REF                       ccagcctgaccaacatggagaaacctcgtctctactaaaaatac-aaaattagtcaggcg    9928
LOC105372935.clincRNA.NCBI.ref      ccagcctgaccaatatggcaaaactccatctctactaaaaatacaaaaattagccgggcg    7798
                                    ************* ****  ****  * **************** ******** * ****

GGT5.NCBI.REF                       tggtggtgcatgcctgtaatcccagctactcggcaggctgaagcaggagaatcgcttgaa    9988
LOC105372935.clincRNA.NCBI.ref      ttttgacgtgtgcctgtagtcccagctacttgggaggctgagacaagagaattgcttgaa    7858
                                    *  **  *  ******** *********** ** *******  ** ****** *******

GGT5.NCBI.REF                       tccgggagg-cgaggttgcaatgagccaagatagtgtcattgcactccagcctgggcaag    10047
LOC105372935.clincRNA.NCBI.ref      cccaagaggtggaggttgcagtgagccgagatctcggc--tgcacttcagcctgggtga-    7915
                                     **  ****  ********* ****** ****   * *  ****** *********  *

GGT5.NCBI.REF                       aagattgaaactctgtctcaaaaaaaaaaaaaaaaaaaaaaaaaaagctggcttcttgagg    10107
LOC105372935.clincRNA.NCBI.ref      cagagtgagactctgtctcaaaaggaataaataaaatacaaagtaa--------------    7961
                                     *** *** **************  ** *** **** * ***  *

GGT5.NCBI.REF                       tgctggggaggaaggattcctgggtgttagggaagaggttgacctcagtgagctgtgcag    10167
```

```
LOC105372935.clincRNA.NCBI.ref      ------------------------------------------------------------    7961

GGT5.NCBI.REF                       gcctgtgccccagggacagggatcatcataatgtaggccaggctgaccagccctcagtgc    10227
LOC105372935.clincRNA.NCBI.ref      -----------------------------aaaaaaatgtagtaagattgcagagtcgtgc    7992
                                                                 **   *    **   **          ****

GGT5.NCBI.REF                       taaagggcccacctcttgctgcccatggacagatggggaggggtaggccagagcattt--    10285
LOC105372935.clincRNA.NCBI.ref      cgcagaagcgtgctggtcctatccatgtagtgaaggctgatttcatacacaaatgtcaga    8052
                                       **   *   **  * **  ***** *  ** **        *  *   *   *

GGT5.NCBI.REF                       --attttctttctttcttttcttttcttttttttttctttttttgaggcagtcttgctttgt    10343
LOC105372935.clincRNA.NCBI.ref      agaacttttcttttctttttcttttcttttttttttttttgagacggagtctcgctctgt    8112
                                      *  ** * * **  ******************* ****  ** * ***** *** ***

GGT5.NCBI.REF                       tgcccaggctggagtgcagtggcatgatttcagctcactgcaacctctgcctgctgggtt    10403
LOC105372935.clincRNA.NCBI.ref      cacccaggctggagtgcagtggtgtgatctaggctcactgcaagctctgcctccctggtt    8172
                                      ********************  **** *  *********** ******** *  ****

GGT5.NCBI.REF                       caagcaattctcctgcctcagcctcctgcgtagctgggattacaggtgcccgccatcaca    10463
LOC105372935.clincRNA.NCBI.ref      tatgccattctcctgcctcagcctcctcagtagctgggactacaggcgcctgccacctag    8232
                                     * ** *********************  ********** ****** *** **** *

GGT5.NCBI.REF                       ttcagctaa--tttttgtatttttagtagagacggggtttcaccatgttggccaagctgg    10521
LOC105372935.clincRNA.NCBI.ref      cccagctaatttttttgtacttttagaggagatggggtttcaccgcgttagccaggatag    8292
                                      *******  ******** ******  **** ***********  *** **** * * *

GGT5.NCBI.REF                       tcttgaactcctgacctcaagtgatctatccgcctcggcctcccaaagtgcagggattat    10581
LOC105372935.clincRNA.NCBI.ref      tctcaatctcctgacctc--gtgatccgcccgtctcggcctcccaaagtgctgggattac    8350
                                    ***  * ***********  ******   *** ****************** *******

GGT5.NCBI.REF                       aggcatgagccaccacacccagcctaggccagagaatttccataggctcagagtcccaag    10641
LOC105372935.clincRNA.NCBI.ref      aggcatgagccaccacacccggcctt----------------------------------    8376
                                    ******************** ****

GGT5.NCBI.REF                       agaagttttggggtccatcagctcaagccctatgccctgctggcagggaaggatcctggg    10701
LOC105372935.clincRNA.NCBI.ref      ------------------------------------------------------------    8376

GGT5.NCBI.REF                       atcacctgccaaaggccagccttgaagtagttgtggccagagtgtccccagtgagatggg    10761
LOC105372935.clincRNA.NCBI.ref      ------------------------------------------------------------    8376

GGT5.NCBI.REF                       gagtggagtcccagggtgggctcctgaggccactgtgagggactgagaggtggccgaggc    10821
LOC105372935.clincRNA.NCBI.ref      ------------------------------------------------------------    8376

GGT5.NCBI.REF                       ctcgttctgcctgtagcatatgagctggtgctgctgggaacaatggcaacagaagtactt    10881
LOC105372935.clincRNA.NCBI.ref      ------------------------------------------------------------    8376

GGT5.NCBI.REF                       tgcaaactgagaagcaccttgtatatggcaaaggtcagtgtaaatggcatcacactttgc    10941
LOC105372935.clincRNA.NCBI.ref      ------------------------------------------------------------    8376

GGT5.NCBI.REF                       aaccgtagtgtgttgtggacctgtgagcagctggatttgatggggaagcctggttgtgtt    11001
LOC105372935.clincRNA.NCBI.ref      ------------------------------------------------------------    8376

GGT5.NCBI.REF                       ctgcaacctaatctgcggtttggtggcagccgacgtgccgcctccgtcaccattctccaa    11061
LOC105372935.clincRNA.NCBI.ref      ------------------------------------------------------------    8376

GGT5.NCBI.REF                       tgagtgccagctgagcaagcctggtggggaggcaggggggaggggagccccacagcgactc    11121
LOC105372935.clincRNA.NCBI.ref      ------------------------------------------------------------    8376

GGT5.NCBI.REF                       tgtgccatgtcccctagagccatcctccagcagcagggctcacccgtggatgccaccatc    11181
LOC105372935.clincRNA.NCBI.ref      ------------------------------------------------------------    8376

GGT5.NCBI.REF                       gcggctctggtctgcaccagcgtcgtcaaccctcagagcatgggcctgggcggaggggtc    11241
LOC105372935.clincRNA.NCBI.ref      ------------------------------------------------------------    8376

GGT5.NCBI.REF                       atcttcaccatctacaatgtgacaacaggtgggcccacatgtgaactgccatggggaaga    11301
LOC105372935.clincRNA.NCBI.ref      ------------------------------------------------------------    8376

GGT5.NCBI.REF                       tgggcttgtccacaccactgcgtaaccccttctccagccagtcccatcaactcaagcccg    11361
LOC105372935.clincRNA.NCBI.ref      ------------------------------------------------------------    8376

GGT5.NCBI.REF                       tttctgggtgttcttgggccccctgccctgccccatttgcctcagctgtgcaaagccctt    11421
LOC105372935.clincRNA.NCBI.ref      ------------------------------------------------------------    8376

GGT5.NCBI.REF                       gtgtcaactcctgtccctgtgttagcccagagtccatctcataatgcagagatggaaact    11481
LOC105372935.clincRNA.NCBI.ref      ------------------------------------------------------------    8376

GGT5.NCBI.REF                       gaggcctagagcaggccaggggctgtctgcagggtccatgaccactcacctgccctgccc    11541
LOC105372935.clincRNA.NCBI.ref      ------------------------------------------------------------    8376
```

```
GGT5.NCBI.REF                     tactccagggaaggtggaggtcatcaatgcccgggagacggtgccggccagccacgcccc    11601
LOC105372935.clincRNA.NCBI.ref    ------------------------------------------------------------    8376

GGT5.NCBI.REF                     gagcctgctggaccagtgtgcacaggctctgccactgggcacaggtgacgccccatggag    11661
LOC105372935.clincRNA.NCBI.ref    ------------------------------------------------------------    8376

GGT5.NCBI.REF                     gtcccccactcccacccccccaggccactaggactaatcagaacacccccgcaaaggagca    11721
LOC105372935.clincRNA.NCBI.ref    ------------------------------------------------------------    8376

GGT5.NCBI.REF                     gcgcctcacggacaggctgtgactccctggcacgggggacacggagcctgtggggaacc    11781
LOC105372935.clincRNA.NCBI.ref    ------------------------------------------------------------    8376

GGT5.NCBI.REF                     cccagccccaacctgcagggattgagaaggtccccccttgggaacgggctctctgaggtga    11841
LOC105372935.clincRNA.NCBI.ref    ------------------------------------------------------------    8376

GGT5.NCBI.REF                     tccagggagagggaccaggctggacaaaggcctggagggttcagtggagatgggttggcg    11901
LOC105372935.clincRNA.NCBI.ref    ------------------------------------------------------------    8376

GGT5.NCBI.REF                     gggcagggtggggctcctggggccagtttgtgtagggccccttgggcttggccatggact    11961
LOC105372935.clincRNA.NCBI.ref    ------------------------------------------------------------    8376

GGT5.NCBI.REF                     ttgtggggagccacaggtgatttgagcagggagggacgtggtcgggtgtgggctccagaa    12021
LOC105372935.clincRNA.NCBI.ref    ------------------------------------------------------------    8376

GGT5.NCBI.REF                     agctcactctggctgctgtaactggggggccacatgtaggggtgatggcaaacctgggaa    12081
LOC105372935.clincRNA.NCBI.ref    ------------------------------------------------------------    8376

GGT5.NCBI.REF                     ggcctggaagccacagggttgagagctgggatgtgccatgcaggggcccagtggatcggg    12141
LOC105372935.clincRNA.NCBI.ref    ------------------------------------------------------------    8376

GGT5.NCBI.REF                     gtgcccggggagctccgtggctatgccgaggcccaccgccgccatggccgcctgccctgg    12201
LOC105372935.clincRNA.NCBI.ref    ------------------------------------------------------------    8376

GGT5.NCBI.REF                     gcgcagctgttccagcccaccatcgcgctgctccgagggggcatgtggtggcccctgtc    12261
LOC105372935.clincRNA.NCBI.ref    ------------------------------------------------------------    8376

GGT5.NCBI.REF                     ctcagccgtttcctgcacaacagcatcctgcggccttccttgcaggcgtcaaccctgcgg    12321
LOC105372935.clincRNA.NCBI.ref    ------------------------------------------------------------    8376

GGT5.NCBI.REF                     tgagccccacatggtggccctgggttcctgggttcaaggccatatcctgtctgggcccag    12381
LOC105372935.clincRNA.NCBI.ref    ------------------------------------------------------------    8376

GGT5.NCBI.REF                     tccacaccccagctctgcctcagtccctcttacactggaggattgaccctcggggtgctg    12441
LOC105372935.clincRNA.NCBI.ref    ------------------------------------------------------------    8376

GGT5.NCBI.REF                     gccaggacaaagagtacttttcatggcccttccagctccccgtggcagagcctaacttgg    12501
LOC105372935.clincRNA.NCBI.ref    ------------------------------------------------------------    8376

GGT5.NCBI.REF                     ggttaggctgcggggagtttattgcccaagtgataggactccaaggtgtggatttgggct    12561
LOC105372935.clincRNA.NCBI.ref    ------------------------------------------------------------    8376

GGT5.NCBI.REF                     ctgccacccgccctggggccccggcctccttcttcctctctctggcatggcaggagggct    12621
LOC105372935.clincRNA.NCBI.ref    ------------------------------------------------------------    8376

GGT5.NCBI.REF                     tcaactgccactctcacctgccccacccagcccactgacaggccctcccaggagtgagat    12681
LOC105372935.clincRNA.NCBI.ref    ------------------------------------------------------------    8376

GGT5.NCBI.REF                     gacaggccctaggtggacccaggccccactgggaaatttgccttccctggaccagggcca    12741
LOC105372935.clincRNA.NCBI.ref    ------------------------------------------------------------    8376

GGT5.NCBI.REF                     tgagctgggtgagtggtgtcagtcccaccacctcctagagaatcagttccccggtgtcca    12801
LOC105372935.clincRNA.NCBI.ref    ------------------------------------------------------------    8376

GGT5.NCBI.REF                     ccccagtgcctcgggaccctgggccagggcgccccccactgtagggtcctgaggactcca    12861
LOC105372935.clincRNA.NCBI.ref    ------------------------------------------------------------    8376

GGT5.NCBI.REF                     cccatgagagccccggaagagtgtggctgcctctgggacctggccccaccctgaggctcc    12921
LOC105372935.clincRNA.NCBI.ref    ------------------------------------------------------------    8376
```

```
GGT5.NCBI.REF                   ccccacccccccagccagctcttcttcaacgggacagaacccctgaggcctcaggacccac    12981
LOC105372935.clincRNA.NCBI.ref  ------------------------------------------------------------    8376

GGT5.NCBI.REF                   tcccatggcctgcactggccaccaccctggagaccgtggccacagagggcgtggaggtct    13041
LOC105372935.clincRNA.NCBI.ref  ------------------------------------------------------------    8376

GGT5.NCBI.REF                   tctacacggggaggctgggccagatgctggtggaggacattgccaaggaaggtcagcctc    13101
LOC105372935.clincRNA.NCBI.ref  ------------------------------------------------------------    8376

GGT5.NCBI.REF                   cctgaggttccataccccatccctgctggaggaagagccaagttctgggcactgggggtt    13161
LOC105372935.clincRNA.NCBI.ref  ------------------------------------------------------------    8376

GGT5.NCBI.REF                   gagggcaggctccttcccaggccctgagggagtgcaggcagtgcccgggaccccgagagg    13221
LOC105372935.clincRNA.NCBI.ref  ------------------------------------------------------------    8376

GGT5.NCBI.REF                   ccatgcacctagctctgccggcccaaggacccctgtggtccaggacctcagagagcagag    13281
LOC105372935.clincRNA.NCBI.ref  ------------------------------------------------------------    8376

GGT5.NCBI.REF                   cctgcgggttagggccagcagaggggcgttaccgatggtcacggcaggtctgggtagccg    13341
LOC105372935.clincRNA.NCBI.ref  ------------------------------------------------------------    8376

GGT5.NCBI.REF                   agctgaccattctcacaggctctcataagccagctccctctcccatcttccctttctttt    13401
LOC105372935.clincRNA.NCBI.ref  ------------------------------------------------------------    8376

GGT5.NCBI.REF                   ccccagcctccctgcctctggtctctgtccacccctccacgcctggccccaaggggccag    13461
LOC105372935.clincRNA.NCBI.ref  ------------------------------------------------------------    8376

GGT5.NCBI.REF                   tcctgaggccactgcctacagacctaccatcatcctgtgtccacaaggcaaaagcatgag    13521
LOC105372935.clincRNA.NCBI.ref  ------------------------------------------------------------    8376

GGT5.NCBI.REF                   tggaatcccattgtgtgacctgacagcagtggctattcctccccgcctcagtttcccatc    13581
LOC105372935.clincRNA.NCBI.ref  ------------------------------------------------------------    8376

GGT5.NCBI.REF                   tgttcatggagccctctccttctcatcccagggagccagctgacgctgcaggacctggcc    13641
LOC105372935.clincRNA.NCBI.ref  ------------------------------------------------------------    8376

GGT5.NCBI.REF                   aagttccagcccgaggtggtggatgccctggaggtgcccctggggactataccctgtac    13701
LOC105372935.clincRNA.NCBI.ref  ------------------------------------------------------------    8376

GGT5.NCBI.REF                   tcaccaccgccgcctgcaggggggtgccattctcagctttatcctcaacgtgctaagaggt    13761
LOC105372935.clincRNA.NCBI.ref  ------------------------------------------------------------    8376

GGT5.NCBI.REF                   aaagcccctgcccagagccctggcgcccacccccctcccccgcacccccggcccccgggaa    13821
LOC105372935.clincRNA.NCBI.ref  ------------------------------------------------------------    8376

GGT5.NCBI.REF                   ctcagactccaagcccaagcccagccaaggcccagctcagcctcctcctccataaaccgg    13881
LOC105372935.clincRNA.NCBI.ref  ------------------------------------------------------------    8376

GGT5.NCBI.REF                   gttcacagatgcacaatatgaagacagagctgtggaaaccaatcaggatggtgggtttta    13941
LOC105372935.clincRNA.NCBI.ref  ------------------------------------------------------------    8376

GGT5.NCBI.REF                   gagctggcctccagctcgacccatacgtgaggctttgctgaagggcgtgatatcattcgt    14001
LOC105372935.clincRNA.NCBI.ref  ------------------------------------------------------------    8376

GGT5.NCBI.REF                   ccagagtcctcctcccactgggtgaccagtcccagacacagaagcaggtgggctgtgtgg    14061
LOC105372935.clincRNA.NCBI.ref  ------------------------------------------------------------    8376

GGT5.NCBI.REF                   gtggtgagctgcctgtcaggggcgtgcacctgggggagcgaagccagcctagcccaaggc    14121
LOC105372935.clincRNA.NCBI.ref  ------------------------------------------------------------    8376

GGT5.NCBI.REF                   agcctgcaggggggttctggcagtgctgaggcctggggctgcctgcgctggcttaatatt    14181
LOC105372935.clincRNA.NCBI.ref  ------------------------------------------------------------    8376

GGT5.NCBI.REF                   agtgtcctgtgtctgccattacaaatcaccacaagcttggtggcttaaaataacagaaat    14241
LOC105372935.clincRNA.NCBI.ref  ------------------------------------------------------------    8376

GGT5.NCBI.REF                   gtgtcctctcacagttctggaagccagaagcccgatatctaggtgtccacagggctgcat    14301
LOC105372935.clincRNA.NCBI.ref  ------------------------------------------------------------    8376
```

```
GGT5.NCBI.REF                   tccattgggggctccaggggagaatctgttctttgccttgttgacctaaataaaaaacag    14361
LOC105372935.clincRNA.NCBI.ref  ------------------------------------------------------------    8376

GGT5.NCBI.REF                   aacaaggctctcaaagaaaatgatgtttgttccagagtaggcatcgcggtaggaataccc    14421
LOC105372935.clincRNA.NCBI.ref  ------------------------------------------------------------    8376

GGT5.NCBI.REF                   atgctagggtaaactatgtgtgtattcagggaggtgaaggaagacaaaagttttcaaaga    14481
LOC105372935.clincRNA.NCBI.ref  ------------------------------------------------------------    8376

GGT5.NCBI.REF                   gaaaatgaggaagattatataactttttttgtttacaatcaatgataatttattgtacatt    14541
LOC105372935.clincRNA.NCBI.ref  -------------cttatatgcttttattgcatttgagcgtacctcttttacagcgaaga    8423
                                              ******   *** ***  *   * *     *  ****  *   *

GGT5.NCBI.REF                   ttatttttttcattgcattattattattattattattaattattgagacggtgtcttgct    14601
LOC105372935.clincRNA.NCBI.ref  tcttttttttaa------------------------------------------------    8435
                                *  ******* *

GGT5.NCBI.REF                   ctgtcgcctagggtagagcacagtggcacaatctcggttcattgcaaactctgcttcctg    14661
LOC105372935.clincRNA.NCBI.ref  ------------------------------------------------------------    8435

GGT5.NCBI.REF                   ggttcaagtgattctcctgactcagcctcccaagtagctgggattacaggcatgggccac    14721
LOC105372935.clincRNA.NCBI.ref  ------------------------------------------------------------    8435

GGT5.NCBI.REF                   cacacgccgctgattttatcttttcttttttttcctttttttttttttttcttttttagaca    14781
LOC105372935.clincRNA.NCBI.ref  -------------------------------------attttattttatttttagagatg    8458
                                                                      **** **** * ***  ***

GGT5.NCBI.REF                   gagtctcaatctgtcacccaggctagagtgcaatggcacgatctcagctcactgcaacct    14841
LOC105372935.clincRNA.NCBI.ref  gagtcttgctttgttgcccaggctggagcactgtggtgtgatcatagctcactgcagcct    8518
                                ******   * ***  ******** ***  *  ***   ****  *********** ***

GGT5.NCBI.REF                   ctgcctcccaggtacaagcaattctcctgcctcagtctcccaagtagctgggattacagg    14901
LOC105372935.clincRNA.NCBI.ref  tgaactcccgggcacaggtgatcctcccacctcagcctcctgaatagctgggactacagg    8578
                                    ***** ** *** *  ** ****  ****** ****  * ********* ******

GGT5.NCBI.REF                   cgcttgccacgacaccgagttaatttttgtatttttggtagagatggggttttgccacgt    14961
LOC105372935.clincRNA.NCBI.ref  catgcaccaccatgcctggcatattttaaagatgtttgtagagatgaggtctcgctatgt    8638
                                *     **** *  **  *   *****     * ** ********* *** * ** * **

GGT5.NCBI.REF                   tggtcaggctggtcttgaacttctgatgtcaggtgatccgcccacctcggcatcccaaag    15021
LOC105372935.clincRNA.NCBI.ref  tgc-caggcttgtctcaaactactgggctcaagccatccatccatttcagcctcccaaag    8697
                                **  ****** ****  **** ***   *** *  ****  ***  ** ** ********

GGT5.NCBI.REF                   tgctgggattacaggcatgagccaccgcgcccagatgatttttatctttttagtagagac    15081
LOC105372935.clincRNA.NCBI.ref  tgcttggattataggcatgagc--------------------------------------    8719
                                **** ****** **********

GGT5.NCBI.REF                   ggggttttaccatgttggccgggctggtcttgaactcttgaccttaactgacccacttgc    15141
LOC105372935.clincRNA.NCBI.ref  ------------------------------------------------------------    8719

GGT5.NCBI.REF                   ctcagcctcccaaagtgctgggattccaggcgtgagccacctctgcctggctattttatt    15201
LOC105372935.clincRNA.NCBI.ref  ---------------------------------------actgcgcctggccatcacact    8740
                                                                        **  ******* **   * *

GGT5.NCBI.REF                   tttatttcattagtttttggggaacaagtggtgttcggttgcatggaaaagttatttagt    15261
LOC105372935.clincRNA.NCBI.ref  gtttttttgtttgtttgt------------------------------------------    8758
                                 ** ***  ** **** *

GGT5.NCBI.REF                   ggtgatttctttttttttttcttttttttttgagatggaatctcactctgtggcccaggc    15321
LOC105372935.clincRNA.NCBI.ref  --------------------ttgtttgttttgagatggagtcttgctctgtcgcccaggc    8798
                                                    ** *** ************ ***  ****** ********

GGT5.NCBI.REF                   tggagagcagtggcatgatcttagctcactgcaacctctgcctctgaggttcaagcaatt    15381
LOC105372935.clincRNA.NCBI.ref  tggagtgcagtagtgggatctcacctcattgcaagctccgccttgtgggttcacgccatt    8858
                                ***** ***** *   ***** * **** ***** *** ****    ****** ** ***

GGT5.NCBI.REF                   ctccagtctcagcctcctgagtagctgggattacaggtgcacgctaccaagcctggttaa    15441
LOC105372935.clincRNA.NCBI.ref  ctcctgcctcagcctcctgagtagctgggattacaggcgcccgccaccacgcctggctaa    8918
                                **** * ****************************** ** *** **** ****** ***

GGT5.NCBI.REF                   ttaattaatttatttatttatttattttttttagtagagatggggtttcaccatgttggc    15501
LOC105372935.clincRNA.NCBI.ref  tt------------------ttttgtatttttagtagagacgggatttcaccgtgttagc    8960
                                **                  **** * ************* *** ******* **** **

GGT5.NCBI.REF                   caggctggtcttaaactcctgacttcaagtgatctgcctgcctcagcctcccaaagtgct    15561
LOC105372935.clincRNA.NCBI.ref  caggatggtctcgatctcctgac----cgtgatctgcccgcctcggcctctcaaagtgct    9016
                                **** ******  * ********     ********** ***** ***** *********

GGT5.NCBI.REF                   ggaattacaggcatgagccaccgcatctggtctttagtggtgatttctgagatcttgatg    15621
LOC105372935.clincRNA.NCBI.ref  gggattacaggcatgagccaataaatatttttataatcattaactaccgag---------    9067
                                ** ****************    ** *  *  * *    * * * * ***

GGT5.NCBI.REF                   cacccatcacacaagcagtgtacactgttcctagtgtgtagtcttttatccctcattccc    15681
LOC105372935.clincRNA.NCBI.ref  ------------------------------------------------------------    9067

GGT5.NCBI.REF                   ctcccacccttccccttgagttcccagagtccattagagcattttcttttcctctgaga    15741
```

```
LOC105372935.clincRNA.NCBI.ref      ------------------------------------------------------------    9067

GGT5.NCBI.REF                       cagagtctcgctctgtcgccaaggctggagtgcaatggtgcgtctcggttcactacaaac    15801
LOC105372935.clincRNA.NCBI.ref      ------------------------------------------------------------    9067

GGT5.NCBI.REF                       tctgcctcctgggttcaagcgattttcctgcctcagcctcccacgtagctgggattacag    15861
LOC105372935.clincRNA.NCBI.ref      ------------------------------------------------------------    9067

GGT5.NCBI.REF                       gcgcctgccaccacaactaattttttttttttgtattattagtaaagatggggtttcacc    15921
LOC105372935.clincRNA.NCBI.ref      ------------------------------------------------------------    9067

GGT5.NCBI.REF                       gtgttgaccaggctgctcttgaactcctgacctcaggtgatctgcctgccttggcctccc    15981
LOC105372935.clincRNA.NCBI.ref      ------------------------------------------------------------    9067

GGT5.NCBI.REF                       aaattgctgggattacaggtgtgagccaccatgctgggccccattatatcattccttctt    16041
LOC105372935.clincRNA.NCBI.ref      ------------------------------------------------------------    9067

GGT5.NCBI.REF                       ttttctggagatggagttttgctcttgtcacccaggctggagtggagtgcaattgcgcga    16101
LOC105372935.clincRNA.NCBI.ref      ------------------------------------------------------------    9067

GGT5.NCBI.REF                       tctcagctcactgcaacctctgcctcccggttcaagcgattctcctgcctcagcctcccg    16161
LOC105372935.clincRNA.NCBI.ref      ------------------------------------------------------------    9067

GGT5.NCBI.REF                       agtagctaggactacccatgtgtgctaccatgcccggctaatttttgtattattagagac    16221
LOC105372935.clincRNA.NCBI.ref      ------------------------------------------------------------    9067

GGT5.NCBI.REF                       agggtttcatcatgttggccagactgctcttgaactcctgacctcaggtgatccacctgc    16281
LOC105372935.clincRNA.NCBI.ref      ------------------------------------------------------------    9067

GGT5.NCBI.REF                       ctcggcctcccaaagtgttgggattataggcataagccaccatgcctggcccattatatc    16341
LOC105372935.clincRNA.NCBI.ref      ------------------------------------------------------------    9067

GGT5.NCBI.REF                       atttttattcctttgcgtcctcatagcttagctttggtatataactgtttcgggcctggc    16401
LOC105372935.clincRNA.NCBI.ref      ------------------------------------------------------------    9067

GGT5.NCBI.REF                       atggtggctcacgcctgtaatcccagcactttgggaggccaaggcaggcggatcatgagt    16461
LOC105372935.clincRNA.NCBI.ref      ------------------------------------------------------------    9067

GGT5.NCBI.REF                       tcaggagatcgagaccatcctgcctaacacagtgaaaccctgtctctactaaaaatacaa    16521
LOC105372935.clincRNA.NCBI.ref      ------------------------------------------------------------    9067

GGT5.NCBI.REF                       aaaattagccaggcgtggtggcgggcacctgtagtcccagttgcttgggaggctgaggca    16581
LOC105372935.clincRNA.NCBI.ref      ----------------------gagaagctggagagaaaaaaggtggatgaagttaaggc    9105
                                                          * * * *** **    *   * * *      * * *

GGT5.NCBI.REF                       ggagaatggtgtgaacccaggagacagagcttgcagtgagctgagatcaca----ccact    16637
LOC105372935.clincRNA.NCBI.ref      agagagactttgtaagtttcttagctgagcttttggagactgtatatcagagatcctatt    9165
                                     ****    *   **         * ******   * **    * **** *    * * *

GGT5.NCBI.REF                       gcactccagcctgggcaacagagtgagactctgtctcaaaaaaaaaaaaaaactgttttg    16697
LOC105372935.clincRNA.NCBI.ref      tgaggtgattttcggctacagagataggcctggtcatttgaaaaataacgga--------    9217
                                      *    *   * *** ******  ** *   ***     ***** **   *

GGT5.NCBI.REF                       aaatgatcatccttggctagtaggatgcatggaaaagtgataagggtggcactggacagg    16757
LOC105372935.clincRNA.NCBI.ref      ------------------------------------------------------------    9217

GGT5.NCBI.REF                       ccactcatgggcagatgtctttgcagaagtatttttttgtgtaagattgtgatggccttct    16817
LOC105372935.clincRNA.NCBI.ref      ------------------------------------------------------------    9217

GGT5.NCBI.REF                       tgtaaggttgtgagctgttgtgtttttgaggcagggtcttgctctgtcacctgggctgga    16877
LOC105372935.clincRNA.NCBI.ref      ------------------------------------------------------------    9217

GGT5.NCBI.REF                       gtgcagtggcactgtcaaggctcactgcagcctcaacctcctggactcaagcaatcctct    16937
LOC105372935.clincRNA.NCBI.ref      ------------------------------------------------------------    9217

GGT5.NCBI.REF                       cacctcagcctcccaaagttttgggattacaggcatgagccactgcacccagccgagaac    16997
LOC105372935.clincRNA.NCBI.ref      ------------------------------------------------------------    9217

GGT5.NCBI.REF                       ggggcacttgtgcacgagagtcctgtcttcatggcctttccctggctctatttttcagag    17057
LOC105372935.clincRNA.NCBI.ref      ------------------------------------------------------------    9217

GGT5.NCBI.REF                       ttttttttttttgttttgttttttttttttttttttgagacagagtcttgctctgttta    17117
LOC105372935.clincRNA.NCBI.ref      ------------------------------------------------------------    9217
```

```
GGT5.NCBI.REF                      ctaggctggagtgcagtggtgcaatcctgcctcactgcaacctcctcctcccgggttcaa    17177
LOC105372935.clincRNA.NCBI.ref     -ttaggtgaagcttgcgtttgaaatcctccttctacttctcatctttctctcttgtttat    9276
                                    *  * ** **        ** ****** * **       * ** * *** *  *** *

GGT5.NCBI.REF                      gcaattctccctcctcagcctccctagtagctggggttacaggcacccaccatcatgcct    17237
LOC105372935.clincRNA.NCBI.ref     tctgaacatccatcttagacaccc------------------------------------    9300
                                    *    *  **  ** ** * ***

GGT5.NCBI.REF                      ggataattttttgtatttttgtagagatggggtttcaccatgttagtcaggctggtc----    17293
LOC105372935.clincRNA.NCBI.ref     ----aatctttgtcactttgtggttgtcattgatttccctgttattgagattagaggttg    9356
                                       *** *****   ***** *   *      *  ** ***** * **  * *

GGT5.NCBI.REF                      ------------------------------------------------------------    17293
LOC105372935.clincRNA.NCBI.ref     gcagactttctctgtaaagggctggagagaaagtacttcagactttgtggtctgtgcagt    9416


GGT5.NCBI.REF                      ------ttgaactcctgacctcagcctcggcctcccaaagtgctgggactacaggtgtga    17347
LOC105372935.clincRNA.NCBI.ref     gtctgttgcaaccacttaactctgccctgtagagcaaaagcagccgtagacagtacatgg    9476
                                         *  ***  ** * *** ***  *     * ****     * *         **

GGT5.NCBI.REF                      gccactgtggctggctgccgctctatttgaaaattttcacagcctcttcccacttccgg    17407
LOC105372935.clincRNA.NCBI.ref     gcagatgagcatggctggggtccagttacatttacttgcaa-------------------    9517
                                   **   ** *  ******  *  *  **      * ** **

GGT5.NCBI.REF                      tggctgccagcattccttggcttgtggctgcctcactccagtctctgtctccatgatcat    17467
LOC105372935.clincRNA.NCBI.ref     ------------------------------------------------------------    9517


GGT5.NCBI.REF                      actggtttctcctctgctgtgtgtcctctcctctatgtgtctgtcttacaaggacactgt    17527
LOC105372935.clincRNA.NCBI.ref     ------------------------------------------------------------    9517


GGT5.NCBI.REF                      ggtcacattcagggcacatctagataatccaggatcatctcctcctctcaaaatctttaa    17587
LOC105372935.clincRNA.NCBI.ref     ----------------------------------------------------------aa    9519
                                                                                             **

GGT5.NCBI.REF                      cgtactttaggccgggtgtggtggctcatgcctgtaatcccagcactttgggaggctaag    17647
LOC105372935.clincRNA.NCBI.ref     agagggttggagactgggtgcgatctcccacctttaatcccggcactttgggaggccgag    9579
                                    *    ** *     * ***    ***   *** ******* **************  **

GGT5.NCBI.REF                      gagggtggatcacttgaggtcaggagttggagaccagcctggccaacacagtaaaacacc    17707
LOC105372935.clincRNA.NCBI.ref     gcaggaggatcacttcaagccaggagttcaagaccagcctgggcaacaaagcaagactcc    9639
                                   *  ** ********* * * ********  ************ ***** ** ** ** **

GGT5.NCBI.REF                      atctctactaaaaatacaaaaat----tagccgggcatgatggcctgtgcctgaagtcct    17763
LOC105372935.clincRNA.NCBI.ref     atctctacaaaaaaataaaaattattatagctaggcatggtggtacacacccgtagtcct    9699
                                   ******** *****   **** *    ****  ****** ***      ** * ******

GGT5.NCBI.REF                      agctacttgggagg------ctgaggcgggagaatcacttcaacccaggaggtggagatt    17817
LOC105372935.clincRNA.NCBI.ref     agctactcaggaagctaaaactgaggcaggagggtcagttgagcccaggagcacgaggct    9759
                                   *******  *** *      ******* ****  *** ** * ********   ***  *

GGT5.NCBI.REF                      gcagtaagctgagatcgtgccacacactccagcctgggtgacagaacaag-acttcctct    17876
LOC105372935.clincRNA.NCBI.ref     gtggtgagctattattgtgccagtgcactccagcctggtgacagagcaagaaccgtctca    9819
                                   *  ** ****   ** ******       *   *  ********* **** **   ***

GGT5.NCBI.REF                      ccaaaaaacacaaaaaaggcggggcatgatggcttacgcctgtaatcctagcactttggg    17936
LOC105372935.clincRNA.NCBI.ref     tcaaaaaataaacaaaaggctgggcacggtggctcacgcctgtaatccctgcactttggg    9879
                                    ******* * * ******* ***** * ***** *************  **********

GGT5.NCBI.REF                      aggccaaggcaggcagatcacttgaggccaggagttcaataccagcctgaacaacatggc    17996
LOC105372935.clincRNA.NCBI.ref     agactaaggtgggcagatca--tgaggtcaggagattgagaccatcctggctaacacggt    9937
                                   ** * ****  *********  ***** ****** *  * **** ****   **** **

GGT5.NCBI.REF                      gaaatcctgtctactaaaaatacaaaaataattagctgtacgtggtggcgaacacatgta    18056
LOC105372935.clincRNA.NCBI.ref     gaaaccctgtctctactaaaaatacaaaaagttagccgggcgtggtggtgggcgcctgta    9997
                                   **** *******     *** * * *** * ***** *  ******** *  * * ****

GGT5.NCBI.REF                      gtctcagctactcaggagactaaggaccaagaatcacttgaacccaggaggcagagattg    18116
LOC105372935.clincRNA.NCBI.ref     gtcccagccactcgggaggctgaggagggagaatcgtttgaacctgggaggcggaggttg    10057
                                   *** **** **** **** ** ****   ******  *******  ****** *** ***

GGT5.NCBI.REF                      cagtgagctgagactgcgccattgcactccagcctgggcaatagagtgagactctgtcta    18176
LOC105372935.clincRNA.NCBI.ref     cagtgagccaaggttgtgccactgcactctagcctgggctacagggcaagactccattaa    10117
                                   ********  **  ** **** ******* ********* * ** *  ******  *  *

GGT5.NCBI.REF                      aaaaaaaaaaaaaaaaaaaaaaaaatccttaacttacttttccacttaaggtattagtcac    18236
LOC105372935.clincRNA.NCBI.ref     aaaaaaaaaaaaaaaccagcaaaaaccaaacaaaacat----------------------    10155
                                   ***************  *  **** **  *    ** *

GGT5.NCBI.REF                      tcttgtgctgtataaggtaatatccacaggttttgggaattaggatgtgggtggatcttt    18296
LOC105372935.clincRNA.NCBI.ref     ------------------------------------------------------------    10155


GGT5.NCBI.REF                      ctgtggtggggggtgggggcaacattcaacccattacatagggtgaccccaaccaacctg    18356
LOC105372935.clincRNA.NCBI.ref     ------------------------------------------------------------    10155


GGT5.NCBI.REF                      tgccccaacctctctccagggttcaacttctcaacagagtctatggccaggcctgaaggg    18416
LOC105372935.clincRNA.NCBI.ref     ------------------------------------------------------------    10155
```

```
GGT5.NCBI.REF                       agggtgaacgtgtaccaccaccttgtagagacgctcaagtttgccaaggggcagaggtgg    18476
LOC105372935.clincRNA.NCBI.ref      ------------------------------------------------------------    10155

GGT5.NCBI.REF                       aggctgggggaccctcgaagccacccgaagctccaggtgaggttgctgaggttgctgggc    18536
LOC105372935.clincRNA.NCBI.ref      ------------------------------------------------------------    10155

GGT5.NCBI.REF                       tggtgggccgtcctcctccctggctcaggacttggcatgaaatgagggtcaggcctggta    18596
LOC105372935.clincRNA.NCBI.ref      ------------------------------------------------------------    10155

GGT5.NCBI.REF                       gggggaagttggagggatatgtatgtggttctaggccagggcaggactgaaagggatccc    18656
LOC105372935.clincRNA.NCBI.ref      ------------------------------------------------------------    10155

GGT5.NCBI.REF                       ggggtggcaggtacaggggtcaggtgcaggagtggcaccatatctcaaaggacctggagg    18716
LOC105372935.clincRNA.NCBI.ref      ------------------------------------------------------------    10155

GGT5.NCBI.REF                       gtgagcagagtctagacctagctgggcttgagggagacctggccacaaggtagaggacag    18776
LOC105372935.clincRNA.NCBI.ref      ------------------------------------------------------------    10155

GGT5.NCBI.REF                       actggaggtggcccccatgggggctgatctcatcctgcccttggttctgcggattctgcc    18836
LOC105372935.clincRNA.NCBI.ref      ------------------------------------------------------------    10155

GGT5.NCBI.REF                       tggcccctcactgaccctgccacctgcccacccaccccagaatgcctcccgggacctgct    18896
LOC105372935.clincRNA.NCBI.ref      ------------------------------------------------------------    10155

GGT5.NCBI.REF                       gggggagaccctggcccagctcatccgccaacagatcgatggccggggggaccaccagct    18956
LOC105372935.clincRNA.NCBI.ref      ------------------------------------------------------------    10155

GGT5.NCBI.REF                       cagccactacagcttggccgaggcctggggccacgggacaggcacgtcccatgtgtctgt    19016
LOC105372935.clincRNA.NCBI.ref      ------------------------------------------------------------    10155

GGT5.NCBI.REF                       gctgggggaggatggcagcgccgtggctgccaccagcaccatcaacacaccgtgcgtagg    19076
LOC105372935.clincRNA.NCBI.ref      ------------------------------------------------------------    10155

GGT5.NCBI.REF                       gcctgggggaaggcggatggcttcactcctcctctcctagacctgcacacccccagcccc    19136
LOC105372935.clincRNA.NCBI.ref      ------------------------------------------------------------    10155

GGT5.NCBI.REF                       atgtcccctcacttgtccccacggggcagcaccttgcttttgccctttttctcctcctct    19196
LOC105372935.clincRNA.NCBI.ref      ------------------------------------------------------------    10155

GGT5.NCBI.REF                       atttcaaaagaggcccccacccctgacatctctggctggaaaggctgctgctggggtggc    19256
LOC105372935.clincRNA.NCBI.ref      ------------------------------------------------------------    10155

GGT5.NCBI.REF                       cccgacccaagatttacctgggaatgggtagcctcactcagaagggtgccctgatgtggg    19316
LOC105372935.clincRNA.NCBI.ref      ----------------------aatgcatgttctctcttataaatgggagctaaacatgg    10193
                                                          ****  *   *** ** * **  * *  ** *    **

GGT5.NCBI.REF                       ggcacaggtgggtctttggggacccctcctgggtggtgccagggagagaatagcggcttc    19376
LOC105372935.clincRNA.NCBI.ref      ggactcattgacttaaagatggcaacaactgggaactgctggatggggag----------    10243
                                    **      **  *    *  * *  *  *****   ***  *   * **

GGT5.NCBI.REF                       agcatgcttcggggcagctgtaaaacgagggggtcctgcaaagcgtgcagggtaagtggt    19436
LOC105372935.clincRNA.NCBI.ref      ------------------------------------------------------------    10243

GGT5.NCBI.REF                       gtctggtgggagccccgggtcctagcccaggctcttctgcctccacggctgcagctttgg    19496
LOC105372935.clincRNA.NCBI.ref      ------------------------------------------------------------    10243

GGT5.NCBI.REF                       agcgatggtgtattcaccacggacaggcatcatcctcaacaacgagctcctggacttatg    19556
LOC105372935.clincRNA.NCBI.ref      ------------------------------------------------------------    10243

GGT5.NCBI.REF                       cgagcgatgcccccggggttccggcaccaccccctcacctggtgagaacaaagcttccca    19616
LOC105372935.clincRNA.NCBI.ref      ------------------------------------------------------------    10243

GGT5.NCBI.REF                       cccggggtccacaagggccccccaccaggggagaggagggagggggctgggctggggttg    19676
LOC105372935.clincRNA.NCBI.ref      --------------------------ggaggggaggggtgaaaggccaactgttggggag    10277
                                                               * ** **** * **  ** *     * ***  *

GGT5.NCBI.REF                       catgctaacccctggatgggtcactgcacttgccaagacgctgtttgctcagcagtgagt    19736
LOC105372935.clincRNA.NCBI.ref      tatgctca----tatccatgtgacaaacctgcacatgtgcccgctgaatctaaaataaaa    10333
                                     ***** *    *      ** **    **   ** *   * * *   **   * * *

GGT5.NCBI.REF                       ggagacagggtgggtggagctcccggaaggtgctggcccccagttccaggcgagcgttcc    19796
LOC105372935.clincRNA.NCBI.ref      gttgaaagtagatttaaaaaaccccaagagggctgg------------------------    10369
                                    *  ** **      *  *   ***  *  * *****
```

```
GGT5.NCBI.REF                     ccatcctccatggtgccctccatcttgatcaacaaagcccaggggtcgaagctagtgatt    19856
LOC105372935.clincRNA.NCBI.ref    --------------------------------------------------------gttt    10373
                                                                                        * **

GGT5.NCBI.REF                     ggcggggctggcggggagctcatcatctctgctgtggcccaggtgagtctggggctcctg    19916
LOC105372935.clincRNA.NCBI.ref    ggcttgtgtgtccatagcttgttaacctccgct---------------------------    10406
                                  ***  *  ** *       *  * * *** ***

GGT5.NCBI.REF                     gctcgagtgtctcctctctgggcagcatactgtctgactgtctctggagtggggatgtga    19976
LOC105372935.clincRNA.NCBI.ref    ------------------------------------------------------------    10406

GGT5.NCBI.REF                     gggctgatgtagggtagcagggtgccccctttctccctgaaaccctcatctctcccccag    20036
LOC105372935.clincRNA.NCBI.ref    ------------------------------------------------------------    10406

GGT5.NCBI.REF                     gccatcatgagcaagctgtggcttggctttgacctgagagcggccattgcagccccccatc    20096
LOC105372935.clincRNA.NCBI.ref    ------------------------------------------------------------    10406

GGT5.NCBI.REF                     ctgcatgtcaacagcaagggctgtgtggagtacgagcccaacttcagccaggtgaggctg    20156
LOC105372935.clincRNA.NCBI.ref    ------------------------------------------------------------    10406

GGT5.NCBI.REF                     aggtccgagctggatgcctagggcagagcccactccccaaatccgtgctgctcaaagcca    20216
LOC105372935.clincRNA.NCBI.ref    ------------------------------------------------------------    10406

GGT5.NCBI.REF                     cctgggaggaactcagtcactgagattcttaggccaggtacacttcaactttgggggcca    20276
LOC105372935.clincRNA.NCBI.ref    -------------------------------------ttagatattaactaatagaaaca    10429
                                                                      ** *  * ****    *   **

GGT5.NCBI.REF                     taggagttggggaccttgatgggtgaggctgtcagtggctccaggccagttctgtggcc    20336
LOC105372935.clincRNA.NCBI.ref    tagtgcttatcttcccaggccacctattttgttcctctccaaggtgatggatagatgaag    10489
                                  ***   **     **  *       *   ***   *  **     *   * *   **

GGT5.NCBI.REF                     tccaagacagagagcagggtattgtctatgctgctcccaggctgaggatctcagca-cct    20395
LOC105372935.clincRNA.NCBI.ref    gcctaatccagccgcctggaagtttgctgacgcttgtcctgtcacggattaatgaagcat    10549
                                   ** *  *     **  ** * * *     *   *  *  *    ****    * * * *

GGT5.NCBI.REF                     tggtctctggtctgtggttgatgccattttcagaagtgagttttcctggctggggcctc    20455
LOC105372935.clincRNA.NCBI.ref    tgtttctgatgaaggtttcatgccgctgtgctgatgtgtcttctcttctct--------    10601
                                  ** * **** *    * ** *****  * * * ** ***  ** ** *  **

GGT5.NCBI.REF                     tcagactctccctcatggtgactttttccttgtgtgatttgtaactaatatttgcaactt    20515
LOC105372935.clincRNA.NCBI.ref    ------------------------------------------------------------    10601

GGT5.NCBI.REF                     tatttgtgaaagaggttgtttttcctgtgacagtctgtaaagtgattttcttctggggca    20575
LOC105372935.clincRNA.NCBI.ref    -----------------------------ctaggcaggaaactgcatatcttctggttta    10632
                                                                 ** * * *** **  * ********   *

GGT5.NCBI.REF                     ctagggcattggtggtaatttttactatatatataatttttttttttttgagacagcat    20635
LOC105372935.clincRNA.NCBI.ref    catgaagatggagtgctaatggaaatgcccaaaaccctt---------------------    10670
                                  *  *   ** *   *  * *   * *    * *   **

GGT5.NCBI.REF                     ctcactctgttgcccaggctggagtgcagtgttgcgatcacagctcactacagccttgac    20695
LOC105372935.clincRNA.NCBI.ref    ------------------------------------------------------------    10670

GGT5.NCBI.REF                     atcctgggctcaagtgatccttcctcctcaggctcccaaggaactaggactacaggtgtg    20755
LOC105372935.clincRNA.NCBI.ref    ------------------------------------------------------------    10670

GGT5.NCBI.REF                     tgccaccacacctggatagtttttattttttttgtttttcgtagaaacagggtctctcacc    20815
LOC105372935.clincRNA.NCBI.ref    ------------------------------------------------------------    10670

GGT5.NCBI.REF                     atgttgcctaggctggtcttgaactcctgagctcaagcaatcttcccgccttggcctccc    20875
LOC105372935.clincRNA.NCBI.ref    ------------------------------------------------------------    10670

GGT5.NCBI.REF                     aaaatgctgggattataggcgtgagccaccatgcccagcctacaaacatttttttttgatt    20935
LOC105372935.clincRNA.NCBI.ref    ------------------------------------------------------------    10670

GGT5.NCBI.REF                     ccccacagaagcccatggtgcagctccaggggctgaattcttctggctgactccccctct    20995
LOC105372935.clincRNA.NCBI.ref    --------------------------------cagagattgacacgctgtcattttccat    10698
                                                                  * **  *   *  **** *     *  *

GGT5.NCBI.REF                     atccttagacagagactcagcttccttggagcatctttgggttgatggatgccctttgtt    21055
LOC105372935.clincRNA.NCBI.ref    ttccgttcctggatctacggagtcttctaagagattttgcaatgaggagaagcactgttt    10758
                                   *** *     **    * *  ** *   **    ****   *** *     *  *  **

GGT5.NCBI.REF                     aggacctatttaaaagattgatagtggccgggcatggtggctcatgcctgtaatcccagc    21115
LOC105372935.clincRNA.NCBI.ref    tcaaactatataactga-------------------------------------------    10775
                                     * **** ***  **

GGT5.NCBI.REF                     actttgggaggccgaggcgggtggatcacctgaggtcaggacttccaggccagcctggcc    21175
LOC105372935.clincRNA.NCBI.ref    ------------------------------------------------------------    10775

GGT5.NCBI.REF                     aacatggtaaaaccctgcctcaactaaaaatacaaaaattagccaggcgtggtggcacac    21235
```

```
LOC105372935.clincRNA.NCBI.ref      ----------------gccttatttataattagggatattatcaaaatatg---------    10810
                                                    **** *  ** ** **   * **** * *    **

GGT5.NCBI.REF                       acctgtaatcccagctactcgggaggctgaagcaggagaaacacttgaacccaggaggta    21295
LOC105372935.clincRNA.NCBI.ref      ------------------------------------------------------------    10810

GGT5.NCBI.REF                       gaggttgcagtgagccaagatcgtgacattgcactccagcctgggtgacaagaacaaaac    21355
LOC105372935.clincRNA.NCBI.ref      ------------------------------------------------------------    10810

GGT5.NCBI.REF                       tccatctcaaaaaaaaaaaaaaaaaaaaaaaaagattgatagcagctgaggttccagatc    21415
LOC105372935.clincRNA.NCBI.ref      ------------------------------------------------------------    10810

GGT5.NCBI.REF                       gtcaaagctgcctctcctgttatgagacttcttcggtctcaagtccaccttccaaagccc    21475
LOC105372935.clincRNA.NCBI.ref      ------------------------------------------------------------    10810

GGT5.NCBI.REF                       ttagaacctgagaaccatggccttagtccaggaacagagtgtagtcccaagagccttagt    21535
LOC105372935.clincRNA.NCBI.ref      ------------------------------------------------------------    10810

GGT5.NCBI.REF                       tttcagaccttgagactaaggacctcagcctgttaggctcaaactcttggagtttgcagg    21595
LOC105372935.clincRNA.NCBI.ref      ------------------------------------------------------------    10810

GGT5.NCBI.REF                       cctccctccgcatgtagggtccttcgcatgacaggaagaccctctcagggctctcgggtt    21655
LOC105372935.clincRNA.NCBI.ref      ------------------------------------------------------------    10810

GGT5.NCBI.REF                       ccagggatcttagatatggaggatttcaagatccagggcacatcctttggagccctcctc    21715
LOC105372935.clincRNA.NCBI.ref      ------------------------------------------------------------    10810

GGT5.NCBI.REF                       ccttgactctttttttattttttattttttttagacagtctcactctgtcactcaggctgga    21775
LOC105372935.clincRNA.NCBI.ref      ------------------------------------------------------------    10810

GGT5.NCBI.REF                       gtgcagtggcatgatcttgcctcaccacaacctccgcttcccaggttcaagcaattctcc    21835
LOC105372935.clincRNA.NCBI.ref      ------------------------------------------------------------    10810

GGT5.NCBI.REF                       tgcctcagcctctgagtagctgggactacacatgtgcgccaccacacctggctaattttt    21895
LOC105372935.clincRNA.NCBI.ref      ------------------------------------------------------------    10810

GGT5.NCBI.REF                       gtatttttagtaaagatggggtttccccatgttggccaggctggtcttgagctcccaatc    21955
LOC105372935.clincRNA.NCBI.ref      ------------------------------------------------------------    10810

GGT5.NCBI.REF                       tcaagtgatctgtccaccttggcctcccaaagtgctgggcttataggcatgagccactgt    22015
LOC105372935.clincRNA.NCBI.ref      ------------------------------------------------------------    10810

GGT5.NCBI.REF                       gcctggccctcccttgactctttttttttctttttttttgagacggagtctcgctctgttg    22075
LOC105372935.clincRNA.NCBI.ref      ------------------------------------------------------------    10810

GGT5.NCBI.REF                       cccaggctggcgtgcagtggcacgatcttggctcactgcaagctctgcctcccgagttca    22135
LOC105372935.clincRNA.NCBI.ref      ------------------------------------------------------------    10810

GGT5.NCBI.REF                       cgccattcccctgcctcatcctcccgcctcatcctcccaagtagctgggactacaggcac    22195
LOC105372935.clincRNA.NCBI.ref      ---------------taaccatgaggcccctcaggtcctgatcagtcagaatggatgctt    10855
                                                   * * * *   *** *      **   *   *  ** *  * **

GGT5.NCBI.REF                       ccaccaccacgctcggctaatttttttgtatttttagtagagacagggtttcaccgtgtta    22255
LOC105372935.clincRNA.NCBI.ref      tcaccagcagacccggccatgt--------------------------------------    10877
                                     ***** **  * **** *  *

GGT5.NCBI.REF                       gccaggatggtctcgatctcctgaccttgtgatccgcccgccttggcctcccaaagtgct    22315
LOC105372935.clincRNA.NCBI.ref      ------------------------------------------------------------    10877

GGT5.NCBI.REF                       gggattataggcgtgagccaccgcgcccggcccctcccttgactcttgactgaaggacct    22375
LOC105372935.clincRNA.NCBI.ref      ------------------------------------------------------------    10877

GGT5.NCBI.REF                       ttgtctttgtgaacatcaattctcaggacctttcaccctggggacgtgaaatgctgagaa    22435
LOC105372935.clincRNA.NCBI.ref      ------------------------------------------------------------    10877

GGT5.NCBI.REF                       tttgggagatgacagtctggggactgggattaatggaatccagtgacccacaaacctaag    22495
LOC105372935.clincRNA.NCBI.ref      ------------------------------------------------------------    10877

GGT5.NCBI.REF                       gttctcagctcccttggggagttggaatgtcagctattcaggtctagggctttccatgga    22555
LOC105372935.clincRNA.NCBI.ref      ------------------------------------------------------------    10877

GGT5.NCBI.REF                       gtaaatcctaaactctgggttggagactttaagcctccaaggaccttcacagctaaggcc    22615
LOC105372935.clincRNA.NCBI.ref      ------------------------------------------------------------    10877
```

```
GGT5.NCBI.REF                      cagggactagggcgaggagagtctttgatcctcagagtcttggagtttggccagtggact    22675
LOC105372935.clincRNA.NCBI.ref     ------------------------------------------------------------    10877

GGT5.NCBI.REF                      ctgaggaatggagtctctgagcactgaaggtccaactttggcttcagcagtaaaggatct    22735
LOC105372935.clincRNA.NCBI.ref     ---------------------ggctgctcggtcctgggtgctcgctgctgtgcaagacat    10916
                                                          ***   *  *     **    * ** **  * **  *

GGT5.NCBI.REF                      tggccttcaagtctaaggacagtgggcaattagtaggtcaggcatggggaactcatagcc    22795
LOC105372935.clincRNA.NCBI.ref     tagccctttagttatgagcctgtgggaacttcaggggttcccagtggggagagcagtggc    10976
                                   * *** *  ***     * * ***** * **    ***      ******   **  * *

GGT5.NCBI.REF                      aaacgtgcagggctccaaagacctcatttgccctgtcagcagctcaggccatgtggcatc    22855
LOC105372935.clincRNA.NCBI.ref     ------------------------------------------------------------    10976

GGT5.NCBI.REF                      acccgatgcatctagatgtctccggaatctcaggccctgcagggtgaggttctcgggcca    22915
LOC105372935.clincRNA.NCBI.ref     ------------------------------------------------------------    10976

GGT5.NCBI.REF                      ttagtttttttttgttttgttttgttttgttttttttggcttgttgttgttgagacaaagtt    22975
LOC105372935.clincRNA.NCBI.ref     ------------------------------------------------------------    10976

GGT5.NCBI.REF                      tcactctgtcacccaggctggagtgcagtggcgcgatctcagcttattgcaacctccacc    23035
LOC105372935.clincRNA.NCBI.ref     ------------------------------------------------------------    10976

GGT5.NCBI.REF                      tcctgggttcaagcaattctcatgtctcagcctcccaagtagctgggattacaagtgtgt    23095
LOC105372935.clincRNA.NCBI.ref     ------------------------------------------------------------    10976

GGT5.NCBI.REF                      gccaccaagcctggctaatttttgtatttttagcagaaacagcgtttctccatgttggcc    23155
LOC105372935.clincRNA.NCBI.ref     ------------------------------------------------------------    10976

GGT5.NCBI.REF                      aggctggtctcaaactcctgacctcaggtgatctgcccaccttggcctcccaaagtgctg    23215
LOC105372935.clincRNA.NCBI.ref     ------------------------------------------------------------    10976

GGT5.NCBI.REF                      ggattacaggcatgaccaccgcgcctggctagggagcagtgtttttaaggacaacttggc    23275
LOC105372935.clincRNA.NCBI.ref     ------------------------------------------------------------    10976

GGT5.NCBI.REF                      gggttgggggaagccaatgagccaggagtgctgataggtcagggatgaaatcatagggag    23335
LOC105372935.clincRNA.NCBI.ref     ------------------------------------------------------------    10976

GGT5.NCBI.REF                      ttggctgcgcgcggtggctcccgcctacaatcccagcactttgggaggctgaggtgggtg    23395
LOC105372935.clincRNA.NCBI.ref     ------------------------------------------------------------    10976

GGT5.NCBI.REF                      gttcacctgaggtcaggagaccagcctggccaacatggcgaaaacctgtctctactaaaa    23455
LOC105372935.clincRNA.NCBI.ref     ------------------------------------------------------------    10976

GGT5.NCBI.REF                      ttacaaaaattagctgggcaaagtggcaggcatctgtaatcccagctactggggaggctg    23515
LOC105372935.clincRNA.NCBI.ref     ---------------------agtgggaggcatctgggggccaaaggtcag---------    11006
                                                        ***** *********    ** *    * *

GGT5.NCBI.REF                      aggcaagagaatcacttgaacctgggaggggaagttgcagtgagccaaggtcgtgccatt    23575
LOC105372935.clincRNA.NCBI.ref     ------------------------------------------------------------    11006

GGT5.NCBI.REF                      gcacgccagcatgggtgacagagcgggactccatctcagaaacagacacacacaaaaaac    23635
LOC105372935.clincRNA.NCBI.ref     ------------------------------------------------------------    11006

GGT5.NCBI.REF                      ccctggaaatcatagaatcatagggagttgaagctttcttcttgagctgagtccgttcct    23695
LOC105372935.clincRNA.NCBI.ref     ------------------------------------------------------------    11006

GGT5.NCBI.REF                      gggtgggggccacaaggtcagatgagccagttaatcgatctggatggtgccagctgatcc    23755
LOC105372935.clincRNA.NCBI.ref     ------------------------------------------------------------    11006

GGT5.NCBI.REF                      atcaagtgcagggtctgcaaaatttctcaagcactgatcttaagagcagtttagggaggg    23815
LOC105372935.clincRNA.NCBI.ref     ------------------------------------------------------------    11006

GGT5.NCBI.REF                      tcagaatctttagccttcagctgcgtgactcctaaaccataatttctaatcttgtggcta    23875
LOC105372935.clincRNA.NCBI.ref     ------------------------------------------------------------    11006

GGT5.NCBI.REF                      atgtgagtcctacgaaggcagtctggtgcccaggcaagaaggaagtctgctttgggaaag    23935
LOC105372935.clincRNA.NCBI.ref     ------------------------------------------------------------    11006

GGT5.NCBI.REF                      ggctgttaccgtctttgttttaaactataaactaagtttctctcaaagttagttcagcct    23995
LOC105372935.clincRNA.NCBI.ref     ------------------------------------------------------------    11006
```

```
GGT5.NCBI.REF                       acacccaggaatgagcaaggacaactgggaggttagaagcaagatggagtcgattaagtt    24055
LOC105372935.clincRNA.NCBI.ref      ------------------------------------------------------------    11006


GGT5.NCBI.REF                       agatctctttccctgtctcagtcataattttgcaaaggcggtttcagtctt-----gctg    24110
LOC105372935.clincRNA.NCBI.ref      -----------------------------tggcaggggtatttcagtattatacaactg    11037
                                                                 * ***  **   ******* **      ***

GGT5.NCBI.REF                       ttttgcccaggctggagtgcagtgtcatgatcaaagctcactgcagcctcaaactcctag    24170
LOC105372935.clincRNA.NCBI.ref      ctgtgaccagacttgtatactggctgaatatcagtgctgtttgtaatttttcactttgag    11097
                                     * ** **** ** *  * * *  * *  ****  ***   ** *   *   ***   **

GGT5.NCBI.REF                       cctcaaggatcctcctgcttcagcctcctgagtagctgggactataagcacagggccac    24230
LOC105372935.clincRNA.NCBI.ref      aacca---------------------------------------------acattaattc    11112
                                       **                                             ***      *

GGT5.NCBI.REF                       cacatccgactaatttttttcttttttcttttctttatttttttaataaagacgtgatctt    24290
LOC105372935.clincRNA.NCBI.ref      catatgaatcaagtgttttgtaactgctattcatttattcagcaaatatttattgatcat    11172
                                    ** **    * * * **** *   * ** *** ******    **  *     ***** *

GGT5.NCBI.REF                       g----ctacattgcccaggctggtcttgaactcctgagctcaaaagatcctcctgcgccg    24346
LOC105372935.clincRNA.NCBI.ref      ctcttctccataagatagtgtgataaacacagtcatgaataaagttattttccacaaaa-    11231
                                         ** ***     **  ** *    *    *     * **   **  ***

GGT5.NCBI.REF                       gcctcccaaagtgctgggattacaggtgtgagccaccacgcccagcccaattttttgattt    24406
LOC105372935.clincRNA.NCBI.ref      ------------------------------------------------------------    11231


GGT5.NCBI.REF                       tcttttagagacggtgtcttgctttgttgccctggctgttctcaaactcctggcctcaag    24466
LOC105372935.clincRNA.NCBI.ref      ------------------------------------------------------------    11231


GGT5.NCBI.REF                       tgatcctgccaccttggctcccaagtggctaagactgtaggtatgtgccaccacgcctgg    24526
LOC105372935.clincRNA.NCBI.ref      ------------------------------------------------------------    11231


GGT5.NCBI.REF                       cttttttttatttcttatttttttattttttagaaacaggatcttgctgcattgcccaggc    24586
LOC105372935.clincRNA.NCBI.ref      ------------------------------------------------------------    11231


GGT5.NCBI.REF                       tggtctcaaactcctggcctcaagtgatcctccttcctcagcctcccaaattgctggcat    24646
LOC105372935.clincRNA.NCBI.ref      ------------------------------------------------------------    11231


GGT5.NCBI.REF                       tacaggtgtgaaccactgtgtctggcctcagtgtctaaatcttgagaagccacatcctcc    24706
LOC105372935.clincRNA.NCBI.ref      ------------------------------------------------------------    11231


GGT5.NCBI.REF                       atcgaaggccccctgaactcagatttgcacctcaaggccctggtgtccttggatccagaa    24766
LOC105372935.clincRNA.NCBI.ref      ------------------------------------------------------------    11231


GGT5.NCBI.REF                       cagacatgaacttgcatttggaggtcttcgggcctgggtggtgtccttcgtacatagtcc    24826
LOC105372935.clincRNA.NCBI.ref      ------------------------------------------------------------    11231


GGT5.NCBI.REF                       ctggaggctgagcttgtcctctatgggcctttggatctcttccatgctcccatctggtcc    24886
LOC105372935.clincRNA.NCBI.ref      ------------------------------------------------------------    11231


GGT5.NCBI.REF                       tcccctcttcctgtgtcttcaggccctcttcctctccgtcttgccttttgatcatttttc    24946
LOC105372935.clincRNA.NCBI.ref      ------------------------------------------------------------    11231


GGT5.NCBI.REF                       tcatttcttgcagaggcctctccctcctggagatgggctgagctgccctcccctgtgaac    25006
LOC105372935.clincRNA.NCBI.ref      ------------------------------------------------------------    11231


GGT5.NCBI.REF                       ctgctttctctcctctccaggaggtgcagaggggactccaagaccgtggccagaaccaga    25066
LOC105372935.clincRNA.NCBI.ref      ------------------------------------------------------------    11231


GGT5.NCBI.REF                       cccagaggcccttcttcctgaacgtggtccaggctgtgtcccaggagggggcctgtgtgt    25126
LOC105372935.clincRNA.NCBI.ref      ------------------------------------------------------------    11231


GGT5.NCBI.REF                       acgccgtctcggacctgaggaagagtggggaggccgcaggctactaagacactgctctgc    25186
LOC105372935.clincRNA.NCBI.ref      ------------------------------------------------------------    11231


GGT5.NCBI.REF                       ccagagctgaagtctggccccaccatgagtcctgtgtccaggccggacatggctggggga    25246
LOC105372935.clincRNA.NCBI.ref      ------------------------------------------------------------    11231


GGT5.NCBI.REF                       ccaactactctggcaggatctggacccctggcaggggagtccagctgagagtggaagagg    25306
LOC105372935.clincRNA.NCBI.ref      ------------------------------------------------------------    11231


GGT5.NCBI.REF                       tggcggggaccagctgggcagatgagaggctgagcctcatccctaaccccctttcccaga    25366
LOC105372935.clincRNA.NCBI.ref      ------------------------------------------------------------    11231
```

```
GGT5.NCBI.REF                    gcccctggtggtcctgaaccggcccctctatccctccgcaggcctcttgcctggggccac    25426
LOC105372935.clincRNA.NCBI.ref   ------------------------------------------------------------    11231

GGT5.NCBI.REF                    tctcccaccctctcgatctgtatatcctccagtccaagattaaagaggcggactgtggcc    25486
LOC105372935.clincRNA.NCBI.ref   ------------------------------------------------------------    11231

GGT5.NCBI.REF                    tga 25489
LOC105372935.clincRNA.NCBI.ref   --- 11231
```
