# Supplementary material for: Formation of human long intergenic non-coding RNA genes, pseudogenes, and protein genes: Ancestral sequences are key players
Source: PLoS One. 2020 Mar 26;15(3):e0230236. doi: 10.1371/journal.pone.0230236 (PMC7098633; doi:10.1371/journal.pone.0230236)

Formation of human long intergenic non-coding RNA genes and pseudogenes: ancestral sequences are key players

Nicholas Delihas

S6 Fig. a. Color highlighted sections represent the *FAM230B-LOC105372935 -GGT2* sequences that are found in *FAM230C-LOC101060145-GGT4P* with the respective percent identities. xxx represents sequences from the clincRNA region (*LOC105372935*) of *FAM230B-LOC105372935 -GGT2* that are missing in FAM230C-*LOC101060145-GGT4P.* The unhighlighted section, I-----I represents the 5' half sequence of *FAM230C* that does not form part of *FAM230B.* **b.** Schematic of *FAM230B-LOC105372935-spacer-GGT2* for comparisons. The % identities shown in Figure S3a are relative to the *FAM230B-LOC105372935 -GGT2* sequence.

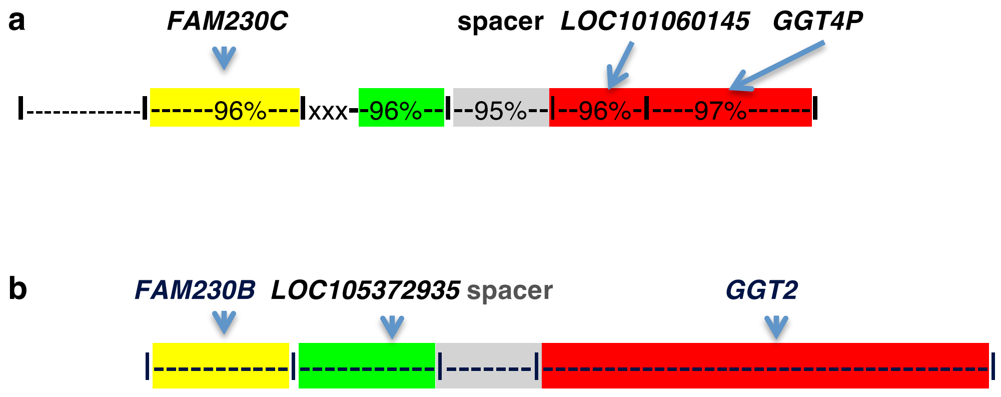

Supplement: S6 Fig — a. Color highlighted sections represent the FAM230B-LOC105372935 -GGT2 sequences that are found in FAM230C-LOC101060145-GGT4P with the respective percent identities. xxx represents sequences from the clincRNA region (LOC105372935) of FAM230BLOC105372935-GGT2 that are missing in FAM230C-LOC101060145-GGT4P. The unhighlighted section, |——| represents the 5’ half sequence of FAM230C that does not form part of FAM230B. b. Schematic of FAM230B-LOC105372935-spacer-GGT2 for comparisons. The % identities shown are relative to the FAM230B-LOC105372935 -GGT2 sequence. (PDF) [file pone.0230236.s006.pdf]
